# Prevalence of sexual violence against children and age at first exposure: a global analysis by location, age, and sex (1990–2023)

**DOI:** 10.1016/S0140-6736(25)00311-3

**Published:** 2025-05-24

**Authors:** Jack Cagney, Cory Spencer, Luisa Flor, Molly Herbert, Mariam Khalil, Erin O'Connell, Erin Mullany, Flavia Bustreo, Joht Singh Chandan, Nicholas Metheny, Felicia Knaul, Emmanuela Gakidou

**Affiliations:** a Institute for Health Metrics and Evaluation, University of Washington, Seattle, WA, USA; b Rollins School of Public Health, Emory University, Atlanta, GA, USA; c Nell Hodgson Woodruff School of Nursing, Emory University, Atlanta, GA, USA; d School of Medicine, University of North Carolina at Chapel Hill, Chapel Hill, NC, USA; e Fondation Botnar, Basel, Switzerland; f Department of Applied Health Sciences, University of Birmingham, Birmingham, UK; g Department of Medicine, David Geffen School of Medicine, UCLA, Los Angeles, CA, USA; h Institute for Advanced Study of the Americas, University of Miami, Coral Gables, FL, USA; i Escuela de Medicina y Ciencias de la Salud, Tecnológico de Monterrey Faculty of Excellence, Mexico City, México; j Tómatelo a Pecho, AC, Mexico City, Mexico

## Abstract

**Background:**

Measuring sexual violence against children (SVAC) is vital to prevention and advocacy efforts, yet existing prevalence studies present estimates for few countries. Here we estimate the prevalence of SVAC for 204 countries by age and sex, from 1990 to 2023, and also report the age at which young survivors of lifetime sexual violence first experienced sexual violence.

**Methods:**

We reviewed publicly available repositories for data on the prevalence of SVAC. To harmonise heterogeneity in the identified input data, we adjusted for alternative case definitions of SVAC and differential disclosure by survey mode. We then used a spatiotemporal Gaussian process regression to estimate a full time series of exposure to SVAC for each age-sex-country combination. We accounted for uncertainty in the underlying data and modelling processes. We also analysed the age at which adolescent and young adult survivors of lifetime sexual violence first experienced this type of violence by sex, data source, and world region.

**Findings:**

We estimate that the global age-standardised prevalence of SVAC was 18·9% (95% uncertainty interval [UI] 16·0–25·2) for females and 14·8% (9·5–23·5) for males in 2023. At the super-region level, these estimates ranged from 12·2% (9·0–17·2) in southeast Asia, east Asia, and Oceania to 26·8% (21·9–32·7) in south Asia for females and from 12·3% (5·2–24·6) in central Europe, eastern Europe, and central Asia to 18·6% (9·7–32·3) in sub-Saharan Africa for males. At the country level, age-standardised estimates ranged from 6·9% (4·8–9·6) in Montenegro to 42·6% (34·4–52·1) in Solomon Islands among females and from 4·2% (1·7–9·2) in Mongolia to 28·3% (13·2–49·8) in Côte d’Ivoire among males. Globally, these estimates remained relatively stable since 1990, with slight variations at the country and regional levels. We also find that the first experience of sexual violence among adolescents and young people occurred before the age of 18 years for 67·3% of female and 71·9% of male survivors.

**Interpretation:**

The prevalence of SVAC is extremely high for both females and males across the globe. Given data sparsity and ongoing measurement challenges, findings probably underestimate the true pervasiveness of SVAC. An overwhelmingly high proportion of survivors first experienced sexual violence during childhood, revealing a narrow yet sensitive window that should be targeted in future prevention efforts. It is a moral imperative to protect children from violence and mitigate its compounding impacts on health across the lifecourse.

**Funding:**

The Gates Foundation.

## Introduction

Sexual violence against children (SVAC) is a pervasive health and human rights issue that curtails the safety and wellbeing of children globally and accounts for a substantial portion of morbidity.^1^, ^2^ Existing studies on the health effects of SVAC have made its long-term somatic (eg, asthma and STIs) and psychiatric (eg, major depression and anxiety) effects increasingly clear,^3^, ^4^ and others have documented SVAC's association with stress-response behaviours such as alcohol and substance use.^5^, ^6^ In addition to its direct impacts on health, violence against children limits individual development, hindering both educational attainment and economic achievement,^7^, ^8^ ultimately leading to diminished wellbeing trajectories for survivors. These consequences extend beyond survivors too, as exposure to SVAC is associated with increased odds of future violence perpetration and criminal offending, thus perpetuating a cycle of violence within communities and across generations.^9^, ^10^ To counter these adverse outcomes and their compounding effects, there have been increasing investments in global movements towards prevention and ending SVAC and other forms of violence against children, including the first-ever Global Ministerial Conference on Ending Violence Against Children in 2024.^11^, ^12^, ^13^, ^14^

Accurately monitoring SVAC is essential to the future success of such prevention efforts, national and international zero-target goals, and support programmes for survivors and their families. Better evidence on the prevalence, characteristics, and consequences of SVAC can guide primary and secondary prevention efforts from health care, educational, criminal justice, and social welfare systems. Prevalence estimates can also provide benchmarks for global initiatives like the Sustainable Development Goals (SDGs), which were established by the UN to reduce global inequalities and include targets like SDG 16.2.3 that measures the proportion of young people who have experienced SVAC.^13^

**Research in context**

**Evidence before this study**

Available studies suggests that sexual violence against children (SVAC) is a widespread human rights and public health issue, yet current estimates remain limited by data sparsity and measurement challenges. Global systematic reviews to date have identified relatively few studies, the majority of which describe populations in high-income countries. Meta-analyses available from existing reviews suggest that 12–19% of girls and around 8% of boys have experienced SVAC; however, there exists a high degree of input data variability due to heterogenous case definitions, survey instruments, and safety and privacy protocols. Several multicountry survey series, including Demographic and Health Surveys (DHS) and Violence Against Children and Youth Surveys (VACS), provide comparable estimates using standard survey instruments across many low-income and middle-income countries. UNICEF released a set of global and regional prevalence estimates for 2023 based, in part, on synthesis of many of these surveys. However, to date, there has not been a systematic effort to synthesise these primary survey data to produce estimates of SVAC by country, age, sex, and year. Even more, underlying SVAC studies and surveys are limited by differential disclosure, a form of self-report bias that has been documented extensively by WHO, among others. Thus, while the existing evidence shows a high burden of SVAC across a range of contexts, there remains a pressing need to synthesise all available data while accounting for between-study differences and differential reporting to create estimates of SVAC that are comparable over time, geography, and demographic characteristics.

**Added value of this study**

This study generates estimates of SVAC prevalence for all age groups and sexes across 204 locations for the years 1990–2023. We reviewed three global epidemiological databases managed by the Institute for Health Metrics and Evaluation, WHO, and the United Nations Entity for Gender Equality and the Empowerment of Women for population-based survey data reporting on exposure to SVAC. Our case definition of SVAC aligns with Sustainable Development Goal Indicator 16.2.3 and the International Classification of Violence Against Children framework. This study provides one of the first sets of national, regional, and global estimates of SVAC prevalence that can be used to track progress towards zero target policy goals. Studies using alternate case definitions were adjusted using data-driven patterns and methods, allowing us to leverage the maximum amount of available data and enhance comparability between sources. Adjustments were also made to account for differential reporting across survey modes in violence surveys, addressing a well established challenge in the field. In addition, we drew upon two multicountry datasets (DHS and VACS) to characterise the age windows at which individuals aged 13–24 years first experience sexual violence, information which is crucial for the prevention of, and screening for, sexual violence against young people.

**Implications of all the available evidence**

After reviewing all available evidence, we find persistent data gaps, emphasising the need for expanded survey and surveillance programmes. The variability in the instruments used across countries also highlights the urgent need for best practices for the measurement of SVAC to be implemented across all national and multinational survey programmes. Despite the inherent data limitations, our synthesis of existing data shows that SVAC prevalence is high among males and females, even in recent generations, and that sexual violence usually first occurs early in life. SVAC has life-long consequences which span health, social, and educational spheres, and the protection of children from sexual violence requires multisectoral interventions and approaches which create safe environments, foster gender-equity, and increase access to support services. The location-specific and population-specific results we present are crucial to informing advocates and policy makers and law makers as they continue to address SVAC. Given the wide range in estimated prevalence across countries, more research is also needed to understand these variations and explore whether they reflect differences in effective child protection policies or are instead driven by barriers to disclosure, reporting, and care.

In order to create comparable, standardised estimates that monitor and inform global indicators like the SDGs, synthesis methods which account for between-study heterogeneity and limitations in individual survey data are needed. The few studies that summarise the prevalence of SVAC globally have shown its widespread nature. In a 2011 meta-analysis of 217 studies published between 1982 and 2008, Stoltenborgh and colleagues estimated the pooled prevalence of SVAC to be 18·0% for girls and 7·6% for boys.^15^ These results were consistent with Pereda and colleagues’ 2009 review of 65 studies from 22 countries, which found a pooled prevalence of 19·7% among girls and 7·9% among boys.^16^ However, these analyses are limited in their geographical coverage, and they show considerable between-study heterogeneity in estimates, with reported prevalence varying widely depending on location, data collection methods, and specific types of sexual violence measured. For example, another systematic review found that the prevalence of SVAC within identified studies ranged from zero to 69% for girls and zero to 47% for boys depending on the study design and the types of violence reported.^17^

Most recently, UNICEF estimated that one in eight girls and women and one in 11 boys and men experienced rape or sexual assault during childhood.^18^ These estimates drew upon nationally representative household surveys published between 2010 and 2022 and applied corrections to account for heterogeneity in case definitions and improve data comparability. However, the report only presented regional and global results for men and women, without any further disaggregation by country, age, or time. Aggregate estimates mask important country-specific or age-specific trends, and further assessment of prevalence over time is needed to monitor progress towards SDG indicators and understand the effectiveness of policies and interventions that work to protect children from harm.

In this study, we sought to address the above-described measurement and data synthesis challenges by using methods from the Global Burden of Diseases, Injuries, and Risk Factors Study (GBD) to estimate SVAC prevalence by sex and age group for 204 countries and territories from 1990 to 2023. Of note, GBD methods allow us to leverage data available in geographically and epidemiologically proximal countries to inform the estimation of SVAC prevalence even in locations that might not have published data available, a strategy which fills in persistent knowledge gaps regarding the burden of SVAC in these areas. This study also aimed to account for differential disclosure across survey modes, a common form of self-report bias in violence studies. We additionally drew upon two multicountry data sources to characterise the distributions of age at first experience of sexual violence among individuals aged 13–24 years who had ever experienced sexual violence, identifying age windows crucial to the prevention of, and screening for, sexual violence against young people. This manuscript was prepared as part of *The Lancet* Commission on Gender-based Violence and Maltreatment of Young People, a collaboration established to generate the information needed to monitor and prevent SVAC, among other forms of violence.^14^

## Methods

### Overview

Here we summarise the main steps in the prevalence estimation process, and the appendix provides further details on data sources and modelling strategies (pp 5–13). All analyses were done in R. This study adheres to the GATHER^19^ recommendations (appendix pp 3–4).

### Definitions and data sources

The case definition of SVAC used in this study is having ever experienced intercourse or other contact sexual violence (ie, fondling and other sexual touching) before the age of 18 years, in which the contact was unwanted (ie, physically forced or coerced). This case definition does not include online abuse or exploitation, as information on these forms of violence is usually measured separately.^20^ It also closely aligns with SDG Indicator 16.2.3^13^ and the International Classification of Violence Against Children (ICVAC),^21^ a framework established by UNICEF that provides a set of internationally agreed upon operational concepts and definitions. In keeping with this system and the UN Convention on the Rights of the Child,^12^, ^21^ we use the term violence to refer to any sexual violence against children, regardless of victim–perpetrator relationship. We consider alternative case definitions in our data seeking, extraction, processing, and modelling stages as described below.

We searched the Global Health Data Exchange, the WHO Global Database on the Prevalence of Violence against Women,^22^ and the United Nations Entity for Gender Equality and the Empowerment of Women (UN Women) Global Database on Violence against Women^23^ for data reporting on exposure to SVAC based on our case definition or an acceptable alternative definition (appendix pp 6–7). Each database was selected for employing targeted and systematic search strategies to identify data on sexual violence against children, resulting in a comprehensive list of sources spanning multiple time periods, geographies, and populations. Briefly, the Global Health Data Exchange is a catalogue of health and demographic surveys, censuses, disease registries, surveillance systems, statistical yearbooks, and scientific publications that have been identified by systematic and targeted data seeking efforts by the Institute for Health Metrics and Evaluation and its network of collaborators. The WHO Global Database on Prevalence of Violence Against Women contains representative prevalence studies of sexual violence that were identified via a systematic review of six electronic databases^24^ and supplemented by targeted, manual searches of grey literature. Lastly, UN Women's Global Database on Violence against Women contains relevant surveillance systems, reports, surveys, laws, and legislations submitted by UN member states. Further details on each database can be found in the appendix (pp 5–6).

**Figure 1:**
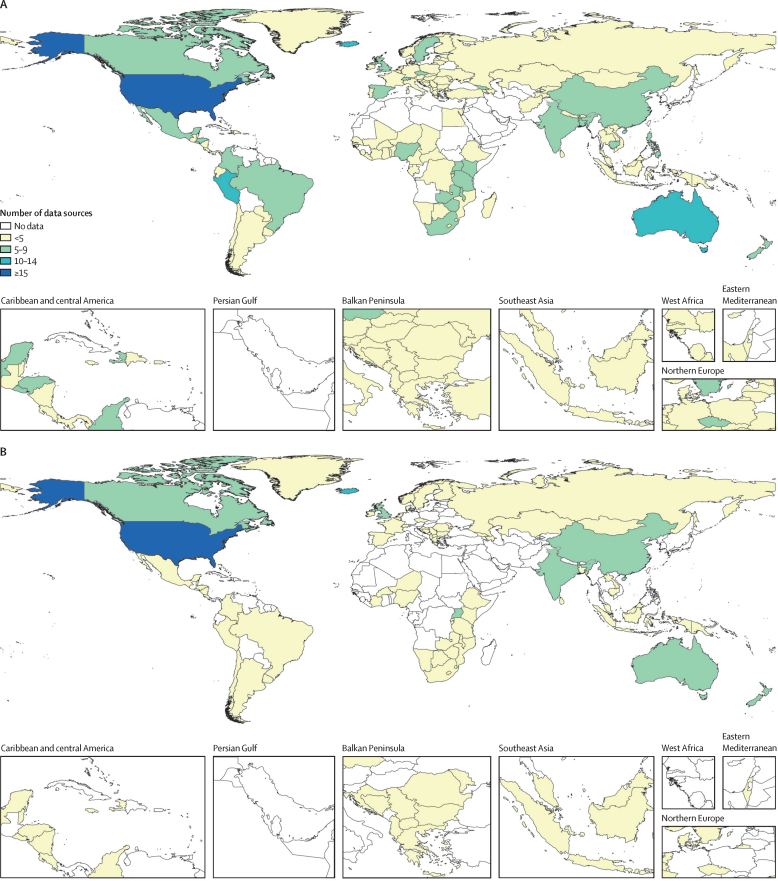

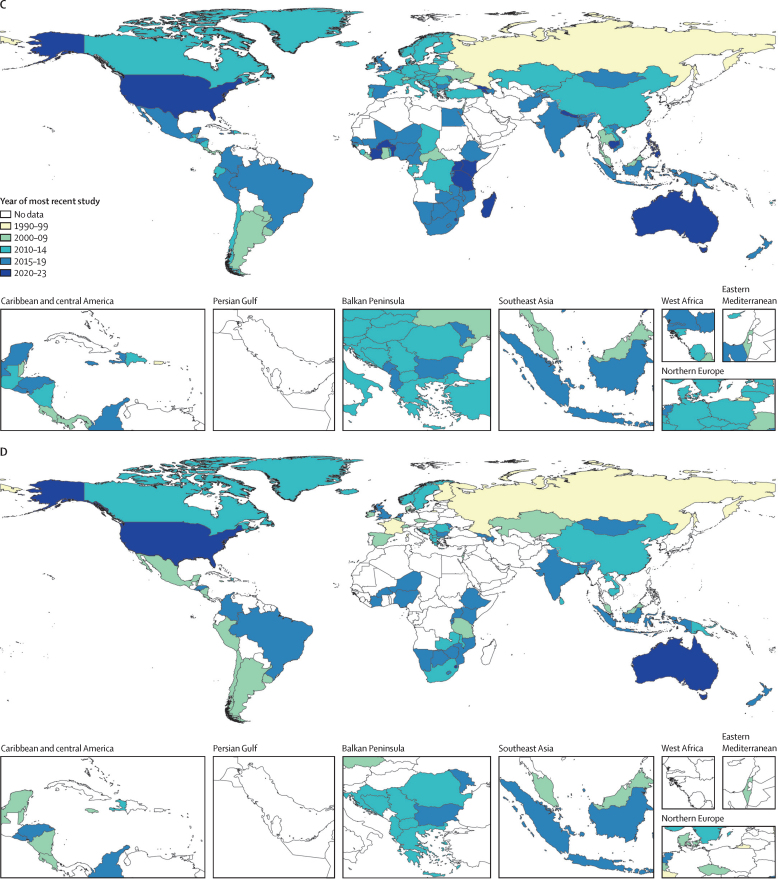
Number of sexual violence against children data sources for females (A) and males (B); most recent year of data for females (C) and males (D) These maps display the sources used in our estimation models for the prevalence of sexual violence against children. Maps A and B denote the total number of sources available in each country for females and males, respectively. Maps C and D display the most recent year of data available in each country for females and males, respectively.

When reviewing each database, we accepted and included all sources that provided self-reported information on SVAC prevalence among individuals aged 10 years and older between 1980 and 2023 and met the following inclusion criteria: (1) were population-based; (2) reported on a sample representative of a national or subnational (ie, first administrative level) location; and (3) measured self-reported instances of violence or abuse. Following current methodological guidelines, administrative records and crime data were excluded due to high under-reporting and geographic variability in comprehensiveness.^25^ Case definitions using alternative age limits (eg, before 16 years) or violence types (eg, forced intercourse only) to define SVAC were accounted for with adjustment factors based upon meta-regressions described below. In total, we identified 451 data sources from 141 countries for females and 195 data sources from 77 countries for males (figure 1). Additional details on inclusion criteria, case definitions, data extraction, and data processing are included in the appendix (pp 6–13). A list of all included data sources can be viewed in the appendix (pp 587–635).

### Data cleaning and adjustments

We used the meta-regression Bayesian, regularised, trimmed (MR-BRT)^26^ tool to generate adjustment factors for data that were reported using an alternative definition to our case definition. Based upon within-study comparisons of different case definitions of SVAC, we calculated adjustment factors for the subtype of SVAC experienced (eg, intercourse-only sexual violence) and the age used to define childhood (eg, occurring before age 16 years *vs* age 18 years; appendix p 9). Regression results suggested that alternative case definitions which included non-contact forms of SVAC (in addition to contact forms of sexual violence) captured more cases of SVAC than our reference definition and should therefore be adjusted downwards (β coefficient 0·434). Conversely, we found that alternative case definitions measuring SVAC as intercourse only or measuring SVAC as experiences occurring before ages of 16 years or 12 years (rather than before age 18 years) captured fewer cases of SVAC than our reference definitions and were adjusted upwards (β –0·426, –0·455, and –1·279, respectively; appendix p 9).

Data were also adjusted for differential disclosure by survey mode. Previous research has shown that due to the sensitive and stigmatised nature of SVAC, survivors might not always disclose SVAC in traditional face-to-face interviews.^25^ Previous studies suggest that the ways in which surveys are administered can influence disclosure patterns and, consequently, prevalence estimates.^27^, ^28^ Drawing upon these studies—primarily, the WHO Multi-Country Study on Women's Health and Domestic Violence against Women^27^ and national violence against women surveys that used the same methodology—we modelled the relationship between SVAC prevalence estimates derived from face-to-face interviews and confidential self-reports. Model results were used to linearly transform input data (appendix pp 9–11).

Given that the definition of SVAC pertains to experiences occurring before the age of 18 years, adults aged 18 years and older have completed the window during which prevalence can increase. In addition, in contiguous surveys of the same population in Türkiye, we observe that prevalence does not vary considerably within a birth cohort after they reach age 18 years (appendix p 11). We therefore extrapolate data reported in 5-year age groups in 5-year increments beginning at age group 20–24 years (appendix pp 11–12). For example, the prevalence reported by a given survey for individuals aged 25–29 years in 2015 can also be considered as an estimate of SVAC prevalence for those aged 20–24 years in 2010 and for those aged 30–34 years in 2020. However, in recognition of demographic changes over time as well as SVAC's association with adverse health outcomes, and in line with previous bias-adjusted cohort-based models,^29^ the uncertainty around these extrapolated data points was inflated by a factor of 2. A factor of 2 was chosen after conducting a series of sensitivity analyses, presented in the appendix (p 12). Singular data points that spanned multiple age groups were not extrapolated. Instead, these data were split into 5-year age groups based on the observed age-sex patterns of SVAC recorded by data sources reporting age-specific prevalence in these same age groups^30^ (appendix p 11).

### Modelling the prevalence of sexual violence against children

We used spatiotemporal Gaussian process regression (ST-GPR),^2^ a three-stage modelling approach that draws strength across data sources, to produce a full-time series with uncertainty by location, age, and sex. The first stage fits a custom linear regression model that predicts SVAC trends using data from both males and females, which allows the global ratio of SVAC exposure between sexes to inform the priors of consequent models and stages. The second stage smooths the linear regression's residuals across time and space to generate non-linear trends that better follow the observed data. The final stage then uses this non-linear trend as a mean function in a Gaussian process regression, which accounts for input data variance and generates uncertainty intervals. Of note, this stage considers the inflated uncertainty of extrapolated data points and down-weights them accordingly.

ST-GPR uses information from directly observed input data and from neighbouring geographies, age groups, and time periods to generate generalisable estimates. However, in extremely data-sparse locations and world regions, the model can overfit observed data points, creating implausible time trends for a lifetime prevalence. We therefore introduced a subsequent modelling step to smooth time trends in the absence of observed data, applying Holt's linear trend method (extended simple exponential smoothing) to forecast and backcast draws from this initial ST-GPR model. This process creates dampened forecasts and smoothed trends using a weighted moving average of past observations, where weights decay exponentially as observations get older.^31^ The appendix provides full details on these modelling strategies (pp 12–13).

These modelling processes generate prevalence estimates for each 5-year age bin (20–24, 25–29, 30–34... 90–94, and ≥95 years) by sex and location. To account for variations in age structures across populations, the modelled, age-specific prevalence estimates were also used to produce age-standardised prevalence rates using the GBD standard population structure (appendix p 5). While we estimate the prevalence of SVAC for all ages 15 years or older, we limit age-standardisation methods to include females and males aged 20 years or older to better align with the study's case definition of SVAC.

### Uncertainty analysis

We captured and propagated uncertainty through all steps of the analysis, including sampling uncertainty from data extraction and modelling uncertainty from the MR-BRT, ST-GPR, and Holt models. Ultimately, we produced 1000 draws of exposure estimates for each geography, year, age, and sex, and 95% UIs were calculated as the 2·5 percentile and 97·5 percentile of the distribution.

### Presentation of estimates

Our modelling processes produced age-sex-location-year-specific estimates for males and females in 5-year age groups (from age 15 years to age ≥95 years) in 204 countries. These estimates were then age-standardised using standard GBD populations. In the main text, we report the age-standardised estimates for females and males older than 20 years as well as estimates for those aged 20–24 years, who represent the youngest age group following the period of childhood defined as aged 0–18 years in ICVAC, the SDGs, and many countries. We present these results globally, by world region, and by country for the year 2023. To monitor how lifetime prevalence of SVAC might be changing over time, we additionally highlight differences in prevalence among those aged 20–24 years between 1990 and 2023. All other age-sex-decade-specific results can be found in the appendix (pp 24–583).

### Reported age at first experience of sexual violence

We also examined data on the age at which young individuals first experienced sexual violence using two multicountry data sources: the Demographic and Health Surveys (DHS) and the Violence Against Children and Youth Surveys (VACS). These sources include nationally representative information for low-income and middle-income countries (LMICs) and constitute two of the largest sets of sexual violence data that are comparable across time periods and locations.^32^, ^33^ Sexual violence data in the DHS come from an optional questionnaire module which asks female respondents aged 15–49 years about different acts of violence perpetrated by partners and non-partners over the lifetime, whereas VACS data cover multiple forms of violence against children (eg, sexual, physical, and emotional) and samples both male and female respondents aged 13–24 years. In both surveys, individuals who have experienced any form of sexual violence at any point in their lifetime are asked to recall the age at which they first experienced this type of violence. The specific survey questions used within both DHS and VACS to identify respondents who had ever experienced sexual violence and the age at which they first experienced such violence are provided in the appendix (pp 18–20).

Drawing upon individual-level data from 88 DHS in 52 countries and 16 VACS in 15 countries, we computed distributions of reported age at first experience among respondents aged 13–24 years who had ever experienced sexual violence. We calculated the percent of first experiences which occurred before ages 12, 16, and 18 years by respondent sex, data source, world region, and across the entire set of available data. Because DHS and VACS data primarily represent populations in LMICs, certain GBD super-regions (ie, high income and north Africa and the Middle East) are not represented within these underlying data and our analysis; distributions presented across the entire set of available data do not represent the global population but rather countries for which these data were available. Additionally, these distributions capture age patterns in respondents’ first experiences of violence only and do not provide information on the incidence of or repeated exposure to violence in childhood.

We specifically examined distributions and calculated percentiles for the subset of female respondents aged 15–24 years within the DHS data. DHS data span ages 15–49 years, and among this full set of data, respondent age was moderately correlated with reported age at first experience (*r*=0·331). This pattern might reflect recall bias in the data, whereby older respondents are less likely to recall sexual violence that occurred earlier in life. VACS data reflect respondents aged 13–24 years only and respondent age was not correlated with reported age at first experience (*r*=0·097 for females and *r*=0·135 for males). Our restriction of the DHS sample therefore minimised the potential for recall bias in older ages as well as enhanced comparability with the female sample from the VACS. DHS distributions among all women aged 15–49 years are additionally presented in the appendix (p 21).

Age distributions reflect unweighted observations from respondents aged 15–24 years (DHS) and 13–24 years (VACS), and observations with a missing or implausible age (eg, negative numbers or numbers greater than participant's current age) were not included. There was a high level of missingness among the age at first experience variable within certain DHS modules, and we tested for associations between missingness in this variable and respondent age, educational level, and urbanicity. Across available data from all DHS modules, respondents with a missing value were slightly older compared with those without a missing value. Differences in the proportion of respondents who had completed primary education and who lived in an urban area were small but significant among those with a missing response compared with those with a non-missing response (appendix pp 21–22). Although these findings do not suggest significant bias, in the absence of reliable predictors to impute or replace missing age at first experience values, we chose to exclude DHS modules for which greater than 50% of responses for the age at first experience of sexual violence variable were missing for our main analysis, resulting in a final data set which combined data from 47 DHS in 34 countries. We also present DHS analyses using all available data regardless of survey-level missingness as a sensitivity analysis in the appendix (p 23). Missingness levels were comparatively lower among the VACS data (female modules: mean 3·38%, range 0–8·94%; male modules: mean 6·61%, range 1·21–19·8%) and no modules were excluded due to unacceptable degrees of missingness. Across all data available from VACS modules, a missing response in the age at first experience variable was not associated with respondent age or whether a respondent had completed primary education but was associated with respondent sex, with males more likely to have a missing value (appendix p 21). Levels of missingness in the age at first experience module are presented by survey module for both DHS and VACS in the appendix (pp 15–18).

More information on this analysis, including data sources, exclusion criteria, and analytical methods, can be found in the appendix (pp 13–23).

### Role of the funding source

The funder of the study had no role in study design, data collection, data analysis, data interpretation, or writing of the report.

## Results

After searching three global databases, we identified 460 sources reporting on SVAC prevalence (451 among females and 195 among males). Maps displaying the count and recency of studies by geographic location are displayed in figure 1.

Among females, data availability was highest in countries in the high-income super-region (139 sources) and lowest in north Africa and the Middle East (five sources). Among the 204 national locations for which we created estimates, we identified no usable data sources for female SVAC in 63 (30·9%) locations (figure 1A). Data coverage improved over time, with 123 (27·3%) identified sources coming from the last decade (2014–23). The sources from this past decade span 76 geographies in 17 world regions. Only 15 locations had data published more recently than 2020 (16 studies, 3·5%), while 119 had data published more recent than 2010 (219 studies, 48·6%, figure 1C). Among the 141 locations with data, we find that 56 (39·7%) have only 1 year of available data.

Similarly, for males, the greatest number of sources were identified in countries in the high-income super-region (95 sources). North Africa and the Middle East had no eligible sources reporting male SVAC prevalence. Data sparsity was much greater for males compared with females, with 127 locations (62·3%) having no usable input data (figure 1B). Approximately a fourth (23·1%, n=45) of identified sources for male SVAC came from the last decade, with three locations having data more recent than 2020 and 55 having data more recent than 2010 (figure 1D). Among the 77 locations with data for males, 43 (55·8%) had only 1 year of eligible information.

Data availability was overall higher for females than males; in 64 locations, there are data for females but none for males. All countries that had data available for males also had data available for females. There were 63 countries with no identified data sources for SVAC prevalence among females or males (figure 1).

The estimated prevalence of SVAC among females and males globally and by country, region, and super-region, in 2023, are presented in table 1. Results for additional age groups, as well as for the years 1990, 2000, 2010, 2020, and 2023, are presented in the appendix (pp 24–583).

**Table 1:** Age-standardised (20 years and older) and age-specific (20–24 years) prevalence of sexual violence against children among females and males globally and by country, region, and super-region, in 2023

		**Females, age-standardised (% [95% UI])**	**Males, age-standardised (% [95% UI])**	**Females aged 20–24 years (% [95% UI])**	**Males aged 20–24 years (% [95% UI])**
**Global**		**18·9 (16·0–25·2)**	**14·8 (9·5–23·5)**	**18·6 (16·4–23·3)**	**13·1 (7·8–21·9)**
**Central Europe, eastern Europe, and central Asia**		**13·8 (7·7–25·8)**	**12·3 (5·2–24·6)**	**13·1 (7·1–25·1)**	**11·4 (4·9–22·9)**
Central Asia		13·5 (6·0–27·5)	7·3 (3·1–15·0)	12·9 (5·5–26·9)	6·4 (2·5–13·7)
	Armenia	12·1 (7·8–20·4)	7·1 (2·7–15·6)	10·1 (7·1–14·0)	6·5 (2·5–14·3)
	Azerbaijan	9·8 (5·0–17·2)	8·6 (4·9–14·2)	8·5 (4·1–15·6)	5·9 (2·7–11·4)
	Georgia	15·0 (11·8–19·1)	7·1 (4·4–11·2)	20·3 (16·0–25·2)	5·0 (2·3– 9·7)
	Kazakhstan	13·9 (4·9–30·8)	7·3 (2·9–15·7)	13·2 (4·6–29·6)	6·9 (2·8–14·9)
	Kyrgyzstan	13·9 (4·9–30·8)	7·1 (2·7–15·6)	13·2 (4·6–29·6)	6·5 (2·5–14·3)
	Mongolia	16·2 (10·4–24·2)	4·2 (1·7– 9·2)	13·9 (8·5–21·8)	4·1 (1·5– 9·3)
	Tajikistan	13·9 (4·9–30·8)	7·1 (2·7–15·6)	13·2 (4·6–29·6)	6·5 (2·5–14·3)
	Turkmenistan	13·9 (4·9–30·8)	7·1 (2·7–15·6)	13·2 (4·6–29·6)	6·5 (2·5–14·3)
	Uzbekistan	13·9 (4·9–30·8)	7·1 (2·7–15·6)	13·2 (4·6–29·6)	6·5 (2·5–14·3)
Central Europe		10·5 (8·0–14·8)	14·8 (6·6–28·7)	9·4 (6·7–13·6)	14·6 (7·1–27·7)
	Albania	9·0 (6·9–11·7)	17·2 (7·2–33·8)	7·7 (5·4–10·4)	17·1 (7·2–33·7)
	Bosnia and Herzegovina	14·7 (5·2–32·2)	21·0 (9·2–39·7)	14·8 (5·2–32·6)	21·4 (9·4–40·5)
	Bulgaria	9·7 (5·4–16·3)	14·0 (5·4–29·2)	8·7 (3·9–15·5)	13·5 (5·2–28·4)
	Croatia	8·5 (4·5–15·0)	13·6 (5·6–27·8)	7·7 (3·6–14·2)	13·1 (5·3–26·9)
	Czechia	14·0 (9·9–19·9)	14·0 (8·2–21·5)	10·6 (4·6–20·2)	13·9 (5·5–26·9)
	Hungary	11·5 (6·0–19·9)	14·7 (6·1–29·7)	10·2 (4·8–18·6)	14·5 (6·0–29·4)
	Montenegro	6·9 (4·8– 9·6)	14·7 (6·1–29·7)	6·0 (3·8– 8·6)	14·5 (6·0–29·4)
	North Macedonia	12·6 (7·3–22·6)	19·6 (12·0–32·0)	12·0 (5·1–23·2)	20·4 (10·4–34·4)
	Poland	10·3 (6·0–17·9)	14·7 (6·1–29·7)	9·1 (4·2–17·3)	14·5 (6·0–29·4)
	Romania	7·4 (3·9–12·9)	14·0 (5·7–28·4)	6·6 (3·1–12·1)	13·5 (5·5–27·6)
	Serbia	13·1 (4·5–29·2)	15·5 (6·4–30·9)	12·7 (4·4–28·5)	15·1 (6·2–30·5)
	Slovakia	10·4 (5·5–18·1)	14·7 (6·1–29·7)	9·3 (4·3–16·9)	14·5 (6·0–29·4)
	Slovenia	12·4 (6·5–21·4)	14·7 (6·1–29·7)	11·1 (5·3–20·1)	14·5 (6·0–29·4)
Eastern Europe		15·6 (6·1–33·1)	13·1 (5·3–26·8)	15·3 (6·1–32·4)	12·7 (5·2–26·1)
	Belarus	15·5 (5·5–33·6)	13·1 (5·3–26·8)	15·3 (5·4–33·3)	12·8 (5·1–26·3)
	Estonia	16·8 (9·1–28·2)	13·1 (5·3–26·8)	15·1 (7·3–26·5)	12·8 (5·1–26·3)
	Latvia	13·7 (7·3–23·4)	13·1 (5·3–26·8)	12·3 (5·8–21·9)	12·8 (5·1–26·3)
	Lithuania	12·5 (6·6–21·6)	13·1 (5·3–26·8)	11·2 (5·3–20·2)	12·8 (5·1–26·3)
	Moldova	22·2 (10·0–42·0)	13·2 (5·9–25·9)	22·3 (14·6–31·9)	11·1 (6·7–17·8)
	Russia	15·5 (5·5–33·6)	13·1 (5·3–26·8)	15·3 (5·4–33·3)	12·8 (5·1–26·3)
	Ukraine	15·5 (5·5–33·6)	13·1 (5·3–26·8)	15·3 (5·4–33·3)	12·8 (5·1–26·3)
**High income**		**23·7 (21·9–27·1)**	**15·0 (10·4–23·2)**	**23·2 (19·3–28·6)**	**13·9 (8·7–22·5)**
Australasia		27·9 (23·1–33·9)	17·7 (13·0–23·9)	25·2 (18·2–34·2)	19·4 (14·3–26·1)
	Australia	27·8 (22·8–34·1)	16·9 (12·1–23·0)	25·4 (18·2–34·2)	18·7 (13·5–25·3)
	New Zealand	28·8 (19·0–41·1)	21·8 (13·8–32·6)	24·5 (11·2–43·4)	22·8 (10·4–40·3)
High-income Asia Pacific		23·7 (9·2–46·8)	15·1 (6·2–30·3)	23·4 (9·0–46·6)	14·3 (5·9–28·9)
	Brunei	23·7 (9·2–46·8)	15·1 (6·2–30·3)	23·4 (9·0–46·6)	14·3 (5·9–28·9)
	Japan	23·7 (9·2–46·8)	15·1 (6·2–30·3)	23·4 (9·0–46·6)	14·3 (5·9–28·9)
	South Korea	23·7 (9·2–46·8)	15·1 (6·2–30·3)	23·4 (9·0–46·6)	14·3 (5·9–28·9)
	Singapore	23·7 (9·2–46·8)	15·1 (6·2–30·3)	23·4 (9·0–46·6)	14·3 (5·9–28·9)
High-income North America		27·2 (22·9–32·4)	15·8 (13·5–18·6)	27·5 (16·9–41·8)	13·9 (7·9–22·3)
	Canada	24·8 (17·5–34·2)	12·4 (8·3–17·6)	23·9 (12·3–38·7)	8·8 (4·3–16·7)
	Greenland	27·4 (8·9–55·3)	16·2 (5·5–34·7)	27·8 (9·1–56·1)	15·8 (5·4–34·1)
	USA	27·5 (22·0–33·9)	16·1 (13·7–19·3)	27·8 (14·9–44·4)	14·4 (7·2–24·2)
Southern Latin America		22·0 (9·0–42·8)	16·0 (7·2–30·4)	22·2 (9·2–43·2)	14·2 (6·1–27·9)
	Argentina	18·5 (7·2–37·7)	16·9 (7·3–32·8)	19·6 (7·7–39·4)	15·6 (6·7–30·5)
	Chile	31·4 (11·5–57·4)	14·5 (6·5–25·6)	31·1 (11·3–57·2)	11·3 (4·8–22·1)
	Uruguay	17·2 (6·5–35·6)	12·5 (5·2–25·2)	15·1 (5·7–32·1)	11·1 (4·6–22·7)
Western Europe		20·7 (17·5–26·1)	14·1 (8·1–24·5)	18·9 (14·0–25·3)	13·2 (7·7–22·4)
	Andorra	20·4 (7·7–41·9)	13·8 (5·6–28·1)	20·1 (7·5–41·5)	12·8 (5·2–26·5)
	Austria	11·9 (6·4–20·5)	13·8 (5·6–28·1)	10·7 (5·1–19·2)	12·8 (5·2–26·5)
	Belgium	19·7 (14·0–26·8)	9·7 (4·1–18·0)	17·1 (11·0–24·3)	7·6 (3·1–15·7)
	Cyprus	10·8 (5·8–18·5)	13·8 (5·6–28·1)	9·7 (4·6–17·2)	12·8 (5·2–26·5)
	Denmark	20·3 (11·4–31·4)	16·0 (5·4–33·7)	18·0 (9·5–29·5)	15·7 (5·2–33·3)
	Finland	20·8 (14·2–29·8)	10·7 (6·1–18·0)	17·6 (8·4–31·4)	10·4 (3·8–22·7)
	France	26·0 (15·5–39·9)	13·8 (5·6–28·1)	23·5 (11·7–38·2)	12·8 (5·2–26·5)
	Germany	19·9 (11·0–32·4)	13·8 (5·6–28·1)	17·9 (8·8–30·8)	12·8 (5·2–26·5)
	Greece	12·1 (6·5–20·8)	15·3 (6·3–30·6)	10·8 (5·1–19·6)	14·9 (6·2–30·1)
	Iceland	22·9 (13·6–37·5)	10·7 (6·7–17·6)	23·6 (10·9–41·3)	9·8 (4·7–18·1)
	Ireland	17·6 (9·1–30·1)	20·8 (13·6–31·6)	16·8 (7·8–30·9)	16·8 (7·3–32·7)
	Israel	21·2 (8·0–43·0)	18·4 (7·8–35·6)	21·0 (7·9–43·0)	19·0 (8·1–36·6)
	Italy	22·1 (15·4–30·4)	13·8 (5·6–28·1)	18·9 (11·9–28·2)	12·8 (5·2–26·5)
	Luxembourg	21·3 (11·8–34·5)	13·8 (5·6–28·1)	19·3 (9·4–32·6)	12·8 (5·2–26·5)
	Malta	16·6 (11·9–22·6)	13·8 (5·6–28·1)	14·5 (9·5–20·6)	12·8 (5·2–26·5)
	Monaco	20·4 (7·7–41·9)	13·8 (5·6–28·1)	20·1 (7·5–41·5)	12·8 (5·2–26·5)
	Netherlands	29·7 (22·7–37·7)	14·1 (8·0–22·7)	25·7 (17·9–35·1)	9·6 (4·3–18·6)
	Norway	25·3 (15·5–38·5)	14·6 (7·9–23·9)	22·8 (12·0–37·3)	10·0 (4·5–19·2)
	Portugal	9·9 (5·3–16·9)	13·8 (5·6–28·1)	8·8 (4·2–15·7)	12·8 (5·2–26·5)
	San Marino	20·4 (7·7–41·9)	13·8 (5·6–28·1)	20·1 (7·5–41·5)	12·8 (5·2–26·5)
	Spain	10·8 (7·3–15·5)	12·2 (7·3–19·9)	9·7 (5·8–15·0)	11·2 (4·6–22·2)
	Sweden	22·6 (16·5–30·4)	13·4 (7·0–22·5)	19·7 (10·7–31·1)	9·2 (3·9–18·6)
	Switzerland	19·7 (10·4–35·2)	13·6 (4·3–29·6)	18·8 (7·0–39·1)	12·8 (4·0–28·0)
	UK	24·4 (14·8–36·4)	16·5 (10·0–25·7)	22·3 (10·0–39·9)	16·9 (6·8–32·7)
**Latin America and Caribbean**		**17·6 (14·9–20·9)**	**13·9 (6·8–25·4)**	**17·6 (14·3–22·5)**	**13·4 (7·9–22·4)**
Andean Latin America		17·5 (12·9–23·3)	15·0 (6·4–29·4)	17·8 (12·3–26·1)	14·3 (6·3–27·7)
	Bolivia	14·7 (5·2–32·1)	14·9 (6·2–30·0)	14·6 (5·1–32·2)	14·3 (5·8–29·0)
	Ecuador	13·9 (10·6–18·4)	14·9 (6·2–30·0)	13·2 (5·4–24·9)	14·3 (5·8–29·0)
	Peru	20·1 (14·6–27·7)	15·1 (6·7–29·2)	21·6 (8·4–43·0)	14·3 (5·9–26·5)
Caribbean		18·1 (9·7–33·6)	16·5 (9·1–29·1)	19·0 (15·4–26·0)	17·8 (15·0–22·5)
	Antigua and Barbuda	15·9 (5·7–34·4)	12·9 (5·2–26·4)	15·7 (5·6–34·1)	12·8 (5·2–26·3)
	The Bahamas	15·9 (5·7–34·4)	12·9 (5·2–26·4)	15·7 (5·6–34·1)	12·8 (5·2–26·3)
	Barbados	15·9 (5·7–34·4)	12·9 (5·2–26·4)	15·7 (5·6–34·1)	12·8 (5·2–26·3)
	Belize	12·1 (7·3–18·8)	11·6 (7·6–17·0)	11·8 (4·5–25·6)	12·3 (5·3–24·2)
	Bermuda	15·9 (5·7–34·4)	12·9 (5·2–26·4)	15·7 (5·6–34·1)	12·8 (5·2–26·3)
	Cuba	15·9 (5·7–34·4)	12·9 (5·2–26·4)	15·7 (5·6–34·1)	12·8 (5·2–26·3)
	Dominica	15·9 (5·7–34·4)	12·9 (5·2–26·4)	15·7 (5·6–34·1)	12·8 (5·2–26·3)
	Dominican Republic	15·9 (5·7–34·4)	12·9 (5·2–26·4)	15·7 (5·6–34·1)	12·8 (5·2–26·3)
	Grenada	20·8 (15·0–28·0)	12·9 (5·2–26·4)	17·8 (11·6–25·7)	12·8 (5·2–26·3)
	Guyana	15·9 (5·7–34·4)	12·9 (5·2–26·4)	15·7 (5·6–34·1)	12·8 (5·2–26·3)
	Haiti	24·6 (15·8–38·9)	26·0 (17·4–38·9)	26·1 (13·4–42·0)	27·2 (15·5–41·0)
	Jamaica	18·3 (14·5–23·3)	18·0 (10·0–29·6)	17·3 (9·5–28·8)	17·8 (7·7–33·0)
	Puerto Rico	12·9 (4·5–29·0)	12·9 (5·2–26·4)	11·8 (4·0–27·0)	12·8 (5·2–26·3)
	Saint Kitts and Nevis	15·9 (5·7–34·4)	12·9 (5·2–26·4)	15·7 (5·6–34·1)	12·8 (5·2–26·3)
	Saint Lucia	15·9 (5·7–34·4)	12·9 (5·2–26·4)	15·7 (5·6–34·1)	12·8 (5·2–26·3)
	Saint Vincent and the Grenadines	15·9 (5·7–34·4)	12·9 (5·2–26·4)	15·7 (5·6–34·1)	12·8 (5·2–26·3)
	Suriname	15·9 (5·7–34·4)	12·9 (5·2–26·4)	15·7 (5·6–34·1)	12·8 (5·2–26·3)
	Trinidad and Tobago	15·9 (5·7–34·4)	12·9 (5·2–26·4)	15·7 (5·6–34·1)	12·8 (5·2–26·3)
	Virgin Islands	18·3 (6·7–38·5)	13·2 (5·4–27·0)	18·5 (6·8–39·0)	12·9 (5·2–26·6)
Central Latin America		17·7 (13·3–23·3)	14·4 (6·3–28·3)	17·4 (13·8–22·5)	13·0 (7·0–23·1)
	Colombia	19·6 (8·2–38·6)	15·6 (7·2–29·7)	19·1 (11·2–30·2)	11·2 (7·1–16·8)
	Costa Rica	30·9 (17·9–48·2)	19·0 (8·4–36·0)	29·2 (12·4–54·0)	18·4 (8·2–34·7)
	El Salvador	13·7 (10·8–17·6)	11·4 (5·6–21·7)	19·5 (12·2–28·7)	10·2 (6·5–15·4)
	Guatemala	11·6 (8·8–15·8)	14·6 (6·0–29·5)	12·9 (5·4–24·9)	14·2 (5·8–28·8)
	Honduras	18·1 (14·1–23·2)	15·8 (8·8–28·0)	17·0 (10·8–25·3)	14·3 (9·5–19·9)
	Mexico	17·4 (9·2–27·7)	13·6 (5·6–27·7)	17·3 (8·6–28·9)	13·1 (5·3–27·0)
	Nicaragua	17·0 (11·8–23·8)	13·3 (5·9–25·3)	17·1 (7·2–33·5)	13·1 (5·4–24·6)
	Panama	15·5 (5·5–33·7)	14·6 (6·0–29·5)	14·6 (5·1–32·2)	14·2 (5·8–28·8)
	Venezuela	16·8 (6·0–35·9)	14·6 (6·0–29·5)	16·4 (5·8–35·4)	14·2 (5·8–28·8)
Tropical Latin America		17·4 (11·3–27·5)	12·5 (6·2–21·8)	17·4 (8·9–30·7)	12·7 (6·4–21·7)
	Brazil	17·7 (11·2–28·2)	12·5 (6·2–21·9)	17·7 (8·9–31·5)	12·7 (6·3–21·4)
	Paraguay	8·9 (6·6–12·6)	13·1 (5·3–26·8)	8·8 (3·5–17·1)	12·9 (5·2–26·6)
**North Africa and Middle East**		**12·3 (4·5–28·2)**	**14·9 (6·0–30·8)**	**13·3 (6·2–28·6)**	**14·2 (5·7–29·7)**
North Africa and Middle East		12·3 (4·5–28·2)	14·9 (6·0–30·8)	13·3 (6·2–28·6)	14·2 (5·7–29·7)
	Afghanistan	12·0 (3·5–29·2)	14·9 (6·0–30·8)	12·7 (3·8–30·7)	14·2 (5·7–29·7)
	Algeria	12·0 (3·5–29·2)	14·9 (6·0–30·8)	12·7 (3·8–30·7)	14·2 (5·7–29·7)
	Bahrain	12·0 (3·5–29·2)	14·9 (6·0–30·8)	12·7 (3·8–30·7)	14·2 (5·7–29·7)
	Egypt	13·9 (4·2–32·9)	14·9 (6·0–30·8)	13·8 (4·1–32·4)	14·2 (5·7–29·7)
	Iran	12·0 (3·5–29·2)	14·9 (6·0–30·8)	12·7 (3·8–30·7)	14·2 (5·7–29·7)
	Iraq	12·0 (3·5–29·2)	14·9 (6·0–30·8)	12·7 (3·8–30·7)	14·2 (5·7–29·7)
	Jordan	12·0 (3·5–29·2)	14·9 (6·0–30·8)	12·7 (3·8–30·7)	14·2 (5·7–29·7)
	Kuwait	12·0 (3·5–29·2)	14·9 (6·0–30·8)	12·7 (3·8–30·7)	14·2 (5·7–29·7)
	Lebanon	12·0 (3·5–29·2)	14·9 (6·0–30·8)	12·7 (3·8–30·7)	14·2 (5·7–29·7)
	Libya	12·0 (3·5–29·2)	14·9 (6·0–30·8)	12·7 (3·8–30·7)	14·2 (5·7–29·7)
	Morocco	12·0 (3·5–29·2)	14·9 (6·0–30·8)	12·7 (3·8–30·7)	14·2 (5·7–29·7)
	Oman	12·0 (3·5–29·2)	14·9 (6·0–30·8)	12·7 (3·8–30·7)	14·2 (5·7–29·7)
	Palestine	12·0 (3·5–29·2)	14·9 (6·0–30·8)	12·7 (3·8–30·7)	14·2 (5·7–29·7)
	Qatar	12·0 (3·5–29·2)	14·9 (6·0–30·8)	12·7 (3·8–30·7)	14·2 (5·7–29·7)
	Saudi Arabia	12·0 (3·5–29·2)	14·9 (6·0–30·8)	12·7 (3·8–30·7)	14·2 (5·7–29·7)
	Sudan	12·0 (3·5–29·2)	14·9 (6·0–30·8)	12·7 (3·8–30·7)	14·2 (5·7–29·7)
	Syria	12·0 (3·5–29·2)	14·9 (6·0–30·8)	12·7 (3·8–30·7)	14·2 (5·7–29·7)
	Tunisia	12·0 (3·5–29·2)	14·9 (6·0–30·8)	12·7 (3·8–30·7)	14·2 (5·7–29·7)
	Türkiye	12·5 (7·9–20·3)	14·9 (6·0–30·8)	16·1 (8·9–26·2)	14·2 (5·7–29·7)
	United Arab Emirates	12·0 (3·5–29·2)	14·9 (6·0–30·8)	12·7 (3·8–30·7)	14·2 (5·7–29·7)
	Yemen	12·0 (3·5–29·2)	14·9 (6·0–30·8)	12·7 (3·8–30·7)	14·2 (5·7–29·7)
**South Asia**		**26·8 (21·9–32·7)**	**15·5 (8·2–27·3)**	**23·0 (16·3–32·5)**	**11·4 (5·3–21·7)**
South Asia		26·8 (21·9–32·7)	15·5 (8·2–27·3)	23·0 (16·3–32·5)	11·4 (5·3–21·7)
	Bangladesh	9·3 (6·6–13·7)	27·7 (17·8–41·2)	7·4 (3·9–12·5)	18·8 (9·0–32·2)
	Bhutan	11·6 (5·8–20·1)	19·5 (7·5–39·6)	10·2 (5·0–17·8)	17·6 (6·7–37·1)
	India	30·8 (25·0–37·5)	13·5 (7·0–23·8)	26·9 (15·3–40·9)	9·4 (4·3–18·3)
	Nepal	14·6 (5·1–32·1)	19·5 (7·5–39·6)	14·1 (4·9–31·3)	17·6 (6·7–37·1)
	Pakistan	14·6 (5·1–32·1)	19·5 (7·5–39·6)	14·1 (4·9–31·3)	17·6 (6·7–37·1)
**Southeast Asia, east Asia, and Oceania**		**12·2 (9·0–17·2)**	**13·6 (10·3–17·8)**	**10·1 (6·2–16·7)**	**11·3 (7·6–16·6)**
East Asia		13·0 (9·6–17·4)	13·4 (9·5–19·2)	9·9 (4·4–18·3)	10·5 (5·3–19·3)
	China	12·9 (9·7–17·4)	13·4 (9·1–19·5)	9·7 (4·3–18·3)	10·3 (4·6–19·7)
	North Korea	14·0 (4·9–31·0)	13·6 (5·5–27·8)	13·7 (4·8–30·5)	13·7 (5·6–28·2)
	Taiwan	14·0 (4·9–31·0)	13·6 (5·5–27·8)	13·7 (4·8–30·5)	13·7 (5·6–28·2)
Oceania		13·2 (10·6–18·2)	13·4 (6·2–25·0)	11·9 (9·0–16·4)	10·5 (4·5–20·5)
	American Samoa	8·0 (4·9–12·8)	13·5 (5·5–27·7)	7·2 (4·2–11·6)	12·3 (4·9–25·7)
	Cook Islands	13·1 (6·2–24·1)	13·5 (5·5–27·7)	12·7 (5·2–25·4)	12·3 (4·9–25·7)
	Fiji	20·7 (14·4–28·4)	13·5 (5·5–27·7)	20·7 (9·8–37·6)	12·3 (4·9–25·7)
	Guam	15·5 (5·5–33·6)	13·5 (5·5–27·7)	15·4 (5·5–33·7)	12·3 (4·9–25·7)
	Kiribati	25·8 (19·2–35·5)	13·5 (5·5–27·7)	24·2 (10·6–43·4)	12·3 (4·9–25·7)
	Marshall Islands	11·8 (5·3–22·0)	13·5 (5·5–27·7)	13·4 (5·5–25·6)	12·3 (4·9–25·7)
	Federated States of Micronesia	14·1 (6·0–27·3)	13·5 (5·5–27·7)	17·5 (8·1–30·7)	12·3 (4·9–25·7)
	Nauru	29·9 (17·4–46·0)	13·5 (5·5–27·7)	27·6 (14·8–44·3)	12·3 (4·9–25·7)
	Niue	15·5 (5·5–33·6)	13·5 (5·5–27·7)	15·4 (5·5–33·7)	12·3 (4·9–25·7)
	Northern Mariana Islands	15·5 (5·5–33·6)	13·5 (5·5–27·7)	15·4 (5·5–33·7)	12·3 (4·9–25·7)
	Palau	16·9 (7·6–31·2)	13·5 (5·5–27·7)	18·8 (8·7–34·8)	12·3 (4·9–25·7)
	Papua New Guinea	10·0 (7·1–15·7)	13·4 (6·2–24·1)	8·4 (4·0–14·8)	10·0 (4·3–19·4)
	Samoa	10·9 (6·3–18·8)	13·5 (5·5–27·7)	10·5 (3·6–22·5)	12·3 (4·9–25·7)
	Solomon Islands	42·6 (34·4–52·1)	13·5 (5·5–27·7)	40·7 (21·2–63·0)	12·3 (4·9–25·7)
	Tokelau	15·5 (5·5–33·6)	13·5 (5·5–27·7)	15·4 (5·5–33·7)	12·3 (4·9–25·7)
	Tonga	13·6 (8·7–21·3)	13·5 (5·5–27·7)	12·3 (4·7–25·6)	12·3 (4·9–25·7)
	Tuvalu	15·5 (5·5–33·6)	13·5 (5·5–27·7)	15·4 (5·5–33·7)	12·3 (4·9–25·7)
	Vanuatu	15·5 (5·5–33·6)	13·5 (5·5–27·7)	15·4 (5·5–33·7)	12·3 (4·9–25·7)
Southeast Asia		10·4 (6·4–18·6)	14·1 (6·3–26·8)	10·3 (6·9–17·5)	12·6 (5·8–23·8)
	Cambodia	8·5 (6·7–10·7)	19·2 (10·2–32·0)	9·7 (5·1–16·0)	13·8 (6·7–24·0)
	Indonesia	11·1 (3·7–25·4)	11·2 (5·1–20·4)	10·9 (3·7–25·2)	9·4 (4·3–16·8)
	Laos	15·7 (10·8–22·0)	22·4 (11·1–39·3)	14·1 (7·5–23·6)	24·4 (13·6–38·2)
	Malaysia	11·6 (4·0–26·4)	14·1 (5·7–28·8)	11·5 (3·9–26·4)	13·2 (5·3–27·3)
	Maldives	17·6 (12·5–26·4)	15·9 (6·6–31·9)	16·7 (6·5–33·6)	14·9 (6·1–30·1)
	Mauritius	11·1 (3·7–25·4)	15·9 (6·6–31·9)	10·9 (3·7–25·2)	14·9 (6·1–30·1)
	Myanmar	11·1 (3·7–25·4)	15·9 (6·6–31·9)	10·9 (3·7–25·2)	14·9 (6·1–30·1)
	Philippines	8·0 (4·3–14·6)	15·9 (6·6–31·9)	7·9 (2·9–16·6)	14·9 (6·1–30·1)
	Seychelles	11·1 (3·7–25·4)	15·9 (6·6–31·9)	10·9 (3·7–25·2)	14·9 (6·1–30·1)
	Sri Lanka	11·5 (6·5–19·2)	19·2 (9·3–33·9)	10·1 (5·7–16·5)	15·8 (6·8–29·7)
	Thailand	13·4 (8·0–22·7)	15·9 (6·6–31·9)	13·2 (4·6–28·6)	14·9 (6·1–30·1)
	Timor-Leste	30·1 (24·6–36·5)	15·9 (6·6–31·9)	27·6 (17·3–40·0)	14·9 (6·1–30·1)
	Viet Nam	7·1 (4·8–10·8)	15·9 (6·6–31·9)	8·4 (4·8–13·7)	14·9 (6·1–30·1)
**Sub-Saharan Africa**		**21·9 (10·7–39·2)**	**18·6 (9·7–32·3)**	**22·6 (13·3–37·2)**	**17·1 (9·9–28·0)**
Central sub-Saharan Africa		18·1 (6·6–38·2)	15·8 (6·6–31·5)	17·4 (6·3–37·0)	15·0 (6·2–30·2)
	Angola	18·3 (6·7–38·5)	15·8 (6·6–31·5)	17·6 (6·3–37·4)	15·0 (6·2–30·2)
	Central African Republic	13·0 (4·5–29·2)	15·8 (6·6–31·5)	12·6 (4·3–28·4)	15·0 (6·2–30·2)
	Congo (Brazzaville)	18·3 (6·7–38·5)	15·8 (6·6–31·5)	17·6 (6·3–37·4)	15·0 (6·2–30·2)
	Democratic Republic of the Congo	18·3 (6·7–38·5)	15·8 (6·6–31·5)	17·6 (6·3–37·4)	15·0 (6·2–30·2)
	Equatorial Guinea	18·3 (6·7–38·5)	15·8 (6·6–31·5)	17·6 (6·3–37·4)	15·0 (6·2–30·2)
	Gabon	18·3 (6·7–38·5)	15·8 (6·6–31·5)	17·6 (6·3–37·4)	15·0 (6·2–30·2)
Eastern sub-Saharan Africa		22·4 (11·3–40·0)	15·4 (8·8–25·8)	23·6 (15·7–35·7)	15·1 (11·0–20·6)
	Burundi	20·8 (7·8–42·5)	13·6 (5·5–27·6)	20·9 (7·9–42·9)	13·6 (5·5–27·7)
	Comoros	13·7 (4·8–30·4)	13·6 (5·5–27·6)	12·7 (4·4–28·7)	13·6 (5·5–27·7)
	Djibouti	20·8 (7·8–42·5)	13·6 (5·5–27·6)	20·9 (7·9–42·9)	13·6 (5·5–27·7)
	Eritrea	20·8 (7·8–42·5)	13·6 (5·5–27·6)	20·9 (7·9–42·9)	13·6 (5·5–27·7)
	Ethiopia	24·2 (8·1–50·2)	8·4 (6·8–10·6)	23·9 (8·0–49·1)	8·4 (5·4–12·3)
	Kenya	27·4 (13·9–45·9)	20·7 (10·8–34·5)	25·0 (14·6–39·0)	17·6 (9·4–28·6)
	Madagascar	20·8 (7·8–42·5)	13·6 (5·5–27·6)	20·9 (7·9–42·9)	13·6 (5·5–27·7)
	Malawi	27·7 (14·0–47·0)	19·6 (9·8–34·5)	28·5 (15·8–45·5)	21·0 (11·8–33·4)
	Mozambique	14·4 (6·5–27·2)	14·8 (6·9–28·2)	15·9 (9·3–25·8)	14·7 (9·6–21·3)
	Rwanda	30·4 (23·9–40·2)	18·7 (8·8–31·8)	34·9 (22·5–49·6)	12·8 (5·9–22·8)
	Somalia	20·8 (7·8–42·5)	13·6 (5·5–27·6)	20·9 (7·9–42·9)	13·6 (5·5–27·7)
	South Sudan	20·8 (7·8–42·5)	13·6 (5·5–27·6)	20·9 (7·9–42·9)	13·6 (5·5–27·7)
	Uganda	18·1 (8·3–33·0)	20·0 (10·7–33·2)	23·2 (11·8–38·4)	22·5 (13·5–34·1)
	Tanzania	18·8 (12·0–29·9)	20·6 (11·9–33·8)	24·7 (10·9–45·6)	20·3 (9·9–35·7)
	Zambia	26·6 (13·3–45·9)	16·4 (8·0–30·1)	27·2 (15·1–43·0)	16·5 (9·3–25·7)
Southern sub-Saharan Africa		25·1 (10·4–48·8)	18·2 (7·5–36·1)	23·4 (12·4–42·1)	16·8 (7·4–32·7)
	Botswana	28·9 (14·3–46·8)	27·1 (14·0–42·2)	22·9 (11·2–38·4)	17·0 (8·5–29·1)
	Eswatini	17·5 (9·9–28·4)	9·9 (4·9–17·2)	11·4 (6·9–17·6)	7·1 (3·8–12·0)
	Lesotho	22·1 (9·4–42·6)	12·0 (5·1–24·2)	20·3 (11·8–32·4)	10·5 (5·7–17·4)
	Namibia	21·4 (9·1–41·5)	15·2 (6·9–29·2)	19·0 (11·2–30·3)	13·8 (8·5–21·5)
	South Africa	25·1 (8·5–52·3)	20·1 (7·5–40·7)	24·5 (8·0–51·2)	19·4 (7·1–40·0)
	Zimbabwe	26·1 (16·1–41·8)	7·9 (3·5–15·8)	21·5 (11·7–34·6)	7·6 (3·7–13·6)
Western sub-Saharan Africa		21·8 (10·7–37·8)	22·4 (11·6–38·4)	23·0 (12·1–39·1)	19·6 (9·3–35·1)
	Benin	16·5 (5·9–35·5)	20·7 (9·0–39·2)	15·8 (5·6–34·4)	19·6 (8·5–37·8)
	Burkina Faso	21·7 (8·1–44·5)	13·2 (5·4–27·0)	21·5 (8·0–44·4)	11·7 (4·7–24·3)
	Cabo Verde	19·2 (7·1–39·9)	20·7 (9·0–39·2)	19·2 (7·1–40·2)	19·6 (8·5–37·8)
	Cameroon	10·4 (5·8–17·1)	20·7 (9·0–39·2)	12·9 (7·5–20·7)	19·6 (8·5–37·8)
	Chad	19·2 (7·1–39·9)	20·7 (9·0–39·2)	19·2 (7·1–40·2)	19·6 (8·5–37·8)
	Côte d'Ivoire	32·4 (13·9–58·2)	28·3 (13·2–49·8)	34·4 (15·0–60·7)	28·8 (13·5–50·6)
	The Gambia	19·2 (7·1–39·9)	20·7 (9·0–39·2)	19·2 (7·1–40·2)	19·6 (8·5–37·8)
	Ghana	19·2 (7·1–39·9)	20·7 (9·0–39·2)	19·2 (7·1–40·2)	19·6 (8·5–37·8)
	Guinea	19·2 (7·1–39·9)	20·7 (9·0–39·2)	19·2 (7·1–40·2)	19·6 (8·5–37·8)
	Guinea-Bissau	19·2 (7·1–39·9)	20·7 (9·0–39·2)	19·2 (7·1–40·2)	19·6 (8·5–37·8)
	Liberia	19·2 (7·1–39·9)	20·7 (9·0–39·2)	19·2 (7·1–40·2)	19·6 (8·5–37·8)
	Mali	19·2 (7·1–39·9)	20·7 (9·0–39·2)	19·2 (7·1–40·2)	19·6 (8·5–37·8)
	Mauritania	19·2 (7·1–39·9)	20·7 (9·0–39·2)	19·2 (7·1–40·2)	19·6 (8·5–37·8)
	Niger	19·9 (7·4–41·1)	17·8 (7·5–34·7)	19·7 (7·3–40·9)	16·8 (7·1–33·3)
	Nigeria	23·9 (13·3–37·7)	24·2 (13·5–38·2)	25·6 (14·5–40·0)	19·0 (9·9–31·5)
	São Tomé and Príncipe	15·2 (9·2–23·9)	11·9 (6·6–19·6)	18·6 (11·6–28·4)	11·0 (6·3–18·1)
	Senegal	19·2 (7·1–39·9)	20·7 (9·0–39·2)	19·2 (7·1–40·2)	19·6 (8·5–37·8)
	Sierra Leone	19·2 (7·1–39·9)	20·7 (9·0–39·2)	19·2 (7·1–40·2)	19·6 (8·5–37·8)
	Togo	19·2 (7·1–39·9)	20·7 (9·0–39·2)	19·2 (7·1–40·2)	19·6 (8·5–37·8)

UI=uncertainty interval.

In 2023, the global age-standardised prevalence of SVAC among adults older than 20 years was 18·9% (95% UI 16·0–25·2) among females and 14·8% (9·5–23·5) among males, with these rates being comparable to those estimated for 1990 (appendix pp 24–583). We observed considerable variation at the super-regional and regional levels in 2023 (table 1). At the super-region level, the age-standardised prevalence of female SVAC ranged from 12·2% (9·0–17·2) in southeast Asia, east Asia, and Oceania to 26·8% (21·9–32·7) in south Asia. For males, the prevalence ranged from 12·3% (5·2–24·6) in central Europe, eastern Europe, and central Asia to 18·6% (9·7–32·3) in sub-Saharan Africa. At the regional level, however, the age-standardised prevalence of SVAC ranged from 10·4% (6·4–18·6) in southeast Asia to 27·9% (23·1–33·9) in Australasia among females and from 7·3% (3·1–15·0) in central Asia to 22·4% (11·6–38·4) in western sub-Saharan Africa among males.

**Figure 2:**
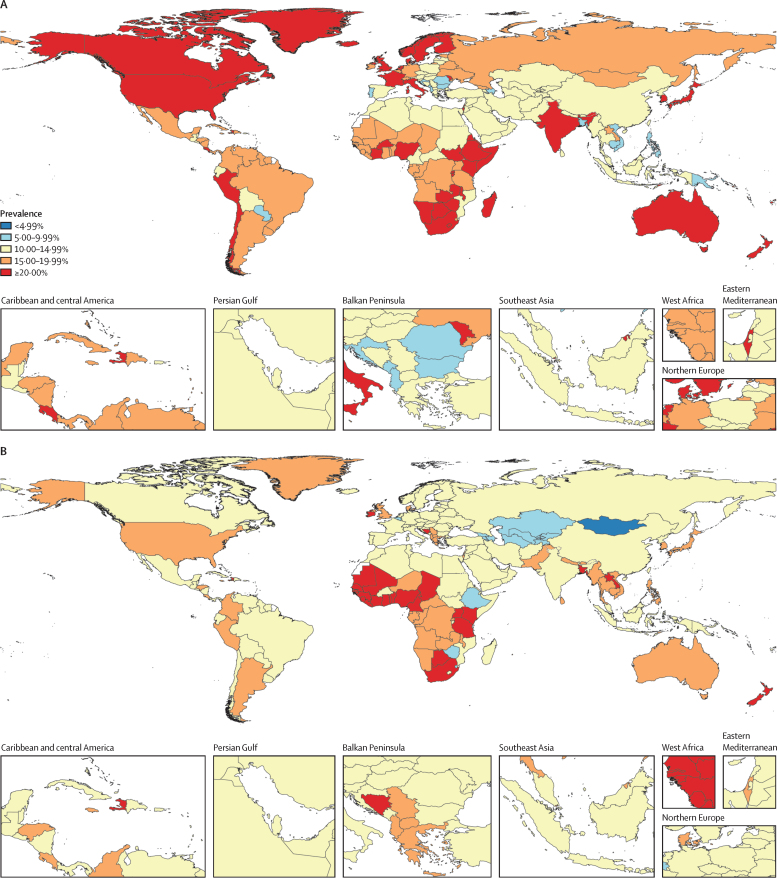
Age-standardised prevalence of sexual violence against children among females (A) and males (B) aged 20 years and older, in 2023

We observe variation within these regional groupings as well (table 1). Among the 204 countries and territories included in this analysis, Montenegro (6·9% [4·8–9·6]), Viet Nam (7·1% [4·8–10·8]), and Romania (7·4% [3·9–12·9]) had the lowest age-standardised prevalence among females while India (30·8% [25·0–37·5]), Costa Rica (30·9% [17·9–48·2]), Chile (31·4% [11·5–57·4]), Côte d’Ivoire (32·4% [13·9–58·2]), and Solomon Islands (42·6% [34·4–52·1) had the highest. Among males, the lowest country-level estimates were in Mongolia (4·2% [1·7–9·2]), Georgia (7·1% [4·4–11·2]), and Armenia (7·1% [2·7–15·6]); the highest estimates, however, were in Nigeria (24·2% [13·5–38·2]), Haiti (26·0% [17·4–38·9]), Botswana (27·1% [14·0–42·2]), Bangladesh (27·7% [17·8–41·2]), and Côte d’Ivoire (28·3% [13·2–49·8]; figure 2).

Those aged 20–24 years represent the youngest GBD age group following the ICVAC period of childhood (0–18 years old). Among this age group in 2023, we find that the global prevalence of SVAC was 18·6% (95% UI 16·4–23·3) among females and 13·1% (7·8–21·9) among males. The highest super-regional prevalence of SVAC was in the high-income region and sub-Saharan Africa respectively for females (23·2% [19·3–28·6]) and males (17·1% [9·9–28·0]) aged 20–24 years. Conversely, the lowest prevalence of SVAC among those aged 20–24 years was in southeast Asia, east Asia, and Oceania for both females (10·1% [6·2–16·7]) and males (11·3% [7·6–16·6]).

Within this specific age group, there was considerable variation in SVAC prevalence estimates across the 204 countries and territories included in this analysis (table 1). Solomon Islands (40·7% [21·2–63·00]), Rwanda (34·9% [22·5–49·6]), Côte d’Ivoire (34·4% [15·0–60·7]), Chile (31·1% [11·3–57·2]), and Costa Rica (29·2% [12·4–54·0]) had the highest estimated prevalence among females aged 20–24 years while Côte d’Ivoire (28·8% [13·5–50·6]), Haiti (27·2% [15·5–41·0]), Laos (24·3% [13·6–38·2]), New Zealand (22·8% [10·4–40·3]), and Uganda (22·5% [13·5–34·1]) had the highest estimated prevalence among males (appendix p 584). The lowest estimates of SVAC prevalence among females aged 20–24 years were in Montenegro (6·0% [3·8–8·6]), Romania (6·6% [3·1–12·1]), and American Samoa (7·2% [4·2–11·6]). Among males, the lowest country-level estimates were in Mongolia (4·1% [1·5–9·3]), Georgia (5·00% [2·3–9·7]), and Azerbaijan (5·9% [2·7–11·4]).

Estimates of the global prevalence of SVAC among those aged 20 to 24 years remained relatively stable over time, with estimates in 1990 (18·2% [10·9–28·4] among females and 15·7% [8·8–25·9] among males) comparable with those in 2023; however, we observed variation across countries, regions, and super-regions (appendix pp 24–583). For example, in Latin America and the Caribbean, prevalence among females increased from 1990 (16·3% [9·0–26·7]) to 2023 (17·6% [14·3–22·5]). Conversely, in southeast Asia, east Asia, and Oceania, the prevalence of SVAC among males aged 20–24 years decreased from 18·7% (11·4–28·4) in 1990 to 11·3% (7·6–16·6) in 2023.

Among the 47 DHS and 16 VACS included in the age at first exposure analysis, a total of 16 786 females aged 13–24 years reported having ever experienced sexual violence in their lifetime and also recalled their age at first exposure (table 2). Across all surveyed countries, we found that 7·7% of experiences of sexual violence against young females had first occurred before age 12, 41·6% before age 16 years, and 67·3% by age 18 years. The distribution of age at first experience varied by country and region for which underlying data were available: in Latin America and the Caribbean and sub-Saharan Africa, 72·9% and 66·8% of first events had occurred by age 18 years, respectively. By comparison, approximately 57·8% of first events had occurred by age 18 years in south Asia (table 2).

**Table 2:** Distribution percentiles of the age at first experience of sexual violence by super-region, sex, and data source among respondents aged 13–24 years

			**Percent of first sexual violence experiences which occurred before age:**			**N**
				12 years	16 years	18 years
Central Europe, eastern Europe, and central Asia						
	Female, VACS		7·4	47·2	76·1	163
	Male, VACS		1·1	33	65·9	88
Latin America and Caribbean						
	Female					
		Aggregate	15·8	50·5	72·9	2141
		VACS	22·3	63·4	83·7	1092
		DHS	9·0	37·2	61·6	1049
	Male, VACS		17·0	57·7	79·4	690
South Asia						
	Female, DHS		1·8	27·5	57·8	109
	Male		NA	NA	NA	NA
Southeast Asia, east Asia, and Oceania						
	Female					
		Aggregate	7·1	33·6	60·3	746
		VACS	10·1	37·1	65·2	89
		DHS	6·7	33·2	59·7	657
	Male, VACS		50·7	70·7	84·0	75
Sub-Saharan Africa						
	Female					
		Aggregate	6·5	40·7	66·8	13 627
		VACS	7·7	46·1	72·6	4697
		DHS	5·90	37·9	63·7	8930
	Male, VACS		12·6	44·3	69·4	2235
All data						
	Female					
		Aggregate	7·7	41·6	67·3	16 786
		VACS	10·3	49·1	74·6	6041
		DHS	6·2	37·4	63·2	10 745
	Male, VACS		14·2	47·6	71·9	3088

The table displays the distribution percentiles of experiences of sexual violence that first occurred before ages 12, 16, and 18 years, among survey respondents aged 13–24 years who had ever experienced sexual violence in their lifetime. These results draw upon surveys with less than 50% missingness in the variable denoting the age at first experience. Female data are derived from the DHS (for individuals aged 15–24 years) and VACS (for individuals aged 13–24 years), while male data are exclusively from the VACS (for individuals aged 13–24 years). In locations with both female DHS and VACS data, results were pooled to create an aggregate percentile distribution. For females, no data were available for countries within the high-income or North Africa and the Middle East super-regions. For males, no data were available for countries within the high-income, North Africa and the Middle East, or south Asia super-regions. DHS=Demographic and Health Surveys. NA=not available. VACS=Violence Against Children and Youth Surveys.

The distribution of young females’ first experiences of sexual violence did not vary substantially by data source; however, reported ages at first experience were on average younger in VACS data compared with DHS. Among all DHS data for females aged 15–24 years, a total of 10 745 women reported having ever experienced sexual violence and recalled their age at first exposure. Among these participants and across all countries of available DHS data, 6·2% had been exposed by age 12 years, 37·4% had been exposed by age 16 years, and 63·2% had been exposed by age 18 years. While VACS data for young females draw upon fewer survey modules than DHS, across the 6041 young females (aged 13–24 years) who reported having experienced sexual violence and recalled their age at first exposure in the VACS data, 10·3% had been first exposed by age 12 years, 49·1% by age 16 years, and 74·6% by age 18 years (table 2). Region-specific distributions from the two-survey series showed similar geographical patterns. Across the three world regions for which both survey series had available data, the percentage of young females exposed by age 18 years was comparatively high for both sources in Latin America and the Caribbean (83·7% for VACS and 61·6% for DHS) and sub-Saharan Africa (72·6% for VACS and 63·7% for DHS) and was the lowest in southeast Asia, east Asia, and Oceania (65·2% for VACS and 59·7% for DHS; table 2). Neither survey series had data available for countries within the high-income or north Africa and the Middle East super-regions.

For young males, only VACS data contained information on age at first experience. Across all available surveys (n=15), 3088 young males aged 13–24 years reported having ever experienced sexual violence in their lifetime and also recalled their age at first exposure (table 2). Distributions were similar to those computed for females, with 14·2% of male respondents having been exposed by age 12 years, 47·6% exposed by age 16 years, and 71·9% exposed by age 18 years. The percentage of first events occurring by age 18 for young males was highest in southeast Asia, east Asia, and Oceania (84·0%) and lowest in central Europe, eastern Europe, and central Asia (65·9%); no data were available for countries within the high-income, north Africa and the Middle East, or south Asia super-regions.

## Discussion

To the best of our knowledge, this is the first study to comprehensively present prevalence estimates for SVAC for all locations, ages, sexes, and years while also accounting for known patterns of differential disclosure of SVAC. After synthesising 460 sources, we find that the prevalence of SVAC is far above the SDG's zero target goals for both females and males. Globally, our age-standardised estimates of adults aged 20 years and older showed SVAC prevalence of 18·9% (95% UI 16·0–25·2) among females and 14·8% (9·5–23·5) among males. We estimate that 18·6% (95% UI 16·4–23·3) of females and 13·1% (7·8–21·9) of males aged 20–24 years were survivors of SVAC, a finding which highlights the pervasiveness of SVAC even in recent generations. Lastly, using retrospective survey data, we find that among females and males aged 13–24 years who reported ever experiencing sexual violence, 67·3% and 71·9%, respectively, had first been exposed by age 18 years.

We observed notable geographical differences in SVAC prevalence in both our input dataset and model estimates. Age-standardised estimates of SVAC prevalence were highest in south Asia (within-region range of 9·3% [6·6–13·7] in Bangladesh to 30·8% [25·0–37·5] in India) for females and sub-Saharan Africa (within-region range of 7·9% [3·5–15·8] in Zimbabwe to 28·3% [13·2–49·8] in Côte d’Ivoire) for males, while estimates were lowest in southeast Asia, east Asia, and Oceania (within-region range of 7·1% [4·8–10·8] in Viet Nam to 42·6% [34·4–52·1] in Solomon Islands) for females and central Europe, eastern Europe, and central Asia (within-region range of 4·2% [1·7–9·2] in Mongolia to 21·0% [9·2–39·7] in Bosnia and Herzegovina) for males. These results are consistent with previous SVAC meta-analyses,^15^, ^16^, ^17^ which have also shown substantial geographical differences in estimated prevalence, albeit using different world region categories. However, across these previous analyses as well as our own, it is difficult to disentangle whether measured differences are due to true differences in prevalence or are instead driven by differential disclosure. Notably, our results are slightly higher than recent publications,^18^ which is most likely due to our differential disclosure adjustment, which found that estimates relying exclusively on face-to-face interviews probably underestimate the true prevalence of SVAC (appendix pp 9–11). To the best of our knowledge, our analysis is the first to systematically account for this factor.

A variety of laws and protections exist for children across the globe, but the extent to which such policies actively lower rates of SVAC has not yet been characterised on a global or internationally comparable scale. Along the same lines, certain cultural views or practices might impact prevalence of SVAC, including levels of gender equality, conceptualisation of masculinity, socioeconomic conditions, isolation of young people, and more.^34^ These same forces—as well as legal and social protections available to survivors—might underlie population-based differences in perpetration as well as willingness to disclose or discuss experiences of violence. Indeed, our study's analysis of differential reporting showed varying rates of disclosure by survey mode alone, indicating that variations in survey administration might be an important factor underlying observed differences in SVAC prevalence. Disclosure of exposure to violence is also likely to differ by other factors.^28^ For example, our analysis of VACS data showed that male respondents were more likely to have a missing response to a question surrounding the age at which they had first experienced violence compared to female respondents. Future work should build upon this finding and explore how rates of reporting vary by age, sex, and geography. Those leading future data collection efforts should also carefully consider their measurement instruments and location of data collection to encourage accurate reporting while prioritising the safety and confidentiality of respondents. Greater consistency and the implementation of best practices in all SVAC measurement across countries can improve our ability to draw true cross-national comparisons, in turn improving our ability to identify and address key drivers of SVAC.

Our age at first experience of sexual violence analysis showed that sexual violence often first occurs during childhood and adolescence. Among 16 786 females aged 13–24 years who had ever experienced sexual violence, 7·7% first experienced violence before age 12 years, increasing to 41·6% before age 16 years, and 67·3% before age 18 years. Among 3088 males aged 13–24 years who reported ever having experienced sexual violence, 14·2% first experienced this violence before age 12 years, 47·6% before age 16 years, and 71·9% before age 18 years. These findings highlight that childhood and adolescence are key intervention windows to prevent SVAC before it ever occurs. Although the survey instruments used for our age at first experience analyses did not provide information on repeated exposure to childhood violence into later adolescence or beyond, females exposed to violence in childhood have been found to be at an elevated risk of revictimisation throughout adolescence and adulthood, and male experiences of SVAC have been linked to future violence perpetration, mechanisms which perpetuate cycles of violence.^9^, ^10^ As outlined by the Safe Futures Hub,^34^ the life-long and intergenerational consequences of SVAC can be avoided through programme implementation, legislative action, and policy development that engage young people early to prevent violence from ever occurring. Such interventions might take the form of school-based programmes that teach children about safe relationships and laws surrounding sexual harassment and create structural changes such as increasing security presence in violence-prone hot spots identified by students.^35^ For example, Safe to Learn is a school-based global initiative which seeks to ensure safe learning environments by embedding violence prevention efforts in school curricula and policies. Programmes that shift focus from victimisation to perpetration are also needed to challenge and change the norms and behaviours that enable SVAC and other forms of violence against children.^36^ Programmes that work with men and boys to combat stereotypes of toxic masculinity have been rated as promising or effective in preventing SVAC and teen dating violence,^37^, ^38^ and interventions which teach adolescents about appropriate behaviour with peers and other young children also have promise in improving youth knowledge and behavioural intentions.^35^ It is important to note that very little is known about the global prevalence of SVAC perpetration, and this data are needed to fully evaluate the growing number of programmes focused on perpetrators. Future SVAC survey efforts should therefore also examine perpetration behaviour and patterns among respondents.

In addition to bolstering systems-based responses, we must also invest in routine data collection. Although we compiled and used all available sources in global repositories, substantial data gaps still exist: 63 countries and territories have no publicly available data based on our review of three global databases (appendix pp 587–635). Notably, north Africa and the Middle East has zero surveys for males and five for females. Even among locations with data, routine or repeated surveys were scarce. Only 39·7% of locations with SVAC data for females and 55·8% for males had 1 year of eligible data, which hinders our ability to effectively track progress over time. Given the high prevalence of SVAC across the globe, particularly in locations where we have survey data (eg, India, South Africa, and the USA), efforts to measure and monitor prevalence in data sparse locations are urgently needed in order to better understand the patterns of SVAC, to highlight it as a priority for policy makers, and to track progress towards eliminating sexual violence against children.

Future data collection and surveillance programs must also use rigorous and inclusive survey instruments to measure all experiences that constitute SVAC, capturing victim–perpetrator relationships, specific acts and force types, and more. For example, many of the survey instruments identified in our analysis did not explicitly measure certain unwanted behaviours like pressured or coercive sex or sexual acts. Instead, surveys often asked respondents more generally about forced or unwanted sexual behaviours, which relies upon respondents’ interpretations of what might constitute unwanted sexual contacts. This lack of clarity means that the included or excluded behaviours are specific to each survey or data source, which hinders comparability. To address this limitation, we estimated adjustment factors based upon within-study comparisons of different case definitions of SVAC. This analysis showed that the specific acts and ages included in SVAC case definitions yield very different estimates of prevalence. Future surveys should therefore employ best practices and adapt a standardised and comprehensive set of definitions to capture the true prevalence of SVAC, enhance comparability of estimates, and minimise the adjustments needed to synthesise evidence.

Our findings should be interpreted within the study's limitations. First, our modelling processes borrow strength from other nearby geographies and world regions with data, which allows us to create estimates in locations without data. However, estimates in locations without directly observed input data rely on data similar in space, time, and age as well as model parameters and should be interpreted within these constraints. Comparisons between female and male SVAC prevalence must also be interpreted considering said data limitations, as there are systematic data gaps which limit our understanding of sexual violence exposure among boys, transgender, and gender-diverse populations. We find that the age-standardised global prevalence of SVAC was on average higher among females compared to males in 2023 (18·9% [95% UI 16·0–25·2] *vs* 14·8% [9·5–23·5]), a pattern which is consistent with previous studies.^15^, ^17^, ^39^ However, in our analyses, of the 77 locations with data for both females and males, 68·8% of them had more data available for female populations, reflecting a substantial compositional bias in the SVAC data landscape. In addition, in our analysis of the ages at which individuals are first exposed to sexual violence, we found a significant association between respondent sex and missing responses, a result which aligns with other studies that have shown males may be more likely to under-report SVAC.^28^ Indeed, high-quality surveys from Asia and the Pacific and several Balkan countries have indicated that SVAC prevalence among boys is comparable with or higher than that among girls.^40^, ^41^ In addition, other studies have shown that transgender and gender non-conforming people are at an elevated risk for child abuse; however, gender identity is often missing in population-based surveys due to privacy, safety, and confidentiality concerns; stigma; political resistance; criminalisation of transgender and non-binary people; and more.^42^, ^43^ It is imperative that future population-based data collection efforts be representative and inclusive of all persons by expanding samples to include all sexes and genders and by including questions on sexual orientation and gender identity. These efforts and subsequent analyses can improve our understanding of the true prevalence and nature of SVAC and guide interventions that reach all young people.

Second, we present results following the regional groupings of the GBD study, which classifies countries by epidemiological and geographical proximity. However, we observed considerable variation of SVAC prevalence within regions (eg, prevalence ranges from 8·8–31·1% among females aged 20–24 years in high-income locations). Many drivers of SVAC are likely not to be associated with geography itself but are instead closely linked to socioeconomic and cultural contexts, which vary widely within world regions and even within countries. Thus, summary results provided at the global and regional level should be interpreted in light of the variation observed across countries. This is particularly true for data-sparse regions (eg, north Africa and Middle East), as estimates for this entire region draw upon only five sources for females and no sources for males, limiting our ability to examine or characterise within-region variation of estimates. It is also true when examining regional or global trends over time, as aggregate values like these might mask progress made within specific locations or societies. Renewed efforts to collect high quality, comparable survey data in under-represented and unrepresented locations can improve our ability to identify true geographical or regional patterns in SVAC prevalence moving forward. These efforts should additionally seek to collect information on the potential risk and protective factors for SVAC to inform prevention and support efforts tailored to local contexts.

Third, our cohort extrapolation process assumes that SVAC prevalence remains stable over time as survivors age (appendix pp 11–12), although it is known that experiencing SVAC is a risk factor for future health issues^3^ that can lead to premature death. Although these negative effects could be partly mitigated via individual resiliency and access to support services, premature mortality of any amount among adult survivors of SVAC would result in decreased prevalence estimates of SVAC among the same population over time. There is limited longitudinal data describing the potential for differential age-specific mortality among survivors of SVAC in adulthood, hindering our ability to accurately account for this in our cohort extrapolation step (ie, modelling an attenuation of SVAC prevalence as a cohort ages over time). Our analysis of contiguous data from Türkiye (appendix p 11) suggests that prevalence remains relatively stable over time for the same cohort of individuals; however, in the absence of additional available data, we have inflated uncertainty around our extrapolated data points so that they will have less weight in the final model than directly measured observations. The use of cohort extrapolation methods greatly increased the data available to our model, a benefit which we believe outweighs any bias our cohort assumption may introduce, particularly when considering that other metrics impacted by differential mortality (eg, educational attainment) have shown comparatively small effects at the population level over time.^29^ In spite of this, the adverse long-term health effects associated with SVAC necessitate further cohort and longitudinal analyses that assess the extent to which SVAC is associated with premature mortality over time. Similarly, this cohort extrapolation process might artificially smooth trends over time, masking progress that has been made in recent generations to prevent and reduce rates of SVAC, particularly in locations with little to no data, and especially when aggregating results to regional and global trends. Repeated surveys are necessary to better understand how SVAC prevalence changes over time.

Fourth, following ICVAC standards, we defined SVAC as sexual violence experienced before the age of 18 years. However, our models produced estimates in 5-year age bins that are consistent with the GBD study, whereby the 15–19 years modelled age group spans both childhood and adulthood (eg, those aged 18 years and 19 years). We were therefore unable to investigate or present prevalence among individuals aged 18 years and 19 years specifically, who represent the population immediately following childhood.

Lastly, it is important to consider that retrospective reports of SVAC are affected by recall and other reporting biases, which might have differential impacts based upon time passed since a sexual violence experience or background characteristics of the respondent. For example, older individuals might be less likely to remember or accurately report sexual violence events that occurred in childhood or adolescence, which could contribute to prevalence estimates that decrease as the age of respondents increases. Indeed, our analysis of ages at first experience of sexual violence reported in the DHS showed a positive correlation between survey respondents’ age at the time of the survey and reported age at first experience of sexual violence. This trend might indicate that older respondents are less likely to recall earlier sexual violence events and is additionally supported by the age pattern modelled in our prevalence analysis: surveys reporting age-specific SVAC estimates usually estimate a higher prevalence among younger age groups than older ones. However, it is also possible that younger respondents might be less likely to report or recognise SVAC if they have had less time to process their experience. Others might be completely unable to recall or remember their trauma due to dissociative amnesia, which can yield inaccurate reports or cases of non-disclosure.^44^, ^45^ Both our prevalence and age at first experience of sexual violence analyses rely on information from adults recalling their experiences while in childhood and should therefore be interpreted with these underlying factors in mind. Outstanding questions surrounding how age impacts recall of traumatic experiences additionally underscore the importance of conducting age-specific analyses for SVAC, as age-aggregated survey data and analyses may mask age-based differences in SVAC prevalence and reporting behaviours. We therefore recommend that surveys reporting on SVAC prevalence do so with age granularity (eg, reporting cases and estimates by age group of survey respondent) and that future modelling efforts present age-specific estimates and trends as well.

The protection of children from violence is a fundamental human right enshrined in the Convention on the Rights of the Child, the Sustainable Development Goals, and other international treaties and standards.^11^, ^12^, ^13^ Despite these protections, SVAC is still highly prevalent, permeating the lives of children globally, across all locations, socioeconomic status, age, and sex. This analysis extends and adds to other international efforts to monitor violence against children, which have previously been limited to analyses focused on specific countries,^46^, ^47^, ^48^ regions,^49^ and populations.^50^, ^51^ In addition to developing routine surveillance, health systems and societies need to put more services and systems in place to support survivors of SVAC for the rest of their lives, including increased investments in systems-based responses and secondary prevention efforts. Our findings are an exhortation for governmental and non-governmental organisations, and indeed society at large, to urgently and significantly commit more resources to supporting survivors and to creating childhoods free of sexual violence.

### Contributors

### Data sharing

To download the results of this analysis, please visit the Global Health Data Exchange website at https://ghdx.healthdata.org/record/ihme-data/global-svac-prevalence-1990-2023. The code used for generating these analyses is available at https://github.com/ihmeuw/.

## Declaration of interests

We declare no competing interests**.**
