# Supplementary material for: Prevalence of sexual violence against children and age at first exposure: a global analysis by location, age, and sex (1990–2023)
Source: Lancet. 2025 May 24;405(10492):1817–36. doi: 10.1016/S0140-6736(25)00311-3 (PMC12100463; doi:10.1016/S0140-6736(25)00311-3)
Supplement: Supplementary appendix [file mmc1.pdf]

THE LANCET

Supplementary appendix

This appendix formed part of the original submission and has been peer reviewed. We post it as supplied by the authors.

Supplement to: Cagney J, Spencer C, Flor L, et al. Prevalence of sexual violence against children and age at first exposure: a global analysis by location, age, and sex (1990–2023). *Lancet* 2025; published online May 7. https://doi.org/10.1016/S0140-6736(25)00311-3.

Supplementary Information: Data Sources and Supplementary Results for “Prevalence of sexual violence against children and age at first exposure: a global analysis by location, age and sex (1990-2023)”

This appendix provides information on input data sources as well as further methodological detail, supplemental figures, and more detailed results. The appendix is organised into broad sections following the structure of the main paper.

Table of Contents

Section 1. GATHER checklist ... 3

Section 2. Geographies estimated ... 5

Section 3. Age-standardization ... 5

Supplementary Table S1. Age group weights for age-standardisation ... 5

Section 4. Input data ... 5

Section 4.1 Data identification ... 5

Section 4.1 Inclusion and exclusion criteria ... 6

Section 4.2 Exposure case definitions ... 6

Reference definition ... 6

Alternative definitions ... 7

Section 4.3 Data extraction ... 7

Microdata Sources ... 7

Tabulated sources ... 7

Supplementary Table S2. Tabulated data extraction template ... 7

Section 5. Supplementary methods ... 8

Section 5.1 Crosswalking and alternative definition adjustments ... 9

Supplementary Table S3: MR-BRT crosswalk adjustment factors for SVAC exposure ... 9

Section 5.2 Differential reporting adjustment ... 9

Figure S5.2.1: Response card from the WHO Multi-country Study on Women’s Health and Domestic Violence against Women ... 10

Section 5.3 Age splitting ... 11

Section 5.4 Data extrapolation via cohort imputation ... 11

Section 5.5 STGPR ... 12

Section 5.6 Exponential smoothing ... 13

Section 6. Age at first experience of sexual violence 13
Section 6.1 Data inputs, locations, and time periods of analysis 13
Figure S6.1.1A: DHS data coverage map 14
Figure S6.1.1B: VACS data coverage map 14
Supplementary Table S4: Demographic and sample information by DHS and VACS module. 15
Supplementary Table S5: DHS questions used to identify respondents who had ever experienced sexual violence and age at which it first occurred. 18
Supplementary Table S6: VACS questions used to identify respondents who had ever experienced sexual violence and age at which it first occurred. 18
Section 6.2 Supplemental results 20
Supplementary Table S7: Percent of first sexual violence experiences which occurred prior to ages 12, 16, and 18 computed using DHS data from 15–49-year-olds. 21
Supplementary Table S8: Associations between missing responses in the age at first exposure to sexual violence variable and respondent age, sex, education, and urbanicity. 22
Supplementary Table S9: Percent of first sexual violence experiences which occurred prior to ages 12, 16, and 18 computed using all DHS data (regardless of variable missingness levels) for 13-24-year-olds. 23
Section 7. Supplementary prevalence results 24
Section 7.1 Summary prevalence tables 24
Section 7.2 Additional prevalence figures 584
Section 7.3 Trends in SVAC prevalence 585
Section 8. Data Sources 587
Supplementary Table S12. Data sources included in the prevalence analysis 587
Supplementary Table S13: DHS and VACS modules included in the age at first sexual violence analysis 636

Section 1. GATHER checklist

Item #	Checklist item	Reporting location
**Objectives and funding**		
1	Define the indicator(s), populations (including age, sex, and geographic entities), and time period(s) for which estimates were made.	Main text: Methods (definitions and data sources)<br>Appendix: Section 2 (Geographies estimated, p 5); Section 4 (Input data, pp 5–8); Section 7 (Supplementary prevalence results, tables S10 and S11)
2	List the funding sources for the work.	Main text: Summary
**Data Inputs**		
For all data inputs from multiple sources that are synthesized as part of the study:		
3	Describe how the data were identified and how the data were accessed.	Main text: Methods (definitions and data sources)<br>Appendix: Section 4 (Input data, pp 5–6)
4	Specify the inclusion and exclusion criteria. Identify all ad-hoc exclusions.	Main text: Methods (definitions and data sources)<br>Appendix: Section 4 (Input data, p 6)
5	Provide information on all included data sources and their main characteristics. For each data source used, report reference information or contact name/institution, population represented, data collection method, year(s) of data collection, sex and age range, diagnostic criteria or measurement method, and sample size, as relevant.	Appendix: Section 6 (Age at first experience of sexual violence, Supplementary table S4); Section 8 (Data sources, Supplementary tables S12 and S13)
6	Identify and describe any categories of input data that have potentially important biases (e.g., based on characteristics listed in item 5).	Main text: Methods (data cleaning and adjustments); Discussion
For data inputs that contribute to the analysis but were not synthesized as part of the study:		
7	Describe and give sources for any other data inputs.	Not applicable
For all data inputs:		
8	Provide all data inputs in a file format from which data can be efficiently extracted (e.g., a spreadsheet rather than a PDF), including all relevant meta-data listed in item 5. For any data inputs that cannot be shared because of ethical or legal reasons, such as third-party ownership, provide a contact name or the name of the institution that retains the right to the data.	Section 8 (Data sources, Supplementary tables S12 and S13)
**Data analysis**		
9	Provide a conceptual overview of the data analysis method. A diagram may be helpful.	Main text: Methods

10	Provide a detailed description of all steps of the analysis, including mathematical formulae. This description should cover, as relevant, data cleaning, data pre-processing, data adjustments and weighting of data sources, and mathematical or statistical model(s).	Main text: Methods<br>Appendix: Section 5 (Supplementary methods, pp 8–13); Section 6 (Age at first experience of sexual violence, pp 13; 20–21)
11	Describe how candidate models were evaluated and how the final model(s) were selected.	Appendix: Section 5 (Supplementary methods, pp 11–13)
12	Provide the results of an evaluation of model performance, if done, as well as the results of any relevant sensitivity analysis.	Appendix: Section 6 (Age at first experience of sexual violence, pp 15–17; 20–23)
13	Describe methods for calculating uncertainty of the estimates. State which sources of uncertainty were, and were not, accounted for in the uncertainty analysis.	Main text: Methods
14	State how analytic or statistical source code used to generate estimates can be accessed.	https://github.com/ihme
Results and Discussion		
15	Provide published estimates in a file format from which data can be efficiently extracted.	https://ghdx.healthdata.org/record/ihme-data/global-svac-prevalence-1990-2023
16	Report a quantitative measure of the uncertainty of the estimates (e.g. uncertainty intervals).	Main text: Results<br>Appendix: Section 7 (Supplementary prevalence results, tables S10 and S11)
17	Interpret results in light of existing evidence. If updating a previous set of estimates, describe the reasons for changes in estimates.	Main test: Discussion
18	Discuss limitations of the estimates. Include a discussion of any modelling assumptions or data limitations that affect interpretation of the estimates.	Main test: Discussion

Section 2. Geographies estimated

We estimated the prevalence of sexual violence against children (SVAC) from 1990-2023 in 204 countries and territories, 21 regions, and 7 super regions by sex and five-year age group (Main Table 1 and Supplementary Tables S10 and S11). We additionally calculated aggregate global estimates by age and sex.

Section 3. Age-standardization

Age-standardised results for females and males aged 20+ were calculated at the 1000 draw level by multiplying each draw in an age group by a set age weight. Shown in Supplementary Table S1, the age weight for each age group is normalised to the population aged 20+ so that the weights add up to 1 using the world population age standard as defined by the GBD. Additional details on the GBD world population standard can be found in the GBD 2021 Demographics Capstone.

Supplementary Table S1. Age group weights for age-standardisation

Age Group	Weight
20 to 24	0.123795
25 to 29	0.120466
30 to 34	0.116186
35 to 39	0.108352
40 to 44	0.09755
45 to 49	0.087458
50 to 54	0.077965
55 to 59	0.068963
60 to 64	0.058432
65 to 69	0.047369
70 to 74	0.035947
75 to 79	0.025351
80 to 84	0.017413
85 to 89	0.009593
90 to 94	0.003914
95 plus	0.001246

Section 4. Input data

Section 4.1 Data identification

Data sources were identified through the Global Health Data Exchange (GHDx), the GBD Collaborator Network, the World Health Organization (WHO) Global Database on the Prevalence of Violence against

Women, and the United Nations Entity for Gender Equality and the Empowerment of Women (UN Women) Global Database on Violence against Women.

The GHDx is a catalogue of datasets from nearly every country in the world. The most common types of data include demographic and health surveys, censuses, disease registries and other epidemiological surveillance systems, statistical yearbooks, and scientific literature. These sources have been identified through systematic reviews, expert knowledge, and targeted data seeking efforts by the Institute for Health Metrics and Evaluation and its individual and institutional collaborators. Currently, the GBD Collaborator network includes over 13,000 individuals in 163 countries who actively contribute to the data that is considered in this study as well as the GBD study overall. All data sources related to sexual or interpersonal violence against children were reviewed, and sources that met inclusion criteria were extracted to be used in the main prevalence analysis.

We additionally reviewed data sources listed in the WHO's Global Database on Prevalence of Violence Against Women, which includes representative prevalence studies of sexual violence by any perpetrator. This database was created via a systematic review of six electronic databases by the WHO, the London School of Hygiene and Tropical Medicine, and the South Africa Medical Research Council. The WHO database also includes sources identified by manual searches for reports and surveys published and/or administered by local, national, and international governments and agencies (e.g., national violence studies, Demographic and Health Surveys).

Lastly, we cross-referenced both the GHDx and the WHO Database with UN Women's Global Database on Violence against Women, which contains relevant surveillance systems, reports, surveys, laws, and legislations submitted by United Nations Member States.

Section 4.1 Inclusion and exclusion criteria

We included prevalence data from individual-level microdata and sex-disaggregated survey or scientific report tabulations. Sources were included if they (1) were representative of a GBD national or subnational location; (2) provided self-reported data on SVAC prevalence among individuals aged 10 years and older between 1980 and 2023; and (3) used an accepted definition of SVAC, described below. Data from Child Protective Services, crime reports, clinical informatics, and other administrative records were excluded due to high levels of underreporting and the geographic variability in comprehensiveness. In total, we identified 451 data sources for females and 195 data sources for males. A complete list of sources for the prevalence analysis is available in Supplementary Table S12.

Section 4.2 Exposure case definitions

Reference definition

We defined SVAC as having ever experienced intercourse or other contact abuse (i.e., fondling and other sexual touching) before the age of 18, in which the contact was unwanted (e.g., forced or coerced).

Alternative definitions

We incorporated exposure data sources identified through the aforementioned data repositories and shared with us by collaborators. We included all sources that provided population-representative data on the proportion of females or males who experienced sexual violence before the age of 18. In addition, we accepted sources reporting on the following non-reference definitions and populations:

1. Proportion of individuals who experienced intercourse-only SVAC
2. Proportion of individuals who experienced contact or non-contact SVAC
3. Proportion of individuals who experienced SVAC by a specific or restricted perpetrator (e.g., sexual violence committed by a caregiver)
4. Proportion of individuals whose first sexual experience or debut was violent (e.g., before age 18 and forced)
5. Proportion of individuals who experienced sexual violence before some age less than 18 (such as before age 12 or 16)
6. Proportion of individuals who experienced SVAC, measured from a student population

Case definitions using alternative violence types and age limits to define SVAC were accounted for with adjustment factors based upon meta-regressions described below in Section 5.1.

Section 4.3 Data extraction

Microdata Sources

Among the surveys for which we had access to microdata, we extracted relevant demographic information, including age, sex, location, and year of data collection, as well as survey metadata, including survey weights, primary sampling units, and strata. Together, this information was used to extract and calculate weighted prevalence estimates by five-year age bin, sex, and location.

Tabulated sources

To facilitate data cleaning and processing, the following metadata was extracted from each source of tabulated data included in this analysis:

Supplementary Table S2. Tabulated data extraction template

Field		Description
**Years**	**Year Start**	Year that the study or data collection began
	**Year End**	Year the study or data collection ended
**Location**	**Location Name**	The national or sub-national location where the study took place
	**Location ID**	The unique location identification number corresponding to the national or sub national location of the study
	**Location Representativeness**	Geographical representativeness of the study (e.g., nationally representative, subnationally representative)
	**Urbanicity**	Urbanicity of where the study took place
**Study Design**		Study design (e.g., cross-sectional survey)

**Participant Characteristics**	**Sex**	Sex of the participants for the given data point
	**Age Start**	Lowest age of the participants for the given data point
	**Age End**	Highest age of the participants for the given data point
	**Age at Abuse Type**	Selected from the following options:<br>before; since
	**Age at Abuse Value**	Numeric age threshold of abuse
**Study Case Information**	**Case Definition**	The exact or paraphrased definition or type of abuse (e.g., any contact or non-contact SVAC) as given by the study
	**Case Diagnostics**	Diagnostics used to define the type of abuse, which include the exact question(s) or definition(s) used
**Violence Type**	**GBV Indicator**	Broad GBV indicator from the following options:<br>sexual violence; physical violence; psychological violence; economic violence; physical and/or sexual violence; all violence (physical, sexual, psychological); all violence (physical, sexual, psychological, economic)
	**Sexual Violence Type**	Type of sexual violence from the following options:<br>any sexual violence; intercourse only; non-intercourse sexual violence; non-contact sexual violence (e.g. pose naked); any contact sexual violence
	**Force Type**	Force type from the following options:<br>any force type including attempts; any force type excluding attempts; physically forced only including attempts; physically forced only excluding attempts; pressured/coerced; attempts only
**Perpetrator Information**	**Perpetrator Identity**	Identity of the perpetrator from the following options:<br>all (not specified); any partner (current or previous); current partner; previous partner (partner at the time); ex-partner at the time; family member; stranger; relative; caregiver; any adult; known person; non-partner
	**Perpetrator Age**	Age of the perpetrator from the following options:<br>same age; older; younger; 5+ years older; <5 years older
	**Perpetrator Sex**	Sex of the perpetrator from the following options:<br>all (not specified); male; female

Section 5. Supplementary methods

Lifetime exposure to SVAC was estimated using a spatiotemporal Gaussian process regression (ST-GPR), further described in section 5.5. Input data were prepared by adjusting data with alternate case definitions (section 5.1) and alternative survey modes (section 5.2) and by splitting data into aggregate age groups by applying modelled reference age patterns, as described in section 5.3.

Section 5.1 Crosswalking and alternative definition adjustments

For alternate case definitions of SVAC, we used data from the CDC Violence against Children and Youth Surveys to run a logit-difference meta-regression with the MR-BRT tool to estimate correction factors (Supplementary Table S3). Our models were fit using 10% trimming and 2 priors, (1) contact only (reference) definitions should be less than definitions including contact and/or non-contact cases, and (2) intercourse only definitions be less than contact only (reference) definitions.

Supplementary Table S3: MR-BRT crosswalk adjustment factors for SVAC exposure

**Data input**	**Reference or alternative case definition**	**Gamma**	**Beta coefficient, logit (SD)***	**Adjustment factor****
Contact SVAC	Ref	0.0134	—	—
Contact or non-contact SVAC	Alt		0.4343 (0.0230)	1.5439
Intercourse only SVAC	Alt		-0.4263 (0.0144)	0.6529
SVAC before 16	Alt		-0.4551 (0.0116)	0.6344
SVAC before 12	Alt		-1.2791 (0.0134)	0.2783

**MR-BRT crosswalk adjustments can be interpreted as the factor the alternative case definition is adjusted by to reflect what it would have been had it been measured using the reference case definition. If the logit beta coefficient is negative, then the alternative is adjusted up to the reference. If the logit beta coefficient is positive, then the alternative is adjusted down to the reference.*

***The adjustment factor column is the exponentiated beta coefficient. For logit beta coefficients, this is the relative odds between the two case definitions*

Note: Due to data limitations and over-adjustment issues, we decided to not adjust data with the restricted perpetrator or sexual debut alternate definitions.

Section 5.2 Differential reporting adjustment

We also accounted for differential reporting, recognizing that some people might not directly disclose experiences of SVAC to an enumerator. To calculate this adjustment factor, we used data from the WHO Multi-country Study on Women's Health and Domestic Violence against Women and 25 national violence against women surveys, all of which measured female SVAC using face-to-face interviews and anonymous self-report cards. Briefly, respondents were given two opportunities to disclose if someone had ever touched them sexually or made them do something sexual that they did not want to do during childhood. First, respondents verbally replied to the question so that interviewers could mark the response. Later, at the end of the interview, respondents were given another, more private opportunity to reply. Using a card with a pictorial representation of yes and no (Supplementary Figure S5.2.1), respondents marked if this type of SVAC had ever or had never happened to them. The card was folded and placed in an envelope so that the response was not directly revealed to the interviewer.

Figure S5.2.1: Response card from the WHO Multi-country Study on Women's Health and Domestic Violence against Women

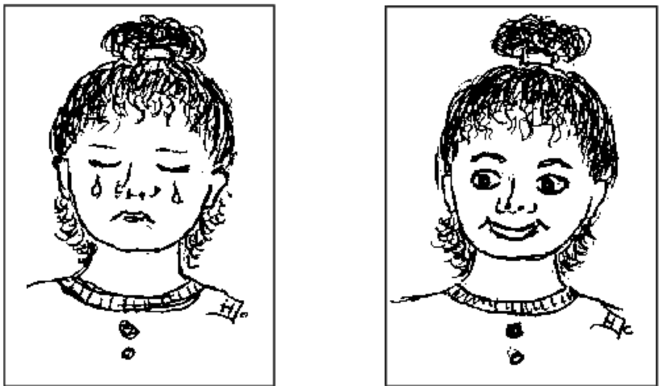

Preliminary results from the WHO Multi-country Study and its national adaptations suggest that the private self-report card was usually able to ascertain more cases of SVAC than the interview alone. Hence, a simple linear regression model was run to estimate the relationship between prevalence estimates derived from each administration method:

$$y_i = mx_i + b$$

where:

- x_i is the prevalence of SVAC derived from the traditional face-to-face interview
- y_i is the prevalence of SVAC derived from private self-report cards
- i is a unique study identifier

We estimated an intercept (b) of 0.058 and a slope (m) of 1.049, and we used these values to predict what prevalence would be in a private, self-report survey given the prevalence from a face-to-face interview. However, our face-to-face input data did not extend beyond 25%, limiting our understanding of reporting patterns in studies already reporting relatively high prevalence estimates. As such, we do not apply the linear shift (intercept) to input values equal to or great than 25% prevalence. Instead, we only use the modelled slope to relatively increase those values:

$$y_i = \begin{cases} mx_i + b, & x_i < 0.25 \\ mx_i, & x_i \geq 0.25 \end{cases}$$

where:

- x_i is the reported face-to-face prevalence of SVAC extracted from each data source
- y_i is the newly adjusted prevalence of SVAC that accounts for differential reporting
- i is a unique study identifier

For example, if a survey used a face-to-face interview and reported a 20.00% prevalence of SVAC, then we would model a new estimate of 26.78%, which would then be used in subsequent modelling steps. Similarly, if another study were to report a prevalence of 38.00%, we would adjust the published value

to be 39.85% and use that in later modelling steps. While input data were limited to female respondents, we apply the same adjustments to estimates of male SVAC. Although previous research suggests non-disclosure might be more common among males, we were unable to estimate male-specific adjustments due to a lack of data.

Section 5.3 Age splitting

Data reported in age groups broader than the GBD's standard 5-year age groups were split using the methods reported in Ng et al. For splitting aggregate data using a reference age pattern, data were first divided into sets: (1) a training dataset, containing data that already fell into 5-year age groups, and (2) a split dataset, containing data reported in aggregate age groups broader than 5-year bins. ST-GPR was used to estimate geography-time-specific age patterns using the training dataset. The ST-GPR model used an age-weight parameter value that minimized the effect of any age smoothing within the model. This parameter choice allowed the estimated age pattern to be driven by data rather than enforced by smoothing parameters of the model. Due to data sparsity within the training dataset, estimated geography-time age patterns were aggregated to the GBD region level. For female SVAC, the age pattern from the GBD world region with the most training data points (High Income North America) was used to adjust all non-standard age data. Due to extreme data sparsity within the male SVAC model, even the aggregated regional age-pattern for the GBD region with the most training data points (High Income North America) was unrealistically variable across neighbouring age groups. Therefore, countries within this region were visually examined and the most stable age pattern (Canada) was selected to adjust non-standard age data.

Section 5.4 Data extrapolation via cohort imputation

Given the case definition of SVAC, we assume that a population's prevalence of SVAC remains roughly constant after reaching 18 years of age, as any sexual violence experienced after this age would be considered violence during adulthood rather than childhood. To support said assumption, we analysed data from two national violence against women studies conducted in Türkiye in 2008 and 2014. Importantly, these surveys used the same methodologies and questionnaires, allowing us to make comparisons over time. The prevalence of female SVAC by age group is shown below, and the results demonstrate that prevalence estimates within a cohort remain relatively stable and comparable over time (note: colors indicate approximate birth cohorts).

	Türkiye	
	2008-2009	2014
15-24	**7.40% (6.5 - 8.3)**	5.0% (3.8 - 6.2)
25-34	**4.70% (4.0 - 5.4)**	**7.1% (6.0 - 8.2)**
35-44	**3.00% (2.4 - 3.6)**	**6.6% (5.5 - 7.7)**
45-59	1.70% (1.2 - 2.2)	**2.2% (1.6 - 2.8)**

Leveraging the assumption that SVAC prevalence should not vary considerably within a birth cohort after they reach age 18, we extrapolated data reported in 5-year age groups in 5-year increments. For example, the prevalence reported by a given survey for 25–29-year-olds in 2015 can also be considered as an estimate of SVAC prevalence for 20–24-year-olds in 2010 and for 30–34-year-olds in 2020. In recognition of demographic changes over time as well as SVAC's association with adverse health outcomes, both of which might influence prevalence, the uncertainty around extrapolated data points were inflated by a factor of 2, which consequently down-weights them in future modeling steps. We did not apply the same assumption or extrapolation to data points representative of 10–19-year-olds, as respondents below the age threshold detailed in the SVAC case definition are still at risk of experiencing violence. Thus, their prevalence is subject to change over time and cannot be subject to the same assumptions described above.

The adjustment factor of 2 was decided via sensitivity analyses. Using the same set of input data, we tested extrapolation inflation factors of 1 (i.e., no inflation), 2, and 4 and ran 100-draw ST-GPR models (described further in Section 5.5) to create a preliminary set of prevalence estimates by age, sex, and time. The correlation between these three sets of estimates was then calculated, with each pair-wise comparison having a correlation around 0.99, suggesting that inflating uncertainty around the input data did not impact point prevalence. Inflation factors did, however, influence model UIs, and we felt that the wider uncertainty intervals yielded by the inflation factor of 2 resulted in more conservative estimates that better account for possible demographic changes over time as well as SVAC's known association with long-term adverse health outcomes (and, in turn, possible premature mortality), both of which might impact prevalence over time.

Section 5.5 STGPR

We used ST-GPR to model lifetime SVAC prevalence. We found that modeling male and female SVAC entirely independently resulted in non-data-driven sex trends, in which priors from the male model fit in the absence of data were higher than female model fits informed by female-only data. In order to leverage data-driven sex trends, we introduced a stage one linear model that predicted trends using SVAC data from both males and females. This method allowed the global ratio of SVAC exposure between sexes to inform the priors of consequent ST-GPR models in the absence of (super) regional data. Briefly, the mean function input to GPR is a complete time series of estimates generated from the linear model described above.

The stage one linear model formula is as follows:

$$logit(p_{g,a,t}) = \beta_0 + \beta_1 S_{A[a],g,t} + \sum_{k=2}^{20} \beta_k I_{A[a]} + \alpha_s + \alpha_r + \alpha_g + \epsilon_{g,a,t}$$

Where $S_{A[a],g,t}$ is the sex of the prevalence point by specific age group A, geography g, and time t, $I_{A[a]}$ is a dummy variable indicating specific age group A that the prevalence point $p_{g,a,t}$ captures, and α_s, α_r, and α_g are super-region, region, and geography random intercepts, respectively. Random effects were used in model fitting and prediction.

Section 5.6 Exponential smoothing

Data sparsity within the SVAC models caused poor model fits over time. Thus, we introduced Holt's linear trend method (extended simple exponential smoothing) to fore- and back-cast draws from the initial ST-GPR model. Holt's linear trend method allows forecasting of data with a linear trend using a weighted average of past observations, with weights decaying exponentially as observations get older (Hyndman et al. 2018). We applied this method to location-age-specific draws from our initial ST-GPR model, with the year range of the ST-GPR draws to be used as the initial time series defined based upon location-age data availability. For location-age combinations with available data spanning more than 3 years, draws were bounded from the minimum year to the maximum year of location-age-specific data. Otherwise, draws were bounded from the minimum year to the maximum year of super-region-age-specific data. For male SVAC, there is one super-region (North Africa and the Middle East) for which we have no data. In this case, we preserved most of the ST-GPR fit by using the time range of 1990-2019 (i.e. forecasting only 2019-2023). To avoid over-forecasting for longer time periods (i.e. in locations where only very old data was available), we used a damping parameter (phi=0.9) to enforce a zero-slope linear trend over time.

Section 6. Age at first experience of sexual violence

Section 6.1 Data inputs, locations, and time periods of analysis

Data providing information on lifetime exposure to sexual violence and age at first experience of sexual violence were sourced from Demographic and Health Surveys (DHS) and Violence Against Children and Youth Surveys (VACS). Survey metadata, including survey location, citation, and time period covered are provided in Supplementary Table S13. In addition, source population demographic information, sample characteristics, and missingness in the age at first experience of sexual violence variable is provided by survey module in Supplementary Table S4. The specific survey questions used within DHS and VACS to identify respondents who had ever experienced sexual violence and the age at which they first experienced such violence are provided in Supplementary Tables S5 and S6, respectively. For VACS data, because respondents are asked about their age of first exposure for each of four separate sexual violence acts and it is possible that respondents have experienced more than one act of violence at different times, we consider the age at first exposure as the earliest age reported across each of the four acts included in the questions.

We identified available microdata from a total of 88 DHS from 52 countries and 16 VACS from 15 countries (Supplementary Figure S6.1.1).

All DHS modules sampled females 15-49, except for DHS AIS Mozambique-2015, which sampled females 15-59 years old and DHS Colombia 2009 and 2015, which sampled females 13-49 years old. All VACS modules sampled females and males aged 13-24, apart from "Swaziland National Study on Violence Against Children and Young Women 2007," which sampled only females 13-24 years old.

Figure S6.1.1A: DHS data coverage map

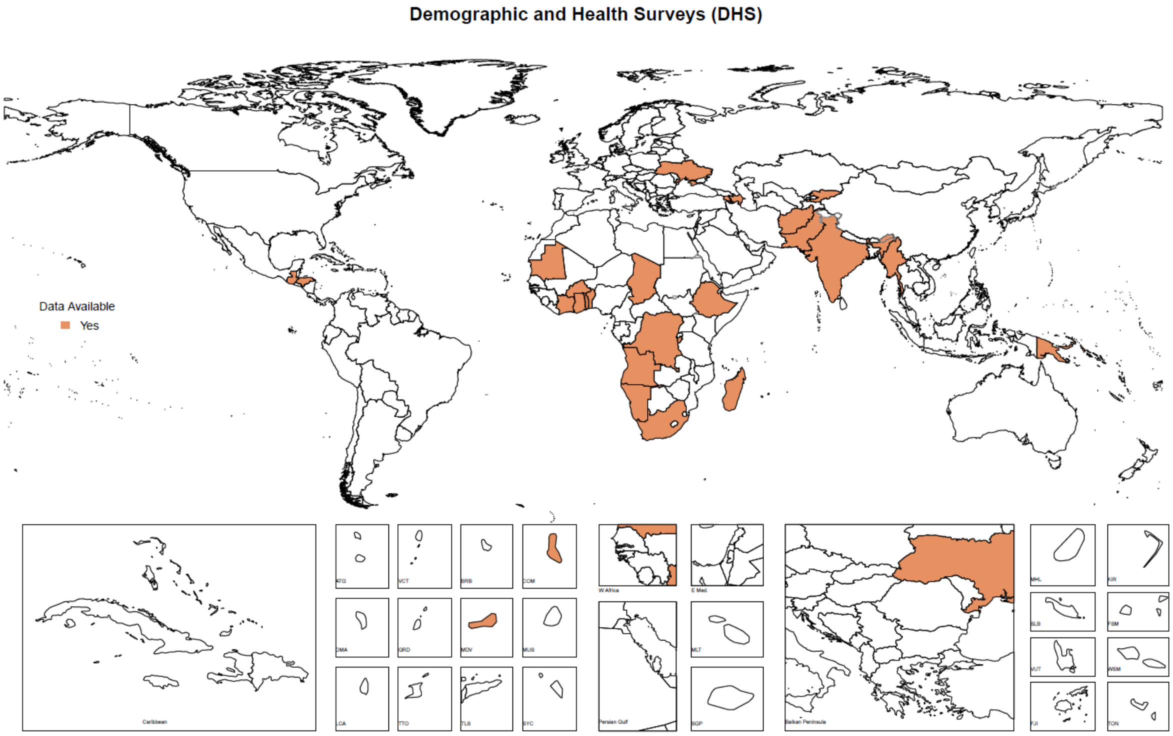

Figure S6.1.1B: VACS data coverage map

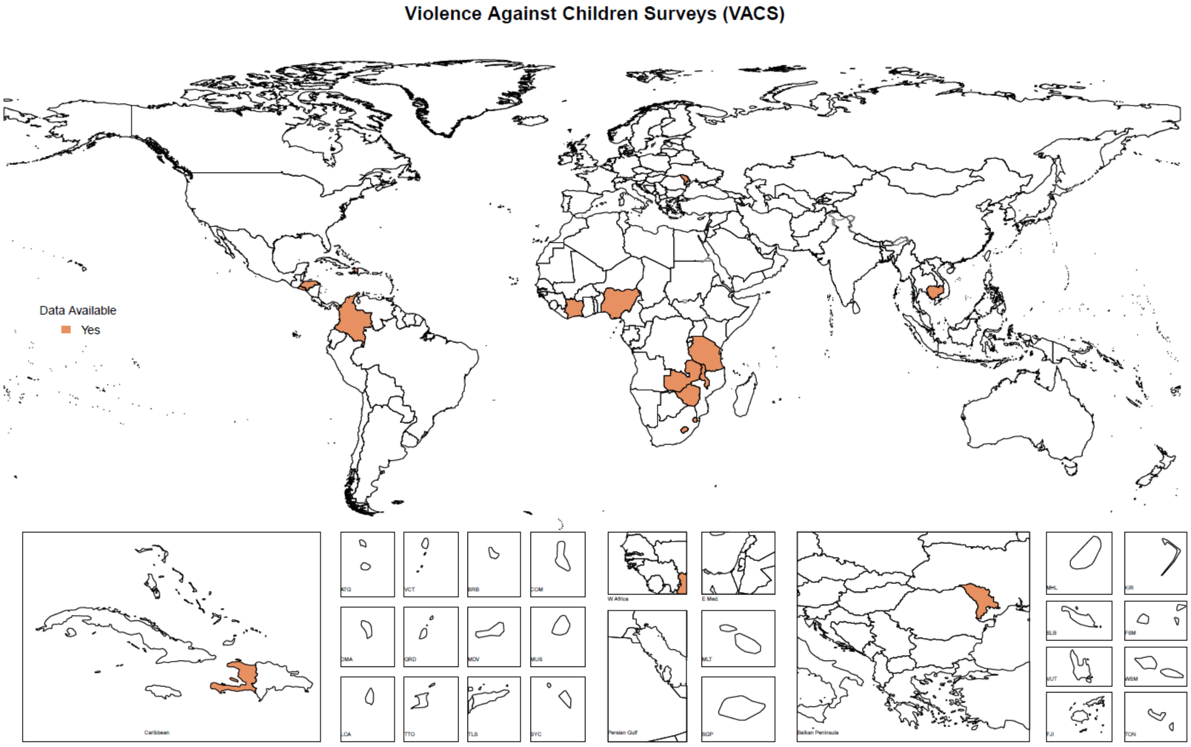

Supplementary Table S4: Demographic and sample information by DHS and VACS module.

Survey	Location	Year	Sample Sex	Sample Size	Age mean (standard deviation)	Percent of reported ages at first SV exposure missing
DHS	Azerbaijan	2006	Females	5617	32 (9.68)	95.3
DHS	Cameroon	2011	Females	5043	29 (9.2)	27.8
DHS	Dominican Republic	2007	Females	10140	30 (9.64)	60.4
DHS	Haiti	2005	Females	3568	29 (9.64)	22.7
DHS	India	2005	Females	83703	30 (8.87)	74.2
DHS	Liberia	2006	Females	4913	30 (9.18)	72.9
DHS	Uganda	2006	Females	2087	29 (8.89)	42.3
DHS	Ukraine	2007	Females	2903	34 (9.14)	69.3
DHS	Zambia	2007	Females	5236	28 (8.72)	63.5
DHS	Zimbabwe	2005	Females	6293	28 (9.03)	40.1
DHS	Ghana	2008	Females	2442	30 (9.21)	12.8
DHS	Nepal	2011	Females	4197	29 (9.12)	88.8
DHS	Timor-Leste	2009	Females	2951	30 (9.49)	72.6
DHS	Colombia	2009	Females	52952	29 (10.76)	62.4
DHS	United Republic of Tanzania	2009	Females	7047	30 (9.19)	51.6
DHS	Kenya	2008	Females	12141	29 (9.02)	47.9
DHS	Malawi	2010	Females	6229	29 (8.89)	67
DHS	Philippines	2008	Females	9316	31 (9.51)	53.2
DHS	Nigeria	2008	Females	23752	29 (9.16)	52.3
DHS	Sao Tome and Principe	2008	Females	1980	30 (9.2)	72
DHS	Mozambique	2011	Females	6835	29 (9.23)	42.2
DHS	Zimbabwe	2010	Females	6542	29 (8.86)	27.9
DHS	Uganda	2011	Females	2056	29 (8.94)	68.3
DHS	Rwanda	2010	Females	5008	29 (9.13)	38
DHS	Haiti	2012	Females	9367	29 (9.52)	16.4
DHS	Tajikistan	2012	Females	5547	30 (9.42)	95.2
DHS	Gabon	2012	Females	5557	30 (9.59)	53.6
DHS	Comoros	2012	Females	3341	28 (8.75)	36.4
DHS	Democratic Republic of the Congo	2013	Females	6811	29 (8.91)	12.1
DHS	Gambia	2013	Females	4525	28 (8.79)	55.9
DHS	Mali	2012	Females	3459	29 (8.62)	91.2
DHS	Nigeria	2013	Females	27634	29 (9.35)	15.5
DHS	Togo	2013	Females	6701	30 (8.95)	6.14
DHS	Zambia	2013	Females	11778	29 (8.85)	18.6
DHS	Kyrgyzstan	2012	Females	6022	31 (9.69)	94.9
DHS	Dominican Republic	2013	Females	6996	30 (9.64)	34.1

DHS	Honduras	2011	Females	15833	29 (9.26)	54.4
VACS	Eswatini	2007	Females	1244	18 (3.39)	0
VACS	United Republic of Tanzania	2009	Males	1771	18 (3.36)	3.14
VACS	United Republic of Tanzania	2009	Females	1968	18 (3.36)	4.99
VACS	Kenya	2010	Males	1365	18 (3.51)	1.21
VACS	Kenya	2010	Females	1151	18 (3.51)	0.532
DHS	Sierra Leone	2013	Females	5185	30 (9.23)	68.9
DHS	Philippines	2013	Females	10963	31 (9.72)	4.23
DHS	Namibia	2013	Females	2931	30 (9.41)	61.2
DHS	Afghanistan	2015	Females	21324	31 (8.46)	61.5
DHS	Cambodia	2014	Females	4307	31 (9.19)	12.7
DHS	Chad	2014	Females	4283	29 (9.04)	62.5
DHS	Guatemala	2014	Females	8595	29 (9.21)	1.48
DHS	Kenya	2014	Females	11314	30 (8.82)	8.85
DHS	Mozambique	2015	Females	3690	34 (11.46)	32
DHS	Myanmar	2015	Females	4530	32 (9.34)	12
DHS	Rwanda	2014	Females	2679	30 (8.83)	0.816
DHS	South Africa	2016	Females	8714	33 (8.79)	87
DHS	Zimbabwe	2015	Females	7223	29 (8.96)	9.23
DHS	Angola	2015	Females	10519	28 (8.92)	18.1
DHS	Armenia	2015	Females	4592	32 (8.89)	77.1
DHS	Benin	2017	Females	5408	30 (9.03)	10.8
DHS	Colombia	2015	Females	38087	29 (10.72)	63.2
DHS	Ethiopia	2016	Females	5860	29 (8.7)	15.8
DHS	Haiti	2016	Females	6321	30 (9.51)	2.47
DHS	Malawi	2015	Females	6379	28 (8.75)	43.1
DHS	Maldives	2016	Females	1189	33 (6.64)	78.9
DHS	United Republic of Tanzania	2015	Females	9322	30 (9.25)	3.2
VACS	Malawi	2013	Males	1133	18 (3.49)	2.48
VACS	Malawi	2013	Females	1029	18 (3.49)	1.48
VACS	Nigeria	2014	Males	2437	18 (3.59)	14.9
VACS	Nigeria	2014	Females	1766	18 (3.59)	2.13
VACS	Haiti	2012	Males	1459	18 (3.38)	5.32
VACS	Haiti	2012	Females	1457	18 (3.38)	8.44
VACS	Cambodia	2013	Males	1255	18 (3.57)	11.8
VACS	Cambodia	2013	Females	1121	18 (3.57)	0
DHS	Burundi	2016	Females	10188	29 (8.92)	22.5
DHS	Uganda	2016	Females	9232	29 (8.98)	20
DHS	Nepal	2016	Females	4444	30 (9.1)	0.84
DHS	Pakistan	2017	Females	4085	33 (8.18)	17.1
DHS	Timor-Leste	2016	Females	5122	30 (9.62)	50.6

DHS	Philippines	2017	Females	17968	31 (9.69)	9.47
DHS	Tajikistan	2017	Females	6353	31 (9.13)	16.4
DHS	Senegal	2017	Females	3415	29 (9.21)	41.3
DHS	Mali	2018	Females	3784	29 (8.78)	16.5
DHS	Nigeria	2018	Females	10678	30 (9.04)	13.6
VACS	Zambia	2014	Males	928	18 (3.42)	1.29
VACS	Zambia	2014	Females	891	18 (3.42)	2.46
VACS	El Salvador	2017	Males	1380	18 (3.38)	7.02
VACS	El Salvador	2017	Females	1056	18 (3.38)	4.76
VACS	Honduras	2017	Males	2659	18 (3.38)	9.49
VACS	Honduras	2017	Females	2537	18 (3.38)	3.56
DHS	Zambia	2018	Females	9503	29 (9.11)	30.1
DHS	Cameroon	2018	Females	6682	29 (9.09)	4.54
DHS	Sierra Leone	2019	Females	5248	30 (9.24)	21.1
DHS	Papua New Guinea	2017	Females	4873	30 (9.05)	44.7
DHS	Senegal	2018	Females	1957	29 (9.46)	39.5
VACS	Kenya	2018	Males	788	59 (127.54)	7.87
VACS	Kenya	2018	Females	1344	59 (127.54)	5.73
VACS	Republic of Moldova	2018	Males	978	44 (61.91)	19.8
VACS	Republic of Moldova	2018	Females	1024	44 (61.91)	8.94
VACS	Zimbabwe	2017	NA	24	40 (16.84)	0
VACS	Zimbabwe	2017	Males	5124	40 (16.84)	2.07
VACS	Zimbabwe	2017	Females	3567	40 (16.84)	2.36
DHS	Liberia	2019	Females	3120	30 (9.65)	17.1
DHS	Gambia	2019	Females	2470	29 (9.07)	6.61
DHS	Senegal	2019	Females	1865	29 (9.39)	77.6
DHS	Rwanda	2019	Females	2788	30 (9.26)	3.26
VACS	Lesotho	2018	Males	1467	44 (17.82)	2.25
VACS	Lesotho	2018	Females	7101	44 (17.82)	3.97
VACS	Colombia	2018	Males	1299	43 (24.78)	4.67
VACS	Colombia	2018	Females	1406	43 (24.78)	1.35
VACS	Côte d'Ivoire	2018	Males	1208	93 (216.64)	6.36
VACS	Côte d'Ivoire	2018	Females	1200	93 (216.64)	2.81
DHS	Mauritania	2019	Females	4184	29 (9.31)	51.7
DHS	Madagascar	2021	Females	7308	29 (9.33)	4.81
DHS	Cambodia	2021	Females	7344	31 (8.92)	89.3
DHS	Philippines	2022	Females	19228	31 (9.82)	62
DHS	Burkina Faso	2021	Females	10863	29 (9.21)	87.4
DHS	Nepal	2022	Females	5178	31 (9.31)	84.2
DHS	Kenya	2022	Females	33852	30 (9.01)	76.4
DHS	Gabon	2020	Females	4163	30 (9.4)	13.6

DHS	Côte d'Ivoire	2021	Females	5040	30 (9.03)	73.2
DHS	United Republic of Tanzania	2022	Females	5563	30 (9.47)	70.2

Supplementary Table S5: DHS questions used to identify respondents who had ever experienced sexual violence and age at which it first occurred.

**Survey Question**	**Answer Options**	**Question Sample**
Does/did your (last) husband/partner ever:<br>• Physically force you to have sexual intercourse with him even when you did not want to?<br>• Force you to perform other sexual acts you did not want to?	EVER: Yes | No | No answer<br><br>If EVER is "Yes," ask about frequency in 12 months preceding the survey:<br>Often | Sometimes | Not in past 12 months	Ever-partnered women only
Did any previous partner physically force you to have sexual intercourse or perform any other sexual acts against your will?	EVER: Yes | No | No answer<br>If EVER is "Yes," ask how long ago did this last happen:<br>0-11 months ago | more than 12 months ago	Ever-partnered women only
At any time in your life, as a child or an adult, has anyone ever forced you in any way to have sexual intercourse or perform any other sexual acts when you did not want to?	Yes | No | No answer	All (if ever-partnered, asks about non-partner)
In the last 12 months, has anyone physically forced you to have sexual intercourse when you did not want to?	Yes | No | No answer	All (if ever-partnered, asks about non-partner)
How old were you the first time you were forced to have sexual intercourse or perform any other sexual acts?	Age in completed years | No answer	Respondents who answered yes to any of sexual violence questions

Supplementary Table S6: VACS questions used to identify respondents who had ever experienced sexual violence and age at which it first occurred.

**Survey Question**	**Answer Options**	**Question Sample**

"Sexual violence – touching"<br><br>Has anyone ever touched you in a sexual way without your permission, but did not try and force you to have sex?<br><br>Touching in a sexual way without permission includes fondling, pinching, grabbing, or touching you on or around your sexual body parts.	Yes | No<br><br>Don't Know | Declined	All respondents
How old were you the first time anyone touched you in a sexual way without your permission but did not try to force you to have sex? Please give your best guess.	*Age in completed years*<br><br>Don't know | Declined	Respondents who answered "Yes" to "sexual violence – touching" question
"Sexual violence – attempted forced sex"<br><br>Has a boyfriend/romantic partner, girlfriend/romantic partner, ex-boyfriend/romantic partner, ex-girlfriend/romantic partner, husband, wife, ex-husband or ex-wife ever tried to make you have sex against your will but did not succeed?<br><br>They might have tried to physically force you to have sex or they might have tried to pressure you to have sex through harassment or threats.<br><br>Has anyone [else] ever tried to make you have sex against your will but did not succeed?<br><br>If the individual did not have an intimate partner: They might have tried to physically force you to have sex or they might have tried to pressure you to have sex through harassment or threats.	Yes | No<br><br>Don't Know | Declined	All respondents
How old were you the first time anyone tried to make you have sex against your will but did not succeed? Please give your best guess.	*Age in completed years*<br><br>Don't know | Declined	Respondents who answered "Yes" to "sexual violence – attempted forced sex" question
"Sexual violence – physically forced sex"<br><br>In the last 12 months, has anyone physically forced you to have sexual intercourse when you did not want to? Has a boyfriend/romantic partner, girlfriend/romantic partner, ex-boyfriend/romantic partner, ex-girlfriend/romantic partner, husband, wife, ex-husband or ex-wife ever physically forced you to have sex and did succeed?	Yes | No<br><br>Don't Know | Declined	All respondents

By physical force, we mean things like being pinned or held down or use of violence like pulling your hair, pushing, shoving, punching, using or threatening to use a weapon, or threatening to physically harm you or a loved one. We include experiences when you may or may not have fought back.<br><br>Has anyone [else] ever physically forced you to have sex against your will and did succeed?		
How old were you the first time anyone physically forced you to have sex? Please give your best guess.	*Age in completed years*<br><br>Don't know | Declined	Respondents who answered "Yes" to "sexual violence – physically forced sex" question
"Sexual violence – pressured sex"<br><br>Has a boyfriend/romantic partner, girlfriend/romantic partner, ex-boyfriend/romantic partner, ex-girlfriend/romantic partner, husband, wife, ex-husband or ex-wife ever pressured you in a non-physical way to have sex against your will and did succeed?<br><br>Pressured means doing things like threatening you, harassing you, telling you lies, making promises about the future they knew were untrue, threatening to end your relationship, or threatening to spread rumors about you.<br><br>Has anyone [else] ever pressured you in a non-physical way to have sex against your will and did succeed?	Yes | No<br><br>Don't Know | Declined	All respondents
How old were you the first time anyone pressured you to have sex through harassment or threats and did succeed? Please give your best guess.	*Age in completed years*<br><br>Don't know | Declined	Respondents who answered "Yes" to "sexual violence – pressured sex" question

Section 6.2 Supplemental results

DHS data spanned females aged 15-49, and we found that respondent age was moderately correlated with reported age at first experience (r = 0.331), indicating a degree of potential recall bias in this question. For this reason and to enhance comparability with the age range of VACS samples, our main analyses present DHS data for women aged 13-24 only. As a sensitivity analysis, we also computed distributions of age at first experience of sexual violence across the entire range of available DHS data (females aged 15-49) and present them here (Supplementary Table S7). As with our main analyses, these results draw upon DHS with an acceptable degree of missingness (less than or equal to 50% missing) in the age at first experience of sexual violence variable.

Supplementary Table S7: Percent of first sexual violence experiences which occurred prior to ages 12, 16, and 18 computed using DHS data from 15–49-year-olds.

**World Region**	**Percent of first sexual violence experiences which occurred before age:**			N
	**12**	**16**	**18**	
Latin America and Caribbean	6.6	25.3	40	3730
South Asia	1.5	18.2	36.8	533
Southeast Asia, East Asia, and Oceania	4.8	18.5	33.2	2915
Sub-Saharan Africa	4.5	26.9	45.2	29610
**All data**	**4.7**	**26**	**43.6**	**36788**
**Main Analysis (13–24-year-olds)**	**6.2**	**37.4**	**63.2**	**10745**

There was a high degree of missingness in the variable capturing age at first experience of sexual violence among certain DHS modules. Using all available individual-level data, we tested for associations between a missing response in the age at first experience variable and participant age, educational level, and urbanicity of their address. We used two-sample t-test for associations with age (a continuous variable) and chi-squared tests for associations with completion of primary education and urbanicity (binary variables). Results demonstrated a small but significant association between a missing response in the age at first experience variable and respondent age; respondents with a missing value in this variable were slightly older compared to respondents with a non-missing response (estimated difference: 1.95 years; 95%UI: 1.83 to 2.06, t(82955)=32.87, p-value < 0.005). Respondents with a missing response were also less likely to have completed primary education (χ^2(1, N=89299) = 168.62, p < 0.005) and more likely to live in an urban area (χ^2(1, N=89329) = 5.27, p = 0.022). These associations are presented in Supplementary Table S8. While these findings do not demonstrate significant bias, in the absence of reliable predictors to impute or replace missing age at first exposure values, we excluded DHS modules for which greater than 50% of responses for the age at first experience of sexual violence were missing for our main analysis. However, as a sensitivity analysis we computed distributions of age at first experience of sexual violence among 13–24-year-old females who had ever experienced sexual violence using all available DHS data regardless of missingness (Supplementary Table S9).

While missing responses in the age at first experience of sexual violence variable in the VACS data were comparatively lower (female modules: mean = 3.38%, range = 0 – 8.94%; male modules: mean = 6.61%, range = 1.21 – 19.8%), we also investigated potential associations between a missing response and respondent age, educational level, and sex. A missing response in the age at first experience variable was not significantly associated with respondent age (estimated difference: 0.264 years; 95%UI: -0.106 to 0.634; t(344.13) = 1.406, p-value = 0.161) nor whether a respondent had completed primary education (χ^2(1,N=5571) = 0.225, p-value = 0.635). However, males were more likely to have a missing response (χ^2(1,9569) = 47.83, p-value < 0.005). These results are shown in Supplementary Table S8.

Supplementary Table S8: Associations between missing responses in the age at first exposure to sexual violence variable and respondent age, sex, education, and urbanicity.

	**Missing response in age at first exposure variable**	**Non-missing response in age at first exposure variable**	**Significance test***	**P-value**
**DHS**				
Age (years)	32.16	30.22	t(82955)=32.87<br><br>Estimated difference:<br>1.95 years (95%UI: 1.83 to 2.06 years)	<0.0005
Education (proportion who completed primary education)	0.534	0.578	$\chi2$ (1, N=89299) = 168.62	<0.0005
Urbanicity (proportion living in an urban area)	0.403	0.400	$\chi2$ (1, N=89329) = 5.27	0.022
**VACS**				
Age (years)	19.64	19.38	t(344.13) = 1.406<br><br>Estimated difference:<br>0.264 years (95%UI: -0.106 to 0.634 years)	0.161
Education (proportion who completed primary education)	0.896	0.885	$\chi2$ (1, N=5571) = 0.225	0.635
Sex (proportion male)	0.500	0.338	$\chi2$ (1, N=9569) = 47.83	<0.0005

* We used two-sample t-test for associations with age (a continuous variable) and chi-squared tests for associations with completion of primary education, sex, and urbanicity (binary variables).

Supplementary Table S9: Percent of first sexual violence experiences which occurred prior to ages 12, 16, and 18 computed using all DHS data (regardless of variable missingness levels) for 13-24-year-olds.

**World Region**	**Percent of first sexual violence experiences which occurred before age:**			**N**
	**12**	**16**	**18**	
Central Europe, Eastern Europe, and Central Asia	5	20	42.5	40
Latin America and Caribbean	20.2	55.4	75.3	2425
North Africa and Middle East	1.4	19.6	51.7	143
South Asia	4.4	37.1	64.4	700
Southeast Asia, East Asia, and Oceania	12.7	39.6	65.4	982
Sub-Saharan Africa	7.1	40.9	66.4	11047
**All data**	**9.3**	**42.7**	**67.5**	**15337**
**Main Analysis (DHS with high levels of missingness excluded)**	**6.2**	**37.4**	**63.2**	**10745**

Section 7. Supplementary prevalence results

Section 7.1 Summary prevalence tables

Supplementary Table S10: Prevalence of female SVAC by age and location for 1990, 2000, 2010, 2020, and 2023

Location	Age Range	1990	2000	2010	2020	2023
Global	20 to 24	18.2 (10.9–28.4)	19.1 (12.5–28.5)	17.8 (12.9–24.8)	18.4 (16.0–23.3)	18.6 (16.4–23.3)
Global	25 to 29	18.4 (10.7–29.1)	19.2 (12.5–28.5)	18.9 (13.3–26.3)	19.2 (16.4–24.5)	19.6 (17.2–24.6)
Global	30 to 34	19.1 (11.0–30.4)	18.9 (12.2–28.1)	19.3 (13.7–26.9)	19.6 (16.1–25.5)	20.0 (16.9–25.7)
Global	35 to 39	19.1 (10.7–30.7)	19.6 (12.4–29.2)	20.4 (14.9–27.4)	21.6 (18.3–27.7)	21.7 (18.4–27.4)
Global	40 to 44	19.8 (10.9–32.0)	19.6 (12.2–29.7)	19.8 (14.9–26.2)	21.4 (19.1–25.3)	21.7 (19.4–25.4)
Global	45 to 49	19.7 (10.7–32.0)	18.9 (11.7–28.7)	19.2 (14.4–25.5)	19.5 (17.3–22.9)	19.8 (17.7–23.1)
Global	50 to 54	19.2 (9.7–32.7)	19.3 (11.2–30.9)	19.0 (12.4–27.9)	18.7 (13.6–27.8)	18.9 (13.5–27.8)
Global	55 to 59	18.5 (9.2–31.9)	18.6 (10.4–30.3)	17.7 (11.6–26.1)	17.8 (12.9–26.6)	17.7 (12.8–25.7)
Global	60 to 64	18.0 (8.7–31.7)	17.9 (9.5–30.1)	17.9 (11.5–26.7)	18.0 (12.8–27.5)	18.1 (12.7–27.1)
Global	65 to 69	16.6 (7.6–30.7)	16.6 (8.0–29.2)	17.0 (9.7–27.5)	16.6 (10.8–26.5)	16.6 (10.7–26.3)
Global	70 to 74	13.7 (5.9–26.9)	13.5 (6.0–25.6)	14.1 (7.6–23.9)	15.0 (8.7–25.8)	14.9 (8.6–26.1)
Global	75 to 79	12.5 (5.1–25.5)	12.6 (5.2–25.0)	13.3 (6.1–24.6)	14.1 (7.3–25.7)	14.3 (7.2–26.1)
Global	80 to 84	9.0 (3.2–19.3)	9.3 (3.5–19.6)	9.9 (4.2–19.5)	10.5 (5.1–20.6)	10.6 (5.3–20.5)
Global	85 to 89	8.1 (3.0–17.2)	8.3 (3.1–17.8)	8.8 (3.8–17.5)	9.0 (4.0–19.2)	9.0 (4.1–19.3)

Supplementary Table S10: Prevalence of female SVAC by age and location for 1990, 2000, 2010, 2020, and 2023						
**Location**	**Age Range**	**1990**	**2000**	**2010**	**2020**	**2023**
Global	90 to 94	7.7 (2.9–16.3)	8.0 (3.0–17.0)	8.3 (3.8–16.3)	8.0 (3.9–16.9)	7.9 (3.9–17.0)
Global	95 plus	7.9 (3.1–16.3)	8.3 (3.2–17.3)	8.6 (4.1–16.5)	8.2 (4.4–16.6)	8.0 (4.2–16.7)
Global	Age-standardized	18.1 (9.8–30.2)	18.3 (11.0–28.6)	18.2 (12.7–25.9)	18.7 (15.5–25.4)	18.9 (16.0–25.2)
Global	All age	18.2 (10.0–30.2)	18.4 (11.2–28.7)	18.3 (12.7–25.9)	18.6 (15.3–25.5)	18.8 (15.7–25.3)
Central Europe, eastern Europe, and central Asia	20 to 24	13.4 (5.2–27.0)	13.3 (5.4–26.6)	13.1 (5.8–24.7)	13.0 (6.8–25.0)	13.1 (7.1–25.1)
Central Europe, eastern Europe, and central Asia	25 to 29	14.1 (5.5–28.0)	13.8 (5.7–27.5)	13.6 (6.2–25.4)	13.5 (7.2–25.7)	13.5 (7.5–25.5)
Central Europe, eastern Europe, and central Asia	30 to 34	15.1 (5.9–29.6)	14.7 (6.0–29.1)	14.4 (6.7–26.4)	14.7 (7.4–28.1)	14.6 (7.9–27.5)
Central Europe, eastern Europe, and central Asia	35 to 39	15.4 (6.1–30.4)	15.4 (6.3–30.4)	15.0 (7.2–27.1)	15.2 (8.3–28.3)	15.3 (8.6–28.4)
Central Europe, eastern Europe, and central Asia	40 to 44	15.7 (6.3–30.9)	15.7 (6.5–30.9)	15.3 (7.3–27.5)	15.4 (8.8–28.1)	15.5 (9.0–28.3)
Central Europe, eastern Europe, and central Asia	45 to 49	15.0 (6.1–29.2)	15.2 (6.3–29.7)	15.1 (7.1–27.7)	15.0 (8.7–27.3)	15.0 (9.2–27.0)
Central Europe, eastern Europe, and central Asia	50 to 54	14.9 (5.8–29.6)	14.9 (6.4–29.1)	14.9 (7.1–27.2)	14.8 (8.6–26.8)	14.8 (9.0–26.5)
Central Europe, eastern Europe, and central Asia	55 to 59	14.0 (5.4–27.8)	13.7 (5.6–27.3)	13.8 (6.4–25.4)	14.1 (7.7–26.6)	14.0 (8.2–25.8)
Central Europe, eastern Europe, and central Asia	60 to 64	13.7 (5.1–27.8)	13.6 (5.2–27.9)	13.3 (6.3–24.4)	13.7 (7.3–26.0)	13.8 (7.7–26.1)
Central Europe, eastern Europe, and central Asia	65 to 69	12.5 (4.6–25.7)	12.3 (4.7–25.4)	12.0 (5.7–22.2)	12.4 (6.9–23.6)	12.5 (7.1–23.6)
Central Europe, eastern Europe, and central Asia	70 to 74	10.1 (3.4–21.8)	9.9 (3.4–21.6)	9.8 (3.8–19.9)	9.8 (4.7–20.0)	9.9 (4.9–20.3)

Supplementary Table S10: Prevalence of female SVAC by age and location for 1990, 2000, 2010, 2020, and 2023						
**Location**	**Age Range**	**1990**	**2000**	**2010**	**2020**	**2023**
Central Europe, eastern Europe, and central Asia	75 to 79	9.3 (3.0–20.8)	9.2 (2.9–20.9)	9.2 (3.1–20.1)	9.4 (3.4–21.1)	9.4 (3.4–21.2)
Central Europe, eastern Europe, and central Asia	80 to 84	6.6 (2.1–15.3)	6.7 (2.0–15.8)	6.8 (2.2–15.2)	6.9 (2.5–16.1)	6.9 (2.5–16.1)
Central Europe, eastern Europe, and central Asia	85 to 89	5.9 (1.8–13.9)	5.9 (1.7–14.0)	5.8 (1.7–13.5)	5.8 (1.8–14.5)	5.9 (1.9–14.5)
Central Europe, eastern Europe, and central Asia	90 to 94	5.3 (1.6–12.6)	5.3 (1.5–12.6)	5.3 (1.6–12.2)	5.3 (1.7–13.3)	5.3 (1.7–13.1)
Central Europe, eastern Europe, and central Asia	95 plus	5.3 (1.6–12.4)	5.2 (1.5–12.3)	5.1 (1.5–11.9)	5.2 (1.6–13.1)	5.2 (1.7–13.0)
Central Europe, eastern Europe, and central Asia	Age-standardized	13.9 (5.4–27.7)	13.8 (5.6–27.7)	13.6 (6.3–25.3)	13.7 (7.4–25.9)	13.8 (7.7–25.8)
Central Europe, eastern Europe, and central Asia	All age	13.8 (5.3–27.4)	13.6 (5.4–27.4)	13.3 (6.1–24.8)	13.4 (7.2–25.4)	13.4 (7.5–25.2)
Central Asia	20 to 24	12.4 (4.3–25.8)	12.3 (4.3–26.4)	12.3 (4.6–24.9)	12.8 (5.3–26.9)	12.9 (5.5–26.9)
Central Asia	25 to 29	13.1 (4.8–26.9)	12.9 (4.6–27.2)	12.8 (5.1–25.4)	13.1 (5.8–27.0)	13.2 (5.8–27.0)
Central Asia	30 to 34	14.1 (5.4–28.6)	13.9 (5.1–28.7)	13.8 (5.6–27.1)	14.0 (6.3–28.5)	14.1 (6.3–28.4)
Central Asia	35 to 39	14.6 (5.8–28.9)	14.4 (5.6–29.0)	14.5 (6.1–27.8)	14.6 (7.0–28.9)	14.6 (6.8–28.8)
Central Asia	40 to 44	15.1 (6.0–29.6)	14.8 (5.9–29.8)	14.8 (6.3–28.3)	15.1 (7.2–29.9)	15.1 (7.1–29.5)
Central Asia	45 to 49	15.0 (5.9–29.6)	14.8 (5.9–29.7)	14.6 (6.4–27.7)	14.9 (7.2–29.4)	14.9 (7.0–29.2)
Central Asia	50 to 54	14.8 (5.8–29.8)	14.9 (6.0–29.7)	14.8 (6.8–27.6)	14.7 (7.4–28.6)	14.8 (7.2–28.7)
Central Asia	55 to 59	14.2 (5.3–28.9)	14.2 (5.3–29.3)	14.0 (6.0–26.6)	13.9 (6.9–27.3)	14.0 (6.6–27.4)

Supplementary Table S10: Prevalence of female SVAC by age and location for 1990, 2000, 2010, 2020, and 2023						
**Location**	**Age Range**	**1990**	**2000**	**2010**	**2020**	**2023**
Central Asia	60 to 64	13.8 (4.7–29.0)	13.7 (4.7–29.2)	13.7 (5.1–27.6)	13.7 (5.5–29.0)	13.7 (5.7–28.8)
Central Asia	65 to 69	13.0 (4.3–27.9)	12.8 (4.2–28.0)	13.0 (4.4–27.0)	12.9 (4.9–28.1)	12.9 (5.1–27.8)
Central Asia	70 to 74	10.3 (3.3–23.0)	10.2 (3.1–23.2)	10.2 (3.2–22.4)	10.3 (3.4–24.2)	10.3 (3.4–23.8)
Central Asia	75 to 79	9.3 (3.0–20.9)	9.3 (2.8–21.3)	9.2 (2.9–20.5)	9.4 (3.1–22.3)	9.4 (3.1–21.9)
Central Asia	80 to 84	6.6 (2.0–15.1)	6.6 (1.9–15.5)	6.5 (2.0–15.0)	6.5 (2.1–16.1)	6.6 (2.1–16.0)
Central Asia	85 to 89	5.9 (1.8–13.7)	5.9 (1.7–13.9)	5.9 (1.8–13.6)	5.9 (1.9–14.6)	5.9 (1.9–14.4)
Central Asia	90 to 94	5.3 (1.6–12.4)	5.3 (1.5–12.6)	5.3 (1.6–12.3)	5.3 (1.7–13.3)	5.3 (1.7–13.1)
Central Asia	95 plus	5.1 (1.6–12.0)	5.1 (1.5–12.3)	5.2 (1.5–12.1)	5.3 (1.7–13.2)	5.3 (1.7–13.0)
Central Asia	Age-standardized	13.5 (5.0–27.4)	13.3 (4.9–27.7)	13.3 (5.3–26.1)	13.5 (6.1–27.7)	13.5 (6.0–27.5)
Central Asia	All age	13.4 (5.0–27.4)	13.4 (4.9–27.8)	13.4 (5.4–26.2)	13.6 (6.2–27.9)	13.6 (6.1–27.7)
Armenia	20 to 24	8.5 (3.0–17.7)	8.5 (3.4–16.7)	8.8 (4.9–14.3)	9.8 (7.1–13.3)	10.1 (7.1–14.0)
Armenia	25 to 29	12.7 (6.3–22.1)	12.3 (7.0–19.9)	11.6 (7.8–16.2)	11.1 (5.1–21.1)	11.1 (4.3–23.0)
Armenia	30 to 34	13.5 (6.7–23.7)	13.1 (7.1–21.1)	12.1 (8.1–16.9)	11.2 (5.6–20.9)	11.2 (4.7–22.8)
Armenia	35 to 39	14.3 (7.2–24.5)	14.0 (8.3–21.7)	13.7 (9.3–18.8)	13.5 (6.1–25.3)	13.4 (5.3–27.3)
Armenia	40 to 44	14.1 (7.1–24.2)	13.8 (8.1–21.5)	13.5 (9.2–18.7)	13.5 (6.1–25.5)	13.6 (5.3–27.6)

Supplementary Table S10: Prevalence of female SVAC by age and location for 1990, 2000, 2010, 2020, and 2023						
**Location**	**Age Range**	**1990**	**2000**	**2010**	**2020**	**2023**
Armenia	45 to 49	17.1 (8.9–28.2)	16.7 (10.4–24.7)	16.3 (11.7–21.4)	15.4 (7.1–28.1)	15.2 (6.0–30.0)
Armenia	50 to 54	17.6 (9.2–29.1)	17.2 (10.7–25.7)	16.8 (12.2–21.9)	15.7 (7.3–28.5)	15.4 (6.1–30.1)
Armenia	55 to 59	14.5 (7.3–24.4)	14.2 (8.6–21.5)	13.8 (9.7–18.6)	13.6 (6.2–25.2)	13.5 (5.3–27.5)
Armenia	60 to 64	12.9 (3.3–31.6)	12.7 (3.6–29.6)	12.5 (4.0–26.9)	12.5 (4.2–28.5)	12.5 (4.2–28.3)
Armenia	65 to 69	12.0 (3.0–29.2)	11.8 (3.3–27.6)	11.6 (3.7–25.3)	11.6 (3.9–26.8)	11.6 (3.9–26.6)
Armenia	70 to 74	9.6 (2.3–24.0)	9.4 (2.6–22.4)	9.2 (2.9–20.6)	9.2 (3.0–22.0)	9.3 (3.0–22.0)
Armenia	75 to 79	8.7 (2.0–22.6)	8.5 (2.3–20.7)	8.3 (2.6–18.8)	8.3 (2.7–20.0)	8.3 (2.7–19.9)
Armenia	80 to 84	6.1 (1.4–16.5)	6.0 (1.6–14.9)	5.8 (1.8–13.6)	5.8 (1.8–14.5)	5.9 (1.9–14.7)
Armenia	85 to 89	5.5 (1.3–14.9)	5.4 (1.4–13.5)	5.3 (1.6–12.3)	5.2 (1.6–13.2)	5.3 (1.7–13.0)
Armenia	90 to 94	5.0 (1.1–13.6)	4.9 (1.3–12.2)	4.7 (1.4–11.1)	4.7 (1.5–11.9)	4.8 (1.5–11.9)
Armenia	95 plus	5.0 (1.1–13.5)	4.8 (1.2–12.3)	4.7 (1.4–11.0)	4.7 (1.5–11.8)	4.7 (1.5–11.7)
Armenia	Age-standardized	13.0 (6.1–23.3)	12.7 (7.1–20.7)	12.4 (9.5–15.6)	12.1 (8.4–18.8)	12.1 (7.8–20.4)
Armenia	All age	13.0 (6.1–23.3)	12.8 (7.0–21.1)	12.4 (9.6–15.7)	12.0 (8.7–17.5)	12.0 (8.2–18.3)
Azerbaijan	20 to 24	9.6 (3.1–21.2)	9.1 (3.3–19.1)	8.4 (4.1–15.0)	8.4 (4.4–14.3)	8.5 (4.1–15.6)
Azerbaijan	25 to 29	10.1 (3.3–22.1)	9.8 (3.6–20.2)	9.2 (4.7–16.1)	9.2 (4.9–15.3)	9.3 (4.5–16.8)

Supplementary Table S10: Prevalence of female SVAC by age and location for 1990, 2000, 2010, 2020, and 2023						
**Location**	**Age Range**	**1990**	**2000**	**2010**	**2020**	**2023**
Azerbaijan	30 to 34	10.8 (3.5–23.5)	10.3 (3.8–21.2)	9.5 (4.8–16.6)	9.5 (5.1–15.8)	9.6 (4.7–17.3)
Azerbaijan	35 to 39	11.4 (3.8–25.1)	11.3 (4.4–23.0)	11.1 (6.0–18.8)	11.0 (6.5–17.1)	11.1 (5.8–19.3)
Azerbaijan	40 to 44	11.8 (3.9–25.4)	11.6 (4.4–23.2)	11.2 (6.1–18.8)	11.1 (6.3–17.9)	11.2 (5.8–19.4)
Azerbaijan	45 to 49	11.5 (3.8–24.8)	11.3 (4.3–22.6)	10.8 (5.8–18.3)	10.7 (6.1–17.4)	10.9 (5.6–18.9)
Azerbaijan	50 to 54	11.5 (3.8–25.1)	11.4 (4.4–23.2)	11.2 (6.1–19.1)	11.2 (6.6–17.4)	11.2 (5.9–19.6)
Azerbaijan	55 to 59	10.7 (3.5–23.2)	10.3 (3.8–21.1)	9.6 (5.0–16.7)	9.6 (5.2–15.8)	9.7 (4.8–17.4)
Azerbaijan	60 to 64	10.6 (3.4–23.8)	10.5 (3.2–23.7)	10.6 (3.3–23.2)	10.6 (3.5–24.9)	10.7 (3.6–24.6)
Azerbaijan	65 to 69	9.8 (3.1–22.3)	9.8 (2.9–22.2)	9.8 (3.1–21.8)	9.9 (3.2–23.4)	9.9 (3.3–23.1)
Azerbaijan	70 to 74	7.8 (2.4–18.1)	7.8 (2.3–18.0)	7.8 (2.4–17.6)	7.8 (2.5–19.0)	7.8 (2.6–18.7)
Azerbaijan	75 to 79	7.0 (2.1–16.3)	7.0 (2.0–16.3)	7.0 (2.1–16.0)	7.0 (2.2–17.2)	7.0 (2.3–17.0)
Azerbaijan	80 to 84	4.9 (1.5–11.7)	4.9 (1.4–11.7)	4.9 (1.4–11.4)	4.9 (1.5–12.4)	4.9 (1.6–12.2)
Azerbaijan	85 to 89	4.4 (1.3–10.6)	4.4 (1.2–10.5)	4.4 (1.3–10.3)	4.4 (1.4–11.2)	4.4 (1.4–11.0)
Azerbaijan	90 to 94	4.0 (1.2– 9.6)	3.9 (1.1– 9.6)	3.9 (1.2– 9.3)	4.0 (1.2–10.1)	4.0 (1.2–10.0)
Azerbaijan	95 plus	3.9 (1.2– 9.5)	3.9 (1.1– 9.5)	3.9 (1.1– 9.3)	3.9 (1.2–10.1)	4.0 (1.2– 9.9)
Azerbaijan	Age-standardized	10.4 (3.5–22.5)	10.1 (3.7–20.7)	9.7 (4.9–16.9)	9.7 (5.2–16.5)	9.8 (5.0–17.2)

Supplementary Table S10: Prevalence of female SVAC by age and location for 1990, 2000, 2010, 2020, and 2023						
**Location**	**Age Range**	**1990**	**2000**	**2010**	**2020**	**2023**
Azerbaijan	All age	10.3 (3.5–22.4)	10.3 (3.8–20.9)	9.8 (5.1–16.8)	9.8 (5.4–16.5)	10.0 (5.1–17.4)
Georgia	20 to 24	11.1 (4.0–23.6)	9.6 (4.4–17.2)	10.4 (7.2–14.7)	17.6 (14.5–21.1)	20.3 (16.0–25.2)
Georgia	25 to 29	13.5 (7.2–22.3)	11.7 (6.0–19.7)	11.8 (8.3–16.3)	14.8 (12.0–18.2)	15.5 (11.8–20.1)
Georgia	30 to 34	14.2 (8.7–21.6)	12.3 (6.7–19.5)	12.1 (8.6–16.2)	14.9 (12.0–18.3)	15.6 (11.8–20.2)
Georgia	35 to 39	15.0 (9.2–22.7)	14.0 (8.8–20.6)	14.5 (10.7–19.1)	15.1 (12.2–18.5)	14.6 (10.8–19.1)
Georgia	40 to 44	15.4 (8.1–25.9)	14.2 (9.5–20.0)	14.5 (10.8–19.1)	15.1 (12.1–18.6)	14.6 (10.9–19.1)
Georgia	45 to 49	15.2 (6.7–28.4)	14.2 (8.9–21.3)	14.3 (10.8–18.6)	15.0 (12.1–18.4)	14.7 (11.0–19.2)
Georgia	50 to 54	15.7 (6.1–31.0)	16.0 (8.2–26.6)	17.0 (12.2–23.1)	15.9 (12.9–19.4)	15.1 (11.3–19.6)
Georgia	55 to 59	15.6 (5.7–31.9)	15.6 (6.7–28.4)	15.2 (10.3–21.7)	14.4 (11.5–17.7)	14.2 (10.6–18.7)
Georgia	60 to 64	15.2 (5.3–31.9)	15.3 (6.1–28.8)	15.0 (9.2–23.1)	14.4 (11.6–17.5)	14.1 (10.6–18.5)
Georgia	65 to 69	14.2 (4.7–30.8)	14.5 (5.1–29.9)	14.9 (6.9–27.3)	15.0 (10.8–21.2)	15.0 (10.7–20.4)
Georgia	70 to 74	11.1 (3.6–24.8)	11.1 (3.4–24.7)	11.1 (3.5–24.3)	11.3 (3.8–26.2)	11.3 (3.8–25.8)
Georgia	75 to 79	10.0 (3.2–22.6)	10.0 (3.0–22.6)	10.0 (3.1–22.1)	10.2 (3.3–23.9)	10.2 (3.4–23.6)
Georgia	80 to 84	7.1 (2.2–16.6)	7.1 (2.1–16.5)	7.1 (2.2–16.2)	7.2 (2.3–17.6)	7.2 (2.3–17.4)
Georgia	85 to 89	6.4 (1.9–15.1)	6.4 (1.8–15.0)	6.4 (1.9–14.7)	6.5 (2.1–16.0)	6.5 (2.1–15.8)

Supplementary Table S10: Prevalence of female SVAC by age and location for 1990, 2000, 2010, 2020, and 2023						
**Location**	**Age Range**	**1990**	**2000**	**2010**	**2020**	**2023**
Georgia	90 to 94	5.8 (1.7–13.7)	5.8 (1.7–13.6)	5.8 (1.7–13.4)	5.9 (1.8–14.6)	5.9 (1.9–14.3)
Georgia	95 plus	5.7 (1.7–13.6)	5.7 (1.6–13.5)	5.7 (1.7–13.3)	5.8 (1.8–14.4)	5.8 (1.9–14.2)
Georgia	Age-standardized	13.8 (6.5–25.0)	12.9 (6.8–21.0)	13.2 (9.3–18.4)	14.8 (12.0–18.2)	15.0 (11.8–19.1)
Georgia	All age	13.8 (6.4–25.4)	13.0 (6.8–21.4)	13.2 (9.1–18.9)	14.2 (11.4–17.6)	14.2 (11.1–18.1)
Kazakhstan	20 to 24	13.1 (4.3–28.5)	13.0 (4.1–28.4)	13.0 (4.2–27.9)	13.2 (4.5–29.9)	13.2 (4.6–29.6)
Kazakhstan	25 to 29	13.4 (4.4–29.2)	13.4 (4.2–29.1)	13.4 (4.4–28.6)	13.6 (4.6–30.6)	13.6 (4.7–30.3)
Kazakhstan	30 to 34	14.6 (4.8–31.3)	14.5 (4.6–31.2)	14.5 (4.8–30.7)	14.7 (5.1–32.8)	14.7 (5.2–32.4)
Kazakhstan	35 to 39	14.8 (4.9–31.8)	14.8 (4.7–31.7)	14.8 (4.9–31.1)	15.0 (5.2–33.2)	15.0 (5.3–32.9)
Kazakhstan	40 to 44	15.4 (5.2–32.9)	15.4 (4.9–32.8)	15.4 (5.1–32.2)	15.6 (5.4–34.3)	15.6 (5.5–33.8)
Kazakhstan	45 to 49	15.1 (5.1–32.4)	15.1 (4.8–32.2)	15.1 (5.0–31.7)	15.3 (5.3–33.8)	15.3 (5.4–33.3)
Kazakhstan	50 to 54	14.9 (5.0–31.9)	14.9 (4.7–31.8)	14.9 (4.9–31.3)	15.0 (5.2–33.4)	15.1 (5.3–32.8)
Kazakhstan	55 to 59	14.5 (4.8–31.2)	14.4 (4.6–31.1)	14.5 (4.8–30.5)	14.6 (5.1–32.6)	14.6 (5.1–32.3)
Kazakhstan	60 to 64	14.1 (4.7–30.4)	14.0 (4.4–30.3)	14.0 (4.6–29.8)	14.2 (4.9–31.8)	14.2 (5.0–31.5)
Kazakhstan	65 to 69	13.1 (4.3–28.6)	13.1 (4.1–28.6)	13.1 (4.2–28.0)	13.2 (4.5–30.0)	13.3 (4.6–29.7)
Kazakhstan	70 to 74	10.5 (3.3–23.6)	10.5 (3.2–23.5)	10.5 (3.3–23.1)	10.6 (3.5–24.8)	10.6 (3.6–24.5)

Supplementary Table S10: Prevalence of female SVAC by age and location for 1990, 2000, 2010, 2020, and 2023						
**Location**	**Age Range**	**1990**	**2000**	**2010**	**2020**	**2023**
Kazakhstan	75 to 79	9.4 (3.0–21.5)	9.4 (2.8–21.4)	9.4 (2.9–21.0)	9.5 (3.1–22.7)	9.6 (3.2–22.4)
Kazakhstan	80 to 84	6.7 (2.0–15.7)	6.7 (1.9–15.6)	6.7 (2.0–15.3)	6.8 (2.1–16.6)	6.8 (2.2–16.4)
Kazakhstan	85 to 89	6.0 (1.8–14.2)	6.0 (1.7–14.2)	6.0 (1.8–13.9)	6.1 (1.9–15.1)	6.1 (1.9–14.8)
Kazakhstan	90 to 94	5.4 (1.6–12.9)	5.4 (1.6–12.9)	5.4 (1.6–12.6)	5.5 (1.7–13.7)	5.5 (1.7–13.5)
Kazakhstan	95 plus	5.4 (1.6–12.8)	5.4 (1.5–12.8)	5.4 (1.6–12.5)	5.4 (1.7–13.6)	5.4 (1.7–13.4)
Kazakhstan	Age-standardized	13.8 (4.6–29.8)	13.7 (4.3–29.7)	13.8 (4.5–29.2)	13.9 (4.8–31.2)	13.9 (4.9–30.8)
Kazakhstan	All age	13.8 (4.6–29.9)	13.8 (4.4–29.9)	13.8 (4.5–29.3)	14.0 (4.8–31.3)	14.0 (4.9–30.9)
Kyrgyzstan	20 to 24	13.1 (4.3–28.5)	13.0 (4.1–28.4)	13.0 (4.2–27.9)	13.2 (4.5–29.9)	13.2 (4.6–29.6)
Kyrgyzstan	25 to 29	13.4 (4.4–29.2)	13.4 (4.2–29.1)	13.4 (4.4–28.6)	13.6 (4.6–30.6)	13.6 (4.7–30.3)
Kyrgyzstan	30 to 34	14.6 (4.8–31.3)	14.5 (4.6–31.2)	14.5 (4.8–30.7)	14.7 (5.1–32.8)	14.7 (5.2–32.4)
Kyrgyzstan	35 to 39	14.8 (4.9–31.8)	14.8 (4.7–31.7)	14.8 (4.9–31.1)	15.0 (5.2–33.2)	15.0 (5.3–32.9)
Kyrgyzstan	40 to 44	15.4 (5.2–32.9)	15.4 (4.9–32.8)	15.4 (5.1–32.2)	15.6 (5.4–34.3)	15.6 (5.5–33.8)
Kyrgyzstan	45 to 49	15.1 (5.1–32.4)	15.1 (4.8–32.2)	15.1 (5.0–31.7)	15.3 (5.3–33.8)	15.3 (5.4–33.3)
Kyrgyzstan	50 to 54	14.9 (5.0–31.9)	14.9 (4.7–31.8)	14.9 (4.9–31.3)	15.0 (5.2–33.4)	15.1 (5.3–32.8)
Kyrgyzstan	55 to 59	14.5 (4.8–31.2)	14.4 (4.6–31.1)	14.5 (4.8–30.5)	14.6 (5.1–32.6)	14.6 (5.1–32.3)

Supplementary Table S10: Prevalence of female SVAC by age and location for 1990, 2000, 2010, 2020, and 2023						
**Location**	**Age Range**	**1990**	**2000**	**2010**	**2020**	**2023**
Kyrgyzstan	60 to 64	14.1 (4.7–30.4)	14.0 (4.4–30.3)	14.0 (4.6–29.8)	14.2 (4.9–31.8)	14.2 (5.0–31.5)
Kyrgyzstan	65 to 69	13.1 (4.3–28.6)	13.1 (4.1–28.6)	13.1 (4.2–28.0)	13.2 (4.5–30.0)	13.3 (4.6–29.7)
Kyrgyzstan	70 to 74	10.5 (3.3–23.6)	10.5 (3.2–23.5)	10.5 (3.3–23.1)	10.6 (3.5–24.8)	10.6 (3.6–24.5)
Kyrgyzstan	75 to 79	9.4 (3.0–21.5)	9.4 (2.8–21.4)	9.4 (2.9–21.0)	9.5 (3.1–22.7)	9.6 (3.2–22.4)
Kyrgyzstan	80 to 84	6.7 (2.0–15.7)	6.7 (1.9–15.6)	6.7 (2.0–15.3)	6.8 (2.1–16.6)	6.8 (2.2–16.4)
Kyrgyzstan	85 to 89	6.0 (1.8–14.2)	6.0 (1.7–14.2)	6.0 (1.8–13.9)	6.1 (1.9–15.1)	6.1 (1.9–14.8)
Kyrgyzstan	90 to 94	5.4 (1.6–12.9)	5.4 (1.6–12.9)	5.4 (1.6–12.6)	5.5 (1.7–13.7)	5.5 (1.7–13.5)
Kyrgyzstan	95 plus	5.4 (1.6–12.8)	5.4 (1.5–12.8)	5.4 (1.6–12.5)	5.4 (1.7–13.6)	5.4 (1.7–13.4)
Kyrgyzstan	Age-standardized	13.8 (4.6–29.8)	13.7 (4.3–29.7)	13.8 (4.5–29.2)	13.9 (4.8–31.2)	13.9 (4.9–30.8)
Kyrgyzstan	All age	13.7 (4.5–29.7)	13.7 (4.3–29.7)	13.8 (4.5–29.3)	14.1 (4.9–31.6)	14.1 (4.9–31.2)
Mongolia	20 to 24	15.5 (5.4–32.3)	15.0 (6.0–29.4)	14.1 (8.1–22.9)	13.7 (9.4–18.9)	13.9 (8.5–21.8)
Mongolia	25 to 29	16.3 (5.7–33.8)	16.2 (6.5–31.4)	15.7 (9.1–25.1)	15.4 (10.8–21.0)	15.5 (9.7–24.0)
Mongolia	30 to 34	17.4 (6.1–35.5)	16.8 (6.9–32.5)	15.9 (9.3–25.5)	15.6 (10.9–21.2)	15.8 (9.8–24.4)
Mongolia	35 to 39	18.5 (6.6–37.4)	18.8 (7.8–35.6)	18.9 (11.3–29.5)	18.8 (13.5–25.1)	18.8 (11.9–28.3)
Mongolia	40 to 44	19.0 (6.8–38.2)	19.1 (8.0–36.2)	19.0 (11.4–29.7)	18.9 (13.6–25.2)	18.9 (12.0–28.4)

Supplementary Table S10: Prevalence of female SVAC by age and location for 1990, 2000, 2010, 2020, and 2023						
**Location**	**Age Range**	**1990**	**2000**	**2010**	**2020**	**2023**
Mongolia	45 to 49	18.6 (6.7–37.5)	18.6 (7.7–35.4)	18.4 (11.0–28.9)	18.2 (13.1–24.4)	18.3 (11.6–27.7)
Mongolia	50 to 54	18.6 (6.7–37.5)	19.0 (7.9–35.9)	19.2 (11.5–30.0)	19.1 (13.8–25.5)	19.1 (12.1–28.8)
Mongolia	55 to 59	17.3 (6.1–35.4)	16.9 (6.9–32.7)	16.2 (9.5–25.9)	15.9 (11.2–21.6)	16.1 (10.0–24.7)
Mongolia	60 to 64	16.9 (5.9–34.7)	16.6 (6.8–32.2)	16.0 (9.4–25.6)	15.8 (11.1–21.4)	15.9 (9.9–24.5)
Mongolia	65 to 69	15.8 (5.3–33.5)	15.8 (5.1–33.5)	15.8 (5.3–32.9)	16.0 (5.6–35.0)	16.0 (5.7–34.6)
Mongolia	70 to 74	12.8 (4.2–28.0)	12.7 (4.0–27.9)	12.8 (4.1–27.4)	12.9 (4.4–29.3)	12.9 (4.4–29.0)
Mongolia	75 to 79	11.5 (3.7–25.6)	11.5 (3.5–25.6)	11.5 (3.7–25.1)	11.6 (3.9–26.9)	11.6 (3.9–26.5)
Mongolia	80 to 84	8.2 (2.5–19.0)	8.2 (2.4–18.9)	8.2 (2.5–18.6)	8.3 (2.7–20.0)	8.3 (2.7–19.7)
Mongolia	85 to 89	7.4 (2.3–17.3)	7.4 (2.2–17.2)	7.4 (2.3–16.9)	7.5 (2.4–18.2)	7.5 (2.4–18.0)
Mongolia	90 to 94	6.7 (2.0–15.7)	6.7 (1.9–15.7)	6.7 (2.0–15.4)	6.8 (2.2–16.6)	6.8 (2.2–16.4)
Mongolia	95 plus	6.7 (2.0–15.6)	6.6 (1.9–15.6)	6.6 (2.0–15.3)	6.7 (2.1–16.5)	6.7 (2.2–16.3)
Mongolia	Age-standardized	16.8 (5.9–33.8)	16.7 (6.7–31.9)	16.3 (9.1–26.3)	16.1 (11.5–22.1)	16.2 (10.4–24.2)
Mongolia	All age	16.7 (5.8–34.1)	16.8 (6.8–32.1)	16.6 (9.5–26.4)	16.5 (11.7–22.1)	16.6 (10.6–24.7)
Tajikistan	20 to 24	13.1 (4.3–28.5)	13.0 (4.1–28.4)	13.0 (4.2–27.9)	13.2 (4.5–29.9)	13.2 (4.6–29.6)
Tajikistan	25 to 29	13.4 (4.4–29.2)	13.4 (4.2–29.1)	13.4 (4.4–28.6)	13.6 (4.6–30.6)	13.6 (4.7–30.3)

Supplementary Table S10: Prevalence of female SVAC by age and location for 1990, 2000, 2010, 2020, and 2023						
**Location**	**Age Range**	**1990**	**2000**	**2010**	**2020**	**2023**
Tajikistan	30 to 34	14.6 (4.8–31.3)	14.5 (4.6–31.2)	14.5 (4.8–30.7)	14.7 (5.1–32.8)	14.7 (5.2–32.4)
Tajikistan	35 to 39	14.8 (4.9–31.8)	14.8 (4.7–31.7)	14.8 (4.9–31.1)	15.0 (5.2–33.2)	15.0 (5.3–32.9)
Tajikistan	40 to 44	15.4 (5.2–32.9)	15.4 (4.9–32.8)	15.4 (5.1–32.2)	15.6 (5.4–34.3)	15.6 (5.5–33.8)
Tajikistan	45 to 49	15.1 (5.1–32.4)	15.1 (4.8–32.2)	15.1 (5.0–31.7)	15.3 (5.3–33.8)	15.3 (5.4–33.3)
Tajikistan	50 to 54	14.9 (5.0–31.9)	14.9 (4.7–31.8)	14.9 (4.9–31.3)	15.0 (5.2–33.4)	15.1 (5.3–32.8)
Tajikistan	55 to 59	14.5 (4.8–31.2)	14.4 (4.6–31.1)	14.5 (4.8–30.5)	14.6 (5.1–32.6)	14.6 (5.1–32.3)
Tajikistan	60 to 64	14.1 (4.7–30.4)	14.0 (4.4–30.3)	14.0 (4.6–29.8)	14.2 (4.9–31.8)	14.2 (5.0–31.5)
Tajikistan	65 to 69	13.1 (4.3–28.6)	13.1 (4.1–28.6)	13.1 (4.2–28.0)	13.2 (4.5–30.0)	13.3 (4.6–29.7)
Tajikistan	70 to 74	10.5 (3.3–23.6)	10.5 (3.2–23.5)	10.5 (3.3–23.1)	10.6 (3.5–24.8)	10.6 (3.6–24.5)
Tajikistan	75 to 79	9.4 (3.0–21.5)	9.4 (2.8–21.4)	9.4 (2.9–21.0)	9.5 (3.1–22.7)	9.6 (3.2–22.4)
Tajikistan	80 to 84	6.7 (2.0–15.7)	6.7 (1.9–15.6)	6.7 (2.0–15.3)	6.8 (2.1–16.6)	6.8 (2.2–16.4)
Tajikistan	85 to 89	6.0 (1.8–14.2)	6.0 (1.7–14.2)	6.0 (1.8–13.9)	6.1 (1.9–15.1)	6.1 (1.9–14.8)
Tajikistan	90 to 94	5.4 (1.6–12.9)	5.4 (1.6–12.9)	5.4 (1.6–12.6)	5.5 (1.7–13.7)	5.5 (1.7–13.5)
Tajikistan	95 plus	5.4 (1.6–12.8)	5.4 (1.5–12.8)	5.4 (1.6–12.5)	5.4 (1.7–13.6)	5.4 (1.7–13.4)
Tajikistan	Age-standardized	13.8 (4.6–29.8)	13.7 (4.3–29.7)	13.8 (4.5–29.2)	13.9 (4.8–31.2)	13.9 (4.9–30.8)

Supplementary Table S10: Prevalence of female SVAC by age and location for 1990, 2000, 2010, 2020, and 2023						
**Location**	**Age Range**	**1990**	**2000**	**2010**	**2020**	**2023**
Tajikistan	All age	13.8 (4.5–29.8)	13.9 (4.4–30.0)	13.9 (4.6–29.6)	14.2 (4.9–31.8)	14.2 (5.0–31.4)
Turkmenistan	20 to 24	13.1 (4.3–28.5)	13.0 (4.1–28.4)	13.0 (4.2–27.9)	13.2 (4.5–29.9)	13.2 (4.6–29.6)
Turkmenistan	25 to 29	13.4 (4.4–29.2)	13.4 (4.2–29.1)	13.4 (4.4–28.6)	13.6 (4.6–30.6)	13.6 (4.7–30.3)
Turkmenistan	30 to 34	14.6 (4.8–31.3)	14.5 (4.6–31.2)	14.5 (4.8–30.7)	14.7 (5.1–32.8)	14.7 (5.2–32.4)
Turkmenistan	35 to 39	14.8 (4.9–31.8)	14.8 (4.7–31.7)	14.8 (4.9–31.1)	15.0 (5.2–33.2)	15.0 (5.3–32.9)
Turkmenistan	40 to 44	15.4 (5.2–32.9)	15.4 (4.9–32.8)	15.4 (5.1–32.2)	15.6 (5.4–34.3)	15.6 (5.5–33.8)
Turkmenistan	45 to 49	15.1 (5.1–32.4)	15.1 (4.8–32.2)	15.1 (5.0–31.7)	15.3 (5.3–33.8)	15.3 (5.4–33.3)
Turkmenistan	50 to 54	14.9 (5.0–31.9)	14.9 (4.7–31.8)	14.9 (4.9–31.3)	15.0 (5.2–33.4)	15.1 (5.3–32.8)
Turkmenistan	55 to 59	14.5 (4.8–31.2)	14.4 (4.6–31.1)	14.5 (4.8–30.5)	14.6 (5.1–32.6)	14.6 (5.1–32.3)
Turkmenistan	60 to 64	14.1 (4.7–30.4)	14.0 (4.4–30.3)	14.0 (4.6–29.8)	14.2 (4.9–31.8)	14.2 (5.0–31.5)
Turkmenistan	65 to 69	13.1 (4.3–28.6)	13.1 (4.1–28.6)	13.1 (4.2–28.0)	13.2 (4.5–30.0)	13.3 (4.6–29.7)
Turkmenistan	70 to 74	10.5 (3.3–23.6)	10.5 (3.2–23.5)	10.5 (3.3–23.1)	10.6 (3.5–24.8)	10.6 (3.6–24.5)
Turkmenistan	75 to 79	9.4 (3.0–21.5)	9.4 (2.8–21.4)	9.4 (2.9–21.0)	9.5 (3.1–22.7)	9.6 (3.2–22.4)
Turkmenistan	80 to 84	6.7 (2.0–15.7)	6.7 (1.9–15.6)	6.7 (2.0–15.3)	6.8 (2.1–16.6)	6.8 (2.2–16.4)
Turkmenistan	85 to 89	6.0 (1.8–14.2)	6.0 (1.7–14.2)	6.0 (1.8–13.9)	6.1 (1.9–15.1)	6.1 (1.9–14.8)

Supplementary Table S10: Prevalence of female SVAC by age and location for 1990, 2000, 2010, 2020, and 2023						
**Location**	**Age Range**	**1990**	**2000**	**2010**	**2020**	**2023**
Turkmenistan	90 to 94	5.4 (1.6–12.9)	5.4 (1.6–12.9)	5.4 (1.6–12.6)	5.5 (1.7–13.7)	5.5 (1.7–13.5)
Turkmenistan	95 plus	5.4 (1.6–12.8)	5.4 (1.5–12.8)	5.4 (1.6–12.5)	5.4 (1.7–13.6)	5.4 (1.7–13.4)
Turkmenistan	Age-standardized	13.8 (4.6–29.8)	13.7 (4.3–29.7)	13.8 (4.5–29.2)	13.9 (4.8–31.2)	13.9 (4.9–30.8)
Turkmenistan	All age	13.8 (4.6–29.9)	13.9 (4.4–30.0)	13.9 (4.6–29.6)	14.1 (4.9–31.7)	14.1 (5.0–31.2)
Uzbekistan	20 to 24	13.1 (4.3–28.5)	13.0 (4.1–28.4)	13.0 (4.2–27.9)	13.2 (4.5–29.9)	13.2 (4.6–29.6)
Uzbekistan	25 to 29	13.4 (4.4–29.2)	13.4 (4.2–29.1)	13.4 (4.4–28.6)	13.6 (4.6–30.6)	13.6 (4.7–30.3)
Uzbekistan	30 to 34	14.6 (4.8–31.3)	14.5 (4.6–31.2)	14.5 (4.8–30.7)	14.7 (5.1–32.8)	14.7 (5.2–32.4)
Uzbekistan	35 to 39	14.8 (4.9–31.8)	14.8 (4.7–31.7)	14.8 (4.9–31.1)	15.0 (5.2–33.2)	15.0 (5.3–32.9)
Uzbekistan	40 to 44	15.4 (5.2–32.9)	15.4 (4.9–32.8)	15.4 (5.1–32.2)	15.6 (5.4–34.3)	15.6 (5.5–33.8)
Uzbekistan	45 to 49	15.1 (5.1–32.4)	15.1 (4.8–32.2)	15.1 (5.0–31.7)	15.3 (5.3–33.8)	15.3 (5.4–33.3)
Uzbekistan	50 to 54	14.9 (5.0–31.9)	14.9 (4.7–31.8)	14.9 (4.9–31.3)	15.0 (5.2–33.4)	15.1 (5.3–32.8)
Uzbekistan	55 to 59	14.5 (4.8–31.2)	14.4 (4.6–31.1)	14.5 (4.8–30.5)	14.6 (5.1–32.6)	14.6 (5.1–32.3)
Uzbekistan	60 to 64	14.1 (4.7–30.4)	14.0 (4.4–30.3)	14.0 (4.6–29.8)	14.2 (4.9–31.8)	14.2 (5.0–31.5)
Uzbekistan	65 to 69	13.1 (4.3–28.6)	13.1 (4.1–28.6)	13.1 (4.2–28.0)	13.2 (4.5–30.0)	13.3 (4.6–29.7)
Uzbekistan	70 to 74	10.5 (3.3–23.6)	10.5 (3.2–23.5)	10.5 (3.3–23.1)	10.6 (3.5–24.8)	10.6 (3.6–24.5)

Supplementary Table S10: Prevalence of female SVAC by age and location for 1990, 2000, 2010, 2020, and 2023						
**Location**	**Age Range**	**1990**	**2000**	**2010**	**2020**	**2023**
Uzbekistan	75 to 79	9.4 (3.0–21.5)	9.4 (2.8–21.4)	9.4 (2.9–21.0)	9.5 (3.1–22.7)	9.6 (3.2–22.4)
Uzbekistan	80 to 84	6.7 (2.0–15.7)	6.7 (1.9–15.6)	6.7 (2.0–15.3)	6.8 (2.1–16.6)	6.8 (2.2–16.4)
Uzbekistan	85 to 89	6.0 (1.8–14.2)	6.0 (1.7–14.2)	6.0 (1.8–13.9)	6.1 (1.9–15.1)	6.1 (1.9–14.8)
Uzbekistan	90 to 94	5.4 (1.6–12.9)	5.4 (1.6–12.9)	5.4 (1.6–12.6)	5.5 (1.7–13.7)	5.5 (1.7–13.5)
Uzbekistan	95 plus	5.4 (1.6–12.8)	5.4 (1.5–12.8)	5.4 (1.6–12.5)	5.4 (1.7–13.6)	5.4 (1.7–13.4)
Uzbekistan	Age-standardized	13.8 (4.6–29.8)	13.7 (4.3–29.7)	13.8 (4.5–29.2)	13.9 (4.8–31.2)	13.9 (4.9–30.8)
Uzbekistan	All age	13.8 (4.5–29.8)	13.9 (4.4–30.0)	13.9 (4.6–29.5)	14.2 (4.9–31.7)	14.2 (5.0–31.3)
Central Europe	20 to 24	11.1 (5.3–20.4)	10.9 (6.7–16.7)	9.5 (7.4–12.0)	9.2 (7.0–12.4)	9.4 (6.7–13.6)
Central Europe	25 to 29	11.9 (5.6–21.7)	11.7 (7.3–17.8)	10.6 (8.5–13.2)	10.1 (7.7–13.5)	10.2 (7.4–14.4)
Central Europe	30 to 34	12.6 (5.9–23.1)	12.0 (7.3–18.7)	11.0 (8.8–13.7)	10.7 (8.1–14.1)	10.9 (7.9–15.4)
Central Europe	35 to 39	13.2 (6.0–24.6)	13.3 (8.2–20.4)	12.3 (10.0–15.1)	12.0 (9.4–15.9)	12.1 (8.8–17.3)
Central Europe	40 to 44	13.2 (6.1–24.6)	13.2 (8.0–20.2)	12.0 (9.7–14.9)	11.9 (9.1–16.2)	12.0 (8.5–18.0)
Central Europe	45 to 49	12.0 (6.0–21.4)	12.5 (7.7–19.1)	11.9 (9.6–14.7)	11.6 (9.4–15.4)	11.7 (8.8–17.1)
Central Europe	50 to 54	11.7 (5.5–21.3)	12.5 (7.9–18.9)	12.0 (9.8–14.7)	11.7 (8.9–15.9)	11.8 (8.3–17.6)
Central Europe	55 to 59	10.6 (4.8–19.8)	10.5 (6.2–16.7)	10.1 (8.0–12.7)	10.5 (8.0–14.2)	10.6 (7.6–16.0)

Supplementary Table S10: Prevalence of female SVAC by age and location for 1990, 2000, 2010, 2020, and 2023						
**Location**	**Age Range**	**1990**	**2000**	**2010**	**2020**	**2023**
Central Europe	60 to 64	10.5 (4.6–19.9)	10.2 (5.8–16.4)	10.0 (8.0–12.4)	10.2 (7.7–14.1)	10.5 (7.5–15.8)
Central Europe	65 to 69	9.7 (4.1–18.8)	9.2 (5.1–15.3)	8.8 (6.8–11.4)	9.1 (6.8–12.7)	9.2 (6.5–14.5)
Central Europe	70 to 74	7.6 (2.9–15.8)	7.1 (3.1–13.4)	6.6 (4.2– 9.8)	7.1 (5.8–10.1)	7.3 (5.6–10.5)
Central Europe	75 to 79	7.5 (2.7–16.3)	7.7 (2.7–16.8)	7.9 (3.1–16.2)	8.2 (3.5–17.3)	8.3 (3.2–17.7)
Central Europe	80 to 84	5.6 (1.8–13.0)	5.8 (1.8–13.5)	6.2 (2.3–13.3)	6.4 (2.7–13.3)	6.5 (2.6–13.7)
Central Europe	85 to 89	4.9 (1.5–11.2)	4.8 (1.4–11.7)	4.9 (1.4–11.4)	4.9 (1.5–12.3)	4.9 (1.5–12.5)
Central Europe	90 to 94	4.5 (1.4–10.4)	4.4 (1.3–10.7)	4.4 (1.3–10.5)	4.5 (1.4–11.3)	4.5 (1.4–11.5)
Central Europe	95 plus	4.4 (1.4–10.2)	4.5 (1.3–10.9)	4.4 (1.3–10.3)	4.5 (1.4–11.3)	4.5 (1.4–11.5)
Central Europe	Age-standardized	11.4 (5.3–21.1)	11.3 (6.8–17.8)	10.5 (8.3–13.4)	10.4 (8.2–13.6)	10.5 (8.0–14.8)
Central Europe	All age	11.2 (5.1–20.9)	11.1 (6.6–17.6)	10.2 (7.9–13.3)	10.0 (8.2–12.6)	10.1 (7.9–13.8)
Albania	20 to 24	9.2 (3.0–20.9)	8.9 (3.4–18.8)	8.2 (4.6–13.6)	7.7 (6.1– 9.3)	7.7 (5.4–10.4)
Albania	25 to 29	10.0 (3.3–22.8)	9.7 (3.8–20.4)	9.1 (5.2–14.9)	8.7 (7.1–10.4)	8.7 (6.3–11.7)
Albania	30 to 34	10.3 (3.4–23.5)	10.0 (3.9–20.9)	9.3 (5.3–15.1)	8.7 (7.1–10.4)	8.7 (6.3–11.7)
Albania	35 to 39	11.8 (4.0–26.0)	11.6 (4.6–23.7)	11.1 (6.5–17.7)	10.7 (8.9–12.6)	10.7 (7.8–14.1)
Albania	40 to 44	12.0 (4.1–26.3)	11.7 (4.6–23.9)	11.1 (6.5–17.8)	10.7 (8.9–12.6)	10.7 (7.8–14.1)

Supplementary Table S10: Prevalence of female SVAC by age and location for 1990, 2000, 2010, 2020, and 2023						
**Location**	**Age Range**	**1990**	**2000**	**2010**	**2020**	**2023**
Albania	45 to 49	11.6 (3.9–25.6)	11.4 (4.5–23.3)	10.8 (6.3–17.3)	10.4 (8.7–12.3)	10.4 (7.6–13.7)
Albania	50 to 54	12.0 (4.1–26.2)	11.7 (4.6–23.9)	11.3 (6.6–18.1)	11.0 (9.2–12.9)	11.0 (8.1–14.4)
Albania	55 to 59	10.4 (3.5–23.5)	10.1 (3.9–21.0)	9.5 (5.4–15.3)	9.0 (7.4–10.7)	9.0 (6.5–12.0)
Albania	60 to 64	10.2 (3.4–23.1)	9.9 (3.9–20.5)	9.3 (5.4–15.2)	8.9 (7.3–10.7)	8.9 (6.5–11.9)
Albania	65 to 69	8.9 (2.9–20.3)	8.6 (3.2–18.4)	8.0 (4.5–13.3)	7.5 (6.0– 9.2)	7.6 (5.3–10.2)
Albania	70 to 74	6.4 (2.0–15.6)	6.2 (2.2–13.8)	5.6 (3.0– 9.6)	5.2 (3.8– 7.0)	5.2 (3.4– 7.4)
Albania	75 to 79	7.1 (1.7–18.0)	7.0 (1.9–17.3)	6.8 (2.0–15.9)	6.8 (2.1–17.4)	6.9 (2.0–17.8)
Albania	80 to 84	5.0 (1.2–13.0)	4.9 (1.3–12.4)	4.7 (1.4–11.4)	4.8 (1.4–12.5)	4.8 (1.4–12.8)
Albania	85 to 89	4.5 (1.0–11.7)	4.4 (1.2–11.2)	4.3 (1.2–10.2)	4.3 (1.3–11.3)	4.3 (1.2–11.5)
Albania	90 to 94	4.0 (0.9–10.6)	3.9 (1.0–10.2)	3.8 (1.1– 9.3)	3.9 (1.1–10.2)	3.9 (1.1–10.5)
Albania	95 plus	4.0 (0.9–10.5)	3.9 (1.0–10.1)	3.8 (1.1– 9.2)	3.8 (1.1–10.1)	3.9 (1.1–10.4)
Albania	Age-standardized	10.2 (3.5–22.6)	10.0 (3.9–20.5)	9.4 (5.3–15.4)	9.0 (7.5–10.5)	9.0 (6.9–11.7)
Albania	All age	10.2 (3.4–22.9)	10.0 (3.9–20.7)	9.4 (5.3–15.3)	8.8 (7.5–10.2)	8.8 (6.9–11.1)
Bosnia and Herzegovina	20 to 24	14.8 (4.9–31.7)	14.8 (4.7–31.6)	14.8 (4.9–31.1)	14.8 (5.1–33.0)	14.8 (5.2–32.6)
Bosnia and Herzegovina	25 to 29	14.9 (5.0–32.0)	14.9 (4.8–31.9)	14.9 (4.9–31.4)	15.0 (5.2–33.3)	15.0 (5.3–32.9)

Supplementary Table S10: Prevalence of female SVAC by age and location for 1990, 2000, 2010, 2020, and 2023						
**Location**	**Age Range**	**1990**	**2000**	**2010**	**2020**	**2023**
Bosnia and Herzegovina	30 to 34	15.9 (5.4–33.7)	15.9 (5.1–33.7)	15.9 (5.3–33.1)	16.0 (5.6–35.1)	16.0 (5.7–34.5)
Bosnia and Herzegovina	35 to 39	15.9 (5.4–33.7)	15.9 (5.1–33.7)	16.0 (5.3–33.2)	16.0 (5.6–35.1)	16.0 (5.7–34.7)
Bosnia and Herzegovina	40 to 44	16.3 (5.5–34.4)	16.3 (5.3–34.4)	16.3 (5.5–33.8)	16.4 (5.8–35.8)	16.4 (5.9–35.4)
Bosnia and Herzegovina	45 to 49	15.7 (5.3–33.4)	15.8 (5.1–33.4)	15.8 (5.3–32.9)	15.9 (5.6–34.8)	15.9 (5.6–34.3)
Bosnia and Herzegovina	50 to 54	15.3 (5.1–32.6)	15.3 (4.9–32.6)	15.3 (5.1–32.0)	15.4 (5.4–34.0)	15.4 (5.5–33.5)
Bosnia and Herzegovina	55 to 59	14.7 (4.9–31.5)	14.7 (4.7–31.5)	14.7 (4.9–31.0)	14.8 (5.1–32.9)	14.8 (5.2–32.5)
Bosnia and Herzegovina	60 to 64	14.1 (4.7–30.5)	14.1 (4.5–30.5)	14.1 (4.6–30.0)	14.2 (4.9–31.9)	14.2 (5.0–31.5)
Bosnia and Herzegovina	65 to 69	13.0 (4.3–28.5)	13.1 (4.1–28.5)	13.1 (4.2–28.0)	13.2 (4.5–29.9)	13.2 (4.6–29.5)
Bosnia and Herzegovina	70 to 74	10.4 (3.3–23.4)	10.4 (3.2–23.4)	10.4 (3.3–23.0)	10.5 (3.5–24.6)	10.5 (3.5–24.3)
Bosnia and Herzegovina	75 to 79	9.3 (2.9–21.2)	9.3 (2.8–21.3)	9.4 (2.9–20.9)	9.4 (3.1–22.5)	9.5 (3.1–22.2)
Bosnia and Herzegovina	80 to 84	6.5 (2.0–15.4)	6.6 (1.9–15.4)	6.6 (2.0–15.2)	6.7 (2.1–16.4)	6.7 (2.2–16.2)
Bosnia and Herzegovina	85 to 89	5.9 (1.8–13.9)	5.9 (1.7–13.9)	5.9 (1.8–13.7)	6.0 (1.9–14.8)	6.0 (1.9–14.6)
Bosnia and Herzegovina	90 to 94	5.3 (1.6–12.6)	5.3 (1.5–12.6)	5.3 (1.6–12.4)	5.4 (1.7–13.4)	5.4 (1.7–13.2)
Bosnia and Herzegovina	95 plus	5.2 (1.6–12.4)	5.2 (1.5–12.5)	5.3 (1.6–12.3)	5.3 (1.7–13.3)	5.3 (1.7–13.1)
Bosnia and Herzegovina	Age-standardized	14.6 (4.9–31.3)	14.6 (4.7–31.3)	14.6 (4.9–30.8)	14.7 (5.1–32.7)	14.7 (5.2–32.2)

Supplementary Table S10: Prevalence of female SVAC by age and location for 1990, 2000, 2010, 2020, and 2023						
**Location**	**Age Range**	**1990**	**2000**	**2010**	**2020**	**2023**
Bosnia and Herzegovina	All age	14.8 (4.9–31.6)	14.6 (4.7–31.3)	14.4 (4.7–30.3)	14.1 (4.9–31.5)	14.0 (4.9–30.8)
Bulgaria	20 to 24	8.5 (3.8–16.0)	8.3 (4.3–14.0)	8.0 (5.9–10.7)	8.4 (4.4–13.9)	8.7 (3.9–15.5)
Bulgaria	25 to 29	9.6 (4.4–17.7)	9.4 (5.0–15.7)	9.1 (6.7–11.9)	9.4 (5.0–15.2)	9.6 (4.4–17.1)
Bulgaria	30 to 34	9.7 (4.4–17.9)	9.5 (5.0–15.9)	9.2 (6.8–12.0)	9.6 (5.1–15.5)	9.8 (4.5–17.5)
Bulgaria	35 to 39	11.4 (5.3–20.7)	11.2 (6.0–18.4)	10.9 (8.4–13.8)	11.1 (6.0–17.8)	11.3 (5.3–19.7)
Bulgaria	40 to 44	11.3 (5.2–20.6)	11.1 (6.0–18.2)	10.8 (8.3–13.7)	11.1 (6.0–17.7)	11.2 (5.3–19.7)
Bulgaria	45 to 49	10.9 (5.0–20.0)	10.8 (5.8–17.6)	10.4 (7.9–13.3)	10.7 (5.8–17.1)	10.9 (5.1–19.1)
Bulgaria	50 to 54	11.5 (5.3–20.8)	11.3 (6.0–18.4)	10.9 (8.4–13.9)	11.1 (6.0–17.7)	11.2 (5.3–19.6)
Bulgaria	55 to 59	9.5 (4.3–17.5)	9.3 (4.9–15.6)	9.0 (6.7–11.8)	9.3 (5.0–15.1)	9.5 (4.4–17.0)
Bulgaria	60 to 64	9.4 (4.3–17.4)	9.2 (4.9–15.4)	8.9 (6.6–11.7)	9.2 (4.9–14.9)	9.4 (4.3–16.7)
Bulgaria	65 to 69	8.1 (3.6–15.2)	7.9 (4.0–13.5)	7.7 (5.5–10.4)	8.0 (4.1–13.3)	8.2 (3.7–14.7)
Bulgaria	70 to 74	5.7 (2.4–11.5)	5.6 (2.7–10.2)	5.5 (3.5– 8.2)	5.9 (2.9–10.4)	6.1 (2.6–11.7)
Bulgaria	75 to 79	7.3 (1.8–18.8)	7.2 (2.0–17.8)	7.0 (2.1–16.4)	7.1 (2.3–17.9)	7.2 (2.2–18.3)
Bulgaria	80 to 84	5.1 (1.2–13.6)	5.1 (1.3–12.8)	4.9 (1.4–11.7)	5.0 (1.5–12.9)	5.0 (1.5–13.2)
Bulgaria	85 to 89	4.6 (1.1–12.3)	4.5 (1.2–11.6)	4.4 (1.3–10.6)	4.5 (1.4–11.6)	4.5 (1.3–11.9)

Supplementary Table S10: Prevalence of female SVAC by age and location for 1990, 2000, 2010, 2020, and 2023						
**Location**	**Age Range**	**1990**	**2000**	**2010**	**2020**	**2023**
Bulgaria	90 to 94	4.2 (1.0–11.1)	4.1 (1.1–10.5)	4.0 (1.2– 9.6)	4.0 (1.2–10.5)	4.1 (1.2–10.8)
Bulgaria	95 plus	4.1 (1.0–11.0)	4.1 (1.1–10.4)	3.9 (1.1– 9.5)	4.0 (1.2–10.4)	4.0 (1.2–10.7)
Bulgaria	Age-standardized	9.7 (4.5–17.6)	9.5 (5.0–15.7)	9.2 (6.8–12.0)	9.5 (5.9–14.6)	9.7 (5.4–16.3)
Bulgaria	All age	9.6 (4.4–17.4)	9.2 (4.9–15.3)	8.8 (6.5–11.7)	8.9 (6.6–12.9)	9.1 (6.5–14.1)
Croatia	20 to 24	9.0 (3.0–19.9)	8.0 (3.6–15.1)	7.1 (5.4– 9.1)	7.4 (3.9–12.0)	7.7 (3.6–14.2)
Croatia	25 to 29	9.5 (3.2–20.9)	8.7 (3.9–16.3)	7.9 (6.1–10.0)	8.2 (4.4–13.1)	8.4 (4.0–15.3)
Croatia	30 to 34	10.0 (3.4–21.9)	9.0 (4.1–16.8)	8.1 (6.3–10.2)	8.4 (4.5–13.4)	8.7 (4.1–15.7)
Croatia	35 to 39	10.7 (3.7–23.3)	10.2 (4.7–18.8)	9.6 (7.5–11.8)	9.7 (5.3–15.3)	9.9 (4.7–17.5)
Croatia	40 to 44	10.9 (3.8–23.6)	10.2 (4.7–18.9)	9.5 (7.5–11.8)	9.7 (5.3–15.3)	9.9 (4.7–17.5)
Croatia	45 to 49	10.5 (3.6–22.9)	9.9 (4.5–18.3)	9.2 (7.2–11.4)	9.4 (5.1–14.8)	9.6 (4.6–17.0)
Croatia	50 to 54	10.5 (3.6–22.9)	10.2 (4.6–18.7)	9.6 (7.6–11.9)	9.8 (5.3–15.4)	9.9 (4.7–17.4)
Croatia	55 to 59	9.5 (3.2–20.9)	8.7 (3.9–16.2)	7.9 (6.1– 9.9)	8.1 (4.4–13.0)	8.4 (3.9–15.2)
Croatia	60 to 64	9.2 (3.1–20.4)	8.5 (3.8–16.0)	7.8 (6.0– 9.9)	8.1 (4.3–12.9)	8.3 (3.9–15.1)
Croatia	65 to 69	8.3 (2.7–18.5)	7.5 (3.3–14.1)	6.7 (5.0– 8.7)	7.0 (3.7–11.4)	7.2 (3.3–13.4)
Croatia	70 to 74	6.3 (2.0–14.2)	5.5 (2.3–10.4)	4.8 (3.3– 6.7)	5.0 (2.5– 8.6)	5.3 (2.3– 9.8)

Supplementary Table S10: Prevalence of female SVAC by age and location for 1990, 2000, 2010, 2020, and 2023						
**Location**	**Age Range**	**1990**	**2000**	**2010**	**2020**	**2023**
Croatia	75 to 79	6.3 (1.9–14.9)	6.3 (1.8–14.9)	6.3 (1.9–14.5)	6.4 (2.0–15.7)	6.4 (2.0–15.5)
Croatia	80 to 84	4.4 (1.3–10.7)	4.4 (1.3–10.6)	4.4 (1.3–10.4)	4.4 (1.4–11.2)	4.4 (1.4–11.1)
Croatia	85 to 89	4.0 (1.2– 9.6)	4.0 (1.1– 9.6)	3.9 (1.2– 9.3)	4.0 (1.2–10.1)	4.0 (1.2–10.0)
Croatia	90 to 94	3.6 (1.0– 8.7)	3.6 (1.0– 8.6)	3.5 (1.0– 8.4)	3.6 (1.1– 9.2)	3.6 (1.1– 9.0)
Croatia	95 plus	3.5 (1.0– 8.6)	3.5 (1.0– 8.6)	3.5 (1.0– 8.4)	3.5 (1.1– 9.1)	3.6 (1.1– 8.9)
Croatia	Age-standardized	9.5 (3.3–20.7)	8.8 (3.9–16.7)	8.1 (6.2–10.3)	8.3 (5.1–12.9)	8.5 (4.5–15.0)
Croatia	All age	9.3 (3.2–20.3)	8.6 (3.8–16.2)	7.8 (5.8–10.1)	7.8 (5.4–11.7)	8.0 (4.9–13.5)
Czechia	20 to 24	15.4 (11.2–20.5)	18.2 (14.8–21.9)	13.3 (9.6–18.1)	10.5 (5.0–18.7)	10.6 (4.6–20.2)
Czechia	25 to 29	14.0 (10.1–18.6)	17.3 (14.0–20.8)	14.9 (11.4–19.2)	11.9 (5.9–20.5)	11.9 (5.4–22.3)
Czechia	30 to 34	15.1 (11.0–20.0)	17.8 (14.4–21.4)	16.3 (13.0–20.4)	15.0 (8.2–24.0)	15.1 (7.4–26.4)
Czechia	35 to 39	13.8 (9.8–18.7)	17.5 (14.2–21.1)	16.5 (12.9–20.1)	17.5 (11.8–25.4)	18.1 (10.6–29.1)
Czechia	40 to 44	13.5 (9.6–18.4)	17.2 (14.0–20.8)	15.4 (11.9–19.1)	14.9 (9.3–22.8)	15.4 (8.4–25.8)
Czechia	45 to 49	14.1 (10.3–18.7)	16.7 (13.6–20.2)	14.7 (11.3–18.6)	14.6 (9.1–22.0)	15.1 (8.6–24.3)
Czechia	50 to 54	13.0 (9.2–17.9)	16.2 (13.0–19.8)	14.8 (11.4–18.4)	14.1 (8.8–21.9)	14.6 (8.0–24.6)
Czechia	55 to 59	12.4 (8.3–17.9)	14.0 (11.1–17.4)	13.1 (9.8–16.6)	13.5 (8.2–21.6)	14.0 (7.5–24.5)

Supplementary Table S10: Prevalence of female SVAC by age and location for 1990, 2000, 2010, 2020, and 2023						
**Location**	**Age Range**	**1990**	**2000**	**2010**	**2020**	**2023**
Czechia	60 to 64	12.1 (7.8–17.9)	14.1 (11.2–17.5)	13.1 (9.8–16.7)	13.8 (8.5–21.7)	14.4 (7.8–24.3)
Czechia	65 to 69	10.6 (6.4–16.7)	12.1 (8.8–16.2)	11.9 (8.6–15.5)	13.1 (7.8–20.8)	13.7 (7.3–23.9)
Czechia	70 to 74	8.3 (4.4–14.0)	9.2 (5.1–15.0)	10.4 (6.4–15.6)	12.1 (7.0–19.3)	12.6 (6.7–22.0)
Czechia	75 to 79	7.9 (4.0–13.5)	9.7 (4.6–16.9)	12.3 (6.5–20.3)	13.4 (7.4–22.1)	13.3 (6.8–22.6)
Czechia	80 to 84	7.5 (2.4–17.2)	8.8 (3.0–18.8)	10.8 (4.7–20.3)	12.4 (6.7–20.7)	12.5 (6.6–20.9)
Czechia	85 to 89	5.9 (1.8–13.8)	6.1 (1.8–14.4)	6.4 (1.9–14.6)	6.6 (2.1–16.3)	6.7 (2.2–16.1)
Czechia	90 to 94	5.3 (1.6–12.4)	5.5 (1.6–13.0)	5.7 (1.7–13.2)	5.9 (1.9–14.7)	6.0 (1.9–14.6)
Czechia	95 plus	5.2 (1.6–12.3)	5.4 (1.6–12.8)	5.6 (1.7–13.0)	5.9 (1.8–14.5)	5.9 (1.9–14.4)
Czechia	Age-standardized	13.2 (9.9–17.2)	15.9 (12.8–19.4)	14.3 (11.7–17.4)	13.7 (10.5–17.8)	14.0 (9.9–19.9)
Czechia	All age	12.7 (9.2–17.0)	15.2 (12.2–18.8)	13.9 (11.5–16.7)	13.6 (11.0–17.2)	14.0 (10.5–18.8)
Hungary	20 to 24	11.3 (3.9–24.5)	10.6 (4.8–19.5)	9.7 (7.4–12.5)	10.0 (5.4–16.1)	10.2 (4.8–18.6)
Hungary	25 to 29	12.1 (4.2–25.9)	11.7 (5.4–21.4)	11.1 (8.5–14.0)	11.2 (6.1–17.7)	11.3 (5.4–20.3)
Hungary	30 to 34	12.8 (4.5–27.2)	12.0 (5.6–22.0)	11.2 (8.6–14.2)	11.5 (6.2–18.1)	11.7 (5.6–20.9)
Hungary	35 to 39	13.7 (4.9–28.9)	13.6 (6.4–24.6)	13.3 (10.4–16.6)	13.3 (7.3–20.7)	13.4 (6.5–23.3)
Hungary	40 to 44	14.0 (5.0–29.4)	13.8 (6.4–24.7)	13.3 (10.4–16.5)	13.4 (7.4–20.7)	13.4 (6.5–23.5)

Supplementary Table S10: Prevalence of female SVAC by age and location for 1990, 2000, 2010, 2020, and 2023						
**Location**	**Age Range**	**1990**	**2000**	**2010**	**2020**	**2023**
Hungary	45 to 49	13.6 (4.8–28.6)	13.3 (6.2–24.0)	12.8 (10.0–15.9)	12.9 (7.1–20.1)	13.0 (6.3–22.7)
Hungary	50 to 54	13.6 (4.8–28.7)	13.6 (6.4–24.5)	13.4 (10.5–16.6)	13.3 (7.4–20.7)	13.4 (6.5–23.4)
Hungary	55 to 59	12.3 (4.3–26.3)	11.7 (5.4–21.4)	11.0 (8.4–13.9)	11.2 (6.1–17.7)	11.4 (5.4–20.3)
Hungary	60 to 64	12.0 (4.1–25.7)	11.5 (5.3–21.1)	10.9 (8.4–13.8)	11.1 (6.0–17.5)	11.2 (5.3–20.1)
Hungary	65 to 69	10.8 (3.7–23.4)	10.1 (4.6–18.7)	9.3 (7.0–12.1)	9.6 (5.1–15.5)	9.8 (4.6–17.9)
Hungary	70 to 74	8.2 (2.7–18.2)	7.4 (3.2–13.7)	6.6 (4.6– 9.3)	6.9 (3.5–11.7)	7.1 (3.1–13.1)
Hungary	75 to 79	8.0 (2.5–18.6)	8.0 (2.4–18.5)	8.0 (2.5–18.1)	8.1 (2.6–19.5)	8.1 (2.6–19.3)
Hungary	80 to 84	5.6 (1.7–13.4)	5.6 (1.6–13.4)	5.6 (1.7–13.1)	5.7 (1.8–14.2)	5.7 (1.8–13.9)
Hungary	85 to 89	5.1 (1.5–12.1)	5.0 (1.4–12.1)	5.1 (1.5–11.8)	5.1 (1.6–12.8)	5.1 (1.6–12.6)
Hungary	90 to 94	4.6 (1.4–11.0)	4.6 (1.3–10.9)	4.6 (1.3–10.7)	4.6 (1.4–11.6)	4.6 (1.5–11.4)
Hungary	95 plus	4.5 (1.3–10.9)	4.5 (1.3–10.8)	4.5 (1.3–10.6)	4.6 (1.4–11.5)	4.6 (1.4–11.3)
Hungary	Age-standardized	12.2 (4.3–25.8)	11.8 (5.4–21.7)	11.2 (8.6–14.4)	11.3 (6.9–17.5)	11.5 (6.0–19.9)
Hungary	All age	11.9 (4.2–25.2)	11.4 (5.2–21.0)	10.8 (8.1–14.0)	10.8 (7.4–16.1)	10.9 (6.7–18.2)
Montenegro	20 to 24	7.8 (2.5–17.9)	7.2 (2.6–15.1)	6.3 (3.5–10.7)	5.9 (4.5– 7.5)	6.0 (3.8– 8.6)
Montenegro	25 to 29	8.2 (2.6–18.7)	7.8 (2.8–16.1)	7.1 (4.0–11.8)	6.6 (5.2– 8.2)	6.7 (4.3– 9.6)

Supplementary Table S10: Prevalence of female SVAC by age and location for 1990, 2000, 2010, 2020, and 2023						
**Location**	**Age Range**	**1990**	**2000**	**2010**	**2020**	**2023**
Montenegro	30 to 34	8.8 (2.8–19.8)	8.2 (2.9–16.8)	7.2 (4.0–12.0)	6.7 (5.2– 8.3)	6.8 (4.4– 9.7)
Montenegro	35 to 39	9.3 (3.0–21.0)	9.1 (3.3–18.5)	8.5 (4.9–14.0)	8.1 (6.5– 9.9)	8.2 (5.4–11.6)
Montenegro	40 to 44	9.5 (3.1–21.5)	9.2 (3.3–18.9)	8.6 (4.9–14.1)	8.1 (6.5–10.0)	8.2 (5.4–11.7)
Montenegro	45 to 49	9.3 (3.0–20.9)	9.0 (3.2–18.3)	8.3 (4.8–13.7)	7.9 (6.3– 9.7)	8.0 (5.2–11.3)
Montenegro	50 to 54	9.2 (3.0–20.8)	9.1 (3.3–18.6)	8.7 (5.0–14.2)	8.3 (6.7–10.2)	8.4 (5.5–11.9)
Montenegro	55 to 59	8.5 (2.7–19.3)	8.1 (2.9–16.6)	7.3 (4.1–12.1)	6.9 (5.4– 8.5)	7.0 (4.5– 9.9)
Montenegro	60 to 64	8.2 (2.6–18.7)	7.9 (2.8–16.3)	7.2 (4.1–12.0)	6.8 (5.3– 8.4)	6.9 (4.4– 9.8)
Montenegro	65 to 69	7.4 (2.4–17.0)	6.9 (2.5–14.5)	6.2 (3.4–10.4)	5.8 (4.4– 7.3)	5.9 (3.7– 8.4)
Montenegro	70 to 74	5.6 (1.8–12.9)	5.1 (1.8–11.1)	4.3 (2.3– 7.6)	4.0 (2.8– 5.6)	4.1 (2.5– 6.3)
Montenegro	75 to 79	5.5 (1.6–13.0)	5.4 (1.6–13.0)	5.4 (1.6–12.6)	5.4 (1.7–13.5)	5.4 (1.7–13.3)
Montenegro	80 to 84	3.8 (1.1– 9.2)	3.8 (1.1– 9.2)	3.8 (1.1– 8.9)	3.8 (1.2– 9.6)	3.8 (1.2– 9.5)
Montenegro	85 to 89	3.4 (1.0– 8.3)	3.4 (0.9– 8.3)	3.4 (1.0– 8.0)	3.4 (1.0– 8.6)	3.4 (1.0– 8.5)
Montenegro	90 to 94	3.1 (0.9– 7.5)	3.0 (0.9– 7.5)	3.0 (0.9– 7.2)	3.0 (0.9– 7.8)	3.0 (0.9– 7.7)
Montenegro	95 plus	3.0 (0.9– 7.4)	3.0 (0.8– 7.4)	3.0 (0.9– 7.2)	3.0 (0.9– 7.7)	3.0 (0.9– 7.6)
Montenegro	Age-standardized	8.3 (2.7–18.8)	7.9 (2.8–16.6)	7.3 (4.1–12.2)	6.8 (5.6– 8.3)	6.9 (4.8– 9.6)

Supplementary Table S10: Prevalence of female SVAC by age and location for 1990, 2000, 2010, 2020, and 2023						
**Location**	**Age Range**	**1990**	**2000**	**2010**	**2020**	**2023**
Montenegro	All age	8.3 (2.7–18.8)	7.9 (2.8–16.5)	7.1 (4.0–12.0)	6.7 (5.5– 8.0)	6.7 (4.8– 9.2)
North Macedonia	20 to 24	12.1 (6.0–20.8)	11.9 (6.8–18.8)	11.7 (8.8–15.1)	11.9 (5.9–20.7)	12.0 (5.1–23.2)
North Macedonia	25 to 29	12.6 (4.5–26.5)	12.4 (5.1–24.3)	12.0 (7.1–19.0)	12.0 (7.0–18.4)	12.1 (6.3–20.2)
North Macedonia	30 to 34	13.5 (4.1–30.9)	13.2 (4.5–28.4)	12.6 (6.0–22.7)	12.0 (8.2–17.1)	11.9 (7.8–17.5)
North Macedonia	35 to 39	14.3 (3.7–34.5)	14.2 (4.2–32.4)	14.0 (4.6–29.6)	14.0 (4.8–31.5)	14.1 (4.8–32.8)
North Macedonia	40 to 44	14.8 (3.9–35.7)	14.7 (4.3–33.4)	14.4 (4.8–30.5)	14.5 (5.0–32.4)	14.6 (4.9–33.3)
North Macedonia	45 to 49	14.4 (3.7–34.6)	14.3 (4.2–32.7)	14.1 (4.6–29.8)	14.1 (4.9–31.7)	14.2 (4.8–32.6)
North Macedonia	50 to 54	14.1 (3.7–33.6)	13.9 (4.1–31.9)	13.7 (4.5–29.2)	13.8 (4.7–31.1)	13.9 (4.7–32.3)
North Macedonia	55 to 59	13.7 (3.5–33.4)	13.5 (3.9–31.2)	13.3 (4.3–28.4)	13.4 (4.6–30.2)	13.4 (4.6–30.9)
North Macedonia	60 to 64	13.2 (3.4–32.3)	13.0 (3.8–30.3)	12.8 (4.2–27.6)	12.9 (4.4–29.4)	13.0 (4.3–29.9)
North Macedonia	65 to 69	12.2 (3.1–30.1)	12.1 (3.5–28.2)	11.9 (3.8–25.8)	12.0 (4.0–27.6)	12.1 (4.0–28.2)
North Macedonia	70 to 74	9.8 (2.4–25.4)	9.7 (2.7–23.1)	9.5 (3.0–21.1)	9.6 (3.1–22.7)	9.6 (3.1–23.2)
North Macedonia	75 to 79	8.8 (2.1–22.3)	8.7 (2.4–21.0)	8.5 (2.6–19.2)	8.6 (2.8–20.6)	8.7 (2.7–21.1)
North Macedonia	80 to 84	6.2 (1.5–16.5)	6.1 (1.6–15.3)	6.0 (1.8–13.9)	6.0 (1.9–15.0)	6.1 (1.9–15.3)
North Macedonia	85 to 89	5.6 (1.3–15.2)	5.5 (1.5–13.9)	5.4 (1.6–12.5)	5.4 (1.7–13.6)	5.5 (1.7–13.9)

Supplementary Table S10: Prevalence of female SVAC by age and location for 1990, 2000, 2010, 2020, and 2023						
**Location**	**Age Range**	**1990**	**2000**	**2010**	**2020**	**2023**
North Macedonia	90 to 94	5.0 (1.2–13.5)	5.0 (1.3–12.6)	4.8 (1.4–11.3)	4.9 (1.5–12.3)	4.9 (1.5–12.6)
North Macedonia	95 plus	5.0 (1.1–13.3)	4.9 (1.3–12.4)	4.8 (1.4–11.2)	4.8 (1.5–12.2)	4.9 (1.5–12.5)
North Macedonia	Age-standardized	13.0 (4.4–27.9)	12.8 (4.8–26.5)	12.5 (5.6–23.6)	12.5 (7.3–22.9)	12.6 (7.3–22.6)
North Macedonia	All age	13.1 (4.5–28.2)	12.9 (4.7–26.7)	12.5 (5.4–24.0)	12.4 (6.2–24.7)	12.4 (6.0–24.5)
Poland	20 to 24	12.6 (5.5–23.5)	11.7 (8.1–15.9)	9.7 (7.4–12.1)	9.0 (4.9–15.6)	9.1 (4.2–17.3)
Poland	25 to 29	13.5 (6.0–24.8)	13.0 (9.2–17.3)	10.9 (8.6–13.3)	9.8 (5.5–16.7)	9.9 (4.8–18.5)
Poland	30 to 34	14.4 (6.5–26.4)	13.7 (9.7–18.1)	11.3 (9.0–13.7)	10.3 (6.1–16.8)	10.4 (5.3–18.7)
Poland	35 to 39	15.1 (6.8–27.4)	14.9 (10.8–19.5)	12.9 (10.4–15.4)	11.5 (7.5–17.1)	11.4 (6.5–18.6)
Poland	40 to 44	14.9 (6.7–27.2)	14.5 (10.4–19.1)	12.7 (10.2–15.2)	11.7 (6.8–19.0)	11.7 (5.8–21.1)
Poland	45 to 49	11.8 (6.4–19.1)	13.1 (9.7–17.1)	12.3 (9.8–14.8)	11.4 (6.6–18.9)	11.4 (5.7–21.0)
Poland	50 to 54	11.3 (6.3–18.1)	12.8 (9.6–16.6)	12.6 (10.1–15.1)	11.8 (6.9–19.3)	11.8 (5.9–21.4)
Poland	55 to 59	10.1 (5.1–17.5)	9.8 (7.1–13.1)	9.9 (7.7–12.3)	10.4 (5.8–17.6)	10.6 (5.1–19.9)
Poland	60 to 64	10.3 (4.6–19.2)	9.7 (6.9–13.0)	9.8 (7.6–12.1)	10.3 (5.8–17.6)	10.5 (5.0–19.8)
Poland	65 to 69	10.2 (4.3–20.1)	9.3 (6.1–13.3)	8.6 (6.5–11.0)	8.9 (4.8–15.5)	9.1 (4.2–17.7)
Poland	70 to 74	8.3 (2.8–18.4)	7.6 (3.3–14.6)	6.8 (4.3–10.1)	7.0 (3.4–12.4)	7.1 (3.0–13.7)

Supplementary Table S10: Prevalence of female SVAC by age and location for 1990, 2000, 2010, 2020, and 2023						
**Location**	**Age Range**	**1990**	**2000**	**2010**	**2020**	**2023**
Poland	75 to 79	8.2 (2.6–18.8)	8.3 (3.0–17.3)	8.3 (3.9–15.4)	8.5 (3.8–16.2)	8.5 (3.3–17.1)
Poland	80 to 84	6.0 (1.9–14.1)	6.4 (2.1–14.4)	7.0 (2.9–14.0)	7.3 (3.5–13.7)	7.4 (3.3–13.9)
Poland	85 to 89	5.0 (1.5–12.0)	5.0 (1.4–12.0)	5.0 (1.5–11.8)	5.1 (1.6–12.9)	5.2 (1.6–13.3)
Poland	90 to 94	4.5 (1.3–10.9)	4.5 (1.3–10.9)	4.5 (1.3–10.7)	4.6 (1.4–11.7)	4.7 (1.4–12.1)
Poland	95 plus	4.5 (1.3–10.8)	4.5 (1.3–10.8)	4.5 (1.3–10.6)	4.6 (1.4–11.6)	4.6 (1.4–12.0)
Poland	Age-standardized	12.3 (5.6–22.3)	12.1 (8.5–16.5)	10.8 (8.9–12.9)	10.2 (6.7–16.1)	10.3 (6.0–17.9)
Poland	All age	12.2 (5.5–22.1)	11.8 (8.2–16.3)	10.5 (8.8–12.5)	9.9 (7.0–15.0)	10.0 (6.6–16.4)
Romania	20 to 24	7.8 (2.6–17.6)	6.9 (3.1–13.0)	6.0 (4.7– 7.6)	6.3 (3.4–10.2)	6.6 (3.1–12.1)
Romania	25 to 29	8.3 (2.8–18.5)	7.6 (3.4–14.3)	6.8 (5.3– 8.5)	7.1 (3.8–11.3)	7.3 (3.4–13.1)
Romania	30 to 34	8.7 (2.9–19.4)	7.8 (3.5–14.7)	6.9 (5.4– 8.6)	7.2 (3.9–11.5)	7.5 (3.5–13.5)
Romania	35 to 39	9.4 (3.1–20.7)	8.8 (4.0–16.5)	8.2 (6.5–10.0)	8.4 (4.5–13.3)	8.5 (4.0–15.1)
Romania	40 to 44	9.5 (3.2–21.0)	8.9 (4.0–16.6)	8.2 (6.5– 9.9)	8.4 (4.6–13.3)	8.6 (4.0–15.2)
Romania	45 to 49	9.2 (3.1–20.4)	8.6 (3.9–16.1)	7.9 (6.3– 9.6)	8.1 (4.4–12.9)	8.3 (3.9–14.7)
Romania	50 to 54	9.2 (3.1–20.4)	8.8 (4.0–16.5)	8.3 (6.6–10.1)	8.4 (4.6–13.4)	8.6 (4.0–15.1)
Romania	55 to 59	8.3 (2.8–18.6)	7.5 (3.4–14.2)	6.8 (5.3– 8.4)	7.0 (3.8–11.2)	7.3 (3.4–13.1)

Supplementary Table S10: Prevalence of female SVAC by age and location for 1990, 2000, 2010, 2020, and 2023						
**Location**	**Age Range**	**1990**	**2000**	**2010**	**2020**	**2023**
Romania	60 to 64	8.1 (2.7–18.1)	7.4 (3.3–14.0)	6.7 (5.3– 8.4)	7.0 (3.7–11.1)	7.2 (3.4–12.9)
Romania	65 to 69	7.2 (2.4–16.4)	6.5 (2.9–12.4)	5.8 (4.4– 7.3)	6.0 (3.2– 9.8)	6.2 (2.9–11.5)
Romania	70 to 74	5.5 (1.7–12.6)	4.7 (2.0– 9.2)	4.1 (2.9– 5.6)	4.3 (2.2– 7.4)	4.5 (2.0– 8.7)
Romania	75 to 79	5.6 (1.7–13.3)	5.6 (1.6–13.2)	5.5 (1.7–12.9)	5.6 (1.8–14.0)	5.6 (1.8–13.8)
Romania	80 to 84	3.9 (1.1– 9.4)	3.9 (1.1– 9.4)	3.9 (1.1– 9.1)	3.9 (1.2– 9.9)	3.9 (1.2– 9.8)
Romania	85 to 89	3.5 (1.0– 8.5)	3.5 (1.0– 8.4)	3.5 (1.0– 8.2)	3.5 (1.1– 8.9)	3.5 (1.1– 8.8)
Romania	90 to 94	3.1 (0.9– 7.7)	3.1 (0.9– 7.6)	3.1 (0.9– 7.4)	3.1 (1.0– 8.1)	3.1 (1.0– 8.0)
Romania	95 plus	3.1 (0.9– 7.6)	3.1 (0.9– 7.6)	3.1 (0.9– 7.4)	3.1 (0.9– 8.0)	3.1 (1.0– 7.9)
Romania	Age-standardized	8.3 (2.8–18.3)	7.6 (3.4–14.5)	6.9 (5.4– 8.7)	7.2 (4.4–11.1)	7.4 (3.9–12.9)
Romania	All age	8.2 (2.8–18.2)	7.5 (3.3–14.3)	6.7 (5.2– 8.6)	6.9 (4.7–10.4)	7.0 (4.2–12.0)
Serbia	20 to 24	12.7 (4.1–27.8)	12.6 (3.9–27.7)	12.6 (4.1–27.1)	12.7 (4.3–28.9)	12.7 (4.4–28.5)
Serbia	25 to 29	13.0 (4.2–28.4)	12.9 (4.0–28.3)	12.9 (4.2–27.7)	13.0 (4.4–29.5)	13.0 (4.5–29.1)
Serbia	30 to 34	14.0 (4.6–30.3)	14.0 (4.4–30.2)	13.9 (4.6–29.6)	14.0 (4.8–31.5)	14.0 (4.9–31.1)
Serbia	35 to 39	14.2 (4.7–30.6)	14.1 (4.5–30.5)	14.1 (4.6–29.9)	14.2 (4.9–31.8)	14.2 (5.0–31.4)
Serbia	40 to 44	14.6 (4.9–31.4)	14.6 (4.6–31.3)	14.5 (4.8–30.7)	14.6 (5.1–32.6)	14.6 (5.1–32.3)

Supplementary Table S10: Prevalence of female SVAC by age and location for 1990, 2000, 2010, 2020, and 2023						
**Location**	**Age Range**	**1990**	**2000**	**2010**	**2020**	**2023**
Serbia	45 to 49	14.2 (4.7–30.7)	14.2 (4.5–30.6)	14.1 (4.6–30.0)	14.2 (4.9–31.9)	14.3 (5.0–31.6)
Serbia	50 to 54	13.9 (4.6–30.0)	13.8 (4.4–30.0)	13.8 (4.5–29.3)	13.9 (4.8–31.3)	13.9 (4.8–30.9)
Serbia	55 to 59	13.4 (4.4–29.1)	13.3 (4.2–29.1)	13.3 (4.3–28.4)	13.4 (4.6–30.3)	13.4 (4.6–30.0)
Serbia	60 to 64	12.9 (4.2–28.2)	12.9 (4.0–28.2)	12.8 (4.2–27.6)	12.9 (4.4–29.5)	13.0 (4.5–29.1)
Serbia	65 to 69	11.9 (3.8–26.4)	11.9 (3.7–26.4)	11.9 (3.8–25.8)	12.0 (4.0–27.6)	12.0 (4.1–27.3)
Serbia	70 to 74	9.5 (3.0–21.6)	9.5 (2.8–21.6)	9.5 (3.0–21.1)	9.6 (3.1–22.7)	9.6 (3.2–22.4)
Serbia	75 to 79	8.5 (2.6–19.6)	8.5 (2.5–19.6)	8.5 (2.6–19.2)	8.6 (2.8–20.7)	8.6 (2.8–20.4)
Serbia	80 to 84	6.0 (1.8–14.1)	6.0 (1.7–14.1)	6.0 (1.8–13.8)	6.0 (1.9–15.0)	6.1 (1.9–14.8)
Serbia	85 to 89	5.3 (1.6–12.7)	5.3 (1.5–12.7)	5.4 (1.6–12.5)	5.4 (1.7–13.5)	5.4 (1.7–13.3)
Serbia	90 to 94	4.8 (1.4–11.5)	4.8 (1.4–11.5)	4.8 (1.4–11.3)	4.9 (1.5–12.3)	4.9 (1.5–12.1)
Serbia	95 plus	4.7 (1.4–11.4)	4.8 (1.4–11.4)	4.8 (1.4–11.2)	4.8 (1.5–12.1)	4.8 (1.5–12.0)
Serbia	Age-standardized	13.0 (4.3–28.4)	13.0 (4.1–28.3)	13.0 (4.2–27.7)	13.0 (4.4–29.6)	13.1 (4.5–29.2)
Serbia	All age	13.0 (4.3–28.4)	12.8 (4.0–28.0)	12.5 (4.1–26.9)	12.5 (4.2–28.4)	12.4 (4.3–28.0)
Slovakia	20 to 24	10.4 (3.5–22.7)	9.6 (4.3–17.8)	8.7 (6.7–11.2)	9.0 (4.8–14.5)	9.3 (4.3–16.9)
Slovakia	25 to 29	11.0 (3.8–23.9)	10.5 (4.8–19.4)	9.9 (7.6–12.4)	10.1 (5.4–15.9)	10.2 (4.9–18.3)

Supplementary Table S10: Prevalence of female SVAC by age and location for 1990, 2000, 2010, 2020, and 2023						
**Location**	**Age Range**	**1990**	**2000**	**2010**	**2020**	**2023**
Slovakia	30 to 34	11.7 (4.0–25.1)	10.9 (5.0–20.1)	10.1 (7.8–12.6)	10.3 (5.6–16.3)	10.6 (5.0–19.0)
Slovakia	35 to 39	12.6 (4.4–26.8)	12.4 (5.7–22.5)	12.0 (9.4–14.8)	12.0 (6.6–18.8)	12.1 (5.9–21.2)
Slovakia	40 to 44	12.7 (4.5–27.1)	12.4 (5.7–22.4)	11.8 (9.3–14.7)	11.9 (6.6–18.7)	12.1 (5.8–21.2)
Slovakia	45 to 49	12.4 (4.3–26.4)	12.0 (5.5–21.8)	11.4 (9.0–14.2)	11.6 (6.3–18.1)	11.7 (5.7–20.6)
Slovakia	50 to 54	12.4 (4.4–26.5)	12.4 (5.7–22.4)	12.1 (9.5–14.9)	12.1 (6.6–18.8)	12.1 (5.9–21.2)
Slovakia	55 to 59	11.2 (3.9–24.3)	10.6 (4.8–19.5)	9.9 (7.6–12.4)	10.1 (5.5–16.0)	10.3 (4.9–18.5)
Slovakia	60 to 64	10.9 (3.7–23.7)	10.4 (4.7–19.2)	9.7 (7.5–12.3)	9.9 (5.4–15.8)	10.1 (4.8–18.2)
Slovakia	65 to 69	9.8 (3.3–21.6)	9.1 (4.1–17.0)	8.4 (6.3–10.8)	8.6 (4.6–14.0)	8.8 (4.1–16.2)
Slovakia	70 to 74	7.5 (2.4–16.7)	6.7 (2.9–12.6)	5.9 (4.1– 8.2)	6.2 (3.1–10.5)	6.5 (2.8–12.1)
Slovakia	75 to 79	7.4 (2.3–17.1)	7.3 (2.1–17.1)	7.3 (2.2–16.7)	7.4 (2.4–18.0)	7.4 (2.4–17.8)
Slovakia	80 to 84	5.1 (1.5–12.3)	5.1 (1.5–12.3)	5.1 (1.5–12.0)	5.2 (1.6–13.0)	5.2 (1.6–12.8)
Slovakia	85 to 89	4.6 (1.4–11.1)	4.6 (1.3–11.1)	4.6 (1.4–10.8)	4.6 (1.4–11.7)	4.7 (1.5–11.6)
Slovakia	90 to 94	4.2 (1.2–10.0)	4.2 (1.2–10.0)	4.1 (1.2– 9.8)	4.2 (1.3–10.6)	4.2 (1.3–10.5)
Slovakia	95 plus	4.1 (1.2–10.0)	4.1 (1.2– 9.9)	4.1 (1.2– 9.7)	4.1 (1.3–10.5)	4.2 (1.3–10.4)
Slovakia	Age-standardized	11.1 (3.9–23.8)	10.6 (4.8–19.8)	10.0 (7.7–12.8)	10.2 (6.2–15.7)	10.4 (5.5–18.1)

Supplementary Table S10: Prevalence of female SVAC by age and location for 1990, 2000, 2010, 2020, and 2023						
**Location**	**Age Range**	**1990**	**2000**	**2010**	**2020**	**2023**
Slovakia	All age	11.0 (3.8–23.6)	10.5 (4.7–19.6)	9.8 (7.5–12.7)	9.9 (6.5–15.0)	10.0 (5.8–17.1)
Slovenia	20 to 24	12.2 (4.2–26.1)	11.5 (5.3–21.0)	10.7 (8.0–13.8)	10.9 (5.9–17.6)	11.1 (5.3–20.1)
Slovenia	25 to 29	13.0 (4.5–27.6)	12.6 (5.8–22.8)	12.0 (9.2–15.3)	12.2 (6.6–19.2)	12.3 (5.9–21.9)
Slovenia	30 to 34	13.7 (4.8–28.8)	13.0 (6.0–23.4)	12.2 (9.3–15.4)	12.4 (6.8–19.6)	12.6 (6.1–22.4)
Slovenia	35 to 39	14.7 (5.2–30.7)	14.7 (6.9–26.3)	14.5 (11.3–18.1)	14.4 (8.0–22.4)	14.4 (7.1–25.2)
Slovenia	40 to 44	14.9 (5.3–31.1)	14.8 (7.0–26.4)	14.3 (11.2–17.9)	14.4 (8.0–22.3)	14.4 (7.1–25.2)
Slovenia	45 to 49	14.5 (5.2–30.3)	14.3 (6.7–25.7)	13.9 (10.8–17.4)	14.0 (7.7–21.7)	14.0 (6.8–24.5)
Slovenia	50 to 54	14.6 (5.2–30.4)	14.7 (7.0–26.3)	14.6 (11.4–18.2)	14.5 (8.1–22.4)	14.5 (7.1–25.2)
Slovenia	55 to 59	13.2 (4.6–27.9)	12.6 (5.8–22.9)	11.9 (9.1–15.1)	12.1 (6.6–19.2)	12.3 (5.9–21.9)
Slovenia	60 to 64	12.8 (4.5–27.3)	12.4 (5.7–22.5)	11.8 (9.0–15.0)	12.0 (6.5–18.9)	12.1 (5.8–21.5)
Slovenia	65 to 69	11.5 (3.9–24.9)	10.9 (5.0–20.0)	10.1 (7.6–13.2)	10.4 (5.6–16.8)	10.6 (5.0–19.2)
Slovenia	70 to 74	8.8 (2.9–19.4)	8.0 (3.5–14.7)	7.2 (5.0–10.1)	7.5 (3.8–12.6)	7.7 (3.4–14.2)
Slovenia	75 to 79	8.6 (2.7–19.7)	8.6 (2.5–19.7)	8.6 (2.6–19.3)	8.7 (2.8–20.8)	8.7 (2.9–20.5)
Slovenia	80 to 84	6.0 (1.8–14.3)	6.0 (1.7–14.3)	6.0 (1.8–14.0)	6.1 (1.9–15.1)	6.1 (2.0–14.9)
Slovenia	85 to 89	5.4 (1.6–12.9)	5.4 (1.6–12.9)	5.4 (1.6–12.6)	5.5 (1.7–13.7)	5.5 (1.7–13.5)

Supplementary Table S10: Prevalence of female SVAC by age and location for 1990, 2000, 2010, 2020, and 2023						
**Location**	**Age Range**	**1990**	**2000**	**2010**	**2020**	**2023**
Slovenia	90 to 94	4.9 (1.5–11.7)	4.9 (1.4–11.7)	4.9 (1.5–11.4)	4.9 (1.5–12.4)	4.9 (1.6–12.2)
Slovenia	95 plus	4.8 (1.4–11.6)	4.8 (1.4–11.6)	4.8 (1.4–11.3)	4.9 (1.5–12.3)	4.9 (1.5–12.1)
Slovenia	Age-standardized	13.0 (4.6–27.4)	12.7 (5.9–23.2)	12.1 (9.2–15.6)	12.3 (7.5–18.9)	12.4 (6.5–21.4)
Slovenia	All age	12.8 (4.5–26.9)	12.3 (5.7–22.6)	11.7 (8.8–15.2)	11.6 (8.0–17.2)	11.6 (7.3–19.3)
Eastern Europe	20 to 24	15.2 (5.1–31.9)	15.1 (5.0–32.0)	15.1 (5.3–31.1)	15.3 (6.0–32.8)	15.3 (6.1–32.4)
Eastern Europe	25 to 29	15.4 (5.2–32.6)	15.4 (5.0–32.6)	15.4 (5.3–31.9)	15.6 (5.9–33.7)	15.6 (6.1–33.2)
Eastern Europe	30 to 34	16.6 (5.7–34.6)	16.5 (5.5–34.5)	16.6 (5.8–33.8)	16.7 (6.3–35.8)	16.7 (6.5–35.3)
Eastern Europe	35 to 39	16.8 (5.8–34.9)	16.7 (5.6–34.9)	16.8 (5.9–34.0)	16.9 (6.4–36.1)	16.9 (6.6–35.7)
Eastern Europe	40 to 44	17.2 (5.9–35.6)	17.2 (5.7–35.7)	17.2 (6.1–34.8)	17.4 (6.6–36.9)	17.4 (6.8–36.5)
Eastern Europe	45 to 49	16.8 (5.8–34.7)	16.7 (5.5–34.9)	16.8 (5.9–34.0)	16.9 (6.4–36.0)	16.9 (6.6–35.7)
Eastern Europe	50 to 54	16.3 (5.6–34.1)	16.3 (5.4–34.2)	16.4 (5.7–33.4)	16.5 (6.3–35.2)	16.5 (6.5–34.8)
Eastern Europe	55 to 59	15.7 (5.3–33.0)	15.7 (5.2–33.0)	15.7 (5.4–32.3)	15.8 (6.0–34.1)	15.8 (6.2–33.5)
Eastern Europe	60 to 64	15.1 (5.1–32.0)	15.1 (4.9–32.1)	15.1 (5.2–31.2)	15.3 (5.7–33.2)	15.3 (5.9–32.7)
Eastern Europe	65 to 69	14.0 (4.7–30.0)	13.9 (4.5–30.0)	13.9 (4.9–28.9)	14.1 (5.2–31.1)	14.1 (5.4–30.7)
Eastern Europe	70 to 74	11.1 (3.6–24.7)	11.1 (3.4–24.7)	11.1 (3.7–23.9)	11.2 (4.0–25.6)	11.2 (4.2–25.3)

Supplementary Table S10: Prevalence of female SVAC by age and location for 1990, 2000, 2010, 2020, and 2023						
**Location**	**Age Range**	**1990**	**2000**	**2010**	**2020**	**2023**
Eastern Europe	75 to 79	10.1 (3.2–22.7)	10.1 (3.0–22.7)	10.1 (3.2–22.3)	10.2 (3.4–24.0)	10.2 (3.4–23.6)
Eastern Europe	80 to 84	7.1 (2.2–16.6)	7.1 (2.1–16.6)	7.1 (2.2–16.3)	7.2 (2.3–17.6)	7.2 (2.3–17.4)
Eastern Europe	85 to 89	6.4 (1.9–15.1)	6.4 (1.9–15.1)	6.4 (1.9–14.8)	6.5 (2.1–16.0)	6.5 (2.1–15.8)
Eastern Europe	90 to 94	5.8 (1.7–13.7)	5.8 (1.7–13.7)	5.8 (1.7–13.4)	5.9 (1.8–14.6)	5.9 (1.9–14.4)
Eastern Europe	95 plus	5.7 (1.7–13.6)	5.7 (1.6–13.6)	5.7 (1.7–13.3)	5.8 (1.8–14.5)	5.8 (1.9–14.2)
Eastern Europe	Age-standardized	15.4 (5.2–32.4)	15.4 (5.0–32.4)	15.4 (5.4–31.6)	15.6 (5.8–33.5)	15.6 (6.1–33.1)
Eastern Europe	All age	15.1 (5.1–31.9)	15.0 (4.9–31.8)	14.9 (5.2–30.8)	15.0 (5.6–32.6)	15.0 (5.8–32.1)
Belarus	20 to 24	15.1 (5.0–32.2)	15.0 (4.8–32.2)	15.1 (5.0–31.6)	15.3 (5.3–33.8)	15.3 (5.4–33.3)
Belarus	25 to 29	15.3 (5.1–32.7)	15.3 (4.9–32.6)	15.3 (5.1–32.1)	15.5 (5.4–34.2)	15.5 (5.5–33.7)
Belarus	30 to 34	16.5 (5.6–34.7)	16.5 (5.3–34.6)	16.5 (5.6–34.1)	16.7 (5.9–36.3)	16.7 (6.0–35.9)
Belarus	35 to 39	16.6 (5.7–35.0)	16.6 (5.4–34.9)	16.6 (5.6–34.3)	16.8 (6.0–36.5)	16.8 (6.1–36.1)
Belarus	40 to 44	17.1 (5.9–35.8)	17.1 (5.6–35.7)	17.1 (5.8–35.2)	17.3 (6.2–37.4)	17.3 (6.2–37.0)
Belarus	45 to 49	16.7 (5.7–35.0)	16.6 (5.4–34.9)	16.7 (5.6–34.4)	16.8 (6.0–36.5)	16.8 (6.1–36.1)
Belarus	50 to 54	16.2 (5.5–34.3)	16.2 (5.2–34.2)	16.2 (5.5–33.7)	16.4 (5.8–35.8)	16.4 (5.9–35.4)
Belarus	55 to 59	15.6 (5.3–33.2)	15.6 (5.0–33.1)	15.6 (5.2–32.6)	15.8 (5.5–34.7)	15.8 (5.6–34.1)

Supplementary Table S10: Prevalence of female SVAC by age and location for 1990, 2000, 2010, 2020, and 2023						
**Location**	**Age Range**	**1990**	**2000**	**2010**	**2020**	**2023**
Belarus	60 to 64	15.0 (5.0–32.2)	15.0 (4.8–32.1)	15.1 (5.0–31.6)	15.2 (5.3–33.7)	15.2 (5.4–33.3)
Belarus	65 to 69	13.9 (4.6–30.2)	13.9 (4.4–30.1)	13.9 (4.6–29.6)	14.1 (4.8–31.6)	14.1 (4.9–31.2)
Belarus	70 to 74	11.1 (3.6–24.8)	11.1 (3.4–24.8)	11.1 (3.5–24.3)	11.2 (3.7–26.1)	11.2 (3.8–25.8)
Belarus	75 to 79	10.0 (3.2–22.7)	10.0 (3.0–22.6)	10.0 (3.1–22.2)	10.1 (3.3–23.9)	10.1 (3.4–23.6)
Belarus	80 to 84	7.1 (2.2–16.6)	7.1 (2.1–16.6)	7.1 (2.2–16.2)	7.2 (2.3–17.6)	7.2 (2.3–17.3)
Belarus	85 to 89	6.4 (1.9–15.0)	6.4 (1.8–15.0)	6.4 (1.9–14.7)	6.5 (2.0–15.9)	6.5 (2.1–15.7)
Belarus	90 to 94	5.8 (1.7–13.7)	5.8 (1.7–13.7)	5.8 (1.7–13.4)	5.8 (1.8–14.5)	5.8 (1.9–14.3)
Belarus	95 plus	5.7 (1.7–13.6)	5.7 (1.6–13.5)	5.7 (1.7–13.3)	5.8 (1.8–14.4)	5.8 (1.8–14.2)
Belarus	Age-standardized	15.3 (5.1–32.5)	15.3 (4.9–32.5)	15.3 (5.1–32.0)	15.5 (5.4–34.0)	15.5 (5.5–33.6)
Belarus	All age	15.0 (5.0–32.0)	14.9 (4.8–31.8)	14.8 (4.9–31.0)	14.9 (5.2–32.9)	14.9 (5.3–32.4)
Estonia	20 to 24	16.4 (5.8–33.5)	15.5 (7.3–27.5)	14.5 (10.8–19.0)	14.9 (8.1–23.7)	15.1 (7.3–26.5)
Estonia	25 to 29	17.4 (6.3–35.2)	17.0 (8.2–29.8)	16.4 (12.5–21.0)	16.5 (9.2–25.8)	16.7 (8.2–28.8)
Estonia	30 to 34	18.3 (6.7–36.8)	17.6 (8.5–30.7)	16.7 (12.7–21.3)	17.0 (9.5–26.4)	17.2 (8.5–29.7)
Estonia	35 to 39	19.7 (7.3–39.0)	19.9 (9.8–34.1)	19.8 (15.3–24.9)	19.7 (11.2–30.0)	19.6 (9.9–33.1)
Estonia	40 to 44	20.0 (7.5–39.5)	20.0 (9.9–34.2)	19.7 (15.2–24.7)	19.7 (11.2–29.9)	19.7 (10.0–33.2)

Supplementary Table S10: Prevalence of female SVAC by age and location for 1990, 2000, 2010, 2020, and 2023						
**Location**	**Age Range**	**1990**	**2000**	**2010**	**2020**	**2023**
Estonia	45 to 49	19.4 (7.2–38.6)	19.3 (9.5–33.2)	18.9 (14.6–24.0)	19.0 (10.7–29.1)	19.0 (9.5–32.3)
Estonia	50 to 54	19.5 (7.3–38.7)	19.9 (9.8–34.0)	19.9 (15.4–24.9)	19.7 (11.2–30.0)	19.6 (9.9–33.1)
Estonia	55 to 59	17.7 (6.4–35.8)	17.1 (8.2–29.9)	16.3 (12.4–20.9)	16.6 (9.2–25.9)	16.7 (8.2–29.0)
Estonia	60 to 64	17.3 (6.2–35.0)	16.8 (8.1–29.4)	16.1 (12.2–20.7)	16.3 (9.0–25.5)	16.4 (8.0–28.5)
Estonia	65 to 69	15.6 (5.5–32.2)	14.8 (6.9–26.4)	13.9 (10.2–18.3)	14.2 (7.7–22.8)	14.4 (6.9–25.5)
Estonia	70 to 74	12.0 (4.1–25.5)	10.8 (4.7–20.0)	9.8 (6.8–13.9)	10.3 (5.4–17.6)	10.6 (4.7–20.0)
Estonia	75 to 79	11.7 (3.8–26.0)	11.7 (3.6–26.0)	11.7 (3.8–25.5)	11.8 (4.0–27.3)	11.8 (4.0–27.0)
Estonia	80 to 84	8.4 (2.6–19.3)	8.4 (2.5–19.3)	8.4 (2.6–18.9)	8.4 (2.7–20.3)	8.4 (2.8–20.0)
Estonia	85 to 89	7.6 (2.3–17.6)	7.5 (2.2–17.5)	7.6 (2.3–17.2)	7.6 (2.4–18.5)	7.6 (2.5–18.3)
Estonia	90 to 94	6.8 (2.1–16.0)	6.8 (2.0–16.0)	6.8 (2.1–15.7)	6.9 (2.2–16.9)	6.9 (2.2–16.7)
Estonia	95 plus	6.8 (2.1–15.9)	6.8 (2.0–15.9)	6.8 (2.1–15.5)	6.8 (2.2–16.8)	6.8 (2.2–16.5)
Estonia	Age-standardized	17.5 (6.4–35.0)	17.2 (8.2–30.2)	16.6 (12.5–21.5)	16.7 (10.3–25.4)	16.8 (9.1–28.2)
Estonia	All age	17.1 (6.4–34.2)	16.6 (7.8–29.1)	15.8 (11.7–20.7)	15.6 (11.1–22.7)	15.7 (10.1–25.0)
Latvia	20 to 24	13.4 (4.7–28.3)	12.6 (5.9–22.8)	11.7 (8.8–15.2)	12.0 (6.5–19.2)	12.3 (5.8–21.9)
Latvia	25 to 29	14.2 (5.0–29.8)	13.8 (6.5–24.8)	13.3 (10.1–16.8)	13.4 (7.3–21.1)	13.5 (6.5–23.9)

Supplementary Table S10: Prevalence of female SVAC by age and location for 1990, 2000, 2010, 2020, and 2023						
**Location**	**Age Range**	**1990**	**2000**	**2010**	**2020**	**2023**
Latvia	30 to 34	15.0 (5.3–31.2)	14.3 (6.7–25.5)	13.4 (10.3–17.1)	13.7 (7.5–21.5)	13.9 (6.8–24.6)
Latvia	35 to 39	16.2 (5.8–33.2)	16.2 (7.7–28.6)	16.0 (12.5–20.0)	16.0 (8.9–24.6)	16.0 (7.9–27.6)
Latvia	40 to 44	16.4 (6.0–33.7)	16.3 (7.8–28.7)	15.9 (12.4–19.8)	15.9 (8.9–24.5)	16.0 (7.9–27.6)
Latvia	45 to 49	16.0 (5.8–32.9)	15.8 (7.5–27.9)	15.4 (11.9–19.3)	15.4 (8.6–23.8)	15.5 (7.6–26.9)
Latvia	50 to 54	16.0 (5.8–33.0)	16.2 (7.7–28.7)	16.1 (12.6–20.1)	16.0 (9.0–24.6)	16.0 (7.9–27.5)
Latvia	55 to 59	14.5 (5.1–30.3)	13.9 (6.5–25.0)	13.2 (10.1–16.8)	13.4 (7.3–21.1)	13.6 (6.6–24.0)
Latvia	60 to 64	14.1 (5.0–29.7)	13.7 (6.4–24.5)	13.0 (9.9–16.6)	13.2 (7.2–20.8)	13.4 (6.4–23.7)
Latvia	65 to 69	12.8 (4.4–27.2)	12.0 (5.6–21.9)	11.3 (8.4–14.7)	11.5 (6.2–18.6)	11.7 (5.5–21.1)
Latvia	70 to 74	9.7 (3.2–21.1)	8.8 (3.9–16.1)	7.9 (5.5–11.2)	8.3 (4.3–14.0)	8.6 (3.8–15.7)
Latvia	75 to 79	9.5 (3.0–21.7)	9.5 (2.9–21.6)	9.5 (3.0–21.2)	9.6 (3.1–22.8)	9.6 (3.2–22.5)
Latvia	80 to 84	6.7 (2.1–15.8)	6.7 (2.0–15.8)	6.7 (2.0–15.5)	6.8 (2.2–16.7)	6.8 (2.2–16.4)
Latvia	85 to 89	6.1 (1.8–14.3)	6.1 (1.7–14.3)	6.1 (1.8–14.0)	6.1 (1.9–15.2)	6.1 (2.0–14.9)
Latvia	90 to 94	5.5 (1.6–13.1)	5.5 (1.6–13.0)	5.5 (1.6–12.7)	5.5 (1.7–13.8)	5.5 (1.8–13.6)
Latvia	95 plus	5.4 (1.6–12.9)	5.4 (1.6–12.9)	5.4 (1.6–12.6)	5.5 (1.7–13.7)	5.5 (1.7–13.5)
Latvia	Age-standardized	14.3 (5.1–29.7)	14.0 (6.5–25.3)	13.4 (10.2–17.2)	13.6 (8.3–20.7)	13.7 (7.3–23.4)

Supplementary Table S10: Prevalence of female SVAC by age and location for 1990, 2000, 2010, 2020, and 2023						
**Location**	**Age Range**	**1990**	**2000**	**2010**	**2020**	**2023**
Latvia	All age	14.0 (5.0–29.0)	13.5 (6.3–24.4)	12.7 (9.5–16.6)	12.7 (8.9–18.5)	12.7 (8.1–20.7)
Lithuania	20 to 24	12.3 (4.2–26.2)	11.5 (5.3–21.0)	10.7 (8.0–13.8)	11.0 (5.9–17.6)	11.2 (5.3–20.2)
Lithuania	25 to 29	13.0 (4.6–27.7)	12.7 (5.9–23.0)	12.1 (9.3–15.3)	12.2 (6.7–19.3)	12.4 (5.9–21.9)
Lithuania	30 to 34	13.8 (4.8–29.0)	13.1 (6.1–23.6)	12.3 (9.5–15.6)	12.5 (6.9–19.7)	12.7 (6.1–22.6)
Lithuania	35 to 39	14.8 (5.3–30.8)	14.8 (7.0–26.4)	14.5 (11.4–18.1)	14.5 (8.1–22.4)	14.5 (7.1–25.3)
Lithuania	40 to 44	15.1 (5.4–31.3)	14.9 (7.0–26.5)	14.4 (11.2–18.0)	14.5 (8.0–22.4)	14.6 (7.1–25.4)
Lithuania	45 to 49	14.7 (5.2–30.6)	14.4 (6.8–25.8)	14.0 (10.9–17.4)	14.1 (7.8–21.8)	14.1 (6.9–24.6)
Lithuania	50 to 54	14.7 (5.2–30.7)	14.8 (7.0–26.5)	14.7 (11.4–18.3)	14.6 (8.1–22.5)	14.6 (7.1–25.3)
Lithuania	55 to 59	13.3 (4.7–28.2)	12.7 (5.9–23.0)	12.0 (9.2–15.2)	12.2 (6.7–19.3)	12.4 (5.9–22.1)
Lithuania	60 to 64	13.0 (4.5–27.5)	12.5 (5.8–22.7)	11.9 (9.1–15.1)	12.1 (6.6–19.1)	12.2 (5.8–21.8)
Lithuania	65 to 69	11.7 (4.0–25.1)	11.0 (5.1–20.1)	10.2 (7.6–13.3)	10.5 (5.6–16.9)	10.7 (5.0–19.3)
Lithuania	70 to 74	8.9 (2.9–19.6)	8.0 (3.5–14.8)	7.2 (5.0–10.1)	7.5 (3.9–12.7)	7.8 (3.4–14.2)
Lithuania	75 to 79	8.7 (2.7–20.0)	8.7 (2.6–19.9)	8.7 (2.7–19.5)	8.8 (2.8–21.0)	8.8 (2.9–20.7)
Lithuania	80 to 84	6.1 (1.9–14.5)	6.1 (1.8–14.4)	6.1 (1.8–14.2)	6.2 (2.0–15.3)	6.2 (2.0–15.1)
Lithuania	85 to 89	5.5 (1.7–13.1)	5.5 (1.6–13.1)	5.5 (1.6–12.8)	5.6 (1.7–13.9)	5.6 (1.8–13.7)

Supplementary Table S10: Prevalence of female SVAC by age and location for 1990, 2000, 2010, 2020, and 2023						
**Location**	**Age Range**	**1990**	**2000**	**2010**	**2020**	**2023**
Lithuania	90 to 94	5.0 (1.5–11.9)	5.0 (1.4–11.9)	5.0 (1.5–11.6)	5.0 (1.6–12.6)	5.0 (1.6–12.4)
Lithuania	95 plus	4.9 (1.5–11.8)	4.9 (1.4–11.8)	4.9 (1.5–11.5)	5.0 (1.5–12.5)	5.0 (1.6–12.3)
Lithuania	Age-standardized	13.1 (4.6–27.6)	12.8 (5.9–23.4)	12.2 (9.3–15.7)	12.4 (7.5–19.0)	12.5 (6.6–21.6)
Lithuania	All age	12.9 (4.6–27.1)	12.4 (5.7–22.8)	11.7 (8.7–15.3)	11.6 (8.1–17.0)	11.7 (7.3–19.3)
Moldova	20 to 24	22.1 (8.2–42.9)	22.3 (9.2–41.2)	22.4 (12.9–35.0)	22.3 (16.3–29.2)	22.3 (14.6–31.9)
Moldova	25 to 29	22.0 (8.0–43.7)	22.0 (7.6–43.6)	22.1 (7.9–43.0)	22.3 (8.4–45.4)	22.3 (8.5–45.0)
Moldova	30 to 34	23.5 (8.6–45.8)	23.5 (8.3–45.8)	23.5 (8.6–45.2)	23.8 (9.1–47.6)	23.8 (9.2–47.2)
Moldova	35 to 39	23.6 (8.7–46.0)	23.6 (8.3–45.9)	23.6 (8.6–45.3)	23.9 (9.1–47.7)	23.9 (9.3–47.3)
Moldova	40 to 44	24.1 (8.9–46.8)	24.1 (8.5–46.7)	24.2 (8.9–46.1)	24.5 (9.4–48.6)	24.5 (9.6–48.1)
Moldova	45 to 49	23.5 (8.6–45.8)	23.5 (8.3–45.8)	23.5 (8.6–45.2)	23.8 (9.1–47.7)	23.8 (9.2–47.2)
Moldova	50 to 54	22.9 (8.4–45.0)	22.9 (8.0–44.9)	23.0 (8.3–44.3)	23.3 (8.8–46.8)	23.3 (9.0–46.4)
Moldova	55 to 59	22.1 (8.0–43.9)	22.1 (7.7–43.8)	22.2 (8.0–43.2)	22.5 (8.5–45.7)	22.5 (8.6–45.3)
Moldova	60 to 64	21.4 (7.7–42.8)	21.4 (7.4–42.7)	21.5 (7.6–42.1)	21.8 (8.1–44.6)	21.8 (8.3–44.2)
Moldova	65 to 69	20.0 (7.1–40.5)	20.0 (6.8–40.5)	20.0 (7.0–39.9)	20.3 (7.5–42.4)	20.3 (7.6–41.9)
Moldova	70 to 74	16.3 (5.5–34.3)	16.2 (5.3–34.3)	16.3 (5.5–33.7)	16.6 (5.8–36.1)	16.6 (5.9–35.6)

Supplementary Table S10: Prevalence of female SVAC by age and location for 1990, 2000, 2010, 2020, and 2023						
**Location**	**Age Range**	**1990**	**2000**	**2010**	**2020**	**2023**
Moldova	75 to 79	14.8 (4.9–31.7)	14.7 (4.7–31.6)	14.8 (4.9–31.1)	15.0 (5.2–33.3)	15.0 (5.3–32.8)
Moldova	80 to 84	10.7 (3.4–23.9)	10.6 (3.2–23.9)	10.7 (3.4–23.5)	10.9 (3.6–25.4)	10.9 (3.7–25.0)
Moldova	85 to 89	9.6 (3.0–21.9)	9.6 (2.9–21.9)	9.7 (3.0–21.5)	9.8 (3.2–23.2)	9.8 (3.3–22.9)
Moldova	90 to 94	8.8 (2.7–20.1)	8.7 (2.6–20.0)	8.8 (2.7–19.7)	8.9 (2.9–21.3)	8.9 (2.9–21.0)
Moldova	95 plus	8.7 (2.7–19.9)	8.7 (2.6–19.9)	8.7 (2.7–19.5)	8.8 (2.9–21.2)	8.8 (2.9–20.9)
Moldova	Age-standardized	21.9 (8.0–42.8)	21.9 (8.2–42.9)	22.0 (8.6–41.1)	22.2 (9.9–42.3)	22.2 (10.0–42.0)
Moldova	All age	21.9 (8.0–42.8)	21.8 (8.0–42.8)	21.6 (8.3–40.7)	21.7 (8.8–42.8)	21.6 (9.1–42.3)
Russia	20 to 24	15.1 (5.0–32.2)	15.0 (4.8–32.2)	15.1 (5.0–31.6)	15.3 (5.3–33.8)	15.3 (5.4–33.3)
Russia	25 to 29	15.3 (5.1–32.7)	15.3 (4.9–32.6)	15.3 (5.1–32.1)	15.5 (5.4–34.2)	15.5 (5.5–33.7)
Russia	30 to 34	16.5 (5.6–34.7)	16.5 (5.3–34.6)	16.5 (5.6–34.1)	16.7 (5.9–36.3)	16.7 (6.0–35.9)
Russia	35 to 39	16.6 (5.7–35.0)	16.6 (5.4–34.9)	16.6 (5.6–34.3)	16.8 (6.0–36.5)	16.8 (6.1–36.1)
Russia	40 to 44	17.1 (5.9–35.8)	17.1 (5.6–35.7)	17.1 (5.8–35.2)	17.3 (6.2–37.4)	17.3 (6.2–37.0)
Russia	45 to 49	16.7 (5.7–35.0)	16.6 (5.4–34.9)	16.7 (5.6–34.4)	16.8 (6.0–36.5)	16.8 (6.1–36.1)
Russia	50 to 54	16.2 (5.5–34.3)	16.2 (5.2–34.2)	16.2 (5.5–33.7)	16.4 (5.8–35.8)	16.4 (5.9–35.4)
Russia	55 to 59	15.6 (5.3–33.2)	15.6 (5.0–33.1)	15.6 (5.2–32.6)	15.8 (5.5–34.7)	15.8 (5.6–34.1)

Supplementary Table S10: Prevalence of female SVAC by age and location for 1990, 2000, 2010, 2020, and 2023						
**Location**	**Age Range**	**1990**	**2000**	**2010**	**2020**	**2023**
Russia	60 to 64	15.0 (5.0–32.2)	15.0 (4.8–32.1)	15.1 (5.0–31.6)	15.2 (5.3–33.7)	15.2 (5.4–33.3)
Russia	65 to 69	13.9 (4.6–30.2)	13.9 (4.4–30.1)	13.9 (4.6–29.6)	14.1 (4.8–31.6)	14.1 (4.9–31.2)
Russia	70 to 74	11.1 (3.6–24.8)	11.1 (3.4–24.8)	11.1 (3.5–24.3)	11.2 (3.7–26.1)	11.2 (3.8–25.8)
Russia	75 to 79	10.0 (3.2–22.7)	10.0 (3.0–22.6)	10.0 (3.1–22.2)	10.1 (3.3–23.9)	10.1 (3.4–23.6)
Russia	80 to 84	7.1 (2.2–16.6)	7.1 (2.1–16.6)	7.1 (2.2–16.2)	7.2 (2.3–17.6)	7.2 (2.3–17.3)
Russia	85 to 89	6.4 (1.9–15.0)	6.4 (1.8–15.0)	6.4 (1.9–14.7)	6.5 (2.0–15.9)	6.5 (2.1–15.7)
Russia	90 to 94	5.8 (1.7–13.7)	5.8 (1.7–13.7)	5.8 (1.7–13.4)	5.8 (1.8–14.5)	5.8 (1.9–14.3)
Russia	95 plus	5.7 (1.7–13.6)	5.7 (1.6–13.5)	5.7 (1.7–13.3)	5.8 (1.8–14.4)	5.8 (1.8–14.2)
Russia	Age-standardized	15.3 (5.1–32.5)	15.3 (4.9–32.5)	15.3 (5.1–32.0)	15.5 (5.4–34.0)	15.5 (5.5–33.6)
Russia	All age	15.1 (5.1–32.1)	15.0 (4.8–31.9)	14.9 (5.0–31.2)	15.0 (5.2–33.1)	15.0 (5.3–32.7)
Ukraine	20 to 24	15.1 (5.0–32.2)	15.0 (4.8–32.2)	15.1 (5.0–31.6)	15.3 (5.3–33.8)	15.3 (5.4–33.3)
Ukraine	25 to 29	15.3 (5.1–32.7)	15.3 (4.9–32.6)	15.3 (5.1–32.1)	15.5 (5.4–34.2)	15.5 (5.5–33.7)
Ukraine	30 to 34	16.5 (5.6–34.7)	16.5 (5.3–34.6)	16.5 (5.6–34.1)	16.7 (5.9–36.3)	16.7 (6.0–35.9)
Ukraine	35 to 39	16.6 (5.7–35.0)	16.6 (5.4–34.9)	16.6 (5.6–34.3)	16.8 (6.0–36.5)	16.8 (6.1–36.1)
Ukraine	40 to 44	17.1 (5.9–35.8)	17.1 (5.6–35.7)	17.1 (5.8–35.2)	17.3 (6.2–37.4)	17.3 (6.2–37.0)

Supplementary Table S10: Prevalence of female SVAC by age and location for 1990, 2000, 2010, 2020, and 2023						
**Location**	**Age Range**	**1990**	**2000**	**2010**	**2020**	**2023**
Ukraine	45 to 49	16.7 (5.7–35.0)	16.6 (5.4–34.9)	16.7 (5.6–34.4)	16.8 (6.0–36.5)	16.8 (6.1–36.1)
Ukraine	50 to 54	16.2 (5.5–34.3)	16.2 (5.2–34.2)	16.2 (5.5–33.7)	16.4 (5.8–35.8)	16.4 (5.9–35.4)
Ukraine	55 to 59	15.6 (5.3–33.2)	15.6 (5.0–33.1)	15.6 (5.2–32.6)	15.8 (5.5–34.7)	15.8 (5.6–34.1)
Ukraine	60 to 64	15.0 (5.0–32.2)	15.0 (4.8–32.1)	15.1 (5.0–31.6)	15.2 (5.3–33.7)	15.2 (5.4–33.3)
Ukraine	65 to 69	13.9 (4.6–30.2)	13.9 (4.4–30.1)	13.9 (4.6–29.6)	14.1 (4.8–31.6)	14.1 (4.9–31.2)
Ukraine	70 to 74	11.1 (3.6–24.8)	11.1 (3.4–24.8)	11.1 (3.5–24.3)	11.2 (3.7–26.1)	11.2 (3.8–25.8)
Ukraine	75 to 79	10.0 (3.2–22.7)	10.0 (3.0–22.6)	10.0 (3.1–22.2)	10.1 (3.3–23.9)	10.1 (3.4–23.6)
Ukraine	80 to 84	7.1 (2.2–16.6)	7.1 (2.1–16.6)	7.1 (2.2–16.2)	7.2 (2.3–17.6)	7.2 (2.3–17.3)
Ukraine	85 to 89	6.4 (1.9–15.0)	6.4 (1.8–15.0)	6.4 (1.9–14.7)	6.5 (2.0–15.9)	6.5 (2.1–15.7)
Ukraine	90 to 94	5.8 (1.7–13.7)	5.8 (1.7–13.7)	5.8 (1.7–13.4)	5.8 (1.8–14.5)	5.8 (1.9–14.3)
Ukraine	95 plus	5.7 (1.7–13.6)	5.7 (1.6–13.5)	5.7 (1.7–13.3)	5.8 (1.8–14.4)	5.8 (1.8–14.2)
Ukraine	Age-standardized	15.3 (5.1–32.5)	15.3 (4.9–32.5)	15.3 (5.1–32.0)	15.5 (5.4–34.0)	15.5 (5.5–33.6)
Ukraine	All age	14.9 (5.0–31.9)	14.9 (4.8–31.7)	14.8 (4.9–31.0)	14.9 (5.2–32.9)	14.8 (5.2–32.4)
High income	20 to 24	23.8 (16.7–33.4)	22.9 (16.9–31.2)	22.8 (19.9–27.0)	23.1 (20.5–26.8)	23.2 (19.3–28.6)
High income	25 to 29	23.6 (16.8–33.1)	23.3 (17.3–31.9)	23.4 (19.1–28.9)	23.9 (22.3–26.4)	23.9 (21.7–27.2)

Supplementary Table S10: Prevalence of female SVAC by age and location for 1990, 2000, 2010, 2020, and 2023						
**Location**	**Age Range**	**1990**	**2000**	**2010**	**2020**	**2023**
High income	30 to 34	25.1 (17.8–35.0)	24.4 (17.6–33.3)	24.1 (19.3–30.1)	24.4 (23.0–26.6)	24.5 (22.9–26.9)
High income	35 to 39	24.6 (16.8–35.2)	25.1 (18.1–34.1)	25.6 (20.0–32.5)	25.6 (23.8–28.9)	25.7 (24.2–28.1)
High income	40 to 44	24.2 (16.0–35.2)	25.2 (18.0–34.4)	26.0 (20.6–32.6)	25.7 (23.9–29.3)	25.7 (24.0–28.5)
High income	45 to 49	23.8 (15.6–34.8)	24.8 (17.5–34.2)	25.5 (20.5–31.6)	25.6 (23.4–29.6)	25.5 (23.7–28.8)
High income	50 to 54	22.5 (14.0–34.1)	24.1 (17.3–33.4)	25.9 (21.3–31.6)	25.8 (22.9–30.8)	25.8 (22.8–31.0)
High income	55 to 59	21.3 (13.0–32.9)	21.7 (15.1–31.0)	23.4 (18.7–29.4)	24.0 (21.2–28.7)	23.8 (21.4–28.6)
High income	60 to 64	20.8 (12.5–32.2)	20.5 (13.4–30.4)	22.5 (17.3–29.1)	23.8 (21.3–28.4)	23.9 (21.6–28.3)
High income	65 to 69	18.9 (10.6–30.4)	18.7 (11.2–29.2)	20.2 (14.7–27.2)	21.9 (19.2–26.8)	22.1 (19.3–26.9)
High income	70 to 74	15.2 (8.1–25.6)	15.0 (8.6–24.6)	15.7 (10.7–22.5)	18.1 (14.6–24.4)	18.5 (15.4–24.6)
High income	75 to 79	14.3 (6.7–26.2)	14.2 (6.9–26.1)	15.3 (8.1–26.8)	16.9 (10.2–29.2)	17.4 (10.7–29.1)
High income	80 to 84	10.0 (3.9–20.2)	10.3 (4.5–20.1)	11.6 (5.8–21.1)	12.6 (7.2–23.2)	12.8 (7.6–22.9)
High income	85 to 89	9.2 (3.6–18.8)	9.5 (4.0–19.1)	10.6 (5.4–19.4)	11.5 (6.4–21.5)	11.6 (6.4–21.4)
High income	90 to 94	8.8 (3.5–17.7)	9.1 (3.7–18.6)	9.8 (5.2–17.6)	9.6 (6.0–17.9)	9.5 (5.7–17.9)
High income	95 plus	8.9 (3.7–17.5)	9.4 (3.9–19.0)	9.7 (5.1–17.5)	9.4 (6.0–17.3)	9.3 (5.7–17.1)
High income	Age-standardized	22.4 (14.8–32.8)	22.5 (15.8–31.7)	23.2 (18.5–29.2)	23.7 (21.8–27.3)	23.7 (21.9–27.1)

Supplementary Table S10: Prevalence of female SVAC by age and location for 1990, 2000, 2010, 2020, and 2023						
**Location**	**Age Range**	**1990**	**2000**	**2010**	**2020**	**2023**
High income	All age	21.7 (14.1–32.3)	21.7 (15.1–31.1)	22.3 (17.5–28.6)	22.6 (20.4–27.3)	22.6 (20.5–27.0)
Australasia	20 to 24	30.1 (21.6–40.6)	26.7 (22.2–31.5)	22.4 (17.7–27.5)	23.9 (18.5–30.4)	25.2 (18.2–34.2)
Australasia	25 to 29	29.3 (19.5–41.5)	28.1 (24.0–32.4)	25.8 (21.2–31.0)	26.4 (20.7–32.9)	27.0 (19.9–34.9)
Australasia	30 to 34	29.6 (18.9–42.7)	28.5 (23.8–33.5)	27.0 (22.7–31.9)	27.2 (21.4–33.8)	27.6 (20.4–35.6)
Australasia	35 to 39	28.5 (18.0–41.5)	28.5 (23.7–33.6)	29.4 (24.3–34.7)	30.4 (24.2–37.1)	30.6 (22.9–40.6)
Australasia	40 to 44	28.0 (17.7–41.1)	27.5 (22.8–32.7)	29.0 (23.8–34.4)	30.6 (25.2–37.0)	30.9 (24.0–39.2)
Australasia	45 to 49	29.6 (20.3–40.6)	28.7 (23.9–33.9)	28.7 (23.5–33.9)	29.6 (25.3–34.4)	29.9 (23.6–37.5)
Australasia	50 to 54	28.3 (20.3–37.9)	28.1 (23.4–33.3)	29.0 (23.3–34.9)	30.1 (24.1–37.1)	30.3 (22.6–40.0)
Australasia	55 to 59	29.6 (20.3–40.8)	26.0 (21.5–31.0)	24.6 (19.1–30.5)	28.2 (21.2–36.7)	29.3 (20.8–39.6)
Australasia	60 to 64	30.2 (19.6–43.5)	26.9 (21.7–32.8)	24.7 (19.1–30.9)	27.9 (21.1–36.5)	29.0 (20.6–39.0)
Australasia	65 to 69	26.2 (14.8–41.3)	23.8 (17.9–30.7)	23.2 (16.4–30.8)	29.3 (22.1–37.6)	31.2 (22.5–41.6)
Australasia	70 to 74	20.5 (8.5–38.0)	20.1 (11.0–32.2)	20.7 (14.1–28.9)	22.2 (15.5–30.4)	22.8 (15.7–32.0)
Australasia	75 to 79	18.3 (7.3–34.6)	18.0 (8.9–30.0)	18.6 (11.7–26.7)	18.4 (10.5–29.6)	18.4 (9.4–31.0)
Australasia	80 to 84	13.7 (4.8–28.9)	14.4 (5.6–28.4)	16.8 (8.1–29.1)	19.0 (10.1–32.0)	19.0 (9.5–32.3)
Australasia	85 to 89	11.9 (4.1–25.8)	12.2 (4.5–25.1)	14.3 (6.1–27.2)	17.9 (10.3–29.8)	18.6 (10.8–29.9)

Supplementary Table S10: Prevalence of female SVAC by age and location for 1990, 2000, 2010, 2020, and 2023						
**Location**	**Age Range**	**1990**	**2000**	**2010**	**2020**	**2023**
Australasia	90 to 94	10.1 (3.3–22.4)	9.4 (3.2–20.6)	9.1 (3.2–19.2)	9.5 (3.4–21.3)	9.7 (3.4–21.7)
Australasia	95 plus	10.0 (3.3–22.2)	9.3 (3.1–20.3)	9.0 (3.1–18.9)	9.4 (3.3–21.0)	9.5 (3.4–21.4)
Australasia	Age-standardized	28.0 (18.3–39.9)	26.6 (21.9–31.8)	25.7 (22.7–28.9)	27.3 (23.5–31.8)	27.9 (23.1–33.9)
Australasia	All age	27.6 (17.9–39.7)	26.1 (21.2–31.4)	25.4 (22.5–28.3)	26.9 (23.6–30.4)	27.4 (23.1–32.8)
Australia	20 to 24	28.6 (18.7–40.7)	26.1 (21.9–30.6)	22.3 (17.0–28.3)	23.9 (18.2–30.7)	25.4 (18.2–34.2)
Australia	25 to 29	28.3 (16.8–42.5)	27.1 (23.0–31.6)	25.9 (20.1–32.4)	26.8 (20.3–34.0)	27.3 (19.4–36.0)
Australia	30 to 34	28.0 (15.5–43.8)	27.6 (22.7–32.8)	27.0 (21.4–33.4)	27.2 (20.7–34.4)	27.6 (19.7–36.4)
Australia	35 to 39	27.6 (15.2–43.1)	27.9 (22.8–33.3)	29.1 (23.4–34.7)	30.0 (23.8–37.1)	30.2 (22.2–39.9)
Australia	40 to 44	27.2 (14.8–43.0)	27.1 (21.9–32.6)	28.6 (22.7–34.5)	29.9 (24.1–36.2)	30.1 (23.3–38.6)
Australia	45 to 49	27.9 (17.9–39.5)	28.1 (23.3–33.2)	28.4 (22.4–34.9)	28.8 (22.8–35.6)	28.9 (21.3–38.6)
Australia	50 to 54	27.2 (18.0–38.0)	27.5 (22.7–32.5)	28.9 (22.7–35.5)	30.0 (23.2–37.8)	30.2 (21.9–40.5)
Australia	55 to 59	28.9 (19.6–40.2)	25.7 (21.4–30.2)	24.4 (18.6–30.6)	28.1 (21.3–36.2)	29.3 (21.1–39.1)
Australia	60 to 64	29.8 (18.7–43.7)	26.8 (21.8–32.3)	24.6 (18.8–31.0)	27.8 (21.2–35.8)	28.9 (20.8–38.6)
Australia	65 to 69	26.5 (15.8–40.4)	24.0 (18.9–29.6)	23.2 (16.7–30.6)	29.7 (23.1–37.3)	31.9 (23.7–41.8)
Australia	70 to 74	19.5 (7.9–36.5)	19.1 (10.9–29.7)	19.6 (13.6–26.7)	21.4 (15.1–28.9)	22.1 (14.6–31.1)

Supplementary Table S10: Prevalence of female SVAC by age and location for 1990, 2000, 2010, 2020, and 2023						
**Location**	**Age Range**	**1990**	**2000**	**2010**	**2020**	**2023**
Australia	75 to 79	17.4 (6.8–33.4)	17.2 (9.0–28.0)	17.9 (11.8–25.4)	17.7 (10.2–27.9)	17.6 (9.0–30.3)
Australia	80 to 84	13.1 (4.5–27.7)	14.0 (5.5–27.6)	16.8 (8.4–28.4)	19.4 (10.5–31.4)	19.4 (10.0–32.7)
Australia	85 to 89	11.3 (3.8–24.5)	11.7 (4.4–24.1)	14.1 (6.2–26.7)	18.4 (10.9–29.5)	19.2 (11.5–30.9)
Australia	90 to 94	9.4 (3.1–20.7)	8.6 (3.0–18.8)	8.3 (3.0–17.3)	8.8 (3.2–19.8)	9.0 (3.2–20.3)
Australia	95 plus	9.3 (3.1–20.5)	8.5 (2.9–18.6)	8.2 (2.9–17.1)	8.7 (3.1–19.6)	8.9 (3.2–20.0)
Australia	Age-standardized	26.9 (16.3–40.7)	26.0 (21.4–30.8)	25.5 (22.2–29.2)	27.1 (22.9–31.8)	27.8 (22.8–34.1)
Australia	All age	26.6 (15.9–40.5)	25.5 (20.7–30.5)	25.1 (22.0–28.4)	26.6 (23.2–30.6)	27.2 (22.8–32.9)
New Zealand	20 to 24	37.4 (30.7–44.5)	29.8 (23.4–37.3)	23.0 (17.8–29.3)	23.8 (11.5–40.1)	24.5 (11.2–43.4)
New Zealand	25 to 29	34.3 (28.3–40.6)	32.8 (27.9–38.4)	25.4 (19.9–31.9)	24.8 (12.2–41.1)	25.3 (11.7–43.9)
New Zealand	30 to 34	37.3 (30.2–44.6)	32.7 (26.4–40.5)	27.0 (21.5–33.3)	27.2 (13.6–44.3)	27.7 (13.2–47.6)
New Zealand	35 to 39	33.3 (27.0–39.9)	31.2 (24.6–38.9)	31.1 (25.5–37.4)	32.6 (17.6–50.7)	32.8 (16.5–52.7)
New Zealand	40 to 44	32.1 (25.6–39.0)	29.7 (23.4–37.0)	31.0 (26.2–36.5)	34.7 (21.4–50.1)	35.2 (20.1–52.6)
New Zealand	45 to 49	38.1 (30.7–46.1)	31.8 (25.1–39.8)	30.1 (25.3–35.3)	34.1 (23.8–44.3)	34.9 (22.7–48.1)
New Zealand	50 to 54	33.8 (27.0–41.1)	31.2 (24.4–39.4)	29.6 (24.6–35.3)	30.4 (19.2–43.5)	30.7 (18.4–46.0)
New Zealand	55 to 59	32.9 (24.1–43.2)	27.7 (20.5–35.5)	25.7 (20.7–30.8)	28.5 (18.2–40.7)	29.3 (16.9–44.5)

Supplementary Table S10: Prevalence of female SVAC by age and location for 1990, 2000, 2010, 2020, and 2023						
**Location**	**Age Range**	**1990**	**2000**	**2010**	**2020**	**2023**
New Zealand	60 to 64	32.3 (23.3–42.9)	27.5 (20.3–35.5)	25.6 (20.5–30.7)	28.4 (18.3–40.3)	29.2 (17.0–44.1)
New Zealand	65 to 69	25.0 (10.1–45.8)	22.6 (11.6–36.2)	23.1 (13.7–34.5)	26.7 (16.3–41.2)	27.6 (15.8–43.2)
New Zealand	70 to 74	24.9 (9.3–47.6)	25.3 (9.7–47.9)	26.0 (11.2–46.8)	26.7 (13.7–46.0)	26.8 (13.4–44.8)
New Zealand	75 to 79	22.4 (8.1–44.2)	22.2 (7.7–43.9)	22.3 (8.0–43.3)	22.4 (8.5–45.6)	22.4 (8.6–45.2)
New Zealand	80 to 84	16.6 (5.7–34.9)	16.5 (5.4–34.7)	16.5 (5.6–34.1)	16.7 (5.9–36.3)	16.7 (6.0–35.9)
New Zealand	85 to 89	15.1 (5.1–32.4)	15.1 (4.8–32.2)	15.1 (5.0–31.6)	15.2 (5.3–33.6)	15.2 (5.4–33.3)
New Zealand	90 to 94	13.8 (4.6–30.0)	13.8 (4.3–29.8)	13.7 (4.5–29.2)	13.9 (4.8–31.2)	13.9 (4.8–30.9)
New Zealand	95 plus	13.7 (4.5–29.8)	13.6 (4.3–29.6)	13.6 (4.5–29.0)	13.8 (4.7–31.0)	13.8 (4.8–30.7)
New Zealand	Age-standardized	33.1 (26.7–40.5)	29.5 (23.5–36.5)	26.7 (21.7–32.5)	28.3 (19.4–39.5)	28.8 (19.0–41.1)
New Zealand	All age	32.6 (25.8–40.5)	29.0 (23.0–36.2)	26.6 (21.6–32.4)	28.0 (20.1–37.9)	28.4 (19.9–39.6)
High-income Asia Pacific	20 to 24	23.3 (8.5–45.5)	23.2 (8.1–45.4)	23.2 (8.4–44.7)	23.4 (8.9–47.0)	23.4 (9.0–46.6)
High-income Asia Pacific	25 to 29	23.7 (8.7–46.2)	23.6 (8.3–46.0)	23.7 (8.6–45.3)	23.8 (9.1–47.6)	23.8 (9.2–47.2)
High-income Asia Pacific	30 to 34	25.3 (9.5–48.4)	25.2 (9.0–48.3)	25.2 (9.4–47.6)	25.4 (9.9–49.9)	25.4 (10.0–49.4)
High-income Asia Pacific	35 to 39	25.4 (9.5–48.6)	25.4 (9.1–48.5)	25.4 (9.4–47.8)	25.6 (10.0–50.2)	25.6 (10.1–49.6)
High-income Asia Pacific	40 to 44	26.0 (9.8–49.5)	26.0 (9.4–49.4)	26.0 (9.8–48.7)	26.2 (10.3–51.1)	26.2 (10.4–50.5)

Supplementary Table S10: Prevalence of female SVAC by age and location for 1990, 2000, 2010, 2020, and 2023						
**Location**	**Age Range**	**1990**	**2000**	**2010**	**2020**	**2023**
High-income Asia Pacific	45 to 49	25.4 (9.5–48.5)	25.3 (9.1–48.4)	25.4 (9.4–47.8)	25.6 (10.0–50.2)	25.6 (10.1–49.6)
High-income Asia Pacific	50 to 54	24.8 (9.2–47.7)	24.7 (8.8–47.6)	24.8 (9.2–47.0)	25.0 (9.7–49.4)	25.0 (9.8–48.9)
High-income Asia Pacific	55 to 59	24.0 (8.9–46.5)	23.9 (8.5–46.5)	24.0 (8.8–45.9)	24.2 (9.3–48.2)	24.2 (9.4–47.8)
High-income Asia Pacific	60 to 64	23.2 (8.5–45.4)	23.2 (8.1–45.3)	23.2 (8.4–44.8)	23.5 (8.9–47.1)	23.5 (9.1–46.7)
High-income Asia Pacific	65 to 69	21.7 (7.8–43.1)	21.6 (7.5–43.0)	21.7 (7.8–42.5)	21.9 (8.2–44.8)	21.9 (8.3–44.4)
High-income Asia Pacific	70 to 74	17.7 (6.1–36.7)	17.6 (5.8–36.7)	17.7 (6.1–36.1)	17.9 (6.4–38.4)	17.9 (6.5–37.9)
High-income Asia Pacific	75 to 79	16.0 (5.4–33.9)	16.0 (5.2–33.8)	16.1 (5.4–33.3)	16.2 (5.7–35.5)	16.2 (5.8–35.1)
High-income Asia Pacific	80 to 84	11.6 (3.7–25.7)	11.6 (3.5–25.7)	11.6 (3.7–25.3)	11.7 (3.9–27.1)	11.7 (4.0–26.7)
High-income Asia Pacific	85 to 89	10.4 (3.3–23.5)	10.4 (3.2–23.4)	10.5 (3.3–23.1)	10.6 (3.5–24.8)	10.6 (3.6–24.5)
High-income Asia Pacific	90 to 94	9.4 (3.0–21.5)	9.4 (2.8–21.5)	9.5 (3.0–21.1)	9.6 (3.1–22.7)	9.6 (3.2–22.4)
High-income Asia Pacific	95 plus	9.3 (2.9–21.3)	9.3 (2.8–21.2)	9.4 (2.9–20.9)	9.5 (3.1–22.5)	9.5 (3.2–22.2)
High-income Asia Pacific	Age-standardized	23.5 (8.7–45.7)	23.5 (8.3–45.6)	23.5 (8.6–45.0)	23.7 (9.1–47.3)	23.7 (9.2–46.8)
High-income Asia Pacific	All age	23.4 (8.6–45.5)	22.9 (8.1–44.7)	22.4 (8.1–43.2)	22.0 (8.4–44.5)	21.8 (8.4–43.7)
Brunei	20 to 24	23.3 (8.5–45.5)	23.2 (8.1–45.4)	23.2 (8.4–44.7)	23.4 (8.9–47.0)	23.4 (9.0–46.6)
Brunei	25 to 29	23.7 (8.7–46.2)	23.6 (8.3–46.0)	23.7 (8.6–45.3)	23.8 (9.1–47.6)	23.8 (9.2–47.2)

Supplementary Table S10: Prevalence of female SVAC by age and location for 1990, 2000, 2010, 2020, and 2023						
**Location**	**Age Range**	**1990**	**2000**	**2010**	**2020**	**2023**
Brunei	30 to 34	25.3 (9.5–48.4)	25.2 (9.0–48.3)	25.2 (9.4–47.6)	25.4 (9.9–49.9)	25.4 (10.0–49.4)
Brunei	35 to 39	25.4 (9.5–48.6)	25.4 (9.1–48.5)	25.4 (9.4–47.8)	25.6 (10.0–50.2)	25.6 (10.1–49.6)
Brunei	40 to 44	26.0 (9.8–49.5)	26.0 (9.4–49.4)	26.0 (9.8–48.7)	26.2 (10.3–51.1)	26.2 (10.4–50.5)
Brunei	45 to 49	25.4 (9.5–48.5)	25.3 (9.1–48.4)	25.4 (9.4–47.8)	25.6 (10.0–50.2)	25.6 (10.1–49.6)
Brunei	50 to 54	24.8 (9.2–47.7)	24.7 (8.8–47.6)	24.8 (9.2–47.0)	25.0 (9.7–49.4)	25.0 (9.8–48.9)
Brunei	55 to 59	24.0 (8.9–46.5)	23.9 (8.5–46.5)	24.0 (8.8–45.9)	24.2 (9.3–48.2)	24.2 (9.4–47.8)
Brunei	60 to 64	23.2 (8.5–45.4)	23.2 (8.1–45.3)	23.2 (8.4–44.8)	23.5 (8.9–47.1)	23.5 (9.1–46.7)
Brunei	65 to 69	21.7 (7.8–43.1)	21.6 (7.5–43.0)	21.7 (7.8–42.5)	21.9 (8.2–44.8)	21.9 (8.3–44.4)
Brunei	70 to 74	17.7 (6.1–36.7)	17.6 (5.8–36.7)	17.7 (6.1–36.1)	17.9 (6.4–38.4)	17.9 (6.5–37.9)
Brunei	75 to 79	16.0 (5.4–33.9)	16.0 (5.2–33.8)	16.1 (5.4–33.3)	16.2 (5.7–35.5)	16.2 (5.8–35.1)
Brunei	80 to 84	11.6 (3.7–25.7)	11.6 (3.5–25.7)	11.6 (3.7–25.3)	11.7 (3.9–27.1)	11.7 (4.0–26.7)
Brunei	85 to 89	10.4 (3.3–23.5)	10.4 (3.2–23.4)	10.5 (3.3–23.1)	10.6 (3.5–24.8)	10.6 (3.6–24.5)
Brunei	90 to 94	9.4 (3.0–21.5)	9.4 (2.8–21.5)	9.5 (3.0–21.1)	9.6 (3.1–22.7)	9.6 (3.2–22.4)
Brunei	95 plus	9.3 (2.9–21.3)	9.3 (2.8–21.2)	9.4 (2.9–20.9)	9.5 (3.1–22.5)	9.5 (3.2–22.2)
Brunei	Age-standardized	23.5 (8.7–45.7)	23.5 (8.3–45.6)	23.5 (8.6–45.0)	23.7 (9.1–47.3)	23.7 (9.2–46.8)

Supplementary Table S10: Prevalence of female SVAC by age and location for 1990, 2000, 2010, 2020, and 2023						
**Location**	**Age Range**	**1990**	**2000**	**2010**	**2020**	**2023**
Brunei	All age	24.2 (9.0–46.7)	24.3 (8.6–46.8)	24.3 (8.9–46.1)	24.2 (9.3–48.1)	24.1 (9.4–47.5)
Japan	20 to 24	23.3 (8.5–45.5)	23.2 (8.1–45.4)	23.2 (8.4–44.7)	23.4 (8.9–47.0)	23.4 (9.0–46.6)
Japan	25 to 29	23.7 (8.7–46.2)	23.6 (8.3–46.0)	23.7 (8.6–45.3)	23.8 (9.1–47.6)	23.8 (9.2–47.2)
Japan	30 to 34	25.3 (9.5–48.4)	25.2 (9.0–48.3)	25.2 (9.4–47.6)	25.4 (9.9–49.9)	25.4 (10.0–49.4)
Japan	35 to 39	25.4 (9.5–48.6)	25.4 (9.1–48.5)	25.4 (9.4–47.8)	25.6 (10.0–50.2)	25.6 (10.1–49.6)
Japan	40 to 44	26.0 (9.8–49.5)	26.0 (9.4–49.4)	26.0 (9.8–48.7)	26.2 (10.3–51.1)	26.2 (10.4–50.5)
Japan	45 to 49	25.4 (9.5–48.5)	25.3 (9.1–48.4)	25.4 (9.4–47.8)	25.6 (10.0–50.2)	25.6 (10.1–49.6)
Japan	50 to 54	24.8 (9.2–47.7)	24.7 (8.8–47.6)	24.8 (9.2–47.0)	25.0 (9.7–49.4)	25.0 (9.8–48.9)
Japan	55 to 59	24.0 (8.9–46.5)	23.9 (8.5–46.5)	24.0 (8.8–45.9)	24.2 (9.3–48.2)	24.2 (9.4–47.8)
Japan	60 to 64	23.2 (8.5–45.4)	23.2 (8.1–45.3)	23.2 (8.4–44.8)	23.5 (8.9–47.1)	23.5 (9.1–46.7)
Japan	65 to 69	21.7 (7.8–43.1)	21.6 (7.5–43.0)	21.7 (7.8–42.5)	21.9 (8.2–44.8)	21.9 (8.3–44.4)
Japan	70 to 74	17.7 (6.1–36.7)	17.6 (5.8–36.7)	17.7 (6.1–36.1)	17.9 (6.4–38.4)	17.9 (6.5–37.9)
Japan	75 to 79	16.0 (5.4–33.9)	16.0 (5.2–33.8)	16.1 (5.4–33.3)	16.2 (5.7–35.5)	16.2 (5.8–35.1)
Japan	80 to 84	11.6 (3.7–25.7)	11.6 (3.5–25.7)	11.6 (3.7–25.3)	11.7 (3.9–27.1)	11.7 (4.0–26.7)
Japan	85 to 89	10.4 (3.3–23.5)	10.4 (3.2–23.4)	10.5 (3.3–23.1)	10.6 (3.5–24.8)	10.6 (3.6–24.5)

Supplementary Table S10: Prevalence of female SVAC by age and location for 1990, 2000, 2010, 2020, and 2023						
**Location**	**Age Range**	**1990**	**2000**	**2010**	**2020**	**2023**
Japan	90 to 94	9.4 (3.0–21.5)	9.4 (2.8–21.5)	9.5 (3.0–21.1)	9.6 (3.1–22.7)	9.6 (3.2–22.4)
Japan	95 plus	9.3 (2.9–21.3)	9.3 (2.8–21.2)	9.4 (2.9–20.9)	9.5 (3.1–22.5)	9.5 (3.2–22.2)
Japan	Age-standardized	23.5 (8.7–45.7)	23.5 (8.3–45.6)	23.5 (8.6–45.0)	23.7 (9.1–47.3)	23.7 (9.2–46.8)
Japan	All age	23.2 (8.6–45.2)	22.6 (7.9–44.2)	22.0 (8.0–42.5)	21.5 (8.1–43.6)	21.3 (8.1–42.8)
South Korea	20 to 24	23.3 (8.5–45.5)	23.2 (8.1–45.4)	23.2 (8.4–44.7)	23.4 (8.9–47.0)	23.4 (9.0–46.6)
South Korea	25 to 29	23.7 (8.7–46.2)	23.6 (8.3–46.0)	23.7 (8.6–45.3)	23.8 (9.1–47.6)	23.8 (9.2–47.2)
South Korea	30 to 34	25.3 (9.5–48.4)	25.2 (9.0–48.3)	25.2 (9.4–47.6)	25.4 (9.9–49.9)	25.4 (10.0–49.4)
South Korea	35 to 39	25.4 (9.5–48.6)	25.4 (9.1–48.5)	25.4 (9.4–47.8)	25.6 (10.0–50.2)	25.6 (10.1–49.6)
South Korea	40 to 44	26.0 (9.8–49.5)	26.0 (9.4–49.4)	26.0 (9.8–48.7)	26.2 (10.3–51.1)	26.2 (10.4–50.5)
South Korea	45 to 49	25.4 (9.5–48.5)	25.3 (9.1–48.4)	25.4 (9.4–47.8)	25.6 (10.0–50.2)	25.6 (10.1–49.6)
South Korea	50 to 54	24.8 (9.2–47.7)	24.7 (8.8–47.6)	24.8 (9.2–47.0)	25.0 (9.7–49.4)	25.0 (9.8–48.9)
South Korea	55 to 59	24.0 (8.9–46.5)	23.9 (8.5–46.5)	24.0 (8.8–45.9)	24.2 (9.3–48.2)	24.2 (9.4–47.8)
South Korea	60 to 64	23.2 (8.5–45.4)	23.2 (8.1–45.3)	23.2 (8.4–44.8)	23.5 (8.9–47.1)	23.5 (9.1–46.7)
South Korea	65 to 69	21.7 (7.8–43.1)	21.6 (7.5–43.0)	21.7 (7.8–42.5)	21.9 (8.2–44.8)	21.9 (8.3–44.4)
South Korea	70 to 74	17.7 (6.1–36.7)	17.6 (5.8–36.7)	17.7 (6.1–36.1)	17.9 (6.4–38.4)	17.9 (6.5–37.9)

Supplementary Table S10: Prevalence of female SVAC by age and location for 1990, 2000, 2010, 2020, and 2023						
**Location**	**Age Range**	**1990**	**2000**	**2010**	**2020**	**2023**
South Korea	75 to 79	16.0 (5.4–33.9)	16.0 (5.2–33.8)	16.1 (5.4–33.3)	16.2 (5.7–35.5)	16.2 (5.8–35.1)
South Korea	80 to 84	11.6 (3.7–25.7)	11.6 (3.5–25.7)	11.6 (3.7–25.3)	11.7 (3.9–27.1)	11.7 (4.0–26.7)
South Korea	85 to 89	10.4 (3.3–23.5)	10.4 (3.2–23.4)	10.5 (3.3–23.1)	10.6 (3.5–24.8)	10.6 (3.6–24.5)
South Korea	90 to 94	9.4 (3.0–21.5)	9.4 (2.8–21.5)	9.5 (3.0–21.1)	9.6 (3.1–22.7)	9.6 (3.2–22.4)
South Korea	95 plus	9.3 (2.9–21.3)	9.3 (2.8–21.2)	9.4 (2.9–20.9)	9.5 (3.1–22.5)	9.5 (3.2–22.2)
South Korea	Age-standardized	23.5 (8.7–45.7)	23.5 (8.3–45.6)	23.5 (8.6–45.0)	23.7 (9.1–47.3)	23.7 (9.2–46.8)
South Korea	All age	23.9 (8.9–46.3)	23.8 (8.4–46.1)	23.5 (8.6–44.9)	23.1 (8.8–46.3)	22.9 (8.8–45.6)
Singapore	20 to 24	23.3 (8.5–45.5)	23.2 (8.1–45.4)	23.2 (8.4–44.7)	23.4 (8.9–47.0)	23.4 (9.0–46.6)
Singapore	25 to 29	23.7 (8.7–46.2)	23.6 (8.3–46.0)	23.7 (8.6–45.3)	23.8 (9.1–47.6)	23.8 (9.2–47.2)
Singapore	30 to 34	25.3 (9.5–48.4)	25.2 (9.0–48.3)	25.2 (9.4–47.6)	25.4 (9.9–49.9)	25.4 (10.0–49.4)
Singapore	35 to 39	25.4 (9.5–48.6)	25.4 (9.1–48.5)	25.4 (9.4–47.8)	25.6 (10.0–50.2)	25.6 (10.1–49.6)
Singapore	40 to 44	26.0 (9.8–49.5)	26.0 (9.4–49.4)	26.0 (9.8–48.7)	26.2 (10.3–51.1)	26.2 (10.4–50.5)
Singapore	45 to 49	25.4 (9.5–48.5)	25.3 (9.1–48.4)	25.4 (9.4–47.8)	25.6 (10.0–50.2)	25.6 (10.1–49.6)
Singapore	50 to 54	24.8 (9.2–47.7)	24.7 (8.8–47.6)	24.8 (9.2–47.0)	25.0 (9.7–49.4)	25.0 (9.8–48.9)
Singapore	55 to 59	24.0 (8.9–46.5)	23.9 (8.5–46.5)	24.0 (8.8–45.9)	24.2 (9.3–48.2)	24.2 (9.4–47.8)

Supplementary Table S10: Prevalence of female SVAC by age and location for 1990, 2000, 2010, 2020, and 2023						
**Location**	**Age Range**	**1990**	**2000**	**2010**	**2020**	**2023**
Singapore	60 to 64	23.2 (8.5–45.4)	23.2 (8.1–45.3)	23.2 (8.4–44.8)	23.5 (8.9–47.1)	23.5 (9.1–46.7)
Singapore	65 to 69	21.7 (7.8–43.1)	21.6 (7.5–43.0)	21.7 (7.8–42.5)	21.9 (8.2–44.8)	21.9 (8.3–44.4)
Singapore	70 to 74	17.7 (6.1–36.7)	17.6 (5.8–36.7)	17.7 (6.1–36.1)	17.9 (6.4–38.4)	17.9 (6.5–37.9)
Singapore	75 to 79	16.0 (5.4–33.9)	16.0 (5.2–33.8)	16.1 (5.4–33.3)	16.2 (5.7–35.5)	16.2 (5.8–35.1)
Singapore	80 to 84	11.6 (3.7–25.7)	11.6 (3.5–25.7)	11.6 (3.7–25.3)	11.7 (3.9–27.1)	11.7 (4.0–26.7)
Singapore	85 to 89	10.4 (3.3–23.5)	10.4 (3.2–23.4)	10.5 (3.3–23.1)	10.6 (3.5–24.8)	10.6 (3.6–24.5)
Singapore	90 to 94	9.4 (3.0–21.5)	9.4 (2.8–21.5)	9.5 (3.0–21.1)	9.6 (3.1–22.7)	9.6 (3.2–22.4)
Singapore	95 plus	9.3 (2.9–21.3)	9.3 (2.8–21.2)	9.4 (2.9–20.9)	9.5 (3.1–22.5)	9.5 (3.2–22.2)
Singapore	Age-standardized	23.5 (8.7–45.7)	23.5 (8.3–45.6)	23.5 (8.6–45.0)	23.7 (9.1–47.3)	23.7 (9.2–46.8)
Singapore	All age	24.0 (8.9–46.4)	23.9 (8.5–46.3)	23.8 (8.7–45.4)	23.7 (9.1–47.2)	23.5 (9.1–46.4)
High-income North America	20 to 24	28.3 (25.4–31.1)	26.3 (23.7–29.0)	27.1 (23.6–30.9)	27.5 (18.7–39.1)	27.5 (16.9–41.8)
High-income North America	25 to 29	27.2 (24.3–30.2)	26.7 (24.0–29.6)	27.2 (24.1–30.3)	27.9 (22.2–34.7)	27.8 (20.4–37.9)
High-income North America	30 to 34	28.1 (25.2–31.3)	27.3 (24.5–30.4)	27.8 (24.7–31.1)	27.5 (24.6–30.9)	27.5 (23.3–32.7)
High-income North America	35 to 39	26.3 (23.5–29.3)	27.3 (24.2–30.6)	28.6 (25.4–31.9)	27.8 (23.9–31.9)	27.8 (22.5–34.1)
High-income North America	40 to 44	24.9 (22.2–27.8)	27.0 (24.0–30.3)	28.9 (25.8–32.2)	27.7 (24.2–31.6)	27.2 (22.4–32.8)

Supplementary Table S10: Prevalence of female SVAC by age and location for 1990, 2000, 2010, 2020, and 2023						
**Location**	**Age Range**	**1990**	**2000**	**2010**	**2020**	**2023**
High-income North America	45 to 49	24.6 (21.9–27.6)	26.7 (23.7–30.0)	28.6 (25.5–31.9)	28.7 (24.9–32.9)	28.7 (23.5–35.0)
High-income North America	50 to 54	21.6 (19.2–24.4)	25.2 (22.9–27.7)	28.9 (26.1–31.9)	28.5 (25.7–31.5)	28.3 (24.1–32.7)
High-income North America	55 to 59	20.9 (17.9–24.2)	22.6 (20.3–25.2)	27.3 (24.4–30.3)	28.1 (25.4–31.1)	27.8 (23.8–32.3)
High-income North America	60 to 64	21.2 (17.5–25.3)	20.3 (18.1–22.8)	25.9 (23.0–28.9)	28.3 (25.5–31.3)	28.4 (24.4–32.8)
High-income North America	65 to 69	18.3 (13.7–23.4)	18.1 (15.1–21.3)	23.3 (20.6–26.4)	26.6 (23.0–30.2)	26.7 (22.0–32.3)
High-income North America	70 to 74	14.6 (10.4–19.6)	15.3 (12.0–18.9)	19.9 (17.2–22.9)	25.0 (21.2–28.9)	25.7 (21.0–31.3)
High-income North America	75 to 79	13.3 (8.9–18.8)	13.1 (9.3–17.8)	17.5 (14.4–20.8)	22.5 (19.3–25.8)	23.4 (19.0–28.5)
High-income North America	80 to 84	8.4 (4.4–14.0)	9.6 (5.9–14.5)	14.4 (11.5–17.8)	18.2 (15.3–21.4)	18.2 (14.6–22.7)
High-income North America	85 to 89	8.4 (4.2–14.8)	9.4 (5.6–14.5)	13.3 (10.2–16.8)	17.2 (13.7–21.3)	17.6 (13.3–22.7)
High-income North America	90 to 94	8.7 (4.3–15.5)	10.1 (5.8–16.0)	12.5 (9.7–16.0)	12.2 (8.6–17.6)	11.9 (7.8–18.4)
High-income North America	95 plus	9.1 (4.4–16.1)	11.0 (6.1–17.5)	12.6 (9.9–16.1)	11.3 (7.5–17.3)	10.9 (6.7–18.5)
High-income North America	Age-standardized	23.8 (21.3–26.5)	24.3 (22.6–26.1)	26.6 (24.6–28.8)	27.3 (24.0–31.1)	27.2 (22.9–32.4)
High-income North America	All age	23.2 (20.6–26.0)	23.6 (21.8–25.5)	26.0 (24.0–28.2)	26.8 (24.1–29.7)	26.7 (23.2–30.8)
Canada	20 to 24	22.9 (17.5–29.5)	21.9 (17.0–27.8)	23.7 (18.7–29.5)	23.9 (13.9–36.2)	23.9 (12.3–38.7)
Canada	25 to 29	22.4 (17.1–29.0)	23.0 (18.0–29.0)	26.1 (21.1–31.7)	26.2 (15.7–38.7)	26.1 (13.9–41.4)

Supplementary Table S10: Prevalence of female SVAC by age and location for 1990, 2000, 2010, 2020, and 2023						
**Location**	**Age Range**	**1990**	**2000**	**2010**	**2020**	**2023**
Canada	30 to 34	23.5 (18.1–30.0)	24.2 (19.3–30.2)	26.3 (21.6–31.6)	25.3 (15.5–36.9)	25.1 (13.7–39.7)
Canada	35 to 39	22.4 (17.0–28.9)	25.0 (20.0–31.0)	29.8 (25.0–34.9)	26.6 (18.6–36.7)	25.7 (16.0–38.8)
Canada	40 to 44	20.8 (15.5–27.1)	24.6 (19.6–30.5)	29.7 (24.9–34.9)	27.5 (19.5–37.7)	26.8 (16.9–40.0)
Canada	45 to 49	20.4 (15.2–26.7)	24.6 (19.6–30.5)	29.4 (24.6–34.5)	27.1 (19.3–37.1)	26.4 (16.8–39.3)
Canada	50 to 54	17.7 (12.7–23.9)	23.1 (18.1–29.0)	30.2 (25.4–35.4)	28.4 (20.4–38.7)	27.6 (17.8–40.6)
Canada	55 to 59	17.3 (11.8–24.4)	20.4 (15.5–26.2)	26.0 (21.4–30.9)	25.7 (18.0–35.4)	25.4 (15.9–38.0)
Canada	60 to 64	17.3 (11.2–25.1)	18.9 (14.1–24.5)	24.9 (20.3–29.9)	26.1 (18.2–35.5)	26.0 (16.3–38.6)
Canada	65 to 69	15.9 (7.5–28.5)	16.7 (11.2–23.4)	21.3 (16.8–26.3)	23.6 (16.0–32.9)	23.9 (14.6–36.1)
Canada	70 to 74	14.1 (5.9–26.8)	14.0 (8.5–21.2)	16.3 (12.2–21.1)	19.2 (12.5–28.4)	19.8 (11.5–31.4)
Canada	75 to 79	13.6 (5.4–26.2)	13.9 (7.8–21.9)	15.3 (10.9–20.6)	16.0 (9.7–24.0)	16.2 (8.9–26.1)
Canada	80 to 84	10.0 (3.5–21.2)	10.0 (4.2–19.2)	11.0 (6.0–18.1)	12.5 (6.6–20.8)	12.8 (6.0–22.1)
Canada	85 to 89	9.0 (3.1–19.4)	8.9 (3.6–17.2)	9.5 (4.6–16.9)	10.6 (5.3–18.8)	10.9 (4.8–20.3)
Canada	90 to 94	8.1 (2.8–17.9)	7.8 (3.0–15.8)	7.7 (3.3–14.8)	8.0 (3.1–16.9)	8.1 (2.8–18.0)
Canada	95 plus	8.1 (2.8–17.7)	7.7 (3.0–15.7)	7.7 (3.3–14.7)	7.9 (3.0–16.8)	8.0 (2.8–17.9)
Canada	Age-standardized	20.0 (15.3–26.0)	21.8 (17.1–27.4)	25.7 (21.5–30.7)	25.1 (18.8–32.2)	24.8 (17.5–34.2)

Supplementary Table S10: Prevalence of female SVAC by age and location for 1990, 2000, 2010, 2020, and 2023						
**Location**	**Age Range**	**1990**	**2000**	**2010**	**2020**	**2023**
Canada	All age	19.8 (15.0–26.0)	21.4 (16.6–26.9)	25.1 (21.0–29.8)	24.2 (18.6–30.9)	23.8 (17.1–32.6)
Greenland	20 to 24	27.3 (8.5–54.7)	27.1 (9.5–51.3)	27.2 (10.3–50.2)	27.7 (9.4–55.2)	27.8 (9.1–56.1)
Greenland	25 to 29	27.5 (8.6–54.9)	27.2 (9.6–51.6)	27.3 (10.4–50.5)	27.9 (9.5–55.4)	28.0 (9.2–56.4)
Greenland	30 to 34	28.9 (9.2–56.8)	28.7 (10.3–53.5)	28.8 (11.1–52.4)	29.3 (10.2–57.4)	29.5 (9.8–58.3)
Greenland	35 to 39	28.9 (9.2–56.7)	28.6 (10.2–53.4)	28.8 (11.1–52.4)	29.3 (10.1–57.3)	29.4 (9.8–58.2)
Greenland	40 to 44	29.4 (9.4–57.4)	29.1 (10.5–54.1)	29.3 (11.4–53.0)	29.8 (10.4–58.0)	29.9 (10.0–58.9)
Greenland	45 to 49	28.5 (9.0–56.3)	28.3 (10.1–53.0)	28.5 (11.0–51.9)	29.0 (10.0–56.9)	29.1 (9.7–57.8)
Greenland	50 to 54	27.8 (8.7–55.3)	27.6 (9.7–52.0)	27.7 (10.6–51.0)	28.3 (9.7–56.0)	28.4 (9.3–56.9)
Greenland	55 to 59	26.9 (8.3–54.1)	26.7 (9.3–50.8)	26.8 (10.1–49.8)	27.4 (9.3–54.8)	27.5 (8.9–55.7)
Greenland	60 to 64	26.1 (8.0–52.9)	25.8 (8.9–49.6)	26.0 (9.7–48.6)	26.5 (8.9–53.6)	26.7 (8.6–54.6)
Greenland	65 to 69	24.4 (7.3–50.6)	24.2 (8.2–47.3)	24.3 (8.9–46.3)	24.9 (8.2–51.4)	25.0 (7.9–52.3)
Greenland	70 to 74	20.1 (5.7–43.9)	19.9 (6.4–40.7)	20.0 (7.0–39.8)	20.5 (6.4–44.7)	20.6 (6.2–45.7)
Greenland	75 to 79	18.3 (5.1–40.9)	18.1 (5.7–37.8)	18.2 (6.2–36.9)	18.7 (5.7–41.7)	18.8 (5.5–42.6)
Greenland	80 to 84	13.4 (3.5–31.8)	13.2 (3.9–29.1)	13.2 (4.3–28.3)	13.7 (3.9–31.9)	13.8 (3.8–32.6)
Greenland	85 to 89	12.2 (3.1–29.3)	11.9 (3.5–26.7)	12.0 (3.8–25.9)	12.4 (3.5–30.0)	12.5 (3.4–30.8)

Supplementary Table S10: Prevalence of female SVAC by age and location for 1990, 2000, 2010, 2020, and 2023						
**Location**	**Age Range**	**1990**	**2000**	**2010**	**2020**	**2023**
Greenland	90 to 94	11.0 (2.8–27.0)	10.8 (3.1–24.5)	10.9 (3.4–23.8)	11.2 (3.1–27.7)	11.3 (3.0–28.4)
Greenland	95 plus	10.9 (2.7–26.8)	10.7 (3.1–24.3)	10.7 (3.4–23.6)	11.1 (3.1–27.4)	11.2 (3.0–28.2)
Greenland	Age-standardized	26.8 (8.4–53.8)	26.6 (9.3–50.5)	26.7 (10.2–49.5)	27.3 (9.3–54.4)	27.4 (8.9–55.3)
Greenland	All age	27.5 (8.6–54.7)	27.3 (9.7–51.6)	27.2 (10.3–50.1)	27.4 (9.3–54.6)	27.5 (9.0–55.5)
USA	20 to 24	28.8 (25.9–31.7)	26.8 (24.0–29.8)	27.5 (23.5–31.7)	27.9 (17.1–41.6)	27.8 (14.9–44.4)
USA	25 to 29	27.7 (25.0–30.5)	27.0 (24.2–30.3)	27.3 (24.1–30.5)	28.0 (20.5–36.7)	28.0 (18.2–40.5)
USA	30 to 34	28.6 (25.8–31.5)	27.7 (24.9–30.8)	28.0 (24.9–31.2)	27.7 (23.4–32.7)	27.7 (21.8–34.9)
USA	35 to 39	26.7 (24.0–29.6)	27.6 (24.6–30.7)	28.5 (25.4–31.7)	27.9 (23.8–32.4)	28.1 (22.3–34.7)
USA	40 to 44	25.3 (22.7–28.1)	27.2 (24.4–30.4)	28.9 (25.8–32.1)	27.7 (24.0–31.8)	27.2 (22.0–33.3)
USA	45 to 49	25.1 (22.4–27.9)	26.9 (24.1–30.1)	28.5 (25.5–31.7)	28.9 (24.8–33.4)	29.0 (23.2–35.8)
USA	50 to 54	22.1 (19.7–24.8)	25.4 (22.6–28.5)	28.8 (25.8–31.7)	28.5 (24.3–32.7)	28.3 (22.4–34.3)
USA	55 to 59	21.3 (18.4–24.5)	22.9 (20.2–25.9)	27.4 (24.7–30.4)	28.4 (24.2–32.6)	28.2 (22.3–34.1)
USA	60 to 64	21.7 (18.0–25.7)	20.5 (17.9–23.5)	26.0 (23.2–28.9)	28.6 (24.3–32.7)	28.7 (22.7–34.7)
USA	65 to 69	18.5 (14.4–23.1)	18.3 (15.3–21.4)	23.6 (20.9–26.5)	27.0 (23.1–31.0)	27.0 (21.4–33.6)
USA	70 to 74	14.7 (10.8–19.2)	15.5 (12.0–19.2)	20.3 (17.6–23.2)	25.7 (21.7–29.7)	26.4 (21.3–32.6)

Supplementary Table S10: Prevalence of female SVAC by age and location for 1990, 2000, 2010, 2020, and 2023						
**Location**	**Age Range**	**1990**	**2000**	**2010**	**2020**	**2023**
USA	75 to 79	13.3 (9.1–18.3)	13.0 (9.4–17.5)	17.7 (14.8–21.1)	23.3 (19.5–27.2)	24.3 (19.1–30.3)
USA	80 to 84	8.2 (4.5–13.6)	9.5 (6.0–14.3)	14.8 (11.9–18.2)	19.0 (15.7–22.5)	18.9 (14.7–24.2)
USA	85 to 89	8.3 (4.3–14.4)	9.5 (5.7–14.6)	13.7 (10.5–17.4)	18.0 (14.3–22.4)	18.5 (13.8–24.3)
USA	90 to 94	8.7 (4.4–15.2)	10.4 (6.0–16.0)	13.0 (9.9–16.7)	12.7 (7.5–19.5)	12.4 (6.5–20.7)
USA	95 plus	9.2 (4.5–16.0)	11.2 (6.4–17.7)	13.2 (10.0–16.9)	11.7 (6.5–18.9)	11.3 (5.4–20.6)
USA	Age-standardized	24.3 (21.9–26.5)	24.6 (23.0–26.3)	26.7 (24.9–28.7)	27.5 (23.4–32.4)	27.5 (22.0–33.9)
USA	All age	23.6 (21.2–26.0)	23.8 (22.1–25.6)	26.1 (24.3–28.1)	27.1 (23.8–30.7)	27.1 (22.7–32.2)
Southern Latin America	20 to 24	22.9 (9.9–41.2)	22.6 (10.7–38.7)	22.9 (10.3–39.8)	22.5 (9.0–43.4)	22.2 (9.2–43.2)
Southern Latin America	25 to 29	20.9 (8.7–38.4)	19.8 (8.3–35.9)	19.8 (8.2–35.7)	20.9 (8.1–41.4)	21.0 (8.4–41.2)
Southern Latin America	30 to 34	23.8 (10.4–42.5)	23.9 (10.9–41.2)	23.4 (10.4–40.5)	23.8 (9.6–45.5)	23.9 (10.0–45.7)
Southern Latin America	35 to 39	22.2 (9.6–40.0)	22.4 (9.7–39.4)	22.2 (9.5–39.0)	22.6 (9.0–43.6)	22.8 (9.5–44.0)
Southern Latin America	40 to 44	24.5 (10.9–43.2)	25.2 (12.0–42.2)	25.4 (11.6–43.1)	24.6 (10.2–45.9)	24.7 (10.5–46.7)
Southern Latin America	45 to 49	23.1 (10.2–41.1)	23.2 (10.9–39.6)	24.1 (10.7–41.4)	23.9 (9.7–45.7)	23.6 (9.9–45.1)
Southern Latin America	50 to 54	23.0 (10.1–41.1)	23.4 (10.8–40.7)	24.0 (10.5–41.8)	24.1 (9.7–46.2)	23.9 (9.9–45.8)
Southern Latin America	55 to 59	21.2 (9.2–38.5)	21.3 (9.4–38.0)	21.8 (9.3–38.7)	22.9 (9.0–44.1)	22.9 (9.3–44.3)

Supplementary Table S10: Prevalence of female SVAC by age and location for 1990, 2000, 2010, 2020, and 2023						
**Location**	**Age Range**	**1990**	**2000**	**2010**	**2020**	**2023**
Southern Latin America	60 to 64	19.1 (8.1–35.3)	18.8 (7.8–34.5)	19.4 (8.0–35.4)	21.1 (8.1–41.3)	21.5 (8.5–42.2)
Southern Latin America	65 to 69	19.2 (7.4–37.4)	19.5 (6.8–39.1)	19.9 (7.1–39.3)	20.5 (7.3–41.5)	20.7 (7.7–42.2)
Southern Latin America	70 to 74	15.7 (5.8–31.8)	15.9 (5.3–33.1)	16.2 (5.5–33.3)	16.6 (5.7–35.3)	16.8 (6.0–35.9)
Southern Latin America	75 to 79	14.2 (5.2–29.2)	14.1 (4.6–30.1)	14.6 (4.9–30.5)	15.1 (5.1–32.6)	15.1 (5.4–33.1)
Southern Latin America	80 to 84	10.3 (3.6–22.0)	10.3 (3.2–22.9)	10.5 (3.4–23.0)	10.9 (3.5–24.7)	11.0 (3.7–25.3)
Southern Latin America	85 to 89	9.4 (3.2–20.2)	9.3 (2.9–20.9)	9.4 (3.0–20.8)	9.9 (3.1–22.7)	9.9 (3.3–23.2)
Southern Latin America	90 to 94	8.5 (2.9–18.5)	8.6 (2.6–19.3)	8.7 (2.7–19.4)	9.0 (2.8–21.0)	9.1 (3.0–21.4)
Southern Latin America	95 plus	8.4 (2.9–18.3)	8.3 (2.5–18.8)	8.5 (2.7–19.0)	8.4 (2.6–20.0)	8.5 (2.9–20.1)
Southern Latin America	Age-standardized	21.5 (9.2–39.0)	21.4 (9.4–38.2)	21.6 (9.2–38.4)	22.0 (8.7–42.9)	22.0 (9.0–42.8)
Southern Latin America	All age	21.4 (9.1–38.9)	21.2 (9.2–38.0)	21.4 (9.1–38.0)	21.6 (8.5–42.5)	21.6 (8.8–42.2)
Argentina	20 to 24	19.9 (8.6–37.0)	20.5 (10.3–33.9)	20.4 (9.8–34.9)	19.8 (8.0–38.7)	19.6 (7.7–39.4)
Argentina	25 to 29	16.1 (6.4–31.5)	15.0 (6.6–27.6)	15.4 (6.6–28.5)	16.8 (6.4–34.5)	17.1 (6.5–35.5)
Argentina	30 to 34	20.2 (8.6–37.5)	20.2 (9.7–34.0)	20.3 (9.5–35.0)	20.4 (8.2–39.8)	20.3 (8.0–40.6)
Argentina	35 to 39	18.2 (7.6–34.4)	17.2 (8.1–29.6)	17.6 (8.1–31.0)	18.7 (7.4–37.2)	19.0 (7.4–38.5)
Argentina	40 to 44	21.6 (9.4–39.4)	21.8 (11.2–35.6)	21.8 (10.7–37.0)	21.6 (8.9–41.5)	21.5 (8.7–42.5)

Supplementary Table S10: Prevalence of female SVAC by age and location for 1990, 2000, 2010, 2020, and 2023						
**Location**	**Age Range**	**1990**	**2000**	**2010**	**2020**	**2023**
Argentina	45 to 49	19.6 (8.3–36.5)	19.2 (9.4–32.3)	19.4 (9.1–33.6)	19.9 (8.0–38.9)	20.0 (7.9–40.1)
Argentina	50 to 54	20.3 (8.7–37.6)	20.4 (9.9–34.2)	20.5 (9.7–35.3)	20.4 (8.2–39.7)	20.3 (8.0–40.6)
Argentina	55 to 59	18.4 (7.6–34.8)	17.9 (8.4–31.1)	18.2 (8.3–32.1)	18.7 (7.3–37.3)	18.8 (7.3–38.2)
Argentina	60 to 64	16.1 (6.3–31.6)	15.1 (6.5–28.0)	15.5 (6.5–28.7)	16.7 (6.3–34.4)	17.0 (6.4–35.5)
Argentina	65 to 69	16.9 (5.8–35.4)	16.9 (5.5–35.3)	16.9 (5.7–34.8)	17.0 (6.1–36.9)	17.0 (6.1–36.5)
Argentina	70 to 74	13.7 (4.5–29.7)	13.6 (4.3–29.6)	13.7 (4.5–29.1)	13.8 (4.7–31.0)	13.8 (4.8–30.7)
Argentina	75 to 79	12.4 (4.0–27.2)	12.3 (3.8–27.2)	12.4 (4.0–26.7)	12.5 (4.2–28.5)	12.5 (4.3–28.2)
Argentina	80 to 84	8.8 (2.8–20.3)	8.8 (2.6–20.2)	8.8 (2.7–19.8)	8.9 (2.9–21.3)	8.9 (2.9–21.1)
Argentina	85 to 89	8.0 (2.5–18.5)	8.0 (2.4–18.4)	8.0 (2.4–18.1)	8.1 (2.6–19.5)	8.1 (2.6–19.2)
Argentina	90 to 94	7.2 (2.2–16.9)	7.2 (2.1–16.8)	7.2 (2.2–16.5)	7.3 (2.3–17.8)	7.3 (2.4–17.6)
Argentina	95 plus	7.2 (2.2–16.7)	7.2 (2.1–16.7)	7.2 (2.2–16.4)	7.2 (2.3–17.7)	7.2 (2.3–17.4)
Argentina	Age-standardized	18.2 (7.5–34.7)	17.9 (8.3–31.7)	18.1 (8.2–32.2)	18.5 (7.2–36.8)	18.5 (7.2–37.7)
Argentina	All age	18.1 (7.4–34.6)	17.7 (8.2–31.5)	17.8 (8.0–31.9)	18.2 (7.1–36.2)	18.2 (7.0–37.1)
Chile	20 to 24	30.4 (11.0–56.8)	30.4 (11.8–55.1)	30.5 (12.0–54.6)	31.0 (11.5–56.5)	31.1 (11.3–57.2)
Chile	25 to 29	30.8 (11.3–57.4)	30.8 (12.1–55.7)	31.0 (12.3–55.1)	31.5 (11.8–57.0)	31.5 (11.5–57.8)

Supplementary Table S10: Prevalence of female SVAC by age and location for 1990, 2000, 2010, 2020, and 2023						
**Location**	**Age Range**	**1990**	**2000**	**2010**	**2020**	**2023**
Chile	30 to 34	32.6 (12.2–59.5)	32.6 (13.0–57.8)	32.8 (13.3–57.3)	33.3 (12.7–59.2)	33.4 (12.4–59.9)
Chile	35 to 39	32.8 (12.3–59.7)	32.8 (13.1–58.0)	32.9 (13.3–57.5)	33.4 (12.8–59.4)	33.5 (12.5–60.1)
Chile	40 to 44	33.5 (12.7–60.6)	33.5 (13.5–58.9)	33.7 (13.8–58.4)	34.2 (13.2–60.2)	34.3 (12.9–61.0)
Chile	45 to 49	32.8 (12.3–59.7)	32.8 (13.1–58.0)	32.9 (13.4–57.5)	33.4 (12.8–59.4)	33.5 (12.5–60.1)
Chile	50 to 54	32.2 (11.9–59.0)	32.1 (12.8–57.3)	32.3 (13.0–56.8)	32.8 (12.5–58.4)	32.9 (12.2–59.3)
Chile	55 to 59	31.3 (11.5–57.9)	31.3 (12.3–56.2)	31.4 (12.5–55.7)	31.9 (12.0–57.6)	32.0 (11.8–58.3)
Chile	60 to 64	30.5 (11.1–56.9)	30.4 (11.9–55.2)	30.6 (12.1–54.7)	31.1 (11.6–56.6)	31.2 (11.3–57.3)
Chile	65 to 69	28.8 (10.2–54.7)	28.7 (11.0–53.0)	28.9 (11.2–52.5)	29.4 (10.7–54.4)	29.5 (10.5–55.2)
Chile	70 to 74	24.0 (8.1–48.2)	24.0 (8.7–46.4)	24.1 (8.8–45.9)	24.5 (8.4–47.9)	24.6 (8.3–48.6)
Chile	75 to 79	22.0 (7.2–45.2)	21.9 (7.7–43.4)	22.0 (7.9–42.9)	22.5 (7.5–44.8)	22.6 (7.4–45.6)
Chile	80 to 84	16.4 (5.0–35.9)	16.3 (5.4–34.3)	16.3 (5.5–33.8)	16.7 (5.3–35.6)	16.8 (5.1–36.3)
Chile	85 to 89	14.9 (4.5–33.2)	14.8 (4.8–31.7)	14.9 (4.9–31.3)	15.2 (4.7–33.0)	15.3 (4.6–33.6)
Chile	90 to 94	13.6 (4.0–30.8)	13.6 (4.3–29.4)	13.6 (4.4–28.9)	13.9 (4.2–30.6)	14.0 (4.1–31.2)
Chile	95 plus	13.5 (4.0–30.6)	13.4 (4.3–29.2)	13.5 (4.4–28.7)	13.8 (4.2–30.3)	13.9 (4.1–31.0)
Chile	Age-standardized	30.7 (11.3–57.0)	30.7 (12.1–55.3)	30.8 (12.3–54.7)	31.3 (11.7–56.6)	31.4 (11.5–57.4)

Supplementary Table S10: Prevalence of female SVAC by age and location for 1990, 2000, 2010, 2020, and 2023						
**Location**	**Age Range**	**1990**	**2000**	**2010**	**2020**	**2023**
Chile	All age	30.9 (11.3–57.2)	30.8 (12.1–55.4)	30.7 (12.2–54.6)	30.9 (11.6–56.0)	30.9 (11.3–56.7)
Uruguay	20 to 24	14.5 (5.5–29.0)	13.4 (5.8–25.2)	13.6 (5.9–25.5)	14.8 (5.6–30.8)	15.1 (5.7–32.1)
Uruguay	25 to 29	17.4 (7.0–33.3)	17.4 (8.2–30.4)	17.5 (8.2–30.8)	17.5 (7.0–35.4)	17.5 (6.8–35.7)
Uruguay	30 to 34	18.0 (7.1–34.5)	17.6 (8.1–30.9)	17.7 (8.1–31.2)	18.2 (7.3–36.5)	18.3 (7.2–37.4)
Uruguay	35 to 39	18.5 (6.4–38.1)	18.5 (6.1–38.0)	18.5 (6.4–37.4)	18.7 (6.7–39.6)	18.6 (6.8–39.2)
Uruguay	40 to 44	18.2 (7.3–35.1)	17.5 (8.0–31.2)	17.7 (8.1–31.4)	18.5 (7.4–37.0)	18.7 (7.3–38.0)
Uruguay	45 to 49	21.7 (9.2–39.9)	22.8 (12.0–36.5)	22.7 (11.6–37.4)	21.6 (9.0–40.3)	21.2 (8.1–40.4)
Uruguay	50 to 54	18.4 (6.4–37.9)	18.3 (6.1–37.8)	18.4 (6.3–37.2)	18.5 (6.7–39.4)	18.5 (6.7–39.0)
Uruguay	55 to 59	17.7 (6.1–36.7)	17.6 (5.8–36.6)	17.7 (6.0–36.1)	17.8 (6.4–38.2)	17.8 (6.5–37.8)
Uruguay	60 to 64	15.5 (6.0–30.7)	14.6 (6.4–26.9)	14.8 (6.5–27.1)	15.9 (6.1–32.6)	16.1 (6.1–33.8)
Uruguay	65 to 69	15.9 (5.3–33.6)	15.8 (5.1–33.5)	15.9 (5.3–33.0)	16.0 (5.6–35.1)	16.0 (5.7–34.6)
Uruguay	70 to 74	12.8 (4.2–28.1)	12.8 (4.0–28.0)	12.8 (4.1–27.5)	12.9 (4.4–29.4)	12.9 (4.5–29.0)
Uruguay	75 to 79	11.6 (3.7–25.7)	11.5 (3.5–25.6)	11.6 (3.7–25.2)	11.7 (3.9–27.0)	11.7 (4.0–26.6)
Uruguay	80 to 84	8.2 (2.6–19.0)	8.2 (2.4–19.0)	8.2 (2.5–18.6)	8.3 (2.7–20.0)	8.3 (2.7–19.8)
Uruguay	85 to 89	7.4 (2.3–17.3)	7.4 (2.2–17.3)	7.4 (2.3–16.9)	7.5 (2.4–18.3)	7.5 (2.4–18.0)

Supplementary Table S10: Prevalence of female SVAC by age and location for 1990, 2000, 2010, 2020, and 2023						
**Location**	**Age Range**	**1990**	**2000**	**2010**	**2020**	**2023**
Uruguay	90 to 94	6.7 (2.1–15.8)	6.7 (2.0–15.8)	6.7 (2.0–15.4)	6.8 (2.2–16.7)	6.8 (2.2–16.4)
Uruguay	95 plus	6.7 (2.0–15.7)	6.7 (1.9–15.6)	6.7 (2.0–15.3)	6.7 (2.1–16.5)	6.7 (2.2–16.3)
Uruguay	Age-standardized	17.0 (6.5–33.8)	16.7 (7.1–31.8)	16.8 (7.1–31.3)	17.2 (6.6–34.9)	17.2 (6.5–35.6)
Uruguay	All age	16.6 (6.3–33.2)	16.3 (6.8–31.1)	16.3 (6.8–30.6)	16.5 (6.3–34.1)	16.5 (6.2–34.4)
Western Europe	20 to 24	20.6 (13.1–31.1)	19.7 (14.6–26.5)	18.7 (15.3–22.4)	18.8 (14.1–24.4)	18.9 (14.0–25.3)
Western Europe	25 to 29	20.9 (13.0–31.9)	20.9 (15.3–28.0)	20.4 (16.5–24.7)	20.7 (15.1–27.0)	20.7 (14.5–28.5)
Western Europe	30 to 34	22.2 (13.3–34.4)	21.8 (15.2–29.7)	20.7 (16.8–25.0)	21.1 (14.9–28.5)	21.3 (14.2–30.1)
Western Europe	35 to 39	22.7 (12.8–35.9)	23.4 (15.7–33.0)	23.8 (19.2–29.1)	24.0 (20.1–29.0)	24.1 (20.0–30.4)
Western Europe	40 to 44	22.5 (12.6–35.8)	23.3 (15.4–33.2)	23.8 (19.1–28.7)	23.8 (19.8–29.2)	23.9 (19.5–30.4)
Western Europe	45 to 49	22.3 (12.9–34.9)	22.8 (15.1–32.6)	23.0 (18.8–27.6)	23.2 (19.3–28.2)	23.0 (19.1–29.0)
Western Europe	50 to 54	21.6 (11.9–34.8)	22.7 (15.1–32.5)	23.8 (19.6–28.5)	24.2 (20.0–29.4)	24.2 (19.9–30.4)
Western Europe	55 to 59	20.0 (11.1–32.1)	19.8 (12.8–29.0)	20.0 (16.1–24.6)	20.5 (16.8–25.5)	20.6 (16.9–26.7)
Western Europe	60 to 64	19.4 (10.2–32.0)	19.3 (11.2–29.9)	19.7 (15.5–24.5)	20.3 (16.5–25.7)	20.4 (16.2–26.8)
Western Europe	65 to 69	18.0 (8.5–31.5)	17.5 (9.4–28.4)	17.0 (13.0–21.9)	17.8 (15.0–22.4)	17.9 (14.8–23.3)
Western Europe	70 to 74	14.4 (6.3–26.8)	13.4 (6.4–23.0)	12.3 (8.6–16.7)	12.9 (10.2–17.8)	13.1 (10.1–18.4)

Supplementary Table S10: Prevalence of female SVAC by age and location for 1990, 2000, 2010, 2020, and 2023						
**Location**	**Age Range**	**1990**	**2000**	**2010**	**2020**	**2023**
Western Europe	75 to 79	14.3 (5.4–28.8)	14.0 (4.9–29.0)	13.6 (5.0–27.4)	13.7 (5.4–29.1)	13.8 (5.8–28.8)
Western Europe	80 to 84	10.3 (3.5–22.2)	10.1 (3.1–22.4)	9.9 (3.2–21.6)	10.0 (3.3–23.6)	10.1 (3.4–23.5)
Western Europe	85 to 89	9.4 (3.1–20.4)	9.1 (2.8–20.6)	9.0 (2.9–19.9)	8.9 (2.9–21.4)	9.0 (3.0–21.3)
Western Europe	90 to 94	8.6 (2.9–19.0)	8.4 (2.5–19.1)	8.2 (2.6–18.3)	8.2 (2.6–19.8)	8.1 (2.7–19.5)
Western Europe	95 plus	8.6 (2.9–18.8)	8.3 (2.5–19.0)	8.2 (2.6–18.2)	8.1 (2.6–19.7)	8.1 (2.7–19.4)
Western Europe	Age-standardized	20.5 (11.7–32.6)	20.5 (13.8–29.5)	20.4 (16.6–24.8)	20.6 (17.6–25.1)	20.7 (17.5–26.1)
Western Europe	All age	19.8 (11.1–31.8)	19.7 (12.7–28.9)	19.4 (15.5–24.1)	19.5 (17.5–22.8)	19.5 (17.7–23.3)
Andorra	20 to 24	20.2 (7.2–40.9)	20.1 (6.8–40.7)	20.0 (7.0–39.8)	20.1 (7.4–41.9)	20.1 (7.5–41.5)
Andorra	25 to 29	20.6 (7.3–41.5)	20.5 (7.0–41.3)	20.4 (7.2–40.5)	20.5 (7.6–42.6)	20.5 (7.7–42.2)
Andorra	30 to 34	22.1 (8.0–43.8)	22.0 (7.6–43.6)	21.9 (7.8–42.7)	22.0 (8.2–44.9)	22.0 (8.3–44.4)
Andorra	35 to 39	22.3 (8.1–44.0)	22.2 (7.7–43.8)	22.1 (7.9–43.0)	22.2 (8.3–45.2)	22.2 (8.4–44.7)
Andorra	40 to 44	22.9 (8.3–44.9)	22.8 (7.9–44.7)	22.7 (8.2–43.9)	22.8 (8.6–46.1)	22.8 (8.8–45.7)
Andorra	45 to 49	22.3 (8.1–44.0)	22.2 (7.7–43.9)	22.1 (7.9–43.1)	22.2 (8.3–45.3)	22.2 (8.5–44.8)
Andorra	50 to 54	21.7 (7.8–43.2)	21.6 (7.5–43.0)	21.6 (7.7–42.3)	21.7 (8.1–44.4)	21.7 (8.2–44.0)
Andorra	55 to 59	20.9 (7.5–42.0)	20.9 (7.1–41.8)	20.8 (7.3–41.1)	20.9 (7.7–43.2)	20.9 (7.9–42.8)

Supplementary Table S10: Prevalence of female SVAC by age and location for 1990, 2000, 2010, 2020, and 2023						
**Location**	**Age Range**	**1990**	**2000**	**2010**	**2020**	**2023**
Andorra	60 to 64	20.2 (7.2–40.8)	20.1 (6.8–40.7)	20.1 (7.0–39.9)	20.1 (7.4–42.0)	20.1 (7.5–41.6)
Andorra	65 to 69	18.8 (6.5–38.5)	18.7 (6.2–38.4)	18.6 (6.4–37.6)	18.7 (6.8–39.7)	18.7 (6.8–39.3)
Andorra	70 to 74	15.1 (5.1–32.3)	15.1 (4.8–32.2)	15.0 (5.0–31.5)	15.1 (5.2–33.4)	15.1 (5.3–32.8)
Andorra	75 to 79	13.6 (4.5–29.6)	13.6 (4.3–29.5)	13.5 (4.4–28.8)	13.6 (4.6–30.6)	13.6 (4.7–30.2)
Andorra	80 to 84	9.7 (3.1–22.1)	9.7 (2.9–22.0)	9.6 (3.0–21.4)	9.7 (3.2–22.9)	9.7 (3.2–22.6)
Andorra	85 to 89	8.7 (2.7–20.1)	8.7 (2.6–19.9)	8.6 (2.7–19.4)	8.7 (2.8–20.8)	8.7 (2.8–20.5)
Andorra	90 to 94	7.9 (2.4–18.3)	7.8 (2.3–18.2)	7.8 (2.4–17.7)	7.8 (2.5–19.0)	7.8 (2.6–18.7)
Andorra	95 plus	7.8 (2.4–18.1)	7.8 (2.3–18.0)	7.7 (2.4–17.5)	7.7 (2.5–18.8)	7.7 (2.5–18.5)
Andorra	Age-standardized	20.5 (7.3–41.2)	20.4 (7.0–41.0)	20.4 (7.2–40.2)	20.5 (7.6–42.4)	20.4 (7.7–41.9)
Andorra	All age	20.6 (7.4–41.4)	20.3 (6.9–40.8)	20.2 (7.1–40.0)	20.0 (7.4–41.6)	20.0 (7.5–41.1)
Austria	20 to 24	12.7 (4.4–27.0)	11.2 (5.1–20.6)	9.8 (7.5–12.4)	10.3 (5.6–16.3)	10.7 (5.1–19.2)
Austria	25 to 29	13.4 (4.8–28.4)	12.3 (5.7–22.3)	11.1 (8.6–13.9)	11.5 (6.3–18.0)	11.8 (5.7–20.9)
Austria	30 to 34	14.2 (5.0–29.7)	12.7 (5.9–22.9)	11.2 (8.8–14.0)	11.7 (6.4–18.4)	12.1 (5.9–21.4)
Austria	35 to 39	15.2 (5.5–31.5)	14.3 (6.7–25.6)	13.3 (10.5–16.4)	13.6 (7.5–21.0)	13.8 (6.8–23.9)
Austria	40 to 44	15.4 (5.6–31.9)	14.4 (6.8–25.6)	13.2 (10.4–16.3)	13.6 (7.5–21.0)	13.9 (6.8–24.0)

Supplementary Table S10: Prevalence of female SVAC by age and location for 1990, 2000, 2010, 2020, and 2023						
**Location**	**Age Range**	**1990**	**2000**	**2010**	**2020**	**2023**
Austria	45 to 49	15.0 (5.4–31.1)	14.0 (6.5–25.0)	12.8 (10.1–15.8)	13.2 (7.3–20.4)	13.5 (6.6–23.3)
Austria	50 to 54	15.0 (5.4–31.2)	14.4 (6.8–25.6)	13.5 (10.7–16.6)	13.7 (7.6–21.2)	13.9 (6.8–24.0)
Austria	55 to 59	13.6 (4.8–28.7)	12.3 (5.7–22.3)	11.0 (8.6–13.7)	11.4 (6.2–17.9)	11.8 (5.7–20.8)
Austria	60 to 64	13.2 (4.7–28.0)	12.1 (5.6–22.0)	10.9 (8.5–13.6)	11.3 (6.2–17.7)	11.6 (5.6–20.6)
Austria	65 to 69	11.9 (4.1–25.6)	10.6 (4.8–19.6)	9.4 (7.2–12.0)	9.8 (5.3–15.7)	10.2 (4.8–18.4)
Austria	70 to 74	9.1 (3.0–20.1)	7.8 (3.4–14.7)	6.6 (4.7– 9.0)	7.1 (3.6–11.9)	7.4 (3.3–13.8)
Austria	75 to 79	9.5 (3.0–21.5)	9.4 (2.8–21.4)	9.3 (2.9–20.8)	9.4 (3.1–22.4)	9.4 (3.1–22.1)
Austria	80 to 84	6.7 (2.0–15.7)	6.6 (1.9–15.6)	6.6 (2.0–15.1)	6.6 (2.1–16.3)	6.6 (2.1–16.1)
Austria	85 to 89	6.0 (1.8–14.2)	6.0 (1.7–14.1)	5.9 (1.8–13.7)	6.0 (1.9–14.8)	6.0 (1.9–14.6)
Austria	90 to 94	5.4 (1.6–12.9)	5.4 (1.5–12.8)	5.3 (1.6–12.5)	5.4 (1.7–13.5)	5.4 (1.7–13.3)
Austria	95 plus	5.4 (1.6–12.8)	5.3 (1.5–12.7)	5.3 (1.6–12.3)	5.3 (1.7–13.3)	5.3 (1.7–13.2)
Austria	Age-standardized	13.5 (4.8–28.2)	12.4 (5.7–22.8)	11.3 (8.7–14.3)	11.6 (7.2–17.7)	11.9 (6.4–20.5)
Austria	All age	13.0 (4.6–27.0)	12.0 (5.5–22.1)	10.9 (8.3–14.2)	11.2 (7.8–16.3)	11.4 (7.2–18.8)
Belgium	20 to 24	14.6 (8.5–22.7)	14.8 (9.4–21.5)	15.6 (12.3–19.7)	16.9 (11.5–23.4)	17.1 (11.0–24.3)
Belgium	25 to 29	16.2 (9.6–24.6)	16.5 (10.8–23.5)	17.6 (14.2–21.7)	19.4 (13.5–26.3)	19.6 (13.0–27.4)

Supplementary Table S10: Prevalence of female SVAC by age and location for 1990, 2000, 2010, 2020, and 2023						
**Location**	**Age Range**	**1990**	**2000**	**2010**	**2020**	**2023**
Belgium	30 to 34	16.5 (9.8–24.9)	16.8 (11.0–23.9)	17.9 (14.4–22.0)	19.5 (13.6–26.5)	19.8 (13.2–27.6)
Belgium	35 to 39	18.7 (11.5–27.5)	19.2 (13.0–26.6)	20.8 (17.1–25.0)	23.2 (16.5–30.8)	23.5 (16.0–32.3)
Belgium	40 to 44	18.5 (11.3–27.2)	18.9 (12.8–26.3)	20.6 (16.9–24.7)	23.0 (16.3–30.5)	23.4 (15.9–32.0)
Belgium	45 to 49	18.0 (11.0–26.7)	18.5 (12.4–25.9)	20.0 (16.4–24.1)	22.3 (15.8–29.7)	22.6 (15.4–31.1)
Belgium	50 to 54	18.7 (11.4–27.6)	19.2 (12.9–26.7)	20.9 (17.2–25.0)	23.5 (16.8–31.1)	23.9 (16.4–32.5)
Belgium	55 to 59	15.8 (9.3–24.1)	16.1 (10.4–23.1)	17.3 (13.9–21.5)	19.1 (13.3–26.1)	19.4 (12.8–27.0)
Belgium	60 to 64	15.6 (9.2–24.0)	16.0 (10.3–22.9)	17.1 (13.7–21.3)	18.9 (13.2–25.9)	19.2 (12.6–26.8)
Belgium	65 to 69	13.9 (7.8–21.7)	14.1 (8.8–20.7)	14.8 (11.4–18.9)	16.1 (10.7–22.5)	16.3 (10.3–23.3)
Belgium	70 to 74	10.4 (5.6–17.5)	10.4 (6.1–16.6)	10.7 (7.4–14.7)	11.2 (6.9–16.8)	11.3 (6.7–17.7)
Belgium	75 to 79	12.7 (3.7–29.5)	12.7 (4.0–28.5)	12.7 (4.1–27.3)	13.0 (3.8–29.8)	13.1 (3.9–30.7)
Belgium	80 to 84	9.1 (2.5–22.1)	9.1 (2.8–21.3)	9.1 (2.8–20.3)	9.3 (2.6–22.4)	9.4 (2.7–23.1)
Belgium	85 to 89	8.2 (2.3–20.2)	8.2 (2.5–19.4)	8.2 (2.5–18.5)	8.4 (2.3–20.4)	8.5 (2.4–21.1)
Belgium	90 to 94	7.5 (2.0–18.5)	7.4 (2.2–17.7)	7.4 (2.3–16.8)	7.6 (2.1–18.7)	7.7 (2.1–19.3)
Belgium	95 plus	7.4 (2.0–18.3)	7.4 (2.2–17.6)	7.3 (2.2–16.7)	7.5 (2.1–18.5)	7.6 (2.1–19.1)
Belgium	Age-standardized	16.2 (9.6–24.6)	16.5 (10.8–23.5)	17.7 (14.3–21.6)	19.4 (14.2–25.9)	19.7 (14.0–26.8)

Supplementary Table S10: Prevalence of female SVAC by age and location for 1990, 2000, 2010, 2020, and 2023						
**Location**	**Age Range**	**1990**	**2000**	**2010**	**2020**	**2023**
Belgium	All age	15.7 (9.2–23.9)	16.0 (10.2–23.1)	16.9 (13.4–20.9)	18.4 (14.0–23.8)	18.6 (13.9–24.5)
Cyprus	20 to 24	11.6 (4.1–25.1)	10.2 (4.6–18.7)	8.8 (6.8–11.0)	9.3 (5.0–14.7)	9.7 (4.6–17.2)
Cyprus	25 to 29	12.4 (4.3–26.4)	11.1 (5.1–20.4)	9.9 (7.8–12.3)	10.3 (5.6–16.2)	10.7 (5.1–18.8)
Cyprus	30 to 34	13.0 (4.6–27.7)	11.5 (5.3–21.0)	10.1 (7.9–12.5)	10.6 (5.8–16.6)	11.0 (5.3–19.4)
Cyprus	35 to 39	14.0 (5.0–29.3)	13.0 (6.1–23.5)	11.9 (9.5–14.6)	12.2 (6.7–18.9)	12.5 (6.1–21.6)
Cyprus	40 to 44	14.2 (5.1–29.8)	13.1 (6.1–23.6)	11.9 (9.5–14.6)	12.3 (6.8–19.0)	12.6 (6.1–21.8)
Cyprus	45 to 49	13.8 (4.9–29.0)	12.7 (5.9–22.9)	11.5 (9.1–14.1)	11.9 (6.5–18.4)	12.2 (5.9–21.1)
Cyprus	50 to 54	13.8 (4.9–29.1)	13.0 (6.1–23.4)	12.0 (9.5–14.8)	12.3 (6.8–19.1)	12.5 (6.1–21.7)
Cyprus	55 to 59	12.5 (4.4–26.7)	11.1 (5.1–20.4)	9.8 (7.7–12.2)	10.3 (5.6–16.2)	10.6 (5.1–18.8)
Cyprus	60 to 64	12.2 (4.3–26.1)	11.0 (5.0–20.2)	9.8 (7.7–12.2)	10.2 (5.6–16.1)	10.5 (5.1–18.6)
Cyprus	65 to 69	11.0 (3.8–23.8)	9.6 (4.4–17.9)	8.4 (6.5–10.6)	8.8 (4.8–14.1)	9.2 (4.4–16.7)
Cyprus	70 to 74	8.3 (2.7–18.6)	7.0 (3.1–13.3)	5.9 (4.3– 7.9)	6.3 (3.3–10.6)	6.7 (3.0–12.5)
Cyprus	75 to 79	8.8 (2.7–20.2)	8.7 (2.6–20.0)	8.7 (2.7–19.5)	8.7 (2.8–20.9)	8.7 (2.9–20.6)
Cyprus	80 to 84	6.2 (1.9–14.6)	6.2 (1.8–14.5)	6.1 (1.8–14.1)	6.1 (1.9–15.2)	6.1 (2.0–15.0)
Cyprus	85 to 89	5.6 (1.7–13.3)	5.5 (1.6–13.2)	5.5 (1.6–12.7)	5.5 (1.7–13.8)	5.5 (1.8–13.6)

Supplementary Table S10: Prevalence of female SVAC by age and location for 1990, 2000, 2010, 2020, and 2023						
**Location**	**Age Range**	**1990**	**2000**	**2010**	**2020**	**2023**
Cyprus	90 to 94	5.0 (1.5–12.0)	5.0 (1.4–11.9)	4.9 (1.5–11.6)	5.0 (1.6–12.5)	5.0 (1.6–12.3)
Cyprus	95 plus	5.0 (1.5–11.9)	4.9 (1.4–11.8)	4.9 (1.5–11.5)	4.9 (1.5–12.4)	4.9 (1.6–12.2)
Cyprus	Age-standardized	12.4 (4.4–26.3)	11.3 (5.2–20.9)	10.1 (7.9–12.7)	10.5 (6.5–16.0)	10.8 (5.8–18.5)
Cyprus	All age	12.3 (4.4–26.1)	11.2 (5.1–20.8)	10.0 (7.8–12.7)	10.4 (6.8–15.6)	10.7 (6.2–18.1)
Denmark	20 to 24	20.6 (7.2–41.1)	19.5 (8.7–34.6)	18.0 (11.3–26.2)	18.0 (10.0–28.3)	18.0 (9.5–29.5)
Denmark	25 to 29	21.8 (7.7–42.9)	20.9 (9.4–36.6)	19.8 (12.9–27.9)	19.9 (11.4–30.7)	20.0 (10.8–32.1)
Denmark	30 to 34	22.6 (7.9–44.6)	21.6 (9.8–37.5)	20.3 (13.3–28.5)	20.4 (11.8–31.2)	20.4 (11.1–32.4)
Denmark	35 to 39	24.5 (8.8–47.1)	24.0 (10.9–40.9)	23.4 (16.0–32.1)	23.8 (14.3–34.6)	23.9 (13.7–36.3)
Denmark	40 to 44	24.7 (8.9–47.3)	24.0 (11.0–40.9)	23.4 (15.9–32.1)	23.7 (13.9–34.8)	23.8 (13.4–36.2)
Denmark	45 to 49	23.9 (8.4–46.4)	23.3 (10.6–39.8)	22.7 (15.4–31.4)	23.0 (13.5–34.0)	23.1 (12.8–35.3)
Denmark	50 to 54	24.2 (8.9–46.5)	23.7 (10.8–40.4)	23.4 (16.0–32.0)	23.9 (14.3–35.0)	24.0 (13.5–36.6)
Denmark	55 to 59	21.5 (7.4–42.5)	20.7 (9.3–36.3)	19.7 (12.8–27.8)	19.9 (11.4–30.6)	20.0 (10.8–31.8)
Denmark	60 to 64	21.0 (7.3–42.0)	20.3 (9.1–35.7)	19.4 (12.6–27.5)	19.7 (11.2–30.0)	19.8 (10.6–31.5)
Denmark	65 to 69	18.9 (6.5–38.3)	18.0 (7.9–32.3)	17.0 (10.5–25.1)	17.1 (9.3–27.1)	17.2 (8.9–28.2)
Denmark	70 to 74	14.6 (4.5–31.7)	13.8 (5.5–26.4)	12.7 (6.7–21.0)	12.7 (6.1–22.4)	12.8 (5.7–23.1)

Supplementary Table S10: Prevalence of female SVAC by age and location for 1990, 2000, 2010, 2020, and 2023						
**Location**	**Age Range**	**1990**	**2000**	**2010**	**2020**	**2023**
Denmark	75 to 79	15.0 (4.1–35.8)	14.6 (4.6–32.1)	14.5 (4.8–30.6)	14.8 (4.5–34.9)	14.8 (4.4–35.1)
Denmark	80 to 84	10.9 (2.7–27.6)	10.5 (3.1–24.2)	10.4 (3.3–23.0)	10.7 (3.0–26.6)	10.7 (3.0–26.8)
Denmark	85 to 89	9.8 (2.4–25.1)	9.5 (2.8–22.0)	9.4 (2.9–21.0)	9.6 (2.6–24.4)	9.7 (2.6–24.9)
Denmark	90 to 94	8.9 (2.2–21.9)	8.6 (2.5–20.1)	8.5 (2.6–19.2)	8.7 (2.4–22.4)	8.8 (2.4–22.6)
Denmark	95 plus	8.8 (2.2–22.2)	8.5 (2.5–20.1)	8.4 (2.6–19.0)	8.6 (2.4–22.0)	8.7 (2.4–22.4)
Denmark	Age-standardized	21.7 (7.8–43.0)	20.9 (9.5–36.6)	20.0 (13.7–27.2)	20.2 (11.9–30.1)	20.3 (11.4–31.4)
Denmark	All age	20.9 (7.4–41.6)	20.3 (9.0–36.0)	19.4 (13.0–26.8)	19.2 (11.0–29.0)	19.2 (10.7–30.1)
Finland	20 to 24	27.9 (19.3–36.8)	22.5 (13.7–33.3)	17.1 (12.1–23.1)	17.0 (9.2–27.9)	17.6 (8.4–31.4)
Finland	25 to 29	29.1 (20.5–38.9)	24.6 (15.6–35.3)	19.0 (13.9–24.9)	18.5 (10.3–30.0)	19.0 (9.3–33.7)
Finland	30 to 34	27.5 (19.4–36.8)	24.8 (16.6–34.4)	19.5 (14.2–25.4)	18.9 (10.8–30.5)	19.5 (10.0–34.0)
Finland	35 to 39	28.2 (19.8–38.1)	27.0 (19.0–36.5)	22.8 (17.5–28.7)	21.6 (12.5–33.0)	21.9 (10.9–36.3)
Finland	40 to 44	25.4 (17.0–36.1)	25.4 (17.7–34.1)	23.0 (17.9–28.8)	22.4 (13.0–34.3)	22.7 (11.6–38.6)
Finland	45 to 49	29.2 (19.6–40.8)	26.3 (18.1–35.5)	22.7 (17.7–28.3)	22.9 (13.8–33.9)	23.3 (12.5–38.7)
Finland	50 to 54	26.4 (17.0–37.9)	24.8 (16.3–34.3)	23.2 (18.1–29.1)	24.9 (16.4–35.6)	25.6 (15.5–38.5)
Finland	55 to 59	25.7 (16.7–36.8)	22.8 (14.4–32.9)	20.2 (15.3–26.0)	22.7 (14.3–33.0)	23.7 (13.8–36.8)

Supplementary Table S10: Prevalence of female SVAC by age and location for 1990, 2000, 2010, 2020, and 2023						
**Location**	**Age Range**	**1990**	**2000**	**2010**	**2020**	**2023**
Finland	60 to 64	26.5 (16.5–38.9)	22.7 (13.5–34.1)	19.6 (14.4–25.7)	21.8 (13.8–31.6)	22.7 (13.0–35.6)
Finland	65 to 69	20.7 (12.2–32.1)	18.0 (10.3–28.5)	16.7 (11.6–23.1)	20.5 (12.7–29.8)	21.8 (12.5–34.6)
Finland	70 to 74	17.6 (9.2–29.4)	14.7 (7.8–24.2)	12.8 (7.8–19.5)	15.4 (8.7–24.5)	16.5 (8.8–27.8)
Finland	75 to 79	17.9 (6.2–37.2)	17.8 (5.9–37.0)	17.8 (6.1–36.2)	18.0 (6.4–38.5)	18.0 (6.5–38.3)
Finland	80 to 84	13.1 (4.3–28.7)	13.0 (4.1–28.5)	13.0 (4.2–27.8)	13.1 (4.5–29.8)	13.2 (4.5–29.6)
Finland	85 to 89	11.9 (3.8–26.3)	11.8 (3.6–26.2)	11.8 (3.8–25.6)	11.9 (4.0–27.4)	11.9 (4.0–27.3)
Finland	90 to 94	10.8 (3.4–24.3)	10.7 (3.3–24.1)	10.7 (3.4–23.5)	10.8 (3.6–25.3)	10.9 (3.6–25.1)
Finland	95 plus	10.7 (3.4–24.1)	10.7 (3.2–23.9)	10.6 (3.3–23.3)	10.7 (3.6–25.1)	10.8 (3.6–24.9)
Finland	Age-standardized	26.1 (18.0–35.7)	23.4 (16.5–31.3)	19.8 (16.0–24.7)	20.2 (15.2–26.9)	20.8 (14.2–29.8)
Finland	All age	25.2 (17.2–35.2)	22.5 (15.6–30.6)	19.2 (15.1–24.1)	19.7 (17.4–23.4)	20.2 (16.6–26.0)
France	20 to 24	24.3 (9.4–45.7)	23.8 (11.8–39.1)	23.2 (17.0–30.6)	23.3 (13.3–35.6)	23.5 (11.7–38.2)
France	25 to 29	25.8 (10.1–47.8)	26.1 (13.2–42.3)	26.1 (19.5–33.7)	25.9 (14.9–38.7)	25.8 (13.2–41.6)
France	30 to 34	27.1 (10.8–49.5)	27.0 (13.9–43.6)	26.7 (20.1–34.2)	26.6 (15.4–39.6)	26.7 (13.8–42.8)
France	35 to 39	29.0 (11.8–52.0)	30.3 (16.0–47.9)	31.3 (24.1–39.5)	30.6 (18.2–44.4)	30.2 (16.1–47.0)
France	40 to 44	29.4 (12.0–52.6)	30.5 (16.0–48.2)	31.2 (24.1–39.2)	30.6 (18.2–44.4)	30.3 (16.1–47.4)

Supplementary Table S10: Prevalence of female SVAC by age and location for 1990, 2000, 2010, 2020, and 2023						
**Location**	**Age Range**	**1990**	**2000**	**2010**	**2020**	**2023**
France	45 to 49	28.7 (11.6–51.6)	29.6 (15.5–47.0)	30.2 (23.1–38.3)	29.7 (17.5–43.3)	29.4 (15.5–46.2)
France	50 to 54	28.8 (11.7–51.8)	30.4 (16.0–48.1)	31.6 (24.4–39.7)	30.8 (18.3–44.5)	30.3 (16.1–47.3)
France	55 to 59	26.2 (10.3–48.3)	26.2 (13.3–42.5)	26.0 (19.4–33.5)	25.9 (14.9–38.7)	25.9 (13.2–41.7)
France	60 to 64	25.6 (10.0–47.5)	25.8 (13.1–42.0)	25.8 (19.3–33.2)	25.6 (14.7–38.3)	25.5 (13.0–41.3)
France	65 to 69	23.2 (8.9–43.9)	22.8 (11.2–37.6)	22.3 (16.3–29.6)	22.4 (12.6–34.4)	22.5 (11.2–37.1)
France	70 to 74	18.0 (6.5–36.0)	16.9 (7.6–30.1)	15.9 (10.3–23.1)	16.6 (8.9–27.1)	16.9 (7.9–29.6)
France	75 to 79	17.2 (5.9–36.0)	17.2 (5.6–36.0)	17.3 (5.9–35.5)	17.4 (6.2–37.6)	17.4 (6.3–37.2)
France	80 to 84	12.5 (4.1–27.6)	12.5 (3.9–27.5)	12.6 (4.1–27.1)	12.7 (4.3–28.9)	12.7 (4.4–28.6)
France	85 to 89	11.3 (3.6–25.3)	11.3 (3.5–25.2)	11.4 (3.6–24.8)	11.4 (3.8–26.5)	11.5 (3.9–26.2)
France	90 to 94	10.3 (3.3–23.2)	10.3 (3.1–23.2)	10.3 (3.3–22.8)	10.4 (3.4–24.4)	10.4 (3.5–24.1)
France	95 plus	10.2 (3.2–23.0)	10.2 (3.1–23.0)	10.2 (3.2–22.6)	10.3 (3.4–24.2)	10.3 (3.5–23.9)
France	Age-standardized	25.9 (10.4–47.4)	26.3 (13.3–42.3)	26.4 (19.9–34.1)	26.2 (17.1–37.2)	26.0 (15.5–39.9)
France	All age	25.0 (10.0–45.8)	25.3 (12.8–41.1)	25.2 (18.8–32.9)	24.6 (18.4–33.5)	24.4 (16.9–35.3)
Germany	20 to 24	19.4 (7.1–38.4)	18.4 (8.8–31.9)	17.3 (13.0–22.4)	17.6 (9.7–27.6)	17.9 (8.8–30.8)
Germany	25 to 29	20.5 (7.7–40.2)	20.0 (9.9–34.2)	19.3 (14.8–24.5)	19.4 (11.0–29.9)	19.6 (9.8–33.1)

Supplementary Table S10: Prevalence of female SVAC by age and location for 1990, 2000, 2010, 2020, and 2023						
**Location**	**Age Range**	**1990**	**2000**	**2010**	**2020**	**2023**
Germany	30 to 34	21.6 (8.1–41.8)	20.7 (10.3–35.3)	19.8 (15.1–25.2)	20.0 (11.3–30.7)	20.3 (10.1–34.1)
Germany	35 to 39	23.1 (8.9–44.1)	23.4 (11.8–38.9)	23.3 (18.1–29.1)	23.1 (13.4–34.6)	23.0 (11.8–37.9)
Germany	40 to 44	23.5 (9.1–44.7)	23.6 (11.9–39.2)	23.3 (18.0–29.3)	23.2 (13.4–34.9)	23.2 (11.9–38.2)
Germany	45 to 49	22.8 (8.8–43.7)	22.8 (11.5–38.1)	22.5 (17.4–28.2)	22.4 (12.9–33.7)	22.4 (11.4–37.1)
Germany	50 to 54	22.9 (8.8–43.9)	23.4 (11.9–39.0)	23.6 (18.4–29.4)	23.3 (13.5–34.8)	23.1 (11.9–38.1)
Germany	55 to 59	20.8 (7.8–40.7)	20.2 (9.9–34.5)	19.3 (14.7–24.8)	19.6 (11.0–30.2)	19.7 (9.9–33.4)
Germany	60 to 64	20.3 (7.6–39.9)	19.9 (9.7–34.0)	19.2 (14.6–24.5)	19.3 (10.9–29.8)	19.5 (9.7–33.0)
Germany	65 to 69	18.4 (6.7–36.8)	17.5 (8.2–30.6)	16.5 (12.2–21.6)	16.8 (9.2–26.6)	17.1 (8.3–29.5)
Germany	70 to 74	14.2 (5.0–29.6)	12.9 (5.6–23.5)	11.7 (8.2–16.4)	12.2 (6.5–20.7)	12.6 (5.7–23.5)
Germany	75 to 79	13.9 (4.6–30.1)	13.9 (4.4–30.0)	13.9 (4.5–29.5)	14.0 (4.8–31.4)	14.0 (4.9–31.0)
Germany	80 to 84	10.0 (3.2–22.6)	10.0 (3.0–22.5)	10.0 (3.1–22.1)	10.0 (3.3–23.7)	10.0 (3.4–23.3)
Germany	85 to 89	9.0 (2.8–20.6)	9.0 (2.7–20.5)	9.0 (2.8–20.1)	9.0 (2.9–21.6)	9.0 (3.0–21.3)
Germany	90 to 94	8.2 (2.5–18.8)	8.1 (2.4–18.8)	8.1 (2.5–18.4)	8.2 (2.6–19.8)	8.2 (2.7–19.5)
Germany	95 plus	8.1 (2.5–18.7)	8.1 (2.4–18.6)	8.1 (2.5–18.2)	8.1 (2.6–19.6)	8.1 (2.7–19.3)
Germany	Age-standardized	20.6 (7.8–39.9)	20.2 (9.9–34.7)	19.7 (14.9–25.3)	19.8 (12.3–29.6)	19.9 (11.0–32.4)

Supplementary Table S10: Prevalence of female SVAC by age and location for 1990, 2000, 2010, 2020, and 2023						
**Location**	**Age Range**	**1990**	**2000**	**2010**	**2020**	**2023**
Germany	All age	19.8 (7.6–38.3)	19.5 (9.4–33.3)	18.7 (13.9–24.4)	18.5 (13.2–26.4)	18.5 (11.9–28.9)
Greece	20 to 24	12.9 (4.5–27.5)	11.3 (5.2–20.8)	9.9 (7.4–12.9)	10.4 (5.6–16.8)	10.8 (5.1–19.6)
Greece	25 to 29	13.7 (4.8–28.8)	12.4 (5.7–22.5)	11.1 (8.5–14.2)	11.6 (6.3–18.3)	11.9 (5.7–21.2)
Greece	30 to 34	14.4 (5.1–30.1)	12.8 (5.9–23.2)	11.3 (8.7–14.4)	11.9 (6.5–18.7)	12.3 (5.9–21.9)
Greece	35 to 39	15.4 (5.5–31.8)	14.4 (6.8–25.9)	13.4 (10.4–16.7)	13.7 (7.6–21.2)	13.9 (6.8–24.3)
Greece	40 to 44	15.6 (5.6–32.3)	14.5 (6.8–26.0)	13.3 (10.3–16.6)	13.7 (7.6–21.3)	14.0 (6.8–24.5)
Greece	45 to 49	15.2 (5.4–31.5)	14.1 (6.6–25.3)	12.9 (10.0–16.2)	13.3 (7.3–20.7)	13.6 (6.6–23.8)
Greece	50 to 54	15.2 (5.4–31.5)	14.4 (6.8–25.8)	13.5 (10.5–16.8)	13.7 (7.6–21.3)	13.9 (6.8–24.2)
Greece	55 to 59	13.7 (4.8–28.9)	12.4 (5.7–22.5)	11.0 (8.4–14.1)	11.5 (6.2–18.3)	11.9 (5.7–21.3)
Greece	60 to 64	13.4 (4.7–28.3)	12.2 (5.6–22.1)	10.9 (8.3–13.9)	11.4 (6.2–18.0)	11.7 (5.6–21.0)
Greece	65 to 69	12.1 (4.1–25.9)	10.7 (4.9–19.7)	9.5 (7.0–12.4)	9.9 (5.3–16.1)	10.3 (4.8–18.7)
Greece	70 to 74	9.2 (3.1–20.2)	7.9 (3.4–14.6)	6.7 (4.7– 9.6)	7.2 (3.7–12.2)	7.5 (3.3–13.9)
Greece	75 to 79	9.6 (3.0–21.8)	9.6 (2.9–21.7)	9.5 (3.0–21.1)	9.6 (3.1–22.7)	9.6 (3.2–22.4)
Greece	80 to 84	6.8 (2.1–15.9)	6.8 (2.0–15.8)	6.7 (2.0–15.4)	6.8 (2.1–16.6)	6.8 (2.2–16.4)
Greece	85 to 89	6.1 (1.8–14.4)	6.1 (1.8–14.4)	6.0 (1.8–13.9)	6.1 (1.9–15.0)	6.1 (1.9–14.8)

Supplementary Table S10: Prevalence of female SVAC by age and location for 1990, 2000, 2010, 2020, and 2023						
**Location**	**Age Range**	**1990**	**2000**	**2010**	**2020**	**2023**
Greece	90 to 94	5.5 (1.7–13.1)	5.5 (1.6–13.0)	5.4 (1.6–12.7)	5.5 (1.7–13.7)	5.5 (1.7–13.5)
Greece	95 plus	5.5 (1.6–13.0)	5.4 (1.6–12.9)	5.4 (1.6–12.6)	5.4 (1.7–13.6)	5.4 (1.7–13.4)
Greece	Age-standardized	13.7 (4.9–28.6)	12.5 (5.8–23.0)	11.3 (8.6–14.6)	11.7 (7.2–18.0)	12.1 (6.5–20.8)
Greece	All age	13.4 (4.8–27.8)	12.2 (5.6–22.4)	11.0 (8.1–14.5)	11.2 (7.9–16.3)	11.4 (7.4–18.5)
Iceland	20 to 24	25.3 (11.4–43.9)	25.0 (14.4–37.7)	24.7 (19.0–31.0)	23.8 (12.5–38.9)	23.6 (10.9–41.3)
Iceland	25 to 29	24.2 (8.0–47.7)	24.1 (10.1–42.6)	24.3 (14.9–36.4)	24.3 (14.5–36.3)	24.3 (13.2–39.1)
Iceland	30 to 34	24.9 (8.4–50.5)	24.7 (9.5–46.5)	24.7 (12.6–41.1)	24.6 (17.0–34.5)	24.6 (16.4–35.2)
Iceland	35 to 39	24.6 (7.5–51.3)	24.2 (8.5–48.4)	24.0 (8.8–45.9)	24.2 (9.3–48.2)	24.3 (9.2–48.9)
Iceland	40 to 44	25.2 (7.8–52.2)	24.8 (8.7–49.3)	24.7 (9.1–46.8)	24.8 (9.6–49.1)	24.9 (9.6–50.1)
Iceland	45 to 49	24.5 (7.5–51.3)	24.2 (8.5–48.1)	24.1 (8.8–45.9)	24.2 (9.3–48.2)	24.3 (9.2–48.9)
Iceland	50 to 54	24.0 (7.3–50.3)	23.7 (8.3–47.4)	23.5 (8.6–45.1)	23.7 (9.0–47.4)	23.7 (9.0–48.4)
Iceland	55 to 59	23.3 (7.0–49.1)	22.9 (7.7–46.0)	22.8 (8.2–44.0)	22.9 (8.7–46.3)	23.0 (8.7–47.0)
Iceland	60 to 64	22.5 (6.7–48.0)	22.2 (7.6–45.3)	22.0 (7.9–43.0)	22.2 (8.3–45.2)	22.2 (8.3–45.9)
Iceland	65 to 69	21.1 (6.2–45.7)	20.7 (7.0–43.0)	20.6 (7.3–40.7)	20.7 (7.7–42.9)	20.8 (7.7–43.7)
Iceland	70 to 74	17.2 (4.8–38.9)	16.9 (5.5–36.2)	16.7 (5.6–34.5)	16.8 (6.0–36.5)	16.9 (6.0–37.2)

Supplementary Table S10: Prevalence of female SVAC by age and location for 1990, 2000, 2010, 2020, and 2023						
**Location**	**Age Range**	**1990**	**2000**	**2010**	**2020**	**2023**
Iceland	75 to 79	15.6 (4.3–36.1)	15.3 (4.9–33.5)	15.1 (5.0–31.7)	15.2 (5.3–33.7)	15.3 (5.3–34.4)
Iceland	80 to 84	11.3 (2.9–27.8)	11.0 (3.3–25.3)	10.9 (3.5–23.9)	11.0 (3.7–25.6)	11.1 (3.7–26.2)
Iceland	85 to 89	10.2 (2.6–25.4)	10.0 (3.0–23.1)	9.8 (3.1–21.8)	9.9 (3.3–23.4)	10.0 (3.3–24.0)
Iceland	90 to 94	9.3 (2.3–23.4)	9.0 (2.7–21.3)	8.9 (2.8–20.0)	9.0 (2.9–21.5)	9.0 (2.9–22.0)
Iceland	95 plus	9.2 (2.3–23.2)	8.9 (2.6–21.1)	8.8 (2.7–19.8)	8.9 (2.9–21.3)	8.9 (2.9–21.8)
Iceland	Age-standardized	23.4 (8.3–46.5)	23.1 (9.3–43.1)	23.0 (11.7–38.9)	22.9 (14.0–37.8)	22.9 (13.6–37.5)
Iceland	All age	23.0 (8.2–46.0)	22.7 (8.9–42.9)	22.4 (10.9–38.9)	22.3 (12.8–38.5)	22.3 (12.3–38.2)
Ireland	20 to 24	27.4 (16.9–41.3)	24.6 (18.0–32.3)	19.6 (12.2–27.9)	17.1 (8.4–30.4)	16.8 (7.8–30.9)
Ireland	25 to 29	28.8 (18.2–42.6)	25.9 (19.2–33.4)	20.7 (13.2–28.9)	17.9 (9.2–30.8)	17.6 (8.6–31.4)
Ireland	30 to 34	30.9 (20.0–44.8)	27.6 (20.7–35.0)	21.6 (14.0–30.0)	18.5 (9.6–31.6)	18.2 (9.0–32.0)
Ireland	35 to 39	30.9 (19.9–44.4)	28.0 (21.2–35.7)	22.7 (15.2–30.8)	19.9 (10.7–32.7)	19.6 (10.0–33.2)
Ireland	40 to 44	29.7 (18.8–43.6)	27.1 (20.3–34.6)	22.3 (14.9–30.3)	19.9 (10.8–32.8)	19.6 (10.1–33.3)
Ireland	45 to 49	31.4 (20.4–45.3)	28.3 (21.5–36.0)	22.6 (15.0–30.7)	19.5 (10.5–32.3)	19.1 (9.8–32.8)
Ireland	50 to 54	29.9 (18.9–43.4)	27.3 (20.6–34.8)	22.5 (15.1–30.5)	19.8 (10.7–32.7)	19.5 (10.1–33.2)
Ireland	55 to 59	25.3 (15.2–38.8)	23.1 (16.6–30.9)	19.4 (12.3–27.5)	17.7 (9.0–30.8)	17.5 (8.5–31.5)

Supplementary Table S10: Prevalence of female SVAC by age and location for 1990, 2000, 2010, 2020, and 2023						
**Location**	**Age Range**	**1990**	**2000**	**2010**	**2020**	**2023**
Ireland	60 to 64	24.8 (14.7–38.2)	22.7 (16.1–30.5)	19.1 (12.1–27.3)	17.4 (8.9–30.4)	17.2 (8.3–31.0)
Ireland	65 to 69	20.9 (11.5–34.6)	19.3 (12.8–27.6)	16.7 (10.1–24.8)	15.8 (7.6–28.5)	15.7 (7.0–29.4)
Ireland	70 to 74	16.1 (8.0–27.9)	15.0 (9.0–22.9)	13.4 (7.2–22.3)	13.0 (5.5–25.8)	13.0 (5.1–26.3)
Ireland	75 to 79	14.7 (7.0–25.1)	14.3 (8.3–22.0)	14.3 (6.9–25.9)	14.6 (5.1–32.0)	14.7 (4.7–33.2)
Ireland	80 to 84	8.7 (2.9–19.4)	8.5 (3.4–16.9)	8.7 (3.2–17.8)	9.3 (2.8–22.4)	9.5 (2.8–22.9)
Ireland	85 to 89	8.1 (2.7–18.3)	7.9 (3.1–15.9)	8.1 (3.0–16.6)	8.6 (2.6–20.9)	8.7 (2.6–21.6)
Ireland	90 to 94	7.7 (2.5–17.3)	7.4 (2.9–15.0)	7.6 (2.8–15.6)	7.9 (2.4–19.6)	8.0 (2.3–20.0)
Ireland	95 plus	7.6 (2.5–16.9)	7.4 (2.9–14.9)	7.5 (2.7–15.5)	7.9 (2.3–19.4)	8.0 (2.3–19.8)
Ireland	Age-standardized	27.2 (17.4–40.2)	24.6 (18.2–32.1)	20.2 (13.5–27.7)	17.9 (9.7–29.6)	17.6 (9.1–30.1)
Ireland	All age	26.4 (17.0–39.2)	24.2 (17.8–31.6)	19.9 (13.5–27.3)	17.6 (9.8–28.9)	17.3 (9.2–29.3)
Israel	20 to 24	21.1 (7.6–42.3)	21.1 (7.2–42.2)	21.0 (7.4–41.3)	21.0 (7.8–43.5)	21.0 (7.9–43.0)
Israel	25 to 29	21.5 (7.7–42.8)	21.4 (7.4–42.7)	21.3 (7.6–41.8)	21.4 (8.0–44.0)	21.4 (8.1–43.5)
Israel	30 to 34	22.9 (8.4–45.0)	22.8 (8.0–44.8)	22.7 (8.2–44.0)	22.8 (8.6–46.1)	22.8 (8.8–45.7)
Israel	35 to 39	23.0 (8.4–45.1)	22.9 (8.0–45.0)	22.8 (8.3–44.2)	22.9 (8.7–46.3)	22.9 (8.8–45.9)
Israel	40 to 44	23.6 (8.7–46.0)	23.5 (8.3–45.8)	23.4 (8.5–45.0)	23.5 (9.0–47.2)	23.5 (9.0–46.8)

Supplementary Table S10: Prevalence of female SVAC by age and location for 1990, 2000, 2010, 2020, and 2023						
**Location**	**Age Range**	**1990**	**2000**	**2010**	**2020**	**2023**
Israel	45 to 49	22.9 (8.4–45.0)	22.9 (8.0–44.9)	22.8 (8.2–44.1)	22.9 (8.7–46.2)	22.9 (8.8–45.8)
Israel	50 to 54	22.4 (8.1–44.2)	22.3 (7.7–44.0)	22.2 (8.0–43.2)	22.3 (8.4–45.4)	22.3 (8.5–44.9)
Israel	55 to 59	21.6 (7.8–43.0)	21.5 (7.4–42.8)	21.4 (7.6–42.0)	21.5 (8.0–44.2)	21.5 (8.1–43.7)
Israel	60 to 64	20.8 (7.4–41.8)	20.7 (7.1–41.7)	20.6 (7.3–40.8)	20.7 (7.7–43.0)	20.7 (7.7–42.6)
Israel	65 to 69	19.3 (6.8–39.5)	19.3 (6.5–39.3)	19.2 (6.7–38.5)	19.3 (7.0–40.6)	19.3 (7.1–40.2)
Israel	70 to 74	15.6 (5.3–33.2)	15.6 (5.0–33.1)	15.5 (5.2–32.3)	15.5 (5.4–34.3)	15.5 (5.5–33.9)
Israel	75 to 79	14.1 (4.7–30.5)	14.0 (4.4–30.4)	14.0 (4.6–29.6)	14.0 (4.8–31.5)	14.0 (4.9–31.0)
Israel	80 to 84	10.1 (3.2–22.8)	10.1 (3.0–22.7)	10.0 (3.1–22.1)	10.0 (3.3–23.6)	10.0 (3.3–23.3)
Israel	85 to 89	9.1 (2.8–20.8)	9.0 (2.7–20.6)	9.0 (2.8–20.1)	9.0 (2.9–21.5)	9.0 (3.0–21.2)
Israel	90 to 94	8.2 (2.5–18.9)	8.2 (2.4–18.8)	8.1 (2.5–18.3)	8.1 (2.6–19.6)	8.1 (2.7–19.3)
Israel	95 plus	8.1 (2.5–18.8)	8.1 (2.4–18.6)	8.0 (2.5–18.1)	8.0 (2.6–19.4)	8.0 (2.6–19.1)
Israel	Age-standardized	21.2 (7.7–42.3)	21.2 (7.3–42.2)	21.1 (7.5–41.4)	21.2 (7.9–43.5)	21.2 (8.0–43.0)
Israel	All age	21.1 (7.6–42.1)	21.0 (7.2–41.8)	20.8 (7.4–40.8)	20.7 (7.7–42.6)	20.6 (7.8–42.1)
Italy	20 to 24	14.4 (7.6–24.4)	14.7 (9.1–22.4)	16.1 (13.0–19.5)	18.5 (12.3–26.2)	18.9 (11.9–28.2)
Italy	25 to 29	15.6 (8.5–26.0)	16.1 (10.3–24.0)	18.1 (14.8–21.6)	21.0 (14.4–29.0)	21.5 (13.9–31.1)

Supplementary Table S10: Prevalence of female SVAC by age and location for 1990, 2000, 2010, 2020, and 2023						
**Location**	**Age Range**	**1990**	**2000**	**2010**	**2020**	**2023**
Italy	30 to 34	16.5 (9.1–27.1)	16.9 (10.9–24.9)	18.4 (15.2–22.0)	21.1 (14.5–29.3)	21.6 (13.9–31.4)
Italy	35 to 39	16.9 (9.5–27.7)	17.9 (11.8–26.2)	21.4 (17.9–25.1)	25.9 (18.6–34.5)	26.7 (18.0–36.9)
Italy	40 to 44	16.6 (9.3–27.3)	17.7 (11.6–25.9)	21.2 (17.7–24.9)	26.0 (18.6–34.6)	26.8 (18.0–37.1)
Italy	45 to 49	16.7 (9.3–27.4)	17.6 (11.5–25.8)	20.6 (17.2–24.3)	24.7 (17.5–33.3)	25.5 (17.0–35.6)
Italy	50 to 54	16.5 (9.2–27.2)	17.6 (11.6–25.7)	21.4 (17.9–25.2)	26.4 (18.9–35.0)	27.2 (18.4–37.5)
Italy	55 to 59	14.3 (7.7–24.4)	15.1 (9.4–22.9)	17.6 (14.4–21.2)	21.5 (14.7–29.5)	22.2 (14.3–31.9)
Italy	60 to 64	14.2 (7.5–24.2)	14.9 (9.2–22.6)	17.4 (14.2–21.0)	21.3 (14.5–29.2)	21.9 (14.1–31.6)
Italy	65 to 69	12.9 (6.4–22.5)	13.3 (7.8–20.9)	15.0 (11.9–18.7)	18.0 (11.7–25.4)	18.6 (11.3–27.5)
Italy	70 to 74	12.1 (4.1–25.3)	11.6 (4.9–22.0)	10.9 (6.4–16.8)	11.4 (5.5–19.7)	11.5 (5.3–21.0)
Italy	75 to 79	13.4 (3.3–32.3)	13.2 (3.8–29.3)	13.2 (4.3–28.2)	13.6 (4.2–32.5)	13.8 (4.1–32.8)
Italy	80 to 84	9.7 (2.3–24.4)	9.5 (2.6–22.0)	9.5 (3.0–21.1)	9.8 (2.9–24.6)	9.9 (2.8–24.9)
Italy	85 to 89	8.8 (2.0–22.3)	8.6 (2.3–20.0)	8.5 (2.6–19.2)	8.8 (2.6–22.5)	8.9 (2.5–22.8)
Italy	90 to 94	7.9 (1.8–20.5)	7.8 (2.1–18.3)	7.7 (2.4–17.6)	8.0 (2.3–20.6)	8.1 (2.3–20.9)
Italy	95 plus	7.9 (1.8–20.3)	7.7 (2.1–18.2)	7.7 (2.3–17.4)	7.9 (2.3–20.5)	8.0 (2.3–20.7)
Italy	Age-standardized	15.3 (8.1–25.4)	15.9 (10.0–24.0)	18.1 (15.0–21.5)	21.5 (15.7–28.3)	22.1 (15.4–30.4)

Supplementary Table S10: Prevalence of female SVAC by age and location for 1990, 2000, 2010, 2020, and 2023						
**Location**	**Age Range**	**1990**	**2000**	**2010**	**2020**	**2023**
Italy	All age	14.9 (7.9–24.9)	15.3 (9.4–23.3)	17.3 (14.1–20.9)	20.0 (15.7–25.1)	20.4 (15.4–26.7)
Luxembourg	20 to 24	20.6 (7.7–40.2)	19.7 (9.6–33.7)	18.7 (13.7–24.7)	19.0 (10.5–29.6)	19.3 (9.4–32.6)
Luxembourg	25 to 29	21.8 (8.2–42.1)	21.5 (10.5–36.6)	21.0 (15.8–27.2)	21.1 (11.8–32.5)	21.1 (10.6–35.5)
Luxembourg	30 to 34	22.9 (8.7–43.7)	22.2 (10.8–37.5)	21.4 (16.0–27.6)	21.6 (12.1–33.1)	21.7 (10.9–36.3)
Luxembourg	35 to 39	24.5 (9.5–46.1)	25.1 (12.6–41.3)	25.3 (19.4–32.0)	24.9 (14.4–37.5)	24.8 (12.8–40.4)
Luxembourg	40 to 44	24.9 (9.7–46.6)	25.2 (12.7–41.5)	25.1 (19.2–31.8)	24.9 (14.4–37.4)	24.8 (12.8–40.5)
Luxembourg	45 to 49	24.2 (9.4–45.7)	24.4 (12.3–40.4)	24.2 (18.6–30.7)	24.1 (13.9–36.3)	24.0 (12.4–39.4)
Luxembourg	50 to 54	24.3 (9.4–45.8)	25.1 (12.6–41.3)	25.4 (19.6–32.1)	25.0 (14.5–37.5)	24.8 (12.8–40.3)
Luxembourg	55 to 59	22.1 (8.4–42.6)	21.6 (10.5–36.7)	20.9 (15.6–27.2)	21.1 (11.8–32.4)	21.2 (10.6–35.5)
Luxembourg	60 to 64	21.5 (8.1–41.7)	21.2 (10.3–36.0)	20.7 (15.4–27.0)	20.7 (11.5–31.9)	20.8 (10.3–34.9)
Luxembourg	65 to 69	19.5 (7.2–38.5)	18.7 (8.9–32.0)	17.8 (12.8–23.9)	18.1 (9.9–28.5)	18.3 (8.8–31.0)
Luxembourg	70 to 74	15.1 (5.3–31.3)	13.9 (6.0–25.0)	12.7 (8.5–18.0)	13.2 (6.9–22.3)	13.5 (6.1–25.1)
Luxembourg	75 to 79	14.7 (4.9–31.5)	14.7 (4.7–31.5)	14.7 (4.8–30.9)	14.8 (5.1–32.9)	14.8 (5.2–32.5)
Luxembourg	80 to 84	10.6 (3.4–23.8)	10.6 (3.2–23.7)	10.6 (3.3–23.3)	10.6 (3.5–24.9)	10.6 (3.6–24.6)
Luxembourg	85 to 89	9.5 (3.0–21.7)	9.5 (2.9–21.6)	9.5 (3.0–21.2)	9.6 (3.1–22.8)	9.6 (3.2–22.5)

Supplementary Table S10: Prevalence of female SVAC by age and location for 1990, 2000, 2010, 2020, and 2023						
**Location**	**Age Range**	**1990**	**2000**	**2010**	**2020**	**2023**
Luxembourg	90 to 94	8.7 (2.7–19.9)	8.6 (2.6–19.8)	8.6 (2.7–19.4)	8.7 (2.8–20.8)	8.7 (2.8–20.6)
Luxembourg	95 plus	8.6 (2.7–19.7)	8.5 (2.5–19.6)	8.6 (2.6–19.2)	8.6 (2.8–20.7)	8.6 (2.8–20.4)
Luxembourg	Age-standardized	21.9 (8.4–41.8)	21.7 (10.6–36.4)	21.3 (16.1–27.4)	21.3 (13.2–31.6)	21.3 (11.8–34.5)
Luxembourg	All age	21.3 (8.3–40.7)	21.2 (10.4–35.5)	20.9 (15.7–27.1)	20.8 (13.7–30.4)	20.9 (12.3–33.1)
Malta	20 to 24	14.5 (8.4–22.3)	14.4 (9.3–20.7)	14.2 (11.5–17.2)	14.4 (9.9–19.7)	14.5 (9.5–20.6)
Malta	25 to 29	16.2 (9.6–24.7)	16.1 (10.6–22.8)	16.1 (13.2–19.2)	16.3 (11.4–22.1)	16.4 (11.0–23.1)
Malta	30 to 34	16.7 (9.8–25.5)	16.6 (10.8–23.4)	16.4 (13.5–19.6)	16.6 (11.5–22.3)	16.6 (11.1–23.1)
Malta	35 to 39	19.4 (11.7–29.3)	19.4 (12.9–27.1)	19.3 (16.1–23.0)	19.7 (13.9–26.2)	19.7 (13.4–27.3)
Malta	40 to 44	19.1 (11.5–28.8)	19.1 (12.7–26.7)	19.1 (15.9–22.8)	19.6 (14.0–26.1)	19.7 (13.4–27.2)
Malta	45 to 49	18.6 (11.1–28.2)	18.6 (12.3–26.1)	18.5 (15.4–22.0)	18.8 (13.3–25.4)	18.9 (12.9–26.3)
Malta	50 to 54	19.3 (11.7–29.1)	19.3 (12.9–27.0)	19.3 (16.0–23.0)	19.8 (14.0–26.1)	19.9 (13.5–27.2)
Malta	55 to 59	15.9 (9.3–24.3)	15.8 (10.3–22.4)	15.8 (13.0–18.9)	16.1 (11.2–21.7)	16.2 (10.8–22.6)
Malta	60 to 64	16.5 (8.9–27.1)	16.4 (9.7–24.8)	16.1 (12.3–20.5)	16.1 (11.2–21.8)	16.2 (10.6–22.8)
Malta	65 to 69	14.2 (7.4–23.6)	14.1 (8.1–21.5)	13.8 (10.3–18.0)	13.8 (9.4–18.9)	13.8 (8.9–19.8)
Malta	70 to 74	10.1 (5.0–17.7)	10.0 (5.5–16.2)	9.8 (6.8–13.7)	9.7 (6.2–14.1)	9.8 (5.9–14.7)

Supplementary Table S10: Prevalence of female SVAC by age and location for 1990, 2000, 2010, 2020, and 2023						
**Location**	**Age Range**	**1990**	**2000**	**2010**	**2020**	**2023**
Malta	75 to 79	12.3 (3.6–29.1)	12.2 (3.8–28.3)	12.1 (3.9–26.2)	12.3 (3.6–29.0)	12.4 (3.6–30.0)
Malta	80 to 84	8.8 (2.5–21.8)	8.8 (2.6–21.1)	8.6 (2.7–19.4)	8.8 (2.5–21.7)	8.9 (2.5–22.5)
Malta	85 to 89	8.0 (2.2–19.9)	7.9 (2.4–19.2)	7.8 (2.4–17.7)	7.9 (2.2–19.8)	8.0 (2.2–20.5)
Malta	90 to 94	7.2 (2.0–18.2)	7.1 (2.1–17.6)	7.0 (2.1–16.1)	7.2 (2.0–18.1)	7.2 (2.0–18.8)
Malta	95 plus	7.1 (1.9–18.0)	7.1 (2.1–17.4)	7.0 (2.1–16.0)	7.1 (2.0–17.9)	7.2 (2.0–18.6)
Malta	Age-standardized	16.5 (9.6–25.4)	16.4 (10.5–23.6)	16.3 (13.2–19.8)	16.5 (12.2–21.9)	16.6 (11.9–22.6)
Malta	All age	16.5 (9.6–25.4)	16.3 (10.3–23.3)	15.8 (12.6–19.5)	15.9 (12.1–20.6)	16.1 (12.0–21.2)
Monaco	20 to 24	20.2 (7.2–40.9)	20.1 (6.8–40.7)	20.0 (7.0–39.8)	20.1 (7.4–41.9)	20.1 (7.5–41.5)
Monaco	25 to 29	20.6 (7.3–41.5)	20.5 (7.0–41.3)	20.4 (7.2–40.5)	20.5 (7.6–42.6)	20.5 (7.7–42.2)
Monaco	30 to 34	22.1 (8.0–43.8)	22.0 (7.6–43.6)	21.9 (7.8–42.7)	22.0 (8.2–44.9)	22.0 (8.3–44.4)
Monaco	35 to 39	22.3 (8.1–44.0)	22.2 (7.7–43.8)	22.1 (7.9–43.0)	22.2 (8.3–45.2)	22.2 (8.4–44.7)
Monaco	40 to 44	22.9 (8.3–44.9)	22.8 (7.9–44.7)	22.7 (8.2–43.9)	22.8 (8.6–46.1)	22.8 (8.8–45.7)
Monaco	45 to 49	22.3 (8.1–44.0)	22.2 (7.7–43.9)	22.1 (7.9–43.1)	22.2 (8.3–45.3)	22.2 (8.5–44.8)
Monaco	50 to 54	21.7 (7.8–43.2)	21.6 (7.5–43.0)	21.6 (7.7–42.3)	21.7 (8.1–44.4)	21.7 (8.2–44.0)
Monaco	55 to 59	20.9 (7.5–42.0)	20.9 (7.1–41.8)	20.8 (7.3–41.1)	20.9 (7.7–43.2)	20.9 (7.9–42.8)

Supplementary Table S10: Prevalence of female SVAC by age and location for 1990, 2000, 2010, 2020, and 2023						
**Location**	**Age Range**	**1990**	**2000**	**2010**	**2020**	**2023**
Monaco	60 to 64	20.2 (7.2–40.8)	20.1 (6.8–40.7)	20.1 (7.0–39.9)	20.1 (7.4–42.0)	20.1 (7.5–41.6)
Monaco	65 to 69	18.8 (6.5–38.5)	18.7 (6.2–38.4)	18.6 (6.4–37.6)	18.7 (6.8–39.7)	18.7 (6.8–39.3)
Monaco	70 to 74	15.1 (5.1–32.3)	15.1 (4.8–32.2)	15.0 (5.0–31.5)	15.1 (5.2–33.4)	15.1 (5.3–32.8)
Monaco	75 to 79	13.6 (4.5–29.6)	13.6 (4.3–29.5)	13.5 (4.4–28.8)	13.6 (4.6–30.6)	13.6 (4.7–30.2)
Monaco	80 to 84	9.7 (3.1–22.1)	9.7 (2.9–22.0)	9.6 (3.0–21.4)	9.7 (3.2–22.9)	9.7 (3.2–22.6)
Monaco	85 to 89	8.7 (2.7–20.1)	8.7 (2.6–19.9)	8.6 (2.7–19.4)	8.7 (2.8–20.8)	8.7 (2.8–20.5)
Monaco	90 to 94	7.9 (2.4–18.3)	7.8 (2.3–18.2)	7.8 (2.4–17.7)	7.8 (2.5–19.0)	7.8 (2.6–18.7)
Monaco	95 plus	7.8 (2.4–18.1)	7.8 (2.3–18.0)	7.7 (2.4–17.5)	7.7 (2.5–18.8)	7.7 (2.5–18.5)
Monaco	Age-standardized	20.5 (7.3–41.2)	20.4 (7.0–41.0)	20.4 (7.2–40.2)	20.5 (7.6–42.4)	20.4 (7.7–41.9)
Monaco	All age	19.2 (6.8–38.9)	19.0 (6.4–38.5)	19.0 (6.6–37.8)	18.9 (6.9–39.7)	18.8 (7.0–39.1)
Netherlands	20 to 24	25.3 (16.1–38.6)	25.1 (17.6–35.3)	24.8 (20.4–29.5)	25.5 (18.5–33.1)	25.7 (17.9–35.1)
Netherlands	25 to 29	28.2 (19.2–41.4)	28.1 (20.6–38.5)	28.1 (23.5–32.9)	28.8 (21.9–36.5)	29.0 (21.2–38.5)
Netherlands	30 to 34	29.5 (20.2–43.1)	29.3 (21.4–40.2)	28.7 (24.1–33.6)	28.9 (22.0–36.5)	28.9 (20.9–38.2)
Netherlands	35 to 39	32.3 (23.3–44.0)	32.5 (24.8–42.3)	33.4 (28.4–38.7)	35.3 (27.4–43.5)	35.6 (26.7–45.3)
Netherlands	40 to 44	31.6 (22.8–43.0)	31.9 (24.3–41.8)	33.0 (28.0–38.1)	35.2 (27.3–43.1)	35.5 (26.7–44.6)

Supplementary Table S10: Prevalence of female SVAC by age and location for 1990, 2000, 2010, 2020, and 2023						
**Location**	**Age Range**	**1990**	**2000**	**2010**	**2020**	**2023**
Netherlands	45 to 49	31.1 (22.1–43.5)	31.3 (23.5–41.5)	32.1 (27.3–37.2)	34.1 (26.4–41.9)	34.3 (25.7–43.9)
Netherlands	50 to 54	31.8 (22.6–43.4)	32.2 (24.2–42.1)	33.5 (28.5–38.7)	36.0 (28.0–44.1)	36.4 (27.4–45.9)
Netherlands	55 to 59	26.3 (17.9–38.5)	26.5 (19.3–36.6)	27.3 (22.8–32.1)	29.6 (22.2–37.5)	30.0 (21.4–39.8)
Netherlands	60 to 64	25.9 (17.3–37.7)	26.2 (18.8–35.7)	27.1 (22.6–31.8)	29.5 (22.1–37.4)	29.9 (21.5–39.6)
Netherlands	65 to 69	22.9 (13.9–36.0)	22.9 (15.4–32.9)	23.3 (18.8–28.0)	25.0 (18.0–32.7)	25.4 (17.2–35.0)
Netherlands	70 to 74	17.5 (7.3–34.6)	17.2 (8.2–31.2)	16.6 (11.2–23.7)	17.3 (11.5–24.2)	17.6 (10.9–26.2)
Netherlands	75 to 79	19.0 (6.4–40.0)	18.7 (7.1–36.7)	17.9 (10.0–29.0)	17.6 (11.7–25.1)	17.7 (11.1–26.1)
Netherlands	80 to 84	14.7 (3.9–35.1)	14.6 (4.3–33.1)	14.4 (4.7–30.4)	14.7 (4.7–34.0)	14.8 (4.5–36.3)
Netherlands	85 to 89	13.4 (3.4–32.6)	13.2 (3.7–30.5)	13.1 (4.2–28.0)	13.3 (4.3–31.4)	13.5 (4.2–32.7)
Netherlands	90 to 94	12.2 (3.0–29.6)	12.0 (3.4–28.2)	11.9 (3.8–25.8)	12.1 (3.8–29.0)	12.3 (3.6–29.9)
Netherlands	95 plus	12.1 (3.0–29.4)	11.9 (3.3–28.4)	11.8 (3.8–25.6)	12.0 (3.8–28.8)	12.1 (3.6–30.3)
Netherlands	Age-standardized	27.7 (19.1–39.4)	27.8 (20.3–37.5)	28.1 (23.3–33.0)	29.5 (23.3–36.2)	29.7 (22.7–37.7)
Netherlands	All age	27.0 (18.5–38.8)	27.2 (19.7–37.0)	27.3 (22.4–32.2)	28.0 (23.2–33.6)	28.2 (22.8–34.6)
Norway	20 to 24	23.9 (9.2–45.4)	23.4 (11.0–40.0)	22.6 (15.9–30.6)	22.6 (13.4–34.5)	22.8 (12.0–37.3)
Norway	25 to 29	25.2 (9.8–47.2)	25.3 (12.1–42.7)	25.2 (18.2–33.4)	25.0 (15.2–37.1)	25.0 (13.3–39.9)

Supplementary Table S10: Prevalence of female SVAC by age and location for 1990, 2000, 2010, 2020, and 2023						
**Location**	**Age Range**	**1990**	**2000**	**2010**	**2020**	**2023**
Norway	30 to 34	26.5 (10.4–49.0)	26.2 (12.6–43.7)	25.6 (18.5–33.8)	25.6 (15.6–37.8)	25.6 (13.8–40.8)
Norway	35 to 39	28.2 (11.4–51.4)	29.3 (14.4–47.7)	30.1 (22.4–38.6)	29.6 (18.5–42.3)	29.3 (16.2–45.2)
Norway	40 to 44	28.7 (11.6–52.0)	29.6 (14.6–48.0)	30.1 (22.4–38.6)	29.7 (18.6–42.4)	29.4 (16.3–45.4)
Norway	45 to 49	28.0 (11.2–51.0)	28.6 (14.0–46.9)	29.0 (21.4–37.5)	28.6 (17.8–41.2)	28.4 (15.6–44.1)
Norway	50 to 54	28.0 (11.2–51.1)	29.3 (14.4–47.7)	30.2 (22.5–38.8)	29.6 (18.6–42.4)	29.3 (16.2–45.2)
Norway	55 to 59	25.8 (10.1–48.1)	25.8 (12.3–43.2)	25.4 (18.4–33.6)	25.3 (15.4–37.5)	25.3 (13.6–40.4)
Norway	60 to 64	25.2 (9.8–47.2)	25.3 (12.1–42.6)	25.1 (18.1–33.3)	25.0 (15.2–37.1)	24.9 (13.3–39.9)
Norway	65 to 69	22.9 (8.7–43.9)	22.4 (10.4–38.6)	21.7 (15.1–29.6)	21.7 (12.7–33.3)	21.9 (11.4–36.1)
Norway	70 to 74	17.9 (6.5–35.8)	16.8 (7.4–31.1)	15.6 (9.8–22.9)	16.1 (8.5–26.4)	16.5 (7.8–29.2)
Norway	75 to 79	17.1 (5.9–35.8)	17.1 (5.6–35.7)	17.2 (5.8–35.2)	17.3 (6.2–37.3)	17.3 (6.2–36.9)
Norway	80 to 84	12.5 (4.0–27.4)	12.4 (3.9–27.4)	12.5 (4.0–26.9)	12.6 (4.3–28.7)	12.6 (4.3–28.4)
Norway	85 to 89	11.3 (3.6–25.1)	11.3 (3.4–25.1)	11.3 (3.6–24.7)	11.4 (3.8–26.4)	11.4 (3.8–26.1)
Norway	90 to 94	10.2 (3.2–23.1)	10.2 (3.1–23.1)	10.3 (3.2–22.7)	10.3 (3.4–24.3)	10.3 (3.5–24.0)
Norway	95 plus	10.1 (3.2–22.9)	10.1 (3.1–22.9)	10.2 (3.2–22.5)	10.2 (3.4–24.1)	10.2 (3.4–23.8)
Norway	Age-standardized	25.3 (10.1–46.9)	25.6 (12.2–42.7)	25.5 (18.5–33.5)	25.3 (17.0–35.8)	25.3 (15.5–38.5)

Supplementary Table S10: Prevalence of female SVAC by age and location for 1990, 2000, 2010, 2020, and 2023						
**Location**	**Age Range**	**1990**	**2000**	**2010**	**2020**	**2023**
Norway	All age	24.3 (9.6–44.9)	24.6 (11.8–41.5)	24.7 (17.7–32.7)	24.3 (17.7–33.2)	24.2 (16.8–35.4)
Portugal	20 to 24	10.8 (3.7–23.4)	9.3 (4.2–17.2)	7.9 (6.2– 9.8)	8.4 (4.5–13.3)	8.8 (4.2–15.7)
Portugal	25 to 29	11.5 (4.0–24.7)	10.2 (4.6–18.8)	9.0 (7.1–11.1)	9.4 (5.1–14.7)	9.7 (4.6–17.2)
Portugal	30 to 34	12.1 (4.2–25.9)	10.5 (4.8–19.4)	9.1 (7.2–11.2)	9.6 (5.2–15.1)	10.0 (4.8–17.7)
Portugal	35 to 39	13.0 (4.5–27.6)	11.9 (5.5–21.7)	10.8 (8.6–13.1)	11.1 (6.1–17.4)	11.4 (5.5–19.9)
Portugal	40 to 44	13.2 (4.6–27.9)	12.0 (5.5–21.8)	10.7 (8.6–13.1)	11.1 (6.1–17.3)	11.5 (5.5–20.0)
Portugal	45 to 49	12.8 (4.5–27.2)	11.6 (5.4–21.2)	10.4 (8.3–12.7)	10.8 (5.9–16.8)	11.1 (5.4–19.4)
Portugal	50 to 54	12.8 (4.5–27.3)	11.9 (5.5–21.6)	10.9 (8.7–13.2)	11.2 (6.1–17.4)	11.4 (5.5–19.9)
Portugal	55 to 59	11.6 (4.0–25.0)	10.2 (4.6–18.8)	8.9 (7.0–11.0)	9.4 (5.1–14.7)	9.7 (4.6–17.1)
Portugal	60 to 64	11.3 (3.9–24.4)	10.0 (4.6–18.6)	8.8 (7.0–10.9)	9.3 (5.0–14.5)	9.6 (4.6–17.0)
Portugal	65 to 69	10.1 (3.5–22.2)	8.8 (4.0–16.4)	7.6 (5.9– 9.5)	8.0 (4.3–12.8)	8.4 (3.9–15.1)
Portugal	70 to 74	7.7 (2.5–17.3)	6.4 (2.8–12.2)	5.3 (3.9– 7.2)	5.8 (3.0– 9.7)	6.1 (2.7–11.5)
Portugal	75 to 79	8.2 (2.5–19.0)	8.2 (2.4–18.8)	8.1 (2.5–18.3)	8.1 (2.6–19.6)	8.1 (2.7–19.4)
Portugal	80 to 84	5.8 (1.7–13.7)	5.7 (1.7–13.6)	5.7 (1.7–13.2)	5.7 (1.8–14.2)	5.7 (1.8–14.0)
Portugal	85 to 89	5.2 (1.6–12.4)	5.2 (1.5–12.3)	5.1 (1.5–11.9)	5.1 (1.6–12.9)	5.1 (1.6–12.7)

Supplementary Table S10: Prevalence of female SVAC by age and location for 1990, 2000, 2010, 2020, and 2023						
**Location**	**Age Range**	**1990**	**2000**	**2010**	**2020**	**2023**
Portugal	90 to 94	4.7 (1.4–11.3)	4.7 (1.3–11.2)	4.6 (1.4–10.8)	4.6 (1.4–11.7)	4.6 (1.5–11.5)
Portugal	95 plus	4.6 (1.4–11.2)	4.6 (1.3–11.1)	4.6 (1.3–10.7)	4.6 (1.4–11.6)	4.6 (1.4–11.4)
Portugal	Age-standardized	11.5 (4.1–24.6)	10.3 (4.7–19.2)	9.1 (7.1–11.4)	9.5 (5.9–14.5)	9.9 (5.3–16.9)
Portugal	All age	11.3 (4.0–24.0)	10.0 (4.5–18.8)	8.9 (6.8–11.4)	9.1 (6.5–13.2)	9.3 (5.9–15.2)
San Marino	20 to 24	20.2 (7.2–40.9)	20.1 (6.8–40.7)	20.0 (7.0–39.8)	20.1 (7.4–41.9)	20.1 (7.5–41.5)
San Marino	25 to 29	20.6 (7.3–41.5)	20.5 (7.0–41.3)	20.4 (7.2–40.5)	20.5 (7.6–42.6)	20.5 (7.7–42.2)
San Marino	30 to 34	22.1 (8.0–43.8)	22.0 (7.6–43.6)	21.9 (7.8–42.7)	22.0 (8.2–44.9)	22.0 (8.3–44.4)
San Marino	35 to 39	22.3 (8.1–44.0)	22.2 (7.7–43.8)	22.1 (7.9–43.0)	22.2 (8.3–45.2)	22.2 (8.4–44.7)
San Marino	40 to 44	22.9 (8.3–44.9)	22.8 (7.9–44.7)	22.7 (8.2–43.9)	22.8 (8.6–46.1)	22.8 (8.8–45.7)
San Marino	45 to 49	22.3 (8.1–44.0)	22.2 (7.7–43.9)	22.1 (7.9–43.1)	22.2 (8.3–45.3)	22.2 (8.5–44.8)
San Marino	50 to 54	21.7 (7.8–43.2)	21.6 (7.5–43.0)	21.6 (7.7–42.3)	21.7 (8.1–44.4)	21.7 (8.2–44.0)
San Marino	55 to 59	20.9 (7.5–42.0)	20.9 (7.1–41.8)	20.8 (7.3–41.1)	20.9 (7.7–43.2)	20.9 (7.9–42.8)
San Marino	60 to 64	20.2 (7.2–40.8)	20.1 (6.8–40.7)	20.1 (7.0–39.9)	20.1 (7.4–42.0)	20.1 (7.5–41.6)
San Marino	65 to 69	18.8 (6.5–38.5)	18.7 (6.2–38.4)	18.6 (6.4–37.6)	18.7 (6.8–39.7)	18.7 (6.8–39.3)
San Marino	70 to 74	15.1 (5.1–32.3)	15.1 (4.8–32.2)	15.0 (5.0–31.5)	15.1 (5.2–33.4)	15.1 (5.3–32.8)

Supplementary Table S10: Prevalence of female SVAC by age and location for 1990, 2000, 2010, 2020, and 2023						
**Location**	**Age Range**	**1990**	**2000**	**2010**	**2020**	**2023**
San Marino	75 to 79	13.6 (4.5–29.6)	13.6 (4.3–29.5)	13.5 (4.4–28.8)	13.6 (4.6–30.6)	13.6 (4.7–30.2)
San Marino	80 to 84	9.7 (3.1–22.1)	9.7 (2.9–22.0)	9.6 (3.0–21.4)	9.7 (3.2–22.9)	9.7 (3.2–22.6)
San Marino	85 to 89	8.7 (2.7–20.1)	8.7 (2.6–19.9)	8.6 (2.7–19.4)	8.7 (2.8–20.8)	8.7 (2.8–20.5)
San Marino	90 to 94	7.9 (2.4–18.3)	7.8 (2.3–18.2)	7.8 (2.4–17.7)	7.8 (2.5–19.0)	7.8 (2.6–18.7)
San Marino	95 plus	7.8 (2.4–18.1)	7.8 (2.3–18.0)	7.7 (2.4–17.5)	7.7 (2.5–18.8)	7.7 (2.5–18.5)
San Marino	Age-standardized	20.5 (7.3–41.2)	20.4 (7.0–41.0)	20.4 (7.2–40.2)	20.5 (7.6–42.4)	20.4 (7.7–41.9)
San Marino	All age	20.0 (7.1–40.3)	19.8 (6.7–39.9)	19.7 (6.9–39.0)	19.5 (7.2–40.7)	19.3 (7.2–40.0)
Spain	20 to 24	25.5 (18.6–34.0)	21.4 (13.7–31.5)	14.5 (10.4–19.3)	10.2 (6.7–14.6)	9.7 (5.8–15.0)
Spain	25 to 29	22.2 (16.5–29.3)	20.6 (13.2–30.2)	15.9 (11.8–20.7)	11.4 (7.7–16.0)	10.7 (6.6–16.2)
Spain	30 to 34	25.9 (19.2–34.2)	22.9 (14.8–33.2)	16.5 (12.2–21.3)	11.4 (7.8–16.0)	10.8 (6.7–16.3)
Spain	35 to 39	22.5 (16.6–29.4)	22.6 (14.8–32.4)	19.0 (14.4–24.0)	13.4 (9.5–18.1)	12.6 (8.2–18.1)
Spain	40 to 44	21.5 (15.8–28.4)	21.8 (14.2–31.5)	18.7 (14.2–23.8)	13.5 (9.6–18.2)	12.7 (8.2–18.4)
Spain	45 to 49	25.5 (19.0–33.3)	24.1 (15.9–34.3)	18.7 (14.2–23.7)	13.0 (9.1–17.6)	12.2 (7.8–17.7)
Spain	50 to 54	22.4 (16.5–29.2)	22.6 (14.8–32.4)	19.2 (14.6–24.2)	13.6 (9.7–18.3)	12.8 (8.3–18.4)
Spain	55 to 59	22.0 (15.7–29.7)	20.4 (13.1–29.9)	15.8 (11.7–20.5)	11.5 (7.8–16.1)	10.9 (6.8–16.5)

Supplementary Table S10: Prevalence of female SVAC by age and location for 1990, 2000, 2010, 2020, and 2023						
**Location**	**Age Range**	**1990**	**2000**	**2010**	**2020**	**2023**
Spain	60 to 64	21.5 (15.1–28.8)	20.2 (12.6–28.6)	15.8 (11.5–20.7)	11.4 (7.6–16.1)	10.8 (6.5–16.6)
Spain	65 to 69	18.0 (11.9–26.0)	16.8 (10.2–25.6)	13.3 (9.4–17.8)	10.0 (6.4–14.5)	9.5 (5.6–15.0)
Spain	70 to 74	13.9 (8.7–21.2)	12.3 (7.0–19.7)	9.4 (6.1–13.8)	7.5 (4.4–11.7)	7.3 (3.9–12.6)
Spain	75 to 79	12.4 (7.2–19.2)	10.6 (5.6–17.9)	8.1 (4.4–13.5)	6.8 (3.8–11.4)	6.7 (3.4–12.0)
Spain	80 to 84	7.4 (3.5–13.7)	6.6 (2.6–13.3)	5.7 (2.0–12.2)	5.2 (1.6–11.9)	5.1 (1.6–12.4)
Spain	85 to 89	7.0 (3.3–13.0)	6.2 (2.4–12.5)	5.2 (1.8–11.2)	4.7 (1.5–10.8)	4.6 (1.4–11.2)
Spain	90 to 94	6.6 (3.1–12.4)	5.8 (2.3–11.8)	4.8 (1.6–10.4)	4.2 (1.3– 9.9)	4.2 (1.3–10.3)
Spain	95 plus	6.6 (3.1–12.3)	5.8 (2.3–11.7)	4.7 (1.6–10.3)	4.2 (1.3– 9.7)	4.1 (1.3–10.1)
Spain	Age-standardized	22.0 (16.2–28.8)	20.5 (15.5–27.6)	16.0 (12.8–19.7)	11.5 (8.3–15.4)	10.8 (7.3–15.5)
Spain	All age	21.2 (15.5–27.8)	19.4 (15.6–25.1)	15.3 (12.6–18.3)	10.9 (8.3–14.3)	10.3 (7.4–14.2)
Sweden	20 to 24	19.7 (7.1–39.9)	19.4 (8.8–34.2)	19.1 (12.8–26.6)	19.6 (11.3–29.6)	19.7 (10.7–31.1)
Sweden	25 to 29	17.1 (10.5–26.5)	18.3 (12.6–25.5)	20.6 (14.8–27.2)	22.0 (13.9–32.4)	22.3 (13.4–33.9)
Sweden	30 to 34	19.1 (11.8–29.0)	19.8 (13.7–27.4)	21.3 (15.4–28.1)	22.3 (14.1–32.6)	22.5 (13.7–34.3)
Sweden	35 to 39	17.6 (10.9–27.1)	20.0 (14.0–27.4)	24.4 (18.0–31.5)	26.8 (17.7–37.7)	27.2 (17.1–39.5)
Sweden	40 to 44	17.0 (10.4–26.4)	19.3 (13.4–26.7)	23.9 (17.7–31.0)	26.7 (17.6–37.7)	27.1 (17.0–39.6)

Supplementary Table S10: Prevalence of female SVAC by age and location for 1990, 2000, 2010, 2020, and 2023						
**Location**	**Age Range**	**1990**	**2000**	**2010**	**2020**	**2023**
Sweden	45 to 49	18.7 (11.6–28.4)	20.6 (14.4–27.9)	23.8 (17.5–30.9)	25.5 (16.9–36.2)	25.8 (16.2–38.1)
Sweden	50 to 54	17.1 (10.5–26.5)	19.7 (13.7–27.1)	24.5 (18.1–31.6)	27.1 (18.0–38.0)	27.5 (17.6–39.9)
Sweden	55 to 59	16.1 (9.5–25.8)	17.4 (11.6–24.7)	20.2 (14.4–27.0)	22.0 (14.0–32.5)	22.4 (13.6–34.2)
Sweden	60 to 64	15.9 (9.3–25.5)	17.3 (11.5–24.5)	20.0 (14.3–26.7)	21.8 (13.8–32.3)	22.1 (13.4–34.0)
Sweden	65 to 69	14.2 (7.7–23.9)	15.1 (9.3–22.3)	17.3 (11.6–23.9)	19.0 (10.7–29.4)	19.3 (10.4–30.6)
Sweden	70 to 74	11.6 (5.4–20.4)	11.6 (6.6–18.6)	12.5 (7.6–18.5)	13.5 (6.9–22.5)	13.7 (6.6–23.5)
Sweden	75 to 79	10.8 (5.1–19.5)	10.9 (5.2–19.5)	12.0 (4.6–23.8)	13.0 (3.9–28.5)	13.2 (3.8–29.2)
Sweden	80 to 84	9.5 (2.8–22.6)	9.5 (2.9–21.6)	9.6 (3.0–21.4)	10.0 (2.8–24.8)	10.1 (2.8–25.1)
Sweden	85 to 89	8.6 (2.5–20.7)	8.6 (2.5–19.7)	8.7 (2.7–19.5)	9.0 (2.5–23.1)	9.1 (2.5–23.3)
Sweden	90 to 94	7.8 (2.3–18.7)	7.8 (2.3–18.0)	7.9 (2.4–17.9)	8.2 (2.3–21.0)	8.3 (2.2–21.1)
Sweden	95 plus	7.7 (2.2–18.6)	7.7 (2.3–17.9)	7.8 (2.4–17.7)	8.1 (2.2–21.1)	8.2 (2.2–21.2)
Sweden	Age-standardized	16.9 (9.8–27.0)	18.2 (12.0–26.0)	20.7 (15.8–26.4)	22.3 (16.8–29.3)	22.6 (16.5–30.4)
Sweden	All age	16.0 (9.4–25.5)	17.2 (11.2–24.9)	19.5 (15.0–24.7)	20.9 (16.1–26.8)	21.2 (15.8–27.9)
Switzerland	20 to 24	18.6 (8.2–34.0)	17.9 (10.7–27.2)	18.0 (9.7–28.8)	18.6 (7.6–37.0)	18.8 (7.0–39.1)
Switzerland	25 to 29	20.5 (9.4–36.3)	20.2 (12.8–29.2)	19.6 (11.8–29.1)	19.5 (8.4–37.2)	19.5 (7.5–39.2)

Supplementary Table S10: Prevalence of female SVAC by age and location for 1990, 2000, 2010, 2020, and 2023						
**Location**	**Age Range**	**1990**	**2000**	**2010**	**2020**	**2023**
Switzerland	30 to 34	22.0 (10.2–39.1)	21.5 (13.7–30.6)	20.5 (12.8–29.7)	20.1 (9.6–36.2)	20.1 (8.6–38.0)
Switzerland	35 to 39	22.7 (10.7–40.3)	22.6 (14.7–31.8)	21.4 (13.3–31.0)	20.2 (10.5–35.0)	20.0 (9.5–36.6)
Switzerland	40 to 44	22.3 (10.5–39.9)	22.1 (14.3–31.6)	22.1 (12.6–33.2)	22.2 (9.3–42.0)	22.2 (8.6–44.2)
Switzerland	45 to 49	21.1 (11.5–35.2)	21.4 (14.3–30.3)	21.7 (12.3–33.7)	21.8 (9.1–41.5)	21.8 (8.5–43.4)
Switzerland	50 to 54	20.8 (11.4–34.0)	21.1 (14.2–29.6)	21.4 (12.0–33.4)	21.4 (9.0–41.0)	21.5 (8.3–42.8)
Switzerland	55 to 59	19.9 (10.2–33.9)	19.5 (12.9–27.9)	19.7 (10.8–31.8)	20.1 (8.2–38.5)	20.2 (7.7–41.0)
Switzerland	60 to 64	19.7 (9.3–34.7)	19.2 (12.6–27.8)	19.3 (10.3–30.5)	19.7 (8.0–38.4)	19.8 (7.5–39.6)
Switzerland	65 to 69	17.9 (7.7–32.8)	17.1 (9.9–26.5)	17.3 (9.1–28.7)	18.0 (7.7–35.7)	18.2 (7.1–37.8)
Switzerland	70 to 74	16.2 (5.9–33.6)	16.8 (6.9–31.7)	17.1 (7.9–30.2)	17.0 (6.9–33.3)	17.0 (6.4–34.3)
Switzerland	75 to 79	14.8 (5.1–31.4)	15.5 (5.8–30.9)	16.2 (7.5–29.0)	16.3 (7.2–31.5)	16.4 (6.5–32.9)
Switzerland	80 to 84	10.9 (3.5–24.5)	11.8 (4.0–25.5)	13.2 (5.5–25.5)	14.0 (6.4–26.6)	14.1 (5.8–27.6)
Switzerland	85 to 89	9.0 (2.8–20.6)	9.0 (2.7–20.5)	9.0 (2.8–20.2)	9.2 (2.9–22.3)	9.3 (2.9–22.4)
Switzerland	90 to 94	8.1 (2.5–18.8)	8.1 (2.4–18.7)	8.2 (2.5–18.5)	8.3 (2.6–20.5)	8.4 (2.6–20.5)
Switzerland	95 plus	8.1 (2.5–18.7)	8.0 (2.4–18.6)	8.1 (2.5–18.3)	8.3 (2.6–20.3)	8.3 (2.5–20.6)
Switzerland	Age-standardized	20.1 (10.1–34.4)	19.9 (12.9–28.6)	19.7 (13.2–27.6)	19.7 (11.1–33.5)	19.7 (10.4–35.2)

Supplementary Table S10: Prevalence of female SVAC by age and location for 1990, 2000, 2010, 2020, and 2023						
**Location**	**Age Range**	**1990**	**2000**	**2010**	**2020**	**2023**
Switzerland	All age	19.5 (9.7–33.4)	19.4 (12.4–28.2)	19.2 (14.0–25.9)	19.2 (12.1–30.6)	19.2 (11.4–31.8)
UK	20 to 24	24.4 (16.3–35.8)	24.9 (17.0–33.3)	23.2 (14.7–34.1)	22.3 (11.1–37.5)	22.3 (10.0–39.9)
UK	25 to 29	22.7 (14.1–35.4)	25.0 (16.6–35.1)	25.2 (16.5–35.2)	24.3 (12.5–40.4)	24.2 (11.7–42.5)
UK	30 to 34	23.6 (14.2–35.2)	25.3 (16.2–36.1)	25.9 (18.0–34.2)	25.4 (13.5–41.6)	25.3 (12.2–43.8)
UK	35 to 39	22.3 (12.8–34.3)	25.4 (15.9–36.6)	27.9 (19.9–37.0)	28.1 (16.2–42.4)	28.0 (14.4–44.7)
UK	40 to 44	21.8 (11.8–34.7)	24.8 (15.3–36.1)	27.7 (18.7–37.8)	28.3 (18.1–40.5)	28.2 (16.7–43.1)
UK	45 to 49	21.3 (11.6–34.2)	22.4 (13.1–34.0)	25.6 (16.9–35.6)	27.3 (14.6–43.2)	27.4 (12.8–45.7)
UK	50 to 54	19.4 (10.1–32.3)	21.3 (11.5–33.7)	25.6 (17.0–35.4)	27.9 (15.1–43.4)	28.0 (13.3–46.7)
UK	55 to 59	19.7 (10.2–33.0)	20.6 (11.1–32.8)	22.7 (14.1–32.8)	24.2 (13.2–38.5)	24.4 (11.3–40.8)
UK	60 to 64	18.4 (9.4–31.7)	19.1 (9.8–31.7)	21.8 (13.3–32.2)	23.9 (13.3–37.3)	24.2 (12.1–39.7)
UK	65 to 69	20.9 (7.7–40.9)	20.7 (8.8–37.4)	20.7 (11.3–33.0)	21.1 (10.0–36.5)	21.2 (9.0–38.4)
UK	70 to 74	16.1 (6.4–31.7)	15.5 (7.2–27.3)	15.4 (7.8–26.2)	16.0 (7.2–29.8)	16.3 (6.5–31.9)
UK	75 to 79	15.4 (5.2–32.8)	15.4 (4.9–32.8)	15.6 (5.2–32.5)	15.7 (5.5–34.6)	15.8 (5.5–35.4)
UK	80 to 84	11.1 (3.6–24.9)	11.2 (3.4–24.9)	11.3 (3.6–24.6)	11.4 (3.8–26.4)	11.5 (3.8–27.0)
UK	85 to 89	10.1 (3.2–22.8)	10.1 (3.0–22.8)	10.2 (3.2–22.5)	10.3 (3.4–24.2)	10.4 (3.4–24.8)

Supplementary Table S10: Prevalence of female SVAC by age and location for 1990, 2000, 2010, 2020, and 2023						
**Location**	**Age Range**	**1990**	**2000**	**2010**	**2020**	**2023**
UK	90 to 94	9.1 (2.9–20.9)	9.2 (2.7–20.9)	9.3 (2.9–20.7)	9.4 (3.1–22.3)	9.4 (3.1–22.8)
UK	95 plus	9.1 (2.8–20.7)	9.1 (2.7–20.7)	9.2 (2.9–20.5)	9.3 (3.0–22.1)	9.3 (3.0–22.6)
UK	Age-standardized	21.2 (13.1–31.9)	22.6 (15.2–31.8)	24.0 (19.1–29.0)	24.4 (16.3–34.3)	24.4 (14.8–36.4)
UK	All age	20.3 (12.1–31.6)	21.5 (13.9–31.6)	22.9 (17.7–29.0)	23.3 (14.9–33.6)	23.3 (13.8–35.4)
Latin America and Caribbean	20 to 24	16.3 (9.0–26.7)	16.7 (11.6–23.3)	17.2 (14.1–20.9)	17.5 (14.7–21.7)	17.6 (14.3–22.5)
Latin America and Caribbean	25 to 29	16.5 (9.0–26.8)	17.2 (11.6–24.5)	18.1 (14.8–22.1)	18.6 (16.0–22.3)	18.7 (15.5–23.1)
Latin America and Caribbean	30 to 34	17.5 (9.7–28.7)	18.2 (12.3–26.1)	19.1 (15.7–23.2)	19.5 (16.5–23.9)	19.6 (16.2–24.5)
Latin America and Caribbean	35 to 39	17.4 (9.5–28.6)	18.4 (12.3–26.5)	20.3 (16.8–24.5)	21.1 (17.8–25.7)	21.2 (17.4–26.2)
Latin America and Caribbean	40 to 44	17.6 (9.5–29.1)	17.9 (11.8–26.0)	18.6 (15.1–22.9)	19.3 (16.2–23.6)	19.4 (15.7–24.8)
Latin America and Caribbean	45 to 49	18.1 (10.0–29.8)	18.2 (12.2–26.5)	18.6 (15.1–22.9)	18.9 (15.9–22.8)	18.9 (15.4–23.9)
Latin America and Caribbean	50 to 54	18.0 (7.3–34.1)	17.8 (9.3–29.8)	17.9 (13.9–23.2)	18.1 (15.7–21.4)	18.2 (15.0–22.1)
Latin America and Caribbean	55 to 59	16.3 (6.4–31.3)	15.8 (7.8–27.6)	15.6 (11.7–21.1)	16.0 (13.5–19.5)	16.1 (13.0–20.1)
Latin America and Caribbean	60 to 64	15.7 (6.1–30.0)	15.3 (7.5–26.9)	15.0 (10.1–22.1)	15.5 (10.2–23.6)	15.6 (9.7–24.3)
Latin America and Caribbean	65 to 69	14.3 (5.4–27.9)	13.8 (6.3–25.2)	13.5 (8.7–20.6)	13.9 (9.1–21.8)	14.0 (8.6–22.3)
Latin America and Caribbean	70 to 74	11.8 (4.3–24.1)	11.4 (4.9–21.7)	11.3 (6.7–18.4)	11.6 (7.4–19.5)	11.7 (7.2–19.9)

Supplementary Table S10: Prevalence of female SVAC by age and location for 1990, 2000, 2010, 2020, and 2023						
**Location**	**Age Range**	**1990**	**2000**	**2010**	**2020**	**2023**
Latin America and Caribbean	75 to 79	10.7 (3.9–22.3)	10.6 (4.2–21.1)	10.5 (5.1–18.9)	10.8 (5.1–20.0)	10.8 (5.1–20.4)
Latin America and Caribbean	80 to 84	7.3 (2.2–16.4)	7.1 (2.3–16.1)	7.0 (2.5–15.1)	7.2 (2.6–16.3)	7.3 (2.6–16.6)
Latin America and Caribbean	85 to 89	6.5 (2.0–15.0)	6.4 (2.0–14.6)	6.3 (2.2–13.8)	6.5 (2.3–15.0)	6.6 (2.4–15.2)
Latin America and Caribbean	90 to 94	5.9 (1.8–13.5)	5.8 (1.8–13.3)	5.8 (2.0–12.7)	6.0 (2.1–13.8)	6.0 (2.1–14.1)
Latin America and Caribbean	95 plus	6.3 (1.8–14.4)	6.1 (1.9–14.3)	5.9 (2.0–13.0)	6.1 (2.0–14.5)	6.2 (2.1–14.8)
Latin America and Caribbean	Age-standardized	16.3 (8.1–28.0)	16.5 (10.2–25.2)	17.0 (13.8–21.5)	17.5 (15.3–20.3)	17.6 (14.9–20.9)
Latin America and Caribbean	All age	16.5 (8.5–27.9)	16.8 (10.7–25.3)	17.3 (14.1–21.5)	17.5 (15.3–20.3)	17.5 (14.9–20.8)
Andean Latin America	20 to 24	18.6 (11.8–28.0)	20.4 (15.3–26.6)	19.2 (14.9–24.6)	18.0 (13.8–24.8)	17.8 (12.3–26.1)
Andean Latin America	25 to 29	18.4 (10.9–28.8)	20.0 (14.6–26.8)	19.1 (15.3–23.9)	18.0 (14.2–24.6)	17.8 (13.0–25.8)
Andean Latin America	30 to 34	19.6 (10.8–31.4)	20.6 (14.4–28.5)	20.1 (15.4–25.3)	18.8 (13.2–27.8)	18.6 (12.2–28.9)
Andean Latin America	35 to 39	20.3 (10.7–33.2)	20.3 (14.3–28.0)	19.7 (15.6–24.5)	19.0 (14.8–25.1)	18.8 (13.9–26.2)
Andean Latin America	40 to 44	20.5 (10.6–33.8)	19.8 (13.4–28.0)	18.8 (15.3–23.1)	19.4 (14.2–26.2)	19.6 (13.4–28.1)
Andean Latin America	45 to 49	21.1 (11.0–34.6)	21.0 (14.4–29.6)	19.3 (15.4–23.9)	18.5 (13.4–25.5)	18.4 (12.1–27.1)
Andean Latin America	50 to 54	18.6 (8.2–33.4)	18.6 (9.8–30.1)	18.4 (11.2–28.0)	18.2 (10.4–29.2)	18.1 (9.5–30.3)
Andean Latin America	55 to 59	15.7 (6.4–30.5)	14.8 (6.7–26.9)	14.9 (7.6–25.7)	15.9 (8.3–28.1)	16.2 (7.9–29.2)

Supplementary Table S10: Prevalence of female SVAC by age and location for 1990, 2000, 2010, 2020, and 2023						
**Location**	**Age Range**	**1990**	**2000**	**2010**	**2020**	**2023**
Andean Latin America	60 to 64	17.1 (7.1–31.8)	17.1 (8.0–30.1)	17.3 (8.6–29.6)	17.7 (8.3–32.5)	17.7 (7.9–33.2)
Andean Latin America	65 to 69	15.6 (6.0–31.1)	15.6 (6.0–31.2)	15.6 (6.4–29.7)	16.0 (6.7–31.9)	16.1 (6.7–32.2)
Andean Latin America	70 to 74	13.0 (4.8–26.9)	13.0 (4.8–26.6)	13.1 (5.3–25.6)	13.3 (5.5–27.4)	13.3 (5.3–27.7)
Andean Latin America	75 to 79	11.9 (4.2–25.1)	11.9 (4.3–24.8)	12.1 (4.8–24.0)	12.2 (5.1–25.5)	12.3 (4.9–25.8)
Andean Latin America	80 to 84	8.8 (3.0–19.3)	9.0 (3.1–19.6)	9.1 (3.4–18.9)	9.2 (3.7–19.9)	9.2 (3.5–20.3)
Andean Latin America	85 to 89	8.2 (2.6–18.2)	8.2 (2.7–18.4)	8.3 (3.1–17.6)	8.5 (3.3–18.7)	8.5 (3.2–18.8)
Andean Latin America	90 to 94	7.6 (2.4–17.5)	7.6 (2.4–17.3)	7.7 (2.7–16.8)	7.8 (2.9–17.7)	7.8 (2.8–18.0)
Andean Latin America	95 plus	8.1 (2.5–18.6)	7.8 (2.3–18.0)	7.8 (2.5–17.5)	8.0 (2.7–18.9)	8.0 (2.7–19.3)
Andean Latin America	Age-standardized	18.1 (10.0–29.9)	18.6 (12.3–27.1)	17.9 (14.7–22.3)	17.5 (14.0–22.3)	17.5 (12.9–23.3)
Andean Latin America	All age	18.5 (10.7–29.5)	19.1 (13.1–27.2)	18.2 (15.1–22.1)	17.6 (14.1–22.2)	17.5 (12.9–23.3)
Bolivia	20 to 24	14.3 (4.7–30.8)	14.4 (4.6–31.0)	14.5 (4.8–30.6)	14.6 (5.1–32.6)	14.6 (5.1–32.2)
Bolivia	25 to 29	14.5 (4.8–31.2)	14.7 (4.7–31.4)	14.7 (4.9–31.0)	14.9 (5.2–33.0)	14.9 (5.2–32.7)
Bolivia	30 to 34	15.5 (5.2–33.1)	15.7 (5.0–33.3)	15.8 (5.3–32.8)	15.9 (5.6–34.9)	15.9 (5.7–34.4)
Bolivia	35 to 39	15.5 (5.2–33.1)	15.7 (5.0–33.3)	15.8 (5.3–32.9)	15.9 (5.6–35.0)	15.9 (5.7–34.4)
Bolivia	40 to 44	15.9 (5.4–33.7)	16.1 (5.2–33.9)	16.2 (5.4–33.5)	16.3 (5.8–35.7)	16.3 (5.8–35.1)

Supplementary Table S10: Prevalence of female SVAC by age and location for 1990, 2000, 2010, 2020, and 2023						
**Location**	**Age Range**	**1990**	**2000**	**2010**	**2020**	**2023**
Bolivia	45 to 49	15.4 (5.2–32.8)	15.5 (5.0–33.0)	15.6 (5.2–32.6)	15.8 (5.5–34.7)	15.8 (5.6–34.2)
Bolivia	50 to 54	14.9 (5.0–32.0)	15.1 (4.8–32.2)	15.2 (5.0–31.8)	15.4 (5.4–33.9)	15.3 (5.4–33.5)
Bolivia	55 to 59	14.4 (4.8–30.9)	14.5 (4.6–31.2)	14.6 (4.8–30.8)	14.8 (5.1–32.9)	14.8 (5.2–32.5)
Bolivia	60 to 64	13.9 (4.6–30.1)	14.0 (4.4–30.3)	14.1 (4.6–29.9)	14.3 (4.9–32.0)	14.3 (5.0–31.6)
Bolivia	65 to 69	12.9 (4.2–28.3)	13.0 (4.1–28.5)	13.2 (4.3–28.2)	13.3 (4.5–30.2)	13.3 (4.6–29.8)
Bolivia	70 to 74	10.4 (3.3–23.4)	10.5 (3.2–23.6)	10.6 (3.3–23.3)	10.7 (3.6–25.1)	10.7 (3.6–24.7)
Bolivia	75 to 79	9.4 (3.0–21.4)	9.5 (2.9–21.6)	9.6 (3.0–21.3)	9.7 (3.2–23.0)	9.7 (3.2–22.7)
Bolivia	80 to 84	6.7 (2.0–15.7)	6.8 (2.0–15.9)	6.8 (2.1–15.7)	6.9 (2.2–17.0)	6.9 (2.2–16.8)
Bolivia	85 to 89	6.1 (1.8–14.3)	6.1 (1.8–14.5)	6.2 (1.9–14.3)	6.3 (2.0–15.5)	6.3 (2.0–15.3)
Bolivia	90 to 94	5.5 (1.7–13.1)	5.6 (1.6–13.2)	5.6 (1.7–13.1)	5.7 (1.8–14.2)	5.7 (1.8–14.0)
Bolivia	95 plus	5.5 (1.6–13.0)	5.5 (1.6–13.1)	5.6 (1.7–13.0)	5.7 (1.8–14.1)	5.7 (1.8–13.9)
Bolivia	Age-standardized	14.3 (4.8–30.7)	14.4 (4.6–30.9)	14.5 (4.8–30.6)	14.7 (5.1–32.6)	14.7 (5.2–32.1)
Bolivia	All age	14.6 (4.9–31.3)	14.7 (4.7–31.4)	14.7 (4.9–31.0)	14.9 (5.2–33.0)	14.9 (5.2–32.5)
Ecuador	20 to 24	14.6 (10.9–19.3)	18.4 (14.9–22.3)	15.4 (10.5–21.4)	13.5 (6.1–24.2)	13.2 (5.4–24.9)
Ecuador	25 to 29	12.5 (9.2–16.9)	16.6 (13.2–20.9)	17.0 (13.0–21.6)	14.3 (7.8–22.9)	13.9 (6.7–24.1)

Supplementary Table S10: Prevalence of female SVAC by age and location for 1990, 2000, 2010, 2020, and 2023						
**Location**	**Age Range**	**1990**	**2000**	**2010**	**2020**	**2023**
Ecuador	30 to 34	11.4 (8.0–15.9)	14.3 (10.8–18.5)	17.9 (14.4–21.9)	15.5 (10.6–22.2)	14.9 (8.9–23.4)
Ecuador	35 to 39	10.8 (7.1–15.4)	12.5 (9.1–16.5)	16.7 (13.0–21.1)	17.3 (12.4–23.1)	16.5 (10.7–24.2)
Ecuador	40 to 44	10.3 (6.5–15.4)	11.3 (7.9–15.3)	14.3 (10.6–18.4)	19.8 (15.3–24.9)	20.7 (14.9–28.0)
Ecuador	45 to 49	10.0 (6.1–15.3)	10.9 (7.3–15.3)	12.2 (8.8–16.2)	13.2 (9.0–18.5)	13.4 (8.5–20.2)
Ecuador	50 to 54	9.6 (5.6–15.0)	10.3 (6.5–15.1)	11.7 (8.1–15.9)	12.8 (8.6–18.1)	13.1 (8.1–19.9)
Ecuador	55 to 59	9.2 (5.3–14.7)	9.8 (5.9–14.5)	11.0 (7.2–15.7)	12.3 (8.2–17.5)	12.7 (7.8–19.7)
Ecuador	60 to 64	8.8 (5.0–14.4)	9.3 (5.6–14.2)	10.3 (6.5–15.3)	11.7 (7.4–17.3)	12.0 (7.3–19.1)
Ecuador	65 to 69	8.8 (4.7–14.9)	8.9 (5.2–14.1)	9.7 (5.8–14.9)	10.4 (6.0–16.8)	10.7 (5.8–18.3)
Ecuador	70 to 74	8.3 (3.9–15.1)	8.4 (4.7–13.6)	9.0 (5.3–14.0)	9.5 (5.3–15.5)	9.6 (5.1–16.8)
Ecuador	75 to 79	8.1 (3.3–16.1)	8.1 (4.3–13.4)	8.6 (4.8–13.7)	9.2 (5.0–15.1)	9.3 (4.9–16.3)
Ecuador	80 to 84	6.7 (2.4–14.1)	7.1 (3.3–12.7)	7.7 (4.3–12.5)	7.9 (4.0–13.5)	7.9 (3.7–14.2)
Ecuador	85 to 89	6.1 (2.0–13.6)	6.6 (2.7–12.8)	7.1 (3.7–12.1)	7.3 (3.6–12.9)	7.3 (3.3–13.6)
Ecuador	90 to 94	5.5 (1.7–12.8)	6.0 (2.2–12.8)	6.6 (3.1–12.0)	6.7 (3.2–12.3)	6.7 (2.9–12.8)
Ecuador	95 plus	5.3 (1.6–12.6)	5.7 (1.9–12.9)	6.2 (2.5–12.5)	6.4 (2.9–12.4)	6.4 (2.8–12.5)
Ecuador	Age-standardized	10.7 (7.6–15.0)	12.5 (9.6–16.2)	13.8 (10.8–17.2)	14.0 (11.5–17.5)	13.9 (10.6–18.4)

Supplementary Table S10: Prevalence of female SVAC by age and location for 1990, 2000, 2010, 2020, and 2023						
**Location**	**Age Range**	**1990**	**2000**	**2010**	**2020**	**2023**
Ecuador	All age	11.3 (8.3–15.3)	13.3 (10.5–16.8)	14.2 (11.2–17.6)	14.1 (11.5–17.6)	14.0 (10.6–18.5)
Peru	20 to 24	21.6 (13.4–32.7)	23.3 (16.8–30.6)	22.7 (12.9–35.7)	21.8 (9.3–40.8)	21.6 (8.4–43.0)
Peru	25 to 29	22.2 (12.8–35.2)	23.2 (16.8–30.8)	21.7 (12.9–33.0)	20.9 (9.4–38.4)	20.9 (8.5–40.1)
Peru	30 to 34	24.7 (13.4–39.4)	25.0 (18.2–33.1)	22.6 (14.6–31.5)	21.4 (10.9–36.8)	21.3 (9.7–39.2)
Peru	35 to 39	26.2 (13.7–42.8)	25.5 (18.0–34.1)	22.4 (15.0–30.9)	20.9 (11.9–34.2)	20.8 (10.8–36.1)
Peru	40 to 44	26.4 (13.6–43.4)	25.0 (17.0–34.4)	21.6 (14.2–30.3)	20.2 (11.2–33.5)	20.1 (10.3–35.5)
Peru	45 to 49	27.8 (14.6–45.1)	27.6 (19.3–37.3)	23.8 (15.8–33.0)	21.9 (12.4–35.5)	21.7 (11.3–37.5)
Peru	50 to 54	23.6 (9.9–42.7)	23.7 (12.4–37.5)	22.5 (13.3–34.1)	21.5 (11.6–34.3)	21.4 (10.4–35.7)
Peru	55 to 59	18.8 (7.2–37.1)	17.3 (7.3–32.1)	16.9 (7.9–30.1)	17.9 (8.4–32.7)	18.2 (7.9–33.4)
Peru	60 to 64	21.5 (8.8–40.2)	21.7 (10.1–37.8)	21.7 (10.3–37.1)	21.6 (8.9–41.6)	21.5 (8.5–42.4)
Peru	65 to 69	19.4 (6.8–39.6)	19.4 (6.5–39.6)	19.4 (6.7–38.8)	19.4 (7.1–40.9)	19.5 (7.1–41.5)
Peru	70 to 74	15.8 (5.3–33.5)	15.8 (5.1–33.5)	15.7 (5.3–32.8)	15.8 (5.5–34.7)	15.8 (5.5–35.4)
Peru	75 to 79	14.3 (4.8–30.9)	14.3 (4.5–30.9)	14.3 (4.7–30.2)	14.3 (4.9–32.0)	14.4 (4.9–32.7)
Peru	80 to 84	10.3 (3.3–23.3)	10.3 (3.1–23.3)	10.3 (3.2–22.7)	10.3 (3.4–24.3)	10.4 (3.4–24.8)
Peru	85 to 89	9.4 (2.9–21.3)	9.4 (2.8–21.3)	9.3 (2.9–20.7)	9.3 (3.0–22.2)	9.4 (3.1–22.7)

Supplementary Table S10: Prevalence of female SVAC by age and location for 1990, 2000, 2010, 2020, and 2023						
**Location**	**Age Range**	**1990**	**2000**	**2010**	**2020**	**2023**
Peru	90 to 94	8.5 (2.6–19.5)	8.5 (2.5–19.5)	8.4 (2.6–19.0)	8.5 (2.7–20.4)	8.5 (2.7–20.8)
Peru	95 plus	8.4 (2.6–19.4)	8.4 (2.5–19.4)	8.4 (2.6–18.8)	8.4 (2.7–20.2)	8.4 (2.7–20.7)
Peru	Age-standardized	22.6 (12.5–36.4)	22.7 (15.3–32.2)	21.0 (17.5–24.8)	20.2 (16.0–26.0)	20.1 (14.6–27.7)
Peru	All age	22.9 (13.2–35.9)	23.2 (16.2–32.0)	21.2 (17.4–25.5)	20.2 (15.9–26.0)	20.1 (14.7–27.6)
Caribbean	20 to 24	16.6 (6.8–32.5)	17.9 (8.0–33.5)	18.7 (11.3–29.8)	18.9 (15.5–26.7)	19.0 (15.4–26.0)
Caribbean	25 to 29	16.6 (6.5–33.2)	17.4 (7.2–33.8)	18.8 (9.5–32.8)	19.4 (11.6–31.9)	19.4 (11.5–32.0)
Caribbean	30 to 34	17.7 (6.8–35.2)	18.0 (7.0–35.7)	19.3 (8.7–35.6)	20.2 (12.0–34.2)	20.2 (12.6–33.2)
Caribbean	35 to 39	17.7 (6.5–35.7)	17.8 (6.5–35.8)	18.6 (7.3–36.1)	20.0 (8.6–39.5)	20.1 (9.0–39.1)
Caribbean	40 to 44	18.0 (6.5–36.2)	18.2 (6.7–36.4)	18.7 (7.2–36.3)	20.0 (8.3–39.9)	20.3 (8.7–39.9)
Caribbean	45 to 49	17.5 (6.2–35.6)	17.7 (6.4–35.7)	18.1 (6.8–35.3)	18.9 (7.5–38.3)	19.2 (8.0–38.5)
Caribbean	50 to 54	17.2 (6.0–35.2)	17.1 (6.0–35.1)	17.7 (6.6–34.7)	18.1 (7.2–37.1)	18.3 (7.5–37.1)
Caribbean	55 to 59	16.7 (5.8–34.5)	16.7 (5.7–34.5)	17.0 (6.2–33.8)	17.3 (6.8–35.8)	17.3 (7.1–35.6)
Caribbean	60 to 64	16.3 (5.6–33.7)	16.3 (5.6–33.9)	16.3 (5.9–32.8)	16.7 (6.6–34.8)	16.7 (6.8–34.6)
Caribbean	65 to 69	15.0 (5.1–31.8)	15.1 (4.9–32.1)	15.1 (5.1–31.4)	15.5 (5.7–33.3)	15.5 (5.8–33.0)
Caribbean	70 to 74	12.0 (3.9–26.5)	12.1 (3.7–26.5)	12.2 (3.9–26.2)	12.3 (4.2–28.0)	12.4 (4.3–27.8)

Supplementary Table S10: Prevalence of female SVAC by age and location for 1990, 2000, 2010, 2020, and 2023						
**Location**	**Age Range**	**1990**	**2000**	**2010**	**2020**	**2023**
Caribbean	75 to 79	10.7 (3.4–24.0)	10.7 (3.3–24.0)	10.9 (3.5–23.8)	11.0 (3.7–25.5)	11.0 (3.7–25.1)
Caribbean	80 to 84	7.6 (2.3–17.6)	7.6 (2.2–17.5)	7.6 (2.3–17.3)	7.7 (2.5–18.7)	7.7 (2.5–18.5)
Caribbean	85 to 89	6.7 (2.1–15.8)	6.7 (2.0–15.7)	6.8 (2.1–15.5)	6.9 (2.2–16.9)	6.9 (2.2–16.6)
Caribbean	90 to 94	6.2 (1.9–14.5)	6.0 (1.7–14.2)	6.1 (1.8–14.0)	6.1 (1.9–15.2)	6.1 (2.0–14.9)
Caribbean	95 plus	6.3 (1.9–14.9)	6.0 (1.7–14.3)	5.9 (1.8–13.7)	6.0 (1.9–14.8)	6.0 (1.9–14.5)
Caribbean	Age-standardized	16.4 (6.0–33.3)	16.7 (6.3–33.6)	17.4 (7.5–32.6)	18.1 (9.4–33.8)	18.1 (9.7–33.6)
Caribbean	All age	16.5 (6.2–33.5)	16.9 (6.4–33.8)	17.5 (7.5–32.6)	17.9 (9.2–33.6)	17.9 (9.4–33.4)
Antigua and Barbuda	20 to 24	15.2 (5.1–32.5)	15.3 (4.9–32.6)	15.4 (5.1–32.3)	15.7 (5.5–34.5)	15.7 (5.6–34.1)
Antigua and Barbuda	25 to 29	15.5 (5.2–33.1)	15.6 (5.0–33.1)	15.7 (5.3–32.8)	16.0 (5.6–35.0)	16.0 (5.7–34.5)
Antigua and Barbuda	30 to 34	16.7 (5.7–35.0)	16.7 (5.5–35.1)	16.9 (5.7–34.8)	17.1 (6.1–37.1)	17.1 (6.2–36.7)
Antigua and Barbuda	35 to 39	16.8 (5.7–35.2)	16.8 (5.5–35.3)	17.0 (5.8–34.9)	17.2 (6.1–37.2)	17.2 (6.2–36.8)
Antigua and Barbuda	40 to 44	17.3 (5.9–36.1)	17.3 (5.7–36.1)	17.5 (6.0–35.7)	17.7 (6.3–38.1)	17.7 (6.4–37.7)
Antigua and Barbuda	45 to 49	16.8 (5.7–35.3)	16.9 (5.5–35.3)	17.0 (5.8–34.9)	17.2 (6.1–37.3)	17.2 (6.2–36.7)
Antigua and Barbuda	50 to 54	16.4 (5.6–34.6)	16.5 (5.4–34.7)	16.6 (5.6–34.3)	16.9 (6.0–36.6)	16.9 (6.1–36.2)
Antigua and Barbuda	55 to 59	15.9 (5.4–33.7)	15.9 (5.1–33.7)	16.1 (5.4–33.4)	16.3 (5.7–35.6)	16.3 (5.8–35.1)

Supplementary Table S10: Prevalence of female SVAC by age and location for 1990, 2000, 2010, 2020, and 2023						
**Location**	**Age Range**	**1990**	**2000**	**2010**	**2020**	**2023**
Antigua and Barbuda	60 to 64	15.4 (5.2–32.9)	15.4 (5.0–32.9)	15.6 (5.2–32.5)	15.8 (5.5–34.7)	15.8 (5.6–34.4)
Antigua and Barbuda	65 to 69	14.4 (4.8–31.0)	14.4 (4.6–31.0)	14.5 (4.8–30.7)	14.7 (5.1–32.8)	14.7 (5.2–32.3)
Antigua and Barbuda	70 to 74	11.6 (3.7–25.7)	11.6 (3.6–25.7)	11.7 (3.7–25.4)	11.8 (4.0–27.3)	11.9 (4.0–27.0)
Antigua and Barbuda	75 to 79	10.4 (3.3–23.5)	10.5 (3.2–23.5)	10.5 (3.3–23.2)	10.7 (3.5–25.0)	10.7 (3.6–24.7)
Antigua and Barbuda	80 to 84	7.4 (2.3–17.3)	7.4 (2.2–17.3)	7.5 (2.3–17.0)	7.6 (2.4–18.5)	7.6 (2.5–18.2)
Antigua and Barbuda	85 to 89	6.7 (2.0–15.7)	6.7 (1.9–15.7)	6.7 (2.0–15.5)	6.8 (2.2–16.8)	6.9 (2.2–16.6)
Antigua and Barbuda	90 to 94	6.0 (1.8–14.3)	6.0 (1.7–14.3)	6.1 (1.8–14.1)	6.2 (2.0–15.3)	6.2 (2.0–15.1)
Antigua and Barbuda	95 plus	6.0 (1.8–14.2)	6.0 (1.7–14.2)	6.0 (1.8–14.0)	6.1 (1.9–15.2)	6.1 (2.0–15.0)
Antigua and Barbuda	Age-standardized	15.5 (5.2–33.0)	15.6 (5.0–33.0)	15.7 (5.3–32.6)	15.9 (5.6–34.9)	15.9 (5.7–34.4)
Antigua and Barbuda	All age	15.5 (5.2–32.8)	15.7 (5.1–33.3)	15.9 (5.3–33.0)	16.0 (5.6–35.0)	15.9 (5.7–34.4)
The Bahamas	20 to 24	15.2 (5.1–32.5)	15.3 (4.9–32.6)	15.4 (5.1–32.3)	15.7 (5.5–34.5)	15.7 (5.6–34.1)
The Bahamas	25 to 29	15.5 (5.2–33.1)	15.6 (5.0–33.1)	15.7 (5.3–32.8)	16.0 (5.6–35.0)	16.0 (5.7–34.5)
The Bahamas	30 to 34	16.7 (5.7–35.0)	16.7 (5.5–35.1)	16.9 (5.7–34.8)	17.1 (6.1–37.1)	17.1 (6.2–36.7)
The Bahamas	35 to 39	16.8 (5.7–35.2)	16.8 (5.5–35.3)	17.0 (5.8–34.9)	17.2 (6.1–37.2)	17.2 (6.2–36.8)
The Bahamas	40 to 44	17.3 (5.9–36.1)	17.3 (5.7–36.1)	17.5 (6.0–35.7)	17.7 (6.3–38.1)	17.7 (6.4–37.7)

Supplementary Table S10: Prevalence of female SVAC by age and location for 1990, 2000, 2010, 2020, and 2023						
**Location**	**Age Range**	**1990**	**2000**	**2010**	**2020**	**2023**
The Bahamas	45 to 49	16.8 (5.7–35.3)	16.9 (5.5–35.3)	17.0 (5.8–34.9)	17.2 (6.1–37.3)	17.2 (6.2–36.7)
The Bahamas	50 to 54	16.4 (5.6–34.6)	16.5 (5.4–34.7)	16.6 (5.6–34.3)	16.9 (6.0–36.6)	16.9 (6.1–36.2)
The Bahamas	55 to 59	15.9 (5.4–33.7)	15.9 (5.1–33.7)	16.1 (5.4–33.4)	16.3 (5.7–35.6)	16.3 (5.8–35.1)
The Bahamas	60 to 64	15.4 (5.2–32.9)	15.4 (5.0–32.9)	15.6 (5.2–32.5)	15.8 (5.5–34.7)	15.8 (5.6–34.4)
The Bahamas	65 to 69	14.4 (4.8–31.0)	14.4 (4.6–31.0)	14.5 (4.8–30.7)	14.7 (5.1–32.8)	14.7 (5.2–32.3)
The Bahamas	70 to 74	11.6 (3.7–25.7)	11.6 (3.6–25.7)	11.7 (3.7–25.4)	11.8 (4.0–27.3)	11.9 (4.0–27.0)
The Bahamas	75 to 79	10.4 (3.3–23.5)	10.5 (3.2–23.5)	10.5 (3.3–23.2)	10.7 (3.5–25.0)	10.7 (3.6–24.7)
The Bahamas	80 to 84	7.4 (2.3–17.3)	7.4 (2.2–17.3)	7.5 (2.3–17.0)	7.6 (2.4–18.5)	7.6 (2.5–18.2)
The Bahamas	85 to 89	6.7 (2.0–15.7)	6.7 (1.9–15.7)	6.7 (2.0–15.5)	6.8 (2.2–16.8)	6.9 (2.2–16.6)
The Bahamas	90 to 94	6.0 (1.8–14.3)	6.0 (1.7–14.3)	6.1 (1.8–14.1)	6.2 (2.0–15.3)	6.2 (2.0–15.1)
The Bahamas	95 plus	6.0 (1.8–14.2)	6.0 (1.7–14.2)	6.0 (1.8–14.0)	6.1 (1.9–15.2)	6.1 (2.0–15.0)
The Bahamas	Age-standardized	15.5 (5.2–33.0)	15.6 (5.0–33.0)	15.7 (5.3–32.6)	15.9 (5.6–34.9)	15.9 (5.7–34.4)
The Bahamas	All age	15.7 (5.3–33.3)	15.8 (5.1–33.5)	16.0 (5.4–33.1)	16.1 (5.7–35.2)	16.0 (5.7–34.6)
Barbados	20 to 24	15.2 (5.1–32.5)	15.3 (4.9–32.6)	15.4 (5.1–32.3)	15.7 (5.5–34.5)	15.7 (5.6–34.1)
Barbados	25 to 29	15.5 (5.2–33.1)	15.6 (5.0–33.1)	15.7 (5.3–32.8)	16.0 (5.6–35.0)	16.0 (5.7–34.5)

Supplementary Table S10: Prevalence of female SVAC by age and location for 1990, 2000, 2010, 2020, and 2023						
**Location**	**Age Range**	**1990**	**2000**	**2010**	**2020**	**2023**
Barbados	30 to 34	16.7 (5.7–35.0)	16.7 (5.5–35.1)	16.9 (5.7–34.8)	17.1 (6.1–37.1)	17.1 (6.2–36.7)
Barbados	35 to 39	16.8 (5.7–35.2)	16.8 (5.5–35.3)	17.0 (5.8–34.9)	17.2 (6.1–37.2)	17.2 (6.2–36.8)
Barbados	40 to 44	17.3 (5.9–36.1)	17.3 (5.7–36.1)	17.5 (6.0–35.7)	17.7 (6.3–38.1)	17.7 (6.4–37.7)
Barbados	45 to 49	16.8 (5.7–35.3)	16.9 (5.5–35.3)	17.0 (5.8–34.9)	17.2 (6.1–37.3)	17.2 (6.2–36.7)
Barbados	50 to 54	16.4 (5.6–34.6)	16.5 (5.4–34.7)	16.6 (5.6–34.3)	16.9 (6.0–36.6)	16.9 (6.1–36.2)
Barbados	55 to 59	15.9 (5.4–33.7)	15.9 (5.1–33.7)	16.1 (5.4–33.4)	16.3 (5.7–35.6)	16.3 (5.8–35.1)
Barbados	60 to 64	15.4 (5.2–32.9)	15.4 (5.0–32.9)	15.6 (5.2–32.5)	15.8 (5.5–34.7)	15.8 (5.6–34.4)
Barbados	65 to 69	14.4 (4.8–31.0)	14.4 (4.6–31.0)	14.5 (4.8–30.7)	14.7 (5.1–32.8)	14.7 (5.2–32.3)
Barbados	70 to 74	11.6 (3.7–25.7)	11.6 (3.6–25.7)	11.7 (3.7–25.4)	11.8 (4.0–27.3)	11.9 (4.0–27.0)
Barbados	75 to 79	10.4 (3.3–23.5)	10.5 (3.2–23.5)	10.5 (3.3–23.2)	10.7 (3.5–25.0)	10.7 (3.6–24.7)
Barbados	80 to 84	7.4 (2.3–17.3)	7.4 (2.2–17.3)	7.5 (2.3–17.0)	7.6 (2.4–18.5)	7.6 (2.5–18.2)
Barbados	85 to 89	6.7 (2.0–15.7)	6.7 (1.9–15.7)	6.7 (2.0–15.5)	6.8 (2.2–16.8)	6.9 (2.2–16.6)
Barbados	90 to 94	6.0 (1.8–14.3)	6.0 (1.7–14.3)	6.1 (1.8–14.1)	6.2 (2.0–15.3)	6.2 (2.0–15.1)
Barbados	95 plus	6.0 (1.8–14.2)	6.0 (1.7–14.2)	6.0 (1.8–14.0)	6.1 (1.9–15.2)	6.1 (2.0–15.0)
Barbados	Age-standardized	15.5 (5.2–33.0)	15.6 (5.0–33.0)	15.7 (5.3–32.6)	15.9 (5.6–34.9)	15.9 (5.7–34.4)

Supplementary Table S10: Prevalence of female SVAC by age and location for 1990, 2000, 2010, 2020, and 2023						
**Location**	**Age Range**	**1990**	**2000**	**2010**	**2020**	**2023**
Barbados	All age	15.2 (5.1–32.3)	15.3 (4.9–32.5)	15.5 (5.2–32.2)	15.6 (5.5–34.1)	15.5 (5.5–33.6)
Belize	20 to 24	12.3 (8.1–18.0)	12.2 (8.4–16.8)	11.5 (6.6–18.6)	11.7 (4.7–23.4)	11.8 (4.5–25.6)
Belize	25 to 29	12.1 (7.8–18.0)	12.9 (9.0–17.8)	12.7 (8.0–18.7)	12.5 (5.5–23.7)	12.5 (5.0–25.6)
Belize	30 to 34	12.0 (7.1–18.6)	12.9 (8.9–18.1)	13.1 (8.9–18.0)	12.7 (6.5–22.1)	12.7 (5.7–23.7)
Belize	35 to 39	11.8 (6.5–19.6)	11.8 (7.9–16.4)	12.5 (8.4–17.3)	12.7 (7.6–19.2)	12.7 (7.0–20.7)
Belize	40 to 44	12.4 (5.9–22.6)	12.1 (7.7–17.6)	12.8 (8.5–17.8)	13.8 (8.3–20.9)	13.9 (7.7–22.6)
Belize	45 to 49	12.1 (4.9–23.5)	11.2 (6.5–17.2)	11.6 (7.6–16.5)	13.1 (7.7–20.4)	13.4 (7.5–22.2)
Belize	50 to 54	12.9 (4.9–25.6)	12.1 (6.5–19.6)	11.8 (7.5–17.2)	12.1 (7.1–19.1)	12.2 (6.6–20.4)
Belize	55 to 59	11.0 (4.0–22.5)	9.9 (5.1–16.6)	10.3 (6.2–15.7)	11.7 (6.7–19.0)	12.1 (6.4–20.8)
Belize	60 to 64	10.6 (3.9–22.2)	9.6 (4.9–16.3)	9.8 (5.5–15.6)	10.8 (6.0–18.4)	11.0 (5.6–19.6)
Belize	65 to 69	12.3 (4.6–25.0)	12.4 (5.8–21.8)	12.4 (6.4–21.1)	12.3 (5.8–22.0)	12.2 (5.3–22.8)
Belize	70 to 74	9.1 (3.3–19.5)	8.8 (3.8–16.5)	8.8 (3.9–16.4)	9.2 (3.5–19.5)	9.3 (3.3–20.3)
Belize	75 to 79	9.8 (3.5–20.6)	10.5 (4.3–20.2)	10.5 (4.5–19.9)	10.0 (3.6–21.8)	9.7 (3.5–21.8)
Belize	80 to 84	6.0 (1.8–14.1)	5.9 (1.7–14.1)	6.0 (1.8–13.8)	6.0 (1.9–14.9)	6.0 (1.9–14.7)
Belize	85 to 89	5.4 (1.6–12.8)	5.3 (1.5–12.7)	5.4 (1.6–12.5)	5.4 (1.7–13.5)	5.4 (1.7–13.3)

Supplementary Table S10: Prevalence of female SVAC by age and location for 1990, 2000, 2010, 2020, and 2023						
**Location**	**Age Range**	**1990**	**2000**	**2010**	**2020**	**2023**
Belize	90 to 94	4.8 (1.4–11.6)	4.8 (1.4–11.6)	4.8 (1.4–11.3)	4.9 (1.5–12.3)	4.9 (1.5–12.1)
Belize	95 plus	4.8 (1.4–11.5)	4.8 (1.4–11.5)	4.8 (1.4–11.2)	4.8 (1.5–12.2)	4.8 (1.5–12.0)
Belize	Age-standardized	11.6 (6.6–19.1)	11.5 (7.6–16.6)	11.7 (8.6–15.3)	12.0 (7.9–17.3)	12.1 (7.3–18.8)
Belize	All age	11.8 (7.2–18.3)	11.9 (8.2–16.6)	12.0 (8.8–15.9)	12.2 (8.0–17.9)	12.3 (7.4–19.6)
Bermuda	20 to 24	15.2 (5.1–32.5)	15.3 (4.9–32.6)	15.4 (5.1–32.3)	15.7 (5.5–34.5)	15.7 (5.6–34.1)
Bermuda	25 to 29	15.5 (5.2–33.1)	15.6 (5.0–33.1)	15.7 (5.3–32.8)	16.0 (5.6–35.0)	16.0 (5.7–34.5)
Bermuda	30 to 34	16.7 (5.7–35.0)	16.7 (5.5–35.1)	16.9 (5.7–34.8)	17.1 (6.1–37.1)	17.1 (6.2–36.7)
Bermuda	35 to 39	16.8 (5.7–35.2)	16.8 (5.5–35.3)	17.0 (5.8–34.9)	17.2 (6.1–37.2)	17.2 (6.2–36.8)
Bermuda	40 to 44	17.3 (5.9–36.1)	17.3 (5.7–36.1)	17.5 (6.0–35.7)	17.7 (6.3–38.1)	17.7 (6.4–37.7)
Bermuda	45 to 49	16.8 (5.7–35.3)	16.9 (5.5–35.3)	17.0 (5.8–34.9)	17.2 (6.1–37.3)	17.2 (6.2–36.7)
Bermuda	50 to 54	16.4 (5.6–34.6)	16.5 (5.4–34.7)	16.6 (5.6–34.3)	16.9 (6.0–36.6)	16.9 (6.1–36.2)
Bermuda	55 to 59	15.9 (5.4–33.7)	15.9 (5.1–33.7)	16.1 (5.4–33.4)	16.3 (5.7–35.6)	16.3 (5.8–35.1)
Bermuda	60 to 64	15.4 (5.2–32.9)	15.4 (5.0–32.9)	15.6 (5.2–32.5)	15.8 (5.5–34.7)	15.8 (5.6–34.4)
Bermuda	65 to 69	14.4 (4.8–31.0)	14.4 (4.6–31.0)	14.5 (4.8–30.7)	14.7 (5.1–32.8)	14.7 (5.2–32.3)
Bermuda	70 to 74	11.6 (3.7–25.7)	11.6 (3.6–25.7)	11.7 (3.7–25.4)	11.8 (4.0–27.3)	11.9 (4.0–27.0)

Supplementary Table S10: Prevalence of female SVAC by age and location for 1990, 2000, 2010, 2020, and 2023						
**Location**	**Age Range**	**1990**	**2000**	**2010**	**2020**	**2023**
Bermuda	75 to 79	10.4 (3.3–23.5)	10.5 (3.2–23.5)	10.5 (3.3–23.2)	10.7 (3.5–25.0)	10.7 (3.6–24.7)
Bermuda	80 to 84	7.4 (2.3–17.3)	7.4 (2.2–17.3)	7.5 (2.3–17.0)	7.6 (2.4–18.5)	7.6 (2.5–18.2)
Bermuda	85 to 89	6.7 (2.0–15.7)	6.7 (1.9–15.7)	6.7 (2.0–15.5)	6.8 (2.2–16.8)	6.9 (2.2–16.6)
Bermuda	90 to 94	6.0 (1.8–14.3)	6.0 (1.7–14.3)	6.1 (1.8–14.1)	6.2 (2.0–15.3)	6.2 (2.0–15.1)
Bermuda	95 plus	6.0 (1.8–14.2)	6.0 (1.7–14.2)	6.0 (1.8–14.0)	6.1 (1.9–15.2)	6.1 (2.0–15.0)
Bermuda	Age-standardized	15.5 (5.2–33.0)	15.6 (5.0–33.0)	15.7 (5.3–32.6)	15.9 (5.6–34.9)	15.9 (5.7–34.4)
Bermuda	All age	15.5 (5.2–33.0)	15.6 (5.0–32.9)	15.4 (5.2–32.1)	15.2 (5.3–33.3)	15.0 (5.3–32.6)
Cuba	20 to 24	15.2 (5.1–32.5)	15.3 (4.9–32.6)	15.4 (5.1–32.3)	15.7 (5.5–34.5)	15.7 (5.6–34.1)
Cuba	25 to 29	15.5 (5.2–33.1)	15.6 (5.0–33.1)	15.7 (5.3–32.8)	16.0 (5.6–35.0)	16.0 (5.7–34.5)
Cuba	30 to 34	16.7 (5.7–35.0)	16.7 (5.5–35.1)	16.9 (5.7–34.8)	17.1 (6.1–37.1)	17.1 (6.2–36.7)
Cuba	35 to 39	16.8 (5.7–35.2)	16.8 (5.5–35.3)	17.0 (5.8–34.9)	17.2 (6.1–37.2)	17.2 (6.2–36.8)
Cuba	40 to 44	17.3 (5.9–36.1)	17.3 (5.7–36.1)	17.5 (6.0–35.7)	17.7 (6.3–38.1)	17.7 (6.4–37.7)
Cuba	45 to 49	16.8 (5.7–35.3)	16.9 (5.5–35.3)	17.0 (5.8–34.9)	17.2 (6.1–37.3)	17.2 (6.2–36.7)
Cuba	50 to 54	16.4 (5.6–34.6)	16.5 (5.4–34.7)	16.6 (5.6–34.3)	16.9 (6.0–36.6)	16.9 (6.1–36.2)
Cuba	55 to 59	15.9 (5.4–33.7)	15.9 (5.1–33.7)	16.1 (5.4–33.4)	16.3 (5.7–35.6)	16.3 (5.8–35.1)

Supplementary Table S10: Prevalence of female SVAC by age and location for 1990, 2000, 2010, 2020, and 2023						
**Location**	**Age Range**	**1990**	**2000**	**2010**	**2020**	**2023**
Cuba	60 to 64	15.4 (5.2–32.9)	15.4 (5.0–32.9)	15.6 (5.2–32.5)	15.8 (5.5–34.7)	15.8 (5.6–34.4)
Cuba	65 to 69	14.4 (4.8–31.0)	14.4 (4.6–31.0)	14.5 (4.8–30.7)	14.7 (5.1–32.8)	14.7 (5.2–32.3)
Cuba	70 to 74	11.6 (3.7–25.7)	11.6 (3.6–25.7)	11.7 (3.7–25.4)	11.8 (4.0–27.3)	11.9 (4.0–27.0)
Cuba	75 to 79	10.4 (3.3–23.5)	10.5 (3.2–23.5)	10.5 (3.3–23.2)	10.7 (3.5–25.0)	10.7 (3.6–24.7)
Cuba	80 to 84	7.4 (2.3–17.3)	7.4 (2.2–17.3)	7.5 (2.3–17.0)	7.6 (2.4–18.5)	7.6 (2.5–18.2)
Cuba	85 to 89	6.7 (2.0–15.7)	6.7 (1.9–15.7)	6.7 (2.0–15.5)	6.8 (2.2–16.8)	6.9 (2.2–16.6)
Cuba	90 to 94	6.0 (1.8–14.3)	6.0 (1.7–14.3)	6.1 (1.8–14.1)	6.2 (2.0–15.3)	6.2 (2.0–15.1)
Cuba	95 plus	6.0 (1.8–14.2)	6.0 (1.7–14.2)	6.0 (1.8–14.0)	6.1 (1.9–15.2)	6.1 (2.0–15.0)
Cuba	Age-standardized	15.5 (5.2–33.0)	15.6 (5.0–33.0)	15.7 (5.3–32.6)	15.9 (5.6–34.9)	15.9 (5.7–34.4)
Cuba	All age	15.5 (5.2–32.9)	15.6 (5.0–33.0)	15.6 (5.2–32.4)	15.5 (5.5–34.1)	15.4 (5.5–33.5)
Dominica	20 to 24	15.2 (5.1–32.5)	15.3 (4.9–32.6)	15.4 (5.1–32.3)	15.7 (5.5–34.5)	15.7 (5.6–34.1)
Dominica	25 to 29	15.5 (5.2–33.1)	15.6 (5.0–33.1)	15.7 (5.3–32.8)	16.0 (5.6–35.0)	16.0 (5.7–34.5)
Dominica	30 to 34	16.7 (5.7–35.0)	16.7 (5.5–35.1)	16.9 (5.7–34.8)	17.1 (6.1–37.1)	17.1 (6.2–36.7)
Dominica	35 to 39	16.8 (5.7–35.2)	16.8 (5.5–35.3)	17.0 (5.8–34.9)	17.2 (6.1–37.2)	17.2 (6.2–36.8)
Dominica	40 to 44	17.3 (5.9–36.1)	17.3 (5.7–36.1)	17.5 (6.0–35.7)	17.7 (6.3–38.1)	17.7 (6.4–37.7)

Supplementary Table S10: Prevalence of female SVAC by age and location for 1990, 2000, 2010, 2020, and 2023						
**Location**	**Age Range**	**1990**	**2000**	**2010**	**2020**	**2023**
Dominica	45 to 49	16.8 (5.7–35.3)	16.9 (5.5–35.3)	17.0 (5.8–34.9)	17.2 (6.1–37.3)	17.2 (6.2–36.7)
Dominica	50 to 54	16.4 (5.6–34.6)	16.5 (5.4–34.7)	16.6 (5.6–34.3)	16.9 (6.0–36.6)	16.9 (6.1–36.2)
Dominica	55 to 59	15.9 (5.4–33.7)	15.9 (5.1–33.7)	16.1 (5.4–33.4)	16.3 (5.7–35.6)	16.3 (5.8–35.1)
Dominica	60 to 64	15.4 (5.2–32.9)	15.4 (5.0–32.9)	15.6 (5.2–32.5)	15.8 (5.5–34.7)	15.8 (5.6–34.4)
Dominica	65 to 69	14.4 (4.8–31.0)	14.4 (4.6–31.0)	14.5 (4.8–30.7)	14.7 (5.1–32.8)	14.7 (5.2–32.3)
Dominica	70 to 74	11.6 (3.7–25.7)	11.6 (3.6–25.7)	11.7 (3.7–25.4)	11.8 (4.0–27.3)	11.9 (4.0–27.0)
Dominica	75 to 79	10.4 (3.3–23.5)	10.5 (3.2–23.5)	10.5 (3.3–23.2)	10.7 (3.5–25.0)	10.7 (3.6–24.7)
Dominica	80 to 84	7.4 (2.3–17.3)	7.4 (2.2–17.3)	7.5 (2.3–17.0)	7.6 (2.4–18.5)	7.6 (2.5–18.2)
Dominica	85 to 89	6.7 (2.0–15.7)	6.7 (1.9–15.7)	6.7 (2.0–15.5)	6.8 (2.2–16.8)	6.9 (2.2–16.6)
Dominica	90 to 94	6.0 (1.8–14.3)	6.0 (1.7–14.3)	6.1 (1.8–14.1)	6.2 (2.0–15.3)	6.2 (2.0–15.1)
Dominica	95 plus	6.0 (1.8–14.2)	6.0 (1.7–14.2)	6.0 (1.8–14.0)	6.1 (1.9–15.2)	6.1 (2.0–15.0)
Dominica	Age-standardized	15.5 (5.2–33.0)	15.6 (5.0–33.0)	15.7 (5.3–32.6)	15.9 (5.6–34.9)	15.9 (5.7–34.4)
Dominica	All age	15.1 (5.1–32.1)	15.1 (4.9–32.1)	15.4 (5.1–32.0)	15.6 (5.5–34.3)	15.6 (5.6–33.9)
Dominican Republic	20 to 24	15.2 (5.1–32.5)	15.3 (4.9–32.6)	15.4 (5.1–32.3)	15.7 (5.5–34.5)	15.7 (5.6–34.1)
Dominican Republic	25 to 29	15.5 (5.2–33.1)	15.6 (5.0–33.1)	15.7 (5.3–32.8)	16.0 (5.6–35.0)	16.0 (5.7–34.5)

Supplementary Table S10: Prevalence of female SVAC by age and location for 1990, 2000, 2010, 2020, and 2023						
**Location**	**Age Range**	**1990**	**2000**	**2010**	**2020**	**2023**
Dominican Republic	30 to 34	16.7 (5.7–35.0)	16.7 (5.5–35.1)	16.9 (5.7–34.8)	17.1 (6.1–37.1)	17.1 (6.2–36.7)
Dominican Republic	35 to 39	16.8 (5.7–35.2)	16.8 (5.5–35.3)	17.0 (5.8–34.9)	17.2 (6.1–37.2)	17.2 (6.2–36.8)
Dominican Republic	40 to 44	17.3 (5.9–36.1)	17.3 (5.7–36.1)	17.5 (6.0–35.7)	17.7 (6.3–38.1)	17.7 (6.4–37.7)
Dominican Republic	45 to 49	16.8 (5.7–35.3)	16.9 (5.5–35.3)	17.0 (5.8–34.9)	17.2 (6.1–37.3)	17.2 (6.2–36.7)
Dominican Republic	50 to 54	16.4 (5.6–34.6)	16.5 (5.4–34.7)	16.6 (5.6–34.3)	16.9 (6.0–36.6)	16.9 (6.1–36.2)
Dominican Republic	55 to 59	15.9 (5.4–33.7)	15.9 (5.1–33.7)	16.1 (5.4–33.4)	16.3 (5.7–35.6)	16.3 (5.8–35.1)
Dominican Republic	60 to 64	15.4 (5.2–32.9)	15.4 (5.0–32.9)	15.6 (5.2–32.5)	15.8 (5.5–34.7)	15.8 (5.6–34.4)
Dominican Republic	65 to 69	14.4 (4.8–31.0)	14.4 (4.6–31.0)	14.5 (4.8–30.7)	14.7 (5.1–32.8)	14.7 (5.2–32.3)
Dominican Republic	70 to 74	11.6 (3.7–25.7)	11.6 (3.6–25.7)	11.7 (3.7–25.4)	11.8 (4.0–27.3)	11.9 (4.0–27.0)
Dominican Republic	75 to 79	10.4 (3.3–23.5)	10.5 (3.2–23.5)	10.5 (3.3–23.2)	10.7 (3.5–25.0)	10.7 (3.6–24.7)
Dominican Republic	80 to 84	7.4 (2.3–17.3)	7.4 (2.2–17.3)	7.5 (2.3–17.0)	7.6 (2.4–18.5)	7.6 (2.5–18.2)
Dominican Republic	85 to 89	6.7 (2.0–15.7)	6.7 (1.9–15.7)	6.7 (2.0–15.5)	6.8 (2.2–16.8)	6.9 (2.2–16.6)
Dominican Republic	90 to 94	6.0 (1.8–14.3)	6.0 (1.7–14.3)	6.1 (1.8–14.1)	6.2 (2.0–15.3)	6.2 (2.0–15.1)
Dominican Republic	95 plus	6.0 (1.8–14.2)	6.0 (1.7–14.2)	6.0 (1.8–14.0)	6.1 (1.9–15.2)	6.1 (2.0–15.0)
Dominican Republic	Age-standardized	15.5 (5.2–33.0)	15.6 (5.0–33.0)	15.7 (5.3–32.6)	15.9 (5.6–34.9)	15.9 (5.7–34.4)

Supplementary Table S10: Prevalence of female SVAC by age and location for 1990, 2000, 2010, 2020, and 2023						
**Location**	**Age Range**	**1990**	**2000**	**2010**	**2020**	**2023**
Dominican Republic	All age	15.8 (5.3–33.4)	15.8 (5.1–33.4)	15.9 (5.3–32.9)	16.0 (5.6–35.0)	16.0 (5.7–34.5)
Grenada	20 to 24	19.5 (7.1–38.6)	19.0 (7.6–36.1)	18.1 (10.3–29.1)	17.6 (12.9–23.4)	17.8 (11.6–25.7)
Grenada	25 to 29	20.4 (7.4–40.2)	20.4 (8.3–38.0)	20.1 (11.7–31.5)	19.8 (14.8–25.9)	19.9 (13.2–28.3)
Grenada	30 to 34	21.7 (7.9–42.1)	21.3 (8.7–39.3)	20.5 (12.0–32.0)	20.0 (15.0–26.1)	20.1 (13.3–28.5)
Grenada	35 to 39	23.0 (8.7–44.6)	23.6 (9.8–42.5)	24.2 (14.6–37.1)	24.2 (18.7–30.4)	24.1 (16.7–33.1)
Grenada	40 to 44	23.6 (9.0–45.6)	24.1 (10.1–43.1)	24.4 (14.7–37.2)	24.4 (18.9–30.4)	24.3 (16.8–33.2)
Grenada	45 to 49	23.1 (8.7–44.7)	23.5 (9.8–42.3)	23.7 (14.2–36.4)	23.6 (18.1–29.7)	23.6 (16.2–32.4)
Grenada	50 to 54	23.1 (8.7–44.8)	23.9 (10.0–42.9)	24.7 (14.9–37.7)	24.9 (19.2–31.1)	24.7 (17.1–33.8)
Grenada	55 to 59	21.6 (7.9–42.0)	21.4 (8.8–39.5)	20.9 (12.3–32.6)	20.6 (15.4–26.7)	20.6 (13.7–29.2)
Grenada	60 to 64	21.1 (7.7–41.3)	21.1 (8.6–39.0)	20.7 (12.1–32.3)	20.4 (15.3–26.5)	20.5 (13.6–28.9)
Grenada	65 to 69	19.7 (6.9–40.1)	19.7 (6.6–40.1)	19.8 (6.9–39.6)	20.1 (7.4–41.9)	20.0 (7.5–41.5)
Grenada	70 to 74	16.1 (5.4–34.0)	16.1 (5.2–34.0)	16.2 (5.4–33.5)	16.3 (5.8–35.7)	16.3 (5.8–35.3)
Grenada	75 to 79	14.6 (4.8–31.4)	14.6 (4.6–31.3)	14.7 (4.8–30.9)	14.8 (5.1–33.0)	14.8 (5.2–32.6)
Grenada	80 to 84	10.5 (3.3–23.7)	10.5 (3.2–23.7)	10.6 (3.3–23.3)	10.7 (3.6–25.1)	10.7 (3.6–24.7)
Grenada	85 to 89	9.5 (3.0–21.7)	9.5 (2.9–21.6)	9.6 (3.0–21.3)	9.7 (3.2–23.0)	9.7 (3.2–22.7)

Supplementary Table S10: Prevalence of female SVAC by age and location for 1990, 2000, 2010, 2020, and 2023						
**Location**	**Age Range**	**1990**	**2000**	**2010**	**2020**	**2023**
Grenada	90 to 94	8.6 (2.7–19.9)	8.6 (2.6–19.8)	8.7 (2.7–19.5)	8.8 (2.9–21.1)	8.8 (2.9–20.8)
Grenada	95 plus	8.6 (2.7–19.7)	8.6 (2.5–19.7)	8.6 (2.7–19.4)	8.7 (2.8–20.9)	8.7 (2.9–20.6)
Grenada	Age-standardized	20.9 (7.6–41.2)	21.0 (8.5–39.2)	20.9 (12.0–33.3)	20.7 (16.0–26.7)	20.8 (15.0–28.0)
Grenada	All age	20.5 (7.4–40.8)	20.8 (8.4–38.9)	20.8 (11.9–33.1)	20.6 (15.8–26.8)	20.6 (14.9–27.7)
Guyana	20 to 24	15.2 (5.1–32.5)	15.3 (4.9–32.6)	15.4 (5.1–32.3)	15.7 (5.5–34.5)	15.7 (5.6–34.1)
Guyana	25 to 29	15.5 (5.2–33.1)	15.6 (5.0–33.1)	15.7 (5.3–32.8)	16.0 (5.6–35.0)	16.0 (5.7–34.5)
Guyana	30 to 34	16.7 (5.7–35.0)	16.7 (5.5–35.1)	16.9 (5.7–34.8)	17.1 (6.1–37.1)	17.1 (6.2–36.7)
Guyana	35 to 39	16.8 (5.7–35.2)	16.8 (5.5–35.3)	17.0 (5.8–34.9)	17.2 (6.1–37.2)	17.2 (6.2–36.8)
Guyana	40 to 44	17.3 (5.9–36.1)	17.3 (5.7–36.1)	17.5 (6.0–35.7)	17.7 (6.3–38.1)	17.7 (6.4–37.7)
Guyana	45 to 49	16.8 (5.7–35.3)	16.9 (5.5–35.3)	17.0 (5.8–34.9)	17.2 (6.1–37.3)	17.2 (6.2–36.7)
Guyana	50 to 54	16.4 (5.6–34.6)	16.5 (5.4–34.7)	16.6 (5.6–34.3)	16.9 (6.0–36.6)	16.9 (6.1–36.2)
Guyana	55 to 59	15.9 (5.4–33.7)	15.9 (5.1–33.7)	16.1 (5.4–33.4)	16.3 (5.7–35.6)	16.3 (5.8–35.1)
Guyana	60 to 64	15.4 (5.2–32.9)	15.4 (5.0–32.9)	15.6 (5.2–32.5)	15.8 (5.5–34.7)	15.8 (5.6–34.4)
Guyana	65 to 69	14.4 (4.8–31.0)	14.4 (4.6–31.0)	14.5 (4.8–30.7)	14.7 (5.1–32.8)	14.7 (5.2–32.3)
Guyana	70 to 74	11.6 (3.7–25.7)	11.6 (3.6–25.7)	11.7 (3.7–25.4)	11.8 (4.0–27.3)	11.9 (4.0–27.0)

Supplementary Table S10: Prevalence of female SVAC by age and location for 1990, 2000, 2010, 2020, and 2023						
**Location**	**Age Range**	**1990**	**2000**	**2010**	**2020**	**2023**
Guyana	75 to 79	10.4 (3.3–23.5)	10.5 (3.2–23.5)	10.5 (3.3–23.2)	10.7 (3.5–25.0)	10.7 (3.6–24.7)
Guyana	80 to 84	7.4 (2.3–17.3)	7.4 (2.2–17.3)	7.5 (2.3–17.0)	7.6 (2.4–18.5)	7.6 (2.5–18.2)
Guyana	85 to 89	6.7 (2.0–15.7)	6.7 (1.9–15.7)	6.7 (2.0–15.5)	6.8 (2.2–16.8)	6.9 (2.2–16.6)
Guyana	90 to 94	6.0 (1.8–14.3)	6.0 (1.7–14.3)	6.1 (1.8–14.1)	6.2 (2.0–15.3)	6.2 (2.0–15.1)
Guyana	95 plus	6.0 (1.8–14.2)	6.0 (1.7–14.2)	6.0 (1.8–14.0)	6.1 (1.9–15.2)	6.1 (2.0–15.0)
Guyana	Age-standardized	15.5 (5.2–33.0)	15.6 (5.0–33.0)	15.7 (5.3–32.6)	15.9 (5.6–34.9)	15.9 (5.7–34.4)
Guyana	All age	15.8 (5.3–33.4)	15.9 (5.1–33.6)	16.0 (5.4–33.2)	16.1 (5.7–35.2)	16.1 (5.7–34.7)
Haiti	20 to 24	24.8 (9.7–46.4)	25.8 (13.1–42.0)	26.6 (20.0–34.2)	26.4 (15.2–39.2)	26.1 (13.4–42.0)
Haiti	25 to 29	24.6 (9.2–47.0)	25.5 (11.0–45.5)	26.4 (15.6–40.6)	26.7 (18.1–36.8)	26.6 (16.3–39.6)
Haiti	30 to 34	25.5 (9.7–48.6)	26.0 (10.4–47.0)	26.7 (13.1–44.8)	26.9 (19.5–37.6)	26.9 (19.1–36.4)
Haiti	35 to 39	25.0 (9.4–48.1)	25.2 (9.0–48.2)	25.5 (9.5–48.0)	25.9 (10.1–50.6)	25.9 (10.3–50.1)
Haiti	40 to 44	25.5 (9.6–48.7)	25.6 (9.2–48.8)	26.0 (9.7–48.6)	26.4 (10.4–51.2)	26.4 (10.5–50.7)
Haiti	45 to 49	24.7 (9.2–47.6)	24.8 (8.9–47.7)	25.2 (9.3–47.5)	25.6 (10.0–50.1)	25.6 (10.1–49.7)
Haiti	50 to 54	24.1 (8.9–46.7)	24.2 (8.6–46.8)	24.5 (9.0–46.6)	24.9 (9.6–49.2)	24.9 (9.8–48.8)
Haiti	55 to 59	23.3 (8.5–45.5)	23.4 (8.2–45.6)	23.7 (8.7–45.4)	24.1 (9.2–48.0)	24.1 (9.4–47.6)

Supplementary Table S10: Prevalence of female SVAC by age and location for 1990, 2000, 2010, 2020, and 2023						
**Location**	**Age Range**	**1990**	**2000**	**2010**	**2020**	**2023**
Haiti	60 to 64	22.5 (8.2–44.5)	22.6 (7.9–44.5)	23.0 (8.3–44.3)	23.3 (8.9–46.9)	23.4 (9.0–46.5)
Haiti	65 to 69	21.1 (7.6–42.3)	21.2 (7.3–42.4)	21.5 (7.7–42.2)	21.9 (8.2–44.7)	21.9 (8.3–44.4)
Haiti	70 to 74	17.3 (5.9–36.0)	17.3 (5.7–36.1)	17.6 (6.0–35.9)	17.9 (6.4–38.4)	17.9 (6.5–38.0)
Haiti	75 to 79	15.7 (5.3–33.3)	15.8 (5.1–33.4)	16.0 (5.4–33.2)	16.3 (5.7–35.6)	16.3 (5.8–35.2)
Haiti	80 to 84	11.4 (3.6–25.3)	11.4 (3.5–25.4)	11.6 (3.7–25.3)	11.8 (4.0–27.3)	11.8 (4.0–27.0)
Haiti	85 to 89	10.3 (3.3–23.2)	10.3 (3.1–23.3)	10.5 (3.3–23.1)	10.7 (3.5–25.0)	10.7 (3.6–24.8)
Haiti	90 to 94	9.4 (2.9–21.3)	9.4 (2.8–21.4)	9.5 (3.0–21.3)	9.7 (3.2–23.0)	9.7 (3.2–22.8)
Haiti	95 plus	9.3 (2.9–21.2)	9.3 (2.8–21.2)	9.5 (3.0–21.1)	9.6 (3.2–22.9)	9.7 (3.2–22.6)
Haiti	Age-standardized	23.5 (9.1–44.9)	23.9 (9.7–44.3)	24.4 (11.7–42.0)	24.6 (15.4–39.8)	24.6 (15.8–38.9)
Haiti	All age	24.2 (9.5–45.7)	24.7 (10.4–44.9)	25.3 (13.4–41.4)	25.5 (17.1–39.1)	25.4 (17.1–38.7)
Jamaica	20 to 24	16.4 (12.0–21.8)	19.6 (15.6–24.2)	19.3 (13.7–26.3)	17.5 (10.8–26.2)	17.3 (9.5–28.8)
Jamaica	25 to 29	15.0 (10.8–20.4)	16.9 (12.8–21.9)	19.4 (14.5–24.9)	19.7 (12.8–28.8)	19.5 (11.2–31.1)
Jamaica	30 to 34	14.9 (10.1–21.2)	15.9 (12.0–20.6)	18.8 (14.5–23.5)	20.2 (13.5–29.3)	20.2 (11.7–32.7)
Jamaica	35 to 39	15.0 (8.9–23.2)	15.2 (11.1–19.9)	18.6 (14.3–23.6)	22.4 (16.0–30.1)	22.7 (14.1–33.7)
Jamaica	40 to 44	15.5 (7.7–26.8)	15.0 (10.2–20.9)	17.8 (13.6–22.5)	21.0 (16.3–26.5)	21.1 (15.0–28.6)

Supplementary Table S10: Prevalence of female SVAC by age and location for 1990, 2000, 2010, 2020, and 2023						
**Location**	**Age Range**	**1990**	**2000**	**2010**	**2020**	**2023**
Jamaica	45 to 49	15.8 (6.9–29.2)	15.1 (9.4–22.8)	17.3 (13.0–22.1)	18.9 (13.9–24.6)	18.6 (12.5–26.6)
Jamaica	50 to 54	17.0 (6.3–33.6)	16.6 (8.1–28.6)	18.2 (13.0–24.3)	19.3 (14.6–24.8)	18.9 (12.9–26.5)
Jamaica	55 to 59	17.8 (6.5–36.4)	17.7 (7.7–32.6)	17.6 (11.4–25.7)	17.2 (12.7–22.5)	17.0 (11.4–24.2)
Jamaica	60 to 64	18.4 (6.7–37.4)	19.2 (8.0–35.7)	19.1 (11.6–29.2)	17.2 (12.4–22.8)	16.6 (10.9–23.8)
Jamaica	65 to 69	16.0 (5.6–33.7)	15.9 (5.5–32.8)	15.5 (6.4–29.6)	15.1 (7.8–26.1)	15.0 (7.7–25.0)
Jamaica	70 to 74	13.0 (4.3–28.5)	13.0 (4.1–28.5)	13.1 (4.3–28.1)	13.2 (4.5–30.0)	13.2 (4.6–29.7)
Jamaica	75 to 79	11.8 (3.8–26.1)	11.8 (3.6–26.1)	11.8 (3.8–25.7)	12.0 (4.0–27.6)	12.0 (4.1–27.2)
Jamaica	80 to 84	8.4 (2.6–19.4)	8.4 (2.5–19.4)	8.5 (2.6–19.0)	8.6 (2.8–20.6)	8.5 (2.8–20.3)
Jamaica	85 to 89	7.6 (2.3–17.6)	7.6 (2.2–17.6)	7.6 (2.3–17.3)	7.7 (2.5–18.7)	7.7 (2.5–18.5)
Jamaica	90 to 94	6.9 (2.1–16.1)	6.9 (2.0–16.1)	6.9 (2.1–15.8)	7.0 (2.2–17.1)	7.0 (2.3–16.9)
Jamaica	95 plus	6.8 (2.1–16.0)	6.8 (2.0–16.0)	6.8 (2.1–15.7)	6.9 (2.2–17.0)	6.9 (2.2–16.7)
Jamaica	Age-standardized	15.5 (9.1–25.0)	16.2 (11.3–22.5)	17.7 (14.2–21.5)	18.5 (15.9–21.5)	18.3 (14.5–23.3)
Jamaica	All age	15.4 (9.7–23.5)	16.1 (11.7–21.8)	17.6 (14.3–21.1)	18.3 (15.8–21.4)	18.2 (14.4–23.1)
Puerto Rico	20 to 24	11.2 (3.6–25.0)	11.3 (3.5–25.2)	11.6 (3.7–25.2)	11.8 (4.0–27.3)	11.8 (4.0–27.0)
Puerto Rico	25 to 29	11.7 (3.8–26.1)	11.9 (3.7–26.3)	12.1 (3.9–26.3)	12.4 (4.2–28.4)	12.4 (4.3–28.0)

Supplementary Table S10: Prevalence of female SVAC by age and location for 1990, 2000, 2010, 2020, and 2023						
**Location**	**Age Range**	**1990**	**2000**	**2010**	**2020**	**2023**
Puerto Rico	30 to 34	12.9 (4.2–28.3)	13.1 (4.1–28.5)	13.4 (4.4–28.6)	13.7 (4.7–30.8)	13.7 (4.8–30.5)
Puerto Rico	35 to 39	13.2 (4.3–28.9)	13.4 (4.2–29.1)	13.7 (4.5–29.1)	14.0 (4.8–31.4)	14.0 (4.9–31.1)
Puerto Rico	40 to 44	13.8 (4.6–30.0)	14.0 (4.4–30.2)	14.3 (4.7–30.3)	14.6 (5.1–32.6)	14.6 (5.1–32.2)
Puerto Rico	45 to 49	13.6 (4.5–29.5)	13.7 (4.3–29.8)	14.0 (4.6–29.8)	14.4 (4.9–32.1)	14.4 (5.0–31.7)
Puerto Rico	50 to 54	13.4 (4.4–29.1)	13.5 (4.2–29.4)	13.8 (4.5–29.4)	14.1 (4.9–31.7)	14.1 (4.9–31.2)
Puerto Rico	55 to 59	13.0 (4.2–28.4)	13.1 (4.1–28.7)	13.4 (4.4–28.7)	13.7 (4.7–30.9)	13.7 (4.8–30.6)
Puerto Rico	60 to 64	12.6 (4.1–27.7)	12.7 (4.0–27.9)	13.0 (4.2–28.0)	13.3 (4.5–30.2)	13.3 (4.6–29.8)
Puerto Rico	65 to 69	11.8 (3.8–26.1)	11.9 (3.7–26.3)	12.2 (3.9–26.3)	12.4 (4.2–28.5)	12.4 (4.3–28.1)
Puerto Rico	70 to 74	9.4 (2.9–21.4)	9.5 (2.8–21.6)	9.7 (3.0–21.6)	9.9 (3.3–23.5)	9.9 (3.3–23.2)
Puerto Rico	75 to 79	8.5 (2.6–19.5)	8.5 (2.5–19.6)	8.7 (2.7–19.6)	8.9 (2.9–21.4)	9.0 (3.0–21.1)
Puerto Rico	80 to 84	6.0 (1.8–14.1)	6.0 (1.7–14.2)	6.2 (1.9–14.2)	6.3 (2.0–15.6)	6.3 (2.0–15.4)
Puerto Rico	85 to 89	5.4 (1.6–12.8)	5.4 (1.6–12.9)	5.5 (1.7–12.9)	5.7 (1.8–14.1)	5.7 (1.8–14.0)
Puerto Rico	90 to 94	4.8 (1.4–11.6)	4.9 (1.4–11.7)	5.0 (1.5–11.7)	5.1 (1.6–12.9)	5.1 (1.6–12.7)
Puerto Rico	95 plus	4.8 (1.4–11.5)	4.8 (1.4–11.6)	5.0 (1.5–11.6)	5.1 (1.6–12.8)	5.1 (1.6–12.6)
Puerto Rico	Age-standardized	12.2 (4.0–26.9)	12.4 (3.8–27.2)	12.7 (4.1–27.2)	12.9 (4.4–29.3)	12.9 (4.5–29.0)

Supplementary Table S10: Prevalence of female SVAC by age and location for 1990, 2000, 2010, 2020, and 2023						
**Location**	**Age Range**	**1990**	**2000**	**2010**	**2020**	**2023**
Puerto Rico	All age	12.2 (4.0–26.9)	12.3 (3.8–27.0)	12.4 (4.0–26.7)	12.3 (4.2–28.1)	12.2 (4.2–27.4)
Saint Kitts and Nevis	20 to 24	15.2 (5.1–32.5)	15.3 (4.9–32.6)	15.4 (5.1–32.3)	15.7 (5.5–34.5)	15.7 (5.6–34.1)
Saint Kitts and Nevis	25 to 29	15.5 (5.2–33.1)	15.6 (5.0–33.1)	15.7 (5.3–32.8)	16.0 (5.6–35.0)	16.0 (5.7–34.5)
Saint Kitts and Nevis	30 to 34	16.7 (5.7–35.0)	16.7 (5.5–35.1)	16.9 (5.7–34.8)	17.1 (6.1–37.1)	17.1 (6.2–36.7)
Saint Kitts and Nevis	35 to 39	16.8 (5.7–35.2)	16.8 (5.5–35.3)	17.0 (5.8–34.9)	17.2 (6.1–37.2)	17.2 (6.2–36.8)
Saint Kitts and Nevis	40 to 44	17.3 (5.9–36.1)	17.3 (5.7–36.1)	17.5 (6.0–35.7)	17.7 (6.3–38.1)	17.7 (6.4–37.7)
Saint Kitts and Nevis	45 to 49	16.8 (5.7–35.3)	16.9 (5.5–35.3)	17.0 (5.8–34.9)	17.2 (6.1–37.3)	17.2 (6.2–36.7)
Saint Kitts and Nevis	50 to 54	16.4 (5.6–34.6)	16.5 (5.4–34.7)	16.6 (5.6–34.3)	16.9 (6.0–36.6)	16.9 (6.1–36.2)
Saint Kitts and Nevis	55 to 59	15.9 (5.4–33.7)	15.9 (5.1–33.7)	16.1 (5.4–33.4)	16.3 (5.7–35.6)	16.3 (5.8–35.1)
Saint Kitts and Nevis	60 to 64	15.4 (5.2–32.9)	15.4 (5.0–32.9)	15.6 (5.2–32.5)	15.8 (5.5–34.7)	15.8 (5.6–34.4)
Saint Kitts and Nevis	65 to 69	14.4 (4.8–31.0)	14.4 (4.6–31.0)	14.5 (4.8–30.7)	14.7 (5.1–32.8)	14.7 (5.2–32.3)
Saint Kitts and Nevis	70 to 74	11.6 (3.7–25.7)	11.6 (3.6–25.7)	11.7 (3.7–25.4)	11.8 (4.0–27.3)	11.9 (4.0–27.0)
Saint Kitts and Nevis	75 to 79	10.4 (3.3–23.5)	10.5 (3.2–23.5)	10.5 (3.3–23.2)	10.7 (3.5–25.0)	10.7 (3.6–24.7)
Saint Kitts and Nevis	80 to 84	7.4 (2.3–17.3)	7.4 (2.2–17.3)	7.5 (2.3–17.0)	7.6 (2.4–18.5)	7.6 (2.5–18.2)
Saint Kitts and Nevis	85 to 89	6.7 (2.0–15.7)	6.7 (1.9–15.7)	6.7 (2.0–15.5)	6.8 (2.2–16.8)	6.9 (2.2–16.6)

Supplementary Table S10: Prevalence of female SVAC by age and location for 1990, 2000, 2010, 2020, and 2023						
**Location**	**Age Range**	**1990**	**2000**	**2010**	**2020**	**2023**
Saint Kitts and Nevis	90 to 94	6.0 (1.8–14.3)	6.0 (1.7–14.3)	6.1 (1.8–14.1)	6.2 (2.0–15.3)	6.2 (2.0–15.1)
Saint Kitts and Nevis	95 plus	6.0 (1.8–14.2)	6.0 (1.7–14.2)	6.0 (1.8–14.0)	6.1 (1.9–15.2)	6.1 (2.0–15.0)
Saint Kitts and Nevis	Age-standardized	15.5 (5.2–33.0)	15.6 (5.0–33.0)	15.7 (5.3–32.6)	15.9 (5.6–34.9)	15.9 (5.7–34.4)
Saint Kitts and Nevis	All age	15.2 (5.1–32.4)	15.6 (5.0–33.0)	15.9 (5.3–33.0)	16.2 (5.7–35.3)	16.1 (5.8–34.8)
Saint Lucia	20 to 24	15.2 (5.1–32.5)	15.3 (4.9–32.6)	15.4 (5.1–32.3)	15.7 (5.5–34.5)	15.7 (5.6–34.1)
Saint Lucia	25 to 29	15.5 (5.2–33.1)	15.6 (5.0–33.1)	15.7 (5.3–32.8)	16.0 (5.6–35.0)	16.0 (5.7–34.5)
Saint Lucia	30 to 34	16.7 (5.7–35.0)	16.7 (5.5–35.1)	16.9 (5.7–34.8)	17.1 (6.1–37.1)	17.1 (6.2–36.7)
Saint Lucia	35 to 39	16.8 (5.7–35.2)	16.8 (5.5–35.3)	17.0 (5.8–34.9)	17.2 (6.1–37.2)	17.2 (6.2–36.8)
Saint Lucia	40 to 44	17.3 (5.9–36.1)	17.3 (5.7–36.1)	17.5 (6.0–35.7)	17.7 (6.3–38.1)	17.7 (6.4–37.7)
Saint Lucia	45 to 49	16.8 (5.7–35.3)	16.9 (5.5–35.3)	17.0 (5.8–34.9)	17.2 (6.1–37.3)	17.2 (6.2–36.7)
Saint Lucia	50 to 54	16.4 (5.6–34.6)	16.5 (5.4–34.7)	16.6 (5.6–34.3)	16.9 (6.0–36.6)	16.9 (6.1–36.2)
Saint Lucia	55 to 59	15.9 (5.4–33.7)	15.9 (5.1–33.7)	16.1 (5.4–33.4)	16.3 (5.7–35.6)	16.3 (5.8–35.1)
Saint Lucia	60 to 64	15.4 (5.2–32.9)	15.4 (5.0–32.9)	15.6 (5.2–32.5)	15.8 (5.5–34.7)	15.8 (5.6–34.4)
Saint Lucia	65 to 69	14.4 (4.8–31.0)	14.4 (4.6–31.0)	14.5 (4.8–30.7)	14.7 (5.1–32.8)	14.7 (5.2–32.3)
Saint Lucia	70 to 74	11.6 (3.7–25.7)	11.6 (3.6–25.7)	11.7 (3.7–25.4)	11.8 (4.0–27.3)	11.9 (4.0–27.0)

Supplementary Table S10: Prevalence of female SVAC by age and location for 1990, 2000, 2010, 2020, and 2023						
**Location**	**Age Range**	**1990**	**2000**	**2010**	**2020**	**2023**
Saint Lucia	75 to 79	10.4 (3.3–23.5)	10.5 (3.2–23.5)	10.5 (3.3–23.2)	10.7 (3.5–25.0)	10.7 (3.6–24.7)
Saint Lucia	80 to 84	7.4 (2.3–17.3)	7.4 (2.2–17.3)	7.5 (2.3–17.0)	7.6 (2.4–18.5)	7.6 (2.5–18.2)
Saint Lucia	85 to 89	6.7 (2.0–15.7)	6.7 (1.9–15.7)	6.7 (2.0–15.5)	6.8 (2.2–16.8)	6.9 (2.2–16.6)
Saint Lucia	90 to 94	6.0 (1.8–14.3)	6.0 (1.7–14.3)	6.1 (1.8–14.1)	6.2 (2.0–15.3)	6.2 (2.0–15.1)
Saint Lucia	95 plus	6.0 (1.8–14.2)	6.0 (1.7–14.2)	6.0 (1.8–14.0)	6.1 (1.9–15.2)	6.1 (2.0–15.0)
Saint Lucia	Age-standardized	15.5 (5.2–33.0)	15.6 (5.0–33.0)	15.7 (5.3–32.6)	15.9 (5.6–34.9)	15.9 (5.7–34.4)
Saint Lucia	All age	15.5 (5.2–33.0)	15.7 (5.1–33.2)	15.8 (5.3–32.8)	16.0 (5.6–34.9)	15.9 (5.7–34.4)
Saint Vincent and the Grenadines	20 to 24	15.2 (5.1–32.5)	15.3 (4.9–32.6)	15.4 (5.1–32.3)	15.7 (5.5–34.5)	15.7 (5.6–34.1)
Saint Vincent and the Grenadines	25 to 29	15.5 (5.2–33.1)	15.6 (5.0–33.1)	15.7 (5.3–32.8)	16.0 (5.6–35.0)	16.0 (5.7–34.5)
Saint Vincent and the Grenadines	30 to 34	16.7 (5.7–35.0)	16.7 (5.5–35.1)	16.9 (5.7–34.8)	17.1 (6.1–37.1)	17.1 (6.2–36.7)
Saint Vincent and the Grenadines	35 to 39	16.8 (5.7–35.2)	16.8 (5.5–35.3)	17.0 (5.8–34.9)	17.2 (6.1–37.2)	17.2 (6.2–36.8)
Saint Vincent and the Grenadines	40 to 44	17.3 (5.9–36.1)	17.3 (5.7–36.1)	17.5 (6.0–35.7)	17.7 (6.3–38.1)	17.7 (6.4–37.7)
Saint Vincent and the Grenadines	45 to 49	16.8 (5.7–35.3)	16.9 (5.5–35.3)	17.0 (5.8–34.9)	17.2 (6.1–37.3)	17.2 (6.2–36.7)
Saint Vincent and the Grenadines	50 to 54	16.4 (5.6–34.6)	16.5 (5.4–34.7)	16.6 (5.6–34.3)	16.9 (6.0–36.6)	16.9 (6.1–36.2)
Saint Vincent and the Grenadines	55 to 59	15.9 (5.4–33.7)	15.9 (5.1–33.7)	16.1 (5.4–33.4)	16.3 (5.7–35.6)	16.3 (5.8–35.1)

Supplementary Table S10: Prevalence of female SVAC by age and location for 1990, 2000, 2010, 2020, and 2023						
**Location**	**Age Range**	**1990**	**2000**	**2010**	**2020**	**2023**
Saint Vincent and the Grenadines	60 to 64	15.4 (5.2–32.9)	15.4 (5.0–32.9)	15.6 (5.2–32.5)	15.8 (5.5–34.7)	15.8 (5.6–34.4)
Saint Vincent and the Grenadines	65 to 69	14.4 (4.8–31.0)	14.4 (4.6–31.0)	14.5 (4.8–30.7)	14.7 (5.1–32.8)	14.7 (5.2–32.3)
Saint Vincent and the Grenadines	70 to 74	11.6 (3.7–25.7)	11.6 (3.6–25.7)	11.7 (3.7–25.4)	11.8 (4.0–27.3)	11.9 (4.0–27.0)
Saint Vincent and the Grenadines	75 to 79	10.4 (3.3–23.5)	10.5 (3.2–23.5)	10.5 (3.3–23.2)	10.7 (3.5–25.0)	10.7 (3.6–24.7)
Saint Vincent and the Grenadines	80 to 84	7.4 (2.3–17.3)	7.4 (2.2–17.3)	7.5 (2.3–17.0)	7.6 (2.4–18.5)	7.6 (2.5–18.2)
Saint Vincent and the Grenadines	85 to 89	6.7 (2.0–15.7)	6.7 (1.9–15.7)	6.7 (2.0–15.5)	6.8 (2.2–16.8)	6.9 (2.2–16.6)
Saint Vincent and the Grenadines	90 to 94	6.0 (1.8–14.3)	6.0 (1.7–14.3)	6.1 (1.8–14.1)	6.2 (2.0–15.3)	6.2 (2.0–15.1)
Saint Vincent and the Grenadines	95 plus	6.0 (1.8–14.2)	6.0 (1.7–14.2)	6.0 (1.8–14.0)	6.1 (1.9–15.2)	6.1 (2.0–15.0)
Saint Vincent and the Grenadines	Age-standardized	15.5 (5.2–33.0)	15.6 (5.0–33.0)	15.7 (5.3–32.6)	15.9 (5.6–34.9)	15.9 (5.7–34.4)
Saint Vincent and the Grenadines	All age	15.5 (5.2–32.8)	15.6 (5.0–33.0)	15.7 (5.3–32.7)	15.9 (5.6–34.7)	15.8 (5.6–34.2)
Suriname	20 to 24	15.2 (5.1–32.5)	15.3 (4.9–32.6)	15.4 (5.1–32.3)	15.7 (5.5–34.5)	15.7 (5.6–34.1)
Suriname	25 to 29	15.5 (5.2–33.1)	15.6 (5.0–33.1)	15.7 (5.3–32.8)	16.0 (5.6–35.0)	16.0 (5.7–34.5)
Suriname	30 to 34	16.7 (5.7–35.0)	16.7 (5.5–35.1)	16.9 (5.7–34.8)	17.1 (6.1–37.1)	17.1 (6.2–36.7)
Suriname	35 to 39	16.8 (5.7–35.2)	16.8 (5.5–35.3)	17.0 (5.8–34.9)	17.2 (6.1–37.2)	17.2 (6.2–36.8)
Suriname	40 to 44	17.3 (5.9–36.1)	17.3 (5.7–36.1)	17.5 (6.0–35.7)	17.7 (6.3–38.1)	17.7 (6.4–37.7)

Supplementary Table S10: Prevalence of female SVAC by age and location for 1990, 2000, 2010, 2020, and 2023						
**Location**	**Age Range**	**1990**	**2000**	**2010**	**2020**	**2023**
Suriname	45 to 49	16.8 (5.7–35.3)	16.9 (5.5–35.3)	17.0 (5.8–34.9)	17.2 (6.1–37.3)	17.2 (6.2–36.7)
Suriname	50 to 54	16.4 (5.6–34.6)	16.5 (5.4–34.7)	16.6 (5.6–34.3)	16.9 (6.0–36.6)	16.9 (6.1–36.2)
Suriname	55 to 59	15.9 (5.4–33.7)	15.9 (5.1–33.7)	16.1 (5.4–33.4)	16.3 (5.7–35.6)	16.3 (5.8–35.1)
Suriname	60 to 64	15.4 (5.2–32.9)	15.4 (5.0–32.9)	15.6 (5.2–32.5)	15.8 (5.5–34.7)	15.8 (5.6–34.4)
Suriname	65 to 69	14.4 (4.8–31.0)	14.4 (4.6–31.0)	14.5 (4.8–30.7)	14.7 (5.1–32.8)	14.7 (5.2–32.3)
Suriname	70 to 74	11.6 (3.7–25.7)	11.6 (3.6–25.7)	11.7 (3.7–25.4)	11.8 (4.0–27.3)	11.9 (4.0–27.0)
Suriname	75 to 79	10.4 (3.3–23.5)	10.5 (3.2–23.5)	10.5 (3.3–23.2)	10.7 (3.5–25.0)	10.7 (3.6–24.7)
Suriname	80 to 84	7.4 (2.3–17.3)	7.4 (2.2–17.3)	7.5 (2.3–17.0)	7.6 (2.4–18.5)	7.6 (2.5–18.2)
Suriname	85 to 89	6.7 (2.0–15.7)	6.7 (1.9–15.7)	6.7 (2.0–15.5)	6.8 (2.2–16.8)	6.9 (2.2–16.6)
Suriname	90 to 94	6.0 (1.8–14.3)	6.0 (1.7–14.3)	6.1 (1.8–14.1)	6.2 (2.0–15.3)	6.2 (2.0–15.1)
Suriname	95 plus	6.0 (1.8–14.2)	6.0 (1.7–14.2)	6.0 (1.8–14.0)	6.1 (1.9–15.2)	6.1 (2.0–15.0)
Suriname	Age-standardized	15.5 (5.2–33.0)	15.6 (5.0–33.0)	15.7 (5.3–32.6)	15.9 (5.6–34.9)	15.9 (5.7–34.4)
Suriname	All age	15.6 (5.3–33.1)	15.8 (5.1–33.4)	15.9 (5.3–33.0)	16.0 (5.6–35.0)	16.0 (5.7–34.5)
Trinidad and Tobago	20 to 24	15.2 (5.1–32.5)	15.3 (4.9–32.6)	15.4 (5.1–32.3)	15.7 (5.5–34.5)	15.7 (5.6–34.1)
Trinidad and Tobago	25 to 29	15.5 (5.2–33.1)	15.6 (5.0–33.1)	15.7 (5.3–32.8)	16.0 (5.6–35.0)	16.0 (5.7–34.5)

Supplementary Table S10: Prevalence of female SVAC by age and location for 1990, 2000, 2010, 2020, and 2023						
**Location**	**Age Range**	**1990**	**2000**	**2010**	**2020**	**2023**
Trinidad and Tobago	30 to 34	16.7 (5.7–35.0)	16.7 (5.5–35.1)	16.9 (5.7–34.8)	17.1 (6.1–37.1)	17.1 (6.2–36.7)
Trinidad and Tobago	35 to 39	16.8 (5.7–35.2)	16.8 (5.5–35.3)	17.0 (5.8–34.9)	17.2 (6.1–37.2)	17.2 (6.2–36.8)
Trinidad and Tobago	40 to 44	17.3 (5.9–36.1)	17.3 (5.7–36.1)	17.5 (6.0–35.7)	17.7 (6.3–38.1)	17.7 (6.4–37.7)
Trinidad and Tobago	45 to 49	16.8 (5.7–35.3)	16.9 (5.5–35.3)	17.0 (5.8–34.9)	17.2 (6.1–37.3)	17.2 (6.2–36.7)
Trinidad and Tobago	50 to 54	16.4 (5.6–34.6)	16.5 (5.4–34.7)	16.6 (5.6–34.3)	16.9 (6.0–36.6)	16.9 (6.1–36.2)
Trinidad and Tobago	55 to 59	15.9 (5.4–33.7)	15.9 (5.1–33.7)	16.1 (5.4–33.4)	16.3 (5.7–35.6)	16.3 (5.8–35.1)
Trinidad and Tobago	60 to 64	15.4 (5.2–32.9)	15.4 (5.0–32.9)	15.6 (5.2–32.5)	15.8 (5.5–34.7)	15.8 (5.6–34.4)
Trinidad and Tobago	65 to 69	14.4 (4.8–31.0)	14.4 (4.6–31.0)	14.5 (4.8–30.7)	14.7 (5.1–32.8)	14.7 (5.2–32.3)
Trinidad and Tobago	70 to 74	11.6 (3.7–25.7)	11.6 (3.6–25.7)	11.7 (3.7–25.4)	11.8 (4.0–27.3)	11.9 (4.0–27.0)
Trinidad and Tobago	75 to 79	10.4 (3.3–23.5)	10.5 (3.2–23.5)	10.5 (3.3–23.2)	10.7 (3.5–25.0)	10.7 (3.6–24.7)
Trinidad and Tobago	80 to 84	7.4 (2.3–17.3)	7.4 (2.2–17.3)	7.5 (2.3–17.0)	7.6 (2.4–18.5)	7.6 (2.5–18.2)
Trinidad and Tobago	85 to 89	6.7 (2.0–15.7)	6.7 (1.9–15.7)	6.7 (2.0–15.5)	6.8 (2.2–16.8)	6.9 (2.2–16.6)
Trinidad and Tobago	90 to 94	6.0 (1.8–14.3)	6.0 (1.7–14.3)	6.1 (1.8–14.1)	6.2 (2.0–15.3)	6.2 (2.0–15.1)
Trinidad and Tobago	95 plus	6.0 (1.8–14.2)	6.0 (1.7–14.2)	6.0 (1.8–14.0)	6.1 (1.9–15.2)	6.1 (2.0–15.0)
Trinidad and Tobago	Age-standardized	15.5 (5.2–33.0)	15.6 (5.0–33.0)	15.7 (5.3–32.6)	15.9 (5.6–34.9)	15.9 (5.7–34.4)

Supplementary Table S10: Prevalence of female SVAC by age and location for 1990, 2000, 2010, 2020, and 2023						
**Location**	**Age Range**	**1990**	**2000**	**2010**	**2020**	**2023**
Trinidad and Tobago	All age	15.7 (5.3–33.3)	15.8 (5.1–33.4)	15.8 (5.3–32.9)	15.9 (5.6–34.8)	15.8 (5.7–34.2)
Virgin Islands	20 to 24	17.9 (6.2–37.1)	18.0 (5.9–37.2)	18.2 (6.3–37.0)	18.6 (6.7–39.5)	18.5 (6.8–39.0)
Virgin Islands	25 to 29	18.0 (6.2–37.3)	18.1 (6.0–37.4)	18.3 (6.3–37.2)	18.7 (6.8–39.7)	18.7 (6.8–39.1)
Virgin Islands	30 to 34	19.1 (6.7–39.1)	19.2 (6.4–39.2)	19.5 (6.8–39.0)	19.8 (7.2–41.5)	19.8 (7.4–41.1)
Virgin Islands	35 to 39	19.1 (6.7–39.0)	19.2 (6.4–39.1)	19.4 (6.8–38.9)	19.8 (7.2–41.4)	19.8 (7.3–41.0)
Virgin Islands	40 to 44	19.5 (6.9–39.7)	19.6 (6.6–39.8)	19.9 (6.9–39.6)	20.2 (7.4–42.1)	20.2 (7.5–41.7)
Virgin Islands	45 to 49	18.9 (6.6–38.8)	19.0 (6.4–38.9)	19.2 (6.7–38.6)	19.6 (7.1–41.1)	19.6 (7.2–40.7)
Virgin Islands	50 to 54	18.5 (6.4–38.0)	18.5 (6.2–38.1)	18.7 (6.5–37.8)	19.1 (6.9–40.3)	19.1 (7.0–39.9)
Virgin Islands	55 to 59	17.8 (6.1–37.0)	17.9 (5.9–37.1)	18.1 (6.2–36.8)	18.4 (6.6–39.2)	18.4 (6.7–38.6)
Virgin Islands	60 to 64	17.3 (5.9–36.1)	17.3 (5.7–36.1)	17.5 (6.0–35.8)	17.8 (6.4–38.3)	17.8 (6.5–37.9)
Virgin Islands	65 to 69	16.1 (5.5–34.1)	16.2 (5.2–34.1)	16.4 (5.5–33.9)	16.7 (5.9–36.2)	16.6 (6.0–35.8)
Virgin Islands	70 to 74	13.0 (4.2–28.5)	13.0 (4.1–28.5)	13.2 (4.3–28.3)	13.4 (4.6–30.4)	13.4 (4.7–30.0)
Virgin Islands	75 to 79	11.8 (3.8–26.1)	11.8 (3.6–26.1)	11.9 (3.8–25.9)	12.2 (4.1–27.9)	12.2 (4.2–27.6)
Virgin Islands	80 to 84	8.4 (2.6–19.3)	8.4 (2.5–19.4)	8.5 (2.6–19.2)	8.7 (2.8–20.9)	8.7 (2.9–20.6)
Virgin Islands	85 to 89	7.6 (2.3–17.6)	7.6 (2.2–17.6)	7.7 (2.4–17.5)	7.8 (2.5–19.0)	7.8 (2.6–18.7)

Supplementary Table S10: Prevalence of female SVAC by age and location for 1990, 2000, 2010, 2020, and 2023						
**Location**	**Age Range**	**1990**	**2000**	**2010**	**2020**	**2023**
Virgin Islands	90 to 94	6.9 (2.1–16.1)	6.9 (2.0–16.1)	7.0 (2.1–15.9)	7.1 (2.3–17.4)	7.1 (2.3–17.1)
Virgin Islands	95 plus	6.8 (2.1–15.9)	6.8 (2.0–16.0)	6.9 (2.1–15.8)	7.0 (2.2–17.2)	7.0 (2.3–17.0)
Virgin Islands	Age-standardized	17.7 (6.1–36.6)	17.8 (5.9–36.7)	18.0 (6.2–36.5)	18.3 (6.6–38.9)	18.3 (6.7–38.5)
Virgin Islands	All age	18.0 (6.2–37.2)	17.9 (5.9–37.0)	17.8 (6.1–36.1)	17.3 (6.2–37.1)	17.1 (6.2–36.3)
Central Latin America	20 to 24	17.3 (8.3–31.0)	17.1 (11.5–24.6)	17.2 (14.0–20.9)	17.4 (14.4–21.5)	17.4 (13.8–22.5)
Central Latin America	25 to 29	17.8 (8.4–32.7)	17.9 (11.2–26.8)	17.9 (14.1–23.0)	18.2 (14.3–23.5)	18.3 (13.7–24.1)
Central Latin America	30 to 34	18.9 (8.8–34.5)	19.0 (12.0–28.6)	18.9 (14.7–24.2)	19.0 (14.8–24.5)	19.0 (14.2–25.3)
Central Latin America	35 to 39	19.0 (8.7–34.9)	19.3 (12.2–29.3)	19.3 (15.2–24.5)	19.4 (14.8–25.0)	19.4 (14.3–25.9)
Central Latin America	40 to 44	19.1 (8.6–35.2)	19.2 (11.9–29.4)	19.3 (15.1–24.7)	19.3 (14.9–24.8)	19.3 (14.3–25.6)
Central Latin America	45 to 49	19.2 (8.6–35.3)	19.3 (12.0–29.5)	19.5 (15.2–25.4)	19.6 (15.2–25.1)	19.6 (14.5–26.0)
Central Latin America	50 to 54	18.6 (8.1–35.1)	18.8 (11.4–29.2)	19.0 (14.7–24.9)	19.3 (14.8–25.0)	19.3 (14.2–25.7)
Central Latin America	55 to 59	17.2 (7.1–33.1)	17.0 (9.8–27.3)	17.2 (12.9–23.2)	17.4 (13.6–22.8)	17.4 (12.8–23.4)
Central Latin America	60 to 64	16.7 (6.9–32.7)	16.7 (9.3–27.2)	16.8 (12.4–22.9)	17.0 (13.1–22.6)	17.0 (12.4–23.4)
Central Latin America	65 to 69	15.2 (6.1–30.2)	15.0 (7.9–25.7)	15.1 (11.0–21.2)	15.3 (11.7–20.9)	15.4 (11.0–21.3)
Central Latin America	70 to 74	12.1 (4.6–24.7)	12.0 (5.7–21.8)	12.1 (7.8–18.8)	12.2 (9.2–18.0)	12.3 (9.2–18.2)

Supplementary Table S10: Prevalence of female SVAC by age and location for 1990, 2000, 2010, 2020, and 2023						
**Location**	**Age Range**	**1990**	**2000**	**2010**	**2020**	**2023**
Central Latin America	75 to 79	11.0 (4.1–22.9)	10.9 (4.5–21.3)	11.0 (4.7–21.6)	11.2 (4.6–21.7)	11.2 (4.8–21.9)
Central Latin America	80 to 84	7.2 (2.3–16.3)	7.1 (2.2–16.1)	7.2 (2.4–16.3)	7.3 (2.3–16.5)	7.3 (2.5–16.3)
Central Latin America	85 to 89	6.3 (2.0–14.5)	6.3 (1.9–14.3)	6.4 (2.1–14.5)	6.5 (2.1–14.7)	6.6 (2.2–14.8)
Central Latin America	90 to 94	5.6 (1.7–12.8)	5.6 (1.7–12.8)	5.8 (1.9–13.2)	5.9 (1.9–13.7)	6.0 (2.0–13.7)
Central Latin America	95 plus	6.6 (2.0–15.3)	6.6 (2.0–15.3)	6.3 (2.0–14.6)	6.6 (2.1–15.3)	6.7 (2.3–15.7)
Central Latin America	Age-standardized	17.3 (7.7–32.5)	17.4 (10.6–27.0)	17.5 (13.5–22.9)	17.6 (13.9–22.7)	17.7 (13.3–23.3)
Central Latin America	All age	17.7 (8.1–33.0)	17.7 (11.0–27.1)	17.7 (13.8–23.0)	17.7 (13.9–22.8)	17.7 (13.3–23.4)
Colombia	20 to 24	19.3 (6.9–38.5)	19.3 (7.6–36.8)	19.0 (9.9–32.3)	19.0 (12.0–28.0)	19.1 (11.2–30.2)
Colombia	25 to 29	19.4 (6.8–39.6)	19.5 (6.5–39.6)	19.7 (6.9–39.3)	20.0 (7.4–41.9)	20.0 (7.4–41.5)
Colombia	30 to 34	20.6 (7.3–41.4)	20.6 (7.0–41.5)	20.8 (7.4–41.1)	21.2 (7.9–43.7)	21.2 (8.0–43.3)
Colombia	35 to 39	20.6 (7.3–41.4)	20.6 (7.0–41.4)	20.8 (7.4–41.1)	21.2 (7.9–43.7)	21.2 (8.0–43.2)
Colombia	40 to 44	21.1 (7.5–42.2)	21.1 (7.2–42.2)	21.3 (7.6–41.8)	21.6 (8.1–44.4)	21.6 (8.2–43.9)
Colombia	45 to 49	20.5 (7.3–41.3)	20.5 (7.0–41.3)	20.7 (7.3–40.9)	21.0 (7.8–43.4)	21.0 (7.9–43.0)
Colombia	50 to 54	20.0 (7.1–40.6)	20.1 (6.8–40.6)	20.2 (7.1–40.2)	20.5 (7.6–42.7)	20.5 (7.7–42.2)
Colombia	55 to 59	19.4 (6.8–39.6)	19.5 (6.5–39.6)	19.6 (6.8–39.2)	19.9 (7.3–41.7)	19.9 (7.4–41.2)

Supplementary Table S10: Prevalence of female SVAC by age and location for 1990, 2000, 2010, 2020, and 2023						
**Location**	**Age Range**	**1990**	**2000**	**2010**	**2020**	**2023**
Colombia	60 to 64	18.8 (6.6–38.7)	18.9 (6.3–38.7)	19.0 (6.6–38.3)	19.3 (7.0–40.7)	19.3 (7.1–40.3)
Colombia	65 to 69	17.6 (6.1–36.6)	17.6 (5.8–36.6)	17.8 (6.1–36.2)	18.1 (6.5–38.6)	18.0 (6.6–38.0)
Colombia	70 to 74	14.3 (4.7–30.8)	14.3 (4.5–30.8)	14.4 (4.7–30.4)	14.6 (5.1–32.6)	14.6 (5.1–32.2)
Colombia	75 to 79	12.9 (4.2–28.2)	12.9 (4.0–28.2)	13.0 (4.2–27.9)	13.2 (4.5–30.0)	13.2 (4.6–29.6)
Colombia	80 to 84	9.2 (2.9–21.0)	9.2 (2.8–21.0)	9.3 (2.9–20.8)	9.5 (3.1–22.5)	9.5 (3.1–22.2)
Colombia	85 to 89	8.3 (2.6–19.1)	8.3 (2.5–19.1)	8.4 (2.6–18.9)	8.5 (2.8–20.5)	8.5 (2.8–20.2)
Colombia	90 to 94	7.5 (2.3–17.5)	7.5 (2.2–17.5)	7.6 (2.3–17.2)	7.7 (2.5–18.8)	7.7 (2.5–18.5)
Colombia	95 plus	7.4 (2.3–17.3)	7.4 (2.2–17.3)	7.5 (2.3–17.1)	7.7 (2.5–18.6)	7.7 (2.5–18.3)
Colombia	Age-standardized	19.1 (6.8–38.7)	19.2 (6.8–38.8)	19.3 (7.2–37.3)	19.6 (8.1–39.1)	19.6 (8.2–38.6)
Colombia	All age	19.5 (7.0–39.4)	19.5 (7.0–39.2)	19.4 (7.3–37.5)	19.5 (8.1–39.0)	19.5 (8.1–38.6)
Costa Rica	20 to 24	33.4 (20.0–49.6)	30.6 (23.7–38.2)	29.0 (18.4–41.2)	29.2 (13.5–50.7)	29.2 (12.4–54.0)
Costa Rica	25 to 29	33.4 (19.2–50.6)	31.1 (24.4–38.5)	29.4 (20.0–39.7)	29.5 (14.1–49.3)	29.7 (12.9–51.3)
Costa Rica	30 to 34	34.8 (19.3–52.7)	33.5 (26.5–41.1)	31.4 (22.9–40.2)	30.7 (17.1–48.2)	30.7 (15.6–50.7)
Costa Rica	35 to 39	36.5 (19.7–55.5)	37.0 (29.3–45.0)	34.6 (25.3–44.2)	31.6 (19.7–46.2)	31.1 (18.1–47.6)
Costa Rica	40 to 44	34.3 (18.3–53.9)	34.6 (26.9–42.7)	35.5 (23.8–47.5)	35.3 (17.1–57.2)	35.2 (15.7–58.7)

Supplementary Table S10: Prevalence of female SVAC by age and location for 1990, 2000, 2010, 2020, and 2023						
**Location**	**Age Range**	**1990**	**2000**	**2010**	**2020**	**2023**
Costa Rica	45 to 49	33.6 (17.9–52.6)	34.0 (26.3–42.2)	35.5 (24.6–46.5)	35.8 (19.1–55.8)	35.8 (17.3–58.4)
Costa Rica	50 to 54	33.0 (17.3–51.9)	33.3 (25.7–41.4)	34.9 (24.3–45.9)	36.0 (20.4–54.7)	36.1 (19.3–56.4)
Costa Rica	55 to 59	31.8 (16.3–51.6)	31.2 (23.2–40.0)	31.3 (19.6–43.8)	31.6 (14.5–53.8)	31.7 (13.2–56.4)
Costa Rica	60 to 64	31.0 (15.7–50.9)	30.5 (22.2–39.8)	30.5 (18.8–43.1)	30.8 (14.0–52.7)	30.9 (13.1–55.1)
Costa Rica	65 to 69	28.9 (14.2–48.6)	28.1 (19.5–38.2)	28.2 (17.0–40.7)	28.8 (12.8–50.9)	28.9 (12.1–53.1)
Costa Rica	70 to 74	24.4 (10.5–44.1)	24.0 (11.4–40.8)	24.1 (11.1–41.4)	24.6 (10.0–46.9)	24.8 (9.6–48.9)
Costa Rica	75 to 79	22.7 (8.3–44.7)	22.7 (7.9–44.6)	22.7 (8.2–43.9)	22.8 (8.5–46.8)	22.9 (8.2–46.5)
Costa Rica	80 to 84	16.9 (5.8–35.4)	16.9 (5.5–35.3)	16.9 (5.7–34.7)	17.0 (6.0–37.4)	17.1 (5.7–37.4)
Costa Rica	85 to 89	15.4 (5.2–32.8)	15.4 (4.9–32.7)	15.4 (5.1–32.1)	15.5 (5.3–34.8)	15.6 (5.1–34.6)
Costa Rica	90 to 94	14.1 (4.7–30.4)	14.0 (4.4–30.4)	14.0 (4.6–29.8)	14.2 (4.8–32.3)	14.2 (4.6–32.4)
Costa Rica	95 plus	14.0 (4.6–30.2)	13.9 (4.4–30.1)	13.9 (4.6–29.6)	14.0 (4.8–32.1)	14.1 (4.6–32.3)
Costa Rica	Age-standardized	32.3 (17.9–50.1)	31.6 (23.8–40.3)	31.1 (23.3–39.7)	30.9 (19.3–46.7)	30.9 (17.9–48.2)
Costa Rica	All age	33.0 (18.7–50.5)	32.1 (24.6–40.3)	31.3 (23.1–40.4)	30.9 (19.2–46.9)	30.9 (18.0–48.3)
El Salvador	20 to 24	13.6 (10.8–16.8)	13.4 (11.7–15.3)	13.8 (11.2–16.7)	18.7 (13.1–25.3)	19.5 (12.2–28.7)
El Salvador	25 to 29	13.6 (10.7–17.1)	13.8 (12.1–15.7)	12.7 (10.5–15.4)	14.9 (10.1–20.8)	15.9 (10.0–23.5)

Supplementary Table S10: Prevalence of female SVAC by age and location for 1990, 2000, 2010, 2020, and 2023						
**Location**	**Age Range**	**1990**	**2000**	**2010**	**2020**	**2023**
El Salvador	30 to 34	13.5 (9.9–17.7)	14.5 (12.7–16.5)	12.9 (10.9–15.1)	11.5 (7.6–15.9)	11.6 (6.7–17.8)
El Salvador	35 to 39	13.4 (8.7–19.5)	14.5 (12.6–16.5)	13.3 (11.2–15.7)	12.9 (8.9–17.2)	13.0 (7.9–19.3)
El Salvador	40 to 44	14.2 (7.6–23.3)	14.5 (12.4–16.8)	13.6 (11.1–16.2)	12.8 (10.0–16.4)	12.8 (9.0–17.7)
El Salvador	45 to 49	15.0 (7.4–25.2)	14.6 (12.2–17.1)	14.3 (11.6–17.0)	13.3 (10.1–17.7)	13.3 (9.1–19.2)
El Salvador	50 to 54	14.0 (5.2–27.9)	13.1 (6.7–22.2)	13.3 (9.7–17.5)	13.8 (10.1–18.3)	14.0 (9.2–20.3)
El Salvador	55 to 59	14.1 (5.0–29.9)	13.3 (5.7–24.8)	13.0 (8.4–19.2)	13.6 (9.9–18.1)	13.7 (9.2–19.4)
El Salvador	60 to 64	13.9 (4.7–29.4)	13.3 (5.0–26.8)	12.6 (6.4–21.9)	12.9 (8.3–18.7)	13.3 (7.9–20.7)
El Salvador	65 to 69	13.3 (4.4–28.6)	13.0 (4.6–27.2)	12.5 (5.2–24.0)	11.8 (7.4–18.9)	11.8 (7.4–18.2)
El Salvador	70 to 74	10.8 (3.4–24.2)	10.8 (3.3–24.1)	10.8 (3.4–23.7)	10.9 (3.6–25.4)	10.9 (3.7–25.0)
El Salvador	75 to 79	9.7 (3.1–22.1)	9.7 (2.9–22.0)	9.7 (3.0–21.6)	9.8 (3.2–23.1)	9.8 (3.3–22.8)
El Salvador	80 to 84	6.9 (2.1–16.1)	6.9 (2.0–16.1)	6.9 (2.1–15.7)	6.9 (2.2–17.0)	6.9 (2.2–16.7)
El Salvador	85 to 89	6.2 (1.9–14.6)	6.2 (1.8–14.6)	6.2 (1.9–14.3)	6.2 (2.0–15.4)	6.2 (2.0–15.2)
El Salvador	90 to 94	5.6 (1.7–13.3)	5.6 (1.6–13.3)	5.6 (1.7–13.0)	5.6 (1.8–14.0)	5.6 (1.8–13.8)
El Salvador	95 plus	5.6 (1.7–13.2)	5.5 (1.6–13.2)	5.5 (1.7–12.9)	5.6 (1.8–13.9)	5.6 (1.8–13.7)
El Salvador	Age-standardized	13.4 (8.3–21.0)	13.5 (10.3–17.7)	12.9 (10.7–15.7)	13.4 (11.0–16.7)	13.7 (10.8–17.6)

Supplementary Table S10: Prevalence of female SVAC by age and location for 1990, 2000, 2010, 2020, and 2023						
**Location**	**Age Range**	**1990**	**2000**	**2010**	**2020**	**2023**
El Salvador	All age	13.5 (9.0–19.9)	13.6 (11.0–17.0)	12.9 (10.9–15.5)	13.6 (11.0–16.9)	13.8 (10.7–17.7)
Guatemala	20 to 24	12.4 (9.7–15.7)	12.1 (10.1–14.3)	12.5 (8.9–16.8)	12.9 (5.8–23.4)	12.9 (5.4–24.9)
Guatemala	25 to 29	11.8 (9.1–15.1)	12.7 (10.6–14.9)	12.1 (9.5–15.5)	12.4 (6.5–20.6)	12.6 (5.8–22.7)
Guatemala	30 to 34	11.0 (7.9–14.8)	13.0 (10.9–15.6)	12.3 (10.1–14.9)	12.4 (8.0–17.2)	12.6 (7.3–19.4)
Guatemala	35 to 39	10.6 (6.5–15.8)	12.8 (10.5–15.3)	12.7 (10.5–15.4)	11.6 (7.9–15.7)	11.6 (7.0–17.4)
Guatemala	40 to 44	11.2 (5.7–19.1)	12.1 (9.6–14.8)	12.9 (10.4–15.5)	11.8 (8.9–15.4)	11.5 (7.8–16.4)
Guatemala	45 to 49	13.4 (6.3–23.3)	12.8 (9.6–16.4)	12.6 (10.1–15.4)	12.5 (9.2–16.4)	12.4 (8.5–17.6)
Guatemala	50 to 54	11.4 (4.0–23.5)	10.3 (5.0–18.1)	10.9 (7.8–14.6)	12.8 (9.2–17.0)	13.1 (8.7–19.2)
Guatemala	55 to 59	11.7 (4.0–25.5)	10.7 (4.4–20.6)	10.3 (6.3–15.7)	11.6 (8.2–15.8)	12.1 (7.9–17.8)
Guatemala	60 to 64	11.8 (3.9–25.6)	11.1 (4.0–23.0)	10.0 (4.9–18.0)	9.8 (6.4–14.6)	10.0 (6.1–15.6)
Guatemala	65 to 69	11.4 (3.7–25.0)	10.9 (3.7–23.7)	10.1 (4.0–20.5)	9.3 (5.4–15.7)	9.2 (5.5–15.4)
Guatemala	70 to 74	9.3 (2.9–21.2)	9.3 (2.8–21.2)	9.3 (2.9–20.7)	9.4 (3.1–22.3)	9.4 (3.1–22.0)
Guatemala	75 to 79	8.4 (2.6–19.3)	8.4 (2.5–19.3)	8.4 (2.6–18.8)	8.4 (2.7–20.2)	8.4 (2.8–20.0)
Guatemala	80 to 84	5.9 (1.8–14.0)	5.9 (1.7–13.9)	5.9 (1.8–13.6)	5.9 (1.9–14.7)	5.9 (1.9–14.5)
Guatemala	85 to 89	5.3 (1.6–12.6)	5.3 (1.5–12.6)	5.3 (1.6–12.3)	5.3 (1.7–13.3)	5.3 (1.7–13.1)

Supplementary Table S10: Prevalence of female SVAC by age and location for 1990, 2000, 2010, 2020, and 2023						
**Location**	**Age Range**	**1990**	**2000**	**2010**	**2020**	**2023**
Guatemala	90 to 94	4.8 (1.4–11.5)	4.8 (1.4–11.4)	4.8 (1.4–11.2)	4.8 (1.5–12.1)	4.8 (1.5–11.9)
Guatemala	95 plus	4.7 (1.4–11.4)	4.7 (1.3–11.4)	4.7 (1.4–11.1)	4.8 (1.5–12.0)	4.8 (1.5–11.8)
Guatemala	Age-standardized	11.3 (7.0–17.9)	11.6 (9.0–15.4)	11.5 (9.3–14.6)	11.6 (8.9–15.2)	11.6 (8.8–15.8)
Guatemala	All age	11.5 (8.0–16.9)	12.0 (9.8–14.8)	11.8 (9.8–14.4)	11.8 (9.0–15.7)	11.9 (8.7–16.2)
Honduras	20 to 24	19.4 (14.4–25.2)	18.5 (15.0–22.6)	17.0 (13.0–21.5)	16.9 (12.1–22.9)	17.0 (10.8–25.3)
Honduras	25 to 29	20.5 (14.8–27.4)	19.0 (15.3–23.1)	17.0 (13.1–21.6)	17.4 (12.9–23.1)	17.9 (12.5–24.8)
Honduras	30 to 34	21.7 (14.9–30.3)	20.1 (16.0–24.7)	17.9 (14.0–22.1)	16.0 (10.6–23.6)	15.9 (9.2–26.1)
Honduras	35 to 39	22.2 (14.0–32.8)	20.0 (15.5–25.1)	18.5 (14.5–23.0)	17.5 (12.2–24.9)	17.5 (10.6–27.9)
Honduras	40 to 44	22.7 (12.6–36.1)	20.6 (15.7–26.4)	19.1 (14.3–24.3)	19.3 (14.3–25.4)	19.7 (13.4–27.9)
Honduras	45 to 49	23.1 (11.7–38.9)	22.2 (16.5–28.5)	20.1 (14.6–26.3)	18.9 (13.9–24.9)	18.9 (12.8–26.7)
Honduras	50 to 54	22.4 (8.9–41.9)	22.6 (11.9–36.6)	21.6 (14.6–30.4)	21.1 (14.4–29.2)	21.2 (13.5–31.4)
Honduras	55 to 59	21.9 (8.3–42.3)	22.6 (10.4–40.1)	22.1 (13.6–33.3)	19.8 (12.9–28.3)	19.3 (11.8–29.8)
Honduras	60 to 64	20.9 (7.6–40.9)	21.8 (8.9–40.4)	22.6 (11.8–37.6)	22.1 (13.9–32.9)	21.8 (13.1–34.4)
Honduras	65 to 69	19.5 (7.0–39.5)	20.4 (7.5–40.0)	21.8 (9.7–39.1)	22.8 (12.4–37.2)	22.7 (12.2–36.0)
Honduras	70 to 74	15.1 (5.0–32.3)	15.0 (4.8–32.1)	15.0 (5.0–31.5)	15.1 (5.3–33.5)	15.1 (5.3–33.1)

Supplementary Table S10: Prevalence of female SVAC by age and location for 1990, 2000, 2010, 2020, and 2023						
**Location**	**Age Range**	**1990**	**2000**	**2010**	**2020**	**2023**
Honduras	75 to 79	13.7 (4.5–29.7)	13.6 (4.3–29.6)	13.6 (4.4–29.0)	13.7 (4.7–30.9)	13.7 (4.8–30.5)
Honduras	80 to 84	9.8 (3.1–22.3)	9.8 (2.9–22.2)	9.8 (3.1–21.7)	9.8 (3.2–23.3)	9.8 (3.3–23.0)
Honduras	85 to 89	8.9 (2.8–20.4)	8.8 (2.6–20.2)	8.8 (2.7–19.8)	8.9 (2.9–21.3)	8.9 (2.9–21.0)
Honduras	90 to 94	8.1 (2.5–18.6)	8.0 (2.4–18.5)	8.0 (2.5–18.1)	8.1 (2.6–19.5)	8.1 (2.6–19.2)
Honduras	95 plus	8.0 (2.5–18.5)	7.9 (2.3–18.4)	7.9 (2.4–18.0)	8.0 (2.6–19.3)	8.0 (2.6–19.1)
Honduras	Age-standardized	20.6 (11.7–33.1)	19.8 (14.4–26.9)	18.6 (15.2–23.1)	18.0 (15.0–21.7)	18.1 (14.1–23.2)
Honduras	All age	20.8 (12.8–31.7)	19.8 (15.4–25.2)	18.4 (15.7–21.7)	18.0 (15.1–21.4)	18.1 (14.0–23.2)
Mexico	20 to 24	16.7 (7.6–30.6)	16.8 (11.6–23.3)	17.0 (10.2–25.2)	17.2 (8.8–28.3)	17.3 (8.6–28.9)
Mexico	25 to 29	17.6 (8.1–31.7)	17.8 (12.7–24.4)	18.1 (11.2–26.6)	18.3 (9.6–29.8)	18.4 (9.4–30.4)
Mexico	30 to 34	18.7 (8.7–33.1)	18.9 (13.5–25.6)	19.1 (12.0–27.9)	19.4 (10.3–30.8)	19.4 (10.1–31.4)
Mexico	35 to 39	19.0 (8.8–33.6)	19.3 (13.9–26.0)	19.6 (12.4–28.1)	19.8 (10.4–31.6)	19.9 (10.1–32.4)
Mexico	40 to 44	18.8 (8.7–33.4)	18.8 (13.4–25.4)	19.0 (12.0–27.6)	19.3 (10.3–31.0)	19.3 (10.1–31.4)
Mexico	45 to 49	19.1 (8.9–34.0)	19.4 (13.9–26.2)	19.7 (12.5–28.2)	20.0 (10.6–31.2)	20.0 (10.4–32.0)
Mexico	50 to 54	18.5 (8.6–33.1)	18.8 (13.4–25.5)	19.1 (12.0–27.8)	19.3 (10.1–30.8)	19.4 (9.9–31.5)
Mexico	55 to 59	16.2 (7.3–29.7)	15.9 (10.8–22.6)	16.1 (9.5–24.1)	16.3 (8.2–27.0)	16.3 (8.0–27.3)

Supplementary Table S10: Prevalence of female SVAC by age and location for 1990, 2000, 2010, 2020, and 2023						
**Location**	**Age Range**	**1990**	**2000**	**2010**	**2020**	**2023**
Mexico	60 to 64	15.8 (7.1–29.1)	15.7 (10.5–22.4)	15.8 (9.3–23.7)	16.0 (7.9–26.7)	16.1 (7.8–27.0)
Mexico	65 to 69	13.8 (6.0–26.2)	13.4 (8.3–20.4)	13.5 (7.3–21.3)	13.7 (6.3–24.5)	13.7 (6.2–24.8)
Mexico	70 to 74	10.8 (4.5–21.2)	10.4 (5.9–16.9)	10.5 (5.2–18.2)	10.6 (4.8–19.6)	10.6 (4.7–20.1)
Mexico	75 to 79	9.7 (3.9–19.7)	9.6 (5.2–16.1)	9.8 (4.6–17.7)	9.9 (4.1–19.5)	10.0 (4.0–19.5)
Mexico	80 to 84	5.7 (1.9–12.9)	5.4 (1.8–12.0)	5.5 (1.8–12.3)	5.6 (1.7–13.7)	5.6 (1.6–13.9)
Mexico	85 to 89	5.1 (1.7–11.6)	4.9 (1.6–10.8)	4.9 (1.6–11.2)	5.0 (1.5–12.2)	5.0 (1.5–12.4)
Mexico	90 to 94	4.6 (1.5–10.5)	4.4 (1.4– 9.8)	4.5 (1.4–10.1)	4.6 (1.3–11.3)	4.6 (1.3–11.6)
Mexico	95 plus	4.5 (1.5–10.3)	4.3 (1.4– 9.7)	4.4 (1.4–10.0)	4.5 (1.3–11.0)	4.5 (1.3–11.3)
Mexico	Age-standardized	16.9 (7.7–30.5)	16.9 (11.9–23.3)	17.1 (10.8–24.8)	17.4 (9.5–27.4)	17.4 (9.2–27.7)
Mexico	All age	17.3 (7.9–31.2)	17.4 (12.3–23.9)	17.5 (11.0–25.3)	17.5 (9.5–27.6)	17.5 (9.3–27.9)
Nicaragua	20 to 24	16.5 (12.0–22.2)	18.0 (13.2–23.5)	18.6 (12.1–26.9)	17.4 (7.9–31.9)	17.1 (7.2–33.5)
Nicaragua	25 to 29	16.4 (11.6–22.5)	17.1 (12.6–22.6)	18.4 (13.3–24.2)	18.0 (9.7–29.8)	17.7 (8.6–31.8)
Nicaragua	30 to 34	17.0 (11.5–23.9)	16.9 (12.5–22.2)	17.8 (13.2–23.3)	18.8 (12.4–27.6)	18.6 (10.7–30.2)
Nicaragua	35 to 39	17.1 (10.7–25.4)	16.4 (11.8–21.8)	17.0 (12.2–22.4)	19.7 (14.2–26.6)	20.5 (13.8–29.0)
Nicaragua	40 to 44	17.6 (9.7–28.5)	16.8 (11.6–22.9)	16.4 (11.7–21.7)	16.0 (10.4–23.3)	15.9 (9.6–24.8)

Supplementary Table S10: Prevalence of female SVAC by age and location for 1990, 2000, 2010, 2020, and 2023						
**Location**	**Age Range**	**1990**	**2000**	**2010**	**2020**	**2023**
Nicaragua	45 to 49	17.8 (8.6–31.1)	17.0 (11.0–24.5)	16.6 (11.6–22.4)	16.8 (11.5–23.3)	16.9 (10.8–25.3)
Nicaragua	50 to 54	18.6 (7.7–34.5)	18.7 (10.7–28.8)	17.8 (12.3–24.3)	16.9 (11.6–23.3)	16.9 (10.6–25.2)
Nicaragua	55 to 59	16.1 (6.0–32.6)	15.9 (7.7–27.5)	16.2 (10.2–24.2)	17.0 (11.3–23.8)	17.2 (10.8–25.8)
Nicaragua	60 to 64	15.4 (5.5–31.4)	15.2 (6.7–28.5)	15.9 (8.8–25.7)	16.4 (9.9–24.9)	16.4 (9.6–26.6)
Nicaragua	65 to 69	15.6 (5.5–33.1)	16.2 (5.9–32.2)	16.9 (7.7–30.3)	17.2 (9.5–27.9)	17.1 (9.3–28.1)
Nicaragua	70 to 74	12.7 (4.2–28.1)	13.3 (4.5–28.6)	14.2 (5.6–28.2)	15.1 (7.2–27.8)	15.1 (7.1–27.2)
Nicaragua	75 to 79	10.9 (3.5–24.4)	10.9 (3.3–24.3)	10.9 (3.4–23.9)	11.0 (3.7–25.6)	11.0 (3.7–25.3)
Nicaragua	80 to 84	7.8 (2.4–18.0)	7.7 (2.3–17.9)	7.7 (2.4–17.6)	7.8 (2.5–18.9)	7.8 (2.6–18.7)
Nicaragua	85 to 89	7.0 (2.1–16.3)	7.0 (2.0–16.3)	7.0 (2.1–16.0)	7.0 (2.2–17.2)	7.1 (2.3–17.0)
Nicaragua	90 to 94	6.3 (1.9–14.9)	6.3 (1.8–14.8)	6.3 (1.9–14.5)	6.4 (2.0–15.7)	6.4 (2.0–15.5)
Nicaragua	95 plus	6.3 (1.9–14.8)	6.2 (1.8–14.7)	6.2 (1.9–14.4)	6.3 (2.0–15.6)	6.3 (2.0–15.4)
Nicaragua	Age-standardized	16.3 (10.0–25.6)	16.3 (11.2–22.6)	16.7 (13.3–20.6)	17.0 (12.6–21.9)	17.0 (11.8–23.8)
Nicaragua	All age	16.5 (11.2–24.0)	16.7 (12.3–22.2)	17.2 (13.8–21.0)	17.3 (13.0–22.5)	17.2 (11.8–23.9)
Panama	20 to 24	14.6 (4.9–31.5)	14.6 (4.6–31.3)	14.5 (4.8–30.6)	14.6 (5.1–32.6)	14.6 (5.1–32.2)
Panama	25 to 29	15.1 (5.1–32.4)	15.1 (4.8–32.2)	15.0 (5.0–31.5)	15.1 (5.3–33.5)	15.1 (5.3–33.2)

Supplementary Table S10: Prevalence of female SVAC by age and location for 1990, 2000, 2010, 2020, and 2023						
**Location**	**Age Range**	**1990**	**2000**	**2010**	**2020**	**2023**
Panama	30 to 34	16.4 (5.6–34.6)	16.4 (5.3–34.5)	16.3 (5.5–33.7)	16.4 (5.8–35.9)	16.4 (5.9–35.5)
Panama	35 to 39	16.7 (5.7–35.1)	16.6 (5.4–34.9)	16.6 (5.6–34.2)	16.7 (5.9–36.3)	16.7 (6.0–35.8)
Panama	40 to 44	17.3 (5.9–36.1)	17.3 (5.7–36.0)	17.2 (5.8–35.2)	17.3 (6.2–37.4)	17.3 (6.3–37.0)
Panama	45 to 49	17.0 (5.8–35.5)	16.9 (5.5–35.4)	16.8 (5.7–34.7)	17.0 (6.0–36.8)	17.0 (6.1–36.4)
Panama	50 to 54	16.7 (5.7–35.0)	16.6 (5.4–34.9)	16.5 (5.6–34.2)	16.7 (5.9–36.3)	16.7 (6.0–35.9)
Panama	55 to 59	16.2 (5.5–34.2)	16.2 (5.2–34.1)	16.1 (5.4–33.4)	16.2 (5.7–35.5)	16.2 (5.8–34.9)
Panama	60 to 64	15.7 (5.3–33.4)	15.7 (5.1–33.3)	15.6 (5.2–32.6)	15.8 (5.5–34.7)	15.8 (5.6–34.1)
Panama	65 to 69	14.7 (4.9–31.5)	14.7 (4.7–31.5)	14.6 (4.8–30.8)	14.7 (5.1–32.8)	14.7 (5.2–32.4)
Panama	70 to 74	11.8 (3.8–26.2)	11.8 (3.6–26.1)	11.7 (3.7–25.5)	11.8 (4.0–27.3)	11.8 (4.0–26.9)
Panama	75 to 79	10.6 (3.4–23.9)	10.6 (3.2–23.8)	10.5 (3.3–23.2)	10.6 (3.5–24.9)	10.6 (3.6–24.6)
Panama	80 to 84	7.5 (2.3–17.5)	7.5 (2.2–17.5)	7.5 (2.3–17.0)	7.5 (2.4–18.4)	7.5 (2.5–18.1)
Panama	85 to 89	6.8 (2.1–15.9)	6.8 (2.0–15.8)	6.7 (2.0–15.4)	6.8 (2.2–16.7)	6.8 (2.2–16.4)
Panama	90 to 94	6.1 (1.9–14.5)	6.1 (1.8–14.4)	6.1 (1.8–14.0)	6.1 (1.9–15.2)	6.1 (2.0–14.9)
Panama	95 plus	6.1 (1.8–14.3)	6.0 (1.7–14.3)	6.0 (1.8–13.9)	6.1 (1.9–15.0)	6.1 (1.9–14.8)
Panama	Age-standardized	15.5 (5.2–32.9)	15.4 (5.0–32.7)	15.4 (5.1–32.0)	15.5 (5.4–34.1)	15.5 (5.5–33.7)

Supplementary Table S10: Prevalence of female SVAC by age and location for 1990, 2000, 2010, 2020, and 2023						
**Location**	**Age Range**	**1990**	**2000**	**2010**	**2020**	**2023**
Panama	All age	15.6 (5.2–33.0)	15.5 (5.0–32.9)	15.4 (5.2–32.2)	15.5 (5.4–34.1)	15.5 (5.5–33.6)
Venezuela	20 to 24	16.3 (5.5–34.4)	16.3 (5.3–34.3)	16.3 (5.5–33.7)	16.4 (5.8–35.8)	16.4 (5.8–35.4)
Venezuela	25 to 29	16.6 (5.7–34.9)	16.6 (5.4–34.9)	16.6 (5.6–34.3)	16.7 (5.9–36.3)	16.7 (6.0–35.9)
Venezuela	30 to 34	17.8 (6.2–37.0)	17.8 (5.9–37.0)	17.8 (6.1–36.3)	17.9 (6.4–38.4)	17.9 (6.5–38.0)
Venezuela	35 to 39	18.0 (6.2–37.2)	18.0 (5.9–37.2)	18.0 (6.2–36.6)	18.1 (6.5–38.6)	18.1 (6.6–38.1)
Venezuela	40 to 44	18.5 (6.4–38.2)	18.5 (6.2–38.1)	18.5 (6.4–37.5)	18.6 (6.7–39.6)	18.6 (6.8–39.2)
Venezuela	45 to 49	18.1 (6.3–37.5)	18.1 (6.0–37.4)	18.1 (6.2–36.8)	18.2 (6.5–38.9)	18.2 (6.6–38.5)
Venezuela	50 to 54	17.8 (6.1–36.9)	17.8 (5.9–36.9)	17.8 (6.1–36.2)	17.8 (6.4–38.3)	17.8 (6.5–37.9)
Venezuela	55 to 59	17.3 (5.9–36.0)	17.3 (5.7–36.0)	17.2 (5.9–35.3)	17.3 (6.2–37.4)	17.3 (6.3–37.0)
Venezuela	60 to 64	16.8 (5.7–35.2)	16.8 (5.5–35.1)	16.7 (5.7–34.5)	16.8 (6.0–36.5)	16.8 (6.1–36.0)
Venezuela	65 to 69	15.7 (5.3–33.3)	15.6 (5.0–33.2)	15.6 (5.2–32.6)	15.7 (5.5–34.6)	15.7 (5.6–34.2)
Venezuela	70 to 74	12.6 (4.1–27.7)	12.6 (3.9–27.7)	12.6 (4.1–27.1)	12.7 (4.3–28.9)	12.7 (4.3–28.5)
Venezuela	75 to 79	11.4 (3.6–25.3)	11.3 (3.5–25.3)	11.3 (3.6–24.8)	11.4 (3.8–26.4)	11.4 (3.9–26.1)
Venezuela	80 to 84	8.1 (2.5–18.7)	8.1 (2.4–18.6)	8.1 (2.5–18.2)	8.1 (2.6–19.6)	8.1 (2.7–19.3)
Venezuela	85 to 89	7.3 (2.2–16.9)	7.2 (2.1–16.9)	7.2 (2.2–16.5)	7.3 (2.3–17.8)	7.3 (2.4–17.5)

Supplementary Table S10: Prevalence of female SVAC by age and location for 1990, 2000, 2010, 2020, and 2023						
**Location**	**Age Range**	**1990**	**2000**	**2010**	**2020**	**2023**
Venezuela	90 to 94	6.6 (2.0–15.4)	6.5 (1.9–15.4)	6.5 (2.0–15.0)	6.6 (2.1–16.2)	6.6 (2.1–16.0)
Venezuela	95 plus	6.5 (2.0–15.3)	6.5 (1.9–15.3)	6.5 (2.0–14.9)	6.5 (2.1–16.1)	6.5 (2.1–15.8)
Venezuela	Age-standardized	16.7 (5.7–35.0)	16.7 (5.5–35.0)	16.7 (5.7–34.3)	16.8 (6.0–36.4)	16.8 (6.0–35.9)
Venezuela	All age	17.0 (5.8–35.5)	17.0 (5.6–35.5)	17.0 (5.8–34.8)	17.0 (6.0–36.7)	16.9 (6.1–36.1)
Tropical Latin America	20 to 24	14.5 (8.6–23.2)	15.0 (11.0–19.9)	16.4 (11.8–21.5)	17.2 (9.5–29.0)	17.4 (8.9–30.7)
Tropical Latin America	25 to 29	14.6 (8.7–23.2)	15.6 (11.5–20.6)	18.0 (13.1–23.6)	19.1 (10.8–31.6)	19.3 (10.1–33.5)
Tropical Latin America	30 to 34	15.7 (9.4–24.6)	16.8 (12.5–21.7)	19.1 (14.7–24.2)	20.2 (12.0–32.2)	20.4 (11.0–33.9)
Tropical Latin America	35 to 39	15.0 (9.0–23.6)	17.1 (12.7–22.0)	21.8 (16.9–27.4)	23.7 (14.2–37.2)	24.0 (13.1–39.3)
Tropical Latin America	40 to 44	15.5 (9.2–24.5)	16.0 (11.8–21.0)	17.8 (12.6–23.5)	19.0 (10.5–31.8)	19.3 (9.9–33.9)
Tropical Latin America	45 to 49	16.5 (9.8–26.0)	16.7 (12.4–21.9)	17.5 (12.5–23.1)	18.1 (10.0–30.4)	18.3 (9.3–32.5)
Tropical Latin America	50 to 54	17.4 (6.2–36.1)	17.0 (7.9–29.8)	16.6 (11.7–22.7)	16.9 (9.4–28.3)	17.0 (8.7–30.2)
Tropical Latin America	55 to 59	15.6 (5.4–33.5)	14.7 (6.7–26.5)	13.9 (9.5–19.7)	14.4 (7.6–24.8)	14.6 (7.2–26.4)
Tropical Latin America	60 to 64	14.5 (4.7–30.8)	13.5 (5.9–25.0)	12.7 (7.7–19.1)	13.3 (6.3–23.1)	13.5 (5.9–24.8)
Tropical Latin America	65 to 69	13.0 (4.1–28.1)	12.0 (5.1–22.6)	11.1 (6.5–17.2)	11.7 (5.4–20.9)	11.9 (5.1–22.3)
Tropical Latin America	70 to 74	11.1 (3.4–24.6)	10.5 (4.3–20.2)	10.0 (5.4–16.5)	10.4 (4.6–19.3)	10.6 (4.4–20.5)

Supplementary Table S10: Prevalence of female SVAC by age and location for 1990, 2000, 2010, 2020, and 2023						
**Location**	**Age Range**	**1990**	**2000**	**2010**	**2020**	**2023**
Tropical Latin America	75 to 79	10.3 (3.1–23.0)	9.9 (4.0–19.1)	9.6 (5.2–15.8)	10.0 (4.4–18.4)	10.1 (4.2–19.7)
Tropical Latin America	80 to 84	6.9 (1.8–16.9)	6.5 (2.2–14.8)	6.3 (2.3–13.2)	6.6 (2.2–14.9)	6.7 (2.2–15.8)
Tropical Latin America	85 to 89	6.3 (1.7–15.3)	6.0 (2.0–13.7)	5.8 (2.1–12.2)	6.0 (2.0–13.8)	6.1 (2.0–14.6)
Tropical Latin America	90 to 94	5.7 (1.5–14.1)	5.5 (1.8–12.6)	5.3 (1.9–11.3)	5.6 (1.8–12.7)	5.6 (1.7–13.5)
Tropical Latin America	95 plus	5.6 (1.5–14.1)	5.4 (1.8–12.4)	5.3 (1.9–11.2)	5.5 (1.8–12.6)	5.6 (1.7–13.5)
Tropical Latin America	Age-standardized	14.7 (7.9–24.5)	15.1 (10.0–21.8)	16.4 (12.8–20.4)	17.3 (11.8–26.1)	17.4 (11.3–27.5)
Tropical Latin America	All age	14.9 (8.3–24.3)	15.4 (10.4–21.6)	16.6 (12.9–20.9)	17.1 (11.9–25.7)	17.2 (11.4–26.6)
Brazil	20 to 24	14.6 (8.6–23.5)	15.2 (11.0–20.2)	16.6 (11.9–22.0)	17.5 (9.6–29.7)	17.7 (8.9–31.5)
Brazil	25 to 29	14.7 (8.7–23.6)	15.8 (11.6–20.9)	18.3 (13.3–24.0)	19.5 (10.8–32.4)	19.7 (10.0–34.4)
Brazil	30 to 34	15.9 (9.5–24.9)	17.0 (12.6–22.0)	19.4 (14.8–24.5)	20.5 (12.0–33.1)	20.8 (11.0–35.0)
Brazil	35 to 39	15.1 (9.0–23.9)	17.3 (12.8–22.4)	22.1 (17.1–27.8)	24.2 (14.4–37.9)	24.5 (13.3–40.2)
Brazil	40 to 44	15.6 (9.3–24.7)	16.2 (12.0–21.3)	18.0 (12.8–23.8)	19.3 (10.5–32.4)	19.5 (9.9–34.5)
Brazil	45 to 49	16.7 (10.0–26.2)	16.9 (12.6–22.2)	17.8 (12.6–23.4)	18.3 (10.0–30.9)	18.5 (9.3–33.0)
Brazil	50 to 54	17.6 (6.3–36.5)	17.1 (8.0–30.1)	16.8 (11.7–23.0)	17.1 (9.4–28.8)	17.2 (8.7–30.6)
Brazil	55 to 59	15.8 (5.4–33.8)	14.9 (6.8–26.7)	14.1 (9.5–19.9)	14.6 (7.6–25.3)	14.7 (7.2–26.8)

Supplementary Table S10: Prevalence of female SVAC by age and location for 1990, 2000, 2010, 2020, and 2023						
**Location**	**Age Range**	**1990**	**2000**	**2010**	**2020**	**2023**
Brazil	60 to 64	14.6 (4.7–31.1)	13.6 (5.9–25.0)	12.7 (7.8–19.1)	13.4 (6.4–23.2)	13.6 (5.9–25.0)
Brazil	65 to 69	13.1 (4.2–28.4)	12.1 (5.1–22.6)	11.1 (6.5–17.2)	11.8 (5.5–21.0)	12.0 (5.1–22.5)
Brazil	70 to 74	11.2 (3.4–24.8)	10.5 (4.3–20.2)	10.0 (5.4–16.4)	10.5 (4.7–19.2)	10.7 (4.4–20.6)
Brazil	75 to 79	10.4 (3.2–23.4)	10.0 (4.1–19.2)	9.7 (5.3–15.7)	10.1 (4.5–18.5)	10.2 (4.2–19.7)
Brazil	80 to 84	7.0 (1.8–17.1)	6.6 (2.2–15.0)	6.3 (2.3–13.3)	6.6 (2.3–14.9)	6.7 (2.2–15.9)
Brazil	85 to 89	6.3 (1.7–15.4)	6.0 (2.0–13.8)	5.8 (2.1–12.3)	6.1 (2.0–13.9)	6.1 (2.0–14.7)
Brazil	90 to 94	5.8 (1.5–14.3)	5.5 (1.8–12.7)	5.4 (1.9–11.4)	5.6 (1.8–12.8)	5.7 (1.7–13.6)
Brazil	95 plus	5.8 (1.5–14.3)	5.5 (1.8–12.6)	5.4 (1.9–11.3)	5.6 (1.8–12.8)	5.6 (1.8–13.6)
Brazil	Age-standardized	14.9 (8.0–24.8)	15.2 (10.1–22.0)	16.6 (13.0–20.7)	17.5 (11.8–26.7)	17.7 (11.2–28.2)
Brazil	All age	15.0 (8.4–24.6)	15.5 (10.5–21.8)	16.8 (13.1–21.2)	17.3 (11.9–26.1)	17.4 (11.4–27.1)
Paraguay	20 to 24	8.8 (7.1–10.8)	9.5 (7.9–11.3)	8.7 (6.8–11.0)	8.7 (3.9–15.6)	8.8 (3.5–17.1)
Paraguay	25 to 29	8.1 (6.1–10.6)	9.5 (8.0–11.3)	9.3 (7.9–11.0)	9.1 (5.0–14.8)	9.2 (4.4–16.5)
Paraguay	30 to 34	8.0 (4.9–12.1)	8.8 (7.3–10.7)	9.4 (8.1–10.9)	9.4 (6.5–12.7)	9.5 (5.9–13.7)
Paraguay	35 to 39	8.2 (3.9–14.4)	8.6 (6.6–11.1)	9.8 (8.5–11.3)	9.4 (7.0–12.3)	9.3 (6.4–13.1)
Paraguay	40 to 44	8.8 (3.6–17.3)	8.2 (5.3–12.2)	9.3 (7.9–10.8)	9.9 (7.3–12.9)	10.0 (6.8–14.1)

Supplementary Table S10: Prevalence of female SVAC by age and location for 1990, 2000, 2010, 2020, and 2023						
**Location**	**Age Range**	**1990**	**2000**	**2010**	**2020**	**2023**
Paraguay	45 to 49	9.2 (3.2–19.4)	8.3 (3.9–14.8)	8.2 (6.0–10.7)	9.5 (6.9–12.6)	9.8 (6.5–14.1)
Paraguay	50 to 54	9.5 (3.2–21.3)	8.7 (3.4–17.6)	8.1 (4.9–12.6)	8.7 (6.2–11.8)	9.0 (5.8–13.3)
Paraguay	55 to 59	9.7 (3.1–21.4)	9.1 (3.2–19.5)	8.3 (3.9–15.3)	7.7 (4.9–11.6)	7.7 (4.7–12.2)
Paraguay	60 to 64	9.8 (3.1–22.1)	9.7 (2.9–22.1)	9.7 (3.0–21.6)	9.8 (3.2–23.3)	9.9 (3.2–23.8)
Paraguay	65 to 69	9.0 (2.8–20.7)	9.0 (2.7–20.6)	9.0 (2.8–20.2)	9.1 (3.0–21.8)	9.2 (3.0–22.3)
Paraguay	70 to 74	7.2 (2.2–16.7)	7.1 (2.1–16.7)	7.1 (2.2–16.3)	7.2 (2.3–17.8)	7.3 (2.3–18.1)
Paraguay	75 to 79	6.4 (1.9–15.1)	6.4 (1.9–15.1)	6.4 (1.9–14.8)	6.5 (2.0–16.0)	6.5 (2.0–16.4)
Paraguay	80 to 84	4.5 (1.3–10.8)	4.5 (1.3–10.8)	4.5 (1.3–10.5)	4.5 (1.4–11.6)	4.6 (1.4–11.8)
Paraguay	85 to 89	4.0 (1.2– 9.7)	4.0 (1.1– 9.7)	4.0 (1.2– 9.5)	4.0 (1.2–10.4)	4.1 (1.2–10.6)
Paraguay	90 to 94	3.6 (1.1– 8.8)	3.6 (1.0– 8.8)	3.6 (1.1– 8.6)	3.6 (1.1– 9.3)	3.7 (1.1– 9.6)
Paraguay	95 plus	3.6 (1.1– 8.7)	3.6 (1.0– 8.7)	3.6 (1.0– 8.5)	3.6 (1.1– 9.3)	3.6 (1.1– 9.5)
Paraguay	Age-standardized	8.5 (4.7–14.7)	8.7 (5.9–12.9)	8.7 (6.7–11.9)	8.9 (6.6–12.3)	8.9 (6.6–12.6)
Paraguay	All age	8.5 (5.2–13.7)	8.8 (6.4–12.1)	8.8 (7.0–11.4)	8.9 (6.6–12.3)	9.0 (6.6–12.6)
North Africa and Middle East	20 to 24	13.4 (4.5–29.9)	13.1 (4.8–27.1)	13.0 (5.5–25.6)	13.2 (6.3–27.5)	13.3 (6.2–28.6)
North Africa and Middle East	25 to 29	12.7 (4.0–29.5)	12.5 (4.3–26.6)	12.7 (4.8–25.7)	13.2 (5.0–29.4)	13.4 (4.9–30.1)

Supplementary Table S10: Prevalence of female SVAC by age and location for 1990, 2000, 2010, 2020, and 2023						
**Location**	**Age Range**	**1990**	**2000**	**2010**	**2020**	**2023**
North Africa and Middle East	30 to 34	13.4 (4.1–30.8)	13.2 (4.6–27.8)	13.4 (5.2–26.6)	13.9 (5.2–30.6)	14.0 (5.1–31.4)
North Africa and Middle East	35 to 39	12.9 (4.0–29.2)	12.7 (4.4–27.1)	12.8 (4.8–26.3)	13.4 (5.0–29.8)	13.6 (4.8–30.7)
North Africa and Middle East	40 to 44	13.0 (4.1–29.4)	12.8 (4.4–27.3)	13.0 (4.9–26.5)	13.5 (5.1–29.5)	13.7 (5.0–30.5)
North Africa and Middle East	45 to 49	12.1 (3.6–28.0)	11.8 (3.9–25.9)	11.8 (4.2–24.6)	12.2 (4.2–27.5)	12.3 (4.0–28.7)
North Africa and Middle East	50 to 54	11.7 (3.5–27.2)	11.4 (3.8–24.9)	11.4 (4.1–23.9)	11.8 (4.1–27.0)	11.9 (4.0–27.7)
North Africa and Middle East	55 to 59	11.0 (3.2–25.8)	10.8 (3.5–23.9)	10.8 (3.7–22.8)	11.1 (3.8–25.3)	11.3 (3.7–26.6)
North Africa and Middle East	60 to 64	11.3 (3.0–27.4)	11.1 (3.3–25.1)	11.0 (3.5–24.2)	11.4 (3.5–27.2)	11.5 (3.5–28.1)
North Africa and Middle East	65 to 69	10.5 (2.8–25.9)	10.2 (3.0–23.3)	10.2 (3.2–22.6)	10.5 (3.3–25.6)	10.6 (3.1–26.4)
North Africa and Middle East	70 to 74	8.4 (2.1–20.9)	8.1 (2.3–18.8)	8.1 (2.5–18.2)	8.3 (2.5–20.9)	8.5 (2.4–21.9)
North Africa and Middle East	75 to 79	7.5 (1.9–19.0)	7.2 (2.1–17.1)	7.2 (2.2–16.4)	7.5 (2.2–18.9)	7.5 (2.2–19.3)
North Africa and Middle East	80 to 84	5.2 (1.3–13.6)	5.1 (1.4–12.4)	5.0 (1.5–11.7)	5.2 (1.5–13.6)	5.3 (1.5–14.3)
North Africa and Middle East	85 to 89	4.7 (1.2–12.5)	4.5 (1.3–11.1)	4.5 (1.3–10.6)	4.7 (1.3–12.5)	4.7 (1.3–12.9)
North Africa and Middle East	90 to 94	4.3 (1.1–11.4)	4.1 (1.1–10.0)	4.1 (1.2– 9.6)	4.2 (1.2–11.2)	4.2 (1.2–11.6)
North Africa and Middle East	95 plus	4.2 (1.0–11.2)	4.1 (1.1– 9.9)	4.0 (1.2– 9.5)	4.1 (1.2–11.2)	4.2 (1.1–11.5)
North Africa and Middle East	Age-standardized	12.0 (3.6–27.6)	11.7 (4.0–25.3)	11.8 (4.4–24.2)	12.2 (4.5–27.3)	12.3 (4.5–28.2)

Supplementary Table S10: Prevalence of female SVAC by age and location for 1990, 2000, 2010, 2020, and 2023						
**Location**	**Age Range**	**1990**	**2000**	**2010**	**2020**	**2023**
North Africa and Middle East	All age	12.4 (3.8–28.5)	12.2 (4.2–26.1)	12.2 (4.6–24.8)	12.5 (4.7–27.8)	12.6 (4.6–28.7)
North Africa and Middle East	20 to 24	13.4 (4.5–29.9)	13.1 (4.8–27.1)	13.0 (5.5–25.6)	13.2 (6.3–27.5)	13.3 (6.2–28.6)
North Africa and Middle East	25 to 29	12.7 (4.0–29.5)	12.5 (4.3–26.6)	12.7 (4.8–25.7)	13.2 (5.0–29.4)	13.4 (4.9–30.1)
North Africa and Middle East	30 to 34	13.4 (4.1–30.8)	13.2 (4.6–27.8)	13.4 (5.2–26.6)	13.9 (5.2–30.6)	14.0 (5.1–31.4)
North Africa and Middle East	35 to 39	12.9 (4.0–29.2)	12.7 (4.4–27.1)	12.8 (4.8–26.3)	13.4 (5.0–29.8)	13.6 (4.8–30.7)
North Africa and Middle East	40 to 44	13.0 (4.1–29.4)	12.8 (4.4–27.3)	13.0 (4.9–26.5)	13.5 (5.1–29.5)	13.7 (5.0–30.5)
North Africa and Middle East	45 to 49	12.1 (3.6–28.0)	11.8 (3.9–25.9)	11.8 (4.2–24.6)	12.2 (4.2–27.5)	12.3 (4.0–28.7)
North Africa and Middle East	50 to 54	11.7 (3.5–27.2)	11.4 (3.8–24.9)	11.4 (4.1–23.9)	11.8 (4.1–27.0)	11.9 (4.0–27.7)
North Africa and Middle East	55 to 59	11.0 (3.2–25.8)	10.8 (3.5–23.9)	10.8 (3.7–22.8)	11.1 (3.8–25.3)	11.3 (3.7–26.6)
North Africa and Middle East	60 to 64	11.3 (3.0–27.4)	11.1 (3.3–25.1)	11.0 (3.5–24.2)	11.4 (3.5–27.2)	11.5 (3.5–28.1)
North Africa and Middle East	65 to 69	10.5 (2.8–25.9)	10.2 (3.0–23.3)	10.2 (3.2–22.6)	10.5 (3.3–25.6)	10.6 (3.1–26.4)
North Africa and Middle East	70 to 74	8.4 (2.1–20.9)	8.1 (2.3–18.8)	8.1 (2.5–18.2)	8.3 (2.5–20.9)	8.5 (2.4–21.9)
North Africa and Middle East	75 to 79	7.5 (1.9–19.0)	7.2 (2.1–17.1)	7.2 (2.2–16.4)	7.5 (2.2–18.9)	7.5 (2.2–19.3)
North Africa and Middle East	80 to 84	5.2 (1.3–13.6)	5.1 (1.4–12.4)	5.0 (1.5–11.7)	5.2 (1.5–13.6)	5.3 (1.5–14.3)
North Africa and Middle East	85 to 89	4.7 (1.2–12.5)	4.5 (1.3–11.1)	4.5 (1.3–10.6)	4.7 (1.3–12.5)	4.7 (1.3–12.9)

Supplementary Table S10: Prevalence of female SVAC by age and location for 1990, 2000, 2010, 2020, and 2023						
**Location**	**Age Range**	**1990**	**2000**	**2010**	**2020**	**2023**
North Africa and Middle East	90 to 94	4.3 (1.1–11.4)	4.1 (1.1–10.0)	4.1 (1.2– 9.6)	4.2 (1.2–11.2)	4.2 (1.2–11.6)
North Africa and Middle East	95 plus	4.2 (1.0–11.2)	4.1 (1.1– 9.9)	4.0 (1.2– 9.5)	4.1 (1.2–11.2)	4.2 (1.1–11.5)
North Africa and Middle East	Age-standardized	12.0 (3.6–27.6)	11.7 (4.0–25.3)	11.8 (4.4–24.2)	12.2 (4.5–27.3)	12.3 (4.5–28.2)
North Africa and Middle East	All age	12.4 (3.8–28.5)	12.2 (4.2–26.1)	12.2 (4.6–24.8)	12.5 (4.7–27.8)	12.6 (4.6–28.7)
Afghanistan	20 to 24	12.5 (3.3–30.9)	12.2 (3.7–27.4)	12.2 (3.9–26.4)	12.5 (3.9–29.6)	12.7 (3.8–30.7)
Afghanistan	25 to 29	12.6 (3.3–30.9)	12.2 (3.7–27.5)	12.2 (3.9–26.5)	12.6 (3.9–30.3)	12.7 (3.8–31.1)
Afghanistan	30 to 34	13.2 (3.5–32.3)	12.9 (4.0–28.7)	12.9 (4.2–27.7)	13.2 (4.2–31.3)	13.4 (4.0–32.2)
Afghanistan	35 to 39	13.0 (3.4–32.1)	12.7 (3.9–28.3)	12.7 (4.1–27.3)	13.0 (4.1–30.9)	13.1 (3.9–31.9)
Afghanistan	40 to 44	13.0 (3.5–31.9)	12.7 (3.9–28.4)	12.7 (4.1–27.4)	13.1 (4.1–31.0)	13.2 (3.9–31.8)
Afghanistan	45 to 49	12.4 (3.2–30.2)	12.1 (3.7–27.1)	12.1 (3.9–26.1)	12.4 (3.9–29.6)	12.5 (3.8–30.0)
Afghanistan	50 to 54	11.9 (3.0–29.6)	11.6 (3.5–26.1)	11.6 (3.7–25.2)	11.9 (3.7–28.6)	12.0 (3.6–29.4)
Afghanistan	55 to 59	11.3 (2.9–28.4)	11.0 (3.3–25.1)	11.0 (3.5–24.2)	11.4 (3.6–27.4)	11.5 (3.5–27.9)
Afghanistan	60 to 64	11.0 (2.9–27.7)	10.7 (3.2–24.4)	10.7 (3.4–23.5)	11.0 (3.4–26.8)	11.1 (3.4–27.1)
Afghanistan	65 to 69	10.2 (2.6–26.0)	9.9 (2.9–22.9)	9.9 (3.1–22.0)	10.2 (3.2–25.1)	10.3 (3.1–25.5)
Afghanistan	70 to 74	8.1 (2.0–20.7)	7.9 (2.3–18.6)	7.9 (2.4–17.9)	8.1 (2.4–20.6)	8.2 (2.4–21.3)

Supplementary Table S10: Prevalence of female SVAC by age and location for 1990, 2000, 2010, 2020, and 2023						
**Location**	**Age Range**	**1990**	**2000**	**2010**	**2020**	**2023**
Afghanistan	75 to 79	7.3 (1.8–19.3)	7.1 (2.0–17.0)	7.1 (2.1–16.2)	7.3 (2.1–18.4)	7.4 (2.1–18.9)
Afghanistan	80 to 84	5.1 (1.2–13.8)	5.0 (1.4–12.1)	5.0 (1.5–11.6)	5.1 (1.5–13.5)	5.2 (1.5–13.9)
Afghanistan	85 to 89	4.6 (1.1–12.8)	4.5 (1.2–11.0)	4.4 (1.3–10.5)	4.6 (1.3–12.2)	4.7 (1.2–12.7)
Afghanistan	90 to 94	4.2 (1.0–11.5)	4.0 (1.1– 9.9)	4.0 (1.2– 9.5)	4.1 (1.2–11.1)	4.2 (1.1–11.4)
Afghanistan	95 plus	4.1 (1.0–11.4)	4.0 (1.1– 9.8)	4.0 (1.2– 9.4)	4.1 (1.2–11.1)	4.2 (1.1–11.5)
Afghanistan	Age-standardized	11.8 (3.1–29.4)	11.5 (3.5–26.0)	11.5 (3.7–25.1)	11.8 (3.7–28.3)	12.0 (3.5–29.2)
Afghanistan	All age	12.0 (3.1–29.8)	11.9 (3.6–26.8)	12.1 (3.9–26.1)	12.4 (3.9–29.4)	12.5 (3.7–30.4)
Algeria	20 to 24	12.5 (3.3–30.9)	12.2 (3.7–27.4)	12.2 (3.9–26.4)	12.5 (3.9–29.6)	12.7 (3.8–30.7)
Algeria	25 to 29	12.6 (3.3–30.9)	12.2 (3.7–27.5)	12.2 (3.9–26.5)	12.6 (3.9–30.3)	12.7 (3.8–31.1)
Algeria	30 to 34	13.2 (3.5–32.3)	12.9 (4.0–28.7)	12.9 (4.2–27.7)	13.2 (4.2–31.3)	13.4 (4.0–32.2)
Algeria	35 to 39	13.0 (3.4–32.1)	12.7 (3.9–28.3)	12.7 (4.1–27.3)	13.0 (4.1–30.9)	13.1 (3.9–31.9)
Algeria	40 to 44	13.0 (3.5–31.9)	12.7 (3.9–28.4)	12.7 (4.1–27.4)	13.1 (4.1–31.0)	13.2 (3.9–31.8)
Algeria	45 to 49	12.4 (3.2–30.2)	12.1 (3.7–27.1)	12.1 (3.9–26.1)	12.4 (3.9–29.6)	12.5 (3.8–30.0)
Algeria	50 to 54	11.9 (3.0–29.6)	11.6 (3.5–26.1)	11.6 (3.7–25.2)	11.9 (3.7–28.6)	12.0 (3.6–29.4)
Algeria	55 to 59	11.3 (2.9–28.4)	11.0 (3.3–25.1)	11.0 (3.5–24.2)	11.4 (3.6–27.4)	11.5 (3.5–27.9)

Supplementary Table S10: Prevalence of female SVAC by age and location for 1990, 2000, 2010, 2020, and 2023						
**Location**	**Age Range**	**1990**	**2000**	**2010**	**2020**	**2023**
Algeria	60 to 64	11.0 (2.9–27.7)	10.7 (3.2–24.4)	10.7 (3.4–23.5)	11.0 (3.4–26.8)	11.1 (3.4–27.1)
Algeria	65 to 69	10.2 (2.6–26.0)	9.9 (2.9–22.9)	9.9 (3.1–22.0)	10.2 (3.2–25.1)	10.3 (3.1–25.5)
Algeria	70 to 74	8.1 (2.0–20.7)	7.9 (2.3–18.6)	7.9 (2.4–17.9)	8.1 (2.4–20.6)	8.2 (2.4–21.3)
Algeria	75 to 79	7.3 (1.8–19.3)	7.1 (2.0–17.0)	7.1 (2.1–16.2)	7.3 (2.1–18.4)	7.4 (2.1–18.9)
Algeria	80 to 84	5.1 (1.2–13.8)	5.0 (1.4–12.1)	5.0 (1.5–11.6)	5.1 (1.5–13.5)	5.2 (1.5–13.9)
Algeria	85 to 89	4.6 (1.1–12.8)	4.5 (1.2–11.0)	4.4 (1.3–10.5)	4.6 (1.3–12.2)	4.7 (1.2–12.7)
Algeria	90 to 94	4.2 (1.0–11.5)	4.0 (1.1– 9.9)	4.0 (1.2– 9.5)	4.1 (1.2–11.1)	4.2 (1.1–11.4)
Algeria	95 plus	4.1 (1.0–11.4)	4.0 (1.1– 9.8)	4.0 (1.2– 9.4)	4.1 (1.2–11.1)	4.2 (1.1–11.5)
Algeria	Age-standardized	11.8 (3.1–29.4)	11.5 (3.5–26.0)	11.5 (3.7–25.1)	11.8 (3.7–28.3)	12.0 (3.5–29.2)
Algeria	All age	12.2 (3.2–30.1)	11.9 (3.6–26.7)	11.9 (3.8–25.7)	12.1 (3.8–28.9)	12.2 (3.6–29.6)
Bahrain	20 to 24	12.5 (3.3–30.9)	12.2 (3.7–27.4)	12.2 (3.9–26.4)	12.5 (3.9–29.6)	12.7 (3.8–30.7)
Bahrain	25 to 29	12.6 (3.3–30.9)	12.2 (3.7–27.5)	12.2 (3.9–26.5)	12.6 (3.9–30.3)	12.7 (3.8–31.1)
Bahrain	30 to 34	13.2 (3.5–32.3)	12.9 (4.0–28.7)	12.9 (4.2–27.7)	13.2 (4.2–31.3)	13.4 (4.0–32.2)
Bahrain	35 to 39	13.0 (3.4–32.1)	12.7 (3.9–28.3)	12.7 (4.1–27.3)	13.0 (4.1–30.9)	13.1 (3.9–31.9)
Bahrain	40 to 44	13.0 (3.5–31.9)	12.7 (3.9–28.4)	12.7 (4.1–27.4)	13.1 (4.1–31.0)	13.2 (3.9–31.8)

Supplementary Table S10: Prevalence of female SVAC by age and location for 1990, 2000, 2010, 2020, and 2023						
**Location**	**Age Range**	**1990**	**2000**	**2010**	**2020**	**2023**
Bahrain	45 to 49	12.4 (3.2–30.2)	12.1 (3.7–27.1)	12.1 (3.9–26.1)	12.4 (3.9–29.6)	12.5 (3.8–30.0)
Bahrain	50 to 54	11.9 (3.0–29.6)	11.6 (3.5–26.1)	11.6 (3.7–25.2)	11.9 (3.7–28.6)	12.0 (3.6–29.4)
Bahrain	55 to 59	11.3 (2.9–28.4)	11.0 (3.3–25.1)	11.0 (3.5–24.2)	11.4 (3.6–27.4)	11.5 (3.5–27.9)
Bahrain	60 to 64	11.0 (2.9–27.7)	10.7 (3.2–24.4)	10.7 (3.4–23.5)	11.0 (3.4–26.8)	11.1 (3.4–27.1)
Bahrain	65 to 69	10.2 (2.6–26.0)	9.9 (2.9–22.9)	9.9 (3.1–22.0)	10.2 (3.2–25.1)	10.3 (3.1–25.5)
Bahrain	70 to 74	8.1 (2.0–20.7)	7.9 (2.3–18.6)	7.9 (2.4–17.9)	8.1 (2.4–20.6)	8.2 (2.4–21.3)
Bahrain	75 to 79	7.3 (1.8–19.3)	7.1 (2.0–17.0)	7.1 (2.1–16.2)	7.3 (2.1–18.4)	7.4 (2.1–18.9)
Bahrain	80 to 84	5.1 (1.2–13.8)	5.0 (1.4–12.1)	5.0 (1.5–11.6)	5.1 (1.5–13.5)	5.2 (1.5–13.9)
Bahrain	85 to 89	4.6 (1.1–12.8)	4.5 (1.2–11.0)	4.4 (1.3–10.5)	4.6 (1.3–12.2)	4.7 (1.2–12.7)
Bahrain	90 to 94	4.2 (1.0–11.5)	4.0 (1.1– 9.9)	4.0 (1.2– 9.5)	4.1 (1.2–11.1)	4.2 (1.1–11.4)
Bahrain	95 plus	4.1 (1.0–11.4)	4.0 (1.1– 9.8)	4.0 (1.2– 9.4)	4.1 (1.2–11.1)	4.2 (1.1–11.5)
Bahrain	Age-standardized	11.8 (3.1–29.4)	11.5 (3.5–26.0)	11.5 (3.7–25.1)	11.8 (3.7–28.3)	12.0 (3.5–29.2)
Bahrain	All age	12.4 (3.3–30.7)	12.2 (3.7–27.3)	12.1 (3.9–26.3)	12.3 (3.8–29.3)	12.3 (3.7–30.1)
Egypt	20 to 24	13.7 (3.7–33.3)	13.4 (4.1–29.6)	13.4 (4.4–28.6)	13.7 (4.3–31.7)	13.8 (4.1–32.4)
Egypt	25 to 29	14.0 (3.8–33.9)	13.6 (4.2–30.2)	13.6 (4.4–29.0)	13.9 (4.5–32.6)	14.0 (4.3–33.0)

Supplementary Table S10: Prevalence of female SVAC by age and location for 1990, 2000, 2010, 2020, and 2023						
**Location**	**Age Range**	**1990**	**2000**	**2010**	**2020**	**2023**
Egypt	30 to 34	15.0 (4.0–35.7)	14.7 (4.6–31.9)	14.6 (4.8–30.8)	15.0 (4.8–34.7)	15.1 (4.6–35.5)
Egypt	35 to 39	15.0 (4.1–35.8)	14.7 (4.6–32.1)	14.7 (4.8–30.9)	15.0 (4.8–34.8)	15.1 (4.6–35.6)
Egypt	40 to 44	15.4 (4.2–36.5)	15.1 (4.7–32.7)	15.1 (5.0–31.6)	15.4 (4.9–35.4)	15.5 (4.7–36.1)
Egypt	45 to 49	14.9 (4.0–35.5)	14.6 (4.5–31.8)	14.6 (4.8–30.7)	14.9 (4.8–34.5)	15.0 (4.6–35.0)
Egypt	50 to 54	14.5 (3.9–35.0)	14.2 (4.4–31.0)	14.2 (4.6–30.0)	14.5 (4.6–33.8)	14.6 (4.4–34.0)
Egypt	55 to 59	14.0 (3.7–33.1)	13.6 (4.2–30.1)	13.6 (4.5–29.1)	14.0 (4.4–32.8)	14.1 (4.3–33.6)
Egypt	60 to 64	13.5 (3.5–32.9)	13.2 (4.1–29.3)	13.2 (4.3–28.3)	13.6 (4.2–31.5)	13.7 (4.1–32.2)
Egypt	65 to 69	12.6 (3.3–31.2)	12.3 (3.7–27.6)	12.3 (4.0–26.6)	12.6 (3.9–30.1)	12.8 (3.9–31.0)
Egypt	70 to 74	10.1 (2.5–25.7)	9.8 (2.9–22.7)	9.8 (3.1–21.8)	10.1 (3.0–24.8)	10.2 (3.0–25.4)
Egypt	75 to 79	9.1 (2.2–23.2)	8.9 (2.6–20.8)	8.8 (2.7–19.8)	9.1 (2.8–22.7)	9.2 (2.7–23.5)
Egypt	80 to 84	6.5 (1.6–17.3)	6.2 (1.8–15.0)	6.2 (1.9–14.4)	6.4 (1.9–16.7)	6.5 (1.8–17.2)
Egypt	85 to 89	5.8 (1.4–15.7)	5.6 (1.6–13.6)	5.6 (1.7–13.0)	5.8 (1.7–15.1)	5.9 (1.7–15.7)
Egypt	90 to 94	5.2 (1.3–14.1)	5.1 (1.4–12.4)	5.1 (1.5–11.8)	5.2 (1.5–13.8)	5.3 (1.5–14.0)
Egypt	95 plus	5.2 (1.2–14.0)	5.0 (1.4–12.3)	5.0 (1.5–11.7)	5.2 (1.5–13.7)	5.3 (1.4–13.9)
Egypt	Age-standardized	13.8 (3.7–33.4)	13.5 (4.2–29.8)	13.5 (4.4–28.7)	13.8 (4.4–32.1)	13.9 (4.2–32.9)

Supplementary Table S10: Prevalence of female SVAC by age and location for 1990, 2000, 2010, 2020, and 2023						
**Location**	**Age Range**	**1990**	**2000**	**2010**	**2020**	**2023**
Egypt	All age	14.2 (3.8–34.2)	13.9 (4.3–30.5)	13.9 (4.5–29.5)	14.2 (4.5–32.8)	14.3 (4.3–33.6)
Iran	20 to 24	12.5 (3.3–30.9)	12.2 (3.7–27.4)	12.2 (3.9–26.4)	12.5 (3.9–29.6)	12.7 (3.8–30.7)
Iran	25 to 29	12.6 (3.3–30.9)	12.2 (3.7–27.5)	12.2 (3.9–26.5)	12.6 (3.9–30.3)	12.7 (3.8–31.1)
Iran	30 to 34	13.2 (3.5–32.3)	12.9 (4.0–28.7)	12.9 (4.2–27.7)	13.2 (4.2–31.3)	13.4 (4.0–32.2)
Iran	35 to 39	13.0 (3.4–32.1)	12.7 (3.9–28.3)	12.7 (4.1–27.3)	13.0 (4.1–30.9)	13.1 (3.9–31.9)
Iran	40 to 44	13.0 (3.5–31.9)	12.7 (3.9–28.4)	12.7 (4.1–27.4)	13.1 (4.1–31.0)	13.2 (3.9–31.8)
Iran	45 to 49	12.4 (3.2–30.2)	12.1 (3.7–27.1)	12.1 (3.9–26.1)	12.4 (3.9–29.6)	12.5 (3.8–30.0)
Iran	50 to 54	11.9 (3.0–29.6)	11.6 (3.5–26.1)	11.6 (3.7–25.2)	11.9 (3.7–28.6)	12.0 (3.6–29.4)
Iran	55 to 59	11.3 (2.9–28.4)	11.0 (3.3–25.1)	11.0 (3.5–24.2)	11.4 (3.6–27.4)	11.5 (3.5–27.9)
Iran	60 to 64	11.0 (2.9–27.7)	10.7 (3.2–24.4)	10.7 (3.4–23.5)	11.0 (3.4–26.8)	11.1 (3.4–27.1)
Iran	65 to 69	10.2 (2.6–26.0)	9.9 (2.9–22.9)	9.9 (3.1–22.0)	10.2 (3.2–25.1)	10.3 (3.1–25.5)
Iran	70 to 74	8.1 (2.0–20.7)	7.9 (2.3–18.6)	7.9 (2.4–17.9)	8.1 (2.4–20.6)	8.2 (2.4–21.3)
Iran	75 to 79	7.3 (1.8–19.3)	7.1 (2.0–17.0)	7.1 (2.1–16.2)	7.3 (2.1–18.4)	7.4 (2.1–18.9)
Iran	80 to 84	5.1 (1.2–13.8)	5.0 (1.4–12.1)	5.0 (1.5–11.6)	5.1 (1.5–13.5)	5.2 (1.5–13.9)
Iran	85 to 89	4.6 (1.1–12.8)	4.5 (1.2–11.0)	4.4 (1.3–10.5)	4.6 (1.3–12.2)	4.7 (1.2–12.7)

Supplementary Table S10: Prevalence of female SVAC by age and location for 1990, 2000, 2010, 2020, and 2023						
**Location**	**Age Range**	**1990**	**2000**	**2010**	**2020**	**2023**
Iran	90 to 94	4.2 (1.0–11.5)	4.0 (1.1– 9.9)	4.0 (1.2– 9.5)	4.1 (1.2–11.1)	4.2 (1.1–11.4)
Iran	95 plus	4.1 (1.0–11.4)	4.0 (1.1– 9.8)	4.0 (1.2– 9.4)	4.1 (1.2–11.1)	4.2 (1.1–11.5)
Iran	Age-standardized	11.8 (3.1–29.4)	11.5 (3.5–26.0)	11.5 (3.7–25.1)	11.8 (3.7–28.3)	12.0 (3.5–29.2)
Iran	All age	12.2 (3.2–30.2)	11.9 (3.6–26.7)	11.9 (3.8–25.7)	12.1 (3.8–28.8)	12.1 (3.6–29.6)
Iraq	20 to 24	12.5 (3.3–30.9)	12.2 (3.7–27.4)	12.2 (3.9–26.4)	12.5 (3.9–29.6)	12.7 (3.8–30.7)
Iraq	25 to 29	12.6 (3.3–30.9)	12.2 (3.7–27.5)	12.2 (3.9–26.5)	12.6 (3.9–30.3)	12.7 (3.8–31.1)
Iraq	30 to 34	13.2 (3.5–32.3)	12.9 (4.0–28.7)	12.9 (4.2–27.7)	13.2 (4.2–31.3)	13.4 (4.0–32.2)
Iraq	35 to 39	13.0 (3.4–32.1)	12.7 (3.9–28.3)	12.7 (4.1–27.3)	13.0 (4.1–30.9)	13.1 (3.9–31.9)
Iraq	40 to 44	13.0 (3.5–31.9)	12.7 (3.9–28.4)	12.7 (4.1–27.4)	13.1 (4.1–31.0)	13.2 (3.9–31.8)
Iraq	45 to 49	12.4 (3.2–30.2)	12.1 (3.7–27.1)	12.1 (3.9–26.1)	12.4 (3.9–29.6)	12.5 (3.8–30.0)
Iraq	50 to 54	11.9 (3.0–29.6)	11.6 (3.5–26.1)	11.6 (3.7–25.2)	11.9 (3.7–28.6)	12.0 (3.6–29.4)
Iraq	55 to 59	11.3 (2.9–28.4)	11.0 (3.3–25.1)	11.0 (3.5–24.2)	11.4 (3.6–27.4)	11.5 (3.5–27.9)
Iraq	60 to 64	11.0 (2.9–27.7)	10.7 (3.2–24.4)	10.7 (3.4–23.5)	11.0 (3.4–26.8)	11.1 (3.4–27.1)
Iraq	65 to 69	10.2 (2.6–26.0)	9.9 (2.9–22.9)	9.9 (3.1–22.0)	10.2 (3.2–25.1)	10.3 (3.1–25.5)
Iraq	70 to 74	8.1 (2.0–20.7)	7.9 (2.3–18.6)	7.9 (2.4–17.9)	8.1 (2.4–20.6)	8.2 (2.4–21.3)

Supplementary Table S10: Prevalence of female SVAC by age and location for 1990, 2000, 2010, 2020, and 2023						
**Location**	**Age Range**	**1990**	**2000**	**2010**	**2020**	**2023**
Iraq	75 to 79	7.3 (1.8–19.3)	7.1 (2.0–17.0)	7.1 (2.1–16.2)	7.3 (2.1–18.4)	7.4 (2.1–18.9)
Iraq	80 to 84	5.1 (1.2–13.8)	5.0 (1.4–12.1)	5.0 (1.5–11.6)	5.1 (1.5–13.5)	5.2 (1.5–13.9)
Iraq	85 to 89	4.6 (1.1–12.8)	4.5 (1.2–11.0)	4.4 (1.3–10.5)	4.6 (1.3–12.2)	4.7 (1.2–12.7)
Iraq	90 to 94	4.2 (1.0–11.5)	4.0 (1.1– 9.9)	4.0 (1.2– 9.5)	4.1 (1.2–11.1)	4.2 (1.1–11.4)
Iraq	95 plus	4.1 (1.0–11.4)	4.0 (1.1– 9.8)	4.0 (1.2– 9.4)	4.1 (1.2–11.1)	4.2 (1.1–11.5)
Iraq	Age-standardized	11.8 (3.1–29.4)	11.5 (3.5–26.0)	11.5 (3.7–25.1)	11.8 (3.7–28.3)	12.0 (3.5–29.2)
Iraq	All age	12.2 (3.2–30.2)	12.0 (3.6–26.9)	12.0 (3.8–26.0)	12.3 (3.8–29.2)	12.3 (3.7–30.1)
Jordan	20 to 24	12.5 (3.3–30.9)	12.2 (3.7–27.4)	12.2 (3.9–26.4)	12.5 (3.9–29.6)	12.7 (3.8–30.7)
Jordan	25 to 29	12.6 (3.3–30.9)	12.2 (3.7–27.5)	12.2 (3.9–26.5)	12.6 (3.9–30.3)	12.7 (3.8–31.1)
Jordan	30 to 34	13.2 (3.5–32.3)	12.9 (4.0–28.7)	12.9 (4.2–27.7)	13.2 (4.2–31.3)	13.4 (4.0–32.2)
Jordan	35 to 39	13.0 (3.4–32.1)	12.7 (3.9–28.3)	12.7 (4.1–27.3)	13.0 (4.1–30.9)	13.1 (3.9–31.9)
Jordan	40 to 44	13.0 (3.5–31.9)	12.7 (3.9–28.4)	12.7 (4.1–27.4)	13.1 (4.1–31.0)	13.2 (3.9–31.8)
Jordan	45 to 49	12.4 (3.2–30.2)	12.1 (3.7–27.1)	12.1 (3.9–26.1)	12.4 (3.9–29.6)	12.5 (3.8–30.0)
Jordan	50 to 54	11.9 (3.0–29.6)	11.6 (3.5–26.1)	11.6 (3.7–25.2)	11.9 (3.7–28.6)	12.0 (3.6–29.4)
Jordan	55 to 59	11.3 (2.9–28.4)	11.0 (3.3–25.1)	11.0 (3.5–24.2)	11.4 (3.6–27.4)	11.5 (3.5–27.9)

Supplementary Table S10: Prevalence of female SVAC by age and location for 1990, 2000, 2010, 2020, and 2023						
**Location**	**Age Range**	**1990**	**2000**	**2010**	**2020**	**2023**
Jordan	60 to 64	11.0 (2.9–27.7)	10.7 (3.2–24.4)	10.7 (3.4–23.5)	11.0 (3.4–26.8)	11.1 (3.4–27.1)
Jordan	65 to 69	10.2 (2.6–26.0)	9.9 (2.9–22.9)	9.9 (3.1–22.0)	10.2 (3.2–25.1)	10.3 (3.1–25.5)
Jordan	70 to 74	8.1 (2.0–20.7)	7.9 (2.3–18.6)	7.9 (2.4–17.9)	8.1 (2.4–20.6)	8.2 (2.4–21.3)
Jordan	75 to 79	7.3 (1.8–19.3)	7.1 (2.0–17.0)	7.1 (2.1–16.2)	7.3 (2.1–18.4)	7.4 (2.1–18.9)
Jordan	80 to 84	5.1 (1.2–13.8)	5.0 (1.4–12.1)	5.0 (1.5–11.6)	5.1 (1.5–13.5)	5.2 (1.5–13.9)
Jordan	85 to 89	4.6 (1.1–12.8)	4.5 (1.2–11.0)	4.4 (1.3–10.5)	4.6 (1.3–12.2)	4.7 (1.2–12.7)
Jordan	90 to 94	4.2 (1.0–11.5)	4.0 (1.1– 9.9)	4.0 (1.2– 9.5)	4.1 (1.2–11.1)	4.2 (1.1–11.4)
Jordan	95 plus	4.1 (1.0–11.4)	4.0 (1.1– 9.8)	4.0 (1.2– 9.4)	4.1 (1.2–11.1)	4.2 (1.1–11.5)
Jordan	Age-standardized	11.8 (3.1–29.4)	11.5 (3.5–26.0)	11.5 (3.7–25.1)	11.8 (3.7–28.3)	12.0 (3.5–29.2)
Jordan	All age	12.3 (3.2–30.4)	12.0 (3.7–27.0)	12.0 (3.9–26.1)	12.3 (3.8–29.2)	12.3 (3.6–30.0)
Kuwait	20 to 24	12.5 (3.3–30.9)	12.2 (3.7–27.4)	12.2 (3.9–26.4)	12.5 (3.9–29.6)	12.7 (3.8–30.7)
Kuwait	25 to 29	12.6 (3.3–30.9)	12.2 (3.7–27.5)	12.2 (3.9–26.5)	12.6 (3.9–30.3)	12.7 (3.8–31.1)
Kuwait	30 to 34	13.2 (3.5–32.3)	12.9 (4.0–28.7)	12.9 (4.2–27.7)	13.2 (4.2–31.3)	13.4 (4.0–32.2)
Kuwait	35 to 39	13.0 (3.4–32.1)	12.7 (3.9–28.3)	12.7 (4.1–27.3)	13.0 (4.1–30.9)	13.1 (3.9–31.9)
Kuwait	40 to 44	13.0 (3.5–31.9)	12.7 (3.9–28.4)	12.7 (4.1–27.4)	13.1 (4.1–31.0)	13.2 (3.9–31.8)

Supplementary Table S10: Prevalence of female SVAC by age and location for 1990, 2000, 2010, 2020, and 2023						
**Location**	**Age Range**	**1990**	**2000**	**2010**	**2020**	**2023**
Kuwait	45 to 49	12.4 (3.2–30.2)	12.1 (3.7–27.1)	12.1 (3.9–26.1)	12.4 (3.9–29.6)	12.5 (3.8–30.0)
Kuwait	50 to 54	11.9 (3.0–29.6)	11.6 (3.5–26.1)	11.6 (3.7–25.2)	11.9 (3.7–28.6)	12.0 (3.6–29.4)
Kuwait	55 to 59	11.3 (2.9–28.4)	11.0 (3.3–25.1)	11.0 (3.5–24.2)	11.4 (3.6–27.4)	11.5 (3.5–27.9)
Kuwait	60 to 64	11.0 (2.9–27.7)	10.7 (3.2–24.4)	10.7 (3.4–23.5)	11.0 (3.4–26.8)	11.1 (3.4–27.1)
Kuwait	65 to 69	10.2 (2.6–26.0)	9.9 (2.9–22.9)	9.9 (3.1–22.0)	10.2 (3.2–25.1)	10.3 (3.1–25.5)
Kuwait	70 to 74	8.1 (2.0–20.7)	7.9 (2.3–18.6)	7.9 (2.4–17.9)	8.1 (2.4–20.6)	8.2 (2.4–21.3)
Kuwait	75 to 79	7.3 (1.8–19.3)	7.1 (2.0–17.0)	7.1 (2.1–16.2)	7.3 (2.1–18.4)	7.4 (2.1–18.9)
Kuwait	80 to 84	5.1 (1.2–13.8)	5.0 (1.4–12.1)	5.0 (1.5–11.6)	5.1 (1.5–13.5)	5.2 (1.5–13.9)
Kuwait	85 to 89	4.6 (1.1–12.8)	4.5 (1.2–11.0)	4.4 (1.3–10.5)	4.6 (1.3–12.2)	4.7 (1.2–12.7)
Kuwait	90 to 94	4.2 (1.0–11.5)	4.0 (1.1– 9.9)	4.0 (1.2– 9.5)	4.1 (1.2–11.1)	4.2 (1.1–11.4)
Kuwait	95 plus	4.1 (1.0–11.4)	4.0 (1.1– 9.8)	4.0 (1.2– 9.4)	4.1 (1.2–11.1)	4.2 (1.1–11.5)
Kuwait	Age-standardized	11.8 (3.1–29.4)	11.5 (3.5–26.0)	11.5 (3.7–25.1)	11.8 (3.7–28.3)	12.0 (3.5–29.2)
Kuwait	All age	12.6 (3.3–31.0)	12.3 (3.7–27.5)	12.3 (3.9–26.5)	12.4 (3.9–29.5)	12.4 (3.7–30.2)
Lebanon	20 to 24	12.5 (3.3–30.9)	12.2 (3.7–27.4)	12.2 (3.9–26.4)	12.5 (3.9–29.6)	12.7 (3.8–30.7)
Lebanon	25 to 29	12.6 (3.3–30.9)	12.2 (3.7–27.5)	12.2 (3.9–26.5)	12.6 (3.9–30.3)	12.7 (3.8–31.1)

Supplementary Table S10: Prevalence of female SVAC by age and location for 1990, 2000, 2010, 2020, and 2023						
**Location**	**Age Range**	**1990**	**2000**	**2010**	**2020**	**2023**
Lebanon	30 to 34	13.2 (3.5–32.3)	12.9 (4.0–28.7)	12.9 (4.2–27.7)	13.2 (4.2–31.3)	13.4 (4.0–32.2)
Lebanon	35 to 39	13.0 (3.4–32.1)	12.7 (3.9–28.3)	12.7 (4.1–27.3)	13.0 (4.1–30.9)	13.1 (3.9–31.9)
Lebanon	40 to 44	13.0 (3.5–31.9)	12.7 (3.9–28.4)	12.7 (4.1–27.4)	13.1 (4.1–31.0)	13.2 (3.9–31.8)
Lebanon	45 to 49	12.4 (3.2–30.2)	12.1 (3.7–27.1)	12.1 (3.9–26.1)	12.4 (3.9–29.6)	12.5 (3.8–30.0)
Lebanon	50 to 54	11.9 (3.0–29.6)	11.6 (3.5–26.1)	11.6 (3.7–25.2)	11.9 (3.7–28.6)	12.0 (3.6–29.4)
Lebanon	55 to 59	11.3 (2.9–28.4)	11.0 (3.3–25.1)	11.0 (3.5–24.2)	11.4 (3.6–27.4)	11.5 (3.5–27.9)
Lebanon	60 to 64	11.0 (2.9–27.7)	10.7 (3.2–24.4)	10.7 (3.4–23.5)	11.0 (3.4–26.8)	11.1 (3.4–27.1)
Lebanon	65 to 69	10.2 (2.6–26.0)	9.9 (2.9–22.9)	9.9 (3.1–22.0)	10.2 (3.2–25.1)	10.3 (3.1–25.5)
Lebanon	70 to 74	8.1 (2.0–20.7)	7.9 (2.3–18.6)	7.9 (2.4–17.9)	8.1 (2.4–20.6)	8.2 (2.4–21.3)
Lebanon	75 to 79	7.3 (1.8–19.3)	7.1 (2.0–17.0)	7.1 (2.1–16.2)	7.3 (2.1–18.4)	7.4 (2.1–18.9)
Lebanon	80 to 84	5.1 (1.2–13.8)	5.0 (1.4–12.1)	5.0 (1.5–11.6)	5.1 (1.5–13.5)	5.2 (1.5–13.9)
Lebanon	85 to 89	4.6 (1.1–12.8)	4.5 (1.2–11.0)	4.4 (1.3–10.5)	4.6 (1.3–12.2)	4.7 (1.2–12.7)
Lebanon	90 to 94	4.2 (1.0–11.5)	4.0 (1.1– 9.9)	4.0 (1.2– 9.5)	4.1 (1.2–11.1)	4.2 (1.1–11.4)
Lebanon	95 plus	4.1 (1.0–11.4)	4.0 (1.1– 9.8)	4.0 (1.2– 9.4)	4.1 (1.2–11.1)	4.2 (1.1–11.5)
Lebanon	Age-standardized	11.8 (3.1–29.4)	11.5 (3.5–26.0)	11.5 (3.7–25.1)	11.8 (3.7–28.3)	12.0 (3.5–29.2)

Supplementary Table S10: Prevalence of female SVAC by age and location for 1990, 2000, 2010, 2020, and 2023						
**Location**	**Age Range**	**1990**	**2000**	**2010**	**2020**	**2023**
Lebanon	All age	12.1 (3.2–29.9)	11.7 (3.5–26.3)	11.6 (3.7–25.1)	11.9 (3.7–28.4)	11.9 (3.5–29.1)
Libya	20 to 24	12.5 (3.3–30.9)	12.2 (3.7–27.4)	12.2 (3.9–26.4)	12.5 (3.9–29.6)	12.7 (3.8–30.7)
Libya	25 to 29	12.6 (3.3–30.9)	12.2 (3.7–27.5)	12.2 (3.9–26.5)	12.6 (3.9–30.3)	12.7 (3.8–31.1)
Libya	30 to 34	13.2 (3.5–32.3)	12.9 (4.0–28.7)	12.9 (4.2–27.7)	13.2 (4.2–31.3)	13.4 (4.0–32.2)
Libya	35 to 39	13.0 (3.4–32.1)	12.7 (3.9–28.3)	12.7 (4.1–27.3)	13.0 (4.1–30.9)	13.1 (3.9–31.9)
Libya	40 to 44	13.0 (3.5–31.9)	12.7 (3.9–28.4)	12.7 (4.1–27.4)	13.1 (4.1–31.0)	13.2 (3.9–31.8)
Libya	45 to 49	12.4 (3.2–30.2)	12.1 (3.7–27.1)	12.1 (3.9–26.1)	12.4 (3.9–29.6)	12.5 (3.8–30.0)
Libya	50 to 54	11.9 (3.0–29.6)	11.6 (3.5–26.1)	11.6 (3.7–25.2)	11.9 (3.7–28.6)	12.0 (3.6–29.4)
Libya	55 to 59	11.3 (2.9–28.4)	11.0 (3.3–25.1)	11.0 (3.5–24.2)	11.4 (3.6–27.4)	11.5 (3.5–27.9)
Libya	60 to 64	11.0 (2.9–27.7)	10.7 (3.2–24.4)	10.7 (3.4–23.5)	11.0 (3.4–26.8)	11.1 (3.4–27.1)
Libya	65 to 69	10.2 (2.6–26.0)	9.9 (2.9–22.9)	9.9 (3.1–22.0)	10.2 (3.2–25.1)	10.3 (3.1–25.5)
Libya	70 to 74	8.1 (2.0–20.7)	7.9 (2.3–18.6)	7.9 (2.4–17.9)	8.1 (2.4–20.6)	8.2 (2.4–21.3)
Libya	75 to 79	7.3 (1.8–19.3)	7.1 (2.0–17.0)	7.1 (2.1–16.2)	7.3 (2.1–18.4)	7.4 (2.1–18.9)
Libya	80 to 84	5.1 (1.2–13.8)	5.0 (1.4–12.1)	5.0 (1.5–11.6)	5.1 (1.5–13.5)	5.2 (1.5–13.9)
Libya	85 to 89	4.6 (1.1–12.8)	4.5 (1.2–11.0)	4.4 (1.3–10.5)	4.6 (1.3–12.2)	4.7 (1.2–12.7)

Supplementary Table S10: Prevalence of female SVAC by age and location for 1990, 2000, 2010, 2020, and 2023						
**Location**	**Age Range**	**1990**	**2000**	**2010**	**2020**	**2023**
Libya	90 to 94	4.2 (1.0–11.5)	4.0 (1.1– 9.9)	4.0 (1.2– 9.5)	4.1 (1.2–11.1)	4.2 (1.1–11.4)
Libya	95 plus	4.1 (1.0–11.4)	4.0 (1.1– 9.8)	4.0 (1.2– 9.4)	4.1 (1.2–11.1)	4.2 (1.1–11.5)
Libya	Age-standardized	11.8 (3.1–29.4)	11.5 (3.5–26.0)	11.5 (3.7–25.1)	11.8 (3.7–28.3)	12.0 (3.5–29.2)
Libya	All age	12.2 (3.2–30.1)	12.0 (3.6–26.9)	12.0 (3.9–26.0)	12.2 (3.8–29.1)	12.3 (3.6–29.9)
Morocco	20 to 24	12.5 (3.3–30.9)	12.2 (3.7–27.4)	12.2 (3.9–26.4)	12.5 (3.9–29.6)	12.7 (3.8–30.7)
Morocco	25 to 29	12.6 (3.3–30.9)	12.2 (3.7–27.5)	12.2 (3.9–26.5)	12.6 (3.9–30.3)	12.7 (3.8–31.1)
Morocco	30 to 34	13.2 (3.5–32.3)	12.9 (4.0–28.7)	12.9 (4.2–27.7)	13.2 (4.2–31.3)	13.4 (4.0–32.2)
Morocco	35 to 39	13.0 (3.4–32.1)	12.7 (3.9–28.3)	12.7 (4.1–27.3)	13.0 (4.1–30.9)	13.1 (3.9–31.9)
Morocco	40 to 44	13.0 (3.5–31.9)	12.7 (3.9–28.4)	12.7 (4.1–27.4)	13.1 (4.1–31.0)	13.2 (3.9–31.8)
Morocco	45 to 49	12.4 (3.2–30.2)	12.1 (3.7–27.1)	12.1 (3.9–26.1)	12.4 (3.9–29.6)	12.5 (3.8–30.0)
Morocco	50 to 54	11.9 (3.0–29.6)	11.6 (3.5–26.1)	11.6 (3.7–25.2)	11.9 (3.7–28.6)	12.0 (3.6–29.4)
Morocco	55 to 59	11.3 (2.9–28.4)	11.0 (3.3–25.1)	11.0 (3.5–24.2)	11.4 (3.6–27.4)	11.5 (3.5–27.9)
Morocco	60 to 64	11.0 (2.9–27.7)	10.7 (3.2–24.4)	10.7 (3.4–23.5)	11.0 (3.4–26.8)	11.1 (3.4–27.1)
Morocco	65 to 69	10.2 (2.6–26.0)	9.9 (2.9–22.9)	9.9 (3.1–22.0)	10.2 (3.2–25.1)	10.3 (3.1–25.5)
Morocco	70 to 74	8.1 (2.0–20.7)	7.9 (2.3–18.6)	7.9 (2.4–17.9)	8.1 (2.4–20.6)	8.2 (2.4–21.3)

Supplementary Table S10: Prevalence of female SVAC by age and location for 1990, 2000, 2010, 2020, and 2023						
**Location**	**Age Range**	**1990**	**2000**	**2010**	**2020**	**2023**
Morocco	75 to 79	7.3 (1.8–19.3)	7.1 (2.0–17.0)	7.1 (2.1–16.2)	7.3 (2.1–18.4)	7.4 (2.1–18.9)
Morocco	80 to 84	5.1 (1.2–13.8)	5.0 (1.4–12.1)	5.0 (1.5–11.6)	5.1 (1.5–13.5)	5.2 (1.5–13.9)
Morocco	85 to 89	4.6 (1.1–12.8)	4.5 (1.2–11.0)	4.4 (1.3–10.5)	4.6 (1.3–12.2)	4.7 (1.2–12.7)
Morocco	90 to 94	4.2 (1.0–11.5)	4.0 (1.1– 9.9)	4.0 (1.2– 9.5)	4.1 (1.2–11.1)	4.2 (1.1–11.4)
Morocco	95 plus	4.1 (1.0–11.4)	4.0 (1.1– 9.8)	4.0 (1.2– 9.4)	4.1 (1.2–11.1)	4.2 (1.1–11.5)
Morocco	Age-standardized	11.8 (3.1–29.4)	11.5 (3.5–26.0)	11.5 (3.7–25.1)	11.8 (3.7–28.3)	12.0 (3.5–29.2)
Morocco	All age	12.2 (3.2–30.1)	11.9 (3.6–26.7)	11.8 (3.8–25.6)	12.0 (3.7–28.6)	12.1 (3.6–29.4)
Oman	20 to 24	12.5 (3.3–30.9)	12.2 (3.7–27.4)	12.2 (3.9–26.4)	12.5 (3.9–29.6)	12.7 (3.8–30.7)
Oman	25 to 29	12.6 (3.3–30.9)	12.2 (3.7–27.5)	12.2 (3.9–26.5)	12.6 (3.9–30.3)	12.7 (3.8–31.1)
Oman	30 to 34	13.2 (3.5–32.3)	12.9 (4.0–28.7)	12.9 (4.2–27.7)	13.2 (4.2–31.3)	13.4 (4.0–32.2)
Oman	35 to 39	13.0 (3.4–32.1)	12.7 (3.9–28.3)	12.7 (4.1–27.3)	13.0 (4.1–30.9)	13.1 (3.9–31.9)
Oman	40 to 44	13.0 (3.5–31.9)	12.7 (3.9–28.4)	12.7 (4.1–27.4)	13.1 (4.1–31.0)	13.2 (3.9–31.8)
Oman	45 to 49	12.4 (3.2–30.2)	12.1 (3.7–27.1)	12.1 (3.9–26.1)	12.4 (3.9–29.6)	12.5 (3.8–30.0)
Oman	50 to 54	11.9 (3.0–29.6)	11.6 (3.5–26.1)	11.6 (3.7–25.2)	11.9 (3.7–28.6)	12.0 (3.6–29.4)
Oman	55 to 59	11.3 (2.9–28.4)	11.0 (3.3–25.1)	11.0 (3.5–24.2)	11.4 (3.6–27.4)	11.5 (3.5–27.9)

Supplementary Table S10: Prevalence of female SVAC by age and location for 1990, 2000, 2010, 2020, and 2023						
**Location**	**Age Range**	**1990**	**2000**	**2010**	**2020**	**2023**
Oman	60 to 64	11.0 (2.9–27.7)	10.7 (3.2–24.4)	10.7 (3.4–23.5)	11.0 (3.4–26.8)	11.1 (3.4–27.1)
Oman	65 to 69	10.2 (2.6–26.0)	9.9 (2.9–22.9)	9.9 (3.1–22.0)	10.2 (3.2–25.1)	10.3 (3.1–25.5)
Oman	70 to 74	8.1 (2.0–20.7)	7.9 (2.3–18.6)	7.9 (2.4–17.9)	8.1 (2.4–20.6)	8.2 (2.4–21.3)
Oman	75 to 79	7.3 (1.8–19.3)	7.1 (2.0–17.0)	7.1 (2.1–16.2)	7.3 (2.1–18.4)	7.4 (2.1–18.9)
Oman	80 to 84	5.1 (1.2–13.8)	5.0 (1.4–12.1)	5.0 (1.5–11.6)	5.1 (1.5–13.5)	5.2 (1.5–13.9)
Oman	85 to 89	4.6 (1.1–12.8)	4.5 (1.2–11.0)	4.4 (1.3–10.5)	4.6 (1.3–12.2)	4.7 (1.2–12.7)
Oman	90 to 94	4.2 (1.0–11.5)	4.0 (1.1– 9.9)	4.0 (1.2– 9.5)	4.1 (1.2–11.1)	4.2 (1.1–11.4)
Oman	95 plus	4.1 (1.0–11.4)	4.0 (1.1– 9.8)	4.0 (1.2– 9.4)	4.1 (1.2–11.1)	4.2 (1.1–11.5)
Oman	Age-standardized	11.8 (3.1–29.4)	11.5 (3.5–26.0)	11.5 (3.7–25.1)	11.8 (3.7–28.3)	12.0 (3.5–29.2)
Oman	All age	12.3 (3.2–30.4)	12.0 (3.7–27.0)	12.1 (3.9–26.2)	12.4 (3.9–29.5)	12.5 (3.7–30.4)
Palestine	20 to 24	12.5 (3.3–30.9)	12.2 (3.7–27.4)	12.2 (3.9–26.4)	12.5 (3.9–29.6)	12.7 (3.8–30.7)
Palestine	25 to 29	12.6 (3.3–30.9)	12.2 (3.7–27.5)	12.2 (3.9–26.5)	12.6 (3.9–30.3)	12.7 (3.8–31.1)
Palestine	30 to 34	13.2 (3.5–32.3)	12.9 (4.0–28.7)	12.9 (4.2–27.7)	13.2 (4.2–31.3)	13.4 (4.0–32.2)
Palestine	35 to 39	13.0 (3.4–32.1)	12.7 (3.9–28.3)	12.7 (4.1–27.3)	13.0 (4.1–30.9)	13.1 (3.9–31.9)
Palestine	40 to 44	13.0 (3.5–31.9)	12.7 (3.9–28.4)	12.7 (4.1–27.4)	13.1 (4.1–31.0)	13.2 (3.9–31.8)

Supplementary Table S10: Prevalence of female SVAC by age and location for 1990, 2000, 2010, 2020, and 2023						
**Location**	**Age Range**	**1990**	**2000**	**2010**	**2020**	**2023**
Palestine	45 to 49	12.4 (3.2–30.2)	12.1 (3.7–27.1)	12.1 (3.9–26.1)	12.4 (3.9–29.6)	12.5 (3.8–30.0)
Palestine	50 to 54	11.9 (3.0–29.6)	11.6 (3.5–26.1)	11.6 (3.7–25.2)	11.9 (3.7–28.6)	12.0 (3.6–29.4)
Palestine	55 to 59	11.3 (2.9–28.4)	11.0 (3.3–25.1)	11.0 (3.5–24.2)	11.4 (3.6–27.4)	11.5 (3.5–27.9)
Palestine	60 to 64	11.0 (2.9–27.7)	10.7 (3.2–24.4)	10.7 (3.4–23.5)	11.0 (3.4–26.8)	11.1 (3.4–27.1)
Palestine	65 to 69	10.2 (2.6–26.0)	9.9 (2.9–22.9)	9.9 (3.1–22.0)	10.2 (3.2–25.1)	10.3 (3.1–25.5)
Palestine	70 to 74	8.1 (2.0–20.7)	7.9 (2.3–18.6)	7.9 (2.4–17.9)	8.1 (2.4–20.6)	8.2 (2.4–21.3)
Palestine	75 to 79	7.3 (1.8–19.3)	7.1 (2.0–17.0)	7.1 (2.1–16.2)	7.3 (2.1–18.4)	7.4 (2.1–18.9)
Palestine	80 to 84	5.1 (1.2–13.8)	5.0 (1.4–12.1)	5.0 (1.5–11.6)	5.1 (1.5–13.5)	5.2 (1.5–13.9)
Palestine	85 to 89	4.6 (1.1–12.8)	4.5 (1.2–11.0)	4.4 (1.3–10.5)	4.6 (1.3–12.2)	4.7 (1.2–12.7)
Palestine	90 to 94	4.2 (1.0–11.5)	4.0 (1.1– 9.9)	4.0 (1.2– 9.5)	4.1 (1.2–11.1)	4.2 (1.1–11.4)
Palestine	95 plus	4.1 (1.0–11.4)	4.0 (1.1– 9.8)	4.0 (1.2– 9.4)	4.1 (1.2–11.1)	4.2 (1.1–11.5)
Palestine	Age-standardized	11.8 (3.1–29.4)	11.5 (3.5–26.0)	11.5 (3.7–25.1)	11.8 (3.7–28.3)	12.0 (3.5–29.2)
Palestine	All age	12.1 (3.2–30.0)	11.9 (3.6–26.8)	12.0 (3.8–25.9)	12.3 (3.8–29.2)	12.4 (3.7–30.1)
Qatar	20 to 24	12.5 (3.3–30.9)	12.2 (3.7–27.4)	12.2 (3.9–26.4)	12.5 (3.9–29.6)	12.7 (3.8–30.7)
Qatar	25 to 29	12.6 (3.3–30.9)	12.2 (3.7–27.5)	12.2 (3.9–26.5)	12.6 (3.9–30.3)	12.7 (3.8–31.1)

Supplementary Table S10: Prevalence of female SVAC by age and location for 1990, 2000, 2010, 2020, and 2023						
**Location**	**Age Range**	**1990**	**2000**	**2010**	**2020**	**2023**
Qatar	30 to 34	13.2 (3.5–32.3)	12.9 (4.0–28.7)	12.9 (4.2–27.7)	13.2 (4.2–31.3)	13.4 (4.0–32.2)
Qatar	35 to 39	13.0 (3.4–32.1)	12.7 (3.9–28.3)	12.7 (4.1–27.3)	13.0 (4.1–30.9)	13.1 (3.9–31.9)
Qatar	40 to 44	13.0 (3.5–31.9)	12.7 (3.9–28.4)	12.7 (4.1–27.4)	13.1 (4.1–31.0)	13.2 (3.9–31.8)
Qatar	45 to 49	12.4 (3.2–30.2)	12.1 (3.7–27.1)	12.1 (3.9–26.1)	12.4 (3.9–29.6)	12.5 (3.8–30.0)
Qatar	50 to 54	11.9 (3.0–29.6)	11.6 (3.5–26.1)	11.6 (3.7–25.2)	11.9 (3.7–28.6)	12.0 (3.6–29.4)
Qatar	55 to 59	11.3 (2.9–28.4)	11.0 (3.3–25.1)	11.0 (3.5–24.2)	11.4 (3.6–27.4)	11.5 (3.5–27.9)
Qatar	60 to 64	11.0 (2.9–27.7)	10.7 (3.2–24.4)	10.7 (3.4–23.5)	11.0 (3.4–26.8)	11.1 (3.4–27.1)
Qatar	65 to 69	10.2 (2.6–26.0)	9.9 (2.9–22.9)	9.9 (3.1–22.0)	10.2 (3.2–25.1)	10.3 (3.1–25.5)
Qatar	70 to 74	8.1 (2.0–20.7)	7.9 (2.3–18.6)	7.9 (2.4–17.9)	8.1 (2.4–20.6)	8.2 (2.4–21.3)
Qatar	75 to 79	7.3 (1.8–19.3)	7.1 (2.0–17.0)	7.1 (2.1–16.2)	7.3 (2.1–18.4)	7.4 (2.1–18.9)
Qatar	80 to 84	5.1 (1.2–13.8)	5.0 (1.4–12.1)	5.0 (1.5–11.6)	5.1 (1.5–13.5)	5.2 (1.5–13.9)
Qatar	85 to 89	4.6 (1.1–12.8)	4.5 (1.2–11.0)	4.4 (1.3–10.5)	4.6 (1.3–12.2)	4.7 (1.2–12.7)
Qatar	90 to 94	4.2 (1.0–11.5)	4.0 (1.1– 9.9)	4.0 (1.2– 9.5)	4.1 (1.2–11.1)	4.2 (1.1–11.4)
Qatar	95 plus	4.1 (1.0–11.4)	4.0 (1.1– 9.8)	4.0 (1.2– 9.4)	4.1 (1.2–11.1)	4.2 (1.1–11.5)
Qatar	Age-standardized	11.8 (3.1–29.4)	11.5 (3.5–26.0)	11.5 (3.7–25.1)	11.8 (3.7–28.3)	12.0 (3.5–29.2)

Supplementary Table S10: Prevalence of female SVAC by age and location for 1990, 2000, 2010, 2020, and 2023						
**Location**	**Age Range**	**1990**	**2000**	**2010**	**2020**	**2023**
Qatar	All age	12.6 (3.3–31.1)	12.3 (3.8–27.6)	12.3 (4.0–26.6)	12.6 (4.0–30.0)	12.7 (3.8–30.9)
Saudi Arabia	20 to 24	12.5 (3.3–30.9)	12.2 (3.7–27.4)	12.2 (3.9–26.4)	12.5 (3.9–29.6)	12.7 (3.8–30.7)
Saudi Arabia	25 to 29	12.6 (3.3–30.9)	12.2 (3.7–27.5)	12.2 (3.9–26.5)	12.6 (3.9–30.3)	12.7 (3.8–31.1)
Saudi Arabia	30 to 34	13.2 (3.5–32.3)	12.9 (4.0–28.7)	12.9 (4.2–27.7)	13.2 (4.2–31.3)	13.4 (4.0–32.2)
Saudi Arabia	35 to 39	13.0 (3.4–32.1)	12.7 (3.9–28.3)	12.7 (4.1–27.3)	13.0 (4.1–30.9)	13.1 (3.9–31.9)
Saudi Arabia	40 to 44	13.0 (3.5–31.9)	12.7 (3.9–28.4)	12.7 (4.1–27.4)	13.1 (4.1–31.0)	13.2 (3.9–31.8)
Saudi Arabia	45 to 49	12.4 (3.2–30.2)	12.1 (3.7–27.1)	12.1 (3.9–26.1)	12.4 (3.9–29.6)	12.5 (3.8–30.0)
Saudi Arabia	50 to 54	11.9 (3.0–29.6)	11.6 (3.5–26.1)	11.6 (3.7–25.2)	11.9 (3.7–28.6)	12.0 (3.6–29.4)
Saudi Arabia	55 to 59	11.3 (2.9–28.4)	11.0 (3.3–25.1)	11.0 (3.5–24.2)	11.4 (3.6–27.4)	11.5 (3.5–27.9)
Saudi Arabia	60 to 64	11.0 (2.9–27.7)	10.7 (3.2–24.4)	10.7 (3.4–23.5)	11.0 (3.4–26.8)	11.1 (3.4–27.1)
Saudi Arabia	65 to 69	10.2 (2.6–26.0)	9.9 (2.9–22.9)	9.9 (3.1–22.0)	10.2 (3.2–25.1)	10.3 (3.1–25.5)
Saudi Arabia	70 to 74	8.1 (2.0–20.7)	7.9 (2.3–18.6)	7.9 (2.4–17.9)	8.1 (2.4–20.6)	8.2 (2.4–21.3)
Saudi Arabia	75 to 79	7.3 (1.8–19.3)	7.1 (2.0–17.0)	7.1 (2.1–16.2)	7.3 (2.1–18.4)	7.4 (2.1–18.9)
Saudi Arabia	80 to 84	5.1 (1.2–13.8)	5.0 (1.4–12.1)	5.0 (1.5–11.6)	5.1 (1.5–13.5)	5.2 (1.5–13.9)
Saudi Arabia	85 to 89	4.6 (1.1–12.8)	4.5 (1.2–11.0)	4.4 (1.3–10.5)	4.6 (1.3–12.2)	4.7 (1.2–12.7)

Supplementary Table S10: Prevalence of female SVAC by age and location for 1990, 2000, 2010, 2020, and 2023						
**Location**	**Age Range**	**1990**	**2000**	**2010**	**2020**	**2023**
Saudi Arabia	90 to 94	4.2 (1.0–11.5)	4.0 (1.1– 9.9)	4.0 (1.2– 9.5)	4.1 (1.2–11.1)	4.2 (1.1–11.4)
Saudi Arabia	95 plus	4.1 (1.0–11.4)	4.0 (1.1– 9.8)	4.0 (1.2– 9.4)	4.1 (1.2–11.1)	4.2 (1.1–11.5)
Saudi Arabia	Age-standardized	11.8 (3.1–29.4)	11.5 (3.5–26.0)	11.5 (3.7–25.1)	11.8 (3.7–28.3)	12.0 (3.5–29.2)
Saudi Arabia	All age	12.4 (3.3–30.5)	12.1 (3.7–27.1)	12.2 (3.9–26.3)	12.5 (3.9–29.6)	12.5 (3.7–30.5)
Sudan	20 to 24	12.5 (3.3–30.9)	12.2 (3.7–27.4)	12.2 (3.9–26.4)	12.5 (3.9–29.6)	12.7 (3.8–30.7)
Sudan	25 to 29	12.6 (3.3–30.9)	12.2 (3.7–27.5)	12.2 (3.9–26.5)	12.6 (3.9–30.3)	12.7 (3.8–31.1)
Sudan	30 to 34	13.2 (3.5–32.3)	12.9 (4.0–28.7)	12.9 (4.2–27.7)	13.2 (4.2–31.3)	13.4 (4.0–32.2)
Sudan	35 to 39	13.0 (3.4–32.1)	12.7 (3.9–28.3)	12.7 (4.1–27.3)	13.0 (4.1–30.9)	13.1 (3.9–31.9)
Sudan	40 to 44	13.0 (3.5–31.9)	12.7 (3.9–28.4)	12.7 (4.1–27.4)	13.1 (4.1–31.0)	13.2 (3.9–31.8)
Sudan	45 to 49	12.4 (3.2–30.2)	12.1 (3.7–27.1)	12.1 (3.9–26.1)	12.4 (3.9–29.6)	12.5 (3.8–30.0)
Sudan	50 to 54	11.9 (3.0–29.6)	11.6 (3.5–26.1)	11.6 (3.7–25.2)	11.9 (3.7–28.6)	12.0 (3.6–29.4)
Sudan	55 to 59	11.3 (2.9–28.4)	11.0 (3.3–25.1)	11.0 (3.5–24.2)	11.4 (3.6–27.4)	11.5 (3.5–27.9)
Sudan	60 to 64	11.0 (2.9–27.7)	10.7 (3.2–24.4)	10.7 (3.4–23.5)	11.0 (3.4–26.8)	11.1 (3.4–27.1)
Sudan	65 to 69	10.2 (2.6–26.0)	9.9 (2.9–22.9)	9.9 (3.1–22.0)	10.2 (3.2–25.1)	10.3 (3.1–25.5)
Sudan	70 to 74	8.1 (2.0–20.7)	7.9 (2.3–18.6)	7.9 (2.4–17.9)	8.1 (2.4–20.6)	8.2 (2.4–21.3)

Supplementary Table S10: Prevalence of female SVAC by age and location for 1990, 2000, 2010, 2020, and 2023						
**Location**	**Age Range**	**1990**	**2000**	**2010**	**2020**	**2023**
Sudan	75 to 79	7.3 (1.8–19.3)	7.1 (2.0–17.0)	7.1 (2.1–16.2)	7.3 (2.1–18.4)	7.4 (2.1–18.9)
Sudan	80 to 84	5.1 (1.2–13.8)	5.0 (1.4–12.1)	5.0 (1.5–11.6)	5.1 (1.5–13.5)	5.2 (1.5–13.9)
Sudan	85 to 89	4.6 (1.1–12.8)	4.5 (1.2–11.0)	4.4 (1.3–10.5)	4.6 (1.3–12.2)	4.7 (1.2–12.7)
Sudan	90 to 94	4.2 (1.0–11.5)	4.0 (1.1– 9.9)	4.0 (1.2– 9.5)	4.1 (1.2–11.1)	4.2 (1.1–11.4)
Sudan	95 plus	4.1 (1.0–11.4)	4.0 (1.1– 9.8)	4.0 (1.2– 9.4)	4.1 (1.2–11.1)	4.2 (1.1–11.5)
Sudan	Age-standardized	11.8 (3.1–29.4)	11.5 (3.5–26.0)	11.5 (3.7–25.1)	11.8 (3.7–28.3)	12.0 (3.5–29.2)
Sudan	All age	12.3 (3.2–30.3)	11.9 (3.6–26.8)	11.9 (3.8–25.7)	12.2 (3.8–28.9)	12.3 (3.6–29.8)
Syria	20 to 24	12.5 (3.3–30.9)	12.2 (3.7–27.4)	12.2 (3.9–26.4)	12.5 (3.9–29.6)	12.7 (3.8–30.7)
Syria	25 to 29	12.6 (3.3–30.9)	12.2 (3.7–27.5)	12.2 (3.9–26.5)	12.6 (3.9–30.3)	12.7 (3.8–31.1)
Syria	30 to 34	13.2 (3.5–32.3)	12.9 (4.0–28.7)	12.9 (4.2–27.7)	13.2 (4.2–31.3)	13.4 (4.0–32.2)
Syria	35 to 39	13.0 (3.4–32.1)	12.7 (3.9–28.3)	12.7 (4.1–27.3)	13.0 (4.1–30.9)	13.1 (3.9–31.9)
Syria	40 to 44	13.0 (3.5–31.9)	12.7 (3.9–28.4)	12.7 (4.1–27.4)	13.1 (4.1–31.0)	13.2 (3.9–31.8)
Syria	45 to 49	12.4 (3.2–30.2)	12.1 (3.7–27.1)	12.1 (3.9–26.1)	12.4 (3.9–29.6)	12.5 (3.8–30.0)
Syria	50 to 54	11.9 (3.0–29.6)	11.6 (3.5–26.1)	11.6 (3.7–25.2)	11.9 (3.7–28.6)	12.0 (3.6–29.4)
Syria	55 to 59	11.3 (2.9–28.4)	11.0 (3.3–25.1)	11.0 (3.5–24.2)	11.4 (3.6–27.4)	11.5 (3.5–27.9)

Supplementary Table S10: Prevalence of female SVAC by age and location for 1990, 2000, 2010, 2020, and 2023						
**Location**	**Age Range**	**1990**	**2000**	**2010**	**2020**	**2023**
Syria	60 to 64	11.0 (2.9–27.7)	10.7 (3.2–24.4)	10.7 (3.4–23.5)	11.0 (3.4–26.8)	11.1 (3.4–27.1)
Syria	65 to 69	10.2 (2.6–26.0)	9.9 (2.9–22.9)	9.9 (3.1–22.0)	10.2 (3.2–25.1)	10.3 (3.1–25.5)
Syria	70 to 74	8.1 (2.0–20.7)	7.9 (2.3–18.6)	7.9 (2.4–17.9)	8.1 (2.4–20.6)	8.2 (2.4–21.3)
Syria	75 to 79	7.3 (1.8–19.3)	7.1 (2.0–17.0)	7.1 (2.1–16.2)	7.3 (2.1–18.4)	7.4 (2.1–18.9)
Syria	80 to 84	5.1 (1.2–13.8)	5.0 (1.4–12.1)	5.0 (1.5–11.6)	5.1 (1.5–13.5)	5.2 (1.5–13.9)
Syria	85 to 89	4.6 (1.1–12.8)	4.5 (1.2–11.0)	4.4 (1.3–10.5)	4.6 (1.3–12.2)	4.7 (1.2–12.7)
Syria	90 to 94	4.2 (1.0–11.5)	4.0 (1.1– 9.9)	4.0 (1.2– 9.5)	4.1 (1.2–11.1)	4.2 (1.1–11.4)
Syria	95 plus	4.1 (1.0–11.4)	4.0 (1.1– 9.8)	4.0 (1.2– 9.4)	4.1 (1.2–11.1)	4.2 (1.1–11.5)
Syria	Age-standardized	11.8 (3.1–29.4)	11.5 (3.5–26.0)	11.5 (3.7–25.1)	11.8 (3.7–28.3)	12.0 (3.5–29.2)
Syria	All age	12.3 (3.2–30.3)	12.0 (3.7–27.0)	12.0 (3.9–26.0)	12.1 (3.8–28.8)	12.1 (3.6–29.5)
Tunisia	20 to 24	12.5 (3.3–30.9)	12.2 (3.7–27.4)	12.2 (3.9–26.4)	12.5 (3.9–29.6)	12.7 (3.8–30.7)
Tunisia	25 to 29	12.6 (3.3–30.9)	12.2 (3.7–27.5)	12.2 (3.9–26.5)	12.6 (3.9–30.3)	12.7 (3.8–31.1)
Tunisia	30 to 34	13.2 (3.5–32.3)	12.9 (4.0–28.7)	12.9 (4.2–27.7)	13.2 (4.2–31.3)	13.4 (4.0–32.2)
Tunisia	35 to 39	13.0 (3.4–32.1)	12.7 (3.9–28.3)	12.7 (4.1–27.3)	13.0 (4.1–30.9)	13.1 (3.9–31.9)
Tunisia	40 to 44	13.0 (3.5–31.9)	12.7 (3.9–28.4)	12.7 (4.1–27.4)	13.1 (4.1–31.0)	13.2 (3.9–31.8)

Supplementary Table S10: Prevalence of female SVAC by age and location for 1990, 2000, 2010, 2020, and 2023						
**Location**	**Age Range**	**1990**	**2000**	**2010**	**2020**	**2023**
Tunisia	45 to 49	12.4 (3.2–30.2)	12.1 (3.7–27.1)	12.1 (3.9–26.1)	12.4 (3.9–29.6)	12.5 (3.8–30.0)
Tunisia	50 to 54	11.9 (3.0–29.6)	11.6 (3.5–26.1)	11.6 (3.7–25.2)	11.9 (3.7–28.6)	12.0 (3.6–29.4)
Tunisia	55 to 59	11.3 (2.9–28.4)	11.0 (3.3–25.1)	11.0 (3.5–24.2)	11.4 (3.6–27.4)	11.5 (3.5–27.9)
Tunisia	60 to 64	11.0 (2.9–27.7)	10.7 (3.2–24.4)	10.7 (3.4–23.5)	11.0 (3.4–26.8)	11.1 (3.4–27.1)
Tunisia	65 to 69	10.2 (2.6–26.0)	9.9 (2.9–22.9)	9.9 (3.1–22.0)	10.2 (3.2–25.1)	10.3 (3.1–25.5)
Tunisia	70 to 74	8.1 (2.0–20.7)	7.9 (2.3–18.6)	7.9 (2.4–17.9)	8.1 (2.4–20.6)	8.2 (2.4–21.3)
Tunisia	75 to 79	7.3 (1.8–19.3)	7.1 (2.0–17.0)	7.1 (2.1–16.2)	7.3 (2.1–18.4)	7.4 (2.1–18.9)
Tunisia	80 to 84	5.1 (1.2–13.8)	5.0 (1.4–12.1)	5.0 (1.5–11.6)	5.1 (1.5–13.5)	5.2 (1.5–13.9)
Tunisia	85 to 89	4.6 (1.1–12.8)	4.5 (1.2–11.0)	4.4 (1.3–10.5)	4.6 (1.3–12.2)	4.7 (1.2–12.7)
Tunisia	90 to 94	4.2 (1.0–11.5)	4.0 (1.1– 9.9)	4.0 (1.2– 9.5)	4.1 (1.2–11.1)	4.2 (1.1–11.4)
Tunisia	95 plus	4.1 (1.0–11.4)	4.0 (1.1– 9.8)	4.0 (1.2– 9.4)	4.1 (1.2–11.1)	4.2 (1.1–11.5)
Tunisia	Age-standardized	11.8 (3.1–29.4)	11.5 (3.5–26.0)	11.5 (3.7–25.1)	11.8 (3.7–28.3)	12.0 (3.5–29.2)
Tunisia	All age	12.2 (3.2–30.1)	11.8 (3.6–26.6)	11.7 (3.8–25.5)	11.9 (3.7–28.4)	11.9 (3.5–29.1)
Türkiye	20 to 24	16.7 (8.1–27.8)	16.5 (9.2–25.9)	16.5 (11.7–22.0)	16.1 (9.6–25.3)	16.1 (8.9–26.2)
Türkiye	25 to 29	12.2 (5.4–22.4)	12.5 (6.4–21.0)	13.8 (9.1–19.5)	15.6 (8.6–24.4)	15.9 (8.4–25.7)

Supplementary Table S10: Prevalence of female SVAC by age and location for 1990, 2000, 2010, 2020, and 2023						
**Location**	**Age Range**	**1990**	**2000**	**2010**	**2020**	**2023**
Türkiye	30 to 34	12.6 (5.6–22.8)	12.9 (6.6–21.4)	14.0 (9.4–19.7)	15.8 (8.8–24.8)	16.1 (8.4–26.0)
Türkiye	35 to 39	10.8 (4.4–21.4)	11.0 (5.2–19.5)	11.9 (7.4–17.5)	13.7 (7.5–21.8)	14.0 (7.3–23.5)
Türkiye	40 to 44	10.7 (4.4–21.2)	10.9 (5.2–19.4)	11.8 (7.3–17.4)	13.6 (7.5–21.7)	14.0 (7.2–23.3)
Türkiye	45 to 49	8.7 (3.2–18.3)	8.5 (3.6–16.1)	8.4 (4.3–14.3)	9.1 (4.1–16.8)	9.2 (3.8–17.7)
Türkiye	50 to 54	8.7 (3.3–18.2)	8.5 (3.7–15.9)	8.5 (4.4–14.3)	9.1 (4.2–16.7)	9.3 (4.0–17.6)
Türkiye	55 to 59	7.9 (2.8–17.0)	7.8 (3.1–15.0)	7.6 (3.7–13.6)	8.2 (3.6–15.8)	8.3 (3.4–16.6)
Türkiye	60 to 64	10.6 (2.7–26.9)	10.5 (2.9–24.8)	10.5 (3.3–23.1)	10.9 (3.2–26.0)	11.0 (3.2–27.3)
Türkiye	65 to 69	9.9 (2.5–25.3)	9.7 (2.7–23.2)	9.7 (3.0–21.6)	10.1 (3.0–24.4)	10.2 (3.0–25.7)
Türkiye	70 to 74	7.8 (1.9–20.6)	7.7 (2.1–18.9)	7.7 (2.4–17.5)	8.0 (2.3–19.9)	8.1 (2.3–21.0)
Türkiye	75 to 79	7.0 (1.7–18.7)	6.9 (1.9–17.1)	6.9 (2.1–15.8)	7.2 (2.1–18.1)	7.3 (2.1–19.1)
Türkiye	80 to 84	4.9 (1.2–13.6)	4.9 (1.3–12.3)	4.8 (1.4–11.3)	5.0 (1.4–13.0)	5.1 (1.4–13.8)
Türkiye	85 to 89	4.4 (1.0–12.3)	4.4 (1.1–11.1)	4.3 (1.3–10.2)	4.5 (1.3–11.8)	4.6 (1.3–12.5)
Türkiye	90 to 94	4.0 (0.9–11.1)	3.9 (1.0–10.1)	3.9 (1.1– 9.3)	4.1 (1.1–10.7)	4.1 (1.1–11.3)
Türkiye	95 plus	4.0 (0.9–11.0)	3.9 (1.0–10.0)	3.9 (1.1– 9.2)	4.0 (1.1–10.6)	4.1 (1.1–11.2)
Türkiye	Age-standardized	10.9 (4.5–21.0)	10.9 (5.3–19.3)	11.4 (7.6–16.5)	12.3 (8.1–19.3)	12.5 (7.9–20.3)

Supplementary Table S10: Prevalence of female SVAC by age and location for 1990, 2000, 2010, 2020, and 2023						
**Location**	**Age Range**	**1990**	**2000**	**2010**	**2020**	**2023**
Türkiye	All age	11.5 (4.9–21.8)	11.4 (5.6–19.6)	11.5 (7.7–16.6)	12.1 (7.8–19.3)	12.2 (7.3–20.4)
United Arab Emirates	20 to 24	12.5 (3.3–30.9)	12.2 (3.7–27.4)	12.2 (3.9–26.4)	12.5 (3.9–29.6)	12.7 (3.8–30.7)
United Arab Emirates	25 to 29	12.6 (3.3–30.9)	12.2 (3.7–27.5)	12.2 (3.9–26.5)	12.6 (3.9–30.3)	12.7 (3.8–31.1)
United Arab Emirates	30 to 34	13.2 (3.5–32.3)	12.9 (4.0–28.7)	12.9 (4.2–27.7)	13.2 (4.2–31.3)	13.4 (4.0–32.2)
United Arab Emirates	35 to 39	13.0 (3.4–32.1)	12.7 (3.9–28.3)	12.7 (4.1–27.3)	13.0 (4.1–30.9)	13.1 (3.9–31.9)
United Arab Emirates	40 to 44	13.0 (3.5–31.9)	12.7 (3.9–28.4)	12.7 (4.1–27.4)	13.1 (4.1–31.0)	13.2 (3.9–31.8)
United Arab Emirates	45 to 49	12.4 (3.2–30.2)	12.1 (3.7–27.1)	12.1 (3.9–26.1)	12.4 (3.9–29.6)	12.5 (3.8–30.0)
United Arab Emirates	50 to 54	11.9 (3.0–29.6)	11.6 (3.5–26.1)	11.6 (3.7–25.2)	11.9 (3.7–28.6)	12.0 (3.6–29.4)
United Arab Emirates	55 to 59	11.3 (2.9–28.4)	11.0 (3.3–25.1)	11.0 (3.5–24.2)	11.4 (3.6–27.4)	11.5 (3.5–27.9)
United Arab Emirates	60 to 64	11.0 (2.9–27.7)	10.7 (3.2–24.4)	10.7 (3.4–23.5)	11.0 (3.4–26.8)	11.1 (3.4–27.1)
United Arab Emirates	65 to 69	10.2 (2.6–26.0)	9.9 (2.9–22.9)	9.9 (3.1–22.0)	10.2 (3.2–25.1)	10.3 (3.1–25.5)
United Arab Emirates	70 to 74	8.1 (2.0–20.7)	7.9 (2.3–18.6)	7.9 (2.4–17.9)	8.1 (2.4–20.6)	8.2 (2.4–21.3)
United Arab Emirates	75 to 79	7.3 (1.8–19.3)	7.1 (2.0–17.0)	7.1 (2.1–16.2)	7.3 (2.1–18.4)	7.4 (2.1–18.9)
United Arab Emirates	80 to 84	5.1 (1.2–13.8)	5.0 (1.4–12.1)	5.0 (1.5–11.6)	5.1 (1.5–13.5)	5.2 (1.5–13.9)
United Arab Emirates	85 to 89	4.6 (1.1–12.8)	4.5 (1.2–11.0)	4.4 (1.3–10.5)	4.6 (1.3–12.2)	4.7 (1.2–12.7)

Supplementary Table S10: Prevalence of female SVAC by age and location for 1990, 2000, 2010, 2020, and 2023						
**Location**	**Age Range**	**1990**	**2000**	**2010**	**2020**	**2023**
United Arab Emirates	90 to 94	4.2 (1.0–11.5)	4.0 (1.1– 9.9)	4.0 (1.2– 9.5)	4.1 (1.2–11.1)	4.2 (1.1–11.4)
United Arab Emirates	95 plus	4.1 (1.0–11.4)	4.0 (1.1– 9.8)	4.0 (1.2– 9.4)	4.1 (1.2–11.1)	4.2 (1.1–11.5)
United Arab Emirates	Age-standardized	11.8 (3.1–29.4)	11.5 (3.5–26.0)	11.5 (3.7–25.1)	11.8 (3.7–28.3)	12.0 (3.5–29.2)
United Arab Emirates	All age	12.6 (3.3–31.0)	12.3 (3.8–27.6)	12.4 (4.0–26.7)	12.6 (3.9–29.9)	12.6 (3.7–30.6)
Yemen	20 to 24	12.5 (3.3–30.9)	12.2 (3.7–27.4)	12.2 (3.9–26.4)	12.5 (3.9–29.6)	12.7 (3.8–30.7)
Yemen	25 to 29	12.6 (3.3–30.9)	12.2 (3.7–27.5)	12.2 (3.9–26.5)	12.6 (3.9–30.3)	12.7 (3.8–31.1)
Yemen	30 to 34	13.2 (3.5–32.3)	12.9 (4.0–28.7)	12.9 (4.2–27.7)	13.2 (4.2–31.3)	13.4 (4.0–32.2)
Yemen	35 to 39	13.0 (3.4–32.1)	12.7 (3.9–28.3)	12.7 (4.1–27.3)	13.0 (4.1–30.9)	13.1 (3.9–31.9)
Yemen	40 to 44	13.0 (3.5–31.9)	12.7 (3.9–28.4)	12.7 (4.1–27.4)	13.1 (4.1–31.0)	13.2 (3.9–31.8)
Yemen	45 to 49	12.4 (3.2–30.2)	12.1 (3.7–27.1)	12.1 (3.9–26.1)	12.4 (3.9–29.6)	12.5 (3.8–30.0)
Yemen	50 to 54	11.9 (3.0–29.6)	11.6 (3.5–26.1)	11.6 (3.7–25.2)	11.9 (3.7–28.6)	12.0 (3.6–29.4)
Yemen	55 to 59	11.3 (2.9–28.4)	11.0 (3.3–25.1)	11.0 (3.5–24.2)	11.4 (3.6–27.4)	11.5 (3.5–27.9)
Yemen	60 to 64	11.0 (2.9–27.7)	10.7 (3.2–24.4)	10.7 (3.4–23.5)	11.0 (3.4–26.8)	11.1 (3.4–27.1)
Yemen	65 to 69	10.2 (2.6–26.0)	9.9 (2.9–22.9)	9.9 (3.1–22.0)	10.2 (3.2–25.1)	10.3 (3.1–25.5)
Yemen	70 to 74	8.1 (2.0–20.7)	7.9 (2.3–18.6)	7.9 (2.4–17.9)	8.1 (2.4–20.6)	8.2 (2.4–21.3)

Supplementary Table S10: Prevalence of female SVAC by age and location for 1990, 2000, 2010, 2020, and 2023						
**Location**	**Age Range**	**1990**	**2000**	**2010**	**2020**	**2023**
Yemen	75 to 79	7.3 (1.8–19.3)	7.1 (2.0–17.0)	7.1 (2.1–16.2)	7.3 (2.1–18.4)	7.4 (2.1–18.9)
Yemen	80 to 84	5.1 (1.2–13.8)	5.0 (1.4–12.1)	5.0 (1.5–11.6)	5.1 (1.5–13.5)	5.2 (1.5–13.9)
Yemen	85 to 89	4.6 (1.1–12.8)	4.5 (1.2–11.0)	4.4 (1.3–10.5)	4.6 (1.3–12.2)	4.7 (1.2–12.7)
Yemen	90 to 94	4.2 (1.0–11.5)	4.0 (1.1– 9.9)	4.0 (1.2– 9.5)	4.1 (1.2–11.1)	4.2 (1.1–11.4)
Yemen	95 plus	4.1 (1.0–11.4)	4.0 (1.1– 9.8)	4.0 (1.2– 9.4)	4.1 (1.2–11.1)	4.2 (1.1–11.5)
Yemen	Age-standardized	11.8 (3.1–29.4)	11.5 (3.5–26.0)	11.5 (3.7–25.1)	11.8 (3.7–28.3)	12.0 (3.5–29.2)
Yemen	All age	12.3 (3.2–30.4)	12.0 (3.6–26.9)	12.0 (3.9–26.0)	12.4 (3.9–29.4)	12.5 (3.7–30.3)
South Asia	20 to 24	25.6 (10.6–45.7)	24.4 (12.1–40.1)	23.2 (16.3–31.0)	22.9 (17.4–29.9)	23.0 (16.3–32.5)
South Asia	25 to 29	27.4 (11.4–48.1)	26.6 (13.5–42.8)	26.0 (18.8–34.1)	25.5 (19.7–32.7)	25.5 (18.3–35.3)
South Asia	30 to 34	28.9 (12.2–50.1)	28.0 (14.3–44.9)	26.6 (19.3–34.8)	26.2 (20.1–33.6)	26.2 (18.9–36.2)
South Asia	35 to 39	31.2 (13.4–52.8)	31.6 (16.5–49.3)	31.6 (23.5–40.4)	30.9 (23.8–39.2)	30.5 (21.9–41.3)
South Asia	40 to 44	31.5 (13.6–53.5)	31.7 (16.6–49.4)	31.7 (23.5–40.6)	31.1 (23.8–39.5)	30.9 (22.2–41.9)
South Asia	45 to 49	30.9 (13.2–52.7)	31.0 (16.1–48.8)	30.8 (22.8–39.4)	30.2 (23.1–38.5)	30.0 (21.2–41.1)
South Asia	50 to 54	29.5 (10.8–54.0)	29.1 (11.4–52.3)	29.0 (12.1–50.5)	29.2 (13.2–52.2)	29.2 (13.5–51.7)
South Asia	55 to 59	28.9 (10.5–53.3)	28.5 (11.0–51.6)	28.4 (11.6–50.0)	28.5 (12.7–51.5)	28.5 (12.9–51.0)

Supplementary Table S10: Prevalence of female SVAC by age and location for 1990, 2000, 2010, 2020, and 2023						
**Location**	**Age Range**	**1990**	**2000**	**2010**	**2020**	**2023**
South Asia	60 to 64	28.1 (10.0–52.4)	28.2 (10.8–51.4)	27.9 (11.3–49.4)	27.9 (12.3–50.9)	28.0 (12.6–50.6)
South Asia	65 to 69	26.4 (9.1–51.4)	26.8 (10.0–49.9)	26.6 (10.2–48.8)	26.9 (10.8–51.2)	26.7 (10.9–50.5)
South Asia	70 to 74	21.8 (7.1–44.1)	22.1 (7.8–43.1)	22.4 (8.2–42.9)	22.5 (8.6–45.1)	22.5 (8.7–44.7)
South Asia	75 to 79	19.8 (6.2–41.0)	20.2 (7.0–40.3)	20.6 (7.4–40.3)	20.6 (7.7–42.3)	20.7 (7.8–42.0)
South Asia	80 to 84	14.8 (4.4–32.3)	14.8 (4.8–31.3)	15.2 (5.1–31.5)	15.4 (5.4–33.7)	15.3 (5.5–33.2)
South Asia	85 to 89	13.2 (3.8–29.5)	13.2 (4.2–28.5)	13.8 (4.6–29.1)	14.0 (4.9–31.2)	14.1 (4.9–30.9)
South Asia	90 to 94	12.0 (3.4–27.3)	12.2 (3.8–26.5)	12.5 (4.1–26.7)	12.7 (4.3–28.7)	12.8 (4.4–28.6)
South Asia	95 plus	11.8 (3.4–26.8)	11.6 (3.6–25.5)	12.2 (4.0–26.0)	12.4 (4.2–28.2)	12.6 (4.4–28.2)
South Asia	Age-standardized	27.9 (11.8–48.9)	27.6 (13.4–45.5)	27.2 (17.1–39.8)	26.9 (21.7–33.9)	26.8 (21.9–32.7)
South Asia	All age	28.2 (12.0–49.0)	27.8 (13.7–45.1)	27.3 (17.7–38.6)	27.0 (22.1–32.9)	27.0 (22.0–32.7)
South Asia	20 to 24	25.6 (10.6–45.7)	24.4 (12.1–40.1)	23.2 (16.3–31.0)	22.9 (17.4–29.9)	23.0 (16.3–32.5)
South Asia	25 to 29	27.4 (11.4–48.1)	26.6 (13.5–42.8)	26.0 (18.8–34.1)	25.5 (19.7–32.7)	25.5 (18.3–35.3)
South Asia	30 to 34	28.9 (12.2–50.1)	28.0 (14.3–44.9)	26.6 (19.3–34.8)	26.2 (20.1–33.6)	26.2 (18.9–36.2)
South Asia	35 to 39	31.2 (13.4–52.8)	31.6 (16.5–49.3)	31.6 (23.5–40.4)	30.9 (23.8–39.2)	30.5 (21.9–41.3)
South Asia	40 to 44	31.5 (13.6–53.5)	31.7 (16.6–49.4)	31.7 (23.5–40.6)	31.1 (23.8–39.5)	30.9 (22.2–41.9)

Supplementary Table S10: Prevalence of female SVAC by age and location for 1990, 2000, 2010, 2020, and 2023						
**Location**	**Age Range**	**1990**	**2000**	**2010**	**2020**	**2023**
South Asia	45 to 49	30.9 (13.2–52.7)	31.0 (16.1–48.8)	30.8 (22.8–39.4)	30.2 (23.1–38.5)	30.0 (21.2–41.1)
South Asia	50 to 54	29.5 (10.8–54.0)	29.1 (11.4–52.3)	29.0 (12.1–50.5)	29.2 (13.2–52.2)	29.2 (13.5–51.7)
South Asia	55 to 59	28.9 (10.5–53.3)	28.5 (11.0–51.6)	28.4 (11.6–50.0)	28.5 (12.7–51.5)	28.5 (12.9–51.0)
South Asia	60 to 64	28.1 (10.0–52.4)	28.2 (10.8–51.4)	27.9 (11.3–49.4)	27.9 (12.3–50.9)	28.0 (12.6–50.6)
South Asia	65 to 69	26.4 (9.1–51.4)	26.8 (10.0–49.9)	26.6 (10.2–48.8)	26.9 (10.8–51.2)	26.7 (10.9–50.5)
South Asia	70 to 74	21.8 (7.1–44.1)	22.1 (7.8–43.1)	22.4 (8.2–42.9)	22.5 (8.6–45.1)	22.5 (8.7–44.7)
South Asia	75 to 79	19.8 (6.2–41.0)	20.2 (7.0–40.3)	20.6 (7.4–40.3)	20.6 (7.7–42.3)	20.7 (7.8–42.0)
South Asia	80 to 84	14.8 (4.4–32.3)	14.8 (4.8–31.3)	15.2 (5.1–31.5)	15.4 (5.4–33.7)	15.3 (5.5–33.2)
South Asia	85 to 89	13.2 (3.8–29.5)	13.2 (4.2–28.5)	13.8 (4.6–29.1)	14.0 (4.9–31.2)	14.1 (4.9–30.9)
South Asia	90 to 94	12.0 (3.4–27.3)	12.2 (3.8–26.5)	12.5 (4.1–26.7)	12.7 (4.3–28.7)	12.8 (4.4–28.6)
South Asia	95 plus	11.8 (3.4–26.8)	11.6 (3.6–25.5)	12.2 (4.0–26.0)	12.4 (4.2–28.2)	12.6 (4.4–28.2)
South Asia	Age-standardized	27.9 (11.8–48.9)	27.6 (13.4–45.5)	27.2 (17.1–39.8)	26.9 (21.7–33.9)	26.8 (21.9–32.7)
South Asia	All age	28.2 (12.0–49.0)	27.8 (13.7–45.1)	27.3 (17.7–38.6)	27.0 (22.1–32.9)	27.0 (22.0–32.7)
Bangladesh	20 to 24	13.9 (9.5–19.7)	12.0 (9.6–14.6)	8.6 (7.3–10.1)	7.5 (4.3–11.9)	7.4 (3.9–12.5)
Bangladesh	25 to 29	14.5 (10.0–20.3)	12.7 (10.2–15.5)	9.7 (8.3–11.2)	8.5 (5.0–13.3)	8.4 (4.5–13.9)

Supplementary Table S10: Prevalence of female SVAC by age and location for 1990, 2000, 2010, 2020, and 2023						
**Location**	**Age Range**	**1990**	**2000**	**2010**	**2020**	**2023**
Bangladesh	30 to 34	15.8 (10.9–21.9)	13.6 (11.0–16.5)	9.9 (8.6–11.4)	8.6 (5.1–13.3)	8.5 (4.5–14.0)
Bangladesh	35 to 39	15.0 (10.4–21.1)	13.7 (11.0–16.7)	11.4 (10.1–12.9)	10.5 (6.3–16.1)	10.5 (5.6–17.1)
Bangladesh	40 to 44	14.4 (10.0–20.3)	13.3 (10.7–16.2)	11.3 (9.9–12.8)	10.7 (6.3–16.3)	10.6 (5.7–17.4)
Bangladesh	45 to 49	15.4 (10.8–21.7)	13.9 (11.1–16.9)	11.0 (9.7–12.5)	10.0 (5.9–15.4)	9.9 (5.3–16.4)
Bangladesh	50 to 54	12.5 (4.4–26.5)	11.9 (5.7–21.1)	11.2 (9.6–12.9)	11.5 (6.3–18.5)	11.6 (5.6–20.2)
Bangladesh	55 to 59	10.9 (3.9–23.7)	10.1 (4.7–18.1)	9.2 (7.7–10.8)	9.5 (5.2–15.7)	9.7 (4.6–17.2)
Bangladesh	60 to 64	10.7 (3.7–23.3)	10.0 (4.7–17.9)	9.1 (7.6–10.7)	9.4 (5.1–15.5)	9.5 (4.5–17.0)
Bangladesh	65 to 69	11.2 (2.9–28.0)	10.9 (3.3–24.7)	10.7 (3.4–23.6)	10.9 (3.3–26.6)	11.0 (3.2–27.5)
Bangladesh	70 to 74	9.0 (2.3–23.2)	8.6 (2.5–20.2)	8.5 (2.6–19.2)	8.7 (2.6–21.5)	8.8 (2.5–22.0)
Bangladesh	75 to 79	8.1 (2.0–20.5)	7.8 (2.2–18.5)	7.6 (2.3–17.4)	7.8 (2.3–19.9)	7.9 (2.2–20.2)
Bangladesh	80 to 84	5.7 (1.3–15.5)	5.5 (1.5–13.3)	5.4 (1.6–12.5)	5.5 (1.6–14.2)	5.6 (1.5–14.6)
Bangladesh	85 to 89	5.1 (1.2–13.9)	4.9 (1.4–12.0)	4.8 (1.4–11.3)	4.9 (1.4–13.1)	5.0 (1.3–13.3)
Bangladesh	90 to 94	4.6 (1.1–12.7)	4.4 (1.2–10.9)	4.3 (1.3–10.2)	4.4 (1.3–11.9)	4.5 (1.2–12.1)
Bangladesh	95 plus	4.6 (1.1–12.6)	4.4 (1.2–10.8)	4.3 (1.3–10.2)	4.4 (1.3–11.6)	4.5 (1.2–11.9)
Bangladesh	Age-standardized	13.3 (8.1–21.1)	12.0 (8.7–16.6)	9.9 (8.4–11.7)	9.3 (6.8–13.1)	9.3 (6.6–13.7)

Supplementary Table S10: Prevalence of female SVAC by age and location for 1990, 2000, 2010, 2020, and 2023						
**Location**	**Age Range**	**1990**	**2000**	**2010**	**2020**	**2023**
Bangladesh	All age	13.9 (9.1–20.8)	12.5 (9.5–16.0)	10.0 (8.7–11.5)	9.3 (6.7–13.0)	9.2 (6.4–13.7)
Bhutan	20 to 24	11.0 (3.8–22.9)	10.8 (4.1–21.6)	10.4 (4.8–19.0)	10.1 (5.3–17.3)	10.2 (5.0–17.8)
Bhutan	25 to 29	11.8 (4.2–24.5)	11.6 (4.5–23.1)	11.3 (5.4–20.4)	11.1 (5.9–18.4)	11.1 (5.5–18.9)
Bhutan	30 to 34	12.2 (4.4–25.3)	12.0 (4.7–23.8)	11.6 (5.6–20.7)	11.3 (6.1–18.6)	11.4 (5.7–19.3)
Bhutan	35 to 39	13.7 (5.2–28.0)	13.6 (5.6–26.1)	13.3 (6.7–23.2)	13.2 (7.4–20.8)	13.2 (6.9–21.5)
Bhutan	40 to 44	13.9 (5.3–28.4)	13.8 (5.7–26.5)	13.5 (6.8–23.5)	13.3 (7.5–21.1)	13.4 (7.0–21.8)
Bhutan	45 to 49	13.6 (5.1–27.7)	13.4 (5.5–25.8)	13.1 (6.6–22.9)	13.0 (7.2–20.5)	13.0 (6.8–21.2)
Bhutan	50 to 54	13.8 (5.3–28.0)	13.7 (5.7–26.2)	13.4 (6.9–23.2)	13.4 (7.6–20.8)	13.4 (7.1–21.8)
Bhutan	55 to 59	12.2 (4.4–25.2)	12.0 (4.7–23.8)	11.7 (5.6–21.0)	11.5 (6.1–19.0)	11.5 (5.7–19.4)
Bhutan	60 to 64	11.9 (4.4–24.9)	11.8 (4.7–23.3)	11.4 (5.6–20.4)	11.3 (6.2–18.4)	11.3 (5.7–18.9)
Bhutan	65 to 69	11.8 (3.2–27.5)	11.7 (3.4–27.0)	11.6 (3.6–25.7)	11.6 (3.7–27.9)	11.7 (3.6–28.4)
Bhutan	70 to 74	9.4 (2.5–22.6)	9.3 (2.7–22.2)	9.2 (2.8–21.0)	9.3 (2.9–23.0)	9.3 (2.8–23.4)
Bhutan	75 to 79	8.5 (2.2–20.6)	8.4 (2.4–20.2)	8.3 (2.5–19.1)	8.3 (2.6–20.9)	8.4 (2.5–21.3)
Bhutan	80 to 84	6.0 (1.5–15.0)	5.9 (1.6–14.6)	5.8 (1.7–13.8)	5.9 (1.8–15.2)	5.9 (1.7–15.5)
Bhutan	85 to 89	5.4 (1.4–13.5)	5.3 (1.4–13.3)	5.2 (1.5–12.5)	5.3 (1.6–13.8)	5.3 (1.5–14.1)

Supplementary Table S10: Prevalence of female SVAC by age and location for 1990, 2000, 2010, 2020, and 2023						
**Location**	**Age Range**	**1990**	**2000**	**2010**	**2020**	**2023**
Bhutan	90 to 94	4.9 (1.2–12.3)	4.8 (1.3–12.0)	4.7 (1.4–11.3)	4.8 (1.4–12.5)	4.8 (1.4–12.8)
Bhutan	95 plus	4.8 (1.2–12.2)	4.8 (1.3–11.9)	4.7 (1.3–11.3)	4.7 (1.4–12.4)	4.8 (1.3–12.7)
Bhutan	Age-standardized	12.1 (4.4–24.8)	12.0 (4.7–23.4)	11.7 (5.5–21.1)	11.5 (6.2–19.6)	11.6 (5.8–20.1)
Bhutan	All age	12.2 (4.4–25.1)	12.0 (4.7–23.6)	11.7 (5.6–21.0)	11.6 (6.2–19.4)	11.7 (5.9–20.2)
India	20 to 24	28.9 (11.2–53.0)	27.9 (13.4–46.3)	26.6 (19.0–35.0)	26.6 (17.4–37.3)	26.9 (15.3–40.9)
India	25 to 29	30.7 (11.9–55.3)	30.4 (14.9–49.5)	30.0 (21.8–38.7)	29.9 (20.0–40.8)	29.9 (17.4–44.5)
India	30 to 34	32.1 (12.8–56.9)	31.4 (15.5–50.6)	30.4 (22.2–39.1)	30.3 (20.3–41.3)	30.5 (17.9–45.1)
India	35 to 39	34.7 (14.2–60.0)	35.4 (18.1–55.3)	36.2 (27.0–45.7)	35.9 (24.7–47.7)	35.5 (21.5–51.0)
India	40 to 44	35.2 (14.5–60.6)	35.6 (18.3–55.5)	36.1 (26.9–45.7)	35.8 (24.6–47.8)	35.6 (21.5–51.1)
India	45 to 49	34.4 (14.1–59.5)	34.6 (17.6–54.5)	35.0 (25.9–44.3)	34.7 (23.8–46.4)	34.5 (20.7–49.8)
India	50 to 54	32.8 (11.9–59.5)	32.7 (12.8–58.0)	32.9 (13.3–57.4)	33.1 (14.0–59.7)	33.1 (14.1–59.2)
India	55 to 59	32.0 (11.5–58.6)	31.9 (12.4–56.9)	32.0 (12.8–56.4)	32.2 (13.5–58.7)	32.2 (13.7–58.2)
India	60 to 64	31.2 (11.0–57.7)	31.1 (11.9–56.0)	31.2 (12.4–55.4)	31.4 (13.1–57.7)	31.4 (13.2–57.3)
India	65 to 69	29.5 (10.3–56.4)	29.4 (11.1–53.9)	29.5 (11.5–53.3)	29.7 (12.1–55.6)	29.7 (12.2–55.2)
India	70 to 74	24.7 (8.1–49.1)	24.5 (8.7–47.3)	24.6 (9.1–46.8)	24.8 (9.6–49.1)	24.8 (9.7–48.6)

Supplementary Table S10: Prevalence of female SVAC by age and location for 1990, 2000, 2010, 2020, and 2023						
**Location**	**Age Range**	**1990**	**2000**	**2010**	**2020**	**2023**
India	75 to 79	22.7 (7.2–46.0)	22.5 (7.8–44.3)	22.6 (8.1–43.8)	22.8 (8.6–46.1)	22.8 (8.7–45.6)
India	80 to 84	16.9 (5.0–36.5)	16.7 (5.5–35.1)	16.8 (5.7–34.6)	16.9 (6.0–36.7)	16.9 (6.1–36.3)
India	85 to 89	15.5 (4.5–34.0)	15.3 (4.9–32.5)	15.3 (5.1–32.1)	15.4 (5.4–34.1)	15.4 (5.5–33.7)
India	90 to 94	14.1 (4.0–31.4)	14.0 (4.4–30.2)	14.0 (4.6–29.7)	14.1 (4.9–31.7)	14.1 (4.9–31.3)
India	95 plus	14.0 (4.0–31.4)	13.8 (4.4–30.0)	13.9 (4.5–29.5)	14.0 (4.8–31.5)	14.0 (4.9–31.1)
India	Age-standardized	31.2 (13.0–53.8)	31.0 (15.0–50.4)	30.9 (19.7–43.7)	30.9 (25.4–36.9)	30.8 (25.0–37.5)
India	All age	31.6 (13.1–55.4)	31.4 (15.2–51.1)	31.1 (20.5–42.7)	31.0 (25.7–36.8)	31.0 (25.1–38.1)
Nepal	20 to 24	14.1 (4.0–31.4)	13.9 (4.4–30.1)	14.0 (4.6–29.6)	14.1 (4.8–31.6)	14.1 (4.9–31.3)
Nepal	25 to 29	14.5 (4.2–32.1)	14.3 (4.5–30.8)	14.4 (4.7–30.4)	14.5 (5.0–32.4)	14.5 (5.1–32.0)
Nepal	30 to 34	15.7 (4.6–34.4)	15.5 (5.0–33.0)	15.6 (5.2–32.5)	15.7 (5.5–34.6)	15.7 (5.6–34.2)
Nepal	35 to 39	15.9 (4.6–34.7)	15.7 (5.1–33.3)	15.8 (5.3–32.9)	16.0 (5.6–35.0)	15.9 (5.7–34.6)
Nepal	40 to 44	16.4 (4.9–35.6)	16.2 (5.3–34.2)	16.3 (5.5–33.7)	16.5 (5.8–35.9)	16.5 (5.9–35.5)
Nepal	45 to 49	16.0 (4.7–34.8)	15.8 (5.1–33.4)	15.8 (5.3–32.9)	16.0 (5.6–35.1)	16.0 (5.7–34.7)
Nepal	50 to 54	15.5 (4.5–34.1)	15.3 (4.9–32.6)	15.4 (5.1–32.1)	15.5 (5.4–34.2)	15.5 (5.5–33.8)
Nepal	55 to 59	14.9 (4.3–33.0)	14.7 (4.7–31.6)	14.8 (4.9–31.1)	14.9 (5.2–33.1)	14.9 (5.2–32.8)

Supplementary Table S10: Prevalence of female SVAC by age and location for 1990, 2000, 2010, 2020, and 2023						
**Location**	**Age Range**	**1990**	**2000**	**2010**	**2020**	**2023**
Nepal	60 to 64	14.4 (4.1–32.1)	14.2 (4.5–30.6)	14.2 (4.7–30.1)	14.4 (5.0–32.2)	14.4 (5.0–31.8)
Nepal	65 to 69	13.4 (3.8–30.2)	13.2 (4.1–28.8)	13.3 (4.3–28.4)	13.4 (4.6–30.3)	13.4 (4.6–30.0)
Nepal	70 to 74	10.7 (3.0–25.0)	10.6 (3.2–23.8)	10.6 (3.4–23.4)	10.7 (3.6–25.1)	10.7 (3.6–24.8)
Nepal	75 to 79	9.7 (2.6–22.8)	9.5 (2.9–21.7)	9.6 (3.0–21.3)	9.7 (3.2–22.9)	9.7 (3.2–22.6)
Nepal	80 to 84	6.9 (1.8–16.6)	6.7 (2.0–15.8)	6.8 (2.0–15.5)	6.8 (2.2–16.8)	6.9 (2.2–16.5)
Nepal	85 to 89	6.2 (1.6–15.5)	6.1 (1.8–14.4)	6.1 (1.8–14.1)	6.2 (1.9–15.3)	6.2 (2.0–15.0)
Nepal	90 to 94	5.6 (1.5–13.8)	5.5 (1.6–13.1)	5.5 (1.6–12.8)	5.6 (1.7–13.9)	5.6 (1.8–13.7)
Nepal	95 plus	5.5 (1.5–13.7)	5.4 (1.6–12.9)	5.5 (1.6–12.7)	5.5 (1.7–13.8)	5.5 (1.8–13.6)
Nepal	Age-standardized	14.6 (4.2–32.5)	14.4 (4.6–30.9)	14.5 (4.8–30.4)	14.6 (5.1–32.5)	14.6 (5.1–32.1)
Nepal	All age	14.9 (4.3–33.1)	14.7 (4.7–31.4)	14.7 (4.9–30.9)	14.8 (5.1–32.8)	14.8 (5.2–32.4)
Pakistan	20 to 24	14.1 (4.0–31.4)	13.9 (4.4–30.1)	14.0 (4.6–29.6)	14.1 (4.8–31.6)	14.1 (4.9–31.3)
Pakistan	25 to 29	14.5 (4.2–32.1)	14.3 (4.5–30.8)	14.4 (4.7–30.4)	14.5 (5.0–32.4)	14.5 (5.1–32.0)
Pakistan	30 to 34	15.7 (4.6–34.4)	15.5 (5.0–33.0)	15.6 (5.2–32.5)	15.7 (5.5–34.6)	15.7 (5.6–34.2)
Pakistan	35 to 39	15.9 (4.6–34.7)	15.7 (5.1–33.3)	15.8 (5.3–32.9)	16.0 (5.6–35.0)	15.9 (5.7–34.6)
Pakistan	40 to 44	16.4 (4.9–35.6)	16.2 (5.3–34.2)	16.3 (5.5–33.7)	16.5 (5.8–35.9)	16.5 (5.9–35.5)

Supplementary Table S10: Prevalence of female SVAC by age and location for 1990, 2000, 2010, 2020, and 2023						
**Location**	**Age Range**	**1990**	**2000**	**2010**	**2020**	**2023**
Pakistan	45 to 49	16.0 (4.7–34.8)	15.8 (5.1–33.4)	15.8 (5.3–32.9)	16.0 (5.6–35.1)	16.0 (5.7–34.7)
Pakistan	50 to 54	15.5 (4.5–34.1)	15.3 (4.9–32.6)	15.4 (5.1–32.1)	15.5 (5.4–34.2)	15.5 (5.5–33.8)
Pakistan	55 to 59	14.9 (4.3–33.0)	14.7 (4.7–31.6)	14.8 (4.9–31.1)	14.9 (5.2–33.1)	14.9 (5.2–32.8)
Pakistan	60 to 64	14.4 (4.1–32.1)	14.2 (4.5–30.6)	14.2 (4.7–30.1)	14.4 (5.0–32.2)	14.4 (5.0–31.8)
Pakistan	65 to 69	13.4 (3.8–30.2)	13.2 (4.1–28.8)	13.3 (4.3–28.4)	13.4 (4.6–30.3)	13.4 (4.6–30.0)
Pakistan	70 to 74	10.7 (3.0–25.0)	10.6 (3.2–23.8)	10.6 (3.4–23.4)	10.7 (3.6–25.1)	10.7 (3.6–24.8)
Pakistan	75 to 79	9.7 (2.6–22.8)	9.5 (2.9–21.7)	9.6 (3.0–21.3)	9.7 (3.2–22.9)	9.7 (3.2–22.6)
Pakistan	80 to 84	6.9 (1.8–16.6)	6.7 (2.0–15.8)	6.8 (2.0–15.5)	6.8 (2.2–16.8)	6.9 (2.2–16.5)
Pakistan	85 to 89	6.2 (1.6–15.5)	6.1 (1.8–14.4)	6.1 (1.8–14.1)	6.2 (1.9–15.3)	6.2 (2.0–15.0)
Pakistan	90 to 94	5.6 (1.5–13.8)	5.5 (1.6–13.1)	5.5 (1.6–12.8)	5.6 (1.7–13.9)	5.6 (1.8–13.7)
Pakistan	95 plus	5.5 (1.5–13.7)	5.4 (1.6–12.9)	5.5 (1.6–12.7)	5.5 (1.7–13.8)	5.5 (1.8–13.6)
Pakistan	Age-standardized	14.6 (4.2–32.5)	14.4 (4.6–30.9)	14.5 (4.8–30.4)	14.6 (5.1–32.5)	14.6 (5.1–32.1)
Pakistan	All age	14.8 (4.3–33.0)	14.6 (4.7–31.4)	14.8 (4.9–31.0)	15.0 (5.2–33.2)	15.0 (5.3–32.8)
Southeast Asia, east Asia, and Oceania	20 to 24	12.8 (9.1–17.8)	15.4 (12.6–18.8)	11.8 (8.7–15.9)	10.1 (6.3–16.3)	10.1 (6.2–16.7)
Southeast Asia, east Asia, and Oceania	25 to 29	12.2 (8.5–17.1)	14.6 (11.3–19.0)	12.5 (9.2–17.0)	11.0 (6.6–17.8)	11.0 (6.4–18.7)

Supplementary Table S10: Prevalence of female SVAC by age and location for 1990, 2000, 2010, 2020, and 2023						
**Location**	**Age Range**	**1990**	**2000**	**2010**	**2020**	**2023**
Southeast Asia, east Asia, and Oceania	30 to 34	11.3 (7.7–16.2)	12.3 (9.4–16.2)	12.7 (9.7–16.9)	13.4 (7.8–21.1)	13.4 (7.4–22.7)
Southeast Asia, east Asia, and Oceania	35 to 39	11.2 (7.5–16.4)	11.8 (8.8–15.8)	12.9 (10.3–16.2)	15.2 (10.1–22.6)	15.9 (9.7–24.5)
Southeast Asia, east Asia, and Oceania	40 to 44	11.8 (7.3–18.0)	11.3 (8.2–15.5)	12.2 (10.2–14.7)	15.0 (12.0–18.6)	15.6 (11.8–20.6)
Southeast Asia, east Asia, and Oceania	45 to 49	11.8 (7.2–18.4)	11.1 (7.9–15.2)	11.5 (9.4–14.0)	11.9 (9.0–16.2)	12.0 (8.6–17.6)
Southeast Asia, east Asia, and Oceania	50 to 54	13.7 (8.1–21.5)	13.1 (9.1–18.4)	11.3 (8.9–14.5)	11.4 (8.6–14.9)	11.5 (8.1–16.1)
Southeast Asia, east Asia, and Oceania	55 to 59	13.9 (7.5–22.9)	13.3 (9.2–18.8)	11.2 (8.9–14.3)	11.0 (8.1–14.7)	11.1 (7.6–16.5)
Southeast Asia, east Asia, and Oceania	60 to 64	13.4 (6.7–23.4)	12.6 (8.6–18.3)	11.3 (8.7–14.7)	11.2 (8.1–15.1)	11.4 (7.5–17.1)
Southeast Asia, east Asia, and Oceania	65 to 69	12.3 (4.9–24.4)	12.0 (6.1–20.8)	11.3 (7.0–17.4)	10.4 (6.8–15.7)	10.2 (6.4–16.0)
Southeast Asia, east Asia, and Oceania	70 to 74	10.5 (3.7–22.1)	10.7 (4.4–20.8)	11.2 (6.1–18.3)	11.7 (5.4–21.3)	11.6 (4.6–22.2)
Southeast Asia, east Asia, and Oceania	75 to 79	9.5 (3.2–21.1)	9.9 (3.7–20.1)	10.5 (5.1–18.7)	11.1 (5.7–19.3)	11.2 (5.4–20.2)
Southeast Asia, east Asia, and Oceania	80 to 84	6.9 (2.2–15.9)	7.5 (2.5–16.5)	8.2 (3.4–16.4)	8.7 (4.4–15.9)	8.7 (4.1–15.7)
Southeast Asia, east Asia, and Oceania	85 to 89	5.7 (1.7–13.3)	5.7 (1.6–13.5)	5.8 (1.7–13.3)	5.9 (1.9–14.7)	5.9 (1.9–14.6)
Southeast Asia, east Asia, and Oceania	90 to 94	5.0 (1.5–11.7)	5.1 (1.4–12.1)	5.2 (1.6–12.1)	5.3 (1.7–13.4)	5.4 (1.7–13.3)
Southeast Asia, east Asia, and Oceania	95 plus	4.4 (1.3–10.6)	4.7 (1.3–11.3)	4.9 (1.5–11.5)	5.3 (1.6–13.2)	5.3 (1.7–13.1)
Southeast Asia, east Asia, and Oceania	Age-standardized	12.0 (7.6–18.4)	12.5 (9.2–17.3)	11.8 (9.4–15.2)	12.0 (9.3–16.3)	12.2 (9.0–17.2)

Supplementary Table S10: Prevalence of female SVAC by age and location for 1990, 2000, 2010, 2020, and 2023						
**Location**	**Age Range**	**1990**	**2000**	**2010**	**2020**	**2023**
Southeast Asia, east Asia, and Oceania	All age	12.1 (8.0–18.1)	12.7 (9.4–17.1)	11.8 (9.5–15.2)	12.0 (9.3–16.0)	12.1 (9.0–16.9)
East Asia	20 to 24	13.8 (10.0–18.6)	18.3 (15.3–21.7)	12.6 (9.0–16.8)	9.9 (4.8–17.4)	9.9 (4.4–18.3)
East Asia	25 to 29	12.9 (9.2–17.6)	16.4 (13.0–20.4)	13.6 (10.3–17.6)	11.2 (5.5–19.4)	11.2 (4.9–21.3)
East Asia	30 to 34	11.5 (8.3–15.6)	12.9 (10.2–16.1)	13.7 (10.6–17.3)	14.3 (7.4–23.7)	14.5 (6.7–26.3)
East Asia	35 to 39	11.2 (8.0–15.4)	12.2 (9.6–15.3)	13.7 (10.9–17.0)	17.4 (10.6–25.2)	18.0 (10.0–28.3)
East Asia	40 to 44	11.9 (7.9–17.4)	11.4 (8.8–14.5)	12.6 (10.3–15.3)	16.8 (12.6–22.9)	17.9 (12.3–26.2)
East Asia	45 to 49	12.1 (7.8–17.7)	11.2 (8.5–14.4)	11.7 (9.6–14.3)	12.3 (8.0–18.5)	12.5 (7.3–21.0)
East Asia	50 to 54	14.6 (9.3–21.8)	13.8 (10.4–17.9)	11.6 (8.7–14.9)	11.5 (8.1–16.4)	11.8 (7.4–18.5)
East Asia	55 to 59	15.0 (8.7–23.8)	14.3 (10.6–18.7)	11.6 (8.7–15.1)	11.1 (7.5–16.7)	11.3 (6.7–18.7)
East Asia	60 to 64	14.4 (7.5–24.5)	13.5 (9.9–17.8)	11.7 (8.4–15.7)	11.6 (7.7–17.6)	11.9 (7.1–20.0)
East Asia	65 to 69	13.1 (5.1–26.5)	12.8 (6.8–21.2)	11.9 (7.6–17.7)	10.6 (7.0–15.4)	10.4 (6.5–16.0)
East Asia	70 to 74	11.2 (3.9–23.7)	11.7 (5.0–21.7)	12.3 (7.0–19.3)	12.7 (6.0–22.4)	12.7 (5.2–23.7)
East Asia	75 to 79	10.2 (3.4–22.3)	10.8 (4.1–22.1)	11.6 (5.9–19.9)	12.3 (6.5–20.5)	12.3 (6.1–21.9)
East Asia	80 to 84	7.5 (2.4–17.3)	8.2 (2.7–17.6)	9.2 (3.9–17.7)	9.7 (5.1–17.1)	9.7 (4.7–17.0)
East Asia	85 to 89	6.1 (1.9–14.5)	6.1 (1.8–14.5)	6.2 (1.9–14.2)	6.2 (2.0–15.5)	6.3 (2.0–15.3)

Supplementary Table S10: Prevalence of female SVAC by age and location for 1990, 2000, 2010, 2020, and 2023						
**Location**	**Age Range**	**1990**	**2000**	**2010**	**2020**	**2023**
East Asia	90 to 94	5.5 (1.7–13.2)	5.6 (1.6–13.2)	5.6 (1.7–13.0)	5.6 (1.8–14.1)	5.7 (1.8–13.9)
East Asia	95 plus	5.5 (1.6–13.1)	5.5 (1.6–13.1)	5.5 (1.6–12.8)	5.6 (1.8–14.0)	5.6 (1.8–13.8)
East Asia	Age-standardized	12.5 (8.7–18.0)	13.5 (10.8–16.9)	12.5 (10.4–14.8)	12.7 (9.9–16.1)	13.0 (9.6–17.4)
East Asia	All age	12.7 (9.0–17.6)	13.6 (10.9–16.8)	12.5 (10.4–14.8)	12.6 (10.1–15.7)	12.9 (9.9–16.6)
China	20 to 24	13.8 (10.0–18.5)	18.5 (15.4–22.1)	12.6 (9.0–16.6)	9.7 (4.7–17.0)	9.7 (4.3–18.3)
China	25 to 29	12.9 (9.3–17.6)	16.5 (13.1–20.5)	13.6 (10.3–17.5)	11.1 (5.5–19.2)	11.0 (4.8–20.8)
China	30 to 34	11.3 (8.2–15.3)	12.8 (10.1–16.0)	13.6 (10.6–17.3)	14.3 (7.5–23.6)	14.5 (6.6–26.1)
China	35 to 39	11.1 (8.0–15.0)	12.1 (9.6–15.0)	13.7 (10.8–17.0)	17.5 (10.7–25.0)	18.1 (10.0–28.4)
China	40 to 44	11.8 (8.0–17.0)	11.2 (8.8–14.2)	12.5 (10.0–15.4)	16.9 (12.1–23.5)	18.0 (11.8–27.0)
China	45 to 49	11.9 (7.9–17.4)	11.0 (8.5–14.1)	11.6 (9.3–14.3)	12.2 (7.6–18.8)	12.4 (6.7–21.5)
China	50 to 54	14.6 (9.3–21.6)	13.8 (10.5–17.8)	11.5 (8.3–15.0)	11.4 (7.7–16.6)	11.7 (7.0–18.8)
China	55 to 59	15.0 (8.7–23.8)	14.3 (10.8–18.5)	11.5 (8.3–15.1)	11.0 (6.9–17.0)	11.2 (6.3–18.9)
China	60 to 64	14.5 (7.6–24.7)	13.5 (10.0–17.7)	11.6 (8.0–15.7)	11.5 (7.2–17.9)	11.8 (6.7–20.4)
China	65 to 69	13.2 (5.1–26.1)	12.8 (6.8–20.9)	11.8 (7.6–17.6)	10.5 (7.0–15.0)	10.3 (6.4–15.9)
China	70 to 74	11.3 (3.9–23.8)	11.7 (5.1–21.6)	12.3 (7.2–19.1)	12.8 (6.1–22.7)	12.7 (5.2–23.9)

Supplementary Table S10: Prevalence of female SVAC by age and location for 1990, 2000, 2010, 2020, and 2023						
**Location**	**Age Range**	**1990**	**2000**	**2010**	**2020**	**2023**
China	75 to 79	10.3 (3.4–22.3)	10.9 (4.1–22.1)	11.7 (6.0–19.9)	12.4 (6.6–20.4)	12.4 (6.1–22.1)
China	80 to 84	7.5 (2.4–17.4)	8.3 (2.7–17.8)	9.3 (4.0–17.8)	9.8 (5.1–16.9)	9.8 (4.8–17.2)
China	85 to 89	6.1 (1.9–14.5)	6.1 (1.8–14.5)	6.2 (1.9–14.2)	6.3 (2.0–15.5)	6.3 (2.0–15.3)
China	90 to 94	5.5 (1.7–13.2)	5.6 (1.6–13.2)	5.6 (1.7–13.0)	5.6 (1.8–14.1)	5.7 (1.8–14.0)
China	95 plus	5.5 (1.7–13.1)	5.5 (1.6–13.1)	5.5 (1.7–12.8)	5.6 (1.8–14.0)	5.6 (1.8–13.8)
China	Age-standardized	12.5 (8.8–17.7)	13.5 (10.9–16.6)	12.4 (10.4–14.7)	12.7 (10.0–15.9)	12.9 (9.7–17.4)
China	All age	12.6 (9.1–17.2)	13.5 (11.0–16.5)	12.4 (10.4–14.6)	12.6 (10.2–15.6)	12.8 (9.9–16.6)
North Korea	20 to 24	13.5 (4.4–29.4)	13.6 (4.3–29.4)	13.6 (4.4–28.9)	13.7 (4.7–30.8)	13.7 (4.8–30.5)
North Korea	25 to 29	13.8 (4.5–29.9)	13.8 (4.3–29.9)	13.8 (4.5–29.3)	13.9 (4.8–31.3)	13.9 (4.9–30.8)
North Korea	30 to 34	14.8 (4.9–31.7)	14.8 (4.7–31.7)	14.8 (4.9–31.2)	14.9 (5.2–33.2)	14.9 (5.3–32.8)
North Korea	35 to 39	14.9 (5.0–31.9)	14.9 (4.7–31.9)	14.9 (4.9–31.3)	15.0 (5.2–33.4)	15.0 (5.3–32.8)
North Korea	40 to 44	15.3 (5.1–32.7)	15.3 (4.9–32.7)	15.4 (5.1–32.1)	15.5 (5.4–34.2)	15.5 (5.5–33.6)
North Korea	45 to 49	15.0 (5.0–32.0)	15.0 (4.8–32.0)	15.0 (5.0–31.5)	15.1 (5.3–33.5)	15.1 (5.3–33.1)
North Korea	50 to 54	14.7 (4.9–31.6)	14.7 (4.7–31.6)	14.7 (4.9–31.0)	14.9 (5.2–33.1)	14.9 (5.2–32.6)
North Korea	55 to 59	14.3 (4.7–30.9)	14.3 (4.5–30.9)	14.4 (4.7–30.4)	14.5 (5.0–32.4)	14.5 (5.1–32.0)

Supplementary Table S10: Prevalence of female SVAC by age and location for 1990, 2000, 2010, 2020, and 2023						
**Location**	**Age Range**	**1990**	**2000**	**2010**	**2020**	**2023**
North Korea	60 to 64	14.0 (4.6–30.2)	14.0 (4.4–30.2)	14.0 (4.6–29.7)	14.1 (4.9–31.7)	14.1 (4.9–31.3)
North Korea	65 to 69	13.1 (4.3–28.6)	13.1 (4.1–28.6)	13.1 (4.3–28.1)	13.3 (4.5–30.1)	13.3 (4.6–29.7)
North Korea	70 to 74	10.6 (3.4–23.7)	10.6 (3.2–23.7)	10.6 (3.3–23.3)	10.7 (3.6–25.1)	10.7 (3.6–24.7)
North Korea	75 to 79	9.6 (3.0–21.7)	9.6 (2.9–21.7)	9.6 (3.0–21.4)	9.7 (3.2–23.0)	9.7 (3.2–22.6)
North Korea	80 to 84	6.8 (2.1–15.9)	6.8 (2.0–15.9)	6.8 (2.1–15.6)	6.9 (2.2–16.9)	6.9 (2.2–16.7)
North Korea	85 to 89	6.1 (1.8–14.4)	6.1 (1.8–14.4)	6.1 (1.8–14.2)	6.2 (2.0–15.4)	6.2 (2.0–15.1)
North Korea	90 to 94	5.5 (1.7–13.1)	5.5 (1.6–13.1)	5.5 (1.7–12.9)	5.6 (1.8–14.0)	5.6 (1.8–13.8)
North Korea	95 plus	5.5 (1.6–13.0)	5.5 (1.6–13.0)	5.5 (1.6–12.8)	5.6 (1.7–13.9)	5.6 (1.8–13.7)
North Korea	Age-standardized	13.9 (4.6–30.0)	13.9 (4.4–30.0)	13.9 (4.6–29.5)	14.0 (4.8–31.4)	14.0 (4.9–31.0)
North Korea	All age	14.0 (4.6–30.2)	14.0 (4.4–30.2)	13.9 (4.6–29.5)	13.9 (4.8–31.2)	13.9 (4.9–30.7)
Taiwan	20 to 24	13.5 (4.4–29.4)	13.6 (4.3–29.4)	13.6 (4.4–28.9)	13.7 (4.7–30.8)	13.7 (4.8–30.5)
Taiwan	25 to 29	13.8 (4.5–29.9)	13.8 (4.3–29.9)	13.8 (4.5–29.3)	13.9 (4.8–31.3)	13.9 (4.9–30.8)
Taiwan	30 to 34	14.8 (4.9–31.7)	14.8 (4.7–31.7)	14.8 (4.9–31.2)	14.9 (5.2–33.2)	14.9 (5.3–32.8)
Taiwan	35 to 39	14.9 (5.0–31.9)	14.9 (4.7–31.9)	14.9 (4.9–31.3)	15.0 (5.2–33.4)	15.0 (5.3–32.8)
Taiwan	40 to 44	15.3 (5.1–32.7)	15.3 (4.9–32.7)	15.4 (5.1–32.1)	15.5 (5.4–34.2)	15.5 (5.5–33.6)

Supplementary Table S10: Prevalence of female SVAC by age and location for 1990, 2000, 2010, 2020, and 2023						
**Location**	**Age Range**	**1990**	**2000**	**2010**	**2020**	**2023**
Taiwan	45 to 49	15.0 (5.0–32.0)	15.0 (4.8–32.0)	15.0 (5.0–31.5)	15.1 (5.3–33.5)	15.1 (5.3–33.1)
Taiwan	50 to 54	14.7 (4.9–31.6)	14.7 (4.7–31.6)	14.7 (4.9–31.0)	14.9 (5.2–33.1)	14.9 (5.2–32.6)
Taiwan	55 to 59	14.3 (4.7–30.9)	14.3 (4.5–30.9)	14.4 (4.7–30.4)	14.5 (5.0–32.4)	14.5 (5.1–32.0)
Taiwan	60 to 64	14.0 (4.6–30.2)	14.0 (4.4–30.2)	14.0 (4.6–29.7)	14.1 (4.9–31.7)	14.1 (4.9–31.3)
Taiwan	65 to 69	13.1 (4.3–28.6)	13.1 (4.1–28.6)	13.1 (4.3–28.1)	13.3 (4.5–30.1)	13.3 (4.6–29.7)
Taiwan	70 to 74	10.6 (3.4–23.7)	10.6 (3.2–23.7)	10.6 (3.3–23.3)	10.7 (3.6–25.1)	10.7 (3.6–24.7)
Taiwan	75 to 79	9.6 (3.0–21.7)	9.6 (2.9–21.7)	9.6 (3.0–21.4)	9.7 (3.2–23.0)	9.7 (3.2–22.6)
Taiwan	80 to 84	6.8 (2.1–15.9)	6.8 (2.0–15.9)	6.8 (2.1–15.6)	6.9 (2.2–16.9)	6.9 (2.2–16.7)
Taiwan	85 to 89	6.1 (1.8–14.4)	6.1 (1.8–14.4)	6.1 (1.8–14.2)	6.2 (2.0–15.4)	6.2 (2.0–15.1)
Taiwan	90 to 94	5.5 (1.7–13.1)	5.5 (1.6–13.1)	5.5 (1.7–12.9)	5.6 (1.8–14.0)	5.6 (1.8–13.8)
Taiwan	95 plus	5.5 (1.6–13.0)	5.5 (1.6–13.0)	5.5 (1.6–12.8)	5.6 (1.7–13.9)	5.6 (1.8–13.7)
Taiwan	Age-standardized	13.9 (4.6–30.0)	13.9 (4.4–30.0)	13.9 (4.6–29.5)	14.0 (4.8–31.4)	14.0 (4.9–31.0)
Taiwan	All age	14.1 (4.7–30.4)	14.0 (4.4–30.3)	13.9 (4.6–29.5)	13.8 (4.7–31.0)	13.7 (4.8–30.3)
Oceania	20 to 24	12.2 (7.9–18.2)	12.2 (8.1–17.6)	11.7 (9.3–14.9)	11.8 (8.9–16.0)	11.9 (9.0–16.4)
Oceania	25 to 29	12.8 (8.1–19.2)	12.9 (8.5–18.8)	12.9 (10.3–16.1)	12.6 (9.4–17.1)	12.7 (9.3–17.5)

Supplementary Table S10: Prevalence of female SVAC by age and location for 1990, 2000, 2010, 2020, and 2023						
**Location**	**Age Range**	**1990**	**2000**	**2010**	**2020**	**2023**
Oceania	30 to 34	13.2 (7.8–20.1)	13.0 (8.4–19.2)	12.9 (9.8–16.8)	13.0 (10.8–16.8)	12.9 (10.5–17.0)
Oceania	35 to 39	14.4 (8.4–22.1)	14.3 (9.5–20.2)	14.3 (11.4–18.0)	14.4 (10.7–19.5)	14.3 (10.3–20.3)
Oceania	40 to 44	14.4 (8.2–22.5)	14.2 (9.2–20.6)	14.0 (11.2–17.6)	14.0 (10.3–19.3)	14.0 (9.9–20.0)
Oceania	45 to 49	14.3 (7.9–22.7)	14.2 (9.2–20.9)	14.0 (11.3–17.4)	14.0 (10.1–19.5)	13.9 (9.7–20.1)
Oceania	50 to 54	15.4 (5.8–31.5)	15.3 (5.8–31.1)	15.0 (6.3–29.3)	15.1 (6.5–30.6)	15.1 (6.6–31.4)
Oceania	55 to 59	14.9 (5.6–30.7)	15.0 (5.8–30.6)	14.7 (6.2–28.7)	14.9 (6.6–29.6)	14.9 (6.7–30.3)
Oceania	60 to 64	14.2 (5.3–29.4)	14.7 (5.6–29.8)	14.4 (6.2–28.1)	14.4 (6.3–29.0)	14.4 (6.4–29.4)
Oceania	65 to 69	13.2 (4.8–27.6)	13.9 (4.9–29.1)	13.9 (4.7–29.1)	13.9 (4.8–30.2)	13.8 (4.9–30.5)
Oceania	70 to 74	10.7 (3.9–22.8)	10.8 (3.6–23.4)	11.3 (3.7–24.3)	11.2 (3.8–25.5)	11.4 (4.0–25.7)
Oceania	75 to 79	9.7 (3.6–20.9)	9.5 (3.2–20.9)	10.2 (3.3–22.1)	10.1 (3.4–23.4)	9.9 (3.3–22.8)
Oceania	80 to 84	7.1 (2.5–15.5)	6.8 (2.2–15.4)	6.9 (2.1–15.8)	7.2 (2.3–17.4)	7.2 (2.4–17.3)
Oceania	85 to 89	6.5 (2.2–14.1)	6.2 (1.9–14.3)	6.0 (1.8–13.9)	6.4 (2.0–15.6)	6.5 (2.1–15.8)
Oceania	90 to 94	5.9 (2.0–13.3)	5.8 (1.8–13.3)	5.4 (1.6–12.7)	5.4 (1.6–13.6)	5.6 (1.8–13.7)
Oceania	95 plus	6.0 (2.0–13.4)	6.0 (1.8–14.1)	5.7 (1.7–13.1)	5.1 (1.5–12.8)	5.2 (1.6–12.9)
Oceania	Age-standardized	13.3 (7.2–22.2)	13.3 (7.7–22.0)	13.2 (9.3–18.9)	13.2 (10.7–18.2)	13.2 (10.6–18.2)

Supplementary Table S10: Prevalence of female SVAC by age and location for 1990, 2000, 2010, 2020, and 2023						
**Location**	**Age Range**	**1990**	**2000**	**2010**	**2020**	**2023**
Oceania	All age	13.4 (7.7–21.5)	13.4 (8.3–20.9)	13.3 (10.1–17.6)	13.3 (11.2–17.1)	13.3 (11.1–17.1)
American Samoa	20 to 24	8.2 (2.6–18.5)	7.9 (2.9–16.6)	7.3 (4.0–12.5)	7.0 (4.7–10.0)	7.2 (4.2–11.6)
American Samoa	25 to 29	8.6 (2.8–19.3)	8.5 (3.2–17.7)	8.1 (4.5–13.7)	7.9 (5.4–11.0)	8.0 (4.8–12.8)
American Samoa	30 to 34	9.1 (2.9–20.3)	8.8 (3.3–18.4)	8.2 (4.6–13.9)	8.0 (5.4–11.1)	8.1 (4.8–13.0)
American Samoa	35 to 39	9.5 (3.1–21.2)	9.7 (3.7–20.1)	9.7 (5.5–16.0)	9.6 (6.7–13.1)	9.6 (5.9–15.1)
American Samoa	40 to 44	9.6 (3.1–21.4)	9.8 (3.7–20.2)	9.7 (5.6–16.1)	9.6 (6.7–13.1)	9.6 (5.9–15.2)
American Samoa	45 to 49	9.2 (3.0–20.6)	9.4 (3.6–19.5)	9.4 (5.4–15.6)	9.3 (6.5–12.7)	9.3 (5.7–14.7)
American Samoa	50 to 54	9.0 (2.9–20.1)	9.4 (3.6–19.5)	9.7 (5.6–16.0)	9.7 (6.8–13.2)	9.7 (5.9–15.1)
American Samoa	55 to 59	8.2 (2.6–18.5)	8.3 (3.1–17.4)	8.2 (4.6–13.7)	8.0 (5.5–11.2)	8.1 (4.8–13.0)
American Samoa	60 to 64	7.7 (2.5–17.5)	7.9 (3.0–16.7)	8.0 (4.5–13.5)	7.9 (5.4–11.0)	7.9 (4.7–12.7)
American Samoa	65 to 69	6.8 (2.1–15.5)	6.9 (2.5–14.6)	6.8 (3.7–11.7)	6.7 (4.4– 9.6)	6.7 (3.9–11.0)
American Samoa	70 to 74	5.0 (1.5–11.7)	4.9 (1.7–10.8)	4.7 (2.3– 8.6)	4.6 (2.7– 7.4)	4.7 (2.5– 8.1)
American Samoa	75 to 79	4.4 (1.3–10.4)	4.5 (1.6– 9.8)	4.5 (2.2– 8.2)	4.5 (2.6– 7.0)	4.5 (2.4– 7.8)
American Samoa	80 to 84	2.6 (0.8– 6.5)	2.5 (0.7– 6.3)	2.4 (0.7– 5.7)	2.4 (0.8– 5.9)	2.4 (0.8– 6.0)
American Samoa	85 to 89	2.3 (0.7– 5.6)	2.2 (0.6– 5.4)	2.1 (0.6– 5.0)	2.1 (0.7– 5.2)	2.1 (0.7– 5.2)

Supplementary Table S10: Prevalence of female SVAC by age and location for 1990, 2000, 2010, 2020, and 2023						
**Location**	**Age Range**	**1990**	**2000**	**2010**	**2020**	**2023**
American Samoa	90 to 94	2.0 (0.6– 4.9)	1.9 (0.5– 4.8)	1.9 (0.6– 4.4)	1.8 (0.6– 4.6)	1.9 (0.6– 4.6)
American Samoa	95 plus	1.9 (0.6– 4.8)	1.9 (0.5– 4.7)	1.8 (0.5– 4.3)	1.8 (0.6– 4.5)	1.8 (0.6– 4.5)
American Samoa	Age-standardized	8.3 (2.7–18.6)	8.3 (3.1–17.3)	8.1 (4.6–13.7)	8.0 (5.5–11.0)	8.0 (4.9–12.8)
American Samoa	All age	8.6 (2.8–19.2)	8.6 (3.3–18.0)	8.4 (4.7–14.1)	8.1 (5.6–11.2)	8.1 (4.9–13.0)
Cook Islands	20 to 24	13.6 (7.8–22.5)	13.3 (7.2–21.8)	12.5 (6.9–20.8)	12.6 (5.4–23.9)	12.7 (5.2–25.4)
Cook Islands	25 to 29	13.8 (6.8–23.6)	13.6 (7.9–21.1)	13.1 (7.6–20.2)	13.1 (6.1–24.2)	13.2 (5.3–25.2)
Cook Islands	30 to 34	14.3 (6.2–26.5)	13.6 (7.8–21.3)	13.0 (8.0–19.5)	13.2 (6.5–22.9)	13.4 (5.7–24.8)
Cook Islands	35 to 39	14.8 (5.7–29.1)	14.5 (7.5–24.1)	14.8 (9.6–21.4)	15.0 (8.0–24.8)	15.0 (7.0–26.0)
Cook Islands	40 to 44	15.0 (5.4–30.3)	14.3 (6.4–25.4)	13.9 (9.0–19.9)	14.7 (7.7–24.2)	14.9 (7.0–25.9)
Cook Islands	45 to 49	14.7 (5.2–30.3)	14.2 (6.0–26.7)	13.7 (8.0–21.4)	14.3 (8.0–22.9)	14.5 (7.6–24.8)
Cook Islands	50 to 54	14.4 (5.0–29.7)	13.8 (5.7–26.6)	13.2 (7.2–21.7)	13.4 (7.5–21.4)	13.6 (7.2–22.7)
Cook Islands	55 to 59	13.4 (4.6–28.3)	12.6 (5.0–24.4)	11.8 (6.2–20.0)	12.4 (6.2–21.4)	12.7 (5.8–22.7)
Cook Islands	60 to 64	13.1 (4.5–28.0)	12.3 (4.8–24.1)	11.6 (5.9–19.8)	12.1 (5.3–22.6)	12.3 (4.8–24.2)
Cook Islands	65 to 69	12.8 (4.2–28.2)	12.8 (4.0–28.0)	12.8 (4.1–27.5)	12.9 (4.4–29.3)	12.9 (4.4–28.9)
Cook Islands	70 to 74	10.3 (3.3–23.2)	10.2 (3.1–23.1)	10.2 (3.2–22.6)	10.3 (3.4–24.2)	10.3 (3.4–23.9)

Supplementary Table S10: Prevalence of female SVAC by age and location for 1990, 2000, 2010, 2020, and 2023						
**Location**	**Age Range**	**1990**	**2000**	**2010**	**2020**	**2023**
Cook Islands	75 to 79	9.2 (2.9–21.1)	9.2 (2.8–21.0)	9.2 (2.9–20.5)	9.3 (3.0–22.0)	9.3 (3.1–21.8)
Cook Islands	80 to 84	6.5 (2.0–15.4)	6.5 (1.9–15.3)	6.5 (2.0–14.9)	6.5 (2.1–16.1)	6.5 (2.1–15.9)
Cook Islands	85 to 89	5.9 (1.8–13.9)	5.8 (1.7–13.8)	5.8 (1.7–13.5)	5.9 (1.8–14.6)	5.9 (1.9–14.3)
Cook Islands	90 to 94	5.3 (1.6–12.6)	5.3 (1.5–12.6)	5.3 (1.6–12.3)	5.3 (1.7–13.3)	5.3 (1.7–13.1)
Cook Islands	95 plus	5.2 (1.6–12.5)	5.2 (1.5–12.5)	5.2 (1.6–12.2)	5.2 (1.6–13.2)	5.3 (1.7–12.9)
Cook Islands	Age-standardized	13.6 (5.8–26.0)	13.1 (6.4–23.0)	12.7 (7.4–20.1)	13.0 (6.8–22.7)	13.1 (6.2–24.1)
Cook Islands	All age	13.8 (6.3–25.6)	13.3 (6.6–23.0)	12.7 (7.4–20.1)	12.7 (6.5–22.4)	12.7 (5.9–23.3)
Fiji	20 to 24	20.4 (14.2–28.0)	21.1 (14.9–28.5)	21.3 (14.9–28.3)	20.8 (10.8–34.8)	20.7 (9.8–37.6)
Fiji	25 to 29	19.9 (13.5–28.4)	21.2 (15.1–28.8)	23.7 (17.4–30.7)	24.5 (13.3–39.6)	24.2 (11.9–42.2)
Fiji	30 to 34	20.2 (12.4–30.5)	20.0 (14.1–27.2)	20.2 (14.7–26.6)	22.0 (13.3–34.7)	22.4 (11.9–38.2)
Fiji	35 to 39	20.3 (10.5–34.1)	20.5 (13.9–28.3)	21.8 (16.0–28.2)	23.0 (15.7–32.1)	23.3 (14.9–34.5)
Fiji	40 to 44	21.1 (9.3–38.0)	20.2 (12.4–29.8)	19.8 (14.4–26.2)	19.7 (12.7–28.5)	19.7 (11.9–30.4)
Fiji	45 to 49	21.2 (8.3–39.8)	20.8 (10.8–33.9)	21.0 (15.2–27.9)	21.9 (14.5–30.6)	22.1 (13.7–33.2)
Fiji	50 to 54	20.8 (7.8–40.6)	20.0 (9.4–34.6)	19.4 (12.9–27.8)	19.7 (12.6–28.3)	19.8 (11.9–30.6)
Fiji	55 to 59	20.4 (7.6–40.1)	19.8 (9.4–34.2)	19.6 (13.2–27.7)	20.5 (13.3–29.2)	20.8 (12.7–31.7)

Supplementary Table S10: Prevalence of female SVAC by age and location for 1990, 2000, 2010, 2020, and 2023						
**Location**	**Age Range**	**1990**	**2000**	**2010**	**2020**	**2023**
Fiji	60 to 64	20.2 (7.6–39.5)	19.7 (9.5–34.3)	19.1 (12.3–27.7)	19.0 (11.4–28.7)	19.0 (10.8–30.8)
Fiji	65 to 69	19.0 (6.6–38.9)	18.9 (6.3–38.8)	19.0 (6.6–38.2)	19.1 (6.9–40.3)	19.1 (7.0–39.9)
Fiji	70 to 74	15.4 (5.2–32.9)	15.4 (4.9–32.8)	15.4 (5.1–32.2)	15.5 (5.4–34.2)	15.5 (5.5–33.7)
Fiji	75 to 79	14.0 (4.6–30.2)	13.9 (4.4–30.2)	14.0 (4.6–29.6)	14.1 (4.8–31.6)	14.1 (4.9–31.2)
Fiji	80 to 84	10.1 (3.2–22.7)	10.0 (3.0–22.7)	10.0 (3.2–22.2)	10.1 (3.3–23.8)	10.1 (3.4–23.5)
Fiji	85 to 89	9.1 (2.8–20.8)	9.1 (2.7–20.7)	9.1 (2.8–20.3)	9.1 (3.0–21.8)	9.1 (3.0–21.5)
Fiji	90 to 94	8.2 (2.6–19.0)	8.2 (2.4–18.9)	8.2 (2.5–18.6)	8.3 (2.7–20.0)	8.3 (2.7–19.7)
Fiji	95 plus	8.2 (2.5–18.9)	8.1 (2.4–18.8)	8.1 (2.5–18.4)	8.2 (2.6–19.8)	8.2 (2.7–19.5)
Fiji	Age-standardized	19.7 (11.2–31.6)	19.7 (13.0–27.9)	20.1 (16.1–24.4)	20.6 (15.7–26.1)	20.7 (14.4–28.4)
Fiji	All age	20.2 (12.3–30.9)	20.2 (14.0–27.6)	20.5 (16.7–24.8)	20.9 (15.8–26.5)	20.9 (14.4–28.8)
Guam	20 to 24	15.4 (5.2–32.7)	15.3 (4.9–32.7)	15.4 (5.1–32.2)	15.4 (5.4–34.1)	15.4 (5.5–33.7)
Guam	25 to 29	15.6 (5.3–33.2)	15.6 (5.0–33.1)	15.7 (5.2–32.7)	15.7 (5.5–34.6)	15.7 (5.6–34.0)
Guam	30 to 34	16.8 (5.7–35.2)	16.7 (5.4–35.1)	16.8 (5.7–34.6)	16.8 (6.0–36.5)	16.8 (6.0–36.0)
Guam	35 to 39	16.8 (5.7–35.3)	16.8 (5.5–35.2)	16.9 (5.7–34.7)	16.9 (6.0–36.6)	16.9 (6.1–36.2)
Guam	40 to 44	17.3 (5.9–36.1)	17.2 (5.6–36.0)	17.3 (5.9–35.4)	17.3 (6.2–37.4)	17.3 (6.3–37.0)

Supplementary Table S10: Prevalence of female SVAC by age and location for 1990, 2000, 2010, 2020, and 2023						
**Location**	**Age Range**	**1990**	**2000**	**2010**	**2020**	**2023**
Guam	45 to 49	16.8 (5.7–35.2)	16.7 (5.4–35.1)	16.8 (5.7–34.5)	16.8 (5.9–36.4)	16.8 (6.0–35.9)
Guam	50 to 54	16.3 (5.5–34.4)	16.3 (5.3–34.3)	16.3 (5.5–33.7)	16.3 (5.7–35.6)	16.3 (5.8–35.2)
Guam	55 to 59	15.7 (5.3–33.3)	15.7 (5.0–33.2)	15.7 (5.2–32.7)	15.7 (5.5–34.5)	15.7 (5.6–34.1)
Guam	60 to 64	15.1 (5.0–32.3)	15.1 (4.8–32.2)	15.1 (5.0–31.6)	15.1 (5.2–33.4)	15.1 (5.3–33.0)
Guam	65 to 69	14.0 (4.6–30.2)	13.9 (4.4–30.1)	13.9 (4.6–29.6)	13.9 (4.8–31.3)	13.9 (4.8–30.9)
Guam	70 to 74	11.1 (3.5–24.8)	11.1 (3.4–24.7)	11.1 (3.5–24.2)	11.0 (3.7–25.7)	11.0 (3.7–25.4)
Guam	75 to 79	9.9 (3.1–22.4)	9.9 (3.0–22.3)	9.9 (3.1–21.9)	9.8 (3.2–23.2)	9.8 (3.3–22.9)
Guam	80 to 84	6.9 (2.1–16.2)	6.9 (2.0–16.2)	6.9 (2.1–15.8)	6.9 (2.2–16.9)	6.9 (2.2–16.6)
Guam	85 to 89	6.2 (1.9–14.6)	6.2 (1.8–14.5)	6.1 (1.8–14.2)	6.1 (1.9–15.1)	6.1 (2.0–14.9)
Guam	90 to 94	5.5 (1.7–13.2)	5.5 (1.6–13.1)	5.5 (1.6–12.8)	5.5 (1.7–13.7)	5.5 (1.7–13.5)
Guam	95 plus	5.5 (1.6–13.0)	5.4 (1.6–13.0)	5.4 (1.6–12.6)	5.4 (1.7–13.5)	5.4 (1.7–13.3)
Guam	Age-standardized	15.5 (5.2–32.8)	15.4 (5.0–32.7)	15.5 (5.2–32.2)	15.5 (5.4–34.0)	15.5 (5.5–33.6)
Guam	All age	15.9 (5.4–33.6)	15.8 (5.1–33.4)	15.7 (5.3–32.6)	15.4 (5.4–33.8)	15.3 (5.4–33.2)
Kiribati	20 to 24	24.5 (10.5–44.8)	24.2 (14.1–36.7)	24.1 (18.3–30.5)	24.1 (12.2–40.6)	24.2 (10.6–43.4)
Kiribati	25 to 29	26.2 (11.5–47.1)	26.8 (15.9–39.8)	27.0 (20.9–33.6)	26.3 (13.6–43.2)	26.1 (11.7–45.8)

Supplementary Table S10: Prevalence of female SVAC by age and location for 1990, 2000, 2010, 2020, and 2023						
**Location**	**Age Range**	**1990**	**2000**	**2010**	**2020**	**2023**
Kiribati	30 to 34	27.6 (12.2–49.0)	27.9 (16.6–41.2)	28.0 (21.7–34.8)	27.5 (14.3–44.8)	27.4 (12.4–47.5)
Kiribati	35 to 39	29.4 (13.2–51.3)	30.9 (18.7–44.9)	31.4 (24.5–38.8)	30.0 (15.8–48.2)	29.5 (13.5–50.4)
Kiribati	40 to 44	29.6 (13.3–51.5)	30.6 (18.4–44.6)	31.0 (24.0–38.3)	29.9 (15.7–48.1)	29.5 (13.5–50.5)
Kiribati	45 to 49	28.9 (12.8–50.6)	29.8 (17.8–43.7)	30.1 (23.2–37.5)	29.1 (15.2–47.2)	28.7 (13.2–49.5)
Kiribati	50 to 54	26.3 (10.0–49.9)	26.4 (9.6–49.9)	26.5 (10.0–49.3)	26.4 (10.4–51.3)	26.4 (10.5–50.9)
Kiribati	55 to 59	25.5 (9.6–48.8)	25.5 (9.2–48.7)	25.7 (9.6–48.2)	25.6 (10.0–50.2)	25.6 (10.1–49.6)
Kiribati	60 to 64	24.8 (9.2–47.7)	24.8 (8.8–47.7)	24.9 (9.2–47.1)	24.8 (9.6–49.1)	24.8 (9.7–48.6)
Kiribati	65 to 69	23.2 (8.5–45.5)	23.2 (8.1–45.4)	23.3 (8.5–44.9)	23.3 (8.8–46.8)	23.2 (9.0–46.4)
Kiribati	70 to 74	19.0 (6.7–39.0)	19.0 (6.4–39.0)	19.1 (6.6–38.4)	19.0 (6.9–40.3)	19.0 (7.0–39.8)
Kiribati	75 to 79	17.3 (5.9–36.1)	17.3 (5.7–36.0)	17.3 (5.9–35.5)	17.3 (6.2–37.3)	17.3 (6.2–36.9)
Kiribati	80 to 84	12.6 (4.1–27.6)	12.6 (3.9–27.6)	12.6 (4.1–27.1)	12.5 (4.2–28.7)	12.5 (4.3–28.3)
Kiribati	85 to 89	11.4 (3.6–25.3)	11.4 (3.5–25.3)	11.4 (3.6–24.8)	11.3 (3.8–26.3)	11.3 (3.8–26.0)
Kiribati	90 to 94	10.3 (3.3–23.2)	10.3 (3.1–23.2)	10.3 (3.3–22.8)	10.3 (3.4–24.2)	10.3 (3.4–23.8)
Kiribati	95 plus	10.2 (3.2–23.0)	10.2 (3.1–23.0)	10.2 (3.2–22.6)	10.2 (3.4–23.9)	10.2 (3.4–23.6)
Kiribati	Age-standardized	25.9 (11.1–46.3)	26.3 (13.7–42.6)	26.4 (20.9–33.5)	25.9 (20.2–33.9)	25.8 (19.2–35.5)

Supplementary Table S10: Prevalence of female SVAC by age and location for 1990, 2000, 2010, 2020, and 2023						
**Location**	**Age Range**	**1990**	**2000**	**2010**	**2020**	**2023**
Kiribati	All age	26.3 (11.3–46.6)	27.0 (14.7–42.3)	27.1 (22.3–32.6)	26.5 (20.1–35.8)	26.3 (19.1–37.6)
Marshall Islands	20 to 24	10.8 (5.0–19.7)	12.0 (6.9–18.8)	13.4 (8.3–19.8)	13.5 (6.5–23.7)	13.4 (5.5–25.6)
Marshall Islands	25 to 29	10.9 (4.4–21.4)	11.4 (6.1–18.7)	12.9 (7.9–19.3)	13.5 (6.5–23.8)	13.5 (5.5–25.7)
Marshall Islands	30 to 34	11.5 (4.2–23.6)	11.2 (5.3–19.7)	12.0 (7.3–18.2)	13.1 (6.7–22.4)	13.3 (6.0–23.9)
Marshall Islands	35 to 39	11.7 (4.1–24.7)	11.3 (4.8–21.8)	11.5 (6.6–18.2)	12.7 (6.9–20.7)	12.9 (6.7–22.0)
Marshall Islands	40 to 44	11.8 (4.0–25.5)	10.9 (4.1–22.3)	10.4 (4.8–18.7)	11.2 (5.8–19.0)	11.5 (5.8–20.0)
Marshall Islands	45 to 49	11.4 (3.8–24.9)	10.5 (3.9–21.7)	9.7 (4.5–17.6)	10.1 (5.1–17.9)	10.5 (5.0–18.8)
Marshall Islands	50 to 54	12.8 (4.5–27.2)	13.1 (5.3–24.6)	12.8 (6.9–20.9)	11.9 (5.9–20.7)	11.7 (5.2–21.3)
Marshall Islands	55 to 59	11.8 (3.9–25.5)	11.6 (4.5–22.7)	11.3 (5.8–19.3)	11.6 (5.1–21.8)	11.7 (4.6–23.0)
Marshall Islands	60 to 64	11.5 (3.8–25.0)	11.4 (4.4–22.4)	11.2 (5.8–19.2)	11.5 (5.0–21.5)	11.5 (4.5–22.7)
Marshall Islands	65 to 69	10.6 (3.4–23.8)	10.6 (3.2–23.7)	10.6 (3.4–23.3)	10.7 (3.5–25.0)	10.7 (3.6–24.7)
Marshall Islands	70 to 74	8.4 (2.6–19.4)	8.4 (2.5–19.3)	8.4 (2.6–19.0)	8.5 (2.7–20.4)	8.5 (2.8–20.1)
Marshall Islands	75 to 79	7.5 (2.3–17.5)	7.5 (2.2–17.5)	7.6 (2.3–17.2)	7.6 (2.4–18.5)	7.6 (2.5–18.3)
Marshall Islands	80 to 84	5.3 (1.6–12.6)	5.3 (1.5–12.6)	5.3 (1.6–12.4)	5.3 (1.7–13.4)	5.3 (1.7–13.1)
Marshall Islands	85 to 89	4.7 (1.4–11.4)	4.7 (1.3–11.4)	4.8 (1.4–11.2)	4.8 (1.5–12.1)	4.8 (1.5–11.9)

Supplementary Table S10: Prevalence of female SVAC by age and location for 1990, 2000, 2010, 2020, and 2023						
**Location**	**Age Range**	**1990**	**2000**	**2010**	**2020**	**2023**
Marshall Islands	90 to 94	4.3 (1.3–10.3)	4.3 (1.2–10.3)	4.3 (1.3–10.1)	4.3 (1.3–10.9)	4.3 (1.4–10.8)
Marshall Islands	95 plus	4.2 (1.3–10.2)	4.2 (1.2–10.2)	4.2 (1.3–10.0)	4.3 (1.3–10.9)	4.3 (1.3–10.7)
Marshall Islands	Age-standardized	11.0 (4.0–22.8)	11.0 (4.8–20.5)	11.3 (6.1–18.6)	11.7 (5.8–20.7)	11.8 (5.3–22.0)
Marshall Islands	All age	11.2 (4.3–22.6)	11.3 (5.3–20.1)	11.9 (6.8–18.8)	12.1 (6.0–20.9)	12.2 (5.5–22.0)
Federated States of Micronesia	20 to 24	12.4 (5.5–22.9)	14.4 (7.3–24.2)	17.6 (10.6–26.4)	18.0 (9.4–30.1)	17.5 (8.1–30.7)
Federated States of Micronesia	25 to 29	12.3 (5.1–23.5)	13.7 (6.7–23.4)	16.9 (9.4–26.5)	18.3 (9.3–31.0)	18.0 (8.2–32.4)
Federated States of Micronesia	30 to 34	12.5 (4.9–25.2)	12.3 (5.5–22.1)	13.5 (6.7–23.5)	15.6 (7.8–27.2)	15.9 (7.4–28.3)
Federated States of Micronesia	35 to 39	12.6 (4.5–26.5)	11.6 (4.8–22.0)	10.7 (5.1–19.1)	10.9 (4.9–20.6)	11.2 (4.4–22.5)
Federated States of Micronesia	40 to 44	13.2 (4.6–28.4)	12.5 (4.7–25.1)	11.8 (5.4–21.6)	12.2 (5.0–23.9)	12.4 (4.8–25.2)
Federated States of Micronesia	45 to 49	13.3 (4.5–28.9)	12.8 (4.6–26.5)	12.2 (5.5–22.5)	12.1 (5.6–23.2)	12.2 (5.0–24.5)
Federated States of Micronesia	50 to 54	13.9 (4.7–29.9)	14.1 (5.3–28.1)	14.2 (6.9–25.0)	14.0 (6.9–24.8)	13.9 (6.3–25.6)
Federated States of Micronesia	55 to 59	14.2 (4.8–30.2)	15.0 (5.8–29.2)	15.8 (7.9–27.2)	15.9 (7.6–28.2)	15.7 (6.7–29.6)
Federated States of Micronesia	60 to 64	13.8 (4.7–29.8)	14.7 (5.6–28.9)	15.6 (7.8–27.0)	15.7 (7.5–27.9)	15.5 (6.6–29.3)
Federated States of Micronesia	65 to 69	11.8 (3.8–26.2)	11.8 (3.6–26.2)	11.9 (3.8–25.9)	12.0 (4.1–27.7)	12.0 (4.1–27.3)
Federated States of Micronesia	70 to 74	9.4 (3.0–21.5)	9.4 (2.8–21.4)	9.5 (3.0–21.2)	9.6 (3.1–22.8)	9.6 (3.2–22.5)

Supplementary Table S10: Prevalence of female SVAC by age and location for 1990, 2000, 2010, 2020, and 2023						
**Location**	**Age Range**	**1990**	**2000**	**2010**	**2020**	**2023**
Federated States of Micronesia	75 to 79	8.5 (2.6–19.5)	8.5 (2.5–19.5)	8.5 (2.6–19.2)	8.6 (2.8–20.7)	8.6 (2.8–20.4)
Federated States of Micronesia	80 to 84	6.0 (1.8–14.1)	6.0 (1.7–14.1)	6.0 (1.8–13.9)	6.1 (1.9–15.1)	6.1 (1.9–14.8)
Federated States of Micronesia	85 to 89	5.4 (1.6–12.8)	5.4 (1.5–12.8)	5.4 (1.6–12.6)	5.5 (1.7–13.6)	5.5 (1.7–13.4)
Federated States of Micronesia	90 to 94	4.8 (1.4–11.6)	4.8 (1.4–11.6)	4.9 (1.4–11.4)	4.9 (1.5–12.4)	4.9 (1.6–12.2)
Federated States of Micronesia	95 plus	4.8 (1.4–11.5)	4.8 (1.4–11.5)	4.8 (1.4–11.3)	4.9 (1.5–12.3)	4.9 (1.5–12.1)
Federated States of Micronesia	Age-standardized	12.4 (4.6–25.8)	12.7 (5.3–24.3)	13.6 (6.9–23.5)	14.1 (6.8–25.9)	14.1 (6.0–27.3)
Federated States of Micronesia	All age	12.5 (4.8–25.5)	13.0 (5.6–24.2)	14.1 (7.3–23.9)	14.6 (7.1–26.1)	14.5 (6.3–27.9)
Nauru	20 to 24	27.2 (10.8–49.9)	27.4 (13.2–45.7)	27.5 (19.1–37.6)	27.6 (16.2–40.6)	27.6 (14.8–44.3)
Nauru	25 to 29	28.8 (11.6–52.0)	29.9 (14.7–49.0)	30.9 (21.9–41.5)	30.7 (18.5–44.5)	30.4 (16.9–47.8)
Nauru	30 to 34	30.2 (12.4–53.8)	30.9 (15.4–50.1)	31.5 (22.4–42.3)	31.4 (18.9–45.3)	31.3 (17.3–48.9)
Nauru	35 to 39	32.3 (13.6–56.0)	34.4 (17.7–54.8)	36.6 (25.3–48.6)	36.1 (21.9–52.3)	35.5 (19.5–54.1)
Nauru	40 to 44	32.8 (13.9–57.0)	34.6 (18.2–54.9)	36.6 (25.5–48.5)	36.1 (22.5–51.6)	35.6 (20.0–54.2)
Nauru	45 to 49	31.9 (13.4–55.9)	33.6 (17.4–53.8)	35.3 (24.7–47.1)	34.9 (21.3–50.0)	34.4 (19.1–53.4)
Nauru	50 to 54	29.2 (11.4–53.7)	29.2 (11.0–53.6)	29.5 (11.5–53.3)	29.7 (12.1–55.5)	29.6 (12.2–55.0)
Nauru	55 to 59	28.3 (11.0–52.5)	28.3 (10.5–52.5)	28.6 (11.0–52.2)	28.8 (11.6–54.4)	28.7 (11.7–53.9)

Supplementary Table S10: Prevalence of female SVAC by age and location for 1990, 2000, 2010, 2020, and 2023						
**Location**	**Age Range**	**1990**	**2000**	**2010**	**2020**	**2023**
Nauru	60 to 64	27.5 (10.6–51.4)	27.5 (10.1–51.4)	27.8 (10.6–51.1)	27.9 (11.2–53.3)	27.9 (11.3–52.8)
Nauru	65 to 69	25.8 (9.7–49.2)	25.8 (9.3–49.2)	26.1 (9.8–48.8)	26.2 (10.3–51.0)	26.2 (10.4–50.5)
Nauru	70 to 74	21.3 (7.6–42.6)	21.3 (7.3–42.6)	21.5 (7.7–42.2)	21.6 (8.1–44.4)	21.6 (8.2–43.9)
Nauru	75 to 79	19.4 (6.8–39.5)	19.4 (6.5–39.5)	19.6 (6.8–39.2)	19.7 (7.2–41.3)	19.7 (7.3–40.9)
Nauru	80 to 84	14.2 (4.7–30.7)	14.2 (4.5–30.7)	14.4 (4.7–30.4)	14.4 (5.0–32.2)	14.4 (5.1–31.8)
Nauru	85 to 89	12.9 (4.2–28.2)	12.9 (4.0–28.2)	13.0 (4.2–27.9)	13.1 (4.4–29.7)	13.1 (4.5–29.3)
Nauru	90 to 94	11.7 (3.8–26.0)	11.7 (3.6–26.0)	11.8 (3.8–25.7)	11.9 (4.0–27.4)	11.9 (4.0–27.0)
Nauru	95 plus	11.6 (3.7–25.7)	11.6 (3.6–25.7)	11.7 (3.7–25.5)	11.8 (3.9–27.1)	11.7 (4.0–26.7)
Nauru	Age-standardized	28.6 (12.0–50.8)	29.4 (14.1–48.3)	30.2 (19.2–43.8)	30.1 (18.3–44.7)	29.9 (17.4–46.0)
Nauru	All age	29.4 (12.3–52.2)	30.4 (14.9–49.7)	31.1 (20.9–43.5)	31.0 (19.1–45.2)	30.7 (17.9–47.3)
Niue	20 to 24	15.4 (5.2–32.7)	15.3 (4.9–32.7)	15.4 (5.1–32.2)	15.4 (5.4–34.1)	15.4 (5.5–33.7)
Niue	25 to 29	15.6 (5.3–33.2)	15.6 (5.0–33.1)	15.7 (5.2–32.7)	15.7 (5.5–34.6)	15.7 (5.6–34.0)
Niue	30 to 34	16.8 (5.7–35.2)	16.7 (5.4–35.1)	16.8 (5.7–34.6)	16.8 (6.0–36.5)	16.8 (6.0–36.0)
Niue	35 to 39	16.8 (5.7–35.3)	16.8 (5.5–35.2)	16.9 (5.7–34.7)	16.9 (6.0–36.6)	16.9 (6.1–36.2)
Niue	40 to 44	17.3 (5.9–36.1)	17.2 (5.6–36.0)	17.3 (5.9–35.4)	17.3 (6.2–37.4)	17.3 (6.3–37.0)

Supplementary Table S10: Prevalence of female SVAC by age and location for 1990, 2000, 2010, 2020, and 2023						
**Location**	**Age Range**	**1990**	**2000**	**2010**	**2020**	**2023**
Niue	45 to 49	16.8 (5.7–35.2)	16.7 (5.4–35.1)	16.8 (5.7–34.5)	16.8 (5.9–36.4)	16.8 (6.0–35.9)
Niue	50 to 54	16.3 (5.5–34.4)	16.3 (5.3–34.3)	16.3 (5.5–33.7)	16.3 (5.7–35.6)	16.3 (5.8–35.2)
Niue	55 to 59	15.7 (5.3–33.3)	15.7 (5.0–33.2)	15.7 (5.2–32.7)	15.7 (5.5–34.5)	15.7 (5.6–34.1)
Niue	60 to 64	15.1 (5.0–32.3)	15.1 (4.8–32.2)	15.1 (5.0–31.6)	15.1 (5.2–33.4)	15.1 (5.3–33.0)
Niue	65 to 69	14.0 (4.6–30.2)	13.9 (4.4–30.1)	13.9 (4.6–29.6)	13.9 (4.8–31.3)	13.9 (4.8–30.9)
Niue	70 to 74	11.1 (3.5–24.8)	11.1 (3.4–24.7)	11.1 (3.5–24.2)	11.0 (3.7–25.7)	11.0 (3.7–25.4)
Niue	75 to 79	9.9 (3.1–22.4)	9.9 (3.0–22.3)	9.9 (3.1–21.9)	9.8 (3.2–23.2)	9.8 (3.3–22.9)
Niue	80 to 84	6.9 (2.1–16.2)	6.9 (2.0–16.2)	6.9 (2.1–15.8)	6.9 (2.2–16.9)	6.9 (2.2–16.6)
Niue	85 to 89	6.2 (1.9–14.6)	6.2 (1.8–14.5)	6.1 (1.8–14.2)	6.1 (1.9–15.1)	6.1 (2.0–14.9)
Niue	90 to 94	5.5 (1.7–13.2)	5.5 (1.6–13.1)	5.5 (1.6–12.8)	5.5 (1.7–13.7)	5.5 (1.7–13.5)
Niue	95 plus	5.5 (1.6–13.0)	5.4 (1.6–13.0)	5.4 (1.6–12.6)	5.4 (1.7–13.5)	5.4 (1.7–13.3)
Niue	Age-standardized	15.5 (5.2–32.8)	15.4 (5.0–32.7)	15.5 (5.2–32.2)	15.5 (5.4–34.0)	15.5 (5.5–33.6)
Niue	All age	15.0 (5.0–31.9)	15.1 (4.9–32.1)	15.0 (5.0–31.3)	15.0 (5.2–33.1)	15.0 (5.3–32.7)
Northern Mariana Islands	20 to 24	15.4 (5.2–32.7)	15.3 (4.9–32.7)	15.4 (5.1–32.2)	15.4 (5.4–34.1)	15.4 (5.5–33.7)
Northern Mariana Islands	25 to 29	15.6 (5.3–33.2)	15.6 (5.0–33.1)	15.7 (5.2–32.7)	15.7 (5.5–34.6)	15.7 (5.6–34.0)

Supplementary Table S10: Prevalence of female SVAC by age and location for 1990, 2000, 2010, 2020, and 2023						
**Location**	**Age Range**	**1990**	**2000**	**2010**	**2020**	**2023**
Northern Mariana Islands	30 to 34	16.8 (5.7–35.2)	16.7 (5.4–35.1)	16.8 (5.7–34.6)	16.8 (6.0–36.5)	16.8 (6.0–36.0)
Northern Mariana Islands	35 to 39	16.8 (5.7–35.3)	16.8 (5.5–35.2)	16.9 (5.7–34.7)	16.9 (6.0–36.6)	16.9 (6.1–36.2)
Northern Mariana Islands	40 to 44	17.3 (5.9–36.1)	17.2 (5.6–36.0)	17.3 (5.9–35.4)	17.3 (6.2–37.4)	17.3 (6.3–37.0)
Northern Mariana Islands	45 to 49	16.8 (5.7–35.2)	16.7 (5.4–35.1)	16.8 (5.7–34.5)	16.8 (5.9–36.4)	16.8 (6.0–35.9)
Northern Mariana Islands	50 to 54	16.3 (5.5–34.4)	16.3 (5.3–34.3)	16.3 (5.5–33.7)	16.3 (5.7–35.6)	16.3 (5.8–35.2)
Northern Mariana Islands	55 to 59	15.7 (5.3–33.3)	15.7 (5.0–33.2)	15.7 (5.2–32.7)	15.7 (5.5–34.5)	15.7 (5.6–34.1)
Northern Mariana Islands	60 to 64	15.1 (5.0–32.3)	15.1 (4.8–32.2)	15.1 (5.0–31.6)	15.1 (5.2–33.4)	15.1 (5.3–33.0)
Northern Mariana Islands	65 to 69	14.0 (4.6–30.2)	13.9 (4.4–30.1)	13.9 (4.6–29.6)	13.9 (4.8–31.3)	13.9 (4.8–30.9)
Northern Mariana Islands	70 to 74	11.1 (3.5–24.8)	11.1 (3.4–24.7)	11.1 (3.5–24.2)	11.0 (3.7–25.7)	11.0 (3.7–25.4)
Northern Mariana Islands	75 to 79	9.9 (3.1–22.4)	9.9 (3.0–22.3)	9.9 (3.1–21.9)	9.8 (3.2–23.2)	9.8 (3.3–22.9)
Northern Mariana Islands	80 to 84	6.9 (2.1–16.2)	6.9 (2.0–16.2)	6.9 (2.1–15.8)	6.9 (2.2–16.9)	6.9 (2.2–16.6)
Northern Mariana Islands	85 to 89	6.2 (1.9–14.6)	6.2 (1.8–14.5)	6.1 (1.8–14.2)	6.1 (1.9–15.1)	6.1 (2.0–14.9)
Northern Mariana Islands	90 to 94	5.5 (1.7–13.2)	5.5 (1.6–13.1)	5.5 (1.6–12.8)	5.5 (1.7–13.7)	5.5 (1.7–13.5)
Northern Mariana Islands	95 plus	5.5 (1.6–13.0)	5.4 (1.6–13.0)	5.4 (1.6–12.6)	5.4 (1.7–13.5)	5.4 (1.7–13.3)
Northern Mariana Islands	Age-standardized	15.5 (5.2–32.8)	15.4 (5.0–32.7)	15.5 (5.2–32.2)	15.5 (5.4–34.0)	15.5 (5.5–33.6)

Supplementary Table S10: Prevalence of female SVAC by age and location for 1990, 2000, 2010, 2020, and 2023						
**Location**	**Age Range**	**1990**	**2000**	**2010**	**2020**	**2023**
Northern Mariana Islands	All age	16.1 (5.4–34.0)	16.0 (5.2–33.9)	16.2 (5.4–33.5)	15.8 (5.5–34.6)	15.6 (5.6–33.9)
Palau	20 to 24	15.9 (8.6–25.7)	17.7 (9.4–28.8)	19.1 (10.5–29.9)	19.0 (9.4–33.8)	18.8 (8.7–34.8)
Palau	25 to 29	15.5 (7.9–26.3)	17.0 (9.4–26.7)	18.7 (10.6–29.1)	19.0 (9.3–33.2)	18.8 (8.4–34.3)
Palau	30 to 34	15.9 (7.2–28.7)	16.9 (9.5–26.6)	19.4 (12.0–28.3)	20.5 (10.3–34.0)	20.4 (9.0–35.6)
Palau	35 to 39	16.1 (6.7–30.7)	16.0 (8.3–26.5)	17.2 (10.2–26.0)	18.4 (9.1–31.3)	18.5 (8.0–32.9)
Palau	40 to 44	16.6 (6.3–32.7)	15.8 (7.3–27.5)	16.0 (9.0–25.0)	17.2 (8.7–28.8)	17.5 (8.1–30.4)
Palau	45 to 49	16.4 (5.8–33.5)	15.6 (6.5–29.1)	15.0 (7.9–24.9)	15.5 (7.5–27.1)	15.8 (7.0–28.9)
Palau	50 to 54	16.3 (5.7–34.1)	15.6 (6.0–30.3)	14.9 (7.3–25.8)	15.1 (7.3–26.9)	15.3 (6.7–28.5)
Palau	55 to 59	17.6 (6.2–35.7)	18.5 (7.7–34.4)	19.0 (10.4–30.5)	18.3 (9.4–30.5)	18.0 (8.2–31.7)
Palau	60 to 64	14.6 (4.9–30.9)	13.8 (4.6–29.5)	13.0 (4.7–26.5)	13.4 (5.0–28.7)	13.6 (5.1–29.1)
Palau	65 to 69	14.6 (4.8–31.3)	14.5 (4.6–31.3)	14.6 (4.8–30.8)	14.7 (5.1–32.7)	14.7 (5.2–32.2)
Palau	70 to 74	11.7 (3.8–26.0)	11.7 (3.6–25.9)	11.7 (3.7–25.5)	11.8 (4.0–27.2)	11.8 (4.0–26.9)
Palau	75 to 79	10.5 (3.4–23.7)	10.5 (3.2–23.7)	10.6 (3.3–23.3)	10.6 (3.5–24.9)	10.6 (3.6–24.6)
Palau	80 to 84	7.5 (2.3–17.4)	7.5 (2.2–17.4)	7.5 (2.3–17.1)	7.6 (2.4–18.4)	7.6 (2.5–18.1)
Palau	85 to 89	6.7 (2.1–15.8)	6.7 (2.0–15.8)	6.7 (2.0–15.5)	6.8 (2.2–16.7)	6.8 (2.2–16.5)

Supplementary Table S10: Prevalence of female SVAC by age and location for 1990, 2000, 2010, 2020, and 2023						
**Location**	**Age Range**	**1990**	**2000**	**2010**	**2020**	**2023**
Palau	90 to 94	6.1 (1.8–14.4)	6.1 (1.8–14.4)	6.1 (1.8–14.1)	6.1 (1.9–15.2)	6.1 (2.0–14.9)
Palau	95 plus	6.0 (1.8–14.3)	6.0 (1.7–14.2)	6.0 (1.8–14.0)	6.1 (1.9–15.1)	6.1 (2.0–14.9)
Palau	Age-standardized	15.4 (6.7–29.4)	15.7 (7.4–27.9)	16.4 (9.1–26.3)	16.9 (8.4–29.9)	16.9 (7.6–31.2)
Palau	All age	15.7 (7.1–29.2)	16.1 (7.8–27.7)	16.7 (9.2–26.6)	16.7 (8.3–29.9)	16.7 (7.4–31.0)
Papua New Guinea	20 to 24	7.9 (4.1–13.4)	7.9 (4.3–12.7)	8.0 (4.8–12.1)	8.3 (4.1–14.0)	8.4 (4.0–14.8)
Papua New Guinea	25 to 29	8.7 (4.7–14.5)	8.7 (5.0–13.7)	8.7 (5.6–12.8)	9.1 (4.7–14.8)	9.2 (4.6–15.8)
Papua New Guinea	30 to 34	9.0 (4.9–15.0)	8.9 (5.2–14.4)	9.0 (5.7–13.4)	9.4 (4.9–15.7)	9.5 (4.7–16.4)
Papua New Guinea	35 to 39	10.3 (5.9–16.7)	10.2 (6.4–15.8)	10.2 (7.0–14.2)	10.5 (6.1–16.6)	10.6 (5.9–17.4)
Papua New Guinea	40 to 44	10.2 (5.9–16.7)	10.2 (6.3–15.9)	10.2 (7.0–14.1)	10.5 (6.0–16.5)	10.6 (5.8–17.2)
Papua New Guinea	45 to 49	9.9 (5.7–16.2)	9.9 (6.1–15.4)	9.8 (6.7–13.8)	10.2 (5.8–16.1)	10.2 (5.6–16.8)
Papua New Guinea	50 to 54	12.1 (3.5–28.9)	12.1 (3.5–27.3)	12.0 (3.8–26.9)	12.3 (3.7–29.7)	12.4 (3.8–29.2)
Papua New Guinea	55 to 59	11.7 (3.4–28.1)	11.7 (3.4–26.5)	11.7 (3.7–26.1)	11.9 (3.6–28.9)	12.0 (3.6–28.4)
Papua New Guinea	60 to 64	11.4 (3.2–27.3)	11.3 (3.2–25.8)	11.3 (3.6–25.4)	11.5 (3.5–28.1)	11.6 (3.5–27.6)
Papua New Guinea	65 to 69	10.5 (3.0–25.6)	10.5 (3.0–24.1)	10.4 (3.3–23.7)	10.7 (3.2–26.3)	10.7 (3.2–25.9)
Papua New Guinea	70 to 74	8.4 (2.3–20.9)	8.3 (2.3–19.6)	8.3 (2.5–19.3)	8.5 (2.5–21.5)	8.5 (2.5–21.2)

Supplementary Table S10: Prevalence of female SVAC by age and location for 1990, 2000, 2010, 2020, and 2023						
**Location**	**Age Range**	**1990**	**2000**	**2010**	**2020**	**2023**
Papua New Guinea	75 to 79	7.5 (2.0–19.0)	7.5 (2.0–17.8)	7.4 (2.2–17.5)	7.6 (2.2–19.5)	7.6 (2.2–19.2)
Papua New Guinea	80 to 84	5.3 (1.4–13.7)	5.2 (1.4–12.8)	5.2 (1.5–12.5)	5.3 (1.5–14.1)	5.3 (1.5–13.8)
Papua New Guinea	85 to 89	4.7 (1.2–12.4)	4.7 (1.2–11.5)	4.6 (1.4–11.3)	4.7 (1.3–12.7)	4.8 (1.3–12.5)
Papua New Guinea	90 to 94	4.3 (1.1–11.2)	4.2 (1.1–10.4)	4.2 (1.2–10.2)	4.3 (1.2–11.6)	4.3 (1.2–11.3)
Papua New Guinea	95 plus	4.2 (1.1–11.1)	4.2 (1.1–10.3)	4.1 (1.2–10.1)	4.2 (1.2–11.4)	4.3 (1.2–11.2)
Papua New Guinea	Age-standardized	9.6 (4.8–17.2)	9.6 (5.1–16.6)	9.6 (6.1–14.9)	9.9 (7.1–15.5)	10.0 (7.1–15.7)
Papua New Guinea	All age	9.5 (5.2–15.9)	9.4 (5.5–14.9)	9.4 (6.6–13.2)	9.9 (7.5–14.0)	10.0 (7.3–14.5)
Samoa	20 to 24	9.5 (4.4–18.1)	9.0 (5.9–12.9)	9.6 (4.5–17.6)	10.3 (3.7–21.8)	10.5 (3.6–22.5)
Samoa	25 to 29	9.8 (4.6–18.5)	9.3 (6.0–13.4)	9.9 (4.6–18.5)	10.5 (3.8–22.7)	10.7 (3.6–24.3)
Samoa	30 to 34	10.5 (5.0–19.7)	9.9 (6.6–14.1)	10.6 (5.1–19.7)	11.4 (4.1–24.2)	11.5 (3.9–25.8)
Samoa	35 to 39	10.5 (4.9–19.6)	9.9 (6.6–14.0)	10.6 (5.0–19.6)	11.4 (4.1–24.2)	11.6 (3.9–25.9)
Samoa	40 to 44	10.4 (4.9–19.5)	9.6 (6.3–13.8)	10.4 (4.9–19.4)	11.5 (4.1–24.4)	11.7 (3.9–26.2)
Samoa	45 to 49	10.7 (5.0–19.8)	10.0 (6.7–14.2)	10.7 (5.1–19.9)	11.5 (4.1–24.4)	11.7 (3.9–26.0)
Samoa	50 to 54	11.8 (3.8–26.2)	11.8 (3.6–26.1)	12.0 (3.8–25.9)	12.1 (4.1–27.8)	12.1 (4.1–27.4)
Samoa	55 to 59	11.5 (3.7–25.5)	11.4 (3.5–25.4)	11.6 (3.7–25.2)	11.7 (3.9–27.0)	11.7 (4.0–26.7)

Supplementary Table S10: Prevalence of female SVAC by age and location for 1990, 2000, 2010, 2020, and 2023						
**Location**	**Age Range**	**1990**	**2000**	**2010**	**2020**	**2023**
Samoa	60 to 64	11.1 (3.5–24.8)	11.0 (3.4–24.7)	11.2 (3.6–24.5)	11.3 (3.8–26.2)	11.3 (3.8–25.9)
Samoa	65 to 69	10.3 (3.3–23.2)	10.2 (3.1–23.1)	10.4 (3.3–22.9)	10.5 (3.5–24.6)	10.5 (3.5–24.3)
Samoa	70 to 74	8.1 (2.5–18.8)	8.1 (2.4–18.7)	8.2 (2.5–18.5)	8.3 (2.7–20.0)	8.3 (2.7–19.7)
Samoa	75 to 79	7.3 (2.2–17.0)	7.3 (2.1–16.9)	7.4 (2.2–16.8)	7.4 (2.4–18.1)	7.4 (2.4–17.9)
Samoa	80 to 84	5.1 (1.5–12.2)	5.1 (1.5–12.1)	5.1 (1.5–12.0)	5.2 (1.6–13.0)	5.2 (1.6–12.8)
Samoa	85 to 89	4.6 (1.4–11.0)	4.6 (1.3–10.9)	4.6 (1.4–10.8)	4.7 (1.4–11.7)	4.7 (1.5–11.6)
Samoa	90 to 94	4.1 (1.2– 9.9)	4.1 (1.2– 9.9)	4.1 (1.2– 9.8)	4.2 (1.3–10.6)	4.2 (1.3–10.5)
Samoa	95 plus	4.1 (1.2– 9.9)	4.1 (1.1– 9.8)	4.1 (1.2– 9.7)	4.1 (1.3–10.5)	4.1 (1.3–10.4)
Samoa	Age-standardized	10.1 (4.4–19.7)	9.7 (6.1–15.0)	10.2 (8.0–13.7)	10.8 (6.9–17.2)	10.9 (6.3–18.8)
Samoa	All age	10.2 (4.6–19.4)	9.7 (6.4–14.4)	10.3 (7.9–14.5)	10.9 (6.8–17.7)	11.0 (6.3–19.2)
Solomon Islands	20 to 24	40.7 (20.7–63.8)	42.0 (27.5–56.6)	42.7 (35.7–49.4)	41.1 (24.0–59.7)	40.7 (21.2–63.0)
Solomon Islands	25 to 29	43.6 (22.8–66.8)	46.5 (31.4–61.3)	47.9 (40.4–55.1)	45.0 (27.1–64.1)	44.0 (23.6–66.2)
Solomon Islands	30 to 34	45.5 (24.2–68.6)	48.2 (33.0–62.9)	49.4 (41.7–56.8)	46.6 (28.5–65.6)	45.8 (25.2–67.8)
Solomon Islands	35 to 39	48.7 (26.6–71.5)	53.3 (37.6–68.1)	55.4 (46.9–63.4)	51.0 (32.1–70.0)	49.5 (27.7–71.3)
Solomon Islands	40 to 44	48.7 (26.6–71.6)	52.7 (37.0–67.6)	54.5 (46.0–62.6)	50.6 (31.7–69.7)	49.3 (27.5–71.2)

Supplementary Table S10: Prevalence of female SVAC by age and location for 1990, 2000, 2010, 2020, and 2023						
**Location**	**Age Range**	**1990**	**2000**	**2010**	**2020**	**2023**
Solomon Islands	45 to 49	47.8 (26.0–70.7)	51.7 (36.1–66.5)	53.4 (45.2–61.2)	49.5 (30.9–68.4)	48.2 (26.9–70.0)
Solomon Islands	50 to 54	41.2 (18.6–67.2)	41.3 (18.0–67.2)	41.6 (18.6–66.8)	41.3 (19.2–68.3)	41.2 (19.4–67.9)
Solomon Islands	55 to 59	40.1 (17.9–66.1)	40.2 (17.2–66.1)	40.5 (17.9–65.7)	40.2 (18.4–67.2)	40.1 (18.6–66.7)
Solomon Islands	60 to 64	39.1 (17.2–65.1)	39.2 (16.6–65.1)	39.4 (17.2–64.6)	39.1 (17.7–66.2)	39.0 (17.9–65.8)
Solomon Islands	65 to 69	37.1 (15.9–63.0)	37.2 (15.4–63.0)	37.4 (16.0–62.5)	37.1 (16.4–64.1)	37.0 (16.6–63.7)
Solomon Islands	70 to 74	31.5 (12.7–56.6)	31.6 (12.2–56.6)	31.8 (12.7–56.1)	31.4 (13.1–57.7)	31.4 (13.2–57.3)
Solomon Islands	75 to 79	29.0 (11.4–53.5)	29.1 (10.9–53.5)	29.3 (11.4–53.0)	28.9 (11.7–54.6)	28.9 (11.8–54.2)
Solomon Islands	80 to 84	22.0 (8.0–43.7)	22.1 (7.7–43.7)	22.2 (8.0–43.2)	21.9 (8.2–44.8)	21.9 (8.3–44.3)
Solomon Islands	85 to 89	20.1 (7.1–40.8)	20.2 (6.8–40.8)	20.3 (7.1–40.3)	20.0 (7.3–41.8)	20.0 (7.4–41.4)
Solomon Islands	90 to 94	18.5 (6.4–38.1)	18.5 (6.2–38.1)	18.6 (6.4–37.6)	18.3 (6.6–39.1)	18.3 (6.7–38.6)
Solomon Islands	95 plus	18.3 (6.3–37.8)	18.4 (6.1–37.8)	18.4 (6.3–37.3)	18.2 (6.5–38.8)	18.1 (6.6–38.3)
Solomon Islands	Age-standardized	42.3 (21.6–65.1)	44.4 (27.1–62.9)	45.4 (38.2–53.5)	43.3 (36.4–50.9)	42.6 (34.4–52.1)
Solomon Islands	All age	43.4 (22.4–65.8)	45.7 (29.3–62.7)	47.2 (41.7–53.1)	44.7 (35.3–56.2)	43.9 (32.6–57.0)
Tokelau	20 to 24	15.4 (5.2–32.7)	15.3 (4.9–32.7)	15.4 (5.1–32.2)	15.4 (5.4–34.1)	15.4 (5.5–33.7)
Tokelau	25 to 29	15.6 (5.3–33.2)	15.6 (5.0–33.1)	15.7 (5.2–32.7)	15.7 (5.5–34.6)	15.7 (5.6–34.0)

Supplementary Table S10: Prevalence of female SVAC by age and location for 1990, 2000, 2010, 2020, and 2023						
**Location**	**Age Range**	**1990**	**2000**	**2010**	**2020**	**2023**
Tokelau	30 to 34	16.8 (5.7–35.2)	16.7 (5.4–35.1)	16.8 (5.7–34.6)	16.8 (6.0–36.5)	16.8 (6.0–36.0)
Tokelau	35 to 39	16.8 (5.7–35.3)	16.8 (5.5–35.2)	16.9 (5.7–34.7)	16.9 (6.0–36.6)	16.9 (6.1–36.2)
Tokelau	40 to 44	17.3 (5.9–36.1)	17.2 (5.6–36.0)	17.3 (5.9–35.4)	17.3 (6.2–37.4)	17.3 (6.3–37.0)
Tokelau	45 to 49	16.8 (5.7–35.2)	16.7 (5.4–35.1)	16.8 (5.7–34.5)	16.8 (5.9–36.4)	16.8 (6.0–35.9)
Tokelau	50 to 54	16.3 (5.5–34.4)	16.3 (5.3–34.3)	16.3 (5.5–33.7)	16.3 (5.7–35.6)	16.3 (5.8–35.2)
Tokelau	55 to 59	15.7 (5.3–33.3)	15.7 (5.0–33.2)	15.7 (5.2–32.7)	15.7 (5.5–34.5)	15.7 (5.6–34.1)
Tokelau	60 to 64	15.1 (5.0–32.3)	15.1 (4.8–32.2)	15.1 (5.0–31.6)	15.1 (5.2–33.4)	15.1 (5.3–33.0)
Tokelau	65 to 69	14.0 (4.6–30.2)	13.9 (4.4–30.1)	13.9 (4.6–29.6)	13.9 (4.8–31.3)	13.9 (4.8–30.9)
Tokelau	70 to 74	11.1 (3.5–24.8)	11.1 (3.4–24.7)	11.1 (3.5–24.2)	11.0 (3.7–25.7)	11.0 (3.7–25.4)
Tokelau	75 to 79	9.9 (3.1–22.4)	9.9 (3.0–22.3)	9.9 (3.1–21.9)	9.8 (3.2–23.2)	9.8 (3.3–22.9)
Tokelau	80 to 84	6.9 (2.1–16.2)	6.9 (2.0–16.2)	6.9 (2.1–15.8)	6.9 (2.2–16.9)	6.9 (2.2–16.6)
Tokelau	85 to 89	6.2 (1.9–14.6)	6.2 (1.8–14.5)	6.1 (1.8–14.2)	6.1 (1.9–15.1)	6.1 (2.0–14.9)
Tokelau	90 to 94	5.5 (1.7–13.2)	5.5 (1.6–13.1)	5.5 (1.6–12.8)	5.5 (1.7–13.7)	5.5 (1.7–13.5)
Tokelau	95 plus	5.5 (1.6–13.0)	5.4 (1.6–13.0)	5.4 (1.6–12.6)	5.4 (1.7–13.5)	5.4 (1.7–13.3)
Tokelau	Age-standardized	15.5 (5.2–32.8)	15.4 (5.0–32.7)	15.5 (5.2–32.2)	15.5 (5.4–34.0)	15.5 (5.5–33.6)

Supplementary Table S10: Prevalence of female SVAC by age and location for 1990, 2000, 2010, 2020, and 2023						
**Location**	**Age Range**	**1990**	**2000**	**2010**	**2020**	**2023**
Tokelau	All age	15.4 (5.2–32.7)	15.3 (4.9–32.6)	15.3 (5.1–31.9)	15.4 (5.4–33.9)	15.4 (5.5–33.4)
Tonga	20 to 24	12.7 (4.6–25.7)	11.8 (5.9–20.5)	11.4 (7.3–16.6)	12.1 (5.5–23.7)	12.3 (4.7–25.6)
Tonga	25 to 29	13.6 (4.9–27.6)	13.0 (6.6–22.3)	12.7 (8.3–18.1)	13.1 (6.0–25.2)	13.3 (5.1–27.4)
Tonga	30 to 34	14.3 (5.2–28.9)	13.4 (6.8–23.0)	13.0 (8.5–18.5)	13.7 (6.2–26.1)	13.9 (5.3–28.5)
Tonga	35 to 39	15.2 (5.6–30.5)	14.8 (7.6–25.1)	14.6 (9.7–20.6)	14.9 (6.9–28.1)	15.0 (5.8–30.3)
Tonga	40 to 44	15.4 (5.7–30.8)	14.7 (7.5–24.9)	14.3 (9.5–20.2)	14.9 (6.9–27.9)	15.0 (5.9–30.4)
Tonga	45 to 49	15.1 (5.6–30.2)	14.5 (7.4–24.5)	14.1 (9.4–19.9)	14.6 (6.7–27.5)	14.7 (5.8–29.8)
Tonga	50 to 54	15.0 (5.0–32.0)	14.9 (4.8–32.0)	15.0 (5.0–31.4)	15.1 (5.2–33.4)	15.1 (5.3–33.0)
Tonga	55 to 59	14.5 (4.8–31.2)	14.4 (4.6–31.1)	14.5 (4.8–30.6)	14.6 (5.0–32.5)	14.6 (5.1–32.1)
Tonga	60 to 64	14.0 (4.6–30.3)	14.0 (4.4–30.2)	14.0 (4.6–29.7)	14.1 (4.8–31.6)	14.1 (4.9–31.1)
Tonga	65 to 69	13.0 (4.3–28.5)	13.0 (4.1–28.4)	13.0 (4.2–27.9)	13.1 (4.5–29.7)	13.1 (4.5–29.4)
Tonga	70 to 74	10.4 (3.3–23.4)	10.4 (3.1–23.3)	10.4 (3.3–22.9)	10.4 (3.5–24.5)	10.4 (3.5–24.2)
Tonga	75 to 79	9.3 (2.9–21.3)	9.3 (2.8–21.2)	9.3 (2.9–20.8)	9.4 (3.1–22.3)	9.4 (3.1–22.0)
Tonga	80 to 84	6.6 (2.0–15.5)	6.6 (1.9–15.4)	6.6 (2.0–15.1)	6.6 (2.1–16.3)	6.6 (2.1–16.0)
Tonga	85 to 89	5.9 (1.8–14.0)	5.9 (1.7–13.9)	5.9 (1.8–13.6)	5.9 (1.9–14.7)	5.9 (1.9–14.4)

Supplementary Table S10: Prevalence of female SVAC by age and location for 1990, 2000, 2010, 2020, and 2023						
**Location**	**Age Range**	**1990**	**2000**	**2010**	**2020**	**2023**
Tonga	90 to 94	5.3 (1.6–12.7)	5.3 (1.5–12.7)	5.3 (1.6–12.4)	5.3 (1.7–13.4)	5.3 (1.7–13.1)
Tonga	95 plus	5.3 (1.6–12.6)	5.3 (1.5–12.5)	5.3 (1.6–12.3)	5.3 (1.7–13.2)	5.3 (1.7–13.0)
Tonga	Age-standardized	13.7 (5.1–27.8)	13.3 (5.9–24.5)	13.1 (8.9–18.3)	13.4 (9.3–19.6)	13.6 (8.7–21.3)
Tonga	All age	13.8 (5.2–27.8)	13.3 (6.0–24.2)	13.0 (9.0–17.9)	13.5 (9.2–20.1)	13.6 (8.7–21.7)
Tuvalu	20 to 24	15.4 (5.2–32.7)	15.3 (4.9–32.7)	15.4 (5.1–32.2)	15.4 (5.4–34.1)	15.4 (5.5–33.7)
Tuvalu	25 to 29	15.6 (5.3–33.2)	15.6 (5.0–33.1)	15.7 (5.2–32.7)	15.7 (5.5–34.6)	15.7 (5.6–34.0)
Tuvalu	30 to 34	16.8 (5.7–35.2)	16.7 (5.4–35.1)	16.8 (5.7–34.6)	16.8 (6.0–36.5)	16.8 (6.0–36.0)
Tuvalu	35 to 39	16.8 (5.7–35.3)	16.8 (5.5–35.2)	16.9 (5.7–34.7)	16.9 (6.0–36.6)	16.9 (6.1–36.2)
Tuvalu	40 to 44	17.3 (5.9–36.1)	17.2 (5.6–36.0)	17.3 (5.9–35.4)	17.3 (6.2–37.4)	17.3 (6.3–37.0)
Tuvalu	45 to 49	16.8 (5.7–35.2)	16.7 (5.4–35.1)	16.8 (5.7–34.5)	16.8 (5.9–36.4)	16.8 (6.0–35.9)
Tuvalu	50 to 54	16.3 (5.5–34.4)	16.3 (5.3–34.3)	16.3 (5.5–33.7)	16.3 (5.7–35.6)	16.3 (5.8–35.2)
Tuvalu	55 to 59	15.7 (5.3–33.3)	15.7 (5.0–33.2)	15.7 (5.2–32.7)	15.7 (5.5–34.5)	15.7 (5.6–34.1)
Tuvalu	60 to 64	15.1 (5.0–32.3)	15.1 (4.8–32.2)	15.1 (5.0–31.6)	15.1 (5.2–33.4)	15.1 (5.3–33.0)
Tuvalu	65 to 69	14.0 (4.6–30.2)	13.9 (4.4–30.1)	13.9 (4.6–29.6)	13.9 (4.8–31.3)	13.9 (4.8–30.9)
Tuvalu	70 to 74	11.1 (3.5–24.8)	11.1 (3.4–24.7)	11.1 (3.5–24.2)	11.0 (3.7–25.7)	11.0 (3.7–25.4)

Supplementary Table S10: Prevalence of female SVAC by age and location for 1990, 2000, 2010, 2020, and 2023						
**Location**	**Age Range**	**1990**	**2000**	**2010**	**2020**	**2023**
Tuvalu	75 to 79	9.9 (3.1–22.4)	9.9 (3.0–22.3)	9.9 (3.1–21.9)	9.8 (3.2–23.2)	9.8 (3.3–22.9)
Tuvalu	80 to 84	6.9 (2.1–16.2)	6.9 (2.0–16.2)	6.9 (2.1–15.8)	6.9 (2.2–16.9)	6.9 (2.2–16.6)
Tuvalu	85 to 89	6.2 (1.9–14.6)	6.2 (1.8–14.5)	6.1 (1.8–14.2)	6.1 (1.9–15.1)	6.1 (2.0–14.9)
Tuvalu	90 to 94	5.5 (1.7–13.2)	5.5 (1.6–13.1)	5.5 (1.6–12.8)	5.5 (1.7–13.7)	5.5 (1.7–13.5)
Tuvalu	95 plus	5.5 (1.6–13.0)	5.4 (1.6–13.0)	5.4 (1.6–12.6)	5.4 (1.7–13.5)	5.4 (1.7–13.3)
Tuvalu	Age-standardized	15.5 (5.2–32.8)	15.4 (5.0–32.7)	15.5 (5.2–32.2)	15.5 (5.4–34.0)	15.5 (5.5–33.6)
Tuvalu	All age	15.8 (5.3–33.4)	15.6 (5.0–33.1)	15.6 (5.2–32.5)	15.5 (5.4–34.0)	15.4 (5.5–33.5)
Vanuatu	20 to 24	15.4 (5.2–32.7)	15.3 (4.9–32.7)	15.4 (5.1–32.2)	15.4 (5.4–34.1)	15.4 (5.5–33.7)
Vanuatu	25 to 29	15.6 (5.3–33.2)	15.6 (5.0–33.1)	15.7 (5.2–32.7)	15.7 (5.5–34.6)	15.7 (5.6–34.0)
Vanuatu	30 to 34	16.8 (5.7–35.2)	16.7 (5.4–35.1)	16.8 (5.7–34.6)	16.8 (6.0–36.5)	16.8 (6.0–36.0)
Vanuatu	35 to 39	16.8 (5.7–35.3)	16.8 (5.5–35.2)	16.9 (5.7–34.7)	16.9 (6.0–36.6)	16.9 (6.1–36.2)
Vanuatu	40 to 44	17.3 (5.9–36.1)	17.2 (5.6–36.0)	17.3 (5.9–35.4)	17.3 (6.2–37.4)	17.3 (6.3–37.0)
Vanuatu	45 to 49	16.8 (5.7–35.2)	16.7 (5.4–35.1)	16.8 (5.7–34.5)	16.8 (5.9–36.4)	16.8 (6.0–35.9)
Vanuatu	50 to 54	16.3 (5.5–34.4)	16.3 (5.3–34.3)	16.3 (5.5–33.7)	16.3 (5.7–35.6)	16.3 (5.8–35.2)
Vanuatu	55 to 59	15.7 (5.3–33.3)	15.7 (5.0–33.2)	15.7 (5.2–32.7)	15.7 (5.5–34.5)	15.7 (5.6–34.1)

Supplementary Table S10: Prevalence of female SVAC by age and location for 1990, 2000, 2010, 2020, and 2023						
**Location**	**Age Range**	**1990**	**2000**	**2010**	**2020**	**2023**
Vanuatu	60 to 64	15.1 (5.0–32.3)	15.1 (4.8–32.2)	15.1 (5.0–31.6)	15.1 (5.2–33.4)	15.1 (5.3–33.0)
Vanuatu	65 to 69	14.0 (4.6–30.2)	13.9 (4.4–30.1)	13.9 (4.6–29.6)	13.9 (4.8–31.3)	13.9 (4.8–30.9)
Vanuatu	70 to 74	11.1 (3.5–24.8)	11.1 (3.4–24.7)	11.1 (3.5–24.2)	11.0 (3.7–25.7)	11.0 (3.7–25.4)
Vanuatu	75 to 79	9.9 (3.1–22.4)	9.9 (3.0–22.3)	9.9 (3.1–21.9)	9.8 (3.2–23.2)	9.8 (3.3–22.9)
Vanuatu	80 to 84	6.9 (2.1–16.2)	6.9 (2.0–16.2)	6.9 (2.1–15.8)	6.9 (2.2–16.9)	6.9 (2.2–16.6)
Vanuatu	85 to 89	6.2 (1.9–14.6)	6.2 (1.8–14.5)	6.1 (1.8–14.2)	6.1 (1.9–15.1)	6.1 (2.0–14.9)
Vanuatu	90 to 94	5.5 (1.7–13.2)	5.5 (1.6–13.1)	5.5 (1.6–12.8)	5.5 (1.7–13.7)	5.5 (1.7–13.5)
Vanuatu	95 plus	5.5 (1.6–13.0)	5.4 (1.6–13.0)	5.4 (1.6–12.6)	5.4 (1.7–13.5)	5.4 (1.7–13.3)
Vanuatu	Age-standardized	15.5 (5.2–32.8)	15.4 (5.0–32.7)	15.5 (5.2–32.2)	15.5 (5.4–34.0)	15.5 (5.5–33.6)
Vanuatu	All age	15.9 (5.4–33.7)	15.9 (5.1–33.6)	15.9 (5.3–33.1)	15.9 (5.6–34.8)	15.9 (5.7–34.4)
Southeast Asia	20 to 24	10.1 (4.7–19.1)	9.9 (5.0–18.2)	9.9 (6.8–16.1)	10.2 (7.3–17.4)	10.3 (6.9–17.5)
Southeast Asia	25 to 29	10.2 (4.7–19.2)	10.1 (5.1–18.4)	10.2 (6.9–16.6)	10.6 (7.4–17.8)	10.7 (7.2–18.1)
Southeast Asia	30 to 34	10.9 (5.0–20.5)	10.7 (5.6–19.2)	10.8 (7.3–17.6)	11.2 (7.7–18.9)	11.3 (7.4–18.6)
Southeast Asia	35 to 39	11.0 (5.0–21.2)	10.8 (5.6–19.4)	10.9 (7.5–17.6)	11.1 (8.2–18.2)	11.1 (8.0–18.3)
Southeast Asia	40 to 44	11.3 (4.9–22.0)	10.9 (5.6–19.8)	11.0 (7.8–17.5)	11.3 (7.8–18.7)	11.3 (7.3–18.3)

Supplementary Table S10: Prevalence of female SVAC by age and location for 1990, 2000, 2010, 2020, and 2023						
**Location**	**Age Range**	**1990**	**2000**	**2010**	**2020**	**2023**
Southeast Asia	45 to 49	11.1 (4.8–21.8)	10.8 (5.4–19.5)	10.7 (7.6–16.9)	11.0 (7.6–18.1)	11.0 (7.1–18.4)
Southeast Asia	50 to 54	10.9 (4.1–22.5)	10.8 (4.1–22.4)	10.5 (4.9–20.2)	10.8 (5.7–21.6)	10.8 (5.7–21.4)
Southeast Asia	55 to 59	10.3 (3.7–21.5)	10.2 (3.8–21.7)	10.1 (4.5–19.6)	10.4 (5.3–21.0)	10.5 (5.2–21.0)
Southeast Asia	60 to 64	10.1 (3.6–21.3)	10.1 (3.6–21.6)	10.1 (4.3–20.3)	10.1 (5.1–20.7)	10.2 (5.0–20.6)
Southeast Asia	65 to 69	9.3 (3.3–20.0)	9.4 (3.1–20.6)	9.4 (3.8–19.5)	9.5 (4.4–20.6)	9.6 (4.5–20.5)
Southeast Asia	70 to 74	7.5 (2.3–17.1)	7.5 (2.2–17.5)	7.6 (2.3–17.2)	7.8 (2.5–18.8)	7.7 (2.5–18.6)
Southeast Asia	75 to 79	6.7 (2.0–15.4)	6.6 (1.9–15.6)	6.7 (2.0–15.4)	6.9 (2.2–16.9)	7.0 (2.2–17.0)
Southeast Asia	80 to 84	4.7 (1.4–11.0)	4.6 (1.3–11.2)	4.7 (1.4–11.0)	4.8 (1.5–12.2)	4.9 (1.5–12.2)
Southeast Asia	85 to 89	4.1 (1.2– 9.8)	4.2 (1.2–10.1)	4.2 (1.2– 9.8)	4.4 (1.3–11.0)	4.4 (1.4–11.1)
Southeast Asia	90 to 94	3.7 (1.1– 8.7)	3.8 (1.1– 9.2)	3.8 (1.1– 9.0)	4.0 (1.2–10.1)	4.0 (1.2–10.2)
Southeast Asia	95 plus	3.7 (1.1– 9.0)	3.8 (1.1– 9.2)	3.9 (1.1– 9.2)	4.1 (1.3–10.3)	4.1 (1.3–10.5)
Southeast Asia	Age-standardized	10.2 (4.3–20.2)	10.0 (4.7–19.3)	10.0 (6.1–17.5)	10.3 (6.7–18.7)	10.4 (6.4–18.6)
Southeast Asia	All age	10.4 (4.5–20.2)	10.2 (4.9–19.2)	10.2 (6.4–17.5)	10.4 (6.8–18.8)	10.4 (6.4–18.7)
Cambodia	20 to 24	7.5 (5.7– 9.8)	8.2 (6.1–10.7)	9.0 (7.6–10.5)	9.5 (6.0–14.2)	9.7 (5.1–16.0)
Cambodia	25 to 29	7.3 (5.6– 9.4)	7.9 (5.9–10.3)	9.2 (7.6–10.9)	9.5 (7.0–12.5)	9.5 (6.2–13.6)

Supplementary Table S10: Prevalence of female SVAC by age and location for 1990, 2000, 2010, 2020, and 2023						
**Location**	**Age Range**	**1990**	**2000**	**2010**	**2020**	**2023**
Cambodia	30 to 34	7.3 (5.6– 9.2)	7.6 (5.8– 9.8)	9.1 (7.5–10.9)	9.3 (6.9–12.4)	9.2 (6.2–13.2)
Cambodia	35 to 39	7.4 (5.4–10.0)	7.6 (5.9– 9.7)	9.9 (8.3–11.6)	9.9 (7.2–13.4)	9.6 (6.2–14.5)
Cambodia	40 to 44	7.6 (4.5–11.9)	7.5 (5.9– 9.5)	9.5 (8.0–11.2)	9.6 (6.8–13.3)	9.4 (6.0–14.5)
Cambodia	45 to 49	7.7 (3.6–14.3)	7.5 (5.3–10.2)	9.2 (7.8–10.8)	8.6 (6.0–11.9)	8.3 (5.2–12.5)
Cambodia	50 to 54	8.6 (3.3–17.4)	7.8 (4.5–12.3)	7.4 (5.8– 9.3)	7.4 (5.1–10.2)	7.5 (4.8–11.1)
Cambodia	55 to 59	8.6 (2.9–18.7)	8.0 (3.7–14.6)	7.3 (5.2– 9.9)	7.4 (5.2–10.2)	7.6 (4.9–11.4)
Cambodia	60 to 64	8.7 (2.9–19.2)	8.4 (3.2–16.8)	7.7 (4.7–12.2)	7.2 (5.3– 9.7)	7.2 (4.8–10.3)
Cambodia	65 to 69	8.2 (2.6–18.7)	8.1 (2.7–17.6)	7.9 (3.6–14.7)	7.6 (5.1–11.1)	7.6 (4.8–11.3)
Cambodia	70 to 74	6.4 (2.0–15.2)	6.4 (1.9–15.1)	6.5 (2.0–14.9)	6.5 (2.1–16.1)	6.6 (2.1–16.5)
Cambodia	75 to 79	5.8 (1.7–13.7)	5.8 (1.7–13.7)	5.8 (1.7–13.5)	5.9 (1.8–14.6)	5.9 (1.8–14.9)
Cambodia	80 to 84	4.0 (1.2– 9.7)	4.0 (1.1– 9.7)	4.0 (1.2– 9.6)	4.1 (1.3–10.4)	4.1 (1.3–10.7)
Cambodia	85 to 89	3.6 (1.1– 8.8)	3.6 (1.0– 8.7)	3.6 (1.1– 8.6)	3.7 (1.1– 9.4)	3.7 (1.1– 9.6)
Cambodia	90 to 94	3.2 (0.9– 7.9)	3.2 (0.9– 7.9)	3.3 (1.0– 7.8)	3.3 (1.0– 8.5)	3.3 (1.0– 8.7)
Cambodia	95 plus	3.2 (0.9– 7.8)	3.2 (0.9– 7.8)	3.2 (0.9– 7.7)	3.3 (1.0– 8.4)	3.3 (1.0– 8.6)
Cambodia	Age-standardized	7.5 (4.7–12.2)	7.6 (5.6–10.4)	8.5 (7.0–10.2)	8.5 (7.2–10.1)	8.5 (6.7–10.7)

Supplementary Table S10: Prevalence of female SVAC by age and location for 1990, 2000, 2010, 2020, and 2023						
**Location**	**Age Range**	**1990**	**2000**	**2010**	**2020**	**2023**
Cambodia	All age	7.5 (5.2–11.2)	7.7 (6.0– 9.9)	8.7 (7.2–10.3)	8.7 (7.3–10.4)	8.6 (6.8–11.1)
Indonesia	20 to 24	10.8 (3.4–24.2)	10.8 (3.3–24.2)	10.8 (3.4–23.7)	10.9 (3.6–25.5)	10.9 (3.7–25.2)
Indonesia	25 to 29	11.0 (3.5–24.6)	11.0 (3.4–24.5)	11.0 (3.5–24.1)	11.1 (3.7–25.9)	11.1 (3.8–25.6)
Indonesia	30 to 34	11.8 (3.8–26.2)	11.8 (3.6–26.2)	11.9 (3.8–25.7)	12.0 (4.0–27.6)	12.0 (4.1–27.3)
Indonesia	35 to 39	11.9 (3.8–26.3)	11.9 (3.7–26.3)	11.9 (3.8–25.8)	12.1 (4.1–27.7)	12.1 (4.1–27.4)
Indonesia	40 to 44	12.2 (4.0–27.0)	12.2 (3.8–26.9)	12.3 (3.9–26.5)	12.4 (4.2–28.4)	12.4 (4.3–28.0)
Indonesia	45 to 49	11.9 (3.8–26.3)	11.8 (3.6–26.2)	11.9 (3.8–25.8)	12.0 (4.0–27.6)	12.0 (4.1–27.2)
Indonesia	50 to 54	11.5 (3.7–25.6)	11.5 (3.5–25.6)	11.5 (3.7–25.1)	11.7 (3.9–27.0)	11.7 (4.0–26.6)
Indonesia	55 to 59	11.1 (3.5–24.8)	11.1 (3.4–24.7)	11.1 (3.5–24.3)	11.2 (3.7–26.1)	11.2 (3.8–25.8)
Indonesia	60 to 64	10.7 (3.4–24.1)	10.7 (3.3–24.0)	10.7 (3.4–23.6)	10.9 (3.6–25.3)	10.9 (3.7–25.0)
Indonesia	65 to 69	9.9 (3.1–22.5)	9.9 (3.0–22.5)	9.9 (3.1–22.0)	10.1 (3.3–23.7)	10.1 (3.4–23.4)
Indonesia	70 to 74	7.9 (2.4–18.3)	7.9 (2.3–18.2)	7.9 (2.4–17.9)	8.0 (2.6–19.3)	8.0 (2.6–19.0)
Indonesia	75 to 79	7.1 (2.2–16.5)	7.1 (2.1–16.5)	7.1 (2.2–16.2)	7.2 (2.3–17.5)	7.2 (2.3–17.3)
Indonesia	80 to 84	5.0 (1.5–11.9)	4.9 (1.4–11.8)	5.0 (1.5–11.6)	5.0 (1.6–12.6)	5.0 (1.6–12.4)
Indonesia	85 to 89	4.5 (1.3–10.7)	4.4 (1.3–10.7)	4.4 (1.3–10.5)	4.5 (1.4–11.4)	4.5 (1.4–11.2)

Supplementary Table S10: Prevalence of female SVAC by age and location for 1990, 2000, 2010, 2020, and 2023						
**Location**	**Age Range**	**1990**	**2000**	**2010**	**2020**	**2023**
Indonesia	90 to 94	4.0 (1.2– 9.7)	4.0 (1.1– 9.7)	4.0 (1.2– 9.5)	4.1 (1.3–10.3)	4.1 (1.3–10.2)
Indonesia	95 plus	4.0 (1.2– 9.6)	4.0 (1.1– 9.6)	4.0 (1.2– 9.4)	4.0 (1.2–10.2)	4.0 (1.3–10.1)
Indonesia	Age-standardized	10.9 (3.5–24.4)	10.9 (3.3–24.4)	10.9 (3.5–23.9)	11.1 (3.7–25.7)	11.1 (3.7–25.4)
Indonesia	All age	11.2 (3.6–24.9)	11.2 (3.4–24.9)	11.2 (3.6–24.5)	11.3 (3.8–26.2)	11.3 (3.8–25.8)
Laos	20 to 24	17.3 (11.7–24.6)	18.0 (12.7–23.9)	15.7 (11.8–20.0)	14.2 (8.8–21.1)	14.1 (7.5–23.6)
Laos	25 to 29	16.5 (10.6–24.0)	17.9 (12.5–24.1)	16.6 (11.8–22.0)	14.5 (10.0–20.0)	14.3 (9.2–20.9)
Laos	30 to 34	15.2 (9.1–23.3)	17.4 (11.8–24.0)	19.0 (14.1–24.6)	17.8 (11.6–26.6)	17.4 (9.9–28.4)
Laos	35 to 39	14.8 (7.8–24.3)	16.1 (10.3–23.1)	18.1 (13.1–23.8)	19.1 (12.5–26.9)	19.3 (11.6–29.4)
Laos	40 to 44	14.9 (6.7–27.2)	15.6 (9.3–23.9)	17.9 (12.7–23.7)	19.2 (12.5–27.1)	19.3 (11.5–29.7)
Laos	45 to 49	15.0 (5.9–29.2)	14.7 (7.8–24.2)	15.9 (10.7–22.1)	17.9 (11.3–25.9)	18.3 (10.7–28.0)
Laos	50 to 54	15.3 (5.6–31.2)	14.7 (6.9–26.1)	14.8 (9.4–21.8)	15.6 (9.4–23.5)	15.9 (8.9–25.4)
Laos	55 to 59	15.3 (5.4–31.4)	14.9 (6.3–28.2)	14.7 (8.6–22.9)	15.2 (8.6–24.1)	15.4 (8.0–26.2)
Laos	60 to 64	14.4 (4.9–30.3)	13.6 (5.2–27.1)	12.8 (6.2–22.3)	13.3 (6.8–22.7)	13.6 (6.4–24.4)
Laos	65 to 69	14.0 (4.7–29.7)	13.7 (4.5–29.4)	13.2 (4.9–27.0)	13.0 (5.3–26.8)	13.1 (5.0–28.2)
Laos	70 to 74	11.4 (3.7–25.5)	11.4 (3.5–25.5)	11.5 (3.7–25.0)	11.6 (3.9–26.9)	11.7 (3.9–27.6)

Supplementary Table S10: Prevalence of female SVAC by age and location for 1990, 2000, 2010, 2020, and 2023						
**Location**	**Age Range**	**1990**	**2000**	**2010**	**2020**	**2023**
Laos	75 to 79	10.3 (3.3–23.3)	10.3 (3.1–23.2)	10.4 (3.3–22.9)	10.5 (3.5–24.6)	10.5 (3.4–25.2)
Laos	80 to 84	7.3 (2.2–17.1)	7.3 (2.1–17.1)	7.4 (2.2–16.8)	7.4 (2.4–18.2)	7.5 (2.4–18.6)
Laos	85 to 89	6.6 (2.0–15.5)	6.6 (1.9–15.5)	6.6 (2.0–15.2)	6.7 (2.1–16.5)	6.8 (2.1–17.0)
Laos	90 to 94	6.0 (1.8–14.1)	6.0 (1.7–14.1)	6.0 (1.8–13.8)	6.1 (1.9–15.0)	6.1 (1.9–15.5)
Laos	95 plus	5.9 (1.8–14.0)	5.9 (1.7–14.0)	5.9 (1.8–13.7)	6.0 (1.9–14.9)	6.1 (1.9–15.3)
Laos	Age-standardized	14.9 (7.6–25.8)	15.5 (9.6–23.4)	15.7 (11.7–20.7)	15.6 (11.6–20.7)	15.7 (10.8–22.0)
Laos	All age	15.4 (8.3–25.9)	16.1 (10.5–23.6)	16.2 (12.5–20.5)	16.0 (12.1–20.7)	16.1 (11.3–22.5)
Malaysia	20 to 24	11.4 (3.7–25.4)	11.4 (3.5–25.4)	11.4 (3.6–25.0)	11.5 (3.9–26.7)	11.5 (3.9–26.4)
Malaysia	25 to 29	11.6 (3.7–25.7)	11.6 (3.6–25.7)	11.6 (3.7–25.3)	11.7 (3.9–27.1)	11.7 (4.0–26.7)
Malaysia	30 to 34	12.4 (4.0–27.4)	12.4 (3.9–27.3)	12.5 (4.0–26.9)	12.6 (4.3–28.7)	12.6 (4.3–28.4)
Malaysia	35 to 39	12.5 (4.1–27.5)	12.5 (3.9–27.4)	12.5 (4.0–27.0)	12.6 (4.3–28.8)	12.6 (4.3–28.5)
Malaysia	40 to 44	12.8 (4.2–28.1)	12.8 (4.0–28.1)	12.8 (4.2–27.6)	13.0 (4.4–29.5)	13.0 (4.5–29.1)
Malaysia	45 to 49	12.4 (4.0–27.4)	12.4 (3.9–27.3)	12.4 (4.0–26.8)	12.6 (4.2–28.7)	12.6 (4.3–28.4)
Malaysia	50 to 54	12.1 (3.9–26.7)	12.1 (3.7–26.7)	12.1 (3.9–26.2)	12.2 (4.1–28.0)	12.2 (4.2–27.7)
Malaysia	55 to 59	11.6 (3.7–25.9)	11.6 (3.6–25.8)	11.7 (3.7–25.4)	11.8 (3.9–27.1)	11.8 (4.0–26.8)

Supplementary Table S10: Prevalence of female SVAC by age and location for 1990, 2000, 2010, 2020, and 2023						
**Location**	**Age Range**	**1990**	**2000**	**2010**	**2020**	**2023**
Malaysia	60 to 64	11.2 (3.6–25.1)	11.2 (3.4–25.0)	11.3 (3.6–24.6)	11.4 (3.8–26.3)	11.4 (3.8–26.0)
Malaysia	65 to 69	10.4 (3.3–23.5)	10.4 (3.2–23.5)	10.4 (3.3–23.0)	10.5 (3.5–24.7)	10.5 (3.5–24.4)
Malaysia	70 to 74	8.3 (2.6–19.1)	8.3 (2.5–19.1)	8.3 (2.6–18.7)	8.4 (2.7–20.2)	8.4 (2.7–19.9)
Malaysia	75 to 79	7.4 (2.3–17.3)	7.4 (2.2–17.3)	7.4 (2.3–17.0)	7.5 (2.4–18.3)	7.5 (2.4–18.0)
Malaysia	80 to 84	5.2 (1.6–12.5)	5.2 (1.5–12.4)	5.2 (1.6–12.2)	5.3 (1.6–13.2)	5.3 (1.7–13.0)
Malaysia	85 to 89	4.7 (1.4–11.3)	4.7 (1.3–11.2)	4.7 (1.4–11.0)	4.7 (1.5–11.9)	4.7 (1.5–11.8)
Malaysia	90 to 94	4.2 (1.3–10.2)	4.2 (1.2–10.2)	4.2 (1.2–10.0)	4.3 (1.3–10.8)	4.3 (1.3–10.7)
Malaysia	95 plus	4.2 (1.2–10.1)	4.2 (1.2–10.1)	4.2 (1.2– 9.9)	4.2 (1.3–10.7)	4.2 (1.3–10.6)
Malaysia	Age-standardized	11.5 (3.7–25.5)	11.5 (3.5–25.5)	11.5 (3.7–25.0)	11.6 (3.9–26.8)	11.6 (4.0–26.4)
Malaysia	All age	11.7 (3.8–26.0)	11.8 (3.6–26.1)	11.7 (3.8–25.5)	11.7 (3.9–27.1)	11.7 (4.0–26.7)
Maldives	20 to 24	16.9 (7.0–31.9)	16.6 (9.9–25.1)	16.5 (11.1–22.8)	16.6 (7.2–31.2)	16.7 (6.5–33.6)
Maldives	25 to 29	18.2 (7.6–33.7)	18.4 (11.2–27.3)	18.3 (12.6–25.0)	18.0 (8.0–33.0)	17.9 (7.2–35.4)
Maldives	30 to 34	19.2 (8.1–35.2)	19.2 (11.8–28.4)	19.1 (13.1–25.9)	18.9 (8.5–34.5)	18.9 (7.6–37.1)
Maldives	35 to 39	20.1 (8.5–36.6)	20.6 (12.7–30.2)	20.5 (14.3–27.7)	19.9 (9.0–36.0)	19.7 (8.1–38.4)
Maldives	40 to 44	20.1 (8.6–36.8)	20.2 (12.4–30.0)	20.2 (13.8–27.4)	19.9 (8.9–36.2)	19.8 (8.0–38.6)

Supplementary Table S10: Prevalence of female SVAC by age and location for 1990, 2000, 2010, 2020, and 2023						
**Location**	**Age Range**	**1990**	**2000**	**2010**	**2020**	**2023**
Maldives	45 to 49	19.9 (8.4–36.4)	20.1 (12.3–29.8)	20.1 (13.8–27.3)	19.7 (8.8–35.8)	19.5 (7.9–38.2)
Maldives	50 to 54	18.2 (6.3–37.6)	18.2 (6.0–37.6)	18.2 (6.3–37.0)	18.3 (6.6–39.0)	18.3 (6.7–38.5)
Maldives	55 to 59	17.6 (6.0–36.6)	17.6 (5.8–36.6)	17.6 (6.0–36.0)	17.7 (6.3–38.0)	17.7 (6.4–37.5)
Maldives	60 to 64	17.0 (5.8–35.7)	17.1 (5.6–35.6)	17.1 (5.8–35.0)	17.1 (6.1–37.0)	17.1 (6.2–36.6)
Maldives	65 to 69	15.9 (5.4–33.7)	15.9 (5.1–33.7)	15.9 (5.3–33.1)	16.0 (5.6–35.0)	16.0 (5.7–34.4)
Maldives	70 to 74	12.8 (4.2–28.1)	12.8 (4.0–28.1)	12.8 (4.2–27.6)	12.9 (4.4–29.3)	12.9 (4.4–28.9)
Maldives	75 to 79	11.6 (3.7–25.8)	11.6 (3.6–25.7)	11.6 (3.7–25.2)	11.6 (3.9–26.9)	11.6 (4.0–26.5)
Maldives	80 to 84	8.3 (2.6–19.1)	8.3 (2.4–19.1)	8.3 (2.5–18.7)	8.3 (2.7–20.0)	8.3 (2.7–19.7)
Maldives	85 to 89	7.5 (2.3–17.4)	7.5 (2.2–17.3)	7.5 (2.3–17.0)	7.5 (2.4–18.2)	7.5 (2.4–18.0)
Maldives	90 to 94	6.7 (2.1–15.8)	6.7 (2.0–15.8)	6.7 (2.0–15.5)	6.8 (2.1–16.6)	6.8 (2.2–16.4)
Maldives	95 plus	6.7 (2.0–15.7)	6.7 (1.9–15.7)	6.7 (2.0–15.4)	6.7 (2.1–16.5)	6.7 (2.2–16.3)
Maldives	Age-standardized	17.8 (7.3–33.8)	17.8 (9.5–29.6)	17.8 (14.1–22.3)	17.7 (13.1–24.9)	17.6 (12.5–26.4)
Maldives	All age	18.2 (7.4–34.5)	18.3 (10.3–28.9)	18.2 (14.5–22.4)	18.1 (12.9–27.3)	18.0 (12.2–28.8)
Mauritius	20 to 24	10.8 (3.4–24.2)	10.8 (3.3–24.2)	10.8 (3.4–23.7)	10.9 (3.6–25.5)	10.9 (3.7–25.2)
Mauritius	25 to 29	11.0 (3.5–24.6)	11.0 (3.4–24.5)	11.0 (3.5–24.1)	11.1 (3.7–25.9)	11.1 (3.8–25.6)

Supplementary Table S10: Prevalence of female SVAC by age and location for 1990, 2000, 2010, 2020, and 2023						
**Location**	**Age Range**	**1990**	**2000**	**2010**	**2020**	**2023**
Mauritius	30 to 34	11.8 (3.8–26.2)	11.8 (3.6–26.2)	11.9 (3.8–25.7)	12.0 (4.0–27.6)	12.0 (4.1–27.3)
Mauritius	35 to 39	11.9 (3.8–26.3)	11.9 (3.7–26.3)	11.9 (3.8–25.8)	12.1 (4.1–27.7)	12.1 (4.1–27.4)
Mauritius	40 to 44	12.2 (4.0–27.0)	12.2 (3.8–26.9)	12.3 (3.9–26.5)	12.4 (4.2–28.4)	12.4 (4.3–28.0)
Mauritius	45 to 49	11.9 (3.8–26.3)	11.8 (3.6–26.2)	11.9 (3.8–25.8)	12.0 (4.0–27.6)	12.0 (4.1–27.2)
Mauritius	50 to 54	11.5 (3.7–25.6)	11.5 (3.5–25.6)	11.5 (3.7–25.1)	11.7 (3.9–27.0)	11.7 (4.0–26.6)
Mauritius	55 to 59	11.1 (3.5–24.8)	11.1 (3.4–24.7)	11.1 (3.5–24.3)	11.2 (3.7–26.1)	11.2 (3.8–25.8)
Mauritius	60 to 64	10.7 (3.4–24.1)	10.7 (3.3–24.0)	10.7 (3.4–23.6)	10.9 (3.6–25.3)	10.9 (3.7–25.0)
Mauritius	65 to 69	9.9 (3.1–22.5)	9.9 (3.0–22.5)	9.9 (3.1–22.0)	10.1 (3.3–23.7)	10.1 (3.4–23.4)
Mauritius	70 to 74	7.9 (2.4–18.3)	7.9 (2.3–18.2)	7.9 (2.4–17.9)	8.0 (2.6–19.3)	8.0 (2.6–19.0)
Mauritius	75 to 79	7.1 (2.2–16.5)	7.1 (2.1–16.5)	7.1 (2.2–16.2)	7.2 (2.3–17.5)	7.2 (2.3–17.3)
Mauritius	80 to 84	5.0 (1.5–11.9)	4.9 (1.4–11.8)	5.0 (1.5–11.6)	5.0 (1.6–12.6)	5.0 (1.6–12.4)
Mauritius	85 to 89	4.5 (1.3–10.7)	4.4 (1.3–10.7)	4.4 (1.3–10.5)	4.5 (1.4–11.4)	4.5 (1.4–11.2)
Mauritius	90 to 94	4.0 (1.2– 9.7)	4.0 (1.1– 9.7)	4.0 (1.2– 9.5)	4.1 (1.3–10.3)	4.1 (1.3–10.2)
Mauritius	95 plus	4.0 (1.2– 9.6)	4.0 (1.1– 9.6)	4.0 (1.2– 9.4)	4.0 (1.2–10.2)	4.0 (1.3–10.1)
Mauritius	Age-standardized	10.9 (3.5–24.4)	10.9 (3.3–24.4)	10.9 (3.5–23.9)	11.1 (3.7–25.7)	11.1 (3.7–25.4)

Supplementary Table S10: Prevalence of female SVAC by age and location for 1990, 2000, 2010, 2020, and 2023						
**Location**	**Age Range**	**1990**	**2000**	**2010**	**2020**	**2023**
Mauritius	All age	11.1 (3.6–24.8)	11.1 (3.4–24.7)	11.0 (3.5–24.1)	10.9 (3.6–25.4)	10.9 (3.7–24.9)
Myanmar	20 to 24	10.8 (3.4–24.2)	10.8 (3.3–24.2)	10.8 (3.4–23.7)	10.9 (3.6–25.5)	10.9 (3.7–25.2)
Myanmar	25 to 29	11.0 (3.5–24.6)	11.0 (3.4–24.5)	11.0 (3.5–24.1)	11.1 (3.7–25.9)	11.1 (3.8–25.6)
Myanmar	30 to 34	11.8 (3.8–26.2)	11.8 (3.6–26.2)	11.9 (3.8–25.7)	12.0 (4.0–27.6)	12.0 (4.1–27.3)
Myanmar	35 to 39	11.9 (3.8–26.3)	11.9 (3.7–26.3)	11.9 (3.8–25.8)	12.1 (4.1–27.7)	12.1 (4.1–27.4)
Myanmar	40 to 44	12.2 (4.0–27.0)	12.2 (3.8–26.9)	12.3 (3.9–26.5)	12.4 (4.2–28.4)	12.4 (4.3–28.0)
Myanmar	45 to 49	11.9 (3.8–26.3)	11.8 (3.6–26.2)	11.9 (3.8–25.8)	12.0 (4.0–27.6)	12.0 (4.1–27.2)
Myanmar	50 to 54	11.5 (3.7–25.6)	11.5 (3.5–25.6)	11.5 (3.7–25.1)	11.7 (3.9–27.0)	11.7 (4.0–26.6)
Myanmar	55 to 59	11.1 (3.5–24.8)	11.1 (3.4–24.7)	11.1 (3.5–24.3)	11.2 (3.7–26.1)	11.2 (3.8–25.8)
Myanmar	60 to 64	10.7 (3.4–24.1)	10.7 (3.3–24.0)	10.7 (3.4–23.6)	10.9 (3.6–25.3)	10.9 (3.7–25.0)
Myanmar	65 to 69	9.9 (3.1–22.5)	9.9 (3.0–22.5)	9.9 (3.1–22.0)	10.1 (3.3–23.7)	10.1 (3.4–23.4)
Myanmar	70 to 74	7.9 (2.4–18.3)	7.9 (2.3–18.2)	7.9 (2.4–17.9)	8.0 (2.6–19.3)	8.0 (2.6–19.0)
Myanmar	75 to 79	7.1 (2.2–16.5)	7.1 (2.1–16.5)	7.1 (2.2–16.2)	7.2 (2.3–17.5)	7.2 (2.3–17.3)
Myanmar	80 to 84	5.0 (1.5–11.9)	4.9 (1.4–11.8)	5.0 (1.5–11.6)	5.0 (1.6–12.6)	5.0 (1.6–12.4)
Myanmar	85 to 89	4.5 (1.3–10.7)	4.4 (1.3–10.7)	4.4 (1.3–10.5)	4.5 (1.4–11.4)	4.5 (1.4–11.2)

Supplementary Table S10: Prevalence of female SVAC by age and location for 1990, 2000, 2010, 2020, and 2023						
**Location**	**Age Range**	**1990**	**2000**	**2010**	**2020**	**2023**
Myanmar	90 to 94	4.0 (1.2– 9.7)	4.0 (1.1– 9.7)	4.0 (1.2– 9.5)	4.1 (1.3–10.3)	4.1 (1.3–10.2)
Myanmar	95 plus	4.0 (1.2– 9.6)	4.0 (1.1– 9.6)	4.0 (1.2– 9.4)	4.0 (1.2–10.2)	4.0 (1.3–10.1)
Myanmar	Age-standardized	10.9 (3.5–24.4)	10.9 (3.3–24.4)	10.9 (3.5–23.9)	11.1 (3.7–25.7)	11.1 (3.7–25.4)
Myanmar	All age	11.1 (3.6–24.8)	11.1 (3.4–24.8)	11.2 (3.6–24.4)	11.2 (3.7–26.0)	11.2 (3.8–25.6)
Philippines	20 to 24	7.5 (4.0–12.2)	7.3 (5.2– 9.7)	7.3 (4.8–10.5)	7.8 (3.2–15.6)	7.9 (2.9–16.6)
Philippines	25 to 29	7.0 (4.1–10.8)	7.1 (5.4– 9.2)	7.8 (5.7–10.3)	8.5 (4.0–15.4)	8.7 (3.6–17.1)
Philippines	30 to 34	7.5 (4.5–11.1)	7.5 (5.7– 9.5)	7.9 (6.1–10.0)	8.6 (5.0–13.8)	8.8 (4.5–15.1)
Philippines	35 to 39	8.0 (4.7–11.8)	7.9 (6.0–10.0)	8.1 (6.0–10.6)	8.4 (5.6–11.9)	8.5 (5.3–12.6)
Philippines	40 to 44	8.0 (4.3–12.9)	7.8 (5.6–10.3)	7.9 (5.2–11.3)	8.5 (3.5–16.9)	8.7 (3.2–18.3)
Philippines	45 to 49	8.1 (4.3–12.9)	7.9 (5.6–10.4)	7.9 (5.2–11.3)	8.5 (3.5–16.8)	8.6 (3.2–18.1)
Philippines	50 to 54	8.1 (4.4–13.1)	7.9 (5.7–10.5)	8.0 (5.2–11.3)	8.4 (3.4–16.7)	8.5 (3.2–17.9)
Philippines	55 to 59	6.7 (3.6–11.1)	6.6 (4.7– 8.8)	6.7 (4.3– 9.6)	7.4 (3.0–14.9)	7.6 (2.8–16.3)
Philippines	60 to 64	6.6 (3.6–11.0)	6.5 (4.6– 8.7)	6.6 (4.3– 9.5)	7.2 (2.9–14.6)	7.4 (2.8–15.9)
Philippines	65 to 69	6.4 (3.3–10.8)	6.3 (4.2– 8.8)	6.3 (4.0– 9.3)	6.9 (2.7–14.0)	7.0 (2.4–15.8)
Philippines	70 to 74	6.2 (1.4–16.5)	6.1 (1.6–14.6)	6.0 (1.8–14.0)	6.1 (1.9–15.1)	6.2 (1.9–15.5)

Supplementary Table S10: Prevalence of female SVAC by age and location for 1990, 2000, 2010, 2020, and 2023						
**Location**	**Age Range**	**1990**	**2000**	**2010**	**2020**	**2023**
Philippines	75 to 79	5.6 (1.3–15.2)	5.4 (1.5–13.3)	5.4 (1.6–12.6)	5.5 (1.7–13.7)	5.5 (1.7–14.0)
Philippines	80 to 84	3.9 (0.9–10.8)	3.8 (1.0– 9.5)	3.8 (1.1– 8.9)	3.8 (1.2– 9.7)	3.8 (1.2–10.0)
Philippines	85 to 89	3.5 (0.8– 9.5)	3.4 (0.9– 8.5)	3.4 (1.0– 8.0)	3.4 (1.0– 8.7)	3.4 (1.0– 9.0)
Philippines	90 to 94	3.2 (0.7– 8.6)	3.1 (0.8– 7.7)	3.0 (0.9– 7.2)	3.1 (0.9– 7.9)	3.1 (0.9– 8.1)
Philippines	95 plus	3.1 (0.7– 8.6)	3.0 (0.8– 7.6)	3.0 (0.9– 7.2)	3.0 (0.9– 7.8)	3.1 (0.9– 8.0)
Philippines	Age-standardized	7.3 (4.0–11.5)	7.2 (5.1– 9.7)	7.3 (5.6– 9.6)	7.9 (4.6–13.5)	8.0 (4.3–14.6)
Philippines	All age	7.4 (4.2–11.7)	7.3 (5.3– 9.7)	7.5 (5.6– 9.9)	8.0 (4.4–13.9)	8.1 (4.1–15.0)
Seychelles	20 to 24	10.8 (3.4–24.2)	10.8 (3.3–24.2)	10.8 (3.4–23.7)	10.9 (3.6–25.5)	10.9 (3.7–25.2)
Seychelles	25 to 29	11.0 (3.5–24.6)	11.0 (3.4–24.5)	11.0 (3.5–24.1)	11.1 (3.7–25.9)	11.1 (3.8–25.6)
Seychelles	30 to 34	11.8 (3.8–26.2)	11.8 (3.6–26.2)	11.9 (3.8–25.7)	12.0 (4.0–27.6)	12.0 (4.1–27.3)
Seychelles	35 to 39	11.9 (3.8–26.3)	11.9 (3.7–26.3)	11.9 (3.8–25.8)	12.1 (4.1–27.7)	12.1 (4.1–27.4)
Seychelles	40 to 44	12.2 (4.0–27.0)	12.2 (3.8–26.9)	12.3 (3.9–26.5)	12.4 (4.2–28.4)	12.4 (4.3–28.0)
Seychelles	45 to 49	11.9 (3.8–26.3)	11.8 (3.6–26.2)	11.9 (3.8–25.8)	12.0 (4.0–27.6)	12.0 (4.1–27.2)
Seychelles	50 to 54	11.5 (3.7–25.6)	11.5 (3.5–25.6)	11.5 (3.7–25.1)	11.7 (3.9–27.0)	11.7 (4.0–26.6)
Seychelles	55 to 59	11.1 (3.5–24.8)	11.1 (3.4–24.7)	11.1 (3.5–24.3)	11.2 (3.7–26.1)	11.2 (3.8–25.8)

Supplementary Table S10: Prevalence of female SVAC by age and location for 1990, 2000, 2010, 2020, and 2023						
**Location**	**Age Range**	**1990**	**2000**	**2010**	**2020**	**2023**
Seychelles	60 to 64	10.7 (3.4–24.1)	10.7 (3.3–24.0)	10.7 (3.4–23.6)	10.9 (3.6–25.3)	10.9 (3.7–25.0)
Seychelles	65 to 69	9.9 (3.1–22.5)	9.9 (3.0–22.5)	9.9 (3.1–22.0)	10.1 (3.3–23.7)	10.1 (3.4–23.4)
Seychelles	70 to 74	7.9 (2.4–18.3)	7.9 (2.3–18.2)	7.9 (2.4–17.9)	8.0 (2.6–19.3)	8.0 (2.6–19.0)
Seychelles	75 to 79	7.1 (2.2–16.5)	7.1 (2.1–16.5)	7.1 (2.2–16.2)	7.2 (2.3–17.5)	7.2 (2.3–17.3)
Seychelles	80 to 84	5.0 (1.5–11.9)	4.9 (1.4–11.8)	5.0 (1.5–11.6)	5.0 (1.6–12.6)	5.0 (1.6–12.4)
Seychelles	85 to 89	4.5 (1.3–10.7)	4.4 (1.3–10.7)	4.4 (1.3–10.5)	4.5 (1.4–11.4)	4.5 (1.4–11.2)
Seychelles	90 to 94	4.0 (1.2– 9.7)	4.0 (1.1– 9.7)	4.0 (1.2– 9.5)	4.1 (1.3–10.3)	4.1 (1.3–10.2)
Seychelles	95 plus	4.0 (1.2– 9.6)	4.0 (1.1– 9.6)	4.0 (1.2– 9.4)	4.0 (1.2–10.2)	4.0 (1.3–10.1)
Seychelles	Age-standardized	10.9 (3.5–24.4)	10.9 (3.3–24.4)	10.9 (3.5–23.9)	11.1 (3.7–25.7)	11.1 (3.7–25.4)
Seychelles	All age	10.8 (3.5–24.2)	10.9 (3.3–24.4)	11.0 (3.5–24.1)	11.1 (3.7–25.7)	11.0 (3.7–25.3)
Sri Lanka	20 to 24	9.0 (2.9–20.3)	8.8 (3.4–17.6)	8.7 (4.7–14.6)	9.7 (5.8–15.1)	10.1 (5.7–16.5)
Sri Lanka	25 to 29	9.4 (3.1–20.8)	9.3 (3.6–18.3)	9.4 (5.4–15.3)	10.8 (6.7–16.0)	11.2 (6.4–17.7)
Sri Lanka	30 to 34	9.8 (3.3–21.4)	9.6 (3.7–19.0)	9.6 (5.5–15.6)	11.0 (6.9–16.3)	11.4 (6.7–17.9)
Sri Lanka	35 to 39	10.3 (3.5–22.2)	10.3 (4.1–20.0)	10.9 (6.6–17.0)	13.1 (8.7–18.9)	13.7 (8.5–20.9)
Sri Lanka	40 to 44	10.4 (3.6–22.3)	10.4 (4.2–20.2)	10.9 (6.6–17.1)	13.2 (8.7–19.0)	13.8 (8.5–21.1)

Supplementary Table S10: Prevalence of female SVAC by age and location for 1990, 2000, 2010, 2020, and 2023						
**Location**	**Age Range**	**1990**	**2000**	**2010**	**2020**	**2023**
Sri Lanka	45 to 49	10.2 (3.5–22.0)	10.2 (4.0–19.9)	10.7 (6.3–16.8)	12.9 (8.4–18.5)	13.4 (8.2–20.6)
Sri Lanka	50 to 54	12.0 (2.9–29.9)	11.7 (3.3–26.7)	11.6 (3.7–25.3)	11.9 (4.0–28.3)	12.0 (3.9–29.0)
Sri Lanka	55 to 59	11.6 (2.8–28.6)	11.3 (3.2–25.9)	11.2 (3.6–24.6)	11.5 (3.8–27.5)	11.6 (3.8–27.7)
Sri Lanka	60 to 64	11.2 (2.7–28.3)	11.0 (3.1–25.2)	10.9 (3.4–23.9)	11.1 (3.7–26.8)	11.3 (3.6–26.9)
Sri Lanka	65 to 69	10.4 (2.5–26.7)	10.2 (2.8–23.7)	10.1 (3.2–22.3)	10.3 (3.4–25.0)	10.5 (3.4–25.2)
Sri Lanka	70 to 74	8.3 (1.9–21.4)	8.1 (2.2–19.1)	8.0 (2.5–18.1)	8.2 (2.6–20.3)	8.3 (2.6–20.9)
Sri Lanka	75 to 79	7.5 (1.7–20.0)	7.3 (2.0–17.4)	7.2 (2.2–16.4)	7.4 (2.3–18.4)	7.5 (2.3–18.7)
Sri Lanka	80 to 84	5.3 (1.2–14.4)	5.1 (1.4–12.5)	5.0 (1.5–11.8)	5.2 (1.6–13.2)	5.2 (1.6–13.5)
Sri Lanka	85 to 89	4.7 (1.1–13.0)	4.6 (1.2–11.3)	4.5 (1.3–10.6)	4.6 (1.4–11.9)	4.7 (1.4–12.2)
Sri Lanka	90 to 94	4.3 (1.0–11.9)	4.1 (1.1–10.3)	4.1 (1.2– 9.6)	4.2 (1.3–10.9)	4.2 (1.3–11.1)
Sri Lanka	95 plus	4.2 (0.9–11.8)	4.1 (1.1–10.2)	4.0 (1.2– 9.5)	4.1 (1.3–10.5)	4.2 (1.3–11.0)
Sri Lanka	Age-standardized	9.9 (3.3–21.9)	9.8 (3.8–20.1)	9.9 (5.0–17.4)	11.1 (6.6–18.2)	11.5 (6.5–19.2)
Sri Lanka	All age	9.9 (3.3–21.7)	9.9 (3.8–20.0)	10.0 (5.1–17.6)	11.1 (6.5–18.8)	11.4 (6.3–19.5)
Thailand	20 to 24	13.1 (6.4–23.5)	12.8 (9.2–17.3)	13.0 (6.4–23.3)	13.2 (4.9–27.4)	13.2 (4.6–28.6)
Thailand	25 to 29	13.4 (6.6–24.0)	13.1 (9.5–17.6)	13.3 (6.6–23.7)	13.5 (5.0–27.9)	13.5 (4.7–29.1)

Supplementary Table S10: Prevalence of female SVAC by age and location for 1990, 2000, 2010, 2020, and 2023						
**Location**	**Age Range**	**1990**	**2000**	**2010**	**2020**	**2023**
Thailand	30 to 34	14.4 (7.1–25.5)	14.0 (10.2–18.7)	14.2 (7.2–25.2)	14.4 (5.4–29.6)	14.5 (5.1–30.9)
Thailand	35 to 39	14.3 (7.1–25.4)	14.0 (10.2–18.6)	14.2 (7.1–25.1)	14.5 (5.4–29.7)	14.5 (5.1–30.9)
Thailand	40 to 44	14.0 (6.9–24.9)	13.5 (9.8–18.0)	13.9 (7.0–24.7)	14.5 (5.5–29.7)	14.6 (5.2–31.1)
Thailand	45 to 49	14.6 (7.2–25.9)	14.3 (10.5–19.0)	14.5 (7.3–25.5)	14.6 (5.5–30.0)	14.7 (5.2–31.2)
Thailand	50 to 54	14.3 (4.7–30.8)	14.3 (4.5–30.8)	14.2 (4.7–30.1)	14.3 (4.9–32.0)	14.3 (5.0–31.6)
Thailand	55 to 59	13.8 (4.6–30.0)	13.8 (4.4–29.9)	13.7 (4.5–29.2)	13.8 (4.7–31.1)	13.8 (4.8–30.8)
Thailand	60 to 64	13.4 (4.4–29.1)	13.4 (4.2–29.1)	13.3 (4.3–28.4)	13.4 (4.6–30.3)	13.4 (4.6–29.8)
Thailand	65 to 69	12.4 (4.0–27.4)	12.4 (3.9–27.4)	12.4 (4.0–26.7)	12.4 (4.2–28.5)	12.4 (4.3–28.1)
Thailand	70 to 74	9.9 (3.1–22.5)	9.9 (3.0–22.5)	9.9 (3.1–21.9)	9.9 (3.3–23.5)	9.9 (3.3–23.1)
Thailand	75 to 79	8.9 (2.8–20.5)	8.9 (2.7–20.5)	8.9 (2.8–19.9)	8.9 (2.9–21.4)	8.9 (3.0–21.1)
Thailand	80 to 84	6.3 (1.9–14.9)	6.3 (1.8–14.9)	6.3 (1.9–14.5)	6.3 (2.0–15.6)	6.3 (2.0–15.4)
Thailand	85 to 89	5.7 (1.7–13.5)	5.7 (1.6–13.5)	5.6 (1.7–13.1)	5.7 (1.8–14.1)	5.7 (1.8–13.9)
Thailand	90 to 94	5.1 (1.5–12.3)	5.1 (1.5–12.2)	5.1 (1.5–11.9)	5.1 (1.6–12.9)	5.1 (1.6–12.7)
Thailand	95 plus	5.1 (1.5–12.2)	5.1 (1.5–12.1)	5.0 (1.5–11.8)	5.1 (1.6–12.7)	5.1 (1.6–12.5)
Thailand	Age-standardized	13.3 (6.1–24.8)	13.1 (8.7–19.3)	13.2 (10.3–17.5)	13.4 (8.8–20.8)	13.4 (8.0–22.7)

Supplementary Table S10: Prevalence of female SVAC by age and location for 1990, 2000, 2010, 2020, and 2023						
**Location**	**Age Range**	**1990**	**2000**	**2010**	**2020**	**2023**
Thailand	All age	13.6 (6.2–25.1)	13.3 (9.2–18.9)	13.3 (10.4–17.6)	13.2 (9.1–19.0)	13.1 (8.1–20.6)
Timor-Leste	20 to 24	26.0 (10.4–48.8)	26.9 (12.5–45.2)	27.8 (19.9–37.0)	27.8 (19.7–36.4)	27.6 (17.3–40.0)
Timor-Leste	25 to 29	27.5 (11.2–50.8)	29.3 (13.9–48.2)	31.3 (22.9–41.1)	31.4 (22.7–40.3)	30.9 (19.8–43.9)
Timor-Leste	30 to 34	28.9 (11.9–52.6)	30.2 (14.5–49.3)	31.7 (23.2–41.5)	31.7 (22.9–40.7)	31.4 (20.1–44.4)
Timor-Leste	35 to 39	31.1 (13.1–55.3)	34.3 (17.1–54.2)	38.1 (28.7–48.7)	38.2 (28.4–47.8)	37.3 (24.7–51.1)
Timor-Leste	40 to 44	31.7 (13.5–56.0)	34.6 (17.3–54.5)	38.1 (28.7–48.6)	38.1 (28.4–47.7)	37.3 (24.8–51.1)
Timor-Leste	45 to 49	30.9 (13.0–55.1)	33.6 (16.6–53.4)	36.9 (27.6–47.3)	36.9 (27.4–46.4)	36.2 (23.9–49.7)
Timor-Leste	50 to 54	27.4 (10.5–51.3)	27.5 (10.1–51.3)	27.9 (10.7–51.2)	28.4 (11.4–53.9)	28.4 (11.6–53.5)
Timor-Leste	55 to 59	26.5 (10.1–50.1)	26.6 (9.7–50.2)	27.0 (10.2–50.1)	27.5 (10.9–52.7)	27.5 (11.1–52.3)
Timor-Leste	60 to 64	25.7 (9.7–49.0)	25.8 (9.3–49.1)	26.2 (9.9–49.0)	26.7 (10.5–51.6)	26.7 (10.7–51.2)
Timor-Leste	65 to 69	24.2 (8.9–46.8)	24.2 (8.6–46.9)	24.7 (9.1–46.8)	25.1 (9.7–49.4)	25.1 (9.9–49.0)
Timor-Leste	70 to 74	19.9 (7.0–40.4)	20.0 (6.8–40.5)	20.3 (7.2–40.4)	20.7 (7.7–42.9)	20.7 (7.8–42.5)
Timor-Leste	75 to 79	18.2 (6.3–37.6)	18.2 (6.0–37.6)	18.6 (6.4–37.5)	18.9 (6.9–40.0)	18.9 (6.9–39.6)
Timor-Leste	80 to 84	13.3 (4.4–29.0)	13.3 (4.2–29.1)	13.6 (4.4–29.0)	13.9 (4.8–31.2)	13.9 (4.8–30.8)
Timor-Leste	85 to 89	12.1 (3.9–26.7)	12.1 (3.7–26.7)	12.3 (4.0–26.7)	12.6 (4.3–28.8)	12.6 (4.3–28.4)

Supplementary Table S10: Prevalence of female SVAC by age and location for 1990, 2000, 2010, 2020, and 2023						
**Location**	**Age Range**	**1990**	**2000**	**2010**	**2020**	**2023**
Timor-Leste	90 to 94	11.0 (3.5–24.6)	11.0 (3.4–24.6)	11.2 (3.6–24.6)	11.5 (3.8–26.6)	11.5 (3.9–26.3)
Timor-Leste	95 plus	10.9 (3.5–24.4)	10.9 (3.3–24.5)	11.1 (3.5–24.4)	11.4 (3.8–26.4)	11.4 (3.9–26.0)
Timor-Leste	Age-standardized	27.2 (11.2–49.6)	28.6 (13.2–47.9)	30.3 (19.8–43.5)	30.5 (26.0–36.1)	30.1 (24.6–36.5)
Timor-Leste	All age	28.1 (11.5–50.2)	29.6 (14.1–48.8)	30.7 (20.6–42.9)	30.7 (26.2–35.5)	30.4 (24.2–38.0)
Viet Nam	20 to 24	7.0 (4.5–10.4)	6.9 (4.8– 9.7)	7.3 (5.1–10.0)	8.2 (5.0–12.5)	8.4 (4.8–13.7)
Viet Nam	25 to 29	7.0 (3.8–11.8)	6.6 (4.6– 9.1)	7.2 (5.1– 9.7)	8.9 (5.8–12.9)	9.2 (5.6–14.4)
Viet Nam	30 to 34	7.2 (3.2–13.6)	6.6 (4.2– 9.7)	6.9 (4.9– 9.3)	9.0 (5.9–13.0)	9.5 (5.8–14.6)
Viet Nam	35 to 39	7.4 (2.8–15.3)	6.8 (3.7–11.0)	6.4 (4.6– 8.6)	6.8 (4.1–10.4)	6.9 (3.9–11.3)
Viet Nam	40 to 44	7.4 (2.5–15.8)	6.7 (3.2–12.1)	6.1 (4.2– 8.4)	6.1 (3.5– 9.5)	6.1 (3.2–10.4)
Viet Nam	45 to 49	7.0 (2.3–15.4)	6.2 (2.8–11.3)	5.5 (3.4– 8.3)	5.4 (2.9– 9.0)	5.4 (2.6–10.0)
Viet Nam	50 to 54	6.7 (2.2–15.1)	6.0 (2.7–11.3)	5.6 (3.4– 8.6)	6.0 (3.4– 9.7)	6.1 (3.2–10.5)
Viet Nam	55 to 59	6.2 (2.0–14.0)	5.5 (2.4–10.6)	5.3 (3.1– 8.4)	6.2 (3.5– 9.8)	6.4 (3.4–11.0)
Viet Nam	60 to 64	7.1 (2.2–16.2)	6.9 (2.3–15.2)	6.7 (3.1–12.5)	6.6 (3.6–10.8)	6.6 (3.3–11.4)
Viet Nam	65 to 69	6.6 (2.0–15.4)	6.5 (1.9–15.4)	6.5 (2.0–15.0)	6.6 (2.1–16.7)	6.7 (2.0–16.6)
Viet Nam	70 to 74	5.2 (1.5–12.3)	5.1 (1.5–12.3)	5.1 (1.5–11.9)	5.2 (1.6–13.3)	5.2 (1.5–13.3)

Supplementary Table S10: Prevalence of female SVAC by age and location for 1990, 2000, 2010, 2020, and 2023						
**Location**	**Age Range**	**1990**	**2000**	**2010**	**2020**	**2023**
Viet Nam	75 to 79	4.6 (1.4–11.1)	4.6 (1.3–11.0)	4.6 (1.4–10.7)	4.6 (1.4–12.0)	4.7 (1.4–12.0)
Viet Nam	80 to 84	3.2 (0.9– 7.8)	3.2 (0.9– 7.8)	3.2 (0.9– 7.6)	3.2 (1.0– 8.5)	3.3 (0.9– 8.5)
Viet Nam	85 to 89	2.9 (0.8– 7.0)	2.8 (0.8– 7.0)	2.8 (0.8– 6.8)	2.9 (0.9– 7.8)	2.9 (0.8– 7.7)
Viet Nam	90 to 94	2.6 (0.7– 6.3)	2.6 (0.7– 6.3)	2.6 (0.7– 6.1)	2.6 (0.8– 6.9)	2.6 (0.7– 6.9)
Viet Nam	95 plus	2.5 (0.7– 6.3)	2.5 (0.7– 6.2)	2.5 (0.7– 6.1)	2.6 (0.8– 6.8)	2.6 (0.7– 6.8)
Viet Nam	Age-standardized	6.8 (2.9–13.5)	6.3 (3.6–10.4)	6.2 (4.3– 8.8)	6.9 (4.9–10.1)	7.1 (4.8–10.8)
Viet Nam	All age	6.8 (3.1–13.2)	6.4 (3.8–10.3)	6.3 (4.3– 8.7)	6.9 (4.9– 9.9)	7.0 (4.8–10.6)
Sub-Saharan Africa	20 to 24	22.8 (10.9–39.5)	23.0 (12.0–38.6)	23.1 (13.7–36.2)	22.7 (13.9–36.5)	22.6 (13.3–37.2)
Sub-Saharan Africa	25 to 29	22.7 (10.2–40.4)	22.6 (11.0–39.0)	22.7 (12.2–37.4)	23.2 (13.0–38.5)	23.3 (12.8–39.0)
Sub-Saharan Africa	30 to 34	23.7 (10.3–43.0)	23.4 (10.4–41.3)	22.8 (11.6–38.3)	23.0 (11.6–40.1)	23.0 (11.4–40.5)
Sub-Saharan Africa	35 to 39	23.6 (10.0–42.5)	23.4 (10.3–42.1)	23.0 (11.5–38.8)	23.1 (11.4–40.6)	23.1 (11.2–41.1)
Sub-Saharan Africa	40 to 44	23.9 (10.0–43.5)	23.6 (10.2–42.3)	23.2 (11.4–39.7)	23.4 (12.3–40.8)	23.6 (11.7–41.4)
Sub-Saharan Africa	45 to 49	23.9 (10.0–44.3)	23.6 (10.1–43.3)	23.1 (10.8–40.7)	23.0 (11.0–41.7)	23.1 (10.8–41.9)
Sub-Saharan Africa	50 to 54	23.4 (9.7–43.8)	23.2 (9.8–43.0)	22.9 (10.7–40.4)	22.8 (10.8–41.2)	22.8 (10.5–41.9)
Sub-Saharan Africa	55 to 59	22.2 (8.6–42.8)	21.8 (8.3–41.8)	21.5 (9.0–39.5)	21.5 (9.9–39.9)	21.6 (9.7–40.0)

Supplementary Table S10: Prevalence of female SVAC by age and location for 1990, 2000, 2010, 2020, and 2023						
**Location**	**Age Range**	**1990**	**2000**	**2010**	**2020**	**2023**
Sub-Saharan Africa	60 to 64	21.7 (8.4–42.4)	21.2 (7.9–41.2)	21.0 (8.5–39.3)	21.1 (9.2–40.0)	21.2 (9.1–40.0)
Sub-Saharan Africa	65 to 69	20.7 (7.6–40.4)	20.3 (7.2–40.6)	20.4 (7.4–39.9)	20.7 (7.8–42.4)	20.8 (8.1–42.0)
Sub-Saharan Africa	70 to 74	17.1 (6.0–34.7)	16.7 (5.5–34.9)	16.6 (5.7–34.0)	16.9 (6.0–36.8)	17.0 (6.1–36.5)
Sub-Saharan Africa	75 to 79	15.5 (5.4–32.0)	15.4 (5.0–32.7)	15.1 (5.1–31.5)	15.4 (5.3–34.0)	15.4 (5.4–33.7)
Sub-Saharan Africa	80 to 84	11.4 (3.7–24.7)	11.3 (3.5–25.1)	11.1 (3.5–24.1)	11.1 (3.7–26.1)	11.2 (3.8–26.0)
Sub-Saharan Africa	85 to 89	10.4 (3.3–22.9)	10.2 (3.2–23.1)	10.2 (3.2–22.4)	10.1 (3.3–24.0)	10.2 (3.4–24.0)
Sub-Saharan Africa	90 to 94	9.5 (3.0–21.2)	9.5 (2.9–21.5)	9.3 (2.9–20.7)	9.3 (3.0–22.3)	9.3 (3.0–22.1)
Sub-Saharan Africa	95 plus	9.6 (3.0–21.3)	9.5 (2.9–21.5)	9.2 (2.9–20.5)	9.3 (3.0–22.3)	9.2 (3.1–22.0)
Sub-Saharan Africa	Age-standardized	22.2 (9.4–40.9)	22.0 (9.6–40.1)	21.8 (10.7–37.8)	21.9 (11.1–38.9)	21.9 (10.7–39.2)
Sub-Saharan Africa	All age	22.7 (9.8–41.2)	22.6 (10.2–40.2)	22.4 (11.5–37.8)	22.5 (12.0–39.0)	22.5 (11.3–39.5)
Central sub-Saharan Africa	20 to 24	17.0 (5.8–35.6)	17.0 (5.6–35.5)	17.1 (5.8–35.1)	17.4 (6.2–37.4)	17.4 (6.3–37.0)
Central sub-Saharan Africa	25 to 29	17.5 (6.0–36.4)	17.5 (5.8–36.4)	17.6 (6.0–36.0)	17.9 (6.4–38.3)	17.9 (6.5–37.9)
Central sub-Saharan Africa	30 to 34	18.9 (6.6–38.8)	18.9 (6.3–38.7)	19.1 (6.6–38.3)	19.3 (7.0–40.7)	19.3 (7.1–40.3)
Central sub-Saharan Africa	35 to 39	19.2 (6.7–39.2)	19.1 (6.4–39.1)	19.3 (6.7–38.7)	19.5 (7.1–41.0)	19.5 (7.2–40.6)
Central sub-Saharan Africa	40 to 44	19.8 (7.0–40.1)	19.8 (6.7–40.1)	19.9 (7.0–39.7)	20.2 (7.4–42.0)	20.1 (7.5–41.6)

Supplementary Table S10: Prevalence of female SVAC by age and location for 1990, 2000, 2010, 2020, and 2023						
**Location**	**Age Range**	**1990**	**2000**	**2010**	**2020**	**2023**
Central sub-Saharan Africa	45 to 49	19.3 (6.8–39.5)	19.4 (6.5–39.4)	19.5 (6.8–39.0)	19.7 (7.2–41.3)	19.7 (7.3–40.9)
Central sub-Saharan Africa	50 to 54	19.0 (6.6–38.9)	19.0 (6.3–38.8)	19.1 (6.6–38.4)	19.3 (7.0–40.7)	19.3 (7.1–40.3)
Central sub-Saharan Africa	55 to 59	18.4 (6.4–38.0)	18.4 (6.1–37.9)	18.6 (6.4–37.5)	18.8 (6.8–39.8)	18.7 (6.9–39.3)
Central sub-Saharan Africa	60 to 64	17.9 (6.2–37.1)	17.9 (5.9–37.0)	18.0 (6.2–36.6)	18.2 (6.5–38.8)	18.2 (6.7–38.4)
Central sub-Saharan Africa	65 to 69	16.7 (5.7–35.1)	16.7 (5.5–35.1)	16.9 (5.7–34.7)	17.0 (6.0–36.9)	17.0 (6.1–36.4)
Central sub-Saharan Africa	70 to 74	13.6 (4.5–29.5)	13.6 (4.3–29.5)	13.7 (4.5–29.1)	13.8 (4.7–31.0)	13.8 (4.8–30.7)
Central sub-Saharan Africa	75 to 79	12.3 (4.0–27.0)	12.3 (3.8–27.1)	12.4 (4.0–26.7)	12.5 (4.2–28.6)	12.5 (4.3–28.2)
Central sub-Saharan Africa	80 to 84	8.8 (2.7–20.2)	8.8 (2.6–20.1)	8.9 (2.8–19.9)	9.0 (2.9–21.4)	9.0 (3.0–21.1)
Central sub-Saharan Africa	85 to 89	7.9 (2.4–18.3)	7.9 (2.3–18.4)	8.0 (2.5–18.2)	8.1 (2.6–19.6)	8.1 (2.7–19.3)
Central sub-Saharan Africa	90 to 94	7.1 (2.2–16.7)	7.2 (2.1–16.8)	7.3 (2.2–16.6)	7.4 (2.4–18.0)	7.4 (2.4–17.7)
Central sub-Saharan Africa	95 plus	7.1 (2.2–16.6)	7.1 (2.1–16.6)	7.2 (2.2–16.5)	7.3 (2.3–17.8)	7.3 (2.4–17.6)
Central sub-Saharan Africa	Age-standardized	17.8 (6.1–36.7)	17.8 (5.9–36.7)	17.9 (6.2–36.3)	18.1 (6.5–38.6)	18.1 (6.6–38.2)
Central sub-Saharan Africa	All age	18.1 (6.3–37.3)	18.0 (6.0–37.2)	18.2 (6.3–36.9)	18.4 (6.7–39.2)	18.4 (6.7–38.8)
Angola	20 to 24	17.3 (5.9–36.0)	17.2 (5.6–35.9)	17.4 (5.9–35.5)	17.6 (6.3–37.8)	17.6 (6.3–37.4)
Angola	25 to 29	17.8 (6.1–36.9)	17.8 (5.9–36.8)	17.9 (6.1–36.4)	18.1 (6.5–38.7)	18.1 (6.6–38.3)

Supplementary Table S10: Prevalence of female SVAC by age and location for 1990, 2000, 2010, 2020, and 2023						
**Location**	**Age Range**	**1990**	**2000**	**2010**	**2020**	**2023**
Angola	30 to 34	19.2 (6.7–39.3)	19.2 (6.4–39.2)	19.3 (6.7–38.8)	19.6 (7.1–41.1)	19.5 (7.2–40.7)
Angola	35 to 39	19.4 (6.8–39.6)	19.4 (6.5–39.5)	19.6 (6.8–39.1)	19.8 (7.2–41.4)	19.8 (7.3–41.0)
Angola	40 to 44	20.1 (7.1–40.7)	20.0 (6.8–40.6)	20.2 (7.1–40.1)	20.4 (7.5–42.5)	20.4 (7.6–42.0)
Angola	45 to 49	19.6 (6.9–40.0)	19.6 (6.6–39.9)	19.8 (6.9–39.5)	20.0 (7.3–41.7)	19.9 (7.4–41.3)
Angola	50 to 54	19.3 (6.8–39.4)	19.2 (6.4–39.3)	19.4 (6.7–38.9)	19.6 (7.1–41.1)	19.6 (7.2–40.7)
Angola	55 to 59	18.7 (6.5–38.4)	18.7 (6.2–38.4)	18.8 (6.5–37.9)	19.0 (6.9–40.2)	19.0 (7.0–39.7)
Angola	60 to 64	18.2 (6.3–37.5)	18.1 (6.0–37.5)	18.3 (6.3–37.0)	18.4 (6.6–39.2)	18.4 (6.7–38.8)
Angola	65 to 69	17.0 (5.8–35.6)	17.0 (5.5–35.5)	17.1 (5.8–35.1)	17.2 (6.1–37.2)	17.2 (6.2–36.8)
Angola	70 to 74	13.8 (4.5–29.8)	13.7 (4.3–29.8)	13.8 (4.5–29.4)	14.0 (4.8–31.4)	14.0 (4.9–31.0)
Angola	75 to 79	12.5 (4.0–27.4)	12.4 (3.9–27.4)	12.5 (4.0–27.0)	12.6 (4.3–28.9)	12.6 (4.3–28.5)
Angola	80 to 84	8.9 (2.8–20.4)	8.9 (2.7–20.4)	9.0 (2.8–20.1)	9.1 (2.9–21.6)	9.1 (3.0–21.3)
Angola	85 to 89	8.1 (2.5–18.6)	8.0 (2.4–18.6)	8.1 (2.5–18.3)	8.2 (2.6–19.7)	8.2 (2.7–19.5)
Angola	90 to 94	7.3 (2.2–17.0)	7.3 (2.1–17.0)	7.3 (2.2–16.7)	7.4 (2.4–18.1)	7.4 (2.4–17.8)
Angola	95 plus	7.2 (2.2–16.9)	7.2 (2.1–16.8)	7.3 (2.2–16.6)	7.3 (2.3–17.9)	7.4 (2.4–17.7)
Angola	Age-standardized	18.0 (6.3–37.2)	18.0 (6.0–37.1)	18.1 (6.2–36.7)	18.3 (6.6–39.0)	18.3 (6.7–38.5)

Supplementary Table S10: Prevalence of female SVAC by age and location for 1990, 2000, 2010, 2020, and 2023						
**Location**	**Age Range**	**1990**	**2000**	**2010**	**2020**	**2023**
Angola	All age	18.4 (6.4–37.9)	18.4 (6.1–37.8)	18.5 (6.4–37.4)	18.7 (6.8–39.7)	18.7 (6.8–39.3)
Central African Republic	20 to 24	12.5 (4.0–27.4)	12.4 (3.9–27.3)	12.5 (4.0–26.9)	12.6 (4.2–28.7)	12.6 (4.3–28.4)
Central African Republic	25 to 29	12.8 (4.2–28.0)	12.7 (4.0–27.9)	12.8 (4.1–27.4)	12.9 (4.4–29.3)	12.9 (4.4–29.0)
Central African Republic	30 to 34	13.8 (4.6–30.0)	13.8 (4.3–29.9)	13.8 (4.5–29.4)	13.9 (4.8–31.3)	13.9 (4.9–30.9)
Central African Republic	35 to 39	14.0 (4.6–30.3)	14.0 (4.4–30.2)	14.0 (4.6–29.7)	14.1 (4.9–31.6)	14.1 (4.9–31.3)
Central African Republic	40 to 44	14.5 (4.8–31.1)	14.4 (4.6–31.1)	14.5 (4.8–30.6)	14.6 (5.0–32.6)	14.6 (5.1–32.0)
Central African Republic	45 to 49	14.1 (4.7–30.5)	14.1 (4.5–30.4)	14.1 (4.6–29.9)	14.2 (4.9–31.9)	14.2 (5.0–31.5)
Central African Republic	50 to 54	13.8 (4.6–30.0)	13.8 (4.3–29.9)	13.8 (4.5–29.4)	13.9 (4.8–31.3)	13.9 (4.9–30.9)
Central African Republic	55 to 59	13.4 (4.4–29.2)	13.4 (4.2–29.1)	13.4 (4.4–28.6)	13.5 (4.6–30.5)	13.5 (4.7–30.2)
Central African Republic	60 to 64	13.0 (4.2–28.4)	12.9 (4.0–28.3)	13.0 (4.2–27.8)	13.1 (4.5–29.7)	13.1 (4.5–29.4)
Central African Republic	65 to 69	12.1 (3.9–26.7)	12.0 (3.7–26.6)	12.1 (3.9–26.2)	12.2 (4.1–28.0)	12.2 (4.2–27.6)
Central African Republic	70 to 74	9.6 (3.0–21.9)	9.6 (2.9–21.8)	9.6 (3.0–21.4)	9.7 (3.2–23.0)	9.7 (3.2–22.7)
Central African Republic	75 to 79	8.7 (2.7–19.9)	8.6 (2.6–19.9)	8.7 (2.7–19.5)	8.7 (2.8–21.0)	8.8 (2.9–20.7)
Central African Republic	80 to 84	6.1 (1.8–14.4)	6.1 (1.8–14.4)	6.1 (1.8–14.1)	6.2 (1.9–15.3)	6.2 (2.0–15.1)
Central African Republic	85 to 89	5.5 (1.7–13.1)	5.5 (1.6–13.0)	5.5 (1.6–12.8)	5.5 (1.7–13.8)	5.6 (1.8–13.6)

Supplementary Table S10: Prevalence of female SVAC by age and location for 1990, 2000, 2010, 2020, and 2023						
**Location**	**Age Range**	**1990**	**2000**	**2010**	**2020**	**2023**
Central African Republic	90 to 94	5.0 (1.5–11.9)	5.0 (1.4–11.8)	5.0 (1.5–11.6)	5.0 (1.6–12.6)	5.0 (1.6–12.4)
Central African Republic	95 plus	4.9 (1.5–11.8)	4.9 (1.4–11.7)	4.9 (1.5–11.5)	5.0 (1.5–12.5)	5.0 (1.6–12.3)
Central African Republic	Age-standardized	12.9 (4.2–28.2)	12.9 (4.0–28.2)	12.9 (4.2–27.7)	13.0 (4.4–29.6)	13.0 (4.5–29.2)
Central African Republic	All age	13.2 (4.3–28.8)	13.2 (4.1–28.7)	13.2 (4.3–28.2)	13.3 (4.6–30.2)	13.3 (4.6–29.8)
Congo (Brazzaville)	20 to 24	17.3 (5.9–36.0)	17.2 (5.6–35.9)	17.4 (5.9–35.5)	17.6 (6.3–37.8)	17.6 (6.3–37.4)
Congo (Brazzaville)	25 to 29	17.8 (6.1–36.9)	17.8 (5.9–36.8)	17.9 (6.1–36.4)	18.1 (6.5–38.7)	18.1 (6.6–38.3)
Congo (Brazzaville)	30 to 34	19.2 (6.7–39.3)	19.2 (6.4–39.2)	19.3 (6.7–38.8)	19.6 (7.1–41.1)	19.5 (7.2–40.7)
Congo (Brazzaville)	35 to 39	19.4 (6.8–39.6)	19.4 (6.5–39.5)	19.6 (6.8–39.1)	19.8 (7.2–41.4)	19.8 (7.3–41.0)
Congo (Brazzaville)	40 to 44	20.1 (7.1–40.7)	20.0 (6.8–40.6)	20.2 (7.1–40.1)	20.4 (7.5–42.5)	20.4 (7.6–42.0)
Congo (Brazzaville)	45 to 49	19.6 (6.9–40.0)	19.6 (6.6–39.9)	19.8 (6.9–39.5)	20.0 (7.3–41.7)	19.9 (7.4–41.3)
Congo (Brazzaville)	50 to 54	19.3 (6.8–39.4)	19.2 (6.4–39.3)	19.4 (6.7–38.9)	19.6 (7.1–41.1)	19.6 (7.2–40.7)
Congo (Brazzaville)	55 to 59	18.7 (6.5–38.4)	18.7 (6.2–38.4)	18.8 (6.5–37.9)	19.0 (6.9–40.2)	19.0 (7.0–39.7)
Congo (Brazzaville)	60 to 64	18.2 (6.3–37.5)	18.1 (6.0–37.5)	18.3 (6.3–37.0)	18.4 (6.6–39.2)	18.4 (6.7–38.8)
Congo (Brazzaville)	65 to 69	17.0 (5.8–35.6)	17.0 (5.5–35.5)	17.1 (5.8–35.1)	17.2 (6.1–37.2)	17.2 (6.2–36.8)
Congo (Brazzaville)	70 to 74	13.8 (4.5–29.8)	13.7 (4.3–29.8)	13.8 (4.5–29.4)	14.0 (4.8–31.4)	14.0 (4.9–31.0)

Supplementary Table S10: Prevalence of female SVAC by age and location for 1990, 2000, 2010, 2020, and 2023						
**Location**	**Age Range**	**1990**	**2000**	**2010**	**2020**	**2023**
Congo (Brazzaville)	75 to 79	12.5 (4.0–27.4)	12.4 (3.9–27.4)	12.5 (4.0–27.0)	12.6 (4.3–28.9)	12.6 (4.3–28.5)
Congo (Brazzaville)	80 to 84	8.9 (2.8–20.4)	8.9 (2.7–20.4)	9.0 (2.8–20.1)	9.1 (2.9–21.6)	9.1 (3.0–21.3)
Congo (Brazzaville)	85 to 89	8.1 (2.5–18.6)	8.0 (2.4–18.6)	8.1 (2.5–18.3)	8.2 (2.6–19.7)	8.2 (2.7–19.5)
Congo (Brazzaville)	90 to 94	7.3 (2.2–17.0)	7.3 (2.1–17.0)	7.3 (2.2–16.7)	7.4 (2.4–18.1)	7.4 (2.4–17.8)
Congo (Brazzaville)	95 plus	7.2 (2.2–16.9)	7.2 (2.1–16.8)	7.3 (2.2–16.6)	7.3 (2.3–17.9)	7.4 (2.4–17.7)
Congo (Brazzaville)	Age-standardized	18.0 (6.3–37.2)	18.0 (6.0–37.1)	18.1 (6.2–36.7)	18.3 (6.6–39.0)	18.3 (6.7–38.5)
Congo (Brazzaville)	All age	18.3 (6.3–37.7)	18.3 (6.1–37.6)	18.5 (6.4–37.3)	18.7 (6.8–39.6)	18.7 (6.8–39.2)
DR Congo	20 to 24	17.3 (5.9–36.0)	17.2 (5.6–35.9)	17.4 (5.9–35.5)	17.6 (6.3–37.8)	17.6 (6.3–37.4)
DR Congo	25 to 29	17.8 (6.1–36.9)	17.8 (5.9–36.8)	17.9 (6.1–36.4)	18.1 (6.5–38.7)	18.1 (6.6–38.3)
DR Congo	30 to 34	19.2 (6.7–39.3)	19.2 (6.4–39.2)	19.3 (6.7–38.8)	19.6 (7.1–41.1)	19.5 (7.2–40.7)
DR Congo	35 to 39	19.4 (6.8–39.6)	19.4 (6.5–39.5)	19.6 (6.8–39.1)	19.8 (7.2–41.4)	19.8 (7.3–41.0)
DR Congo	40 to 44	20.1 (7.1–40.7)	20.0 (6.8–40.6)	20.2 (7.1–40.1)	20.4 (7.5–42.5)	20.4 (7.6–42.0)
DR Congo	45 to 49	19.6 (6.9–40.0)	19.6 (6.6–39.9)	19.8 (6.9–39.5)	20.0 (7.3–41.7)	19.9 (7.4–41.3)
DR Congo	50 to 54	19.3 (6.8–39.4)	19.2 (6.4–39.3)	19.4 (6.7–38.9)	19.6 (7.1–41.1)	19.6 (7.2–40.7)
DR Congo	55 to 59	18.7 (6.5–38.4)	18.7 (6.2–38.4)	18.8 (6.5–37.9)	19.0 (6.9–40.2)	19.0 (7.0–39.7)

Supplementary Table S10: Prevalence of female SVAC by age and location for 1990, 2000, 2010, 2020, and 2023						
**Location**	**Age Range**	**1990**	**2000**	**2010**	**2020**	**2023**
DR Congo	60 to 64	18.2 (6.3–37.5)	18.1 (6.0–37.5)	18.3 (6.3–37.0)	18.4 (6.6–39.2)	18.4 (6.7–38.8)
DR Congo	65 to 69	17.0 (5.8–35.6)	17.0 (5.5–35.5)	17.1 (5.8–35.1)	17.2 (6.1–37.2)	17.2 (6.2–36.8)
DR Congo	70 to 74	13.8 (4.5–29.8)	13.7 (4.3–29.8)	13.8 (4.5–29.4)	14.0 (4.8–31.4)	14.0 (4.9–31.0)
DR Congo	75 to 79	12.5 (4.0–27.4)	12.4 (3.9–27.4)	12.5 (4.0–27.0)	12.6 (4.3–28.9)	12.6 (4.3–28.5)
DR Congo	80 to 84	8.9 (2.8–20.4)	8.9 (2.7–20.4)	9.0 (2.8–20.1)	9.1 (2.9–21.6)	9.1 (3.0–21.3)
DR Congo	85 to 89	8.1 (2.5–18.6)	8.0 (2.4–18.6)	8.1 (2.5–18.3)	8.2 (2.6–19.7)	8.2 (2.7–19.5)
DR Congo	90 to 94	7.3 (2.2–17.0)	7.3 (2.1–17.0)	7.3 (2.2–16.7)	7.4 (2.4–18.1)	7.4 (2.4–17.8)
DR Congo	95 plus	7.2 (2.2–16.9)	7.2 (2.1–16.8)	7.3 (2.2–16.6)	7.3 (2.3–17.9)	7.4 (2.4–17.7)
DR Congo	Age-standardized	18.0 (6.3–37.2)	18.0 (6.0–37.1)	18.1 (6.2–36.7)	18.3 (6.6–39.0)	18.3 (6.7–38.5)
DR Congo	All age	18.4 (6.4–37.8)	18.3 (6.1–37.7)	18.4 (6.4–37.2)	18.7 (6.8–39.6)	18.7 (6.8–39.2)
Equatorial Guinea	20 to 24	17.3 (5.9–36.0)	17.2 (5.6–35.9)	17.4 (5.9–35.5)	17.6 (6.3–37.8)	17.6 (6.3–37.4)
Equatorial Guinea	25 to 29	17.8 (6.1–36.9)	17.8 (5.9–36.8)	17.9 (6.1–36.4)	18.1 (6.5–38.7)	18.1 (6.6–38.3)
Equatorial Guinea	30 to 34	19.2 (6.7–39.3)	19.2 (6.4–39.2)	19.3 (6.7–38.8)	19.6 (7.1–41.1)	19.5 (7.2–40.7)
Equatorial Guinea	35 to 39	19.4 (6.8–39.6)	19.4 (6.5–39.5)	19.6 (6.8–39.1)	19.8 (7.2–41.4)	19.8 (7.3–41.0)
Equatorial Guinea	40 to 44	20.1 (7.1–40.7)	20.0 (6.8–40.6)	20.2 (7.1–40.1)	20.4 (7.5–42.5)	20.4 (7.6–42.0)

Supplementary Table S10: Prevalence of female SVAC by age and location for 1990, 2000, 2010, 2020, and 2023						
**Location**	**Age Range**	**1990**	**2000**	**2010**	**2020**	**2023**
Equatorial Guinea	45 to 49	19.6 (6.9–40.0)	19.6 (6.6–39.9)	19.8 (6.9–39.5)	20.0 (7.3–41.7)	19.9 (7.4–41.3)
Equatorial Guinea	50 to 54	19.3 (6.8–39.4)	19.2 (6.4–39.3)	19.4 (6.7–38.9)	19.6 (7.1–41.1)	19.6 (7.2–40.7)
Equatorial Guinea	55 to 59	18.7 (6.5–38.4)	18.7 (6.2–38.4)	18.8 (6.5–37.9)	19.0 (6.9–40.2)	19.0 (7.0–39.7)
Equatorial Guinea	60 to 64	18.2 (6.3–37.5)	18.1 (6.0–37.5)	18.3 (6.3–37.0)	18.4 (6.6–39.2)	18.4 (6.7–38.8)
Equatorial Guinea	65 to 69	17.0 (5.8–35.6)	17.0 (5.5–35.5)	17.1 (5.8–35.1)	17.2 (6.1–37.2)	17.2 (6.2–36.8)
Equatorial Guinea	70 to 74	13.8 (4.5–29.8)	13.7 (4.3–29.8)	13.8 (4.5–29.4)	14.0 (4.8–31.4)	14.0 (4.9–31.0)
Equatorial Guinea	75 to 79	12.5 (4.0–27.4)	12.4 (3.9–27.4)	12.5 (4.0–27.0)	12.6 (4.3–28.9)	12.6 (4.3–28.5)
Equatorial Guinea	80 to 84	8.9 (2.8–20.4)	8.9 (2.7–20.4)	9.0 (2.8–20.1)	9.1 (2.9–21.6)	9.1 (3.0–21.3)
Equatorial Guinea	85 to 89	8.1 (2.5–18.6)	8.0 (2.4–18.6)	8.1 (2.5–18.3)	8.2 (2.6–19.7)	8.2 (2.7–19.5)
Equatorial Guinea	90 to 94	7.3 (2.2–17.0)	7.3 (2.1–17.0)	7.3 (2.2–16.7)	7.4 (2.4–18.1)	7.4 (2.4–17.8)
Equatorial Guinea	95 plus	7.2 (2.2–16.9)	7.2 (2.1–16.8)	7.3 (2.2–16.6)	7.3 (2.3–17.9)	7.4 (2.4–17.7)
Equatorial Guinea	Age-standardized	18.0 (6.3–37.2)	18.0 (6.0–37.1)	18.1 (6.2–36.7)	18.3 (6.6–39.0)	18.3 (6.7–38.5)
Equatorial Guinea	All age	18.3 (6.4–37.7)	18.3 (6.1–37.7)	18.4 (6.4–37.3)	18.7 (6.8–39.6)	18.7 (6.8–39.3)
Gabon	20 to 24	17.3 (5.9–36.0)	17.2 (5.6–35.9)	17.4 (5.9–35.5)	17.6 (6.3–37.8)	17.6 (6.3–37.4)
Gabon	25 to 29	17.8 (6.1–36.9)	17.8 (5.9–36.8)	17.9 (6.1–36.4)	18.1 (6.5–38.7)	18.1 (6.6–38.3)

Supplementary Table S10: Prevalence of female SVAC by age and location for 1990, 2000, 2010, 2020, and 2023						
**Location**	**Age Range**	**1990**	**2000**	**2010**	**2020**	**2023**
Gabon	30 to 34	19.2 (6.7–39.3)	19.2 (6.4–39.2)	19.3 (6.7–38.8)	19.6 (7.1–41.1)	19.5 (7.2–40.7)
Gabon	35 to 39	19.4 (6.8–39.6)	19.4 (6.5–39.5)	19.6 (6.8–39.1)	19.8 (7.2–41.4)	19.8 (7.3–41.0)
Gabon	40 to 44	20.1 (7.1–40.7)	20.0 (6.8–40.6)	20.2 (7.1–40.1)	20.4 (7.5–42.5)	20.4 (7.6–42.0)
Gabon	45 to 49	19.6 (6.9–40.0)	19.6 (6.6–39.9)	19.8 (6.9–39.5)	20.0 (7.3–41.7)	19.9 (7.4–41.3)
Gabon	50 to 54	19.3 (6.8–39.4)	19.2 (6.4–39.3)	19.4 (6.7–38.9)	19.6 (7.1–41.1)	19.6 (7.2–40.7)
Gabon	55 to 59	18.7 (6.5–38.4)	18.7 (6.2–38.4)	18.8 (6.5–37.9)	19.0 (6.9–40.2)	19.0 (7.0–39.7)
Gabon	60 to 64	18.2 (6.3–37.5)	18.1 (6.0–37.5)	18.3 (6.3–37.0)	18.4 (6.6–39.2)	18.4 (6.7–38.8)
Gabon	65 to 69	17.0 (5.8–35.6)	17.0 (5.5–35.5)	17.1 (5.8–35.1)	17.2 (6.1–37.2)	17.2 (6.2–36.8)
Gabon	70 to 74	13.8 (4.5–29.8)	13.7 (4.3–29.8)	13.8 (4.5–29.4)	14.0 (4.8–31.4)	14.0 (4.9–31.0)
Gabon	75 to 79	12.5 (4.0–27.4)	12.4 (3.9–27.4)	12.5 (4.0–27.0)	12.6 (4.3–28.9)	12.6 (4.3–28.5)
Gabon	80 to 84	8.9 (2.8–20.4)	8.9 (2.7–20.4)	9.0 (2.8–20.1)	9.1 (2.9–21.6)	9.1 (3.0–21.3)
Gabon	85 to 89	8.1 (2.5–18.6)	8.0 (2.4–18.6)	8.1 (2.5–18.3)	8.2 (2.6–19.7)	8.2 (2.7–19.5)
Gabon	90 to 94	7.3 (2.2–17.0)	7.3 (2.1–17.0)	7.3 (2.2–16.7)	7.4 (2.4–18.1)	7.4 (2.4–17.8)
Gabon	95 plus	7.2 (2.2–16.9)	7.2 (2.1–16.8)	7.3 (2.2–16.6)	7.3 (2.3–17.9)	7.4 (2.4–17.7)
Gabon	Age-standardized	18.0 (6.3–37.2)	18.0 (6.0–37.1)	18.1 (6.2–36.7)	18.3 (6.6–39.0)	18.3 (6.7–38.5)

Supplementary Table S10: Prevalence of female SVAC by age and location for 1990, 2000, 2010, 2020, and 2023						
**Location**	**Age Range**	**1990**	**2000**	**2010**	**2020**	**2023**
Gabon	All age	18.1 (6.3–37.3)	18.1 (6.0–37.4)	18.3 (6.3–37.1)	18.6 (6.7–39.5)	18.6 (6.8–39.1)
Eastern sub-Saharan Africa	20 to 24	21.9 (10.1–38.6)	22.4 (11.3–37.5)	23.6 (15.2–35.0)	23.7 (16.6–35.3)	23.6 (15.7–35.7)
Eastern sub-Saharan Africa	25 to 29	21.8 (9.3–39.7)	21.7 (9.9–38.2)	22.9 (12.7–37.0)	24.0 (13.3–39.7)	24.1 (12.5–40.5)
Eastern sub-Saharan Africa	30 to 34	22.9 (9.6–41.6)	22.6 (9.6–40.8)	23.0 (11.5–39.0)	24.2 (13.2–40.8)	24.4 (12.9–41.3)
Eastern sub-Saharan Africa	35 to 39	23.2 (9.3–42.3)	22.5 (9.3–41.5)	22.3 (10.2–39.1)	22.9 (11.3–40.9)	23.1 (11.2–41.3)
Eastern sub-Saharan Africa	40 to 44	23.6 (9.3–43.5)	23.2 (9.5–42.6)	22.8 (10.2–40.4)	23.1 (11.4–41.0)	23.2 (11.4–40.8)
Eastern sub-Saharan Africa	45 to 49	23.3 (9.2–43.7)	23.0 (9.2–42.8)	22.9 (9.7–41.6)	23.2 (10.0–44.3)	23.4 (10.3–44.5)
Eastern sub-Saharan Africa	50 to 54	23.1 (8.9–44.1)	22.8 (8.9–43.5)	22.9 (9.7–41.7)	23.0 (10.2–44.0)	23.1 (10.3–44.2)
Eastern sub-Saharan Africa	55 to 59	22.4 (8.5–43.5)	22.1 (8.2–42.9)	22.0 (9.2–40.7)	22.3 (9.8–42.4)	22.3 (10.1–42.6)
Eastern sub-Saharan Africa	60 to 64	22.0 (8.0–43.5)	21.5 (7.5–42.5)	21.6 (8.1–41.2)	21.9 (8.6–43.1)	21.9 (8.8–43.1)
Eastern sub-Saharan Africa	65 to 69	20.6 (7.3–40.8)	20.3 (7.1–40.8)	20.4 (7.5–39.6)	20.7 (8.1–41.6)	20.7 (8.3–41.3)
Eastern sub-Saharan Africa	70 to 74	16.8 (5.8–35.8)	16.7 (5.3–34.9)	16.6 (5.7–34.0)	17.0 (6.0–36.8)	17.1 (6.1–37.0)
Eastern sub-Saharan Africa	75 to 79	15.0 (5.0–32.1)	15.1 (4.7–32.1)	15.1 (5.1–31.5)	15.4 (5.2–34.1)	15.4 (5.4–34.0)
Eastern sub-Saharan Africa	80 to 84	10.7 (3.4–24.1)	10.8 (3.3–24.2)	10.9 (3.5–23.8)	11.0 (3.7–25.8)	11.1 (3.8–25.9)
Eastern sub-Saharan Africa	85 to 89	9.7 (3.0–22.0)	9.5 (2.9–21.7)	9.8 (3.1–21.7)	10.0 (3.3–23.6)	10.0 (3.3–23.4)

Supplementary Table S10: Prevalence of female SVAC by age and location for 1990, 2000, 2010, 2020, and 2023						
**Location**	**Age Range**	**1990**	**2000**	**2010**	**2020**	**2023**
Eastern sub-Saharan Africa	90 to 94	8.7 (2.7–19.8)	8.7 (2.6–20.0)	8.8 (2.8–19.7)	9.0 (2.9–21.6)	9.0 (3.0–21.5)
Eastern sub-Saharan Africa	95 plus	8.7 (2.7–19.9)	8.8 (2.6–20.2)	8.7 (2.7–19.4)	8.8 (2.9–21.1)	8.8 (2.9–21.3)
Eastern sub-Saharan Africa	Age-standardized	21.8 (8.7–40.1)	21.5 (8.9–40.0)	21.9 (10.4–37.9)	22.3 (11.3–39.7)	22.4 (11.3–40.0)
Eastern sub-Saharan Africa	All age	22.2 (9.2–40.4)	22.1 (9.6–39.9)	22.6 (11.6–37.9)	23.1 (12.8–39.6)	23.1 (12.3–39.8)
Burundi	20 to 24	20.4 (7.2–41.1)	20.4 (6.9–41.2)	20.8 (7.3–41.0)	21.0 (7.8–43.3)	20.9 (7.9–42.9)
Burundi	25 to 29	20.6 (7.3–41.4)	20.6 (7.0–41.4)	20.9 (7.4–41.3)	21.2 (7.9–43.6)	21.1 (8.0–43.2)
Burundi	30 to 34	21.8 (7.8–43.3)	21.8 (7.5–43.3)	22.1 (7.9–43.1)	22.4 (8.4–45.5)	22.3 (8.5–45.0)
Burundi	35 to 39	21.7 (7.8–43.2)	21.7 (7.5–43.2)	22.1 (7.9–43.0)	22.3 (8.4–45.3)	22.3 (8.5–44.9)
Burundi	40 to 44	22.2 (8.0–43.9)	22.2 (7.7–43.9)	22.6 (8.1–43.7)	22.8 (8.6–46.1)	22.7 (8.7–45.6)
Burundi	45 to 49	21.6 (7.8–43.1)	21.7 (7.5–43.1)	22.0 (7.9–42.9)	22.2 (8.3–45.2)	22.2 (8.5–44.8)
Burundi	50 to 54	21.2 (7.6–42.5)	21.3 (7.3–42.5)	21.6 (7.7–42.3)	21.8 (8.1–44.6)	21.8 (8.3–44.1)
Burundi	55 to 59	20.6 (7.3–41.5)	20.6 (7.0–41.5)	21.0 (7.4–41.3)	21.1 (7.8–43.6)	21.1 (8.0–43.2)
Burundi	60 to 64	20.0 (7.1–40.5)	20.0 (6.8–40.5)	20.3 (7.2–40.4)	20.5 (7.6–42.6)	20.5 (7.7–42.2)
Burundi	65 to 69	18.7 (6.5–38.5)	18.8 (6.3–38.5)	19.0 (6.6–38.3)	19.2 (7.0–40.5)	19.2 (7.1–40.1)
Burundi	70 to 74	15.2 (5.1–32.5)	15.2 (4.9–32.5)	15.5 (5.2–32.3)	15.6 (5.5–34.4)	15.6 (5.5–34.0)

Supplementary Table S10: Prevalence of female SVAC by age and location for 1990, 2000, 2010, 2020, and 2023						
**Location**	**Age Range**	**1990**	**2000**	**2010**	**2020**	**2023**
Burundi	75 to 79	13.8 (4.5–29.9)	13.8 (4.4–29.9)	14.0 (4.6–29.8)	14.2 (4.9–31.7)	14.1 (4.9–31.2)
Burundi	80 to 84	9.9 (3.1–22.5)	9.9 (3.0–22.5)	10.1 (3.2–22.3)	10.2 (3.4–24.0)	10.2 (3.4–23.7)
Burundi	85 to 89	9.0 (2.8–20.5)	9.0 (2.7–20.5)	9.1 (2.8–20.4)	9.2 (3.0–22.0)	9.2 (3.1–21.7)
Burundi	90 to 94	8.1 (2.5–18.8)	8.1 (2.4–18.8)	8.3 (2.5–18.7)	8.4 (2.7–20.1)	8.4 (2.7–19.9)
Burundi	95 plus	8.1 (2.5–18.7)	8.1 (2.4–18.7)	8.2 (2.5–18.5)	8.3 (2.7–20.0)	8.3 (2.7–19.7)
Burundi	Age-standardized	20.3 (7.2–40.8)	20.3 (6.9–40.8)	20.6 (7.3–40.7)	20.8 (7.7–42.9)	20.8 (7.8–42.5)
Burundi	All age	20.7 (7.4–41.5)	20.8 (7.1–41.7)	21.1 (7.5–41.5)	21.4 (8.0–43.9)	21.4 (8.1–43.5)
Comoros	20 to 24	13.3 (4.4–29.0)	13.3 (4.2–28.9)	13.2 (4.3–28.2)	12.9 (4.4–29.4)	12.7 (4.4–28.7)
Comoros	25 to 29	13.9 (4.6–30.0)	13.8 (4.4–30.0)	13.8 (4.5–29.3)	13.5 (4.6–30.5)	13.3 (4.6–29.8)
Comoros	30 to 34	15.1 (5.1–32.3)	15.1 (4.8–32.2)	15.1 (5.0–31.6)	14.7 (5.1–32.8)	14.6 (5.1–32.0)
Comoros	35 to 39	15.4 (5.2–32.8)	15.3 (4.9–32.7)	15.3 (5.1–32.0)	15.0 (5.2–33.3)	14.8 (5.2–32.6)
Comoros	40 to 44	16.0 (5.4–33.8)	15.9 (5.1–33.7)	15.9 (5.3–33.1)	15.6 (5.5–34.4)	15.4 (5.5–33.7)
Comoros	45 to 49	15.6 (5.3–33.3)	15.6 (5.0–33.2)	15.6 (5.2–32.5)	15.3 (5.3–33.8)	15.1 (5.3–33.1)
Comoros	50 to 54	15.4 (5.2–32.8)	15.3 (4.9–32.7)	15.3 (5.1–32.0)	15.0 (5.2–33.3)	14.8 (5.2–32.6)
Comoros	55 to 59	14.9 (5.0–32.0)	14.9 (4.8–31.9)	14.9 (4.9–31.3)	14.6 (5.0–32.5)	14.4 (5.0–31.8)

Supplementary Table S10: Prevalence of female SVAC by age and location for 1990, 2000, 2010, 2020, and 2023						
**Location**	**Age Range**	**1990**	**2000**	**2010**	**2020**	**2023**
Comoros	60 to 64	14.5 (4.8–31.2)	14.4 (4.6–31.1)	14.4 (4.8–30.5)	14.1 (4.9–31.7)	14.0 (4.9–30.9)
Comoros	65 to 69	13.5 (4.4–29.4)	13.5 (4.2–29.3)	13.4 (4.4–28.7)	13.2 (4.5–29.9)	13.0 (4.5–29.2)
Comoros	70 to 74	10.8 (3.4–24.3)	10.8 (3.3–24.2)	10.8 (3.4–23.7)	10.5 (3.5–24.7)	10.4 (3.5–24.1)
Comoros	75 to 79	9.8 (3.1–22.1)	9.7 (2.9–22.1)	9.7 (3.0–21.6)	9.5 (3.1–22.6)	9.4 (3.1–22.0)
Comoros	80 to 84	6.9 (2.1–16.2)	6.9 (2.0–16.1)	6.9 (2.1–15.7)	6.7 (2.1–16.5)	6.6 (2.1–16.0)
Comoros	85 to 89	6.2 (1.9–14.7)	6.2 (1.8–14.6)	6.2 (1.9–14.3)	6.0 (1.9–15.0)	6.0 (1.9–14.6)
Comoros	90 to 94	5.6 (1.7–13.4)	5.6 (1.6–13.3)	5.6 (1.7–13.0)	5.5 (1.7–13.6)	5.4 (1.7–13.3)
Comoros	95 plus	5.6 (1.7–13.2)	5.5 (1.6–13.2)	5.5 (1.7–12.9)	5.4 (1.7–13.5)	5.3 (1.7–13.2)
Comoros	Age-standardized	14.2 (4.7–30.6)	14.2 (4.5–30.5)	14.2 (4.7–29.9)	13.9 (4.8–31.1)	13.7 (4.8–30.4)
Comoros	All age	14.4 (4.8–31.0)	14.3 (4.6–30.8)	14.3 (4.7–30.2)	14.0 (4.8–31.4)	13.8 (4.8–30.7)
Djibouti	20 to 24	20.4 (7.2–41.1)	20.4 (6.9–41.2)	20.8 (7.3–41.0)	21.0 (7.8–43.3)	20.9 (7.9–42.9)
Djibouti	25 to 29	20.6 (7.3–41.4)	20.6 (7.0–41.4)	20.9 (7.4–41.3)	21.2 (7.9–43.6)	21.1 (8.0–43.2)
Djibouti	30 to 34	21.8 (7.8–43.3)	21.8 (7.5–43.3)	22.1 (7.9–43.1)	22.4 (8.4–45.5)	22.3 (8.5–45.0)
Djibouti	35 to 39	21.7 (7.8–43.2)	21.7 (7.5–43.2)	22.1 (7.9–43.0)	22.3 (8.4–45.3)	22.3 (8.5–44.9)
Djibouti	40 to 44	22.2 (8.0–43.9)	22.2 (7.7–43.9)	22.6 (8.1–43.7)	22.8 (8.6–46.1)	22.7 (8.7–45.6)

Supplementary Table S10: Prevalence of female SVAC by age and location for 1990, 2000, 2010, 2020, and 2023						
**Location**	**Age Range**	**1990**	**2000**	**2010**	**2020**	**2023**
Djibouti	45 to 49	21.6 (7.8–43.1)	21.7 (7.5–43.1)	22.0 (7.9–42.9)	22.2 (8.3–45.2)	22.2 (8.5–44.8)
Djibouti	50 to 54	21.2 (7.6–42.5)	21.3 (7.3–42.5)	21.6 (7.7–42.3)	21.8 (8.1–44.6)	21.8 (8.3–44.1)
Djibouti	55 to 59	20.6 (7.3–41.5)	20.6 (7.0–41.5)	21.0 (7.4–41.3)	21.1 (7.8–43.6)	21.1 (8.0–43.2)
Djibouti	60 to 64	20.0 (7.1–40.5)	20.0 (6.8–40.5)	20.3 (7.2–40.4)	20.5 (7.6–42.6)	20.5 (7.7–42.2)
Djibouti	65 to 69	18.7 (6.5–38.5)	18.8 (6.3–38.5)	19.0 (6.6–38.3)	19.2 (7.0–40.5)	19.2 (7.1–40.1)
Djibouti	70 to 74	15.2 (5.1–32.5)	15.2 (4.9–32.5)	15.5 (5.2–32.3)	15.6 (5.5–34.4)	15.6 (5.5–34.0)
Djibouti	75 to 79	13.8 (4.5–29.9)	13.8 (4.4–29.9)	14.0 (4.6–29.8)	14.2 (4.9–31.7)	14.1 (4.9–31.2)
Djibouti	80 to 84	9.9 (3.1–22.5)	9.9 (3.0–22.5)	10.1 (3.2–22.3)	10.2 (3.4–24.0)	10.2 (3.4–23.7)
Djibouti	85 to 89	9.0 (2.8–20.5)	9.0 (2.7–20.5)	9.1 (2.8–20.4)	9.2 (3.0–22.0)	9.2 (3.1–21.7)
Djibouti	90 to 94	8.1 (2.5–18.8)	8.1 (2.4–18.8)	8.3 (2.5–18.7)	8.4 (2.7–20.1)	8.4 (2.7–19.9)
Djibouti	95 plus	8.1 (2.5–18.7)	8.1 (2.4–18.7)	8.2 (2.5–18.5)	8.3 (2.7–20.0)	8.3 (2.7–19.7)
Djibouti	Age-standardized	20.3 (7.2–40.8)	20.3 (6.9–40.8)	20.6 (7.3–40.7)	20.8 (7.7–42.9)	20.8 (7.8–42.5)
Djibouti	All age	20.9 (7.5–41.9)	20.9 (7.1–41.9)	21.3 (7.6–41.7)	21.5 (8.0–44.1)	21.4 (8.1–43.6)
Eritrea	20 to 24	20.4 (7.2–41.1)	20.4 (6.9–41.2)	20.8 (7.3–41.0)	21.0 (7.8–43.3)	20.9 (7.9–42.9)
Eritrea	25 to 29	20.6 (7.3–41.4)	20.6 (7.0–41.4)	20.9 (7.4–41.3)	21.2 (7.9–43.6)	21.1 (8.0–43.2)

Supplementary Table S10: Prevalence of female SVAC by age and location for 1990, 2000, 2010, 2020, and 2023						
**Location**	**Age Range**	**1990**	**2000**	**2010**	**2020**	**2023**
Eritrea	30 to 34	21.8 (7.8–43.3)	21.8 (7.5–43.3)	22.1 (7.9–43.1)	22.4 (8.4–45.5)	22.3 (8.5–45.0)
Eritrea	35 to 39	21.7 (7.8–43.2)	21.7 (7.5–43.2)	22.1 (7.9–43.0)	22.3 (8.4–45.3)	22.3 (8.5–44.9)
Eritrea	40 to 44	22.2 (8.0–43.9)	22.2 (7.7–43.9)	22.6 (8.1–43.7)	22.8 (8.6–46.1)	22.7 (8.7–45.6)
Eritrea	45 to 49	21.6 (7.8–43.1)	21.7 (7.5–43.1)	22.0 (7.9–42.9)	22.2 (8.3–45.2)	22.2 (8.5–44.8)
Eritrea	50 to 54	21.2 (7.6–42.5)	21.3 (7.3–42.5)	21.6 (7.7–42.3)	21.8 (8.1–44.6)	21.8 (8.3–44.1)
Eritrea	55 to 59	20.6 (7.3–41.5)	20.6 (7.0–41.5)	21.0 (7.4–41.3)	21.1 (7.8–43.6)	21.1 (8.0–43.2)
Eritrea	60 to 64	20.0 (7.1–40.5)	20.0 (6.8–40.5)	20.3 (7.2–40.4)	20.5 (7.6–42.6)	20.5 (7.7–42.2)
Eritrea	65 to 69	18.7 (6.5–38.5)	18.8 (6.3–38.5)	19.0 (6.6–38.3)	19.2 (7.0–40.5)	19.2 (7.1–40.1)
Eritrea	70 to 74	15.2 (5.1–32.5)	15.2 (4.9–32.5)	15.5 (5.2–32.3)	15.6 (5.5–34.4)	15.6 (5.5–34.0)
Eritrea	75 to 79	13.8 (4.5–29.9)	13.8 (4.4–29.9)	14.0 (4.6–29.8)	14.2 (4.9–31.7)	14.1 (4.9–31.2)
Eritrea	80 to 84	9.9 (3.1–22.5)	9.9 (3.0–22.5)	10.1 (3.2–22.3)	10.2 (3.4–24.0)	10.2 (3.4–23.7)
Eritrea	85 to 89	9.0 (2.8–20.5)	9.0 (2.7–20.5)	9.1 (2.8–20.4)	9.2 (3.0–22.0)	9.2 (3.1–21.7)
Eritrea	90 to 94	8.1 (2.5–18.8)	8.1 (2.4–18.8)	8.3 (2.5–18.7)	8.4 (2.7–20.1)	8.4 (2.7–19.9)
Eritrea	95 plus	8.1 (2.5–18.7)	8.1 (2.4–18.7)	8.2 (2.5–18.5)	8.3 (2.7–20.0)	8.3 (2.7–19.7)
Eritrea	Age-standardized	20.3 (7.2–40.8)	20.3 (6.9–40.8)	20.6 (7.3–40.7)	20.8 (7.7–42.9)	20.8 (7.8–42.5)

Supplementary Table S10: Prevalence of female SVAC by age and location for 1990, 2000, 2010, 2020, and 2023						
**Location**	**Age Range**	**1990**	**2000**	**2010**	**2020**	**2023**
Eritrea	All age	20.9 (7.5–41.9)	20.9 (7.1–41.8)	21.2 (7.5–41.6)	21.4 (8.0–43.9)	21.3 (8.1–43.4)
Ethiopia	20 to 24	24.9 (7.3–52.1)	24.3 (8.2–48.1)	23.7 (8.7–45.4)	23.8 (8.3–48.9)	23.9 (8.0–49.1)
Ethiopia	25 to 29	25.2 (7.3–52.5)	24.7 (8.3–48.4)	24.1 (8.8–46.0)	24.2 (8.5–50.3)	24.3 (8.1–50.4)
Ethiopia	30 to 34	26.8 (7.9–54.5)	26.3 (8.9–50.7)	25.6 (9.6–48.1)	25.7 (9.0–52.2)	25.8 (8.5–52.2)
Ethiopia	35 to 39	26.9 (7.8–54.7)	26.4 (8.8–50.9)	25.7 (9.6–48.3)	25.8 (9.1–52.3)	25.9 (8.7–52.8)
Ethiopia	40 to 44	27.6 (8.2–56.1)	27.1 (9.1–52.2)	26.4 (9.9–49.2)	26.5 (9.3–52.8)	26.6 (9.0–53.4)
Ethiopia	45 to 49	27.0 (7.9–54.7)	26.5 (8.8–51.0)	25.8 (9.6–48.4)	25.9 (9.1–52.4)	26.0 (8.8–52.6)
Ethiopia	50 to 54	26.5 (8.0–54.2)	25.9 (8.9–50.3)	25.3 (9.4–47.7)	25.4 (9.0–51.7)	25.5 (8.6–51.9)
Ethiopia	55 to 59	25.8 (7.7–53.6)	25.3 (8.6–49.4)	24.6 (9.1–46.7)	24.7 (8.7–50.2)	24.8 (8.3–50.6)
Ethiopia	60 to 64	25.1 (7.4–52.7)	24.6 (8.2–48.4)	23.9 (8.8–45.8)	24.0 (8.4–49.8)	24.1 (8.1–50.1)
Ethiopia	65 to 69	23.6 (6.6–50.2)	23.1 (7.6–46.6)	22.5 (8.1–43.6)	22.6 (7.8–47.9)	22.7 (7.5–47.8)
Ethiopia	70 to 74	19.4 (5.2–44.1)	18.9 (5.9–39.8)	18.5 (6.4–37.3)	18.5 (6.1–41.5)	18.6 (5.8–42.8)
Ethiopia	75 to 79	17.6 (4.6–40.8)	17.2 (5.3–37.0)	16.8 (5.7–34.6)	16.8 (5.3–38.6)	16.9 (5.1–40.5)
Ethiopia	80 to 84	12.8 (3.3–31.1)	12.5 (3.7–28.2)	12.2 (3.9–26.4)	12.2 (3.9–29.7)	12.3 (3.6–30.0)
Ethiopia	85 to 89	11.7 (2.9–28.2)	11.4 (3.3–26.0)	11.1 (3.5–24.2)	11.1 (3.4–27.6)	11.1 (3.2–28.2)

Supplementary Table S10: Prevalence of female SVAC by age and location for 1990, 2000, 2010, 2020, and 2023						
**Location**	**Age Range**	**1990**	**2000**	**2010**	**2020**	**2023**
Ethiopia	90 to 94	10.6 (2.7–25.6)	10.3 (2.9–23.0)	10.1 (3.2–22.3)	10.1 (3.0–25.2)	10.1 (2.9–26.4)
Ethiopia	95 plus	10.5 (2.5–25.6)	10.2 (2.9–23.5)	10.0 (3.1–22.1)	10.0 (3.0–25.0)	10.0 (2.8–25.9)
Ethiopia	Age-standardized	25.2 (7.4–52.2)	24.7 (8.2–48.3)	24.0 (8.8–45.7)	24.1 (8.4–49.9)	24.2 (8.1–50.2)
Ethiopia	All age	25.8 (7.6–53.1)	25.2 (8.5–49.3)	24.5 (9.1–46.6)	24.7 (8.6–50.8)	24.8 (8.3–51.1)
Kenya	20 to 24	27.4 (14.5–45.5)	27.6 (17.5–39.2)	27.6 (19.3–36.4)	25.4 (16.1–37.3)	25.0 (14.6–39.0)
Kenya	25 to 29	27.5 (11.1–51.1)	27.7 (14.2–44.3)	28.7 (18.1–40.8)	29.5 (16.8–44.7)	29.6 (15.5–46.8)
Kenya	30 to 34	28.7 (11.0–54.1)	28.5 (13.1–48.8)	28.7 (16.9–44.2)	29.6 (19.4–42.3)	29.8 (18.9–44.3)
Kenya	35 to 39	29.0 (10.2–55.4)	28.5 (11.8–50.8)	28.0 (14.1–46.0)	28.1 (14.7–46.7)	28.2 (13.3–48.2)
Kenya	40 to 44	29.8 (10.3–56.5)	29.3 (11.7–53.1)	28.8 (13.4–49.4)	28.3 (15.3–46.3)	28.3 (14.8–45.7)
Kenya	45 to 49	29.5 (9.7–58.1)	29.4 (11.0–54.2)	29.6 (11.5–53.4)	29.8 (12.2–55.7)	29.8 (12.2–55.6)
Kenya	50 to 54	29.0 (9.4–57.4)	28.9 (10.7–53.6)	29.1 (11.3–52.7)	29.2 (11.9–55.0)	29.2 (11.9–54.9)
Kenya	55 to 59	28.3 (9.1–56.4)	28.1 (10.4–52.5)	28.3 (10.9–51.8)	28.5 (11.5–54.0)	28.5 (11.5–53.8)
Kenya	60 to 64	27.6 (8.8–54.6)	27.4 (10.0–51.5)	27.6 (10.5–50.8)	27.8 (11.1–53.1)	27.7 (11.1–52.9)
Kenya	65 to 69	26.0 (8.1–53.7)	25.8 (9.3–49.4)	26.0 (9.7–48.7)	26.2 (10.3–50.9)	26.2 (10.3–50.9)
Kenya	70 to 74	21.6 (6.4–46.8)	21.4 (7.3–42.9)	21.5 (7.7–42.2)	21.7 (8.1–44.4)	21.7 (8.1–44.1)

Supplementary Table S10: Prevalence of female SVAC by age and location for 1990, 2000, 2010, 2020, and 2023						
**Location**	**Age Range**	**1990**	**2000**	**2010**	**2020**	**2023**
Kenya	75 to 79	19.8 (5.7–44.1)	19.5 (6.5–40.0)	19.7 (6.9–39.3)	19.8 (7.2–41.5)	19.8 (7.3–41.4)
Kenya	80 to 84	14.6 (3.9–34.7)	14.4 (4.5–31.1)	14.5 (4.8–30.5)	14.6 (5.0–32.5)	14.6 (5.1–32.3)
Kenya	85 to 89	13.3 (3.5–32.1)	13.1 (4.0–28.7)	13.1 (4.3–28.1)	13.2 (4.5–30.0)	13.3 (4.5–29.8)
Kenya	90 to 94	12.1 (3.2–29.8)	11.9 (3.6–26.5)	12.0 (3.8–26.0)	12.1 (4.1–27.8)	12.1 (4.1–27.5)
Kenya	95 plus	12.1 (3.1–29.5)	11.8 (3.6–26.3)	11.9 (3.8–25.8)	12.0 (4.0–27.6)	12.0 (4.1–27.3)
Kenya	Age-standardized	27.5 (10.9–51.0)	27.3 (12.2–47.6)	27.4 (14.6–44.4)	27.4 (14.8–45.5)	27.4 (13.9–45.9)
Kenya	All age	27.9 (11.8–50.5)	27.7 (13.5–45.9)	28.0 (16.7–42.7)	27.8 (16.0–44.1)	27.8 (14.7–45.3)
Madagascar	20 to 24	20.4 (7.2–41.1)	20.4 (6.9–41.2)	20.8 (7.3–41.0)	21.0 (7.8–43.3)	20.9 (7.9–42.9)
Madagascar	25 to 29	20.6 (7.3–41.4)	20.6 (7.0–41.4)	20.9 (7.4–41.3)	21.2 (7.9–43.6)	21.1 (8.0–43.2)
Madagascar	30 to 34	21.8 (7.8–43.3)	21.8 (7.5–43.3)	22.1 (7.9–43.1)	22.4 (8.4–45.5)	22.3 (8.5–45.0)
Madagascar	35 to 39	21.7 (7.8–43.2)	21.7 (7.5–43.2)	22.1 (7.9–43.0)	22.3 (8.4–45.3)	22.3 (8.5–44.9)
Madagascar	40 to 44	22.2 (8.0–43.9)	22.2 (7.7–43.9)	22.6 (8.1–43.7)	22.8 (8.6–46.1)	22.7 (8.7–45.6)
Madagascar	45 to 49	21.6 (7.8–43.1)	21.7 (7.5–43.1)	22.0 (7.9–42.9)	22.2 (8.3–45.2)	22.2 (8.5–44.8)
Madagascar	50 to 54	21.2 (7.6–42.5)	21.3 (7.3–42.5)	21.6 (7.7–42.3)	21.8 (8.1–44.6)	21.8 (8.3–44.1)
Madagascar	55 to 59	20.6 (7.3–41.5)	20.6 (7.0–41.5)	21.0 (7.4–41.3)	21.1 (7.8–43.6)	21.1 (8.0–43.2)

Supplementary Table S10: Prevalence of female SVAC by age and location for 1990, 2000, 2010, 2020, and 2023						
**Location**	**Age Range**	**1990**	**2000**	**2010**	**2020**	**2023**
Madagascar	60 to 64	20.0 (7.1–40.5)	20.0 (6.8–40.5)	20.3 (7.2–40.4)	20.5 (7.6–42.6)	20.5 (7.7–42.2)
Madagascar	65 to 69	18.7 (6.5–38.5)	18.8 (6.3–38.5)	19.0 (6.6–38.3)	19.2 (7.0–40.5)	19.2 (7.1–40.1)
Madagascar	70 to 74	15.2 (5.1–32.5)	15.2 (4.9–32.5)	15.5 (5.2–32.3)	15.6 (5.5–34.4)	15.6 (5.5–34.0)
Madagascar	75 to 79	13.8 (4.5–29.9)	13.8 (4.4–29.9)	14.0 (4.6–29.8)	14.2 (4.9–31.7)	14.1 (4.9–31.2)
Madagascar	80 to 84	9.9 (3.1–22.5)	9.9 (3.0–22.5)	10.1 (3.2–22.3)	10.2 (3.4–24.0)	10.2 (3.4–23.7)
Madagascar	85 to 89	9.0 (2.8–20.5)	9.0 (2.7–20.5)	9.1 (2.8–20.4)	9.2 (3.0–22.0)	9.2 (3.1–21.7)
Madagascar	90 to 94	8.1 (2.5–18.8)	8.1 (2.4–18.8)	8.3 (2.5–18.7)	8.4 (2.7–20.1)	8.4 (2.7–19.9)
Madagascar	95 plus	8.1 (2.5–18.7)	8.1 (2.4–18.7)	8.2 (2.5–18.5)	8.3 (2.7–20.0)	8.3 (2.7–19.7)
Madagascar	Age-standardized	20.3 (7.2–40.8)	20.3 (6.9–40.8)	20.6 (7.3–40.7)	20.8 (7.7–42.9)	20.8 (7.8–42.5)
Madagascar	All age	20.7 (7.4–41.6)	20.8 (7.1–41.7)	21.2 (7.5–41.6)	21.4 (8.0–43.9)	21.3 (8.1–43.4)
Malawi	20 to 24	27.6 (11.1–50.5)	28.1 (13.8–46.8)	28.6 (20.1–38.5)	28.6 (17.1–42.1)	28.5 (15.8–45.5)
Malawi	25 to 29	27.6 (10.8–50.7)	28.0 (12.0–49.4)	28.5 (16.0–44.8)	28.6 (19.3–39.5)	28.6 (18.0–42.3)
Malawi	30 to 34	28.7 (11.2–53.0)	28.7 (10.7–53.0)	29.0 (11.2–52.7)	29.3 (11.9–55.1)	29.3 (12.1–54.7)
Malawi	35 to 39	28.6 (11.2–53.0)	28.7 (10.7–52.9)	29.0 (11.2–52.7)	29.3 (11.9–55.1)	29.3 (12.1–54.7)
Malawi	40 to 44	29.2 (11.5–53.7)	29.3 (11.0–53.7)	29.6 (11.6–53.5)	30.0 (12.3–55.9)	29.9 (12.4–55.5)

Supplementary Table S10: Prevalence of female SVAC by age and location for 1990, 2000, 2010, 2020, and 2023						
**Location**	**Age Range**	**1990**	**2000**	**2010**	**2020**	**2023**
Malawi	45 to 49	28.6 (11.1–52.9)	28.6 (10.7–52.8)	28.9 (11.2–52.6)	29.3 (11.9–55.0)	29.2 (12.0–54.6)
Malawi	50 to 54	28.0 (10.8–52.2)	28.0 (10.4–52.1)	28.4 (10.9–51.9)	28.7 (11.6–54.3)	28.7 (11.7–53.9)
Malawi	55 to 59	27.3 (10.5–51.1)	27.3 (10.0–51.1)	27.6 (10.5–50.8)	27.9 (11.2–53.3)	27.9 (11.3–52.9)
Malawi	60 to 64	26.5 (10.1–50.1)	26.6 (9.7–50.1)	26.9 (10.2–49.9)	27.2 (10.8–52.3)	27.2 (10.9–51.8)
Malawi	65 to 69	25.0 (9.3–48.0)	25.0 (8.9–48.0)	25.3 (9.4–47.7)	25.6 (10.0–50.1)	25.6 (10.1–49.7)
Malawi	70 to 74	20.6 (7.3–41.5)	20.6 (7.0–41.5)	20.9 (7.4–41.2)	21.2 (7.9–43.6)	21.1 (8.0–43.2)
Malawi	75 to 79	18.8 (6.6–38.6)	18.8 (6.3–38.6)	19.1 (6.6–38.4)	19.3 (7.0–40.7)	19.3 (7.1–40.3)
Malawi	80 to 84	13.8 (4.6–30.0)	13.8 (4.4–29.9)	14.0 (4.6–29.7)	14.2 (4.9–31.8)	14.2 (5.0–31.3)
Malawi	85 to 89	12.6 (4.1–27.6)	12.6 (3.9–27.6)	12.7 (4.1–27.4)	12.9 (4.4–29.4)	12.9 (4.4–29.0)
Malawi	90 to 94	11.4 (3.7–25.5)	11.4 (3.5–25.4)	11.6 (3.7–25.2)	11.8 (3.9–27.1)	11.7 (4.0–26.7)
Malawi	95 plus	11.3 (3.6–25.3)	11.3 (3.5–25.3)	11.5 (3.7–25.1)	11.7 (3.9–26.9)	11.7 (4.0–26.6)
Malawi	Age-standardized	27.0 (10.7–49.7)	27.1 (11.3–49.4)	27.5 (12.7–47.4)	27.7 (14.3–47.6)	27.7 (14.0–47.0)
Malawi	All age	27.6 (11.3–50.6)	27.8 (12.1–48.7)	28.2 (14.4–46.1)	28.4 (15.9–45.8)	28.4 (16.0–46.0)
Mozambique	20 to 24	13.5 (6.4–24.9)	13.7 (7.9–21.4)	14.9 (8.4–23.3)	15.8 (9.8–24.3)	15.9 (9.3–25.8)
Mozambique	25 to 29	14.1 (5.3–28.7)	13.6 (6.6–23.6)	13.6 (7.0–22.3)	14.4 (5.7–28.2)	14.5 (5.1–29.7)

Supplementary Table S10: Prevalence of female SVAC by age and location for 1990, 2000, 2010, 2020, and 2023						
**Location**	**Age Range**	**1990**	**2000**	**2010**	**2020**	**2023**
Mozambique	30 to 34	15.1 (5.0–32.6)	14.4 (6.0–27.2)	13.8 (7.4–22.5)	14.5 (6.7–26.5)	14.7 (5.8–27.9)
Mozambique	35 to 39	15.6 (4.8–34.9)	14.9 (5.7–29.7)	14.1 (6.8–24.6)	14.1 (7.1–23.9)	14.2 (6.6–25.6)
Mozambique	40 to 44	16.2 (4.9–36.8)	15.6 (5.4–31.6)	14.8 (6.4–27.8)	14.1 (7.7–23.4)	14.0 (7.6–23.0)
Mozambique	45 to 49	16.3 (4.5–37.6)	16.0 (5.1–34.1)	16.0 (5.4–33.3)	16.1 (5.7–35.3)	16.1 (5.7–35.0)
Mozambique	50 to 54	16.0 (4.4–37.4)	15.7 (5.0–33.5)	15.7 (5.2–32.7)	15.8 (5.5–34.7)	15.8 (5.6–34.5)
Mozambique	55 to 59	15.5 (4.2–35.9)	15.2 (4.8–32.7)	15.2 (5.1–31.9)	15.3 (5.3–33.9)	15.3 (5.4–33.7)
Mozambique	60 to 64	15.0 (4.1–34.9)	14.7 (4.6–31.8)	14.8 (4.9–31.1)	14.9 (5.2–33.0)	14.9 (5.2–33.1)
Mozambique	65 to 69	14.0 (3.7–33.5)	13.7 (4.3–30.0)	13.7 (4.5–29.2)	13.8 (4.8–31.2)	13.9 (4.8–30.9)
Mozambique	70 to 74	11.3 (2.9–27.9)	11.0 (3.3–24.8)	11.0 (3.5–24.1)	11.1 (3.7–25.8)	11.1 (3.7–25.6)
Mozambique	75 to 79	10.2 (2.6–25.6)	9.9 (3.0–22.6)	9.9 (3.1–22.0)	10.0 (3.3–23.6)	10.0 (3.3–23.4)
Mozambique	80 to 84	7.2 (1.8–19.2)	7.0 (2.0–16.6)	7.0 (2.1–16.1)	7.1 (2.3–17.4)	7.1 (2.3–17.2)
Mozambique	85 to 89	6.5 (1.6–17.2)	6.3 (1.8–15.1)	6.3 (1.9–14.6)	6.4 (2.0–15.8)	6.4 (2.0–15.6)
Mozambique	90 to 94	5.9 (1.4–15.7)	5.7 (1.6–13.7)	5.7 (1.7–13.3)	5.8 (1.8–14.4)	5.8 (1.8–14.3)
Mozambique	95 plus	5.8 (1.4–15.7)	5.7 (1.6–13.6)	5.7 (1.7–13.2)	5.7 (1.8–14.2)	5.7 (1.8–14.1)
Mozambique	Age-standardized	14.6 (5.0–30.5)	14.2 (5.6–27.8)	14.1 (6.6–25.6)	14.4 (6.9–27.3)	14.4 (6.5–27.2)

Supplementary Table S10: Prevalence of female SVAC by age and location for 1990, 2000, 2010, 2020, and 2023						
**Location**	**Age Range**	**1990**	**2000**	**2010**	**2020**	**2023**
Mozambique	All age	14.8 (5.3–30.7)	14.3 (6.2–26.7)	14.3 (7.3–24.8)	14.7 (7.5–26.4)	14.8 (6.9–27.5)
Rwanda	20 to 24	24.4 (14.2–36.5)	25.1 (16.0–35.4)	27.4 (21.4–33.8)	33.8 (23.4–45.2)	34.9 (22.5–49.6)
Rwanda	25 to 29	25.9 (15.7–38.6)	26.4 (17.1–37.0)	27.9 (21.6–35.2)	32.3 (24.1–41.1)	33.2 (24.0–44.2)
Rwanda	30 to 34	28.6 (17.3–43.6)	28.4 (18.4–40.6)	27.9 (21.2–35.3)	28.8 (15.9–45.0)	29.1 (14.2–48.7)
Rwanda	35 to 39	32.9 (21.8–48.5)	32.6 (22.8–45.0)	31.8 (24.9–39.4)	31.9 (17.9–48.5)	32.0 (16.2–51.8)
Rwanda	40 to 44	32.5 (21.2–48.1)	32.2 (22.1–44.9)	31.5 (24.5–39.1)	31.8 (17.9–48.6)	31.9 (16.2–51.7)
Rwanda	45 to 49	31.5 (20.2–46.9)	31.2 (21.5–43.9)	30.6 (23.8–38.1)	30.9 (17.3–47.5)	31.1 (15.6–50.8)
Rwanda	50 to 54	33.1 (21.8–48.5)	32.8 (22.8–45.3)	32.0 (25.0–39.7)	31.9 (18.0–48.7)	31.9 (16.1–51.7)
Rwanda	55 to 59	26.8 (16.2–41.7)	26.6 (17.3–38.6)	26.2 (19.7–33.4)	27.2 (14.8–43.1)	27.6 (13.3–46.7)
Rwanda	60 to 64	29.9 (9.3–57.8)	29.7 (10.4–55.7)	29.4 (11.4–53.1)	29.6 (12.1–55.5)	30.0 (11.8–56.4)
Rwanda	65 to 69	28.2 (8.6–55.8)	28.1 (9.5–53.9)	27.7 (10.6–51.0)	28.0 (11.2–53.4)	28.3 (11.0–54.2)
Rwanda	70 to 74	23.5 (6.8–49.3)	23.3 (7.5–47.4)	23.0 (8.3–44.5)	23.3 (8.8–46.8)	23.5 (8.8–48.3)
Rwanda	75 to 79	21.6 (6.2–47.4)	21.4 (6.8–45.1)	21.1 (7.5–41.5)	21.3 (7.9–43.9)	21.5 (7.8–45.4)
Rwanda	80 to 84	15.9 (4.2–37.2)	15.8 (4.7–35.3)	15.6 (5.2–32.5)	15.8 (5.5–34.7)	15.9 (5.5–35.4)
Rwanda	85 to 89	14.5 (3.8–34.9)	14.3 (4.2–32.7)	14.2 (4.7–30.1)	14.4 (5.0–32.1)	14.5 (5.0–33.1)

Supplementary Table S10: Prevalence of female SVAC by age and location for 1990, 2000, 2010, 2020, and 2023						
**Location**	**Age Range**	**1990**	**2000**	**2010**	**2020**	**2023**
Rwanda	90 to 94	13.2 (3.4–32.4)	13.1 (3.8–30.0)	13.0 (4.2–27.8)	13.1 (4.5–29.8)	13.2 (4.5–30.7)
Rwanda	95 plus	13.1 (3.4–31.5)	13.0 (3.7–29.7)	12.8 (4.2–27.6)	13.0 (4.4–29.6)	13.1 (4.4–30.1)
Rwanda	Age-standardized	28.3 (18.1–42.2)	28.3 (19.3–40.2)	28.4 (22.5–35.0)	30.0 (24.5–37.9)	30.4 (23.9–40.2)
Rwanda	All age	28.2 (18.6–41.4)	28.4 (20.0–39.2)	28.7 (23.6–34.3)	30.8 (24.9–39.5)	31.2 (23.9–42.0)
Somalia	20 to 24	20.4 (7.2–41.1)	20.4 (6.9–41.2)	20.8 (7.3–41.0)	21.0 (7.8–43.3)	20.9 (7.9–42.9)
Somalia	25 to 29	20.6 (7.3–41.4)	20.6 (7.0–41.4)	20.9 (7.4–41.3)	21.2 (7.9–43.6)	21.1 (8.0–43.2)
Somalia	30 to 34	21.8 (7.8–43.3)	21.8 (7.5–43.3)	22.1 (7.9–43.1)	22.4 (8.4–45.5)	22.3 (8.5–45.0)
Somalia	35 to 39	21.7 (7.8–43.2)	21.7 (7.5–43.2)	22.1 (7.9–43.0)	22.3 (8.4–45.3)	22.3 (8.5–44.9)
Somalia	40 to 44	22.2 (8.0–43.9)	22.2 (7.7–43.9)	22.6 (8.1–43.7)	22.8 (8.6–46.1)	22.7 (8.7–45.6)
Somalia	45 to 49	21.6 (7.8–43.1)	21.7 (7.5–43.1)	22.0 (7.9–42.9)	22.2 (8.3–45.2)	22.2 (8.5–44.8)
Somalia	50 to 54	21.2 (7.6–42.5)	21.3 (7.3–42.5)	21.6 (7.7–42.3)	21.8 (8.1–44.6)	21.8 (8.3–44.1)
Somalia	55 to 59	20.6 (7.3–41.5)	20.6 (7.0–41.5)	21.0 (7.4–41.3)	21.1 (7.8–43.6)	21.1 (8.0–43.2)
Somalia	60 to 64	20.0 (7.1–40.5)	20.0 (6.8–40.5)	20.3 (7.2–40.4)	20.5 (7.6–42.6)	20.5 (7.7–42.2)
Somalia	65 to 69	18.7 (6.5–38.5)	18.8 (6.3–38.5)	19.0 (6.6–38.3)	19.2 (7.0–40.5)	19.2 (7.1–40.1)
Somalia	70 to 74	15.2 (5.1–32.5)	15.2 (4.9–32.5)	15.5 (5.2–32.3)	15.6 (5.5–34.4)	15.6 (5.5–34.0)

Supplementary Table S10: Prevalence of female SVAC by age and location for 1990, 2000, 2010, 2020, and 2023						
**Location**	**Age Range**	**1990**	**2000**	**2010**	**2020**	**2023**
Somalia	75 to 79	13.8 (4.5–29.9)	13.8 (4.4–29.9)	14.0 (4.6–29.8)	14.2 (4.9–31.7)	14.1 (4.9–31.2)
Somalia	80 to 84	9.9 (3.1–22.5)	9.9 (3.0–22.5)	10.1 (3.2–22.3)	10.2 (3.4–24.0)	10.2 (3.4–23.7)
Somalia	85 to 89	9.0 (2.8–20.5)	9.0 (2.7–20.5)	9.1 (2.8–20.4)	9.2 (3.0–22.0)	9.2 (3.1–21.7)
Somalia	90 to 94	8.1 (2.5–18.8)	8.1 (2.4–18.8)	8.3 (2.5–18.7)	8.4 (2.7–20.1)	8.4 (2.7–19.9)
Somalia	95 plus	8.1 (2.5–18.7)	8.1 (2.4–18.7)	8.2 (2.5–18.5)	8.3 (2.7–20.0)	8.3 (2.7–19.7)
Somalia	Age-standardized	20.3 (7.2–40.8)	20.3 (6.9–40.8)	20.6 (7.3–40.7)	20.8 (7.7–42.9)	20.8 (7.8–42.5)
Somalia	All age	21.0 (7.5–42.0)	20.9 (7.1–41.9)	21.2 (7.5–41.7)	21.4 (8.0–44.0)	21.4 (8.1–43.5)
South Sudan	20 to 24	20.4 (7.2–41.1)	20.4 (6.9–41.2)	20.8 (7.3–41.0)	21.0 (7.8–43.3)	20.9 (7.9–42.9)
South Sudan	25 to 29	20.6 (7.3–41.4)	20.6 (7.0–41.4)	20.9 (7.4–41.3)	21.2 (7.9–43.6)	21.1 (8.0–43.2)
South Sudan	30 to 34	21.8 (7.8–43.3)	21.8 (7.5–43.3)	22.1 (7.9–43.1)	22.4 (8.4–45.5)	22.3 (8.5–45.0)
South Sudan	35 to 39	21.7 (7.8–43.2)	21.7 (7.5–43.2)	22.1 (7.9–43.0)	22.3 (8.4–45.3)	22.3 (8.5–44.9)
South Sudan	40 to 44	22.2 (8.0–43.9)	22.2 (7.7–43.9)	22.6 (8.1–43.7)	22.8 (8.6–46.1)	22.7 (8.7–45.6)
South Sudan	45 to 49	21.6 (7.8–43.1)	21.7 (7.5–43.1)	22.0 (7.9–42.9)	22.2 (8.3–45.2)	22.2 (8.5–44.8)
South Sudan	50 to 54	21.2 (7.6–42.5)	21.3 (7.3–42.5)	21.6 (7.7–42.3)	21.8 (8.1–44.6)	21.8 (8.3–44.1)
South Sudan	55 to 59	20.6 (7.3–41.5)	20.6 (7.0–41.5)	21.0 (7.4–41.3)	21.1 (7.8–43.6)	21.1 (8.0–43.2)

Supplementary Table S10: Prevalence of female SVAC by age and location for 1990, 2000, 2010, 2020, and 2023						
**Location**	**Age Range**	**1990**	**2000**	**2010**	**2020**	**2023**
South Sudan	60 to 64	20.0 (7.1–40.5)	20.0 (6.8–40.5)	20.3 (7.2–40.4)	20.5 (7.6–42.6)	20.5 (7.7–42.2)
South Sudan	65 to 69	18.7 (6.5–38.5)	18.8 (6.3–38.5)	19.0 (6.6–38.3)	19.2 (7.0–40.5)	19.2 (7.1–40.1)
South Sudan	70 to 74	15.2 (5.1–32.5)	15.2 (4.9–32.5)	15.5 (5.2–32.3)	15.6 (5.5–34.4)	15.6 (5.5–34.0)
South Sudan	75 to 79	13.8 (4.5–29.9)	13.8 (4.4–29.9)	14.0 (4.6–29.8)	14.2 (4.9–31.7)	14.1 (4.9–31.2)
South Sudan	80 to 84	9.9 (3.1–22.5)	9.9 (3.0–22.5)	10.1 (3.2–22.3)	10.2 (3.4–24.0)	10.2 (3.4–23.7)
South Sudan	85 to 89	9.0 (2.8–20.5)	9.0 (2.7–20.5)	9.1 (2.8–20.4)	9.2 (3.0–22.0)	9.2 (3.1–21.7)
South Sudan	90 to 94	8.1 (2.5–18.8)	8.1 (2.4–18.8)	8.3 (2.5–18.7)	8.4 (2.7–20.1)	8.4 (2.7–19.9)
South Sudan	95 plus	8.1 (2.5–18.7)	8.1 (2.4–18.7)	8.2 (2.5–18.5)	8.3 (2.7–20.0)	8.3 (2.7–19.7)
South Sudan	Age-standardized	20.3 (7.2–40.8)	20.3 (6.9–40.8)	20.6 (7.3–40.7)	20.8 (7.7–42.9)	20.8 (7.8–42.5)
South Sudan	All age	20.7 (7.4–41.5)	20.8 (7.1–41.7)	21.2 (7.6–41.7)	21.5 (8.0–44.1)	21.4 (8.1–43.6)
Uganda	20 to 24	16.4 (7.8–28.4)	18.4 (11.2–27.6)	22.1 (14.3–31.2)	23.3 (13.1–36.5)	23.2 (11.8–38.4)
Uganda	25 to 29	16.3 (7.2–30.7)	17.3 (9.9–26.8)	20.3 (12.1–30.1)	22.7 (14.1–34.5)	23.0 (13.5–35.8)
Uganda	30 to 34	16.7 (6.3–32.9)	16.2 (8.1–27.6)	17.2 (9.9–26.6)	18.4 (8.6–32.4)	18.7 (7.4–34.4)
Uganda	35 to 39	15.6 (5.6–31.5)	14.8 (6.9–25.7)	15.8 (8.9–25.0)	17.8 (9.1–29.7)	18.2 (8.5–32.2)
Uganda	40 to 44	16.5 (6.0–32.3)	15.5 (7.0–27.4)	15.7 (8.1–26.4)	17.1 (8.5–29.7)	17.5 (7.8–31.6)

Supplementary Table S10: Prevalence of female SVAC by age and location for 1990, 2000, 2010, 2020, and 2023						
**Location**	**Age Range**	**1990**	**2000**	**2010**	**2020**	**2023**
Uganda	45 to 49	16.1 (6.0–32.6)	15.0 (6.4–28.0)	14.9 (6.8–26.9)	15.5 (7.0–29.4)	15.8 (6.5–30.2)
Uganda	50 to 54	17.4 (6.0–36.3)	17.3 (5.7–36.1)	17.5 (5.9–35.7)	17.7 (6.3–38.0)	17.8 (6.3–38.8)
Uganda	55 to 59	16.8 (5.8–35.4)	16.8 (5.5–35.2)	16.9 (5.7–34.8)	17.2 (6.1–37.1)	17.2 (6.1–37.8)
Uganda	60 to 64	16.3 (5.6–34.5)	16.3 (5.3–34.3)	16.4 (5.5–34.0)	16.7 (5.9–36.2)	16.7 (5.9–36.9)
Uganda	65 to 69	15.2 (5.1–32.6)	15.2 (4.9–32.4)	15.3 (5.1–32.1)	15.5 (5.4–34.3)	15.6 (5.4–35.0)
Uganda	70 to 74	12.3 (4.0–27.2)	12.2 (3.8–26.9)	12.3 (4.0–26.6)	12.5 (4.2–28.6)	12.6 (4.2–29.3)
Uganda	75 to 79	11.1 (3.6–24.8)	11.0 (3.4–24.7)	11.1 (3.5–24.4)	11.3 (3.8–26.3)	11.4 (3.8–26.8)
Uganda	80 to 84	7.9 (2.4–18.3)	7.9 (2.3–18.2)	7.9 (2.4–18.0)	8.1 (2.6–19.5)	8.1 (2.6–20.0)
Uganda	85 to 89	7.1 (2.2–16.6)	7.1 (2.1–16.5)	7.1 (2.2–16.3)	7.3 (2.3–17.7)	7.3 (2.3–18.2)
Uganda	90 to 94	6.4 (2.0–15.2)	6.4 (1.9–15.1)	6.5 (2.0–14.9)	6.6 (2.1–16.2)	6.6 (2.1–16.7)
Uganda	95 plus	6.4 (1.9–15.1)	6.3 (1.8–15.0)	6.4 (1.9–14.8)	6.5 (2.1–16.1)	6.6 (2.1–16.5)
Uganda	Age-standardized	15.8 (6.2–30.9)	15.8 (7.3–28.5)	16.9 (9.0–28.1)	18.0 (9.1–31.9)	18.1 (8.3–33.0)
Uganda	All age	16.1 (6.7–30.4)	16.5 (8.4–27.7)	18.2 (10.6–28.1)	19.4 (10.3–32.4)	19.5 (9.4–34.6)
Tanzania	20 to 24	17.0 (7.0–31.8)	19.5 (11.4–30.5)	25.4 (17.3–34.5)	25.4 (13.2–43.9)	24.7 (10.9–45.6)
Tanzania	25 to 29	14.9 (7.6–25.4)	15.4 (8.9–23.8)	20.1 (12.7–28.8)	23.9 (12.8–38.1)	24.0 (11.1–40.2)

Supplementary Table S10: Prevalence of female SVAC by age and location for 1990, 2000, 2010, 2020, and 2023						
**Location**	**Age Range**	**1990**	**2000**	**2010**	**2020**	**2023**
Tanzania	30 to 34	15.5 (8.6–25.1)	15.4 (8.9–23.7)	18.3 (10.9–27.9)	23.4 (14.5–35.5)	24.2 (14.2–38.8)
Tanzania	35 to 39	15.5 (8.5–25.7)	14.2 (8.1–22.8)	14.2 (7.5–23.1)	15.1 (7.3–27.8)	15.5 (6.9–29.1)
Tanzania	40 to 44	16.0 (7.9–27.5)	14.5 (8.4–22.3)	14.4 (7.2–24.8)	14.8 (7.5–26.4)	15.0 (7.2–26.5)
Tanzania	45 to 49	16.9 (7.5–32.0)	16.3 (8.7–26.7)	17.1 (7.9–29.8)	18.1 (6.6–35.5)	18.2 (6.7–37.8)
Tanzania	50 to 54	17.3 (6.6–34.0)	16.5 (7.6–29.2)	16.7 (8.2–28.4)	17.6 (7.1–33.4)	17.8 (6.5–34.4)
Tanzania	55 to 59	17.1 (6.2–34.8)	16.6 (6.8–31.0)	16.3 (8.3–27.4)	16.8 (7.6–31.0)	17.0 (6.8–32.4)
Tanzania	60 to 64	16.9 (5.9–35.2)	16.6 (6.2–32.5)	16.2 (7.7–28.9)	16.3 (8.0–28.6)	16.4 (7.4–29.9)
Tanzania	65 to 69	15.9 (5.5–33.5)	15.9 (5.6–32.7)	15.9 (6.7–30.2)	15.7 (8.0–27.4)	15.7 (7.9–26.7)
Tanzania	70 to 74	12.8 (4.2–28.1)	12.8 (4.0–28.1)	13.0 (4.2–27.9)	13.1 (4.5–29.8)	13.1 (4.5–29.6)
Tanzania	75 to 79	11.6 (3.7–25.8)	11.6 (3.6–25.7)	11.8 (3.8–25.6)	11.9 (4.0–27.4)	11.9 (4.0–27.4)
Tanzania	80 to 84	8.3 (2.6–19.1)	8.3 (2.4–19.1)	8.4 (2.6–18.9)	8.5 (2.7–20.4)	8.5 (2.8–20.2)
Tanzania	85 to 89	7.5 (2.3–17.4)	7.5 (2.2–17.3)	7.6 (2.3–17.2)	7.6 (2.5–18.6)	7.7 (2.5–18.4)
Tanzania	90 to 94	6.7 (2.1–15.8)	6.7 (2.0–15.8)	6.8 (2.1–15.7)	6.9 (2.2–17.0)	6.9 (2.2–16.8)
Tanzania	95 plus	6.7 (2.0–15.7)	6.7 (1.9–15.7)	6.8 (2.1–15.6)	6.9 (2.2–16.8)	6.9 (2.2–16.7)
Tanzania	Age-standardized	15.7 (7.5–28.1)	15.6 (8.0–26.1)	17.3 (11.1–25.6)	18.7 (12.5–29.0)	18.8 (12.0–29.9)

Supplementary Table S10: Prevalence of female SVAC by age and location for 1990, 2000, 2010, 2020, and 2023						
**Location**	**Age Range**	**1990**	**2000**	**2010**	**2020**	**2023**
Tanzania	All age	15.9 (7.8–27.7)	16.2 (8.7–26.0)	18.5 (13.2–25.3)	20.1 (15.0–28.7)	20.2 (13.9–29.7)
Zambia	20 to 24	26.4 (10.3–49.0)	26.8 (12.5–45.4)	27.1 (18.2–38.2)	27.2 (16.5–39.5)	27.2 (15.1–43.0)
Zambia	25 to 29	26.5 (10.1–49.2)	26.8 (11.2–48.0)	27.1 (14.4–44.1)	27.2 (18.4–38.1)	27.1 (17.1–40.0)
Zambia	30 to 34	27.6 (10.6–51.6)	27.6 (10.2–51.6)	28.0 (10.7–51.3)	28.3 (11.4–53.8)	28.3 (11.5–53.3)
Zambia	35 to 39	27.6 (10.6–51.6)	27.6 (10.2–51.6)	28.0 (10.7–51.3)	28.3 (11.4–53.8)	28.3 (11.5–53.3)
Zambia	40 to 44	28.2 (10.9–52.4)	28.3 (10.5–52.4)	28.6 (11.0–52.1)	28.9 (11.7–54.6)	28.9 (11.9–54.2)
Zambia	45 to 49	27.6 (10.6–51.6)	27.6 (10.2–51.5)	27.9 (10.7–51.3)	28.3 (11.3–53.7)	28.2 (11.5–53.2)
Zambia	50 to 54	27.0 (10.3–50.9)	27.1 (9.9–50.8)	27.4 (10.4–50.5)	27.7 (11.1–53.0)	27.7 (11.2–52.6)
Zambia	55 to 59	26.3 (10.0–49.8)	26.3 (9.6–49.8)	26.6 (10.0–49.5)	27.0 (10.7–52.0)	26.9 (10.8–51.5)
Zambia	60 to 64	25.6 (9.6–48.9)	25.6 (9.2–48.8)	25.9 (9.7–48.5)	26.2 (10.3–51.0)	26.2 (10.4–50.6)
Zambia	65 to 69	24.1 (8.9–46.7)	24.1 (8.5–46.7)	24.4 (9.0–46.4)	24.7 (9.5–48.9)	24.7 (9.6–48.4)
Zambia	70 to 74	19.8 (7.0–40.3)	19.9 (6.7–40.3)	20.1 (7.0–40.0)	20.4 (7.5–42.4)	20.3 (7.6–42.0)
Zambia	75 to 79	18.1 (6.3–37.4)	18.1 (6.0–37.4)	18.3 (6.3–37.1)	18.6 (6.7–39.5)	18.6 (6.8–39.0)
Zambia	80 to 84	13.2 (4.3–28.9)	13.2 (4.1–28.9)	13.4 (4.4–28.6)	13.6 (4.7–30.7)	13.6 (4.7–30.3)
Zambia	85 to 89	12.0 (3.9–26.6)	12.0 (3.7–26.6)	12.2 (3.9–26.3)	12.4 (4.2–28.3)	12.3 (4.2–27.9)

Supplementary Table S10: Prevalence of female SVAC by age and location for 1990, 2000, 2010, 2020, and 2023						
**Location**	**Age Range**	**1990**	**2000**	**2010**	**2020**	**2023**
Zambia	90 to 94	10.9 (3.5–24.5)	10.9 (3.3–24.5)	11.1 (3.5–24.3)	11.2 (3.8–26.1)	11.2 (3.8–25.8)
Zambia	95 plus	10.9 (3.5–24.3)	10.9 (3.3–24.3)	11.0 (3.5–24.1)	11.2 (3.7–26.0)	11.2 (3.8–25.6)
Zambia	Age-standardized	26.0 (10.2–48.4)	26.1 (10.6–48.4)	26.4 (11.8–46.3)	26.6 (13.4–46.6)	26.6 (13.3–45.9)
Zambia	All age	26.6 (10.7–49.5)	26.8 (11.4–47.8)	27.1 (13.3–45.3)	27.4 (15.3–44.6)	27.3 (15.3–44.8)
Southern sub-Saharan Africa	20 to 24	27.2 (13.3–46.4)	26.8 (13.4–46.0)	25.3 (12.6–42.7)	23.6 (12.4–42.8)	23.4 (12.4–42.1)
Southern sub-Saharan Africa	25 to 29	26.7 (11.9–47.7)	26.4 (11.8–47.5)	25.9 (11.7–45.7)	24.8 (11.5–46.5)	24.5 (11.4–46.5)
Southern sub-Saharan Africa	30 to 34	27.3 (11.6–49.0)	27.2 (11.5–49.4)	27.4 (12.0–48.7)	27.7 (12.8–51.2)	27.8 (13.2–51.3)
Southern sub-Saharan Africa	35 to 39	26.9 (10.3–49.4)	26.7 (10.2–49.9)	26.7 (10.5–49.1)	27.0 (10.5–53.6)	27.1 (10.4–53.7)
Southern sub-Saharan Africa	40 to 44	27.6 (10.9–50.6)	27.4 (10.6–50.9)	27.4 (10.8–50.0)	27.7 (10.8–53.8)	27.7 (10.8–53.7)
Southern sub-Saharan Africa	45 to 49	27.0 (10.4–49.8)	26.8 (10.3–50.1)	26.8 (10.4–49.2)	27.0 (10.5–53.1)	27.1 (10.5–52.9)
Southern sub-Saharan Africa	50 to 54	26.5 (10.2–48.8)	26.3 (10.0–49.5)	26.3 (10.3–48.7)	26.5 (10.1–52.7)	26.6 (10.1–52.6)
Southern sub-Saharan Africa	55 to 59	25.6 (9.6–48.0)	25.5 (9.6–48.3)	25.5 (9.7–47.6)	25.7 (9.7–51.5)	25.8 (9.6–51.7)
Southern sub-Saharan Africa	60 to 64	24.9 (9.2–47.0)	24.8 (9.3–47.4)	24.7 (9.3–46.5)	25.0 (9.4–51.1)	25.1 (9.1–51.6)
Southern sub-Saharan Africa	65 to 69	23.4 (8.5–44.9)	23.2 (8.6–45.2)	23.1 (8.6–44.3)	23.5 (8.4–48.5)	23.5 (8.5–48.5)
Southern sub-Saharan Africa	70 to 74	19.3 (6.7–38.6)	19.1 (6.7–39.0)	19.0 (6.7–38.1)	19.3 (6.6–42.5)	19.4 (6.6–42.6)

Supplementary Table S10: Prevalence of female SVAC by age and location for 1990, 2000, 2010, 2020, and 2023						
**Location**	**Age Range**	**1990**	**2000**	**2010**	**2020**	**2023**
Southern sub-Saharan Africa	75 to 79	17.5 (6.0–35.4)	17.4 (5.9–36.2)	17.3 (6.0–35.3)	17.5 (5.9–39.5)	17.6 (5.9–39.4)
Southern sub-Saharan Africa	80 to 84	12.8 (4.0–27.9)	12.6 (4.1–27.9)	12.6 (4.1–27.1)	12.8 (3.9–30.6)	12.9 (4.0–30.5)
Southern sub-Saharan Africa	85 to 89	11.6 (3.6–25.8)	11.4 (3.6–25.6)	11.4 (3.6–24.9)	11.6 (3.6–28.1)	11.7 (3.5–28.3)
Southern sub-Saharan Africa	90 to 94	10.5 (3.2–23.9)	10.4 (3.2–23.6)	10.4 (3.3–22.9)	10.6 (3.2–25.4)	10.7 (3.1–26.0)
Southern sub-Saharan Africa	95 plus	10.4 (3.1–23.8)	10.3 (3.2–23.5)	10.3 (3.2–22.7)	10.5 (3.0–25.8)	10.5 (3.1–26.0)
Southern sub-Saharan Africa	Age-standardized	25.7 (10.7–46.9)	25.4 (10.5–47.2)	25.2 (10.4–45.6)	25.1 (10.5–49.2)	25.1 (10.4–48.8)
Southern sub-Saharan Africa	All age	26.2 (11.4–47.3)	25.9 (10.8–47.4)	25.6 (10.8–45.8)	25.4 (10.8–49.4)	25.4 (10.6–49.1)
Botswana	20 to 24	27.3 (13.9–44.3)	26.6 (14.5–41.7)	25.0 (16.0–35.3)	23.0 (12.4–36.5)	22.9 (11.2–38.4)
Botswana	25 to 29	32.3 (17.3–50.6)	31.4 (17.8–47.3)	28.7 (19.3–40.2)	24.2 (14.1–36.7)	23.3 (12.5–37.4)
Botswana	30 to 34	32.7 (17.8–50.5)	32.4 (19.0–47.7)	31.7 (20.9–43.8)	31.3 (17.0–48.3)	31.3 (15.0–51.0)
Botswana	35 to 39	37.9 (22.6–55.7)	37.5 (23.6–52.9)	36.5 (25.4–48.6)	35.1 (20.0–52.8)	34.9 (17.9–54.9)
Botswana	40 to 44	37.7 (22.3–55.6)	37.4 (23.4–52.8)	36.4 (25.2–48.4)	35.3 (20.1–53.0)	35.1 (17.5–55.1)
Botswana	45 to 49	36.6 (21.7–54.6)	36.3 (22.7–51.7)	35.3 (24.3–47.4)	34.4 (19.4–51.9)	34.3 (17.6–54.4)
Botswana	50 to 54	37.9 (22.7–55.7)	37.6 (23.8–52.9)	36.5 (25.2–48.6)	35.2 (20.1–52.9)	35.0 (17.9–55.0)
Botswana	55 to 59	31.6 (17.3–49.4)	31.3 (18.3–46.3)	30.6 (19.9–42.6)	30.3 (16.4–47.4)	30.4 (14.3–50.1)

Supplementary Table S10: Prevalence of female SVAC by age and location for 1990, 2000, 2010, 2020, and 2023						
**Location**	**Age Range**	**1990**	**2000**	**2010**	**2020**	**2023**
Botswana	60 to 64	31.2 (16.7–48.9)	30.9 (17.8–46.6)	30.2 (19.4–42.1)	29.9 (16.0–46.8)	29.9 (14.0–49.2)
Botswana	65 to 69	27.1 (13.2–44.9)	26.9 (14.2–42.6)	26.4 (15.9–38.8)	26.4 (13.6–42.7)	26.5 (11.9–45.3)
Botswana	70 to 74	19.9 (8.6–36.2)	19.8 (9.1–34.0)	19.6 (10.3–32.1)	20.0 (9.3–35.8)	20.2 (8.1–37.1)
Botswana	75 to 79	19.0 (8.0–34.6)	18.8 (8.6–32.8)	18.6 (9.7–30.8)	18.9 (8.7–34.1)	19.0 (7.5–35.3)
Botswana	80 to 84	12.7 (3.9–29.0)	12.6 (4.1–27.9)	12.4 (4.4–25.4)	12.7 (4.6–27.6)	12.8 (4.5–28.7)
Botswana	85 to 89	11.6 (3.5–27.2)	11.5 (3.7–25.7)	11.3 (4.0–23.5)	11.5 (4.2–25.4)	11.6 (4.1–26.5)
Botswana	90 to 94	10.7 (3.1–25.2)	10.5 (3.4–23.9)	10.4 (3.7–21.7)	10.6 (3.8–23.6)	10.7 (3.7–24.0)
Botswana	95 plus	10.6 (3.1–24.9)	10.4 (3.3–23.7)	10.3 (3.6–21.5)	10.4 (3.7–23.3)	10.5 (3.7–24.1)
Botswana	Age-standardized	31.9 (17.8–49.0)	31.5 (18.4–46.2)	30.4 (20.0–42.0)	29.1 (16.4–44.4)	28.9 (14.3–46.8)
Botswana	All age	32.1 (18.0–49.4)	31.6 (18.5–46.5)	30.5 (20.1–41.9)	29.5 (16.7–44.9)	29.5 (14.6–47.3)
Eswatini	20 to 24	40.3 (24.7–58.2)	35.3 (23.0–49.1)	23.0 (17.2–29.4)	12.4 (8.2–17.4)	11.4 (6.9–17.6)
Eswatini	25 to 29	23.1 (8.5–43.8)	22.0 (9.9–39.2)	19.5 (13.0–27.9)	17.6 (10.1–27.1)	17.6 (9.2–30.1)
Eswatini	30 to 34	18.2 (6.2–37.2)	17.9 (7.4–33.7)	17.7 (11.3–26.4)	20.7 (12.8–30.2)	21.7 (12.4–34.6)
Eswatini	35 to 39	17.9 (6.2–36.6)	17.9 (7.7–33.6)	18.2 (11.9–26.6)	23.0 (15.6–31.6)	24.9 (15.8–36.6)
Eswatini	40 to 44	20.4 (7.1–40.4)	19.7 (8.6–36.2)	18.1 (11.8–26.4)	18.2 (10.7–27.7)	18.8 (10.0–31.5)

Supplementary Table S10: Prevalence of female SVAC by age and location for 1990, 2000, 2010, 2020, and 2023						
**Location**	**Age Range**	**1990**	**2000**	**2010**	**2020**	**2023**
Eswatini	45 to 49	19.7 (6.9–39.6)	19.0 (8.1–35.3)	17.5 (11.3–25.9)	17.7 (10.3–27.1)	18.2 (9.6–30.5)
Eswatini	50 to 54	20.1 (6.9–40.0)	19.5 (8.4–35.9)	18.2 (11.9–26.5)	18.3 (10.7–27.8)	18.7 (10.0–31.4)
Eswatini	55 to 59	17.7 (6.1–36.1)	17.0 (6.9–32.0)	15.4 (9.4–23.6)	15.7 (8.8–24.7)	16.2 (8.1–28.2)
Eswatini	60 to 64	17.4 (6.1–35.3)	16.7 (6.8–31.1)	15.2 (9.2–23.4)	15.4 (8.6–24.3)	15.9 (7.9–27.7)
Eswatini	65 to 69	15.5 (5.3–32.2)	14.8 (6.1–27.3)	13.3 (7.6–21.1)	13.6 (7.2–22.9)	14.1 (6.7–25.4)
Eswatini	70 to 74	15.4 (4.0–36.0)	15.2 (4.5–33.8)	14.9 (4.9–31.2)	14.9 (5.2–33.1)	14.9 (5.2–32.8)
Eswatini	75 to 79	14.0 (3.5–33.2)	13.7 (4.0–31.1)	13.5 (4.4–28.7)	13.5 (4.6–30.5)	13.5 (4.7–30.1)
Eswatini	80 to 84	10.1 (2.4–25.3)	9.9 (2.8–22.9)	9.7 (3.0–21.5)	9.7 (3.2–23.0)	9.7 (3.2–22.7)
Eswatini	85 to 89	9.2 (2.2–23.2)	9.0 (2.5–21.4)	8.7 (2.7–19.6)	8.8 (2.8–21.0)	8.8 (2.9–20.8)
Eswatini	90 to 94	8.3 (2.0–21.2)	8.1 (2.2–19.5)	7.9 (2.4–17.9)	8.0 (2.6–19.3)	8.0 (2.6–19.0)
Eswatini	95 plus	8.3 (1.9–21.1)	8.1 (2.2–19.0)	7.9 (2.4–17.8)	7.9 (2.5–19.1)	7.9 (2.6–18.9)
Eswatini	Age-standardized	21.3 (8.9–39.3)	20.2 (9.7–34.9)	17.7 (11.7–25.8)	17.1 (10.6–25.2)	17.5 (9.9–28.4)
Eswatini	All age	24.0 (11.0–42.3)	22.4 (11.6–37.7)	18.8 (12.8–26.5)	17.4 (10.7–25.6)	17.8 (10.0–28.8)
Lesotho	20 to 24	21.4 (7.8–41.7)	21.1 (8.5–39.6)	20.6 (10.6–34.8)	20.2 (12.6–29.9)	20.3 (11.8–32.4)
Lesotho	25 to 29	22.0 (7.9–43.6)	22.0 (7.6–43.5)	22.0 (7.9–42.9)	22.1 (8.3–45.1)	22.1 (8.4–44.6)

Supplementary Table S10: Prevalence of female SVAC by age and location for 1990, 2000, 2010, 2020, and 2023						
**Location**	**Age Range**	**1990**	**2000**	**2010**	**2020**	**2023**
Lesotho	30 to 34	23.6 (8.7–46.0)	23.6 (8.3–45.9)	23.6 (8.6–45.3)	23.7 (9.1–47.5)	23.7 (9.2–47.1)
Lesotho	35 to 39	23.8 (8.8–46.4)	23.8 (8.4–46.3)	23.9 (8.7–45.7)	24.0 (9.2–47.9)	24.0 (9.3–47.4)
Lesotho	40 to 44	24.6 (9.1–47.4)	24.5 (8.7–47.3)	24.6 (9.1–46.7)	24.7 (9.5–48.9)	24.7 (9.6–48.5)
Lesotho	45 to 49	24.0 (8.9–46.7)	24.0 (8.5–46.6)	24.1 (8.8–46.0)	24.2 (9.3–48.1)	24.2 (9.4–47.7)
Lesotho	50 to 54	23.6 (8.7–46.0)	23.6 (8.3–45.9)	23.6 (8.6–45.3)	23.7 (9.1–47.5)	23.7 (9.2–47.0)
Lesotho	55 to 59	22.9 (8.4–45.0)	22.9 (8.0–45.0)	23.0 (8.3–44.4)	23.1 (8.7–46.5)	23.1 (8.8–46.1)
Lesotho	60 to 64	22.3 (8.1–44.1)	22.3 (7.7–44.0)	22.3 (8.0–43.4)	22.4 (8.4–45.6)	22.4 (8.5–45.1)
Lesotho	65 to 69	20.9 (7.5–42.0)	20.9 (7.1–41.9)	21.0 (7.4–41.3)	21.0 (7.8–43.4)	21.0 (7.9–43.0)
Lesotho	70 to 74	17.1 (5.9–35.8)	17.1 (5.6–35.7)	17.1 (5.8–35.1)	17.2 (6.1–37.2)	17.2 (6.2–36.8)
Lesotho	75 to 79	15.5 (5.2–33.1)	15.5 (5.0–33.0)	15.5 (5.2–32.5)	15.6 (5.5–34.4)	15.6 (5.5–34.0)
Lesotho	80 to 84	11.3 (3.6–25.1)	11.2 (3.4–25.0)	11.3 (3.6–24.6)	11.3 (3.8–26.3)	11.3 (3.8–25.9)
Lesotho	85 to 89	10.2 (3.2–23.0)	10.2 (3.1–22.9)	10.2 (3.2–22.5)	10.2 (3.4–24.1)	10.2 (3.4–23.8)
Lesotho	90 to 94	9.3 (2.9–21.1)	9.2 (2.8–21.0)	9.2 (2.9–20.6)	9.3 (3.0–22.1)	9.3 (3.1–21.8)
Lesotho	95 plus	9.2 (2.9–20.9)	9.2 (2.7–20.9)	9.2 (2.8–20.5)	9.2 (3.0–22.0)	9.2 (3.1–21.7)
Lesotho	Age-standardized	22.2 (8.2–43.4)	22.1 (8.0–43.5)	22.1 (8.4–41.7)	22.1 (9.3–43.1)	22.1 (9.4–42.6)

Supplementary Table S10: Prevalence of female SVAC by age and location for 1990, 2000, 2010, 2020, and 2023						
**Location**	**Age Range**	**1990**	**2000**	**2010**	**2020**	**2023**
Lesotho	All age	22.4 (8.3–43.8)	22.2 (8.3–43.3)	22.2 (8.8–41.5)	22.3 (9.8–42.3)	22.3 (9.9–42.2)
Namibia	20 to 24	20.5 (7.3–40.9)	20.2 (7.9–38.1)	19.5 (9.6–34.4)	18.9 (11.8–28.3)	19.0 (11.2–30.3)
Namibia	25 to 29	21.3 (7.6–42.5)	21.2 (7.3–42.4)	21.3 (7.6–41.8)	21.3 (7.9–43.8)	21.3 (8.0–43.4)
Namibia	30 to 34	22.9 (8.4–45.0)	22.9 (8.0–44.9)	22.9 (8.3–44.3)	22.9 (8.7–46.3)	22.9 (8.8–45.9)
Namibia	35 to 39	23.2 (8.5–45.4)	23.2 (8.1–45.3)	23.2 (8.4–44.7)	23.2 (8.8–46.7)	23.2 (8.9–46.3)
Namibia	40 to 44	23.9 (8.8–46.5)	23.9 (8.4–46.4)	24.0 (8.8–45.8)	23.9 (9.2–47.8)	23.9 (9.3–47.4)
Namibia	45 to 49	23.4 (8.6–45.8)	23.4 (8.2–45.7)	23.5 (8.5–45.1)	23.4 (8.9–47.1)	23.4 (9.0–46.7)
Namibia	50 to 54	23.0 (8.4–45.2)	23.0 (8.0–45.1)	23.0 (8.3–44.4)	23.0 (8.7–46.5)	23.0 (8.8–46.0)
Namibia	55 to 59	22.4 (8.1–44.2)	22.4 (7.8–44.1)	22.4 (8.1–43.5)	22.4 (8.4–45.5)	22.4 (8.5–45.1)
Namibia	60 to 64	21.8 (7.8–43.3)	21.7 (7.5–43.2)	21.8 (7.8–42.6)	21.8 (8.1–44.6)	21.8 (8.3–44.1)
Namibia	65 to 69	20.4 (7.2–41.2)	20.4 (6.9–41.1)	20.4 (7.2–40.5)	20.4 (7.5–42.5)	20.4 (7.6–42.1)
Namibia	70 to 74	16.7 (5.7–35.0)	16.6 (5.4–35.0)	16.7 (5.6–34.4)	16.7 (5.9–36.2)	16.7 (6.0–35.9)
Namibia	75 to 79	15.1 (5.1–32.4)	15.1 (4.8–32.3)	15.1 (5.0–31.7)	15.1 (5.3–33.5)	15.1 (5.3–33.1)
Namibia	80 to 84	10.9 (3.5–24.5)	10.9 (3.3–24.4)	10.9 (3.5–24.0)	10.9 (3.6–25.5)	10.9 (3.7–25.2)
Namibia	85 to 89	9.9 (3.1–22.4)	9.9 (3.0–22.3)	9.9 (3.1–21.9)	9.9 (3.2–23.4)	9.9 (3.3–23.0)

Supplementary Table S10: Prevalence of female SVAC by age and location for 1990, 2000, 2010, 2020, and 2023						
**Location**	**Age Range**	**1990**	**2000**	**2010**	**2020**	**2023**
Namibia	90 to 94	9.0 (2.8–20.6)	9.0 (2.7–20.5)	9.0 (2.8–20.1)	9.0 (2.9–21.4)	9.0 (3.0–21.2)
Namibia	95 plus	8.9 (2.8–20.4)	8.9 (2.6–20.3)	8.9 (2.8–19.9)	8.9 (2.9–21.3)	8.9 (2.9–21.0)
Namibia	Age-standardized	21.6 (7.9–42.4)	21.5 (7.7–42.5)	21.4 (8.0–40.8)	21.4 (8.8–41.9)	21.4 (9.1–41.5)
Namibia	All age	21.8 (8.0–42.9)	21.7 (8.0–42.6)	21.6 (8.4–40.8)	21.6 (9.2–41.6)	21.6 (9.4–41.5)
South Africa	20 to 24	24.1 (8.1–48.0)	23.9 (8.5–46.5)	24.0 (8.8–45.9)	24.4 (8.4–51.3)	24.5 (8.0–51.2)
South Africa	25 to 29	24.6 (8.3–48.7)	24.4 (8.7–47.2)	24.5 (9.0–46.6)	24.9 (8.7–52.2)	25.0 (8.5–52.5)
South Africa	30 to 34	26.3 (9.1–51.5)	26.1 (9.5–49.5)	26.2 (9.8–48.9)	26.6 (9.4–55.0)	26.7 (9.2–55.1)
South Africa	35 to 39	26.5 (9.2–51.8)	26.3 (9.6–49.8)	26.4 (9.9–49.3)	26.8 (9.5–54.7)	26.9 (9.4–55.2)
South Africa	40 to 44	27.2 (9.5–52.4)	27.1 (9.9–50.9)	27.2 (10.3–50.3)	27.5 (9.7–55.7)	27.6 (9.4–55.7)
South Africa	45 to 49	26.7 (9.3–52.0)	26.5 (9.7–50.1)	26.6 (10.0–49.5)	26.9 (9.5–54.8)	27.0 (9.4–54.8)
South Africa	50 to 54	26.1 (9.1–51.0)	26.0 (9.4–49.4)	26.1 (9.8–48.8)	26.4 (9.4–54.2)	26.5 (8.9–53.6)
South Africa	55 to 59	25.4 (8.7–50.3)	25.3 (9.1–48.4)	25.4 (9.4–47.8)	25.7 (9.0–52.4)	25.8 (8.8–53.4)
South Africa	60 to 64	24.7 (8.4–49.0)	24.6 (8.8–47.4)	24.7 (9.1–46.8)	25.0 (8.7–52.2)	25.1 (8.5–52.7)
South Africa	65 to 69	23.3 (7.7–47.0)	23.1 (8.1–45.2)	23.1 (8.4–44.6)	23.5 (8.0–50.0)	23.6 (7.8–50.3)
South Africa	70 to 74	19.2 (6.1–40.5)	19.0 (6.3–38.8)	19.0 (6.6–38.2)	19.3 (6.3–43.5)	19.4 (6.3–43.8)

Supplementary Table S10: Prevalence of female SVAC by age and location for 1990, 2000, 2010, 2020, and 2023						
**Location**	**Age Range**	**1990**	**2000**	**2010**	**2020**	**2023**
South Africa	75 to 79	17.4 (5.4–37.3)	17.3 (5.7–36.0)	17.3 (5.9–35.4)	17.6 (5.5–39.5)	17.7 (5.4–40.4)
South Africa	80 to 84	12.7 (3.7–28.8)	12.6 (3.9–27.6)	12.6 (4.1–27.1)	12.9 (3.9–30.4)	13.0 (3.8–31.5)
South Africa	85 to 89	11.6 (3.4–26.6)	11.4 (3.5–25.3)	11.4 (3.6–24.9)	11.7 (3.5–27.7)	11.8 (3.4–29.0)
South Africa	90 to 94	10.5 (3.0–24.4)	10.3 (3.1–23.3)	10.4 (3.3–22.9)	10.6 (3.1–25.7)	10.7 (3.0–26.3)
South Africa	95 plus	10.4 (3.0–24.3)	10.3 (3.1–23.1)	10.3 (3.2–22.7)	10.5 (3.0–25.7)	10.6 (2.9–26.6)
South Africa	Age-standardized	24.7 (8.5–48.8)	24.6 (8.8–47.2)	24.6 (9.1–46.6)	25.0 (8.7–51.9)	25.1 (8.5–52.3)
South Africa	All age	24.9 (8.5–49.1)	24.8 (8.9–47.6)	24.8 (9.2–46.9)	25.2 (8.8–52.3)	25.4 (8.6–52.7)
Zimbabwe	20 to 24	39.5 (23.8–57.9)	37.5 (23.8–53.1)	32.0 (24.0–40.7)	22.9 (14.0–34.1)	21.5 (11.7–34.6)
Zimbabwe	25 to 29	36.6 (16.2–60.6)	35.6 (17.4–57.4)	32.9 (20.4–48.0)	26.0 (18.4–35.1)	23.9 (15.4–34.7)
Zimbabwe	30 to 34	33.4 (11.7–59.9)	33.4 (13.3–57.9)	33.8 (17.8–53.5)	34.9 (25.1–47.2)	34.9 (24.3–47.5)
Zimbabwe	35 to 39	28.9 (9.3–56.7)	28.6 (9.9–55.0)	28.0 (10.6–52.1)	27.8 (11.1–53.1)	27.8 (11.2–53.5)
Zimbabwe	40 to 44	29.7 (9.4–57.9)	29.5 (10.2–55.6)	28.8 (11.0–53.1)	28.5 (11.5–54.1)	28.5 (11.6–54.3)
Zimbabwe	45 to 49	29.0 (9.2–57.0)	28.8 (9.9–55.2)	28.2 (10.7–52.3)	27.9 (11.2–53.3)	28.0 (11.2–53.5)
Zimbabwe	50 to 54	28.6 (9.3–56.3)	28.3 (9.9–54.5)	27.7 (10.5–51.6)	27.4 (10.9–52.6)	27.4 (11.0–52.8)
Zimbabwe	55 to 59	27.8 (8.7–55.5)	27.5 (9.5–53.5)	27.0 (10.1–50.7)	26.7 (10.5–51.7)	26.7 (10.6–52.0)

Supplementary Table S10: Prevalence of female SVAC by age and location for 1990, 2000, 2010, 2020, and 2023						
**Location**	**Age Range**	**1990**	**2000**	**2010**	**2020**	**2023**
Zimbabwe	60 to 64	27.0 (8.3–54.7)	26.8 (9.0–53.3)	26.2 (9.7–49.7)	26.0 (10.2–50.7)	26.0 (10.3–50.9)
Zimbabwe	65 to 69	25.4 (7.8–52.4)	25.2 (8.4–50.4)	24.7 (9.0–47.6)	24.4 (9.4–48.5)	24.4 (9.5–48.9)
Zimbabwe	70 to 74	21.1 (6.1–46.0)	20.9 (6.5–44.0)	20.4 (7.1–40.9)	20.1 (7.4–42.0)	20.1 (7.4–42.1)
Zimbabwe	75 to 79	19.2 (5.5–43.2)	19.0 (6.0–41.1)	18.5 (6.3–38.0)	18.4 (6.6–39.1)	18.3 (6.7–38.9)
Zimbabwe	80 to 84	14.1 (3.8–33.4)	13.9 (4.0–32.1)	13.6 (4.4–29.5)	13.4 (4.6–30.4)	13.4 (4.6–30.0)
Zimbabwe	85 to 89	12.8 (3.4–31.2)	12.6 (3.6–29.6)	12.3 (3.9–27.1)	12.2 (4.1–28.0)	12.2 (4.1–27.6)
Zimbabwe	90 to 94	11.7 (3.1–28.4)	11.5 (3.4–27.2)	11.2 (3.5–25.0)	11.1 (3.7–25.8)	11.1 (3.7–25.6)
Zimbabwe	95 plus	11.6 (3.0–28.7)	11.4 (3.2–27.0)	11.1 (3.5–24.8)	11.0 (3.7–25.6)	11.0 (3.7–25.2)
Zimbabwe	Age-standardized	30.3 (12.7–52.3)	29.8 (13.2–50.7)	28.5 (14.2–47.2)	26.5 (16.0–43.0)	26.1 (16.1–41.8)
Zimbabwe	All age	32.6 (15.3–54.4)	31.9 (15.7–52.5)	30.1 (16.8–46.8)	27.1 (18.0–41.1)	26.6 (17.7–40.4)
Western sub-Saharan Africa	20 to 24	24.0 (11.7–40.3)	24.3 (13.8–38.3)	23.7 (14.8–36.1)	23.0 (13.1–37.8)	23.0 (12.1–39.1)
Western sub-Saharan Africa	25 to 29	23.8 (10.8–41.5)	23.9 (13.0–38.5)	23.1 (13.5–36.3)	23.5 (14.5–36.7)	23.8 (14.5–37.2)
Western sub-Saharan Africa	30 to 34	24.7 (10.7–43.9)	24.2 (11.9–40.6)	22.6 (12.7–35.9)	21.8 (11.4–37.5)	21.9 (10.4–38.3)
Western sub-Saharan Africa	35 to 39	24.4 (10.6–43.3)	24.3 (12.0–40.7)	23.6 (14.0–36.6)	23.2 (12.8–38.5)	23.2 (11.8–40.0)
Western sub-Saharan Africa	40 to 44	24.2 (10.5–43.4)	23.8 (11.6–40.2)	23.5 (13.3–37.3)	23.7 (13.3–38.6)	23.8 (12.7–39.5)

Supplementary Table S10: Prevalence of female SVAC by age and location for 1990, 2000, 2010, 2020, and 2023						
**Location**	**Age Range**	**1990**	**2000**	**2010**	**2020**	**2023**
Western sub-Saharan Africa	45 to 49	24.8 (10.8–44.4)	24.4 (11.6–41.8)	23.3 (12.8–37.6)	22.8 (12.3–38.3)	22.8 (11.4–38.8)
Western sub-Saharan Africa	50 to 54	24.1 (10.4–43.3)	23.7 (11.1–40.6)	22.9 (12.6–36.7)	22.5 (12.0–38.2)	22.5 (10.8–38.6)
Western sub-Saharan Africa	55 to 59	22.1 (8.6–42.1)	21.5 (8.6–40.5)	20.5 (9.5–36.0)	20.5 (10.2–36.2)	20.7 (9.6–36.6)
Western sub-Saharan Africa	60 to 64	21.5 (8.3–41.2)	20.7 (8.2–39.3)	19.9 (9.1–35.5)	20.0 (9.9–35.7)	20.2 (9.2–36.1)
Western sub-Saharan Africa	65 to 69	21.1 (7.9–41.3)	20.3 (7.4–40.5)	20.5 (7.3–40.2)	20.7 (7.7–42.5)	20.9 (7.8–43.2)
Western sub-Saharan Africa	70 to 74	17.4 (6.2–35.5)	16.7 (5.8–34.7)	16.6 (5.7–33.9)	16.8 (6.0–36.2)	17.0 (6.1–36.9)
Western sub-Saharan Africa	75 to 79	15.9 (5.6–32.9)	15.6 (5.3–32.8)	15.0 (5.1–31.3)	15.3 (5.4–33.6)	15.3 (5.4–33.9)
Western sub-Saharan Africa	80 to 84	11.6 (3.9–25.3)	11.5 (3.7–25.3)	11.0 (3.5–24.0)	11.1 (3.7–25.6)	11.2 (3.8–26.2)
Western sub-Saharan Africa	85 to 89	10.5 (3.4–23.2)	10.5 (3.4–23.4)	10.3 (3.3–22.7)	10.0 (3.3–23.5)	10.1 (3.4–23.9)
Western sub-Saharan Africa	90 to 94	9.6 (3.0–21.3)	9.6 (3.0–21.6)	9.5 (3.0–21.1)	9.2 (3.0–21.8)	9.2 (3.0–22.1)
Western sub-Saharan Africa	95 plus	9.6 (3.0–21.4)	9.5 (2.9–21.5)	9.5 (3.0–21.1)	9.5 (3.1–22.5)	9.4 (3.1–22.7)
Western sub-Saharan Africa	Age-standardized	22.9 (9.9–41.4)	22.6 (10.9–39.2)	21.9 (12.0–35.8)	21.7 (11.5–37.1)	21.8 (10.7–37.8)
Western sub-Saharan Africa	All age	23.5 (10.5–41.8)	23.4 (11.7–39.4)	22.6 (13.0–36.0)	22.4 (12.3–37.6)	22.5 (11.5–38.0)
Benin	20 to 24	16.1 (5.4–34.0)	16.0 (5.2–33.8)	15.9 (5.3–33.1)	15.9 (5.6–34.8)	15.8 (5.6–34.4)
Benin	25 to 29	16.5 (5.6–34.8)	16.5 (5.4–34.7)	16.4 (5.5–33.9)	16.3 (5.8–35.7)	16.3 (5.8–35.2)

Supplementary Table S10: Prevalence of female SVAC by age and location for 1990, 2000, 2010, 2020, and 2023						
**Location**	**Age Range**	**1990**	**2000**	**2010**	**2020**	**2023**
Benin	30 to 34	17.9 (6.2–37.1)	17.8 (5.9–37.0)	17.8 (6.1–36.2)	17.7 (6.3–38.0)	17.6 (6.4–37.5)
Benin	35 to 39	18.1 (6.3–37.5)	18.1 (6.0–37.4)	18.0 (6.2–36.6)	17.9 (6.4–38.4)	17.9 (6.5–37.9)
Benin	40 to 44	18.8 (6.5–38.5)	18.7 (6.2–38.4)	18.6 (6.4–37.6)	18.5 (6.7–39.4)	18.5 (6.8–39.0)
Benin	45 to 49	18.3 (6.3–37.8)	18.3 (6.1–37.7)	18.2 (6.2–36.9)	18.1 (6.5–38.7)	18.1 (6.5–38.2)
Benin	50 to 54	17.9 (6.2–37.1)	17.8 (5.9–37.0)	17.8 (6.1–36.2)	17.7 (6.3–38.0)	17.6 (6.4–37.5)
Benin	55 to 59	17.3 (5.9–36.1)	17.3 (5.7–36.0)	17.2 (5.8–35.2)	17.1 (6.1–37.0)	17.1 (6.1–36.3)
Benin	60 to 64	16.7 (5.7–35.2)	16.7 (5.4–35.0)	16.6 (5.6–34.3)	16.5 (5.8–36.0)	16.5 (5.9–35.6)
Benin	65 to 69	15.6 (5.2–33.2)	15.6 (5.0–33.1)	15.5 (5.2–32.3)	15.4 (5.4–34.0)	15.4 (5.4–33.6)
Benin	70 to 74	12.6 (4.1–27.6)	12.5 (3.9–27.6)	12.5 (4.0–26.9)	12.4 (4.2–28.4)	12.4 (4.2–28.0)
Benin	75 to 79	11.4 (3.6–25.3)	11.3 (3.5–25.2)	11.3 (3.6–24.6)	11.2 (3.7–26.0)	11.2 (3.8–25.7)
Benin	80 to 84	8.1 (2.5–18.7)	8.1 (2.4–18.6)	8.0 (2.5–18.1)	8.0 (2.6–19.3)	8.0 (2.6–19.0)
Benin	85 to 89	7.3 (2.2–17.0)	7.3 (2.1–17.0)	7.2 (2.2–16.5)	7.2 (2.3–17.6)	7.2 (2.3–17.3)
Benin	90 to 94	6.6 (2.0–15.5)	6.6 (1.9–15.5)	6.5 (2.0–15.0)	6.5 (2.1–16.0)	6.5 (2.1–15.7)
Benin	95 plus	6.6 (2.0–15.4)	6.5 (1.9–15.3)	6.5 (2.0–14.9)	6.4 (2.0–15.9)	6.4 (2.1–15.7)
Benin	Age-standardized	16.7 (5.7–35.0)	16.7 (5.5–34.9)	16.6 (5.6–34.2)	16.5 (5.9–35.9)	16.5 (5.9–35.5)

Supplementary Table S10: Prevalence of female SVAC by age and location for 1990, 2000, 2010, 2020, and 2023						
**Location**	**Age Range**	**1990**	**2000**	**2010**	**2020**	**2023**
Benin	All age	16.9 (5.8–35.4)	16.9 (5.5–35.3)	16.8 (5.7–34.6)	16.8 (6.0–36.4)	16.8 (6.0–36.0)
Burkina Faso	20 to 24	21.5 (6.5–45.8)	21.4 (6.7–45.5)	21.3 (6.8–44.8)	21.3 (7.9–43.9)	21.5 (8.0–44.4)
Burkina Faso	25 to 29	21.8 (6.7–46.3)	21.7 (6.9–46.0)	21.6 (6.9–45.4)	21.7 (8.1–44.4)	21.8 (8.2–44.9)
Burkina Faso	30 to 34	23.2 (7.2–48.5)	23.2 (7.4–48.2)	23.1 (7.5–47.6)	23.1 (8.8–46.6)	23.2 (8.8–47.0)
Burkina Faso	35 to 39	23.3 (7.3–48.7)	23.3 (7.5–48.4)	23.2 (7.5–47.7)	23.2 (8.8–46.8)	23.3 (8.9–47.2)
Burkina Faso	40 to 44	24.0 (7.5–49.6)	23.9 (7.7–49.3)	23.8 (7.8–48.6)	23.8 (9.1–47.7)	24.0 (9.2–48.1)
Burkina Faso	45 to 49	23.3 (7.3–48.7)	23.3 (7.5–48.4)	23.2 (7.5–47.7)	23.2 (8.8–46.8)	23.3 (8.9–47.2)
Burkina Faso	50 to 54	22.8 (7.1–47.9)	22.8 (7.3–47.6)	22.7 (7.3–46.9)	22.7 (8.6–45.9)	22.8 (8.6–46.3)
Burkina Faso	55 to 59	22.1 (6.8–46.8)	22.0 (7.0–46.4)	21.9 (7.0–45.8)	21.9 (8.2–44.8)	22.1 (8.3–45.3)
Burkina Faso	60 to 64	21.4 (6.5–45.7)	21.3 (6.7–45.4)	21.2 (6.8–44.8)	21.3 (7.9–43.8)	21.4 (8.0–44.2)
Burkina Faso	65 to 69	20.0 (6.0–43.5)	20.0 (6.2–43.2)	19.9 (6.2–42.6)	19.9 (7.3–41.7)	20.0 (7.3–42.1)
Burkina Faso	70 to 74	16.4 (4.7–37.3)	16.3 (4.8–37.0)	16.2 (4.9–36.4)	16.2 (5.7–35.5)	16.3 (5.8–35.9)
Burkina Faso	75 to 79	14.9 (4.2–34.5)	14.8 (4.3–34.2)	14.7 (4.3–33.6)	14.7 (5.1–32.8)	14.8 (5.1–33.2)
Burkina Faso	80 to 84	10.8 (2.9–26.4)	10.7 (3.0–26.1)	10.7 (3.0–25.6)	10.6 (3.5–24.9)	10.7 (3.5–25.2)
Burkina Faso	85 to 89	9.8 (2.6–24.2)	9.7 (2.6–23.9)	9.6 (2.7–23.5)	9.6 (3.1–22.8)	9.7 (3.2–23.1)

Supplementary Table S10: Prevalence of female SVAC by age and location for 1990, 2000, 2010, 2020, and 2023						
**Location**	**Age Range**	**1990**	**2000**	**2010**	**2020**	**2023**
Burkina Faso	90 to 94	8.9 (2.3–22.2)	8.8 (2.4–22.0)	8.7 (2.4–21.6)	8.7 (2.8–20.9)	8.8 (2.9–21.3)
Burkina Faso	95 plus	8.8 (2.3–22.1)	8.8 (2.4–21.8)	8.7 (2.4–21.4)	8.6 (2.8–20.8)	8.7 (2.8–21.0)
Burkina Faso	Age-standardized	21.7 (6.7–46.0)	21.6 (6.8–45.7)	21.5 (6.9–45.0)	21.6 (8.1–44.1)	21.7 (8.1–44.5)
Burkina Faso	All age	22.1 (6.8–46.7)	22.0 (7.0–46.3)	22.0 (7.0–45.8)	22.0 (8.3–44.8)	22.1 (8.3–45.3)
Cabo Verde	20 to 24	19.4 (6.8–39.6)	19.3 (6.5–39.4)	19.2 (6.7–38.6)	19.2 (7.0–40.6)	19.2 (7.1–40.2)
Cabo Verde	25 to 29	19.7 (6.9–40.0)	19.6 (6.6–39.9)	19.5 (6.8–39.0)	19.5 (7.1–41.0)	19.5 (7.2–40.6)
Cabo Verde	30 to 34	21.0 (7.5–42.0)	20.9 (7.1–41.9)	20.8 (7.3–41.1)	20.8 (7.7–43.0)	20.7 (7.8–42.5)
Cabo Verde	35 to 39	21.0 (7.5–42.2)	21.0 (7.2–42.0)	20.9 (7.4–41.2)	20.8 (7.7–43.1)	20.8 (7.8–42.6)
Cabo Verde	40 to 44	21.6 (7.8–43.0)	21.5 (7.4–42.8)	21.4 (7.6–42.0)	21.3 (7.9–43.9)	21.3 (8.1–43.4)
Cabo Verde	45 to 49	20.9 (7.5–42.0)	20.9 (7.1–41.9)	20.7 (7.3–41.0)	20.7 (7.6–42.9)	20.7 (7.7–42.5)
Cabo Verde	50 to 54	20.3 (7.2–41.0)	20.3 (6.9–40.9)	20.1 (7.1–40.0)	20.1 (7.4–41.9)	20.1 (7.5–41.5)
Cabo Verde	55 to 59	19.6 (6.9–39.8)	19.5 (6.6–39.7)	19.4 (6.7–38.8)	19.3 (7.0–40.7)	19.3 (7.1–40.3)
Cabo Verde	60 to 64	18.9 (6.6–38.7)	18.8 (6.3–38.6)	18.7 (6.4–37.7)	18.6 (6.7–39.5)	18.6 (6.8–39.0)
Cabo Verde	65 to 69	17.6 (6.0–36.6)	17.5 (5.8–36.5)	17.4 (5.9–35.6)	17.3 (6.2–37.4)	17.3 (6.3–36.8)
Cabo Verde	70 to 74	14.2 (4.7–30.7)	14.2 (4.5–30.6)	14.1 (4.6–29.9)	14.0 (4.8–31.5)	14.0 (4.9–31.0)

Supplementary Table S10: Prevalence of female SVAC by age and location for 1990, 2000, 2010, 2020, and 2023						
**Location**	**Age Range**	**1990**	**2000**	**2010**	**2020**	**2023**
Cabo Verde	75 to 79	12.9 (4.2–28.3)	12.9 (4.0–28.1)	12.7 (4.1–27.4)	12.7 (4.3–29.0)	12.7 (4.4–28.6)
Cabo Verde	80 to 84	9.3 (2.9–21.1)	9.2 (2.8–21.0)	9.1 (2.8–20.4)	9.1 (3.0–21.7)	9.1 (3.0–21.4)
Cabo Verde	85 to 89	8.4 (2.6–19.3)	8.3 (2.5–19.2)	8.2 (2.5–18.6)	8.2 (2.6–19.8)	8.2 (2.7–19.5)
Cabo Verde	90 to 94	7.6 (2.3–17.6)	7.5 (2.2–17.5)	7.5 (2.3–17.0)	7.4 (2.4–18.1)	7.4 (2.4–17.9)
Cabo Verde	95 plus	7.5 (2.3–17.5)	7.5 (2.2–17.4)	7.4 (2.3–16.9)	7.4 (2.4–18.0)	7.4 (2.4–17.7)
Cabo Verde	Age-standardized	19.4 (6.8–39.4)	19.4 (6.5–39.3)	19.2 (6.7–38.5)	19.2 (7.0–40.3)	19.2 (7.1–39.9)
Cabo Verde	All age	19.3 (6.8–39.2)	19.3 (6.5–39.3)	19.2 (6.7–38.5)	19.3 (7.1–40.6)	19.3 (7.2–40.1)
Cameroon	20 to 24	11.9 (3.9–25.7)	12.4 (4.8–25.2)	13.0 (7.2–21.5)	13.0 (8.5–18.5)	12.9 (7.5–20.7)
Cameroon	25 to 29	11.4 (3.7–25.2)	11.4 (4.3–23.3)	11.1 (5.9–19.0)	10.9 (6.8–16.4)	11.0 (6.1–18.1)
Cameroon	30 to 34	11.8 (3.9–25.9)	11.4 (4.3–23.3)	10.6 (5.6–18.5)	10.4 (6.3–15.8)	10.5 (5.8–17.6)
Cameroon	35 to 39	12.1 (4.0–26.5)	11.9 (4.5–24.4)	11.5 (6.1–19.8)	11.3 (7.0–17.1)	11.4 (6.4–18.9)
Cameroon	40 to 44	12.4 (4.1–27.0)	12.3 (4.7–24.9)	11.9 (6.4–20.2)	11.7 (7.4–17.4)	11.8 (6.6–19.2)
Cameroon	45 to 49	11.8 (3.8–25.8)	11.6 (4.3–23.7)	11.1 (5.7–19.4)	10.9 (6.5–16.8)	11.0 (5.9–18.5)
Cameroon	50 to 54	10.6 (3.5–23.3)	9.7 (3.4–20.7)	8.6 (3.9–15.7)	8.3 (4.2–14.3)	8.5 (4.1–15.5)
Cameroon	55 to 59	10.6 (3.5–23.2)	10.1 (3.6–21.0)	9.3 (4.6–16.6)	9.0 (5.0–14.9)	9.2 (4.6–16.1)

Supplementary Table S10: Prevalence of female SVAC by age and location for 1990, 2000, 2010, 2020, and 2023						
**Location**	**Age Range**	**1990**	**2000**	**2010**	**2020**	**2023**
Cameroon	60 to 64	10.0 (3.3–22.1)	9.5 (3.3–20.1)	8.6 (4.0–15.7)	8.4 (4.3–14.5)	8.5 (4.1–15.5)
Cameroon	65 to 69	9.8 (3.1–22.1)	9.7 (2.9–22.1)	9.7 (3.0–21.6)	9.8 (3.2–23.2)	9.8 (3.3–22.8)
Cameroon	70 to 74	7.7 (2.4–18.0)	7.7 (2.3–17.9)	7.7 (2.4–17.5)	7.8 (2.5–18.8)	7.8 (2.5–18.6)
Cameroon	75 to 79	6.9 (2.1–16.3)	6.9 (2.0–16.2)	6.9 (2.1–15.9)	7.0 (2.2–17.1)	7.0 (2.3–16.8)
Cameroon	80 to 84	4.9 (1.5–11.7)	4.8 (1.4–11.6)	4.8 (1.4–11.3)	4.9 (1.5–12.3)	4.9 (1.5–12.1)
Cameroon	85 to 89	4.4 (1.3–10.5)	4.4 (1.2–10.5)	4.3 (1.3–10.2)	4.4 (1.4–11.1)	4.4 (1.4–10.9)
Cameroon	90 to 94	3.9 (1.2– 9.5)	3.9 (1.1– 9.5)	3.9 (1.1– 9.3)	3.9 (1.2–10.0)	3.9 (1.2– 9.9)
Cameroon	95 plus	3.9 (1.2– 9.4)	3.9 (1.1– 9.4)	3.9 (1.1– 9.2)	3.9 (1.2–10.0)	3.9 (1.2– 9.8)
Cameroon	Age-standardized	11.0 (3.6–23.9)	10.8 (4.0–22.1)	10.4 (5.3–18.0)	10.3 (6.2–16.0)	10.4 (5.8–17.1)
Cameroon	All age	11.4 (3.7–24.6)	11.3 (4.2–23.1)	11.1 (5.7–19.0)	10.9 (6.7–16.4)	10.9 (6.2–17.9)
Chad	20 to 24	19.4 (6.8–39.6)	19.3 (6.5–39.4)	19.2 (6.7–38.6)	19.2 (7.0–40.6)	19.2 (7.1–40.2)
Chad	25 to 29	19.7 (6.9–40.0)	19.6 (6.6–39.9)	19.5 (6.8–39.0)	19.5 (7.1–41.0)	19.5 (7.2–40.6)
Chad	30 to 34	21.0 (7.5–42.0)	20.9 (7.1–41.9)	20.8 (7.3–41.1)	20.8 (7.7–43.0)	20.7 (7.8–42.5)
Chad	35 to 39	21.0 (7.5–42.2)	21.0 (7.2–42.0)	20.9 (7.4–41.2)	20.8 (7.7–43.1)	20.8 (7.8–42.6)
Chad	40 to 44	21.6 (7.8–43.0)	21.5 (7.4–42.8)	21.4 (7.6–42.0)	21.3 (7.9–43.9)	21.3 (8.1–43.4)

Supplementary Table S10: Prevalence of female SVAC by age and location for 1990, 2000, 2010, 2020, and 2023						
**Location**	**Age Range**	**1990**	**2000**	**2010**	**2020**	**2023**
Chad	45 to 49	20.9 (7.5–42.0)	20.9 (7.1–41.9)	20.7 (7.3–41.0)	20.7 (7.6–42.9)	20.7 (7.7–42.5)
Chad	50 to 54	20.3 (7.2–41.0)	20.3 (6.9–40.9)	20.1 (7.1–40.0)	20.1 (7.4–41.9)	20.1 (7.5–41.5)
Chad	55 to 59	19.6 (6.9–39.8)	19.5 (6.6–39.7)	19.4 (6.7–38.8)	19.3 (7.0–40.7)	19.3 (7.1–40.3)
Chad	60 to 64	18.9 (6.6–38.7)	18.8 (6.3–38.6)	18.7 (6.4–37.7)	18.6 (6.7–39.5)	18.6 (6.8–39.0)
Chad	65 to 69	17.6 (6.0–36.6)	17.5 (5.8–36.5)	17.4 (5.9–35.6)	17.3 (6.2–37.4)	17.3 (6.3–36.8)
Chad	70 to 74	14.2 (4.7–30.7)	14.2 (4.5–30.6)	14.1 (4.6–29.9)	14.0 (4.8–31.5)	14.0 (4.9–31.0)
Chad	75 to 79	12.9 (4.2–28.3)	12.9 (4.0–28.1)	12.7 (4.1–27.4)	12.7 (4.3–29.0)	12.7 (4.4–28.6)
Chad	80 to 84	9.3 (2.9–21.1)	9.2 (2.8–21.0)	9.1 (2.8–20.4)	9.1 (3.0–21.7)	9.1 (3.0–21.4)
Chad	85 to 89	8.4 (2.6–19.3)	8.3 (2.5–19.2)	8.2 (2.5–18.6)	8.2 (2.6–19.8)	8.2 (2.7–19.5)
Chad	90 to 94	7.6 (2.3–17.6)	7.5 (2.2–17.5)	7.5 (2.3–17.0)	7.4 (2.4–18.1)	7.4 (2.4–17.9)
Chad	95 plus	7.5 (2.3–17.5)	7.5 (2.2–17.4)	7.4 (2.3–16.9)	7.4 (2.4–18.0)	7.4 (2.4–17.7)
Chad	Age-standardized	19.4 (6.8–39.4)	19.4 (6.5–39.3)	19.2 (6.7–38.5)	19.2 (7.0–40.3)	19.2 (7.1–39.9)
Chad	All age	19.7 (7.0–40.0)	19.8 (6.7–40.0)	19.7 (6.9–39.3)	19.8 (7.2–41.4)	19.7 (7.3–40.9)
Côte d'Ivoire	20 to 24	33.4 (13.8–58.9)	33.4 (13.2–58.8)	33.8 (13.8–58.5)	34.4 (14.8–61.2)	34.4 (15.0–60.7)
Côte d'Ivoire	25 to 29	33.1 (13.6–58.4)	33.1 (13.0–58.4)	33.4 (13.6–58.0)	34.0 (14.5–60.7)	34.0 (14.8–60.3)

Supplementary Table S10: Prevalence of female SVAC by age and location for 1990, 2000, 2010, 2020, and 2023						
**Location**	**Age Range**	**1990**	**2000**	**2010**	**2020**	**2023**
Côte d'Ivoire	30 to 34	34.2 (14.2–59.8)	34.2 (13.7–59.7)	34.6 (14.3–59.4)	35.2 (15.2–62.0)	35.1 (15.4–61.5)
Côte d'Ivoire	35 to 39	33.8 (14.0–59.3)	33.8 (13.4–59.3)	34.1 (14.0–58.9)	34.7 (15.0–61.5)	34.7 (15.2–61.1)
Côte d'Ivoire	40 to 44	34.1 (14.2–59.7)	34.1 (13.6–59.6)	34.4 (14.2–59.2)	35.0 (15.1–61.8)	35.0 (15.3–61.3)
Côte d'Ivoire	45 to 49	33.0 (13.6–58.4)	33.1 (13.0–58.4)	33.3 (13.6–58.0)	33.9 (14.5–60.6)	33.8 (14.7–60.2)
Côte d'Ivoire	50 to 54	32.1 (13.0–57.3)	32.1 (12.5–57.3)	32.4 (13.0–56.8)	32.9 (13.9–59.5)	32.9 (14.1–59.0)
Côte d'Ivoire	55 to 59	31.0 (12.4–56.0)	31.0 (11.9–55.9)	31.3 (12.4–55.5)	31.8 (13.3–58.1)	31.8 (13.5–57.7)
Côte d'Ivoire	60 to 64	30.1 (11.9–54.8)	30.1 (11.4–54.7)	30.3 (11.9–54.3)	30.8 (12.7–56.9)	30.8 (12.9–56.5)
Côte d'Ivoire	65 to 69	28.3 (11.0–52.6)	28.3 (10.5–52.5)	28.5 (11.0–52.1)	29.0 (11.7–54.7)	29.0 (11.9–54.3)
Côte d'Ivoire	70 to 74	23.6 (8.7–46.0)	23.6 (8.3–46.0)	23.8 (8.7–45.5)	24.2 (9.3–48.2)	24.2 (9.4–47.7)
Côte d'Ivoire	75 to 79	21.6 (7.8–43.1)	21.6 (7.4–43.0)	21.8 (7.8–42.6)	22.2 (8.3–45.2)	22.2 (8.4–44.7)
Côte d'Ivoire	80 to 84	16.0 (5.4–33.9)	16.0 (5.2–33.9)	16.2 (5.4–33.5)	16.5 (5.8–35.9)	16.5 (5.9–35.5)
Côte d'Ivoire	85 to 89	14.6 (4.9–31.4)	14.6 (4.6–31.4)	14.7 (4.9–31.0)	15.0 (5.2–33.3)	15.0 (5.3–32.9)
Côte d'Ivoire	90 to 94	13.3 (4.4–29.1)	13.3 (4.2–29.0)	13.4 (4.4–28.7)	13.7 (4.7–30.9)	13.7 (4.8–30.5)
Côte d'Ivoire	95 plus	13.2 (4.3–28.9)	13.2 (4.1–28.8)	13.3 (4.3–28.5)	13.6 (4.7–30.7)	13.6 (4.7–30.3)
Côte d'Ivoire	Age-standardized	31.6 (12.8–56.4)	31.6 (12.3–56.4)	31.9 (12.9–56.0)	32.4 (13.7–58.6)	32.4 (13.9–58.2)

Supplementary Table S10: Prevalence of female SVAC by age and location for 1990, 2000, 2010, 2020, and 2023						
**Location**	**Age Range**	**1990**	**2000**	**2010**	**2020**	**2023**
Côte d'Ivoire	All age	32.9 (13.5–58.2)	32.9 (12.9–58.1)	33.1 (13.5–57.6)	33.7 (14.4–60.3)	33.7 (14.6–59.9)
The Gambia	20 to 24	19.4 (6.8–39.6)	19.3 (6.5–39.4)	19.2 (6.7–38.6)	19.2 (7.0–40.6)	19.2 (7.1–40.2)
The Gambia	25 to 29	19.7 (6.9–40.0)	19.6 (6.6–39.9)	19.5 (6.8–39.0)	19.5 (7.1–41.0)	19.5 (7.2–40.6)
The Gambia	30 to 34	21.0 (7.5–42.0)	20.9 (7.1–41.9)	20.8 (7.3–41.1)	20.8 (7.7–43.0)	20.7 (7.8–42.5)
The Gambia	35 to 39	21.0 (7.5–42.2)	21.0 (7.2–42.0)	20.9 (7.4–41.2)	20.8 (7.7–43.1)	20.8 (7.8–42.6)
The Gambia	40 to 44	21.6 (7.8–43.0)	21.5 (7.4–42.8)	21.4 (7.6–42.0)	21.3 (7.9–43.9)	21.3 (8.1–43.4)
The Gambia	45 to 49	20.9 (7.5–42.0)	20.9 (7.1–41.9)	20.7 (7.3–41.0)	20.7 (7.6–42.9)	20.7 (7.7–42.5)
The Gambia	50 to 54	20.3 (7.2–41.0)	20.3 (6.9–40.9)	20.1 (7.1–40.0)	20.1 (7.4–41.9)	20.1 (7.5–41.5)
The Gambia	55 to 59	19.6 (6.9–39.8)	19.5 (6.6–39.7)	19.4 (6.7–38.8)	19.3 (7.0–40.7)	19.3 (7.1–40.3)
The Gambia	60 to 64	18.9 (6.6–38.7)	18.8 (6.3–38.6)	18.7 (6.4–37.7)	18.6 (6.7–39.5)	18.6 (6.8–39.0)
The Gambia	65 to 69	17.6 (6.0–36.6)	17.5 (5.8–36.5)	17.4 (5.9–35.6)	17.3 (6.2–37.4)	17.3 (6.3–36.8)
The Gambia	70 to 74	14.2 (4.7–30.7)	14.2 (4.5–30.6)	14.1 (4.6–29.9)	14.0 (4.8–31.5)	14.0 (4.9–31.0)
The Gambia	75 to 79	12.9 (4.2–28.3)	12.9 (4.0–28.1)	12.7 (4.1–27.4)	12.7 (4.3–29.0)	12.7 (4.4–28.6)
The Gambia	80 to 84	9.3 (2.9–21.1)	9.2 (2.8–21.0)	9.1 (2.8–20.4)	9.1 (3.0–21.7)	9.1 (3.0–21.4)
The Gambia	85 to 89	8.4 (2.6–19.3)	8.3 (2.5–19.2)	8.2 (2.5–18.6)	8.2 (2.6–19.8)	8.2 (2.7–19.5)

Supplementary Table S10: Prevalence of female SVAC by age and location for 1990, 2000, 2010, 2020, and 2023						
**Location**	**Age Range**	**1990**	**2000**	**2010**	**2020**	**2023**
The Gambia	90 to 94	7.6 (2.3–17.6)	7.5 (2.2–17.5)	7.5 (2.3–17.0)	7.4 (2.4–18.1)	7.4 (2.4–17.9)
The Gambia	95 plus	7.5 (2.3–17.5)	7.5 (2.2–17.4)	7.4 (2.3–16.9)	7.4 (2.4–18.0)	7.4 (2.4–17.7)
The Gambia	Age-standardized	19.4 (6.8–39.4)	19.4 (6.5–39.3)	19.2 (6.7–38.5)	19.2 (7.0–40.3)	19.2 (7.1–39.9)
The Gambia	All age	19.9 (7.0–40.3)	19.8 (6.7–40.1)	19.6 (6.9–39.2)	19.6 (7.2–41.2)	19.7 (7.3–40.7)
Ghana	20 to 24	19.4 (6.8–39.6)	19.3 (6.5–39.4)	19.2 (6.7–38.6)	19.2 (7.0–40.6)	19.2 (7.1–40.2)
Ghana	25 to 29	19.7 (6.9–40.0)	19.6 (6.6–39.9)	19.5 (6.8–39.0)	19.5 (7.1–41.0)	19.5 (7.2–40.6)
Ghana	30 to 34	21.0 (7.5–42.0)	20.9 (7.1–41.9)	20.8 (7.3–41.1)	20.8 (7.7–43.0)	20.7 (7.8–42.5)
Ghana	35 to 39	21.0 (7.5–42.2)	21.0 (7.2–42.0)	20.9 (7.4–41.2)	20.8 (7.7–43.1)	20.8 (7.8–42.6)
Ghana	40 to 44	21.6 (7.8–43.0)	21.5 (7.4–42.8)	21.4 (7.6–42.0)	21.3 (7.9–43.9)	21.3 (8.1–43.4)
Ghana	45 to 49	20.9 (7.5–42.0)	20.9 (7.1–41.9)	20.7 (7.3–41.0)	20.7 (7.6–42.9)	20.7 (7.7–42.5)
Ghana	50 to 54	20.3 (7.2–41.0)	20.3 (6.9–40.9)	20.1 (7.1–40.0)	20.1 (7.4–41.9)	20.1 (7.5–41.5)
Ghana	55 to 59	19.6 (6.9–39.8)	19.5 (6.6–39.7)	19.4 (6.7–38.8)	19.3 (7.0–40.7)	19.3 (7.1–40.3)
Ghana	60 to 64	18.9 (6.6–38.7)	18.8 (6.3–38.6)	18.7 (6.4–37.7)	18.6 (6.7–39.5)	18.6 (6.8–39.0)
Ghana	65 to 69	17.6 (6.0–36.6)	17.5 (5.8–36.5)	17.4 (5.9–35.6)	17.3 (6.2–37.4)	17.3 (6.3–36.8)
Ghana	70 to 74	14.2 (4.7–30.7)	14.2 (4.5–30.6)	14.1 (4.6–29.9)	14.0 (4.8–31.5)	14.0 (4.9–31.0)

Supplementary Table S10: Prevalence of female SVAC by age and location for 1990, 2000, 2010, 2020, and 2023						
**Location**	**Age Range**	**1990**	**2000**	**2010**	**2020**	**2023**
Ghana	75 to 79	12.9 (4.2–28.3)	12.9 (4.0–28.1)	12.7 (4.1–27.4)	12.7 (4.3–29.0)	12.7 (4.4–28.6)
Ghana	80 to 84	9.3 (2.9–21.1)	9.2 (2.8–21.0)	9.1 (2.8–20.4)	9.1 (3.0–21.7)	9.1 (3.0–21.4)
Ghana	85 to 89	8.4 (2.6–19.3)	8.3 (2.5–19.2)	8.2 (2.5–18.6)	8.2 (2.6–19.8)	8.2 (2.7–19.5)
Ghana	90 to 94	7.6 (2.3–17.6)	7.5 (2.2–17.5)	7.5 (2.3–17.0)	7.4 (2.4–18.1)	7.4 (2.4–17.9)
Ghana	95 plus	7.5 (2.3–17.5)	7.5 (2.2–17.4)	7.4 (2.3–16.9)	7.4 (2.4–18.0)	7.4 (2.4–17.7)
Ghana	Age-standardized	19.4 (6.8–39.4)	19.4 (6.5–39.3)	19.2 (6.7–38.5)	19.2 (7.0–40.3)	19.2 (7.1–39.9)
Ghana	All age	19.9 (7.0–40.3)	19.8 (6.7–40.2)	19.7 (6.9–39.3)	19.6 (7.2–41.2)	19.6 (7.3–40.7)
Guinea	20 to 24	19.4 (6.8–39.6)	19.3 (6.5–39.4)	19.2 (6.7–38.6)	19.2 (7.0–40.6)	19.2 (7.1–40.2)
Guinea	25 to 29	19.7 (6.9–40.0)	19.6 (6.6–39.9)	19.5 (6.8–39.0)	19.5 (7.1–41.0)	19.5 (7.2–40.6)
Guinea	30 to 34	21.0 (7.5–42.0)	20.9 (7.1–41.9)	20.8 (7.3–41.1)	20.8 (7.7–43.0)	20.7 (7.8–42.5)
Guinea	35 to 39	21.0 (7.5–42.2)	21.0 (7.2–42.0)	20.9 (7.4–41.2)	20.8 (7.7–43.1)	20.8 (7.8–42.6)
Guinea	40 to 44	21.6 (7.8–43.0)	21.5 (7.4–42.8)	21.4 (7.6–42.0)	21.3 (7.9–43.9)	21.3 (8.1–43.4)
Guinea	45 to 49	20.9 (7.5–42.0)	20.9 (7.1–41.9)	20.7 (7.3–41.0)	20.7 (7.6–42.9)	20.7 (7.7–42.5)
Guinea	50 to 54	20.3 (7.2–41.0)	20.3 (6.9–40.9)	20.1 (7.1–40.0)	20.1 (7.4–41.9)	20.1 (7.5–41.5)
Guinea	55 to 59	19.6 (6.9–39.8)	19.5 (6.6–39.7)	19.4 (6.7–38.8)	19.3 (7.0–40.7)	19.3 (7.1–40.3)

Supplementary Table S10: Prevalence of female SVAC by age and location for 1990, 2000, 2010, 2020, and 2023						
**Location**	**Age Range**	**1990**	**2000**	**2010**	**2020**	**2023**
Guinea	60 to 64	18.9 (6.6–38.7)	18.8 (6.3–38.6)	18.7 (6.4–37.7)	18.6 (6.7–39.5)	18.6 (6.8–39.0)
Guinea	65 to 69	17.6 (6.0–36.6)	17.5 (5.8–36.5)	17.4 (5.9–35.6)	17.3 (6.2–37.4)	17.3 (6.3–36.8)
Guinea	70 to 74	14.2 (4.7–30.7)	14.2 (4.5–30.6)	14.1 (4.6–29.9)	14.0 (4.8–31.5)	14.0 (4.9–31.0)
Guinea	75 to 79	12.9 (4.2–28.3)	12.9 (4.0–28.1)	12.7 (4.1–27.4)	12.7 (4.3–29.0)	12.7 (4.4–28.6)
Guinea	80 to 84	9.3 (2.9–21.1)	9.2 (2.8–21.0)	9.1 (2.8–20.4)	9.1 (3.0–21.7)	9.1 (3.0–21.4)
Guinea	85 to 89	8.4 (2.6–19.3)	8.3 (2.5–19.2)	8.2 (2.5–18.6)	8.2 (2.6–19.8)	8.2 (2.7–19.5)
Guinea	90 to 94	7.6 (2.3–17.6)	7.5 (2.2–17.5)	7.5 (2.3–17.0)	7.4 (2.4–18.1)	7.4 (2.4–17.9)
Guinea	95 plus	7.5 (2.3–17.5)	7.5 (2.2–17.4)	7.4 (2.3–16.9)	7.4 (2.4–18.0)	7.4 (2.4–17.7)
Guinea	Age-standardized	19.4 (6.8–39.4)	19.4 (6.5–39.3)	19.2 (6.7–38.5)	19.2 (7.0–40.3)	19.2 (7.1–39.9)
Guinea	All age	19.8 (7.0–40.1)	19.6 (6.6–39.7)	19.5 (6.8–39.0)	19.7 (7.2–41.2)	19.7 (7.3–40.8)
Guinea-Bissau	20 to 24	19.4 (6.8–39.6)	19.3 (6.5–39.4)	19.2 (6.7–38.6)	19.2 (7.0–40.6)	19.2 (7.1–40.2)
Guinea-Bissau	25 to 29	19.7 (6.9–40.0)	19.6 (6.6–39.9)	19.5 (6.8–39.0)	19.5 (7.1–41.0)	19.5 (7.2–40.6)
Guinea-Bissau	30 to 34	21.0 (7.5–42.0)	20.9 (7.1–41.9)	20.8 (7.3–41.1)	20.8 (7.7–43.0)	20.7 (7.8–42.5)
Guinea-Bissau	35 to 39	21.0 (7.5–42.2)	21.0 (7.2–42.0)	20.9 (7.4–41.2)	20.8 (7.7–43.1)	20.8 (7.8–42.6)
Guinea-Bissau	40 to 44	21.6 (7.8–43.0)	21.5 (7.4–42.8)	21.4 (7.6–42.0)	21.3 (7.9–43.9)	21.3 (8.1–43.4)

Supplementary Table S10: Prevalence of female SVAC by age and location for 1990, 2000, 2010, 2020, and 2023						
**Location**	**Age Range**	**1990**	**2000**	**2010**	**2020**	**2023**
Guinea-Bissau	45 to 49	20.9 (7.5–42.0)	20.9 (7.1–41.9)	20.7 (7.3–41.0)	20.7 (7.6–42.9)	20.7 (7.7–42.5)
Guinea-Bissau	50 to 54	20.3 (7.2–41.0)	20.3 (6.9–40.9)	20.1 (7.1–40.0)	20.1 (7.4–41.9)	20.1 (7.5–41.5)
Guinea-Bissau	55 to 59	19.6 (6.9–39.8)	19.5 (6.6–39.7)	19.4 (6.7–38.8)	19.3 (7.0–40.7)	19.3 (7.1–40.3)
Guinea-Bissau	60 to 64	18.9 (6.6–38.7)	18.8 (6.3–38.6)	18.7 (6.4–37.7)	18.6 (6.7–39.5)	18.6 (6.8–39.0)
Guinea-Bissau	65 to 69	17.6 (6.0–36.6)	17.5 (5.8–36.5)	17.4 (5.9–35.6)	17.3 (6.2–37.4)	17.3 (6.3–36.8)
Guinea-Bissau	70 to 74	14.2 (4.7–30.7)	14.2 (4.5–30.6)	14.1 (4.6–29.9)	14.0 (4.8–31.5)	14.0 (4.9–31.0)
Guinea-Bissau	75 to 79	12.9 (4.2–28.3)	12.9 (4.0–28.1)	12.7 (4.1–27.4)	12.7 (4.3–29.0)	12.7 (4.4–28.6)
Guinea-Bissau	80 to 84	9.3 (2.9–21.1)	9.2 (2.8–21.0)	9.1 (2.8–20.4)	9.1 (3.0–21.7)	9.1 (3.0–21.4)
Guinea-Bissau	85 to 89	8.4 (2.6–19.3)	8.3 (2.5–19.2)	8.2 (2.5–18.6)	8.2 (2.6–19.8)	8.2 (2.7–19.5)
Guinea-Bissau	90 to 94	7.6 (2.3–17.6)	7.5 (2.2–17.5)	7.5 (2.3–17.0)	7.4 (2.4–18.1)	7.4 (2.4–17.9)
Guinea-Bissau	95 plus	7.5 (2.3–17.5)	7.5 (2.2–17.4)	7.4 (2.3–16.9)	7.4 (2.4–18.0)	7.4 (2.4–17.7)
Guinea-Bissau	Age-standardized	19.4 (6.8–39.4)	19.4 (6.5–39.3)	19.2 (6.7–38.5)	19.2 (7.0–40.3)	19.2 (7.1–39.9)
Guinea-Bissau	All age	19.9 (7.1–40.4)	19.9 (6.7–40.2)	19.8 (6.9–39.4)	19.8 (7.3–41.4)	19.8 (7.4–41.0)
Liberia	20 to 24	19.4 (6.8–39.6)	19.3 (6.5–39.4)	19.2 (6.7–38.6)	19.2 (7.0–40.6)	19.2 (7.1–40.2)
Liberia	25 to 29	19.7 (6.9–40.0)	19.6 (6.6–39.9)	19.5 (6.8–39.0)	19.5 (7.1–41.0)	19.5 (7.2–40.6)

Supplementary Table S10: Prevalence of female SVAC by age and location for 1990, 2000, 2010, 2020, and 2023						
**Location**	**Age Range**	**1990**	**2000**	**2010**	**2020**	**2023**
Liberia	30 to 34	21.0 (7.5–42.0)	20.9 (7.1–41.9)	20.8 (7.3–41.1)	20.8 (7.7–43.0)	20.7 (7.8–42.5)
Liberia	35 to 39	21.0 (7.5–42.2)	21.0 (7.2–42.0)	20.9 (7.4–41.2)	20.8 (7.7–43.1)	20.8 (7.8–42.6)
Liberia	40 to 44	21.6 (7.8–43.0)	21.5 (7.4–42.8)	21.4 (7.6–42.0)	21.3 (7.9–43.9)	21.3 (8.1–43.4)
Liberia	45 to 49	20.9 (7.5–42.0)	20.9 (7.1–41.9)	20.7 (7.3–41.0)	20.7 (7.6–42.9)	20.7 (7.7–42.5)
Liberia	50 to 54	20.3 (7.2–41.0)	20.3 (6.9–40.9)	20.1 (7.1–40.0)	20.1 (7.4–41.9)	20.1 (7.5–41.5)
Liberia	55 to 59	19.6 (6.9–39.8)	19.5 (6.6–39.7)	19.4 (6.7–38.8)	19.3 (7.0–40.7)	19.3 (7.1–40.3)
Liberia	60 to 64	18.9 (6.6–38.7)	18.8 (6.3–38.6)	18.7 (6.4–37.7)	18.6 (6.7–39.5)	18.6 (6.8–39.0)
Liberia	65 to 69	17.6 (6.0–36.6)	17.5 (5.8–36.5)	17.4 (5.9–35.6)	17.3 (6.2–37.4)	17.3 (6.3–36.8)
Liberia	70 to 74	14.2 (4.7–30.7)	14.2 (4.5–30.6)	14.1 (4.6–29.9)	14.0 (4.8–31.5)	14.0 (4.9–31.0)
Liberia	75 to 79	12.9 (4.2–28.3)	12.9 (4.0–28.1)	12.7 (4.1–27.4)	12.7 (4.3–29.0)	12.7 (4.4–28.6)
Liberia	80 to 84	9.3 (2.9–21.1)	9.2 (2.8–21.0)	9.1 (2.8–20.4)	9.1 (3.0–21.7)	9.1 (3.0–21.4)
Liberia	85 to 89	8.4 (2.6–19.3)	8.3 (2.5–19.2)	8.2 (2.5–18.6)	8.2 (2.6–19.8)	8.2 (2.7–19.5)
Liberia	90 to 94	7.6 (2.3–17.6)	7.5 (2.2–17.5)	7.5 (2.3–17.0)	7.4 (2.4–18.1)	7.4 (2.4–17.9)
Liberia	95 plus	7.5 (2.3–17.5)	7.5 (2.2–17.4)	7.4 (2.3–16.9)	7.4 (2.4–18.0)	7.4 (2.4–17.7)
Liberia	Age-standardized	19.4 (6.8–39.4)	19.4 (6.5–39.3)	19.2 (6.7–38.5)	19.2 (7.0–40.3)	19.2 (7.1–39.9)

Supplementary Table S10: Prevalence of female SVAC by age and location for 1990, 2000, 2010, 2020, and 2023						
**Location**	**Age Range**	**1990**	**2000**	**2010**	**2020**	**2023**
Liberia	All age	19.8 (7.0–40.2)	19.8 (6.7–40.0)	19.7 (6.9–39.4)	19.8 (7.3–41.4)	19.8 (7.4–41.0)
Mali	20 to 24	19.4 (6.8–39.6)	19.3 (6.5–39.4)	19.2 (6.7–38.6)	19.2 (7.0–40.6)	19.2 (7.1–40.2)
Mali	25 to 29	19.7 (6.9–40.0)	19.6 (6.6–39.9)	19.5 (6.8–39.0)	19.5 (7.1–41.0)	19.5 (7.2–40.6)
Mali	30 to 34	21.0 (7.5–42.0)	20.9 (7.1–41.9)	20.8 (7.3–41.1)	20.8 (7.7–43.0)	20.7 (7.8–42.5)
Mali	35 to 39	21.0 (7.5–42.2)	21.0 (7.2–42.0)	20.9 (7.4–41.2)	20.8 (7.7–43.1)	20.8 (7.8–42.6)
Mali	40 to 44	21.6 (7.8–43.0)	21.5 (7.4–42.8)	21.4 (7.6–42.0)	21.3 (7.9–43.9)	21.3 (8.1–43.4)
Mali	45 to 49	20.9 (7.5–42.0)	20.9 (7.1–41.9)	20.7 (7.3–41.0)	20.7 (7.6–42.9)	20.7 (7.7–42.5)
Mali	50 to 54	20.3 (7.2–41.0)	20.3 (6.9–40.9)	20.1 (7.1–40.0)	20.1 (7.4–41.9)	20.1 (7.5–41.5)
Mali	55 to 59	19.6 (6.9–39.8)	19.5 (6.6–39.7)	19.4 (6.7–38.8)	19.3 (7.0–40.7)	19.3 (7.1–40.3)
Mali	60 to 64	18.9 (6.6–38.7)	18.8 (6.3–38.6)	18.7 (6.4–37.7)	18.6 (6.7–39.5)	18.6 (6.8–39.0)
Mali	65 to 69	17.6 (6.0–36.6)	17.5 (5.8–36.5)	17.4 (5.9–35.6)	17.3 (6.2–37.4)	17.3 (6.3–36.8)
Mali	70 to 74	14.2 (4.7–30.7)	14.2 (4.5–30.6)	14.1 (4.6–29.9)	14.0 (4.8–31.5)	14.0 (4.9–31.0)
Mali	75 to 79	12.9 (4.2–28.3)	12.9 (4.0–28.1)	12.7 (4.1–27.4)	12.7 (4.3–29.0)	12.7 (4.4–28.6)
Mali	80 to 84	9.3 (2.9–21.1)	9.2 (2.8–21.0)	9.1 (2.8–20.4)	9.1 (3.0–21.7)	9.1 (3.0–21.4)
Mali	85 to 89	8.4 (2.6–19.3)	8.3 (2.5–19.2)	8.2 (2.5–18.6)	8.2 (2.6–19.8)	8.2 (2.7–19.5)

Supplementary Table S10: Prevalence of female SVAC by age and location for 1990, 2000, 2010, 2020, and 2023						
**Location**	**Age Range**	**1990**	**2000**	**2010**	**2020**	**2023**
Mali	90 to 94	7.6 (2.3–17.6)	7.5 (2.2–17.5)	7.5 (2.3–17.0)	7.4 (2.4–18.1)	7.4 (2.4–17.9)
Mali	95 plus	7.5 (2.3–17.5)	7.5 (2.2–17.4)	7.4 (2.3–16.9)	7.4 (2.4–18.0)	7.4 (2.4–17.7)
Mali	Age-standardized	19.4 (6.8–39.4)	19.4 (6.5–39.3)	19.2 (6.7–38.5)	19.2 (7.0–40.3)	19.2 (7.1–39.9)
Mali	All age	19.9 (7.0–40.3)	19.9 (6.7–40.2)	19.7 (6.9–39.3)	19.7 (7.2–41.2)	19.7 (7.3–40.8)
Mauritania	20 to 24	19.4 (6.8–39.6)	19.3 (6.5–39.4)	19.2 (6.7–38.6)	19.2 (7.0–40.6)	19.2 (7.1–40.2)
Mauritania	25 to 29	19.7 (6.9–40.0)	19.6 (6.6–39.9)	19.5 (6.8–39.0)	19.5 (7.1–41.0)	19.5 (7.2–40.6)
Mauritania	30 to 34	21.0 (7.5–42.0)	20.9 (7.1–41.9)	20.8 (7.3–41.1)	20.8 (7.7–43.0)	20.7 (7.8–42.5)
Mauritania	35 to 39	21.0 (7.5–42.2)	21.0 (7.2–42.0)	20.9 (7.4–41.2)	20.8 (7.7–43.1)	20.8 (7.8–42.6)
Mauritania	40 to 44	21.6 (7.8–43.0)	21.5 (7.4–42.8)	21.4 (7.6–42.0)	21.3 (7.9–43.9)	21.3 (8.1–43.4)
Mauritania	45 to 49	20.9 (7.5–42.0)	20.9 (7.1–41.9)	20.7 (7.3–41.0)	20.7 (7.6–42.9)	20.7 (7.7–42.5)
Mauritania	50 to 54	20.3 (7.2–41.0)	20.3 (6.9–40.9)	20.1 (7.1–40.0)	20.1 (7.4–41.9)	20.1 (7.5–41.5)
Mauritania	55 to 59	19.6 (6.9–39.8)	19.5 (6.6–39.7)	19.4 (6.7–38.8)	19.3 (7.0–40.7)	19.3 (7.1–40.3)
Mauritania	60 to 64	18.9 (6.6–38.7)	18.8 (6.3–38.6)	18.7 (6.4–37.7)	18.6 (6.7–39.5)	18.6 (6.8–39.0)
Mauritania	65 to 69	17.6 (6.0–36.6)	17.5 (5.8–36.5)	17.4 (5.9–35.6)	17.3 (6.2–37.4)	17.3 (6.3–36.8)
Mauritania	70 to 74	14.2 (4.7–30.7)	14.2 (4.5–30.6)	14.1 (4.6–29.9)	14.0 (4.8–31.5)	14.0 (4.9–31.0)

Supplementary Table S10: Prevalence of female SVAC by age and location for 1990, 2000, 2010, 2020, and 2023						
**Location**	**Age Range**	**1990**	**2000**	**2010**	**2020**	**2023**
Mauritania	75 to 79	12.9 (4.2–28.3)	12.9 (4.0–28.1)	12.7 (4.1–27.4)	12.7 (4.3–29.0)	12.7 (4.4–28.6)
Mauritania	80 to 84	9.3 (2.9–21.1)	9.2 (2.8–21.0)	9.1 (2.8–20.4)	9.1 (3.0–21.7)	9.1 (3.0–21.4)
Mauritania	85 to 89	8.4 (2.6–19.3)	8.3 (2.5–19.2)	8.2 (2.5–18.6)	8.2 (2.6–19.8)	8.2 (2.7–19.5)
Mauritania	90 to 94	7.6 (2.3–17.6)	7.5 (2.2–17.5)	7.5 (2.3–17.0)	7.4 (2.4–18.1)	7.4 (2.4–17.9)
Mauritania	95 plus	7.5 (2.3–17.5)	7.5 (2.2–17.4)	7.4 (2.3–16.9)	7.4 (2.4–18.0)	7.4 (2.4–17.7)
Mauritania	Age-standardized	19.4 (6.8–39.4)	19.4 (6.5–39.3)	19.2 (6.7–38.5)	19.2 (7.0–40.3)	19.2 (7.1–39.9)
Mauritania	All age	19.7 (7.0–40.0)	19.8 (6.7–40.0)	19.7 (6.9–39.2)	19.6 (7.2–41.1)	19.6 (7.3–40.6)
Niger	20 to 24	19.7 (6.9–40.0)	19.6 (6.6–39.9)	19.6 (6.8–39.2)	19.7 (7.2–41.3)	19.7 (7.3–40.9)
Niger	25 to 29	20.0 (7.1–40.5)	20.0 (6.7–40.4)	20.0 (7.0–39.8)	20.0 (7.3–41.9)	20.0 (7.5–41.4)
Niger	30 to 34	21.4 (7.7–42.7)	21.3 (7.3–42.6)	21.3 (7.6–41.9)	21.4 (8.0–44.0)	21.4 (8.0–43.5)
Niger	35 to 39	21.5 (7.7–42.9)	21.5 (7.4–42.8)	21.5 (7.6–42.1)	21.5 (8.0–44.2)	21.5 (8.1–43.7)
Niger	40 to 44	22.1 (8.0–43.8)	22.1 (7.6–43.7)	22.0 (7.9–43.0)	22.1 (8.3–45.1)	22.1 (8.4–44.6)
Niger	45 to 49	21.5 (7.7–42.9)	21.5 (7.4–42.8)	21.5 (7.6–42.1)	21.5 (8.0–44.2)	21.5 (8.1–43.7)
Niger	50 to 54	21.0 (7.5–42.1)	21.0 (7.2–42.0)	20.9 (7.4–41.3)	21.0 (7.8–43.3)	21.0 (7.9–42.9)
Niger	55 to 59	20.3 (7.2–41.0)	20.2 (6.9–40.9)	20.2 (7.1–40.2)	20.2 (7.4–42.2)	20.2 (7.6–41.8)

Supplementary Table S10: Prevalence of female SVAC by age and location for 1990, 2000, 2010, 2020, and 2023						
**Location**	**Age Range**	**1990**	**2000**	**2010**	**2020**	**2023**
Niger	60 to 64	19.6 (6.9–39.9)	19.6 (6.6–39.8)	19.6 (6.8–39.1)	19.6 (7.2–41.2)	19.6 (7.3–40.7)
Niger	65 to 69	18.3 (6.4–37.8)	18.3 (6.1–37.7)	18.3 (6.3–37.0)	18.3 (6.6–39.0)	18.3 (6.6–38.6)
Niger	70 to 74	14.9 (5.0–31.9)	14.9 (4.7–31.8)	14.8 (4.9–31.2)	14.8 (5.1–33.0)	14.8 (5.2–32.6)
Niger	75 to 79	13.5 (4.4–29.4)	13.5 (4.2–29.3)	13.4 (4.4–28.7)	13.4 (4.6–30.4)	13.4 (4.7–30.0)
Niger	80 to 84	9.7 (3.1–22.0)	9.7 (2.9–21.9)	9.6 (3.0–21.4)	9.7 (3.2–22.9)	9.7 (3.2–22.6)
Niger	85 to 89	8.8 (2.7–20.1)	8.7 (2.6–20.0)	8.7 (2.7–19.6)	8.7 (2.8–20.9)	8.7 (2.9–20.6)
Niger	90 to 94	7.9 (2.5–18.4)	7.9 (2.3–18.3)	7.9 (2.4–17.9)	7.9 (2.5–19.2)	7.9 (2.6–18.9)
Niger	95 plus	7.9 (2.4–18.3)	7.9 (2.3–18.2)	7.8 (2.4–17.8)	7.8 (2.5–19.0)	7.8 (2.6–18.7)
Niger	Age-standardized	19.9 (7.1–40.3)	19.9 (6.7–40.2)	19.9 (7.0–39.5)	19.9 (7.3–41.5)	19.9 (7.4–41.1)
Niger	All age	20.4 (7.3–41.2)	20.4 (6.9–41.1)	20.4 (7.2–40.4)	20.4 (7.5–42.4)	20.4 (7.6–42.0)
Nigeria	20 to 24	27.8 (15.1–44.8)	28.4 (19.1–40.0)	27.7 (20.0–36.2)	25.9 (16.4–37.8)	25.6 (14.5–40.0)
Nigeria	25 to 29	27.7 (13.7–46.8)	27.4 (17.0–39.7)	26.3 (17.6–36.3)	27.2 (19.3–36.2)	27.6 (18.3–39.1)
Nigeria	30 to 34	28.3 (12.9–48.5)	27.0 (15.9–40.2)	23.9 (15.9–33.4)	22.3 (12.4–34.8)	22.4 (10.8–37.1)
Nigeria	35 to 39	28.0 (12.7–49.0)	27.2 (16.0–40.2)	25.9 (17.7–35.5)	25.2 (15.0–37.9)	25.2 (13.9–40.3)
Nigeria	40 to 44	27.0 (12.1–47.8)	25.8 (15.0–39.2)	25.1 (16.5–35.4)	25.7 (16.0–37.7)	26.0 (14.7–40.8)

Supplementary Table S10: Prevalence of female SVAC by age and location for 1990, 2000, 2010, 2020, and 2023						
**Location**	**Age Range**	**1990**	**2000**	**2010**	**2020**	**2023**
Nigeria	45 to 49	29.0 (12.9–50.5)	28.1 (15.8–43.2)	25.7 (16.7–36.3)	24.6 (14.4–37.0)	24.6 (12.9–39.1)
Nigeria	50 to 54	28.4 (12.6–49.6)	27.8 (15.6–42.4)	25.8 (16.8–36.3)	24.7 (13.9–38.0)	24.7 (12.8–40.1)
Nigeria	55 to 59	24.9 (9.1–47.8)	23.4 (9.9–42.3)	21.4 (11.6–34.2)	21.4 (11.4–34.6)	21.8 (10.4–36.5)
Nigeria	60 to 64	24.4 (8.8–46.9)	23.0 (9.7–41.7)	21.2 (11.5–33.9)	21.2 (11.2–34.3)	21.5 (10.3–36.1)
Nigeria	65 to 69	24.1 (8.7–46.9)	24.0 (8.5–46.6)	23.9 (8.8–45.7)	24.0 (9.2–48.0)	24.1 (9.2–48.8)
Nigeria	70 to 74	19.9 (7.1–40.4)	19.8 (6.7–40.1)	19.7 (6.9–39.3)	19.8 (7.2–41.5)	19.9 (7.2–42.3)
Nigeria	75 to 79	18.1 (6.3–37.6)	18.0 (6.0–37.3)	18.0 (6.1–36.5)	18.0 (6.4–38.6)	18.1 (6.4–39.4)
Nigeria	80 to 84	13.3 (4.4–29.0)	13.2 (4.1–28.8)	13.1 (4.3–28.1)	13.2 (4.5–29.9)	13.3 (4.5–30.6)
Nigeria	85 to 89	12.1 (3.9–26.7)	12.0 (3.7–26.5)	11.9 (3.8–25.8)	12.0 (4.0–27.7)	12.0 (4.0–28.2)
Nigeria	90 to 94	11.0 (3.5–24.6)	10.9 (3.3–24.4)	10.8 (3.4–23.8)	10.9 (3.6–25.6)	11.0 (3.6–26.1)
Nigeria	95 plus	10.9 (3.5–24.4)	10.8 (3.3–24.2)	10.7 (3.4–23.6)	10.8 (3.6–25.4)	10.9 (3.5–25.9)
Nigeria	Age-standardized	26.4 (12.1–44.9)	25.7 (14.6–39.3)	24.3 (16.4–33.8)	23.8 (14.2–36.0)	23.9 (13.3–37.7)
Nigeria	All age	26.9 (12.8–45.5)	26.6 (16.1–39.5)	25.3 (17.5–33.9)	24.6 (15.1–36.5)	24.7 (14.2–37.7)
São Tomé and Príncipe	20 to 24	15.8 (8.3–26.4)	15.5 (10.1–22.2)	16.6 (11.2–23.1)	18.3 (12.4–25.3)	18.6 (11.6–28.4)
São Tomé and Príncipe	25 to 29	16.4 (7.3–30.0)	15.8 (9.5–23.7)	15.3 (10.1–21.7)	15.0 (9.6–22.0)	15.0 (9.0–24.1)

Supplementary Table S10: Prevalence of female SVAC by age and location for 1990, 2000, 2010, 2020, and 2023						
**Location**	**Age Range**	**1990**	**2000**	**2010**	**2020**	**2023**
São Tomé and Príncipe	30 to 34	17.1 (6.6–33.2)	16.4 (8.7–26.9)	15.9 (10.3–22.7)	16.2 (10.6–23.2)	16.4 (9.8–25.5)
São Tomé and Príncipe	35 to 39	17.4 (6.4–35.2)	16.9 (7.7–30.6)	16.1 (9.9–24.5)	16.0 (10.4–22.9)	16.1 (9.6–25.2)
São Tomé and Príncipe	40 to 44	17.8 (6.2–36.4)	17.2 (6.9–32.9)	15.8 (8.5–26.5)	14.3 (8.8–21.6)	14.3 (8.2–23.4)
São Tomé and Príncipe	45 to 49	17.7 (6.2–36.1)	17.9 (6.8–34.8)	17.9 (8.9–31.4)	17.7 (11.4–26.2)	17.7 (10.5–28.0)
São Tomé and Príncipe	50 to 54	16.8 (6.0–35.1)	16.5 (6.1–32.8)	15.9 (7.4–28.5)	15.5 (8.7–24.8)	15.6 (8.3–25.5)
São Tomé and Príncipe	55 to 59	16.0 (5.6–33.7)	15.5 (5.6–31.1)	14.7 (6.8–26.5)	14.2 (7.9–22.9)	14.3 (7.6–23.6)
São Tomé and Príncipe	60 to 64	15.5 (5.4–32.9)	15.2 (5.4–30.6)	14.6 (6.5–26.8)	14.2 (7.6–23.9)	14.3 (7.4–24.3)
São Tomé and Príncipe	65 to 69	13.9 (4.6–29.5)	13.2 (4.3–28.8)	12.2 (4.4–25.5)	11.8 (4.7–24.8)	11.9 (4.6–24.7)
São Tomé and Príncipe	70 to 74	11.7 (3.8–25.9)	11.6 (3.6–25.8)	11.6 (3.7–25.3)	11.6 (3.9–26.9)	11.6 (4.0–26.5)
São Tomé and Príncipe	75 to 79	10.5 (3.3–23.7)	10.5 (3.2–23.6)	10.5 (3.3–23.1)	10.5 (3.5–24.6)	10.5 (3.5–24.3)
São Tomé and Príncipe	80 to 84	7.5 (2.3–17.4)	7.4 (2.2–17.3)	7.4 (2.3–16.9)	7.4 (2.4–18.1)	7.4 (2.4–17.9)
São Tomé and Príncipe	85 to 89	6.7 (2.1–15.8)	6.7 (2.0–15.7)	6.7 (2.0–15.4)	6.7 (2.1–16.5)	6.7 (2.2–16.3)
São Tomé and Príncipe	90 to 94	6.1 (1.8–14.4)	6.1 (1.8–14.3)	6.0 (1.8–14.0)	6.1 (1.9–15.0)	6.1 (1.9–14.8)
São Tomé and Príncipe	95 plus	6.0 (1.8–14.3)	6.0 (1.7–14.2)	6.0 (1.8–13.9)	6.0 (1.9–14.9)	6.0 (1.9–14.7)
São Tomé and Príncipe	Age-standardized	16.0 (6.3–31.4)	15.6 (7.5–27.7)	15.2 (9.0–23.9)	15.1 (9.5–22.9)	15.2 (9.2–23.9)

Supplementary Table S10: Prevalence of female SVAC by age and location for 1990, 2000, 2010, 2020, and 2023						
**Location**	**Age Range**	**1990**	**2000**	**2010**	**2020**	**2023**
São Tomé and Príncipe	All age	16.1 (6.6–30.5)	15.7 (8.1–27.0)	15.6 (9.7–23.6)	15.7 (10.0–23.3)	15.7 (9.6–24.4)
Senegal	20 to 24	19.4 (6.8–39.6)	19.3 (6.5–39.4)	19.2 (6.7–38.6)	19.2 (7.0–40.6)	19.2 (7.1–40.2)
Senegal	25 to 29	19.7 (6.9–40.0)	19.6 (6.6–39.9)	19.5 (6.8–39.0)	19.5 (7.1–41.0)	19.5 (7.2–40.6)
Senegal	30 to 34	21.0 (7.5–42.0)	20.9 (7.1–41.9)	20.8 (7.3–41.1)	20.8 (7.7–43.0)	20.7 (7.8–42.5)
Senegal	35 to 39	21.0 (7.5–42.2)	21.0 (7.2–42.0)	20.9 (7.4–41.2)	20.8 (7.7–43.1)	20.8 (7.8–42.6)
Senegal	40 to 44	21.6 (7.8–43.0)	21.5 (7.4–42.8)	21.4 (7.6–42.0)	21.3 (7.9–43.9)	21.3 (8.1–43.4)
Senegal	45 to 49	20.9 (7.5–42.0)	20.9 (7.1–41.9)	20.7 (7.3–41.0)	20.7 (7.6–42.9)	20.7 (7.7–42.5)
Senegal	50 to 54	20.3 (7.2–41.0)	20.3 (6.9–40.9)	20.1 (7.1–40.0)	20.1 (7.4–41.9)	20.1 (7.5–41.5)
Senegal	55 to 59	19.6 (6.9–39.8)	19.5 (6.6–39.7)	19.4 (6.7–38.8)	19.3 (7.0–40.7)	19.3 (7.1–40.3)
Senegal	60 to 64	18.9 (6.6–38.7)	18.8 (6.3–38.6)	18.7 (6.4–37.7)	18.6 (6.7–39.5)	18.6 (6.8–39.0)
Senegal	65 to 69	17.6 (6.0–36.6)	17.5 (5.8–36.5)	17.4 (5.9–35.6)	17.3 (6.2–37.4)	17.3 (6.3–36.8)
Senegal	70 to 74	14.2 (4.7–30.7)	14.2 (4.5–30.6)	14.1 (4.6–29.9)	14.0 (4.8–31.5)	14.0 (4.9–31.0)
Senegal	75 to 79	12.9 (4.2–28.3)	12.9 (4.0–28.1)	12.7 (4.1–27.4)	12.7 (4.3–29.0)	12.7 (4.4–28.6)
Senegal	80 to 84	9.3 (2.9–21.1)	9.2 (2.8–21.0)	9.1 (2.8–20.4)	9.1 (3.0–21.7)	9.1 (3.0–21.4)
Senegal	85 to 89	8.4 (2.6–19.3)	8.3 (2.5–19.2)	8.2 (2.5–18.6)	8.2 (2.6–19.8)	8.2 (2.7–19.5)

Supplementary Table S10: Prevalence of female SVAC by age and location for 1990, 2000, 2010, 2020, and 2023						
**Location**	**Age Range**	**1990**	**2000**	**2010**	**2020**	**2023**
Senegal	90 to 94	7.6 (2.3–17.6)	7.5 (2.2–17.5)	7.5 (2.3–17.0)	7.4 (2.4–18.1)	7.4 (2.4–17.9)
Senegal	95 plus	7.5 (2.3–17.5)	7.5 (2.2–17.4)	7.4 (2.3–16.9)	7.4 (2.4–18.0)	7.4 (2.4–17.7)
Senegal	Age-standardized	19.4 (6.8–39.4)	19.4 (6.5–39.3)	19.2 (6.7–38.5)	19.2 (7.0–40.3)	19.2 (7.1–39.9)
Senegal	All age	19.8 (7.0–40.2)	19.8 (6.7–40.1)	19.6 (6.9–39.2)	19.6 (7.2–41.1)	19.6 (7.3–40.7)
Sierra Leone	20 to 24	19.4 (6.8–39.6)	19.3 (6.5–39.4)	19.2 (6.7–38.6)	19.2 (7.0–40.6)	19.2 (7.1–40.2)
Sierra Leone	25 to 29	19.7 (6.9–40.0)	19.6 (6.6–39.9)	19.5 (6.8–39.0)	19.5 (7.1–41.0)	19.5 (7.2–40.6)
Sierra Leone	30 to 34	21.0 (7.5–42.0)	20.9 (7.1–41.9)	20.8 (7.3–41.1)	20.8 (7.7–43.0)	20.7 (7.8–42.5)
Sierra Leone	35 to 39	21.0 (7.5–42.2)	21.0 (7.2–42.0)	20.9 (7.4–41.2)	20.8 (7.7–43.1)	20.8 (7.8–42.6)
Sierra Leone	40 to 44	21.6 (7.8–43.0)	21.5 (7.4–42.8)	21.4 (7.6–42.0)	21.3 (7.9–43.9)	21.3 (8.1–43.4)
Sierra Leone	45 to 49	20.9 (7.5–42.0)	20.9 (7.1–41.9)	20.7 (7.3–41.0)	20.7 (7.6–42.9)	20.7 (7.7–42.5)
Sierra Leone	50 to 54	20.3 (7.2–41.0)	20.3 (6.9–40.9)	20.1 (7.1–40.0)	20.1 (7.4–41.9)	20.1 (7.5–41.5)
Sierra Leone	55 to 59	19.6 (6.9–39.8)	19.5 (6.6–39.7)	19.4 (6.7–38.8)	19.3 (7.0–40.7)	19.3 (7.1–40.3)
Sierra Leone	60 to 64	18.9 (6.6–38.7)	18.8 (6.3–38.6)	18.7 (6.4–37.7)	18.6 (6.7–39.5)	18.6 (6.8–39.0)
Sierra Leone	65 to 69	17.6 (6.0–36.6)	17.5 (5.8–36.5)	17.4 (5.9–35.6)	17.3 (6.2–37.4)	17.3 (6.3–36.8)
Sierra Leone	70 to 74	14.2 (4.7–30.7)	14.2 (4.5–30.6)	14.1 (4.6–29.9)	14.0 (4.8–31.5)	14.0 (4.9–31.0)

Supplementary Table S10: Prevalence of female SVAC by age and location for 1990, 2000, 2010, 2020, and 2023						
**Location**	**Age Range**	**1990**	**2000**	**2010**	**2020**	**2023**
Sierra Leone	75 to 79	12.9 (4.2–28.3)	12.9 (4.0–28.1)	12.7 (4.1–27.4)	12.7 (4.3–29.0)	12.7 (4.4–28.6)
Sierra Leone	80 to 84	9.3 (2.9–21.1)	9.2 (2.8–21.0)	9.1 (2.8–20.4)	9.1 (3.0–21.7)	9.1 (3.0–21.4)
Sierra Leone	85 to 89	8.4 (2.6–19.3)	8.3 (2.5–19.2)	8.2 (2.5–18.6)	8.2 (2.6–19.8)	8.2 (2.7–19.5)
Sierra Leone	90 to 94	7.6 (2.3–17.6)	7.5 (2.2–17.5)	7.5 (2.3–17.0)	7.4 (2.4–18.1)	7.4 (2.4–17.9)
Sierra Leone	95 plus	7.5 (2.3–17.5)	7.5 (2.2–17.4)	7.4 (2.3–16.9)	7.4 (2.4–18.0)	7.4 (2.4–17.7)
Sierra Leone	Age-standardized	19.4 (6.8–39.4)	19.4 (6.5–39.3)	19.2 (6.7–38.5)	19.2 (7.0–40.3)	19.2 (7.1–39.9)
Sierra Leone	All age	19.8 (7.0–40.1)	19.7 (6.7–39.9)	19.6 (6.8–39.1)	19.7 (7.2–41.3)	19.7 (7.3–40.9)
Togo	20 to 24	19.4 (6.8–39.6)	19.3 (6.5–39.4)	19.2 (6.7–38.6)	19.2 (7.0–40.6)	19.2 (7.1–40.2)
Togo	25 to 29	19.7 (6.9–40.0)	19.6 (6.6–39.9)	19.5 (6.8–39.0)	19.5 (7.1–41.0)	19.5 (7.2–40.6)
Togo	30 to 34	21.0 (7.5–42.0)	20.9 (7.1–41.9)	20.8 (7.3–41.1)	20.8 (7.7–43.0)	20.7 (7.8–42.5)
Togo	35 to 39	21.0 (7.5–42.2)	21.0 (7.2–42.0)	20.9 (7.4–41.2)	20.8 (7.7–43.1)	20.8 (7.8–42.6)
Togo	40 to 44	21.6 (7.8–43.0)	21.5 (7.4–42.8)	21.4 (7.6–42.0)	21.3 (7.9–43.9)	21.3 (8.1–43.4)
Togo	45 to 49	20.9 (7.5–42.0)	20.9 (7.1–41.9)	20.7 (7.3–41.0)	20.7 (7.6–42.9)	20.7 (7.7–42.5)
Togo	50 to 54	20.3 (7.2–41.0)	20.3 (6.9–40.9)	20.1 (7.1–40.0)	20.1 (7.4–41.9)	20.1 (7.5–41.5)
Togo	55 to 59	19.6 (6.9–39.8)	19.5 (6.6–39.7)	19.4 (6.7–38.8)	19.3 (7.0–40.7)	19.3 (7.1–40.3)

Supplementary Table S10: Prevalence of female SVAC by age and location for 1990, 2000, 2010, 2020, and 2023						
**Location**	**Age Range**	**1990**	**2000**	**2010**	**2020**	**2023**
Togo	60 to 64	18.9 (6.6–38.7)	18.8 (6.3–38.6)	18.7 (6.4–37.7)	18.6 (6.7–39.5)	18.6 (6.8–39.0)
Togo	65 to 69	17.6 (6.0–36.6)	17.5 (5.8–36.5)	17.4 (5.9–35.6)	17.3 (6.2–37.4)	17.3 (6.3–36.8)
Togo	70 to 74	14.2 (4.7–30.7)	14.2 (4.5–30.6)	14.1 (4.6–29.9)	14.0 (4.8–31.5)	14.0 (4.9–31.0)
Togo	75 to 79	12.9 (4.2–28.3)	12.9 (4.0–28.1)	12.7 (4.1–27.4)	12.7 (4.3–29.0)	12.7 (4.4–28.6)
Togo	80 to 84	9.3 (2.9–21.1)	9.2 (2.8–21.0)	9.1 (2.8–20.4)	9.1 (3.0–21.7)	9.1 (3.0–21.4)
Togo	85 to 89	8.4 (2.6–19.3)	8.3 (2.5–19.2)	8.2 (2.5–18.6)	8.2 (2.6–19.8)	8.2 (2.7–19.5)
Togo	90 to 94	7.6 (2.3–17.6)	7.5 (2.2–17.5)	7.5 (2.3–17.0)	7.4 (2.4–18.1)	7.4 (2.4–17.9)
Togo	95 plus	7.5 (2.3–17.5)	7.5 (2.2–17.4)	7.4 (2.3–16.9)	7.4 (2.4–18.0)	7.4 (2.4–17.7)
Togo	Age-standardized	19.4 (6.8–39.4)	19.4 (6.5–39.3)	19.2 (6.7–38.5)	19.2 (7.0–40.3)	19.2 (7.1–39.9)
Togo	All age	20.0 (7.1–40.4)	19.9 (6.7–40.3)	19.8 (6.9–39.4)	19.7 (7.2–41.3)	19.7 (7.3–40.8)

Supplementary Table S11: Prevalence of male SVAC by age and location for 1990, 2000, 2010, 2020, and 2023						
**Location**	**Age Range**	**1990**	**2000**	**2010**	**2020**	**2023**
Global	20 to 24	15.7 (8.8–25.9)	14.8 (8.8–23.9)	13.1 (8.8–19.7)	12.9 (8.1–21.0)	13.1 (7.8–21.9)
Global	25 to 29	14.7 (7.6–25.2)	14.5 (8.2–24.0)	13.6 (8.7–20.8)	13.3 (8.7–21.4)	13.4 (8.3–22.2)
Global	30 to 34	17.1 (9.2–28.7)	17.5 (10.7–26.9)	16.4 (11.1–23.8)	16.0 (11.7–23.5)	16.1 (11.3–24.0)
Global	35 to 39	15.9 (8.6–26.7)	16.4 (9.7–25.7)	15.9 (10.7–22.9)	16.1 (11.0–24.3)	16.2 (11.1–24.5)
Global	40 to 44	15.6 (8.4–26.4)	16.7 (9.5–27.0)	18.1 (12.1–26.2)	18.6 (11.9–28.6)	18.7 (11.3–29.3)
Global	45 to 49	14.9 (7.8–25.3)	15.2 (8.6–24.6)	16.5 (11.4–23.5)	17.2 (13.0–24.4)	17.3 (12.5–25.2)
Global	50 to 54	14.1 (6.8–25.2)	14.2 (8.1–23.6)	15.0 (10.2–23.0)	15.7 (10.7–24.3)	15.9 (10.0–25.4)
Global	55 to 59	14.3 (7.1–25.3)	14.3 (8.0–23.8)	14.3 (9.9–21.6)	14.8 (10.0–23.0)	14.8 (9.5–23.2)
Global	60 to 64	13.9 (6.7–25.1)	13.6 (7.0–23.7)	13.7 (8.9–21.4)	14.2 (9.6–22.9)	14.3 (9.1–22.9)
Global	65 to 69	13.2 (6.1–24.6)	12.9 (6.0–23.9)	13.0 (6.7–22.6)	13.0 (6.8–23.5)	13.2 (6.9–24.4)
Global	70 to 74	10.7 (4.8–20.8)	10.7 (4.7–20.6)	10.8 (5.1–19.6)	11.0 (5.8–20.1)	11.0 (5.9–20.2)
Global	75 to 79	9.5 (3.9–18.9)	9.5 (4.0–18.8)	9.7 (4.4–18.2)	10.0 (4.9–18.8)	10.0 (5.0–18.6)
Global	80 to 84	7.0 (2.7–14.2)	7.1 (3.0–14.4)	7.3 (3.2–14.0)	7.4 (3.3–15.2)	7.4 (3.4–14.9)
Global	85 to 89	6.4 (2.5–13.2)	6.6 (2.7–13.3)	6.8 (3.2–12.8)	6.7 (3.1–13.8)	6.7 (3.1–13.6)
Global	90 to 94	6.0 (2.4–12.5)	6.2 (2.5–12.7)	6.4 (3.2–11.8)	6.1 (3.2–12.1)	6.0 (3.2–12.0)

Supplementary Table S11: Prevalence of male SVAC by age and location for 1990, 2000, 2010, 2020, and 2023						
**Location**	**Age Range**	**1990**	**2000**	**2010**	**2020**	**2023**
Global	95 plus	6.3 (2.5–12.8)	6.5 (2.6–13.1)	6.5 (3.3–12.0)	6.3 (3.7–12.0)	6.1 (3.4–11.9)
Global	Age-standardized	14.6 (7.7–25.0)	14.7 (8.3–24.2)	14.5 (9.5–22.0)	14.7 (9.8–22.8)	14.8 (9.5–23.5)
Global	All age	15.0 (7.9–25.4)	15.0 (8.6–24.6)	14.7 (9.7–22.2)	14.8 (9.9–23.0)	14.9 (9.6–23.6)
Central Europe, eastern Europe, and central Asia	20 to 24	12.0 (4.9–23.6)	12.0 (4.8–23.7)	11.6 (4.9–22.4)	11.4 (4.9–23.0)	11.4 (4.9–22.9)
Central Europe, eastern Europe, and central Asia	25 to 29	12.3 (5.0–24.1)	12.2 (4.9–24.2)	12.1 (5.0–23.4)	11.6 (5.1–23.6)	11.6 (5.0–23.4)
Central Europe, eastern Europe, and central Asia	30 to 34	13.5 (5.5–26.3)	13.2 (5.2–26.0)	13.2 (5.6–25.3)	12.9 (5.7–25.9)	12.8 (5.6–25.3)
Central Europe, eastern Europe, and central Asia	35 to 39	13.7 (5.5–26.7)	13.4 (5.2–26.3)	13.4 (5.4–25.8)	13.3 (5.5–27.0)	13.2 (5.5–26.5)
Central Europe, eastern Europe, and central Asia	40 to 44	14.4 (5.8–27.6)	14.1 (5.4–27.7)	13.9 (5.6–26.6)	14.0 (5.9–27.9)	13.9 (6.0–27.6)
Central Europe, eastern Europe, and central Asia	45 to 49	13.9 (5.6–26.8)	13.9 (5.4–27.2)	13.6 (5.5–25.9)	13.6 (5.8–27.3)	13.6 (5.9–27.1)
Central Europe, eastern Europe, and central Asia	50 to 54	13.5 (5.4–26.3)	13.7 (5.3–27.0)	13.5 (5.4–25.9)	13.3 (5.6–26.9)	13.3 (5.7–26.6)
Central Europe, eastern Europe, and central Asia	55 to 59	13.2 (5.3–25.8)	13.2 (5.1–26.2)	13.3 (5.3–25.6)	13.0 (5.5–26.3)	13.0 (5.6–25.9)
Central Europe, eastern Europe, and central Asia	60 to 64	12.8 (5.1–25.1)	12.7 (4.8–25.4)	13.0 (5.1–25.2)	12.8 (5.2–26.2)	12.6 (5.3–25.6)
Central Europe, eastern Europe, and central Asia	65 to 69	12.2 (4.8–24.1)	12.0 (4.5–24.3)	12.1 (4.7–23.8)	12.1 (4.9–25.0)	12.0 (5.0–24.6)
Central Europe, eastern Europe, and central Asia	70 to 74	9.7 (3.7–19.7)	9.6 (3.5–20.0)	9.6 (3.6–19.5)	9.8 (3.9–20.9)	9.7 (3.9–20.5)
Central Europe, eastern Europe, and central Asia	75 to 79	8.7 (3.3–17.9)	8.8 (3.2–18.6)	8.8 (3.3–18.0)	8.9 (3.5–19.0)	9.0 (3.6–18.9)

Supplementary Table S11: Prevalence of male SVAC by age and location for 1990, 2000, 2010, 2020, and 2023						
**Location**	**Age Range**	**1990**	**2000**	**2010**	**2020**	**2023**
Central Europe, eastern Europe, and central Asia	80 to 84	6.2 (2.3–12.9)	6.2 (2.2–13.4)	6.2 (2.3–13.0)	6.3 (2.4–13.9)	6.3 (2.5–13.7)
Central Europe, eastern Europe, and central Asia	85 to 89	5.4 (2.0–11.5)	5.5 (1.9–12.0)	5.7 (2.0–12.0)	5.7 (2.1–12.8)	5.6 (2.1–12.5)
Central Europe, eastern Europe, and central Asia	90 to 94	4.8 (1.7–10.2)	4.9 (1.7–10.8)	5.1 (1.8–10.8)	5.1 (1.9–11.5)	5.1 (1.9–11.4)
Central Europe, eastern Europe, and central Asia	95 plus	4.6 (1.6– 9.8)	4.7 (1.6–10.3)	4.9 (1.8–10.5)	5.1 (1.9–11.6)	5.1 (1.9–11.3)
Central Europe, eastern Europe, and central Asia	Age-standardized	12.7 (5.1–24.8)	12.6 (4.9–24.9)	12.5 (5.1–24.1)	12.3 (5.2–25.0)	12.3 (5.2–24.6)
Central Europe, eastern Europe, and central Asia	All age	12.9 (5.2–25.1)	12.8 (4.9–25.3)	12.6 (5.1–24.3)	12.5 (5.3–25.3)	12.4 (5.3–24.9)
Central Asia	20 to 24	6.5 (2.5–13.6)	6.4 (2.4–13.2)	6.3 (2.4–12.9)	6.4 (2.5–13.8)	6.4 (2.5–13.7)
Central Asia	25 to 29	6.7 (2.6–13.9)	6.6 (2.5–13.5)	6.5 (2.5–13.2)	6.5 (2.6–14.0)	6.6 (2.6–14.1)
Central Asia	30 to 34	7.6 (2.8–15.9)	7.6 (2.9–15.9)	7.6 (3.1–15.0)	7.6 (3.4–15.5)	7.6 (3.3–15.6)
Central Asia	35 to 39	7.8 (3.1–15.7)	7.7 (3.2–15.4)	7.6 (3.2–14.8)	7.7 (3.4–15.6)	7.7 (3.3–15.8)
Central Asia	40 to 44	8.3 (3.2–17.2)	8.5 (3.3–17.4)	8.5 (3.7–16.4)	8.5 (4.0–16.8)	8.5 (4.0–16.7)
Central Asia	45 to 49	8.1 (3.3–16.3)	8.2 (3.4–16.1)	8.2 (3.7–15.6)	8.2 (3.8–16.2)	8.2 (3.8–16.2)
Central Asia	50 to 54	8.1 (3.2–16.3)	8.0 (3.4–15.9)	8.0 (3.5–15.3)	8.0 (3.7–16.0)	8.0 (3.5–16.1)
Central Asia	55 to 59	8.0 (3.1–16.2)	7.9 (3.2–16.0)	8.1 (3.6–15.5)	8.3 (4.1–15.9)	8.2 (4.0–15.8)
Central Asia	60 to 64	7.6 (2.9–15.5)	7.5 (2.9–15.4)	7.6 (3.0–14.9)	7.7 (3.1–16.2)	7.7 (3.2–16.1)

Supplementary Table S11: Prevalence of male SVAC by age and location for 1990, 2000, 2010, 2020, and 2023						
**Location**	**Age Range**	**1990**	**2000**	**2010**	**2020**	**2023**
Central Asia	65 to 69	7.1 (2.6–14.9)	7.2 (2.7–15.1)	7.1 (2.8–14.4)	7.2 (3.0–15.3)	7.2 (3.1–15.1)
Central Asia	70 to 74	5.5 (2.0–11.8)	5.5 (1.9–11.9)	5.6 (2.0–11.7)	5.6 (2.1–12.5)	5.6 (2.1–12.4)
Central Asia	75 to 79	4.9 (1.8–10.7)	4.9 (1.7–10.7)	5.0 (1.8–10.6)	4.9 (1.8–11.2)	5.0 (1.9–11.1)
Central Asia	80 to 84	3.4 (1.2– 7.5)	3.4 (1.1– 7.5)	3.4 (1.2– 7.4)	3.5 (1.3– 8.0)	3.4 (1.3– 7.8)
Central Asia	85 to 89	3.1 (1.1– 6.8)	3.1 (1.0– 6.8)	3.1 (1.1– 6.6)	3.1 (1.1– 7.2)	3.1 (1.2– 7.1)
Central Asia	90 to 94	2.8 (1.0– 6.2)	2.8 (0.9– 6.1)	2.7 (1.0– 6.0)	2.8 (1.0– 6.5)	2.8 (1.0– 6.4)
Central Asia	95 plus	2.7 (1.0– 6.1)	2.7 (0.9– 6.1)	2.7 (1.0– 5.9)	2.7 (1.0– 6.3)	2.7 (1.0– 6.3)
Central Asia	Age-standardized	7.3 (2.8–15.0)	7.2 (2.8–14.8)	7.2 (3.0–14.1)	7.2 (3.2–14.9)	7.3 (3.1–15.0)
Central Asia	All age	7.3 (2.8–15.1)	7.3 (2.9–15.0)	7.3 (3.0–14.3)	7.4 (3.3–15.1)	7.4 (3.2–15.2)
Armenia	20 to 24	6.5 (2.4–14.0)	6.5 (2.3–13.9)	6.5 (2.3–13.6)	6.5 (2.4–14.5)	6.5 (2.5–14.3)
Armenia	25 to 29	6.7 (2.4–14.4)	6.7 (2.3–14.3)	6.7 (2.4–14.0)	6.7 (2.5–14.9)	6.7 (2.6–14.7)
Armenia	30 to 34	7.4 (2.7–15.8)	7.4 (2.6–15.7)	7.4 (2.7–15.4)	7.4 (2.8–16.4)	7.4 (2.9–16.2)
Armenia	35 to 39	7.6 (2.8–16.2)	7.6 (2.7–16.1)	7.6 (2.8–15.8)	7.6 (2.9–16.8)	7.6 (2.9–16.6)
Armenia	40 to 44	8.1 (3.0–17.0)	8.1 (2.9–17.0)	8.0 (2.9–16.6)	8.1 (3.1–17.7)	8.1 (3.1–17.5)
Armenia	45 to 49	8.0 (2.9–16.8)	7.9 (2.8–16.7)	7.9 (2.9–16.4)	8.0 (3.0–17.5)	8.0 (3.1–17.3)

Supplementary Table S11: Prevalence of male SVAC by age and location for 1990, 2000, 2010, 2020, and 2023						
**Location**	**Age Range**	**1990**	**2000**	**2010**	**2020**	**2023**
Armenia	50 to 54	7.9 (2.9–16.6)	7.9 (2.8–16.6)	7.9 (2.9–16.3)	7.9 (3.0–17.4)	7.9 (3.0–17.1)
Armenia	55 to 59	7.7 (2.8–16.3)	7.7 (2.7–16.2)	7.7 (2.8–15.9)	7.7 (2.9–17.0)	7.7 (3.0–16.8)
Armenia	60 to 64	7.5 (2.7–15.8)	7.4 (2.6–15.8)	7.5 (2.7–15.5)	7.5 (2.8–16.6)	7.5 (2.9–16.4)
Armenia	65 to 69	6.9 (2.5–14.8)	6.9 (2.4–14.7)	6.9 (2.5–14.5)	7.0 (2.6–15.5)	7.0 (2.7–15.3)
Armenia	70 to 74	5.5 (2.0–11.8)	5.4 (1.9–11.8)	5.4 (1.9–11.5)	5.5 (2.0–12.4)	5.5 (2.1–12.2)
Armenia	75 to 79	4.9 (1.7–10.6)	4.9 (1.7–10.6)	4.9 (1.7–10.4)	4.9 (1.8–11.1)	4.9 (1.8–11.0)
Armenia	80 to 84	3.4 (1.2– 7.5)	3.4 (1.1– 7.4)	3.4 (1.2– 7.3)	3.4 (1.2– 7.8)	3.4 (1.3– 7.7)
Armenia	85 to 89	3.0 (1.1– 6.7)	3.0 (1.0– 6.7)	3.0 (1.1– 6.5)	3.0 (1.1– 7.0)	3.0 (1.1– 7.0)
Armenia	90 to 94	2.7 (1.0– 6.0)	2.7 (0.9– 6.0)	2.7 (0.9– 5.9)	2.7 (1.0– 6.4)	2.7 (1.0– 6.3)
Armenia	95 plus	2.7 (0.9– 6.0)	2.7 (0.9– 6.0)	2.7 (0.9– 5.9)	2.7 (1.0– 6.3)	2.7 (1.0– 6.2)
Armenia	Age-standardized	7.1 (2.6–15.1)	7.1 (2.5–15.1)	7.1 (2.6–14.8)	7.1 (2.7–15.8)	7.1 (2.7–15.6)
Armenia	All age	7.2 (2.6–15.3)	7.2 (2.5–15.3)	7.1 (2.6–14.8)	7.1 (2.7–15.8)	7.1 (2.7–15.6)
Azerbaijan	20 to 24	7.1 (2.6–15.0)	6.5 (2.5–13.3)	5.9 (2.7–10.8)	5.8 (2.8–11.0)	5.9 (2.7–11.4)
Azerbaijan	25 to 29	7.3 (2.7–15.5)	6.7 (2.6–13.7)	6.0 (2.8–11.0)	6.0 (2.9–11.2)	6.1 (2.8–11.8)
Azerbaijan	30 to 34	9.1 (3.5–18.4)	9.2 (3.9–17.4)	9.1 (5.2–14.8)	9.1 (5.4–14.2)	9.2 (5.0–15.4)

Supplementary Table S11: Prevalence of male SVAC by age and location for 1990, 2000, 2010, 2020, and 2023						
**Location**	**Age Range**	**1990**	**2000**	**2010**	**2020**	**2023**
Azerbaijan	35 to 39	9.1 (3.5–18.4)	8.9 (3.7–17.1)	8.5 (4.6–14.0)	8.4 (4.9–13.4)	8.5 (4.6–14.6)
Azerbaijan	40 to 44	10.6 (4.1–21.2)	11.2 (5.0–21.1)	11.9 (7.2–18.6)	11.9 (7.7–17.3)	11.8 (6.9–18.7)
Azerbaijan	45 to 49	10.1 (3.9–20.6)	10.4 (4.5–19.6)	10.6 (6.3–16.8)	10.5 (6.7–15.6)	10.5 (6.0–17.2)
Azerbaijan	50 to 54	9.7 (3.7–19.6)	9.7 (4.2–18.3)	9.5 (5.5–15.4)	9.5 (5.7–14.7)	9.6 (5.2–15.9)
Azerbaijan	55 to 59	10.3 (4.0–20.7)	11.1 (5.0–20.9)	12.0 (7.4–18.6)	12.1 (7.9–17.5)	11.9 (7.1–18.9)
Azerbaijan	60 to 64	9.1 (3.4–19.0)	9.1 (3.3–19.0)	9.1 (3.4–18.6)	9.2 (3.5–19.9)	9.2 (3.6–19.6)
Azerbaijan	65 to 69	8.4 (3.1–17.7)	8.4 (3.0–17.7)	8.4 (3.1–17.4)	8.5 (3.3–18.5)	8.5 (3.3–18.3)
Azerbaijan	70 to 74	6.7 (2.4–14.2)	6.6 (2.3–14.2)	6.7 (2.4–13.9)	6.7 (2.5–14.9)	6.7 (2.6–14.7)
Azerbaijan	75 to 79	6.0 (2.1–12.8)	5.9 (2.1–12.8)	6.0 (2.1–12.6)	6.0 (2.2–13.5)	6.0 (2.3–13.3)
Azerbaijan	80 to 84	4.1 (1.5– 9.1)	4.1 (1.4– 9.1)	4.1 (1.5– 8.9)	4.2 (1.5– 9.6)	4.2 (1.6– 9.4)
Azerbaijan	85 to 89	3.7 (1.3– 8.2)	3.7 (1.3– 8.2)	3.7 (1.3– 8.0)	3.7 (1.4– 8.6)	3.7 (1.4– 8.5)
Azerbaijan	90 to 94	3.3 (1.2– 7.4)	3.3 (1.1– 7.4)	3.3 (1.2– 7.2)	3.4 (1.2– 7.8)	3.4 (1.2– 7.7)
Azerbaijan	95 plus	3.3 (1.2– 7.3)	3.3 (1.1– 7.3)	3.3 (1.2– 7.2)	3.3 (1.2– 7.7)	3.3 (1.2– 7.6)
Azerbaijan	Age-standardized	8.6 (3.4–17.5)	8.6 (3.6–16.4)	8.5 (4.7–14.2)	8.5 (5.0–13.9)	8.6 (4.9–14.2)
Azerbaijan	All age	8.6 (3.4–17.4)	8.7 (3.7–16.6)	8.6 (4.8–13.9)	8.8 (5.2–14.2)	8.9 (5.1–14.5)

Supplementary Table S11: Prevalence of male SVAC by age and location for 1990, 2000, 2010, 2020, and 2023						
**Location**	**Age Range**	**1990**	**2000**	**2010**	**2020**	**2023**
Georgia	20 to 24	6.1 (2.2–13.0)	5.7 (2.1–12.0)	5.2 (2.2–10.3)	5.0 (2.4– 9.7)	5.0 (2.3– 9.7)
Georgia	25 to 29	6.3 (2.3–13.6)	5.9 (2.2–12.4)	5.4 (2.3–10.5)	5.1 (2.5– 9.9)	5.2 (2.3– 9.8)
Georgia	30 to 34	7.5 (2.7–15.7)	7.5 (3.0–14.8)	7.3 (3.6–13.3)	7.2 (4.5–11.1)	7.2 (4.3–12.0)
Georgia	35 to 39	7.6 (2.9–16.0)	7.4 (2.9–14.6)	7.0 (3.4–12.5)	6.7 (3.9–10.8)	6.8 (3.8–11.1)
Georgia	40 to 44	8.6 (3.2–17.7)	8.9 (3.6–17.5)	9.3 (4.9–16.1)	9.4 (6.3–13.5)	9.3 (5.9–14.2)
Georgia	45 to 49	8.4 (3.1–17.2)	8.4 (3.4–16.5)	8.4 (4.3–14.8)	8.3 (5.4–12.4)	8.3 (5.0–13.1)
Georgia	50 to 54	8.1 (3.0–16.9)	8.0 (3.2–15.7)	7.7 (3.9–13.9)	7.5 (4.7–11.4)	7.6 (4.5–12.3)
Georgia	55 to 59	8.5 (3.2–17.4)	8.9 (3.6–17.5)	9.4 (5.0–16.1)	9.6 (6.6–13.7)	9.5 (6.0–14.2)
Georgia	60 to 64	8.2 (3.0–16.7)	8.4 (3.4–16.5)	8.6 (4.4–14.9)	8.6 (5.7–12.5)	8.6 (5.3–13.2)
Georgia	65 to 69	7.9 (2.9–16.2)	8.3 (3.4–16.5)	9.0 (4.7–15.4)	9.2 (6.3–13.2)	9.1 (5.7–13.8)
Georgia	70 to 74	5.8 (2.1–12.5)	5.8 (2.0–12.5)	5.8 (2.1–12.2)	5.8 (2.2–13.1)	5.8 (2.2–13.0)
Georgia	75 to 79	5.2 (1.9–11.2)	5.2 (1.8–11.2)	5.2 (1.8–11.0)	5.2 (1.9–11.8)	5.2 (2.0–11.7)
Georgia	80 to 84	3.6 (1.3– 7.9)	3.6 (1.2– 7.9)	3.6 (1.3– 7.8)	3.6 (1.3– 8.3)	3.6 (1.3– 8.2)
Georgia	85 to 89	3.2 (1.1– 7.1)	3.2 (1.1– 7.1)	3.2 (1.1– 7.0)	3.2 (1.2– 7.5)	3.2 (1.2– 7.4)
Georgia	90 to 94	2.9 (1.0– 6.4)	2.9 (1.0– 6.4)	2.9 (1.0– 6.3)	2.9 (1.1– 6.8)	2.9 (1.1– 6.7)

Supplementary Table S11: Prevalence of male SVAC by age and location for 1990, 2000, 2010, 2020, and 2023						
**Location**	**Age Range**	**1990**	**2000**	**2010**	**2020**	**2023**
Georgia	95 plus	2.9 (1.0– 6.4)	2.9 (1.0– 6.4)	2.9 (1.0– 6.2)	2.9 (1.1– 6.7)	2.9 (1.1– 6.6)
Georgia	Age-standardized	7.3 (2.8–15.2)	7.3 (2.9–14.4)	7.2 (3.5–12.7)	7.1 (4.4–11.1)	7.1 (4.4–11.2)
Georgia	All age	7.4 (2.8–15.4)	7.4 (3.0–14.7)	7.2 (3.5–12.8)	7.3 (4.6–11.4)	7.3 (4.5–11.4)
Kazakhstan	20 to 24	6.7 (2.8–13.3)	6.6 (3.0–12.2)	6.7 (2.9–12.5)	6.9 (2.7–14.7)	6.9 (2.8–14.9)
Kazakhstan	25 to 29	6.8 (2.9–13.5)	6.7 (3.0–12.3)	6.8 (3.0–12.7)	7.0 (2.8–15.0)	7.0 (2.8–15.1)
Kazakhstan	30 to 34	7.8 (2.9–16.5)	7.8 (2.7–16.4)	7.8 (2.8–16.1)	7.8 (3.0–17.2)	7.8 (3.0–17.0)
Kazakhstan	35 to 39	7.8 (3.4–14.9)	7.7 (3.9–13.2)	7.8 (3.7–13.9)	8.0 (3.2–16.8)	8.0 (3.3–16.8)
Kazakhstan	40 to 44	8.2 (3.0–17.3)	8.2 (2.9–17.3)	8.2 (3.0–17.0)	8.3 (3.2–18.1)	8.3 (3.2–17.9)
Kazakhstan	45 to 49	8.1 (3.5–15.6)	8.0 (3.9–14.1)	8.1 (3.7–14.6)	8.2 (3.3–17.2)	8.2 (3.3–17.3)
Kazakhstan	50 to 54	8.0 (3.5–15.6)	8.0 (3.8–14.3)	8.0 (3.6–14.7)	8.1 (3.3–17.0)	8.1 (3.3–17.1)
Kazakhstan	55 to 59	7.3 (2.9–14.5)	7.1 (2.9–13.9)	7.2 (2.9–14.3)	7.4 (2.9–16.0)	7.5 (2.9–16.1)
Kazakhstan	60 to 64	7.0 (2.8–14.0)	6.9 (2.9–13.4)	7.0 (2.8–13.6)	7.2 (2.8–15.5)	7.2 (2.9–15.6)
Kazakhstan	65 to 69	6.7 (2.4–14.3)	6.7 (2.3–14.3)	6.7 (2.4–14.0)	6.8 (2.5–15.0)	6.8 (2.6–14.8)
Kazakhstan	70 to 74	5.3 (1.9–11.4)	5.2 (1.8–11.4)	5.3 (1.9–11.2)	5.3 (2.0–12.0)	5.3 (2.0–11.8)
Kazakhstan	75 to 79	4.7 (1.7–10.2)	4.7 (1.6–10.2)	4.7 (1.7–10.0)	4.7 (1.8–10.8)	4.7 (1.8–10.6)

Supplementary Table S11: Prevalence of male SVAC by age and location for 1990, 2000, 2010, 2020, and 2023						
**Location**	**Age Range**	**1990**	**2000**	**2010**	**2020**	**2023**
Kazakhstan	80 to 84	3.3 (1.1– 7.2)	3.2 (1.1– 7.2)	3.3 (1.1– 7.0)	3.3 (1.2– 7.6)	3.3 (1.2– 7.5)
Kazakhstan	85 to 89	2.9 (1.0– 6.5)	2.9 (1.0– 6.4)	2.9 (1.0– 6.3)	2.9 (1.1– 6.8)	2.9 (1.1– 6.7)
Kazakhstan	90 to 94	2.6 (0.9– 5.8)	2.6 (0.9– 5.8)	2.6 (0.9– 5.7)	2.6 (1.0– 6.1)	2.6 (1.0– 6.1)
Kazakhstan	95 plus	2.6 (0.9– 5.8)	2.6 (0.9– 5.8)	2.6 (0.9– 5.6)	2.6 (0.9– 6.1)	2.6 (1.0– 6.0)
Kazakhstan	Age-standardized	7.2 (2.9–14.3)	7.1 (3.1–13.7)	7.1 (2.9–13.9)	7.3 (2.9–15.7)	7.3 (2.9–15.7)
Kazakhstan	All age	7.3 (3.0–14.6)	7.3 (3.2–14.0)	7.3 (3.0–14.1)	7.5 (2.9–16.1)	7.5 (3.0–16.1)
Kyrgyzstan	20 to 24	6.5 (2.4–14.0)	6.5 (2.3–13.9)	6.5 (2.3–13.6)	6.5 (2.4–14.5)	6.5 (2.5–14.3)
Kyrgyzstan	25 to 29	6.7 (2.4–14.4)	6.7 (2.3–14.3)	6.7 (2.4–14.0)	6.7 (2.5–14.9)	6.7 (2.6–14.7)
Kyrgyzstan	30 to 34	7.4 (2.7–15.8)	7.4 (2.6–15.7)	7.4 (2.7–15.4)	7.4 (2.8–16.4)	7.4 (2.9–16.2)
Kyrgyzstan	35 to 39	7.6 (2.8–16.2)	7.6 (2.7–16.1)	7.6 (2.8–15.8)	7.6 (2.9–16.8)	7.6 (2.9–16.6)
Kyrgyzstan	40 to 44	8.1 (3.0–17.0)	8.1 (2.9–17.0)	8.0 (2.9–16.6)	8.1 (3.1–17.7)	8.1 (3.1–17.5)
Kyrgyzstan	45 to 49	8.0 (2.9–16.8)	7.9 (2.8–16.7)	7.9 (2.9–16.4)	8.0 (3.0–17.5)	8.0 (3.1–17.3)
Kyrgyzstan	50 to 54	7.9 (2.9–16.6)	7.9 (2.8–16.6)	7.9 (2.9–16.3)	7.9 (3.0–17.4)	7.9 (3.0–17.1)
Kyrgyzstan	55 to 59	7.7 (2.8–16.3)	7.7 (2.7–16.2)	7.7 (2.8–15.9)	7.7 (2.9–17.0)	7.7 (3.0–16.8)
Kyrgyzstan	60 to 64	7.5 (2.7–15.8)	7.4 (2.6–15.8)	7.5 (2.7–15.5)	7.5 (2.8–16.6)	7.5 (2.9–16.4)

Supplementary Table S11: Prevalence of male SVAC by age and location for 1990, 2000, 2010, 2020, and 2023						
**Location**	**Age Range**	**1990**	**2000**	**2010**	**2020**	**2023**
Kyrgyzstan	65 to 69	6.9 (2.5–14.8)	6.9 (2.4–14.7)	6.9 (2.5–14.5)	7.0 (2.6–15.5)	7.0 (2.7–15.3)
Kyrgyzstan	70 to 74	5.5 (2.0–11.8)	5.4 (1.9–11.8)	5.4 (1.9–11.5)	5.5 (2.0–12.4)	5.5 (2.1–12.2)
Kyrgyzstan	75 to 79	4.9 (1.7–10.6)	4.9 (1.7–10.6)	4.9 (1.7–10.4)	4.9 (1.8–11.1)	4.9 (1.8–11.0)
Kyrgyzstan	80 to 84	3.4 (1.2– 7.5)	3.4 (1.1– 7.4)	3.4 (1.2– 7.3)	3.4 (1.2– 7.8)	3.4 (1.3– 7.7)
Kyrgyzstan	85 to 89	3.0 (1.1– 6.7)	3.0 (1.0– 6.7)	3.0 (1.1– 6.5)	3.0 (1.1– 7.0)	3.0 (1.1– 7.0)
Kyrgyzstan	90 to 94	2.7 (1.0– 6.0)	2.7 (0.9– 6.0)	2.7 (0.9– 5.9)	2.7 (1.0– 6.4)	2.7 (1.0– 6.3)
Kyrgyzstan	95 plus	2.7 (0.9– 6.0)	2.7 (0.9– 6.0)	2.7 (0.9– 5.9)	2.7 (1.0– 6.3)	2.7 (1.0– 6.2)
Kyrgyzstan	Age-standardized	7.1 (2.6–15.1)	7.1 (2.5–15.1)	7.1 (2.6–14.8)	7.1 (2.7–15.8)	7.1 (2.7–15.6)
Kyrgyzstan	All age	7.2 (2.6–15.3)	7.2 (2.5–15.2)	7.2 (2.6–14.9)	7.3 (2.8–16.1)	7.3 (2.8–15.9)
Mongolia	20 to 24	4.1 (1.4– 8.9)	4.1 (1.4– 8.9)	4.1 (1.4– 8.7)	4.1 (1.5– 9.4)	4.1 (1.5– 9.3)
Mongolia	25 to 29	4.2 (1.5– 9.1)	4.2 (1.5– 9.0)	4.2 (1.7– 8.3)	4.2 (1.8– 8.6)	4.2 (1.8– 8.5)
Mongolia	30 to 34	4.5 (1.6– 9.7)	4.4 (1.6– 9.7)	4.3 (1.7– 8.9)	4.3 (1.8– 9.2)	4.4 (1.8– 9.3)
Mongolia	35 to 39	4.6 (1.6– 9.9)	4.6 (1.6–10.0)	4.5 (1.7– 9.3)	4.5 (1.9– 9.6)	4.6 (1.8– 9.7)
Mongolia	40 to 44	4.9 (1.8–10.4)	4.9 (1.7–10.6)	4.9 (1.9–10.1)	4.9 (2.0–10.5)	4.9 (2.0–10.4)
Mongolia	45 to 49	4.6 (1.6– 9.8)	4.5 (1.6– 9.9)	4.4 (1.7– 9.1)	4.4 (1.8– 9.3)	4.4 (1.8– 9.4)

Supplementary Table S11: Prevalence of male SVAC by age and location for 1990, 2000, 2010, 2020, and 2023						
**Location**	**Age Range**	**1990**	**2000**	**2010**	**2020**	**2023**
Mongolia	50 to 54	4.6 (1.6–10.0)	4.6 (1.6– 9.9)	4.6 (1.6– 9.7)	4.6 (1.7–10.4)	4.6 (1.7–10.3)
Mongolia	55 to 59	4.4 (1.6– 9.6)	4.4 (1.5– 9.6)	4.4 (1.6– 9.4)	4.4 (1.6–10.1)	4.4 (1.7–10.0)
Mongolia	60 to 64	4.3 (1.5– 9.3)	4.3 (1.5– 9.3)	4.3 (1.5– 9.1)	4.3 (1.6– 9.8)	4.3 (1.6– 9.6)
Mongolia	65 to 69	3.9 (1.4– 8.6)	3.9 (1.3– 8.6)	3.9 (1.4– 8.4)	4.0 (1.5– 9.1)	4.0 (1.5– 8.9)
Mongolia	70 to 74	3.1 (1.1– 6.8)	3.1 (1.0– 6.8)	3.1 (1.1– 6.6)	3.1 (1.1– 7.1)	3.1 (1.1– 7.0)
Mongolia	75 to 79	2.7 (1.0– 6.1)	2.7 (0.9– 6.0)	2.7 (0.9– 5.9)	2.7 (1.0– 6.4)	2.7 (1.0– 6.3)
Mongolia	80 to 84	1.9 (0.7– 4.2)	1.9 (0.6– 4.2)	1.9 (0.6– 4.1)	1.9 (0.7– 4.4)	1.9 (0.7– 4.4)
Mongolia	85 to 89	1.7 (0.6– 3.8)	1.7 (0.6– 3.8)	1.7 (0.6– 3.7)	1.7 (0.6– 4.0)	1.7 (0.6– 3.9)
Mongolia	90 to 94	1.5 (0.5– 3.4)	1.5 (0.5– 3.4)	1.5 (0.5– 3.3)	1.5 (0.5– 3.6)	1.5 (0.6– 3.5)
Mongolia	95 plus	1.5 (0.5– 3.4)	1.5 (0.5– 3.3)	1.5 (0.5– 3.3)	1.5 (0.5– 3.5)	1.5 (0.5– 3.5)
Mongolia	Age-standardized	4.2 (1.5– 9.3)	4.2 (1.5– 9.2)	4.2 (1.6– 8.8)	4.2 (1.7– 9.1)	4.2 (1.7– 9.2)
Mongolia	All age	4.3 (1.5– 9.4)	4.3 (1.5– 9.5)	4.3 (1.6– 9.0)	4.3 (1.7– 9.4)	4.4 (1.7– 9.4)
Tajikistan	20 to 24	6.5 (2.4–14.0)	6.5 (2.3–13.9)	6.5 (2.3–13.6)	6.5 (2.4–14.5)	6.5 (2.5–14.3)
Tajikistan	25 to 29	6.7 (2.4–14.4)	6.7 (2.3–14.3)	6.7 (2.4–14.0)	6.7 (2.5–14.9)	6.7 (2.6–14.7)
Tajikistan	30 to 34	7.4 (2.7–15.8)	7.4 (2.6–15.7)	7.4 (2.7–15.4)	7.4 (2.8–16.4)	7.4 (2.9–16.2)

Supplementary Table S11: Prevalence of male SVAC by age and location for 1990, 2000, 2010, 2020, and 2023						
**Location**	**Age Range**	**1990**	**2000**	**2010**	**2020**	**2023**
Tajikistan	35 to 39	7.6 (2.8–16.2)	7.6 (2.7–16.1)	7.6 (2.8–15.8)	7.6 (2.9–16.8)	7.6 (2.9–16.6)
Tajikistan	40 to 44	8.1 (3.0–17.0)	8.1 (2.9–17.0)	8.0 (2.9–16.6)	8.1 (3.1–17.7)	8.1 (3.1–17.5)
Tajikistan	45 to 49	8.0 (2.9–16.8)	7.9 (2.8–16.7)	7.9 (2.9–16.4)	8.0 (3.0–17.5)	8.0 (3.1–17.3)
Tajikistan	50 to 54	7.9 (2.9–16.6)	7.9 (2.8–16.6)	7.9 (2.9–16.3)	7.9 (3.0–17.4)	7.9 (3.0–17.1)
Tajikistan	55 to 59	7.7 (2.8–16.3)	7.7 (2.7–16.2)	7.7 (2.8–15.9)	7.7 (2.9–17.0)	7.7 (3.0–16.8)
Tajikistan	60 to 64	7.5 (2.7–15.8)	7.4 (2.6–15.8)	7.5 (2.7–15.5)	7.5 (2.8–16.6)	7.5 (2.9–16.4)
Tajikistan	65 to 69	6.9 (2.5–14.8)	6.9 (2.4–14.7)	6.9 (2.5–14.5)	7.0 (2.6–15.5)	7.0 (2.7–15.3)
Tajikistan	70 to 74	5.5 (2.0–11.8)	5.4 (1.9–11.8)	5.4 (1.9–11.5)	5.5 (2.0–12.4)	5.5 (2.1–12.2)
Tajikistan	75 to 79	4.9 (1.7–10.6)	4.9 (1.7–10.6)	4.9 (1.7–10.4)	4.9 (1.8–11.1)	4.9 (1.8–11.0)
Tajikistan	80 to 84	3.4 (1.2– 7.5)	3.4 (1.1– 7.4)	3.4 (1.2– 7.3)	3.4 (1.2– 7.8)	3.4 (1.3– 7.7)
Tajikistan	85 to 89	3.0 (1.1– 6.7)	3.0 (1.0– 6.7)	3.0 (1.1– 6.5)	3.0 (1.1– 7.0)	3.0 (1.1– 7.0)
Tajikistan	90 to 94	2.7 (1.0– 6.0)	2.7 (0.9– 6.0)	2.7 (0.9– 5.9)	2.7 (1.0– 6.4)	2.7 (1.0– 6.3)
Tajikistan	95 plus	2.7 (0.9– 6.0)	2.7 (0.9– 6.0)	2.7 (0.9– 5.9)	2.7 (1.0– 6.3)	2.7 (1.0– 6.2)
Tajikistan	Age-standardized	7.1 (2.6–15.1)	7.1 (2.5–15.1)	7.1 (2.6–14.8)	7.1 (2.7–15.8)	7.1 (2.7–15.6)
Tajikistan	All age	7.1 (2.6–15.2)	7.2 (2.5–15.2)	7.2 (2.6–14.9)	7.2 (2.7–16.0)	7.3 (2.8–15.8)

Supplementary Table S11: Prevalence of male SVAC by age and location for 1990, 2000, 2010, 2020, and 2023						
**Location**	**Age Range**	**1990**	**2000**	**2010**	**2020**	**2023**
Turkmenistan	20 to 24	6.5 (2.4–14.0)	6.5 (2.3–13.9)	6.5 (2.3–13.6)	6.5 (2.4–14.5)	6.5 (2.5–14.3)
Turkmenistan	25 to 29	6.7 (2.4–14.4)	6.7 (2.3–14.3)	6.7 (2.4–14.0)	6.7 (2.5–14.9)	6.7 (2.6–14.7)
Turkmenistan	30 to 34	7.4 (2.7–15.8)	7.4 (2.6–15.7)	7.4 (2.7–15.4)	7.4 (2.8–16.4)	7.4 (2.9–16.2)
Turkmenistan	35 to 39	7.6 (2.8–16.2)	7.6 (2.7–16.1)	7.6 (2.8–15.8)	7.6 (2.9–16.8)	7.6 (2.9–16.6)
Turkmenistan	40 to 44	8.1 (3.0–17.0)	8.1 (2.9–17.0)	8.0 (2.9–16.6)	8.1 (3.1–17.7)	8.1 (3.1–17.5)
Turkmenistan	45 to 49	8.0 (2.9–16.8)	7.9 (2.8–16.7)	7.9 (2.9–16.4)	8.0 (3.0–17.5)	8.0 (3.1–17.3)
Turkmenistan	50 to 54	7.9 (2.9–16.6)	7.9 (2.8–16.6)	7.9 (2.9–16.3)	7.9 (3.0–17.4)	7.9 (3.0–17.1)
Turkmenistan	55 to 59	7.7 (2.8–16.3)	7.7 (2.7–16.2)	7.7 (2.8–15.9)	7.7 (2.9–17.0)	7.7 (3.0–16.8)
Turkmenistan	60 to 64	7.5 (2.7–15.8)	7.4 (2.6–15.8)	7.5 (2.7–15.5)	7.5 (2.8–16.6)	7.5 (2.9–16.4)
Turkmenistan	65 to 69	6.9 (2.5–14.8)	6.9 (2.4–14.7)	6.9 (2.5–14.5)	7.0 (2.6–15.5)	7.0 (2.7–15.3)
Turkmenistan	70 to 74	5.5 (2.0–11.8)	5.4 (1.9–11.8)	5.4 (1.9–11.5)	5.5 (2.0–12.4)	5.5 (2.1–12.2)
Turkmenistan	75 to 79	4.9 (1.7–10.6)	4.9 (1.7–10.6)	4.9 (1.7–10.4)	4.9 (1.8–11.1)	4.9 (1.8–11.0)
Turkmenistan	80 to 84	3.4 (1.2– 7.5)	3.4 (1.1– 7.4)	3.4 (1.2– 7.3)	3.4 (1.2– 7.8)	3.4 (1.3– 7.7)
Turkmenistan	85 to 89	3.0 (1.1– 6.7)	3.0 (1.0– 6.7)	3.0 (1.1– 6.5)	3.0 (1.1– 7.0)	3.0 (1.1– 7.0)
Turkmenistan	90 to 94	2.7 (1.0– 6.0)	2.7 (0.9– 6.0)	2.7 (0.9– 5.9)	2.7 (1.0– 6.4)	2.7 (1.0– 6.3)

Supplementary Table S11: Prevalence of male SVAC by age and location for 1990, 2000, 2010, 2020, and 2023						
**Location**	**Age Range**	**1990**	**2000**	**2010**	**2020**	**2023**
Turkmenistan	95 plus	2.7 (0.9– 6.0)	2.7 (0.9– 6.0)	2.7 (0.9– 5.9)	2.7 (1.0– 6.3)	2.7 (1.0– 6.2)
Turkmenistan	Age-standardized	7.1 (2.6–15.1)	7.1 (2.5–15.1)	7.1 (2.6–14.8)	7.1 (2.7–15.8)	7.1 (2.7–15.6)
Turkmenistan	All age	7.2 (2.6–15.2)	7.2 (2.5–15.3)	7.2 (2.6–15.0)	7.3 (2.8–16.1)	7.3 (2.8–15.9)
Uzbekistan	20 to 24	6.5 (2.4–14.0)	6.5 (2.3–13.9)	6.5 (2.3–13.6)	6.5 (2.4–14.5)	6.5 (2.5–14.3)
Uzbekistan	25 to 29	6.7 (2.4–14.4)	6.7 (2.3–14.3)	6.7 (2.4–14.0)	6.7 (2.5–14.9)	6.7 (2.6–14.7)
Uzbekistan	30 to 34	7.4 (2.7–15.8)	7.4 (2.6–15.7)	7.4 (2.7–15.4)	7.4 (2.8–16.4)	7.4 (2.9–16.2)
Uzbekistan	35 to 39	7.6 (2.8–16.2)	7.6 (2.7–16.1)	7.6 (2.8–15.8)	7.6 (2.9–16.8)	7.6 (2.9–16.6)
Uzbekistan	40 to 44	8.1 (3.0–17.0)	8.1 (2.9–17.0)	8.0 (2.9–16.6)	8.1 (3.1–17.7)	8.1 (3.1–17.5)
Uzbekistan	45 to 49	8.0 (2.9–16.8)	7.9 (2.8–16.7)	7.9 (2.9–16.4)	8.0 (3.0–17.5)	8.0 (3.1–17.3)
Uzbekistan	50 to 54	7.9 (2.9–16.6)	7.9 (2.8–16.6)	7.9 (2.9–16.3)	7.9 (3.0–17.4)	7.9 (3.0–17.1)
Uzbekistan	55 to 59	7.7 (2.8–16.3)	7.7 (2.7–16.2)	7.7 (2.8–15.9)	7.7 (2.9–17.0)	7.7 (3.0–16.8)
Uzbekistan	60 to 64	7.5 (2.7–15.8)	7.4 (2.6–15.8)	7.5 (2.7–15.5)	7.5 (2.8–16.6)	7.5 (2.9–16.4)
Uzbekistan	65 to 69	6.9 (2.5–14.8)	6.9 (2.4–14.7)	6.9 (2.5–14.5)	7.0 (2.6–15.5)	7.0 (2.7–15.3)
Uzbekistan	70 to 74	5.5 (2.0–11.8)	5.4 (1.9–11.8)	5.4 (1.9–11.5)	5.5 (2.0–12.4)	5.5 (2.1–12.2)
Uzbekistan	75 to 79	4.9 (1.7–10.6)	4.9 (1.7–10.6)	4.9 (1.7–10.4)	4.9 (1.8–11.1)	4.9 (1.8–11.0)

Supplementary Table S11: Prevalence of male SVAC by age and location for 1990, 2000, 2010, 2020, and 2023						
**Location**	**Age Range**	**1990**	**2000**	**2010**	**2020**	**2023**
Uzbekistan	80 to 84	3.4 (1.2– 7.5)	3.4 (1.1– 7.4)	3.4 (1.2– 7.3)	3.4 (1.2– 7.8)	3.4 (1.3– 7.7)
Uzbekistan	85 to 89	3.0 (1.1– 6.7)	3.0 (1.0– 6.7)	3.0 (1.1– 6.5)	3.0 (1.1– 7.0)	3.0 (1.1– 7.0)
Uzbekistan	90 to 94	2.7 (1.0– 6.0)	2.7 (0.9– 6.0)	2.7 (0.9– 5.9)	2.7 (1.0– 6.4)	2.7 (1.0– 6.3)
Uzbekistan	95 plus	2.7 (0.9– 6.0)	2.7 (0.9– 6.0)	2.7 (0.9– 5.9)	2.7 (1.0– 6.3)	2.7 (1.0– 6.2)
Uzbekistan	Age-standardized	7.1 (2.6–15.1)	7.1 (2.5–15.1)	7.1 (2.6–14.8)	7.1 (2.7–15.8)	7.1 (2.7–15.6)
Uzbekistan	All age	7.1 (2.6–15.2)	7.2 (2.5–15.3)	7.2 (2.6–15.0)	7.3 (2.8–16.1)	7.3 (2.8–15.9)
Central Europe	20 to 24	14.6 (6.5–27.1)	14.4 (6.5–27.2)	14.6 (7.1–26.5)	14.7 (7.3–27.9)	14.6 (7.1–27.7)
Central Europe	25 to 29	14.9 (6.6–27.5)	14.7 (6.6–27.7)	14.9 (7.1–27.2)	15.0 (7.4–28.4)	15.0 (7.0–28.6)
Central Europe	30 to 34	15.8 (6.9–29.2)	15.7 (6.8–29.6)	15.7 (7.4–28.5)	15.9 (7.8–30.0)	15.9 (7.5–30.0)
Central Europe	35 to 39	15.8 (6.7–29.3)	16.0 (6.5–30.6)	15.9 (6.7–29.8)	16.0 (6.8–31.8)	16.0 (6.8–31.6)
Central Europe	40 to 44	16.0 (6.8–29.8)	16.2 (6.5–31.1)	16.3 (6.8–30.2)	16.4 (7.1–31.9)	16.4 (7.3–31.8)
Central Europe	45 to 49	15.6 (6.6–29.1)	15.7 (6.3–30.1)	15.9 (6.6–29.6)	15.9 (7.0–31.1)	16.0 (7.1–30.9)
Central Europe	50 to 54	15.3 (6.4–28.8)	15.2 (6.2–29.2)	15.5 (6.4–29.0)	15.6 (6.7–30.6)	15.6 (6.9–30.3)
Central Europe	55 to 59	14.8 (6.1–28.0)	14.7 (6.0–28.5)	14.9 (6.2–28.3)	15.2 (6.5–30.0)	15.2 (6.6–29.9)
Central Europe	60 to 64	14.3 (5.9–27.2)	14.4 (5.7–28.2)	14.4 (6.0–27.3)	14.6 (6.2–29.2)	14.7 (6.3–29.1)

Supplementary Table S11: Prevalence of male SVAC by age and location for 1990, 2000, 2010, 2020, and 2023						
**Location**	**Age Range**	**1990**	**2000**	**2010**	**2020**	**2023**
Central Europe	65 to 69	13.4 (5.4–25.7)	13.5 (5.2–26.8)	13.5 (5.4–26.0)	13.7 (5.7–27.6)	13.7 (5.8–27.5)
Central Europe	70 to 74	10.8 (4.2–21.3)	10.8 (4.1–22.2)	10.9 (4.2–21.8)	11.0 (4.5–22.8)	11.0 (4.5–22.9)
Central Europe	75 to 79	9.7 (3.8–19.4)	9.8 (3.6–20.2)	9.9 (3.8–19.9)	10.0 (4.1–20.9)	10.0 (4.1–20.7)
Central Europe	80 to 84	6.9 (2.6–14.1)	7.0 (2.5–14.9)	7.0 (2.6–14.6)	7.2 (2.8–15.6)	7.2 (2.9–15.5)
Central Europe	85 to 89	6.2 (2.3–12.9)	6.2 (2.2–13.4)	6.2 (2.2–13.1)	6.3 (2.4–14.1)	6.4 (2.4–14.0)
Central Europe	90 to 94	5.6 (2.1–11.7)	5.6 (1.9–12.2)	5.6 (2.0–11.9)	5.7 (2.1–12.8)	5.7 (2.2–12.7)
Central Europe	95 plus	5.6 (2.1–11.7)	5.6 (1.9–12.1)	5.6 (2.0–11.8)	5.6 (2.1–12.7)	5.6 (2.1–12.5)
Central Europe	Age-standardized	14.6 (6.2–27.3)	14.6 (6.0–28.1)	14.7 (6.5–27.4)	14.8 (6.7–28.9)	14.8 (6.6–28.7)
Central Europe	All age	14.7 (6.3–27.6)	14.7 (6.0–28.3)	14.6 (6.4–27.4)	14.6 (6.5–28.7)	14.6 (6.5–28.4)
Albania	20 to 24	16.9 (6.8–32.6)	16.9 (6.5–32.7)	17.0 (6.8–32.3)	17.1 (7.1–34.1)	17.1 (7.2–33.7)
Albania	25 to 29	17.1 (6.9–33.0)	17.1 (6.6–33.1)	17.3 (6.9–32.7)	17.4 (7.2–34.5)	17.4 (7.3–34.2)
Albania	30 to 34	18.3 (7.4–34.9)	18.3 (7.2–35.0)	18.5 (7.5–34.6)	18.6 (7.8–36.4)	18.6 (7.9–36.1)
Albania	35 to 39	18.3 (7.4–35.0)	18.4 (7.2–35.0)	18.5 (7.5–34.7)	18.6 (7.8–36.5)	18.6 (7.9–36.0)
Albania	40 to 44	18.8 (7.7–35.7)	18.8 (7.4–35.7)	19.0 (7.7–35.5)	19.1 (8.1–37.3)	19.1 (8.1–36.9)
Albania	45 to 49	18.2 (7.4–34.8)	18.3 (7.1–34.9)	18.4 (7.4–34.6)	18.6 (7.8–36.4)	18.5 (7.9–35.9)

Supplementary Table S11: Prevalence of male SVAC by age and location for 1990, 2000, 2010, 2020, and 2023						
**Location**	**Age Range**	**1990**	**2000**	**2010**	**2020**	**2023**
Albania	50 to 54	17.8 (7.2–34.1)	17.8 (6.9–34.1)	18.0 (7.2–33.9)	18.1 (7.6–35.7)	18.1 (7.7–35.3)
Albania	55 to 59	17.2 (6.9–33.1)	17.2 (6.7–33.2)	17.4 (6.9–32.9)	17.5 (7.3–34.7)	17.5 (7.3–34.3)
Albania	60 to 64	16.6 (6.6–32.2)	16.7 (6.4–32.3)	16.8 (6.7–32.0)	16.9 (7.0–33.8)	16.9 (7.1–33.4)
Albania	65 to 69	15.5 (6.1–30.4)	15.5 (5.9–30.4)	15.7 (6.2–30.2)	15.8 (6.5–31.9)	15.8 (6.5–31.5)
Albania	70 to 74	12.5 (4.8–25.2)	12.5 (4.6–25.2)	12.6 (4.8–25.0)	12.7 (5.1–26.5)	12.7 (5.1–26.2)
Albania	75 to 79	11.2 (4.3–23.0)	11.3 (4.1–23.0)	11.4 (4.3–22.8)	11.5 (4.5–24.2)	11.5 (4.6–24.0)
Albania	80 to 84	8.0 (2.9–16.9)	8.0 (2.8–16.9)	8.1 (3.0–16.7)	8.2 (3.1–17.9)	8.2 (3.2–17.6)
Albania	85 to 89	7.2 (2.6–15.3)	7.2 (2.5–15.3)	7.3 (2.7–15.2)	7.3 (2.8–16.2)	7.4 (2.8–16.0)
Albania	90 to 94	6.5 (2.4–13.9)	6.5 (2.3–14.0)	6.6 (2.4–13.8)	6.6 (2.5–14.8)	6.6 (2.5–14.6)
Albania	95 plus	6.5 (2.3–13.8)	6.5 (2.3–13.9)	6.5 (2.4–13.7)	6.6 (2.5–14.7)	6.6 (2.5–14.5)
Albania	Age-standardized	16.9 (6.8–32.7)	17.0 (6.6–32.7)	17.1 (6.8–32.4)	17.2 (7.2–34.1)	17.2 (7.2–33.8)
Albania	All age	17.3 (7.0–33.3)	17.3 (6.7–33.2)	17.1 (6.8–32.4)	16.9 (7.0–33.6)	16.8 (7.1–33.2)
Bosnia and Herzegovina	20 to 24	21.0 (8.8–39.1)	21.1 (8.5–39.2)	21.4 (8.9–39.0)	21.5 (9.3–40.9)	21.4 (9.4–40.5)
Bosnia and Herzegovina	25 to 29	21.1 (8.8–39.3)	21.2 (8.5–39.4)	21.5 (8.9–39.2)	21.6 (9.3–41.1)	21.6 (9.5–40.7)
Bosnia and Herzegovina	30 to 34	22.3 (9.4–41.1)	22.5 (9.2–41.2)	22.8 (9.6–41.1)	22.9 (10.0–42.9)	22.8 (10.1–42.5)

Supplementary Table S11: Prevalence of male SVAC by age and location for 1990, 2000, 2010, 2020, and 2023						
**Location**	**Age Range**	**1990**	**2000**	**2010**	**2020**	**2023**
Bosnia and Herzegovina	35 to 39	22.2 (9.4–40.9)	22.4 (9.1–41.1)	22.7 (9.5–40.9)	22.8 (10.0–42.8)	22.7 (10.1–42.4)
Bosnia and Herzegovina	40 to 44	22.6 (9.6–41.5)	22.8 (9.3–41.6)	23.1 (9.7–41.5)	23.2 (10.2–43.4)	23.1 (10.3–43.0)
Bosnia and Herzegovina	45 to 49	21.9 (9.2–40.4)	22.0 (8.9–40.6)	22.3 (9.4–40.4)	22.4 (9.8–42.3)	22.4 (9.9–41.9)
Bosnia and Herzegovina	50 to 54	21.3 (8.9–39.5)	21.4 (8.6–39.6)	21.7 (9.0–39.5)	21.8 (9.4–41.3)	21.8 (9.5–41.0)
Bosnia and Herzegovina	55 to 59	20.5 (8.5–38.4)	20.6 (8.3–38.5)	21.0 (8.7–38.4)	21.0 (9.0–40.2)	21.0 (9.1–39.8)
Bosnia and Herzegovina	60 to 64	19.8 (8.2–37.4)	20.0 (7.9–37.5)	20.3 (8.3–37.4)	20.3 (8.7–39.1)	20.3 (8.7–38.7)
Bosnia and Herzegovina	65 to 69	18.5 (7.5–35.3)	18.6 (7.3–35.4)	18.9 (7.7–35.3)	19.0 (8.0–37.0)	18.9 (8.1–36.6)
Bosnia and Herzegovina	70 to 74	15.0 (5.9–29.6)	15.1 (5.7–29.7)	15.3 (6.0–29.6)	15.4 (6.3–31.1)	15.3 (6.3–30.8)
Bosnia and Herzegovina	75 to 79	13.6 (5.3–27.1)	13.6 (5.1–27.2)	13.9 (5.4–27.1)	13.9 (5.6–28.6)	13.9 (5.7–28.3)
Bosnia and Herzegovina	80 to 84	9.7 (3.6–20.2)	9.8 (3.5–20.3)	9.9 (3.7–20.2)	10.0 (3.9–21.4)	10.0 (3.9–21.2)
Bosnia and Herzegovina	85 to 89	8.8 (3.3–18.4)	8.8 (3.2–18.5)	9.0 (3.3–18.4)	9.0 (3.5–19.5)	9.0 (3.5–19.3)
Bosnia and Herzegovina	90 to 94	8.0 (2.9–16.8)	8.0 (2.8–16.9)	8.1 (3.0–16.8)	8.2 (3.1–17.9)	8.2 (3.2–17.6)
Bosnia and Herzegovina	95 plus	7.9 (2.9–16.7)	7.9 (2.8–16.7)	8.1 (3.0–16.7)	8.1 (3.1–17.7)	8.1 (3.1–17.5)
Bosnia and Herzegovina	Age-standardized	20.6 (8.6–38.3)	20.7 (8.3–38.4)	21.0 (8.7–38.3)	21.0 (9.1–40.1)	21.0 (9.2–39.7)
Bosnia and Herzegovina	All age	21.1 (8.8–39.1)	21.0 (8.4–38.9)	21.0 (8.7–38.3)	20.7 (8.9–39.5)	20.5 (8.9–38.9)

Supplementary Table S11: Prevalence of male SVAC by age and location for 1990, 2000, 2010, 2020, and 2023						
**Location**	**Age Range**	**1990**	**2000**	**2010**	**2020**	**2023**
Bulgaria	20 to 24	13.9 (4.6–30.0)	13.8 (4.9–28.8)	13.6 (5.2–27.0)	13.5 (5.4–28.2)	13.5 (5.2–28.4)
Bulgaria	25 to 29	14.2 (4.7–30.6)	14.1 (5.0–29.4)	13.9 (5.3–27.5)	13.8 (5.5–28.8)	13.8 (5.3–29.0)
Bulgaria	30 to 34	15.4 (5.1–32.6)	15.2 (5.5–31.3)	15.0 (5.8–29.4)	14.9 (6.1–30.7)	15.0 (5.8–31.0)
Bulgaria	35 to 39	15.5 (5.2–32.9)	15.4 (5.5–31.6)	15.2 (5.9–29.7)	15.1 (6.1–31.0)	15.1 (5.9–31.3)
Bulgaria	40 to 44	16.0 (5.4–33.8)	15.9 (5.8–32.5)	15.7 (6.1–30.5)	15.6 (6.4–31.9)	15.6 (6.1–32.2)
Bulgaria	45 to 49	15.6 (5.2–33.1)	15.5 (5.6–31.8)	15.3 (5.9–29.9)	15.2 (6.2–31.2)	15.3 (5.9–31.5)
Bulgaria	50 to 54	15.3 (5.1–32.5)	15.2 (5.4–31.2)	14.9 (5.8–29.3)	14.9 (6.0–30.7)	14.9 (5.8–31.0)
Bulgaria	55 to 59	14.8 (4.9–31.7)	14.7 (5.3–30.4)	14.5 (5.6–28.5)	14.4 (5.8–29.9)	14.5 (5.6–30.2)
Bulgaria	60 to 64	14.4 (4.7–30.8)	14.2 (5.1–29.6)	14.0 (5.4–27.8)	14.0 (5.6–29.1)	14.0 (5.4–29.4)
Bulgaria	65 to 69	13.4 (4.4–29.1)	13.3 (4.7–27.9)	13.1 (5.0–26.1)	13.0 (5.2–27.4)	13.1 (5.0–27.7)
Bulgaria	70 to 74	10.8 (3.4–24.0)	10.6 (3.6–22.9)	10.4 (3.9–21.4)	10.4 (4.1–22.5)	10.5 (3.9–22.8)
Bulgaria	75 to 79	9.7 (3.0–21.9)	9.6 (3.2–20.9)	9.4 (3.5–19.5)	9.4 (3.6–20.5)	9.4 (3.5–20.8)
Bulgaria	80 to 84	6.9 (2.1–16.0)	6.8 (2.2–15.2)	6.6 (2.4–14.1)	6.6 (2.5–14.9)	6.7 (2.4–15.1)
Bulgaria	85 to 89	6.2 (1.9–14.5)	6.1 (2.0–13.8)	6.0 (2.1–12.8)	6.0 (2.2–13.5)	6.0 (2.1–13.7)
Bulgaria	90 to 94	5.6 (1.7–13.2)	5.5 (1.8–12.6)	5.4 (1.9–11.6)	5.4 (2.0–12.3)	5.4 (1.9–12.5)

Supplementary Table S11: Prevalence of male SVAC by age and location for 1990, 2000, 2010, 2020, and 2023						
**Location**	**Age Range**	**1990**	**2000**	**2010**	**2020**	**2023**
Bulgaria	95 plus	5.5 (1.6–13.1)	5.5 (1.8–12.4)	5.3 (1.9–11.5)	5.3 (2.0–12.2)	5.4 (1.9–12.4)
Bulgaria	Age-standardized	14.4 (4.7–30.8)	14.2 (5.1–29.5)	14.0 (5.4–27.7)	14.0 (5.6–28.9)	14.0 (5.4–29.2)
Bulgaria	All age	14.4 (4.7–30.8)	14.1 (5.0–29.3)	13.8 (5.3–27.4)	13.7 (5.5–28.4)	13.7 (5.3–28.7)
Croatia	20 to 24	13.0 (5.0–26.1)	13.0 (4.8–26.1)	13.0 (5.0–25.7)	13.1 (5.2–27.2)	13.1 (5.3–26.9)
Croatia	25 to 29	13.3 (5.2–26.7)	13.3 (5.0–26.7)	13.3 (5.1–26.2)	13.4 (5.4–27.8)	13.4 (5.5–27.5)
Croatia	30 to 34	14.4 (5.7–28.6)	14.4 (5.4–28.6)	14.5 (5.6–28.1)	14.6 (5.9–29.8)	14.6 (6.0–29.5)
Croatia	35 to 39	14.6 (5.7–28.9)	14.6 (5.5–28.8)	14.6 (5.7–28.4)	14.7 (6.0–30.1)	14.7 (6.1–29.8)
Croatia	40 to 44	15.1 (5.9–29.7)	15.1 (5.7–29.7)	15.1 (5.9–29.2)	15.2 (6.2–30.9)	15.2 (6.3–30.6)
Croatia	45 to 49	14.7 (5.8–29.1)	14.7 (5.5–29.0)	14.7 (5.7–28.6)	14.9 (6.0–30.3)	14.9 (6.1–30.0)
Croatia	50 to 54	14.4 (5.6–28.5)	14.4 (5.4–28.5)	14.4 (5.6–28.1)	14.6 (5.9–29.8)	14.6 (6.0–29.5)
Croatia	55 to 59	13.9 (5.4–27.8)	13.9 (5.2–27.7)	14.0 (5.4–27.3)	14.1 (5.7–29.0)	14.1 (5.8–28.7)
Croatia	60 to 64	13.5 (5.2–27.0)	13.5 (5.0–27.0)	13.6 (5.2–26.6)	13.7 (5.5–28.2)	13.7 (5.6–27.9)
Croatia	65 to 69	12.6 (4.8–25.4)	12.6 (4.7–25.4)	12.6 (4.8–25.0)	12.8 (5.1–26.6)	12.8 (5.2–26.3)
Croatia	70 to 74	10.1 (3.8–20.8)	10.1 (3.6–20.8)	10.1 (3.8–20.5)	10.2 (4.0–21.8)	10.2 (4.0–21.6)
Croatia	75 to 79	9.1 (3.4–18.9)	9.1 (3.2–18.9)	9.1 (3.4–18.6)	9.2 (3.5–19.9)	9.2 (3.6–19.7)

Supplementary Table S11: Prevalence of male SVAC by age and location for 1990, 2000, 2010, 2020, and 2023						
**Location**	**Age Range**	**1990**	**2000**	**2010**	**2020**	**2023**
Croatia	80 to 84	6.4 (2.3–13.7)	6.4 (2.2–13.7)	6.4 (2.3–13.5)	6.5 (2.4–14.5)	6.5 (2.5–14.3)
Croatia	85 to 89	5.8 (2.1–12.4)	5.7 (2.0–12.4)	5.8 (2.1–12.2)	5.8 (2.2–13.1)	5.8 (2.2–12.9)
Croatia	90 to 94	5.2 (1.9–11.3)	5.2 (1.8–11.2)	5.2 (1.9–11.0)	5.3 (2.0–11.9)	5.3 (2.0–11.7)
Croatia	95 plus	5.1 (1.8–11.2)	5.1 (1.8–11.1)	5.2 (1.8–11.0)	5.2 (1.9–11.8)	5.2 (2.0–11.6)
Croatia	Age-standardized	13.5 (5.2–26.9)	13.5 (5.0–26.9)	13.5 (5.2–26.5)	13.6 (5.5–28.1)	13.6 (5.6–27.8)
Croatia	All age	13.7 (5.3–27.2)	13.6 (5.1–27.0)	13.4 (5.2–26.3)	13.3 (5.3–27.5)	13.2 (5.4–27.1)
Czechia	20 to 24	14.2 (9.3–21.5)	13.9 (8.9–19.9)	13.7 (7.2–23.2)	13.8 (5.9–26.0)	13.9 (5.5–26.9)
Czechia	25 to 29	14.4 (9.5–21.6)	14.7 (9.9–20.2)	14.6 (8.4–23.3)	14.5 (6.4–26.5)	14.4 (5.9–26.9)
Czechia	30 to 34	13.9 (8.6–21.6)	13.8 (9.2–19.3)	14.0 (8.5–21.4)	14.6 (6.9–25.6)	14.7 (6.4–26.7)
Czechia	35 to 39	14.2 (8.5–22.2)	15.5 (10.5–22.0)	15.6 (9.9–22.2)	15.2 (8.2–25.3)	15.1 (7.8–26.7)
Czechia	40 to 44	12.8 (7.6–20.1)	13.5 (8.9–19.1)	14.4 (9.2–20.9)	14.9 (8.9–23.2)	15.0 (8.6–24.2)
Czechia	45 to 49	12.7 (7.3–20.1)	13.2 (8.5–18.9)	14.3 (9.0–20.8)	15.0 (9.1–22.9)	15.1 (8.7–24.1)
Czechia	50 to 54	12.6 (6.8–20.9)	12.6 (8.1–18.4)	13.7 (8.4–19.9)	14.4 (8.6–22.8)	14.5 (8.1–23.9)
Czechia	55 to 59	12.4 (6.0–21.6)	11.9 (7.3–17.9)	12.9 (8.0–19.2)	14.6 (8.3–22.7)	14.9 (7.9–24.1)
Czechia	60 to 64	12.5 (5.8–22.6)	11.9 (7.0–18.2)	12.2 (7.5–18.3)	13.1 (7.3–20.4)	13.3 (7.1–21.4)

Supplementary Table S11: Prevalence of male SVAC by age and location for 1990, 2000, 2010, 2020, and 2023						
**Location**	**Age Range**	**1990**	**2000**	**2010**	**2020**	**2023**
Czechia	65 to 69	12.5 (5.1–24.2)	12.1 (5.6–21.4)	12.1 (6.6–19.7)	12.6 (6.8–21.1)	12.8 (6.4–21.7)
Czechia	70 to 74	10.6 (4.1–21.6)	10.7 (4.5–20.3)	10.7 (5.5–18.4)	10.8 (5.5–19.4)	10.9 (5.0–20.5)
Czechia	75 to 79	9.6 (3.7–19.9)	9.9 (3.9–19.7)	10.2 (4.8–18.5)	10.3 (5.5–18.0)	10.3 (5.2–17.9)
Czechia	80 to 84	6.9 (2.5–14.5)	7.2 (2.7–15.3)	7.8 (3.2–15.4)	8.3 (4.0–15.8)	8.3 (4.0–15.4)
Czechia	85 to 89	6.0 (2.2–12.8)	6.0 (2.1–12.8)	6.0 (2.2–12.7)	6.1 (2.3–13.7)	6.1 (2.3–13.5)
Czechia	90 to 94	5.4 (1.9–11.6)	5.4 (1.9–11.7)	5.4 (1.9–11.5)	5.5 (2.1–12.4)	5.5 (2.1–12.3)
Czechia	95 plus	5.3 (1.9–11.5)	5.3 (1.8–11.6)	5.4 (1.9–11.4)	5.5 (2.0–12.3)	5.5 (2.1–12.2)
Czechia	Age-standardized	13.0 (8.4–19.6)	13.2 (9.5–17.7)	13.5 (10.1–17.4)	13.9 (8.9–20.3)	14.0 (8.2–21.5)
Czechia	All age	13.0 (8.3–19.6)	13.1 (9.5–17.7)	13.4 (9.8–17.4)	13.7 (8.6–20.4)	13.7 (8.2–21.2)
Hungary	20 to 24	14.3 (5.6–28.3)	14.3 (5.4–28.3)	14.4 (5.6–28.1)	14.5 (5.9–29.7)	14.5 (6.0–29.4)
Hungary	25 to 29	14.5 (5.7–28.7)	14.5 (5.5–28.8)	14.7 (5.7–28.5)	14.8 (6.0–30.2)	14.8 (6.1–29.9)
Hungary	30 to 34	15.5 (6.1–30.5)	15.6 (5.9–30.5)	15.7 (6.2–30.2)	15.9 (6.5–32.0)	15.9 (6.6–31.7)
Hungary	35 to 39	15.6 (6.2–30.5)	15.6 (6.0–30.6)	15.8 (6.2–30.3)	15.9 (6.5–32.1)	15.9 (6.6–31.7)
Hungary	40 to 44	16.0 (6.4–31.2)	16.0 (6.1–31.3)	16.2 (6.4–31.0)	16.3 (6.7–32.8)	16.3 (6.8–32.5)
Hungary	45 to 49	15.5 (6.1–30.4)	15.6 (5.9–30.5)	15.7 (6.2–30.2)	15.9 (6.5–32.0)	15.9 (6.6–31.7)

Supplementary Table S11: Prevalence of male SVAC by age and location for 1990, 2000, 2010, 2020, and 2023						
**Location**	**Age Range**	**1990**	**2000**	**2010**	**2020**	**2023**
Hungary	50 to 54	15.2 (6.0–29.8)	15.2 (5.8–29.9)	15.3 (6.0–29.6)	15.5 (6.3–31.4)	15.5 (6.4–31.1)
Hungary	55 to 59	14.7 (5.8–29.0)	14.7 (5.6–29.0)	14.8 (5.8–28.8)	15.0 (6.1–30.5)	15.0 (6.2–30.2)
Hungary	60 to 64	14.2 (5.6–28.2)	14.3 (5.4–28.3)	14.4 (5.6–28.0)	14.6 (5.9–29.8)	14.6 (6.0–29.4)
Hungary	65 to 69	13.3 (5.1–26.6)	13.3 (5.0–26.6)	13.4 (5.2–26.4)	13.6 (5.5–28.1)	13.6 (5.5–27.8)
Hungary	70 to 74	10.6 (4.0–21.9)	10.7 (3.9–21.9)	10.8 (4.0–21.7)	10.9 (4.3–23.2)	10.9 (4.3–22.9)
Hungary	75 to 79	9.6 (3.6–20.0)	9.6 (3.5–20.0)	9.7 (3.6–19.8)	9.9 (3.8–21.2)	9.9 (3.9–21.0)
Hungary	80 to 84	6.8 (2.5–14.5)	6.8 (2.4–14.6)	6.9 (2.5–14.4)	7.0 (2.6–15.5)	7.0 (2.7–15.3)
Hungary	85 to 89	6.1 (2.2–13.2)	6.1 (2.1–13.2)	6.2 (2.2–13.0)	6.3 (2.4–14.0)	6.3 (2.4–13.9)
Hungary	90 to 94	5.5 (2.0–12.0)	5.5 (1.9–12.0)	5.6 (2.0–11.8)	5.7 (2.1–12.8)	5.7 (2.2–12.6)
Hungary	95 plus	5.5 (2.0–11.8)	5.5 (1.9–11.9)	5.6 (2.0–11.7)	5.6 (2.1–12.7)	5.6 (2.1–12.5)
Hungary	Age-standardized	14.4 (5.7–28.5)	14.4 (5.5–28.5)	14.6 (5.7–28.3)	14.7 (6.0–30.0)	14.7 (6.1–29.7)
Hungary	All age	14.5 (5.7–28.6)	14.4 (5.5–28.5)	14.5 (5.7–28.2)	14.5 (5.9–29.6)	14.5 (6.0–29.3)
Montenegro	20 to 24	14.3 (5.6–28.3)	14.3 (5.4–28.3)	14.4 (5.6–28.1)	14.5 (5.9–29.7)	14.5 (6.0–29.4)
Montenegro	25 to 29	14.5 (5.7–28.7)	14.5 (5.5–28.8)	14.7 (5.7–28.5)	14.8 (6.0–30.2)	14.8 (6.1–29.9)
Montenegro	30 to 34	15.5 (6.1–30.5)	15.6 (5.9–30.5)	15.7 (6.2–30.2)	15.9 (6.5–32.0)	15.9 (6.6–31.7)

Supplementary Table S11: Prevalence of male SVAC by age and location for 1990, 2000, 2010, 2020, and 2023						
**Location**	**Age Range**	**1990**	**2000**	**2010**	**2020**	**2023**
Montenegro	35 to 39	15.6 (6.2–30.5)	15.6 (6.0–30.6)	15.8 (6.2–30.3)	15.9 (6.5–32.1)	15.9 (6.6–31.7)
Montenegro	40 to 44	16.0 (6.4–31.2)	16.0 (6.1–31.3)	16.2 (6.4–31.0)	16.3 (6.7–32.8)	16.3 (6.8–32.5)
Montenegro	45 to 49	15.5 (6.1–30.4)	15.6 (5.9–30.5)	15.7 (6.2–30.2)	15.9 (6.5–32.0)	15.9 (6.6–31.7)
Montenegro	50 to 54	15.2 (6.0–29.8)	15.2 (5.8–29.9)	15.3 (6.0–29.6)	15.5 (6.3–31.4)	15.5 (6.4–31.1)
Montenegro	55 to 59	14.7 (5.8–29.0)	14.7 (5.6–29.0)	14.8 (5.8–28.8)	15.0 (6.1–30.5)	15.0 (6.2–30.2)
Montenegro	60 to 64	14.2 (5.6–28.2)	14.3 (5.4–28.3)	14.4 (5.6–28.0)	14.6 (5.9–29.8)	14.6 (6.0–29.4)
Montenegro	65 to 69	13.3 (5.1–26.6)	13.3 (5.0–26.6)	13.4 (5.2–26.4)	13.6 (5.5–28.1)	13.6 (5.5–27.8)
Montenegro	70 to 74	10.6 (4.0–21.9)	10.7 (3.9–21.9)	10.8 (4.0–21.7)	10.9 (4.3–23.2)	10.9 (4.3–22.9)
Montenegro	75 to 79	9.6 (3.6–20.0)	9.6 (3.5–20.0)	9.7 (3.6–19.8)	9.9 (3.8–21.2)	9.9 (3.9–21.0)
Montenegro	80 to 84	6.8 (2.5–14.5)	6.8 (2.4–14.6)	6.9 (2.5–14.4)	7.0 (2.6–15.5)	7.0 (2.7–15.3)
Montenegro	85 to 89	6.1 (2.2–13.2)	6.1 (2.1–13.2)	6.2 (2.2–13.0)	6.3 (2.4–14.0)	6.3 (2.4–13.9)
Montenegro	90 to 94	5.5 (2.0–12.0)	5.5 (1.9–12.0)	5.6 (2.0–11.8)	5.7 (2.1–12.8)	5.7 (2.2–12.6)
Montenegro	95 plus	5.5 (2.0–11.8)	5.5 (1.9–11.9)	5.6 (2.0–11.7)	5.6 (2.1–12.7)	5.6 (2.1–12.5)
Montenegro	Age-standardized	14.4 (5.7–28.5)	14.4 (5.5–28.5)	14.6 (5.7–28.3)	14.7 (6.0–30.0)	14.7 (6.1–29.7)
Montenegro	All age	14.6 (5.7–28.8)	14.6 (5.5–28.8)	14.5 (5.7–28.2)	14.5 (5.9–29.6)	14.5 (6.0–29.3)

Supplementary Table S11: Prevalence of male SVAC by age and location for 1990, 2000, 2010, 2020, and 2023						
**Location**	**Age Range**	**1990**	**2000**	**2010**	**2020**	**2023**
North Macedonia	20 to 24	21.6 (12.5–33.0)	21.4 (14.0–30.3)	21.1 (16.7–26.1)	20.6 (11.8–31.8)	20.4 (10.4–34.4)
North Macedonia	25 to 29	20.7 (8.7–37.3)	20.6 (10.0–35.0)	20.7 (12.7–31.0)	20.9 (12.7–31.3)	20.9 (11.3–33.5)
North Macedonia	30 to 34	21.1 (7.8–40.6)	21.1 (8.6–38.5)	21.1 (11.3–34.6)	21.3 (14.7–30.1)	21.4 (14.1–31.0)
North Macedonia	35 to 39	20.5 (6.9–42.4)	20.4 (7.6–40.2)	20.2 (8.3–37.3)	20.5 (8.8–39.4)	20.6 (8.7–40.3)
North Macedonia	40 to 44	21.0 (7.2–42.2)	20.9 (7.9–40.5)	20.8 (8.6–38.2)	21.1 (9.1–40.3)	21.2 (9.1–41.3)
North Macedonia	45 to 49	20.5 (6.9–41.9)	20.4 (7.6–40.3)	20.2 (8.3–37.3)	20.5 (8.8–39.4)	20.7 (8.7–40.6)
North Macedonia	50 to 54	20.0 (6.7–41.7)	19.9 (7.4–39.4)	19.8 (8.1–36.7)	20.0 (8.5–38.7)	20.2 (8.5–39.5)
North Macedonia	55 to 59	19.3 (6.5–40.5)	19.2 (7.1–38.4)	19.2 (7.8–35.7)	19.4 (8.2–37.7)	19.5 (8.2–38.4)
North Macedonia	60 to 64	18.7 (6.3–39.8)	18.6 (6.9–37.7)	18.6 (7.5–34.8)	18.8 (7.9–36.8)	18.9 (7.9–37.7)
North Macedonia	65 to 69	17.4 (5.8–37.2)	17.4 (6.3–35.6)	17.3 (6.9–32.9)	17.6 (7.3–34.8)	17.7 (7.3–35.7)
North Macedonia	70 to 74	14.1 (4.5–31.3)	14.1 (4.9–29.5)	14.0 (5.4–27.4)	14.2 (5.7–29.1)	14.3 (5.7–30.2)
North Macedonia	75 to 79	12.8 (4.0–28.6)	12.7 (4.4–27.0)	12.7 (4.8–25.0)	12.8 (5.1–26.7)	12.9 (5.1–27.2)
North Macedonia	80 to 84	9.2 (2.8–21.1)	9.1 (3.0–20.0)	9.0 (3.3–18.5)	9.2 (3.5–19.9)	9.2 (3.5–20.2)
North Macedonia	85 to 89	8.3 (2.5–19.1)	8.2 (2.7–18.2)	8.2 (3.0–16.8)	8.3 (3.2–18.1)	8.3 (3.2–18.4)
North Macedonia	90 to 94	7.5 (2.3–17.9)	7.5 (2.5–16.7)	7.4 (2.7–15.3)	7.5 (2.8–16.5)	7.5 (2.8–16.8)

Supplementary Table S11: Prevalence of male SVAC by age and location for 1990, 2000, 2010, 2020, and 2023						
**Location**	**Age Range**	**1990**	**2000**	**2010**	**2020**	**2023**
North Macedonia	95 plus	7.4 (2.2–17.4)	7.4 (2.4–16.5)	7.3 (2.7–15.2)	7.4 (2.8–16.4)	7.5 (2.8–16.7)
North Macedonia	Age-standardized	19.6 (8.3–36.6)	19.5 (9.0–34.9)	19.4 (10.3–32.1)	19.6 (12.3–31.6)	19.6 (12.0–32.0)
North Macedonia	All age	19.9 (8.5–36.9)	19.7 (9.0–35.3)	19.4 (9.9–32.5)	19.2 (11.0–32.7)	19.1 (10.6–33.2)
Poland	20 to 24	14.3 (5.6–28.3)	14.3 (5.4–28.3)	14.4 (5.6–28.1)	14.5 (5.9–29.7)	14.5 (6.0–29.4)
Poland	25 to 29	14.5 (5.7–28.7)	14.5 (5.5–28.8)	14.7 (5.7–28.5)	14.8 (6.0–30.2)	14.8 (6.1–29.9)
Poland	30 to 34	15.5 (6.1–30.5)	15.6 (5.9–30.5)	15.7 (6.2–30.2)	15.9 (6.5–32.0)	15.9 (6.6–31.7)
Poland	35 to 39	15.6 (6.2–30.5)	15.6 (6.0–30.6)	15.8 (6.2–30.3)	15.9 (6.5–32.1)	15.9 (6.6–31.7)
Poland	40 to 44	16.0 (6.4–31.2)	16.0 (6.1–31.3)	16.2 (6.4–31.0)	16.3 (6.7–32.8)	16.3 (6.8–32.5)
Poland	45 to 49	15.5 (6.1–30.4)	15.6 (5.9–30.5)	15.7 (6.2–30.2)	15.9 (6.5–32.0)	15.9 (6.6–31.7)
Poland	50 to 54	15.2 (6.0–29.8)	15.2 (5.8–29.9)	15.3 (6.0–29.6)	15.5 (6.3–31.4)	15.5 (6.4–31.1)
Poland	55 to 59	14.7 (5.8–29.0)	14.7 (5.6–29.0)	14.8 (5.8–28.8)	15.0 (6.1–30.5)	15.0 (6.2–30.2)
Poland	60 to 64	14.2 (5.6–28.2)	14.3 (5.4–28.3)	14.4 (5.6–28.0)	14.6 (5.9–29.8)	14.6 (6.0–29.4)
Poland	65 to 69	13.3 (5.1–26.6)	13.3 (5.0–26.6)	13.4 (5.2–26.4)	13.6 (5.5–28.1)	13.6 (5.5–27.8)
Poland	70 to 74	10.6 (4.0–21.9)	10.7 (3.9–21.9)	10.8 (4.0–21.7)	10.9 (4.3–23.2)	10.9 (4.3–22.9)
Poland	75 to 79	9.6 (3.6–20.0)	9.6 (3.5–20.0)	9.7 (3.6–19.8)	9.9 (3.8–21.2)	9.9 (3.9–21.0)

Supplementary Table S11: Prevalence of male SVAC by age and location for 1990, 2000, 2010, 2020, and 2023						
**Location**	**Age Range**	**1990**	**2000**	**2010**	**2020**	**2023**
Poland	80 to 84	6.8 (2.5–14.5)	6.8 (2.4–14.6)	6.9 (2.5–14.4)	7.0 (2.6–15.5)	7.0 (2.7–15.3)
Poland	85 to 89	6.1 (2.2–13.2)	6.1 (2.1–13.2)	6.2 (2.2–13.0)	6.3 (2.4–14.0)	6.3 (2.4–13.9)
Poland	90 to 94	5.5 (2.0–12.0)	5.5 (1.9–12.0)	5.6 (2.0–11.8)	5.7 (2.1–12.8)	5.7 (2.2–12.6)
Poland	95 plus	5.5 (2.0–11.8)	5.5 (1.9–11.9)	5.6 (2.0–11.7)	5.6 (2.1–12.7)	5.6 (2.1–12.5)
Poland	Age-standardized	14.4 (5.7–28.5)	14.4 (5.5–28.5)	14.6 (5.7–28.3)	14.7 (6.0–30.0)	14.7 (6.1–29.7)
Poland	All age	14.6 (5.7–28.9)	14.6 (5.5–28.8)	14.6 (5.7–28.3)	14.6 (5.9–29.8)	14.6 (6.0–29.4)
Romania	20 to 24	13.4 (5.2–26.8)	13.4 (5.0–26.8)	13.4 (5.2–26.4)	13.5 (5.4–28.0)	13.5 (5.5–27.6)
Romania	25 to 29	13.7 (5.3–27.4)	13.7 (5.1–27.3)	13.8 (5.3–26.9)	13.9 (5.6–28.5)	13.9 (5.6–28.2)
Romania	30 to 34	14.8 (5.8–29.3)	14.8 (5.6–29.3)	14.9 (5.8–28.8)	15.0 (6.1–30.5)	15.0 (6.2–30.2)
Romania	35 to 39	15.0 (5.9–29.5)	15.0 (5.7–29.5)	15.0 (5.9–29.1)	15.1 (6.2–30.8)	15.1 (6.2–30.5)
Romania	40 to 44	15.5 (6.1–30.4)	15.5 (5.9–30.3)	15.5 (6.1–29.9)	15.6 (6.4–31.6)	15.6 (6.5–31.3)
Romania	45 to 49	15.1 (5.9–29.7)	15.1 (5.7–29.7)	15.1 (5.9–29.3)	15.3 (6.2–31.0)	15.3 (6.3–30.7)
Romania	50 to 54	14.7 (5.8–29.1)	14.7 (5.6–29.1)	14.8 (5.8–28.7)	14.9 (6.1–30.4)	14.9 (6.1–30.1)
Romania	55 to 59	14.3 (5.6–28.4)	14.3 (5.4–28.3)	14.3 (5.6–27.9)	14.5 (5.9–29.6)	14.5 (5.9–29.3)
Romania	60 to 64	13.9 (5.4–27.6)	13.8 (5.2–27.6)	13.9 (5.4–27.2)	14.0 (5.7–28.8)	14.0 (5.7–28.6)

Supplementary Table S11: Prevalence of male SVAC by age and location for 1990, 2000, 2010, 2020, and 2023						
**Location**	**Age Range**	**1990**	**2000**	**2010**	**2020**	**2023**
Romania	65 to 69	12.9 (5.0–26.0)	12.9 (4.8–25.9)	13.0 (5.0–25.6)	13.1 (5.2–27.2)	13.1 (5.3–26.9)
Romania	70 to 74	10.3 (3.9–21.3)	10.3 (3.7–21.3)	10.4 (3.9–20.9)	10.5 (4.1–22.3)	10.5 (4.1–22.1)
Romania	75 to 79	9.3 (3.5–19.4)	9.3 (3.3–19.3)	9.3 (3.5–19.1)	9.4 (3.6–20.4)	9.4 (3.7–20.1)
Romania	80 to 84	6.6 (2.4–14.0)	6.6 (2.3–14.0)	6.6 (2.4–13.8)	6.7 (2.5–14.8)	6.7 (2.5–14.6)
Romania	85 to 89	5.9 (2.1–12.7)	5.9 (2.0–12.7)	5.9 (2.1–12.5)	6.0 (2.2–13.4)	6.0 (2.3–13.3)
Romania	90 to 94	5.3 (1.9–11.5)	5.3 (1.8–11.5)	5.3 (1.9–11.3)	5.4 (2.0–12.2)	5.4 (2.0–12.0)
Romania	95 plus	5.3 (1.9–11.4)	5.3 (1.8–11.4)	5.3 (1.9–11.2)	5.4 (2.0–12.1)	5.4 (2.0–11.9)
Romania	Age-standardized	13.9 (5.4–27.5)	13.8 (5.2–27.5)	13.9 (5.4–27.1)	14.0 (5.7–28.7)	14.0 (5.7–28.4)
Romania	All age	14.0 (5.5–27.8)	13.9 (5.2–27.6)	13.8 (5.4–27.0)	13.9 (5.6–28.5)	13.8 (5.7–28.1)
Serbia	20 to 24	15.0 (5.9–29.5)	15.0 (5.7–29.5)	15.0 (5.9–29.1)	15.1 (6.2–30.8)	15.1 (6.2–30.5)
Serbia	25 to 29	15.3 (6.0–30.0)	15.3 (5.8–30.0)	15.3 (6.0–29.6)	15.5 (6.3–31.3)	15.4 (6.4–31.0)
Serbia	30 to 34	16.4 (6.5–31.9)	16.4 (6.3–31.9)	16.5 (6.5–31.5)	16.6 (6.9–33.3)	16.6 (6.9–32.9)
Serbia	35 to 39	16.5 (6.6–32.1)	16.5 (6.3–32.1)	16.6 (6.6–31.7)	16.7 (6.9–33.4)	16.7 (7.0–33.1)
Serbia	40 to 44	17.0 (6.8–32.8)	17.0 (6.6–32.8)	17.1 (6.8–32.5)	17.2 (7.2–34.3)	17.2 (7.2–33.9)
Serbia	45 to 49	16.5 (6.6–32.1)	16.5 (6.4–32.1)	16.6 (6.6–31.7)	16.8 (6.9–33.5)	16.8 (7.0–33.2)

Supplementary Table S11: Prevalence of male SVAC by age and location for 1990, 2000, 2010, 2020, and 2023						
**Location**	**Age Range**	**1990**	**2000**	**2010**	**2020**	**2023**
Serbia	50 to 54	16.1 (6.4–31.4)	16.1 (6.2–31.4)	16.3 (6.4–31.1)	16.4 (6.7–32.9)	16.4 (6.8–32.5)
Serbia	55 to 59	15.6 (6.2–30.6)	15.6 (6.0–30.6)	15.7 (6.2–30.3)	15.8 (6.5–32.0)	15.8 (6.6–31.7)
Serbia	60 to 64	15.1 (6.0–29.8)	15.1 (5.7–29.8)	15.2 (6.0–29.4)	15.4 (6.3–31.1)	15.4 (6.3–30.8)
Serbia	65 to 69	14.1 (5.5–28.0)	14.1 (5.3–28.0)	14.2 (5.5–27.7)	14.3 (5.8–29.3)	14.3 (5.9–29.0)
Serbia	70 to 74	11.3 (4.3–23.1)	11.3 (4.1–23.1)	11.4 (4.3–22.8)	11.5 (4.5–24.2)	11.5 (4.6–23.9)
Serbia	75 to 79	10.2 (3.8–21.0)	10.2 (3.7–21.0)	10.3 (3.8–20.8)	10.4 (4.0–22.1)	10.4 (4.1–21.9)
Serbia	80 to 84	7.2 (2.6–15.3)	7.2 (2.5–15.3)	7.3 (2.6–15.1)	7.3 (2.8–16.2)	7.3 (2.8–16.0)
Serbia	85 to 89	6.5 (2.4–13.9)	6.5 (2.3–13.9)	6.5 (2.4–13.7)	6.6 (2.5–14.7)	6.6 (2.5–14.5)
Serbia	90 to 94	5.9 (2.1–12.6)	5.9 (2.0–12.6)	5.9 (2.1–12.5)	6.0 (2.2–13.4)	6.0 (2.3–13.2)
Serbia	95 plus	5.8 (2.1–12.5)	5.8 (2.0–12.5)	5.9 (2.1–12.4)	5.9 (2.2–13.3)	5.9 (2.2–13.1)
Serbia	Age-standardized	15.3 (6.0–29.9)	15.3 (5.8–29.9)	15.4 (6.0–29.6)	15.5 (6.3–31.2)	15.5 (6.4–30.9)
Serbia	All age	15.4 (6.1–30.3)	15.3 (5.8–30.0)	15.2 (6.0–29.3)	15.2 (6.2–30.8)	15.2 (6.3–30.4)
Slovakia	20 to 24	14.3 (5.6–28.3)	14.3 (5.4–28.3)	14.4 (5.6–28.1)	14.5 (5.9–29.7)	14.5 (6.0–29.4)
Slovakia	25 to 29	14.5 (5.7–28.7)	14.5 (5.5–28.8)	14.7 (5.7–28.5)	14.8 (6.0–30.2)	14.8 (6.1–29.9)
Slovakia	30 to 34	15.5 (6.1–30.5)	15.6 (5.9–30.5)	15.7 (6.2–30.2)	15.9 (6.5–32.0)	15.9 (6.6–31.7)

Supplementary Table S11: Prevalence of male SVAC by age and location for 1990, 2000, 2010, 2020, and 2023						
**Location**	**Age Range**	**1990**	**2000**	**2010**	**2020**	**2023**
Slovakia	35 to 39	15.6 (6.2–30.5)	15.6 (6.0–30.6)	15.8 (6.2–30.3)	15.9 (6.5–32.1)	15.9 (6.6–31.7)
Slovakia	40 to 44	16.0 (6.4–31.2)	16.0 (6.1–31.3)	16.2 (6.4–31.0)	16.3 (6.7–32.8)	16.3 (6.8–32.5)
Slovakia	45 to 49	15.5 (6.1–30.4)	15.6 (5.9–30.5)	15.7 (6.2–30.2)	15.9 (6.5–32.0)	15.9 (6.6–31.7)
Slovakia	50 to 54	15.2 (6.0–29.8)	15.2 (5.8–29.9)	15.3 (6.0–29.6)	15.5 (6.3–31.4)	15.5 (6.4–31.1)
Slovakia	55 to 59	14.7 (5.8–29.0)	14.7 (5.6–29.0)	14.8 (5.8–28.8)	15.0 (6.1–30.5)	15.0 (6.2–30.2)
Slovakia	60 to 64	14.2 (5.6–28.2)	14.3 (5.4–28.3)	14.4 (5.6–28.0)	14.6 (5.9–29.8)	14.6 (6.0–29.4)
Slovakia	65 to 69	13.3 (5.1–26.6)	13.3 (5.0–26.6)	13.4 (5.2–26.4)	13.6 (5.5–28.1)	13.6 (5.5–27.8)
Slovakia	70 to 74	10.6 (4.0–21.9)	10.7 (3.9–21.9)	10.8 (4.0–21.7)	10.9 (4.3–23.2)	10.9 (4.3–22.9)
Slovakia	75 to 79	9.6 (3.6–20.0)	9.6 (3.5–20.0)	9.7 (3.6–19.8)	9.9 (3.8–21.2)	9.9 (3.9–21.0)
Slovakia	80 to 84	6.8 (2.5–14.5)	6.8 (2.4–14.6)	6.9 (2.5–14.4)	7.0 (2.6–15.5)	7.0 (2.7–15.3)
Slovakia	85 to 89	6.1 (2.2–13.2)	6.1 (2.1–13.2)	6.2 (2.2–13.0)	6.3 (2.4–14.0)	6.3 (2.4–13.9)
Slovakia	90 to 94	5.5 (2.0–12.0)	5.5 (1.9–12.0)	5.6 (2.0–11.8)	5.7 (2.1–12.8)	5.7 (2.2–12.6)
Slovakia	95 plus	5.5 (2.0–11.8)	5.5 (1.9–11.9)	5.6 (2.0–11.7)	5.6 (2.1–12.7)	5.6 (2.1–12.5)
Slovakia	Age-standardized	14.4 (5.7–28.5)	14.4 (5.5–28.5)	14.6 (5.7–28.3)	14.7 (6.0–30.0)	14.7 (6.1–29.7)
Slovakia	All age	14.6 (5.7–28.7)	14.6 (5.5–28.8)	14.7 (5.7–28.4)	14.7 (6.0–29.9)	14.6 (6.0–29.5)

Supplementary Table S11: Prevalence of male SVAC by age and location for 1990, 2000, 2010, 2020, and 2023						
**Location**	**Age Range**	**1990**	**2000**	**2010**	**2020**	**2023**
Slovenia	20 to 24	14.3 (5.6–28.3)	14.3 (5.4–28.3)	14.4 (5.6–28.1)	14.5 (5.9–29.7)	14.5 (6.0–29.4)
Slovenia	25 to 29	14.5 (5.7–28.7)	14.5 (5.5–28.8)	14.7 (5.7–28.5)	14.8 (6.0–30.2)	14.8 (6.1–29.9)
Slovenia	30 to 34	15.5 (6.1–30.5)	15.6 (5.9–30.5)	15.7 (6.2–30.2)	15.9 (6.5–32.0)	15.9 (6.6–31.7)
Slovenia	35 to 39	15.6 (6.2–30.5)	15.6 (6.0–30.6)	15.8 (6.2–30.3)	15.9 (6.5–32.1)	15.9 (6.6–31.7)
Slovenia	40 to 44	16.0 (6.4–31.2)	16.0 (6.1–31.3)	16.2 (6.4–31.0)	16.3 (6.7–32.8)	16.3 (6.8–32.5)
Slovenia	45 to 49	15.5 (6.1–30.4)	15.6 (5.9–30.5)	15.7 (6.2–30.2)	15.9 (6.5–32.0)	15.9 (6.6–31.7)
Slovenia	50 to 54	15.2 (6.0–29.8)	15.2 (5.8–29.9)	15.3 (6.0–29.6)	15.5 (6.3–31.4)	15.5 (6.4–31.1)
Slovenia	55 to 59	14.7 (5.8–29.0)	14.7 (5.6–29.0)	14.8 (5.8–28.8)	15.0 (6.1–30.5)	15.0 (6.2–30.2)
Slovenia	60 to 64	14.2 (5.6–28.2)	14.3 (5.4–28.3)	14.4 (5.6–28.0)	14.6 (5.9–29.8)	14.6 (6.0–29.4)
Slovenia	65 to 69	13.3 (5.1–26.6)	13.3 (5.0–26.6)	13.4 (5.2–26.4)	13.6 (5.5–28.1)	13.6 (5.5–27.8)
Slovenia	70 to 74	10.6 (4.0–21.9)	10.7 (3.9–21.9)	10.8 (4.0–21.7)	10.9 (4.3–23.2)	10.9 (4.3–22.9)
Slovenia	75 to 79	9.6 (3.6–20.0)	9.6 (3.5–20.0)	9.7 (3.6–19.8)	9.9 (3.8–21.2)	9.9 (3.9–21.0)
Slovenia	80 to 84	6.8 (2.5–14.5)	6.8 (2.4–14.6)	6.9 (2.5–14.4)	7.0 (2.6–15.5)	7.0 (2.7–15.3)
Slovenia	85 to 89	6.1 (2.2–13.2)	6.1 (2.1–13.2)	6.2 (2.2–13.0)	6.3 (2.4–14.0)	6.3 (2.4–13.9)
Slovenia	90 to 94	5.5 (2.0–12.0)	5.5 (1.9–12.0)	5.6 (2.0–11.8)	5.7 (2.1–12.8)	5.7 (2.2–12.6)

Supplementary Table S11: Prevalence of male SVAC by age and location for 1990, 2000, 2010, 2020, and 2023						
**Location**	**Age Range**	**1990**	**2000**	**2010**	**2020**	**2023**
Slovenia	95 plus	5.5 (2.0–11.8)	5.5 (1.9–11.9)	5.6 (2.0–11.7)	5.6 (2.1–12.7)	5.6 (2.1–12.5)
Slovenia	Age-standardized	14.4 (5.7–28.5)	14.4 (5.5–28.5)	14.6 (5.7–28.3)	14.7 (6.0–30.0)	14.7 (6.1–29.7)
Slovenia	All age	14.6 (5.7–28.8)	14.6 (5.5–28.8)	14.5 (5.7–28.2)	14.4 (5.9–29.4)	14.3 (5.9–29.0)
Eastern Europe	20 to 24	12.7 (4.9–25.4)	12.6 (4.7–25.4)	12.7 (4.9–24.9)	12.7 (5.1–26.3)	12.7 (5.2–26.1)
Eastern Europe	25 to 29	12.9 (5.0–26.0)	12.9 (4.8–25.9)	12.9 (5.0–25.5)	13.0 (5.2–27.0)	13.0 (5.2–26.7)
Eastern Europe	30 to 34	13.9 (5.4–27.8)	13.9 (5.2–27.7)	14.0 (5.4–27.3)	14.0 (5.7–28.9)	14.0 (5.7–28.6)
Eastern Europe	35 to 39	14.0 (5.5–27.9)	14.0 (5.3–27.9)	14.1 (5.4–27.5)	14.1 (5.7–29.0)	14.1 (5.8–28.7)
Eastern Europe	40 to 44	14.5 (5.7–28.7)	14.5 (5.5–28.6)	14.5 (5.6–28.2)	14.6 (5.9–29.9)	14.6 (6.0–29.5)
Eastern Europe	45 to 49	14.1 (5.5–28.0)	14.1 (5.3–28.0)	14.1 (5.5–27.6)	14.2 (5.7–29.2)	14.2 (5.8–28.9)
Eastern Europe	50 to 54	13.8 (5.4–27.5)	13.8 (5.2–27.4)	13.8 (5.3–27.0)	13.9 (5.6–28.6)	13.9 (5.7–28.3)
Eastern Europe	55 to 59	13.3 (5.2–26.7)	13.3 (5.0–26.7)	13.4 (5.1–26.3)	13.5 (5.4–27.9)	13.5 (5.5–27.6)
Eastern Europe	60 to 64	12.9 (5.0–26.0)	12.9 (4.8–25.9)	13.0 (5.0–25.6)	13.1 (5.2–27.1)	13.1 (5.3–26.8)
Eastern Europe	65 to 69	12.0 (4.6–24.4)	12.0 (4.4–24.4)	12.1 (4.6–24.0)	12.2 (4.8–25.5)	12.2 (4.9–25.2)
Eastern Europe	70 to 74	9.6 (3.6–20.0)	9.6 (3.4–19.9)	9.6 (3.6–19.6)	9.7 (3.8–20.9)	9.7 (3.8–20.7)
Eastern Europe	75 to 79	8.7 (3.2–18.1)	8.6 (3.1–18.1)	8.7 (3.2–17.8)	8.7 (3.4–19.0)	8.7 (3.4–18.8)

Supplementary Table S11: Prevalence of male SVAC by age and location for 1990, 2000, 2010, 2020, and 2023						
**Location**	**Age Range**	**1990**	**2000**	**2010**	**2020**	**2023**
Eastern Europe	80 to 84	6.1 (2.2–13.1)	6.1 (2.1–13.1)	6.1 (2.2–12.9)	6.2 (2.3–13.8)	6.2 (2.3–13.6)
Eastern Europe	85 to 89	5.5 (2.0–11.8)	5.5 (1.9–11.8)	5.5 (2.0–11.6)	5.5 (2.1–12.5)	5.5 (2.1–12.3)
Eastern Europe	90 to 94	4.9 (1.8–10.7)	4.9 (1.7–10.7)	5.0 (1.8–10.5)	5.0 (1.8–11.3)	5.0 (1.9–11.2)
Eastern Europe	95 plus	4.9 (1.8–10.7)	4.9 (1.7–10.6)	4.9 (1.7–10.4)	4.9 (1.8–11.2)	4.9 (1.9–11.1)
Eastern Europe	Age-standardized	13.0 (5.0–26.0)	13.0 (4.8–26.0)	13.0 (5.0–25.6)	13.1 (5.3–27.1)	13.1 (5.3–26.8)
Eastern Europe	All age	13.2 (5.1–26.5)	13.2 (4.9–26.3)	13.1 (5.0–25.8)	13.2 (5.3–27.2)	13.1 (5.3–26.9)
Belarus	20 to 24	12.7 (4.9–25.5)	12.6 (4.7–25.5)	12.7 (4.8–25.1)	12.8 (5.1–26.6)	12.8 (5.1–26.3)
Belarus	25 to 29	12.9 (5.0–26.0)	12.9 (4.8–25.9)	12.9 (5.0–25.5)	13.0 (5.2–27.0)	13.0 (5.2–26.7)
Belarus	30 to 34	13.9 (5.4–27.8)	13.9 (5.2–27.7)	14.0 (5.4–27.3)	14.0 (5.7–28.8)	14.0 (5.7–28.5)
Belarus	35 to 39	14.0 (5.5–27.9)	14.0 (5.3–27.9)	14.1 (5.4–27.5)	14.1 (5.7–29.0)	14.1 (5.8–28.7)
Belarus	40 to 44	14.5 (5.7–28.7)	14.5 (5.4–28.6)	14.5 (5.6–28.2)	14.6 (5.9–29.8)	14.6 (6.0–29.5)
Belarus	45 to 49	14.1 (5.5–28.0)	14.1 (5.3–28.0)	14.1 (5.5–27.6)	14.2 (5.7–29.2)	14.2 (5.8–28.9)
Belarus	50 to 54	13.8 (5.4–27.5)	13.8 (5.1–27.4)	13.8 (5.3–27.0)	13.9 (5.6–28.6)	13.9 (5.7–28.3)
Belarus	55 to 59	13.3 (5.2–26.7)	13.3 (5.0–26.7)	13.4 (5.1–26.3)	13.5 (5.4–27.8)	13.5 (5.5–27.5)
Belarus	60 to 64	12.9 (5.0–26.0)	12.9 (4.8–25.9)	13.0 (5.0–25.6)	13.0 (5.2–27.1)	13.0 (5.3–26.8)

Supplementary Table S11: Prevalence of male SVAC by age and location for 1990, 2000, 2010, 2020, and 2023						
**Location**	**Age Range**	**1990**	**2000**	**2010**	**2020**	**2023**
Belarus	65 to 69	12.0 (4.6–24.4)	12.0 (4.4–24.4)	12.1 (4.6–24.0)	12.1 (4.8–25.5)	12.2 (4.9–25.2)
Belarus	70 to 74	9.6 (3.6–19.9)	9.6 (3.4–19.9)	9.6 (3.6–19.6)	9.7 (3.8–20.9)	9.7 (3.8–20.6)
Belarus	75 to 79	8.7 (3.2–18.1)	8.6 (3.1–18.1)	8.7 (3.2–17.8)	8.7 (3.4–19.0)	8.7 (3.4–18.8)
Belarus	80 to 84	6.1 (2.2–13.1)	6.1 (2.1–13.1)	6.1 (2.2–12.8)	6.1 (2.3–13.8)	6.2 (2.3–13.6)
Belarus	85 to 89	5.5 (2.0–11.8)	5.5 (1.9–11.8)	5.5 (2.0–11.6)	5.5 (2.1–12.4)	5.5 (2.1–12.3)
Belarus	90 to 94	4.9 (1.8–10.7)	4.9 (1.7–10.7)	4.9 (1.8–10.5)	5.0 (1.8–11.3)	5.0 (1.9–11.2)
Belarus	95 plus	4.9 (1.7–10.6)	4.9 (1.7–10.6)	4.9 (1.7–10.4)	4.9 (1.8–11.2)	4.9 (1.9–11.1)
Belarus	Age-standardized	13.0 (5.0–26.0)	13.0 (4.8–26.0)	13.0 (5.0–25.6)	13.1 (5.2–27.1)	13.1 (5.3–26.8)
Belarus	All age	13.2 (5.1–26.4)	13.1 (4.9–26.3)	13.1 (5.0–25.8)	13.2 (5.3–27.3)	13.1 (5.3–26.9)
Estonia	20 to 24	12.7 (4.9–25.5)	12.6 (4.7–25.5)	12.7 (4.8–25.1)	12.8 (5.1–26.6)	12.8 (5.1–26.3)
Estonia	25 to 29	12.9 (5.0–26.0)	12.9 (4.8–25.9)	12.9 (5.0–25.5)	13.0 (5.2–27.0)	13.0 (5.2–26.7)
Estonia	30 to 34	13.9 (5.4–27.8)	13.9 (5.2–27.7)	14.0 (5.4–27.3)	14.0 (5.7–28.8)	14.0 (5.7–28.5)
Estonia	35 to 39	14.0 (5.5–27.9)	14.0 (5.3–27.9)	14.1 (5.4–27.5)	14.1 (5.7–29.0)	14.1 (5.8–28.7)
Estonia	40 to 44	14.5 (5.7–28.7)	14.5 (5.4–28.6)	14.5 (5.6–28.2)	14.6 (5.9–29.8)	14.6 (6.0–29.5)
Estonia	45 to 49	14.1 (5.5–28.0)	14.1 (5.3–28.0)	14.1 (5.5–27.6)	14.2 (5.7–29.2)	14.2 (5.8–28.9)

Supplementary Table S11: Prevalence of male SVAC by age and location for 1990, 2000, 2010, 2020, and 2023						
**Location**	**Age Range**	**1990**	**2000**	**2010**	**2020**	**2023**
Estonia	50 to 54	13.8 (5.4–27.5)	13.8 (5.1–27.4)	13.8 (5.3–27.0)	13.9 (5.6–28.6)	13.9 (5.7–28.3)
Estonia	55 to 59	13.3 (5.2–26.7)	13.3 (5.0–26.7)	13.4 (5.1–26.3)	13.5 (5.4–27.8)	13.5 (5.5–27.5)
Estonia	60 to 64	12.9 (5.0–26.0)	12.9 (4.8–25.9)	13.0 (5.0–25.6)	13.0 (5.2–27.1)	13.0 (5.3–26.8)
Estonia	65 to 69	12.0 (4.6–24.4)	12.0 (4.4–24.4)	12.1 (4.6–24.0)	12.1 (4.8–25.5)	12.2 (4.9–25.2)
Estonia	70 to 74	9.6 (3.6–19.9)	9.6 (3.4–19.9)	9.6 (3.6–19.6)	9.7 (3.8–20.9)	9.7 (3.8–20.6)
Estonia	75 to 79	8.7 (3.2–18.1)	8.6 (3.1–18.1)	8.7 (3.2–17.8)	8.7 (3.4–19.0)	8.7 (3.4–18.8)
Estonia	80 to 84	6.1 (2.2–13.1)	6.1 (2.1–13.1)	6.1 (2.2–12.8)	6.1 (2.3–13.8)	6.2 (2.3–13.6)
Estonia	85 to 89	5.5 (2.0–11.8)	5.5 (1.9–11.8)	5.5 (2.0–11.6)	5.5 (2.1–12.4)	5.5 (2.1–12.3)
Estonia	90 to 94	4.9 (1.8–10.7)	4.9 (1.7–10.7)	4.9 (1.8–10.5)	5.0 (1.8–11.3)	5.0 (1.9–11.2)
Estonia	95 plus	4.9 (1.7–10.6)	4.9 (1.7–10.6)	4.9 (1.7–10.4)	4.9 (1.8–11.2)	4.9 (1.9–11.1)
Estonia	Age-standardized	13.0 (5.0–26.0)	13.0 (4.8–26.0)	13.0 (5.0–25.6)	13.1 (5.2–27.1)	13.1 (5.3–26.8)
Estonia	All age	13.2 (5.1–26.4)	13.0 (4.9–26.1)	13.0 (5.0–25.5)	12.9 (5.2–26.8)	12.9 (5.2–26.5)
Latvia	20 to 24	12.7 (4.9–25.5)	12.6 (4.7–25.5)	12.7 (4.8–25.1)	12.8 (5.1–26.6)	12.8 (5.1–26.3)
Latvia	25 to 29	12.9 (5.0–26.0)	12.9 (4.8–25.9)	12.9 (5.0–25.5)	13.0 (5.2–27.0)	13.0 (5.2–26.7)
Latvia	30 to 34	13.9 (5.4–27.8)	13.9 (5.2–27.7)	14.0 (5.4–27.3)	14.0 (5.7–28.8)	14.0 (5.7–28.5)

Supplementary Table S11: Prevalence of male SVAC by age and location for 1990, 2000, 2010, 2020, and 2023						
**Location**	**Age Range**	**1990**	**2000**	**2010**	**2020**	**2023**
Latvia	35 to 39	14.0 (5.5–27.9)	14.0 (5.3–27.9)	14.1 (5.4–27.5)	14.1 (5.7–29.0)	14.1 (5.8–28.7)
Latvia	40 to 44	14.5 (5.7–28.7)	14.5 (5.4–28.6)	14.5 (5.6–28.2)	14.6 (5.9–29.8)	14.6 (6.0–29.5)
Latvia	45 to 49	14.1 (5.5–28.0)	14.1 (5.3–28.0)	14.1 (5.5–27.6)	14.2 (5.7–29.2)	14.2 (5.8–28.9)
Latvia	50 to 54	13.8 (5.4–27.5)	13.8 (5.1–27.4)	13.8 (5.3–27.0)	13.9 (5.6–28.6)	13.9 (5.7–28.3)
Latvia	55 to 59	13.3 (5.2–26.7)	13.3 (5.0–26.7)	13.4 (5.1–26.3)	13.5 (5.4–27.8)	13.5 (5.5–27.5)
Latvia	60 to 64	12.9 (5.0–26.0)	12.9 (4.8–25.9)	13.0 (5.0–25.6)	13.0 (5.2–27.1)	13.0 (5.3–26.8)
Latvia	65 to 69	12.0 (4.6–24.4)	12.0 (4.4–24.4)	12.1 (4.6–24.0)	12.1 (4.8–25.5)	12.2 (4.9–25.2)
Latvia	70 to 74	9.6 (3.6–19.9)	9.6 (3.4–19.9)	9.6 (3.6–19.6)	9.7 (3.8–20.9)	9.7 (3.8–20.6)
Latvia	75 to 79	8.7 (3.2–18.1)	8.6 (3.1–18.1)	8.7 (3.2–17.8)	8.7 (3.4–19.0)	8.7 (3.4–18.8)
Latvia	80 to 84	6.1 (2.2–13.1)	6.1 (2.1–13.1)	6.1 (2.2–12.8)	6.1 (2.3–13.8)	6.2 (2.3–13.6)
Latvia	85 to 89	5.5 (2.0–11.8)	5.5 (1.9–11.8)	5.5 (2.0–11.6)	5.5 (2.1–12.4)	5.5 (2.1–12.3)
Latvia	90 to 94	4.9 (1.8–10.7)	4.9 (1.7–10.7)	4.9 (1.8–10.5)	5.0 (1.8–11.3)	5.0 (1.9–11.2)
Latvia	95 plus	4.9 (1.7–10.6)	4.9 (1.7–10.6)	4.9 (1.7–10.4)	4.9 (1.8–11.2)	4.9 (1.9–11.1)
Latvia	Age-standardized	13.0 (5.0–26.0)	13.0 (4.8–26.0)	13.0 (5.0–25.6)	13.1 (5.2–27.1)	13.1 (5.3–26.8)
Latvia	All age	13.1 (5.1–26.3)	13.1 (4.9–26.2)	12.9 (5.0–25.5)	12.9 (5.2–26.8)	12.9 (5.2–26.5)

Supplementary Table S11: Prevalence of male SVAC by age and location for 1990, 2000, 2010, 2020, and 2023						
**Location**	**Age Range**	**1990**	**2000**	**2010**	**2020**	**2023**
Lithuania	20 to 24	12.7 (4.9–25.5)	12.6 (4.7–25.5)	12.7 (4.8–25.1)	12.8 (5.1–26.6)	12.8 (5.1–26.3)
Lithuania	25 to 29	12.9 (5.0–26.0)	12.9 (4.8–25.9)	12.9 (5.0–25.5)	13.0 (5.2–27.0)	13.0 (5.2–26.7)
Lithuania	30 to 34	13.9 (5.4–27.8)	13.9 (5.2–27.7)	14.0 (5.4–27.3)	14.0 (5.7–28.8)	14.0 (5.7–28.5)
Lithuania	35 to 39	14.0 (5.5–27.9)	14.0 (5.3–27.9)	14.1 (5.4–27.5)	14.1 (5.7–29.0)	14.1 (5.8–28.7)
Lithuania	40 to 44	14.5 (5.7–28.7)	14.5 (5.4–28.6)	14.5 (5.6–28.2)	14.6 (5.9–29.8)	14.6 (6.0–29.5)
Lithuania	45 to 49	14.1 (5.5–28.0)	14.1 (5.3–28.0)	14.1 (5.5–27.6)	14.2 (5.7–29.2)	14.2 (5.8–28.9)
Lithuania	50 to 54	13.8 (5.4–27.5)	13.8 (5.1–27.4)	13.8 (5.3–27.0)	13.9 (5.6–28.6)	13.9 (5.7–28.3)
Lithuania	55 to 59	13.3 (5.2–26.7)	13.3 (5.0–26.7)	13.4 (5.1–26.3)	13.5 (5.4–27.8)	13.5 (5.5–27.5)
Lithuania	60 to 64	12.9 (5.0–26.0)	12.9 (4.8–25.9)	13.0 (5.0–25.6)	13.0 (5.2–27.1)	13.0 (5.3–26.8)
Lithuania	65 to 69	12.0 (4.6–24.4)	12.0 (4.4–24.4)	12.1 (4.6–24.0)	12.1 (4.8–25.5)	12.2 (4.9–25.2)
Lithuania	70 to 74	9.6 (3.6–19.9)	9.6 (3.4–19.9)	9.6 (3.6–19.6)	9.7 (3.8–20.9)	9.7 (3.8–20.6)
Lithuania	75 to 79	8.7 (3.2–18.1)	8.6 (3.1–18.1)	8.7 (3.2–17.8)	8.7 (3.4–19.0)	8.7 (3.4–18.8)
Lithuania	80 to 84	6.1 (2.2–13.1)	6.1 (2.1–13.1)	6.1 (2.2–12.8)	6.1 (2.3–13.8)	6.2 (2.3–13.6)
Lithuania	85 to 89	5.5 (2.0–11.8)	5.5 (1.9–11.8)	5.5 (2.0–11.6)	5.5 (2.1–12.4)	5.5 (2.1–12.3)
Lithuania	90 to 94	4.9 (1.8–10.7)	4.9 (1.7–10.7)	4.9 (1.8–10.5)	5.0 (1.8–11.3)	5.0 (1.9–11.2)

Supplementary Table S11: Prevalence of male SVAC by age and location for 1990, 2000, 2010, 2020, and 2023						
**Location**	**Age Range**	**1990**	**2000**	**2010**	**2020**	**2023**
Lithuania	95 plus	4.9 (1.7–10.6)	4.9 (1.7–10.6)	4.9 (1.7–10.4)	4.9 (1.8–11.2)	4.9 (1.9–11.1)
Lithuania	Age-standardized	13.0 (5.0–26.0)	13.0 (4.8–26.0)	13.0 (5.0–25.6)	13.1 (5.2–27.1)	13.1 (5.3–26.8)
Lithuania	All age	13.1 (5.1–26.2)	13.1 (4.9–26.1)	12.9 (5.0–25.5)	12.9 (5.2–26.8)	12.9 (5.2–26.5)
Moldova	20 to 24	12.5 (4.9–24.7)	12.1 (5.1–22.8)	11.4 (6.1–19.3)	11.0 (7.0–16.3)	11.1 (6.7–17.8)
Moldova	25 to 29	13.2 (5.1–26.5)	13.2 (4.9–26.4)	13.2 (5.1–26.0)	13.3 (5.3–27.6)	13.3 (5.4–27.3)
Moldova	30 to 34	14.3 (5.6–28.4)	14.3 (5.4–28.3)	14.3 (5.6–27.9)	14.4 (5.8–29.5)	14.4 (5.9–29.2)
Moldova	35 to 39	14.5 (5.7–28.6)	14.4 (5.4–28.6)	14.5 (5.6–28.1)	14.6 (5.9–29.8)	14.6 (6.0–29.5)
Moldova	40 to 44	15.0 (5.9–29.5)	14.9 (5.6–29.4)	15.0 (5.8–29.0)	15.1 (6.1–30.6)	15.1 (6.2–30.3)
Moldova	45 to 49	14.6 (5.7–28.8)	14.6 (5.5–28.8)	14.6 (5.7–28.4)	14.7 (6.0–30.0)	14.7 (6.0–29.7)
Moldova	50 to 54	14.3 (5.6–28.3)	14.2 (5.4–28.2)	14.3 (5.5–27.8)	14.4 (5.8–29.4)	14.4 (5.9–29.1)
Moldova	55 to 59	13.8 (5.4–27.5)	13.8 (5.2–27.5)	13.8 (5.3–27.1)	13.9 (5.6–28.6)	13.9 (5.7–28.3)
Moldova	60 to 64	13.4 (5.2–26.8)	13.4 (5.0–26.7)	13.4 (5.1–26.3)	13.5 (5.4–27.9)	13.5 (5.5–27.6)
Moldova	65 to 69	12.4 (4.8–25.1)	12.4 (4.6–25.1)	12.4 (4.7–24.7)	12.5 (5.0–26.2)	12.5 (5.0–25.9)
Moldova	70 to 74	9.9 (3.7–20.5)	9.9 (3.6–20.5)	9.9 (3.7–20.1)	10.0 (3.9–21.4)	10.0 (3.9–21.2)
Moldova	75 to 79	8.9 (3.3–18.6)	8.9 (3.2–18.6)	8.9 (3.3–18.3)	9.0 (3.5–19.5)	9.0 (3.5–19.3)

Supplementary Table S11: Prevalence of male SVAC by age and location for 1990, 2000, 2010, 2020, and 2023						
**Location**	**Age Range**	**1990**	**2000**	**2010**	**2020**	**2023**
Moldova	80 to 84	6.3 (2.3–13.5)	6.3 (2.2–13.4)	6.3 (2.3–13.2)	6.3 (2.4–14.1)	6.3 (2.4–14.0)
Moldova	85 to 89	5.6 (2.0–12.2)	5.6 (1.9–12.2)	5.6 (2.0–11.9)	5.7 (2.1–12.8)	5.7 (2.2–12.6)
Moldova	90 to 94	5.1 (1.8–11.1)	5.1 (1.7–11.0)	5.1 (1.8–10.8)	5.1 (1.9–11.6)	5.1 (1.9–11.5)
Moldova	95 plus	5.0 (1.8–11.0)	5.0 (1.7–10.9)	5.0 (1.8–10.7)	5.1 (1.9–11.5)	5.1 (1.9–11.4)
Moldova	Age-standardized	13.3 (5.2–26.3)	13.2 (5.1–26.4)	13.2 (5.3–25.2)	13.2 (5.8–26.1)	13.2 (5.9–25.9)
Moldova	All age	13.6 (5.3–26.8)	13.4 (5.2–26.7)	13.3 (5.4–25.3)	13.4 (5.6–27.0)	13.4 (5.8–26.8)
Russia	20 to 24	12.7 (4.9–25.5)	12.6 (4.7–25.5)	12.7 (4.8–25.1)	12.8 (5.1–26.6)	12.8 (5.1–26.3)
Russia	25 to 29	12.9 (5.0–26.0)	12.9 (4.8–25.9)	12.9 (5.0–25.5)	13.0 (5.2–27.0)	13.0 (5.2–26.7)
Russia	30 to 34	13.9 (5.4–27.8)	13.9 (5.2–27.7)	14.0 (5.4–27.3)	14.0 (5.7–28.8)	14.0 (5.7–28.5)
Russia	35 to 39	14.0 (5.5–27.9)	14.0 (5.3–27.9)	14.1 (5.4–27.5)	14.1 (5.7–29.0)	14.1 (5.8–28.7)
Russia	40 to 44	14.5 (5.7–28.7)	14.5 (5.4–28.6)	14.5 (5.6–28.2)	14.6 (5.9–29.8)	14.6 (6.0–29.5)
Russia	45 to 49	14.1 (5.5–28.0)	14.1 (5.3–28.0)	14.1 (5.5–27.6)	14.2 (5.7–29.2)	14.2 (5.8–28.9)
Russia	50 to 54	13.8 (5.4–27.5)	13.8 (5.1–27.4)	13.8 (5.3–27.0)	13.9 (5.6–28.6)	13.9 (5.7–28.3)
Russia	55 to 59	13.3 (5.2–26.7)	13.3 (5.0–26.7)	13.4 (5.1–26.3)	13.5 (5.4–27.8)	13.5 (5.5–27.5)
Russia	60 to 64	12.9 (5.0–26.0)	12.9 (4.8–25.9)	13.0 (5.0–25.6)	13.0 (5.2–27.1)	13.0 (5.3–26.8)

Supplementary Table S11: Prevalence of male SVAC by age and location for 1990, 2000, 2010, 2020, and 2023						
**Location**	**Age Range**	**1990**	**2000**	**2010**	**2020**	**2023**
Russia	65 to 69	12.0 (4.6–24.4)	12.0 (4.4–24.4)	12.1 (4.6–24.0)	12.1 (4.8–25.5)	12.2 (4.9–25.2)
Russia	70 to 74	9.6 (3.6–19.9)	9.6 (3.4–19.9)	9.6 (3.6–19.6)	9.7 (3.8–20.9)	9.7 (3.8–20.6)
Russia	75 to 79	8.7 (3.2–18.1)	8.6 (3.1–18.1)	8.7 (3.2–17.8)	8.7 (3.4–19.0)	8.7 (3.4–18.8)
Russia	80 to 84	6.1 (2.2–13.1)	6.1 (2.1–13.1)	6.1 (2.2–12.8)	6.1 (2.3–13.8)	6.2 (2.3–13.6)
Russia	85 to 89	5.5 (2.0–11.8)	5.5 (1.9–11.8)	5.5 (2.0–11.6)	5.5 (2.1–12.4)	5.5 (2.1–12.3)
Russia	90 to 94	4.9 (1.8–10.7)	4.9 (1.7–10.7)	4.9 (1.8–10.5)	5.0 (1.8–11.3)	5.0 (1.9–11.2)
Russia	95 plus	4.9 (1.7–10.6)	4.9 (1.7–10.6)	4.9 (1.7–10.4)	4.9 (1.8–11.2)	4.9 (1.9–11.1)
Russia	Age-standardized	13.0 (5.0–26.0)	13.0 (4.8–26.0)	13.0 (5.0–25.6)	13.1 (5.2–27.1)	13.1 (5.3–26.8)
Russia	All age	13.3 (5.1–26.6)	13.2 (4.9–26.4)	13.1 (5.0–25.8)	13.2 (5.3–27.3)	13.2 (5.3–26.9)
Ukraine	20 to 24	12.7 (4.9–25.5)	12.6 (4.7–25.5)	12.7 (4.8–25.1)	12.8 (5.1–26.6)	12.8 (5.1–26.3)
Ukraine	25 to 29	12.9 (5.0–26.0)	12.9 (4.8–25.9)	12.9 (5.0–25.5)	13.0 (5.2–27.0)	13.0 (5.2–26.7)
Ukraine	30 to 34	13.9 (5.4–27.8)	13.9 (5.2–27.7)	14.0 (5.4–27.3)	14.0 (5.7–28.8)	14.0 (5.7–28.5)
Ukraine	35 to 39	14.0 (5.5–27.9)	14.0 (5.3–27.9)	14.1 (5.4–27.5)	14.1 (5.7–29.0)	14.1 (5.8–28.7)
Ukraine	40 to 44	14.5 (5.7–28.7)	14.5 (5.4–28.6)	14.5 (5.6–28.2)	14.6 (5.9–29.8)	14.6 (6.0–29.5)
Ukraine	45 to 49	14.1 (5.5–28.0)	14.1 (5.3–28.0)	14.1 (5.5–27.6)	14.2 (5.7–29.2)	14.2 (5.8–28.9)

Supplementary Table S11: Prevalence of male SVAC by age and location for 1990, 2000, 2010, 2020, and 2023						
**Location**	**Age Range**	**1990**	**2000**	**2010**	**2020**	**2023**
Ukraine	50 to 54	13.8 (5.4–27.5)	13.8 (5.1–27.4)	13.8 (5.3–27.0)	13.9 (5.6–28.6)	13.9 (5.7–28.3)
Ukraine	55 to 59	13.3 (5.2–26.7)	13.3 (5.0–26.7)	13.4 (5.1–26.3)	13.5 (5.4–27.8)	13.5 (5.5–27.5)
Ukraine	60 to 64	12.9 (5.0–26.0)	12.9 (4.8–25.9)	13.0 (5.0–25.6)	13.0 (5.2–27.1)	13.0 (5.3–26.8)
Ukraine	65 to 69	12.0 (4.6–24.4)	12.0 (4.4–24.4)	12.1 (4.6–24.0)	12.1 (4.8–25.5)	12.2 (4.9–25.2)
Ukraine	70 to 74	9.6 (3.6–19.9)	9.6 (3.4–19.9)	9.6 (3.6–19.6)	9.7 (3.8–20.9)	9.7 (3.8–20.6)
Ukraine	75 to 79	8.7 (3.2–18.1)	8.6 (3.1–18.1)	8.7 (3.2–17.8)	8.7 (3.4–19.0)	8.7 (3.4–18.8)
Ukraine	80 to 84	6.1 (2.2–13.1)	6.1 (2.1–13.1)	6.1 (2.2–12.8)	6.1 (2.3–13.8)	6.2 (2.3–13.6)
Ukraine	85 to 89	5.5 (2.0–11.8)	5.5 (1.9–11.8)	5.5 (2.0–11.6)	5.5 (2.1–12.4)	5.5 (2.1–12.3)
Ukraine	90 to 94	4.9 (1.8–10.7)	4.9 (1.7–10.7)	4.9 (1.8–10.5)	5.0 (1.8–11.3)	5.0 (1.9–11.2)
Ukraine	95 plus	4.9 (1.7–10.6)	4.9 (1.7–10.6)	4.9 (1.7–10.4)	4.9 (1.8–11.2)	4.9 (1.9–11.1)
Ukraine	Age-standardized	13.0 (5.0–26.0)	13.0 (4.8–26.0)	13.0 (5.0–25.6)	13.1 (5.2–27.1)	13.1 (5.3–26.8)
Ukraine	All age	13.2 (5.1–26.4)	13.1 (4.9–26.2)	13.0 (5.0–25.6)	13.1 (5.3–27.1)	13.1 (5.3–26.8)
High income	20 to 24	14.1 (8.9–22.1)	14.5 (9.7–22.0)	14.1 (9.5–21.1)	13.9 (8.8–22.1)	13.9 (8.7–22.5)
High income	25 to 29	14.2 (8.9–22.4)	14.6 (9.8–22.2)	14.9 (10.8–21.2)	14.3 (9.4–22.3)	14.1 (9.2–22.6)
High income	30 to 34	15.4 (10.0–23.6)	15.6 (10.6–23.5)	15.9 (11.5–22.6)	15.7 (10.5–24.2)	15.5 (10.4–24.4)

Supplementary Table S11: Prevalence of male SVAC by age and location for 1990, 2000, 2010, 2020, and 2023						
**Location**	**Age Range**	**1990**	**2000**	**2010**	**2020**	**2023**
High income	35 to 39	15.1 (9.4–23.8)	15.3 (10.3–23.0)	15.6 (10.9–22.9)	16.1 (11.0–24.6)	16.1 (11.0–25.0)
High income	40 to 44	16.4 (10.6–25.3)	16.5 (11.3–24.5)	16.6 (11.8–24.1)	16.8 (12.4–24.5)	16.9 (12.2–24.3)
High income	45 to 49	15.7 (9.9–24.7)	15.8 (10.5–24.0)	16.0 (11.2–23.2)	16.4 (11.9–24.6)	16.6 (11.9–24.6)
High income	50 to 54	15.1 (8.8–25.0)	15.3 (10.1–23.8)	15.7 (11.0–22.7)	15.8 (11.3–23.9)	15.9 (11.4–23.9)
High income	55 to 59	15.6 (9.3–25.5)	15.6 (10.1–24.4)	15.9 (11.0–23.1)	16.0 (11.8–23.6)	16.0 (11.7–23.7)
High income	60 to 64	14.9 (8.7–24.4)	15.0 (9.1–24.2)	15.3 (10.3–22.9)	15.7 (10.9–23.9)	15.8 (10.7–24.0)
High income	65 to 69	14.1 (8.0–23.1)	14.2 (8.3–23.1)	14.6 (9.6–22.4)	14.9 (10.7–22.5)	14.9 (11.0–22.4)
High income	70 to 74	10.7 (5.5–18.5)	11.2 (5.9–19.7)	11.9 (6.9–19.6)	12.5 (8.3–19.9)	12.5 (8.5–19.9)
High income	75 to 79	9.2 (4.1–17.3)	9.6 (4.7–17.7)	10.5 (5.6–18.0)	11.2 (6.8–19.3)	11.3 (6.8–19.1)
High income	80 to 84	7.1 (3.0–13.9)	7.7 (3.8–14.0)	8.4 (4.7–14.3)	8.6 (5.1–15.2)	8.5 (5.0–15.1)
High income	85 to 89	6.6 (2.7–13.1)	7.1 (3.3–13.3)	7.9 (4.7–13.3)	7.9 (4.7–14.0)	7.7 (4.4–13.8)
High income	90 to 94	6.3 (2.6–12.7)	6.8 (3.0–13.2)	7.6 (4.7–12.1)	6.9 (4.9–11.3)	6.7 (4.5–11.4)
High income	95 plus	6.7 (2.8–13.2)	7.2 (3.2–13.9)	7.4 (4.4–12.1)	7.0 (5.1–10.8)	6.7 (4.6–10.9)
High income	Age-standardized	14.5 (9.0–23.1)	14.7 (9.7–22.7)	15.0 (10.3–22.0)	15.0 (10.5–23.1)	15.0 (10.4–23.2)
High income	All age	14.5 (9.0–23.1)	14.7 (9.6–22.7)	14.9 (10.2–21.9)	14.8 (10.4–22.7)	14.8 (10.4–22.8)

Supplementary Table S11: Prevalence of male SVAC by age and location for 1990, 2000, 2010, 2020, and 2023						
**Location**	**Age Range**	**1990**	**2000**	**2010**	**2020**	**2023**
Australasia	20 to 24	13.2 (8.1–20.2)	13.0 (9.4–17.2)	14.7 (10.9–19.2)	18.6 (14.2–24.6)	19.4 (14.3–26.1)
Australasia	25 to 29	13.2 (8.2–20.5)	12.4 (9.2–16.0)	12.3 (8.9–16.2)	13.0 (8.7–18.3)	13.0 (8.4–19.1)
Australasia	30 to 34	16.0 (9.7–24.1)	15.5 (11.3–20.5)	16.1 (12.5–20.1)	20.1 (15.8–25.8)	21.2 (15.4–28.5)
Australasia	35 to 39	15.2 (8.3–24.7)	14.4 (10.2–19.4)	14.3 (10.3–18.9)	15.7 (11.3–21.2)	16.1 (11.2–22.5)
Australasia	40 to 44	19.1 (10.7–30.1)	19.3 (14.6–24.6)	19.6 (14.8–25.0)	20.1 (15.7–25.3)	20.0 (15.3–25.7)
Australasia	45 to 49	17.7 (9.7–28.4)	17.5 (12.9–22.8)	18.0 (13.1–23.4)	19.6 (14.8–25.4)	19.9 (14.7–26.6)
Australasia	50 to 54	17.7 (9.7–28.6)	17.0 (12.2–22.6)	16.6 (11.8–22.0)	18.0 (12.8–24.8)	18.3 (12.8–25.7)
Australasia	55 to 59	16.8 (8.6–28.1)	17.7 (12.4–24.1)	19.4 (14.8–24.8)	20.3 (15.2–26.6)	20.2 (14.6–27.2)
Australasia	60 to 64	16.4 (7.7–29.4)	16.9 (10.3–25.2)	17.8 (13.3–23.3)	18.5 (13.7–24.1)	18.5 (13.4–25.2)
Australasia	65 to 69	16.4 (7.8–28.9)	17.9 (11.0–26.2)	19.9 (15.2–25.7)	21.8 (16.9–27.6)	21.9 (16.4–28.4)
Australasia	70 to 74	11.7 (5.2–21.8)	12.0 (6.5–19.8)	13.0 (8.4–19.0)	14.1 (9.7–19.8)	14.2 (9.7–20.7)
Australasia	75 to 79	9.1 (3.6–18.5)	8.5 (3.7–16.0)	8.3 (4.0–14.7)	8.6 (4.1–16.2)	8.7 (3.9–16.6)
Australasia	80 to 84	6.5 (2.3–13.9)	6.2 (2.2–12.9)	6.0 (2.3–12.3)	6.2 (2.4–13.3)	6.3 (2.5–13.5)
Australasia	85 to 89	5.8 (2.1–12.5)	5.5 (2.0–11.6)	5.4 (2.0–11.0)	5.5 (2.1–11.9)	5.6 (2.2–12.0)
Australasia	90 to 94	5.2 (1.9–11.3)	4.9 (1.8–10.5)	4.8 (1.8–10.0)	4.9 (1.9–10.7)	5.0 (1.9–10.9)

Supplementary Table S11: Prevalence of male SVAC by age and location for 1990, 2000, 2010, 2020, and 2023						
**Location**	**Age Range**	**1990**	**2000**	**2010**	**2020**	**2023**
Australasia	95 plus	5.1 (1.8–11.0)	4.8 (1.7–10.2)	4.8 (1.8– 9.9)	4.9 (1.9–10.5)	4.9 (1.9–10.7)
Australasia	Age-standardized	15.3 (8.6–24.5)	15.1 (10.7–20.3)	15.7 (11.8–20.2)	17.4 (13.2–22.7)	17.7 (13.0–23.9)
Australasia	All age	15.4 (8.7–24.7)	15.2 (10.8–20.6)	15.8 (11.8–20.5)	17.2 (12.9–22.6)	17.4 (12.7–23.6)
Australia	20 to 24	11.5 (5.9–19.4)	11.2 (8.0–15.1)	13.0 (8.6–18.2)	17.6 (13.2–23.0)	18.7 (13.5–25.3)
Australia	25 to 29	11.6 (5.9–20.0)	10.7 (7.3–14.9)	10.5 (6.9–14.8)	10.9 (7.2–16.0)	11.1 (7.0–16.8)
Australia	30 to 34	14.6 (7.9–23.7)	14.1 (10.5–18.3)	14.9 (11.1–19.2)	19.5 (15.2–24.8)	20.7 (15.1–27.7)
Australia	35 to 39	13.7 (7.3–22.6)	12.8 (9.3–17.0)	12.9 (9.1–17.3)	14.5 (10.6–19.3)	14.9 (10.2–21.0)
Australia	40 to 44	18.3 (10.4–29.0)	18.7 (14.7–23.1)	19.3 (14.4–24.7)	19.8 (15.9–24.6)	19.7 (15.0–25.3)
Australia	45 to 49	16.5 (9.2–26.6)	16.4 (12.7–20.7)	17.1 (12.4–22.5)	19.2 (14.8–24.5)	19.6 (14.2–26.6)
Australia	50 to 54	16.7 (9.2–26.9)	15.8 (12.1–20.2)	15.4 (10.9–20.5)	16.9 (12.6–22.1)	17.3 (12.2–23.8)
Australia	55 to 59	15.8 (8.3–26.2)	17.1 (12.6–22.5)	19.2 (14.3–24.5)	19.9 (15.3–25.7)	19.8 (14.0–26.8)
Australia	60 to 64	15.3 (7.0–27.8)	16.0 (9.9–23.6)	17.1 (12.5–22.4)	17.8 (13.4–23.1)	17.7 (12.3–24.6)
Australia	65 to 69	15.2 (7.0–27.3)	16.8 (10.5–24.4)	19.2 (14.3–24.8)	21.6 (16.8–27.1)	21.7 (15.8–28.8)
Australia	70 to 74	10.6 (4.6–20.6)	11.1 (6.3–17.6)	12.2 (8.2–17.3)	13.5 (9.6–18.4)	13.7 (9.1–19.5)
Australia	75 to 79	7.8 (3.0–15.9)	7.2 (3.2–13.3)	6.9 (3.5–12.1)	7.3 (3.5–13.5)	7.5 (3.3–14.6)

Supplementary Table S11: Prevalence of male SVAC by age and location for 1990, 2000, 2010, 2020, and 2023						
**Location**	**Age Range**	**1990**	**2000**	**2010**	**2020**	**2023**
Australia	80 to 84	5.5 (1.9–12.2)	5.2 (1.9–11.0)	5.1 (1.9–10.5)	5.3 (2.1–11.5)	5.4 (2.1–11.7)
Australia	85 to 89	4.9 (1.7–10.8)	4.6 (1.7– 9.9)	4.5 (1.7– 9.4)	4.7 (1.9–10.3)	4.8 (1.9–10.5)
Australia	90 to 94	4.4 (1.5– 9.7)	4.2 (1.5– 8.9)	4.1 (1.6– 8.5)	4.3 (1.7– 9.3)	4.3 (1.7– 9.5)
Australia	95 plus	4.3 (1.5– 9.5)	4.1 (1.5– 8.8)	4.0 (1.5– 8.4)	4.2 (1.6– 9.2)	4.3 (1.7– 9.3)
Australia	Age-standardized	14.0 (7.4–23.4)	13.8 (10.0–18.4)	14.6 (10.6–19.2)	16.5 (12.6–21.5)	16.9 (12.1–23.0)
Australia	All age	14.1 (7.4–23.6)	14.0 (10.1–18.7)	14.7 (10.8–19.4)	16.3 (12.4–21.3)	16.6 (11.9–22.5)
New Zealand	20 to 24	21.2 (13.3–32.4)	22.0 (13.3–32.6)	23.1 (12.9–36.5)	23.0 (10.8–39.7)	22.8 (10.4–40.3)
New Zealand	25 to 29	21.3 (13.7–31.9)	21.1 (14.6–28.2)	22.0 (13.3–33.4)	22.5 (10.7–38.5)	22.5 (10.0–39.0)
New Zealand	30 to 34	22.8 (13.2–34.8)	22.3 (14.7–31.0)	22.6 (14.0–33.0)	23.2 (11.2–39.4)	23.3 (10.7–40.7)
New Zealand	35 to 39	23.0 (12.5–36.3)	22.0 (13.9–31.7)	21.4 (14.3–29.7)	22.1 (11.5–36.1)	22.4 (10.6–38.4)
New Zealand	40 to 44	23.2 (11.8–38.3)	21.8 (13.5–31.9)	20.8 (14.1–28.6)	21.5 (12.4–33.2)	21.9 (11.2–35.3)
New Zealand	45 to 49	23.7 (11.3–40.2)	23.1 (13.4–34.9)	22.2 (14.4–31.8)	21.6 (13.2–31.9)	21.6 (12.4–32.8)
New Zealand	50 to 54	23.4 (10.9–40.5)	23.0 (12.6–36.3)	22.9 (14.1–33.5)	23.4 (12.1–38.2)	23.5 (10.9–39.7)
New Zealand	55 to 59	21.6 (9.8–38.3)	20.7 (10.8–33.6)	20.8 (12.5–30.9)	22.0 (12.0–35.6)	22.3 (10.9–36.8)
New Zealand	60 to 64	21.9 (9.8–38.5)	21.5 (11.3–34.3)	21.6 (13.0–32.2)	22.3 (12.5–34.5)	22.4 (11.9–35.6)

Supplementary Table S11: Prevalence of male SVAC by age and location for 1990, 2000, 2010, 2020, and 2023						
**Location**	**Age Range**	**1990**	**2000**	**2010**	**2020**	**2023**
New Zealand	65 to 69	22.4 (10.2–39.7)	23.2 (12.1–37.3)	23.5 (13.6–35.9)	22.9 (12.5–36.1)	22.6 (11.6–37.3)
New Zealand	70 to 74	17.0 (6.8–32.9)	17.0 (6.5–32.8)	17.0 (6.8–32.3)	17.1 (7.1–34.1)	17.1 (7.2–33.7)
New Zealand	75 to 79	15.4 (6.1–30.3)	15.4 (5.8–30.2)	15.4 (6.0–29.7)	15.5 (6.3–31.4)	15.5 (6.4–31.1)
New Zealand	80 to 84	11.1 (4.2–22.8)	11.1 (4.0–22.7)	11.1 (4.2–22.3)	11.2 (4.4–23.7)	11.2 (4.4–23.4)
New Zealand	85 to 89	10.0 (3.8–20.8)	10.0 (3.6–20.7)	10.0 (3.7–20.3)	10.1 (3.9–21.7)	10.1 (4.0–21.4)
New Zealand	90 to 94	9.1 (3.4–19.0)	9.1 (3.2–18.9)	9.1 (3.4–18.6)	9.2 (3.5–19.8)	9.2 (3.6–19.6)
New Zealand	95 plus	9.0 (3.4–18.9)	9.0 (3.2–18.8)	9.0 (3.3–18.4)	9.1 (3.5–19.7)	9.1 (3.5–19.5)
New Zealand	Age-standardized	21.7 (13.7–32.0)	21.3 (13.8–30.7)	21.3 (15.2–28.6)	21.7 (14.6–31.6)	21.8 (13.8–32.6)
New Zealand	All age	21.7 (13.9–32.1)	21.3 (13.5–31.0)	21.2 (14.6–29.0)	21.4 (14.0–31.7)	21.4 (13.2–32.8)
High-income Asia Pacific	20 to 24	14.1 (5.5–28.1)	14.1 (5.3–28.1)	14.2 (5.5–27.7)	14.3 (5.8–29.4)	14.3 (5.9–28.9)
High-income Asia Pacific	25 to 29	14.5 (5.7–28.7)	14.5 (5.5–28.7)	14.6 (5.7–28.3)	14.7 (6.0–30.0)	14.7 (6.0–29.7)
High-income Asia Pacific	30 to 34	15.7 (6.2–30.8)	15.7 (6.0–30.8)	15.8 (6.2–30.4)	16.0 (6.5–32.2)	16.0 (6.6–31.8)
High-income Asia Pacific	35 to 39	15.9 (6.3–31.1)	16.0 (6.1–31.2)	16.1 (6.3–30.8)	16.2 (6.7–32.6)	16.2 (6.8–32.3)
High-income Asia Pacific	40 to 44	16.6 (6.6–32.2)	16.6 (6.4–32.2)	16.7 (6.6–31.8)	16.8 (7.0–33.6)	16.8 (7.1–33.3)
High-income Asia Pacific	45 to 49	16.2 (6.5–31.6)	16.3 (6.2–31.6)	16.4 (6.5–31.3)	16.5 (6.8–33.1)	16.5 (6.9–32.8)

Supplementary Table S11: Prevalence of male SVAC by age and location for 1990, 2000, 2010, 2020, and 2023						
**Location**	**Age Range**	**1990**	**2000**	**2010**	**2020**	**2023**
High-income Asia Pacific	50 to 54	15.9 (6.3–31.1)	16.0 (6.1–31.2)	16.1 (6.4–30.9)	16.3 (6.7–32.6)	16.3 (6.8–32.3)
High-income Asia Pacific	55 to 59	15.5 (6.1–30.5)	15.6 (5.9–30.5)	15.7 (6.2–30.2)	15.8 (6.5–32.0)	15.9 (6.6–31.7)
High-income Asia Pacific	60 to 64	15.1 (6.0–29.8)	15.2 (5.8–29.8)	15.3 (6.0–29.5)	15.4 (6.3–31.3)	15.4 (6.4–31.0)
High-income Asia Pacific	65 to 69	14.1 (5.5–28.1)	14.2 (5.3–28.2)	14.3 (5.6–27.9)	14.5 (5.9–29.6)	14.5 (5.9–29.2)
High-income Asia Pacific	70 to 74	11.3 (4.3–23.2)	11.4 (4.2–23.2)	11.5 (4.3–23.0)	11.6 (4.6–24.5)	11.6 (4.6–24.3)
High-income Asia Pacific	75 to 79	10.2 (3.8–21.1)	10.3 (3.7–21.2)	10.4 (3.9–21.0)	10.5 (4.1–22.4)	10.5 (4.2–22.2)
High-income Asia Pacific	80 to 84	7.2 (2.6–15.4)	7.3 (2.6–15.4)	7.4 (2.7–15.3)	7.4 (2.8–16.4)	7.5 (2.9–16.2)
High-income Asia Pacific	85 to 89	6.5 (2.4–13.9)	6.5 (2.3–14.0)	6.6 (2.4–13.9)	6.7 (2.5–14.9)	6.7 (2.6–14.7)
High-income Asia Pacific	90 to 94	5.9 (2.1–12.7)	5.9 (2.1–12.7)	6.0 (2.1–12.6)	6.1 (2.3–13.6)	6.1 (2.3–13.4)
High-income Asia Pacific	95 plus	5.8 (2.1–12.6)	5.9 (2.0–12.6)	5.9 (2.1–12.5)	6.0 (2.2–13.4)	6.0 (2.3–13.3)
High-income Asia Pacific	Age-standardized	14.8 (5.8–29.2)	14.9 (5.6–29.2)	15.0 (5.9–28.9)	15.1 (6.2–30.6)	15.1 (6.2–30.3)
High-income Asia Pacific	All age	15.0 (5.9–29.6)	14.9 (5.7–29.3)	14.8 (5.8–28.6)	14.6 (5.9–29.7)	14.5 (6.0–29.2)
Brunei	20 to 24	14.1 (5.5–28.1)	14.1 (5.3–28.1)	14.2 (5.5–27.7)	14.3 (5.8–29.4)	14.3 (5.9–28.9)
Brunei	25 to 29	14.5 (5.7–28.7)	14.5 (5.5–28.7)	14.6 (5.7–28.3)	14.7 (6.0–30.0)	14.7 (6.0–29.7)
Brunei	30 to 34	15.7 (6.2–30.8)	15.7 (6.0–30.8)	15.8 (6.2–30.4)	16.0 (6.5–32.2)	16.0 (6.6–31.8)

Supplementary Table S11: Prevalence of male SVAC by age and location for 1990, 2000, 2010, 2020, and 2023						
**Location**	**Age Range**	**1990**	**2000**	**2010**	**2020**	**2023**
Brunei	35 to 39	15.9 (6.3–31.1)	16.0 (6.1–31.2)	16.1 (6.3–30.8)	16.2 (6.7–32.6)	16.2 (6.8–32.3)
Brunei	40 to 44	16.6 (6.6–32.2)	16.6 (6.4–32.2)	16.7 (6.6–31.8)	16.8 (7.0–33.6)	16.8 (7.1–33.3)
Brunei	45 to 49	16.2 (6.5–31.6)	16.3 (6.2–31.6)	16.4 (6.5–31.3)	16.5 (6.8–33.1)	16.5 (6.9–32.8)
Brunei	50 to 54	15.9 (6.3–31.1)	16.0 (6.1–31.2)	16.1 (6.4–30.9)	16.3 (6.7–32.6)	16.3 (6.8–32.3)
Brunei	55 to 59	15.5 (6.1–30.5)	15.6 (5.9–30.5)	15.7 (6.2–30.2)	15.8 (6.5–32.0)	15.9 (6.6–31.7)
Brunei	60 to 64	15.1 (6.0–29.8)	15.2 (5.8–29.8)	15.3 (6.0–29.5)	15.4 (6.3–31.3)	15.4 (6.4–31.0)
Brunei	65 to 69	14.1 (5.5–28.1)	14.2 (5.3–28.2)	14.3 (5.6–27.9)	14.5 (5.9–29.6)	14.5 (5.9–29.2)
Brunei	70 to 74	11.3 (4.3–23.2)	11.4 (4.2–23.2)	11.5 (4.3–23.0)	11.6 (4.6–24.5)	11.6 (4.6–24.3)
Brunei	75 to 79	10.2 (3.8–21.1)	10.3 (3.7–21.2)	10.4 (3.9–21.0)	10.5 (4.1–22.4)	10.5 (4.2–22.2)
Brunei	80 to 84	7.2 (2.6–15.4)	7.3 (2.6–15.4)	7.4 (2.7–15.3)	7.4 (2.8–16.4)	7.5 (2.9–16.2)
Brunei	85 to 89	6.5 (2.4–13.9)	6.5 (2.3–14.0)	6.6 (2.4–13.9)	6.7 (2.5–14.9)	6.7 (2.6–14.7)
Brunei	90 to 94	5.9 (2.1–12.7)	5.9 (2.1–12.7)	6.0 (2.1–12.6)	6.1 (2.3–13.6)	6.1 (2.3–13.4)
Brunei	95 plus	5.8 (2.1–12.6)	5.9 (2.0–12.6)	5.9 (2.1–12.5)	6.0 (2.2–13.4)	6.0 (2.3–13.3)
Brunei	Age-standardized	14.8 (5.8–29.2)	14.9 (5.6–29.2)	15.0 (5.9–28.9)	15.1 (6.2–30.6)	15.1 (6.2–30.3)
Brunei	All age	15.1 (6.0–29.8)	15.3 (5.8–30.0)	15.4 (6.0–29.6)	15.5 (6.3–31.3)	15.5 (6.4–30.9)

Supplementary Table S11: Prevalence of male SVAC by age and location for 1990, 2000, 2010, 2020, and 2023						
**Location**	**Age Range**	**1990**	**2000**	**2010**	**2020**	**2023**
Japan	20 to 24	14.1 (5.5–28.1)	14.1 (5.3–28.1)	14.2 (5.5–27.7)	14.3 (5.8–29.4)	14.3 (5.9–28.9)
Japan	25 to 29	14.5 (5.7–28.7)	14.5 (5.5–28.7)	14.6 (5.7–28.3)	14.7 (6.0–30.0)	14.7 (6.0–29.7)
Japan	30 to 34	15.7 (6.2–30.8)	15.7 (6.0–30.8)	15.8 (6.2–30.4)	16.0 (6.5–32.2)	16.0 (6.6–31.8)
Japan	35 to 39	15.9 (6.3–31.1)	16.0 (6.1–31.2)	16.1 (6.3–30.8)	16.2 (6.7–32.6)	16.2 (6.8–32.3)
Japan	40 to 44	16.6 (6.6–32.2)	16.6 (6.4–32.2)	16.7 (6.6–31.8)	16.8 (7.0–33.6)	16.8 (7.1–33.3)
Japan	45 to 49	16.2 (6.5–31.6)	16.3 (6.2–31.6)	16.4 (6.5–31.3)	16.5 (6.8–33.1)	16.5 (6.9–32.8)
Japan	50 to 54	15.9 (6.3–31.1)	16.0 (6.1–31.2)	16.1 (6.4–30.9)	16.3 (6.7–32.6)	16.3 (6.8–32.3)
Japan	55 to 59	15.5 (6.1–30.5)	15.6 (5.9–30.5)	15.7 (6.2–30.2)	15.8 (6.5–32.0)	15.9 (6.6–31.7)
Japan	60 to 64	15.1 (6.0–29.8)	15.2 (5.8–29.8)	15.3 (6.0–29.5)	15.4 (6.3–31.3)	15.4 (6.4–31.0)
Japan	65 to 69	14.1 (5.5–28.1)	14.2 (5.3–28.2)	14.3 (5.6–27.9)	14.5 (5.9–29.6)	14.5 (5.9–29.2)
Japan	70 to 74	11.3 (4.3–23.2)	11.4 (4.2–23.2)	11.5 (4.3–23.0)	11.6 (4.6–24.5)	11.6 (4.6–24.3)
Japan	75 to 79	10.2 (3.8–21.1)	10.3 (3.7–21.2)	10.4 (3.9–21.0)	10.5 (4.1–22.4)	10.5 (4.2–22.2)
Japan	80 to 84	7.2 (2.6–15.4)	7.3 (2.6–15.4)	7.4 (2.7–15.3)	7.4 (2.8–16.4)	7.5 (2.9–16.2)
Japan	85 to 89	6.5 (2.4–13.9)	6.5 (2.3–14.0)	6.6 (2.4–13.9)	6.7 (2.5–14.9)	6.7 (2.6–14.7)
Japan	90 to 94	5.9 (2.1–12.7)	5.9 (2.1–12.7)	6.0 (2.1–12.6)	6.1 (2.3–13.6)	6.1 (2.3–13.4)

Supplementary Table S11: Prevalence of male SVAC by age and location for 1990, 2000, 2010, 2020, and 2023						
**Location**	**Age Range**	**1990**	**2000**	**2010**	**2020**	**2023**
Japan	95 plus	5.8 (2.1–12.6)	5.9 (2.0–12.6)	5.9 (2.1–12.5)	6.0 (2.2–13.4)	6.0 (2.3–13.3)
Japan	Age-standardized	14.8 (5.8–29.2)	14.9 (5.6–29.2)	15.0 (5.9–28.9)	15.1 (6.2–30.6)	15.1 (6.2–30.3)
Japan	All age	15.0 (5.9–29.5)	14.8 (5.6–29.1)	14.6 (5.7–28.2)	14.3 (5.8–29.2)	14.2 (5.8–28.7)
South Korea	20 to 24	14.1 (5.5–28.1)	14.1 (5.3–28.1)	14.2 (5.5–27.7)	14.3 (5.8–29.4)	14.3 (5.9–28.9)
South Korea	25 to 29	14.5 (5.7–28.7)	14.5 (5.5–28.7)	14.6 (5.7–28.3)	14.7 (6.0–30.0)	14.7 (6.0–29.7)
South Korea	30 to 34	15.7 (6.2–30.8)	15.7 (6.0–30.8)	15.8 (6.2–30.4)	16.0 (6.5–32.2)	16.0 (6.6–31.8)
South Korea	35 to 39	15.9 (6.3–31.1)	16.0 (6.1–31.2)	16.1 (6.3–30.8)	16.2 (6.7–32.6)	16.2 (6.8–32.3)
South Korea	40 to 44	16.6 (6.6–32.2)	16.6 (6.4–32.2)	16.7 (6.6–31.8)	16.8 (7.0–33.6)	16.8 (7.1–33.3)
South Korea	45 to 49	16.2 (6.5–31.6)	16.3 (6.2–31.6)	16.4 (6.5–31.3)	16.5 (6.8–33.1)	16.5 (6.9–32.8)
South Korea	50 to 54	15.9 (6.3–31.1)	16.0 (6.1–31.2)	16.1 (6.4–30.9)	16.3 (6.7–32.6)	16.3 (6.8–32.3)
South Korea	55 to 59	15.5 (6.1–30.5)	15.6 (5.9–30.5)	15.7 (6.2–30.2)	15.8 (6.5–32.0)	15.9 (6.6–31.7)
South Korea	60 to 64	15.1 (6.0–29.8)	15.2 (5.8–29.8)	15.3 (6.0–29.5)	15.4 (6.3–31.3)	15.4 (6.4–31.0)
South Korea	65 to 69	14.1 (5.5–28.1)	14.2 (5.3–28.2)	14.3 (5.6–27.9)	14.5 (5.9–29.6)	14.5 (5.9–29.2)
South Korea	70 to 74	11.3 (4.3–23.2)	11.4 (4.2–23.2)	11.5 (4.3–23.0)	11.6 (4.6–24.5)	11.6 (4.6–24.3)
South Korea	75 to 79	10.2 (3.8–21.1)	10.3 (3.7–21.2)	10.4 (3.9–21.0)	10.5 (4.1–22.4)	10.5 (4.2–22.2)

Supplementary Table S11: Prevalence of male SVAC by age and location for 1990, 2000, 2010, 2020, and 2023						
**Location**	**Age Range**	**1990**	**2000**	**2010**	**2020**	**2023**
South Korea	80 to 84	7.2 (2.6–15.4)	7.3 (2.6–15.4)	7.4 (2.7–15.3)	7.4 (2.8–16.4)	7.5 (2.9–16.2)
South Korea	85 to 89	6.5 (2.4–13.9)	6.5 (2.3–14.0)	6.6 (2.4–13.9)	6.7 (2.5–14.9)	6.7 (2.6–14.7)
South Korea	90 to 94	5.9 (2.1–12.7)	5.9 (2.1–12.7)	6.0 (2.1–12.6)	6.1 (2.3–13.6)	6.1 (2.3–13.4)
South Korea	95 plus	5.8 (2.1–12.6)	5.9 (2.0–12.6)	5.9 (2.1–12.5)	6.0 (2.2–13.4)	6.0 (2.3–13.3)
South Korea	Age-standardized	14.8 (5.8–29.2)	14.9 (5.6–29.2)	15.0 (5.9–28.9)	15.1 (6.2–30.6)	15.1 (6.2–30.3)
South Korea	All age	15.2 (6.0–29.8)	15.3 (5.8–29.9)	15.2 (6.0–29.4)	15.2 (6.2–30.7)	15.1 (6.2–30.2)
Singapore	20 to 24	14.1 (5.5–28.1)	14.1 (5.3–28.1)	14.2 (5.5–27.7)	14.3 (5.8–29.4)	14.3 (5.9–28.9)
Singapore	25 to 29	14.5 (5.7–28.7)	14.5 (5.5–28.7)	14.6 (5.7–28.3)	14.7 (6.0–30.0)	14.7 (6.0–29.7)
Singapore	30 to 34	15.7 (6.2–30.8)	15.7 (6.0–30.8)	15.8 (6.2–30.4)	16.0 (6.5–32.2)	16.0 (6.6–31.8)
Singapore	35 to 39	15.9 (6.3–31.1)	16.0 (6.1–31.2)	16.1 (6.3–30.8)	16.2 (6.7–32.6)	16.2 (6.8–32.3)
Singapore	40 to 44	16.6 (6.6–32.2)	16.6 (6.4–32.2)	16.7 (6.6–31.8)	16.8 (7.0–33.6)	16.8 (7.1–33.3)
Singapore	45 to 49	16.2 (6.5–31.6)	16.3 (6.2–31.6)	16.4 (6.5–31.3)	16.5 (6.8–33.1)	16.5 (6.9–32.8)
Singapore	50 to 54	15.9 (6.3–31.1)	16.0 (6.1–31.2)	16.1 (6.4–30.9)	16.3 (6.7–32.6)	16.3 (6.8–32.3)
Singapore	55 to 59	15.5 (6.1–30.5)	15.6 (5.9–30.5)	15.7 (6.2–30.2)	15.8 (6.5–32.0)	15.9 (6.6–31.7)
Singapore	60 to 64	15.1 (6.0–29.8)	15.2 (5.8–29.8)	15.3 (6.0–29.5)	15.4 (6.3–31.3)	15.4 (6.4–31.0)

Supplementary Table S11: Prevalence of male SVAC by age and location for 1990, 2000, 2010, 2020, and 2023						
**Location**	**Age Range**	**1990**	**2000**	**2010**	**2020**	**2023**
Singapore	65 to 69	14.1 (5.5–28.1)	14.2 (5.3–28.2)	14.3 (5.6–27.9)	14.5 (5.9–29.6)	14.5 (5.9–29.2)
Singapore	70 to 74	11.3 (4.3–23.2)	11.4 (4.2–23.2)	11.5 (4.3–23.0)	11.6 (4.6–24.5)	11.6 (4.6–24.3)
Singapore	75 to 79	10.2 (3.8–21.1)	10.3 (3.7–21.2)	10.4 (3.9–21.0)	10.5 (4.1–22.4)	10.5 (4.2–22.2)
Singapore	80 to 84	7.2 (2.6–15.4)	7.3 (2.6–15.4)	7.4 (2.7–15.3)	7.4 (2.8–16.4)	7.5 (2.9–16.2)
Singapore	85 to 89	6.5 (2.4–13.9)	6.5 (2.3–14.0)	6.6 (2.4–13.9)	6.7 (2.5–14.9)	6.7 (2.6–14.7)
Singapore	90 to 94	5.9 (2.1–12.7)	5.9 (2.1–12.7)	6.0 (2.1–12.6)	6.1 (2.3–13.6)	6.1 (2.3–13.4)
Singapore	95 plus	5.8 (2.1–12.6)	5.9 (2.0–12.6)	5.9 (2.1–12.5)	6.0 (2.2–13.4)	6.0 (2.3–13.3)
Singapore	Age-standardized	14.8 (5.8–29.2)	14.9 (5.6–29.2)	15.0 (5.9–28.9)	15.1 (6.2–30.6)	15.1 (6.2–30.3)
Singapore	All age	15.1 (6.0–29.7)	15.2 (5.8–29.9)	15.3 (6.0–29.5)	15.4 (6.3–31.1)	15.3 (6.3–30.6)
High-income North America	20 to 24	15.9 (13.7–18.2)	16.8 (14.6–19.0)	15.2 (12.4–18.4)	13.9 (8.7–21.0)	13.9 (7.9–22.3)
High-income North America	25 to 29	15.6 (13.1–18.4)	16.6 (14.4–19.0)	16.9 (14.6–19.2)	14.7 (10.7–19.8)	14.3 (9.7–20.9)
High-income North America	30 to 34	15.4 (13.1–18.1)	16.2 (14.2–18.5)	17.2 (15.1–19.5)	15.8 (13.3–18.8)	15.3 (12.2–19.3)
High-income North America	35 to 39	15.0 (12.7–17.7)	15.8 (13.8–18.0)	16.9 (14.8–19.1)	17.4 (14.9–20.2)	17.4 (14.1–21.5)
High-income North America	40 to 44	15.2 (13.2–17.2)	16.0 (14.2–18.1)	16.9 (14.6–19.3)	17.3 (14.3–20.8)	17.3 (13.4–22.2)
High-income North America	45 to 49	14.5 (12.4–16.7)	15.6 (13.7–17.7)	16.3 (14.2–18.7)	17.4 (14.5–20.6)	17.8 (14.1–22.3)

Supplementary Table S11: Prevalence of male SVAC by age and location for 1990, 2000, 2010, 2020, and 2023						
**Location**	**Age Range**	**1990**	**2000**	**2010**	**2020**	**2023**
High-income North America	50 to 54	13.6 (11.5–16.1)	15.0 (13.2–17.1)	16.2 (14.1–18.5)	16.3 (13.4–20.0)	16.4 (12.3–21.9)
High-income North America	55 to 59	13.9 (11.4–16.7)	14.8 (13.1–16.8)	16.1 (14.0–18.4)	16.5 (13.5–20.2)	16.5 (12.3–22.0)
High-income North America	60 to 64	12.8 (9.6–16.3)	14.0 (11.9–16.2)	15.4 (13.3–17.8)	16.6 (13.8–19.7)	16.9 (13.1–21.6)
High-income North America	65 to 69	12.7 (8.9–17.1)	13.4 (10.9–16.3)	15.2 (12.9–17.7)	15.7 (12.8–18.9)	15.7 (12.0–19.9)
High-income North America	70 to 74	9.3 (5.9–13.6)	11.1 (8.1–14.7)	13.8 (11.5–16.2)	14.8 (12.0–18.0)	14.8 (11.2–18.8)
High-income North America	75 to 79	7.7 (4.3–12.7)	9.2 (6.0–13.3)	12.3 (9.8–15.0)	14.3 (11.5–17.4)	14.3 (10.7–18.9)
High-income North America	80 to 84	7.2 (3.5–12.9)	8.9 (5.4–13.4)	12.0 (9.4–14.7)	12.7 (10.1–15.7)	12.4 (9.2–16.5)
High-income North America	85 to 89	7.0 (3.0–13.1)	8.6 (5.0–13.5)	11.5 (9.0–14.5)	12.1 (9.4–15.4)	11.7 (8.6–15.7)
High-income North America	90 to 94	7.2 (2.9–14.1)	9.1 (4.9–14.7)	11.1 (8.6–14.0)	9.7 (6.8–14.0)	9.1 (6.0–14.1)
High-income North America	95 plus	7.7 (3.1–15.0)	9.9 (5.2–16.2)	11.2 (8.8–14.2)	9.4 (6.2–14.1)	8.7 (5.4–14.3)
High-income North America	Age-standardized	14.1 (12.4–16.2)	15.2 (13.7–16.7)	16.0 (14.2–18.1)	15.8 (14.2–17.8)	15.8 (13.5–18.6)
High-income North America	All age	14.2 (12.5–16.2)	15.1 (13.6–16.6)	15.9 (14.1–18.0)	15.8 (14.4–17.5)	15.8 (13.7–18.4)
Canada	20 to 24	11.1 (7.7–15.5)	9.1 (6.2–12.7)	8.0 (5.1–11.7)	8.6 (4.3–15.0)	8.8 (4.3–16.7)
Canada	25 to 29	11.9 (8.4–16.5)	10.0 (7.0–13.7)	8.4 (5.6–11.9)	8.8 (4.6–15.1)	9.0 (4.4–16.8)
Canada	30 to 34	13.1 (9.7–17.4)	13.4 (10.1–17.8)	13.5 (10.7–16.8)	13.2 (8.2–20.2)	13.3 (7.5–21.8)

Supplementary Table S11: Prevalence of male SVAC by age and location for 1990, 2000, 2010, 2020, and 2023						
**Location**	**Age Range**	**1990**	**2000**	**2010**	**2020**	**2023**
Canada	35 to 39	13.0 (9.4–17.3)	13.2 (9.8–17.4)	12.4 (9.6–15.4)	11.4 (7.3–16.8)	11.3 (6.5–18.2)
Canada	40 to 44	15.2 (11.5–19.8)	16.4 (12.6–21.0)	17.5 (14.2–21.1)	15.0 (10.0–21.3)	14.5 (8.6–22.5)
Canada	45 to 49	14.5 (10.8–19.0)	15.3 (11.7–19.6)	16.0 (12.9–19.3)	14.6 (9.9–20.8)	14.3 (8.7–22.0)
Canada	50 to 54	13.5 (9.8–18.2)	14.5 (10.9–18.6)	14.7 (11.8–17.9)	13.8 (9.3–19.7)	13.6 (8.3–21.2)
Canada	55 to 59	15.8 (11.6–21.1)	17.2 (13.2–21.9)	18.4 (15.2–22.0)	16.6 (11.7–22.9)	16.2 (10.1–24.5)
Canada	60 to 64	14.8 (10.4–20.3)	16.1 (12.2–20.7)	17.0 (14.0–20.4)	15.2 (10.5–21.3)	14.8 (9.2–22.7)
Canada	65 to 69	14.2 (7.0–24.8)	16.5 (11.4–22.6)	18.5 (15.4–22.1)	15.9 (10.9–22.0)	15.3 (9.5–22.9)
Canada	70 to 74	11.4 (5.1–21.2)	12.2 (7.5–18.1)	13.0 (10.0–16.4)	13.0 (8.5–18.6)	13.0 (7.7–20.2)
Canada	75 to 79	8.8 (3.6–16.9)	8.6 (4.8–13.8)	9.4 (6.4–13.3)	10.9 (6.7–16.5)	11.2 (6.3–18.2)
Canada	80 to 84	6.8 (2.7–13.7)	7.2 (3.5–12.9)	8.4 (4.9–13.1)	9.7 (5.4–15.6)	9.9 (5.1–16.8)
Canada	85 to 89	6.1 (2.4–12.5)	6.3 (2.9–11.4)	6.7 (3.5–11.4)	7.3 (3.7–12.8)	7.4 (3.4–13.5)
Canada	90 to 94	5.6 (2.2–11.6)	5.6 (2.5–10.4)	5.3 (2.6– 9.5)	5.2 (2.2–10.4)	5.2 (2.1–10.8)
Canada	95 plus	5.5 (2.2–11.5)	5.5 (2.5–10.3)	5.3 (2.6– 9.4)	5.2 (2.2–10.3)	5.2 (2.1–10.7)
Canada	Age-standardized	13.0 (9.7–17.0)	13.2 (10.0–17.0)	13.3 (10.7–16.2)	12.5 (9.2–16.7)	12.4 (8.3–17.6)
Canada	All age	13.1 (9.8–17.1)	13.5 (10.3–17.5)	13.9 (11.2–16.8)	12.9 (9.8–16.8)	12.8 (9.1–17.6)

Supplementary Table S11: Prevalence of male SVAC by age and location for 1990, 2000, 2010, 2020, and 2023						
**Location**	**Age Range**	**1990**	**2000**	**2010**	**2020**	**2023**
Greenland	20 to 24	15.6 (5.1–33.1)	15.4 (5.6–30.6)	15.4 (6.0–29.7)	15.7 (5.5–33.3)	15.8 (5.4–34.1)
Greenland	25 to 29	15.9 (5.2–33.6)	15.7 (5.7–31.0)	15.7 (6.2–30.1)	16.0 (5.6–33.8)	16.1 (5.5–34.6)
Greenland	30 to 34	17.1 (5.7–35.6)	16.8 (6.2–32.9)	16.8 (6.7–32.1)	17.2 (6.1–35.9)	17.3 (6.0–36.6)
Greenland	35 to 39	17.2 (5.7–35.8)	17.0 (6.3–33.2)	17.0 (6.8–32.3)	17.3 (6.2–36.1)	17.4 (6.0–36.9)
Greenland	40 to 44	17.7 (5.9–36.7)	17.5 (6.5–34.0)	17.5 (7.0–33.1)	17.9 (6.4–37.0)	18.0 (6.2–37.8)
Greenland	45 to 49	17.3 (5.7–36.0)	17.1 (6.3–33.3)	17.1 (6.8–32.5)	17.5 (6.3–36.3)	17.5 (6.1–37.1)
Greenland	50 to 54	16.9 (5.6–35.4)	16.7 (6.2–32.8)	16.8 (6.7–31.9)	17.1 (6.1–35.8)	17.2 (5.9–36.5)
Greenland	55 to 59	16.5 (5.4–34.5)	16.2 (6.0–32.0)	16.3 (6.4–31.2)	16.7 (5.9–35.0)	16.7 (5.7–35.7)
Greenland	60 to 64	16.0 (5.2–33.7)	15.8 (5.8–31.2)	15.8 (6.2–30.4)	16.2 (5.7–34.2)	16.3 (5.6–35.0)
Greenland	65 to 69	15.0 (4.8–31.9)	14.8 (5.4–29.5)	14.8 (5.8–28.8)	15.2 (5.3–32.4)	15.3 (5.2–33.2)
Greenland	70 to 74	12.1 (3.8–26.6)	11.9 (4.2–24.4)	12.0 (4.5–23.8)	12.3 (4.2–27.1)	12.4 (4.0–27.8)
Greenland	75 to 79	10.9 (3.4–24.4)	10.8 (3.7–22.3)	10.8 (4.1–21.8)	11.1 (3.7–24.9)	11.2 (3.6–25.5)
Greenland	80 to 84	7.8 (2.3–18.0)	7.7 (2.6–16.4)	7.7 (2.8–15.9)	7.9 (2.6–18.4)	8.0 (2.5–18.9)
Greenland	85 to 89	7.0 (2.1–16.3)	6.9 (2.3–14.9)	6.9 (2.5–14.5)	7.2 (2.3–16.8)	7.2 (2.2–17.3)
Greenland	90 to 94	6.4 (1.9–14.9)	6.2 (2.1–13.5)	6.3 (2.3–13.2)	6.5 (2.1–15.3)	6.5 (2.0–15.7)

Supplementary Table S11: Prevalence of male SVAC by age and location for 1990, 2000, 2010, 2020, and 2023						
**Location**	**Age Range**	**1990**	**2000**	**2010**	**2020**	**2023**
Greenland	95 plus	6.3 (1.8–14.8)	6.2 (2.1–13.4)	6.2 (2.2–13.1)	6.4 (2.1–15.2)	6.5 (2.0–15.6)
Greenland	Age-standardized	16.0 (5.2–33.6)	15.8 (5.8–31.1)	15.8 (6.2–30.3)	16.1 (5.7–34.0)	16.2 (5.5–34.7)
Greenland	All age	16.5 (5.4–34.6)	16.4 (6.0–32.2)	16.2 (6.4–31.0)	16.3 (5.8–34.3)	16.4 (5.6–35.0)
USA	20 to 24	16.4 (14.1–18.9)	17.5 (15.2–20.0)	15.9 (12.7–19.6)	14.5 (8.2–22.8)	14.4 (7.2–24.2)
USA	25 to 29	16.0 (13.6–18.6)	17.2 (14.8–19.9)	17.7 (15.3–20.3)	15.3 (10.3–21.3)	14.9 (9.0–22.6)
USA	30 to 34	15.7 (13.4–18.2)	16.5 (14.1–19.2)	17.6 (15.4–20.0)	16.1 (12.6–19.9)	15.5 (11.2–20.5)
USA	35 to 39	15.3 (13.0–17.8)	16.1 (13.9–18.8)	17.4 (15.1–19.8)	18.0 (14.8–21.5)	18.1 (13.9–22.9)
USA	40 to 44	15.2 (12.9–17.5)	16.0 (13.8–18.6)	16.8 (14.5–19.4)	17.6 (14.6–21.0)	17.7 (13.7–22.4)
USA	45 to 49	14.5 (12.3–16.9)	15.6 (13.3–18.2)	16.4 (14.1–18.9)	17.7 (14.6–21.2)	18.2 (13.9–23.2)
USA	50 to 54	13.6 (11.6–16.0)	15.1 (12.8–17.6)	16.3 (14.2–18.7)	16.6 (13.8–20.1)	16.7 (12.7–21.9)
USA	55 to 59	13.6 (11.2–16.4)	14.5 (12.3–17.0)	15.8 (13.7–18.2)	16.5 (13.7–19.9)	16.5 (12.5–21.6)
USA	60 to 64	12.6 (9.5–16.0)	13.7 (11.6–16.0)	15.2 (13.0–17.6)	16.7 (13.8–20.1)	17.1 (13.2–22.1)
USA	65 to 69	12.5 (9.0–16.4)	13.1 (10.6–15.7)	14.8 (12.6–17.2)	15.7 (12.8–18.9)	15.8 (12.1–20.1)
USA	70 to 74	9.1 (5.9–12.9)	11.0 (8.2–14.4)	13.8 (11.7–16.3)	15.1 (12.2–18.2)	15.0 (11.4–19.2)
USA	75 to 79	7.6 (4.4–12.2)	9.3 (6.1–13.4)	12.7 (10.1–15.4)	14.7 (11.9–18.0)	14.8 (11.0–19.3)

Supplementary Table S11: Prevalence of male SVAC by age and location for 1990, 2000, 2010, 2020, and 2023						
**Location**	**Age Range**	**1990**	**2000**	**2010**	**2020**	**2023**
USA	80 to 84	7.2 (3.5–13.0)	9.0 (5.6–13.6)	12.4 (9.9–15.2)	13.2 (10.5–16.2)	12.7 (9.4–17.0)
USA	85 to 89	7.1 (3.1–13.4)	8.9 (5.2–13.9)	12.1 (9.3–15.3)	12.8 (10.0–16.2)	12.4 (9.0–16.7)
USA	90 to 94	7.4 (3.0–14.4)	9.5 (5.1–15.2)	11.7 (9.0–14.9)	10.3 (6.3–15.5)	9.7 (5.2–16.0)
USA	95 plus	7.9 (3.2–15.4)	10.2 (5.4–16.7)	11.9 (9.2–15.1)	9.9 (5.9–15.3)	9.2 (4.8–15.8)
USA	Age-standardized	14.3 (12.6–16.1)	15.4 (13.7–17.2)	16.3 (14.4–18.4)	16.2 (14.4–18.5)	16.1 (13.7–19.3)
USA	All age	14.3 (12.7–16.1)	15.2 (13.5–17.1)	16.1 (14.2–18.2)	16.2 (14.6–18.1)	16.1 (13.9–18.9)
Southern Latin America	20 to 24	13.8 (6.0–25.9)	13.4 (6.3–23.7)	13.3 (6.3–23.4)	14.0 (6.1–27.1)	14.2 (6.1–27.9)
Southern Latin America	25 to 29	15.5 (6.9–28.3)	15.5 (7.7–26.5)	15.5 (7.7–26.4)	15.4 (6.9–29.2)	15.4 (6.8–29.8)
Southern Latin America	30 to 34	15.7 (7.1–28.3)	15.2 (7.4–26.3)	15.4 (8.0–25.8)	16.1 (7.5–29.2)	16.2 (7.5–30.3)
Southern Latin America	35 to 39	15.9 (7.2–28.8)	15.2 (7.5–26.2)	15.4 (8.0–25.5)	16.2 (7.4–29.6)	16.3 (7.5–30.8)
Southern Latin America	40 to 44	18.2 (8.5–32.2)	18.4 (9.3–31.0)	18.7 (10.6–29.6)	18.7 (9.1–33.1)	18.6 (8.9–34.1)
Southern Latin America	45 to 49	17.8 (7.7–33.0)	17.9 (7.9–32.9)	18.0 (9.1–30.5)	18.1 (8.4–33.0)	18.1 (8.4–33.6)
Southern Latin America	50 to 54	17.8 (8.1–31.8)	17.8 (8.8–30.3)	17.6 (9.4–28.9)	17.8 (8.4–31.7)	17.8 (8.3–32.8)
Southern Latin America	55 to 59	17.6 (7.6–32.5)	18.0 (8.1–33.0)	18.3 (9.4–30.9)	18.2 (8.9–32.4)	18.0 (8.7–33.1)
Southern Latin America	60 to 64	17.3 (7.6–31.7)	17.4 (8.1–31.0)	17.4 (7.9–31.0)	17.2 (7.4–33.2)	17.1 (7.4–33.2)

Supplementary Table S11: Prevalence of male SVAC by age and location for 1990, 2000, 2010, 2020, and 2023						
**Location**	**Age Range**	**1990**	**2000**	**2010**	**2020**	**2023**
Southern Latin America	65 to 69	15.5 (6.2–30.4)	15.5 (5.9–30.3)	15.5 (6.1–29.8)	15.6 (6.4–31.5)	15.5 (6.4–31.1)
Southern Latin America	70 to 74	12.5 (4.8–25.2)	12.4 (4.6–25.1)	12.4 (4.7–24.6)	12.5 (5.0–26.2)	12.5 (5.0–25.8)
Southern Latin America	75 to 79	11.2 (4.3–22.9)	11.3 (4.1–23.0)	11.2 (4.2–22.5)	11.3 (4.4–23.9)	11.3 (4.5–23.6)
Southern Latin America	80 to 84	8.0 (2.9–16.8)	8.0 (2.8–16.8)	8.0 (2.9–16.5)	8.0 (3.1–17.6)	8.0 (3.1–17.3)
Southern Latin America	85 to 89	7.2 (2.6–15.2)	7.2 (2.5–15.2)	7.2 (2.6–15.0)	7.2 (2.7–15.9)	7.2 (2.8–15.7)
Southern Latin America	90 to 94	6.5 (2.3–13.8)	6.4 (2.2–13.7)	6.4 (2.3–13.4)	6.5 (2.4–14.4)	6.5 (2.5–14.3)
Southern Latin America	95 plus	6.4 (2.3–13.6)	6.3 (2.2–13.4)	6.2 (2.2–13.1)	6.4 (2.4–14.2)	6.4 (2.4–14.1)
Southern Latin America	Age-standardized	15.8 (6.9–29.2)	15.7 (7.4–27.9)	15.7 (7.8–26.8)	16.0 (7.3–29.8)	16.0 (7.2–30.4)
Southern Latin America	All age	15.9 (7.0–29.3)	15.7 (7.4–27.9)	15.8 (7.9–26.8)	16.0 (7.3–29.9)	16.0 (7.2–30.4)
Argentina	20 to 24	15.2 (6.7–27.8)	14.7 (7.1–25.6)	14.9 (7.0–26.0)	15.5 (6.7–29.9)	15.6 (6.7–30.5)
Argentina	25 to 29	17.6 (8.1–31.4)	17.9 (9.1–29.9)	17.9 (8.9–30.2)	17.5 (7.8–32.8)	17.3 (7.6–33.3)
Argentina	30 to 34	16.1 (7.2–29.4)	15.3 (7.3–27.0)	15.6 (7.3–27.3)	16.6 (7.2–31.7)	16.8 (7.3–32.4)
Argentina	35 to 39	16.5 (7.4–30.0)	15.8 (7.6–27.5)	16.1 (7.5–27.9)	16.9 (7.4–32.3)	17.1 (7.5–33.0)
Argentina	40 to 44	18.8 (8.5–33.7)	18.7 (9.1–32.2)	18.8 (8.9–32.4)	19.0 (8.4–35.6)	19.0 (8.4–36.0)
Argentina	45 to 49	18.5 (7.5–35.2)	18.5 (7.2–35.2)	18.5 (7.5–34.7)	18.6 (7.8–36.5)	18.6 (7.9–36.1)

Supplementary Table S11: Prevalence of male SVAC by age and location for 1990, 2000, 2010, 2020, and 2023						
**Location**	**Age Range**	**1990**	**2000**	**2010**	**2020**	**2023**
Argentina	50 to 54	18.9 (8.6–33.8)	19.0 (9.3–32.5)	19.0 (9.1–32.4)	19.0 (8.4–35.5)	18.9 (8.4–35.8)
Argentina	55 to 59	17.9 (7.3–34.4)	17.9 (7.0–34.3)	18.0 (7.2–33.8)	18.1 (7.6–35.6)	18.1 (7.7–35.3)
Argentina	60 to 64	18.6 (8.4–33.4)	18.9 (9.3–32.5)	18.9 (9.0–32.4)	18.6 (8.2–35.0)	18.4 (8.1–35.1)
Argentina	65 to 69	16.4 (6.5–31.9)	16.4 (6.3–31.9)	16.4 (6.5–31.4)	16.5 (6.8–33.1)	16.5 (6.9–32.8)
Argentina	70 to 74	13.2 (5.1–26.5)	13.2 (4.9–26.5)	13.2 (5.1–26.1)	13.3 (5.3–27.6)	13.3 (5.4–27.3)
Argentina	75 to 79	11.9 (4.6–24.2)	11.9 (4.4–24.2)	12.0 (4.5–23.8)	12.0 (4.8–25.3)	12.0 (4.8–24.9)
Argentina	80 to 84	8.5 (3.1–17.9)	8.5 (3.0–17.8)	8.5 (3.1–17.5)	8.6 (3.3–18.7)	8.6 (3.3–18.5)
Argentina	85 to 89	7.7 (2.8–16.2)	7.7 (2.7–16.2)	7.7 (2.8–15.9)	7.7 (2.9–17.0)	7.7 (3.0–16.8)
Argentina	90 to 94	6.9 (2.5–14.8)	6.9 (2.4–14.8)	6.9 (2.5–14.5)	7.0 (2.6–15.5)	7.0 (2.7–15.3)
Argentina	95 plus	6.9 (2.5–14.7)	6.9 (2.4–14.6)	6.9 (2.5–14.4)	6.9 (2.6–15.4)	6.9 (2.7–15.2)
Argentina	Age-standardized	16.7 (7.3–30.8)	16.5 (7.7–29.5)	16.6 (7.5–29.6)	16.9 (7.3–32.8)	16.9 (7.3–32.8)
Argentina	All age	16.8 (7.4–30.9)	16.6 (7.7–29.6)	16.7 (7.6–29.6)	16.9 (7.3–32.9)	16.9 (7.3–32.9)
Chile	20 to 24	11.7 (4.7–23.3)	10.6 (4.6–20.4)	10.1 (4.9–18.0)	11.0 (4.9–21.1)	11.3 (4.8–22.1)
Chile	25 to 29	12.0 (4.8–23.9)	10.9 (4.8–20.7)	10.4 (5.0–18.4)	11.2 (5.1–21.5)	11.6 (5.0–22.6)
Chile	30 to 34	15.2 (6.5–28.4)	15.1 (7.8–25.3)	15.1 (9.4–22.2)	15.3 (7.6–26.0)	15.3 (6.9–27.8)

Supplementary Table S11: Prevalence of male SVAC by age and location for 1990, 2000, 2010, 2020, and 2023						
**Location**	**Age Range**	**1990**	**2000**	**2010**	**2020**	**2023**
Chile	35 to 39	14.9 (6.2–28.0)	14.3 (7.1–24.3)	14.0 (8.2–21.2)	14.6 (7.1–25.3)	14.7 (6.4–26.8)
Chile	40 to 44	18.0 (7.9–32.7)	19.1 (10.3–30.4)	19.6 (13.2–27.1)	19.1 (9.9–31.5)	18.7 (8.7–32.7)
Chile	45 to 49	17.0 (7.3–31.0)	17.3 (9.2–28.2)	17.5 (11.4–24.7)	17.4 (8.9–29.1)	17.3 (7.9–30.8)
Chile	50 to 54	16.0 (6.9–29.6)	15.8 (8.1–26.2)	15.7 (9.9–22.8)	16.0 (8.0–26.9)	16.1 (7.3–28.9)
Chile	55 to 59	17.8 (7.8–32.3)	19.1 (10.3–30.4)	19.9 (13.6–27.4)	19.1 (9.9–31.3)	18.6 (8.7–32.5)
Chile	60 to 64	15.1 (5.9–29.7)	15.0 (5.7–29.6)	15.1 (5.9–29.2)	15.2 (6.2–30.8)	15.2 (6.3–30.6)
Chile	65 to 69	14.0 (5.5–27.9)	14.0 (5.3–27.9)	14.1 (5.4–27.4)	14.1 (5.7–29.1)	14.1 (5.8–28.7)
Chile	70 to 74	11.2 (4.3–23.0)	11.2 (4.1–22.9)	11.2 (4.2–22.6)	11.3 (4.5–24.0)	11.3 (4.5–23.7)
Chile	75 to 79	10.1 (3.8–20.9)	10.1 (3.7–20.9)	10.1 (3.8–20.5)	10.2 (4.0–21.9)	10.2 (4.0–21.6)
Chile	80 to 84	7.2 (2.6–15.2)	7.2 (2.5–15.2)	7.2 (2.6–14.9)	7.2 (2.7–16.0)	7.2 (2.8–15.8)
Chile	85 to 89	6.4 (2.3–13.8)	6.4 (2.2–13.8)	6.4 (2.3–13.5)	6.5 (2.4–14.5)	6.5 (2.5–14.3)
Chile	90 to 94	5.8 (2.1–12.5)	5.8 (2.0–12.5)	5.8 (2.1–12.3)	5.9 (2.2–13.2)	5.9 (2.2–13.0)
Chile	95 plus	5.8 (2.1–12.4)	5.8 (2.0–12.4)	5.8 (2.1–12.2)	5.8 (2.2–13.1)	5.8 (2.2–12.9)
Chile	Age-standardized	14.4 (6.1–27.3)	14.3 (6.9–25.0)	14.2 (8.3–22.1)	14.5 (7.3–25.0)	14.5 (6.5–25.6)
Chile	All age	14.3 (6.1–27.3)	14.4 (7.0–25.1)	14.4 (8.5–22.2)	14.5 (7.3–25.1)	14.6 (6.6–25.8)

Supplementary Table S11: Prevalence of male SVAC by age and location for 1990, 2000, 2010, 2020, and 2023						
**Location**	**Age Range**	**1990**	**2000**	**2010**	**2020**	**2023**
Uruguay	20 to 24	10.7 (4.5–21.0)	10.2 (4.6–19.0)	10.3 (4.6–18.9)	10.9 (4.5–22.2)	11.1 (4.6–22.7)
Uruguay	25 to 29	11.7 (4.9–22.7)	11.5 (5.3–21.0)	11.6 (5.2–20.8)	11.9 (5.0–23.8)	11.9 (5.0–24.2)
Uruguay	30 to 34	13.2 (5.1–26.4)	13.2 (4.9–26.4)	13.2 (5.1–26.0)	13.3 (5.3–27.5)	13.3 (5.4–27.2)
Uruguay	35 to 39	14.1 (6.1–26.6)	14.3 (6.8–25.1)	14.4 (6.8–25.1)	14.2 (6.1–27.5)	14.1 (6.0–27.9)
Uruguay	40 to 44	12.6 (5.3–24.3)	11.9 (5.4–22.0)	12.1 (5.4–21.9)	12.9 (5.4–25.7)	13.1 (5.5–26.3)
Uruguay	45 to 49	14.6 (6.3–27.4)	14.8 (7.1–25.9)	14.8 (7.0–25.8)	14.6 (6.3–28.2)	14.5 (6.2–28.6)
Uruguay	50 to 54	13.5 (5.3–27.1)	13.5 (5.0–27.0)	13.6 (5.2–26.6)	13.7 (5.5–28.2)	13.7 (5.6–27.9)
Uruguay	55 to 59	14.2 (6.1–26.9)	14.6 (6.9–25.7)	14.6 (6.9–25.5)	14.2 (6.1–27.6)	14.1 (6.0–27.9)
Uruguay	60 to 64	12.0 (5.0–23.3)	11.6 (5.1–21.6)	11.7 (5.1–21.5)	12.2 (5.1–24.5)	12.3 (5.1–24.9)
Uruguay	65 to 69	11.8 (4.5–24.0)	11.8 (4.3–23.9)	11.8 (4.5–23.5)	11.9 (4.7–25.0)	11.9 (4.7–24.7)
Uruguay	70 to 74	9.4 (3.5–19.5)	9.4 (3.4–19.5)	9.4 (3.5–19.1)	9.5 (3.7–20.4)	9.5 (3.7–20.2)
Uruguay	75 to 79	8.4 (3.1–17.7)	8.4 (3.0–17.7)	8.4 (3.1–17.4)	8.5 (3.3–18.5)	8.5 (3.3–18.2)
Uruguay	80 to 84	5.9 (2.1–12.8)	5.9 (2.1–12.7)	5.9 (2.1–12.5)	6.0 (2.2–13.4)	6.0 (2.3–13.2)
Uruguay	85 to 89	5.3 (1.9–11.5)	5.3 (1.8–11.5)	5.3 (1.9–11.3)	5.4 (2.0–12.1)	5.4 (2.0–12.0)
Uruguay	90 to 94	4.8 (1.7–10.5)	4.8 (1.6–10.4)	4.8 (1.7–10.2)	4.8 (1.8–11.0)	4.8 (1.8–10.8)

Supplementary Table S11: Prevalence of male SVAC by age and location for 1990, 2000, 2010, 2020, and 2023						
**Location**	**Age Range**	**1990**	**2000**	**2010**	**2020**	**2023**
Uruguay	95 plus	4.8 (1.7–10.4)	4.7 (1.6–10.3)	4.8 (1.7–10.1)	4.8 (1.8–10.9)	4.8 (1.8–10.7)
Uruguay	Age-standardized	12.3 (5.1–24.1)	12.2 (5.3–22.8)	12.3 (5.3–22.7)	12.5 (5.2–25.0)	12.5 (5.2–25.2)
Uruguay	All age	12.3 (5.1–24.0)	12.1 (5.3–22.7)	12.2 (5.3–22.7)	12.3 (5.1–24.8)	12.3 (5.1–25.0)
Western Europe	20 to 24	12.8 (6.2–23.0)	13.0 (6.9–22.8)	13.2 (8.4–21.3)	13.2 (8.1–22.3)	13.2 (7.7–22.4)
Western Europe	25 to 29	13.0 (6.2–23.3)	13.2 (6.8–23.1)	13.4 (8.2–21.8)	13.5 (8.3–22.6)	13.6 (8.0–22.7)
Western Europe	30 to 34	15.2 (8.1–25.9)	15.2 (8.3–25.4)	15.0 (9.5–23.6)	15.1 (8.9–25.9)	15.1 (8.9–25.9)
Western Europe	35 to 39	14.8 (7.4–26.2)	14.7 (7.5–25.6)	14.6 (8.8–23.7)	14.9 (8.7–25.6)	14.9 (8.4–26.0)
Western Europe	40 to 44	17.0 (9.2–28.9)	16.4 (8.9–27.7)	16.0 (9.9–25.8)	15.9 (10.1–26.2)	15.9 (9.7–26.5)
Western Europe	45 to 49	15.9 (8.3–27.1)	15.4 (8.0–26.6)	15.2 (8.8–25.3)	15.3 (9.1–26.5)	15.3 (9.0–26.5)
Western Europe	50 to 54	15.2 (7.6–26.7)	14.9 (7.6–25.9)	14.8 (8.4–24.8)	14.9 (8.6–26.4)	15.0 (8.6–26.1)
Western Europe	55 to 59	16.4 (8.8–27.8)	15.7 (8.4–26.7)	15.3 (9.0–25.3)	15.2 (8.9–26.4)	15.2 (8.8–26.1)
Western Europe	60 to 64	15.7 (8.4–26.8)	15.2 (7.7–26.4)	15.0 (8.7–24.9)	14.8 (8.6–26.2)	14.8 (8.5–26.1)
Western Europe	65 to 69	14.7 (7.6–26.1)	14.3 (7.6–24.8)	14.1 (8.2–23.9)	14.0 (8.1–24.9)	14.0 (7.9–24.7)
Western Europe	70 to 74	11.3 (5.2–21.3)	11.1 (4.7–21.3)	11.0 (4.8–20.9)	11.2 (5.1–21.7)	11.1 (5.2–21.9)
Western Europe	75 to 79	9.7 (3.8–19.6)	9.6 (3.6–19.7)	9.6 (3.7–19.2)	9.7 (3.8–20.4)	9.8 (4.0–20.6)

Supplementary Table S11: Prevalence of male SVAC by age and location for 1990, 2000, 2010, 2020, and 2023						
**Location**	**Age Range**	**1990**	**2000**	**2010**	**2020**	**2023**
Western Europe	80 to 84	7.0 (2.6–14.6)	7.0 (2.5–14.8)	6.9 (2.6–14.5)	7.0 (2.6–15.3)	7.0 (2.8–15.5)
Western Europe	85 to 89	6.3 (2.4–13.2)	6.3 (2.2–13.4)	6.3 (2.3–13.1)	6.3 (2.4–13.8)	6.3 (2.5–14.1)
Western Europe	90 to 94	5.8 (2.2–12.0)	5.7 (2.0–12.2)	5.7 (2.1–12.0)	5.7 (2.1–12.5)	5.7 (2.3–12.7)
Western Europe	95 plus	5.8 (2.2–12.1)	5.7 (2.0–12.2)	5.7 (2.1–12.0)	5.7 (2.1–12.5)	5.7 (2.2–12.6)
Western Europe	Age-standardized	14.4 (7.3–25.0)	14.2 (7.3–24.7)	14.1 (8.5–23.3)	14.1 (8.3–24.5)	14.1 (8.1–24.5)
Western Europe	All age	14.4 (7.3–25.1)	14.2 (7.3–24.8)	13.9 (8.2–23.3)	13.8 (8.0–24.3)	13.8 (7.8–24.3)
Andorra	20 to 24	12.8 (4.9–25.8)	12.8 (4.8–25.8)	12.8 (4.9–25.3)	12.8 (5.1–26.8)	12.8 (5.2–26.5)
Andorra	25 to 29	13.2 (5.1–26.4)	13.2 (4.9–26.4)	13.1 (5.0–25.9)	13.2 (5.3–27.4)	13.2 (5.4–27.1)
Andorra	30 to 34	14.4 (5.6–28.6)	14.4 (5.4–28.6)	14.4 (5.6–28.0)	14.5 (5.9–29.6)	14.5 (5.9–29.3)
Andorra	35 to 39	14.7 (5.8–29.1)	14.7 (5.6–29.1)	14.7 (5.7–28.5)	14.8 (6.0–30.1)	14.8 (6.1–29.8)
Andorra	40 to 44	15.4 (6.1–30.2)	15.4 (5.9–30.2)	15.4 (6.0–29.6)	15.5 (6.3–31.3)	15.4 (6.4–31.0)
Andorra	45 to 49	15.2 (6.0–29.8)	15.2 (5.7–29.8)	15.1 (5.9–29.2)	15.2 (6.2–30.9)	15.2 (6.3–30.6)
Andorra	50 to 54	15.0 (5.9–29.5)	15.0 (5.7–29.5)	14.9 (5.8–28.9)	15.0 (6.1–30.6)	15.0 (6.2–30.2)
Andorra	55 to 59	14.7 (5.7–29.0)	14.7 (5.5–29.0)	14.6 (5.7–28.4)	14.7 (6.0–30.0)	14.7 (6.0–29.7)
Andorra	60 to 64	14.3 (5.6–28.4)	14.3 (5.4–28.4)	14.3 (5.5–27.8)	14.4 (5.8–29.4)	14.4 (5.9–29.0)

Supplementary Table S11: Prevalence of male SVAC by age and location for 1990, 2000, 2010, 2020, and 2023						
**Location**	**Age Range**	**1990**	**2000**	**2010**	**2020**	**2023**
Andorra	65 to 69	13.4 (5.2–26.9)	13.4 (5.0–26.8)	13.4 (5.1–26.3)	13.5 (5.4–27.8)	13.4 (5.5–27.5)
Andorra	70 to 74	10.8 (4.1–22.1)	10.8 (3.9–22.1)	10.7 (4.0–21.6)	10.8 (4.2–22.9)	10.8 (4.3–22.7)
Andorra	75 to 79	9.7 (3.6–20.1)	9.7 (3.5–20.1)	9.7 (3.6–19.6)	9.7 (3.8–20.9)	9.7 (3.8–20.6)
Andorra	80 to 84	6.9 (2.5–14.6)	6.9 (2.4–14.6)	6.8 (2.5–14.3)	6.9 (2.6–15.3)	6.9 (2.6–15.1)
Andorra	85 to 89	6.2 (2.2–13.3)	6.2 (2.1–13.3)	6.2 (2.2–13.0)	6.2 (2.3–13.9)	6.2 (2.4–13.7)
Andorra	90 to 94	5.6 (2.0–12.1)	5.6 (1.9–12.1)	5.6 (2.0–11.8)	5.6 (2.1–12.6)	5.6 (2.1–12.5)
Andorra	95 plus	5.6 (2.0–12.0)	5.6 (1.9–12.0)	5.5 (2.0–11.7)	5.6 (2.1–12.5)	5.6 (2.1–12.4)
Andorra	Age-standardized	13.8 (5.4–27.4)	13.8 (5.2–27.4)	13.7 (5.3–26.8)	13.8 (5.6–28.4)	13.8 (5.6–28.1)
Andorra	All age	13.9 (5.4–27.6)	13.9 (5.2–27.6)	13.9 (5.4–27.1)	13.8 (5.6–28.4)	13.8 (5.6–28.1)
Austria	20 to 24	12.8 (4.9–25.8)	12.8 (4.8–25.8)	12.8 (4.9–25.3)	12.8 (5.1–26.8)	12.8 (5.2–26.5)
Austria	25 to 29	13.2 (5.1–26.4)	13.2 (4.9–26.4)	13.1 (5.0–25.9)	13.2 (5.3–27.4)	13.2 (5.4–27.1)
Austria	30 to 34	14.4 (5.6–28.6)	14.4 (5.4–28.6)	14.4 (5.6–28.0)	14.5 (5.9–29.6)	14.5 (5.9–29.3)
Austria	35 to 39	14.7 (5.8–29.1)	14.7 (5.6–29.1)	14.7 (5.7–28.5)	14.8 (6.0–30.1)	14.8 (6.1–29.8)
Austria	40 to 44	15.4 (6.1–30.2)	15.4 (5.9–30.2)	15.4 (6.0–29.6)	15.5 (6.3–31.3)	15.4 (6.4–31.0)
Austria	45 to 49	15.2 (6.0–29.8)	15.2 (5.7–29.8)	15.1 (5.9–29.2)	15.2 (6.2–30.9)	15.2 (6.3–30.6)

Supplementary Table S11: Prevalence of male SVAC by age and location for 1990, 2000, 2010, 2020, and 2023						
**Location**	**Age Range**	**1990**	**2000**	**2010**	**2020**	**2023**
Austria	50 to 54	15.0 (5.9–29.5)	15.0 (5.7–29.5)	14.9 (5.8–28.9)	15.0 (6.1–30.6)	15.0 (6.2–30.2)
Austria	55 to 59	14.7 (5.7–29.0)	14.7 (5.5–29.0)	14.6 (5.7–28.4)	14.7 (6.0–30.0)	14.7 (6.0–29.7)
Austria	60 to 64	14.3 (5.6–28.4)	14.3 (5.4–28.4)	14.3 (5.5–27.8)	14.4 (5.8–29.4)	14.4 (5.9–29.0)
Austria	65 to 69	13.4 (5.2–26.9)	13.4 (5.0–26.8)	13.4 (5.1–26.3)	13.5 (5.4–27.8)	13.4 (5.5–27.5)
Austria	70 to 74	10.8 (4.1–22.1)	10.8 (3.9–22.1)	10.7 (4.0–21.6)	10.8 (4.2–22.9)	10.8 (4.3–22.7)
Austria	75 to 79	9.7 (3.6–20.1)	9.7 (3.5–20.1)	9.7 (3.6–19.6)	9.7 (3.8–20.9)	9.7 (3.8–20.6)
Austria	80 to 84	6.9 (2.5–14.6)	6.9 (2.4–14.6)	6.8 (2.5–14.3)	6.9 (2.6–15.3)	6.9 (2.6–15.1)
Austria	85 to 89	6.2 (2.2–13.3)	6.2 (2.1–13.3)	6.2 (2.2–13.0)	6.2 (2.3–13.9)	6.2 (2.4–13.7)
Austria	90 to 94	5.6 (2.0–12.1)	5.6 (1.9–12.1)	5.6 (2.0–11.8)	5.6 (2.1–12.6)	5.6 (2.1–12.5)
Austria	95 plus	5.6 (2.0–12.0)	5.6 (1.9–12.0)	5.5 (2.0–11.7)	5.6 (2.1–12.5)	5.6 (2.1–12.4)
Austria	Age-standardized	13.8 (5.4–27.4)	13.8 (5.2–27.4)	13.7 (5.3–26.8)	13.8 (5.6–28.4)	13.8 (5.6–28.1)
Austria	All age	13.8 (5.4–27.4)	13.8 (5.2–27.5)	13.7 (5.3–26.8)	13.6 (5.5–28.0)	13.6 (5.5–27.6)
Belgium	20 to 24	8.0 (3.1–16.4)	7.2 (3.0–14.3)	6.8 (3.1–12.6)	7.4 (3.1–14.9)	7.6 (3.1–15.7)
Belgium	25 to 29	8.2 (3.2–16.9)	7.4 (3.1–14.7)	7.0 (3.2–12.8)	7.6 (3.2–15.3)	7.8 (3.2–16.1)
Belgium	30 to 34	10.1 (4.1–19.8)	9.7 (4.7–16.9)	9.4 (5.6–14.4)	9.8 (4.6–17.5)	10.0 (4.2–18.8)

Supplementary Table S11: Prevalence of male SVAC by age and location for 1990, 2000, 2010, 2020, and 2023						
**Location**	**Age Range**	**1990**	**2000**	**2010**	**2020**	**2023**
Belgium	35 to 39	10.0 (4.0–19.7)	9.2 (4.4–16.4)	8.8 (4.9–14.0)	9.4 (4.4–17.0)	9.7 (4.0–18.1)
Belgium	40 to 44	12.0 (5.0–22.9)	12.1 (6.2–20.3)	12.1 (7.7–17.4)	12.2 (6.0–21.1)	12.1 (5.3–22.3)
Belgium	45 to 49	11.3 (4.7–21.8)	11.0 (5.5–18.9)	10.8 (6.7–16.0)	11.2 (5.4–19.5)	11.3 (4.9–21.1)
Belgium	50 to 54	10.8 (4.4–20.9)	10.2 (5.0–17.6)	9.8 (5.9–14.8)	10.4 (4.9–18.3)	10.5 (4.5–19.9)
Belgium	55 to 59	11.9 (4.9–22.7)	12.2 (6.3–20.3)	12.3 (8.0–17.5)	12.2 (6.0–21.2)	12.1 (5.4–22.3)
Belgium	60 to 64	11.2 (4.6–21.5)	11.1 (5.7–18.8)	11.0 (7.1–15.9)	11.2 (5.5–19.6)	11.2 (4.9–20.8)
Belgium	65 to 69	11.1 (4.6–21.4)	11.6 (6.0–19.3)	11.8 (7.8–16.7)	11.6 (5.7–20.0)	11.5 (5.0–21.1)
Belgium	70 to 74	8.1 (3.2–16.3)	7.9 (3.7–14.4)	7.8 (4.2–12.7)	8.0 (3.7–14.8)	8.1 (3.3–15.3)
Belgium	75 to 79	7.3 (2.7–15.4)	7.2 (2.5–15.4)	7.2 (2.6–15.0)	7.3 (2.8–16.1)	7.3 (2.8–15.9)
Belgium	80 to 84	5.1 (1.8–11.1)	5.1 (1.7–11.0)	5.1 (1.8–10.7)	5.1 (1.9–11.5)	5.1 (1.9–11.4)
Belgium	85 to 89	4.6 (1.6–10.0)	4.6 (1.6– 9.9)	4.5 (1.6– 9.7)	4.6 (1.7–10.4)	4.6 (1.7–10.3)
Belgium	90 to 94	4.1 (1.5– 9.1)	4.1 (1.4– 9.0)	4.1 (1.4– 8.8)	4.1 (1.5– 9.5)	4.1 (1.5– 9.3)
Belgium	95 plus	4.1 (1.5– 9.0)	4.1 (1.4– 8.9)	4.1 (1.4– 8.7)	4.1 (1.5– 9.4)	4.1 (1.5– 9.2)
Belgium	Age-standardized	9.9 (4.1–19.3)	9.5 (4.5–17.0)	9.3 (5.3–14.7)	9.6 (4.6–17.3)	9.7 (4.1–18.0)
Belgium	All age	10.0 (4.1–19.5)	9.7 (4.6–17.2)	9.5 (5.5–14.8)	9.8 (4.7–17.4)	9.8 (4.2–18.2)

Supplementary Table S11: Prevalence of male SVAC by age and location for 1990, 2000, 2010, 2020, and 2023						
**Location**	**Age Range**	**1990**	**2000**	**2010**	**2020**	**2023**
Cyprus	20 to 24	12.8 (4.9–25.8)	12.8 (4.8–25.8)	12.8 (4.9–25.3)	12.8 (5.1–26.8)	12.8 (5.2–26.5)
Cyprus	25 to 29	13.2 (5.1–26.4)	13.2 (4.9–26.4)	13.1 (5.0–25.9)	13.2 (5.3–27.4)	13.2 (5.4–27.1)
Cyprus	30 to 34	14.4 (5.6–28.6)	14.4 (5.4–28.6)	14.4 (5.6–28.0)	14.5 (5.9–29.6)	14.5 (5.9–29.3)
Cyprus	35 to 39	14.7 (5.8–29.1)	14.7 (5.6–29.1)	14.7 (5.7–28.5)	14.8 (6.0–30.1)	14.8 (6.1–29.8)
Cyprus	40 to 44	15.4 (6.1–30.2)	15.4 (5.9–30.2)	15.4 (6.0–29.6)	15.5 (6.3–31.3)	15.4 (6.4–31.0)
Cyprus	45 to 49	15.2 (6.0–29.8)	15.2 (5.7–29.8)	15.1 (5.9–29.2)	15.2 (6.2–30.9)	15.2 (6.3–30.6)
Cyprus	50 to 54	15.0 (5.9–29.5)	15.0 (5.7–29.5)	14.9 (5.8–28.9)	15.0 (6.1–30.6)	15.0 (6.2–30.2)
Cyprus	55 to 59	14.7 (5.7–29.0)	14.7 (5.5–29.0)	14.6 (5.7–28.4)	14.7 (6.0–30.0)	14.7 (6.0–29.7)
Cyprus	60 to 64	14.3 (5.6–28.4)	14.3 (5.4–28.4)	14.3 (5.5–27.8)	14.4 (5.8–29.4)	14.4 (5.9–29.0)
Cyprus	65 to 69	13.4 (5.2–26.9)	13.4 (5.0–26.8)	13.4 (5.1–26.3)	13.5 (5.4–27.8)	13.4 (5.5–27.5)
Cyprus	70 to 74	10.8 (4.1–22.1)	10.8 (3.9–22.1)	10.7 (4.0–21.6)	10.8 (4.2–22.9)	10.8 (4.3–22.7)
Cyprus	75 to 79	9.7 (3.6–20.1)	9.7 (3.5–20.1)	9.7 (3.6–19.6)	9.7 (3.8–20.9)	9.7 (3.8–20.6)
Cyprus	80 to 84	6.9 (2.5–14.6)	6.9 (2.4–14.6)	6.8 (2.5–14.3)	6.9 (2.6–15.3)	6.9 (2.6–15.1)
Cyprus	85 to 89	6.2 (2.2–13.3)	6.2 (2.1–13.3)	6.2 (2.2–13.0)	6.2 (2.3–13.9)	6.2 (2.4–13.7)
Cyprus	90 to 94	5.6 (2.0–12.1)	5.6 (1.9–12.1)	5.6 (2.0–11.8)	5.6 (2.1–12.6)	5.6 (2.1–12.5)

Supplementary Table S11: Prevalence of male SVAC by age and location for 1990, 2000, 2010, 2020, and 2023						
**Location**	**Age Range**	**1990**	**2000**	**2010**	**2020**	**2023**
Cyprus	95 plus	5.6 (2.0–12.0)	5.6 (1.9–12.0)	5.5 (2.0–11.7)	5.6 (2.1–12.5)	5.6 (2.1–12.4)
Cyprus	Age-standardized	13.8 (5.4–27.4)	13.8 (5.2–27.4)	13.7 (5.3–26.8)	13.8 (5.6–28.4)	13.8 (5.6–28.1)
Cyprus	All age	13.8 (5.4–27.4)	13.8 (5.2–27.5)	13.7 (5.3–26.8)	13.7 (5.5–28.3)	13.7 (5.6–27.9)
Denmark	20 to 24	15.9 (5.4–32.9)	15.6 (5.9–30.9)	15.5 (6.1–29.8)	15.7 (5.3–33.0)	15.7 (5.2–33.3)
Denmark	25 to 29	16.1 (5.5–33.2)	15.8 (5.9–31.3)	15.7 (6.2–30.2)	15.9 (5.4–33.3)	15.9 (5.3–33.7)
Denmark	30 to 34	17.3 (6.0–35.2)	17.0 (6.5–33.2)	16.8 (6.7–32.1)	17.0 (5.9–35.3)	17.1 (5.8–35.7)
Denmark	35 to 39	17.4 (6.0–35.4)	17.1 (6.5–33.4)	16.9 (6.8–32.2)	17.1 (5.9–35.5)	17.2 (5.8–35.9)
Denmark	40 to 44	17.9 (6.2–36.3)	17.6 (6.7–34.2)	17.5 (7.0–33.1)	17.7 (6.1–36.4)	17.7 (6.0–36.8)
Denmark	45 to 49	17.5 (6.0–35.5)	17.1 (6.5–33.5)	17.0 (6.8–32.4)	17.2 (6.0–35.4)	17.3 (5.9–35.8)
Denmark	50 to 54	17.1 (5.9–34.9)	16.8 (6.4–32.9)	16.7 (6.7–31.8)	16.9 (5.8–35.1)	16.9 (5.7–35.4)
Denmark	55 to 59	16.6 (5.7–34.1)	16.3 (6.2–32.1)	16.2 (6.4–31.0)	16.4 (5.6–34.2)	16.4 (5.5–34.6)
Denmark	60 to 64	16.1 (5.5–33.3)	15.8 (6.0–31.3)	15.7 (6.2–30.2)	15.9 (5.4–33.4)	16.0 (5.3–33.8)
Denmark	65 to 69	15.1 (5.1–31.4)	14.8 (5.5–29.5)	14.7 (5.7–28.5)	14.9 (5.0–31.6)	14.9 (4.9–31.9)
Denmark	70 to 74	12.1 (4.0–26.1)	11.8 (4.3–24.4)	11.8 (4.5–23.5)	11.9 (3.9–26.2)	12.0 (3.8–26.5)
Denmark	75 to 79	10.9 (3.5–23.8)	10.7 (3.8–22.2)	10.6 (4.0–21.4)	10.8 (3.5–23.9)	10.8 (3.4–24.2)

Supplementary Table S11: Prevalence of male SVAC by age and location for 1990, 2000, 2010, 2020, and 2023						
**Location**	**Age Range**	**1990**	**2000**	**2010**	**2020**	**2023**
Denmark	80 to 84	7.8 (2.4–17.5)	7.6 (2.6–16.3)	7.5 (2.7–15.6)	7.6 (2.4–17.6)	7.7 (2.3–17.9)
Denmark	85 to 89	7.0 (2.2–15.9)	6.8 (2.3–14.8)	6.8 (2.5–14.1)	6.9 (2.1–16.0)	6.9 (2.1–16.2)
Denmark	90 to 94	6.3 (1.9–14.5)	6.2 (2.1–13.5)	6.1 (2.2–12.9)	6.2 (1.9–14.6)	6.3 (1.9–14.8)
Denmark	95 plus	6.3 (1.9–14.4)	6.1 (2.1–13.4)	6.1 (2.2–12.8)	6.2 (1.9–14.5)	6.2 (1.9–14.7)
Denmark	Age-standardized	16.2 (5.5–33.2)	15.8 (6.0–31.3)	15.7 (6.2–30.2)	15.9 (5.4–33.1)	16.0 (5.4–33.7)
Denmark	All age	16.1 (5.5–33.0)	15.8 (6.0–31.1)	15.6 (6.2–29.9)	15.4 (5.3–32.2)	15.4 (5.1–32.6)
Finland	20 to 24	9.9 (6.5–14.3)	9.5 (4.7–16.6)	9.9 (4.1–19.8)	10.3 (3.7–21.4)	10.4 (3.8–22.7)
Finland	25 to 29	11.0 (7.5–15.6)	10.8 (5.8–17.9)	10.8 (4.7–21.2)	10.9 (4.1–22.2)	10.9 (4.0–23.3)
Finland	30 to 34	11.5 (8.0–16.0)	11.4 (6.7–17.4)	11.2 (5.3–21.0)	11.5 (4.6–22.6)	11.6 (4.3–23.7)
Finland	35 to 39	9.5 (6.5–13.6)	10.1 (6.3–15.2)	10.7 (5.5–18.7)	11.3 (4.8–22.6)	11.4 (4.6–23.4)
Finland	40 to 44	10.2 (6.7–14.9)	10.5 (6.6–15.3)	10.9 (6.2–17.6)	11.3 (5.3–21.2)	11.5 (5.0–22.7)
Finland	45 to 49	9.7 (6.1–14.8)	10.0 (6.3–15.0)	10.7 (6.5–16.4)	11.0 (5.6–18.9)	11.1 (5.3–20.1)
Finland	50 to 54	10.6 (6.5–16.2)	10.3 (6.5–15.1)	10.6 (6.5–16.0)	10.9 (6.4–17.4)	10.9 (6.2–18.6)
Finland	55 to 59	12.9 (7.0–20.9)	11.5 (6.9–17.4)	10.7 (6.7–16.1)	11.3 (6.3–18.1)	11.4 (5.9–19.2)
Finland	60 to 64	11.3 (6.3–18.3)	10.7 (5.9–17.0)	10.2 (6.1–15.9)	10.6 (6.0–17.4)	10.8 (5.7–18.2)

Supplementary Table S11: Prevalence of male SVAC by age and location for 1990, 2000, 2010, 2020, and 2023						
**Location**	**Age Range**	**1990**	**2000**	**2010**	**2020**	**2023**
Finland	65 to 69	11.1 (5.9–18.7)	10.6 (5.4–18.0)	9.9 (5.4–16.2)	9.5 (5.4–15.6)	9.5 (5.0–15.9)
Finland	70 to 74	8.4 (3.2–17.6)	8.6 (3.4–17.1)	8.8 (4.2–16.0)	9.0 (4.7–15.6)	9.1 (4.6–15.8)
Finland	75 to 79	7.5 (2.7–15.7)	7.6 (2.9–16.0)	7.8 (3.3–15.4)	8.0 (3.9–15.4)	8.0 (3.7–15.0)
Finland	80 to 84	5.2 (1.8–11.2)	5.1 (1.8–11.2)	5.2 (1.8–11.0)	5.2 (1.9–11.8)	5.2 (2.0–11.8)
Finland	85 to 89	4.6 (1.7–10.1)	4.6 (1.6–10.1)	4.6 (1.6– 9.9)	4.7 (1.7–10.7)	4.7 (1.7–10.6)
Finland	90 to 94	4.2 (1.5– 9.1)	4.2 (1.4– 9.1)	4.2 (1.5– 9.0)	4.2 (1.6– 9.7)	4.2 (1.6– 9.7)
Finland	95 plus	4.1 (1.5– 9.1)	4.1 (1.4– 9.0)	4.1 (1.5– 8.9)	4.2 (1.5– 9.6)	4.2 (1.6– 9.6)
Finland	Age-standardized	10.3 (7.2–14.4)	10.2 (7.5–13.3)	10.3 (7.9–14.1)	10.6 (6.4–17.0)	10.7 (6.1–18.0)
Finland	All age	10.4 (7.3–14.4)	10.2 (7.6–13.3)	10.2 (8.0–12.8)	10.3 (6.4–15.6)	10.3 (5.9–16.6)
France	20 to 24	12.8 (4.9–25.8)	12.8 (4.8–25.8)	12.8 (4.9–25.3)	12.8 (5.1–26.8)	12.8 (5.2–26.5)
France	25 to 29	13.2 (5.1–26.4)	13.2 (4.9–26.4)	13.1 (5.0–25.9)	13.2 (5.3–27.4)	13.2 (5.4–27.1)
France	30 to 34	14.4 (5.6–28.6)	14.4 (5.4–28.6)	14.4 (5.6–28.0)	14.5 (5.9–29.6)	14.5 (5.9–29.3)
France	35 to 39	14.7 (5.8–29.1)	14.7 (5.6–29.1)	14.7 (5.7–28.5)	14.8 (6.0–30.1)	14.8 (6.1–29.8)
France	40 to 44	15.4 (6.1–30.2)	15.4 (5.9–30.2)	15.4 (6.0–29.6)	15.5 (6.3–31.3)	15.4 (6.4–31.0)
France	45 to 49	15.2 (6.0–29.8)	15.2 (5.7–29.8)	15.1 (5.9–29.2)	15.2 (6.2–30.9)	15.2 (6.3–30.6)

Supplementary Table S11: Prevalence of male SVAC by age and location for 1990, 2000, 2010, 2020, and 2023						
**Location**	**Age Range**	**1990**	**2000**	**2010**	**2020**	**2023**
France	50 to 54	15.0 (5.9–29.5)	15.0 (5.7–29.5)	14.9 (5.8–28.9)	15.0 (6.1–30.6)	15.0 (6.2–30.2)
France	55 to 59	14.7 (5.7–29.0)	14.7 (5.5–29.0)	14.6 (5.7–28.4)	14.7 (6.0–30.0)	14.7 (6.0–29.7)
France	60 to 64	14.3 (5.6–28.4)	14.3 (5.4–28.4)	14.3 (5.5–27.8)	14.4 (5.8–29.4)	14.4 (5.9–29.0)
France	65 to 69	13.4 (5.2–26.9)	13.4 (5.0–26.8)	13.4 (5.1–26.3)	13.5 (5.4–27.8)	13.4 (5.5–27.5)
France	70 to 74	10.8 (4.1–22.1)	10.8 (3.9–22.1)	10.7 (4.0–21.6)	10.8 (4.2–22.9)	10.8 (4.3–22.7)
France	75 to 79	9.7 (3.6–20.1)	9.7 (3.5–20.1)	9.7 (3.6–19.6)	9.7 (3.8–20.9)	9.7 (3.8–20.6)
France	80 to 84	6.9 (2.5–14.6)	6.9 (2.4–14.6)	6.8 (2.5–14.3)	6.9 (2.6–15.3)	6.9 (2.6–15.1)
France	85 to 89	6.2 (2.2–13.3)	6.2 (2.1–13.3)	6.2 (2.2–13.0)	6.2 (2.3–13.9)	6.2 (2.4–13.7)
France	90 to 94	5.6 (2.0–12.1)	5.6 (1.9–12.1)	5.6 (2.0–11.8)	5.6 (2.1–12.6)	5.6 (2.1–12.5)
France	95 plus	5.6 (2.0–12.0)	5.6 (1.9–12.0)	5.5 (2.0–11.7)	5.6 (2.1–12.5)	5.6 (2.1–12.4)
France	Age-standardized	13.8 (5.4–27.4)	13.8 (5.2–27.4)	13.7 (5.3–26.8)	13.8 (5.6–28.4)	13.8 (5.6–28.1)
France	All age	13.8 (5.4–27.4)	13.7 (5.1–27.3)	13.6 (5.2–26.5)	13.5 (5.4–27.8)	13.4 (5.5–27.4)
Germany	20 to 24	12.8 (4.9–25.8)	12.8 (4.8–25.8)	12.8 (4.9–25.3)	12.8 (5.1–26.8)	12.8 (5.2–26.5)
Germany	25 to 29	13.2 (5.1–26.4)	13.2 (4.9–26.4)	13.1 (5.0–25.9)	13.2 (5.3–27.4)	13.2 (5.4–27.1)
Germany	30 to 34	14.4 (5.6–28.6)	14.4 (5.4–28.6)	14.4 (5.6–28.0)	14.5 (5.9–29.6)	14.5 (5.9–29.3)

Supplementary Table S11: Prevalence of male SVAC by age and location for 1990, 2000, 2010, 2020, and 2023						
**Location**	**Age Range**	**1990**	**2000**	**2010**	**2020**	**2023**
Germany	35 to 39	14.7 (5.8–29.1)	14.7 (5.6–29.1)	14.7 (5.7–28.5)	14.8 (6.0–30.1)	14.8 (6.1–29.8)
Germany	40 to 44	15.4 (6.1–30.2)	15.4 (5.9–30.2)	15.4 (6.0–29.6)	15.5 (6.3–31.3)	15.4 (6.4–31.0)
Germany	45 to 49	15.2 (6.0–29.8)	15.2 (5.7–29.8)	15.1 (5.9–29.2)	15.2 (6.2–30.9)	15.2 (6.3–30.6)
Germany	50 to 54	15.0 (5.9–29.5)	15.0 (5.7–29.5)	14.9 (5.8–28.9)	15.0 (6.1–30.6)	15.0 (6.2–30.2)
Germany	55 to 59	14.7 (5.7–29.0)	14.7 (5.5–29.0)	14.6 (5.7–28.4)	14.7 (6.0–30.0)	14.7 (6.0–29.7)
Germany	60 to 64	14.3 (5.6–28.4)	14.3 (5.4–28.4)	14.3 (5.5–27.8)	14.4 (5.8–29.4)	14.4 (5.9–29.0)
Germany	65 to 69	13.4 (5.2–26.9)	13.4 (5.0–26.8)	13.4 (5.1–26.3)	13.5 (5.4–27.8)	13.4 (5.5–27.5)
Germany	70 to 74	10.8 (4.1–22.1)	10.8 (3.9–22.1)	10.7 (4.0–21.6)	10.8 (4.2–22.9)	10.8 (4.3–22.7)
Germany	75 to 79	9.7 (3.6–20.1)	9.7 (3.5–20.1)	9.7 (3.6–19.6)	9.7 (3.8–20.9)	9.7 (3.8–20.6)
Germany	80 to 84	6.9 (2.5–14.6)	6.9 (2.4–14.6)	6.8 (2.5–14.3)	6.9 (2.6–15.3)	6.9 (2.6–15.1)
Germany	85 to 89	6.2 (2.2–13.3)	6.2 (2.1–13.3)	6.2 (2.2–13.0)	6.2 (2.3–13.9)	6.2 (2.4–13.7)
Germany	90 to 94	5.6 (2.0–12.1)	5.6 (1.9–12.1)	5.6 (2.0–11.8)	5.6 (2.1–12.6)	5.6 (2.1–12.5)
Germany	95 plus	5.6 (2.0–12.0)	5.6 (1.9–12.0)	5.5 (2.0–11.7)	5.6 (2.1–12.5)	5.6 (2.1–12.4)
Germany	Age-standardized	13.8 (5.4–27.4)	13.8 (5.2–27.4)	13.7 (5.3–26.8)	13.8 (5.6–28.4)	13.8 (5.6–28.1)
Germany	All age	13.8 (5.4–27.5)	13.9 (5.2–27.5)	13.6 (5.2–26.5)	13.4 (5.4–27.7)	13.4 (5.5–27.3)

Supplementary Table S11: Prevalence of male SVAC by age and location for 1990, 2000, 2010, 2020, and 2023						
**Location**	**Age Range**	**1990**	**2000**	**2010**	**2020**	**2023**
Greece	20 to 24	14.7 (5.8–29.1)	14.8 (5.6–29.1)	14.9 (5.8–28.8)	14.9 (6.1–30.4)	14.9 (6.2–30.1)
Greece	25 to 29	14.9 (5.9–29.5)	15.0 (5.7–29.5)	15.1 (5.9–29.2)	15.2 (6.2–30.8)	15.2 (6.3–30.5)
Greece	30 to 34	16.1 (6.4–31.4)	16.1 (6.2–31.4)	16.2 (6.4–31.1)	16.3 (6.7–32.8)	16.3 (6.8–32.4)
Greece	35 to 39	16.2 (6.5–31.6)	16.3 (6.2–31.6)	16.4 (6.5–31.3)	16.4 (6.8–33.0)	16.4 (6.9–32.6)
Greece	40 to 44	16.8 (6.7–32.5)	16.8 (6.5–32.5)	16.9 (6.7–32.2)	17.0 (7.0–33.9)	17.0 (7.1–33.5)
Greece	45 to 49	16.4 (6.5–31.9)	16.4 (6.3–31.9)	16.5 (6.5–31.5)	16.6 (6.8–33.2)	16.6 (6.9–32.9)
Greece	50 to 54	16.1 (6.4–31.4)	16.1 (6.2–31.4)	16.2 (6.4–31.0)	16.3 (6.7–32.7)	16.3 (6.8–32.3)
Greece	55 to 59	15.6 (6.2–30.6)	15.7 (6.0–30.6)	15.7 (6.2–30.2)	15.8 (6.5–31.9)	15.8 (6.6–31.6)
Greece	60 to 64	15.2 (6.0–29.9)	15.2 (5.8–29.9)	15.3 (6.0–29.5)	15.4 (6.3–31.2)	15.4 (6.4–30.8)
Greece	65 to 69	14.2 (5.5–28.2)	14.2 (5.3–28.2)	14.3 (5.5–27.8)	14.3 (5.8–29.4)	14.3 (5.9–29.0)
Greece	70 to 74	11.4 (4.3–23.2)	11.4 (4.2–23.2)	11.4 (4.3–22.9)	11.5 (4.5–24.3)	11.5 (4.6–24.0)
Greece	75 to 79	10.3 (3.9–21.2)	10.3 (3.7–21.2)	10.3 (3.8–20.8)	10.4 (4.0–22.1)	10.3 (4.1–21.9)
Greece	80 to 84	7.3 (2.7–15.4)	7.3 (2.6–15.4)	7.3 (2.6–15.2)	7.3 (2.8–16.2)	7.3 (2.8–16.0)
Greece	85 to 89	6.6 (2.4–14.0)	6.6 (2.3–14.0)	6.6 (2.4–13.8)	6.6 (2.5–14.7)	6.6 (2.5–14.5)
Greece	90 to 94	5.9 (2.1–12.8)	5.9 (2.1–12.8)	5.9 (2.1–12.5)	6.0 (2.2–13.4)	6.0 (2.3–13.2)

Supplementary Table S11: Prevalence of male SVAC by age and location for 1990, 2000, 2010, 2020, and 2023						
**Location**	**Age Range**	**1990**	**2000**	**2010**	**2020**	**2023**
Greece	95 plus	5.9 (2.1–12.7)	5.9 (2.0–12.7)	5.9 (2.1–12.4)	5.9 (2.2–13.3)	5.9 (2.2–13.1)
Greece	Age-standardized	15.1 (6.0–29.7)	15.1 (5.8–29.7)	15.2 (6.0–29.3)	15.3 (6.3–31.0)	15.3 (6.3–30.6)
Greece	All age	15.0 (5.9–29.5)	15.0 (5.7–29.4)	14.9 (5.8–28.7)	14.8 (6.0–30.0)	14.7 (6.1–29.6)
Iceland	20 to 24	10.5 (4.7–19.3)	10.0 (5.8–15.5)	9.4 (7.5–11.6)	9.7 (5.2–16.0)	9.8 (4.7–18.1)
Iceland	25 to 29	10.9 (4.0–22.2)	10.4 (4.7–19.1)	9.7 (6.4–14.3)	9.7 (6.0–14.3)	9.8 (5.6–16.1)
Iceland	30 to 34	11.8 (4.1–25.2)	11.3 (4.5–21.5)	10.3 (5.6–17.2)	9.6 (7.0–13.1)	9.7 (6.5–13.9)
Iceland	35 to 39	12.4 (4.0–26.7)	12.0 (4.5–23.6)	11.4 (5.1–21.4)	11.0 (5.9–19.0)	10.9 (5.8–18.1)
Iceland	40 to 44	13.1 (4.2–28.6)	12.9 (4.8–26.5)	12.8 (4.9–25.4)	12.9 (5.1–26.8)	12.9 (5.2–26.5)
Iceland	45 to 49	12.9 (4.1–28.2)	12.6 (4.6–26.0)	12.6 (4.8–24.9)	12.6 (5.0–26.3)	12.6 (5.0–26.0)
Iceland	50 to 54	12.6 (4.0–27.5)	12.4 (4.5–25.7)	12.3 (4.7–24.5)	12.4 (4.9–25.9)	12.3 (4.9–25.6)
Iceland	55 to 59	12.3 (3.9–26.9)	12.0 (4.4–25.0)	12.0 (4.5–23.8)	12.0 (4.7–25.2)	12.0 (4.8–24.9)
Iceland	60 to 64	11.9 (3.8–26.3)	11.7 (4.3–24.4)	11.6 (4.4–23.2)	11.7 (4.6–24.6)	11.6 (4.6–24.3)
Iceland	65 to 69	11.1 (3.5–24.9)	10.9 (3.9–22.9)	10.8 (4.1–21.8)	10.8 (4.2–23.1)	10.8 (4.3–22.8)
Iceland	70 to 74	8.9 (2.7–20.4)	8.7 (3.1–18.6)	8.6 (3.2–17.7)	8.6 (3.3–18.8)	8.6 (3.3–18.6)
Iceland	75 to 79	8.0 (2.4–18.5)	7.8 (2.7–16.9)	7.7 (2.8–16.0)	7.8 (2.9–17.0)	7.7 (3.0–16.8)

Supplementary Table S11: Prevalence of male SVAC by age and location for 1990, 2000, 2010, 2020, and 2023						
**Location**	**Age Range**	**1990**	**2000**	**2010**	**2020**	**2023**
Iceland	80 to 84	5.6 (1.7–13.4)	5.5 (1.8–12.2)	5.4 (1.9–11.5)	5.4 (2.0–12.3)	5.4 (2.0–12.1)
Iceland	85 to 89	5.1 (1.5–12.0)	4.9 (1.6–11.0)	4.9 (1.7–10.4)	4.9 (1.8–11.1)	4.9 (1.8–10.9)
Iceland	90 to 94	4.6 (1.3–11.0)	4.4 (1.5–10.0)	4.4 (1.6– 9.4)	4.4 (1.6–10.1)	4.4 (1.6– 9.9)
Iceland	95 plus	4.5 (1.3–10.9)	4.4 (1.5– 9.9)	4.4 (1.5– 9.3)	4.4 (1.6–10.0)	4.4 (1.6– 9.8)
Iceland	Age-standardized	11.5 (4.3–23.7)	11.1 (4.6–21.5)	10.8 (5.5–19.0)	10.7 (6.7–18.0)	10.7 (6.7–17.6)
Iceland	All age	11.5 (4.3–23.6)	11.2 (4.6–21.7)	10.8 (5.4–19.4)	10.7 (6.3–18.6)	10.7 (6.3–18.3)
Ireland	20 to 24	14.8 (6.9–26.9)	13.6 (7.1–22.7)	14.5 (6.9–25.0)	16.4 (7.0–31.7)	16.8 (7.3–32.7)
Ireland	25 to 29	15.2 (7.1–27.5)	13.9 (7.3–23.1)	14.8 (7.1–25.5)	16.8 (7.3–32.4)	17.3 (7.6–33.4)
Ireland	30 to 34	22.2 (12.1–36.3)	22.2 (15.5–29.7)	22.1 (12.7–34.3)	21.9 (10.2–38.1)	21.8 (9.8–38.8)
Ireland	35 to 39	20.7 (11.0–34.5)	20.1 (13.5–27.6)	20.4 (11.5–32.5)	21.2 (9.7–37.1)	21.4 (9.6–38.0)
Ireland	40 to 44	28.0 (15.8–43.6)	29.5 (22.4–37.6)	28.3 (17.1–41.9)	26.1 (12.5–44.8)	25.5 (11.7–45.5)
Ireland	45 to 49	25.3 (14.0–40.1)	25.9 (18.9–34.0)	25.4 (15.0–38.6)	24.3 (11.4–42.6)	24.1 (10.8–43.5)
Ireland	50 to 54	23.0 (12.7–37.4)	22.9 (16.2–30.5)	22.9 (13.2–35.4)	22.8 (10.7–39.3)	22.8 (10.4–40.2)
Ireland	55 to 59	28.2 (16.0–43.8)	30.3 (23.2–38.2)	28.7 (17.4–42.3)	25.8 (12.5–44.4)	25.1 (11.5–45.1)
Ireland	60 to 64	25.7 (14.3–40.4)	26.9 (20.0–34.9)	25.9 (15.4–39.1)	24.1 (11.3–42.1)	23.6 (10.6–42.7)

Supplementary Table S11: Prevalence of male SVAC by age and location for 1990, 2000, 2010, 2020, and 2023						
**Location**	**Age Range**	**1990**	**2000**	**2010**	**2020**	**2023**
Ireland	65 to 69	26.8 (15.0–42.1)	29.1 (22.2–37.0)	27.4 (16.4–40.7)	24.2 (11.5–42.2)	23.4 (10.5–42.7)
Ireland	70 to 74	17.9 (9.3–30.5)	18.3 (12.1–25.5)	18.0 (9.9–29.1)	17.3 (7.6–31.4)	17.1 (7.4–31.7)
Ireland	75 to 79	12.3 (5.6–22.9)	11.6 (5.8–19.9)	12.1 (5.5–21.6)	13.2 (5.5–26.4)	13.4 (5.7–26.8)
Ireland	80 to 84	9.1 (3.9–17.8)	8.8 (3.9–16.4)	9.0 (3.8–17.3)	9.6 (3.9–20.2)	9.7 (3.9–20.3)
Ireland	85 to 89	8.4 (3.5–16.5)	8.1 (3.6–15.3)	8.3 (3.5–16.0)	8.7 (3.5–18.6)	8.8 (3.5–18.6)
Ireland	90 to 94	7.8 (3.2–15.4)	7.6 (3.4–14.3)	7.7 (3.2–14.9)	8.0 (3.2–17.2)	8.1 (3.2–17.1)
Ireland	95 plus	7.7 (3.2–15.3)	7.5 (3.3–14.2)	7.7 (3.2–14.8)	7.9 (3.2–17.0)	8.0 (3.2–17.0)
Ireland	Age-standardized	21.2 (11.4–34.5)	21.3 (15.2–28.4)	21.1 (16.4–27.9)	20.9 (14.6–30.5)	20.8 (13.6–31.6)
Ireland	All age	21.2 (11.5–34.6)	21.5 (15.3–28.6)	21.6 (16.4–29.2)	21.3 (13.9–32.3)	21.1 (13.1–33.4)
Israel	20 to 24	19.0 (7.8–36.1)	19.2 (7.6–36.3)	19.0 (7.7–35.4)	19.0 (8.0–37.1)	19.0 (8.1–36.6)
Israel	25 to 29	18.9 (7.7–36.0)	19.1 (7.5–36.2)	18.9 (7.7–35.3)	18.9 (8.0–37.0)	18.9 (8.1–36.6)
Israel	30 to 34	20.0 (8.3–37.6)	20.2 (8.0–37.8)	20.0 (8.2–36.9)	20.0 (8.5–38.6)	19.9 (8.6–38.2)
Israel	35 to 39	19.9 (8.2–37.4)	20.0 (8.0–37.6)	19.8 (8.1–36.7)	19.8 (8.4–38.4)	19.8 (8.5–38.0)
Israel	40 to 44	20.3 (8.4–38.0)	20.4 (8.1–38.2)	20.2 (8.3–37.3)	20.2 (8.6–39.0)	20.2 (8.7–38.6)
Israel	45 to 49	19.6 (8.1–37.0)	19.7 (7.8–37.1)	19.5 (8.0–36.3)	19.6 (8.3–38.0)	19.5 (8.4–37.6)

Supplementary Table S11: Prevalence of male SVAC by age and location for 1990, 2000, 2010, 2020, and 2023						
**Location**	**Age Range**	**1990**	**2000**	**2010**	**2020**	**2023**
Israel	50 to 54	19.0 (7.8–36.1)	19.2 (7.6–36.3)	19.0 (7.7–35.4)	19.0 (8.0–37.1)	19.0 (8.1–36.8)
Israel	55 to 59	18.4 (7.5–35.1)	18.5 (7.3–35.3)	18.3 (7.4–34.4)	18.4 (7.7–36.1)	18.4 (7.8–35.7)
Israel	60 to 64	17.8 (7.2–34.2)	17.9 (7.0–34.3)	17.7 (7.1–33.5)	17.8 (7.4–35.1)	17.7 (7.5–34.8)
Israel	65 to 69	16.6 (6.6–32.2)	16.7 (6.4–32.3)	16.5 (6.5–31.5)	16.5 (6.8–33.1)	16.5 (6.9–32.8)
Israel	70 to 74	13.3 (5.2–26.7)	13.4 (5.0–26.8)	13.3 (5.1–26.1)	13.3 (5.3–27.5)	13.3 (5.4–27.2)
Israel	75 to 79	12.0 (4.6–24.3)	12.1 (4.4–24.4)	11.9 (4.5–23.8)	12.0 (4.7–25.1)	12.0 (4.8–24.8)
Israel	80 to 84	8.5 (3.2–17.9)	8.6 (3.1–18.0)	8.5 (3.1–17.5)	8.5 (3.3–18.6)	8.5 (3.3–18.3)
Israel	85 to 89	7.7 (2.8–16.3)	7.7 (2.7–16.4)	7.7 (2.8–15.9)	7.7 (2.9–16.9)	7.7 (3.0–16.7)
Israel	90 to 94	7.0 (2.5–14.8)	7.0 (2.5–14.9)	6.9 (2.5–14.5)	6.9 (2.6–15.4)	6.9 (2.7–15.1)
Israel	95 plus	6.9 (2.5–14.7)	6.9 (2.4–14.8)	6.9 (2.5–14.4)	6.9 (2.6–15.3)	6.9 (2.6–15.1)
Israel	Age-standardized	18.4 (7.5–35.0)	18.6 (7.3–35.2)	18.4 (7.4–34.4)	18.4 (7.7–36.0)	18.4 (7.8–35.6)
Israel	All age	18.4 (7.5–35.1)	18.6 (7.3–35.3)	18.4 (7.4–34.3)	18.2 (7.7–35.7)	18.2 (7.7–35.3)
Italy	20 to 24	12.8 (4.9–25.8)	12.8 (4.8–25.8)	12.8 (4.9–25.3)	12.8 (5.1–26.8)	12.8 (5.2–26.5)
Italy	25 to 29	13.2 (5.1–26.4)	13.2 (4.9–26.4)	13.1 (5.0–25.9)	13.2 (5.3–27.4)	13.2 (5.4–27.1)
Italy	30 to 34	14.4 (5.6–28.6)	14.4 (5.4–28.6)	14.4 (5.6–28.0)	14.5 (5.9–29.6)	14.5 (5.9–29.3)

Supplementary Table S11: Prevalence of male SVAC by age and location for 1990, 2000, 2010, 2020, and 2023						
**Location**	**Age Range**	**1990**	**2000**	**2010**	**2020**	**2023**
Italy	35 to 39	14.7 (5.8–29.1)	14.7 (5.6–29.1)	14.7 (5.7–28.5)	14.8 (6.0–30.1)	14.8 (6.1–29.8)
Italy	40 to 44	15.4 (6.1–30.2)	15.4 (5.9–30.2)	15.4 (6.0–29.6)	15.5 (6.3–31.3)	15.4 (6.4–31.0)
Italy	45 to 49	15.2 (6.0–29.8)	15.2 (5.7–29.8)	15.1 (5.9–29.2)	15.2 (6.2–30.9)	15.2 (6.3–30.6)
Italy	50 to 54	15.0 (5.9–29.5)	15.0 (5.7–29.5)	14.9 (5.8–28.9)	15.0 (6.1–30.6)	15.0 (6.2–30.2)
Italy	55 to 59	14.7 (5.7–29.0)	14.7 (5.5–29.0)	14.6 (5.7–28.4)	14.7 (6.0–30.0)	14.7 (6.0–29.7)
Italy	60 to 64	14.3 (5.6–28.4)	14.3 (5.4–28.4)	14.3 (5.5–27.8)	14.4 (5.8–29.4)	14.4 (5.9–29.0)
Italy	65 to 69	13.4 (5.2–26.9)	13.4 (5.0–26.8)	13.4 (5.1–26.3)	13.5 (5.4–27.8)	13.4 (5.5–27.5)
Italy	70 to 74	10.8 (4.1–22.1)	10.8 (3.9–22.1)	10.7 (4.0–21.6)	10.8 (4.2–22.9)	10.8 (4.3–22.7)
Italy	75 to 79	9.7 (3.6–20.1)	9.7 (3.5–20.1)	9.7 (3.6–19.6)	9.7 (3.8–20.9)	9.7 (3.8–20.6)
Italy	80 to 84	6.9 (2.5–14.6)	6.9 (2.4–14.6)	6.8 (2.5–14.3)	6.9 (2.6–15.3)	6.9 (2.6–15.1)
Italy	85 to 89	6.2 (2.2–13.3)	6.2 (2.1–13.3)	6.2 (2.2–13.0)	6.2 (2.3–13.9)	6.2 (2.4–13.7)
Italy	90 to 94	5.6 (2.0–12.1)	5.6 (1.9–12.1)	5.6 (2.0–11.8)	5.6 (2.1–12.6)	5.6 (2.1–12.5)
Italy	95 plus	5.6 (2.0–12.0)	5.6 (1.9–12.0)	5.5 (2.0–11.7)	5.6 (2.1–12.5)	5.6 (2.1–12.4)
Italy	Age-standardized	13.8 (5.4–27.4)	13.8 (5.2–27.4)	13.7 (5.3–26.8)	13.8 (5.6–28.4)	13.8 (5.6–28.1)
Italy	All age	13.7 (5.4–27.3)	13.7 (5.1–27.2)	13.5 (5.2–26.5)	13.4 (5.4–27.7)	13.4 (5.4–27.2)

Supplementary Table S11: Prevalence of male SVAC by age and location for 1990, 2000, 2010, 2020, and 2023						
**Location**	**Age Range**	**1990**	**2000**	**2010**	**2020**	**2023**
Luxembourg	20 to 24	12.8 (4.9–25.8)	12.8 (4.8–25.8)	12.8 (4.9–25.3)	12.8 (5.1–26.8)	12.8 (5.2–26.5)
Luxembourg	25 to 29	13.2 (5.1–26.4)	13.2 (4.9–26.4)	13.1 (5.0–25.9)	13.2 (5.3–27.4)	13.2 (5.4–27.1)
Luxembourg	30 to 34	14.4 (5.6–28.6)	14.4 (5.4–28.6)	14.4 (5.6–28.0)	14.5 (5.9–29.6)	14.5 (5.9–29.3)
Luxembourg	35 to 39	14.7 (5.8–29.1)	14.7 (5.6–29.1)	14.7 (5.7–28.5)	14.8 (6.0–30.1)	14.8 (6.1–29.8)
Luxembourg	40 to 44	15.4 (6.1–30.2)	15.4 (5.9–30.2)	15.4 (6.0–29.6)	15.5 (6.3–31.3)	15.4 (6.4–31.0)
Luxembourg	45 to 49	15.2 (6.0–29.8)	15.2 (5.7–29.8)	15.1 (5.9–29.2)	15.2 (6.2–30.9)	15.2 (6.3–30.6)
Luxembourg	50 to 54	15.0 (5.9–29.5)	15.0 (5.7–29.5)	14.9 (5.8–28.9)	15.0 (6.1–30.6)	15.0 (6.2–30.2)
Luxembourg	55 to 59	14.7 (5.7–29.0)	14.7 (5.5–29.0)	14.6 (5.7–28.4)	14.7 (6.0–30.0)	14.7 (6.0–29.7)
Luxembourg	60 to 64	14.3 (5.6–28.4)	14.3 (5.4–28.4)	14.3 (5.5–27.8)	14.4 (5.8–29.4)	14.4 (5.9–29.0)
Luxembourg	65 to 69	13.4 (5.2–26.9)	13.4 (5.0–26.8)	13.4 (5.1–26.3)	13.5 (5.4–27.8)	13.4 (5.5–27.5)
Luxembourg	70 to 74	10.8 (4.1–22.1)	10.8 (3.9–22.1)	10.7 (4.0–21.6)	10.8 (4.2–22.9)	10.8 (4.3–22.7)
Luxembourg	75 to 79	9.7 (3.6–20.1)	9.7 (3.5–20.1)	9.7 (3.6–19.6)	9.7 (3.8–20.9)	9.7 (3.8–20.6)
Luxembourg	80 to 84	6.9 (2.5–14.6)	6.9 (2.4–14.6)	6.8 (2.5–14.3)	6.9 (2.6–15.3)	6.9 (2.6–15.1)
Luxembourg	85 to 89	6.2 (2.2–13.3)	6.2 (2.1–13.3)	6.2 (2.2–13.0)	6.2 (2.3–13.9)	6.2 (2.4–13.7)
Luxembourg	90 to 94	5.6 (2.0–12.1)	5.6 (1.9–12.1)	5.6 (2.0–11.8)	5.6 (2.1–12.6)	5.6 (2.1–12.5)

Supplementary Table S11: Prevalence of male SVAC by age and location for 1990, 2000, 2010, 2020, and 2023						
**Location**	**Age Range**	**1990**	**2000**	**2010**	**2020**	**2023**
Luxembourg	95 plus	5.6 (2.0–12.0)	5.6 (1.9–12.0)	5.5 (2.0–11.7)	5.6 (2.1–12.5)	5.6 (2.1–12.4)
Luxembourg	Age-standardized	13.8 (5.4–27.4)	13.8 (5.2–27.4)	13.7 (5.3–26.8)	13.8 (5.6–28.4)	13.8 (5.6–28.1)
Luxembourg	All age	13.9 (5.4–27.6)	13.9 (5.2–27.6)	13.8 (5.3–27.0)	13.8 (5.6–28.4)	13.8 (5.6–28.0)
Malta	20 to 24	12.8 (4.9–25.8)	12.8 (4.8–25.8)	12.8 (4.9–25.3)	12.8 (5.1–26.8)	12.8 (5.2–26.5)
Malta	25 to 29	13.2 (5.1–26.4)	13.2 (4.9–26.4)	13.1 (5.0–25.9)	13.2 (5.3–27.4)	13.2 (5.4–27.1)
Malta	30 to 34	14.4 (5.6–28.6)	14.4 (5.4–28.6)	14.4 (5.6–28.0)	14.5 (5.9–29.6)	14.5 (5.9–29.3)
Malta	35 to 39	14.7 (5.8–29.1)	14.7 (5.6–29.1)	14.7 (5.7–28.5)	14.8 (6.0–30.1)	14.8 (6.1–29.8)
Malta	40 to 44	15.4 (6.1–30.2)	15.4 (5.9–30.2)	15.4 (6.0–29.6)	15.5 (6.3–31.3)	15.4 (6.4–31.0)
Malta	45 to 49	15.2 (6.0–29.8)	15.2 (5.7–29.8)	15.1 (5.9–29.2)	15.2 (6.2–30.9)	15.2 (6.3–30.6)
Malta	50 to 54	15.0 (5.9–29.5)	15.0 (5.7–29.5)	14.9 (5.8–28.9)	15.0 (6.1–30.6)	15.0 (6.2–30.2)
Malta	55 to 59	14.7 (5.7–29.0)	14.7 (5.5–29.0)	14.6 (5.7–28.4)	14.7 (6.0–30.0)	14.7 (6.0–29.7)
Malta	60 to 64	14.3 (5.6–28.4)	14.3 (5.4–28.4)	14.3 (5.5–27.8)	14.4 (5.8–29.4)	14.4 (5.9–29.0)
Malta	65 to 69	13.4 (5.2–26.9)	13.4 (5.0–26.8)	13.4 (5.1–26.3)	13.5 (5.4–27.8)	13.4 (5.5–27.5)
Malta	70 to 74	10.8 (4.1–22.1)	10.8 (3.9–22.1)	10.7 (4.0–21.6)	10.8 (4.2–22.9)	10.8 (4.3–22.7)
Malta	75 to 79	9.7 (3.6–20.1)	9.7 (3.5–20.1)	9.7 (3.6–19.6)	9.7 (3.8–20.9)	9.7 (3.8–20.6)

Supplementary Table S11: Prevalence of male SVAC by age and location for 1990, 2000, 2010, 2020, and 2023						
**Location**	**Age Range**	**1990**	**2000**	**2010**	**2020**	**2023**
Malta	80 to 84	6.9 (2.5–14.6)	6.9 (2.4–14.6)	6.8 (2.5–14.3)	6.9 (2.6–15.3)	6.9 (2.6–15.1)
Malta	85 to 89	6.2 (2.2–13.3)	6.2 (2.1–13.3)	6.2 (2.2–13.0)	6.2 (2.3–13.9)	6.2 (2.4–13.7)
Malta	90 to 94	5.6 (2.0–12.1)	5.6 (1.9–12.1)	5.6 (2.0–11.8)	5.6 (2.1–12.6)	5.6 (2.1–12.5)
Malta	95 plus	5.6 (2.0–12.0)	5.6 (1.9–12.0)	5.5 (2.0–11.7)	5.6 (2.1–12.5)	5.6 (2.1–12.4)
Malta	Age-standardized	13.8 (5.4–27.4)	13.8 (5.2–27.4)	13.7 (5.3–26.8)	13.8 (5.6–28.4)	13.8 (5.6–28.1)
Malta	All age	13.9 (5.4–27.7)	13.9 (5.2–27.6)	13.7 (5.3–26.8)	13.7 (5.5–28.1)	13.7 (5.6–27.9)
Monaco	20 to 24	12.8 (4.9–25.8)	12.8 (4.8–25.8)	12.8 (4.9–25.3)	12.8 (5.1–26.8)	12.8 (5.2–26.5)
Monaco	25 to 29	13.2 (5.1–26.4)	13.2 (4.9–26.4)	13.1 (5.0–25.9)	13.2 (5.3–27.4)	13.2 (5.4–27.1)
Monaco	30 to 34	14.4 (5.6–28.6)	14.4 (5.4–28.6)	14.4 (5.6–28.0)	14.5 (5.9–29.6)	14.5 (5.9–29.3)
Monaco	35 to 39	14.7 (5.8–29.1)	14.7 (5.6–29.1)	14.7 (5.7–28.5)	14.8 (6.0–30.1)	14.8 (6.1–29.8)
Monaco	40 to 44	15.4 (6.1–30.2)	15.4 (5.9–30.2)	15.4 (6.0–29.6)	15.5 (6.3–31.3)	15.4 (6.4–31.0)
Monaco	45 to 49	15.2 (6.0–29.8)	15.2 (5.7–29.8)	15.1 (5.9–29.2)	15.2 (6.2–30.9)	15.2 (6.3–30.6)
Monaco	50 to 54	15.0 (5.9–29.5)	15.0 (5.7–29.5)	14.9 (5.8–28.9)	15.0 (6.1–30.6)	15.0 (6.2–30.2)
Monaco	55 to 59	14.7 (5.7–29.0)	14.7 (5.5–29.0)	14.6 (5.7–28.4)	14.7 (6.0–30.0)	14.7 (6.0–29.7)
Monaco	60 to 64	14.3 (5.6–28.4)	14.3 (5.4–28.4)	14.3 (5.5–27.8)	14.4 (5.8–29.4)	14.4 (5.9–29.0)

Supplementary Table S11: Prevalence of male SVAC by age and location for 1990, 2000, 2010, 2020, and 2023						
**Location**	**Age Range**	**1990**	**2000**	**2010**	**2020**	**2023**
Monaco	65 to 69	13.4 (5.2–26.9)	13.4 (5.0–26.8)	13.4 (5.1–26.3)	13.5 (5.4–27.8)	13.4 (5.5–27.5)
Monaco	70 to 74	10.8 (4.1–22.1)	10.8 (3.9–22.1)	10.7 (4.0–21.6)	10.8 (4.2–22.9)	10.8 (4.3–22.7)
Monaco	75 to 79	9.7 (3.6–20.1)	9.7 (3.5–20.1)	9.7 (3.6–19.6)	9.7 (3.8–20.9)	9.7 (3.8–20.6)
Monaco	80 to 84	6.9 (2.5–14.6)	6.9 (2.4–14.6)	6.8 (2.5–14.3)	6.9 (2.6–15.3)	6.9 (2.6–15.1)
Monaco	85 to 89	6.2 (2.2–13.3)	6.2 (2.1–13.3)	6.2 (2.2–13.0)	6.2 (2.3–13.9)	6.2 (2.4–13.7)
Monaco	90 to 94	5.6 (2.0–12.1)	5.6 (1.9–12.1)	5.6 (2.0–11.8)	5.6 (2.1–12.6)	5.6 (2.1–12.5)
Monaco	95 plus	5.6 (2.0–12.0)	5.6 (1.9–12.0)	5.5 (2.0–11.7)	5.6 (2.1–12.5)	5.6 (2.1–12.4)
Monaco	Age-standardized	13.8 (5.4–27.4)	13.8 (5.2–27.4)	13.7 (5.3–26.8)	13.8 (5.6–28.4)	13.8 (5.6–28.1)
Monaco	All age	13.5 (5.2–26.9)	13.4 (5.0–26.7)	13.4 (5.2–26.2)	13.3 (5.3–27.4)	13.2 (5.4–27.0)
Netherlands	20 to 24	9.7 (3.8–18.9)	9.5 (4.2–17.5)	9.2 (4.7–15.9)	9.4 (4.5–17.7)	9.6 (4.3–18.6)
Netherlands	25 to 29	10.0 (4.1–19.5)	9.9 (4.4–17.8)	9.5 (4.9–16.3)	9.7 (4.6–18.1)	9.8 (4.5–18.7)
Netherlands	30 to 34	15.9 (8.1–27.1)	15.7 (8.8–24.7)	15.2 (10.1–21.6)	14.7 (8.8–22.5)	14.7 (7.9–23.4)
Netherlands	35 to 39	14.5 (7.1–25.2)	14.3 (7.6–23.0)	13.8 (8.8–20.1)	13.5 (7.8–21.1)	13.5 (7.1–21.9)
Netherlands	40 to 44	21.2 (11.4–34.5)	20.9 (12.4–31.7)	20.3 (14.2–27.6)	19.1 (12.4–27.2)	19.0 (11.3–28.0)
Netherlands	45 to 49	18.7 (9.5–30.9)	18.4 (10.5–28.9)	17.8 (12.0–24.9)	16.9 (10.8–25.1)	16.8 (9.8–26.3)

Supplementary Table S11: Prevalence of male SVAC by age and location for 1990, 2000, 2010, 2020, and 2023						
**Location**	**Age Range**	**1990**	**2000**	**2010**	**2020**	**2023**
Netherlands	50 to 54	16.7 (8.5–28.0)	16.4 (9.1–25.9)	15.9 (10.4–22.5)	15.3 (9.3–23.5)	15.2 (8.7–24.0)
Netherlands	55 to 59	21.6 (11.8–35.1)	21.3 (12.7–32.3)	20.8 (14.7–27.9)	19.4 (12.8–27.4)	19.3 (11.7–28.2)
Netherlands	60 to 64	19.4 (10.2–31.4)	19.1 (11.0–29.7)	18.5 (12.8–25.4)	17.5 (11.1–25.6)	17.3 (10.4–26.2)
Netherlands	65 to 69	20.9 (11.0–33.5)	20.6 (12.2–31.5)	20.1 (14.3–27.0)	18.7 (12.1–26.5)	18.5 (11.1–27.5)
Netherlands	70 to 74	12.0 (4.4–24.6)	11.9 (5.0–22.9)	11.8 (6.0–20.5)	11.8 (6.4–19.9)	11.9 (6.1–20.6)
Netherlands	75 to 79	9.7 (3.2–21.3)	9.5 (3.5–19.6)	9.0 (3.8–17.3)	8.8 (3.8–17.6)	8.9 (3.7–18.6)
Netherlands	80 to 84	7.6 (2.3–17.6)	7.5 (2.4–16.8)	7.4 (2.7–15.3)	7.4 (2.7–17.0)	7.5 (2.6–17.6)
Netherlands	85 to 89	6.8 (2.0–15.9)	6.7 (2.2–15.1)	6.6 (2.4–13.9)	6.7 (2.4–15.5)	6.7 (2.3–16.0)
Netherlands	90 to 94	6.2 (1.8–15.0)	6.1 (1.9–13.9)	6.0 (2.1–12.6)	6.0 (2.2–14.1)	6.1 (2.0–14.2)
Netherlands	95 plus	6.1 (1.8–14.5)	6.0 (1.9–13.5)	5.9 (2.1–12.5)	6.0 (2.1–14.0)	6.0 (2.0–14.3)
Netherlands	Age-standardized	15.3 (7.9–25.7)	15.1 (8.6–23.9)	14.7 (9.9–20.7)	14.2 (8.5–21.8)	14.1 (8.0–22.7)
Netherlands	All age	15.5 (8.0–26.0)	15.6 (8.9–24.4)	15.4 (10.5–21.6)	14.5 (8.8–22.1)	14.3 (8.3–22.9)
Norway	20 to 24	11.5 (4.5–23.2)	10.3 (4.4–19.6)	9.3 (4.8–15.7)	9.7 (4.8–17.3)	10.0 (4.5–19.2)
Norway	25 to 29	11.9 (4.7–24.0)	10.6 (4.6–19.9)	9.5 (5.0–16.1)	10.0 (5.0–17.8)	10.3 (4.6–19.6)
Norway	30 to 34	15.0 (6.1–28.7)	15.1 (7.4–26.1)	15.1 (9.9–21.4)	15.2 (8.7–23.8)	15.2 (7.9–25.2)

Supplementary Table S11: Prevalence of male SVAC by age and location for 1990, 2000, 2010, 2020, and 2023						
**Location**	**Age Range**	**1990**	**2000**	**2010**	**2020**	**2023**
Norway	35 to 39	14.9 (6.0–28.6)	14.4 (6.9–24.9)	13.9 (8.8–20.3)	14.2 (7.9–22.4)	14.4 (7.3–24.2)
Norway	40 to 44	17.5 (7.3–32.7)	18.6 (9.4–31.5)	19.5 (13.6–26.8)	19.3 (11.7–28.6)	19.0 (10.3–30.8)
Norway	45 to 49	16.7 (6.9–31.3)	17.1 (8.5–29.4)	17.3 (11.6–24.0)	17.3 (10.1–26.7)	17.3 (9.2–28.5)
Norway	50 to 54	16.0 (6.6–30.3)	15.9 (7.8–27.3)	15.6 (10.3–22.1)	15.8 (9.2–24.7)	15.9 (8.3–26.2)
Norway	55 to 59	17.4 (7.2–32.6)	18.7 (9.5–31.4)	19.9 (14.1–27.1)	19.6 (11.8–29.0)	19.2 (10.5–30.9)
Norway	60 to 64	16.6 (6.8–31.2)	17.3 (8.7–29.6)	18.0 (12.3–24.9)	17.8 (10.6–26.7)	17.6 (9.5–29.1)
Norway	65 to 69	16.4 (6.8–31.1)	17.9 (9.0–30.4)	19.3 (13.7–26.1)	18.8 (11.5–27.9)	18.4 (10.4–29.8)
Norway	70 to 74	12.2 (4.8–24.0)	12.5 (5.8–21.9)	12.6 (7.8–18.9)	12.7 (6.9–20.5)	12.7 (6.3–21.4)
Norway	75 to 79	10.5 (4.0–21.7)	10.5 (3.8–21.6)	10.5 (4.0–21.3)	10.6 (4.1–22.6)	10.6 (4.2–22.4)
Norway	80 to 84	7.5 (2.7–15.8)	7.5 (2.6–15.8)	7.5 (2.7–15.5)	7.5 (2.9–16.6)	7.5 (2.9–16.4)
Norway	85 to 89	6.7 (2.4–14.4)	6.7 (2.3–14.3)	6.7 (2.4–14.1)	6.8 (2.6–15.1)	6.8 (2.6–14.9)
Norway	90 to 94	6.1 (2.2–13.1)	6.1 (2.1–13.0)	6.1 (2.2–12.8)	6.1 (2.3–13.7)	6.1 (2.3–13.5)
Norway	95 plus	6.0 (2.2–13.0)	6.0 (2.1–12.9)	6.0 (2.2–12.7)	6.1 (2.3–13.6)	6.1 (2.3–13.4)
Norway	Age-standardized	14.5 (6.1–27.5)	14.5 (7.0–25.3)	14.5 (9.8–20.6)	14.6 (8.7–22.6)	14.6 (7.9–23.9)
Norway	All age	14.5 (6.1–27.3)	14.7 (7.1–25.5)	15.0 (10.3–21.1)	14.9 (9.0–23.0)	14.8 (8.1–24.2)

Supplementary Table S11: Prevalence of male SVAC by age and location for 1990, 2000, 2010, 2020, and 2023						
**Location**	**Age Range**	**1990**	**2000**	**2010**	**2020**	**2023**
Portugal	20 to 24	12.8 (4.9–25.8)	12.8 (4.8–25.8)	12.8 (4.9–25.3)	12.8 (5.1–26.8)	12.8 (5.2–26.5)
Portugal	25 to 29	13.2 (5.1–26.4)	13.2 (4.9–26.4)	13.1 (5.0–25.9)	13.2 (5.3–27.4)	13.2 (5.4–27.1)
Portugal	30 to 34	14.4 (5.6–28.6)	14.4 (5.4–28.6)	14.4 (5.6–28.0)	14.5 (5.9–29.6)	14.5 (5.9–29.3)
Portugal	35 to 39	14.7 (5.8–29.1)	14.7 (5.6–29.1)	14.7 (5.7–28.5)	14.8 (6.0–30.1)	14.8 (6.1–29.8)
Portugal	40 to 44	15.4 (6.1–30.2)	15.4 (5.9–30.2)	15.4 (6.0–29.6)	15.5 (6.3–31.3)	15.4 (6.4–31.0)
Portugal	45 to 49	15.2 (6.0–29.8)	15.2 (5.7–29.8)	15.1 (5.9–29.2)	15.2 (6.2–30.9)	15.2 (6.3–30.6)
Portugal	50 to 54	15.0 (5.9–29.5)	15.0 (5.7–29.5)	14.9 (5.8–28.9)	15.0 (6.1–30.6)	15.0 (6.2–30.2)
Portugal	55 to 59	14.7 (5.7–29.0)	14.7 (5.5–29.0)	14.6 (5.7–28.4)	14.7 (6.0–30.0)	14.7 (6.0–29.7)
Portugal	60 to 64	14.3 (5.6–28.4)	14.3 (5.4–28.4)	14.3 (5.5–27.8)	14.4 (5.8–29.4)	14.4 (5.9–29.0)
Portugal	65 to 69	13.4 (5.2–26.9)	13.4 (5.0–26.8)	13.4 (5.1–26.3)	13.5 (5.4–27.8)	13.4 (5.5–27.5)
Portugal	70 to 74	10.8 (4.1–22.1)	10.8 (3.9–22.1)	10.7 (4.0–21.6)	10.8 (4.2–22.9)	10.8 (4.3–22.7)
Portugal	75 to 79	9.7 (3.6–20.1)	9.7 (3.5–20.1)	9.7 (3.6–19.6)	9.7 (3.8–20.9)	9.7 (3.8–20.6)
Portugal	80 to 84	6.9 (2.5–14.6)	6.9 (2.4–14.6)	6.8 (2.5–14.3)	6.9 (2.6–15.3)	6.9 (2.6–15.1)
Portugal	85 to 89	6.2 (2.2–13.3)	6.2 (2.1–13.3)	6.2 (2.2–13.0)	6.2 (2.3–13.9)	6.2 (2.4–13.7)
Portugal	90 to 94	5.6 (2.0–12.1)	5.6 (1.9–12.1)	5.6 (2.0–11.8)	5.6 (2.1–12.6)	5.6 (2.1–12.5)

Supplementary Table S11: Prevalence of male SVAC by age and location for 1990, 2000, 2010, 2020, and 2023						
**Location**	**Age Range**	**1990**	**2000**	**2010**	**2020**	**2023**
Portugal	95 plus	5.6 (2.0–12.0)	5.6 (1.9–12.0)	5.5 (2.0–11.7)	5.6 (2.1–12.5)	5.6 (2.1–12.4)
Portugal	Age-standardized	13.8 (5.4–27.4)	13.8 (5.2–27.4)	13.7 (5.3–26.8)	13.8 (5.6–28.4)	13.8 (5.6–28.1)
Portugal	All age	13.8 (5.4–27.4)	13.7 (5.1–27.2)	13.6 (5.2–26.6)	13.5 (5.4–27.7)	13.4 (5.5–27.3)
San Marino	20 to 24	12.8 (4.9–25.8)	12.8 (4.8–25.8)	12.8 (4.9–25.3)	12.8 (5.1–26.8)	12.8 (5.2–26.5)
San Marino	25 to 29	13.2 (5.1–26.4)	13.2 (4.9–26.4)	13.1 (5.0–25.9)	13.2 (5.3–27.4)	13.2 (5.4–27.1)
San Marino	30 to 34	14.4 (5.6–28.6)	14.4 (5.4–28.6)	14.4 (5.6–28.0)	14.5 (5.9–29.6)	14.5 (5.9–29.3)
San Marino	35 to 39	14.7 (5.8–29.1)	14.7 (5.6–29.1)	14.7 (5.7–28.5)	14.8 (6.0–30.1)	14.8 (6.1–29.8)
San Marino	40 to 44	15.4 (6.1–30.2)	15.4 (5.9–30.2)	15.4 (6.0–29.6)	15.5 (6.3–31.3)	15.4 (6.4–31.0)
San Marino	45 to 49	15.2 (6.0–29.8)	15.2 (5.7–29.8)	15.1 (5.9–29.2)	15.2 (6.2–30.9)	15.2 (6.3–30.6)
San Marino	50 to 54	15.0 (5.9–29.5)	15.0 (5.7–29.5)	14.9 (5.8–28.9)	15.0 (6.1–30.6)	15.0 (6.2–30.2)
San Marino	55 to 59	14.7 (5.7–29.0)	14.7 (5.5–29.0)	14.6 (5.7–28.4)	14.7 (6.0–30.0)	14.7 (6.0–29.7)
San Marino	60 to 64	14.3 (5.6–28.4)	14.3 (5.4–28.4)	14.3 (5.5–27.8)	14.4 (5.8–29.4)	14.4 (5.9–29.0)
San Marino	65 to 69	13.4 (5.2–26.9)	13.4 (5.0–26.8)	13.4 (5.1–26.3)	13.5 (5.4–27.8)	13.4 (5.5–27.5)
San Marino	70 to 74	10.8 (4.1–22.1)	10.8 (3.9–22.1)	10.7 (4.0–21.6)	10.8 (4.2–22.9)	10.8 (4.3–22.7)
San Marino	75 to 79	9.7 (3.6–20.1)	9.7 (3.5–20.1)	9.7 (3.6–19.6)	9.7 (3.8–20.9)	9.7 (3.8–20.6)

Supplementary Table S11: Prevalence of male SVAC by age and location for 1990, 2000, 2010, 2020, and 2023						
**Location**	**Age Range**	**1990**	**2000**	**2010**	**2020**	**2023**
San Marino	80 to 84	6.9 (2.5–14.6)	6.9 (2.4–14.6)	6.8 (2.5–14.3)	6.9 (2.6–15.3)	6.9 (2.6–15.1)
San Marino	85 to 89	6.2 (2.2–13.3)	6.2 (2.1–13.3)	6.2 (2.2–13.0)	6.2 (2.3–13.9)	6.2 (2.4–13.7)
San Marino	90 to 94	5.6 (2.0–12.1)	5.6 (1.9–12.1)	5.6 (2.0–11.8)	5.6 (2.1–12.6)	5.6 (2.1–12.5)
San Marino	95 plus	5.6 (2.0–12.0)	5.6 (1.9–12.0)	5.5 (2.0–11.7)	5.6 (2.1–12.5)	5.6 (2.1–12.4)
San Marino	Age-standardized	13.8 (5.4–27.4)	13.8 (5.2–27.4)	13.7 (5.3–26.8)	13.8 (5.6–28.4)	13.8 (5.6–28.1)
San Marino	All age	13.7 (5.3–27.3)	13.6 (5.1–27.1)	13.6 (5.3–26.7)	13.6 (5.5–27.9)	13.5 (5.5–27.4)
Spain	20 to 24	10.4 (5.4–18.2)	10.4 (5.5–17.6)	10.9 (4.9–19.8)	11.2 (4.7–21.9)	11.2 (4.6–22.2)
Spain	25 to 29	11.0 (5.8–19.2)	11.4 (6.1–18.8)	12.0 (5.7–21.4)	12.4 (5.4–23.7)	12.4 (5.4–24.1)
Spain	30 to 34	16.5 (11.0–23.6)	15.0 (9.0–22.6)	14.0 (7.1–25.3)	13.7 (6.0–26.8)	13.7 (5.9–27.0)
Spain	35 to 39	14.6 (9.5–21.7)	13.0 (7.4–20.3)	12.3 (5.9–23.0)	12.1 (5.0–24.8)	12.1 (5.0–25.1)
Spain	40 to 44	20.9 (14.5–28.7)	16.9 (10.1–25.5)	14.2 (7.0–26.7)	13.5 (5.7–27.5)	13.4 (5.6–27.6)
Spain	45 to 49	18.3 (12.4–25.7)	15.0 (8.7–23.1)	12.9 (6.2–24.6)	12.4 (5.1–25.3)	12.3 (5.0–25.4)
Spain	50 to 54	16.5 (11.1–23.5)	14.0 (8.1–21.8)	12.6 (6.0–23.8)	12.2 (5.0–25.3)	12.1 (4.9–25.4)
Spain	55 to 59	21.6 (15.2–29.4)	16.9 (10.0–25.8)	13.8 (6.5–26.0)	12.9 (5.2–27.0)	12.7 (5.1–27.1)
Spain	60 to 64	19.6 (13.7–27.0)	16.2 (9.6–24.7)	13.8 (6.6–25.9)	13.2 (5.5–27.2)	13.1 (5.3–27.2)

Supplementary Table S11: Prevalence of male SVAC by age and location for 1990, 2000, 2010, 2020, and 2023						
**Location**	**Age Range**	**1990**	**2000**	**2010**	**2020**	**2023**
Spain	65 to 69	21.1 (15.0–28.5)	16.7 (9.8–25.6)	13.6 (6.3–26.1)	12.7 (5.0–27.0)	12.6 (4.9–27.1)
Spain	70 to 74	13.6 (8.5–20.9)	12.5 (6.9–20.1)	11.5 (5.3–21.4)	11.3 (4.6–22.5)	11.3 (4.5–22.6)
Spain	75 to 79	8.5 (4.1–15.5)	8.2 (3.7–15.2)	8.3 (3.2–16.5)	8.5 (3.0–18.0)	8.5 (3.0–18.6)
Spain	80 to 84	6.9 (3.2–13.0)	6.7 (2.8–13.2)	6.7 (2.4–14.3)	6.7 (2.2–15.5)	6.8 (2.1–15.7)
Spain	85 to 89	6.4 (3.0–12.3)	6.2 (2.6–12.4)	6.2 (2.2–13.3)	6.2 (2.0–14.1)	6.2 (2.0–14.4)
Spain	90 to 94	6.1 (2.8–11.5)	5.8 (2.4–11.7)	5.7 (2.0–12.4)	5.7 (1.8–12.8)	5.8 (1.8–13.1)
Spain	95 plus	6.0 (2.8–11.5)	5.8 (2.4–11.6)	5.7 (2.0–12.3)	5.7 (1.8–13.2)	5.7 (1.8–13.3)
Spain	Age-standardized	15.6 (10.4–22.9)	13.6 (9.0–19.2)	12.5 (8.3–19.1)	12.2 (7.4–19.7)	12.2 (7.3–19.9)
Spain	All age	15.8 (10.6–23.0)	13.6 (9.0–19.1)	12.5 (8.2–19.7)	12.0 (7.2–20.3)	12.0 (7.0–20.3)
Sweden	20 to 24	10.2 (3.7–21.2)	9.6 (3.9–18.9)	9.0 (4.2–16.2)	9.2 (4.0–18.3)	9.2 (3.9–18.6)
Sweden	25 to 29	8.6 (3.5–16.9)	8.2 (3.7–15.2)	8.4 (4.1–14.9)	9.1 (4.1–17.9)	9.2 (4.0–18.5)
Sweden	30 to 34	10.8 (5.4–18.5)	11.4 (6.7–17.5)	12.6 (8.0–18.3)	13.6 (7.5–22.0)	13.8 (7.2–22.9)
Sweden	35 to 39	10.3 (5.0–18.1)	10.6 (6.0–16.7)	11.6 (6.9–17.5)	12.5 (6.7–20.9)	12.7 (6.4–21.8)
Sweden	40 to 44	13.3 (7.2–21.4)	14.7 (9.3–21.3)	16.7 (11.2–23.1)	17.8 (10.2–27.3)	18.0 (9.9–28.1)
Sweden	45 to 49	12.2 (6.3–20.0)	13.0 (8.0–19.6)	14.7 (9.6–20.7)	15.8 (8.9–24.8)	16.0 (8.6–25.6)

Supplementary Table S11: Prevalence of male SVAC by age and location for 1990, 2000, 2010, 2020, and 2023						
**Location**	**Age Range**	**1990**	**2000**	**2010**	**2020**	**2023**
Sweden	50 to 54	11.2 (5.7–18.8)	11.7 (7.0–18.0)	13.1 (8.4–18.8)	14.2 (7.8–22.6)	14.4 (7.5–23.4)
Sweden	55 to 59	13.5 (7.4–21.5)	15.0 (9.6–21.6)	17.0 (11.6–23.4)	18.0 (10.5–27.3)	18.2 (10.2–28.2)
Sweden	60 to 64	12.3 (6.5–20.1)	13.4 (8.4–19.8)	15.2 (10.2–21.1)	16.2 (9.4–25.0)	16.4 (9.0–25.7)
Sweden	65 to 69	12.8 (7.1–20.3)	14.4 (9.3–20.6)	16.4 (11.3–22.6)	17.4 (10.2–26.2)	17.5 (9.9–27.1)
Sweden	70 to 74	9.0 (4.2–16.0)	9.5 (5.3–15.3)	10.4 (6.0–16.1)	11.1 (5.6–19.1)	11.2 (5.5–19.7)
Sweden	75 to 79	7.1 (2.7–14.6)	7.2 (2.9–14.3)	7.8 (3.0–15.6)	8.4 (2.9–19.4)	8.5 (2.8–20.0)
Sweden	80 to 84	5.9 (2.0–13.3)	5.9 (2.1–12.8)	6.1 (2.2–12.7)	6.3 (2.1–14.8)	6.3 (2.0–15.0)
Sweden	85 to 89	5.3 (1.8–12.1)	5.3 (1.8–11.6)	5.4 (1.9–11.5)	5.6 (1.9–13.8)	5.7 (1.8–13.8)
Sweden	90 to 94	4.8 (1.6–10.8)	4.8 (1.7–10.5)	4.9 (1.7–10.5)	5.1 (1.7–12.2)	5.1 (1.6–12.3)
Sweden	95 plus	4.8 (1.6–10.8)	4.8 (1.6–10.4)	4.9 (1.7–10.4)	5.1 (1.6–12.0)	5.1 (1.6–12.4)
Sweden	Age-standardized	10.9 (5.3–18.9)	11.3 (6.6–17.9)	12.4 (7.7–18.6)	13.2 (7.1–21.8)	13.4 (7.0–22.5)
Sweden	All age	10.8 (5.4–18.8)	11.4 (6.7–17.9)	12.7 (8.0–18.8)	13.2 (7.1–21.8)	13.4 (7.0–22.5)
Switzerland	20 to 24	12.5 (4.8–25.1)	12.5 (4.6–25.2)	12.5 (4.8–24.8)	12.7 (4.2–27.4)	12.8 (4.0–28.0)
Switzerland	25 to 29	12.8 (5.0–25.8)	12.8 (4.8–25.8)	12.8 (4.9–25.4)	13.1 (4.3–28.2)	13.1 (4.1–28.6)
Switzerland	30 to 34	14.0 (5.5–27.7)	14.0 (5.3–27.8)	14.0 (5.4–27.4)	14.3 (4.8–30.3)	14.3 (4.5–31.1)

Supplementary Table S11: Prevalence of male SVAC by age and location for 1990, 2000, 2010, 2020, and 2023						
**Location**	**Age Range**	**1990**	**2000**	**2010**	**2020**	**2023**
Switzerland	35 to 39	14.3 (5.6–28.2)	14.3 (5.4–28.3)	14.3 (5.5–27.9)	14.6 (4.9–30.9)	14.6 (4.6–31.5)
Switzerland	40 to 44	14.9 (5.9–29.3)	14.9 (5.6–29.4)	14.9 (5.8–28.9)	15.2 (5.1–31.9)	15.2 (4.9–32.5)
Switzerland	45 to 49	14.6 (5.8–28.9)	14.6 (5.5–28.9)	14.6 (5.7–28.5)	14.9 (5.0–31.5)	14.9 (4.7–32.1)
Switzerland	50 to 54	14.4 (5.7–28.4)	14.4 (5.4–28.6)	14.4 (5.6–28.1)	14.7 (5.0–31.2)	14.8 (4.7–31.7)
Switzerland	55 to 59	14.1 (5.5–27.9)	14.1 (5.3–28.0)	14.1 (5.5–27.6)	14.4 (4.8–30.2)	14.4 (4.6–31.2)
Switzerland	60 to 64	13.8 (5.4–27.4)	13.8 (5.2–27.4)	13.8 (5.3–27.0)	14.0 (4.7–29.9)	14.1 (4.5–30.4)
Switzerland	65 to 69	12.9 (5.0–25.6)	12.9 (4.8–25.9)	12.9 (4.9–25.4)	13.1 (4.3–28.4)	13.2 (4.1–28.8)
Switzerland	70 to 74	10.3 (3.9–21.2)	10.3 (3.7–21.2)	10.3 (3.9–20.9)	10.5 (3.4–23.4)	10.6 (3.2–24.0)
Switzerland	75 to 79	9.3 (3.5–19.3)	9.3 (3.3–19.3)	9.3 (3.4–19.0)	9.5 (3.0–21.1)	9.5 (3.0–21.7)
Switzerland	80 to 84	6.6 (2.4–14.0)	6.6 (2.3–14.0)	6.6 (2.4–13.8)	6.7 (2.0–15.4)	6.8 (2.0–15.9)
Switzerland	85 to 89	5.9 (2.1–12.6)	5.9 (2.1–12.7)	5.9 (2.1–12.5)	6.1 (1.9–14.1)	6.1 (1.8–14.4)
Switzerland	90 to 94	5.4 (1.9–11.6)	5.4 (1.8–11.6)	5.3 (1.9–11.4)	5.5 (1.7–12.9)	5.5 (1.6–13.1)
Switzerland	95 plus	5.3 (1.9–11.4)	5.3 (1.8–11.5)	5.3 (1.9–11.3)	5.4 (1.6–12.7)	5.5 (1.6–13.1)
Switzerland	Age-standardized	13.3 (5.2–26.5)	13.3 (5.0–26.6)	13.3 (5.1–26.2)	13.6 (4.5–29.1)	13.6 (4.3–29.6)
Switzerland	All age	13.3 (5.2–26.5)	13.3 (5.0–26.6)	13.3 (5.1–26.0)	13.4 (4.5–28.6)	13.4 (4.2–29.0)

Supplementary Table S11: Prevalence of male SVAC by age and location for 1990, 2000, 2010, 2020, and 2023						
**Location**	**Age Range**	**1990**	**2000**	**2010**	**2020**	**2023**
UK	20 to 24	15.5 (9.9–23.9)	17.8 (11.6–25.0)	17.9 (9.3–30.1)	17.1 (7.3–31.8)	16.9 (6.8–32.7)
UK	25 to 29	14.8 (8.9–23.8)	16.8 (10.9–23.7)	17.3 (9.8–27.9)	17.0 (7.5–30.9)	16.9 (6.9–32.0)
UK	30 to 34	18.2 (11.7–27.3)	19.0 (12.3–27.5)	18.4 (11.5–28.0)	17.9 (8.4–30.7)	17.8 (7.9–32.3)
UK	35 to 39	16.0 (9.1–25.4)	16.6 (9.7–25.7)	17.2 (10.4–25.6)	17.7 (9.6–29.3)	17.8 (9.0–31.0)
UK	40 to 44	20.7 (12.7–30.7)	19.3 (11.8–28.8)	18.2 (11.0–26.8)	17.9 (10.9–28.3)	17.9 (10.0–28.8)
UK	45 to 49	17.6 (10.3–27.8)	16.3 (9.0–26.6)	16.2 (8.5–27.3)	16.9 (7.4–30.5)	17.1 (7.3–32.5)
UK	50 to 54	16.3 (9.4–25.9)	15.6 (8.4–25.4)	15.9 (8.3–26.4)	16.5 (7.7–29.5)	16.7 (7.7–31.2)
UK	55 to 59	19.8 (11.8–30.5)	17.4 (9.1–28.2)	16.5 (8.3–27.8)	16.5 (7.9–29.1)	16.5 (7.9–30.0)
UK	60 to 64	19.0 (11.4–29.8)	17.9 (9.5–29.3)	17.1 (8.1–30.5)	16.8 (6.8–31.8)	16.8 (6.7–32.7)
UK	65 to 69	14.4 (6.3–26.9)	14.1 (6.5–25.5)	14.4 (6.3–26.5)	14.9 (6.2–29.8)	15.0 (6.2–30.6)
UK	70 to 74	11.8 (5.1–22.5)	11.6 (5.3–21.4)	11.8 (5.1–22.1)	12.1 (4.9–24.9)	12.2 (4.9–25.5)
UK	75 to 79	11.0 (4.2–22.6)	11.0 (4.0–22.5)	11.0 (4.1–22.1)	11.1 (4.3–23.5)	11.1 (4.4–24.0)
UK	80 to 84	7.8 (2.9–16.5)	7.8 (2.8–16.5)	7.8 (2.9–16.2)	7.9 (3.0–17.3)	7.9 (3.0–17.6)
UK	85 to 89	7.0 (2.6–15.0)	7.0 (2.5–15.0)	7.0 (2.6–14.7)	7.1 (2.7–15.7)	7.1 (2.7–16.0)
UK	90 to 94	6.4 (2.3–13.7)	6.4 (2.2–13.6)	6.4 (2.3–13.4)	6.4 (2.4–14.3)	6.4 (2.4–14.6)

Supplementary Table S11: Prevalence of male SVAC by age and location for 1990, 2000, 2010, 2020, and 2023						
**Location**	**Age Range**	**1990**	**2000**	**2010**	**2020**	**2023**
UK	95 plus	6.3 (2.3–13.5)	6.3 (2.2–13.5)	6.3 (2.3–13.3)	6.4 (2.4–14.2)	6.4 (2.4–14.5)
UK	Age-standardized	16.5 (10.0–25.5)	16.6 (10.7–24.2)	16.5 (12.3–22.7)	16.4 (10.9–24.8)	16.5 (10.0–25.7)
UK	All age	16.4 (9.8–25.6)	16.3 (10.2–24.4)	16.1 (11.4–23.2)	15.9 (10.0–25.4)	15.9 (9.1–26.3)
Latin America and Caribbean	20 to 24	13.3 (7.1–22.6)	13.2 (6.9–22.3)	13.2 (7.2–22.1)	13.3 (7.7–22.3)	13.4 (7.9–22.4)
Latin America and Caribbean	25 to 29	13.7 (7.4–23.4)	13.6 (7.0–23.7)	13.8 (7.0–23.6)	14.1 (7.0–25.2)	14.1 (7.1–25.3)
Latin America and Caribbean	30 to 34	14.8 (8.1–25.0)	14.8 (7.7–25.6)	14.9 (7.6–25.4)	15.2 (7.5–27.2)	15.3 (7.7–27.4)
Latin America and Caribbean	35 to 39	14.3 (7.8–24.3)	14.3 (7.2–25.0)	14.4 (7.0–25.1)	14.7 (7.0–26.8)	14.9 (7.1–27.2)
Latin America and Caribbean	40 to 44	14.8 (8.0–25.1)	14.8 (7.4–25.8)	14.8 (7.2–25.9)	15.2 (7.2–27.6)	15.3 (7.3–27.8)
Latin America and Caribbean	45 to 49	14.2 (7.6–24.4)	14.3 (7.0–25.2)	14.4 (6.8–25.4)	14.7 (6.8–27.0)	14.7 (6.8–27.4)
Latin America and Caribbean	50 to 54	13.7 (7.0–24.1)	13.7 (6.3–24.9)	13.8 (6.2–25.2)	14.1 (6.2–26.8)	14.2 (6.4–27.2)
Latin America and Caribbean	55 to 59	14.2 (7.5–24.5)	14.2 (6.8–25.0)	14.2 (6.6–25.3)	14.4 (6.6–26.8)	14.5 (6.7–27.3)
Latin America and Caribbean	60 to 64	12.8 (6.5–22.6)	12.8 (5.9–23.2)	12.8 (5.7–23.5)	13.1 (5.8–24.9)	13.1 (5.9–25.3)
Latin America and Caribbean	65 to 69	12.2 (6.4–21.4)	12.3 (5.7–22.3)	12.3 (5.5–22.4)	12.5 (5.7–23.7)	12.6 (5.7–24.1)
Latin America and Caribbean	70 to 74	11.8 (6.0–20.5)	11.8 (5.5–21.4)	11.8 (5.3–21.7)	11.8 (5.2–22.9)	11.8 (5.2–23.1)
Latin America and Caribbean	75 to 79	9.2 (4.3–17.1)	9.2 (4.0–17.6)	9.3 (3.8–18.0)	9.4 (3.9–19.2)	9.4 (4.0–19.2)

Supplementary Table S11: Prevalence of male SVAC by age and location for 1990, 2000, 2010, 2020, and 2023						
**Location**	**Age Range**	**1990**	**2000**	**2010**	**2020**	**2023**
Latin America and Caribbean	80 to 84	6.5 (2.7–13.1)	6.5 (2.5–13.3)	6.5 (2.4–13.6)	6.6 (2.5–14.5)	6.6 (2.6–14.3)
Latin America and Caribbean	85 to 89	5.8 (2.4–11.9)	5.8 (2.2–12.0)	5.9 (2.1–12.3)	5.9 (2.2–13.2)	5.9 (2.3–13.1)
Latin America and Caribbean	90 to 94	5.3 (2.1–11.0)	5.3 (2.0–11.0)	5.3 (1.9–11.2)	5.4 (2.0–12.0)	5.4 (2.1–11.9)
Latin America and Caribbean	95 plus	5.4 (2.2–11.2)	5.3 (2.0–11.1)	5.3 (1.9–11.1)	5.4 (2.0–12.0)	5.4 (2.1–11.9)
Latin America and Caribbean	Age-standardized	13.5 (7.2–23.3)	13.5 (6.6–23.8)	13.5 (6.5–23.7)	13.8 (6.7–25.1)	13.9 (6.8–25.4)
Latin America and Caribbean	All age	13.7 (7.3–23.6)	13.7 (6.8–23.9)	13.7 (6.7–23.9)	13.9 (6.7–25.3)	13.9 (6.9–25.6)
Andean Latin America	20 to 24	14.1 (6.1–26.7)	13.9 (6.5–25.0)	14.0 (6.9–24.2)	14.3 (6.3–27.1)	14.3 (6.3–27.7)
Andean Latin America	25 to 29	14.0 (5.9–26.4)	13.6 (6.2–24.8)	13.7 (6.5–24.0)	14.2 (6.2–27.1)	14.3 (6.2–27.9)
Andean Latin America	30 to 34	16.7 (7.3–30.7)	17.0 (8.0–30.0)	17.0 (8.4–29.1)	16.8 (7.5–31.4)	16.7 (7.4–32.0)
Andean Latin America	35 to 39	15.8 (6.7–29.8)	15.5 (7.1–28.1)	15.6 (7.2–27.6)	15.9 (6.9–30.5)	16.0 (6.9–31.0)
Andean Latin America	40 to 44	15.5 (6.5–29.0)	14.9 (6.7–27.1)	14.9 (6.8–26.4)	15.6 (6.8–30.0)	15.8 (6.9–30.7)
Andean Latin America	45 to 49	17.5 (7.5–32.6)	17.9 (8.3–32.0)	18.0 (8.3–31.5)	17.6 (7.7–33.6)	17.5 (7.7–33.6)
Andean Latin America	50 to 54	16.1 (6.4–31.4)	16.1 (6.2–31.3)	16.1 (6.4–30.9)	16.2 (6.7–32.6)	16.2 (6.8–32.3)
Andean Latin America	55 to 59	15.6 (6.2–30.6)	15.6 (5.9–30.5)	15.6 (6.1–30.0)	15.7 (6.4–31.7)	15.7 (6.5–31.4)
Andean Latin America	60 to 64	15.1 (6.0–29.8)	15.1 (5.7–29.7)	15.1 (5.9–29.2)	15.2 (6.2–30.9)	15.2 (6.3–30.6)

Supplementary Table S11: Prevalence of male SVAC by age and location for 1990, 2000, 2010, 2020, and 2023						
**Location**	**Age Range**	**1990**	**2000**	**2010**	**2020**	**2023**
Andean Latin America	65 to 69	14.1 (5.5–28.0)	14.1 (5.3–27.9)	14.1 (5.5–27.5)	14.2 (5.7–29.1)	14.2 (5.8–28.7)
Andean Latin America	70 to 74	11.3 (4.3–23.0)	11.3 (4.1–23.0)	11.3 (4.3–22.6)	11.4 (4.5–24.0)	11.4 (4.5–23.7)
Andean Latin America	75 to 79	10.2 (3.8–21.0)	10.1 (3.7–20.9)	10.2 (3.8–20.6)	10.2 (4.0–21.9)	10.2 (4.0–21.6)
Andean Latin America	80 to 84	7.2 (2.6–15.3)	7.2 (2.5–15.2)	7.2 (2.6–15.0)	7.2 (2.7–16.0)	7.2 (2.8–15.8)
Andean Latin America	85 to 89	6.5 (2.3–13.9)	6.5 (2.3–13.8)	6.5 (2.3–13.6)	6.5 (2.5–14.5)	6.5 (2.5–14.3)
Andean Latin America	90 to 94	5.9 (2.1–12.6)	5.8 (2.0–12.6)	5.8 (2.1–12.3)	5.9 (2.2–13.2)	5.9 (2.2–13.1)
Andean Latin America	95 plus	5.8 (2.1–12.6)	5.8 (2.0–12.5)	5.8 (2.1–12.3)	5.9 (2.2–13.2)	5.9 (2.2–13.0)
Andean Latin America	Age-standardized	14.9 (6.2–28.4)	14.8 (6.6–27.5)	14.8 (6.6–26.8)	15.0 (6.5–29.2)	15.0 (6.4–29.4)
Andean Latin America	All age	15.0 (6.3–28.3)	14.9 (6.7–27.4)	14.9 (6.7–26.8)	15.1 (6.5–29.4)	15.1 (6.5–29.6)
Bolivia	20 to 24	14.2 (5.5–28.1)	14.1 (5.3–28.1)	14.2 (5.5–27.7)	14.3 (5.8–29.3)	14.3 (5.8–29.0)
Bolivia	25 to 29	14.5 (5.7–28.8)	14.5 (5.5–28.7)	14.5 (5.7–28.3)	14.6 (5.9–29.9)	14.6 (6.0–29.6)
Bolivia	30 to 34	15.8 (6.3–30.9)	15.8 (6.0–30.8)	15.8 (6.2–30.4)	15.9 (6.5–32.1)	15.9 (6.6–31.8)
Bolivia	35 to 39	15.9 (6.3–31.1)	15.9 (6.1–31.1)	16.0 (6.3–30.6)	16.1 (6.6–32.3)	16.1 (6.7–32.0)
Bolivia	40 to 44	16.5 (6.6–32.0)	16.5 (6.3–32.0)	16.5 (6.5–31.5)	16.6 (6.9–33.2)	16.6 (6.9–32.9)
Bolivia	45 to 49	16.3 (6.5–31.7)	16.3 (6.2–31.6)	16.3 (6.4–31.2)	16.4 (6.8–32.9)	16.4 (6.8–32.6)

Supplementary Table S11: Prevalence of male SVAC by age and location for 1990, 2000, 2010, 2020, and 2023						
**Location**	**Age Range**	**1990**	**2000**	**2010**	**2020**	**2023**
Bolivia	50 to 54	15.9 (6.3–31.1)	15.9 (6.1–31.0)	15.9 (6.3–30.6)	16.0 (6.6–32.3)	16.0 (6.7–31.9)
Bolivia	55 to 59	15.4 (6.1–30.2)	15.4 (5.8–30.2)	15.4 (6.0–29.7)	15.5 (6.3–31.4)	15.5 (6.4–31.1)
Bolivia	60 to 64	14.9 (5.9–29.4)	14.9 (5.6–29.4)	14.9 (5.8–28.9)	15.0 (6.1–30.6)	15.0 (6.2–30.2)
Bolivia	65 to 69	13.9 (5.4–27.7)	13.9 (5.2–27.6)	13.9 (5.4–27.2)	14.0 (5.6–28.8)	14.0 (5.7–28.5)
Bolivia	70 to 74	11.1 (4.2–22.7)	11.1 (4.0–22.7)	11.1 (4.2–22.3)	11.2 (4.4–23.7)	11.2 (4.5–23.4)
Bolivia	75 to 79	10.0 (3.8–20.7)	10.0 (3.6–20.7)	10.0 (3.7–20.3)	10.1 (3.9–21.6)	10.1 (4.0–21.4)
Bolivia	80 to 84	7.1 (2.6–15.1)	7.1 (2.5–15.0)	7.1 (2.6–14.8)	7.1 (2.7–15.8)	7.1 (2.7–15.6)
Bolivia	85 to 89	6.4 (2.3–13.6)	6.4 (2.2–13.6)	6.4 (2.3–13.4)	6.4 (2.4–14.3)	6.4 (2.4–14.1)
Bolivia	90 to 94	5.7 (2.1–12.4)	5.7 (2.0–12.4)	5.7 (2.1–12.1)	5.8 (2.2–13.0)	5.8 (2.2–12.8)
Bolivia	95 plus	5.7 (2.1–12.3)	5.7 (2.0–12.3)	5.7 (2.0–12.0)	5.7 (2.1–12.9)	5.7 (2.2–12.7)
Bolivia	Age-standardized	14.8 (5.8–29.1)	14.8 (5.6–29.1)	14.8 (5.8–28.7)	14.9 (6.1–30.3)	14.9 (6.2–30.0)
Bolivia	All age	15.0 (5.9–29.6)	15.0 (5.7–29.5)	15.0 (5.9–29.0)	15.1 (6.1–30.6)	15.1 (6.2–30.3)
Ecuador	20 to 24	14.2 (5.5–28.1)	14.1 (5.3–28.1)	14.2 (5.5–27.7)	14.3 (5.8–29.3)	14.3 (5.8–29.0)
Ecuador	25 to 29	14.5 (5.7–28.8)	14.5 (5.5–28.7)	14.5 (5.7–28.3)	14.6 (5.9–29.9)	14.6 (6.0–29.6)
Ecuador	30 to 34	15.8 (6.3–30.9)	15.8 (6.0–30.8)	15.8 (6.2–30.4)	15.9 (6.5–32.1)	15.9 (6.6–31.8)

Supplementary Table S11: Prevalence of male SVAC by age and location for 1990, 2000, 2010, 2020, and 2023						
**Location**	**Age Range**	**1990**	**2000**	**2010**	**2020**	**2023**
Ecuador	35 to 39	15.9 (6.3–31.1)	15.9 (6.1–31.1)	16.0 (6.3–30.6)	16.1 (6.6–32.3)	16.1 (6.7–32.0)
Ecuador	40 to 44	16.5 (6.6–32.0)	16.5 (6.3–32.0)	16.5 (6.5–31.5)	16.6 (6.9–33.2)	16.6 (6.9–32.9)
Ecuador	45 to 49	16.3 (6.5–31.7)	16.3 (6.2–31.6)	16.3 (6.4–31.2)	16.4 (6.8–32.9)	16.4 (6.8–32.6)
Ecuador	50 to 54	15.9 (6.3–31.1)	15.9 (6.1–31.0)	15.9 (6.3–30.6)	16.0 (6.6–32.3)	16.0 (6.7–31.9)
Ecuador	55 to 59	15.4 (6.1–30.2)	15.4 (5.8–30.2)	15.4 (6.0–29.7)	15.5 (6.3–31.4)	15.5 (6.4–31.1)
Ecuador	60 to 64	14.9 (5.9–29.4)	14.9 (5.6–29.4)	14.9 (5.8–28.9)	15.0 (6.1–30.6)	15.0 (6.2–30.2)
Ecuador	65 to 69	13.9 (5.4–27.7)	13.9 (5.2–27.6)	13.9 (5.4–27.2)	14.0 (5.6–28.8)	14.0 (5.7–28.5)
Ecuador	70 to 74	11.1 (4.2–22.7)	11.1 (4.0–22.7)	11.1 (4.2–22.3)	11.2 (4.4–23.7)	11.2 (4.5–23.4)
Ecuador	75 to 79	10.0 (3.8–20.7)	10.0 (3.6–20.7)	10.0 (3.7–20.3)	10.1 (3.9–21.6)	10.1 (4.0–21.4)
Ecuador	80 to 84	7.1 (2.6–15.1)	7.1 (2.5–15.0)	7.1 (2.6–14.8)	7.1 (2.7–15.8)	7.1 (2.7–15.6)
Ecuador	85 to 89	6.4 (2.3–13.6)	6.4 (2.2–13.6)	6.4 (2.3–13.4)	6.4 (2.4–14.3)	6.4 (2.4–14.1)
Ecuador	90 to 94	5.7 (2.1–12.4)	5.7 (2.0–12.4)	5.7 (2.1–12.1)	5.8 (2.2–13.0)	5.8 (2.2–12.8)
Ecuador	95 plus	5.7 (2.1–12.3)	5.7 (2.0–12.3)	5.7 (2.0–12.0)	5.7 (2.1–12.9)	5.7 (2.2–12.7)
Ecuador	Age-standardized	14.8 (5.8–29.1)	14.8 (5.6–29.1)	14.8 (5.8–28.7)	14.9 (6.1–30.3)	14.9 (6.2–30.0)
Ecuador	All age	15.0 (5.9–29.5)	15.0 (5.7–29.4)	15.0 (5.8–28.9)	15.0 (6.1–30.4)	15.0 (6.2–30.1)

Supplementary Table S11: Prevalence of male SVAC by age and location for 1990, 2000, 2010, 2020, and 2023						
**Location**	**Age Range**	**1990**	**2000**	**2010**	**2020**	**2023**
Peru	20 to 24	14.1 (6.3–26.2)	13.8 (7.4–22.5)	13.8 (7.4–22.4)	14.3 (6.4–26.2)	14.3 (5.9–26.5)
Peru	25 to 29	13.7 (6.0–25.5)	12.9 (6.7–21.5)	13.0 (6.9–21.6)	13.8 (6.2–25.6)	14.0 (5.8–26.3)
Peru	30 to 34	17.3 (7.8–31.7)	17.8 (9.5–28.9)	17.9 (9.5–29.1)	17.5 (8.1–31.6)	17.3 (7.5–31.8)
Peru	35 to 39	15.7 (6.9–28.7)	15.2 (7.6–26.0)	15.3 (7.8–25.7)	15.8 (7.1–29.9)	15.9 (7.1–30.4)
Peru	40 to 44	14.8 (6.4–27.2)	13.6 (6.8–23.4)	13.6 (7.0–23.2)	14.9 (6.7–28.2)	15.2 (6.7–29.0)
Peru	45 to 49	18.4 (8.1–33.1)	19.2 (9.6–32.4)	19.3 (9.8–32.1)	18.6 (8.4–34.2)	18.3 (8.2–34.6)
Peru	50 to 54	16.2 (6.5–31.6)	16.2 (6.2–31.6)	16.3 (6.4–31.1)	16.4 (6.7–32.8)	16.4 (6.8–32.5)
Peru	55 to 59	15.7 (6.2–30.8)	15.7 (6.0–30.7)	15.8 (6.2–30.3)	15.9 (6.5–32.0)	15.9 (6.6–31.7)
Peru	60 to 64	15.3 (6.0–30.0)	15.2 (5.8–29.9)	15.3 (6.0–29.5)	15.4 (6.3–31.2)	15.4 (6.4–30.9)
Peru	65 to 69	14.2 (5.6–28.2)	14.2 (5.3–28.2)	14.2 (5.5–27.7)	14.3 (5.8–29.4)	14.3 (5.9–28.9)
Peru	70 to 74	11.4 (4.3–23.2)	11.4 (4.2–23.2)	11.4 (4.3–22.8)	11.5 (4.5–24.2)	11.5 (4.6–24.0)
Peru	75 to 79	10.3 (3.9–21.2)	10.2 (3.7–21.1)	10.3 (3.8–20.8)	10.3 (4.0–22.1)	10.3 (4.1–21.9)
Peru	80 to 84	7.3 (2.7–15.4)	7.2 (2.5–15.4)	7.3 (2.6–15.1)	7.3 (2.8–16.2)	7.3 (2.8–16.0)
Peru	85 to 89	6.5 (2.4–14.0)	6.5 (2.3–13.9)	6.5 (2.4–13.7)	6.6 (2.5–14.7)	6.6 (2.5–14.5)
Peru	90 to 94	5.9 (2.1–12.7)	5.9 (2.0–12.7)	5.9 (2.1–12.4)	5.9 (2.2–13.3)	6.0 (2.3–13.2)

Supplementary Table S11: Prevalence of male SVAC by age and location for 1990, 2000, 2010, 2020, and 2023						
**Location**	**Age Range**	**1990**	**2000**	**2010**	**2020**	**2023**
Peru	95 plus	5.9 (2.1–12.6)	5.8 (2.0–12.6)	5.9 (2.1–12.3)	5.9 (2.2–13.2)	5.9 (2.2–13.1)
Peru	Age-standardized	15.0 (6.5–27.8)	14.8 (7.0–26.2)	14.8 (7.3–25.6)	15.1 (6.7–28.6)	15.1 (6.7–29.2)
Peru	All age	15.1 (6.5–27.8)	14.9 (7.2–25.8)	14.9 (7.5–25.4)	15.2 (6.7–28.7)	15.2 (6.8–29.4)
Caribbean	20 to 24	15.0 (6.8–28.0)	16.3 (8.1–28.6)	17.2 (11.7–25.4)	17.7 (15.5–22.8)	17.8 (15.0–22.5)
Caribbean	25 to 29	15.1 (6.3–28.6)	15.7 (7.0–29.0)	17.1 (8.9–28.9)	17.8 (10.7–29.0)	17.9 (10.7–29.0)
Caribbean	30 to 34	16.2 (6.7–30.7)	16.2 (6.8–30.8)	17.5 (8.4–31.1)	18.4 (10.7–30.8)	18.5 (11.3–30.4)
Caribbean	35 to 39	16.3 (6.6–31.1)	16.1 (6.4–30.9)	16.8 (7.0–31.1)	18.1 (8.1–34.3)	18.3 (8.2–34.4)
Caribbean	40 to 44	16.4 (6.6–31.3)	16.7 (6.6–31.8)	16.8 (6.9–31.2)	18.1 (8.1–34.4)	18.4 (8.4–34.5)
Caribbean	45 to 49	15.9 (6.4–30.7)	16.1 (6.3–31.1)	16.1 (6.5–30.5)	16.9 (7.2–33.1)	17.3 (7.5–33.5)
Caribbean	50 to 54	15.5 (6.2–30.0)	15.3 (5.9–29.8)	15.8 (6.3–29.9)	16.0 (6.7–31.8)	16.2 (6.9–31.7)
Caribbean	55 to 59	15.0 (6.0–29.3)	14.8 (5.7–28.9)	15.2 (6.0–29.0)	15.3 (6.3–30.6)	15.3 (6.4–30.4)
Caribbean	60 to 64	14.5 (5.8–28.4)	14.3 (5.5–28.1)	14.3 (5.6–27.6)	14.8 (6.1–29.8)	14.8 (6.2–29.5)
Caribbean	65 to 69	13.4 (5.2–26.5)	13.4 (5.1–26.6)	13.4 (5.2–26.0)	13.7 (5.6–27.9)	13.7 (5.7–27.6)
Caribbean	70 to 74	10.5 (4.0–21.5)	10.7 (3.9–21.7)	10.6 (4.0–21.2)	10.7 (4.2–22.5)	10.8 (4.3–22.6)
Caribbean	75 to 79	9.3 (3.5–19.2)	9.4 (3.4–19.4)	9.5 (3.6–19.3)	9.5 (3.7–20.4)	9.5 (3.8–20.2)

Supplementary Table S11: Prevalence of male SVAC by age and location for 1990, 2000, 2010, 2020, and 2023						
**Location**	**Age Range**	**1990**	**2000**	**2010**	**2020**	**2023**
Caribbean	80 to 84	6.4 (2.3–13.6)	6.5 (2.3–13.8)	6.7 (2.4–13.9)	6.6 (2.5–14.7)	6.7 (2.6–14.5)
Caribbean	85 to 89	5.6 (2.0–12.1)	5.7 (2.0–12.2)	5.8 (2.1–12.2)	5.9 (2.2–13.2)	5.9 (2.2–13.0)
Caribbean	90 to 94	5.1 (1.8–11.1)	5.0 (1.7–10.9)	5.2 (1.8–10.9)	5.2 (1.9–11.8)	5.2 (2.0–11.6)
Caribbean	95 plus	5.4 (1.9–11.6)	5.0 (1.7–10.9)	5.1 (1.8–10.8)	5.1 (1.9–11.6)	5.1 (1.9–11.4)
Caribbean	Age-standardized	14.8 (6.1–28.5)	15.1 (6.2–28.7)	15.7 (7.3–28.0)	16.3 (8.8–29.2)	16.5 (9.1–29.1)
Caribbean	All age	15.0 (6.2–28.8)	15.3 (6.3–29.0)	15.8 (7.4–28.2)	16.3 (8.8–29.2)	16.4 (9.0–29.1)
Antigua and Barbuda	20 to 24	12.5 (4.8–25.2)	12.5 (4.6–25.3)	12.6 (4.8–25.0)	12.8 (5.1–26.7)	12.8 (5.2–26.3)
Antigua and Barbuda	25 to 29	12.7 (4.9–25.6)	12.8 (4.7–25.7)	12.9 (4.9–25.4)	13.0 (5.2–27.1)	13.1 (5.3–26.8)
Antigua and Barbuda	30 to 34	13.7 (5.3–27.3)	13.7 (5.1–27.3)	13.8 (5.3–27.1)	14.0 (5.6–28.8)	14.0 (5.7–28.4)
Antigua and Barbuda	35 to 39	13.7 (5.3–27.3)	13.7 (5.1–27.3)	13.8 (5.3–27.1)	14.0 (5.6–28.8)	14.0 (5.7–28.4)
Antigua and Barbuda	40 to 44	14.1 (5.5–28.0)	14.1 (5.3–28.0)	14.2 (5.5–27.7)	14.4 (5.8–29.4)	14.4 (5.9–29.1)
Antigua and Barbuda	45 to 49	13.6 (5.3–27.2)	13.6 (5.1–27.2)	13.7 (5.3–26.9)	13.9 (5.6–28.6)	13.9 (5.7–28.3)
Antigua and Barbuda	50 to 54	13.3 (5.1–26.6)	13.3 (4.9–26.6)	13.4 (5.1–26.3)	13.5 (5.4–27.9)	13.5 (5.5–27.7)
Antigua and Barbuda	55 to 59	12.8 (4.9–25.8)	12.8 (4.7–25.7)	12.9 (4.9–25.4)	13.0 (5.2–27.1)	13.0 (5.3–26.8)
Antigua and Barbuda	60 to 64	12.4 (4.7–25.0)	12.4 (4.6–25.0)	12.5 (4.8–24.7)	12.6 (5.0–26.3)	12.6 (5.1–26.0)

Supplementary Table S11: Prevalence of male SVAC by age and location for 1990, 2000, 2010, 2020, and 2023						
**Location**	**Age Range**	**1990**	**2000**	**2010**	**2020**	**2023**
Antigua and Barbuda	65 to 69	11.5 (4.4–23.5)	11.5 (4.2–23.5)	11.6 (4.4–23.2)	11.7 (4.6–24.7)	11.7 (4.7–24.4)
Antigua and Barbuda	70 to 74	9.2 (3.4–19.1)	9.2 (3.3–19.1)	9.2 (3.4–18.9)	9.3 (3.6–20.2)	9.3 (3.7–20.0)
Antigua and Barbuda	75 to 79	8.2 (3.0–17.4)	8.3 (2.9–17.3)	8.3 (3.0–17.1)	8.4 (3.2–18.3)	8.4 (3.3–18.1)
Antigua and Barbuda	80 to 84	5.8 (2.1–12.5)	5.8 (2.0–12.5)	5.8 (2.1–12.3)	5.9 (2.2–13.2)	5.9 (2.2–13.1)
Antigua and Barbuda	85 to 89	5.2 (1.9–11.3)	5.2 (1.8–11.3)	5.2 (1.9–11.1)	5.3 (2.0–12.0)	5.3 (2.0–11.8)
Antigua and Barbuda	90 to 94	4.7 (1.7–10.2)	4.7 (1.6–10.2)	4.7 (1.7–10.1)	4.8 (1.8–10.9)	4.8 (1.8–10.7)
Antigua and Barbuda	95 plus	4.7 (1.7–10.1)	4.7 (1.6–10.1)	4.7 (1.7–10.0)	4.7 (1.8–10.8)	4.7 (1.8–10.6)
Antigua and Barbuda	Age-standardized	12.6 (4.9–25.4)	12.6 (4.7–25.4)	12.7 (4.9–25.1)	12.9 (5.1–26.7)	12.9 (5.2–26.4)
Antigua and Barbuda	All age	12.7 (4.9–25.5)	12.8 (4.8–25.7)	12.9 (5.0–25.5)	13.0 (5.2–26.9)	13.0 (5.2–26.5)
The Bahamas	20 to 24	12.5 (4.8–25.2)	12.5 (4.6–25.3)	12.6 (4.8–25.0)	12.8 (5.1–26.7)	12.8 (5.2–26.3)
The Bahamas	25 to 29	12.7 (4.9–25.6)	12.8 (4.7–25.7)	12.9 (4.9–25.4)	13.0 (5.2–27.1)	13.1 (5.3–26.8)
The Bahamas	30 to 34	13.7 (5.3–27.3)	13.7 (5.1–27.3)	13.8 (5.3–27.1)	14.0 (5.6–28.8)	14.0 (5.7–28.4)
The Bahamas	35 to 39	13.7 (5.3–27.3)	13.7 (5.1–27.3)	13.8 (5.3–27.1)	14.0 (5.6–28.8)	14.0 (5.7–28.4)
The Bahamas	40 to 44	14.1 (5.5–28.0)	14.1 (5.3–28.0)	14.2 (5.5–27.7)	14.4 (5.8–29.4)	14.4 (5.9–29.1)
The Bahamas	45 to 49	13.6 (5.3–27.2)	13.6 (5.1–27.2)	13.7 (5.3–26.9)	13.9 (5.6–28.6)	13.9 (5.7–28.3)

Supplementary Table S11: Prevalence of male SVAC by age and location for 1990, 2000, 2010, 2020, and 2023						
**Location**	**Age Range**	**1990**	**2000**	**2010**	**2020**	**2023**
The Bahamas	50 to 54	13.3 (5.1–26.6)	13.3 (4.9–26.6)	13.4 (5.1–26.3)	13.5 (5.4–27.9)	13.5 (5.5–27.7)
The Bahamas	55 to 59	12.8 (4.9–25.8)	12.8 (4.7–25.7)	12.9 (4.9–25.4)	13.0 (5.2–27.1)	13.0 (5.3–26.8)
The Bahamas	60 to 64	12.4 (4.7–25.0)	12.4 (4.6–25.0)	12.5 (4.8–24.7)	12.6 (5.0–26.3)	12.6 (5.1–26.0)
The Bahamas	65 to 69	11.5 (4.4–23.5)	11.5 (4.2–23.5)	11.6 (4.4–23.2)	11.7 (4.6–24.7)	11.7 (4.7–24.4)
The Bahamas	70 to 74	9.2 (3.4–19.1)	9.2 (3.3–19.1)	9.2 (3.4–18.9)	9.3 (3.6–20.2)	9.3 (3.7–20.0)
The Bahamas	75 to 79	8.2 (3.0–17.4)	8.3 (2.9–17.3)	8.3 (3.0–17.1)	8.4 (3.2–18.3)	8.4 (3.3–18.1)
The Bahamas	80 to 84	5.8 (2.1–12.5)	5.8 (2.0–12.5)	5.8 (2.1–12.3)	5.9 (2.2–13.2)	5.9 (2.2–13.1)
The Bahamas	85 to 89	5.2 (1.9–11.3)	5.2 (1.8–11.3)	5.2 (1.9–11.1)	5.3 (2.0–12.0)	5.3 (2.0–11.8)
The Bahamas	90 to 94	4.7 (1.7–10.2)	4.7 (1.6–10.2)	4.7 (1.7–10.1)	4.8 (1.8–10.9)	4.8 (1.8–10.7)
The Bahamas	95 plus	4.7 (1.7–10.1)	4.7 (1.6–10.1)	4.7 (1.7–10.0)	4.7 (1.8–10.8)	4.7 (1.8–10.6)
The Bahamas	Age-standardized	12.6 (4.9–25.4)	12.6 (4.7–25.4)	12.7 (4.9–25.1)	12.9 (5.1–26.7)	12.9 (5.2–26.4)
The Bahamas	All age	12.9 (5.0–25.9)	13.0 (4.8–26.0)	13.1 (5.0–25.7)	13.1 (5.2–27.1)	13.1 (5.3–26.8)
Barbados	20 to 24	12.5 (4.8–25.2)	12.5 (4.6–25.3)	12.6 (4.8–25.0)	12.8 (5.1–26.7)	12.8 (5.2–26.3)
Barbados	25 to 29	12.7 (4.9–25.6)	12.8 (4.7–25.7)	12.9 (4.9–25.4)	13.0 (5.2–27.1)	13.1 (5.3–26.8)
Barbados	30 to 34	13.7 (5.3–27.3)	13.7 (5.1–27.3)	13.8 (5.3–27.1)	14.0 (5.6–28.8)	14.0 (5.7–28.4)

Supplementary Table S11: Prevalence of male SVAC by age and location for 1990, 2000, 2010, 2020, and 2023						
**Location**	**Age Range**	**1990**	**2000**	**2010**	**2020**	**2023**
Barbados	35 to 39	13.7 (5.3–27.3)	13.7 (5.1–27.3)	13.8 (5.3–27.1)	14.0 (5.6–28.8)	14.0 (5.7–28.4)
Barbados	40 to 44	14.1 (5.5–28.0)	14.1 (5.3–28.0)	14.2 (5.5–27.7)	14.4 (5.8–29.4)	14.4 (5.9–29.1)
Barbados	45 to 49	13.6 (5.3–27.2)	13.6 (5.1–27.2)	13.7 (5.3–26.9)	13.9 (5.6–28.6)	13.9 (5.7–28.3)
Barbados	50 to 54	13.3 (5.1–26.6)	13.3 (4.9–26.6)	13.4 (5.1–26.3)	13.5 (5.4–27.9)	13.5 (5.5–27.7)
Barbados	55 to 59	12.8 (4.9–25.8)	12.8 (4.7–25.7)	12.9 (4.9–25.4)	13.0 (5.2–27.1)	13.0 (5.3–26.8)
Barbados	60 to 64	12.4 (4.7–25.0)	12.4 (4.6–25.0)	12.5 (4.8–24.7)	12.6 (5.0–26.3)	12.6 (5.1–26.0)
Barbados	65 to 69	11.5 (4.4–23.5)	11.5 (4.2–23.5)	11.6 (4.4–23.2)	11.7 (4.6–24.7)	11.7 (4.7–24.4)
Barbados	70 to 74	9.2 (3.4–19.1)	9.2 (3.3–19.1)	9.2 (3.4–18.9)	9.3 (3.6–20.2)	9.3 (3.7–20.0)
Barbados	75 to 79	8.2 (3.0–17.4)	8.3 (2.9–17.3)	8.3 (3.0–17.1)	8.4 (3.2–18.3)	8.4 (3.3–18.1)
Barbados	80 to 84	5.8 (2.1–12.5)	5.8 (2.0–12.5)	5.8 (2.1–12.3)	5.9 (2.2–13.2)	5.9 (2.2–13.1)
Barbados	85 to 89	5.2 (1.9–11.3)	5.2 (1.8–11.3)	5.2 (1.9–11.1)	5.3 (2.0–12.0)	5.3 (2.0–11.8)
Barbados	90 to 94	4.7 (1.7–10.2)	4.7 (1.6–10.2)	4.7 (1.7–10.1)	4.8 (1.8–10.9)	4.8 (1.8–10.7)
Barbados	95 plus	4.7 (1.7–10.1)	4.7 (1.6–10.1)	4.7 (1.7–10.0)	4.7 (1.8–10.8)	4.7 (1.8–10.6)
Barbados	Age-standardized	12.6 (4.9–25.4)	12.6 (4.7–25.4)	12.7 (4.9–25.1)	12.9 (5.1–26.7)	12.9 (5.2–26.4)
Barbados	All age	12.5 (4.8–25.2)	12.6 (4.7–25.4)	12.7 (4.9–25.1)	12.7 (5.1–26.3)	12.6 (5.1–25.9)

Supplementary Table S11: Prevalence of male SVAC by age and location for 1990, 2000, 2010, 2020, and 2023						
**Location**	**Age Range**	**1990**	**2000**	**2010**	**2020**	**2023**
Belize	20 to 24	11.4 (7.5–16.9)	12.0 (8.2–16.8)	12.6 (7.5–19.7)	12.4 (5.6–23.3)	12.3 (5.3–24.2)
Belize	25 to 29	11.3 (7.4–17.0)	11.8 (8.0–16.4)	12.5 (7.9–18.5)	12.6 (6.1–22.7)	12.5 (5.6–23.9)
Belize	30 to 34	11.3 (6.8–16.9)	10.8 (7.3–15.1)	11.3 (7.4–16.4)	12.4 (6.4–20.6)	12.6 (6.1–22.0)
Belize	35 to 39	11.6 (6.6–18.7)	11.7 (7.9–16.4)	12.0 (7.9–16.6)	12.6 (7.6–19.2)	12.7 (7.2–20.3)
Belize	40 to 44	12.0 (6.0–20.5)	11.6 (7.5–16.9)	11.6 (7.6–16.1)	11.8 (7.1–18.3)	12.0 (6.7–19.6)
Belize	45 to 49	11.8 (5.3–21.5)	11.2 (6.6–17.0)	11.0 (7.1–15.7)	10.9 (6.5–17.1)	10.9 (6.1–18.1)
Belize	50 to 54	11.8 (5.0–22.3)	11.4 (6.3–18.0)	11.5 (7.3–16.8)	12.2 (7.3–19.2)	12.3 (6.9–20.5)
Belize	55 to 59	10.4 (4.4–19.8)	9.6 (5.0–15.7)	10.0 (6.2–14.9)	11.1 (6.3–17.6)	11.4 (5.9–18.7)
Belize	60 to 64	11.0 (4.6–21.1)	10.5 (5.3–17.7)	10.5 (6.0–16.6)	10.8 (5.8–17.7)	10.9 (5.6–18.5)
Belize	65 to 69	10.2 (4.3–19.7)	9.8 (4.8–17.1)	10.0 (5.3–16.7)	10.5 (5.3–18.1)	10.6 (5.0–19.3)
Belize	70 to 74	9.3 (3.9–18.0)	9.6 (4.6–17.2)	9.7 (4.7–17.0)	9.4 (4.0–19.0)	9.3 (3.9–19.1)
Belize	75 to 79	8.0 (3.3–15.7)	8.0 (3.7–14.6)	8.1 (3.8–14.4)	8.1 (3.4–16.4)	8.0 (3.3–16.7)
Belize	80 to 84	5.4 (1.9–11.6)	5.4 (1.9–11.6)	5.4 (1.9–11.4)	5.4 (2.0–12.2)	5.4 (2.0–12.1)
Belize	85 to 89	4.8 (1.7–10.5)	4.8 (1.7–10.5)	4.8 (1.7–10.3)	4.9 (1.8–11.0)	4.9 (1.8–10.9)
Belize	90 to 94	4.4 (1.5– 9.5)	4.3 (1.5– 9.5)	4.4 (1.5– 9.3)	4.4 (1.6–10.0)	4.4 (1.6– 9.9)

Supplementary Table S11: Prevalence of male SVAC by age and location for 1990, 2000, 2010, 2020, and 2023						
**Location**	**Age Range**	**1990**	**2000**	**2010**	**2020**	**2023**
Belize	95 plus	4.3 (1.5– 9.4)	4.3 (1.5– 9.4)	4.3 (1.5– 9.2)	4.4 (1.6– 9.9)	4.4 (1.6– 9.8)
Belize	Age-standardized	11.0 (7.2–16.6)	10.9 (7.5–15.3)	11.2 (8.4–14.2)	11.5 (8.0–16.3)	11.6 (7.6–17.0)
Belize	All age	11.2 (7.9–15.9)	11.2 (8.1–15.4)	11.5 (8.8–14.6)	11.8 (8.3–16.6)	11.9 (7.7–17.6)
Bermuda	20 to 24	12.5 (4.8–25.2)	12.5 (4.6–25.3)	12.6 (4.8–25.0)	12.8 (5.1–26.7)	12.8 (5.2–26.3)
Bermuda	25 to 29	12.7 (4.9–25.6)	12.8 (4.7–25.7)	12.9 (4.9–25.4)	13.0 (5.2–27.1)	13.1 (5.3–26.8)
Bermuda	30 to 34	13.7 (5.3–27.3)	13.7 (5.1–27.3)	13.8 (5.3–27.1)	14.0 (5.6–28.8)	14.0 (5.7–28.4)
Bermuda	35 to 39	13.7 (5.3–27.3)	13.7 (5.1–27.3)	13.8 (5.3–27.1)	14.0 (5.6–28.8)	14.0 (5.7–28.4)
Bermuda	40 to 44	14.1 (5.5–28.0)	14.1 (5.3–28.0)	14.2 (5.5–27.7)	14.4 (5.8–29.4)	14.4 (5.9–29.1)
Bermuda	45 to 49	13.6 (5.3–27.2)	13.6 (5.1–27.2)	13.7 (5.3–26.9)	13.9 (5.6–28.6)	13.9 (5.7–28.3)
Bermuda	50 to 54	13.3 (5.1–26.6)	13.3 (4.9–26.6)	13.4 (5.1–26.3)	13.5 (5.4–27.9)	13.5 (5.5–27.7)
Bermuda	55 to 59	12.8 (4.9–25.8)	12.8 (4.7–25.7)	12.9 (4.9–25.4)	13.0 (5.2–27.1)	13.0 (5.3–26.8)
Bermuda	60 to 64	12.4 (4.7–25.0)	12.4 (4.6–25.0)	12.5 (4.8–24.7)	12.6 (5.0–26.3)	12.6 (5.1–26.0)
Bermuda	65 to 69	11.5 (4.4–23.5)	11.5 (4.2–23.5)	11.6 (4.4–23.2)	11.7 (4.6–24.7)	11.7 (4.7–24.4)
Bermuda	70 to 74	9.2 (3.4–19.1)	9.2 (3.3–19.1)	9.2 (3.4–18.9)	9.3 (3.6–20.2)	9.3 (3.7–20.0)
Bermuda	75 to 79	8.2 (3.0–17.4)	8.3 (2.9–17.3)	8.3 (3.0–17.1)	8.4 (3.2–18.3)	8.4 (3.3–18.1)

Supplementary Table S11: Prevalence of male SVAC by age and location for 1990, 2000, 2010, 2020, and 2023						
**Location**	**Age Range**	**1990**	**2000**	**2010**	**2020**	**2023**
Bermuda	80 to 84	5.8 (2.1–12.5)	5.8 (2.0–12.5)	5.8 (2.1–12.3)	5.9 (2.2–13.2)	5.9 (2.2–13.1)
Bermuda	85 to 89	5.2 (1.9–11.3)	5.2 (1.8–11.3)	5.2 (1.9–11.1)	5.3 (2.0–12.0)	5.3 (2.0–11.8)
Bermuda	90 to 94	4.7 (1.7–10.2)	4.7 (1.6–10.2)	4.7 (1.7–10.1)	4.8 (1.8–10.9)	4.8 (1.8–10.7)
Bermuda	95 plus	4.7 (1.7–10.1)	4.7 (1.6–10.1)	4.7 (1.7–10.0)	4.7 (1.8–10.8)	4.7 (1.8–10.6)
Bermuda	Age-standardized	12.6 (4.9–25.4)	12.6 (4.7–25.4)	12.7 (4.9–25.1)	12.9 (5.1–26.7)	12.9 (5.2–26.4)
Bermuda	All age	12.9 (5.0–25.8)	12.8 (4.8–25.7)	12.7 (4.9–25.1)	12.6 (5.0–26.1)	12.4 (5.0–25.6)
Cuba	20 to 24	12.5 (4.8–25.2)	12.5 (4.6–25.3)	12.6 (4.8–25.0)	12.8 (5.1–26.7)	12.8 (5.2–26.3)
Cuba	25 to 29	12.7 (4.9–25.6)	12.8 (4.7–25.7)	12.9 (4.9–25.4)	13.0 (5.2–27.1)	13.1 (5.3–26.8)
Cuba	30 to 34	13.7 (5.3–27.3)	13.7 (5.1–27.3)	13.8 (5.3–27.1)	14.0 (5.6–28.8)	14.0 (5.7–28.4)
Cuba	35 to 39	13.7 (5.3–27.3)	13.7 (5.1–27.3)	13.8 (5.3–27.1)	14.0 (5.6–28.8)	14.0 (5.7–28.4)
Cuba	40 to 44	14.1 (5.5–28.0)	14.1 (5.3–28.0)	14.2 (5.5–27.7)	14.4 (5.8–29.4)	14.4 (5.9–29.1)
Cuba	45 to 49	13.6 (5.3–27.2)	13.6 (5.1–27.2)	13.7 (5.3–26.9)	13.9 (5.6–28.6)	13.9 (5.7–28.3)
Cuba	50 to 54	13.3 (5.1–26.6)	13.3 (4.9–26.6)	13.4 (5.1–26.3)	13.5 (5.4–27.9)	13.5 (5.5–27.7)
Cuba	55 to 59	12.8 (4.9–25.8)	12.8 (4.7–25.7)	12.9 (4.9–25.4)	13.0 (5.2–27.1)	13.0 (5.3–26.8)
Cuba	60 to 64	12.4 (4.7–25.0)	12.4 (4.6–25.0)	12.5 (4.8–24.7)	12.6 (5.0–26.3)	12.6 (5.1–26.0)

Supplementary Table S11: Prevalence of male SVAC by age and location for 1990, 2000, 2010, 2020, and 2023						
**Location**	**Age Range**	**1990**	**2000**	**2010**	**2020**	**2023**
Cuba	65 to 69	11.5 (4.4–23.5)	11.5 (4.2–23.5)	11.6 (4.4–23.2)	11.7 (4.6–24.7)	11.7 (4.7–24.4)
Cuba	70 to 74	9.2 (3.4–19.1)	9.2 (3.3–19.1)	9.2 (3.4–18.9)	9.3 (3.6–20.2)	9.3 (3.7–20.0)
Cuba	75 to 79	8.2 (3.0–17.4)	8.3 (2.9–17.3)	8.3 (3.0–17.1)	8.4 (3.2–18.3)	8.4 (3.3–18.1)
Cuba	80 to 84	5.8 (2.1–12.5)	5.8 (2.0–12.5)	5.8 (2.1–12.3)	5.9 (2.2–13.2)	5.9 (2.2–13.1)
Cuba	85 to 89	5.2 (1.9–11.3)	5.2 (1.8–11.3)	5.2 (1.9–11.1)	5.3 (2.0–12.0)	5.3 (2.0–11.8)
Cuba	90 to 94	4.7 (1.7–10.2)	4.7 (1.6–10.2)	4.7 (1.7–10.1)	4.8 (1.8–10.9)	4.8 (1.8–10.7)
Cuba	95 plus	4.7 (1.7–10.1)	4.7 (1.6–10.1)	4.7 (1.7–10.0)	4.7 (1.8–10.8)	4.7 (1.8–10.6)
Cuba	Age-standardized	12.6 (4.9–25.4)	12.6 (4.7–25.4)	12.7 (4.9–25.1)	12.9 (5.1–26.7)	12.9 (5.2–26.4)
Cuba	All age	12.6 (4.9–25.4)	12.7 (4.7–25.5)	12.7 (4.9–25.1)	12.7 (5.1–26.3)	12.6 (5.1–25.9)
Dominica	20 to 24	12.5 (4.8–25.2)	12.5 (4.6–25.3)	12.6 (4.8–25.0)	12.8 (5.1–26.7)	12.8 (5.2–26.3)
Dominica	25 to 29	12.7 (4.9–25.6)	12.8 (4.7–25.7)	12.9 (4.9–25.4)	13.0 (5.2–27.1)	13.1 (5.3–26.8)
Dominica	30 to 34	13.7 (5.3–27.3)	13.7 (5.1–27.3)	13.8 (5.3–27.1)	14.0 (5.6–28.8)	14.0 (5.7–28.4)
Dominica	35 to 39	13.7 (5.3–27.3)	13.7 (5.1–27.3)	13.8 (5.3–27.1)	14.0 (5.6–28.8)	14.0 (5.7–28.4)
Dominica	40 to 44	14.1 (5.5–28.0)	14.1 (5.3–28.0)	14.2 (5.5–27.7)	14.4 (5.8–29.4)	14.4 (5.9–29.1)
Dominica	45 to 49	13.6 (5.3–27.2)	13.6 (5.1–27.2)	13.7 (5.3–26.9)	13.9 (5.6–28.6)	13.9 (5.7–28.3)

Supplementary Table S11: Prevalence of male SVAC by age and location for 1990, 2000, 2010, 2020, and 2023						
**Location**	**Age Range**	**1990**	**2000**	**2010**	**2020**	**2023**
Dominica	50 to 54	13.3 (5.1–26.6)	13.3 (4.9–26.6)	13.4 (5.1–26.3)	13.5 (5.4–27.9)	13.5 (5.5–27.7)
Dominica	55 to 59	12.8 (4.9–25.8)	12.8 (4.7–25.7)	12.9 (4.9–25.4)	13.0 (5.2–27.1)	13.0 (5.3–26.8)
Dominica	60 to 64	12.4 (4.7–25.0)	12.4 (4.6–25.0)	12.5 (4.8–24.7)	12.6 (5.0–26.3)	12.6 (5.1–26.0)
Dominica	65 to 69	11.5 (4.4–23.5)	11.5 (4.2–23.5)	11.6 (4.4–23.2)	11.7 (4.6–24.7)	11.7 (4.7–24.4)
Dominica	70 to 74	9.2 (3.4–19.1)	9.2 (3.3–19.1)	9.2 (3.4–18.9)	9.3 (3.6–20.2)	9.3 (3.7–20.0)
Dominica	75 to 79	8.2 (3.0–17.4)	8.3 (2.9–17.3)	8.3 (3.0–17.1)	8.4 (3.2–18.3)	8.4 (3.3–18.1)
Dominica	80 to 84	5.8 (2.1–12.5)	5.8 (2.0–12.5)	5.8 (2.1–12.3)	5.9 (2.2–13.2)	5.9 (2.2–13.1)
Dominica	85 to 89	5.2 (1.9–11.3)	5.2 (1.8–11.3)	5.2 (1.9–11.1)	5.3 (2.0–12.0)	5.3 (2.0–11.8)
Dominica	90 to 94	4.7 (1.7–10.2)	4.7 (1.6–10.2)	4.7 (1.7–10.1)	4.8 (1.8–10.9)	4.8 (1.8–10.7)
Dominica	95 plus	4.7 (1.7–10.1)	4.7 (1.6–10.1)	4.7 (1.7–10.0)	4.7 (1.8–10.8)	4.7 (1.8–10.6)
Dominica	Age-standardized	12.6 (4.9–25.4)	12.6 (4.7–25.4)	12.7 (4.9–25.1)	12.9 (5.1–26.7)	12.9 (5.2–26.4)
Dominica	All age	12.6 (4.8–25.3)	12.6 (4.7–25.4)	12.7 (4.9–25.1)	12.9 (5.1–26.7)	12.8 (5.2–26.3)
Dominican Republic	20 to 24	12.5 (4.8–25.2)	12.5 (4.6–25.3)	12.6 (4.8–25.0)	12.8 (5.1–26.7)	12.8 (5.2–26.3)
Dominican Republic	25 to 29	12.7 (4.9–25.6)	12.8 (4.7–25.7)	12.9 (4.9–25.4)	13.0 (5.2–27.1)	13.1 (5.3–26.8)
Dominican Republic	30 to 34	13.7 (5.3–27.3)	13.7 (5.1–27.3)	13.8 (5.3–27.1)	14.0 (5.6–28.8)	14.0 (5.7–28.4)

Supplementary Table S11: Prevalence of male SVAC by age and location for 1990, 2000, 2010, 2020, and 2023						
**Location**	**Age Range**	**1990**	**2000**	**2010**	**2020**	**2023**
Dominican Republic	35 to 39	13.7 (5.3–27.3)	13.7 (5.1–27.3)	13.8 (5.3–27.1)	14.0 (5.6–28.8)	14.0 (5.7–28.4)
Dominican Republic	40 to 44	14.1 (5.5–28.0)	14.1 (5.3–28.0)	14.2 (5.5–27.7)	14.4 (5.8–29.4)	14.4 (5.9–29.1)
Dominican Republic	45 to 49	13.6 (5.3–27.2)	13.6 (5.1–27.2)	13.7 (5.3–26.9)	13.9 (5.6–28.6)	13.9 (5.7–28.3)
Dominican Republic	50 to 54	13.3 (5.1–26.6)	13.3 (4.9–26.6)	13.4 (5.1–26.3)	13.5 (5.4–27.9)	13.5 (5.5–27.7)
Dominican Republic	55 to 59	12.8 (4.9–25.8)	12.8 (4.7–25.7)	12.9 (4.9–25.4)	13.0 (5.2–27.1)	13.0 (5.3–26.8)
Dominican Republic	60 to 64	12.4 (4.7–25.0)	12.4 (4.6–25.0)	12.5 (4.8–24.7)	12.6 (5.0–26.3)	12.6 (5.1–26.0)
Dominican Republic	65 to 69	11.5 (4.4–23.5)	11.5 (4.2–23.5)	11.6 (4.4–23.2)	11.7 (4.6–24.7)	11.7 (4.7–24.4)
Dominican Republic	70 to 74	9.2 (3.4–19.1)	9.2 (3.3–19.1)	9.2 (3.4–18.9)	9.3 (3.6–20.2)	9.3 (3.7–20.0)
Dominican Republic	75 to 79	8.2 (3.0–17.4)	8.3 (2.9–17.3)	8.3 (3.0–17.1)	8.4 (3.2–18.3)	8.4 (3.3–18.1)
Dominican Republic	80 to 84	5.8 (2.1–12.5)	5.8 (2.0–12.5)	5.8 (2.1–12.3)	5.9 (2.2–13.2)	5.9 (2.2–13.1)
Dominican Republic	85 to 89	5.2 (1.9–11.3)	5.2 (1.8–11.3)	5.2 (1.9–11.1)	5.3 (2.0–12.0)	5.3 (2.0–11.8)
Dominican Republic	90 to 94	4.7 (1.7–10.2)	4.7 (1.6–10.2)	4.7 (1.7–10.1)	4.8 (1.8–10.9)	4.8 (1.8–10.7)
Dominican Republic	95 plus	4.7 (1.7–10.1)	4.7 (1.6–10.1)	4.7 (1.7–10.0)	4.7 (1.8–10.8)	4.7 (1.8–10.6)
Dominican Republic	Age-standardized	12.6 (4.9–25.4)	12.6 (4.7–25.4)	12.7 (4.9–25.1)	12.9 (5.1–26.7)	12.9 (5.2–26.4)
Dominican Republic	All age	12.8 (4.9–25.8)	12.8 (4.8–25.8)	12.9 (5.0–25.5)	13.0 (5.2–27.0)	13.0 (5.3–26.7)

Supplementary Table S11: Prevalence of male SVAC by age and location for 1990, 2000, 2010, 2020, and 2023						
**Location**	**Age Range**	**1990**	**2000**	**2010**	**2020**	**2023**
Grenada	20 to 24	12.5 (4.8–25.2)	12.5 (4.6–25.3)	12.6 (4.8–25.0)	12.8 (5.1–26.7)	12.8 (5.2–26.3)
Grenada	25 to 29	12.7 (4.9–25.6)	12.8 (4.7–25.7)	12.9 (4.9–25.4)	13.0 (5.2–27.1)	13.1 (5.3–26.8)
Grenada	30 to 34	13.7 (5.3–27.3)	13.7 (5.1–27.3)	13.8 (5.3–27.1)	14.0 (5.6–28.8)	14.0 (5.7–28.4)
Grenada	35 to 39	13.7 (5.3–27.3)	13.7 (5.1–27.3)	13.8 (5.3–27.1)	14.0 (5.6–28.8)	14.0 (5.7–28.4)
Grenada	40 to 44	14.1 (5.5–28.0)	14.1 (5.3–28.0)	14.2 (5.5–27.7)	14.4 (5.8–29.4)	14.4 (5.9–29.1)
Grenada	45 to 49	13.6 (5.3–27.2)	13.6 (5.1–27.2)	13.7 (5.3–26.9)	13.9 (5.6–28.6)	13.9 (5.7–28.3)
Grenada	50 to 54	13.3 (5.1–26.6)	13.3 (4.9–26.6)	13.4 (5.1–26.3)	13.5 (5.4–27.9)	13.5 (5.5–27.7)
Grenada	55 to 59	12.8 (4.9–25.8)	12.8 (4.7–25.7)	12.9 (4.9–25.4)	13.0 (5.2–27.1)	13.0 (5.3–26.8)
Grenada	60 to 64	12.4 (4.7–25.0)	12.4 (4.6–25.0)	12.5 (4.8–24.7)	12.6 (5.0–26.3)	12.6 (5.1–26.0)
Grenada	65 to 69	11.5 (4.4–23.5)	11.5 (4.2–23.5)	11.6 (4.4–23.2)	11.7 (4.6–24.7)	11.7 (4.7–24.4)
Grenada	70 to 74	9.2 (3.4–19.1)	9.2 (3.3–19.1)	9.2 (3.4–18.9)	9.3 (3.6–20.2)	9.3 (3.7–20.0)
Grenada	75 to 79	8.2 (3.0–17.4)	8.3 (2.9–17.3)	8.3 (3.0–17.1)	8.4 (3.2–18.3)	8.4 (3.3–18.1)
Grenada	80 to 84	5.8 (2.1–12.5)	5.8 (2.0–12.5)	5.8 (2.1–12.3)	5.9 (2.2–13.2)	5.9 (2.2–13.1)
Grenada	85 to 89	5.2 (1.9–11.3)	5.2 (1.8–11.3)	5.2 (1.9–11.1)	5.3 (2.0–12.0)	5.3 (2.0–11.8)
Grenada	90 to 94	4.7 (1.7–10.2)	4.7 (1.6–10.2)	4.7 (1.7–10.1)	4.8 (1.8–10.9)	4.8 (1.8–10.7)

Supplementary Table S11: Prevalence of male SVAC by age and location for 1990, 2000, 2010, 2020, and 2023						
**Location**	**Age Range**	**1990**	**2000**	**2010**	**2020**	**2023**
Grenada	95 plus	4.7 (1.7–10.1)	4.7 (1.6–10.1)	4.7 (1.7–10.0)	4.7 (1.8–10.8)	4.7 (1.8–10.6)
Grenada	Age-standardized	12.6 (4.9–25.4)	12.6 (4.7–25.4)	12.7 (4.9–25.1)	12.9 (5.1–26.7)	12.9 (5.2–26.4)
Grenada	All age	12.7 (4.9–25.5)	12.8 (4.8–25.7)	12.9 (5.0–25.5)	13.0 (5.2–26.9)	12.9 (5.2–26.5)
Guyana	20 to 24	12.5 (4.8–25.2)	12.5 (4.6–25.3)	12.6 (4.8–25.0)	12.8 (5.1–26.7)	12.8 (5.2–26.3)
Guyana	25 to 29	12.7 (4.9–25.6)	12.8 (4.7–25.7)	12.9 (4.9–25.4)	13.0 (5.2–27.1)	13.1 (5.3–26.8)
Guyana	30 to 34	13.7 (5.3–27.3)	13.7 (5.1–27.3)	13.8 (5.3–27.1)	14.0 (5.6–28.8)	14.0 (5.7–28.4)
Guyana	35 to 39	13.7 (5.3–27.3)	13.7 (5.1–27.3)	13.8 (5.3–27.1)	14.0 (5.6–28.8)	14.0 (5.7–28.4)
Guyana	40 to 44	14.1 (5.5–28.0)	14.1 (5.3–28.0)	14.2 (5.5–27.7)	14.4 (5.8–29.4)	14.4 (5.9–29.1)
Guyana	45 to 49	13.6 (5.3–27.2)	13.6 (5.1–27.2)	13.7 (5.3–26.9)	13.9 (5.6–28.6)	13.9 (5.7–28.3)
Guyana	50 to 54	13.3 (5.1–26.6)	13.3 (4.9–26.6)	13.4 (5.1–26.3)	13.5 (5.4–27.9)	13.5 (5.5–27.7)
Guyana	55 to 59	12.8 (4.9–25.8)	12.8 (4.7–25.7)	12.9 (4.9–25.4)	13.0 (5.2–27.1)	13.0 (5.3–26.8)
Guyana	60 to 64	12.4 (4.7–25.0)	12.4 (4.6–25.0)	12.5 (4.8–24.7)	12.6 (5.0–26.3)	12.6 (5.1–26.0)
Guyana	65 to 69	11.5 (4.4–23.5)	11.5 (4.2–23.5)	11.6 (4.4–23.2)	11.7 (4.6–24.7)	11.7 (4.7–24.4)
Guyana	70 to 74	9.2 (3.4–19.1)	9.2 (3.3–19.1)	9.2 (3.4–18.9)	9.3 (3.6–20.2)	9.3 (3.7–20.0)
Guyana	75 to 79	8.2 (3.0–17.4)	8.3 (2.9–17.3)	8.3 (3.0–17.1)	8.4 (3.2–18.3)	8.4 (3.3–18.1)

Supplementary Table S11: Prevalence of male SVAC by age and location for 1990, 2000, 2010, 2020, and 2023						
**Location**	**Age Range**	**1990**	**2000**	**2010**	**2020**	**2023**
Guyana	80 to 84	5.8 (2.1–12.5)	5.8 (2.0–12.5)	5.8 (2.1–12.3)	5.9 (2.2–13.2)	5.9 (2.2–13.1)
Guyana	85 to 89	5.2 (1.9–11.3)	5.2 (1.8–11.3)	5.2 (1.9–11.1)	5.3 (2.0–12.0)	5.3 (2.0–11.8)
Guyana	90 to 94	4.7 (1.7–10.2)	4.7 (1.6–10.2)	4.7 (1.7–10.1)	4.8 (1.8–10.9)	4.8 (1.8–10.7)
Guyana	95 plus	4.7 (1.7–10.1)	4.7 (1.6–10.1)	4.7 (1.7–10.0)	4.7 (1.8–10.8)	4.7 (1.8–10.6)
Guyana	Age-standardized	12.6 (4.9–25.4)	12.6 (4.7–25.4)	12.7 (4.9–25.1)	12.9 (5.1–26.7)	12.9 (5.2–26.4)
Guyana	All age	12.9 (5.0–25.9)	13.0 (4.8–26.0)	13.1 (5.0–25.7)	13.1 (5.2–27.1)	13.1 (5.3–26.8)
Haiti	20 to 24	26.2 (11.9–45.1)	27.0 (15.2–41.0)	27.7 (21.5–34.7)	27.5 (17.3–39.0)	27.2 (15.5–41.0)
Haiti	25 to 29	26.0 (11.5–45.8)	26.7 (13.1–44.5)	27.5 (17.2–40.5)	27.8 (19.4–37.5)	27.7 (18.0–39.6)
Haiti	30 to 34	27.1 (12.1–47.3)	27.4 (12.8–46.3)	28.0 (15.0–44.1)	28.1 (20.6–38.2)	28.0 (20.4–37.3)
Haiti	35 to 39	26.7 (11.8–47.2)	26.9 (11.4–47.3)	27.2 (12.0–47.2)	27.6 (12.6–49.5)	27.6 (12.7–49.1)
Haiti	40 to 44	27.2 (12.1–47.9)	27.3 (11.7–48.0)	27.7 (12.2–47.9)	28.1 (12.9–50.1)	28.1 (13.1–49.8)
Haiti	45 to 49	26.4 (11.6–46.8)	26.5 (11.3–46.9)	26.9 (11.8–46.8)	27.3 (12.5–49.0)	27.3 (12.6–48.7)
Haiti	50 to 54	25.7 (11.2–45.9)	25.9 (10.9–46.0)	26.2 (11.4–45.9)	26.6 (12.1–48.1)	26.6 (12.2–47.8)
Haiti	55 to 59	24.9 (10.8–44.7)	25.0 (10.4–44.8)	25.4 (10.9–44.7)	25.7 (11.6–47.0)	25.8 (11.7–46.7)
Haiti	60 to 64	24.1 (10.4–43.6)	24.2 (10.0–43.7)	24.6 (10.5–43.6)	24.9 (11.1–45.9)	25.0 (11.3–45.5)

Supplementary Table S11: Prevalence of male SVAC by age and location for 1990, 2000, 2010, 2020, and 2023						
**Location**	**Age Range**	**1990**	**2000**	**2010**	**2020**	**2023**
Haiti	65 to 69	22.6 (9.6–41.5)	22.7 (9.3–41.5)	23.0 (9.7–41.4)	23.4 (10.3–43.7)	23.4 (10.4–43.4)
Haiti	70 to 74	18.5 (7.5–35.3)	18.6 (7.3–35.3)	18.9 (7.6–35.2)	19.2 (8.1–37.4)	19.2 (8.2–37.1)
Haiti	75 to 79	16.8 (6.7–32.5)	16.9 (6.5–32.6)	17.1 (6.8–32.5)	17.4 (7.2–34.6)	17.5 (7.4–34.3)
Haiti	80 to 84	12.2 (4.7–24.7)	12.2 (4.5–24.7)	12.4 (4.7–24.7)	12.7 (5.0–26.4)	12.7 (5.1–26.2)
Haiti	85 to 89	11.0 (4.2–22.6)	11.1 (4.0–22.7)	11.3 (4.2–22.6)	11.5 (4.5–24.2)	11.5 (4.6–24.0)
Haiti	90 to 94	10.0 (3.8–20.7)	10.1 (3.6–20.8)	10.2 (3.8–20.7)	10.4 (4.1–22.3)	10.5 (4.1–22.1)
Haiti	95 plus	9.9 (3.7–20.6)	10.0 (3.6–20.6)	10.2 (3.8–20.6)	10.3 (4.0–22.1)	10.4 (4.1–21.9)
Haiti	Age-standardized	25.1 (11.4–44.0)	25.3 (11.9–43.6)	25.8 (13.7–41.4)	26.1 (17.0–39.6)	26.0 (17.4–38.9)
Haiti	All age	25.7 (11.9–44.9)	26.1 (12.5–44.1)	26.7 (15.4–41.0)	26.9 (19.0–38.8)	26.8 (18.9–38.6)
Jamaica	20 to 24	18.5 (10.1–30.4)	18.8 (13.5–25.0)	18.6 (10.2–30.0)	18.0 (7.8–32.5)	17.8 (7.7–33.0)
Jamaica	25 to 29	18.2 (8.3–32.2)	18.5 (10.6–28.6)	18.8 (10.7–28.6)	18.6 (8.6–32.9)	18.5 (7.8–33.7)
Jamaica	30 to 34	19.0 (8.1–34.8)	19.1 (9.6–31.8)	19.2 (11.5–28.5)	19.5 (10.2–32.2)	19.5 (9.0–33.3)
Jamaica	35 to 39	19.1 (8.0–35.4)	19.2 (8.9–33.9)	19.3 (11.1–30.0)	19.5 (11.1–30.6)	19.5 (10.4–31.8)
Jamaica	40 to 44	19.5 (8.2–36.7)	19.6 (8.4–35.2)	19.7 (10.2–32.6)	19.6 (12.1–29.6)	19.6 (11.9–29.7)
Jamaica	45 to 49	18.9 (7.7–35.8)	18.9 (7.4–35.8)	19.1 (7.7–35.6)	19.2 (8.1–37.5)	19.2 (8.2–37.1)

Supplementary Table S11: Prevalence of male SVAC by age and location for 1990, 2000, 2010, 2020, and 2023						
**Location**	**Age Range**	**1990**	**2000**	**2010**	**2020**	**2023**
Jamaica	50 to 54	18.4 (7.5–35.2)	18.5 (7.2–35.2)	18.6 (7.5–34.9)	18.8 (7.9–36.8)	18.8 (8.0–36.4)
Jamaica	55 to 59	17.9 (7.2–34.3)	17.9 (7.0–34.2)	18.0 (7.3–34.0)	18.2 (7.6–35.8)	18.2 (7.7–35.5)
Jamaica	60 to 64	17.3 (7.0–33.4)	17.3 (6.7–33.3)	17.5 (7.0–33.1)	17.6 (7.3–34.9)	17.6 (7.4–34.6)
Jamaica	65 to 69	16.1 (6.4–31.5)	16.1 (6.2–31.5)	16.3 (6.5–31.2)	16.5 (6.8–33.0)	16.5 (6.9–32.7)
Jamaica	70 to 74	13.0 (5.0–26.1)	13.0 (4.8–26.1)	13.1 (5.0–25.9)	13.3 (5.3–27.5)	13.3 (5.4–27.2)
Jamaica	75 to 79	11.7 (4.5–23.9)	11.7 (4.3–23.8)	11.9 (4.5–23.6)	12.0 (4.7–25.1)	12.0 (4.8–24.9)
Jamaica	80 to 84	8.4 (3.1–17.6)	8.4 (3.0–17.5)	8.4 (3.1–17.4)	8.5 (3.3–18.6)	8.5 (3.3–18.4)
Jamaica	85 to 89	7.5 (2.8–16.0)	7.5 (2.7–15.9)	7.6 (2.8–15.8)	7.7 (2.9–16.9)	7.7 (3.0–16.7)
Jamaica	90 to 94	6.8 (2.5–14.5)	6.8 (2.4–14.5)	6.9 (2.5–14.4)	6.9 (2.6–15.4)	7.0 (2.7–15.2)
Jamaica	95 plus	6.8 (2.5–14.4)	6.7 (2.4–14.4)	6.8 (2.5–14.3)	6.9 (2.6–15.3)	6.9 (2.6–15.1)
Jamaica	Age-standardized	17.8 (8.2–31.7)	17.9 (9.2–30.5)	18.0 (11.1–27.8)	18.0 (10.6–29.4)	18.0 (10.0–29.6)
Jamaica	All age	17.9 (8.6–31.2)	18.0 (9.7–30.0)	18.1 (11.4–27.5)	18.0 (10.8–29.2)	18.0 (10.0–29.6)
Puerto Rico	20 to 24	12.5 (4.8–25.2)	12.5 (4.6–25.3)	12.6 (4.8–25.0)	12.8 (5.1–26.7)	12.8 (5.2–26.3)
Puerto Rico	25 to 29	12.7 (4.9–25.6)	12.8 (4.7–25.7)	12.9 (4.9–25.4)	13.0 (5.2–27.1)	13.1 (5.3–26.8)
Puerto Rico	30 to 34	13.7 (5.3–27.3)	13.7 (5.1–27.3)	13.8 (5.3–27.1)	14.0 (5.6–28.8)	14.0 (5.7–28.4)

Supplementary Table S11: Prevalence of male SVAC by age and location for 1990, 2000, 2010, 2020, and 2023						
**Location**	**Age Range**	**1990**	**2000**	**2010**	**2020**	**2023**
Puerto Rico	35 to 39	13.7 (5.3–27.3)	13.7 (5.1–27.3)	13.8 (5.3–27.1)	14.0 (5.6–28.8)	14.0 (5.7–28.4)
Puerto Rico	40 to 44	14.1 (5.5–28.0)	14.1 (5.3–28.0)	14.2 (5.5–27.7)	14.4 (5.8–29.4)	14.4 (5.9–29.1)
Puerto Rico	45 to 49	13.6 (5.3–27.2)	13.6 (5.1–27.2)	13.7 (5.3–26.9)	13.9 (5.6–28.6)	13.9 (5.7–28.3)
Puerto Rico	50 to 54	13.3 (5.1–26.6)	13.3 (4.9–26.6)	13.4 (5.1–26.3)	13.5 (5.4–27.9)	13.5 (5.5–27.7)
Puerto Rico	55 to 59	12.8 (4.9–25.8)	12.8 (4.7–25.7)	12.9 (4.9–25.4)	13.0 (5.2–27.1)	13.0 (5.3–26.8)
Puerto Rico	60 to 64	12.4 (4.7–25.0)	12.4 (4.6–25.0)	12.5 (4.8–24.7)	12.6 (5.0–26.3)	12.6 (5.1–26.0)
Puerto Rico	65 to 69	11.5 (4.4–23.5)	11.5 (4.2–23.5)	11.6 (4.4–23.2)	11.7 (4.6–24.7)	11.7 (4.7–24.4)
Puerto Rico	70 to 74	9.2 (3.4–19.1)	9.2 (3.3–19.1)	9.2 (3.4–18.9)	9.3 (3.6–20.2)	9.3 (3.7–20.0)
Puerto Rico	75 to 79	8.2 (3.0–17.4)	8.3 (2.9–17.3)	8.3 (3.0–17.1)	8.4 (3.2–18.3)	8.4 (3.3–18.1)
Puerto Rico	80 to 84	5.8 (2.1–12.5)	5.8 (2.0–12.5)	5.8 (2.1–12.3)	5.9 (2.2–13.2)	5.9 (2.2–13.1)
Puerto Rico	85 to 89	5.2 (1.9–11.3)	5.2 (1.8–11.3)	5.2 (1.9–11.1)	5.3 (2.0–12.0)	5.3 (2.0–11.8)
Puerto Rico	90 to 94	4.7 (1.7–10.2)	4.7 (1.6–10.2)	4.7 (1.7–10.1)	4.8 (1.8–10.9)	4.8 (1.8–10.7)
Puerto Rico	95 plus	4.7 (1.7–10.1)	4.7 (1.6–10.1)	4.7 (1.7–10.0)	4.7 (1.8–10.8)	4.7 (1.8–10.6)
Puerto Rico	Age-standardized	12.6 (4.9–25.4)	12.6 (4.7–25.4)	12.7 (4.9–25.1)	12.9 (5.1–26.7)	12.9 (5.2–26.4)
Puerto Rico	All age	12.6 (4.9–25.4)	12.6 (4.7–25.3)	12.5 (4.8–24.7)	12.2 (4.9–25.5)	12.1 (4.9–24.9)

Supplementary Table S11: Prevalence of male SVAC by age and location for 1990, 2000, 2010, 2020, and 2023						
**Location**	**Age Range**	**1990**	**2000**	**2010**	**2020**	**2023**
Saint Kitts and Nevis	20 to 24	12.5 (4.8–25.2)	12.5 (4.6–25.3)	12.6 (4.8–25.0)	12.8 (5.1–26.7)	12.8 (5.2–26.3)
Saint Kitts and Nevis	25 to 29	12.7 (4.9–25.6)	12.8 (4.7–25.7)	12.9 (4.9–25.4)	13.0 (5.2–27.1)	13.1 (5.3–26.8)
Saint Kitts and Nevis	30 to 34	13.7 (5.3–27.3)	13.7 (5.1–27.3)	13.8 (5.3–27.1)	14.0 (5.6–28.8)	14.0 (5.7–28.4)
Saint Kitts and Nevis	35 to 39	13.7 (5.3–27.3)	13.7 (5.1–27.3)	13.8 (5.3–27.1)	14.0 (5.6–28.8)	14.0 (5.7–28.4)
Saint Kitts and Nevis	40 to 44	14.1 (5.5–28.0)	14.1 (5.3–28.0)	14.2 (5.5–27.7)	14.4 (5.8–29.4)	14.4 (5.9–29.1)
Saint Kitts and Nevis	45 to 49	13.6 (5.3–27.2)	13.6 (5.1–27.2)	13.7 (5.3–26.9)	13.9 (5.6–28.6)	13.9 (5.7–28.3)
Saint Kitts and Nevis	50 to 54	13.3 (5.1–26.6)	13.3 (4.9–26.6)	13.4 (5.1–26.3)	13.5 (5.4–27.9)	13.5 (5.5–27.7)
Saint Kitts and Nevis	55 to 59	12.8 (4.9–25.8)	12.8 (4.7–25.7)	12.9 (4.9–25.4)	13.0 (5.2–27.1)	13.0 (5.3–26.8)
Saint Kitts and Nevis	60 to 64	12.4 (4.7–25.0)	12.4 (4.6–25.0)	12.5 (4.8–24.7)	12.6 (5.0–26.3)	12.6 (5.1–26.0)
Saint Kitts and Nevis	65 to 69	11.5 (4.4–23.5)	11.5 (4.2–23.5)	11.6 (4.4–23.2)	11.7 (4.6–24.7)	11.7 (4.7–24.4)
Saint Kitts and Nevis	70 to 74	9.2 (3.4–19.1)	9.2 (3.3–19.1)	9.2 (3.4–18.9)	9.3 (3.6–20.2)	9.3 (3.7–20.0)
Saint Kitts and Nevis	75 to 79	8.2 (3.0–17.4)	8.3 (2.9–17.3)	8.3 (3.0–17.1)	8.4 (3.2–18.3)	8.4 (3.3–18.1)
Saint Kitts and Nevis	80 to 84	5.8 (2.1–12.5)	5.8 (2.0–12.5)	5.8 (2.1–12.3)	5.9 (2.2–13.2)	5.9 (2.2–13.1)
Saint Kitts and Nevis	85 to 89	5.2 (1.9–11.3)	5.2 (1.8–11.3)	5.2 (1.9–11.1)	5.3 (2.0–12.0)	5.3 (2.0–11.8)
Saint Kitts and Nevis	90 to 94	4.7 (1.7–10.2)	4.7 (1.6–10.2)	4.7 (1.7–10.1)	4.8 (1.8–10.9)	4.8 (1.8–10.7)

Supplementary Table S11: Prevalence of male SVAC by age and location for 1990, 2000, 2010, 2020, and 2023						
**Location**	**Age Range**	**1990**	**2000**	**2010**	**2020**	**2023**
Saint Kitts and Nevis	95 plus	4.7 (1.7–10.1)	4.7 (1.6–10.1)	4.7 (1.7–10.0)	4.7 (1.8–10.8)	4.7 (1.8–10.6)
Saint Kitts and Nevis	Age-standardized	12.6 (4.9–25.4)	12.6 (4.7–25.4)	12.7 (4.9–25.1)	12.9 (5.1–26.7)	12.9 (5.2–26.4)
Saint Kitts and Nevis	All age	12.5 (4.8–25.2)	12.8 (4.8–25.8)	13.0 (5.0–25.6)	13.1 (5.3–27.2)	13.1 (5.3–26.8)
Saint Lucia	20 to 24	12.5 (4.8–25.2)	12.5 (4.6–25.3)	12.6 (4.8–25.0)	12.8 (5.1–26.7)	12.8 (5.2–26.3)
Saint Lucia	25 to 29	12.7 (4.9–25.6)	12.8 (4.7–25.7)	12.9 (4.9–25.4)	13.0 (5.2–27.1)	13.1 (5.3–26.8)
Saint Lucia	30 to 34	13.7 (5.3–27.3)	13.7 (5.1–27.3)	13.8 (5.3–27.1)	14.0 (5.6–28.8)	14.0 (5.7–28.4)
Saint Lucia	35 to 39	13.7 (5.3–27.3)	13.7 (5.1–27.3)	13.8 (5.3–27.1)	14.0 (5.6–28.8)	14.0 (5.7–28.4)
Saint Lucia	40 to 44	14.1 (5.5–28.0)	14.1 (5.3–28.0)	14.2 (5.5–27.7)	14.4 (5.8–29.4)	14.4 (5.9–29.1)
Saint Lucia	45 to 49	13.6 (5.3–27.2)	13.6 (5.1–27.2)	13.7 (5.3–26.9)	13.9 (5.6–28.6)	13.9 (5.7–28.3)
Saint Lucia	50 to 54	13.3 (5.1–26.6)	13.3 (4.9–26.6)	13.4 (5.1–26.3)	13.5 (5.4–27.9)	13.5 (5.5–27.7)
Saint Lucia	55 to 59	12.8 (4.9–25.8)	12.8 (4.7–25.7)	12.9 (4.9–25.4)	13.0 (5.2–27.1)	13.0 (5.3–26.8)
Saint Lucia	60 to 64	12.4 (4.7–25.0)	12.4 (4.6–25.0)	12.5 (4.8–24.7)	12.6 (5.0–26.3)	12.6 (5.1–26.0)
Saint Lucia	65 to 69	11.5 (4.4–23.5)	11.5 (4.2–23.5)	11.6 (4.4–23.2)	11.7 (4.6–24.7)	11.7 (4.7–24.4)
Saint Lucia	70 to 74	9.2 (3.4–19.1)	9.2 (3.3–19.1)	9.2 (3.4–18.9)	9.3 (3.6–20.2)	9.3 (3.7–20.0)
Saint Lucia	75 to 79	8.2 (3.0–17.4)	8.3 (2.9–17.3)	8.3 (3.0–17.1)	8.4 (3.2–18.3)	8.4 (3.3–18.1)

Supplementary Table S11: Prevalence of male SVAC by age and location for 1990, 2000, 2010, 2020, and 2023						
**Location**	**Age Range**	**1990**	**2000**	**2010**	**2020**	**2023**
Saint Lucia	80 to 84	5.8 (2.1–12.5)	5.8 (2.0–12.5)	5.8 (2.1–12.3)	5.9 (2.2–13.2)	5.9 (2.2–13.1)
Saint Lucia	85 to 89	5.2 (1.9–11.3)	5.2 (1.8–11.3)	5.2 (1.9–11.1)	5.3 (2.0–12.0)	5.3 (2.0–11.8)
Saint Lucia	90 to 94	4.7 (1.7–10.2)	4.7 (1.6–10.2)	4.7 (1.7–10.1)	4.8 (1.8–10.9)	4.8 (1.8–10.7)
Saint Lucia	95 plus	4.7 (1.7–10.1)	4.7 (1.6–10.1)	4.7 (1.7–10.0)	4.7 (1.8–10.8)	4.7 (1.8–10.6)
Saint Lucia	Age-standardized	12.6 (4.9–25.4)	12.6 (4.7–25.4)	12.7 (4.9–25.1)	12.9 (5.1–26.7)	12.9 (5.2–26.4)
Saint Lucia	All age	12.7 (4.9–25.6)	12.8 (4.8–25.7)	12.9 (5.0–25.5)	13.0 (5.2–27.0)	13.0 (5.3–26.6)
Saint Vincent and the Grenadines	20 to 24	12.5 (4.8–25.2)	12.5 (4.6–25.3)	12.6 (4.8–25.0)	12.8 (5.1–26.7)	12.8 (5.2–26.3)
Saint Vincent and the Grenadines	25 to 29	12.7 (4.9–25.6)	12.8 (4.7–25.7)	12.9 (4.9–25.4)	13.0 (5.2–27.1)	13.1 (5.3–26.8)
Saint Vincent and the Grenadines	30 to 34	13.7 (5.3–27.3)	13.7 (5.1–27.3)	13.8 (5.3–27.1)	14.0 (5.6–28.8)	14.0 (5.7–28.4)
Saint Vincent and the Grenadines	35 to 39	13.7 (5.3–27.3)	13.7 (5.1–27.3)	13.8 (5.3–27.1)	14.0 (5.6–28.8)	14.0 (5.7–28.4)
Saint Vincent and the Grenadines	40 to 44	14.1 (5.5–28.0)	14.1 (5.3–28.0)	14.2 (5.5–27.7)	14.4 (5.8–29.4)	14.4 (5.9–29.1)
Saint Vincent and the Grenadines	45 to 49	13.6 (5.3–27.2)	13.6 (5.1–27.2)	13.7 (5.3–26.9)	13.9 (5.6–28.6)	13.9 (5.7–28.3)
Saint Vincent and the Grenadines	50 to 54	13.3 (5.1–26.6)	13.3 (4.9–26.6)	13.4 (5.1–26.3)	13.5 (5.4–27.9)	13.5 (5.5–27.7)
Saint Vincent and the Grenadines	55 to 59	12.8 (4.9–25.8)	12.8 (4.7–25.7)	12.9 (4.9–25.4)	13.0 (5.2–27.1)	13.0 (5.3–26.8)
Saint Vincent and the Grenadines	60 to 64	12.4 (4.7–25.0)	12.4 (4.6–25.0)	12.5 (4.8–24.7)	12.6 (5.0–26.3)	12.6 (5.1–26.0)

Supplementary Table S11: Prevalence of male SVAC by age and location for 1990, 2000, 2010, 2020, and 2023						
**Location**	**Age Range**	**1990**	**2000**	**2010**	**2020**	**2023**
Saint Vincent and the Grenadines	65 to 69	11.5 (4.4–23.5)	11.5 (4.2–23.5)	11.6 (4.4–23.2)	11.7 (4.6–24.7)	11.7 (4.7–24.4)
Saint Vincent and the Grenadines	70 to 74	9.2 (3.4–19.1)	9.2 (3.3–19.1)	9.2 (3.4–18.9)	9.3 (3.6–20.2)	9.3 (3.7–20.0)
Saint Vincent and the Grenadines	75 to 79	8.2 (3.0–17.4)	8.3 (2.9–17.3)	8.3 (3.0–17.1)	8.4 (3.2–18.3)	8.4 (3.3–18.1)
Saint Vincent and the Grenadines	80 to 84	5.8 (2.1–12.5)	5.8 (2.0–12.5)	5.8 (2.1–12.3)	5.9 (2.2–13.2)	5.9 (2.2–13.1)
Saint Vincent and the Grenadines	85 to 89	5.2 (1.9–11.3)	5.2 (1.8–11.3)	5.2 (1.9–11.1)	5.3 (2.0–12.0)	5.3 (2.0–11.8)
Saint Vincent and the Grenadines	90 to 94	4.7 (1.7–10.2)	4.7 (1.6–10.2)	4.7 (1.7–10.1)	4.8 (1.8–10.9)	4.8 (1.8–10.7)
Saint Vincent and the Grenadines	95 plus	4.7 (1.7–10.1)	4.7 (1.6–10.1)	4.7 (1.7–10.0)	4.7 (1.8–10.8)	4.7 (1.8–10.6)
Saint Vincent and the Grenadines	Age-standardized	12.6 (4.9–25.4)	12.6 (4.7–25.4)	12.7 (4.9–25.1)	12.9 (5.1–26.7)	12.9 (5.2–26.4)
Saint Vincent and the Grenadines	All age	12.7 (4.9–25.6)	12.8 (4.8–25.7)	12.9 (4.9–25.4)	12.9 (5.1–26.7)	12.8 (5.2–26.3)
Suriname	20 to 24	12.5 (4.8–25.2)	12.5 (4.6–25.3)	12.6 (4.8–25.0)	12.8 (5.1–26.7)	12.8 (5.2–26.3)
Suriname	25 to 29	12.7 (4.9–25.6)	12.8 (4.7–25.7)	12.9 (4.9–25.4)	13.0 (5.2–27.1)	13.1 (5.3–26.8)
Suriname	30 to 34	13.7 (5.3–27.3)	13.7 (5.1–27.3)	13.8 (5.3–27.1)	14.0 (5.6–28.8)	14.0 (5.7–28.4)
Suriname	35 to 39	13.7 (5.3–27.3)	13.7 (5.1–27.3)	13.8 (5.3–27.1)	14.0 (5.6–28.8)	14.0 (5.7–28.4)
Suriname	40 to 44	14.1 (5.5–28.0)	14.1 (5.3–28.0)	14.2 (5.5–27.7)	14.4 (5.8–29.4)	14.4 (5.9–29.1)
Suriname	45 to 49	13.6 (5.3–27.2)	13.6 (5.1–27.2)	13.7 (5.3–26.9)	13.9 (5.6–28.6)	13.9 (5.7–28.3)

Supplementary Table S11: Prevalence of male SVAC by age and location for 1990, 2000, 2010, 2020, and 2023						
**Location**	**Age Range**	**1990**	**2000**	**2010**	**2020**	**2023**
Suriname	50 to 54	13.3 (5.1–26.6)	13.3 (4.9–26.6)	13.4 (5.1–26.3)	13.5 (5.4–27.9)	13.5 (5.5–27.7)
Suriname	55 to 59	12.8 (4.9–25.8)	12.8 (4.7–25.7)	12.9 (4.9–25.4)	13.0 (5.2–27.1)	13.0 (5.3–26.8)
Suriname	60 to 64	12.4 (4.7–25.0)	12.4 (4.6–25.0)	12.5 (4.8–24.7)	12.6 (5.0–26.3)	12.6 (5.1–26.0)
Suriname	65 to 69	11.5 (4.4–23.5)	11.5 (4.2–23.5)	11.6 (4.4–23.2)	11.7 (4.6–24.7)	11.7 (4.7–24.4)
Suriname	70 to 74	9.2 (3.4–19.1)	9.2 (3.3–19.1)	9.2 (3.4–18.9)	9.3 (3.6–20.2)	9.3 (3.7–20.0)
Suriname	75 to 79	8.2 (3.0–17.4)	8.3 (2.9–17.3)	8.3 (3.0–17.1)	8.4 (3.2–18.3)	8.4 (3.3–18.1)
Suriname	80 to 84	5.8 (2.1–12.5)	5.8 (2.0–12.5)	5.8 (2.1–12.3)	5.9 (2.2–13.2)	5.9 (2.2–13.1)
Suriname	85 to 89	5.2 (1.9–11.3)	5.2 (1.8–11.3)	5.2 (1.9–11.1)	5.3 (2.0–12.0)	5.3 (2.0–11.8)
Suriname	90 to 94	4.7 (1.7–10.2)	4.7 (1.6–10.2)	4.7 (1.7–10.1)	4.8 (1.8–10.9)	4.8 (1.8–10.7)
Suriname	95 plus	4.7 (1.7–10.1)	4.7 (1.6–10.1)	4.7 (1.7–10.0)	4.7 (1.8–10.8)	4.7 (1.8–10.6)
Suriname	Age-standardized	12.6 (4.9–25.4)	12.6 (4.7–25.4)	12.7 (4.9–25.1)	12.9 (5.1–26.7)	12.9 (5.2–26.4)
Suriname	All age	12.8 (4.9–25.6)	12.9 (4.8–25.8)	13.0 (5.0–25.6)	13.0 (5.2–27.0)	13.0 (5.3–26.7)
Trinidad and Tobago	20 to 24	12.5 (4.8–25.2)	12.5 (4.6–25.3)	12.6 (4.8–25.0)	12.8 (5.1–26.7)	12.8 (5.2–26.3)
Trinidad and Tobago	25 to 29	12.7 (4.9–25.6)	12.8 (4.7–25.7)	12.9 (4.9–25.4)	13.0 (5.2–27.1)	13.1 (5.3–26.8)
Trinidad and Tobago	30 to 34	13.7 (5.3–27.3)	13.7 (5.1–27.3)	13.8 (5.3–27.1)	14.0 (5.6–28.8)	14.0 (5.7–28.4)

Supplementary Table S11: Prevalence of male SVAC by age and location for 1990, 2000, 2010, 2020, and 2023						
**Location**	**Age Range**	**1990**	**2000**	**2010**	**2020**	**2023**
Trinidad and Tobago	35 to 39	13.7 (5.3–27.3)	13.7 (5.1–27.3)	13.8 (5.3–27.1)	14.0 (5.6–28.8)	14.0 (5.7–28.4)
Trinidad and Tobago	40 to 44	14.1 (5.5–28.0)	14.1 (5.3–28.0)	14.2 (5.5–27.7)	14.4 (5.8–29.4)	14.4 (5.9–29.1)
Trinidad and Tobago	45 to 49	13.6 (5.3–27.2)	13.6 (5.1–27.2)	13.7 (5.3–26.9)	13.9 (5.6–28.6)	13.9 (5.7–28.3)
Trinidad and Tobago	50 to 54	13.3 (5.1–26.6)	13.3 (4.9–26.6)	13.4 (5.1–26.3)	13.5 (5.4–27.9)	13.5 (5.5–27.7)
Trinidad and Tobago	55 to 59	12.8 (4.9–25.8)	12.8 (4.7–25.7)	12.9 (4.9–25.4)	13.0 (5.2–27.1)	13.0 (5.3–26.8)
Trinidad and Tobago	60 to 64	12.4 (4.7–25.0)	12.4 (4.6–25.0)	12.5 (4.8–24.7)	12.6 (5.0–26.3)	12.6 (5.1–26.0)
Trinidad and Tobago	65 to 69	11.5 (4.4–23.5)	11.5 (4.2–23.5)	11.6 (4.4–23.2)	11.7 (4.6–24.7)	11.7 (4.7–24.4)
Trinidad and Tobago	70 to 74	9.2 (3.4–19.1)	9.2 (3.3–19.1)	9.2 (3.4–18.9)	9.3 (3.6–20.2)	9.3 (3.7–20.0)
Trinidad and Tobago	75 to 79	8.2 (3.0–17.4)	8.3 (2.9–17.3)	8.3 (3.0–17.1)	8.4 (3.2–18.3)	8.4 (3.3–18.1)
Trinidad and Tobago	80 to 84	5.8 (2.1–12.5)	5.8 (2.0–12.5)	5.8 (2.1–12.3)	5.9 (2.2–13.2)	5.9 (2.2–13.1)
Trinidad and Tobago	85 to 89	5.2 (1.9–11.3)	5.2 (1.8–11.3)	5.2 (1.9–11.1)	5.3 (2.0–12.0)	5.3 (2.0–11.8)
Trinidad and Tobago	90 to 94	4.7 (1.7–10.2)	4.7 (1.6–10.2)	4.7 (1.7–10.1)	4.8 (1.8–10.9)	4.8 (1.8–10.7)
Trinidad and Tobago	95 plus	4.7 (1.7–10.1)	4.7 (1.6–10.1)	4.7 (1.7–10.0)	4.7 (1.8–10.8)	4.7 (1.8–10.6)
Trinidad and Tobago	Age-standardized	12.6 (4.9–25.4)	12.6 (4.7–25.4)	12.7 (4.9–25.1)	12.9 (5.1–26.7)	12.9 (5.2–26.4)
Trinidad and Tobago	All age	12.8 (5.0–25.8)	12.9 (4.8–25.8)	12.9 (5.0–25.5)	13.0 (5.2–26.9)	12.9 (5.2–26.5)

Supplementary Table S11: Prevalence of male SVAC by age and location for 1990, 2000, 2010, 2020, and 2023						
**Location**	**Age Range**	**1990**	**2000**	**2010**	**2020**	**2023**
Virgin Islands	20 to 24	12.8 (4.9–25.7)	12.8 (4.7–25.7)	12.8 (4.9–25.3)	12.9 (5.2–26.9)	12.9 (5.2–26.6)
Virgin Islands	25 to 29	13.0 (5.0–26.2)	13.0 (4.8–26.1)	13.1 (5.0–25.8)	13.2 (5.3–27.4)	13.2 (5.4–27.1)
Virgin Islands	30 to 34	14.0 (5.5–27.9)	14.0 (5.3–27.9)	14.1 (5.5–27.6)	14.3 (5.8–29.2)	14.3 (5.8–28.8)
Virgin Islands	35 to 39	14.1 (5.5–28.0)	14.1 (5.3–28.0)	14.2 (5.5–27.7)	14.3 (5.8–29.4)	14.3 (5.9–29.0)
Virgin Islands	40 to 44	14.5 (5.7–28.7)	14.5 (5.5–28.7)	14.6 (5.7–28.4)	14.8 (6.0–30.1)	14.8 (6.1–29.8)
Virgin Islands	45 to 49	14.1 (5.5–28.0)	14.1 (5.3–28.0)	14.2 (5.5–27.7)	14.3 (5.8–29.4)	14.3 (5.9–29.1)
Virgin Islands	50 to 54	13.7 (5.3–27.4)	13.8 (5.1–27.4)	13.8 (5.3–27.1)	14.0 (5.6–28.8)	14.0 (5.7–28.5)
Virgin Islands	55 to 59	13.3 (5.1–26.6)	13.3 (4.9–26.6)	13.4 (5.1–26.3)	13.5 (5.4–27.9)	13.5 (5.5–27.6)
Virgin Islands	60 to 64	12.8 (5.0–25.8)	12.8 (4.8–25.8)	12.9 (4.9–25.5)	13.1 (5.2–27.1)	13.1 (5.3–26.8)
Virgin Islands	65 to 69	11.9 (4.6–24.2)	11.9 (4.4–24.2)	12.0 (4.6–23.9)	12.1 (4.8–25.5)	12.1 (4.9–25.1)
Virgin Islands	70 to 74	9.5 (3.6–19.8)	9.5 (3.4–19.8)	9.6 (3.6–19.5)	9.7 (3.7–20.8)	9.7 (3.8–20.6)
Virgin Islands	75 to 79	8.6 (3.2–18.0)	8.6 (3.0–17.9)	8.6 (3.2–17.7)	8.7 (3.3–18.9)	8.7 (3.4–18.7)
Virgin Islands	80 to 84	6.0 (2.2–12.9)	6.0 (2.1–12.9)	6.1 (2.2–12.7)	6.1 (2.3–13.7)	6.1 (2.3–13.5)
Virgin Islands	85 to 89	5.4 (1.9–11.7)	5.4 (1.9–11.7)	5.4 (1.9–11.5)	5.5 (2.0–12.4)	5.5 (2.1–12.2)
Virgin Islands	90 to 94	4.9 (1.7–10.6)	4.9 (1.7–10.6)	4.9 (1.7–10.4)	5.0 (1.8–11.2)	5.0 (1.9–11.1)

Supplementary Table S11: Prevalence of male SVAC by age and location for 1990, 2000, 2010, 2020, and 2023						
**Location**	**Age Range**	**1990**	**2000**	**2010**	**2020**	**2023**
Virgin Islands	95 plus	4.8 (1.7–10.5)	4.8 (1.7–10.5)	4.9 (1.7–10.3)	4.9 (1.8–11.1)	4.9 (1.8–11.0)
Virgin Islands	Age-standardized	13.0 (5.0–26.1)	13.0 (4.8–26.0)	13.1 (5.0–25.7)	13.2 (5.3–27.3)	13.2 (5.4–27.0)
Virgin Islands	All age	13.3 (5.2–26.6)	13.2 (4.9–26.4)	13.1 (5.0–25.7)	12.7 (5.1–26.5)	12.6 (5.1–25.9)
Central Latin America	20 to 24	13.6 (5.4–26.5)	13.4 (5.6–26.0)	13.1 (5.9–24.1)	12.9 (6.9–23.3)	13.0 (7.0–23.1)
Central Latin America	25 to 29	14.2 (5.6–27.9)	14.2 (5.5–27.8)	14.2 (5.7–27.2)	14.3 (6.1–28.4)	14.3 (6.4–28.1)
Central Latin America	30 to 34	15.4 (6.1–30.1)	15.4 (5.9–30.0)	15.4 (6.2–29.5)	15.5 (6.4–31.2)	15.5 (6.5–30.9)
Central Latin America	35 to 39	15.5 (6.2–30.3)	15.5 (6.0–30.3)	15.5 (6.2–29.6)	15.6 (6.5–31.4)	15.7 (6.6–31.2)
Central Latin America	40 to 44	16.0 (6.4–31.2)	16.1 (6.2–31.3)	16.1 (6.4–30.7)	16.2 (6.7–32.4)	16.2 (6.8–32.2)
Central Latin America	45 to 49	15.6 (6.2–30.4)	15.6 (6.0–30.4)	15.6 (6.2–30.0)	15.7 (6.5–31.6)	15.7 (6.5–31.3)
Central Latin America	50 to 54	15.3 (6.1–30.0)	15.3 (5.9–30.1)	15.4 (6.1–29.7)	15.5 (6.3–31.2)	15.4 (6.4–30.8)
Central Latin America	55 to 59	14.8 (5.8–29.2)	14.8 (5.6–29.2)	14.9 (5.8–28.9)	15.0 (6.1–30.5)	15.0 (6.2–30.1)
Central Latin America	60 to 64	14.4 (5.6–28.5)	14.4 (5.4–28.4)	14.4 (5.6–28.1)	14.5 (5.9–29.7)	14.5 (6.0–29.4)
Central Latin America	65 to 69	13.4 (5.2–26.7)	13.4 (5.0–26.7)	13.4 (5.2–26.3)	13.5 (5.4–27.9)	13.5 (5.5–27.7)
Central Latin America	70 to 74	10.7 (4.0–21.9)	10.7 (3.9–22.0)	10.7 (4.0–21.6)	10.8 (4.2–23.0)	10.8 (4.3–22.8)
Central Latin America	75 to 79	9.6 (3.6–19.9)	9.6 (3.5–19.9)	9.7 (3.6–19.7)	9.7 (3.8–20.9)	9.7 (3.8–20.7)

Supplementary Table S11: Prevalence of male SVAC by age and location for 1990, 2000, 2010, 2020, and 2023						
**Location**	**Age Range**	**1990**	**2000**	**2010**	**2020**	**2023**
Central Latin America	80 to 84	6.8 (2.5–14.4)	6.8 (2.4–14.5)	6.8 (2.5–14.2)	6.9 (2.6–15.2)	6.9 (2.6–15.0)
Central Latin America	85 to 89	6.0 (2.2–13.0)	6.1 (2.1–13.0)	6.1 (2.2–12.8)	6.2 (2.3–13.8)	6.2 (2.3–13.6)
Central Latin America	90 to 94	5.4 (2.0–11.7)	5.5 (1.9–11.8)	5.5 (2.0–11.7)	5.6 (2.1–12.5)	5.6 (2.1–12.4)
Central Latin America	95 plus	5.6 (2.0–12.1)	5.6 (1.9–12.1)	5.6 (2.0–11.8)	5.7 (2.1–12.8)	5.7 (2.2–12.7)
Central Latin America	Age-standardized	14.3 (5.6–28.0)	14.3 (5.5–28.0)	14.3 (5.7–27.3)	14.3 (6.1–28.7)	14.4 (6.3–28.3)
Central Latin America	All age	14.5 (5.7–28.3)	14.5 (5.6–28.3)	14.4 (5.8–27.5)	14.5 (6.2–28.8)	14.5 (6.3–28.5)
Colombia	20 to 24	14.4 (5.7–28.0)	13.3 (5.8–24.8)	11.7 (6.6–19.1)	11.0 (7.7–15.3)	11.2 (7.1–16.8)
Colombia	25 to 29	15.8 (6.3–31.0)	15.8 (6.0–30.9)	15.9 (6.3–30.5)	16.0 (6.6–32.2)	16.0 (6.6–31.9)
Colombia	30 to 34	17.1 (6.9–33.0)	17.1 (6.6–33.0)	17.1 (6.8–32.5)	17.2 (7.2–34.3)	17.2 (7.3–33.8)
Colombia	35 to 39	17.3 (6.9–33.3)	17.2 (6.7–33.2)	17.3 (6.9–32.8)	17.4 (7.2–34.6)	17.4 (7.3–34.2)
Colombia	40 to 44	17.8 (7.2–34.2)	17.8 (6.9–34.1)	17.9 (7.2–33.7)	18.0 (7.5–35.5)	18.0 (7.6–35.1)
Colombia	45 to 49	17.4 (7.0–33.5)	17.4 (6.7–33.4)	17.4 (7.0–33.0)	17.5 (7.3–34.8)	17.5 (7.4–34.4)
Colombia	50 to 54	17.0 (6.8–32.9)	17.0 (6.6–32.9)	17.1 (6.8–32.4)	17.2 (7.1–34.2)	17.2 (7.2–33.7)
Colombia	55 to 59	16.5 (6.6–32.1)	16.5 (6.3–32.0)	16.5 (6.6–31.6)	16.7 (6.9–33.3)	16.6 (7.0–33.0)
Colombia	60 to 64	16.0 (6.4–31.2)	16.0 (6.1–31.2)	16.0 (6.3–30.7)	16.1 (6.6–32.5)	16.1 (6.7–32.1)

Supplementary Table S11: Prevalence of male SVAC by age and location for 1990, 2000, 2010, 2020, and 2023						
**Location**	**Age Range**	**1990**	**2000**	**2010**	**2020**	**2023**
Colombia	65 to 69	14.9 (5.9–29.4)	14.9 (5.6–29.4)	14.9 (5.8–28.9)	15.1 (6.1–30.6)	15.0 (6.2–30.3)
Colombia	70 to 74	12.0 (4.6–24.3)	12.0 (4.4–24.2)	12.0 (4.6–23.9)	12.1 (4.8–25.4)	12.1 (4.8–25.1)
Colombia	75 to 79	10.8 (4.1–22.2)	10.8 (3.9–22.1)	10.8 (4.1–21.8)	10.9 (4.3–23.2)	10.9 (4.3–22.9)
Colombia	80 to 84	7.7 (2.8–16.2)	7.6 (2.7–16.2)	7.7 (2.8–15.9)	7.7 (2.9–17.0)	7.7 (3.0–16.8)
Colombia	85 to 89	6.9 (2.5–14.7)	6.9 (2.4–14.7)	6.9 (2.5–14.4)	7.0 (2.6–15.4)	7.0 (2.7–15.2)
Colombia	90 to 94	6.2 (2.3–13.4)	6.2 (2.2–13.3)	6.2 (2.2–13.1)	6.3 (2.4–14.0)	6.3 (2.4–13.9)
Colombia	95 plus	6.2 (2.2–13.3)	6.2 (2.1–13.2)	6.2 (2.2–13.0)	6.2 (2.3–13.9)	6.2 (2.4–13.8)
Colombia	Age-standardized	15.9 (6.4–30.6)	15.7 (6.3–30.4)	15.5 (6.4–28.9)	15.6 (7.0–29.9)	15.6 (7.2–29.7)
Colombia	All age	16.0 (6.5–31.0)	15.9 (6.5–30.7)	15.6 (6.5–29.0)	15.5 (7.1–29.8)	15.6 (7.2–29.7)
Costa Rica	20 to 24	18.0 (8.7–31.7)	17.6 (9.6–28.0)	17.8 (9.4–29.1)	18.3 (8.4–33.7)	18.4 (8.2–34.7)
Costa Rica	25 to 29	20.3 (9.5–35.6)	20.7 (10.6–34.1)	20.6 (10.3–34.2)	20.2 (9.1–37.2)	20.0 (9.0–37.8)
Costa Rica	30 to 34	20.4 (9.6–35.7)	20.3 (10.5–33.3)	20.4 (10.3–33.8)	20.6 (9.4–37.7)	20.6 (9.3–38.2)
Costa Rica	35 to 39	19.1 (9.1–33.5)	18.4 (9.7–29.8)	18.7 (9.6–30.8)	19.5 (8.9–35.9)	19.7 (8.9–36.8)
Costa Rica	40 to 44	20.9 (8.7–39.0)	20.9 (8.4–38.9)	21.0 (8.7–38.4)	21.1 (9.1–40.3)	21.1 (9.2–39.9)
Costa Rica	45 to 49	19.7 (9.3–34.6)	19.2 (10.0–31.5)	19.4 (9.8–32.1)	20.0 (9.1–36.5)	20.1 (9.1–37.5)

Supplementary Table S11: Prevalence of male SVAC by age and location for 1990, 2000, 2010, 2020, and 2023						
**Location**	**Age Range**	**1990**	**2000**	**2010**	**2020**	**2023**
Costa Rica	50 to 54	20.0 (8.2–37.6)	20.0 (7.9–37.5)	20.0 (8.2–37.0)	20.1 (8.6–38.8)	20.1 (8.7–38.5)
Costa Rica	55 to 59	19.4 (8.0–36.6)	19.4 (7.7–36.6)	19.4 (7.9–36.1)	19.5 (8.3–37.9)	19.5 (8.4–37.5)
Costa Rica	60 to 64	18.8 (7.7–35.8)	18.8 (7.4–35.7)	18.8 (7.6–35.2)	18.9 (8.0–37.0)	18.9 (8.1–36.6)
Costa Rica	65 to 69	17.6 (7.1–33.8)	17.6 (6.8–33.8)	17.6 (7.0–33.3)	17.7 (7.4–35.0)	17.7 (7.5–34.7)
Costa Rica	70 to 74	14.2 (5.6–28.2)	14.2 (5.3–28.2)	14.2 (5.5–27.7)	14.3 (5.8–29.3)	14.3 (5.8–28.9)
Costa Rica	75 to 79	12.9 (5.0–25.9)	12.8 (4.8–25.8)	12.9 (4.9–25.4)	12.9 (5.2–26.9)	12.9 (5.2–26.6)
Costa Rica	80 to 84	9.2 (3.4–19.2)	9.2 (3.3–19.1)	9.2 (3.4–18.8)	9.2 (3.6–20.0)	9.2 (3.6–19.8)
Costa Rica	85 to 89	8.3 (3.1–17.4)	8.3 (2.9–17.4)	8.3 (3.0–17.1)	8.3 (3.2–18.2)	8.3 (3.2–18.0)
Costa Rica	90 to 94	7.5 (2.8–15.9)	7.5 (2.6–15.9)	7.5 (2.7–15.6)	7.5 (2.9–16.6)	7.6 (2.9–16.4)
Costa Rica	95 plus	7.4 (2.7–15.8)	7.4 (2.6–15.7)	7.4 (2.7–15.5)	7.5 (2.8–16.5)	7.5 (2.9–16.3)
Costa Rica	Age-standardized	18.8 (8.4–34.2)	18.7 (8.9–32.6)	18.8 (8.7–32.6)	19.0 (8.4–35.8)	19.0 (8.4–36.0)
Costa Rica	All age	19.1 (8.7–34.4)	18.9 (9.2–32.6)	19.0 (8.9–32.8)	19.1 (8.4–36.0)	19.1 (8.5–36.2)
El Salvador	20 to 24	11.1 (4.3–22.2)	10.8 (4.7–20.4)	10.3 (6.2–16.2)	10.1 (7.1–13.6)	10.2 (6.5–15.4)
El Salvador	25 to 29	11.4 (4.4–23.1)	11.2 (4.5–22.3)	10.9 (5.0–20.2)	10.4 (6.5–16.4)	10.3 (6.6–16.2)
El Salvador	30 to 34	12.5 (4.8–25.2)	12.5 (4.6–25.2)	12.5 (4.8–24.7)	12.5 (5.0–26.2)	12.5 (5.0–25.9)

Supplementary Table S11: Prevalence of male SVAC by age and location for 1990, 2000, 2010, 2020, and 2023						
**Location**	**Age Range**	**1990**	**2000**	**2010**	**2020**	**2023**
El Salvador	35 to 39	12.6 (4.9–25.5)	12.6 (4.7–25.4)	12.6 (4.8–25.0)	12.7 (5.1–26.5)	12.7 (5.1–26.2)
El Salvador	40 to 44	13.1 (5.1–26.3)	13.1 (4.9–26.2)	13.1 (5.0–25.8)	13.1 (5.3–27.3)	13.1 (5.3–27.0)
El Salvador	45 to 49	12.8 (4.9–25.7)	12.8 (4.7–25.7)	12.8 (4.9–25.2)	12.8 (5.1–26.7)	12.8 (5.2–26.4)
El Salvador	50 to 54	12.5 (4.8–25.2)	12.5 (4.6–25.2)	12.5 (4.8–24.7)	12.5 (5.0–26.2)	12.5 (5.0–25.9)
El Salvador	55 to 59	12.1 (4.6–24.5)	12.1 (4.4–24.4)	12.1 (4.6–24.0)	12.1 (4.8–25.4)	12.1 (4.9–25.1)
El Salvador	60 to 64	11.7 (4.5–23.8)	11.7 (4.3–23.8)	11.7 (4.4–23.3)	11.7 (4.6–24.7)	11.7 (4.7–24.4)
El Salvador	65 to 69	10.9 (4.1–22.3)	10.8 (3.9–22.2)	10.9 (4.1–21.8)	10.9 (4.3–23.2)	10.9 (4.3–22.9)
El Salvador	70 to 74	8.6 (3.2–18.1)	8.6 (3.1–18.0)	8.6 (3.2–17.7)	8.7 (3.3–18.8)	8.6 (3.4–18.6)
El Salvador	75 to 79	7.7 (2.8–16.4)	7.7 (2.7–16.3)	7.7 (2.8–16.0)	7.8 (3.0–17.1)	7.8 (3.0–16.8)
El Salvador	80 to 84	5.4 (1.9–11.7)	5.4 (1.9–11.7)	5.4 (1.9–11.5)	5.4 (2.0–12.3)	5.4 (2.1–12.1)
El Salvador	85 to 89	4.9 (1.7–10.6)	4.9 (1.7–10.6)	4.9 (1.7–10.4)	4.9 (1.8–11.1)	4.9 (1.8–10.9)
El Salvador	90 to 94	4.4 (1.6– 9.6)	4.4 (1.5– 9.6)	4.4 (1.5– 9.4)	4.4 (1.6–10.0)	4.4 (1.6– 9.9)
El Salvador	95 plus	4.4 (1.5– 9.5)	4.3 (1.5– 9.5)	4.3 (1.5– 9.3)	4.4 (1.6–10.0)	4.4 (1.6– 9.8)
El Salvador	Age-standardized	11.6 (4.5–23.3)	11.6 (4.6–23.3)	11.5 (4.9–21.9)	11.4 (5.5–21.8)	11.4 (5.6–21.7)
El Salvador	All age	11.7 (4.6–23.5)	11.6 (4.7–23.0)	11.4 (5.0–21.6)	11.3 (5.8–21.1)	11.4 (5.8–21.0)

Supplementary Table S11: Prevalence of male SVAC by age and location for 1990, 2000, 2010, 2020, and 2023						
**Location**	**Age Range**	**1990**	**2000**	**2010**	**2020**	**2023**
Guatemala	20 to 24	14.1 (5.5–28.1)	14.1 (5.3–28.0)	14.1 (5.5–27.6)	14.2 (5.7–29.1)	14.2 (5.8–28.8)
Guatemala	25 to 29	14.5 (5.7–28.7)	14.5 (5.5–28.7)	14.5 (5.6–28.2)	14.5 (5.9–29.7)	14.5 (5.9–29.3)
Guatemala	30 to 34	15.7 (6.2–30.7)	15.7 (6.0–30.7)	15.7 (6.2–30.1)	15.7 (6.4–31.7)	15.7 (6.5–31.4)
Guatemala	35 to 39	15.8 (6.3–30.9)	15.8 (6.0–30.9)	15.8 (6.2–30.3)	15.8 (6.5–32.0)	15.8 (6.6–31.6)
Guatemala	40 to 44	16.3 (6.5–31.7)	16.3 (6.3–31.7)	16.3 (6.4–31.2)	16.4 (6.7–32.8)	16.3 (6.8–32.5)
Guatemala	45 to 49	15.9 (6.3–31.0)	15.9 (6.1–31.0)	15.9 (6.2–30.5)	15.9 (6.5–32.1)	15.9 (6.6–31.8)
Guatemala	50 to 54	15.6 (6.2–30.5)	15.5 (5.9–30.5)	15.5 (6.1–29.9)	15.6 (6.4–31.5)	15.6 (6.5–31.2)
Guatemala	55 to 59	15.1 (5.9–29.7)	15.1 (5.7–29.6)	15.0 (5.9–29.1)	15.1 (6.1–30.7)	15.1 (6.2–30.4)
Guatemala	60 to 64	14.6 (5.7–28.9)	14.6 (5.5–28.8)	14.6 (5.7–28.3)	14.6 (5.9–29.9)	14.6 (6.0–29.4)
Guatemala	65 to 69	13.6 (5.3–27.1)	13.6 (5.1–27.1)	13.6 (5.2–26.6)	13.6 (5.5–28.1)	13.6 (5.5–27.9)
Guatemala	70 to 74	10.9 (4.1–22.3)	10.9 (4.0–22.3)	10.8 (4.1–21.8)	10.9 (4.3–23.2)	10.9 (4.3–22.9)
Guatemala	75 to 79	9.8 (3.7–20.3)	9.8 (3.5–20.3)	9.8 (3.6–19.9)	9.8 (3.8–21.1)	9.8 (3.8–20.8)
Guatemala	80 to 84	6.9 (2.5–14.7)	6.9 (2.4–14.7)	6.9 (2.5–14.4)	6.9 (2.6–15.4)	6.9 (2.6–15.2)
Guatemala	85 to 89	6.2 (2.2–13.3)	6.2 (2.2–13.3)	6.2 (2.2–13.0)	6.2 (2.3–13.9)	6.2 (2.4–13.7)
Guatemala	90 to 94	5.6 (2.0–12.1)	5.6 (1.9–12.1)	5.6 (2.0–11.8)	5.6 (2.1–12.7)	5.6 (2.1–12.5)

Supplementary Table S11: Prevalence of male SVAC by age and location for 1990, 2000, 2010, 2020, and 2023						
**Location**	**Age Range**	**1990**	**2000**	**2010**	**2020**	**2023**
Guatemala	95 plus	5.6 (2.0–12.0)	5.6 (1.9–12.0)	5.6 (2.0–11.7)	5.6 (2.1–12.6)	5.6 (2.1–12.4)
Guatemala	Age-standardized	14.6 (5.7–28.8)	14.6 (5.5–28.8)	14.6 (5.7–28.3)	14.6 (6.0–29.8)	14.6 (6.0–29.5)
Guatemala	All age	14.9 (5.8–29.3)	14.8 (5.6–29.1)	14.7 (5.7–28.5)	14.8 (6.0–30.1)	14.8 (6.1–29.8)
Honduras	20 to 24	15.5 (6.3–29.8)	15.1 (6.8–27.0)	14.5 (9.5–21.3)	14.2 (10.9–17.9)	14.3 (9.5–19.9)
Honduras	25 to 29	15.8 (6.3–31.0)	15.6 (6.5–29.3)	15.1 (7.8–25.7)	14.4 (10.8–19.5)	14.3 (10.7–18.7)
Honduras	30 to 34	17.2 (6.9–33.2)	17.2 (6.7–33.2)	17.3 (6.9–32.7)	17.4 (7.2–34.5)	17.3 (7.3–34.1)
Honduras	35 to 39	17.4 (7.0–33.5)	17.4 (6.7–33.5)	17.4 (7.0–33.0)	17.5 (7.3–34.8)	17.5 (7.3–34.4)
Honduras	40 to 44	18.0 (7.3–34.4)	17.9 (7.0–34.4)	18.0 (7.2–33.9)	18.1 (7.6–35.7)	18.1 (7.7–35.3)
Honduras	45 to 49	17.5 (7.1–33.7)	17.5 (6.8–33.7)	17.6 (7.0–33.2)	17.7 (7.4–35.0)	17.6 (7.4–34.6)
Honduras	50 to 54	17.2 (6.9–33.1)	17.1 (6.6–33.1)	17.2 (6.9–32.6)	17.3 (7.2–34.4)	17.3 (7.3–34.0)
Honduras	55 to 59	16.6 (6.7–32.3)	16.6 (6.4–32.2)	16.7 (6.6–31.8)	16.8 (6.9–33.5)	16.8 (7.0–33.2)
Honduras	60 to 64	16.1 (6.4–31.5)	16.1 (6.2–31.4)	16.2 (6.4–31.0)	16.3 (6.7–32.7)	16.2 (6.8–32.3)
Honduras	65 to 69	15.0 (5.9–29.6)	15.0 (5.7–29.6)	15.1 (5.9–29.2)	15.2 (6.2–30.8)	15.1 (6.2–30.5)
Honduras	70 to 74	12.1 (4.6–24.5)	12.1 (4.4–24.4)	12.1 (4.6–24.1)	12.2 (4.8–25.5)	12.2 (4.9–25.2)
Honduras	75 to 79	10.9 (4.1–22.3)	10.9 (4.0–22.3)	10.9 (4.1–21.9)	11.0 (4.3–23.3)	11.0 (4.4–23.0)

Supplementary Table S11: Prevalence of male SVAC by age and location for 1990, 2000, 2010, 2020, and 2023						
**Location**	**Age Range**	**1990**	**2000**	**2010**	**2020**	**2023**
Honduras	80 to 84	7.7 (2.8–16.3)	7.7 (2.7–16.3)	7.7 (2.8–16.0)	7.8 (3.0–17.1)	7.8 (3.0–16.9)
Honduras	85 to 89	7.0 (2.5–14.8)	6.9 (2.4–14.8)	7.0 (2.5–14.5)	7.0 (2.6–15.5)	7.0 (2.7–15.3)
Honduras	90 to 94	6.3 (2.3–13.5)	6.3 (2.2–13.5)	6.3 (2.3–13.2)	6.3 (2.4–14.2)	6.3 (2.4–14.0)
Honduras	95 plus	6.2 (2.3–13.4)	6.2 (2.2–13.3)	6.2 (2.2–13.1)	6.3 (2.4–14.0)	6.3 (2.4–13.8)
Honduras	Age-standardized	16.1 (6.5–30.7)	16.0 (6.7–30.7)	15.9 (7.3–29.0)	15.8 (8.4–28.4)	15.8 (8.8–28.0)
Honduras	All age	16.3 (6.6–31.0)	16.2 (6.9–30.2)	16.0 (7.5–28.7)	15.9 (9.2–27.1)	15.9 (9.7–26.8)
Mexico	20 to 24	13.0 (5.0–26.1)	13.0 (4.8–26.1)	13.0 (5.0–25.7)	13.1 (5.3–27.3)	13.1 (5.3–27.0)
Mexico	25 to 29	13.3 (5.2–26.7)	13.3 (5.0–26.7)	13.4 (5.1–26.3)	13.5 (5.4–27.9)	13.5 (5.5–27.5)
Mexico	30 to 34	14.4 (5.6–28.6)	14.4 (5.4–28.6)	14.5 (5.6–28.2)	14.6 (5.9–29.8)	14.6 (6.0–29.4)
Mexico	35 to 39	14.6 (5.7–28.9)	14.6 (5.5–28.9)	14.7 (5.7–28.5)	14.8 (6.0–30.1)	14.8 (6.1–29.8)
Mexico	40 to 44	15.1 (5.9–29.7)	15.1 (5.7–29.7)	15.2 (5.9–29.3)	15.3 (6.2–31.0)	15.3 (6.3–30.7)
Mexico	45 to 49	14.7 (5.8–29.1)	14.7 (5.6–29.1)	14.8 (5.8–28.7)	14.9 (6.0–30.3)	14.9 (6.1–30.0)
Mexico	50 to 54	14.4 (5.6–28.6)	14.4 (5.4–28.5)	14.5 (5.6–28.1)	14.6 (5.9–29.8)	14.6 (6.0–29.3)
Mexico	55 to 59	13.9 (5.4–27.8)	14.0 (5.2–27.8)	14.0 (5.4–27.4)	14.1 (5.7–29.0)	14.1 (5.8–28.6)
Mexico	60 to 64	13.5 (5.2–27.0)	13.5 (5.0–27.0)	13.6 (5.2–26.6)	13.7 (5.5–28.2)	13.7 (5.6–27.9)

Supplementary Table S11: Prevalence of male SVAC by age and location for 1990, 2000, 2010, 2020, and 2023						
**Location**	**Age Range**	**1990**	**2000**	**2010**	**2020**	**2023**
Mexico	65 to 69	12.6 (4.8–25.4)	12.6 (4.7–25.3)	12.6 (4.8–25.0)	12.7 (5.1–26.5)	12.7 (5.1–26.2)
Mexico	70 to 74	10.0 (3.8–20.7)	10.0 (3.6–20.7)	10.1 (3.8–20.4)	10.1 (3.9–21.7)	10.1 (4.0–21.5)
Mexico	75 to 79	9.0 (3.3–18.8)	9.0 (3.2–18.8)	9.0 (3.3–18.5)	9.1 (3.5–19.8)	9.1 (3.6–19.5)
Mexico	80 to 84	6.4 (2.3–13.6)	6.4 (2.2–13.6)	6.4 (2.3–13.4)	6.4 (2.4–14.3)	6.4 (2.4–14.1)
Mexico	85 to 89	5.7 (2.1–12.3)	5.7 (2.0–12.3)	5.7 (2.0–12.1)	5.8 (2.2–13.0)	5.8 (2.2–12.8)
Mexico	90 to 94	5.2 (1.8–11.2)	5.2 (1.8–11.2)	5.2 (1.8–11.0)	5.2 (1.9–11.8)	5.2 (2.0–11.6)
Mexico	95 plus	5.1 (1.8–11.1)	5.1 (1.8–11.1)	5.1 (1.8–10.9)	5.2 (1.9–11.7)	5.2 (1.9–11.5)
Mexico	Age-standardized	13.5 (5.2–26.9)	13.5 (5.0–26.9)	13.5 (5.2–26.5)	13.6 (5.5–28.1)	13.6 (5.6–27.7)
Mexico	All age	13.7 (5.3–27.2)	13.7 (5.1–27.3)	13.7 (5.3–26.8)	13.8 (5.5–28.3)	13.7 (5.6–28.0)
Nicaragua	20 to 24	13.0 (5.7–24.3)	12.8 (6.8–21.0)	12.9 (6.8–21.0)	13.1 (5.8–24.3)	13.1 (5.4–24.6)
Nicaragua	25 to 29	13.0 (5.6–24.4)	12.7 (6.6–21.1)	12.7 (7.1–20.2)	13.1 (6.1–24.1)	13.2 (5.4–24.5)
Nicaragua	30 to 34	15.0 (6.6–27.6)	15.1 (7.8–24.7)	14.8 (8.2–23.1)	14.5 (7.2–25.3)	14.4 (6.4–25.7)
Nicaragua	35 to 39	14.7 (6.4–27.5)	14.5 (7.3–24.6)	14.3 (7.7–23.5)	14.2 (7.3–24.2)	14.2 (6.7–25.2)
Nicaragua	40 to 44	14.9 (6.5–27.6)	14.8 (7.2–25.6)	14.8 (7.4–25.2)	15.0 (6.7–28.7)	15.1 (6.6–29.3)
Nicaragua	45 to 49	12.7 (5.5–23.9)	11.7 (5.7–20.6)	11.8 (5.8–20.3)	12.8 (5.6–24.9)	13.1 (5.7–25.7)

Supplementary Table S11: Prevalence of male SVAC by age and location for 1990, 2000, 2010, 2020, and 2023						
**Location**	**Age Range**	**1990**	**2000**	**2010**	**2020**	**2023**
Nicaragua	50 to 54	14.9 (6.4–27.7)	15.1 (7.3–26.3)	15.2 (7.5–26.0)	15.0 (6.6–28.6)	14.9 (6.5–29.2)
Nicaragua	55 to 59	13.8 (5.4–27.5)	13.8 (5.1–27.4)	13.8 (5.3–27.0)	13.9 (5.6–28.6)	13.9 (5.7–28.1)
Nicaragua	60 to 64	13.3 (5.2–26.7)	13.3 (5.0–26.7)	13.4 (5.1–26.3)	13.4 (5.4–27.8)	13.4 (5.5–27.5)
Nicaragua	65 to 69	12.4 (4.8–25.1)	12.4 (4.6–25.0)	12.4 (4.7–24.6)	12.5 (5.0–26.1)	12.5 (5.0–25.8)
Nicaragua	70 to 74	9.9 (3.7–20.5)	9.9 (3.6–20.4)	9.9 (3.7–20.1)	10.0 (3.9–21.4)	10.0 (3.9–21.1)
Nicaragua	75 to 79	8.9 (3.3–18.6)	8.9 (3.2–18.6)	8.9 (3.3–18.2)	9.0 (3.4–19.4)	9.0 (3.5–19.2)
Nicaragua	80 to 84	6.3 (2.3–13.4)	6.3 (2.2–13.4)	6.3 (2.3–13.2)	6.3 (2.4–14.1)	6.3 (2.4–13.9)
Nicaragua	85 to 89	5.6 (2.0–12.2)	5.6 (1.9–12.1)	5.6 (2.0–11.9)	5.7 (2.1–12.7)	5.7 (2.1–12.6)
Nicaragua	90 to 94	5.1 (1.8–11.0)	5.1 (1.7–11.0)	5.1 (1.8–10.8)	5.1 (1.9–11.6)	5.1 (1.9–11.4)
Nicaragua	95 plus	5.0 (1.8–10.9)	5.0 (1.7–10.9)	5.0 (1.8–10.7)	5.1 (1.9–11.5)	5.1 (1.9–11.3)
Nicaragua	Age-standardized	13.3 (5.7–25.5)	13.2 (6.3–23.3)	13.1 (6.7–22.3)	13.3 (6.2–24.6)	13.3 (5.9–25.3)
Nicaragua	All age	13.6 (5.8–25.6)	13.4 (6.6–23.0)	13.3 (7.0–22.3)	13.5 (6.3–24.8)	13.6 (6.1–25.5)
Panama	20 to 24	14.1 (5.5–28.1)	14.1 (5.3–28.0)	14.1 (5.5–27.6)	14.2 (5.7–29.1)	14.2 (5.8–28.8)
Panama	25 to 29	14.5 (5.7–28.7)	14.5 (5.5–28.7)	14.5 (5.6–28.2)	14.5 (5.9–29.7)	14.5 (5.9–29.3)
Panama	30 to 34	15.7 (6.2–30.7)	15.7 (6.0–30.7)	15.7 (6.2–30.1)	15.7 (6.4–31.7)	15.7 (6.5–31.4)

Supplementary Table S11: Prevalence of male SVAC by age and location for 1990, 2000, 2010, 2020, and 2023						
**Location**	**Age Range**	**1990**	**2000**	**2010**	**2020**	**2023**
Panama	35 to 39	15.8 (6.3–30.9)	15.8 (6.0–30.9)	15.8 (6.2–30.3)	15.8 (6.5–32.0)	15.8 (6.6–31.6)
Panama	40 to 44	16.3 (6.5–31.7)	16.3 (6.3–31.7)	16.3 (6.4–31.2)	16.4 (6.7–32.8)	16.3 (6.8–32.5)
Panama	45 to 49	15.9 (6.3–31.0)	15.9 (6.1–31.0)	15.9 (6.2–30.5)	15.9 (6.5–32.1)	15.9 (6.6–31.8)
Panama	50 to 54	15.6 (6.2–30.5)	15.5 (5.9–30.5)	15.5 (6.1–29.9)	15.6 (6.4–31.5)	15.6 (6.5–31.2)
Panama	55 to 59	15.1 (5.9–29.7)	15.1 (5.7–29.6)	15.0 (5.9–29.1)	15.1 (6.1–30.7)	15.1 (6.2–30.4)
Panama	60 to 64	14.6 (5.7–28.9)	14.6 (5.5–28.8)	14.6 (5.7–28.3)	14.6 (5.9–29.9)	14.6 (6.0–29.4)
Panama	65 to 69	13.6 (5.3–27.1)	13.6 (5.1–27.1)	13.6 (5.2–26.6)	13.6 (5.5–28.1)	13.6 (5.5–27.9)
Panama	70 to 74	10.9 (4.1–22.3)	10.9 (4.0–22.3)	10.8 (4.1–21.8)	10.9 (4.3–23.2)	10.9 (4.3–22.9)
Panama	75 to 79	9.8 (3.7–20.3)	9.8 (3.5–20.3)	9.8 (3.6–19.9)	9.8 (3.8–21.1)	9.8 (3.8–20.8)
Panama	80 to 84	6.9 (2.5–14.7)	6.9 (2.4–14.7)	6.9 (2.5–14.4)	6.9 (2.6–15.4)	6.9 (2.6–15.2)
Panama	85 to 89	6.2 (2.2–13.3)	6.2 (2.2–13.3)	6.2 (2.2–13.0)	6.2 (2.3–13.9)	6.2 (2.4–13.7)
Panama	90 to 94	5.6 (2.0–12.1)	5.6 (1.9–12.1)	5.6 (2.0–11.8)	5.6 (2.1–12.7)	5.6 (2.1–12.5)
Panama	95 plus	5.6 (2.0–12.0)	5.6 (1.9–12.0)	5.6 (2.0–11.7)	5.6 (2.1–12.6)	5.6 (2.1–12.4)
Panama	Age-standardized	14.6 (5.7–28.8)	14.6 (5.5–28.8)	14.6 (5.7–28.3)	14.6 (6.0–29.8)	14.6 (6.0–29.5)
Panama	All age	14.8 (5.8–29.1)	14.8 (5.6–29.1)	14.7 (5.8–28.6)	14.7 (6.0–29.9)	14.7 (6.0–29.6)

Supplementary Table S11: Prevalence of male SVAC by age and location for 1990, 2000, 2010, 2020, and 2023						
**Location**	**Age Range**	**1990**	**2000**	**2010**	**2020**	**2023**
Venezuela	20 to 24	14.1 (5.5–28.1)	14.1 (5.3–28.0)	14.1 (5.5–27.6)	14.2 (5.7–29.1)	14.2 (5.8–28.8)
Venezuela	25 to 29	14.5 (5.7–28.7)	14.5 (5.5–28.7)	14.5 (5.6–28.2)	14.5 (5.9–29.7)	14.5 (5.9–29.3)
Venezuela	30 to 34	15.7 (6.2–30.7)	15.7 (6.0–30.7)	15.7 (6.2–30.1)	15.7 (6.4–31.7)	15.7 (6.5–31.4)
Venezuela	35 to 39	15.8 (6.3–30.9)	15.8 (6.0–30.9)	15.8 (6.2–30.3)	15.8 (6.5–32.0)	15.8 (6.6–31.6)
Venezuela	40 to 44	16.3 (6.5–31.7)	16.3 (6.3–31.7)	16.3 (6.4–31.2)	16.4 (6.7–32.8)	16.3 (6.8–32.5)
Venezuela	45 to 49	15.9 (6.3–31.0)	15.9 (6.1–31.0)	15.9 (6.2–30.5)	15.9 (6.5–32.1)	15.9 (6.6–31.8)
Venezuela	50 to 54	15.6 (6.2–30.5)	15.5 (5.9–30.5)	15.5 (6.1–29.9)	15.6 (6.4–31.5)	15.6 (6.5–31.2)
Venezuela	55 to 59	15.1 (5.9–29.7)	15.1 (5.7–29.6)	15.0 (5.9–29.1)	15.1 (6.1–30.7)	15.1 (6.2–30.4)
Venezuela	60 to 64	14.6 (5.7–28.9)	14.6 (5.5–28.8)	14.6 (5.7–28.3)	14.6 (5.9–29.9)	14.6 (6.0–29.4)
Venezuela	65 to 69	13.6 (5.3–27.1)	13.6 (5.1–27.1)	13.6 (5.2–26.6)	13.6 (5.5–28.1)	13.6 (5.5–27.9)
Venezuela	70 to 74	10.9 (4.1–22.3)	10.9 (4.0–22.3)	10.8 (4.1–21.8)	10.9 (4.3–23.2)	10.9 (4.3–22.9)
Venezuela	75 to 79	9.8 (3.7–20.3)	9.8 (3.5–20.3)	9.8 (3.6–19.9)	9.8 (3.8–21.1)	9.8 (3.8–20.8)
Venezuela	80 to 84	6.9 (2.5–14.7)	6.9 (2.4–14.7)	6.9 (2.5–14.4)	6.9 (2.6–15.4)	6.9 (2.6–15.2)
Venezuela	85 to 89	6.2 (2.2–13.3)	6.2 (2.2–13.3)	6.2 (2.2–13.0)	6.2 (2.3–13.9)	6.2 (2.4–13.7)
Venezuela	90 to 94	5.6 (2.0–12.1)	5.6 (1.9–12.1)	5.6 (2.0–11.8)	5.6 (2.1–12.7)	5.6 (2.1–12.5)

Supplementary Table S11: Prevalence of male SVAC by age and location for 1990, 2000, 2010, 2020, and 2023						
**Location**	**Age Range**	**1990**	**2000**	**2010**	**2020**	**2023**
Venezuela	95 plus	5.6 (2.0–12.0)	5.6 (1.9–12.0)	5.6 (2.0–11.7)	5.6 (2.1–12.6)	5.6 (2.1–12.4)
Venezuela	Age-standardized	14.6 (5.7–28.8)	14.6 (5.5–28.8)	14.6 (5.7–28.3)	14.6 (6.0–29.8)	14.6 (6.0–29.5)
Venezuela	All age	14.9 (5.9–29.4)	14.9 (5.7–29.4)	14.9 (5.8–28.8)	14.9 (6.1–30.3)	14.9 (6.1–29.9)
Tropical Latin America	20 to 24	12.3 (6.5–19.9)	12.3 (6.7–19.5)	12.3 (7.1–19.2)	12.7 (6.5–21.4)	12.7 (6.4–21.7)
Tropical Latin America	25 to 29	12.7 (6.7–20.5)	12.7 (6.9–19.9)	12.7 (7.4–19.6)	13.0 (6.7–21.9)	13.1 (6.5–22.0)
Tropical Latin America	30 to 34	13.5 (7.2–21.3)	13.4 (7.4–20.7)	13.5 (8.2–20.5)	13.8 (7.3–22.5)	13.9 (7.0–22.9)
Tropical Latin America	35 to 39	12.5 (6.6–20.1)	12.5 (6.8–19.6)	12.5 (7.3–19.4)	12.9 (6.7–21.7)	13.0 (6.5–21.9)
Tropical Latin America	40 to 44	13.1 (6.8–21.1)	13.1 (7.0–20.8)	13.1 (7.5–20.6)	13.5 (7.0–22.8)	13.6 (6.8–23.3)
Tropical Latin America	45 to 49	11.9 (6.0–19.6)	11.9 (6.1–19.3)	11.9 (6.6–19.1)	12.3 (6.2–21.5)	12.4 (6.1–21.8)
Tropical Latin America	50 to 54	11.4 (5.4–19.8)	11.3 (5.5–19.3)	11.4 (6.0–19.2)	11.8 (5.7–21.6)	11.9 (5.5–21.9)
Tropical Latin America	55 to 59	13.1 (6.5–21.8)	13.1 (6.6–21.6)	13.1 (7.3–21.0)	13.4 (6.7–23.6)	13.4 (6.5–23.8)
Tropical Latin America	60 to 64	10.3 (5.0–17.9)	10.3 (5.1–17.7)	10.4 (5.5–17.3)	10.8 (5.2–19.8)	10.9 (5.0–20.0)
Tropical Latin America	65 to 69	10.5 (5.2–17.8)	10.5 (5.3–17.6)	10.5 (5.7–17.2)	10.9 (5.3–19.5)	11.0 (5.3–19.8)
Tropical Latin America	70 to 74	13.3 (6.3–23.2)	13.3 (6.4–22.5)	13.2 (6.9–22.1)	13.1 (6.2–23.4)	13.1 (6.0–24.1)
Tropical Latin America	75 to 79	8.6 (3.9–15.9)	8.6 (3.9–15.5)	8.6 (4.1–15.3)	8.7 (3.9–16.8)	8.8 (3.9–17.2)

Supplementary Table S11: Prevalence of male SVAC by age and location for 1990, 2000, 2010, 2020, and 2023						
**Location**	**Age Range**	**1990**	**2000**	**2010**	**2020**	**2023**
Tropical Latin America	80 to 84	6.0 (2.2–12.9)	6.0 (2.2–12.5)	5.9 (2.3–12.3)	6.1 (2.3–13.0)	6.1 (2.2–13.4)
Tropical Latin America	85 to 89	5.5 (2.0–11.8)	5.4 (2.0–11.5)	5.4 (2.1–11.3)	5.5 (2.0–11.9)	5.6 (2.0–12.3)
Tropical Latin America	90 to 94	5.0 (1.8–10.8)	5.0 (1.9–10.6)	5.0 (1.9–10.4)	5.1 (1.9–10.9)	5.1 (1.8–11.3)
Tropical Latin America	95 plus	5.0 (1.8–10.6)	5.0 (1.9–10.5)	4.9 (1.9–10.3)	5.0 (1.9–10.9)	5.1 (1.8–11.2)
Tropical Latin America	Age-standardized	12.1 (6.2–19.8)	12.0 (6.3–19.6)	12.1 (6.8–19.2)	12.4 (6.3–21.6)	12.5 (6.2–21.8)
Tropical Latin America	All age	12.3 (6.4–20.0)	12.3 (6.5–19.8)	12.2 (6.9–19.5)	12.5 (6.3–21.6)	12.5 (6.2–21.9)
Brazil	20 to 24	12.3 (6.3–20.0)	12.3 (6.6–19.6)	12.3 (7.3–19.1)	12.7 (6.5–21.2)	12.7 (6.3–21.4)
Brazil	25 to 29	12.7 (6.5–20.5)	12.7 (6.9–20.1)	12.7 (7.5–19.6)	13.0 (6.7–21.6)	13.1 (6.5–21.8)
Brazil	30 to 34	13.4 (7.1–21.5)	13.4 (7.5–20.8)	13.5 (8.2–20.4)	13.8 (7.3–22.3)	13.9 (7.0–22.9)
Brazil	35 to 39	12.5 (6.4–20.1)	12.4 (6.7–19.7)	12.5 (7.4–19.3)	12.9 (6.7–21.4)	13.0 (6.4–21.8)
Brazil	40 to 44	13.1 (6.7–21.2)	13.1 (7.0–20.8)	13.1 (7.6–20.4)	13.5 (7.0–22.8)	13.6 (6.7–23.0)
Brazil	45 to 49	11.8 (5.8–19.5)	11.8 (6.1–19.2)	11.8 (6.6–18.9)	12.3 (6.2–21.3)	12.4 (6.1–21.8)
Brazil	50 to 54	11.3 (5.2–19.9)	11.3 (5.5–19.4)	11.3 (6.0–19.0)	11.7 (5.6–21.6)	11.8 (5.5–21.9)
Brazil	55 to 59	13.1 (6.4–21.9)	13.1 (6.6–21.5)	13.1 (7.3–21.0)	13.4 (6.7–23.4)	13.4 (6.5–23.9)
Brazil	60 to 64	10.3 (4.8–17.9)	10.3 (5.0–17.5)	10.3 (5.5–17.1)	10.7 (5.2–19.6)	10.8 (5.0–19.9)

Supplementary Table S11: Prevalence of male SVAC by age and location for 1990, 2000, 2010, 2020, and 2023						
**Location**	**Age Range**	**1990**	**2000**	**2010**	**2020**	**2023**
Brazil	65 to 69	10.5 (5.0–17.8)	10.5 (5.2–17.4)	10.5 (5.7–17.2)	10.8 (5.4–19.4)	10.9 (5.3–19.7)
Brazil	70 to 74	13.4 (6.2–23.5)	13.4 (6.5–22.8)	13.3 (7.0–22.2)	13.2 (6.2–23.5)	13.2 (6.1–24.3)
Brazil	75 to 79	8.6 (3.8–16.0)	8.6 (3.9–15.5)	8.6 (4.1–15.2)	8.7 (3.9–16.8)	8.8 (3.9–17.1)
Brazil	80 to 84	6.0 (2.1–13.1)	5.9 (2.2–12.6)	5.9 (2.3–12.3)	6.1 (2.2–13.0)	6.1 (2.2–13.3)
Brazil	85 to 89	5.5 (1.9–12.0)	5.4 (2.0–11.6)	5.4 (2.1–11.2)	5.5 (2.0–11.9)	5.6 (2.0–12.2)
Brazil	90 to 94	5.0 (1.8–11.1)	5.0 (1.8–10.7)	5.0 (1.9–10.3)	5.1 (1.9–10.9)	5.1 (1.8–11.2)
Brazil	95 plus	5.0 (1.7–11.0)	5.0 (1.8–10.6)	4.9 (1.9–10.3)	5.0 (1.9–10.9)	5.1 (1.8–11.2)
Brazil	Age-standardized	12.0 (6.0–19.8)	12.0 (6.2–19.5)	12.0 (6.8–19.1)	12.4 (6.3–21.3)	12.5 (6.2–21.9)
Brazil	All age	12.3 (6.2–20.1)	12.2 (6.4–19.8)	12.2 (6.9–19.2)	12.4 (6.3–21.4)	12.5 (6.2–22.0)
Paraguay	20 to 24	12.8 (4.9–25.7)	12.8 (4.7–25.7)	12.8 (4.9–25.3)	12.9 (5.1–26.9)	12.9 (5.2–26.6)
Paraguay	25 to 29	13.0 (5.0–26.1)	13.0 (4.8–26.0)	13.0 (5.0–25.6)	13.1 (5.2–27.3)	13.1 (5.3–27.0)
Paraguay	30 to 34	14.0 (5.4–27.8)	13.9 (5.2–27.7)	14.0 (5.4–27.3)	14.1 (5.7–29.0)	14.1 (5.8–28.6)
Paraguay	35 to 39	14.0 (5.5–27.9)	14.0 (5.2–27.8)	14.0 (5.4–27.4)	14.2 (5.7–29.1)	14.2 (5.8–28.8)
Paraguay	40 to 44	14.4 (5.6–28.6)	14.4 (5.4–28.5)	14.4 (5.6–28.1)	14.6 (5.9–29.8)	14.6 (5.9–29.3)
Paraguay	45 to 49	14.0 (5.5–27.9)	14.0 (5.2–27.8)	14.0 (5.4–27.4)	14.1 (5.7–29.0)	14.1 (5.8–28.7)

Supplementary Table S11: Prevalence of male SVAC by age and location for 1990, 2000, 2010, 2020, and 2023						
**Location**	**Age Range**	**1990**	**2000**	**2010**	**2020**	**2023**
Paraguay	50 to 54	13.7 (5.3–27.3)	13.7 (5.1–27.3)	13.7 (5.3–26.8)	13.8 (5.6–28.5)	13.8 (5.6–28.1)
Paraguay	55 to 59	13.3 (5.1–26.6)	13.3 (4.9–26.6)	13.3 (5.1–26.1)	13.4 (5.4–27.7)	13.4 (5.4–27.4)
Paraguay	60 to 64	12.9 (5.0–25.9)	12.9 (4.8–25.9)	12.9 (4.9–25.5)	13.0 (5.2–27.0)	13.0 (5.3–26.7)
Paraguay	65 to 69	12.1 (4.6–24.5)	12.1 (4.4–24.4)	12.1 (4.6–24.0)	12.2 (4.8–25.5)	12.2 (4.9–25.3)
Paraguay	70 to 74	9.7 (3.6–20.2)	9.7 (3.5–20.1)	9.7 (3.6–19.8)	9.8 (3.8–21.1)	9.8 (3.8–20.8)
Paraguay	75 to 79	8.7 (3.2–18.3)	8.7 (3.1–18.2)	8.7 (3.2–17.9)	8.8 (3.4–19.2)	8.8 (3.4–18.9)
Paraguay	80 to 84	6.1 (2.2–13.2)	6.1 (2.1–13.2)	6.1 (2.2–12.9)	6.2 (2.3–13.8)	6.2 (2.4–13.7)
Paraguay	85 to 89	5.5 (2.0–11.9)	5.5 (1.9–11.9)	5.5 (2.0–11.7)	5.6 (2.1–12.5)	5.6 (2.1–12.4)
Paraguay	90 to 94	5.0 (1.8–10.9)	5.0 (1.7–10.8)	5.0 (1.8–10.6)	5.0 (1.9–11.4)	5.0 (1.9–11.3)
Paraguay	95 plus	5.0 (1.8–10.8)	4.9 (1.7–10.7)	5.0 (1.8–10.5)	5.0 (1.9–11.3)	5.0 (1.9–11.2)
Paraguay	Age-standardized	13.0 (5.0–26.0)	13.0 (4.8–26.0)	13.0 (5.0–25.6)	13.1 (5.3–27.2)	13.1 (5.3–26.8)
Paraguay	All age	13.2 (5.1–26.4)	13.2 (4.9–26.4)	13.2 (5.1–25.9)	13.3 (5.3–27.5)	13.3 (5.4–27.2)
North Africa and Middle East	20 to 24	14.0 (5.5–27.9)	14.0 (5.3–27.9)	14.1 (5.5–27.5)	14.2 (5.7–29.3)	14.2 (5.7–29.7)
North Africa and Middle East	25 to 29	14.4 (5.6–28.5)	14.4 (5.4–28.5)	14.4 (5.6–28.1)	14.5 (5.8–29.9)	14.6 (5.8–30.3)
North Africa and Middle East	30 to 34	15.6 (6.2–30.5)	15.6 (5.9–30.5)	15.7 (6.2–30.1)	15.8 (6.4–31.9)	15.8 (6.4–32.5)

Supplementary Table S11: Prevalence of male SVAC by age and location for 1990, 2000, 2010, 2020, and 2023						
**Location**	**Age Range**	**1990**	**2000**	**2010**	**2020**	**2023**
North Africa and Middle East	35 to 39	15.8 (6.3–30.9)	15.8 (6.0–30.9)	15.9 (6.2–30.5)	16.0 (6.5–32.2)	16.0 (6.5–32.8)
North Africa and Middle East	40 to 44	16.4 (6.5–31.8)	16.4 (6.3–31.9)	16.5 (6.5–31.5)	16.6 (6.8–33.2)	16.6 (6.8–33.9)
North Africa and Middle East	45 to 49	16.0 (6.4–31.2)	16.0 (6.1–31.3)	16.1 (6.4–30.9)	16.2 (6.6–32.7)	16.3 (6.6–33.3)
North Africa and Middle East	50 to 54	15.7 (6.2–30.7)	15.7 (6.0–30.8)	15.8 (6.2–30.4)	15.9 (6.5–32.2)	16.0 (6.5–32.8)
North Africa and Middle East	55 to 59	15.3 (6.0–30.0)	15.3 (5.8–30.1)	15.4 (6.0–29.7)	15.5 (6.3–31.5)	15.6 (6.2–32.1)
North Africa and Middle East	60 to 64	14.9 (5.8–29.3)	14.9 (5.6–29.4)	15.0 (5.9–29.0)	15.1 (6.1–30.9)	15.2 (6.2–31.4)
North Africa and Middle East	65 to 69	13.9 (5.4–27.7)	13.9 (5.2–27.7)	14.0 (5.4–27.4)	14.1 (5.7–29.1)	14.2 (5.7–29.9)
North Africa and Middle East	70 to 74	11.1 (4.2–22.8)	11.2 (4.1–22.8)	11.3 (4.2–22.6)	11.4 (4.5–24.1)	11.4 (4.5–24.6)
North Africa and Middle East	75 to 79	10.0 (3.8–20.8)	10.1 (3.6–20.8)	10.2 (3.8–20.6)	10.3 (4.0–22.0)	10.3 (3.9–22.7)
North Africa and Middle East	80 to 84	7.1 (2.6–15.1)	7.1 (2.5–15.2)	7.2 (2.6–15.0)	7.3 (2.7–16.1)	7.3 (2.8–16.5)
North Africa and Middle East	85 to 89	6.4 (2.3–13.7)	6.4 (2.2–13.7)	6.5 (2.3–13.6)	6.5 (2.5–14.6)	6.6 (2.5–15.0)
North Africa and Middle East	90 to 94	5.8 (2.1–12.5)	5.8 (2.0–12.5)	5.9 (2.1–12.3)	5.9 (2.2–13.3)	6.0 (2.2–13.6)
North Africa and Middle East	95 plus	5.7 (2.1–12.4)	5.8 (2.0–12.4)	5.8 (2.1–12.2)	5.9 (2.2–13.2)	5.9 (2.2–13.5)
North Africa and Middle East	Age-standardized	14.7 (5.8–28.9)	14.7 (5.6–28.9)	14.7 (5.8–28.6)	14.9 (6.0–30.3)	14.9 (6.0–30.8)
North Africa and Middle East	All age	14.9 (5.9–29.3)	14.9 (5.6–29.3)	15.0 (5.9–29.0)	15.2 (6.2–30.9)	15.2 (6.2–31.4)

Supplementary Table S11: Prevalence of male SVAC by age and location for 1990, 2000, 2010, 2020, and 2023						
**Location**	**Age Range**	**1990**	**2000**	**2010**	**2020**	**2023**
North Africa and Middle East	20 to 24	14.0 (5.5–27.9)	14.0 (5.3–27.9)	14.1 (5.5–27.5)	14.2 (5.7–29.3)	14.2 (5.7–29.7)
North Africa and Middle East	25 to 29	14.4 (5.6–28.5)	14.4 (5.4–28.5)	14.4 (5.6–28.1)	14.5 (5.8–29.9)	14.6 (5.8–30.3)
North Africa and Middle East	30 to 34	15.6 (6.2–30.5)	15.6 (5.9–30.5)	15.7 (6.2–30.1)	15.8 (6.4–31.9)	15.8 (6.4–32.5)
North Africa and Middle East	35 to 39	15.8 (6.3–30.9)	15.8 (6.0–30.9)	15.9 (6.2–30.5)	16.0 (6.5–32.2)	16.0 (6.5–32.8)
North Africa and Middle East	40 to 44	16.4 (6.5–31.8)	16.4 (6.3–31.9)	16.5 (6.5–31.5)	16.6 (6.8–33.2)	16.6 (6.8–33.9)
North Africa and Middle East	45 to 49	16.0 (6.4–31.2)	16.0 (6.1–31.3)	16.1 (6.4–30.9)	16.2 (6.6–32.7)	16.3 (6.6–33.3)
North Africa and Middle East	50 to 54	15.7 (6.2–30.7)	15.7 (6.0–30.8)	15.8 (6.2–30.4)	15.9 (6.5–32.2)	16.0 (6.5–32.8)
North Africa and Middle East	55 to 59	15.3 (6.0–30.0)	15.3 (5.8–30.1)	15.4 (6.0–29.7)	15.5 (6.3–31.5)	15.6 (6.2–32.1)
North Africa and Middle East	60 to 64	14.9 (5.8–29.3)	14.9 (5.6–29.4)	15.0 (5.9–29.0)	15.1 (6.1–30.9)	15.2 (6.2–31.4)
North Africa and Middle East	65 to 69	13.9 (5.4–27.7)	13.9 (5.2–27.7)	14.0 (5.4–27.4)	14.1 (5.7–29.1)	14.2 (5.7–29.9)
North Africa and Middle East	70 to 74	11.1 (4.2–22.8)	11.2 (4.1–22.8)	11.3 (4.2–22.6)	11.4 (4.5–24.1)	11.4 (4.5–24.6)
North Africa and Middle East	75 to 79	10.0 (3.8–20.8)	10.1 (3.6–20.8)	10.2 (3.8–20.6)	10.3 (4.0–22.0)	10.3 (3.9–22.7)
North Africa and Middle East	80 to 84	7.1 (2.6–15.1)	7.1 (2.5–15.2)	7.2 (2.6–15.0)	7.3 (2.7–16.1)	7.3 (2.8–16.5)
North Africa and Middle East	85 to 89	6.4 (2.3–13.7)	6.4 (2.2–13.7)	6.5 (2.3–13.6)	6.5 (2.5–14.6)	6.6 (2.5–15.0)
North Africa and Middle East	90 to 94	5.8 (2.1–12.5)	5.8 (2.0–12.5)	5.9 (2.1–12.3)	5.9 (2.2–13.3)	6.0 (2.2–13.6)

Supplementary Table S11: Prevalence of male SVAC by age and location for 1990, 2000, 2010, 2020, and 2023						
**Location**	**Age Range**	**1990**	**2000**	**2010**	**2020**	**2023**
North Africa and Middle East	95 plus	5.7 (2.1–12.4)	5.8 (2.0–12.4)	5.8 (2.1–12.2)	5.9 (2.2–13.2)	5.9 (2.2–13.5)
North Africa and Middle East	Age-standardized	14.7 (5.8–28.9)	14.7 (5.6–28.9)	14.7 (5.8–28.6)	14.9 (6.0–30.3)	14.9 (6.0–30.8)
North Africa and Middle East	All age	14.9 (5.9–29.3)	14.9 (5.6–29.3)	15.0 (5.9–29.0)	15.2 (6.2–30.9)	15.2 (6.2–31.4)
Afghanistan	20 to 24	14.0 (5.5–27.9)	14.0 (5.3–27.9)	14.1 (5.5–27.5)	14.2 (5.7–29.3)	14.2 (5.7–29.7)
Afghanistan	25 to 29	14.4 (5.6–28.5)	14.4 (5.4–28.5)	14.4 (5.6–28.1)	14.5 (5.8–29.9)	14.6 (5.8–30.3)
Afghanistan	30 to 34	15.6 (6.2–30.5)	15.6 (5.9–30.5)	15.7 (6.2–30.1)	15.8 (6.4–31.9)	15.8 (6.4–32.5)
Afghanistan	35 to 39	15.8 (6.3–30.9)	15.8 (6.0–30.9)	15.9 (6.2–30.5)	16.0 (6.5–32.2)	16.0 (6.5–32.8)
Afghanistan	40 to 44	16.4 (6.5–31.8)	16.4 (6.3–31.9)	16.5 (6.5–31.5)	16.6 (6.8–33.2)	16.6 (6.8–33.9)
Afghanistan	45 to 49	16.0 (6.4–31.2)	16.0 (6.1–31.3)	16.1 (6.4–30.9)	16.2 (6.6–32.7)	16.3 (6.6–33.3)
Afghanistan	50 to 54	15.7 (6.2–30.7)	15.7 (6.0–30.8)	15.8 (6.2–30.4)	15.9 (6.5–32.2)	16.0 (6.5–32.8)
Afghanistan	55 to 59	15.3 (6.0–30.0)	15.3 (5.8–30.1)	15.4 (6.0–29.7)	15.5 (6.3–31.5)	15.6 (6.2–32.1)
Afghanistan	60 to 64	14.9 (5.8–29.3)	14.9 (5.6–29.4)	15.0 (5.9–29.0)	15.1 (6.1–30.9)	15.2 (6.2–31.4)
Afghanistan	65 to 69	13.9 (5.4–27.7)	13.9 (5.2–27.7)	14.0 (5.4–27.4)	14.1 (5.7–29.1)	14.2 (5.7–29.9)
Afghanistan	70 to 74	11.1 (4.2–22.8)	11.2 (4.1–22.8)	11.3 (4.2–22.6)	11.4 (4.5–24.1)	11.4 (4.5–24.6)
Afghanistan	75 to 79	10.0 (3.8–20.8)	10.1 (3.6–20.8)	10.2 (3.8–20.6)	10.3 (4.0–22.0)	10.3 (3.9–22.7)

Supplementary Table S11: Prevalence of male SVAC by age and location for 1990, 2000, 2010, 2020, and 2023						
**Location**	**Age Range**	**1990**	**2000**	**2010**	**2020**	**2023**
Afghanistan	80 to 84	7.1 (2.6–15.1)	7.1 (2.5–15.2)	7.2 (2.6–15.0)	7.3 (2.7–16.1)	7.3 (2.8–16.5)
Afghanistan	85 to 89	6.4 (2.3–13.7)	6.4 (2.2–13.7)	6.5 (2.3–13.6)	6.5 (2.5–14.6)	6.6 (2.5–15.0)
Afghanistan	90 to 94	5.8 (2.1–12.5)	5.8 (2.0–12.5)	5.9 (2.1–12.3)	5.9 (2.2–13.3)	6.0 (2.2–13.6)
Afghanistan	95 plus	5.7 (2.1–12.4)	5.8 (2.0–12.4)	5.8 (2.1–12.2)	5.9 (2.2–13.2)	5.9 (2.2–13.5)
Afghanistan	Age-standardized	14.7 (5.8–28.9)	14.7 (5.6–28.9)	14.7 (5.8–28.6)	14.9 (6.0–30.3)	14.9 (6.0–30.8)
Afghanistan	All age	14.7 (5.8–29.1)	14.6 (5.5–28.8)	15.0 (5.9–29.0)	15.1 (6.1–30.8)	15.2 (6.1–31.4)
Algeria	20 to 24	14.0 (5.5–27.9)	14.0 (5.3–27.9)	14.1 (5.5–27.5)	14.2 (5.7–29.3)	14.2 (5.7–29.7)
Algeria	25 to 29	14.4 (5.6–28.5)	14.4 (5.4–28.5)	14.4 (5.6–28.1)	14.5 (5.8–29.9)	14.6 (5.8–30.3)
Algeria	30 to 34	15.6 (6.2–30.5)	15.6 (5.9–30.5)	15.7 (6.2–30.1)	15.8 (6.4–31.9)	15.8 (6.4–32.5)
Algeria	35 to 39	15.8 (6.3–30.9)	15.8 (6.0–30.9)	15.9 (6.2–30.5)	16.0 (6.5–32.2)	16.0 (6.5–32.8)
Algeria	40 to 44	16.4 (6.5–31.8)	16.4 (6.3–31.9)	16.5 (6.5–31.5)	16.6 (6.8–33.2)	16.6 (6.8–33.9)
Algeria	45 to 49	16.0 (6.4–31.2)	16.0 (6.1–31.3)	16.1 (6.4–30.9)	16.2 (6.6–32.7)	16.3 (6.6–33.3)
Algeria	50 to 54	15.7 (6.2–30.7)	15.7 (6.0–30.8)	15.8 (6.2–30.4)	15.9 (6.5–32.2)	16.0 (6.5–32.8)
Algeria	55 to 59	15.3 (6.0–30.0)	15.3 (5.8–30.1)	15.4 (6.0–29.7)	15.5 (6.3–31.5)	15.6 (6.2–32.1)
Algeria	60 to 64	14.9 (5.8–29.3)	14.9 (5.6–29.4)	15.0 (5.9–29.0)	15.1 (6.1–30.9)	15.2 (6.2–31.4)

Supplementary Table S11: Prevalence of male SVAC by age and location for 1990, 2000, 2010, 2020, and 2023						
**Location**	**Age Range**	**1990**	**2000**	**2010**	**2020**	**2023**
Algeria	65 to 69	13.9 (5.4–27.7)	13.9 (5.2–27.7)	14.0 (5.4–27.4)	14.1 (5.7–29.1)	14.2 (5.7–29.9)
Algeria	70 to 74	11.1 (4.2–22.8)	11.2 (4.1–22.8)	11.3 (4.2–22.6)	11.4 (4.5–24.1)	11.4 (4.5–24.6)
Algeria	75 to 79	10.0 (3.8–20.8)	10.1 (3.6–20.8)	10.2 (3.8–20.6)	10.3 (4.0–22.0)	10.3 (3.9–22.7)
Algeria	80 to 84	7.1 (2.6–15.1)	7.1 (2.5–15.2)	7.2 (2.6–15.0)	7.3 (2.7–16.1)	7.3 (2.8–16.5)
Algeria	85 to 89	6.4 (2.3–13.7)	6.4 (2.2–13.7)	6.5 (2.3–13.6)	6.5 (2.5–14.6)	6.6 (2.5–15.0)
Algeria	90 to 94	5.8 (2.1–12.5)	5.8 (2.0–12.5)	5.9 (2.1–12.3)	5.9 (2.2–13.3)	6.0 (2.2–13.6)
Algeria	95 plus	5.7 (2.1–12.4)	5.8 (2.0–12.4)	5.8 (2.1–12.2)	5.9 (2.2–13.2)	5.9 (2.2–13.5)
Algeria	Age-standardized	14.7 (5.8–28.9)	14.7 (5.6–28.9)	14.7 (5.8–28.6)	14.9 (6.0–30.3)	14.9 (6.0–30.8)
Algeria	All age	14.8 (5.8–29.1)	14.8 (5.6–29.2)	14.9 (5.8–28.9)	15.1 (6.1–30.7)	15.2 (6.1–31.3)
Bahrain	20 to 24	14.0 (5.5–27.9)	14.0 (5.3–27.9)	14.1 (5.5–27.5)	14.2 (5.7–29.3)	14.2 (5.7–29.7)
Bahrain	25 to 29	14.4 (5.6–28.5)	14.4 (5.4–28.5)	14.4 (5.6–28.1)	14.5 (5.8–29.9)	14.6 (5.8–30.3)
Bahrain	30 to 34	15.6 (6.2–30.5)	15.6 (5.9–30.5)	15.7 (6.2–30.1)	15.8 (6.4–31.9)	15.8 (6.4–32.5)
Bahrain	35 to 39	15.8 (6.3–30.9)	15.8 (6.0–30.9)	15.9 (6.2–30.5)	16.0 (6.5–32.2)	16.0 (6.5–32.8)
Bahrain	40 to 44	16.4 (6.5–31.8)	16.4 (6.3–31.9)	16.5 (6.5–31.5)	16.6 (6.8–33.2)	16.6 (6.8–33.9)
Bahrain	45 to 49	16.0 (6.4–31.2)	16.0 (6.1–31.3)	16.1 (6.4–30.9)	16.2 (6.6–32.7)	16.3 (6.6–33.3)

Supplementary Table S11: Prevalence of male SVAC by age and location for 1990, 2000, 2010, 2020, and 2023						
**Location**	**Age Range**	**1990**	**2000**	**2010**	**2020**	**2023**
Bahrain	50 to 54	15.7 (6.2–30.7)	15.7 (6.0–30.8)	15.8 (6.2–30.4)	15.9 (6.5–32.2)	16.0 (6.5–32.8)
Bahrain	55 to 59	15.3 (6.0–30.0)	15.3 (5.8–30.1)	15.4 (6.0–29.7)	15.5 (6.3–31.5)	15.6 (6.2–32.1)
Bahrain	60 to 64	14.9 (5.8–29.3)	14.9 (5.6–29.4)	15.0 (5.9–29.0)	15.1 (6.1–30.9)	15.2 (6.2–31.4)
Bahrain	65 to 69	13.9 (5.4–27.7)	13.9 (5.2–27.7)	14.0 (5.4–27.4)	14.1 (5.7–29.1)	14.2 (5.7–29.9)
Bahrain	70 to 74	11.1 (4.2–22.8)	11.2 (4.1–22.8)	11.3 (4.2–22.6)	11.4 (4.5–24.1)	11.4 (4.5–24.6)
Bahrain	75 to 79	10.0 (3.8–20.8)	10.1 (3.6–20.8)	10.2 (3.8–20.6)	10.3 (4.0–22.0)	10.3 (3.9–22.7)
Bahrain	80 to 84	7.1 (2.6–15.1)	7.1 (2.5–15.2)	7.2 (2.6–15.0)	7.3 (2.7–16.1)	7.3 (2.8–16.5)
Bahrain	85 to 89	6.4 (2.3–13.7)	6.4 (2.2–13.7)	6.5 (2.3–13.6)	6.5 (2.5–14.6)	6.6 (2.5–15.0)
Bahrain	90 to 94	5.8 (2.1–12.5)	5.8 (2.0–12.5)	5.9 (2.1–12.3)	5.9 (2.2–13.3)	6.0 (2.2–13.6)
Bahrain	95 plus	5.7 (2.1–12.4)	5.8 (2.0–12.4)	5.8 (2.1–12.2)	5.9 (2.2–13.2)	5.9 (2.2–13.5)
Bahrain	Age-standardized	14.7 (5.8–28.9)	14.7 (5.6–28.9)	14.7 (5.8–28.6)	14.9 (6.0–30.3)	14.9 (6.0–30.8)
Bahrain	All age	15.1 (6.0–29.8)	15.2 (5.8–29.9)	15.3 (6.0–29.5)	15.5 (6.3–31.4)	15.5 (6.3–32.0)
Egypt	20 to 24	14.0 (5.5–27.9)	14.0 (5.3–27.9)	14.1 (5.5–27.5)	14.2 (5.7–29.3)	14.2 (5.7–29.7)
Egypt	25 to 29	14.4 (5.6–28.5)	14.4 (5.4–28.5)	14.4 (5.6–28.1)	14.5 (5.8–29.9)	14.6 (5.8–30.3)
Egypt	30 to 34	15.6 (6.2–30.5)	15.6 (5.9–30.5)	15.7 (6.2–30.1)	15.8 (6.4–31.9)	15.8 (6.4–32.5)

Supplementary Table S11: Prevalence of male SVAC by age and location for 1990, 2000, 2010, 2020, and 2023						
**Location**	**Age Range**	**1990**	**2000**	**2010**	**2020**	**2023**
Egypt	35 to 39	15.8 (6.3–30.9)	15.8 (6.0–30.9)	15.9 (6.2–30.5)	16.0 (6.5–32.2)	16.0 (6.5–32.8)
Egypt	40 to 44	16.4 (6.5–31.8)	16.4 (6.3–31.9)	16.5 (6.5–31.5)	16.6 (6.8–33.2)	16.6 (6.8–33.9)
Egypt	45 to 49	16.0 (6.4–31.2)	16.0 (6.1–31.3)	16.1 (6.4–30.9)	16.2 (6.6–32.7)	16.3 (6.6–33.3)
Egypt	50 to 54	15.7 (6.2–30.7)	15.7 (6.0–30.8)	15.8 (6.2–30.4)	15.9 (6.5–32.2)	16.0 (6.5–32.8)
Egypt	55 to 59	15.3 (6.0–30.0)	15.3 (5.8–30.1)	15.4 (6.0–29.7)	15.5 (6.3–31.5)	15.6 (6.2–32.1)
Egypt	60 to 64	14.9 (5.8–29.3)	14.9 (5.6–29.4)	15.0 (5.9–29.0)	15.1 (6.1–30.9)	15.2 (6.2–31.4)
Egypt	65 to 69	13.9 (5.4–27.7)	13.9 (5.2–27.7)	14.0 (5.4–27.4)	14.1 (5.7–29.1)	14.2 (5.7–29.9)
Egypt	70 to 74	11.1 (4.2–22.8)	11.2 (4.1–22.8)	11.3 (4.2–22.6)	11.4 (4.5–24.1)	11.4 (4.5–24.6)
Egypt	75 to 79	10.0 (3.8–20.8)	10.1 (3.6–20.8)	10.2 (3.8–20.6)	10.3 (4.0–22.0)	10.3 (3.9–22.7)
Egypt	80 to 84	7.1 (2.6–15.1)	7.1 (2.5–15.2)	7.2 (2.6–15.0)	7.3 (2.7–16.1)	7.3 (2.8–16.5)
Egypt	85 to 89	6.4 (2.3–13.7)	6.4 (2.2–13.7)	6.5 (2.3–13.6)	6.5 (2.5–14.6)	6.6 (2.5–15.0)
Egypt	90 to 94	5.8 (2.1–12.5)	5.8 (2.0–12.5)	5.9 (2.1–12.3)	5.9 (2.2–13.3)	6.0 (2.2–13.6)
Egypt	95 plus	5.7 (2.1–12.4)	5.8 (2.0–12.4)	5.8 (2.1–12.2)	5.9 (2.2–13.2)	5.9 (2.2–13.5)
Egypt	Age-standardized	14.7 (5.8–28.9)	14.7 (5.6–28.9)	14.7 (5.8–28.6)	14.9 (6.0–30.3)	14.9 (6.0–30.8)
Egypt	All age	14.9 (5.9–29.4)	15.0 (5.7–29.5)	15.0 (5.9–29.1)	15.2 (6.2–30.9)	15.2 (6.2–31.5)

Supplementary Table S11: Prevalence of male SVAC by age and location for 1990, 2000, 2010, 2020, and 2023						
**Location**	**Age Range**	**1990**	**2000**	**2010**	**2020**	**2023**
Iran	20 to 24	14.0 (5.5–27.9)	14.0 (5.3–27.9)	14.1 (5.5–27.5)	14.2 (5.7–29.3)	14.2 (5.7–29.7)
Iran	25 to 29	14.4 (5.6–28.5)	14.4 (5.4–28.5)	14.4 (5.6–28.1)	14.5 (5.8–29.9)	14.6 (5.8–30.3)
Iran	30 to 34	15.6 (6.2–30.5)	15.6 (5.9–30.5)	15.7 (6.2–30.1)	15.8 (6.4–31.9)	15.8 (6.4–32.5)
Iran	35 to 39	15.8 (6.3–30.9)	15.8 (6.0–30.9)	15.9 (6.2–30.5)	16.0 (6.5–32.2)	16.0 (6.5–32.8)
Iran	40 to 44	16.4 (6.5–31.8)	16.4 (6.3–31.9)	16.5 (6.5–31.5)	16.6 (6.8–33.2)	16.6 (6.8–33.9)
Iran	45 to 49	16.0 (6.4–31.2)	16.0 (6.1–31.3)	16.1 (6.4–30.9)	16.2 (6.6–32.7)	16.3 (6.6–33.3)
Iran	50 to 54	15.7 (6.2–30.7)	15.7 (6.0–30.8)	15.8 (6.2–30.4)	15.9 (6.5–32.2)	16.0 (6.5–32.8)
Iran	55 to 59	15.3 (6.0–30.0)	15.3 (5.8–30.1)	15.4 (6.0–29.7)	15.5 (6.3–31.5)	15.6 (6.2–32.1)
Iran	60 to 64	14.9 (5.8–29.3)	14.9 (5.6–29.4)	15.0 (5.9–29.0)	15.1 (6.1–30.9)	15.2 (6.2–31.4)
Iran	65 to 69	13.9 (5.4–27.7)	13.9 (5.2–27.7)	14.0 (5.4–27.4)	14.1 (5.7–29.1)	14.2 (5.7–29.9)
Iran	70 to 74	11.1 (4.2–22.8)	11.2 (4.1–22.8)	11.3 (4.2–22.6)	11.4 (4.5–24.1)	11.4 (4.5–24.6)
Iran	75 to 79	10.0 (3.8–20.8)	10.1 (3.6–20.8)	10.2 (3.8–20.6)	10.3 (4.0–22.0)	10.3 (3.9–22.7)
Iran	80 to 84	7.1 (2.6–15.1)	7.1 (2.5–15.2)	7.2 (2.6–15.0)	7.3 (2.7–16.1)	7.3 (2.8–16.5)
Iran	85 to 89	6.4 (2.3–13.7)	6.4 (2.2–13.7)	6.5 (2.3–13.6)	6.5 (2.5–14.6)	6.6 (2.5–15.0)
Iran	90 to 94	5.8 (2.1–12.5)	5.8 (2.0–12.5)	5.9 (2.1–12.3)	5.9 (2.2–13.3)	6.0 (2.2–13.6)

Supplementary Table S11: Prevalence of male SVAC by age and location for 1990, 2000, 2010, 2020, and 2023						
**Location**	**Age Range**	**1990**	**2000**	**2010**	**2020**	**2023**
Iran	95 plus	5.7 (2.1–12.4)	5.8 (2.0–12.4)	5.8 (2.1–12.2)	5.9 (2.2–13.2)	5.9 (2.2–13.5)
Iran	Age-standardized	14.7 (5.8–28.9)	14.7 (5.6–28.9)	14.7 (5.8–28.6)	14.9 (6.0–30.3)	14.9 (6.0–30.8)
Iran	All age	14.8 (5.8–29.2)	14.8 (5.6–29.2)	14.9 (5.8–28.9)	15.2 (6.2–30.9)	15.3 (6.2–31.5)
Iraq	20 to 24	14.0 (5.5–27.9)	14.0 (5.3–27.9)	14.1 (5.5–27.5)	14.2 (5.7–29.3)	14.2 (5.7–29.7)
Iraq	25 to 29	14.4 (5.6–28.5)	14.4 (5.4–28.5)	14.4 (5.6–28.1)	14.5 (5.8–29.9)	14.6 (5.8–30.3)
Iraq	30 to 34	15.6 (6.2–30.5)	15.6 (5.9–30.5)	15.7 (6.2–30.1)	15.8 (6.4–31.9)	15.8 (6.4–32.5)
Iraq	35 to 39	15.8 (6.3–30.9)	15.8 (6.0–30.9)	15.9 (6.2–30.5)	16.0 (6.5–32.2)	16.0 (6.5–32.8)
Iraq	40 to 44	16.4 (6.5–31.8)	16.4 (6.3–31.9)	16.5 (6.5–31.5)	16.6 (6.8–33.2)	16.6 (6.8–33.9)
Iraq	45 to 49	16.0 (6.4–31.2)	16.0 (6.1–31.3)	16.1 (6.4–30.9)	16.2 (6.6–32.7)	16.3 (6.6–33.3)
Iraq	50 to 54	15.7 (6.2–30.7)	15.7 (6.0–30.8)	15.8 (6.2–30.4)	15.9 (6.5–32.2)	16.0 (6.5–32.8)
Iraq	55 to 59	15.3 (6.0–30.0)	15.3 (5.8–30.1)	15.4 (6.0–29.7)	15.5 (6.3–31.5)	15.6 (6.2–32.1)
Iraq	60 to 64	14.9 (5.8–29.3)	14.9 (5.6–29.4)	15.0 (5.9–29.0)	15.1 (6.1–30.9)	15.2 (6.2–31.4)
Iraq	65 to 69	13.9 (5.4–27.7)	13.9 (5.2–27.7)	14.0 (5.4–27.4)	14.1 (5.7–29.1)	14.2 (5.7–29.9)
Iraq	70 to 74	11.1 (4.2–22.8)	11.2 (4.1–22.8)	11.3 (4.2–22.6)	11.4 (4.5–24.1)	11.4 (4.5–24.6)
Iraq	75 to 79	10.0 (3.8–20.8)	10.1 (3.6–20.8)	10.2 (3.8–20.6)	10.3 (4.0–22.0)	10.3 (3.9–22.7)

Supplementary Table S11: Prevalence of male SVAC by age and location for 1990, 2000, 2010, 2020, and 2023						
**Location**	**Age Range**	**1990**	**2000**	**2010**	**2020**	**2023**
Iraq	80 to 84	7.1 (2.6–15.1)	7.1 (2.5–15.2)	7.2 (2.6–15.0)	7.3 (2.7–16.1)	7.3 (2.8–16.5)
Iraq	85 to 89	6.4 (2.3–13.7)	6.4 (2.2–13.7)	6.5 (2.3–13.6)	6.5 (2.5–14.6)	6.6 (2.5–15.0)
Iraq	90 to 94	5.8 (2.1–12.5)	5.8 (2.0–12.5)	5.9 (2.1–12.3)	5.9 (2.2–13.3)	6.0 (2.2–13.6)
Iraq	95 plus	5.7 (2.1–12.4)	5.8 (2.0–12.4)	5.8 (2.1–12.2)	5.9 (2.2–13.2)	5.9 (2.2–13.5)
Iraq	Age-standardized	14.7 (5.8–28.9)	14.7 (5.6–28.9)	14.7 (5.8–28.6)	14.9 (6.0–30.3)	14.9 (6.0–30.8)
Iraq	All age	14.8 (5.8–29.2)	14.9 (5.6–29.4)	15.1 (5.9–29.2)	15.2 (6.2–30.9)	15.2 (6.2–31.4)
Jordan	20 to 24	14.0 (5.5–27.9)	14.0 (5.3–27.9)	14.1 (5.5–27.5)	14.2 (5.7–29.3)	14.2 (5.7–29.7)
Jordan	25 to 29	14.4 (5.6–28.5)	14.4 (5.4–28.5)	14.4 (5.6–28.1)	14.5 (5.8–29.9)	14.6 (5.8–30.3)
Jordan	30 to 34	15.6 (6.2–30.5)	15.6 (5.9–30.5)	15.7 (6.2–30.1)	15.8 (6.4–31.9)	15.8 (6.4–32.5)
Jordan	35 to 39	15.8 (6.3–30.9)	15.8 (6.0–30.9)	15.9 (6.2–30.5)	16.0 (6.5–32.2)	16.0 (6.5–32.8)
Jordan	40 to 44	16.4 (6.5–31.8)	16.4 (6.3–31.9)	16.5 (6.5–31.5)	16.6 (6.8–33.2)	16.6 (6.8–33.9)
Jordan	45 to 49	16.0 (6.4–31.2)	16.0 (6.1–31.3)	16.1 (6.4–30.9)	16.2 (6.6–32.7)	16.3 (6.6–33.3)
Jordan	50 to 54	15.7 (6.2–30.7)	15.7 (6.0–30.8)	15.8 (6.2–30.4)	15.9 (6.5–32.2)	16.0 (6.5–32.8)
Jordan	55 to 59	15.3 (6.0–30.0)	15.3 (5.8–30.1)	15.4 (6.0–29.7)	15.5 (6.3–31.5)	15.6 (6.2–32.1)
Jordan	60 to 64	14.9 (5.8–29.3)	14.9 (5.6–29.4)	15.0 (5.9–29.0)	15.1 (6.1–30.9)	15.2 (6.2–31.4)

Supplementary Table S11: Prevalence of male SVAC by age and location for 1990, 2000, 2010, 2020, and 2023						
**Location**	**Age Range**	**1990**	**2000**	**2010**	**2020**	**2023**
Jordan	65 to 69	13.9 (5.4–27.7)	13.9 (5.2–27.7)	14.0 (5.4–27.4)	14.1 (5.7–29.1)	14.2 (5.7–29.9)
Jordan	70 to 74	11.1 (4.2–22.8)	11.2 (4.1–22.8)	11.3 (4.2–22.6)	11.4 (4.5–24.1)	11.4 (4.5–24.6)
Jordan	75 to 79	10.0 (3.8–20.8)	10.1 (3.6–20.8)	10.2 (3.8–20.6)	10.3 (4.0–22.0)	10.3 (3.9–22.7)
Jordan	80 to 84	7.1 (2.6–15.1)	7.1 (2.5–15.2)	7.2 (2.6–15.0)	7.3 (2.7–16.1)	7.3 (2.8–16.5)
Jordan	85 to 89	6.4 (2.3–13.7)	6.4 (2.2–13.7)	6.5 (2.3–13.6)	6.5 (2.5–14.6)	6.6 (2.5–15.0)
Jordan	90 to 94	5.8 (2.1–12.5)	5.8 (2.0–12.5)	5.9 (2.1–12.3)	5.9 (2.2–13.3)	6.0 (2.2–13.6)
Jordan	95 plus	5.7 (2.1–12.4)	5.8 (2.0–12.4)	5.8 (2.1–12.2)	5.9 (2.2–13.2)	5.9 (2.2–13.5)
Jordan	Age-standardized	14.7 (5.8–28.9)	14.7 (5.6–28.9)	14.7 (5.8–28.6)	14.9 (6.0–30.3)	14.9 (6.0–30.8)
Jordan	All age	14.9 (5.8–29.3)	14.9 (5.6–29.3)	15.0 (5.9–29.0)	15.2 (6.2–30.9)	15.3 (6.2–31.5)
Kuwait	20 to 24	14.0 (5.5–27.9)	14.0 (5.3–27.9)	14.1 (5.5–27.5)	14.2 (5.7–29.3)	14.2 (5.7–29.7)
Kuwait	25 to 29	14.4 (5.6–28.5)	14.4 (5.4–28.5)	14.4 (5.6–28.1)	14.5 (5.8–29.9)	14.6 (5.8–30.3)
Kuwait	30 to 34	15.6 (6.2–30.5)	15.6 (5.9–30.5)	15.7 (6.2–30.1)	15.8 (6.4–31.9)	15.8 (6.4–32.5)
Kuwait	35 to 39	15.8 (6.3–30.9)	15.8 (6.0–30.9)	15.9 (6.2–30.5)	16.0 (6.5–32.2)	16.0 (6.5–32.8)
Kuwait	40 to 44	16.4 (6.5–31.8)	16.4 (6.3–31.9)	16.5 (6.5–31.5)	16.6 (6.8–33.2)	16.6 (6.8–33.9)
Kuwait	45 to 49	16.0 (6.4–31.2)	16.0 (6.1–31.3)	16.1 (6.4–30.9)	16.2 (6.6–32.7)	16.3 (6.6–33.3)

Supplementary Table S11: Prevalence of male SVAC by age and location for 1990, 2000, 2010, 2020, and 2023						
**Location**	**Age Range**	**1990**	**2000**	**2010**	**2020**	**2023**
Kuwait	50 to 54	15.7 (6.2–30.7)	15.7 (6.0–30.8)	15.8 (6.2–30.4)	15.9 (6.5–32.2)	16.0 (6.5–32.8)
Kuwait	55 to 59	15.3 (6.0–30.0)	15.3 (5.8–30.1)	15.4 (6.0–29.7)	15.5 (6.3–31.5)	15.6 (6.2–32.1)
Kuwait	60 to 64	14.9 (5.8–29.3)	14.9 (5.6–29.4)	15.0 (5.9–29.0)	15.1 (6.1–30.9)	15.2 (6.2–31.4)
Kuwait	65 to 69	13.9 (5.4–27.7)	13.9 (5.2–27.7)	14.0 (5.4–27.4)	14.1 (5.7–29.1)	14.2 (5.7–29.9)
Kuwait	70 to 74	11.1 (4.2–22.8)	11.2 (4.1–22.8)	11.3 (4.2–22.6)	11.4 (4.5–24.1)	11.4 (4.5–24.6)
Kuwait	75 to 79	10.0 (3.8–20.8)	10.1 (3.6–20.8)	10.2 (3.8–20.6)	10.3 (4.0–22.0)	10.3 (3.9–22.7)
Kuwait	80 to 84	7.1 (2.6–15.1)	7.1 (2.5–15.2)	7.2 (2.6–15.0)	7.3 (2.7–16.1)	7.3 (2.8–16.5)
Kuwait	85 to 89	6.4 (2.3–13.7)	6.4 (2.2–13.7)	6.5 (2.3–13.6)	6.5 (2.5–14.6)	6.6 (2.5–15.0)
Kuwait	90 to 94	5.8 (2.1–12.5)	5.8 (2.0–12.5)	5.9 (2.1–12.3)	5.9 (2.2–13.3)	6.0 (2.2–13.6)
Kuwait	95 plus	5.7 (2.1–12.4)	5.8 (2.0–12.4)	5.8 (2.1–12.2)	5.9 (2.2–13.2)	5.9 (2.2–13.5)
Kuwait	Age-standardized	14.7 (5.8–28.9)	14.7 (5.6–28.9)	14.7 (5.8–28.6)	14.9 (6.0–30.3)	14.9 (6.0–30.8)
Kuwait	All age	15.2 (6.0–29.9)	15.3 (5.8–30.0)	15.4 (6.0–29.6)	15.5 (6.3–31.5)	15.5 (6.3–32.0)
Lebanon	20 to 24	14.0 (5.5–27.9)	14.0 (5.3–27.9)	14.1 (5.5–27.5)	14.2 (5.7–29.3)	14.2 (5.7–29.7)
Lebanon	25 to 29	14.4 (5.6–28.5)	14.4 (5.4–28.5)	14.4 (5.6–28.1)	14.5 (5.8–29.9)	14.6 (5.8–30.3)
Lebanon	30 to 34	15.6 (6.2–30.5)	15.6 (5.9–30.5)	15.7 (6.2–30.1)	15.8 (6.4–31.9)	15.8 (6.4–32.5)

Supplementary Table S11: Prevalence of male SVAC by age and location for 1990, 2000, 2010, 2020, and 2023						
**Location**	**Age Range**	**1990**	**2000**	**2010**	**2020**	**2023**
Lebanon	35 to 39	15.8 (6.3–30.9)	15.8 (6.0–30.9)	15.9 (6.2–30.5)	16.0 (6.5–32.2)	16.0 (6.5–32.8)
Lebanon	40 to 44	16.4 (6.5–31.8)	16.4 (6.3–31.9)	16.5 (6.5–31.5)	16.6 (6.8–33.2)	16.6 (6.8–33.9)
Lebanon	45 to 49	16.0 (6.4–31.2)	16.0 (6.1–31.3)	16.1 (6.4–30.9)	16.2 (6.6–32.7)	16.3 (6.6–33.3)
Lebanon	50 to 54	15.7 (6.2–30.7)	15.7 (6.0–30.8)	15.8 (6.2–30.4)	15.9 (6.5–32.2)	16.0 (6.5–32.8)
Lebanon	55 to 59	15.3 (6.0–30.0)	15.3 (5.8–30.1)	15.4 (6.0–29.7)	15.5 (6.3–31.5)	15.6 (6.2–32.1)
Lebanon	60 to 64	14.9 (5.8–29.3)	14.9 (5.6–29.4)	15.0 (5.9–29.0)	15.1 (6.1–30.9)	15.2 (6.2–31.4)
Lebanon	65 to 69	13.9 (5.4–27.7)	13.9 (5.2–27.7)	14.0 (5.4–27.4)	14.1 (5.7–29.1)	14.2 (5.7–29.9)
Lebanon	70 to 74	11.1 (4.2–22.8)	11.2 (4.1–22.8)	11.3 (4.2–22.6)	11.4 (4.5–24.1)	11.4 (4.5–24.6)
Lebanon	75 to 79	10.0 (3.8–20.8)	10.1 (3.6–20.8)	10.2 (3.8–20.6)	10.3 (4.0–22.0)	10.3 (3.9–22.7)
Lebanon	80 to 84	7.1 (2.6–15.1)	7.1 (2.5–15.2)	7.2 (2.6–15.0)	7.3 (2.7–16.1)	7.3 (2.8–16.5)
Lebanon	85 to 89	6.4 (2.3–13.7)	6.4 (2.2–13.7)	6.5 (2.3–13.6)	6.5 (2.5–14.6)	6.6 (2.5–15.0)
Lebanon	90 to 94	5.8 (2.1–12.5)	5.8 (2.0–12.5)	5.9 (2.1–12.3)	5.9 (2.2–13.3)	6.0 (2.2–13.6)
Lebanon	95 plus	5.7 (2.1–12.4)	5.8 (2.0–12.4)	5.8 (2.1–12.2)	5.9 (2.2–13.2)	5.9 (2.2–13.5)
Lebanon	Age-standardized	14.7 (5.8–28.9)	14.7 (5.6–28.9)	14.7 (5.8–28.6)	14.9 (6.0–30.3)	14.9 (6.0–30.8)
Lebanon	All age	14.8 (5.8–29.2)	14.7 (5.6–29.0)	14.6 (5.7–28.4)	14.9 (6.0–30.3)	15.0 (6.1–31.0)

Supplementary Table S11: Prevalence of male SVAC by age and location for 1990, 2000, 2010, 2020, and 2023						
**Location**	**Age Range**	**1990**	**2000**	**2010**	**2020**	**2023**
Libya	20 to 24	14.0 (5.5–27.9)	14.0 (5.3–27.9)	14.1 (5.5–27.5)	14.2 (5.7–29.3)	14.2 (5.7–29.7)
Libya	25 to 29	14.4 (5.6–28.5)	14.4 (5.4–28.5)	14.4 (5.6–28.1)	14.5 (5.8–29.9)	14.6 (5.8–30.3)
Libya	30 to 34	15.6 (6.2–30.5)	15.6 (5.9–30.5)	15.7 (6.2–30.1)	15.8 (6.4–31.9)	15.8 (6.4–32.5)
Libya	35 to 39	15.8 (6.3–30.9)	15.8 (6.0–30.9)	15.9 (6.2–30.5)	16.0 (6.5–32.2)	16.0 (6.5–32.8)
Libya	40 to 44	16.4 (6.5–31.8)	16.4 (6.3–31.9)	16.5 (6.5–31.5)	16.6 (6.8–33.2)	16.6 (6.8–33.9)
Libya	45 to 49	16.0 (6.4–31.2)	16.0 (6.1–31.3)	16.1 (6.4–30.9)	16.2 (6.6–32.7)	16.3 (6.6–33.3)
Libya	50 to 54	15.7 (6.2–30.7)	15.7 (6.0–30.8)	15.8 (6.2–30.4)	15.9 (6.5–32.2)	16.0 (6.5–32.8)
Libya	55 to 59	15.3 (6.0–30.0)	15.3 (5.8–30.1)	15.4 (6.0–29.7)	15.5 (6.3–31.5)	15.6 (6.2–32.1)
Libya	60 to 64	14.9 (5.8–29.3)	14.9 (5.6–29.4)	15.0 (5.9–29.0)	15.1 (6.1–30.9)	15.2 (6.2–31.4)
Libya	65 to 69	13.9 (5.4–27.7)	13.9 (5.2–27.7)	14.0 (5.4–27.4)	14.1 (5.7–29.1)	14.2 (5.7–29.9)
Libya	70 to 74	11.1 (4.2–22.8)	11.2 (4.1–22.8)	11.3 (4.2–22.6)	11.4 (4.5–24.1)	11.4 (4.5–24.6)
Libya	75 to 79	10.0 (3.8–20.8)	10.1 (3.6–20.8)	10.2 (3.8–20.6)	10.3 (4.0–22.0)	10.3 (3.9–22.7)
Libya	80 to 84	7.1 (2.6–15.1)	7.1 (2.5–15.2)	7.2 (2.6–15.0)	7.3 (2.7–16.1)	7.3 (2.8–16.5)
Libya	85 to 89	6.4 (2.3–13.7)	6.4 (2.2–13.7)	6.5 (2.3–13.6)	6.5 (2.5–14.6)	6.6 (2.5–15.0)
Libya	90 to 94	5.8 (2.1–12.5)	5.8 (2.0–12.5)	5.9 (2.1–12.3)	5.9 (2.2–13.3)	6.0 (2.2–13.6)

Supplementary Table S11: Prevalence of male SVAC by age and location for 1990, 2000, 2010, 2020, and 2023						
**Location**	**Age Range**	**1990**	**2000**	**2010**	**2020**	**2023**
Libya	95 plus	5.7 (2.1–12.4)	5.8 (2.0–12.4)	5.8 (2.1–12.2)	5.9 (2.2–13.2)	5.9 (2.2–13.5)
Libya	Age-standardized	14.7 (5.8–28.9)	14.7 (5.6–28.9)	14.7 (5.8–28.6)	14.9 (6.0–30.3)	14.9 (6.0–30.8)
Libya	All age	14.9 (5.9–29.3)	14.8 (5.6–29.2)	15.0 (5.9–29.0)	15.3 (6.2–31.0)	15.3 (6.2–31.6)
Morocco	20 to 24	14.0 (5.5–27.9)	14.0 (5.3–27.9)	14.1 (5.5–27.5)	14.2 (5.7–29.3)	14.2 (5.7–29.7)
Morocco	25 to 29	14.4 (5.6–28.5)	14.4 (5.4–28.5)	14.4 (5.6–28.1)	14.5 (5.8–29.9)	14.6 (5.8–30.3)
Morocco	30 to 34	15.6 (6.2–30.5)	15.6 (5.9–30.5)	15.7 (6.2–30.1)	15.8 (6.4–31.9)	15.8 (6.4–32.5)
Morocco	35 to 39	15.8 (6.3–30.9)	15.8 (6.0–30.9)	15.9 (6.2–30.5)	16.0 (6.5–32.2)	16.0 (6.5–32.8)
Morocco	40 to 44	16.4 (6.5–31.8)	16.4 (6.3–31.9)	16.5 (6.5–31.5)	16.6 (6.8–33.2)	16.6 (6.8–33.9)
Morocco	45 to 49	16.0 (6.4–31.2)	16.0 (6.1–31.3)	16.1 (6.4–30.9)	16.2 (6.6–32.7)	16.3 (6.6–33.3)
Morocco	50 to 54	15.7 (6.2–30.7)	15.7 (6.0–30.8)	15.8 (6.2–30.4)	15.9 (6.5–32.2)	16.0 (6.5–32.8)
Morocco	55 to 59	15.3 (6.0–30.0)	15.3 (5.8–30.1)	15.4 (6.0–29.7)	15.5 (6.3–31.5)	15.6 (6.2–32.1)
Morocco	60 to 64	14.9 (5.8–29.3)	14.9 (5.6–29.4)	15.0 (5.9–29.0)	15.1 (6.1–30.9)	15.2 (6.2–31.4)
Morocco	65 to 69	13.9 (5.4–27.7)	13.9 (5.2–27.7)	14.0 (5.4–27.4)	14.1 (5.7–29.1)	14.2 (5.7–29.9)
Morocco	70 to 74	11.1 (4.2–22.8)	11.2 (4.1–22.8)	11.3 (4.2–22.6)	11.4 (4.5–24.1)	11.4 (4.5–24.6)
Morocco	75 to 79	10.0 (3.8–20.8)	10.1 (3.6–20.8)	10.2 (3.8–20.6)	10.3 (4.0–22.0)	10.3 (3.9–22.7)

Supplementary Table S11: Prevalence of male SVAC by age and location for 1990, 2000, 2010, 2020, and 2023						
**Location**	**Age Range**	**1990**	**2000**	**2010**	**2020**	**2023**
Morocco	80 to 84	7.1 (2.6–15.1)	7.1 (2.5–15.2)	7.2 (2.6–15.0)	7.3 (2.7–16.1)	7.3 (2.8–16.5)
Morocco	85 to 89	6.4 (2.3–13.7)	6.4 (2.2–13.7)	6.5 (2.3–13.6)	6.5 (2.5–14.6)	6.6 (2.5–15.0)
Morocco	90 to 94	5.8 (2.1–12.5)	5.8 (2.0–12.5)	5.9 (2.1–12.3)	5.9 (2.2–13.3)	6.0 (2.2–13.6)
Morocco	95 plus	5.7 (2.1–12.4)	5.8 (2.0–12.4)	5.8 (2.1–12.2)	5.9 (2.2–13.2)	5.9 (2.2–13.5)
Morocco	Age-standardized	14.7 (5.8–28.9)	14.7 (5.6–28.9)	14.7 (5.8–28.6)	14.9 (6.0–30.3)	14.9 (6.0–30.8)
Morocco	All age	14.8 (5.8–29.1)	14.8 (5.6–29.2)	14.9 (5.8–28.9)	15.0 (6.1–30.7)	15.1 (6.1–31.2)
Oman	20 to 24	14.0 (5.5–27.9)	14.0 (5.3–27.9)	14.1 (5.5–27.5)	14.2 (5.7–29.3)	14.2 (5.7–29.7)
Oman	25 to 29	14.4 (5.6–28.5)	14.4 (5.4–28.5)	14.4 (5.6–28.1)	14.5 (5.8–29.9)	14.6 (5.8–30.3)
Oman	30 to 34	15.6 (6.2–30.5)	15.6 (5.9–30.5)	15.7 (6.2–30.1)	15.8 (6.4–31.9)	15.8 (6.4–32.5)
Oman	35 to 39	15.8 (6.3–30.9)	15.8 (6.0–30.9)	15.9 (6.2–30.5)	16.0 (6.5–32.2)	16.0 (6.5–32.8)
Oman	40 to 44	16.4 (6.5–31.8)	16.4 (6.3–31.9)	16.5 (6.5–31.5)	16.6 (6.8–33.2)	16.6 (6.8–33.9)
Oman	45 to 49	16.0 (6.4–31.2)	16.0 (6.1–31.3)	16.1 (6.4–30.9)	16.2 (6.6–32.7)	16.3 (6.6–33.3)
Oman	50 to 54	15.7 (6.2–30.7)	15.7 (6.0–30.8)	15.8 (6.2–30.4)	15.9 (6.5–32.2)	16.0 (6.5–32.8)
Oman	55 to 59	15.3 (6.0–30.0)	15.3 (5.8–30.1)	15.4 (6.0–29.7)	15.5 (6.3–31.5)	15.6 (6.2–32.1)
Oman	60 to 64	14.9 (5.8–29.3)	14.9 (5.6–29.4)	15.0 (5.9–29.0)	15.1 (6.1–30.9)	15.2 (6.2–31.4)

Supplementary Table S11: Prevalence of male SVAC by age and location for 1990, 2000, 2010, 2020, and 2023						
**Location**	**Age Range**	**1990**	**2000**	**2010**	**2020**	**2023**
Oman	65 to 69	13.9 (5.4–27.7)	13.9 (5.2–27.7)	14.0 (5.4–27.4)	14.1 (5.7–29.1)	14.2 (5.7–29.9)
Oman	70 to 74	11.1 (4.2–22.8)	11.2 (4.1–22.8)	11.3 (4.2–22.6)	11.4 (4.5–24.1)	11.4 (4.5–24.6)
Oman	75 to 79	10.0 (3.8–20.8)	10.1 (3.6–20.8)	10.2 (3.8–20.6)	10.3 (4.0–22.0)	10.3 (3.9–22.7)
Oman	80 to 84	7.1 (2.6–15.1)	7.1 (2.5–15.2)	7.2 (2.6–15.0)	7.3 (2.7–16.1)	7.3 (2.8–16.5)
Oman	85 to 89	6.4 (2.3–13.7)	6.4 (2.2–13.7)	6.5 (2.3–13.6)	6.5 (2.5–14.6)	6.6 (2.5–15.0)
Oman	90 to 94	5.8 (2.1–12.5)	5.8 (2.0–12.5)	5.9 (2.1–12.3)	5.9 (2.2–13.3)	6.0 (2.2–13.6)
Oman	95 plus	5.7 (2.1–12.4)	5.8 (2.0–12.4)	5.8 (2.1–12.2)	5.9 (2.2–13.2)	5.9 (2.2–13.5)
Oman	Age-standardized	14.7 (5.8–28.9)	14.7 (5.6–28.9)	14.7 (5.8–28.6)	14.9 (6.0–30.3)	14.9 (6.0–30.8)
Oman	All age	15.1 (6.0–29.7)	15.1 (5.7–29.8)	15.1 (5.9–29.2)	15.4 (6.3–31.4)	15.6 (6.3–32.1)
Palestine	20 to 24	14.0 (5.5–27.9)	14.0 (5.3–27.9)	14.1 (5.5–27.5)	14.2 (5.7–29.3)	14.2 (5.7–29.7)
Palestine	25 to 29	14.4 (5.6–28.5)	14.4 (5.4–28.5)	14.4 (5.6–28.1)	14.5 (5.8–29.9)	14.6 (5.8–30.3)
Palestine	30 to 34	15.6 (6.2–30.5)	15.6 (5.9–30.5)	15.7 (6.2–30.1)	15.8 (6.4–31.9)	15.8 (6.4–32.5)
Palestine	35 to 39	15.8 (6.3–30.9)	15.8 (6.0–30.9)	15.9 (6.2–30.5)	16.0 (6.5–32.2)	16.0 (6.5–32.8)
Palestine	40 to 44	16.4 (6.5–31.8)	16.4 (6.3–31.9)	16.5 (6.5–31.5)	16.6 (6.8–33.2)	16.6 (6.8–33.9)
Palestine	45 to 49	16.0 (6.4–31.2)	16.0 (6.1–31.3)	16.1 (6.4–30.9)	16.2 (6.6–32.7)	16.3 (6.6–33.3)

Supplementary Table S11: Prevalence of male SVAC by age and location for 1990, 2000, 2010, 2020, and 2023						
**Location**	**Age Range**	**1990**	**2000**	**2010**	**2020**	**2023**
Palestine	50 to 54	15.7 (6.2–30.7)	15.7 (6.0–30.8)	15.8 (6.2–30.4)	15.9 (6.5–32.2)	16.0 (6.5–32.8)
Palestine	55 to 59	15.3 (6.0–30.0)	15.3 (5.8–30.1)	15.4 (6.0–29.7)	15.5 (6.3–31.5)	15.6 (6.2–32.1)
Palestine	60 to 64	14.9 (5.8–29.3)	14.9 (5.6–29.4)	15.0 (5.9–29.0)	15.1 (6.1–30.9)	15.2 (6.2–31.4)
Palestine	65 to 69	13.9 (5.4–27.7)	13.9 (5.2–27.7)	14.0 (5.4–27.4)	14.1 (5.7–29.1)	14.2 (5.7–29.9)
Palestine	70 to 74	11.1 (4.2–22.8)	11.2 (4.1–22.8)	11.3 (4.2–22.6)	11.4 (4.5–24.1)	11.4 (4.5–24.6)
Palestine	75 to 79	10.0 (3.8–20.8)	10.1 (3.6–20.8)	10.2 (3.8–20.6)	10.3 (4.0–22.0)	10.3 (3.9–22.7)
Palestine	80 to 84	7.1 (2.6–15.1)	7.1 (2.5–15.2)	7.2 (2.6–15.0)	7.3 (2.7–16.1)	7.3 (2.8–16.5)
Palestine	85 to 89	6.4 (2.3–13.7)	6.4 (2.2–13.7)	6.5 (2.3–13.6)	6.5 (2.5–14.6)	6.6 (2.5–15.0)
Palestine	90 to 94	5.8 (2.1–12.5)	5.8 (2.0–12.5)	5.9 (2.1–12.3)	5.9 (2.2–13.3)	6.0 (2.2–13.6)
Palestine	95 plus	5.7 (2.1–12.4)	5.8 (2.0–12.4)	5.8 (2.1–12.2)	5.9 (2.2–13.2)	5.9 (2.2–13.5)
Palestine	Age-standardized	14.7 (5.8–28.9)	14.7 (5.6–28.9)	14.7 (5.8–28.6)	14.9 (6.0–30.3)	14.9 (6.0–30.8)
Palestine	All age	14.7 (5.8–29.0)	14.9 (5.6–29.3)	15.0 (5.9–29.0)	15.1 (6.1–30.8)	15.2 (6.1–31.3)
Qatar	20 to 24	14.0 (5.5–27.9)	14.0 (5.3–27.9)	14.1 (5.5–27.5)	14.2 (5.7–29.3)	14.2 (5.7–29.7)
Qatar	25 to 29	14.4 (5.6–28.5)	14.4 (5.4–28.5)	14.4 (5.6–28.1)	14.5 (5.8–29.9)	14.6 (5.8–30.3)
Qatar	30 to 34	15.6 (6.2–30.5)	15.6 (5.9–30.5)	15.7 (6.2–30.1)	15.8 (6.4–31.9)	15.8 (6.4–32.5)

Supplementary Table S11: Prevalence of male SVAC by age and location for 1990, 2000, 2010, 2020, and 2023						
**Location**	**Age Range**	**1990**	**2000**	**2010**	**2020**	**2023**
Qatar	35 to 39	15.8 (6.3–30.9)	15.8 (6.0–30.9)	15.9 (6.2–30.5)	16.0 (6.5–32.2)	16.0 (6.5–32.8)
Qatar	40 to 44	16.4 (6.5–31.8)	16.4 (6.3–31.9)	16.5 (6.5–31.5)	16.6 (6.8–33.2)	16.6 (6.8–33.9)
Qatar	45 to 49	16.0 (6.4–31.2)	16.0 (6.1–31.3)	16.1 (6.4–30.9)	16.2 (6.6–32.7)	16.3 (6.6–33.3)
Qatar	50 to 54	15.7 (6.2–30.7)	15.7 (6.0–30.8)	15.8 (6.2–30.4)	15.9 (6.5–32.2)	16.0 (6.5–32.8)
Qatar	55 to 59	15.3 (6.0–30.0)	15.3 (5.8–30.1)	15.4 (6.0–29.7)	15.5 (6.3–31.5)	15.6 (6.2–32.1)
Qatar	60 to 64	14.9 (5.8–29.3)	14.9 (5.6–29.4)	15.0 (5.9–29.0)	15.1 (6.1–30.9)	15.2 (6.2–31.4)
Qatar	65 to 69	13.9 (5.4–27.7)	13.9 (5.2–27.7)	14.0 (5.4–27.4)	14.1 (5.7–29.1)	14.2 (5.7–29.9)
Qatar	70 to 74	11.1 (4.2–22.8)	11.2 (4.1–22.8)	11.3 (4.2–22.6)	11.4 (4.5–24.1)	11.4 (4.5–24.6)
Qatar	75 to 79	10.0 (3.8–20.8)	10.1 (3.6–20.8)	10.2 (3.8–20.6)	10.3 (4.0–22.0)	10.3 (3.9–22.7)
Qatar	80 to 84	7.1 (2.6–15.1)	7.1 (2.5–15.2)	7.2 (2.6–15.0)	7.3 (2.7–16.1)	7.3 (2.8–16.5)
Qatar	85 to 89	6.4 (2.3–13.7)	6.4 (2.2–13.7)	6.5 (2.3–13.6)	6.5 (2.5–14.6)	6.6 (2.5–15.0)
Qatar	90 to 94	5.8 (2.1–12.5)	5.8 (2.0–12.5)	5.9 (2.1–12.3)	5.9 (2.2–13.3)	6.0 (2.2–13.6)
Qatar	95 plus	5.7 (2.1–12.4)	5.8 (2.0–12.4)	5.8 (2.1–12.2)	5.9 (2.2–13.2)	5.9 (2.2–13.5)
Qatar	Age-standardized	14.7 (5.8–28.9)	14.7 (5.6–28.9)	14.7 (5.8–28.6)	14.9 (6.0–30.3)	14.9 (6.0–30.8)
Qatar	All age	15.3 (6.0–30.0)	15.4 (5.9–30.2)	15.4 (6.0–29.6)	15.5 (6.3–31.5)	15.7 (6.4–32.3)

Supplementary Table S11: Prevalence of male SVAC by age and location for 1990, 2000, 2010, 2020, and 2023						
**Location**	**Age Range**	**1990**	**2000**	**2010**	**2020**	**2023**
Saudi Arabia	20 to 24	14.0 (5.5–27.9)	14.0 (5.3–27.9)	14.1 (5.5–27.5)	14.2 (5.7–29.3)	14.2 (5.7–29.7)
Saudi Arabia	25 to 29	14.4 (5.6–28.5)	14.4 (5.4–28.5)	14.4 (5.6–28.1)	14.5 (5.8–29.9)	14.6 (5.8–30.3)
Saudi Arabia	30 to 34	15.6 (6.2–30.5)	15.6 (5.9–30.5)	15.7 (6.2–30.1)	15.8 (6.4–31.9)	15.8 (6.4–32.5)
Saudi Arabia	35 to 39	15.8 (6.3–30.9)	15.8 (6.0–30.9)	15.9 (6.2–30.5)	16.0 (6.5–32.2)	16.0 (6.5–32.8)
Saudi Arabia	40 to 44	16.4 (6.5–31.8)	16.4 (6.3–31.9)	16.5 (6.5–31.5)	16.6 (6.8–33.2)	16.6 (6.8–33.9)
Saudi Arabia	45 to 49	16.0 (6.4–31.2)	16.0 (6.1–31.3)	16.1 (6.4–30.9)	16.2 (6.6–32.7)	16.3 (6.6–33.3)
Saudi Arabia	50 to 54	15.7 (6.2–30.7)	15.7 (6.0–30.8)	15.8 (6.2–30.4)	15.9 (6.5–32.2)	16.0 (6.5–32.8)
Saudi Arabia	55 to 59	15.3 (6.0–30.0)	15.3 (5.8–30.1)	15.4 (6.0–29.7)	15.5 (6.3–31.5)	15.6 (6.2–32.1)
Saudi Arabia	60 to 64	14.9 (5.8–29.3)	14.9 (5.6–29.4)	15.0 (5.9–29.0)	15.1 (6.1–30.9)	15.2 (6.2–31.4)
Saudi Arabia	65 to 69	13.9 (5.4–27.7)	13.9 (5.2–27.7)	14.0 (5.4–27.4)	14.1 (5.7–29.1)	14.2 (5.7–29.9)
Saudi Arabia	70 to 74	11.1 (4.2–22.8)	11.2 (4.1–22.8)	11.3 (4.2–22.6)	11.4 (4.5–24.1)	11.4 (4.5–24.6)
Saudi Arabia	75 to 79	10.0 (3.8–20.8)	10.1 (3.6–20.8)	10.2 (3.8–20.6)	10.3 (4.0–22.0)	10.3 (3.9–22.7)
Saudi Arabia	80 to 84	7.1 (2.6–15.1)	7.1 (2.5–15.2)	7.2 (2.6–15.0)	7.3 (2.7–16.1)	7.3 (2.8–16.5)
Saudi Arabia	85 to 89	6.4 (2.3–13.7)	6.4 (2.2–13.7)	6.5 (2.3–13.6)	6.5 (2.5–14.6)	6.6 (2.5–15.0)
Saudi Arabia	90 to 94	5.8 (2.1–12.5)	5.8 (2.0–12.5)	5.9 (2.1–12.3)	5.9 (2.2–13.3)	6.0 (2.2–13.6)

Supplementary Table S11: Prevalence of male SVAC by age and location for 1990, 2000, 2010, 2020, and 2023						
**Location**	**Age Range**	**1990**	**2000**	**2010**	**2020**	**2023**
Saudi Arabia	95 plus	5.7 (2.1–12.4)	5.8 (2.0–12.4)	5.8 (2.1–12.2)	5.9 (2.2–13.2)	5.9 (2.2–13.5)
Saudi Arabia	Age-standardized	14.7 (5.8–28.9)	14.7 (5.6–28.9)	14.7 (5.8–28.6)	14.9 (6.0–30.3)	14.9 (6.0–30.8)
Saudi Arabia	All age	15.0 (5.9–29.6)	15.1 (5.7–29.7)	15.2 (6.0–29.4)	15.4 (6.3–31.3)	15.5 (6.3–31.9)
Sudan	20 to 24	14.0 (5.5–27.9)	14.0 (5.3–27.9)	14.1 (5.5–27.5)	14.2 (5.7–29.3)	14.2 (5.7–29.7)
Sudan	25 to 29	14.4 (5.6–28.5)	14.4 (5.4–28.5)	14.4 (5.6–28.1)	14.5 (5.8–29.9)	14.6 (5.8–30.3)
Sudan	30 to 34	15.6 (6.2–30.5)	15.6 (5.9–30.5)	15.7 (6.2–30.1)	15.8 (6.4–31.9)	15.8 (6.4–32.5)
Sudan	35 to 39	15.8 (6.3–30.9)	15.8 (6.0–30.9)	15.9 (6.2–30.5)	16.0 (6.5–32.2)	16.0 (6.5–32.8)
Sudan	40 to 44	16.4 (6.5–31.8)	16.4 (6.3–31.9)	16.5 (6.5–31.5)	16.6 (6.8–33.2)	16.6 (6.8–33.9)
Sudan	45 to 49	16.0 (6.4–31.2)	16.0 (6.1–31.3)	16.1 (6.4–30.9)	16.2 (6.6–32.7)	16.3 (6.6–33.3)
Sudan	50 to 54	15.7 (6.2–30.7)	15.7 (6.0–30.8)	15.8 (6.2–30.4)	15.9 (6.5–32.2)	16.0 (6.5–32.8)
Sudan	55 to 59	15.3 (6.0–30.0)	15.3 (5.8–30.1)	15.4 (6.0–29.7)	15.5 (6.3–31.5)	15.6 (6.2–32.1)
Sudan	60 to 64	14.9 (5.8–29.3)	14.9 (5.6–29.4)	15.0 (5.9–29.0)	15.1 (6.1–30.9)	15.2 (6.2–31.4)
Sudan	65 to 69	13.9 (5.4–27.7)	13.9 (5.2–27.7)	14.0 (5.4–27.4)	14.1 (5.7–29.1)	14.2 (5.7–29.9)
Sudan	70 to 74	11.1 (4.2–22.8)	11.2 (4.1–22.8)	11.3 (4.2–22.6)	11.4 (4.5–24.1)	11.4 (4.5–24.6)
Sudan	75 to 79	10.0 (3.8–20.8)	10.1 (3.6–20.8)	10.2 (3.8–20.6)	10.3 (4.0–22.0)	10.3 (3.9–22.7)

Supplementary Table S11: Prevalence of male SVAC by age and location for 1990, 2000, 2010, 2020, and 2023						
**Location**	**Age Range**	**1990**	**2000**	**2010**	**2020**	**2023**
Sudan	80 to 84	7.1 (2.6–15.1)	7.1 (2.5–15.2)	7.2 (2.6–15.0)	7.3 (2.7–16.1)	7.3 (2.8–16.5)
Sudan	85 to 89	6.4 (2.3–13.7)	6.4 (2.2–13.7)	6.5 (2.3–13.6)	6.5 (2.5–14.6)	6.6 (2.5–15.0)
Sudan	90 to 94	5.8 (2.1–12.5)	5.8 (2.0–12.5)	5.9 (2.1–12.3)	5.9 (2.2–13.3)	6.0 (2.2–13.6)
Sudan	95 plus	5.7 (2.1–12.4)	5.8 (2.0–12.4)	5.8 (2.1–12.2)	5.9 (2.2–13.2)	5.9 (2.2–13.5)
Sudan	Age-standardized	14.7 (5.8–28.9)	14.7 (5.6–28.9)	14.7 (5.8–28.6)	14.9 (6.0–30.3)	14.9 (6.0–30.8)
Sudan	All age	14.8 (5.8–29.2)	14.8 (5.6–29.1)	14.9 (5.8–28.8)	15.0 (6.1–30.6)	15.1 (6.1–31.1)
Syria	20 to 24	14.0 (5.5–27.9)	14.0 (5.3–27.9)	14.1 (5.5–27.5)	14.2 (5.7–29.3)	14.2 (5.7–29.7)
Syria	25 to 29	14.4 (5.6–28.5)	14.4 (5.4–28.5)	14.4 (5.6–28.1)	14.5 (5.8–29.9)	14.6 (5.8–30.3)
Syria	30 to 34	15.6 (6.2–30.5)	15.6 (5.9–30.5)	15.7 (6.2–30.1)	15.8 (6.4–31.9)	15.8 (6.4–32.5)
Syria	35 to 39	15.8 (6.3–30.9)	15.8 (6.0–30.9)	15.9 (6.2–30.5)	16.0 (6.5–32.2)	16.0 (6.5–32.8)
Syria	40 to 44	16.4 (6.5–31.8)	16.4 (6.3–31.9)	16.5 (6.5–31.5)	16.6 (6.8–33.2)	16.6 (6.8–33.9)
Syria	45 to 49	16.0 (6.4–31.2)	16.0 (6.1–31.3)	16.1 (6.4–30.9)	16.2 (6.6–32.7)	16.3 (6.6–33.3)
Syria	50 to 54	15.7 (6.2–30.7)	15.7 (6.0–30.8)	15.8 (6.2–30.4)	15.9 (6.5–32.2)	16.0 (6.5–32.8)
Syria	55 to 59	15.3 (6.0–30.0)	15.3 (5.8–30.1)	15.4 (6.0–29.7)	15.5 (6.3–31.5)	15.6 (6.2–32.1)
Syria	60 to 64	14.9 (5.8–29.3)	14.9 (5.6–29.4)	15.0 (5.9–29.0)	15.1 (6.1–30.9)	15.2 (6.2–31.4)

Supplementary Table S11: Prevalence of male SVAC by age and location for 1990, 2000, 2010, 2020, and 2023						
**Location**	**Age Range**	**1990**	**2000**	**2010**	**2020**	**2023**
Syria	65 to 69	13.9 (5.4–27.7)	13.9 (5.2–27.7)	14.0 (5.4–27.4)	14.1 (5.7–29.1)	14.2 (5.7–29.9)
Syria	70 to 74	11.1 (4.2–22.8)	11.2 (4.1–22.8)	11.3 (4.2–22.6)	11.4 (4.5–24.1)	11.4 (4.5–24.6)
Syria	75 to 79	10.0 (3.8–20.8)	10.1 (3.6–20.8)	10.2 (3.8–20.6)	10.3 (4.0–22.0)	10.3 (3.9–22.7)
Syria	80 to 84	7.1 (2.6–15.1)	7.1 (2.5–15.2)	7.2 (2.6–15.0)	7.3 (2.7–16.1)	7.3 (2.8–16.5)
Syria	85 to 89	6.4 (2.3–13.7)	6.4 (2.2–13.7)	6.5 (2.3–13.6)	6.5 (2.5–14.6)	6.6 (2.5–15.0)
Syria	90 to 94	5.8 (2.1–12.5)	5.8 (2.0–12.5)	5.9 (2.1–12.3)	5.9 (2.2–13.3)	6.0 (2.2–13.6)
Syria	95 plus	5.7 (2.1–12.4)	5.8 (2.0–12.4)	5.8 (2.1–12.2)	5.9 (2.2–13.2)	5.9 (2.2–13.5)
Syria	Age-standardized	14.7 (5.8–28.9)	14.7 (5.6–28.9)	14.7 (5.8–28.6)	14.9 (6.0–30.3)	14.9 (6.0–30.8)
Syria	All age	14.8 (5.8–29.2)	14.9 (5.6–29.3)	15.0 (5.9–29.0)	15.2 (6.2–30.9)	15.1 (6.1–31.3)
Tunisia	20 to 24	14.0 (5.5–27.9)	14.0 (5.3–27.9)	14.1 (5.5–27.5)	14.2 (5.7–29.3)	14.2 (5.7–29.7)
Tunisia	25 to 29	14.4 (5.6–28.5)	14.4 (5.4–28.5)	14.4 (5.6–28.1)	14.5 (5.8–29.9)	14.6 (5.8–30.3)
Tunisia	30 to 34	15.6 (6.2–30.5)	15.6 (5.9–30.5)	15.7 (6.2–30.1)	15.8 (6.4–31.9)	15.8 (6.4–32.5)
Tunisia	35 to 39	15.8 (6.3–30.9)	15.8 (6.0–30.9)	15.9 (6.2–30.5)	16.0 (6.5–32.2)	16.0 (6.5–32.8)
Tunisia	40 to 44	16.4 (6.5–31.8)	16.4 (6.3–31.9)	16.5 (6.5–31.5)	16.6 (6.8–33.2)	16.6 (6.8–33.9)
Tunisia	45 to 49	16.0 (6.4–31.2)	16.0 (6.1–31.3)	16.1 (6.4–30.9)	16.2 (6.6–32.7)	16.3 (6.6–33.3)

Supplementary Table S11: Prevalence of male SVAC by age and location for 1990, 2000, 2010, 2020, and 2023						
**Location**	**Age Range**	**1990**	**2000**	**2010**	**2020**	**2023**
Tunisia	50 to 54	15.7 (6.2–30.7)	15.7 (6.0–30.8)	15.8 (6.2–30.4)	15.9 (6.5–32.2)	16.0 (6.5–32.8)
Tunisia	55 to 59	15.3 (6.0–30.0)	15.3 (5.8–30.1)	15.4 (6.0–29.7)	15.5 (6.3–31.5)	15.6 (6.2–32.1)
Tunisia	60 to 64	14.9 (5.8–29.3)	14.9 (5.6–29.4)	15.0 (5.9–29.0)	15.1 (6.1–30.9)	15.2 (6.2–31.4)
Tunisia	65 to 69	13.9 (5.4–27.7)	13.9 (5.2–27.7)	14.0 (5.4–27.4)	14.1 (5.7–29.1)	14.2 (5.7–29.9)
Tunisia	70 to 74	11.1 (4.2–22.8)	11.2 (4.1–22.8)	11.3 (4.2–22.6)	11.4 (4.5–24.1)	11.4 (4.5–24.6)
Tunisia	75 to 79	10.0 (3.8–20.8)	10.1 (3.6–20.8)	10.2 (3.8–20.6)	10.3 (4.0–22.0)	10.3 (3.9–22.7)
Tunisia	80 to 84	7.1 (2.6–15.1)	7.1 (2.5–15.2)	7.2 (2.6–15.0)	7.3 (2.7–16.1)	7.3 (2.8–16.5)
Tunisia	85 to 89	6.4 (2.3–13.7)	6.4 (2.2–13.7)	6.5 (2.3–13.6)	6.5 (2.5–14.6)	6.6 (2.5–15.0)
Tunisia	90 to 94	5.8 (2.1–12.5)	5.8 (2.0–12.5)	5.9 (2.1–12.3)	5.9 (2.2–13.3)	6.0 (2.2–13.6)
Tunisia	95 plus	5.7 (2.1–12.4)	5.8 (2.0–12.4)	5.8 (2.1–12.2)	5.9 (2.2–13.2)	5.9 (2.2–13.5)
Tunisia	Age-standardized	14.7 (5.8–28.9)	14.7 (5.6–28.9)	14.7 (5.8–28.6)	14.9 (6.0–30.3)	14.9 (6.0–30.8)
Tunisia	All age	14.8 (5.8–29.1)	14.8 (5.6–29.2)	14.9 (5.8–28.8)	15.0 (6.1–30.6)	15.1 (6.1–31.1)
Türkiye	20 to 24	14.0 (5.5–27.9)	14.0 (5.3–27.9)	14.1 (5.5–27.5)	14.2 (5.7–29.3)	14.2 (5.7–29.7)
Türkiye	25 to 29	14.4 (5.6–28.5)	14.4 (5.4–28.5)	14.4 (5.6–28.1)	14.5 (5.8–29.9)	14.6 (5.8–30.3)
Türkiye	30 to 34	15.6 (6.2–30.5)	15.6 (5.9–30.5)	15.7 (6.2–30.1)	15.8 (6.4–31.9)	15.8 (6.4–32.5)

Supplementary Table S11: Prevalence of male SVAC by age and location for 1990, 2000, 2010, 2020, and 2023						
**Location**	**Age Range**	**1990**	**2000**	**2010**	**2020**	**2023**
Türkiye	35 to 39	15.8 (6.3–30.9)	15.8 (6.0–30.9)	15.9 (6.2–30.5)	16.0 (6.5–32.2)	16.0 (6.5–32.8)
Türkiye	40 to 44	16.4 (6.5–31.8)	16.4 (6.3–31.9)	16.5 (6.5–31.5)	16.6 (6.8–33.2)	16.6 (6.8–33.9)
Türkiye	45 to 49	16.0 (6.4–31.2)	16.0 (6.1–31.3)	16.1 (6.4–30.9)	16.2 (6.6–32.7)	16.3 (6.6–33.3)
Türkiye	50 to 54	15.7 (6.2–30.7)	15.7 (6.0–30.8)	15.8 (6.2–30.4)	15.9 (6.5–32.2)	16.0 (6.5–32.8)
Türkiye	55 to 59	15.3 (6.0–30.0)	15.3 (5.8–30.1)	15.4 (6.0–29.7)	15.5 (6.3–31.5)	15.6 (6.2–32.1)
Türkiye	60 to 64	14.9 (5.8–29.3)	14.9 (5.6–29.4)	15.0 (5.9–29.0)	15.1 (6.1–30.9)	15.2 (6.2–31.4)
Türkiye	65 to 69	13.9 (5.4–27.7)	13.9 (5.2–27.7)	14.0 (5.4–27.4)	14.1 (5.7–29.1)	14.2 (5.7–29.9)
Türkiye	70 to 74	11.1 (4.2–22.8)	11.2 (4.1–22.8)	11.3 (4.2–22.6)	11.4 (4.5–24.1)	11.4 (4.5–24.6)
Türkiye	75 to 79	10.0 (3.8–20.8)	10.1 (3.6–20.8)	10.2 (3.8–20.6)	10.3 (4.0–22.0)	10.3 (3.9–22.7)
Türkiye	80 to 84	7.1 (2.6–15.1)	7.1 (2.5–15.2)	7.2 (2.6–15.0)	7.3 (2.7–16.1)	7.3 (2.8–16.5)
Türkiye	85 to 89	6.4 (2.3–13.7)	6.4 (2.2–13.7)	6.5 (2.3–13.6)	6.5 (2.5–14.6)	6.6 (2.5–15.0)
Türkiye	90 to 94	5.8 (2.1–12.5)	5.8 (2.0–12.5)	5.9 (2.1–12.3)	5.9 (2.2–13.3)	6.0 (2.2–13.6)
Türkiye	95 plus	5.7 (2.1–12.4)	5.8 (2.0–12.4)	5.8 (2.1–12.2)	5.9 (2.2–13.2)	5.9 (2.2–13.5)
Türkiye	Age-standardized	14.7 (5.8–28.9)	14.7 (5.6–28.9)	14.7 (5.8–28.6)	14.9 (6.0–30.3)	14.9 (6.0–30.8)
Türkiye	All age	14.9 (5.9–29.4)	14.9 (5.7–29.4)	15.0 (5.9–29.0)	15.0 (6.1–30.6)	15.1 (6.1–31.1)

Supplementary Table S11: Prevalence of male SVAC by age and location for 1990, 2000, 2010, 2020, and 2023						
**Location**	**Age Range**	**1990**	**2000**	**2010**	**2020**	**2023**
United Arab Emirates	20 to 24	14.0 (5.5–27.9)	14.0 (5.3–27.9)	14.1 (5.5–27.5)	14.2 (5.7–29.3)	14.2 (5.7–29.7)
United Arab Emirates	25 to 29	14.4 (5.6–28.5)	14.4 (5.4–28.5)	14.4 (5.6–28.1)	14.5 (5.8–29.9)	14.6 (5.8–30.3)
United Arab Emirates	30 to 34	15.6 (6.2–30.5)	15.6 (5.9–30.5)	15.7 (6.2–30.1)	15.8 (6.4–31.9)	15.8 (6.4–32.5)
United Arab Emirates	35 to 39	15.8 (6.3–30.9)	15.8 (6.0–30.9)	15.9 (6.2–30.5)	16.0 (6.5–32.2)	16.0 (6.5–32.8)
United Arab Emirates	40 to 44	16.4 (6.5–31.8)	16.4 (6.3–31.9)	16.5 (6.5–31.5)	16.6 (6.8–33.2)	16.6 (6.8–33.9)
United Arab Emirates	45 to 49	16.0 (6.4–31.2)	16.0 (6.1–31.3)	16.1 (6.4–30.9)	16.2 (6.6–32.7)	16.3 (6.6–33.3)
United Arab Emirates	50 to 54	15.7 (6.2–30.7)	15.7 (6.0–30.8)	15.8 (6.2–30.4)	15.9 (6.5–32.2)	16.0 (6.5–32.8)
United Arab Emirates	55 to 59	15.3 (6.0–30.0)	15.3 (5.8–30.1)	15.4 (6.0–29.7)	15.5 (6.3–31.5)	15.6 (6.2–32.1)
United Arab Emirates	60 to 64	14.9 (5.8–29.3)	14.9 (5.6–29.4)	15.0 (5.9–29.0)	15.1 (6.1–30.9)	15.2 (6.2–31.4)
United Arab Emirates	65 to 69	13.9 (5.4–27.7)	13.9 (5.2–27.7)	14.0 (5.4–27.4)	14.1 (5.7–29.1)	14.2 (5.7–29.9)
United Arab Emirates	70 to 74	11.1 (4.2–22.8)	11.2 (4.1–22.8)	11.3 (4.2–22.6)	11.4 (4.5–24.1)	11.4 (4.5–24.6)
United Arab Emirates	75 to 79	10.0 (3.8–20.8)	10.1 (3.6–20.8)	10.2 (3.8–20.6)	10.3 (4.0–22.0)	10.3 (3.9–22.7)
United Arab Emirates	80 to 84	7.1 (2.6–15.1)	7.1 (2.5–15.2)	7.2 (2.6–15.0)	7.3 (2.7–16.1)	7.3 (2.8–16.5)
United Arab Emirates	85 to 89	6.4 (2.3–13.7)	6.4 (2.2–13.7)	6.5 (2.3–13.6)	6.5 (2.5–14.6)	6.6 (2.5–15.0)
United Arab Emirates	90 to 94	5.8 (2.1–12.5)	5.8 (2.0–12.5)	5.9 (2.1–12.3)	5.9 (2.2–13.3)	6.0 (2.2–13.6)

Supplementary Table S11: Prevalence of male SVAC by age and location for 1990, 2000, 2010, 2020, and 2023						
**Location**	**Age Range**	**1990**	**2000**	**2010**	**2020**	**2023**
United Arab Emirates	95 plus	5.7 (2.1–12.4)	5.8 (2.0–12.4)	5.8 (2.1–12.2)	5.9 (2.2–13.2)	5.9 (2.2–13.5)
United Arab Emirates	Age-standardized	14.7 (5.8–28.9)	14.7 (5.6–28.9)	14.7 (5.8–28.6)	14.9 (6.0–30.3)	14.9 (6.0–30.8)
United Arab Emirates	All age	15.3 (6.0–30.0)	15.3 (5.8–30.0)	15.2 (5.9–29.3)	15.9 (6.5–32.1)	15.9 (6.5–32.7)
Yemen	20 to 24	14.0 (5.5–27.9)	14.0 (5.3–27.9)	14.1 (5.5–27.5)	14.2 (5.7–29.3)	14.2 (5.7–29.7)
Yemen	25 to 29	14.4 (5.6–28.5)	14.4 (5.4–28.5)	14.4 (5.6–28.1)	14.5 (5.8–29.9)	14.6 (5.8–30.3)
Yemen	30 to 34	15.6 (6.2–30.5)	15.6 (5.9–30.5)	15.7 (6.2–30.1)	15.8 (6.4–31.9)	15.8 (6.4–32.5)
Yemen	35 to 39	15.8 (6.3–30.9)	15.8 (6.0–30.9)	15.9 (6.2–30.5)	16.0 (6.5–32.2)	16.0 (6.5–32.8)
Yemen	40 to 44	16.4 (6.5–31.8)	16.4 (6.3–31.9)	16.5 (6.5–31.5)	16.6 (6.8–33.2)	16.6 (6.8–33.9)
Yemen	45 to 49	16.0 (6.4–31.2)	16.0 (6.1–31.3)	16.1 (6.4–30.9)	16.2 (6.6–32.7)	16.3 (6.6–33.3)
Yemen	50 to 54	15.7 (6.2–30.7)	15.7 (6.0–30.8)	15.8 (6.2–30.4)	15.9 (6.5–32.2)	16.0 (6.5–32.8)
Yemen	55 to 59	15.3 (6.0–30.0)	15.3 (5.8–30.1)	15.4 (6.0–29.7)	15.5 (6.3–31.5)	15.6 (6.2–32.1)
Yemen	60 to 64	14.9 (5.8–29.3)	14.9 (5.6–29.4)	15.0 (5.9–29.0)	15.1 (6.1–30.9)	15.2 (6.2–31.4)
Yemen	65 to 69	13.9 (5.4–27.7)	13.9 (5.2–27.7)	14.0 (5.4–27.4)	14.1 (5.7–29.1)	14.2 (5.7–29.9)
Yemen	70 to 74	11.1 (4.2–22.8)	11.2 (4.1–22.8)	11.3 (4.2–22.6)	11.4 (4.5–24.1)	11.4 (4.5–24.6)
Yemen	75 to 79	10.0 (3.8–20.8)	10.1 (3.6–20.8)	10.2 (3.8–20.6)	10.3 (4.0–22.0)	10.3 (3.9–22.7)

Supplementary Table S11: Prevalence of male SVAC by age and location for 1990, 2000, 2010, 2020, and 2023						
**Location**	**Age Range**	**1990**	**2000**	**2010**	**2020**	**2023**
Yemen	80 to 84	7.1 (2.6–15.1)	7.1 (2.5–15.2)	7.2 (2.6–15.0)	7.3 (2.7–16.1)	7.3 (2.8–16.5)
Yemen	85 to 89	6.4 (2.3–13.7)	6.4 (2.2–13.7)	6.5 (2.3–13.6)	6.5 (2.5–14.6)	6.6 (2.5–15.0)
Yemen	90 to 94	5.8 (2.1–12.5)	5.8 (2.0–12.5)	5.9 (2.1–12.3)	5.9 (2.2–13.3)	6.0 (2.2–13.6)
Yemen	95 plus	5.7 (2.1–12.4)	5.8 (2.0–12.4)	5.8 (2.1–12.2)	5.9 (2.2–13.2)	5.9 (2.2–13.5)
Yemen	Age-standardized	14.7 (5.8–28.9)	14.7 (5.6–28.9)	14.7 (5.8–28.6)	14.9 (6.0–30.3)	14.9 (6.0–30.8)
Yemen	All age	15.0 (5.9–29.5)	14.9 (5.6–29.3)	14.9 (5.8–28.9)	15.1 (6.2–30.8)	15.2 (6.1–31.4)
South Asia	20 to 24	12.7 (4.7–25.8)	11.9 (5.0–22.7)	11.0 (5.4–19.2)	11.3 (5.4–20.8)	11.4 (5.3–21.7)
South Asia	25 to 29	13.1 (4.9–26.4)	12.3 (5.2–23.2)	11.4 (5.7–19.6)	11.6 (5.6–21.3)	11.7 (5.5–22.1)
South Asia	30 to 34	16.7 (6.5–31.7)	16.8 (8.1–28.9)	16.9 (11.3–24.3)	17.1 (11.7–24.9)	17.2 (11.1–25.7)
South Asia	35 to 39	16.3 (6.3–31.0)	16.1 (7.6–28.0)	15.8 (9.7–23.8)	16.0 (9.3–24.9)	16.1 (8.9–26.1)
South Asia	40 to 44	19.3 (7.7–35.8)	20.3 (10.2–34.1)	21.6 (13.9–31.0)	21.7 (14.1–31.4)	21.8 (13.5–32.6)
South Asia	45 to 49	18.2 (7.2–34.5)	18.6 (9.2–31.8)	19.4 (12.2–28.3)	19.5 (12.1–29.4)	19.6 (11.6–30.0)
South Asia	50 to 54	16.6 (6.0–33.0)	16.6 (6.5–31.9)	16.8 (6.8–31.6)	17.0 (6.6–34.6)	17.0 (6.6–35.3)
South Asia	55 to 59	16.1 (5.7–32.3)	16.1 (6.2–31.0)	16.2 (6.5–30.7)	16.5 (6.5–33.7)	16.6 (6.3–34.3)
South Asia	60 to 64	15.6 (5.6–31.3)	15.5 (6.0–30.0)	15.7 (6.2–29.8)	16.0 (6.3–33.1)	16.1 (6.0–33.6)

Supplementary Table S11: Prevalence of male SVAC by age and location for 1990, 2000, 2010, 2020, and 2023						
**Location**	**Age Range**	**1990**	**2000**	**2010**	**2020**	**2023**
South Asia	65 to 69	14.8 (5.2–29.8)	14.4 (5.5–28.3)	14.6 (5.8–28.1)	14.8 (5.7–30.7)	15.1 (5.7–32.0)
South Asia	70 to 74	12.2 (4.2–25.3)	11.8 (4.4–23.7)	11.8 (4.5–23.3)	11.9 (4.4–25.6)	12.0 (4.3–26.3)
South Asia	75 to 79	11.2 (3.9–23.4)	10.6 (3.9–21.7)	10.6 (4.0–21.2)	10.8 (3.9–23.7)	10.8 (3.9–24.2)
South Asia	80 to 84	8.0 (2.6–17.6)	7.8 (2.8–16.3)	7.6 (2.8–15.6)	7.7 (2.8–17.6)	7.8 (2.7–18.0)
South Asia	85 to 89	7.6 (2.5–16.7)	7.1 (2.5–15.1)	6.9 (2.5–14.2)	7.1 (2.5–16.2)	7.1 (2.4–16.5)
South Asia	90 to 94	6.9 (2.2–15.0)	6.4 (2.2–13.6)	6.3 (2.3–13.1)	6.5 (2.3–14.8)	6.5 (2.2–14.9)
South Asia	95 plus	6.7 (2.2–14.7)	6.5 (2.3–13.8)	6.3 (2.3–13.2)	6.7 (2.4–15.4)	6.7 (2.3–15.7)
South Asia	Age-standardized	15.3 (6.0–29.5)	15.2 (6.7–27.6)	15.2 (8.2–25.0)	15.4 (8.6–25.9)	15.5 (8.2–27.3)
South Asia	All age	15.4 (6.0–29.5)	15.3 (6.9–27.6)	15.2 (8.4–24.7)	15.4 (8.8–26.0)	15.6 (8.4–27.2)
South Asia	20 to 24	12.7 (4.7–25.8)	11.9 (5.0–22.7)	11.0 (5.4–19.2)	11.3 (5.4–20.8)	11.4 (5.3–21.7)
South Asia	25 to 29	13.1 (4.9–26.4)	12.3 (5.2–23.2)	11.4 (5.7–19.6)	11.6 (5.6–21.3)	11.7 (5.5–22.1)
South Asia	30 to 34	16.7 (6.5–31.7)	16.8 (8.1–28.9)	16.9 (11.3–24.3)	17.1 (11.7–24.9)	17.2 (11.1–25.7)
South Asia	35 to 39	16.3 (6.3–31.0)	16.1 (7.6–28.0)	15.8 (9.7–23.8)	16.0 (9.3–24.9)	16.1 (8.9–26.1)
South Asia	40 to 44	19.3 (7.7–35.8)	20.3 (10.2–34.1)	21.6 (13.9–31.0)	21.7 (14.1–31.4)	21.8 (13.5–32.6)
South Asia	45 to 49	18.2 (7.2–34.5)	18.6 (9.2–31.8)	19.4 (12.2–28.3)	19.5 (12.1–29.4)	19.6 (11.6–30.0)

Supplementary Table S11: Prevalence of male SVAC by age and location for 1990, 2000, 2010, 2020, and 2023						
**Location**	**Age Range**	**1990**	**2000**	**2010**	**2020**	**2023**
South Asia	50 to 54	16.6 (6.0–33.0)	16.6 (6.5–31.9)	16.8 (6.8–31.6)	17.0 (6.6–34.6)	17.0 (6.6–35.3)
South Asia	55 to 59	16.1 (5.7–32.3)	16.1 (6.2–31.0)	16.2 (6.5–30.7)	16.5 (6.5–33.7)	16.6 (6.3–34.3)
South Asia	60 to 64	15.6 (5.6–31.3)	15.5 (6.0–30.0)	15.7 (6.2–29.8)	16.0 (6.3–33.1)	16.1 (6.0–33.6)
South Asia	65 to 69	14.8 (5.2–29.8)	14.4 (5.5–28.3)	14.6 (5.8–28.1)	14.8 (5.7–30.7)	15.1 (5.7–32.0)
South Asia	70 to 74	12.2 (4.2–25.3)	11.8 (4.4–23.7)	11.8 (4.5–23.3)	11.9 (4.4–25.6)	12.0 (4.3–26.3)
South Asia	75 to 79	11.2 (3.9–23.4)	10.6 (3.9–21.7)	10.6 (4.0–21.2)	10.8 (3.9–23.7)	10.8 (3.9–24.2)
South Asia	80 to 84	8.0 (2.6–17.6)	7.8 (2.8–16.3)	7.6 (2.8–15.6)	7.7 (2.8–17.6)	7.8 (2.7–18.0)
South Asia	85 to 89	7.6 (2.5–16.7)	7.1 (2.5–15.1)	6.9 (2.5–14.2)	7.1 (2.5–16.2)	7.1 (2.4–16.5)
South Asia	90 to 94	6.9 (2.2–15.0)	6.4 (2.2–13.6)	6.3 (2.3–13.1)	6.5 (2.3–14.8)	6.5 (2.2–14.9)
South Asia	95 plus	6.7 (2.2–14.7)	6.5 (2.3–13.8)	6.3 (2.3–13.2)	6.7 (2.4–15.4)	6.7 (2.3–15.7)
South Asia	Age-standardized	15.3 (6.0–29.5)	15.2 (6.7–27.6)	15.2 (8.2–25.0)	15.4 (8.6–25.9)	15.5 (8.2–27.3)
South Asia	All age	15.4 (6.0–29.5)	15.3 (6.9–27.6)	15.2 (8.4–24.7)	15.4 (8.8–26.0)	15.6 (8.4–27.2)
Bangladesh	20 to 24	21.2 (8.7–39.1)	19.3 (9.5–32.5)	17.6 (10.4–26.6)	18.5 (9.6–30.8)	18.8 (9.0–32.2)
Bangladesh	25 to 29	22.0 (9.0–40.1)	19.9 (9.9–33.3)	18.1 (10.8–27.2)	19.1 (10.0–31.5)	19.4 (9.4–32.9)
Bangladesh	30 to 34	30.2 (13.9–51.1)	30.8 (17.5–46.4)	31.4 (23.8–40.0)	31.2 (19.9–45.4)	31.2 (18.9–47.3)

Supplementary Table S11: Prevalence of male SVAC by age and location for 1990, 2000, 2010, 2020, and 2023						
**Location**	**Age Range**	**1990**	**2000**	**2010**	**2020**	**2023**
Bangladesh	35 to 39	29.2 (13.2–50.4)	28.8 (16.1–44.2)	28.5 (20.8–37.3)	28.9 (16.9–42.2)	29.0 (16.4–44.0)
Bangladesh	40 to 44	35.9 (16.8–58.0)	38.2 (22.4–55.4)	40.8 (29.9–51.9)	40.2 (25.4–55.5)	40.0 (23.8–57.1)
Bangladesh	45 to 49	33.8 (15.4–55.3)	35.0 (20.2–51.7)	36.5 (26.9–46.7)	36.2 (22.3–51.0)	36.2 (20.9–52.9)
Bangladesh	50 to 54	30.0 (12.3–53.1)	29.9 (13.1–51.3)	30.0 (13.6–50.8)	30.2 (13.3–54.4)	30.3 (12.9–55.8)
Bangladesh	55 to 59	29.3 (11.9–52.8)	29.2 (12.7–50.3)	29.2 (13.1–49.8)	29.5 (12.9–54.0)	29.6 (12.6–54.7)
Bangladesh	60 to 64	28.5 (11.6–50.7)	28.4 (12.3–49.4)	28.5 (12.7–48.8)	28.7 (12.5–52.5)	28.8 (12.1–53.9)
Bangladesh	65 to 69	26.9 (10.7–48.7)	26.8 (11.4–47.2)	26.9 (11.8–46.7)	27.1 (11.6–50.3)	27.2 (11.2–51.1)
Bangladesh	70 to 74	22.3 (8.4–42.5)	22.2 (9.0–40.8)	22.2 (9.3–40.3)	22.5 (9.3–43.8)	22.5 (9.0–44.6)
Bangladesh	75 to 79	20.4 (7.6–39.1)	20.2 (8.1–37.9)	20.3 (8.3–37.4)	20.5 (8.4–40.9)	20.6 (8.3–41.6)
Bangladesh	80 to 84	15.0 (5.3–30.5)	14.9 (5.6–29.3)	14.9 (5.8–28.9)	15.1 (5.8–31.6)	15.2 (5.6–32.2)
Bangladesh	85 to 89	13.7 (4.7–28.3)	13.5 (5.0–27.0)	13.5 (5.2–26.6)	13.8 (5.2–29.5)	13.9 (5.0–30.2)
Bangladesh	90 to 94	12.5 (4.2–26.3)	12.3 (4.5–24.9)	12.3 (4.7–24.5)	12.5 (4.7–26.9)	12.6 (4.7–27.5)
Bangladesh	95 plus	12.4 (4.2–25.8)	12.2 (4.5–24.7)	12.2 (4.7–24.3)	12.4 (4.6–27.1)	12.5 (4.6–27.7)
Bangladesh	Age-standardized	27.5 (12.6–46.7)	27.3 (14.1–44.1)	27.3 (18.3–38.1)	27.6 (18.5–40.2)	27.7 (17.8–41.2)
Bangladesh	All age	27.0 (12.3–46.1)	27.1 (14.3–43.2)	27.2 (18.9–37.2)	27.7 (18.9–39.8)	27.8 (18.1–41.0)

Supplementary Table S11: Prevalence of male SVAC by age and location for 1990, 2000, 2010, 2020, and 2023						
**Location**	**Age Range**	**1990**	**2000**	**2010**	**2020**	**2023**
Bhutan	20 to 24	17.6 (6.3–34.8)	17.4 (6.7–33.5)	17.4 (7.0–33.0)	17.5 (6.8–36.0)	17.6 (6.7–37.1)
Bhutan	25 to 29	18.3 (6.6–35.9)	18.1 (7.1–34.6)	18.2 (7.3–34.1)	18.2 (7.2–37.1)	18.3 (7.2–37.8)
Bhutan	30 to 34	20.3 (7.5–39.1)	20.1 (8.0–37.8)	20.2 (8.3–37.3)	20.2 (8.2–40.4)	20.3 (7.8–41.6)
Bhutan	35 to 39	21.0 (7.8–40.1)	20.8 (8.4–38.8)	20.9 (8.6–38.3)	20.9 (8.4–41.0)	21.0 (8.1–41.7)
Bhutan	40 to 44	22.2 (8.4–42.0)	22.0 (8.9–40.6)	22.1 (9.2–40.1)	22.1 (9.2–42.8)	22.2 (9.0–44.1)
Bhutan	45 to 49	21.9 (8.2–41.6)	21.8 (8.8–40.2)	21.8 (9.1–39.7)	21.8 (8.9–42.4)	21.9 (8.7–43.6)
Bhutan	50 to 54	21.5 (8.0–41.5)	21.3 (8.6–39.5)	21.4 (8.9–39.1)	21.4 (8.7–42.2)	21.5 (8.3–43.5)
Bhutan	55 to 59	20.8 (7.7–40.0)	20.7 (8.3–38.6)	20.8 (8.6–38.1)	20.8 (8.3–41.2)	20.8 (8.0–42.0)
Bhutan	60 to 64	20.2 (7.5–39.1)	20.1 (8.0–37.7)	20.2 (8.3–37.2)	20.2 (8.2–40.4)	20.3 (7.9–41.8)
Bhutan	65 to 69	18.9 (6.9–37.3)	18.8 (7.4–35.7)	18.9 (7.6–35.2)	18.9 (7.5–37.9)	18.9 (7.2–38.5)
Bhutan	70 to 74	15.4 (5.4–31.2)	15.2 (5.8–30.0)	15.3 (6.0–29.5)	15.3 (5.8–31.7)	15.4 (5.6–32.3)
Bhutan	75 to 79	13.9 (4.8–28.6)	13.8 (5.2–27.5)	13.8 (5.3–27.1)	13.9 (5.2–29.7)	14.0 (5.0–30.4)
Bhutan	80 to 84	10.0 (3.4–21.4)	9.9 (3.6–20.5)	9.9 (3.7–20.2)	10.0 (3.7–22.3)	10.0 (3.6–22.9)
Bhutan	85 to 89	9.1 (2.9–19.9)	8.9 (3.2–18.7)	9.0 (3.3–18.4)	9.0 (3.2–20.4)	9.1 (3.1–21.4)
Bhutan	90 to 94	8.2 (2.7–18.2)	8.1 (2.9–17.1)	8.1 (3.0–16.8)	8.2 (2.9–18.7)	8.2 (2.8–19.5)

Supplementary Table S11: Prevalence of male SVAC by age and location for 1990, 2000, 2010, 2020, and 2023						
**Location**	**Age Range**	**1990**	**2000**	**2010**	**2020**	**2023**
Bhutan	95 plus	8.2 (2.7–17.9)	8.0 (2.8–16.9)	8.1 (2.9–16.6)	8.1 (2.9–18.3)	8.1 (2.8–19.0)
Bhutan	Age-standardized	19.5 (7.2–37.9)	19.3 (7.7–36.4)	19.4 (7.9–36.0)	19.4 (7.8–39.0)	19.5 (7.5–39.6)
Bhutan	All age	19.5 (7.2–37.9)	19.4 (7.7–36.5)	19.4 (7.9–36.0)	19.5 (7.9–39.3)	19.7 (7.6–40.0)
India	20 to 24	10.9 (3.9–22.7)	10.1 (4.1–19.6)	9.3 (4.6–16.4)	9.4 (4.6–17.5)	9.4 (4.3–18.3)
India	25 to 29	11.3 (4.0–23.4)	10.5 (4.3–20.2)	9.6 (4.8–16.8)	9.6 (4.7–17.8)	9.7 (4.5–18.7)
India	30 to 34	14.6 (5.4–29.0)	14.7 (6.8–26.4)	14.9 (9.2–22.3)	15.1 (9.2–23.0)	15.2 (8.5–24.3)
India	35 to 39	14.4 (5.2–28.8)	14.0 (6.5–25.4)	13.7 (8.2–21.0)	13.8 (8.2–21.9)	13.9 (7.6–22.6)
India	40 to 44	17.2 (6.5–33.2)	18.1 (8.8–31.1)	19.4 (12.6–27.8)	19.7 (12.4–28.9)	19.8 (11.6–30.1)
India	45 to 49	16.2 (6.0–31.8)	16.6 (8.0–29.2)	17.2 (10.9–25.2)	17.4 (10.9–26.1)	17.5 (10.1–27.3)
India	50 to 54	14.7 (5.1–30.0)	14.6 (5.5–28.8)	14.6 (5.7–28.4)	14.8 (5.6–31.4)	14.9 (5.5–32.1)
India	55 to 59	14.3 (4.9–29.2)	14.1 (5.3–28.0)	14.2 (5.5–27.6)	14.4 (5.4–30.4)	14.5 (5.3–31.0)
India	60 to 64	13.8 (4.7–28.3)	13.7 (5.1–27.3)	13.7 (5.3–26.9)	13.9 (5.3–29.7)	14.0 (5.0–30.2)
India	65 to 69	12.9 (4.3–26.7)	12.7 (4.7–25.6)	12.8 (4.9–25.2)	13.0 (4.8–28.0)	13.1 (4.7–28.7)
India	70 to 74	10.3 (3.4–22.2)	10.2 (3.7–20.9)	10.2 (3.8–20.6)	10.3 (3.7–23.1)	10.4 (3.6–23.6)
India	75 to 79	9.3 (3.1–19.9)	9.1 (3.3–19.0)	9.1 (3.4–18.7)	9.3 (3.3–21.0)	9.4 (3.3–21.6)

Supplementary Table S11: Prevalence of male SVAC by age and location for 1990, 2000, 2010, 2020, and 2023						
**Location**	**Age Range**	**1990**	**2000**	**2010**	**2020**	**2023**
India	80 to 84	6.5 (2.1–14.8)	6.4 (2.2–13.8)	6.4 (2.3–13.5)	6.6 (2.3–15.3)	6.6 (2.2–15.7)
India	85 to 89	5.9 (1.9–13.5)	5.8 (2.0–12.5)	5.8 (2.1–12.2)	5.9 (2.0–13.9)	6.0 (2.0–14.3)
India	90 to 94	5.3 (1.7–11.9)	5.2 (1.8–11.3)	5.2 (1.9–11.1)	5.3 (1.8–12.4)	5.4 (1.8–12.7)
India	95 plus	5.3 (1.7–11.9)	5.2 (1.8–11.2)	5.2 (1.8–11.0)	5.3 (1.8–12.4)	5.3 (1.8–12.9)
India	Age-standardized	13.4 (5.0–26.7)	13.3 (5.8–24.7)	13.2 (7.1–22.1)	13.4 (7.5–23.0)	13.5 (7.0–23.8)
India	All age	13.5 (5.0–26.9)	13.4 (5.9–24.7)	13.2 (7.2–21.6)	13.5 (7.6–22.8)	13.6 (7.2–23.6)
Nepal	20 to 24	17.6 (6.3–34.8)	17.4 (6.7–33.5)	17.4 (7.0–33.0)	17.5 (6.8–36.0)	17.6 (6.7–37.1)
Nepal	25 to 29	18.3 (6.6–35.9)	18.1 (7.1–34.6)	18.2 (7.3–34.1)	18.2 (7.2–37.1)	18.3 (7.2–37.8)
Nepal	30 to 34	20.3 (7.5–39.1)	20.1 (8.0–37.8)	20.2 (8.3–37.3)	20.2 (8.2–40.4)	20.3 (7.8–41.6)
Nepal	35 to 39	21.0 (7.8–40.1)	20.8 (8.4–38.8)	20.9 (8.6–38.3)	20.9 (8.4–41.0)	21.0 (8.1–41.7)
Nepal	40 to 44	22.2 (8.4–42.0)	22.0 (8.9–40.6)	22.1 (9.2–40.1)	22.1 (9.2–42.8)	22.2 (9.0–44.1)
Nepal	45 to 49	21.9 (8.2–41.6)	21.8 (8.8–40.2)	21.8 (9.1–39.7)	21.8 (8.9–42.4)	21.9 (8.7–43.6)
Nepal	50 to 54	21.5 (8.0–41.5)	21.3 (8.6–39.5)	21.4 (8.9–39.1)	21.4 (8.7–42.2)	21.5 (8.3–43.5)
Nepal	55 to 59	20.8 (7.7–40.0)	20.7 (8.3–38.6)	20.8 (8.6–38.1)	20.8 (8.3–41.2)	20.8 (8.0–42.0)
Nepal	60 to 64	20.2 (7.5–39.1)	20.1 (8.0–37.7)	20.2 (8.3–37.2)	20.2 (8.2–40.4)	20.3 (7.9–41.8)

Supplementary Table S11: Prevalence of male SVAC by age and location for 1990, 2000, 2010, 2020, and 2023						
**Location**	**Age Range**	**1990**	**2000**	**2010**	**2020**	**2023**
Nepal	65 to 69	18.9 (6.9–37.3)	18.8 (7.4–35.7)	18.9 (7.6–35.2)	18.9 (7.5–37.9)	18.9 (7.2–38.5)
Nepal	70 to 74	15.4 (5.4–31.2)	15.2 (5.8–30.0)	15.3 (6.0–29.5)	15.3 (5.8–31.7)	15.4 (5.6–32.3)
Nepal	75 to 79	13.9 (4.8–28.6)	13.8 (5.2–27.5)	13.8 (5.3–27.1)	13.9 (5.2–29.7)	14.0 (5.0–30.4)
Nepal	80 to 84	10.0 (3.4–21.4)	9.9 (3.6–20.5)	9.9 (3.7–20.2)	10.0 (3.7–22.3)	10.0 (3.6–22.9)
Nepal	85 to 89	9.1 (2.9–19.9)	8.9 (3.2–18.7)	9.0 (3.3–18.4)	9.0 (3.2–20.4)	9.1 (3.1–21.4)
Nepal	90 to 94	8.2 (2.7–18.2)	8.1 (2.9–17.1)	8.1 (3.0–16.8)	8.2 (2.9–18.7)	8.2 (2.8–19.5)
Nepal	95 plus	8.2 (2.7–17.9)	8.0 (2.8–16.9)	8.1 (2.9–16.6)	8.1 (2.9–18.3)	8.1 (2.8–19.0)
Nepal	Age-standardized	19.5 (7.2–37.9)	19.3 (7.7–36.4)	19.4 (7.9–36.0)	19.4 (7.8–39.0)	19.5 (7.5–39.6)
Nepal	All age	19.7 (7.2–38.2)	19.5 (7.7–36.7)	19.5 (8.0–36.2)	19.5 (7.8–39.2)	19.6 (7.6–39.9)
Pakistan	20 to 24	17.6 (6.3–34.8)	17.4 (6.7–33.5)	17.4 (7.0–33.0)	17.5 (6.8–36.0)	17.6 (6.7–37.1)
Pakistan	25 to 29	18.3 (6.6–35.9)	18.1 (7.1–34.6)	18.2 (7.3–34.1)	18.2 (7.2–37.1)	18.3 (7.2–37.8)
Pakistan	30 to 34	20.3 (7.5–39.1)	20.1 (8.0–37.8)	20.2 (8.3–37.3)	20.2 (8.2–40.4)	20.3 (7.8–41.6)
Pakistan	35 to 39	21.0 (7.8–40.1)	20.8 (8.4–38.8)	20.9 (8.6–38.3)	20.9 (8.4–41.0)	21.0 (8.1–41.7)
Pakistan	40 to 44	22.2 (8.4–42.0)	22.0 (8.9–40.6)	22.1 (9.2–40.1)	22.1 (9.2–42.8)	22.2 (9.0–44.1)
Pakistan	45 to 49	21.9 (8.2–41.6)	21.8 (8.8–40.2)	21.8 (9.1–39.7)	21.8 (8.9–42.4)	21.9 (8.7–43.6)

Supplementary Table S11: Prevalence of male SVAC by age and location for 1990, 2000, 2010, 2020, and 2023						
**Location**	**Age Range**	**1990**	**2000**	**2010**	**2020**	**2023**
Pakistan	50 to 54	21.5 (8.0–41.5)	21.3 (8.6–39.5)	21.4 (8.9–39.1)	21.4 (8.7–42.2)	21.5 (8.3–43.5)
Pakistan	55 to 59	20.8 (7.7–40.0)	20.7 (8.3–38.6)	20.8 (8.6–38.1)	20.8 (8.3–41.2)	20.8 (8.0–42.0)
Pakistan	60 to 64	20.2 (7.5–39.1)	20.1 (8.0–37.7)	20.2 (8.3–37.2)	20.2 (8.2–40.4)	20.3 (7.9–41.8)
Pakistan	65 to 69	18.9 (6.9–37.3)	18.8 (7.4–35.7)	18.9 (7.6–35.2)	18.9 (7.5–37.9)	18.9 (7.2–38.5)
Pakistan	70 to 74	15.4 (5.4–31.2)	15.2 (5.8–30.0)	15.3 (6.0–29.5)	15.3 (5.8–31.7)	15.4 (5.6–32.3)
Pakistan	75 to 79	13.9 (4.8–28.6)	13.8 (5.2–27.5)	13.8 (5.3–27.1)	13.9 (5.2–29.7)	14.0 (5.0–30.4)
Pakistan	80 to 84	10.0 (3.4–21.4)	9.9 (3.6–20.5)	9.9 (3.7–20.2)	10.0 (3.7–22.3)	10.0 (3.6–22.9)
Pakistan	85 to 89	9.1 (2.9–19.9)	8.9 (3.2–18.7)	9.0 (3.3–18.4)	9.0 (3.2–20.4)	9.1 (3.1–21.4)
Pakistan	90 to 94	8.2 (2.7–18.2)	8.1 (2.9–17.1)	8.1 (3.0–16.8)	8.2 (2.9–18.7)	8.2 (2.8–19.5)
Pakistan	95 plus	8.2 (2.7–17.9)	8.0 (2.8–16.9)	8.1 (2.9–16.6)	8.1 (2.9–18.3)	8.1 (2.8–19.0)
Pakistan	Age-standardized	19.5 (7.2–37.9)	19.3 (7.7–36.4)	19.4 (7.9–36.0)	19.4 (7.8–39.0)	19.5 (7.5–39.6)
Pakistan	All age	19.4 (7.1–37.8)	19.4 (7.7–36.5)	19.5 (8.0–36.2)	19.6 (7.9–39.3)	19.7 (7.6–40.0)
Southeast Asia, east Asia, and Oceania	20 to 24	18.7 (11.4–28.4)	17.3 (12.8–23.1)	12.7 (10.3–15.5)	11.3 (8.2–15.3)	11.3 (7.6–16.6)
Southeast Asia, east Asia, and Oceania	25 to 29	15.6 (8.5–25.4)	15.3 (10.0–22.5)	13.1 (10.4–16.3)	12.1 (9.5–15.7)	12.1 (9.0–16.3)
Southeast Asia, east Asia, and Oceania	30 to 34	19.3 (11.0–30.4)	19.6 (14.4–26.0)	16.3 (13.2–20.1)	14.9 (11.8–19.2)	14.7 (11.1–20.1)

Supplementary Table S11: Prevalence of male SVAC by age and location for 1990, 2000, 2010, 2020, and 2023						
**Location**	**Age Range**	**1990**	**2000**	**2010**	**2020**	**2023**
Southeast Asia, east Asia, and Oceania	35 to 39	16.1 (9.5–24.9)	17.3 (12.3–23.7)	15.7 (12.6–19.4)	15.8 (12.8–19.4)	15.9 (12.0–21.4)
Southeast Asia, east Asia, and Oceania	40 to 44	12.1 (7.0–19.1)	14.8 (9.5–21.4)	17.8 (13.1–23.3)	18.7 (12.5–27.0)	18.6 (11.9–27.7)
Southeast Asia, east Asia, and Oceania	45 to 49	11.1 (6.2–17.8)	12.5 (8.1–18.3)	15.5 (12.4–19.2)	16.7 (13.3–21.9)	16.7 (12.6–22.9)
Southeast Asia, east Asia, and Oceania	50 to 54	10.7 (5.6–18.7)	11.0 (7.4–15.7)	13.1 (10.6–16.3)	15.0 (10.6–20.3)	15.2 (9.7–22.3)
Southeast Asia, east Asia, and Oceania	55 to 59	11.5 (5.9–20.5)	11.1 (7.4–16.0)	11.5 (8.7–15.7)	12.6 (8.2–19.0)	12.7 (7.3–20.2)
Southeast Asia, east Asia, and Oceania	60 to 64	11.6 (5.6–20.7)	10.8 (6.3–17.1)	10.7 (7.8–14.6)	11.7 (8.5–16.5)	11.9 (7.7–18.0)
Southeast Asia, east Asia, and Oceania	65 to 69	11.0 (4.5–21.8)	10.3 (4.5–19.3)	10.0 (5.3–16.5)	10.5 (5.2–18.9)	10.8 (4.9–20.2)
Southeast Asia, east Asia, and Oceania	70 to 74	9.3 (3.6–19.0)	9.1 (3.7–17.8)	8.8 (4.4–15.5)	9.0 (4.7–15.9)	9.1 (4.4–16.3)
Southeast Asia, east Asia, and Oceania	75 to 79	8.5 (3.2–18.0)	8.4 (3.2–17.2)	8.2 (3.6–15.6)	8.2 (4.1–15.1)	8.2 (3.9–14.9)
Southeast Asia, east Asia, and Oceania	80 to 84	6.1 (2.2–13.0)	6.0 (2.1–12.9)	6.0 (2.2–12.7)	6.1 (2.3–13.6)	6.1 (2.3–13.5)
Southeast Asia, east Asia, and Oceania	85 to 89	5.5 (2.0–11.8)	5.5 (1.9–11.8)	5.4 (1.9–11.5)	5.5 (2.0–12.3)	5.5 (2.1–12.2)
Southeast Asia, east Asia, and Oceania	90 to 94	5.0 (1.8–10.8)	5.0 (1.7–10.9)	4.9 (1.8–10.5)	5.0 (1.8–11.3)	5.0 (1.9–11.2)
Southeast Asia, east Asia, and Oceania	95 plus	5.2 (1.9–11.3)	5.3 (1.8–11.4)	5.3 (1.9–11.2)	5.2 (1.9–11.8)	5.2 (2.0–11.6)
Southeast Asia, east Asia, and Oceania	Age-standardized	13.9 (8.0–22.2)	14.1 (9.7–20.2)	13.4 (11.2–16.3)	13.5 (11.0–16.7)	13.6 (10.3–17.8)
Southeast Asia, east Asia, and Oceania	All age	14.8 (8.7–23.2)	14.8 (10.2–20.9)	13.8 (11.5–16.6)	13.7 (11.1–16.8)	13.7 (10.3–18.0)

Supplementary Table S11: Prevalence of male SVAC by age and location for 1990, 2000, 2010, 2020, and 2023						
**Location**	**Age Range**	**1990**	**2000**	**2010**	**2020**	**2023**
East Asia	20 to 24	20.5 (12.9–30.5)	19.6 (15.4–24.5)	12.8 (8.6–18.4)	10.5 (5.8–18.1)	10.5 (5.3–19.3)
East Asia	25 to 29	16.4 (9.4–25.9)	16.2 (11.4–22.1)	13.3 (9.4–17.5)	11.7 (6.7–19.9)	11.7 (6.1–21.4)
East Asia	30 to 34	20.8 (12.5–32.0)	21.0 (16.6–26.2)	17.0 (13.5–21.3)	14.8 (9.3–23.6)	14.6 (8.1–25.5)
East Asia	35 to 39	16.4 (10.3–24.4)	18.1 (14.0–22.9)	16.1 (12.4–20.4)	16.3 (11.1–23.7)	16.4 (10.1–26.1)
East Asia	40 to 44	10.8 (6.8–16.1)	14.0 (9.8–19.0)	17.9 (13.6–22.8)	19.4 (13.8–26.1)	19.3 (13.1–27.7)
East Asia	45 to 49	9.6 (5.9–14.6)	11.4 (8.1–15.6)	15.1 (11.5–19.3)	16.9 (10.7–26.4)	16.9 (9.6–28.8)
East Asia	50 to 54	9.5 (5.3–15.5)	9.8 (6.8–13.5)	12.4 (7.5–19.5)	14.9 (8.7–23.7)	15.3 (8.4–25.2)
East Asia	55 to 59	10.7 (5.7–18.5)	10.1 (6.9–13.7)	10.7 (6.2–17.5)	11.9 (5.5–22.1)	12.1 (5.2–23.0)
East Asia	60 to 64	10.9 (5.5–18.9)	9.8 (6.3–14.5)	9.7 (5.9–14.8)	10.7 (5.5–19.6)	11.0 (5.3–20.6)
East Asia	65 to 69	10.4 (4.1–20.6)	9.5 (4.4–17.2)	9.1 (5.1–14.6)	9.8 (4.8–17.5)	10.1 (4.4–18.5)
East Asia	70 to 74	8.9 (3.4–18.4)	8.6 (3.6–16.8)	8.3 (4.3–14.3)	8.5 (4.6–14.4)	8.7 (4.3–15.2)
East Asia	75 to 79	8.2 (3.1–17.1)	8.1 (3.1–16.4)	7.9 (3.6–14.7)	7.8 (4.0–13.9)	7.8 (3.9–13.9)
East Asia	80 to 84	5.8 (2.1–12.5)	5.8 (2.0–12.5)	5.8 (2.1–12.3)	5.9 (2.2–13.3)	5.9 (2.2–13.2)
East Asia	85 to 89	5.2 (1.9–11.3)	5.2 (1.8–11.3)	5.3 (1.9–11.1)	5.3 (2.0–12.0)	5.3 (2.0–11.9)
East Asia	90 to 94	4.7 (1.7–10.2)	4.7 (1.6–10.2)	4.7 (1.7–10.1)	4.8 (1.8–10.9)	4.8 (1.8–10.8)

Supplementary Table S11: Prevalence of male SVAC by age and location for 1990, 2000, 2010, 2020, and 2023						
**Location**	**Age Range**	**1990**	**2000**	**2010**	**2020**	**2023**
East Asia	95 plus	4.6 (1.7–10.1)	4.7 (1.6–10.2)	4.7 (1.7–10.0)	4.8 (1.8–10.8)	4.8 (1.8–10.7)
East Asia	Age-standardized	13.9 (8.6–21.1)	14.3 (10.8–18.8)	13.4 (11.2–15.9)	13.3 (10.2–18.0)	13.4 (9.5–19.2)
East Asia	All age	15.0 (9.4–22.4)	15.0 (11.4–19.5)	13.6 (11.4–16.2)	13.4 (10.3–17.9)	13.4 (9.6–19.2)
China	20 to 24	20.7 (13.1–30.6)	19.8 (15.6–24.6)	12.7 (8.2–18.7)	10.3 (5.1–18.7)	10.3 (4.6–19.7)
China	25 to 29	16.5 (9.5–25.9)	16.3 (11.6–22.0)	13.3 (9.1–17.9)	11.6 (5.9–20.4)	11.6 (5.1–22.1)
China	30 to 34	21.0 (12.7–32.1)	21.2 (16.8–26.2)	17.1 (13.2–21.5)	14.8 (8.8–24.1)	14.6 (7.5–26.1)
China	35 to 39	16.4 (10.4–24.4)	18.2 (14.1–23.0)	16.1 (12.3–20.6)	16.3 (10.8–24.3)	16.4 (9.7–26.6)
China	40 to 44	10.7 (6.7–15.8)	13.9 (9.8–18.8)	18.0 (13.7–22.8)	19.5 (14.0–26.2)	19.5 (13.3–27.7)
China	45 to 49	9.4 (5.9–14.2)	11.3 (8.1–15.2)	15.2 (11.4–19.6)	17.0 (10.3–26.9)	17.0 (9.0–29.5)
China	50 to 54	9.3 (5.3–15.1)	9.7 (6.6–13.5)	12.4 (7.0–20.0)	14.9 (8.3–24.1)	15.3 (8.1–25.6)
China	55 to 59	10.6 (5.7–18.3)	9.9 (6.8–13.7)	10.6 (5.6–17.8)	11.8 (5.0–22.5)	12.1 (5.0–23.4)
China	60 to 64	10.8 (5.5–18.7)	9.7 (6.3–14.1)	9.6 (5.5–15.0)	10.6 (5.0–20.0)	10.9 (4.8–21.2)
China	65 to 69	10.3 (4.2–20.5)	9.5 (4.4–17.0)	9.0 (5.0–14.4)	9.8 (4.8–17.4)	10.1 (4.4–18.5)
China	70 to 74	8.9 (3.4–18.3)	8.6 (3.6–16.7)	8.3 (4.3–14.1)	8.5 (4.6–14.3)	8.7 (4.3–15.2)
China	75 to 79	8.2 (3.1–17.1)	8.1 (3.1–16.4)	7.9 (3.6–14.6)	7.8 (4.0–13.7)	7.8 (3.9–13.8)

Supplementary Table S11: Prevalence of male SVAC by age and location for 1990, 2000, 2010, 2020, and 2023						
**Location**	**Age Range**	**1990**	**2000**	**2010**	**2020**	**2023**
China	80 to 84	5.8 (2.1–12.5)	5.8 (2.0–12.5)	5.8 (2.1–12.3)	5.9 (2.2–13.3)	5.9 (2.2–13.2)
China	85 to 89	5.2 (1.9–11.2)	5.2 (1.8–11.3)	5.3 (1.9–11.1)	5.3 (2.0–12.0)	5.3 (2.0–11.9)
China	90 to 94	4.7 (1.7–10.2)	4.7 (1.6–10.2)	4.7 (1.7–10.1)	4.8 (1.8–10.9)	4.8 (1.8–10.8)
China	95 plus	4.6 (1.7–10.1)	4.7 (1.6–10.2)	4.7 (1.7–10.0)	4.8 (1.8–10.8)	4.8 (1.8–10.7)
China	Age-standardized	13.9 (8.7–20.9)	14.3 (10.9–18.6)	13.3 (11.0–16.1)	13.3 (9.9–18.5)	13.4 (9.1–19.5)
China	All age	15.0 (9.6–22.2)	15.0 (11.5–19.3)	13.6 (11.3–16.4)	13.4 (9.9–18.3)	13.3 (9.3–19.4)
North Korea	20 to 24	13.5 (5.2–27.0)	13.6 (5.1–27.1)	13.6 (5.2–26.7)	13.7 (5.5–28.3)	13.7 (5.6–28.2)
North Korea	25 to 29	13.7 (5.3–27.4)	13.8 (5.2–27.5)	13.8 (5.3–27.1)	14.0 (5.6–28.7)	14.0 (5.7–28.6)
North Korea	30 to 34	14.7 (5.8–29.0)	14.8 (5.6–29.2)	14.8 (5.8–28.8)	15.0 (6.1–30.5)	15.0 (6.1–30.3)
North Korea	35 to 39	14.6 (5.7–28.9)	14.7 (5.6–29.0)	14.8 (5.8–28.7)	14.9 (6.1–30.4)	15.0 (6.1–30.2)
North Korea	40 to 44	14.8 (5.8–29.2)	14.9 (5.6–29.4)	15.0 (5.9–29.1)	15.2 (6.2–30.8)	15.2 (6.2–30.7)
North Korea	45 to 49	14.1 (5.5–28.1)	14.3 (5.4–28.3)	14.4 (5.6–28.0)	14.5 (5.9–29.7)	14.6 (5.9–29.5)
North Korea	50 to 54	13.6 (5.3–27.2)	13.7 (5.1–27.3)	13.8 (5.3–27.1)	14.0 (5.6–28.8)	14.0 (5.7–28.6)
North Korea	55 to 59	13.0 (5.0–26.1)	13.1 (4.9–26.3)	13.2 (5.1–26.0)	13.4 (5.3–27.7)	13.4 (5.4–27.5)
North Korea	60 to 64	12.5 (4.8–25.2)	12.6 (4.6–25.3)	12.6 (4.8–25.0)	12.8 (5.1–26.6)	12.8 (5.1–26.5)

Supplementary Table S11: Prevalence of male SVAC by age and location for 1990, 2000, 2010, 2020, and 2023						
**Location**	**Age Range**	**1990**	**2000**	**2010**	**2020**	**2023**
North Korea	65 to 69	11.6 (4.4–23.5)	11.6 (4.3–23.6)	11.7 (4.4–23.4)	11.8 (4.7–24.9)	11.9 (4.7–25.0)
North Korea	70 to 74	9.2 (3.4–19.2)	9.2 (3.3–19.2)	9.3 (3.4–19.0)	9.4 (3.6–20.3)	9.4 (3.7–20.2)
North Korea	75 to 79	8.3 (3.0–17.4)	8.3 (2.9–17.4)	8.3 (3.1–17.2)	8.4 (3.2–18.4)	8.5 (3.3–18.3)
North Korea	80 to 84	5.8 (2.1–12.5)	5.8 (2.0–12.5)	5.9 (2.1–12.4)	5.9 (2.2–13.3)	6.0 (2.2–13.2)
North Korea	85 to 89	5.2 (1.9–11.3)	5.2 (1.8–11.3)	5.3 (1.9–11.2)	5.3 (2.0–12.0)	5.3 (2.0–11.9)
North Korea	90 to 94	4.7 (1.7–10.2)	4.7 (1.6–10.3)	4.8 (1.7–10.1)	4.8 (1.8–10.9)	4.8 (1.8–10.8)
North Korea	95 plus	4.7 (1.7–10.1)	4.7 (1.6–10.2)	4.7 (1.7–10.0)	4.8 (1.8–10.8)	4.8 (1.8–10.7)
North Korea	Age-standardized	13.2 (5.1–26.5)	13.3 (5.0–26.6)	13.4 (5.2–26.3)	13.5 (5.4–27.9)	13.6 (5.5–27.8)
North Korea	All age	13.7 (5.3–27.3)	13.8 (5.2–27.4)	13.8 (5.3–27.0)	13.8 (5.6–28.4)	13.8 (5.6–28.2)
Taiwan	20 to 24	13.5 (5.2–27.0)	13.6 (5.1–27.1)	13.6 (5.2–26.7)	13.7 (5.5–28.3)	13.7 (5.6–28.2)
Taiwan	25 to 29	13.7 (5.3–27.4)	13.8 (5.2–27.5)	13.8 (5.3–27.1)	14.0 (5.6–28.7)	14.0 (5.7–28.6)
Taiwan	30 to 34	14.7 (5.8–29.0)	14.8 (5.6–29.2)	14.8 (5.8–28.8)	15.0 (6.1–30.5)	15.0 (6.1–30.3)
Taiwan	35 to 39	14.6 (5.7–28.9)	14.7 (5.6–29.0)	14.8 (5.8–28.7)	14.9 (6.1–30.4)	15.0 (6.1–30.2)
Taiwan	40 to 44	14.8 (5.8–29.2)	14.9 (5.6–29.4)	15.0 (5.9–29.1)	15.2 (6.2–30.8)	15.2 (6.2–30.7)
Taiwan	45 to 49	14.1 (5.5–28.1)	14.3 (5.4–28.3)	14.4 (5.6–28.0)	14.5 (5.9–29.7)	14.6 (5.9–29.5)

Supplementary Table S11: Prevalence of male SVAC by age and location for 1990, 2000, 2010, 2020, and 2023						
**Location**	**Age Range**	**1990**	**2000**	**2010**	**2020**	**2023**
Taiwan	50 to 54	13.6 (5.3–27.2)	13.7 (5.1–27.3)	13.8 (5.3–27.1)	14.0 (5.6–28.8)	14.0 (5.7–28.6)
Taiwan	55 to 59	13.0 (5.0–26.1)	13.1 (4.9–26.3)	13.2 (5.1–26.0)	13.4 (5.3–27.7)	13.4 (5.4–27.5)
Taiwan	60 to 64	12.5 (4.8–25.2)	12.6 (4.6–25.3)	12.6 (4.8–25.0)	12.8 (5.1–26.6)	12.8 (5.1–26.5)
Taiwan	65 to 69	11.6 (4.4–23.5)	11.6 (4.3–23.6)	11.7 (4.4–23.4)	11.8 (4.7–24.9)	11.9 (4.7–25.0)
Taiwan	70 to 74	9.2 (3.4–19.2)	9.2 (3.3–19.2)	9.3 (3.4–19.0)	9.4 (3.6–20.3)	9.4 (3.7–20.2)
Taiwan	75 to 79	8.3 (3.0–17.4)	8.3 (2.9–17.4)	8.3 (3.1–17.2)	8.4 (3.2–18.4)	8.5 (3.3–18.3)
Taiwan	80 to 84	5.8 (2.1–12.5)	5.8 (2.0–12.5)	5.9 (2.1–12.4)	5.9 (2.2–13.3)	6.0 (2.2–13.2)
Taiwan	85 to 89	5.2 (1.9–11.3)	5.2 (1.8–11.3)	5.3 (1.9–11.2)	5.3 (2.0–12.0)	5.3 (2.0–11.9)
Taiwan	90 to 94	4.7 (1.7–10.2)	4.7 (1.6–10.3)	4.8 (1.7–10.1)	4.8 (1.8–10.9)	4.8 (1.8–10.8)
Taiwan	95 plus	4.7 (1.7–10.1)	4.7 (1.6–10.2)	4.7 (1.7–10.0)	4.8 (1.8–10.8)	4.8 (1.8–10.7)
Taiwan	Age-standardized	13.2 (5.1–26.5)	13.3 (5.0–26.6)	13.4 (5.2–26.3)	13.5 (5.4–27.9)	13.6 (5.5–27.8)
Taiwan	All age	13.5 (5.3–27.0)	13.5 (5.1–26.9)	13.4 (5.2–26.3)	13.3 (5.3–27.5)	13.2 (5.3–27.1)
Oceania	20 to 24	11.2 (4.4–22.7)	10.5 (4.3–20.6)	9.9 (4.6–17.8)	10.3 (4.7–19.4)	10.5 (4.5–20.5)
Oceania	25 to 29	11.7 (4.6–23.5)	10.9 (4.5–21.3)	10.3 (4.9–18.3)	10.6 (5.0–20.0)	10.8 (4.7–20.9)
Oceania	30 to 34	14.3 (6.0–27.6)	14.6 (6.7–26.0)	14.7 (8.8–22.1)	14.8 (7.9–24.7)	14.8 (6.8–25.4)

Supplementary Table S11: Prevalence of male SVAC by age and location for 1990, 2000, 2010, 2020, and 2023						
**Location**	**Age Range**	**1990**	**2000**	**2010**	**2020**	**2023**
Oceania	35 to 39	14.3 (6.0–27.6)	14.1 (6.4–25.6)	13.9 (8.1–21.6)	14.2 (7.4–23.9)	14.3 (6.6–25.1)
Oceania	40 to 44	16.5 (7.1–30.9)	17.4 (8.3–30.4)	18.3 (11.3–26.9)	18.1 (9.9–29.5)	17.9 (8.5–30.0)
Oceania	45 to 49	15.8 (6.7–29.9)	16.2 (7.6–28.7)	16.6 (10.0–25.1)	16.6 (8.9–27.4)	16.5 (7.8–28.3)
Oceania	50 to 54	14.6 (5.7–29.0)	14.6 (5.5–28.9)	14.7 (5.7–28.5)	14.8 (6.0–30.1)	14.8 (6.0–29.9)
Oceania	55 to 59	14.2 (5.5–28.2)	14.2 (5.3–28.1)	14.2 (5.5–27.7)	14.3 (5.8–29.3)	14.3 (5.8–29.1)
Oceania	60 to 64	13.7 (5.3–27.4)	13.7 (5.1–27.3)	13.7 (5.3–26.9)	13.8 (5.6–28.5)	13.9 (5.6–28.4)
Oceania	65 to 69	12.8 (4.9–25.7)	12.8 (4.7–25.7)	12.8 (4.9–25.3)	12.9 (5.1–26.8)	12.9 (5.2–26.6)
Oceania	70 to 74	10.2 (3.8–21.0)	10.2 (3.7–21.0)	10.2 (3.8–20.7)	10.3 (4.0–22.0)	10.3 (4.0–21.8)
Oceania	75 to 79	9.2 (3.4–19.1)	9.2 (3.3–19.1)	9.2 (3.4–18.8)	9.2 (3.6–20.0)	9.3 (3.6–19.9)
Oceania	80 to 84	6.5 (2.3–13.8)	6.5 (2.2–13.8)	6.5 (2.3–13.6)	6.5 (2.4–14.5)	6.5 (2.5–14.4)
Oceania	85 to 89	5.8 (2.1–12.5)	5.8 (2.0–12.5)	5.8 (2.1–12.3)	5.9 (2.2–13.1)	5.9 (2.2–13.1)
Oceania	90 to 94	5.2 (1.9–11.4)	5.2 (1.8–11.3)	5.2 (1.9–11.1)	5.3 (2.0–11.9)	5.3 (2.0–11.8)
Oceania	95 plus	5.2 (1.9–11.3)	5.2 (1.8–11.2)	5.2 (1.9–11.0)	5.2 (1.9–11.8)	5.2 (2.0–11.7)
Oceania	Age-standardized	13.3 (5.5–26.1)	13.3 (5.7–25.3)	13.2 (6.8–22.7)	13.4 (6.7–24.1)	13.4 (6.2–25.0)
Oceania	All age	13.4 (5.5–26.3)	13.3 (5.8–25.1)	13.3 (7.1–22.2)	13.5 (6.7–23.9)	13.5 (6.3–24.8)

Supplementary Table S11: Prevalence of male SVAC by age and location for 1990, 2000, 2010, 2020, and 2023						
**Location**	**Age Range**	**1990**	**2000**	**2010**	**2020**	**2023**
American Samoa	20 to 24	12.2 (4.7–24.7)	12.2 (4.5–24.6)	12.2 (4.6–24.2)	12.2 (4.9–25.7)	12.3 (4.9–25.7)
American Samoa	25 to 29	12.6 (4.9–25.5)	12.6 (4.7–25.4)	12.6 (4.8–25.0)	12.7 (5.1–26.5)	12.7 (5.1–26.3)
American Samoa	30 to 34	14.1 (5.5–28.0)	14.1 (5.3–27.9)	14.1 (5.4–27.5)	14.2 (5.7–29.1)	14.2 (5.7–28.9)
American Samoa	35 to 39	14.5 (5.7–28.7)	14.5 (5.5–28.7)	14.5 (5.6–28.3)	14.6 (5.9–29.9)	14.7 (6.0–29.7)
American Samoa	40 to 44	15.3 (6.0–30.1)	15.3 (5.8–30.1)	15.3 (6.0–29.6)	15.5 (6.3–31.3)	15.5 (6.4–31.2)
American Samoa	45 to 49	15.1 (5.9–29.7)	15.1 (5.7–29.6)	15.1 (5.9–29.2)	15.2 (6.2–30.9)	15.2 (6.2–30.7)
American Samoa	50 to 54	14.7 (5.8–29.1)	14.7 (5.6–29.1)	14.7 (5.7–28.6)	14.9 (6.0–30.3)	14.9 (6.1–30.1)
American Samoa	55 to 59	14.2 (5.6–28.3)	14.2 (5.4–28.2)	14.3 (5.5–27.8)	14.4 (5.8–29.5)	14.4 (5.8–29.3)
American Samoa	60 to 64	13.8 (5.4–27.5)	13.8 (5.2–27.5)	13.8 (5.3–27.0)	13.9 (5.6–28.7)	13.9 (5.6–28.5)
American Samoa	65 to 69	12.8 (4.9–25.8)	12.8 (4.8–25.8)	12.9 (4.9–25.4)	12.9 (5.2–26.9)	13.0 (5.2–26.7)
American Samoa	70 to 74	10.2 (3.8–21.1)	10.2 (3.7–21.1)	10.3 (3.8–20.7)	10.3 (4.0–22.1)	10.3 (4.1–21.9)
American Samoa	75 to 79	9.2 (3.4–19.2)	9.2 (3.3–19.2)	9.2 (3.4–18.8)	9.3 (3.6–20.1)	9.3 (3.6–20.0)
American Samoa	80 to 84	6.5 (2.4–13.9)	6.5 (2.3–13.9)	6.5 (2.3–13.6)	6.6 (2.5–14.6)	6.6 (2.5–14.5)
American Samoa	85 to 89	5.8 (2.1–12.6)	5.8 (2.0–12.6)	5.8 (2.1–12.3)	5.9 (2.2–13.2)	5.9 (2.2–13.1)
American Samoa	90 to 94	5.3 (1.9–11.4)	5.3 (1.8–11.4)	5.3 (1.9–11.2)	5.3 (2.0–12.0)	5.3 (2.0–11.9)

**Supplementary Table S11: Prevalence of male SVAC by age and location for 1990, 2000, 2010, 2020, and 2023**						
**Location**	**Age Range**	**1990**	**2000**	**2010**	**2020**	**2023**
American Samoa	95 plus	5.2 (1.9–11.3)	5.2 (1.8–11.3)	5.2 (1.9–11.1)	5.3 (2.0–11.9)	5.3 (2.0–11.8)
American Samoa	Age-standardized	13.4 (5.2–26.7)	13.4 (5.0–26.7)	13.4 (5.2–26.3)	13.5 (5.4–27.8)	13.5 (5.5–27.7)
American Samoa	All age	13.6 (5.3–27.1)	13.6 (5.1–27.2)	13.7 (5.3–26.8)	13.8 (5.5–28.3)	13.7 (5.6–28.1)
Cook Islands	20 to 24	12.2 (4.7–24.7)	12.2 (4.5–24.6)	12.2 (4.6–24.2)	12.2 (4.9–25.7)	12.3 (4.9–25.7)
Cook Islands	25 to 29	12.6 (4.9–25.5)	12.6 (4.7–25.4)	12.6 (4.8–25.0)	12.7 (5.1–26.5)	12.7 (5.1–26.3)
Cook Islands	30 to 34	14.1 (5.5–28.0)	14.1 (5.3–27.9)	14.1 (5.4–27.5)	14.2 (5.7–29.1)	14.2 (5.7–28.9)
Cook Islands	35 to 39	14.5 (5.7–28.7)	14.5 (5.5–28.7)	14.5 (5.6–28.3)	14.6 (5.9–29.9)	14.7 (6.0–29.7)
Cook Islands	40 to 44	15.3 (6.0–30.1)	15.3 (5.8–30.1)	15.3 (6.0–29.6)	15.5 (6.3–31.3)	15.5 (6.4–31.2)
Cook Islands	45 to 49	15.1 (5.9–29.7)	15.1 (5.7–29.6)	15.1 (5.9–29.2)	15.2 (6.2–30.9)	15.2 (6.2–30.7)
Cook Islands	50 to 54	14.7 (5.8–29.1)	14.7 (5.6–29.1)	14.7 (5.7–28.6)	14.9 (6.0–30.3)	14.9 (6.1–30.1)
Cook Islands	55 to 59	14.2 (5.6–28.3)	14.2 (5.4–28.2)	14.3 (5.5–27.8)	14.4 (5.8–29.5)	14.4 (5.8–29.3)
Cook Islands	60 to 64	13.8 (5.4–27.5)	13.8 (5.2–27.5)	13.8 (5.3–27.0)	13.9 (5.6–28.7)	13.9 (5.6–28.5)
Cook Islands	65 to 69	12.8 (4.9–25.8)	12.8 (4.8–25.8)	12.9 (4.9–25.4)	12.9 (5.2–26.9)	13.0 (5.2–26.7)
Cook Islands	70 to 74	10.2 (3.8–21.1)	10.2 (3.7–21.1)	10.3 (3.8–20.7)	10.3 (4.0–22.1)	10.3 (4.1–21.9)
Cook Islands	75 to 79	9.2 (3.4–19.2)	9.2 (3.3–19.2)	9.2 (3.4–18.8)	9.3 (3.6–20.1)	9.3 (3.6–20.0)

Supplementary Table S11: Prevalence of male SVAC by age and location for 1990, 2000, 2010, 2020, and 2023						
**Location**	**Age Range**	**1990**	**2000**	**2010**	**2020**	**2023**
Cook Islands	80 to 84	6.5 (2.4–13.9)	6.5 (2.3–13.9)	6.5 (2.3–13.6)	6.6 (2.5–14.6)	6.6 (2.5–14.5)
Cook Islands	85 to 89	5.8 (2.1–12.6)	5.8 (2.0–12.6)	5.8 (2.1–12.3)	5.9 (2.2–13.2)	5.9 (2.2–13.1)
Cook Islands	90 to 94	5.3 (1.9–11.4)	5.3 (1.8–11.4)	5.3 (1.9–11.2)	5.3 (2.0–12.0)	5.3 (2.0–11.9)
Cook Islands	95 plus	5.2 (1.9–11.3)	5.2 (1.8–11.3)	5.2 (1.9–11.1)	5.3 (2.0–11.9)	5.3 (2.0–11.8)
Cook Islands	Age-standardized	13.4 (5.2–26.7)	13.4 (5.0–26.7)	13.4 (5.2–26.3)	13.5 (5.4–27.8)	13.5 (5.5–27.7)
Cook Islands	All age	13.5 (5.2–26.9)	13.5 (5.1–27.0)	13.5 (5.2–26.5)	13.4 (5.4–27.7)	13.3 (5.4–27.3)
Fiji	20 to 24	12.2 (4.7–24.7)	12.2 (4.5–24.6)	12.2 (4.6–24.2)	12.2 (4.9–25.7)	12.3 (4.9–25.7)
Fiji	25 to 29	12.6 (4.9–25.5)	12.6 (4.7–25.4)	12.6 (4.8–25.0)	12.7 (5.1–26.5)	12.7 (5.1–26.3)
Fiji	30 to 34	14.1 (5.5–28.0)	14.1 (5.3–27.9)	14.1 (5.4–27.5)	14.2 (5.7–29.1)	14.2 (5.7–28.9)
Fiji	35 to 39	14.5 (5.7–28.7)	14.5 (5.5–28.7)	14.5 (5.6–28.3)	14.6 (5.9–29.9)	14.7 (6.0–29.7)
Fiji	40 to 44	15.3 (6.0–30.1)	15.3 (5.8–30.1)	15.3 (6.0–29.6)	15.5 (6.3–31.3)	15.5 (6.4–31.2)
Fiji	45 to 49	15.1 (5.9–29.7)	15.1 (5.7–29.6)	15.1 (5.9–29.2)	15.2 (6.2–30.9)	15.2 (6.2–30.7)
Fiji	50 to 54	14.7 (5.8–29.1)	14.7 (5.6–29.1)	14.7 (5.7–28.6)	14.9 (6.0–30.3)	14.9 (6.1–30.1)
Fiji	55 to 59	14.2 (5.6–28.3)	14.2 (5.4–28.2)	14.3 (5.5–27.8)	14.4 (5.8–29.5)	14.4 (5.8–29.3)
Fiji	60 to 64	13.8 (5.4–27.5)	13.8 (5.2–27.5)	13.8 (5.3–27.0)	13.9 (5.6–28.7)	13.9 (5.6–28.5)

Supplementary Table S11: Prevalence of male SVAC by age and location for 1990, 2000, 2010, 2020, and 2023						
**Location**	**Age Range**	**1990**	**2000**	**2010**	**2020**	**2023**
Fiji	65 to 69	12.8 (4.9–25.8)	12.8 (4.8–25.8)	12.9 (4.9–25.4)	12.9 (5.2–26.9)	13.0 (5.2–26.7)
Fiji	70 to 74	10.2 (3.8–21.1)	10.2 (3.7–21.1)	10.3 (3.8–20.7)	10.3 (4.0–22.1)	10.3 (4.1–21.9)
Fiji	75 to 79	9.2 (3.4–19.2)	9.2 (3.3–19.2)	9.2 (3.4–18.8)	9.3 (3.6–20.1)	9.3 (3.6–20.0)
Fiji	80 to 84	6.5 (2.4–13.9)	6.5 (2.3–13.9)	6.5 (2.3–13.6)	6.6 (2.5–14.6)	6.6 (2.5–14.5)
Fiji	85 to 89	5.8 (2.1–12.6)	5.8 (2.0–12.6)	5.8 (2.1–12.3)	5.9 (2.2–13.2)	5.9 (2.2–13.1)
Fiji	90 to 94	5.3 (1.9–11.4)	5.3 (1.8–11.4)	5.3 (1.9–11.2)	5.3 (2.0–12.0)	5.3 (2.0–11.9)
Fiji	95 plus	5.2 (1.9–11.3)	5.2 (1.8–11.3)	5.2 (1.9–11.1)	5.3 (2.0–11.9)	5.3 (2.0–11.8)
Fiji	Age-standardized	13.4 (5.2–26.7)	13.4 (5.0–26.7)	13.4 (5.2–26.3)	13.5 (5.4–27.8)	13.5 (5.5–27.7)
Fiji	All age	13.6 (5.3–27.2)	13.7 (5.1–27.2)	13.7 (5.3–26.8)	13.8 (5.6–28.4)	13.8 (5.6–28.2)
Guam	20 to 24	12.2 (4.7–24.7)	12.2 (4.5–24.6)	12.2 (4.6–24.2)	12.2 (4.9–25.7)	12.3 (4.9–25.7)
Guam	25 to 29	12.6 (4.9–25.5)	12.6 (4.7–25.4)	12.6 (4.8–25.0)	12.7 (5.1–26.5)	12.7 (5.1–26.3)
Guam	30 to 34	14.1 (5.5–28.0)	14.1 (5.3–27.9)	14.1 (5.4–27.5)	14.2 (5.7–29.1)	14.2 (5.7–28.9)
Guam	35 to 39	14.5 (5.7–28.7)	14.5 (5.5–28.7)	14.5 (5.6–28.3)	14.6 (5.9–29.9)	14.7 (6.0–29.7)
Guam	40 to 44	15.3 (6.0–30.1)	15.3 (5.8–30.1)	15.3 (6.0–29.6)	15.5 (6.3–31.3)	15.5 (6.4–31.2)
Guam	45 to 49	15.1 (5.9–29.7)	15.1 (5.7–29.6)	15.1 (5.9–29.2)	15.2 (6.2–30.9)	15.2 (6.2–30.7)

Supplementary Table S11: Prevalence of male SVAC by age and location for 1990, 2000, 2010, 2020, and 2023						
**Location**	**Age Range**	**1990**	**2000**	**2010**	**2020**	**2023**
Guam	50 to 54	14.7 (5.8–29.1)	14.7 (5.6–29.1)	14.7 (5.7–28.6)	14.9 (6.0–30.3)	14.9 (6.1–30.1)
Guam	55 to 59	14.2 (5.6–28.3)	14.2 (5.4–28.2)	14.3 (5.5–27.8)	14.4 (5.8–29.5)	14.4 (5.8–29.3)
Guam	60 to 64	13.8 (5.4–27.5)	13.8 (5.2–27.5)	13.8 (5.3–27.0)	13.9 (5.6–28.7)	13.9 (5.6–28.5)
Guam	65 to 69	12.8 (4.9–25.8)	12.8 (4.8–25.8)	12.9 (4.9–25.4)	12.9 (5.2–26.9)	13.0 (5.2–26.7)
Guam	70 to 74	10.2 (3.8–21.1)	10.2 (3.7–21.1)	10.3 (3.8–20.7)	10.3 (4.0–22.1)	10.3 (4.1–21.9)
Guam	75 to 79	9.2 (3.4–19.2)	9.2 (3.3–19.2)	9.2 (3.4–18.8)	9.3 (3.6–20.1)	9.3 (3.6–20.0)
Guam	80 to 84	6.5 (2.4–13.9)	6.5 (2.3–13.9)	6.5 (2.3–13.6)	6.6 (2.5–14.6)	6.6 (2.5–14.5)
Guam	85 to 89	5.8 (2.1–12.6)	5.8 (2.0–12.6)	5.8 (2.1–12.3)	5.9 (2.2–13.2)	5.9 (2.2–13.1)
Guam	90 to 94	5.3 (1.9–11.4)	5.3 (1.8–11.4)	5.3 (1.9–11.2)	5.3 (2.0–12.0)	5.3 (2.0–11.9)
Guam	95 plus	5.2 (1.9–11.3)	5.2 (1.8–11.3)	5.2 (1.9–11.1)	5.3 (2.0–11.9)	5.3 (2.0–11.8)
Guam	Age-standardized	13.4 (5.2–26.7)	13.4 (5.0–26.7)	13.4 (5.2–26.3)	13.5 (5.4–27.8)	13.5 (5.5–27.7)
Guam	All age	13.6 (5.3–27.1)	13.7 (5.1–27.3)	13.7 (5.3–26.8)	13.6 (5.5–28.0)	13.6 (5.5–27.8)
Kiribati	20 to 24	12.2 (4.7–24.7)	12.2 (4.5–24.6)	12.2 (4.6–24.2)	12.2 (4.9–25.7)	12.3 (4.9–25.7)
Kiribati	25 to 29	12.6 (4.9–25.5)	12.6 (4.7–25.4)	12.6 (4.8–25.0)	12.7 (5.1–26.5)	12.7 (5.1–26.3)
Kiribati	30 to 34	14.1 (5.5–28.0)	14.1 (5.3–27.9)	14.1 (5.4–27.5)	14.2 (5.7–29.1)	14.2 (5.7–28.9)

Supplementary Table S11: Prevalence of male SVAC by age and location for 1990, 2000, 2010, 2020, and 2023						
**Location**	**Age Range**	**1990**	**2000**	**2010**	**2020**	**2023**
Kiribati	35 to 39	14.5 (5.7–28.7)	14.5 (5.5–28.7)	14.5 (5.6–28.3)	14.6 (5.9–29.9)	14.7 (6.0–29.7)
Kiribati	40 to 44	15.3 (6.0–30.1)	15.3 (5.8–30.1)	15.3 (6.0–29.6)	15.5 (6.3–31.3)	15.5 (6.4–31.2)
Kiribati	45 to 49	15.1 (5.9–29.7)	15.1 (5.7–29.6)	15.1 (5.9–29.2)	15.2 (6.2–30.9)	15.2 (6.2–30.7)
Kiribati	50 to 54	14.7 (5.8–29.1)	14.7 (5.6–29.1)	14.7 (5.7–28.6)	14.9 (6.0–30.3)	14.9 (6.1–30.1)
Kiribati	55 to 59	14.2 (5.6–28.3)	14.2 (5.4–28.2)	14.3 (5.5–27.8)	14.4 (5.8–29.5)	14.4 (5.8–29.3)
Kiribati	60 to 64	13.8 (5.4–27.5)	13.8 (5.2–27.5)	13.8 (5.3–27.0)	13.9 (5.6–28.7)	13.9 (5.6–28.5)
Kiribati	65 to 69	12.8 (4.9–25.8)	12.8 (4.8–25.8)	12.9 (4.9–25.4)	12.9 (5.2–26.9)	13.0 (5.2–26.7)
Kiribati	70 to 74	10.2 (3.8–21.1)	10.2 (3.7–21.1)	10.3 (3.8–20.7)	10.3 (4.0–22.1)	10.3 (4.1–21.9)
Kiribati	75 to 79	9.2 (3.4–19.2)	9.2 (3.3–19.2)	9.2 (3.4–18.8)	9.3 (3.6–20.1)	9.3 (3.6–20.0)
Kiribati	80 to 84	6.5 (2.4–13.9)	6.5 (2.3–13.9)	6.5 (2.3–13.6)	6.6 (2.5–14.6)	6.6 (2.5–14.5)
Kiribati	85 to 89	5.8 (2.1–12.6)	5.8 (2.0–12.6)	5.8 (2.1–12.3)	5.9 (2.2–13.2)	5.9 (2.2–13.1)
Kiribati	90 to 94	5.3 (1.9–11.4)	5.3 (1.8–11.4)	5.3 (1.9–11.2)	5.3 (2.0–12.0)	5.3 (2.0–11.9)
Kiribati	95 plus	5.2 (1.9–11.3)	5.2 (1.8–11.3)	5.2 (1.9–11.1)	5.3 (2.0–11.9)	5.3 (2.0–11.8)
Kiribati	Age-standardized	13.4 (5.2–26.7)	13.4 (5.0–26.7)	13.4 (5.2–26.3)	13.5 (5.4–27.8)	13.5 (5.5–27.7)
Kiribati	All age	13.5 (5.3–27.0)	13.7 (5.1–27.3)	13.6 (5.3–26.7)	13.7 (5.5–28.3)	13.8 (5.6–28.2)

Supplementary Table S11: Prevalence of male SVAC by age and location for 1990, 2000, 2010, 2020, and 2023						
**Location**	**Age Range**	**1990**	**2000**	**2010**	**2020**	**2023**
Marshall Islands	20 to 24	12.2 (4.7–24.7)	12.2 (4.5–24.6)	12.2 (4.6–24.2)	12.2 (4.9–25.7)	12.3 (4.9–25.7)
Marshall Islands	25 to 29	12.6 (4.9–25.5)	12.6 (4.7–25.4)	12.6 (4.8–25.0)	12.7 (5.1–26.5)	12.7 (5.1–26.3)
Marshall Islands	30 to 34	14.1 (5.5–28.0)	14.1 (5.3–27.9)	14.1 (5.4–27.5)	14.2 (5.7–29.1)	14.2 (5.7–28.9)
Marshall Islands	35 to 39	14.5 (5.7–28.7)	14.5 (5.5–28.7)	14.5 (5.6–28.3)	14.6 (5.9–29.9)	14.7 (6.0–29.7)
Marshall Islands	40 to 44	15.3 (6.0–30.1)	15.3 (5.8–30.1)	15.3 (6.0–29.6)	15.5 (6.3–31.3)	15.5 (6.4–31.2)
Marshall Islands	45 to 49	15.1 (5.9–29.7)	15.1 (5.7–29.6)	15.1 (5.9–29.2)	15.2 (6.2–30.9)	15.2 (6.2–30.7)
Marshall Islands	50 to 54	14.7 (5.8–29.1)	14.7 (5.6–29.1)	14.7 (5.7–28.6)	14.9 (6.0–30.3)	14.9 (6.1–30.1)
Marshall Islands	55 to 59	14.2 (5.6–28.3)	14.2 (5.4–28.2)	14.3 (5.5–27.8)	14.4 (5.8–29.5)	14.4 (5.8–29.3)
Marshall Islands	60 to 64	13.8 (5.4–27.5)	13.8 (5.2–27.5)	13.8 (5.3–27.0)	13.9 (5.6–28.7)	13.9 (5.6–28.5)
Marshall Islands	65 to 69	12.8 (4.9–25.8)	12.8 (4.8–25.8)	12.9 (4.9–25.4)	12.9 (5.2–26.9)	13.0 (5.2–26.7)
Marshall Islands	70 to 74	10.2 (3.8–21.1)	10.2 (3.7–21.1)	10.3 (3.8–20.7)	10.3 (4.0–22.1)	10.3 (4.1–21.9)
Marshall Islands	75 to 79	9.2 (3.4–19.2)	9.2 (3.3–19.2)	9.2 (3.4–18.8)	9.3 (3.6–20.1)	9.3 (3.6–20.0)
Marshall Islands	80 to 84	6.5 (2.4–13.9)	6.5 (2.3–13.9)	6.5 (2.3–13.6)	6.6 (2.5–14.6)	6.6 (2.5–14.5)
Marshall Islands	85 to 89	5.8 (2.1–12.6)	5.8 (2.0–12.6)	5.8 (2.1–12.3)	5.9 (2.2–13.2)	5.9 (2.2–13.1)
Marshall Islands	90 to 94	5.3 (1.9–11.4)	5.3 (1.8–11.4)	5.3 (1.9–11.2)	5.3 (2.0–12.0)	5.3 (2.0–11.9)

Supplementary Table S11: Prevalence of male SVAC by age and location for 1990, 2000, 2010, 2020, and 2023						
**Location**	**Age Range**	**1990**	**2000**	**2010**	**2020**	**2023**
Marshall Islands	95 plus	5.2 (1.9–11.3)	5.2 (1.8–11.3)	5.2 (1.9–11.1)	5.3 (2.0–11.9)	5.3 (2.0–11.8)
Marshall Islands	Age-standardized	13.4 (5.2–26.7)	13.4 (5.0–26.7)	13.4 (5.2–26.3)	13.5 (5.4–27.8)	13.5 (5.5–27.7)
Marshall Islands	All age	13.5 (5.3–27.0)	13.6 (5.1–27.1)	13.7 (5.3–26.8)	13.8 (5.6–28.5)	13.8 (5.6–28.3)
Federated States of Micronesia	20 to 24	12.2 (4.7–24.7)	12.2 (4.5–24.6)	12.2 (4.6–24.2)	12.2 (4.9–25.7)	12.3 (4.9–25.7)
Federated States of Micronesia	25 to 29	12.6 (4.9–25.5)	12.6 (4.7–25.4)	12.6 (4.8–25.0)	12.7 (5.1–26.5)	12.7 (5.1–26.3)
Federated States of Micronesia	30 to 34	14.1 (5.5–28.0)	14.1 (5.3–27.9)	14.1 (5.4–27.5)	14.2 (5.7–29.1)	14.2 (5.7–28.9)
Federated States of Micronesia	35 to 39	14.5 (5.7–28.7)	14.5 (5.5–28.7)	14.5 (5.6–28.3)	14.6 (5.9–29.9)	14.7 (6.0–29.7)
Federated States of Micronesia	40 to 44	15.3 (6.0–30.1)	15.3 (5.8–30.1)	15.3 (6.0–29.6)	15.5 (6.3–31.3)	15.5 (6.4–31.2)
Federated States of Micronesia	45 to 49	15.1 (5.9–29.7)	15.1 (5.7–29.6)	15.1 (5.9–29.2)	15.2 (6.2–30.9)	15.2 (6.2–30.7)
Federated States of Micronesia	50 to 54	14.7 (5.8–29.1)	14.7 (5.6–29.1)	14.7 (5.7–28.6)	14.9 (6.0–30.3)	14.9 (6.1–30.1)
Federated States of Micronesia	55 to 59	14.2 (5.6–28.3)	14.2 (5.4–28.2)	14.3 (5.5–27.8)	14.4 (5.8–29.5)	14.4 (5.8–29.3)
Federated States of Micronesia	60 to 64	13.8 (5.4–27.5)	13.8 (5.2–27.5)	13.8 (5.3–27.0)	13.9 (5.6–28.7)	13.9 (5.6–28.5)
Federated States of Micronesia	65 to 69	12.8 (4.9–25.8)	12.8 (4.8–25.8)	12.9 (4.9–25.4)	12.9 (5.2–26.9)	13.0 (5.2–26.7)
Federated States of Micronesia	70 to 74	10.2 (3.8–21.1)	10.2 (3.7–21.1)	10.3 (3.8–20.7)	10.3 (4.0–22.1)	10.3 (4.1–21.9)
Federated States of Micronesia	75 to 79	9.2 (3.4–19.2)	9.2 (3.3–19.2)	9.2 (3.4–18.8)	9.3 (3.6–20.1)	9.3 (3.6–20.0)

Supplementary Table S11: Prevalence of male SVAC by age and location for 1990, 2000, 2010, 2020, and 2023						
**Location**	**Age Range**	**1990**	**2000**	**2010**	**2020**	**2023**
Federated States of Micronesia	80 to 84	6.5 (2.4–13.9)	6.5 (2.3–13.9)	6.5 (2.3–13.6)	6.6 (2.5–14.6)	6.6 (2.5–14.5)
Federated States of Micronesia	85 to 89	5.8 (2.1–12.6)	5.8 (2.0–12.6)	5.8 (2.1–12.3)	5.9 (2.2–13.2)	5.9 (2.2–13.1)
Federated States of Micronesia	90 to 94	5.3 (1.9–11.4)	5.3 (1.8–11.4)	5.3 (1.9–11.2)	5.3 (2.0–12.0)	5.3 (2.0–11.9)
Federated States of Micronesia	95 plus	5.2 (1.9–11.3)	5.2 (1.8–11.3)	5.2 (1.9–11.1)	5.3 (2.0–11.9)	5.3 (2.0–11.8)
Federated States of Micronesia	Age-standardized	13.4 (5.2–26.7)	13.4 (5.0–26.7)	13.4 (5.2–26.3)	13.5 (5.4–27.8)	13.5 (5.5–27.7)
Federated States of Micronesia	All age	13.5 (5.3–27.0)	13.6 (5.1–27.0)	13.7 (5.3–26.8)	13.8 (5.5–28.4)	13.8 (5.6–28.2)
Nauru	20 to 24	12.2 (4.7–24.7)	12.2 (4.5–24.6)	12.2 (4.6–24.2)	12.2 (4.9–25.7)	12.3 (4.9–25.7)
Nauru	25 to 29	12.6 (4.9–25.5)	12.6 (4.7–25.4)	12.6 (4.8–25.0)	12.7 (5.1–26.5)	12.7 (5.1–26.3)
Nauru	30 to 34	14.1 (5.5–28.0)	14.1 (5.3–27.9)	14.1 (5.4–27.5)	14.2 (5.7–29.1)	14.2 (5.7–28.9)
Nauru	35 to 39	14.5 (5.7–28.7)	14.5 (5.5–28.7)	14.5 (5.6–28.3)	14.6 (5.9–29.9)	14.7 (6.0–29.7)
Nauru	40 to 44	15.3 (6.0–30.1)	15.3 (5.8–30.1)	15.3 (6.0–29.6)	15.5 (6.3–31.3)	15.5 (6.4–31.2)
Nauru	45 to 49	15.1 (5.9–29.7)	15.1 (5.7–29.6)	15.1 (5.9–29.2)	15.2 (6.2–30.9)	15.2 (6.2–30.7)
Nauru	50 to 54	14.7 (5.8–29.1)	14.7 (5.6–29.1)	14.7 (5.7–28.6)	14.9 (6.0–30.3)	14.9 (6.1–30.1)
Nauru	55 to 59	14.2 (5.6–28.3)	14.2 (5.4–28.2)	14.3 (5.5–27.8)	14.4 (5.8–29.5)	14.4 (5.8–29.3)
Nauru	60 to 64	13.8 (5.4–27.5)	13.8 (5.2–27.5)	13.8 (5.3–27.0)	13.9 (5.6–28.7)	13.9 (5.6–28.5)

Supplementary Table S11: Prevalence of male SVAC by age and location for 1990, 2000, 2010, 2020, and 2023						
**Location**	**Age Range**	**1990**	**2000**	**2010**	**2020**	**2023**
Nauru	65 to 69	12.8 (4.9–25.8)	12.8 (4.8–25.8)	12.9 (4.9–25.4)	12.9 (5.2–26.9)	13.0 (5.2–26.7)
Nauru	70 to 74	10.2 (3.8–21.1)	10.2 (3.7–21.1)	10.3 (3.8–20.7)	10.3 (4.0–22.1)	10.3 (4.1–21.9)
Nauru	75 to 79	9.2 (3.4–19.2)	9.2 (3.3–19.2)	9.2 (3.4–18.8)	9.3 (3.6–20.1)	9.3 (3.6–20.0)
Nauru	80 to 84	6.5 (2.4–13.9)	6.5 (2.3–13.9)	6.5 (2.3–13.6)	6.6 (2.5–14.6)	6.6 (2.5–14.5)
Nauru	85 to 89	5.8 (2.1–12.6)	5.8 (2.0–12.6)	5.8 (2.1–12.3)	5.9 (2.2–13.2)	5.9 (2.2–13.1)
Nauru	90 to 94	5.3 (1.9–11.4)	5.3 (1.8–11.4)	5.3 (1.9–11.2)	5.3 (2.0–12.0)	5.3 (2.0–11.9)
Nauru	95 plus	5.2 (1.9–11.3)	5.2 (1.8–11.3)	5.2 (1.9–11.1)	5.3 (2.0–11.9)	5.3 (2.0–11.8)
Nauru	Age-standardized	13.4 (5.2–26.7)	13.4 (5.0–26.7)	13.4 (5.2–26.3)	13.5 (5.4–27.8)	13.5 (5.5–27.7)
Nauru	All age	13.6 (5.3–27.2)	13.6 (5.1–27.1)	13.6 (5.3–26.7)	13.8 (5.5–28.3)	13.8 (5.6–28.2)
Niue	20 to 24	12.2 (4.7–24.7)	12.2 (4.5–24.6)	12.2 (4.6–24.2)	12.2 (4.9–25.7)	12.3 (4.9–25.7)
Niue	25 to 29	12.6 (4.9–25.5)	12.6 (4.7–25.4)	12.6 (4.8–25.0)	12.7 (5.1–26.5)	12.7 (5.1–26.3)
Niue	30 to 34	14.1 (5.5–28.0)	14.1 (5.3–27.9)	14.1 (5.4–27.5)	14.2 (5.7–29.1)	14.2 (5.7–28.9)
Niue	35 to 39	14.5 (5.7–28.7)	14.5 (5.5–28.7)	14.5 (5.6–28.3)	14.6 (5.9–29.9)	14.7 (6.0–29.7)
Niue	40 to 44	15.3 (6.0–30.1)	15.3 (5.8–30.1)	15.3 (6.0–29.6)	15.5 (6.3–31.3)	15.5 (6.4–31.2)
Niue	45 to 49	15.1 (5.9–29.7)	15.1 (5.7–29.6)	15.1 (5.9–29.2)	15.2 (6.2–30.9)	15.2 (6.2–30.7)

Supplementary Table S11: Prevalence of male SVAC by age and location for 1990, 2000, 2010, 2020, and 2023						
**Location**	**Age Range**	**1990**	**2000**	**2010**	**2020**	**2023**
Niue	50 to 54	14.7 (5.8–29.1)	14.7 (5.6–29.1)	14.7 (5.7–28.6)	14.9 (6.0–30.3)	14.9 (6.1–30.1)
Niue	55 to 59	14.2 (5.6–28.3)	14.2 (5.4–28.2)	14.3 (5.5–27.8)	14.4 (5.8–29.5)	14.4 (5.8–29.3)
Niue	60 to 64	13.8 (5.4–27.5)	13.8 (5.2–27.5)	13.8 (5.3–27.0)	13.9 (5.6–28.7)	13.9 (5.6–28.5)
Niue	65 to 69	12.8 (4.9–25.8)	12.8 (4.8–25.8)	12.9 (4.9–25.4)	12.9 (5.2–26.9)	13.0 (5.2–26.7)
Niue	70 to 74	10.2 (3.8–21.1)	10.2 (3.7–21.1)	10.3 (3.8–20.7)	10.3 (4.0–22.1)	10.3 (4.1–21.9)
Niue	75 to 79	9.2 (3.4–19.2)	9.2 (3.3–19.2)	9.2 (3.4–18.8)	9.3 (3.6–20.1)	9.3 (3.6–20.0)
Niue	80 to 84	6.5 (2.4–13.9)	6.5 (2.3–13.9)	6.5 (2.3–13.6)	6.6 (2.5–14.6)	6.6 (2.5–14.5)
Niue	85 to 89	5.8 (2.1–12.6)	5.8 (2.0–12.6)	5.8 (2.1–12.3)	5.9 (2.2–13.2)	5.9 (2.2–13.1)
Niue	90 to 94	5.3 (1.9–11.4)	5.3 (1.8–11.4)	5.3 (1.9–11.2)	5.3 (2.0–12.0)	5.3 (2.0–11.9)
Niue	95 plus	5.2 (1.9–11.3)	5.2 (1.8–11.3)	5.2 (1.9–11.1)	5.3 (2.0–11.9)	5.3 (2.0–11.8)
Niue	Age-standardized	13.4 (5.2–26.7)	13.4 (5.0–26.7)	13.4 (5.2–26.3)	13.5 (5.4–27.8)	13.5 (5.5–27.7)
Niue	All age	13.4 (5.2–26.8)	13.5 (5.1–27.0)	13.5 (5.2–26.5)	13.7 (5.5–28.3)	13.7 (5.5–28.0)
Northern Mariana Islands	20 to 24	12.2 (4.7–24.7)	12.2 (4.5–24.6)	12.2 (4.6–24.2)	12.2 (4.9–25.7)	12.3 (4.9–25.7)
Northern Mariana Islands	25 to 29	12.6 (4.9–25.5)	12.6 (4.7–25.4)	12.6 (4.8–25.0)	12.7 (5.1–26.5)	12.7 (5.1–26.3)
Northern Mariana Islands	30 to 34	14.1 (5.5–28.0)	14.1 (5.3–27.9)	14.1 (5.4–27.5)	14.2 (5.7–29.1)	14.2 (5.7–28.9)

Supplementary Table S11: Prevalence of male SVAC by age and location for 1990, 2000, 2010, 2020, and 2023						
**Location**	**Age Range**	**1990**	**2000**	**2010**	**2020**	**2023**
Northern Mariana Islands	35 to 39	14.5 (5.7–28.7)	14.5 (5.5–28.7)	14.5 (5.6–28.3)	14.6 (5.9–29.9)	14.7 (6.0–29.7)
Northern Mariana Islands	40 to 44	15.3 (6.0–30.1)	15.3 (5.8–30.1)	15.3 (6.0–29.6)	15.5 (6.3–31.3)	15.5 (6.4–31.2)
Northern Mariana Islands	45 to 49	15.1 (5.9–29.7)	15.1 (5.7–29.6)	15.1 (5.9–29.2)	15.2 (6.2–30.9)	15.2 (6.2–30.7)
Northern Mariana Islands	50 to 54	14.7 (5.8–29.1)	14.7 (5.6–29.1)	14.7 (5.7–28.6)	14.9 (6.0–30.3)	14.9 (6.1–30.1)
Northern Mariana Islands	55 to 59	14.2 (5.6–28.3)	14.2 (5.4–28.2)	14.3 (5.5–27.8)	14.4 (5.8–29.5)	14.4 (5.8–29.3)
Northern Mariana Islands	60 to 64	13.8 (5.4–27.5)	13.8 (5.2–27.5)	13.8 (5.3–27.0)	13.9 (5.6–28.7)	13.9 (5.6–28.5)
Northern Mariana Islands	65 to 69	12.8 (4.9–25.8)	12.8 (4.8–25.8)	12.9 (4.9–25.4)	12.9 (5.2–26.9)	13.0 (5.2–26.7)
Northern Mariana Islands	70 to 74	10.2 (3.8–21.1)	10.2 (3.7–21.1)	10.3 (3.8–20.7)	10.3 (4.0–22.1)	10.3 (4.1–21.9)
Northern Mariana Islands	75 to 79	9.2 (3.4–19.2)	9.2 (3.3–19.2)	9.2 (3.4–18.8)	9.3 (3.6–20.1)	9.3 (3.6–20.0)
Northern Mariana Islands	80 to 84	6.5 (2.4–13.9)	6.5 (2.3–13.9)	6.5 (2.3–13.6)	6.6 (2.5–14.6)	6.6 (2.5–14.5)
Northern Mariana Islands	85 to 89	5.8 (2.1–12.6)	5.8 (2.0–12.6)	5.8 (2.1–12.3)	5.9 (2.2–13.2)	5.9 (2.2–13.1)
Northern Mariana Islands	90 to 94	5.3 (1.9–11.4)	5.3 (1.8–11.4)	5.3 (1.9–11.2)	5.3 (2.0–12.0)	5.3 (2.0–11.9)
Northern Mariana Islands	95 plus	5.2 (1.9–11.3)	5.2 (1.8–11.3)	5.2 (1.9–11.1)	5.3 (2.0–11.9)	5.3 (2.0–11.8)
Northern Mariana Islands	Age-standardized	13.4 (5.2–26.7)	13.4 (5.0–26.7)	13.4 (5.2–26.3)	13.5 (5.4–27.8)	13.5 (5.5–27.7)
Northern Mariana Islands	All age	13.8 (5.4–27.6)	13.9 (5.2–27.7)	14.2 (5.5–27.6)	13.9 (5.6–28.6)	13.8 (5.6–28.3)

Supplementary Table S11: Prevalence of male SVAC by age and location for 1990, 2000, 2010, 2020, and 2023						
**Location**	**Age Range**	**1990**	**2000**	**2010**	**2020**	**2023**
Palau	20 to 24	12.2 (4.7–24.7)	12.2 (4.5–24.6)	12.2 (4.6–24.2)	12.2 (4.9–25.7)	12.3 (4.9–25.7)
Palau	25 to 29	12.6 (4.9–25.5)	12.6 (4.7–25.4)	12.6 (4.8–25.0)	12.7 (5.1–26.5)	12.7 (5.1–26.3)
Palau	30 to 34	14.1 (5.5–28.0)	14.1 (5.3–27.9)	14.1 (5.4–27.5)	14.2 (5.7–29.1)	14.2 (5.7–28.9)
Palau	35 to 39	14.5 (5.7–28.7)	14.5 (5.5–28.7)	14.5 (5.6–28.3)	14.6 (5.9–29.9)	14.7 (6.0–29.7)
Palau	40 to 44	15.3 (6.0–30.1)	15.3 (5.8–30.1)	15.3 (6.0–29.6)	15.5 (6.3–31.3)	15.5 (6.4–31.2)
Palau	45 to 49	15.1 (5.9–29.7)	15.1 (5.7–29.6)	15.1 (5.9–29.2)	15.2 (6.2–30.9)	15.2 (6.2–30.7)
Palau	50 to 54	14.7 (5.8–29.1)	14.7 (5.6–29.1)	14.7 (5.7–28.6)	14.9 (6.0–30.3)	14.9 (6.1–30.1)
Palau	55 to 59	14.2 (5.6–28.3)	14.2 (5.4–28.2)	14.3 (5.5–27.8)	14.4 (5.8–29.5)	14.4 (5.8–29.3)
Palau	60 to 64	13.8 (5.4–27.5)	13.8 (5.2–27.5)	13.8 (5.3–27.0)	13.9 (5.6–28.7)	13.9 (5.6–28.5)
Palau	65 to 69	12.8 (4.9–25.8)	12.8 (4.8–25.8)	12.9 (4.9–25.4)	12.9 (5.2–26.9)	13.0 (5.2–26.7)
Palau	70 to 74	10.2 (3.8–21.1)	10.2 (3.7–21.1)	10.3 (3.8–20.7)	10.3 (4.0–22.1)	10.3 (4.1–21.9)
Palau	75 to 79	9.2 (3.4–19.2)	9.2 (3.3–19.2)	9.2 (3.4–18.8)	9.3 (3.6–20.1)	9.3 (3.6–20.0)
Palau	80 to 84	6.5 (2.4–13.9)	6.5 (2.3–13.9)	6.5 (2.3–13.6)	6.6 (2.5–14.6)	6.6 (2.5–14.5)
Palau	85 to 89	5.8 (2.1–12.6)	5.8 (2.0–12.6)	5.8 (2.1–12.3)	5.9 (2.2–13.2)	5.9 (2.2–13.1)
Palau	90 to 94	5.3 (1.9–11.4)	5.3 (1.8–11.4)	5.3 (1.9–11.2)	5.3 (2.0–12.0)	5.3 (2.0–11.9)

Supplementary Table S11: Prevalence of male SVAC by age and location for 1990, 2000, 2010, 2020, and 2023						
**Location**	**Age Range**	**1990**	**2000**	**2010**	**2020**	**2023**
Palau	95 plus	5.2 (1.9–11.3)	5.2 (1.8–11.3)	5.2 (1.9–11.1)	5.3 (2.0–11.9)	5.3 (2.0–11.8)
Palau	Age-standardized	13.4 (5.2–26.7)	13.4 (5.0–26.7)	13.4 (5.2–26.3)	13.5 (5.4–27.8)	13.5 (5.5–27.7)
Palau	All age	13.5 (5.3–27.0)	13.8 (5.2–27.4)	13.8 (5.3–27.0)	13.8 (5.6–28.5)	13.8 (5.6–28.2)
Papua New Guinea	20 to 24	10.8 (4.3–21.8)	9.8 (4.2–18.8)	9.2 (4.6–15.9)	9.7 (4.5–18.2)	10.0 (4.3–19.4)
Papua New Guinea	25 to 29	11.2 (4.5–22.5)	10.2 (4.4–19.3)	9.5 (4.8–16.3)	10.1 (4.7–18.8)	10.4 (4.4–20.0)
Papua New Guinea	30 to 34	14.5 (6.1–27.5)	14.8 (7.3–25.2)	14.9 (9.6–21.4)	15.0 (8.0–24.4)	14.9 (7.1–26.1)
Papua New Guinea	35 to 39	14.3 (5.8–27.3)	14.0 (6.7–24.1)	13.7 (8.5–20.3)	14.0 (7.4–23.3)	14.1 (6.6–24.8)
Papua New Guinea	40 to 44	17.1 (7.3–31.7)	18.4 (9.4–30.7)	19.4 (12.9–27.1)	18.9 (10.4–29.9)	18.5 (9.0–31.6)
Papua New Guinea	45 to 49	16.1 (6.8–30.2)	16.8 (8.4–28.3)	17.3 (11.3–24.5)	17.1 (9.2–27.5)	16.9 (8.1–29.1)
Papua New Guinea	50 to 54	14.6 (5.7–28.9)	14.6 (5.5–28.8)	14.6 (5.7–28.4)	14.7 (6.0–30.0)	14.7 (6.0–29.9)
Papua New Guinea	55 to 59	14.1 (5.5–28.1)	14.1 (5.3–28.1)	14.2 (5.5–27.6)	14.3 (5.8–29.2)	14.3 (5.8–29.0)
Papua New Guinea	60 to 64	13.7 (5.3–27.3)	13.7 (5.1–27.3)	13.7 (5.3–26.9)	13.8 (5.6–28.5)	13.8 (5.6–28.3)
Papua New Guinea	65 to 69	12.7 (4.9–25.7)	12.7 (4.7–25.6)	12.8 (4.9–25.2)	12.8 (5.1–26.7)	12.9 (5.1–26.6)
Papua New Guinea	70 to 74	10.2 (3.8–21.0)	10.2 (3.7–21.0)	10.2 (3.8–20.6)	10.3 (4.0–21.9)	10.3 (4.0–21.8)
Papua New Guinea	75 to 79	9.2 (3.4–19.1)	9.1 (3.3–19.0)	9.2 (3.4–18.7)	9.2 (3.6–20.0)	9.2 (3.6–19.8)

Supplementary Table S11: Prevalence of male SVAC by age and location for 1990, 2000, 2010, 2020, and 2023						
**Location**	**Age Range**	**1990**	**2000**	**2010**	**2020**	**2023**
Papua New Guinea	80 to 84	6.5 (2.3–13.8)	6.4 (2.2–13.8)	6.4 (2.3–13.5)	6.5 (2.4–14.5)	6.5 (2.5–14.4)
Papua New Guinea	85 to 89	5.8 (2.1–12.5)	5.8 (2.0–12.5)	5.8 (2.1–12.2)	5.8 (2.2–13.1)	5.9 (2.2–13.1)
Papua New Guinea	90 to 94	5.2 (1.9–11.3)	5.2 (1.8–11.3)	5.2 (1.9–11.1)	5.3 (2.0–11.9)	5.3 (2.0–11.8)
Papua New Guinea	95 plus	5.2 (1.9–11.2)	5.2 (1.8–11.2)	5.2 (1.8–11.0)	5.2 (1.9–11.8)	5.2 (2.0–11.7)
Papua New Guinea	Age-standardized	13.3 (5.5–25.8)	13.2 (6.0–24.3)	13.2 (7.4–21.5)	13.4 (6.8–23.5)	13.4 (6.2–24.1)
Papua New Guinea	All age	13.3 (5.5–25.8)	13.2 (6.0–23.9)	13.2 (7.6–20.6)	13.4 (6.9–23.2)	13.5 (6.2–24.3)
Samoa	20 to 24	12.2 (4.7–24.7)	12.2 (4.5–24.6)	12.2 (4.6–24.2)	12.2 (4.9–25.7)	12.3 (4.9–25.7)
Samoa	25 to 29	12.6 (4.9–25.5)	12.6 (4.7–25.4)	12.6 (4.8–25.0)	12.7 (5.1–26.5)	12.7 (5.1–26.3)
Samoa	30 to 34	14.1 (5.5–28.0)	14.1 (5.3–27.9)	14.1 (5.4–27.5)	14.2 (5.7–29.1)	14.2 (5.7–28.9)
Samoa	35 to 39	14.5 (5.7–28.7)	14.5 (5.5–28.7)	14.5 (5.6–28.3)	14.6 (5.9–29.9)	14.7 (6.0–29.7)
Samoa	40 to 44	15.3 (6.0–30.1)	15.3 (5.8–30.1)	15.3 (6.0–29.6)	15.5 (6.3–31.3)	15.5 (6.4–31.2)
Samoa	45 to 49	15.1 (5.9–29.7)	15.1 (5.7–29.6)	15.1 (5.9–29.2)	15.2 (6.2–30.9)	15.2 (6.2–30.7)
Samoa	50 to 54	14.7 (5.8–29.1)	14.7 (5.6–29.1)	14.7 (5.7–28.6)	14.9 (6.0–30.3)	14.9 (6.1–30.1)
Samoa	55 to 59	14.2 (5.6–28.3)	14.2 (5.4–28.2)	14.3 (5.5–27.8)	14.4 (5.8–29.5)	14.4 (5.8–29.3)
Samoa	60 to 64	13.8 (5.4–27.5)	13.8 (5.2–27.5)	13.8 (5.3–27.0)	13.9 (5.6–28.7)	13.9 (5.6–28.5)

Supplementary Table S11: Prevalence of male SVAC by age and location for 1990, 2000, 2010, 2020, and 2023						
**Location**	**Age Range**	**1990**	**2000**	**2010**	**2020**	**2023**
Samoa	65 to 69	12.8 (4.9–25.8)	12.8 (4.8–25.8)	12.9 (4.9–25.4)	12.9 (5.2–26.9)	13.0 (5.2–26.7)
Samoa	70 to 74	10.2 (3.8–21.1)	10.2 (3.7–21.1)	10.3 (3.8–20.7)	10.3 (4.0–22.1)	10.3 (4.1–21.9)
Samoa	75 to 79	9.2 (3.4–19.2)	9.2 (3.3–19.2)	9.2 (3.4–18.8)	9.3 (3.6–20.1)	9.3 (3.6–20.0)
Samoa	80 to 84	6.5 (2.4–13.9)	6.5 (2.3–13.9)	6.5 (2.3–13.6)	6.6 (2.5–14.6)	6.6 (2.5–14.5)
Samoa	85 to 89	5.8 (2.1–12.6)	5.8 (2.0–12.6)	5.8 (2.1–12.3)	5.9 (2.2–13.2)	5.9 (2.2–13.1)
Samoa	90 to 94	5.3 (1.9–11.4)	5.3 (1.8–11.4)	5.3 (1.9–11.2)	5.3 (2.0–12.0)	5.3 (2.0–11.9)
Samoa	95 plus	5.2 (1.9–11.3)	5.2 (1.8–11.3)	5.2 (1.9–11.1)	5.3 (2.0–11.9)	5.3 (2.0–11.8)
Samoa	Age-standardized	13.4 (5.2–26.7)	13.4 (5.0–26.7)	13.4 (5.2–26.3)	13.5 (5.4–27.8)	13.5 (5.5–27.7)
Samoa	All age	13.4 (5.2–26.8)	13.5 (5.0–26.9)	13.6 (5.3–26.6)	13.7 (5.5–28.2)	13.7 (5.5–28.0)
Solomon Islands	20 to 24	12.2 (4.7–24.7)	12.2 (4.5–24.6)	12.2 (4.6–24.2)	12.2 (4.9–25.7)	12.3 (4.9–25.7)
Solomon Islands	25 to 29	12.6 (4.9–25.5)	12.6 (4.7–25.4)	12.6 (4.8–25.0)	12.7 (5.1–26.5)	12.7 (5.1–26.3)
Solomon Islands	30 to 34	14.1 (5.5–28.0)	14.1 (5.3–27.9)	14.1 (5.4–27.5)	14.2 (5.7–29.1)	14.2 (5.7–28.9)
Solomon Islands	35 to 39	14.5 (5.7–28.7)	14.5 (5.5–28.7)	14.5 (5.6–28.3)	14.6 (5.9–29.9)	14.7 (6.0–29.7)
Solomon Islands	40 to 44	15.3 (6.0–30.1)	15.3 (5.8–30.1)	15.3 (6.0–29.6)	15.5 (6.3–31.3)	15.5 (6.4–31.2)
Solomon Islands	45 to 49	15.1 (5.9–29.7)	15.1 (5.7–29.6)	15.1 (5.9–29.2)	15.2 (6.2–30.9)	15.2 (6.2–30.7)

Supplementary Table S11: Prevalence of male SVAC by age and location for 1990, 2000, 2010, 2020, and 2023						
**Location**	**Age Range**	**1990**	**2000**	**2010**	**2020**	**2023**
Solomon Islands	50 to 54	14.7 (5.8–29.1)	14.7 (5.6–29.1)	14.7 (5.7–28.6)	14.9 (6.0–30.3)	14.9 (6.1–30.1)
Solomon Islands	55 to 59	14.2 (5.6–28.3)	14.2 (5.4–28.2)	14.3 (5.5–27.8)	14.4 (5.8–29.5)	14.4 (5.8–29.3)
Solomon Islands	60 to 64	13.8 (5.4–27.5)	13.8 (5.2–27.5)	13.8 (5.3–27.0)	13.9 (5.6–28.7)	13.9 (5.6–28.5)
Solomon Islands	65 to 69	12.8 (4.9–25.8)	12.8 (4.8–25.8)	12.9 (4.9–25.4)	12.9 (5.2–26.9)	13.0 (5.2–26.7)
Solomon Islands	70 to 74	10.2 (3.8–21.1)	10.2 (3.7–21.1)	10.3 (3.8–20.7)	10.3 (4.0–22.1)	10.3 (4.1–21.9)
Solomon Islands	75 to 79	9.2 (3.4–19.2)	9.2 (3.3–19.2)	9.2 (3.4–18.8)	9.3 (3.6–20.1)	9.3 (3.6–20.0)
Solomon Islands	80 to 84	6.5 (2.4–13.9)	6.5 (2.3–13.9)	6.5 (2.3–13.6)	6.6 (2.5–14.6)	6.6 (2.5–14.5)
Solomon Islands	85 to 89	5.8 (2.1–12.6)	5.8 (2.0–12.6)	5.8 (2.1–12.3)	5.9 (2.2–13.2)	5.9 (2.2–13.1)
Solomon Islands	90 to 94	5.3 (1.9–11.4)	5.3 (1.8–11.4)	5.3 (1.9–11.2)	5.3 (2.0–12.0)	5.3 (2.0–11.9)
Solomon Islands	95 plus	5.2 (1.9–11.3)	5.2 (1.8–11.3)	5.2 (1.9–11.1)	5.3 (2.0–11.9)	5.3 (2.0–11.8)
Solomon Islands	Age-standardized	13.4 (5.2–26.7)	13.4 (5.0–26.7)	13.4 (5.2–26.3)	13.5 (5.4–27.8)	13.5 (5.5–27.7)
Solomon Islands	All age	13.5 (5.2–27.0)	13.4 (5.0–26.8)	13.6 (5.2–26.6)	13.8 (5.5–28.3)	13.8 (5.6–28.2)
Tokelau	20 to 24	12.2 (4.7–24.7)	12.2 (4.5–24.6)	12.2 (4.6–24.2)	12.2 (4.9–25.7)	12.3 (4.9–25.7)
Tokelau	25 to 29	12.6 (4.9–25.5)	12.6 (4.7–25.4)	12.6 (4.8–25.0)	12.7 (5.1–26.5)	12.7 (5.1–26.3)
Tokelau	30 to 34	14.1 (5.5–28.0)	14.1 (5.3–27.9)	14.1 (5.4–27.5)	14.2 (5.7–29.1)	14.2 (5.7–28.9)

Supplementary Table S11: Prevalence of male SVAC by age and location for 1990, 2000, 2010, 2020, and 2023						
**Location**	**Age Range**	**1990**	**2000**	**2010**	**2020**	**2023**
Tokelau	35 to 39	14.5 (5.7–28.7)	14.5 (5.5–28.7)	14.5 (5.6–28.3)	14.6 (5.9–29.9)	14.7 (6.0–29.7)
Tokelau	40 to 44	15.3 (6.0–30.1)	15.3 (5.8–30.1)	15.3 (6.0–29.6)	15.5 (6.3–31.3)	15.5 (6.4–31.2)
Tokelau	45 to 49	15.1 (5.9–29.7)	15.1 (5.7–29.6)	15.1 (5.9–29.2)	15.2 (6.2–30.9)	15.2 (6.2–30.7)
Tokelau	50 to 54	14.7 (5.8–29.1)	14.7 (5.6–29.1)	14.7 (5.7–28.6)	14.9 (6.0–30.3)	14.9 (6.1–30.1)
Tokelau	55 to 59	14.2 (5.6–28.3)	14.2 (5.4–28.2)	14.3 (5.5–27.8)	14.4 (5.8–29.5)	14.4 (5.8–29.3)
Tokelau	60 to 64	13.8 (5.4–27.5)	13.8 (5.2–27.5)	13.8 (5.3–27.0)	13.9 (5.6–28.7)	13.9 (5.6–28.5)
Tokelau	65 to 69	12.8 (4.9–25.8)	12.8 (4.8–25.8)	12.9 (4.9–25.4)	12.9 (5.2–26.9)	13.0 (5.2–26.7)
Tokelau	70 to 74	10.2 (3.8–21.1)	10.2 (3.7–21.1)	10.3 (3.8–20.7)	10.3 (4.0–22.1)	10.3 (4.1–21.9)
Tokelau	75 to 79	9.2 (3.4–19.2)	9.2 (3.3–19.2)	9.2 (3.4–18.8)	9.3 (3.6–20.1)	9.3 (3.6–20.0)
Tokelau	80 to 84	6.5 (2.4–13.9)	6.5 (2.3–13.9)	6.5 (2.3–13.6)	6.6 (2.5–14.6)	6.6 (2.5–14.5)
Tokelau	85 to 89	5.8 (2.1–12.6)	5.8 (2.0–12.6)	5.8 (2.1–12.3)	5.9 (2.2–13.2)	5.9 (2.2–13.1)
Tokelau	90 to 94	5.3 (1.9–11.4)	5.3 (1.8–11.4)	5.3 (1.9–11.2)	5.3 (2.0–12.0)	5.3 (2.0–11.9)
Tokelau	95 plus	5.2 (1.9–11.3)	5.2 (1.8–11.3)	5.2 (1.9–11.1)	5.3 (2.0–11.9)	5.3 (2.0–11.8)
Tokelau	Age-standardized	13.4 (5.2–26.7)	13.4 (5.0–26.7)	13.4 (5.2–26.3)	13.5 (5.4–27.8)	13.5 (5.5–27.7)
Tokelau	All age	13.3 (5.2–26.6)	13.2 (4.9–26.4)	13.3 (5.1–26.1)	13.5 (5.4–27.8)	13.5 (5.5–27.7)

Supplementary Table S11: Prevalence of male SVAC by age and location for 1990, 2000, 2010, 2020, and 2023						
**Location**	**Age Range**	**1990**	**2000**	**2010**	**2020**	**2023**
Tonga	20 to 24	12.2 (4.7–24.7)	12.2 (4.5–24.6)	12.2 (4.6–24.2)	12.2 (4.9–25.7)	12.3 (4.9–25.7)
Tonga	25 to 29	12.6 (4.9–25.5)	12.6 (4.7–25.4)	12.6 (4.8–25.0)	12.7 (5.1–26.5)	12.7 (5.1–26.3)
Tonga	30 to 34	14.1 (5.5–28.0)	14.1 (5.3–27.9)	14.1 (5.4–27.5)	14.2 (5.7–29.1)	14.2 (5.7–28.9)
Tonga	35 to 39	14.5 (5.7–28.7)	14.5 (5.5–28.7)	14.5 (5.6–28.3)	14.6 (5.9–29.9)	14.7 (6.0–29.7)
Tonga	40 to 44	15.3 (6.0–30.1)	15.3 (5.8–30.1)	15.3 (6.0–29.6)	15.5 (6.3–31.3)	15.5 (6.4–31.2)
Tonga	45 to 49	15.1 (5.9–29.7)	15.1 (5.7–29.6)	15.1 (5.9–29.2)	15.2 (6.2–30.9)	15.2 (6.2–30.7)
Tonga	50 to 54	14.7 (5.8–29.1)	14.7 (5.6–29.1)	14.7 (5.7–28.6)	14.9 (6.0–30.3)	14.9 (6.1–30.1)
Tonga	55 to 59	14.2 (5.6–28.3)	14.2 (5.4–28.2)	14.3 (5.5–27.8)	14.4 (5.8–29.5)	14.4 (5.8–29.3)
Tonga	60 to 64	13.8 (5.4–27.5)	13.8 (5.2–27.5)	13.8 (5.3–27.0)	13.9 (5.6–28.7)	13.9 (5.6–28.5)
Tonga	65 to 69	12.8 (4.9–25.8)	12.8 (4.8–25.8)	12.9 (4.9–25.4)	12.9 (5.2–26.9)	13.0 (5.2–26.7)
Tonga	70 to 74	10.2 (3.8–21.1)	10.2 (3.7–21.1)	10.3 (3.8–20.7)	10.3 (4.0–22.1)	10.3 (4.1–21.9)
Tonga	75 to 79	9.2 (3.4–19.2)	9.2 (3.3–19.2)	9.2 (3.4–18.8)	9.3 (3.6–20.1)	9.3 (3.6–20.0)
Tonga	80 to 84	6.5 (2.4–13.9)	6.5 (2.3–13.9)	6.5 (2.3–13.6)	6.6 (2.5–14.6)	6.6 (2.5–14.5)
Tonga	85 to 89	5.8 (2.1–12.6)	5.8 (2.0–12.6)	5.8 (2.1–12.3)	5.9 (2.2–13.2)	5.9 (2.2–13.1)
Tonga	90 to 94	5.3 (1.9–11.4)	5.3 (1.8–11.4)	5.3 (1.9–11.2)	5.3 (2.0–12.0)	5.3 (2.0–11.9)

Supplementary Table S11: Prevalence of male SVAC by age and location for 1990, 2000, 2010, 2020, and 2023						
**Location**	**Age Range**	**1990**	**2000**	**2010**	**2020**	**2023**
Tonga	95 plus	5.2 (1.9–11.3)	5.2 (1.8–11.3)	5.2 (1.9–11.1)	5.3 (2.0–11.9)	5.3 (2.0–11.8)
Tonga	Age-standardized	13.4 (5.2–26.7)	13.4 (5.0–26.7)	13.4 (5.2–26.3)	13.5 (5.4–27.8)	13.5 (5.5–27.7)
Tonga	All age	13.4 (5.2–26.7)	13.4 (5.0–26.7)	13.5 (5.2–26.4)	13.6 (5.5–28.0)	13.6 (5.5–27.8)
Tuvalu	20 to 24	12.2 (4.7–24.7)	12.2 (4.5–24.6)	12.2 (4.6–24.2)	12.2 (4.9–25.7)	12.3 (4.9–25.7)
Tuvalu	25 to 29	12.6 (4.9–25.5)	12.6 (4.7–25.4)	12.6 (4.8–25.0)	12.7 (5.1–26.5)	12.7 (5.1–26.3)
Tuvalu	30 to 34	14.1 (5.5–28.0)	14.1 (5.3–27.9)	14.1 (5.4–27.5)	14.2 (5.7–29.1)	14.2 (5.7–28.9)
Tuvalu	35 to 39	14.5 (5.7–28.7)	14.5 (5.5–28.7)	14.5 (5.6–28.3)	14.6 (5.9–29.9)	14.7 (6.0–29.7)
Tuvalu	40 to 44	15.3 (6.0–30.1)	15.3 (5.8–30.1)	15.3 (6.0–29.6)	15.5 (6.3–31.3)	15.5 (6.4–31.2)
Tuvalu	45 to 49	15.1 (5.9–29.7)	15.1 (5.7–29.6)	15.1 (5.9–29.2)	15.2 (6.2–30.9)	15.2 (6.2–30.7)
Tuvalu	50 to 54	14.7 (5.8–29.1)	14.7 (5.6–29.1)	14.7 (5.7–28.6)	14.9 (6.0–30.3)	14.9 (6.1–30.1)
Tuvalu	55 to 59	14.2 (5.6–28.3)	14.2 (5.4–28.2)	14.3 (5.5–27.8)	14.4 (5.8–29.5)	14.4 (5.8–29.3)
Tuvalu	60 to 64	13.8 (5.4–27.5)	13.8 (5.2–27.5)	13.8 (5.3–27.0)	13.9 (5.6–28.7)	13.9 (5.6–28.5)
Tuvalu	65 to 69	12.8 (4.9–25.8)	12.8 (4.8–25.8)	12.9 (4.9–25.4)	12.9 (5.2–26.9)	13.0 (5.2–26.7)
Tuvalu	70 to 74	10.2 (3.8–21.1)	10.2 (3.7–21.1)	10.3 (3.8–20.7)	10.3 (4.0–22.1)	10.3 (4.1–21.9)
Tuvalu	75 to 79	9.2 (3.4–19.2)	9.2 (3.3–19.2)	9.2 (3.4–18.8)	9.3 (3.6–20.1)	9.3 (3.6–20.0)

**Supplementary Table S11: Prevalence of male SVAC by age and location for 1990, 2000, 2010, 2020, and 2023**						
**Location**	**Age Range**	**1990**	**2000**	**2010**	**2020**	**2023**
Tuvalu	80 to 84	6.5 (2.4–13.9)	6.5 (2.3–13.9)	6.5 (2.3–13.6)	6.6 (2.5–14.6)	6.6 (2.5–14.5)
Tuvalu	85 to 89	5.8 (2.1–12.6)	5.8 (2.0–12.6)	5.8 (2.1–12.3)	5.9 (2.2–13.2)	5.9 (2.2–13.1)
Tuvalu	90 to 94	5.3 (1.9–11.4)	5.3 (1.8–11.4)	5.3 (1.9–11.2)	5.3 (2.0–12.0)	5.3 (2.0–11.9)
Tuvalu	95 plus	5.2 (1.9–11.3)	5.2 (1.8–11.3)	5.2 (1.9–11.1)	5.3 (2.0–11.9)	5.3 (2.0–11.8)
Tuvalu	Age-standardized	13.4 (5.2–26.7)	13.4 (5.0–26.7)	13.4 (5.2–26.3)	13.5 (5.4–27.8)	13.5 (5.5–27.7)
Tuvalu	All age	13.6 (5.3–27.1)	13.5 (5.1–27.0)	13.6 (5.2–26.6)	13.6 (5.5–28.0)	13.6 (5.5–27.9)
Vanuatu	20 to 24	12.2 (4.7–24.7)	12.2 (4.5–24.6)	12.2 (4.6–24.2)	12.2 (4.9–25.7)	12.3 (4.9–25.7)
Vanuatu	25 to 29	12.6 (4.9–25.5)	12.6 (4.7–25.4)	12.6 (4.8–25.0)	12.7 (5.1–26.5)	12.7 (5.1–26.3)
Vanuatu	30 to 34	14.1 (5.5–28.0)	14.1 (5.3–27.9)	14.1 (5.4–27.5)	14.2 (5.7–29.1)	14.2 (5.7–28.9)
Vanuatu	35 to 39	14.5 (5.7–28.7)	14.5 (5.5–28.7)	14.5 (5.6–28.3)	14.6 (5.9–29.9)	14.7 (6.0–29.7)
Vanuatu	40 to 44	15.3 (6.0–30.1)	15.3 (5.8–30.1)	15.3 (6.0–29.6)	15.5 (6.3–31.3)	15.5 (6.4–31.2)
Vanuatu	45 to 49	15.1 (5.9–29.7)	15.1 (5.7–29.6)	15.1 (5.9–29.2)	15.2 (6.2–30.9)	15.2 (6.2–30.7)
Vanuatu	50 to 54	14.7 (5.8–29.1)	14.7 (5.6–29.1)	14.7 (5.7–28.6)	14.9 (6.0–30.3)	14.9 (6.1–30.1)
Vanuatu	55 to 59	14.2 (5.6–28.3)	14.2 (5.4–28.2)	14.3 (5.5–27.8)	14.4 (5.8–29.5)	14.4 (5.8–29.3)
Vanuatu	60 to 64	13.8 (5.4–27.5)	13.8 (5.2–27.5)	13.8 (5.3–27.0)	13.9 (5.6–28.7)	13.9 (5.6–28.5)

Supplementary Table S11: Prevalence of male SVAC by age and location for 1990, 2000, 2010, 2020, and 2023						
**Location**	**Age Range**	**1990**	**2000**	**2010**	**2020**	**2023**
Vanuatu	65 to 69	12.8 (4.9–25.8)	12.8 (4.8–25.8)	12.9 (4.9–25.4)	12.9 (5.2–26.9)	13.0 (5.2–26.7)
Vanuatu	70 to 74	10.2 (3.8–21.1)	10.2 (3.7–21.1)	10.3 (3.8–20.7)	10.3 (4.0–22.1)	10.3 (4.1–21.9)
Vanuatu	75 to 79	9.2 (3.4–19.2)	9.2 (3.3–19.2)	9.2 (3.4–18.8)	9.3 (3.6–20.1)	9.3 (3.6–20.0)
Vanuatu	80 to 84	6.5 (2.4–13.9)	6.5 (2.3–13.9)	6.5 (2.3–13.6)	6.6 (2.5–14.6)	6.6 (2.5–14.5)
Vanuatu	85 to 89	5.8 (2.1–12.6)	5.8 (2.0–12.6)	5.8 (2.1–12.3)	5.9 (2.2–13.2)	5.9 (2.2–13.1)
Vanuatu	90 to 94	5.3 (1.9–11.4)	5.3 (1.8–11.4)	5.3 (1.9–11.2)	5.3 (2.0–12.0)	5.3 (2.0–11.9)
Vanuatu	95 plus	5.2 (1.9–11.3)	5.2 (1.8–11.3)	5.2 (1.9–11.1)	5.3 (2.0–11.9)	5.3 (2.0–11.8)
Vanuatu	Age-standardized	13.4 (5.2–26.7)	13.4 (5.0–26.7)	13.4 (5.2–26.3)	13.5 (5.4–27.8)	13.5 (5.5–27.7)
Vanuatu	All age	13.6 (5.3–27.1)	13.6 (5.1–27.0)	13.6 (5.3–26.6)	13.7 (5.5–28.2)	13.7 (5.6–28.1)
Southeast Asia	20 to 24	12.9 (5.5–25.2)	12.6 (5.3–24.1)	12.5 (6.0–22.1)	12.5 (6.1–23.3)	12.6 (5.8–23.8)
Southeast Asia	25 to 29	13.2 (5.6–25.8)	12.9 (5.4–24.6)	12.7 (6.1–22.4)	12.9 (6.3–23.9)	12.9 (5.9–24.3)
Southeast Asia	30 to 34	15.0 (6.5–28.5)	15.0 (6.6–28.0)	14.9 (7.7–25.5)	15.1 (7.5–26.9)	15.1 (7.1–27.5)
Southeast Asia	35 to 39	15.0 (6.5–28.6)	14.9 (6.4–27.9)	14.6 (7.2–25.4)	14.9 (7.2–26.9)	14.9 (6.9–27.6)
Southeast Asia	40 to 44	16.5 (7.3–30.5)	17.0 (7.7–31.0)	17.3 (9.4–28.4)	17.3 (9.1–29.7)	17.2 (8.5–30.4)
Southeast Asia	45 to 49	16.0 (7.1–29.6)	16.2 (7.3–29.8)	16.4 (8.5–27.6)	16.4 (8.4–28.6)	16.3 (7.8–29.2)

Supplementary Table S11: Prevalence of male SVAC by age and location for 1990, 2000, 2010, 2020, and 2023						
**Location**	**Age Range**	**1990**	**2000**	**2010**	**2020**	**2023**
Southeast Asia	50 to 54	15.0 (6.0–29.1)	15.0 (5.8–29.4)	15.1 (6.0–29.1)	15.1 (6.2–30.6)	15.1 (6.3–30.6)
Southeast Asia	55 to 59	14.7 (5.9–28.6)	14.6 (5.5–28.6)	14.6 (5.7–28.3)	14.7 (6.0–30.0)	14.7 (6.1–29.8)
Southeast Asia	60 to 64	14.5 (5.8–28.2)	14.4 (5.5–28.3)	14.4 (5.7–27.9)	14.5 (5.9–29.5)	14.5 (6.0–29.3)
Southeast Asia	65 to 69	13.3 (5.2–26.3)	13.4 (5.0–26.7)	13.3 (5.1–26.0)	13.4 (5.4–27.7)	13.4 (5.5–27.6)
Southeast Asia	70 to 74	10.7 (4.1–21.7)	10.7 (3.9–21.9)	10.7 (4.0–21.5)	10.8 (4.2–22.9)	10.8 (4.3–22.9)
Southeast Asia	75 to 79	9.8 (3.7–19.9)	9.6 (3.5–20.0)	9.7 (3.6–19.8)	9.8 (3.8–21.1)	9.8 (3.9–21.1)
Southeast Asia	80 to 84	7.0 (2.6–14.6)	6.8 (2.4–14.5)	6.8 (2.5–14.3)	7.0 (2.6–15.5)	7.0 (2.7–15.5)
Southeast Asia	85 to 89	6.2 (2.3–13.2)	6.2 (2.2–13.3)	6.1 (2.2–12.9)	6.4 (2.4–14.2)	6.4 (2.4–14.2)
Southeast Asia	90 to 94	5.5 (2.0–11.8)	5.7 (2.0–12.2)	5.6 (2.0–11.9)	5.8 (2.2–13.1)	5.9 (2.2–13.2)
Southeast Asia	95 plus	5.5 (2.0–11.8)	5.6 (1.9–12.1)	5.8 (2.1–12.2)	6.0 (2.2–13.4)	6.0 (2.3–13.4)
Southeast Asia	Age-standardized	14.1 (5.9–26.6)	14.0 (5.9–26.8)	14.0 (6.6–25.2)	14.1 (6.6–26.5)	14.1 (6.3–26.8)
Southeast Asia	All age	14.2 (6.1–27.1)	14.3 (6.0–27.1)	14.3 (6.8–25.3)	14.4 (6.7–26.9)	14.4 (6.5–27.2)
Cambodia	20 to 24	12.9 (6.1–22.8)	12.9 (6.6–21.2)	12.9 (7.8–19.5)	13.5 (7.2–22.4)	13.8 (6.7–24.0)
Cambodia	25 to 29	13.3 (6.0–24.2)	13.2 (6.5–22.8)	13.2 (7.8–20.5)	13.7 (7.6–21.9)	13.9 (7.0–23.5)
Cambodia	30 to 34	22.6 (12.0–35.8)	22.6 (13.4–34.1)	22.9 (16.3–30.6)	23.1 (13.7–34.5)	23.0 (12.5–36.5)

Supplementary Table S11: Prevalence of male SVAC by age and location for 1990, 2000, 2010, 2020, and 2023						
**Location**	**Age Range**	**1990**	**2000**	**2010**	**2020**	**2023**
Cambodia	35 to 39	20.6 (11.1–33.2)	20.6 (12.2–31.4)	20.8 (14.4–28.3)	21.3 (12.3–32.7)	21.4 (11.2–34.8)
Cambodia	40 to 44	28.4 (15.7–43.5)	28.5 (17.6–41.7)	29.0 (21.2–37.8)	28.6 (17.6–41.7)	28.3 (15.9–43.3)
Cambodia	45 to 49	25.6 (14.4–39.9)	25.7 (15.7–37.6)	26.1 (18.8–34.1)	26.1 (15.7–38.7)	25.9 (14.2–40.9)
Cambodia	50 to 54	19.5 (6.7–39.2)	19.4 (7.3–38.3)	19.3 (7.8–36.5)	19.5 (8.0–38.8)	19.5 (7.7–39.3)
Cambodia	55 to 59	18.9 (6.5–38.3)	18.8 (7.1–37.4)	18.8 (7.5–35.6)	18.9 (7.7–37.9)	19.0 (7.5–38.4)
Cambodia	60 to 64	18.3 (6.3–37.4)	18.3 (6.8–36.5)	18.2 (7.3–34.7)	18.3 (7.5–37.0)	18.4 (7.2–37.5)
Cambodia	65 to 69	17.2 (5.8–35.4)	17.1 (6.3–34.5)	17.0 (6.7–32.8)	17.1 (6.9–35.0)	17.2 (6.7–35.5)
Cambodia	70 to 74	13.9 (4.5–29.7)	13.8 (4.9–28.9)	13.7 (5.3–27.3)	13.8 (5.4–29.3)	13.9 (5.2–29.8)
Cambodia	75 to 79	12.6 (4.0–27.2)	12.5 (4.4–26.5)	12.4 (4.7–25.0)	12.5 (4.8–26.9)	12.6 (4.6–27.3)
Cambodia	80 to 84	9.0 (2.8–20.3)	8.9 (3.0–19.6)	8.8 (3.2–18.4)	8.9 (3.3–20.0)	9.0 (3.2–20.4)
Cambodia	85 to 89	8.1 (2.5–18.5)	8.1 (2.7–17.9)	8.0 (2.9–16.8)	8.0 (3.0–18.2)	8.1 (2.9–18.6)
Cambodia	90 to 94	7.4 (2.2–16.9)	7.3 (2.4–16.3)	7.2 (2.6–15.3)	7.3 (2.7–16.6)	7.3 (2.6–17.0)
Cambodia	95 plus	7.3 (2.2–16.7)	7.2 (2.4–16.2)	7.2 (2.6–15.2)	7.2 (2.6–16.5)	7.3 (2.5–16.8)
Cambodia	Age-standardized	18.9 (9.5–32.4)	18.8 (10.3–30.3)	19.0 (12.0–28.0)	19.2 (10.9–30.7)	19.2 (10.2–32.0)
Cambodia	All age	18.4 (9.4–30.8)	18.7 (10.5–29.3)	18.8 (12.3–26.7)	19.5 (11.4–30.8)	19.8 (10.5–32.8)

Supplementary Table S11: Prevalence of male SVAC by age and location for 1990, 2000, 2010, 2020, and 2023						
**Location**	**Age Range**	**1990**	**2000**	**2010**	**2020**	**2023**
Indonesia	20 to 24	9.6 (3.7–19.3)	9.1 (4.2–16.4)	8.8 (5.2–13.6)	9.2 (4.8–15.7)	9.4 (4.3–16.8)
Indonesia	25 to 29	9.9 (3.8–19.8)	9.4 (4.3–16.9)	9.0 (5.4–13.8)	9.5 (5.0–16.2)	9.7 (4.4–17.3)
Indonesia	30 to 34	11.9 (4.8–23.2)	11.9 (5.7–20.8)	11.9 (7.4–17.6)	12.0 (6.3–20.2)	12.1 (5.5–21.5)
Indonesia	35 to 39	11.6 (4.6–22.9)	11.3 (5.3–20.0)	11.0 (6.5–16.8)	11.3 (5.8–19.3)	11.5 (5.1–20.5)
Indonesia	40 to 44	13.9 (5.8–26.5)	14.9 (7.4–25.3)	15.6 (10.5–21.8)	15.2 (8.3–24.8)	15.0 (7.1–26.1)
Indonesia	45 to 49	13.1 (5.4–25.2)	13.5 (6.6–23.3)	13.8 (8.9–19.9)	13.7 (7.3–22.6)	13.7 (6.4–24.1)
Indonesia	50 to 54	12.0 (4.6–24.3)	12.0 (4.4–24.2)	12.0 (4.5–23.8)	12.1 (4.8–25.3)	12.1 (4.8–25.4)
Indonesia	55 to 59	11.6 (4.4–23.6)	11.6 (4.2–23.6)	11.6 (4.4–23.1)	11.7 (4.6–24.6)	11.7 (4.6–24.6)
Indonesia	60 to 64	11.2 (4.3–22.9)	11.2 (4.1–22.9)	11.2 (4.2–22.5)	11.3 (4.4–23.9)	11.3 (4.5–23.7)
Indonesia	65 to 69	10.4 (3.9–21.5)	10.4 (3.8–21.4)	10.4 (3.9–21.0)	10.5 (4.1–22.4)	10.5 (4.1–22.2)
Indonesia	70 to 74	8.3 (3.0–17.4)	8.2 (2.9–17.3)	8.2 (3.0–17.0)	8.3 (3.2–18.2)	8.3 (3.2–18.0)
Indonesia	75 to 79	7.4 (2.7–15.7)	7.4 (2.6–15.7)	7.4 (2.7–15.4)	7.5 (2.8–16.4)	7.5 (2.8–16.3)
Indonesia	80 to 84	5.2 (1.9–11.2)	5.2 (1.8–11.2)	5.2 (1.8–11.0)	5.2 (1.9–11.8)	5.2 (2.0–11.7)
Indonesia	85 to 89	4.7 (1.7–10.1)	4.6 (1.6–10.1)	4.6 (1.6– 9.9)	4.7 (1.7–10.6)	4.7 (1.7–10.6)
Indonesia	90 to 94	4.2 (1.5– 9.2)	4.2 (1.4– 9.2)	4.2 (1.5– 9.0)	4.2 (1.6– 9.6)	4.2 (1.6– 9.6)

Supplementary Table S11: Prevalence of male SVAC by age and location for 1990, 2000, 2010, 2020, and 2023						
**Location**	**Age Range**	**1990**	**2000**	**2010**	**2020**	**2023**
Indonesia	95 plus	4.2 (1.5– 9.1)	4.1 (1.4– 9.1)	4.2 (1.5– 8.9)	4.2 (1.5– 9.6)	4.2 (1.6– 9.5)
Indonesia	Age-standardized	11.0 (4.5–21.9)	11.0 (4.9–20.4)	11.0 (6.3–17.6)	11.1 (5.7–19.5)	11.2 (5.1–20.4)
Indonesia	All age	11.2 (4.5–21.9)	11.2 (5.1–20.4)	11.3 (6.6–17.5)	11.5 (5.9–19.8)	11.5 (5.2–20.8)
Laos	20 to 24	22.1 (9.7–39.6)	23.3 (11.9–38.8)	24.5 (16.1–34.4)	24.6 (15.3–36.4)	24.4 (13.6–38.2)
Laos	25 to 29	22.0 (9.6–40.4)	22.8 (10.3–39.6)	23.7 (13.0–37.6)	24.5 (15.6–35.4)	24.4 (14.9–36.2)
Laos	30 to 34	22.7 (9.6–41.6)	22.7 (9.3–41.6)	22.8 (9.6–41.1)	23.1 (10.1–43.3)	23.1 (10.2–43.1)
Laos	35 to 39	22.9 (9.7–41.9)	22.9 (9.4–41.8)	22.9 (9.7–41.3)	23.3 (10.2–43.6)	23.3 (10.3–43.4)
Laos	40 to 44	23.6 (10.1–42.9)	23.6 (9.7–42.8)	23.6 (10.0–42.3)	24.0 (10.6–44.6)	24.0 (10.7–44.3)
Laos	45 to 49	23.0 (9.8–42.1)	23.0 (9.4–42.0)	23.1 (9.7–41.5)	23.5 (10.3–43.8)	23.5 (10.4–43.6)
Laos	50 to 54	22.6 (9.6–41.4)	22.5 (9.2–41.3)	22.6 (9.5–40.9)	23.0 (10.1–43.1)	23.0 (10.1–42.9)
Laos	55 to 59	21.9 (9.2–40.5)	21.9 (8.9–40.4)	22.0 (9.2–39.9)	22.3 (9.7–42.2)	22.3 (9.8–41.9)
Laos	60 to 64	21.3 (8.9–39.6)	21.3 (8.6–39.5)	21.4 (8.9–39.0)	21.7 (9.4–41.2)	21.7 (9.5–41.0)
Laos	65 to 69	20.0 (8.2–37.5)	19.9 (7.9–37.5)	20.0 (8.2–37.0)	20.3 (8.7–39.2)	20.4 (8.7–39.0)
Laos	70 to 74	16.2 (6.5–31.6)	16.2 (6.2–31.6)	16.3 (6.4–31.1)	16.6 (6.8–33.2)	16.6 (6.9–32.9)
Laos	75 to 79	14.7 (5.8–29.1)	14.7 (5.6–29.0)	14.7 (5.7–28.6)	15.0 (6.1–30.6)	15.0 (6.1–30.4)

Supplementary Table S11: Prevalence of male SVAC by age and location for 1990, 2000, 2010, 2020, and 2023						
**Location**	**Age Range**	**1990**	**2000**	**2010**	**2020**	**2023**
Laos	80 to 84	10.6 (4.0–21.8)	10.6 (3.8–21.7)	10.6 (4.0–21.4)	10.8 (4.2–23.0)	10.8 (4.3–23.0)
Laos	85 to 89	9.6 (3.6–19.9)	9.6 (3.4–19.8)	9.6 (3.6–19.5)	9.8 (3.8–21.0)	9.8 (3.8–20.9)
Laos	90 to 94	8.7 (3.2–18.2)	8.7 (3.1–18.2)	8.7 (3.2–17.9)	8.9 (3.4–19.3)	8.9 (3.4–19.1)
Laos	95 plus	8.6 (3.2–18.1)	8.6 (3.1–18.0)	8.6 (3.2–17.7)	8.8 (3.4–19.1)	8.8 (3.4–19.1)
Laos	Age-standardized	21.6 (9.3–38.9)	21.8 (9.4–39.5)	22.1 (10.4–38.2)	22.5 (11.6–38.8)	22.4 (11.1–39.3)
Laos	All age	22.1 (9.6–39.6)	22.4 (10.0–40.1)	22.9 (11.4–38.7)	23.2 (12.4–39.2)	23.1 (11.5–40.0)
Malaysia	20 to 24	13.1 (5.1–26.3)	13.1 (4.9–26.3)	13.1 (5.0–25.8)	13.2 (5.3–27.4)	13.2 (5.3–27.3)
Malaysia	25 to 29	13.5 (5.2–27.0)	13.5 (5.0–27.0)	13.5 (5.2–26.5)	13.6 (5.5–28.2)	13.7 (5.5–28.1)
Malaysia	30 to 34	14.8 (5.8–29.2)	14.8 (5.6–29.1)	14.8 (5.8–28.7)	14.9 (6.1–30.4)	14.9 (6.1–30.2)
Malaysia	35 to 39	15.1 (5.9–29.7)	15.0 (5.7–29.6)	15.1 (5.9–29.1)	15.2 (6.2–30.9)	15.2 (6.2–30.9)
Malaysia	40 to 44	15.7 (6.2–30.8)	15.7 (6.0–30.7)	15.7 (6.2–30.2)	15.9 (6.5–32.0)	15.9 (6.5–31.9)
Malaysia	45 to 49	15.4 (6.1–30.2)	15.4 (5.8–30.2)	15.4 (6.0–29.7)	15.6 (6.4–31.5)	15.6 (6.4–31.5)
Malaysia	50 to 54	15.1 (5.9–29.7)	15.1 (5.7–29.7)	15.1 (5.9–29.2)	15.2 (6.2–30.9)	15.3 (6.3–31.0)
Malaysia	55 to 59	14.6 (5.7–29.0)	14.6 (5.5–28.9)	14.6 (5.7–28.5)	14.8 (6.0–30.2)	14.8 (6.0–30.0)
Malaysia	60 to 64	14.2 (5.6–28.2)	14.2 (5.3–28.2)	14.2 (5.5–27.7)	14.4 (5.8–29.4)	14.4 (5.8–29.2)

Supplementary Table S11: Prevalence of male SVAC by age and location for 1990, 2000, 2010, 2020, and 2023						
**Location**	**Age Range**	**1990**	**2000**	**2010**	**2020**	**2023**
Malaysia	65 to 69	13.2 (5.1–26.5)	13.2 (4.9–26.5)	13.2 (5.1–26.0)	13.4 (5.4–27.7)	13.4 (5.4–27.5)
Malaysia	70 to 74	10.6 (4.0–21.7)	10.6 (3.8–21.7)	10.6 (4.0–21.3)	10.7 (4.2–22.7)	10.7 (4.2–22.6)
Malaysia	75 to 79	9.5 (3.6–19.8)	9.5 (3.4–19.7)	9.5 (3.5–19.4)	9.6 (3.7–20.7)	9.6 (3.7–20.6)
Malaysia	80 to 84	6.7 (2.4–14.3)	6.7 (2.3–14.3)	6.7 (2.4–14.0)	6.8 (2.6–15.1)	6.8 (2.6–15.0)
Malaysia	85 to 89	6.0 (2.2–13.0)	6.0 (2.1–13.0)	6.0 (2.2–12.7)	6.1 (2.3–13.7)	6.1 (2.3–13.6)
Malaysia	90 to 94	5.5 (2.0–11.8)	5.4 (1.9–11.8)	5.4 (1.9–11.5)	5.5 (2.1–12.4)	5.5 (2.1–12.3)
Malaysia	95 plus	5.4 (1.9–11.7)	5.4 (1.9–11.7)	5.4 (1.9–11.4)	5.5 (2.0–12.3)	5.5 (2.0–12.3)
Malaysia	Age-standardized	13.9 (5.4–27.7)	13.9 (5.2–27.6)	14.0 (5.4–27.2)	14.1 (5.7–28.9)	14.1 (5.7–28.8)
Malaysia	All age	14.2 (5.5–28.1)	14.3 (5.4–28.2)	14.2 (5.5–27.6)	14.3 (5.8–29.2)	14.3 (5.8–29.1)
Maldives	20 to 24	14.8 (5.8–29.3)	14.8 (5.6–29.2)	14.7 (5.7–28.5)	14.8 (6.0–30.3)	14.9 (6.1–30.1)
Maldives	25 to 29	15.2 (6.0–30.0)	15.2 (5.8–29.9)	15.1 (5.9–29.3)	15.3 (6.2–31.0)	15.3 (6.3–31.1)
Maldives	30 to 34	16.7 (6.7–32.4)	16.7 (6.4–32.3)	16.6 (6.6–31.7)	16.8 (6.9–33.5)	16.8 (7.0–33.3)
Maldives	35 to 39	17.1 (6.9–33.0)	17.1 (6.6–32.9)	17.0 (6.8–32.3)	17.2 (7.1–34.1)	17.2 (7.2–34.0)
Maldives	40 to 44	17.9 (7.2–34.3)	17.9 (7.0–34.2)	17.8 (7.1–33.6)	18.0 (7.5–35.5)	18.0 (7.6–35.3)
Maldives	45 to 49	17.6 (7.1–33.8)	17.6 (6.8–33.8)	17.5 (7.0–33.1)	17.7 (7.4–35.0)	17.7 (7.4–34.8)

Supplementary Table S11: Prevalence of male SVAC by age and location for 1990, 2000, 2010, 2020, and 2023						
**Location**	**Age Range**	**1990**	**2000**	**2010**	**2020**	**2023**
Maldives	50 to 54	17.2 (6.9–33.2)	17.2 (6.7–33.2)	17.2 (6.8–32.6)	17.3 (7.2–34.4)	17.3 (7.3–34.5)
Maldives	55 to 59	16.7 (6.7–32.5)	16.7 (6.4–32.4)	16.7 (6.6–31.8)	16.8 (7.0–33.6)	16.8 (7.0–33.4)
Maldives	60 to 64	16.3 (6.5–31.7)	16.3 (6.2–31.7)	16.2 (6.4–31.1)	16.4 (6.7–32.8)	16.4 (6.8–32.6)
Maldives	65 to 69	15.2 (6.0–29.9)	15.2 (5.8–29.9)	15.1 (5.9–29.2)	15.3 (6.2–31.0)	15.3 (6.3–30.8)
Maldives	70 to 74	12.2 (4.7–24.7)	12.2 (4.5–24.7)	12.1 (4.6–24.1)	12.3 (4.9–25.7)	12.3 (4.9–25.7)
Maldives	75 to 79	11.0 (4.2–22.6)	11.0 (4.0–22.5)	10.9 (4.1–22.0)	11.1 (4.3–23.5)	11.1 (4.4–23.3)
Maldives	80 to 84	7.8 (2.9–16.5)	7.8 (2.8–16.5)	7.8 (2.8–16.1)	7.8 (3.0–17.2)	7.9 (3.0–17.1)
Maldives	85 to 89	7.0 (2.6–15.0)	7.0 (2.5–15.0)	7.0 (2.5–14.6)	7.1 (2.7–15.6)	7.1 (2.7–15.5)
Maldives	90 to 94	6.4 (2.3–13.6)	6.4 (2.2–13.6)	6.3 (2.3–13.3)	6.4 (2.4–14.3)	6.4 (2.4–14.1)
Maldives	95 plus	6.3 (2.3–13.5)	6.3 (2.2–13.5)	6.3 (2.3–13.2)	6.3 (2.4–14.1)	6.3 (2.4–14.0)
Maldives	Age-standardized	15.9 (6.3–30.9)	15.9 (6.1–30.9)	15.8 (6.2–30.3)	15.9 (6.6–32.0)	15.9 (6.6–31.9)
Maldives	All age	16.0 (6.4–31.2)	16.0 (6.1–31.1)	15.9 (6.3–30.5)	16.3 (6.7–32.7)	16.4 (6.8–32.6)
Mauritius	20 to 24	14.8 (5.8–29.3)	14.8 (5.6–29.2)	14.7 (5.7–28.5)	14.8 (6.0–30.3)	14.9 (6.1–30.1)
Mauritius	25 to 29	15.2 (6.0–30.0)	15.2 (5.8–29.9)	15.1 (5.9–29.3)	15.3 (6.2–31.0)	15.3 (6.3–31.1)
Mauritius	30 to 34	16.7 (6.7–32.4)	16.7 (6.4–32.3)	16.6 (6.6–31.7)	16.8 (6.9–33.5)	16.8 (7.0–33.3)

Supplementary Table S11: Prevalence of male SVAC by age and location for 1990, 2000, 2010, 2020, and 2023						
**Location**	**Age Range**	**1990**	**2000**	**2010**	**2020**	**2023**
Mauritius	35 to 39	17.1 (6.9–33.0)	17.1 (6.6–32.9)	17.0 (6.8–32.3)	17.2 (7.1–34.1)	17.2 (7.2–34.0)
Mauritius	40 to 44	17.9 (7.2–34.3)	17.9 (7.0–34.2)	17.8 (7.1–33.6)	18.0 (7.5–35.5)	18.0 (7.6–35.3)
Mauritius	45 to 49	17.6 (7.1–33.8)	17.6 (6.8–33.8)	17.5 (7.0–33.1)	17.7 (7.4–35.0)	17.7 (7.4–34.8)
Mauritius	50 to 54	17.2 (6.9–33.2)	17.2 (6.7–33.2)	17.2 (6.8–32.6)	17.3 (7.2–34.4)	17.3 (7.3–34.5)
Mauritius	55 to 59	16.7 (6.7–32.5)	16.7 (6.4–32.4)	16.7 (6.6–31.8)	16.8 (7.0–33.6)	16.8 (7.0–33.4)
Mauritius	60 to 64	16.3 (6.5–31.7)	16.3 (6.2–31.7)	16.2 (6.4–31.1)	16.4 (6.7–32.8)	16.4 (6.8–32.6)
Mauritius	65 to 69	15.2 (6.0–29.9)	15.2 (5.8–29.9)	15.1 (5.9–29.2)	15.3 (6.2–31.0)	15.3 (6.3–30.8)
Mauritius	70 to 74	12.2 (4.7–24.7)	12.2 (4.5–24.7)	12.1 (4.6–24.1)	12.3 (4.9–25.7)	12.3 (4.9–25.7)
Mauritius	75 to 79	11.0 (4.2–22.6)	11.0 (4.0–22.5)	10.9 (4.1–22.0)	11.1 (4.3–23.5)	11.1 (4.4–23.3)
Mauritius	80 to 84	7.8 (2.9–16.5)	7.8 (2.8–16.5)	7.8 (2.8–16.1)	7.8 (3.0–17.2)	7.9 (3.0–17.1)
Mauritius	85 to 89	7.0 (2.6–15.0)	7.0 (2.5–15.0)	7.0 (2.5–14.6)	7.1 (2.7–15.6)	7.1 (2.7–15.5)
Mauritius	90 to 94	6.4 (2.3–13.6)	6.4 (2.2–13.6)	6.3 (2.3–13.3)	6.4 (2.4–14.3)	6.4 (2.4–14.1)
Mauritius	95 plus	6.3 (2.3–13.5)	6.3 (2.2–13.5)	6.3 (2.3–13.2)	6.3 (2.4–14.1)	6.3 (2.4–14.0)
Mauritius	Age-standardized	15.9 (6.3–30.9)	15.9 (6.1–30.9)	15.8 (6.2–30.3)	15.9 (6.6–32.0)	15.9 (6.6–31.9)
Mauritius	All age	16.1 (6.4–31.4)	16.2 (6.2–31.5)	16.1 (6.4–30.9)	16.1 (6.6–32.3)	16.0 (6.6–32.1)

Supplementary Table S11: Prevalence of male SVAC by age and location for 1990, 2000, 2010, 2020, and 2023						
**Location**	**Age Range**	**1990**	**2000**	**2010**	**2020**	**2023**
Myanmar	20 to 24	14.8 (5.8–29.3)	14.8 (5.6–29.2)	14.7 (5.7–28.5)	14.8 (6.0–30.3)	14.9 (6.1–30.1)
Myanmar	25 to 29	15.2 (6.0–30.0)	15.2 (5.8–29.9)	15.1 (5.9–29.3)	15.3 (6.2–31.0)	15.3 (6.3–31.1)
Myanmar	30 to 34	16.7 (6.7–32.4)	16.7 (6.4–32.3)	16.6 (6.6–31.7)	16.8 (6.9–33.5)	16.8 (7.0–33.3)
Myanmar	35 to 39	17.1 (6.9–33.0)	17.1 (6.6–32.9)	17.0 (6.8–32.3)	17.2 (7.1–34.1)	17.2 (7.2–34.0)
Myanmar	40 to 44	17.9 (7.2–34.3)	17.9 (7.0–34.2)	17.8 (7.1–33.6)	18.0 (7.5–35.5)	18.0 (7.6–35.3)
Myanmar	45 to 49	17.6 (7.1–33.8)	17.6 (6.8–33.8)	17.5 (7.0–33.1)	17.7 (7.4–35.0)	17.7 (7.4–34.8)
Myanmar	50 to 54	17.2 (6.9–33.2)	17.2 (6.7–33.2)	17.2 (6.8–32.6)	17.3 (7.2–34.4)	17.3 (7.3–34.5)
Myanmar	55 to 59	16.7 (6.7–32.5)	16.7 (6.4–32.4)	16.7 (6.6–31.8)	16.8 (7.0–33.6)	16.8 (7.0–33.4)
Myanmar	60 to 64	16.3 (6.5–31.7)	16.3 (6.2–31.7)	16.2 (6.4–31.1)	16.4 (6.7–32.8)	16.4 (6.8–32.6)
Myanmar	65 to 69	15.2 (6.0–29.9)	15.2 (5.8–29.9)	15.1 (5.9–29.2)	15.3 (6.2–31.0)	15.3 (6.3–30.8)
Myanmar	70 to 74	12.2 (4.7–24.7)	12.2 (4.5–24.7)	12.1 (4.6–24.1)	12.3 (4.9–25.7)	12.3 (4.9–25.7)
Myanmar	75 to 79	11.0 (4.2–22.6)	11.0 (4.0–22.5)	10.9 (4.1–22.0)	11.1 (4.3–23.5)	11.1 (4.4–23.3)
Myanmar	80 to 84	7.8 (2.9–16.5)	7.8 (2.8–16.5)	7.8 (2.8–16.1)	7.8 (3.0–17.2)	7.9 (3.0–17.1)
Myanmar	85 to 89	7.0 (2.6–15.0)	7.0 (2.5–15.0)	7.0 (2.5–14.6)	7.1 (2.7–15.6)	7.1 (2.7–15.5)
Myanmar	90 to 94	6.4 (2.3–13.6)	6.4 (2.2–13.6)	6.3 (2.3–13.3)	6.4 (2.4–14.3)	6.4 (2.4–14.1)

Supplementary Table S11: Prevalence of male SVAC by age and location for 1990, 2000, 2010, 2020, and 2023						
**Location**	**Age Range**	**1990**	**2000**	**2010**	**2020**	**2023**
Myanmar	95 plus	6.3 (2.3–13.5)	6.3 (2.2–13.5)	6.3 (2.3–13.2)	6.3 (2.4–14.1)	6.3 (2.4–14.0)
Myanmar	Age-standardized	15.9 (6.3–30.9)	15.9 (6.1–30.9)	15.8 (6.2–30.3)	15.9 (6.6–32.0)	15.9 (6.6–31.9)
Myanmar	All age	16.1 (6.4–31.3)	16.1 (6.2–31.4)	16.1 (6.4–30.8)	16.2 (6.7–32.5)	16.2 (6.7–32.4)
Philippines	20 to 24	14.8 (5.8–29.3)	14.8 (5.6–29.2)	14.7 (5.7–28.5)	14.8 (6.0–30.3)	14.9 (6.1–30.1)
Philippines	25 to 29	15.2 (6.0–30.0)	15.2 (5.8–29.9)	15.1 (5.9–29.3)	15.3 (6.2–31.0)	15.3 (6.3–31.1)
Philippines	30 to 34	16.7 (6.7–32.4)	16.7 (6.4–32.3)	16.6 (6.6–31.7)	16.8 (6.9–33.5)	16.8 (7.0–33.3)
Philippines	35 to 39	17.1 (6.9–33.0)	17.1 (6.6–32.9)	17.0 (6.8–32.3)	17.2 (7.1–34.1)	17.2 (7.2–34.0)
Philippines	40 to 44	17.9 (7.2–34.3)	17.9 (7.0–34.2)	17.8 (7.1–33.6)	18.0 (7.5–35.5)	18.0 (7.6–35.3)
Philippines	45 to 49	17.6 (7.1–33.8)	17.6 (6.8–33.8)	17.5 (7.0–33.1)	17.7 (7.4–35.0)	17.7 (7.4–34.8)
Philippines	50 to 54	17.2 (6.9–33.2)	17.2 (6.7–33.2)	17.2 (6.8–32.6)	17.3 (7.2–34.4)	17.3 (7.3–34.5)
Philippines	55 to 59	16.7 (6.7–32.5)	16.7 (6.4–32.4)	16.7 (6.6–31.8)	16.8 (7.0–33.6)	16.8 (7.0–33.4)
Philippines	60 to 64	16.3 (6.5–31.7)	16.3 (6.2–31.7)	16.2 (6.4–31.1)	16.4 (6.7–32.8)	16.4 (6.8–32.6)
Philippines	65 to 69	15.2 (6.0–29.9)	15.2 (5.8–29.9)	15.1 (5.9–29.2)	15.3 (6.2–31.0)	15.3 (6.3–30.8)
Philippines	70 to 74	12.2 (4.7–24.7)	12.2 (4.5–24.7)	12.1 (4.6–24.1)	12.3 (4.9–25.7)	12.3 (4.9–25.7)
Philippines	75 to 79	11.0 (4.2–22.6)	11.0 (4.0–22.5)	10.9 (4.1–22.0)	11.1 (4.3–23.5)	11.1 (4.4–23.3)

Supplementary Table S11: Prevalence of male SVAC by age and location for 1990, 2000, 2010, 2020, and 2023						
**Location**	**Age Range**	**1990**	**2000**	**2010**	**2020**	**2023**
Philippines	80 to 84	7.8 (2.9–16.5)	7.8 (2.8–16.5)	7.8 (2.8–16.1)	7.8 (3.0–17.2)	7.9 (3.0–17.1)
Philippines	85 to 89	7.0 (2.6–15.0)	7.0 (2.5–15.0)	7.0 (2.5–14.6)	7.1 (2.7–15.6)	7.1 (2.7–15.5)
Philippines	90 to 94	6.4 (2.3–13.6)	6.4 (2.2–13.6)	6.3 (2.3–13.3)	6.4 (2.4–14.3)	6.4 (2.4–14.1)
Philippines	95 plus	6.3 (2.3–13.5)	6.3 (2.2–13.5)	6.3 (2.3–13.2)	6.3 (2.4–14.1)	6.3 (2.4–14.0)
Philippines	Age-standardized	15.9 (6.3–30.9)	15.9 (6.1–30.9)	15.8 (6.2–30.3)	15.9 (6.6–32.0)	15.9 (6.6–31.9)
Philippines	All age	16.1 (6.4–31.3)	16.1 (6.2–31.4)	16.1 (6.4–30.8)	16.2 (6.7–32.6)	16.2 (6.7–32.4)
Seychelles	20 to 24	14.8 (5.8–29.3)	14.8 (5.6–29.2)	14.7 (5.7–28.5)	14.8 (6.0–30.3)	14.9 (6.1–30.1)
Seychelles	25 to 29	15.2 (6.0–30.0)	15.2 (5.8–29.9)	15.1 (5.9–29.3)	15.3 (6.2–31.0)	15.3 (6.3–31.1)
Seychelles	30 to 34	16.7 (6.7–32.4)	16.7 (6.4–32.3)	16.6 (6.6–31.7)	16.8 (6.9–33.5)	16.8 (7.0–33.3)
Seychelles	35 to 39	17.1 (6.9–33.0)	17.1 (6.6–32.9)	17.0 (6.8–32.3)	17.2 (7.1–34.1)	17.2 (7.2–34.0)
Seychelles	40 to 44	17.9 (7.2–34.3)	17.9 (7.0–34.2)	17.8 (7.1–33.6)	18.0 (7.5–35.5)	18.0 (7.6–35.3)
Seychelles	45 to 49	17.6 (7.1–33.8)	17.6 (6.8–33.8)	17.5 (7.0–33.1)	17.7 (7.4–35.0)	17.7 (7.4–34.8)
Seychelles	50 to 54	17.2 (6.9–33.2)	17.2 (6.7–33.2)	17.2 (6.8–32.6)	17.3 (7.2–34.4)	17.3 (7.3–34.5)
Seychelles	55 to 59	16.7 (6.7–32.5)	16.7 (6.4–32.4)	16.7 (6.6–31.8)	16.8 (7.0–33.6)	16.8 (7.0–33.4)
Seychelles	60 to 64	16.3 (6.5–31.7)	16.3 (6.2–31.7)	16.2 (6.4–31.1)	16.4 (6.7–32.8)	16.4 (6.8–32.6)

Supplementary Table S11: Prevalence of male SVAC by age and location for 1990, 2000, 2010, 2020, and 2023						
**Location**	**Age Range**	**1990**	**2000**	**2010**	**2020**	**2023**
Seychelles	65 to 69	15.2 (6.0–29.9)	15.2 (5.8–29.9)	15.1 (5.9–29.2)	15.3 (6.2–31.0)	15.3 (6.3–30.8)
Seychelles	70 to 74	12.2 (4.7–24.7)	12.2 (4.5–24.7)	12.1 (4.6–24.1)	12.3 (4.9–25.7)	12.3 (4.9–25.7)
Seychelles	75 to 79	11.0 (4.2–22.6)	11.0 (4.0–22.5)	10.9 (4.1–22.0)	11.1 (4.3–23.5)	11.1 (4.4–23.3)
Seychelles	80 to 84	7.8 (2.9–16.5)	7.8 (2.8–16.5)	7.8 (2.8–16.1)	7.8 (3.0–17.2)	7.9 (3.0–17.1)
Seychelles	85 to 89	7.0 (2.6–15.0)	7.0 (2.5–15.0)	7.0 (2.5–14.6)	7.1 (2.7–15.6)	7.1 (2.7–15.5)
Seychelles	90 to 94	6.4 (2.3–13.6)	6.4 (2.2–13.6)	6.3 (2.3–13.3)	6.4 (2.4–14.3)	6.4 (2.4–14.1)
Seychelles	95 plus	6.3 (2.3–13.5)	6.3 (2.2–13.5)	6.3 (2.3–13.2)	6.3 (2.4–14.1)	6.3 (2.4–14.0)
Seychelles	Age-standardized	15.9 (6.3–30.9)	15.9 (6.1–30.9)	15.8 (6.2–30.3)	15.9 (6.6–32.0)	15.9 (6.6–31.9)
Seychelles	All age	15.9 (6.3–31.0)	16.1 (6.2–31.3)	16.1 (6.4–30.8)	16.3 (6.7–32.7)	16.3 (6.8–32.6)
Sri Lanka	20 to 24	20.0 (9.0–35.8)	18.8 (9.8–30.8)	16.8 (9.0–27.3)	15.9 (7.3–29.3)	15.8 (6.8–29.7)
Sri Lanka	25 to 29	17.5 (7.3–32.7)	16.7 (8.2–28.3)	15.5 (8.2–25.6)	15.2 (7.1–29.0)	15.2 (6.6–29.4)
Sri Lanka	30 to 34	20.3 (8.8–36.9)	20.6 (11.1–32.8)	21.7 (13.2–31.7)	22.7 (12.5–35.5)	22.9 (12.0–37.1)
Sri Lanka	35 to 39	18.8 (8.0–34.5)	18.9 (10.0–30.6)	19.6 (11.6–29.5)	20.6 (10.7–33.6)	20.8 (10.5–34.6)
Sri Lanka	40 to 44	21.4 (9.4–38.7)	22.5 (12.3–35.4)	26.0 (16.6–36.7)	28.5 (16.5–42.3)	28.9 (15.8–44.1)
Sri Lanka	45 to 49	21.7 (9.6–38.8)	22.4 (12.1–35.3)	24.3 (15.2–34.8)	25.7 (14.3–39.5)	26.0 (13.8–40.7)

Supplementary Table S11: Prevalence of male SVAC by age and location for 1990, 2000, 2010, 2020, and 2023						
**Location**	**Age Range**	**1990**	**2000**	**2010**	**2020**	**2023**
Sri Lanka	50 to 54	16.8 (6.4–33.1)	16.4 (7.2–30.4)	16.4 (7.2–29.9)	17.1 (6.5–34.7)	17.2 (6.5–35.6)
Sri Lanka	55 to 59	16.5 (6.2–32.8)	16.2 (7.0–29.9)	16.1 (7.1–29.6)	16.7 (6.5–34.2)	16.8 (6.4–35.2)
Sri Lanka	60 to 64	21.6 (9.1–39.4)	21.0 (10.0–35.7)	20.0 (9.4–34.3)	19.7 (7.9–39.1)	19.7 (7.7–39.7)
Sri Lanka	65 to 69	16.2 (5.5–34.9)	15.8 (5.9–31.5)	15.5 (6.1–29.9)	15.9 (5.5–33.9)	16.0 (5.5–34.0)
Sri Lanka	70 to 74	13.1 (4.1–29.3)	12.7 (4.6–26.2)	12.5 (4.8–24.8)	12.8 (4.3–28.5)	12.9 (4.3–28.9)
Sri Lanka	75 to 79	11.8 (3.8–26.7)	11.5 (4.1–23.9)	11.3 (4.2–22.6)	11.5 (3.9–25.3)	11.6 (3.8–26.1)
Sri Lanka	80 to 84	8.4 (2.5–19.8)	8.1 (2.8–17.6)	8.0 (2.9–16.5)	8.2 (2.7–19.1)	8.3 (2.7–19.2)
Sri Lanka	85 to 89	7.6 (2.3–18.0)	7.3 (2.5–16.0)	7.2 (2.6–15.0)	7.4 (2.4–17.0)	7.5 (2.4–17.4)
Sri Lanka	90 to 94	6.9 (2.0–16.3)	6.6 (2.3–14.5)	6.5 (2.4–13.7)	6.7 (2.3–15.9)	6.8 (2.2–16.2)
Sri Lanka	95 plus	6.8 (2.1–16.1)	6.6 (2.3–14.4)	6.5 (2.3–13.6)	6.7 (2.2–15.7)	6.7 (2.2–15.9)
Sri Lanka	Age-standardized	18.5 (7.8–34.0)	18.3 (9.0–30.9)	18.5 (10.6–29.0)	19.1 (9.6–32.6)	19.2 (9.3–33.9)
Sri Lanka	All age	18.9 (8.1–34.8)	18.7 (9.5–31.4)	18.9 (10.8–29.5)	19.3 (9.8–33.1)	19.3 (9.3–34.0)
Thailand	20 to 24	14.8 (5.8–29.3)	14.8 (5.6–29.2)	14.7 (5.7–28.5)	14.8 (6.0–30.3)	14.9 (6.1–30.1)
Thailand	25 to 29	15.2 (6.0–30.0)	15.2 (5.8–29.9)	15.1 (5.9–29.3)	15.3 (6.2–31.0)	15.3 (6.3–31.1)
Thailand	30 to 34	16.7 (6.7–32.4)	16.7 (6.4–32.3)	16.6 (6.6–31.7)	16.8 (6.9–33.5)	16.8 (7.0–33.3)

Supplementary Table S11: Prevalence of male SVAC by age and location for 1990, 2000, 2010, 2020, and 2023						
**Location**	**Age Range**	**1990**	**2000**	**2010**	**2020**	**2023**
Thailand	35 to 39	17.1 (6.9–33.0)	17.1 (6.6–32.9)	17.0 (6.8–32.3)	17.2 (7.1–34.1)	17.2 (7.2–34.0)
Thailand	40 to 44	17.9 (7.2–34.3)	17.9 (7.0–34.2)	17.8 (7.1–33.6)	18.0 (7.5–35.5)	18.0 (7.6–35.3)
Thailand	45 to 49	17.6 (7.1–33.8)	17.6 (6.8–33.8)	17.5 (7.0–33.1)	17.7 (7.4–35.0)	17.7 (7.4–34.8)
Thailand	50 to 54	17.2 (6.9–33.2)	17.2 (6.7–33.2)	17.2 (6.8–32.6)	17.3 (7.2–34.4)	17.3 (7.3–34.5)
Thailand	55 to 59	16.7 (6.7–32.5)	16.7 (6.4–32.4)	16.7 (6.6–31.8)	16.8 (7.0–33.6)	16.8 (7.0–33.4)
Thailand	60 to 64	16.3 (6.5–31.7)	16.3 (6.2–31.7)	16.2 (6.4–31.1)	16.4 (6.7–32.8)	16.4 (6.8–32.6)
Thailand	65 to 69	15.2 (6.0–29.9)	15.2 (5.8–29.9)	15.1 (5.9–29.2)	15.3 (6.2–31.0)	15.3 (6.3–30.8)
Thailand	70 to 74	12.2 (4.7–24.7)	12.2 (4.5–24.7)	12.1 (4.6–24.1)	12.3 (4.9–25.7)	12.3 (4.9–25.7)
Thailand	75 to 79	11.0 (4.2–22.6)	11.0 (4.0–22.5)	10.9 (4.1–22.0)	11.1 (4.3–23.5)	11.1 (4.4–23.3)
Thailand	80 to 84	7.8 (2.9–16.5)	7.8 (2.8–16.5)	7.8 (2.8–16.1)	7.8 (3.0–17.2)	7.9 (3.0–17.1)
Thailand	85 to 89	7.0 (2.6–15.0)	7.0 (2.5–15.0)	7.0 (2.5–14.6)	7.1 (2.7–15.6)	7.1 (2.7–15.5)
Thailand	90 to 94	6.4 (2.3–13.6)	6.4 (2.2–13.6)	6.3 (2.3–13.3)	6.4 (2.4–14.3)	6.4 (2.4–14.1)
Thailand	95 plus	6.3 (2.3–13.5)	6.3 (2.2–13.5)	6.3 (2.3–13.2)	6.3 (2.4–14.1)	6.3 (2.4–14.0)
Thailand	Age-standardized	15.9 (6.3–30.9)	15.9 (6.1–30.9)	15.8 (6.2–30.3)	15.9 (6.6–32.0)	15.9 (6.6–31.9)
Thailand	All age	16.1 (6.4–31.3)	16.2 (6.2–31.4)	16.1 (6.4–30.8)	16.0 (6.6–32.2)	15.9 (6.6–31.8)

Supplementary Table S11: Prevalence of male SVAC by age and location for 1990, 2000, 2010, 2020, and 2023						
**Location**	**Age Range**	**1990**	**2000**	**2010**	**2020**	**2023**
Timor-Leste	20 to 24	14.8 (5.8–29.3)	14.8 (5.6–29.2)	14.7 (5.7–28.5)	14.8 (6.0–30.3)	14.9 (6.1–30.1)
Timor-Leste	25 to 29	15.2 (6.0–30.0)	15.2 (5.8–29.9)	15.1 (5.9–29.3)	15.3 (6.2–31.0)	15.3 (6.3–31.1)
Timor-Leste	30 to 34	16.7 (6.7–32.4)	16.7 (6.4–32.3)	16.6 (6.6–31.7)	16.8 (6.9–33.5)	16.8 (7.0–33.3)
Timor-Leste	35 to 39	17.1 (6.9–33.0)	17.1 (6.6–32.9)	17.0 (6.8–32.3)	17.2 (7.1–34.1)	17.2 (7.2–34.0)
Timor-Leste	40 to 44	17.9 (7.2–34.3)	17.9 (7.0–34.2)	17.8 (7.1–33.6)	18.0 (7.5–35.5)	18.0 (7.6–35.3)
Timor-Leste	45 to 49	17.6 (7.1–33.8)	17.6 (6.8–33.8)	17.5 (7.0–33.1)	17.7 (7.4–35.0)	17.7 (7.4–34.8)
Timor-Leste	50 to 54	17.2 (6.9–33.2)	17.2 (6.7–33.2)	17.2 (6.8–32.6)	17.3 (7.2–34.4)	17.3 (7.3–34.5)
Timor-Leste	55 to 59	16.7 (6.7–32.5)	16.7 (6.4–32.4)	16.7 (6.6–31.8)	16.8 (7.0–33.6)	16.8 (7.0–33.4)
Timor-Leste	60 to 64	16.3 (6.5–31.7)	16.3 (6.2–31.7)	16.2 (6.4–31.1)	16.4 (6.7–32.8)	16.4 (6.8–32.6)
Timor-Leste	65 to 69	15.2 (6.0–29.9)	15.2 (5.8–29.9)	15.1 (5.9–29.2)	15.3 (6.2–31.0)	15.3 (6.3–30.8)
Timor-Leste	70 to 74	12.2 (4.7–24.7)	12.2 (4.5–24.7)	12.1 (4.6–24.1)	12.3 (4.9–25.7)	12.3 (4.9–25.7)
Timor-Leste	75 to 79	11.0 (4.2–22.6)	11.0 (4.0–22.5)	10.9 (4.1–22.0)	11.1 (4.3–23.5)	11.1 (4.4–23.3)
Timor-Leste	80 to 84	7.8 (2.9–16.5)	7.8 (2.8–16.5)	7.8 (2.8–16.1)	7.8 (3.0–17.2)	7.9 (3.0–17.1)
Timor-Leste	85 to 89	7.0 (2.6–15.0)	7.0 (2.5–15.0)	7.0 (2.5–14.6)	7.1 (2.7–15.6)	7.1 (2.7–15.5)
Timor-Leste	90 to 94	6.4 (2.3–13.6)	6.4 (2.2–13.6)	6.3 (2.3–13.3)	6.4 (2.4–14.3)	6.4 (2.4–14.1)

Supplementary Table S11: Prevalence of male SVAC by age and location for 1990, 2000, 2010, 2020, and 2023						
**Location**	**Age Range**	**1990**	**2000**	**2010**	**2020**	**2023**
Timor-Leste	95 plus	6.3 (2.3–13.5)	6.3 (2.2–13.5)	6.3 (2.3–13.2)	6.3 (2.4–14.1)	6.3 (2.4–14.0)
Timor-Leste	Age-standardized	15.9 (6.3–30.9)	15.9 (6.1–30.9)	15.8 (6.2–30.3)	15.9 (6.6–32.0)	15.9 (6.6–31.9)
Timor-Leste	All age	16.2 (6.4–31.5)	16.1 (6.2–31.3)	15.9 (6.3–30.5)	16.0 (6.6–32.2)	16.0 (6.6–32.1)
Viet Nam	20 to 24	14.8 (5.8–29.3)	14.8 (5.6–29.2)	14.7 (5.7–28.5)	14.8 (6.0–30.3)	14.9 (6.1–30.1)
Viet Nam	25 to 29	15.2 (6.0–30.0)	15.2 (5.8–29.9)	15.1 (5.9–29.3)	15.3 (6.2–31.0)	15.3 (6.3–31.1)
Viet Nam	30 to 34	16.7 (6.7–32.4)	16.7 (6.4–32.3)	16.6 (6.6–31.7)	16.8 (6.9–33.5)	16.8 (7.0–33.3)
Viet Nam	35 to 39	17.1 (6.9–33.0)	17.1 (6.6–32.9)	17.0 (6.8–32.3)	17.2 (7.1–34.1)	17.2 (7.2–34.0)
Viet Nam	40 to 44	17.9 (7.2–34.3)	17.9 (7.0–34.2)	17.8 (7.1–33.6)	18.0 (7.5–35.5)	18.0 (7.6–35.3)
Viet Nam	45 to 49	17.6 (7.1–33.8)	17.6 (6.8–33.8)	17.5 (7.0–33.1)	17.7 (7.4–35.0)	17.7 (7.4–34.8)
Viet Nam	50 to 54	17.2 (6.9–33.2)	17.2 (6.7–33.2)	17.2 (6.8–32.6)	17.3 (7.2–34.4)	17.3 (7.3–34.5)
Viet Nam	55 to 59	16.7 (6.7–32.5)	16.7 (6.4–32.4)	16.7 (6.6–31.8)	16.8 (7.0–33.6)	16.8 (7.0–33.4)
Viet Nam	60 to 64	16.3 (6.5–31.7)	16.3 (6.2–31.7)	16.2 (6.4–31.1)	16.4 (6.7–32.8)	16.4 (6.8–32.6)
Viet Nam	65 to 69	15.2 (6.0–29.9)	15.2 (5.8–29.9)	15.1 (5.9–29.2)	15.3 (6.2–31.0)	15.3 (6.3–30.8)
Viet Nam	70 to 74	12.2 (4.7–24.7)	12.2 (4.5–24.7)	12.1 (4.6–24.1)	12.3 (4.9–25.7)	12.3 (4.9–25.7)
Viet Nam	75 to 79	11.0 (4.2–22.6)	11.0 (4.0–22.5)	10.9 (4.1–22.0)	11.1 (4.3–23.5)	11.1 (4.4–23.3)

Supplementary Table S11: Prevalence of male SVAC by age and location for 1990, 2000, 2010, 2020, and 2023						
**Location**	**Age Range**	**1990**	**2000**	**2010**	**2020**	**2023**
Viet Nam	80 to 84	7.8 (2.9–16.5)	7.8 (2.8–16.5)	7.8 (2.8–16.1)	7.8 (3.0–17.2)	7.9 (3.0–17.1)
Viet Nam	85 to 89	7.0 (2.6–15.0)	7.0 (2.5–15.0)	7.0 (2.5–14.6)	7.1 (2.7–15.6)	7.1 (2.7–15.5)
Viet Nam	90 to 94	6.4 (2.3–13.6)	6.4 (2.2–13.6)	6.3 (2.3–13.3)	6.4 (2.4–14.3)	6.4 (2.4–14.1)
Viet Nam	95 plus	6.3 (2.3–13.5)	6.3 (2.2–13.5)	6.3 (2.3–13.2)	6.3 (2.4–14.1)	6.3 (2.4–14.0)
Viet Nam	Age-standardized	15.9 (6.3–30.9)	15.9 (6.1–30.9)	15.8 (6.2–30.3)	15.9 (6.6–32.0)	15.9 (6.6–31.9)
Viet Nam	All age	15.9 (6.3–31.1)	16.1 (6.2–31.3)	16.1 (6.3–30.7)	16.3 (6.7–32.7)	16.3 (6.8–32.6)
Sub-Saharan Africa	20 to 24	18.8 (10.1–31.1)	18.4 (10.1–30.2)	17.4 (10.4–27.2)	17.0 (10.3–27.6)	17.1 (9.9–28.0)
Sub-Saharan Africa	25 to 29	19.1 (10.0–32.2)	18.8 (9.9–31.6)	18.0 (9.7–29.6)	17.5 (9.6–29.8)	17.5 (9.2–29.7)
Sub-Saharan Africa	30 to 34	20.4 (10.4–34.5)	20.3 (10.7–33.6)	19.9 (11.0–31.9)	19.5 (10.7–32.9)	19.5 (10.4–33.3)
Sub-Saharan Africa	35 to 39	20.4 (10.1–35.0)	20.2 (10.5–34.1)	19.8 (10.8–32.1)	19.7 (10.4–33.8)	19.7 (10.1–34.1)
Sub-Saharan Africa	40 to 44	21.2 (10.4–36.6)	21.1 (10.8–35.5)	21.3 (12.1–34.0)	21.5 (11.9–35.8)	21.5 (11.4–36.3)
Sub-Saharan Africa	45 to 49	20.8 (9.9–36.2)	20.6 (10.3–35.2)	20.6 (11.3–33.5)	20.6 (11.2–34.8)	20.6 (10.8–35.2)
Sub-Saharan Africa	50 to 54	20.3 (9.5–36.0)	20.2 (9.8–35.1)	20.0 (10.5–33.4)	19.9 (10.6–34.2)	20.0 (10.2–34.6)
Sub-Saharan Africa	55 to 59	20.2 (9.4–35.9)	20.4 (9.7–35.2)	20.3 (10.5–33.9)	20.2 (11.0–34.6)	20.3 (10.5–34.8)
Sub-Saharan Africa	60 to 64	19.6 (8.9–35.3)	19.7 (9.2–34.5)	19.5 (9.8–33.1)	19.3 (10.0–33.7)	19.3 (9.7–34.0)

Supplementary Table S11: Prevalence of male SVAC by age and location for 1990, 2000, 2010, 2020, and 2023						
**Location**	**Age Range**	**1990**	**2000**	**2010**	**2020**	**2023**
Sub-Saharan Africa	65 to 69	17.9 (7.5–33.3)	18.3 (7.4–34.4)	18.1 (7.6–33.1)	17.8 (7.8–34.2)	17.9 (8.0–34.2)
Sub-Saharan Africa	70 to 74	14.4 (5.8–27.7)	14.9 (5.7–29.0)	14.8 (5.8–28.2)	14.6 (6.0–29.7)	14.6 (6.1–29.7)
Sub-Saharan Africa	75 to 79	13.7 (5.4–26.8)	13.4 (5.1–26.6)	13.5 (5.3–26.1)	13.3 (5.4–27.4)	13.3 (5.5–27.4)
Sub-Saharan Africa	80 to 84	10.3 (3.9–20.9)	9.8 (3.6–20.2)	9.9 (3.7–20.0)	9.8 (3.8–21.0)	9.8 (3.9–21.0)
Sub-Saharan Africa	85 to 89	9.5 (3.5–19.4)	9.3 (3.4–19.3)	9.1 (3.4–18.5)	9.1 (3.5–19.7)	9.1 (3.6–19.6)
Sub-Saharan Africa	90 to 94	8.8 (3.2–18.1)	8.7 (3.1–18.1)	8.5 (3.1–17.3)	8.6 (3.3–18.6)	8.5 (3.3–18.6)
Sub-Saharan Africa	95 plus	8.7 (3.2–18.0)	8.7 (3.1–18.2)	8.8 (3.3–17.9)	8.7 (3.4–19.0)	8.8 (3.4–19.1)
Sub-Saharan Africa	Age-standardized	19.2 (9.4–33.4)	19.1 (9.6–32.9)	18.8 (9.9–31.1)	18.6 (9.9–32.0)	18.6 (9.7–32.3)
Sub-Saharan Africa	All age	19.6 (9.8–33.6)	19.4 (10.0–32.9)	19.0 (10.4–31.0)	18.8 (10.3–31.8)	18.8 (9.9–32.2)
Central sub-Saharan Africa	20 to 24	15.1 (5.9–29.7)	15.1 (5.7–29.7)	15.1 (5.9–29.2)	15.0 (6.1–30.6)	15.0 (6.2–30.2)
Central sub-Saharan Africa	25 to 29	15.5 (6.1–30.4)	15.5 (5.9–30.3)	15.5 (6.1–29.8)	15.4 (6.3–31.3)	15.4 (6.4–30.9)
Central sub-Saharan Africa	30 to 34	16.8 (6.7–32.5)	16.8 (6.5–32.5)	16.8 (6.7–32.0)	16.7 (6.9–33.5)	16.7 (7.0–33.1)
Central sub-Saharan Africa	35 to 39	17.0 (6.8–32.9)	17.0 (6.6–32.9)	17.0 (6.8–32.4)	17.0 (7.0–33.9)	17.0 (7.1–33.5)
Central sub-Saharan Africa	40 to 44	17.7 (7.2–34.0)	17.7 (6.9–34.0)	17.7 (7.1–33.5)	17.7 (7.4–35.0)	17.7 (7.5–34.6)
Central sub-Saharan Africa	45 to 49	17.4 (7.0–33.5)	17.4 (6.7–33.4)	17.4 (7.0–33.0)	17.4 (7.2–34.5)	17.3 (7.3–34.1)

Supplementary Table S11: Prevalence of male SVAC by age and location for 1990, 2000, 2010, 2020, and 2023						
**Location**	**Age Range**	**1990**	**2000**	**2010**	**2020**	**2023**
Central sub-Saharan Africa	50 to 54	17.1 (6.9–33.0)	17.1 (6.6–33.0)	17.1 (6.8–32.5)	17.1 (7.1–34.0)	17.0 (7.2–33.6)
Central sub-Saharan Africa	55 to 59	16.7 (6.7–32.3)	16.7 (6.4–32.3)	16.7 (6.6–31.8)	16.6 (6.9–33.3)	16.6 (6.9–32.9)
Central sub-Saharan Africa	60 to 64	16.2 (6.5–31.6)	16.2 (6.2–31.6)	16.3 (6.4–31.1)	16.2 (6.7–32.6)	16.2 (6.7–32.2)
Central sub-Saharan Africa	65 to 69	15.2 (6.0–29.9)	15.2 (5.8–29.8)	15.2 (6.0–29.4)	15.2 (6.2–30.8)	15.1 (6.2–30.4)
Central sub-Saharan Africa	70 to 74	12.2 (4.7–24.8)	12.2 (4.5–24.8)	12.3 (4.7–24.4)	12.2 (4.8–25.6)	12.2 (4.9–25.3)
Central sub-Saharan Africa	75 to 79	11.1 (4.2–22.7)	11.1 (4.0–22.6)	11.1 (4.2–22.3)	11.0 (4.3–23.4)	11.0 (4.4–23.1)
Central sub-Saharan Africa	80 to 84	7.9 (2.9–16.6)	7.9 (2.8–16.6)	7.9 (2.9–16.3)	7.9 (3.0–17.3)	7.8 (3.0–17.0)
Central sub-Saharan Africa	85 to 89	7.1 (2.6–15.1)	7.1 (2.5–15.1)	7.1 (2.6–14.9)	7.1 (2.7–15.7)	7.1 (2.7–15.5)
Central sub-Saharan Africa	90 to 94	6.5 (2.3–13.8)	6.4 (2.2–13.8)	6.5 (2.3–13.6)	6.4 (2.4–14.3)	6.4 (2.4–14.1)
Central sub-Saharan Africa	95 plus	6.4 (2.3–13.7)	6.4 (2.2–13.7)	6.4 (2.3–13.5)	6.4 (2.4–14.2)	6.4 (2.4–14.0)
Central sub-Saharan Africa	Age-standardized	15.9 (6.3–31.0)	15.9 (6.1–30.9)	15.9 (6.3–30.4)	15.9 (6.5–31.9)	15.8 (6.6–31.5)
Central sub-Saharan Africa	All age	16.1 (6.4–31.4)	16.2 (6.2–31.4)	16.2 (6.4–31.0)	16.2 (6.7–32.5)	16.1 (6.7–32.1)
Angola	20 to 24	15.1 (5.9–29.7)	15.1 (5.7–29.7)	15.1 (5.9–29.2)	15.0 (6.1–30.6)	15.0 (6.2–30.2)
Angola	25 to 29	15.5 (6.1–30.4)	15.5 (5.9–30.3)	15.5 (6.1–29.8)	15.4 (6.3–31.3)	15.4 (6.4–30.9)
Angola	30 to 34	16.8 (6.7–32.5)	16.8 (6.5–32.5)	16.8 (6.7–32.0)	16.7 (6.9–33.5)	16.7 (7.0–33.1)

Supplementary Table S11: Prevalence of male SVAC by age and location for 1990, 2000, 2010, 2020, and 2023						
**Location**	**Age Range**	**1990**	**2000**	**2010**	**2020**	**2023**
Angola	35 to 39	17.0 (6.8–32.9)	17.0 (6.6–32.9)	17.0 (6.8–32.4)	17.0 (7.0–33.9)	17.0 (7.1–33.5)
Angola	40 to 44	17.7 (7.2–34.0)	17.7 (6.9–34.0)	17.7 (7.1–33.5)	17.7 (7.4–35.0)	17.7 (7.5–34.6)
Angola	45 to 49	17.4 (7.0–33.5)	17.4 (6.7–33.4)	17.4 (7.0–33.0)	17.4 (7.2–34.5)	17.3 (7.3–34.1)
Angola	50 to 54	17.1 (6.9–33.0)	17.1 (6.6–33.0)	17.1 (6.8–32.5)	17.1 (7.1–34.0)	17.0 (7.2–33.6)
Angola	55 to 59	16.7 (6.7–32.3)	16.7 (6.4–32.3)	16.7 (6.6–31.8)	16.6 (6.9–33.3)	16.6 (6.9–32.9)
Angola	60 to 64	16.2 (6.5–31.6)	16.2 (6.2–31.6)	16.3 (6.4–31.1)	16.2 (6.7–32.6)	16.2 (6.7–32.2)
Angola	65 to 69	15.2 (6.0–29.9)	15.2 (5.8–29.8)	15.2 (6.0–29.4)	15.2 (6.2–30.8)	15.1 (6.2–30.4)
Angola	70 to 74	12.2 (4.7–24.8)	12.2 (4.5–24.8)	12.3 (4.7–24.4)	12.2 (4.8–25.6)	12.2 (4.9–25.3)
Angola	75 to 79	11.1 (4.2–22.7)	11.1 (4.0–22.6)	11.1 (4.2–22.3)	11.0 (4.3–23.4)	11.0 (4.4–23.1)
Angola	80 to 84	7.9 (2.9–16.6)	7.9 (2.8–16.6)	7.9 (2.9–16.3)	7.9 (3.0–17.3)	7.8 (3.0–17.0)
Angola	85 to 89	7.1 (2.6–15.1)	7.1 (2.5–15.1)	7.1 (2.6–14.9)	7.1 (2.7–15.7)	7.1 (2.7–15.5)
Angola	90 to 94	6.5 (2.3–13.8)	6.4 (2.2–13.8)	6.5 (2.3–13.6)	6.4 (2.4–14.3)	6.4 (2.4–14.1)
Angola	95 plus	6.4 (2.3–13.7)	6.4 (2.2–13.7)	6.4 (2.3–13.5)	6.4 (2.4–14.2)	6.4 (2.4–14.0)
Angola	Age-standardized	15.9 (6.3–31.0)	15.9 (6.1–30.9)	15.9 (6.3–30.4)	15.9 (6.5–31.9)	15.8 (6.6–31.5)
Angola	All age	16.2 (6.5–31.5)	16.2 (6.2–31.5)	16.2 (6.4–31.0)	16.2 (6.7–32.5)	16.1 (6.7–32.1)

Supplementary Table S11: Prevalence of male SVAC by age and location for 1990, 2000, 2010, 2020, and 2023						
**Location**	**Age Range**	**1990**	**2000**	**2010**	**2020**	**2023**
Central African Republic	20 to 24	15.1 (5.9–29.7)	15.1 (5.7–29.7)	15.1 (5.9–29.2)	15.0 (6.1–30.6)	15.0 (6.2–30.2)
Central African Republic	25 to 29	15.5 (6.1–30.4)	15.5 (5.9–30.3)	15.5 (6.1–29.8)	15.4 (6.3–31.3)	15.4 (6.4–30.9)
Central African Republic	30 to 34	16.8 (6.7–32.5)	16.8 (6.5–32.5)	16.8 (6.7–32.0)	16.7 (6.9–33.5)	16.7 (7.0–33.1)
Central African Republic	35 to 39	17.0 (6.8–32.9)	17.0 (6.6–32.9)	17.0 (6.8–32.4)	17.0 (7.0–33.9)	17.0 (7.1–33.5)
Central African Republic	40 to 44	17.7 (7.2–34.0)	17.7 (6.9–34.0)	17.7 (7.1–33.5)	17.7 (7.4–35.0)	17.7 (7.5–34.6)
Central African Republic	45 to 49	17.4 (7.0–33.5)	17.4 (6.7–33.4)	17.4 (7.0–33.0)	17.4 (7.2–34.5)	17.3 (7.3–34.1)
Central African Republic	50 to 54	17.1 (6.9–33.0)	17.1 (6.6–33.0)	17.1 (6.8–32.5)	17.1 (7.1–34.0)	17.0 (7.2–33.6)
Central African Republic	55 to 59	16.7 (6.7–32.3)	16.7 (6.4–32.3)	16.7 (6.6–31.8)	16.6 (6.9–33.3)	16.6 (6.9–32.9)
Central African Republic	60 to 64	16.2 (6.5–31.6)	16.2 (6.2–31.6)	16.3 (6.4–31.1)	16.2 (6.7–32.6)	16.2 (6.7–32.2)
Central African Republic	65 to 69	15.2 (6.0–29.9)	15.2 (5.8–29.8)	15.2 (6.0–29.4)	15.2 (6.2–30.8)	15.1 (6.2–30.4)
Central African Republic	70 to 74	12.2 (4.7–24.8)	12.2 (4.5–24.8)	12.3 (4.7–24.4)	12.2 (4.8–25.6)	12.2 (4.9–25.3)
Central African Republic	75 to 79	11.1 (4.2–22.7)	11.1 (4.0–22.6)	11.1 (4.2–22.3)	11.0 (4.3–23.4)	11.0 (4.4–23.1)
Central African Republic	80 to 84	7.9 (2.9–16.6)	7.9 (2.8–16.6)	7.9 (2.9–16.3)	7.9 (3.0–17.3)	7.8 (3.0–17.0)
Central African Republic	85 to 89	7.1 (2.6–15.1)	7.1 (2.5–15.1)	7.1 (2.6–14.9)	7.1 (2.7–15.7)	7.1 (2.7–15.5)
Central African Republic	90 to 94	6.5 (2.3–13.8)	6.4 (2.2–13.8)	6.5 (2.3–13.6)	6.4 (2.4–14.3)	6.4 (2.4–14.1)

Supplementary Table S11: Prevalence of male SVAC by age and location for 1990, 2000, 2010, 2020, and 2023						
**Location**	**Age Range**	**1990**	**2000**	**2010**	**2020**	**2023**
Central African Republic	95 plus	6.4 (2.3–13.7)	6.4 (2.2–13.7)	6.4 (2.3–13.5)	6.4 (2.4–14.2)	6.4 (2.4–14.0)
Central African Republic	Age-standardized	15.9 (6.3–31.0)	15.9 (6.1–30.9)	15.9 (6.3–30.4)	15.9 (6.5–31.9)	15.8 (6.6–31.5)
Central African Republic	All age	16.2 (6.4–31.5)	16.2 (6.2–31.5)	16.3 (6.4–31.1)	16.3 (6.7–32.6)	16.2 (6.7–32.2)
Congo (Brazzaville)	20 to 24	15.1 (5.9–29.7)	15.1 (5.7–29.7)	15.1 (5.9–29.2)	15.0 (6.1–30.6)	15.0 (6.2–30.2)
Congo (Brazzaville)	25 to 29	15.5 (6.1–30.4)	15.5 (5.9–30.3)	15.5 (6.1–29.8)	15.4 (6.3–31.3)	15.4 (6.4–30.9)
Congo (Brazzaville)	30 to 34	16.8 (6.7–32.5)	16.8 (6.5–32.5)	16.8 (6.7–32.0)	16.7 (6.9–33.5)	16.7 (7.0–33.1)
Congo (Brazzaville)	35 to 39	17.0 (6.8–32.9)	17.0 (6.6–32.9)	17.0 (6.8–32.4)	17.0 (7.0–33.9)	17.0 (7.1–33.5)
Congo (Brazzaville)	40 to 44	17.7 (7.2–34.0)	17.7 (6.9–34.0)	17.7 (7.1–33.5)	17.7 (7.4–35.0)	17.7 (7.5–34.6)
Congo (Brazzaville)	45 to 49	17.4 (7.0–33.5)	17.4 (6.7–33.4)	17.4 (7.0–33.0)	17.4 (7.2–34.5)	17.3 (7.3–34.1)
Congo (Brazzaville)	50 to 54	17.1 (6.9–33.0)	17.1 (6.6–33.0)	17.1 (6.8–32.5)	17.1 (7.1–34.0)	17.0 (7.2–33.6)
Congo (Brazzaville)	55 to 59	16.7 (6.7–32.3)	16.7 (6.4–32.3)	16.7 (6.6–31.8)	16.6 (6.9–33.3)	16.6 (6.9–32.9)
Congo (Brazzaville)	60 to 64	16.2 (6.5–31.6)	16.2 (6.2–31.6)	16.3 (6.4–31.1)	16.2 (6.7–32.6)	16.2 (6.7–32.2)
Congo (Brazzaville)	65 to 69	15.2 (6.0–29.9)	15.2 (5.8–29.8)	15.2 (6.0–29.4)	15.2 (6.2–30.8)	15.1 (6.2–30.4)
Congo (Brazzaville)	70 to 74	12.2 (4.7–24.8)	12.2 (4.5–24.8)	12.3 (4.7–24.4)	12.2 (4.8–25.6)	12.2 (4.9–25.3)
Congo (Brazzaville)	75 to 79	11.1 (4.2–22.7)	11.1 (4.0–22.6)	11.1 (4.2–22.3)	11.0 (4.3–23.4)	11.0 (4.4–23.1)

Supplementary Table S11: Prevalence of male SVAC by age and location for 1990, 2000, 2010, 2020, and 2023						
**Location**	**Age Range**	**1990**	**2000**	**2010**	**2020**	**2023**
Congo (Brazzaville)	80 to 84	7.9 (2.9–16.6)	7.9 (2.8–16.6)	7.9 (2.9–16.3)	7.9 (3.0–17.3)	7.8 (3.0–17.0)
Congo (Brazzaville)	85 to 89	7.1 (2.6–15.1)	7.1 (2.5–15.1)	7.1 (2.6–14.9)	7.1 (2.7–15.7)	7.1 (2.7–15.5)
Congo (Brazzaville)	90 to 94	6.5 (2.3–13.8)	6.4 (2.2–13.8)	6.5 (2.3–13.6)	6.4 (2.4–14.3)	6.4 (2.4–14.1)
Congo (Brazzaville)	95 plus	6.4 (2.3–13.7)	6.4 (2.2–13.7)	6.4 (2.3–13.5)	6.4 (2.4–14.2)	6.4 (2.4–14.0)
Congo (Brazzaville)	Age-standardized	15.9 (6.3–31.0)	15.9 (6.1–30.9)	15.9 (6.3–30.4)	15.9 (6.5–31.9)	15.8 (6.6–31.5)
Congo (Brazzaville)	All age	16.1 (6.4–31.4)	16.1 (6.2–31.4)	16.2 (6.4–31.1)	16.3 (6.7–32.6)	16.2 (6.8–32.2)
DR Congo	20 to 24	15.1 (5.9–29.7)	15.1 (5.7–29.7)	15.1 (5.9–29.2)	15.0 (6.1–30.6)	15.0 (6.2–30.2)
DR Congo	25 to 29	15.5 (6.1–30.4)	15.5 (5.9–30.3)	15.5 (6.1–29.8)	15.4 (6.3–31.3)	15.4 (6.4–30.9)
DR Congo	30 to 34	16.8 (6.7–32.5)	16.8 (6.5–32.5)	16.8 (6.7–32.0)	16.7 (6.9–33.5)	16.7 (7.0–33.1)
DR Congo	35 to 39	17.0 (6.8–32.9)	17.0 (6.6–32.9)	17.0 (6.8–32.4)	17.0 (7.0–33.9)	17.0 (7.1–33.5)
DR Congo	40 to 44	17.7 (7.2–34.0)	17.7 (6.9–34.0)	17.7 (7.1–33.5)	17.7 (7.4–35.0)	17.7 (7.5–34.6)
DR Congo	45 to 49	17.4 (7.0–33.5)	17.4 (6.7–33.4)	17.4 (7.0–33.0)	17.4 (7.2–34.5)	17.3 (7.3–34.1)
DR Congo	50 to 54	17.1 (6.9–33.0)	17.1 (6.6–33.0)	17.1 (6.8–32.5)	17.1 (7.1–34.0)	17.0 (7.2–33.6)
DR Congo	55 to 59	16.7 (6.7–32.3)	16.7 (6.4–32.3)	16.7 (6.6–31.8)	16.6 (6.9–33.3)	16.6 (6.9–32.9)
DR Congo	60 to 64	16.2 (6.5–31.6)	16.2 (6.2–31.6)	16.3 (6.4–31.1)	16.2 (6.7–32.6)	16.2 (6.7–32.2)

Supplementary Table S11: Prevalence of male SVAC by age and location for 1990, 2000, 2010, 2020, and 2023						
**Location**	**Age Range**	**1990**	**2000**	**2010**	**2020**	**2023**
DR Congo	65 to 69	15.2 (6.0–29.9)	15.2 (5.8–29.8)	15.2 (6.0–29.4)	15.2 (6.2–30.8)	15.1 (6.2–30.4)
DR Congo	70 to 74	12.2 (4.7–24.8)	12.2 (4.5–24.8)	12.3 (4.7–24.4)	12.2 (4.8–25.6)	12.2 (4.9–25.3)
DR Congo	75 to 79	11.1 (4.2–22.7)	11.1 (4.0–22.6)	11.1 (4.2–22.3)	11.0 (4.3–23.4)	11.0 (4.4–23.1)
DR Congo	80 to 84	7.9 (2.9–16.6)	7.9 (2.8–16.6)	7.9 (2.9–16.3)	7.9 (3.0–17.3)	7.8 (3.0–17.0)
DR Congo	85 to 89	7.1 (2.6–15.1)	7.1 (2.5–15.1)	7.1 (2.6–14.9)	7.1 (2.7–15.7)	7.1 (2.7–15.5)
DR Congo	90 to 94	6.5 (2.3–13.8)	6.4 (2.2–13.8)	6.5 (2.3–13.6)	6.4 (2.4–14.3)	6.4 (2.4–14.1)
DR Congo	95 plus	6.4 (2.3–13.7)	6.4 (2.2–13.7)	6.4 (2.3–13.5)	6.4 (2.4–14.2)	6.4 (2.4–14.0)
DR Congo	Age-standardized	15.9 (6.3–31.0)	15.9 (6.1–30.9)	15.9 (6.3–30.4)	15.9 (6.5–31.9)	15.8 (6.6–31.5)
DR Congo	All age	16.1 (6.4–31.4)	16.2 (6.2–31.4)	16.2 (6.4–31.0)	16.2 (6.7–32.5)	16.1 (6.7–32.1)
Equatorial Guinea	20 to 24	15.1 (5.9–29.7)	15.1 (5.7–29.7)	15.1 (5.9–29.2)	15.0 (6.1–30.6)	15.0 (6.2–30.2)
Equatorial Guinea	25 to 29	15.5 (6.1–30.4)	15.5 (5.9–30.3)	15.5 (6.1–29.8)	15.4 (6.3–31.3)	15.4 (6.4–30.9)
Equatorial Guinea	30 to 34	16.8 (6.7–32.5)	16.8 (6.5–32.5)	16.8 (6.7–32.0)	16.7 (6.9–33.5)	16.7 (7.0–33.1)
Equatorial Guinea	35 to 39	17.0 (6.8–32.9)	17.0 (6.6–32.9)	17.0 (6.8–32.4)	17.0 (7.0–33.9)	17.0 (7.1–33.5)
Equatorial Guinea	40 to 44	17.7 (7.2–34.0)	17.7 (6.9–34.0)	17.7 (7.1–33.5)	17.7 (7.4–35.0)	17.7 (7.5–34.6)
Equatorial Guinea	45 to 49	17.4 (7.0–33.5)	17.4 (6.7–33.4)	17.4 (7.0–33.0)	17.4 (7.2–34.5)	17.3 (7.3–34.1)

Supplementary Table S11: Prevalence of male SVAC by age and location for 1990, 2000, 2010, 2020, and 2023						
**Location**	**Age Range**	**1990**	**2000**	**2010**	**2020**	**2023**
Equatorial Guinea	50 to 54	17.1 (6.9–33.0)	17.1 (6.6–33.0)	17.1 (6.8–32.5)	17.1 (7.1–34.0)	17.0 (7.2–33.6)
Equatorial Guinea	55 to 59	16.7 (6.7–32.3)	16.7 (6.4–32.3)	16.7 (6.6–31.8)	16.6 (6.9–33.3)	16.6 (6.9–32.9)
Equatorial Guinea	60 to 64	16.2 (6.5–31.6)	16.2 (6.2–31.6)	16.3 (6.4–31.1)	16.2 (6.7–32.6)	16.2 (6.7–32.2)
Equatorial Guinea	65 to 69	15.2 (6.0–29.9)	15.2 (5.8–29.8)	15.2 (6.0–29.4)	15.2 (6.2–30.8)	15.1 (6.2–30.4)
Equatorial Guinea	70 to 74	12.2 (4.7–24.8)	12.2 (4.5–24.8)	12.3 (4.7–24.4)	12.2 (4.8–25.6)	12.2 (4.9–25.3)
Equatorial Guinea	75 to 79	11.1 (4.2–22.7)	11.1 (4.0–22.6)	11.1 (4.2–22.3)	11.0 (4.3–23.4)	11.0 (4.4–23.1)
Equatorial Guinea	80 to 84	7.9 (2.9–16.6)	7.9 (2.8–16.6)	7.9 (2.9–16.3)	7.9 (3.0–17.3)	7.8 (3.0–17.0)
Equatorial Guinea	85 to 89	7.1 (2.6–15.1)	7.1 (2.5–15.1)	7.1 (2.6–14.9)	7.1 (2.7–15.7)	7.1 (2.7–15.5)
Equatorial Guinea	90 to 94	6.5 (2.3–13.8)	6.4 (2.2–13.8)	6.5 (2.3–13.6)	6.4 (2.4–14.3)	6.4 (2.4–14.1)
Equatorial Guinea	95 plus	6.4 (2.3–13.7)	6.4 (2.2–13.7)	6.4 (2.3–13.5)	6.4 (2.4–14.2)	6.4 (2.4–14.0)
Equatorial Guinea	Age-standardized	15.9 (6.3–31.0)	15.9 (6.1–30.9)	15.9 (6.3–30.4)	15.9 (6.5–31.9)	15.8 (6.6–31.5)
Equatorial Guinea	All age	16.1 (6.4–31.4)	16.1 (6.2–31.3)	16.0 (6.3–30.7)	16.0 (6.6–32.3)	16.1 (6.7–32.0)
Gabon	20 to 24	15.1 (5.9–29.7)	15.1 (5.7–29.7)	15.1 (5.9–29.2)	15.0 (6.1–30.6)	15.0 (6.2–30.2)
Gabon	25 to 29	15.5 (6.1–30.4)	15.5 (5.9–30.3)	15.5 (6.1–29.8)	15.4 (6.3–31.3)	15.4 (6.4–30.9)
Gabon	30 to 34	16.8 (6.7–32.5)	16.8 (6.5–32.5)	16.8 (6.7–32.0)	16.7 (6.9–33.5)	16.7 (7.0–33.1)

Supplementary Table S11: Prevalence of male SVAC by age and location for 1990, 2000, 2010, 2020, and 2023						
**Location**	**Age Range**	**1990**	**2000**	**2010**	**2020**	**2023**
Gabon	35 to 39	17.0 (6.8–32.9)	17.0 (6.6–32.9)	17.0 (6.8–32.4)	17.0 (7.0–33.9)	17.0 (7.1–33.5)
Gabon	40 to 44	17.7 (7.2–34.0)	17.7 (6.9–34.0)	17.7 (7.1–33.5)	17.7 (7.4–35.0)	17.7 (7.5–34.6)
Gabon	45 to 49	17.4 (7.0–33.5)	17.4 (6.7–33.4)	17.4 (7.0–33.0)	17.4 (7.2–34.5)	17.3 (7.3–34.1)
Gabon	50 to 54	17.1 (6.9–33.0)	17.1 (6.6–33.0)	17.1 (6.8–32.5)	17.1 (7.1–34.0)	17.0 (7.2–33.6)
Gabon	55 to 59	16.7 (6.7–32.3)	16.7 (6.4–32.3)	16.7 (6.6–31.8)	16.6 (6.9–33.3)	16.6 (6.9–32.9)
Gabon	60 to 64	16.2 (6.5–31.6)	16.2 (6.2–31.6)	16.3 (6.4–31.1)	16.2 (6.7–32.6)	16.2 (6.7–32.2)
Gabon	65 to 69	15.2 (6.0–29.9)	15.2 (5.8–29.8)	15.2 (6.0–29.4)	15.2 (6.2–30.8)	15.1 (6.2–30.4)
Gabon	70 to 74	12.2 (4.7–24.8)	12.2 (4.5–24.8)	12.3 (4.7–24.4)	12.2 (4.8–25.6)	12.2 (4.9–25.3)
Gabon	75 to 79	11.1 (4.2–22.7)	11.1 (4.0–22.6)	11.1 (4.2–22.3)	11.0 (4.3–23.4)	11.0 (4.4–23.1)
Gabon	80 to 84	7.9 (2.9–16.6)	7.9 (2.8–16.6)	7.9 (2.9–16.3)	7.9 (3.0–17.3)	7.8 (3.0–17.0)
Gabon	85 to 89	7.1 (2.6–15.1)	7.1 (2.5–15.1)	7.1 (2.6–14.9)	7.1 (2.7–15.7)	7.1 (2.7–15.5)
Gabon	90 to 94	6.5 (2.3–13.8)	6.4 (2.2–13.8)	6.5 (2.3–13.6)	6.4 (2.4–14.3)	6.4 (2.4–14.1)
Gabon	95 plus	6.4 (2.3–13.7)	6.4 (2.2–13.7)	6.4 (2.3–13.5)	6.4 (2.4–14.2)	6.4 (2.4–14.0)
Gabon	Age-standardized	15.9 (6.3–31.0)	15.9 (6.1–30.9)	15.9 (6.3–30.4)	15.9 (6.5–31.9)	15.8 (6.6–31.5)
Gabon	All age	16.0 (6.4–31.2)	16.1 (6.2–31.4)	16.2 (6.4–31.0)	16.2 (6.7–32.5)	16.1 (6.7–32.1)

Supplementary Table S11: Prevalence of male SVAC by age and location for 1990, 2000, 2010, 2020, and 2023						
**Location**	**Age Range**	**1990**	**2000**	**2010**	**2020**	**2023**
Eastern sub-Saharan Africa	20 to 24	15.5 (8.9–25.0)	15.5 (10.2–22.7)	15.3 (11.7–20.1)	15.0 (11.7–19.8)	15.1 (11.0–20.6)
Eastern sub-Saharan Africa	25 to 29	15.4 (7.9–26.2)	15.7 (8.9–25.1)	15.9 (10.2–23.8)	15.7 (9.8–24.3)	15.7 (9.3–24.9)
Eastern sub-Saharan Africa	30 to 34	16.3 (8.2–28.1)	16.4 (9.0–27.1)	16.7 (10.0–26.0)	16.4 (10.6–25.5)	16.3 (10.4–25.2)
Eastern sub-Saharan Africa	35 to 39	15.7 (7.7–27.5)	15.9 (8.2–27.3)	16.5 (9.4–26.7)	16.8 (9.6–28.0)	16.7 (9.3–27.9)
Eastern sub-Saharan Africa	40 to 44	16.1 (7.7–28.6)	16.2 (8.0–27.8)	16.8 (9.2–27.6)	17.4 (10.1–28.8)	17.5 (10.0–29.2)
Eastern sub-Saharan Africa	45 to 49	15.7 (7.2–28.6)	15.7 (7.5–27.9)	16.2 (8.2–27.4)	16.6 (8.7–29.2)	16.7 (8.5–29.5)
Eastern sub-Saharan Africa	50 to 54	15.5 (6.8–28.8)	15.5 (6.9–28.3)	15.9 (7.5–27.8)	16.0 (8.1–28.9)	16.1 (8.3–28.9)
Eastern sub-Saharan Africa	55 to 59	15.3 (6.6–28.5)	15.2 (6.5–28.2)	15.5 (7.2–27.4)	15.8 (7.9–28.6)	15.8 (8.0–28.5)
Eastern sub-Saharan Africa	60 to 64	14.6 (5.9–27.9)	14.7 (6.1–28.4)	14.8 (6.4–27.0)	15.0 (7.2–28.1)	15.1 (7.2–27.8)
Eastern sub-Saharan Africa	65 to 69	13.8 (5.4–26.6)	13.8 (5.5–27.0)	13.9 (5.7–26.1)	14.1 (6.4–26.9)	14.1 (6.6–26.6)
Eastern sub-Saharan Africa	70 to 74	10.8 (4.1–21.6)	11.0 (4.0–22.3)	11.1 (4.2–22.0)	11.3 (4.5–23.6)	11.4 (4.5–23.5)
Eastern sub-Saharan Africa	75 to 79	10.8 (4.1–21.7)	10.0 (3.6–20.5)	9.8 (3.7–19.8)	9.9 (3.9–21.1)	10.1 (4.0–21.1)
Eastern sub-Saharan Africa	80 to 84	8.3 (3.0–17.2)	7.3 (2.6–15.4)	7.1 (2.6–14.6)	7.1 (2.7–15.6)	7.1 (2.7–15.5)
Eastern sub-Saharan Africa	85 to 89	7.6 (2.7–16.0)	7.4 (2.6–15.8)	6.7 (2.5–14.0)	6.5 (2.5–14.3)	6.5 (2.5–14.2)
Eastern sub-Saharan Africa	90 to 94	6.8 (2.4–14.5)	7.0 (2.4–14.9)	6.4 (2.3–13.3)	6.0 (2.3–13.4)	6.0 (2.3–13.2)

Supplementary Table S11: Prevalence of male SVAC by age and location for 1990, 2000, 2010, 2020, and 2023						
**Location**	**Age Range**	**1990**	**2000**	**2010**	**2020**	**2023**
Eastern sub-Saharan Africa	95 plus	6.7 (2.4–14.2)	6.9 (2.4–14.7)	6.9 (2.5–14.4)	6.4 (2.4–14.1)	6.2 (2.4–13.7)
Eastern sub-Saharan Africa	Age-standardized	15.0 (7.2–26.6)	15.1 (7.7–25.9)	15.3 (8.6–25.0)	15.4 (9.2–25.4)	15.4 (8.8–25.8)
Eastern sub-Saharan Africa	All age	15.4 (7.7–26.7)	15.5 (8.4–25.9)	15.8 (9.5–24.6)	15.8 (9.8–25.1)	15.8 (9.5–25.3)
Burundi	20 to 24	13.3 (5.1–26.6)	13.4 (5.0–26.7)	13.5 (5.2–26.5)	13.6 (5.5–28.1)	13.6 (5.5–27.7)
Burundi	25 to 29	13.4 (5.2–26.9)	13.5 (5.1–27.0)	13.7 (5.3–26.9)	13.8 (5.5–28.4)	13.8 (5.6–28.1)
Burundi	30 to 34	14.4 (5.6–28.6)	14.5 (5.5–28.7)	14.7 (5.7–28.5)	14.8 (6.0–30.1)	14.7 (6.1–29.8)
Burundi	35 to 39	14.4 (5.6–28.6)	14.5 (5.5–28.7)	14.7 (5.7–28.5)	14.8 (6.0–30.1)	14.7 (6.1–29.8)
Burundi	40 to 44	14.8 (5.8–29.2)	14.9 (5.6–29.4)	15.1 (5.9–29.2)	15.1 (6.2–30.8)	15.1 (6.2–30.4)
Burundi	45 to 49	14.3 (5.6–28.4)	14.4 (5.4–28.5)	14.6 (5.7–28.3)	14.6 (5.9–29.9)	14.6 (6.0–29.5)
Burundi	50 to 54	13.9 (5.4–27.7)	14.0 (5.3–27.8)	14.2 (5.5–27.6)	14.2 (5.7–29.2)	14.2 (5.8–28.8)
Burundi	55 to 59	13.4 (5.2–26.9)	13.5 (5.0–27.0)	13.7 (5.3–26.8)	13.7 (5.5–28.2)	13.7 (5.6–27.9)
Burundi	60 to 64	13.0 (5.0–26.1)	13.0 (4.8–26.2)	13.2 (5.1–25.9)	13.2 (5.3–27.4)	13.2 (5.3–27.0)
Burundi	65 to 69	12.1 (4.6–24.4)	12.1 (4.5–24.5)	12.2 (4.7–24.3)	12.3 (4.9–25.7)	12.2 (4.9–25.4)
Burundi	70 to 74	9.6 (3.6–19.9)	9.7 (3.5–20.0)	9.8 (3.6–19.8)	9.8 (3.8–21.0)	9.7 (3.8–20.7)
Burundi	75 to 79	8.6 (3.2–18.1)	8.7 (3.1–18.2)	8.8 (3.2–18.0)	8.8 (3.4–19.1)	8.8 (3.4–18.8)

Supplementary Table S11: Prevalence of male SVAC by age and location for 1990, 2000, 2010, 2020, and 2023						
**Location**	**Age Range**	**1990**	**2000**	**2010**	**2020**	**2023**
Burundi	80 to 84	6.1 (2.2–13.1)	6.1 (2.1–13.1)	6.2 (2.2–13.0)	6.2 (2.3–13.8)	6.2 (2.3–13.6)
Burundi	85 to 89	5.5 (2.0–11.8)	5.5 (1.9–11.8)	5.5 (2.0–11.7)	5.6 (2.1–12.5)	5.5 (2.1–12.3)
Burundi	90 to 94	4.9 (1.8–10.7)	4.9 (1.7–10.7)	5.0 (1.8–10.6)	5.0 (1.9–11.4)	5.0 (1.9–11.2)
Burundi	95 plus	4.9 (1.7–10.6)	4.9 (1.7–10.7)	5.0 (1.8–10.5)	5.0 (1.8–11.3)	5.0 (1.9–11.1)
Burundi	Age-standardized	13.3 (5.2–26.6)	13.4 (5.0–26.7)	13.5 (5.2–26.5)	13.6 (5.5–28.0)	13.6 (5.5–27.6)
Burundi	All age	13.6 (5.3–27.1)	13.8 (5.2–27.4)	13.9 (5.4–27.2)	14.0 (5.7–28.8)	14.0 (5.7–28.5)
Comoros	20 to 24	13.3 (5.1–26.6)	13.4 (5.0–26.7)	13.5 (5.2–26.5)	13.6 (5.5–28.1)	13.6 (5.5–27.7)
Comoros	25 to 29	13.4 (5.2–26.9)	13.5 (5.1–27.0)	13.7 (5.3–26.9)	13.8 (5.5–28.4)	13.8 (5.6–28.1)
Comoros	30 to 34	14.4 (5.6–28.6)	14.5 (5.5–28.7)	14.7 (5.7–28.5)	14.8 (6.0–30.1)	14.7 (6.1–29.8)
Comoros	35 to 39	14.4 (5.6–28.6)	14.5 (5.5–28.7)	14.7 (5.7–28.5)	14.8 (6.0–30.1)	14.7 (6.1–29.8)
Comoros	40 to 44	14.8 (5.8–29.2)	14.9 (5.6–29.4)	15.1 (5.9–29.2)	15.1 (6.2–30.8)	15.1 (6.2–30.4)
Comoros	45 to 49	14.3 (5.6–28.4)	14.4 (5.4–28.5)	14.6 (5.7–28.3)	14.6 (5.9–29.9)	14.6 (6.0–29.5)
Comoros	50 to 54	13.9 (5.4–27.7)	14.0 (5.3–27.8)	14.2 (5.5–27.6)	14.2 (5.7–29.2)	14.2 (5.8–28.8)
Comoros	55 to 59	13.4 (5.2–26.9)	13.5 (5.0–27.0)	13.7 (5.3–26.8)	13.7 (5.5–28.2)	13.7 (5.6–27.9)
Comoros	60 to 64	13.0 (5.0–26.1)	13.0 (4.8–26.2)	13.2 (5.1–25.9)	13.2 (5.3–27.4)	13.2 (5.3–27.0)

Supplementary Table S11: Prevalence of male SVAC by age and location for 1990, 2000, 2010, 2020, and 2023						
**Location**	**Age Range**	**1990**	**2000**	**2010**	**2020**	**2023**
Comoros	65 to 69	12.1 (4.6–24.4)	12.1 (4.5–24.5)	12.2 (4.7–24.3)	12.3 (4.9–25.7)	12.2 (4.9–25.4)
Comoros	70 to 74	9.6 (3.6–19.9)	9.7 (3.5–20.0)	9.8 (3.6–19.8)	9.8 (3.8–21.0)	9.7 (3.8–20.7)
Comoros	75 to 79	8.6 (3.2–18.1)	8.7 (3.1–18.2)	8.8 (3.2–18.0)	8.8 (3.4–19.1)	8.8 (3.4–18.8)
Comoros	80 to 84	6.1 (2.2–13.1)	6.1 (2.1–13.1)	6.2 (2.2–13.0)	6.2 (2.3–13.8)	6.2 (2.3–13.6)
Comoros	85 to 89	5.5 (2.0–11.8)	5.5 (1.9–11.8)	5.5 (2.0–11.7)	5.6 (2.1–12.5)	5.5 (2.1–12.3)
Comoros	90 to 94	4.9 (1.8–10.7)	4.9 (1.7–10.7)	5.0 (1.8–10.6)	5.0 (1.9–11.4)	5.0 (1.9–11.2)
Comoros	95 plus	4.9 (1.7–10.6)	4.9 (1.7–10.7)	5.0 (1.8–10.5)	5.0 (1.8–11.3)	5.0 (1.9–11.1)
Comoros	Age-standardized	13.3 (5.2–26.6)	13.4 (5.0–26.7)	13.5 (5.2–26.5)	13.6 (5.5–28.0)	13.6 (5.5–27.6)
Comoros	All age	13.6 (5.3–27.1)	13.7 (5.1–27.3)	13.9 (5.4–27.1)	13.9 (5.6–28.6)	13.9 (5.7–28.2)
Djibouti	20 to 24	13.3 (5.1–26.6)	13.4 (5.0–26.7)	13.5 (5.2–26.5)	13.6 (5.5–28.1)	13.6 (5.5–27.7)
Djibouti	25 to 29	13.4 (5.2–26.9)	13.5 (5.1–27.0)	13.7 (5.3–26.9)	13.8 (5.5–28.4)	13.8 (5.6–28.1)
Djibouti	30 to 34	14.4 (5.6–28.6)	14.5 (5.5–28.7)	14.7 (5.7–28.5)	14.8 (6.0–30.1)	14.7 (6.1–29.8)
Djibouti	35 to 39	14.4 (5.6–28.6)	14.5 (5.5–28.7)	14.7 (5.7–28.5)	14.8 (6.0–30.1)	14.7 (6.1–29.8)
Djibouti	40 to 44	14.8 (5.8–29.2)	14.9 (5.6–29.4)	15.1 (5.9–29.2)	15.1 (6.2–30.8)	15.1 (6.2–30.4)
Djibouti	45 to 49	14.3 (5.6–28.4)	14.4 (5.4–28.5)	14.6 (5.7–28.3)	14.6 (5.9–29.9)	14.6 (6.0–29.5)

Supplementary Table S11: Prevalence of male SVAC by age and location for 1990, 2000, 2010, 2020, and 2023						
**Location**	**Age Range**	**1990**	**2000**	**2010**	**2020**	**2023**
Djibouti	50 to 54	13.9 (5.4–27.7)	14.0 (5.3–27.8)	14.2 (5.5–27.6)	14.2 (5.7–29.2)	14.2 (5.8–28.8)
Djibouti	55 to 59	13.4 (5.2–26.9)	13.5 (5.0–27.0)	13.7 (5.3–26.8)	13.7 (5.5–28.2)	13.7 (5.6–27.9)
Djibouti	60 to 64	13.0 (5.0–26.1)	13.0 (4.8–26.2)	13.2 (5.1–25.9)	13.2 (5.3–27.4)	13.2 (5.3–27.0)
Djibouti	65 to 69	12.1 (4.6–24.4)	12.1 (4.5–24.5)	12.2 (4.7–24.3)	12.3 (4.9–25.7)	12.2 (4.9–25.4)
Djibouti	70 to 74	9.6 (3.6–19.9)	9.7 (3.5–20.0)	9.8 (3.6–19.8)	9.8 (3.8–21.0)	9.7 (3.8–20.7)
Djibouti	75 to 79	8.6 (3.2–18.1)	8.7 (3.1–18.2)	8.8 (3.2–18.0)	8.8 (3.4–19.1)	8.8 (3.4–18.8)
Djibouti	80 to 84	6.1 (2.2–13.1)	6.1 (2.1–13.1)	6.2 (2.2–13.0)	6.2 (2.3–13.8)	6.2 (2.3–13.6)
Djibouti	85 to 89	5.5 (2.0–11.8)	5.5 (1.9–11.8)	5.5 (2.0–11.7)	5.6 (2.1–12.5)	5.5 (2.1–12.3)
Djibouti	90 to 94	4.9 (1.8–10.7)	4.9 (1.7–10.7)	5.0 (1.8–10.6)	5.0 (1.9–11.4)	5.0 (1.9–11.2)
Djibouti	95 plus	4.9 (1.7–10.6)	4.9 (1.7–10.7)	5.0 (1.8–10.5)	5.0 (1.8–11.3)	5.0 (1.9–11.1)
Djibouti	Age-standardized	13.3 (5.2–26.6)	13.4 (5.0–26.7)	13.5 (5.2–26.5)	13.6 (5.5–28.0)	13.6 (5.5–27.6)
Djibouti	All age	13.8 (5.4–27.4)	13.9 (5.2–27.6)	14.0 (5.4–27.4)	14.1 (5.7–28.9)	14.0 (5.7–28.5)
Eritrea	20 to 24	13.3 (5.1–26.6)	13.4 (5.0–26.7)	13.5 (5.2–26.5)	13.6 (5.5–28.1)	13.6 (5.5–27.7)
Eritrea	25 to 29	13.4 (5.2–26.9)	13.5 (5.1–27.0)	13.7 (5.3–26.9)	13.8 (5.5–28.4)	13.8 (5.6–28.1)
Eritrea	30 to 34	14.4 (5.6–28.6)	14.5 (5.5–28.7)	14.7 (5.7–28.5)	14.8 (6.0–30.1)	14.7 (6.1–29.8)

Supplementary Table S11: Prevalence of male SVAC by age and location for 1990, 2000, 2010, 2020, and 2023						
**Location**	**Age Range**	**1990**	**2000**	**2010**	**2020**	**2023**
Eritrea	35 to 39	14.4 (5.6–28.6)	14.5 (5.5–28.7)	14.7 (5.7–28.5)	14.8 (6.0–30.1)	14.7 (6.1–29.8)
Eritrea	40 to 44	14.8 (5.8–29.2)	14.9 (5.6–29.4)	15.1 (5.9–29.2)	15.1 (6.2–30.8)	15.1 (6.2–30.4)
Eritrea	45 to 49	14.3 (5.6–28.4)	14.4 (5.4–28.5)	14.6 (5.7–28.3)	14.6 (5.9–29.9)	14.6 (6.0–29.5)
Eritrea	50 to 54	13.9 (5.4–27.7)	14.0 (5.3–27.8)	14.2 (5.5–27.6)	14.2 (5.7–29.2)	14.2 (5.8–28.8)
Eritrea	55 to 59	13.4 (5.2–26.9)	13.5 (5.0–27.0)	13.7 (5.3–26.8)	13.7 (5.5–28.2)	13.7 (5.6–27.9)
Eritrea	60 to 64	13.0 (5.0–26.1)	13.0 (4.8–26.2)	13.2 (5.1–25.9)	13.2 (5.3–27.4)	13.2 (5.3–27.0)
Eritrea	65 to 69	12.1 (4.6–24.4)	12.1 (4.5–24.5)	12.2 (4.7–24.3)	12.3 (4.9–25.7)	12.2 (4.9–25.4)
Eritrea	70 to 74	9.6 (3.6–19.9)	9.7 (3.5–20.0)	9.8 (3.6–19.8)	9.8 (3.8–21.0)	9.7 (3.8–20.7)
Eritrea	75 to 79	8.6 (3.2–18.1)	8.7 (3.1–18.2)	8.8 (3.2–18.0)	8.8 (3.4–19.1)	8.8 (3.4–18.8)
Eritrea	80 to 84	6.1 (2.2–13.1)	6.1 (2.1–13.1)	6.2 (2.2–13.0)	6.2 (2.3–13.8)	6.2 (2.3–13.6)
Eritrea	85 to 89	5.5 (2.0–11.8)	5.5 (1.9–11.8)	5.5 (2.0–11.7)	5.6 (2.1–12.5)	5.5 (2.1–12.3)
Eritrea	90 to 94	4.9 (1.8–10.7)	4.9 (1.7–10.7)	5.0 (1.8–10.6)	5.0 (1.9–11.4)	5.0 (1.9–11.2)
Eritrea	95 plus	4.9 (1.7–10.6)	4.9 (1.7–10.7)	5.0 (1.8–10.5)	5.0 (1.8–11.3)	5.0 (1.9–11.1)
Eritrea	Age-standardized	13.3 (5.2–26.6)	13.4 (5.0–26.7)	13.5 (5.2–26.5)	13.6 (5.5–28.0)	13.6 (5.5–27.6)
Eritrea	All age	13.8 (5.4–27.5)	13.8 (5.2–27.5)	14.0 (5.4–27.4)	14.1 (5.7–29.0)	14.1 (5.8–28.6)

Supplementary Table S11: Prevalence of male SVAC by age and location for 1990, 2000, 2010, 2020, and 2023						
**Location**	**Age Range**	**1990**	**2000**	**2010**	**2020**	**2023**
Ethiopia	20 to 24	8.3 (6.7–10.1)	9.6 (7.7–11.7)	9.3 (7.4–11.3)	8.5 (6.1–11.4)	8.4 (5.4–12.3)
Ethiopia	25 to 29	7.8 (6.1– 9.8)	9.0 (7.2–10.9)	9.6 (7.8–11.8)	8.8 (6.9–11.0)	8.6 (6.3–11.5)
Ethiopia	30 to 34	7.7 (5.5–10.4)	8.3 (6.7–10.0)	9.5 (7.7–11.6)	9.3 (7.2–11.7)	9.1 (6.5–12.3)
Ethiopia	35 to 39	7.7 (4.9–11.2)	7.9 (6.2– 9.7)	9.0 (7.3–11.1)	10.0 (8.0–12.3)	9.9 (7.3–13.2)
Ethiopia	40 to 44	8.0 (4.4–13.3)	7.7 (5.6–10.4)	8.3 (6.8–10.1)	9.9 (8.1–12.0)	10.2 (7.8–13.2)
Ethiopia	45 to 49	8.3 (3.9–15.3)	7.8 (5.0–11.6)	7.8 (6.2– 9.7)	8.5 (6.7–10.7)	8.9 (6.4–12.0)
Ethiopia	50 to 54	8.5 (3.6–16.6)	8.0 (4.2–13.3)	7.9 (5.7–10.6)	7.9 (6.2– 9.8)	7.9 (5.7–10.5)
Ethiopia	55 to 59	8.4 (3.3–17.1)	7.9 (3.6–14.4)	7.6 (4.9–11.2)	7.8 (6.2– 9.9)	8.0 (6.2–10.3)
Ethiopia	60 to 64	8.4 (3.2–17.2)	8.1 (3.5–15.7)	7.7 (4.5–12.3)	7.4 (5.2–10.2)	7.5 (4.7–11.4)
Ethiopia	65 to 69	7.9 (2.9–16.4)	7.9 (3.1–16.1)	7.7 (3.5–14.5)	7.5 (4.8–11.8)	7.5 (4.8–11.5)
Ethiopia	70 to 74	6.2 (2.3–13.4)	6.2 (2.2–13.4)	6.3 (2.3–13.2)	6.3 (2.4–14.1)	6.3 (2.4–14.0)
Ethiopia	75 to 79	5.6 (2.0–12.0)	5.6 (1.9–12.0)	5.6 (2.0–11.9)	5.7 (2.1–12.7)	5.7 (2.1–12.6)
Ethiopia	80 to 84	3.9 (1.4– 8.5)	3.9 (1.3– 8.5)	3.9 (1.4– 8.4)	3.9 (1.4– 9.0)	3.9 (1.5– 8.9)
Ethiopia	85 to 89	3.5 (1.2– 7.7)	3.5 (1.2– 7.7)	3.5 (1.2– 7.5)	3.5 (1.3– 8.1)	3.5 (1.3– 8.0)
Ethiopia	90 to 94	3.1 (1.1– 6.9)	3.1 (1.1– 6.9)	3.1 (1.1– 6.8)	3.2 (1.2– 7.3)	3.2 (1.2– 7.2)

Supplementary Table S11: Prevalence of male SVAC by age and location for 1990, 2000, 2010, 2020, and 2023						
**Location**	**Age Range**	**1990**	**2000**	**2010**	**2020**	**2023**
Ethiopia	95 plus	3.1 (1.1– 6.9)	3.1 (1.0– 6.9)	3.1 (1.1– 6.7)	3.1 (1.1– 7.3)	3.1 (1.2– 7.2)
Ethiopia	Age-standardized	7.8 (4.8–12.5)	8.0 (5.8–11.0)	8.3 (6.6–10.6)	8.4 (7.1–10.1)	8.4 (6.8–10.6)
Ethiopia	All age	8.0 (5.3–12.0)	8.4 (6.6–10.8)	8.8 (7.1–10.7)	8.7 (7.5–10.2)	8.7 (7.1–10.8)
Kenya	20 to 24	23.1 (13.3–37.0)	22.5 (15.0–31.3)	20.7 (14.0–28.0)	17.9 (10.5–27.5)	17.6 (9.4–28.6)
Kenya	25 to 29	21.9 (10.0–39.1)	22.1 (12.2–34.4)	22.5 (14.6–32.1)	22.1 (12.7–34.3)	22.0 (11.4–35.5)
Kenya	30 to 34	22.6 (9.4–41.9)	22.6 (11.3–37.8)	23.0 (14.1–33.9)	22.9 (14.1–33.8)	22.8 (13.4–34.8)
Kenya	35 to 39	22.5 (8.7–43.2)	22.5 (10.3–39.2)	23.0 (12.8–36.4)	23.1 (13.2–36.2)	23.0 (12.2–37.1)
Kenya	40 to 44	22.9 (8.7–44.1)	22.9 (9.9–40.8)	23.3 (11.8–38.7)	23.3 (13.8–36.1)	23.3 (13.6–35.5)
Kenya	45 to 49	22.1 (7.9–43.9)	21.9 (8.8–40.7)	22.1 (9.2–40.1)	22.2 (9.7–42.0)	22.2 (9.7–42.3)
Kenya	50 to 54	21.6 (7.6–43.2)	21.4 (8.6–40.0)	21.6 (9.0–39.4)	21.7 (9.4–41.3)	21.7 (9.5–41.1)
Kenya	55 to 59	21.0 (7.4–42.3)	20.8 (8.3–39.0)	21.0 (8.7–38.5)	21.1 (9.1–40.3)	21.1 (9.1–40.1)
Kenya	60 to 64	20.4 (7.1–41.3)	20.2 (8.0–38.1)	20.4 (8.4–37.6)	20.5 (8.8–39.4)	20.5 (8.8–39.2)
Kenya	65 to 69	19.1 (6.6–39.3)	18.9 (7.4–36.1)	19.1 (7.7–35.6)	19.2 (8.1–37.3)	19.2 (8.1–37.1)
Kenya	70 to 74	15.5 (5.1–33.2)	15.3 (5.8–30.3)	15.5 (6.1–29.8)	15.5 (6.3–31.4)	15.6 (6.4–31.2)
Kenya	75 to 79	14.0 (4.6–30.6)	13.9 (5.2–27.8)	14.0 (5.4–27.4)	14.1 (5.7–28.9)	14.1 (5.7–28.7)

Supplementary Table S11: Prevalence of male SVAC by age and location for 1990, 2000, 2010, 2020, and 2023						
**Location**	**Age Range**	**1990**	**2000**	**2010**	**2020**	**2023**
Kenya	80 to 84	10.1 (3.2–23.1)	10.0 (3.5–20.7)	10.1 (3.7–20.4)	10.1 (3.9–21.7)	10.1 (3.9–21.5)
Kenya	85 to 89	9.1 (2.8–21.3)	9.0 (3.2–18.9)	9.1 (3.4–18.6)	9.1 (3.5–19.8)	9.1 (3.5–19.6)
Kenya	90 to 94	8.3 (2.5–18.9)	8.2 (2.9–17.3)	8.2 (3.0–17.0)	8.3 (3.2–18.1)	8.3 (3.2–17.9)
Kenya	95 plus	8.2 (2.5–19.2)	8.1 (2.8–17.2)	8.2 (3.0–16.8)	8.2 (3.1–17.9)	8.2 (3.1–17.8)
Kenya	Age-standardized	21.2 (9.0–38.6)	21.0 (10.2–36.2)	21.1 (11.9–33.7)	20.7 (11.7–34.7)	20.7 (10.8–34.5)
Kenya	All age	21.9 (9.9–39.1)	21.9 (11.4–35.7)	21.8 (13.4–32.9)	21.2 (12.3–33.8)	21.1 (11.2–34.7)
Madagascar	20 to 24	13.3 (5.1–26.6)	13.4 (5.0–26.7)	13.5 (5.2–26.5)	13.6 (5.5–28.1)	13.6 (5.5–27.7)
Madagascar	25 to 29	13.4 (5.2–26.9)	13.5 (5.1–27.0)	13.7 (5.3–26.9)	13.8 (5.5–28.4)	13.8 (5.6–28.1)
Madagascar	30 to 34	14.4 (5.6–28.6)	14.5 (5.5–28.7)	14.7 (5.7–28.5)	14.8 (6.0–30.1)	14.7 (6.1–29.8)
Madagascar	35 to 39	14.4 (5.6–28.6)	14.5 (5.5–28.7)	14.7 (5.7–28.5)	14.8 (6.0–30.1)	14.7 (6.1–29.8)
Madagascar	40 to 44	14.8 (5.8–29.2)	14.9 (5.6–29.4)	15.1 (5.9–29.2)	15.1 (6.2–30.8)	15.1 (6.2–30.4)
Madagascar	45 to 49	14.3 (5.6–28.4)	14.4 (5.4–28.5)	14.6 (5.7–28.3)	14.6 (5.9–29.9)	14.6 (6.0–29.5)
Madagascar	50 to 54	13.9 (5.4–27.7)	14.0 (5.3–27.8)	14.2 (5.5–27.6)	14.2 (5.7–29.2)	14.2 (5.8–28.8)
Madagascar	55 to 59	13.4 (5.2–26.9)	13.5 (5.0–27.0)	13.7 (5.3–26.8)	13.7 (5.5–28.2)	13.7 (5.6–27.9)
Madagascar	60 to 64	13.0 (5.0–26.1)	13.0 (4.8–26.2)	13.2 (5.1–25.9)	13.2 (5.3–27.4)	13.2 (5.3–27.0)

Supplementary Table S11: Prevalence of male SVAC by age and location for 1990, 2000, 2010, 2020, and 2023						
**Location**	**Age Range**	**1990**	**2000**	**2010**	**2020**	**2023**
Madagascar	65 to 69	12.1 (4.6–24.4)	12.1 (4.5–24.5)	12.2 (4.7–24.3)	12.3 (4.9–25.7)	12.2 (4.9–25.4)
Madagascar	70 to 74	9.6 (3.6–19.9)	9.7 (3.5–20.0)	9.8 (3.6–19.8)	9.8 (3.8–21.0)	9.7 (3.8–20.7)
Madagascar	75 to 79	8.6 (3.2–18.1)	8.7 (3.1–18.2)	8.8 (3.2–18.0)	8.8 (3.4–19.1)	8.8 (3.4–18.8)
Madagascar	80 to 84	6.1 (2.2–13.1)	6.1 (2.1–13.1)	6.2 (2.2–13.0)	6.2 (2.3–13.8)	6.2 (2.3–13.6)
Madagascar	85 to 89	5.5 (2.0–11.8)	5.5 (1.9–11.8)	5.5 (2.0–11.7)	5.6 (2.1–12.5)	5.5 (2.1–12.3)
Madagascar	90 to 94	4.9 (1.8–10.7)	4.9 (1.7–10.7)	5.0 (1.8–10.6)	5.0 (1.9–11.4)	5.0 (1.9–11.2)
Madagascar	95 plus	4.9 (1.7–10.6)	4.9 (1.7–10.7)	5.0 (1.8–10.5)	5.0 (1.8–11.3)	5.0 (1.9–11.1)
Madagascar	Age-standardized	13.3 (5.2–26.6)	13.4 (5.0–26.7)	13.5 (5.2–26.5)	13.6 (5.5–28.0)	13.6 (5.5–27.6)
Madagascar	All age	13.6 (5.3–27.1)	13.7 (5.1–27.4)	13.9 (5.4–27.2)	14.0 (5.6–28.8)	14.0 (5.7–28.4)
Malawi	20 to 24	19.8 (8.4–36.4)	20.6 (10.5–33.8)	21.2 (15.1–28.7)	21.2 (12.9–31.0)	21.0 (11.8–33.4)
Malawi	25 to 29	19.7 (8.3–36.7)	20.2 (8.9–35.9)	20.8 (11.3–33.1)	21.0 (12.9–31.2)	21.0 (12.3–32.2)
Malawi	30 to 34	20.3 (8.4–38.0)	20.4 (8.1–38.1)	20.7 (8.5–38.0)	20.9 (9.0–40.0)	20.9 (9.1–39.5)
Malawi	35 to 39	20.2 (8.4–38.0)	20.3 (8.1–38.1)	20.6 (8.5–37.9)	20.8 (8.9–39.9)	20.8 (9.0–39.5)
Malawi	40 to 44	20.7 (8.6–38.6)	20.8 (8.3–38.7)	21.1 (8.7–38.6)	21.3 (9.2–40.6)	21.2 (9.3–40.2)
Malawi	45 to 49	20.0 (8.3–37.7)	20.1 (8.0–37.7)	20.4 (8.4–37.6)	20.6 (8.8–39.6)	20.6 (8.9–39.2)

Supplementary Table S11: Prevalence of male SVAC by age and location for 1990, 2000, 2010, 2020, and 2023						
**Location**	**Age Range**	**1990**	**2000**	**2010**	**2020**	**2023**
Malawi	50 to 54	19.5 (8.0–36.9)	19.6 (7.8–37.0)	19.9 (8.1–36.8)	20.1 (8.6–38.8)	20.0 (8.6–38.4)
Malawi	55 to 59	18.9 (7.7–35.9)	19.0 (7.5–35.9)	19.2 (7.8–35.8)	19.4 (8.2–37.7)	19.4 (8.3–37.3)
Malawi	60 to 64	18.3 (7.4–34.9)	18.4 (7.2–35.0)	18.6 (7.5–34.8)	18.8 (7.9–36.8)	18.7 (8.0–36.3)
Malawi	65 to 69	17.1 (6.8–33.0)	17.1 (6.6–33.1)	17.4 (6.9–32.9)	17.5 (7.3–34.8)	17.5 (7.4–34.4)
Malawi	70 to 74	13.8 (5.4–27.5)	13.8 (5.2–27.5)	14.0 (5.4–27.4)	14.2 (5.7–29.1)	14.1 (5.8–28.7)
Malawi	75 to 79	12.5 (4.8–25.1)	12.5 (4.6–25.2)	12.7 (4.8–25.1)	12.8 (5.1–26.7)	12.8 (5.2–26.4)
Malawi	80 to 84	8.9 (3.3–18.6)	8.9 (3.2–18.6)	9.1 (3.3–18.5)	9.2 (3.5–19.8)	9.1 (3.6–19.6)
Malawi	85 to 89	8.0 (3.0–16.9)	8.0 (2.8–17.0)	8.2 (3.0–16.8)	8.3 (3.2–18.1)	8.2 (3.2–17.8)
Malawi	90 to 94	7.3 (2.7–15.4)	7.3 (2.6–15.5)	7.4 (2.7–15.4)	7.5 (2.8–16.5)	7.5 (2.9–16.3)
Malawi	95 plus	7.2 (2.6–15.3)	7.2 (2.5–15.3)	7.3 (2.7–15.2)	7.4 (2.8–16.4)	7.4 (2.8–16.1)
Malawi	Age-standardized	18.9 (8.0–34.9)	19.1 (8.4–35.4)	19.5 (9.4–33.8)	19.7 (10.2–34.4)	19.6 (9.8–34.5)
Malawi	All age	19.6 (8.4–36.3)	19.9 (9.0–35.5)	20.3 (10.8–34.1)	20.5 (11.2–34.2)	20.4 (10.9–33.9)
Mozambique	20 to 24	14.6 (5.8–28.4)	14.8 (6.3–27.5)	14.8 (8.1–24.3)	14.7 (10.4–20.3)	14.7 (9.6–21.3)
Mozambique	25 to 29	14.6 (5.7–28.9)	14.7 (5.5–29.0)	14.8 (5.8–28.8)	15.0 (6.1–30.5)	15.0 (6.2–30.2)
Mozambique	30 to 34	15.6 (6.2–30.6)	15.7 (6.0–30.7)	15.9 (6.3–30.5)	16.1 (6.6–32.3)	16.0 (6.7–32.0)

Supplementary Table S11: Prevalence of male SVAC by age and location for 1990, 2000, 2010, 2020, and 2023						
**Location**	**Age Range**	**1990**	**2000**	**2010**	**2020**	**2023**
Mozambique	35 to 39	15.7 (6.2–30.7)	15.7 (6.0–30.8)	15.9 (6.3–30.5)	16.1 (6.6–32.4)	16.1 (6.7–32.0)
Mozambique	40 to 44	16.1 (6.4–31.4)	16.2 (6.2–31.5)	16.3 (6.5–31.2)	16.5 (6.8–33.1)	16.5 (6.9–32.7)
Mozambique	45 to 49	15.6 (6.2–30.6)	15.7 (6.0–30.6)	15.8 (6.2–30.4)	16.0 (6.6–32.2)	16.0 (6.6–31.8)
Mozambique	50 to 54	15.2 (6.0–29.9)	15.2 (5.8–29.9)	15.4 (6.0–29.7)	15.6 (6.4–31.5)	15.5 (6.4–31.1)
Mozambique	55 to 59	14.7 (5.8–29.0)	14.7 (5.6–29.1)	14.9 (5.8–28.8)	15.0 (6.1–30.6)	15.0 (6.2–30.2)
Mozambique	60 to 64	14.2 (5.5–28.2)	14.2 (5.3–28.2)	14.4 (5.6–28.0)	14.5 (5.9–29.7)	14.5 (5.9–29.3)
Mozambique	65 to 69	13.2 (5.1–26.4)	13.2 (4.9–26.5)	13.4 (5.1–26.3)	13.5 (5.4–27.9)	13.5 (5.5–27.6)
Mozambique	70 to 74	10.5 (4.0–21.7)	10.6 (3.8–21.7)	10.7 (4.0–21.5)	10.8 (4.2–23.0)	10.8 (4.3–22.6)
Mozambique	75 to 79	9.5 (3.5–19.7)	9.5 (3.4–19.8)	9.6 (3.6–19.6)	9.7 (3.8–20.9)	9.7 (3.8–20.6)
Mozambique	80 to 84	6.7 (2.4–14.3)	6.7 (2.3–14.3)	6.8 (2.5–14.2)	6.9 (2.6–15.2)	6.8 (2.6–15.0)
Mozambique	85 to 89	6.0 (2.2–12.9)	6.0 (2.1–13.0)	6.1 (2.2–12.8)	6.2 (2.3–13.8)	6.2 (2.3–13.6)
Mozambique	90 to 94	5.4 (2.0–11.8)	5.5 (1.9–11.8)	5.5 (2.0–11.7)	5.6 (2.1–12.5)	5.6 (2.1–12.4)
Mozambique	95 plus	5.4 (1.9–11.7)	5.4 (1.9–11.7)	5.5 (1.9–11.6)	5.5 (2.1–12.4)	5.5 (2.1–12.3)
Mozambique	Age-standardized	14.5 (5.7–28.3)	14.6 (5.8–28.6)	14.7 (6.1–27.6)	14.8 (6.8–28.5)	14.8 (6.9–28.2)
Mozambique	All age	14.9 (5.9–29.0)	15.0 (6.0–29.1)	15.2 (6.6–28.1)	15.3 (7.5–28.2)	15.2 (7.8–28.0)

Supplementary Table S11: Prevalence of male SVAC by age and location for 1990, 2000, 2010, 2020, and 2023						
**Location**	**Age Range**	**1990**	**2000**	**2010**	**2020**	**2023**
Rwanda	20 to 24	12.7 (5.6–23.1)	12.6 (6.1–21.7)	12.3 (6.9–19.7)	12.6 (6.4–21.4)	12.8 (5.9–22.8)
Rwanda	25 to 29	13.4 (5.8–24.8)	13.2 (6.1–23.7)	12.8 (6.9–21.1)	12.8 (6.9–21.4)	12.9 (6.2–22.4)
Rwanda	30 to 34	22.3 (12.0–35.7)	22.1 (12.8–32.8)	21.5 (14.0–30.4)	20.7 (11.0–33.3)	20.5 (9.6–35.1)
Rwanda	35 to 39	20.3 (10.4–33.9)	20.1 (11.1–31.1)	19.7 (12.2–28.7)	19.5 (10.2–31.9)	19.5 (8.9–33.6)
Rwanda	40 to 44	29.2 (15.9–44.6)	28.8 (17.0–41.2)	27.8 (18.3–38.5)	25.9 (14.3–40.4)	25.5 (12.4–41.8)
Rwanda	45 to 49	26.0 (14.1–40.9)	25.7 (15.0–38.1)	25.0 (16.1–35.2)	23.8 (12.9–37.7)	23.5 (11.2–39.3)
Rwanda	50 to 54	23.4 (12.5–37.2)	23.1 (13.5–34.5)	22.6 (14.9–31.6)	21.9 (11.9–34.9)	21.8 (10.3–37.4)
Rwanda	55 to 59	29.9 (16.8–45.1)	29.5 (17.7–42.1)	28.4 (18.8–39.1)	26.2 (14.5–40.8)	25.8 (12.4–42.0)
Rwanda	60 to 64	19.6 (6.6–40.8)	19.4 (7.2–39.0)	19.0 (7.7–35.5)	18.8 (7.9–36.8)	18.9 (7.9–37.8)
Rwanda	65 to 69	18.3 (6.1–38.7)	18.1 (6.6–36.5)	17.8 (7.1–33.5)	17.6 (7.3–34.8)	17.6 (7.2–35.9)
Rwanda	70 to 74	14.8 (4.8–32.1)	14.7 (5.2–30.4)	14.4 (5.6–28.0)	14.2 (5.7–29.1)	14.2 (5.7–29.6)
Rwanda	75 to 79	13.5 (4.3–28.7)	13.3 (4.6–27.9)	13.0 (5.0–25.6)	12.8 (5.1–26.7)	12.9 (5.0–27.1)
Rwanda	80 to 84	9.7 (2.9–22.3)	9.5 (3.2–20.6)	9.3 (3.4–18.9)	9.2 (3.5–19.9)	9.2 (3.5–20.2)
Rwanda	85 to 89	8.7 (2.6–20.3)	8.6 (2.9–19.0)	8.4 (3.1–17.2)	8.3 (3.2–18.1)	8.3 (3.2–18.4)
Rwanda	90 to 94	7.9 (2.4–19.0)	7.8 (2.6–17.5)	7.6 (2.8–15.7)	7.5 (2.8–16.5)	7.5 (2.8–16.8)

Supplementary Table S11: Prevalence of male SVAC by age and location for 1990, 2000, 2010, 2020, and 2023						
**Location**	**Age Range**	**1990**	**2000**	**2010**	**2020**	**2023**
Rwanda	95 plus	7.9 (2.3–18.4)	7.8 (2.6–17.3)	7.5 (2.7–15.6)	7.4 (2.8–16.4)	7.4 (2.8–16.6)
Rwanda	Age-standardized	20.2 (10.4–33.5)	19.9 (11.0–31.4)	19.4 (11.7–29.0)	18.8 (10.1–30.6)	18.7 (8.8–31.8)
Rwanda	All age	19.1 (9.8–31.7)	19.2 (10.6–30.0)	18.3 (11.0–27.4)	18.3 (9.9–30.2)	18.5 (8.7–31.2)
Somalia	20 to 24	13.3 (5.1–26.6)	13.4 (5.0–26.7)	13.5 (5.2–26.5)	13.6 (5.5–28.1)	13.6 (5.5–27.7)
Somalia	25 to 29	13.4 (5.2–26.9)	13.5 (5.1–27.0)	13.7 (5.3–26.9)	13.8 (5.5–28.4)	13.8 (5.6–28.1)
Somalia	30 to 34	14.4 (5.6–28.6)	14.5 (5.5–28.7)	14.7 (5.7–28.5)	14.8 (6.0–30.1)	14.7 (6.1–29.8)
Somalia	35 to 39	14.4 (5.6–28.6)	14.5 (5.5–28.7)	14.7 (5.7–28.5)	14.8 (6.0–30.1)	14.7 (6.1–29.8)
Somalia	40 to 44	14.8 (5.8–29.2)	14.9 (5.6–29.4)	15.1 (5.9–29.2)	15.1 (6.2–30.8)	15.1 (6.2–30.4)
Somalia	45 to 49	14.3 (5.6–28.4)	14.4 (5.4–28.5)	14.6 (5.7–28.3)	14.6 (5.9–29.9)	14.6 (6.0–29.5)
Somalia	50 to 54	13.9 (5.4–27.7)	14.0 (5.3–27.8)	14.2 (5.5–27.6)	14.2 (5.7–29.2)	14.2 (5.8–28.8)
Somalia	55 to 59	13.4 (5.2–26.9)	13.5 (5.0–27.0)	13.7 (5.3–26.8)	13.7 (5.5–28.2)	13.7 (5.6–27.9)
Somalia	60 to 64	13.0 (5.0–26.1)	13.0 (4.8–26.2)	13.2 (5.1–25.9)	13.2 (5.3–27.4)	13.2 (5.3–27.0)
Somalia	65 to 69	12.1 (4.6–24.4)	12.1 (4.5–24.5)	12.2 (4.7–24.3)	12.3 (4.9–25.7)	12.2 (4.9–25.4)
Somalia	70 to 74	9.6 (3.6–19.9)	9.7 (3.5–20.0)	9.8 (3.6–19.8)	9.8 (3.8–21.0)	9.7 (3.8–20.7)
Somalia	75 to 79	8.6 (3.2–18.1)	8.7 (3.1–18.2)	8.8 (3.2–18.0)	8.8 (3.4–19.1)	8.8 (3.4–18.8)

Supplementary Table S11: Prevalence of male SVAC by age and location for 1990, 2000, 2010, 2020, and 2023						
**Location**	**Age Range**	**1990**	**2000**	**2010**	**2020**	**2023**
Somalia	80 to 84	6.1 (2.2–13.1)	6.1 (2.1–13.1)	6.2 (2.2–13.0)	6.2 (2.3–13.8)	6.2 (2.3–13.6)
Somalia	85 to 89	5.5 (2.0–11.8)	5.5 (1.9–11.8)	5.5 (2.0–11.7)	5.6 (2.1–12.5)	5.5 (2.1–12.3)
Somalia	90 to 94	4.9 (1.8–10.7)	4.9 (1.7–10.7)	5.0 (1.8–10.6)	5.0 (1.9–11.4)	5.0 (1.9–11.2)
Somalia	95 plus	4.9 (1.7–10.6)	4.9 (1.7–10.7)	5.0 (1.8–10.5)	5.0 (1.8–11.3)	5.0 (1.9–11.1)
Somalia	Age-standardized	13.3 (5.2–26.6)	13.4 (5.0–26.7)	13.5 (5.2–26.5)	13.6 (5.5–28.0)	13.6 (5.5–27.6)
Somalia	All age	13.8 (5.4–27.5)	13.8 (5.2–27.5)	14.0 (5.4–27.3)	14.1 (5.7–28.9)	14.1 (5.7–28.6)
South Sudan	20 to 24	13.3 (5.1–26.6)	13.4 (5.0–26.7)	13.5 (5.2–26.5)	13.6 (5.5–28.1)	13.6 (5.5–27.7)
South Sudan	25 to 29	13.4 (5.2–26.9)	13.5 (5.1–27.0)	13.7 (5.3–26.9)	13.8 (5.5–28.4)	13.8 (5.6–28.1)
South Sudan	30 to 34	14.4 (5.6–28.6)	14.5 (5.5–28.7)	14.7 (5.7–28.5)	14.8 (6.0–30.1)	14.7 (6.1–29.8)
South Sudan	35 to 39	14.4 (5.6–28.6)	14.5 (5.5–28.7)	14.7 (5.7–28.5)	14.8 (6.0–30.1)	14.7 (6.1–29.8)
South Sudan	40 to 44	14.8 (5.8–29.2)	14.9 (5.6–29.4)	15.1 (5.9–29.2)	15.1 (6.2–30.8)	15.1 (6.2–30.4)
South Sudan	45 to 49	14.3 (5.6–28.4)	14.4 (5.4–28.5)	14.6 (5.7–28.3)	14.6 (5.9–29.9)	14.6 (6.0–29.5)
South Sudan	50 to 54	13.9 (5.4–27.7)	14.0 (5.3–27.8)	14.2 (5.5–27.6)	14.2 (5.7–29.2)	14.2 (5.8–28.8)
South Sudan	55 to 59	13.4 (5.2–26.9)	13.5 (5.0–27.0)	13.7 (5.3–26.8)	13.7 (5.5–28.2)	13.7 (5.6–27.9)
South Sudan	60 to 64	13.0 (5.0–26.1)	13.0 (4.8–26.2)	13.2 (5.1–25.9)	13.2 (5.3–27.4)	13.2 (5.3–27.0)

Supplementary Table S11: Prevalence of male SVAC by age and location for 1990, 2000, 2010, 2020, and 2023						
**Location**	**Age Range**	**1990**	**2000**	**2010**	**2020**	**2023**
South Sudan	65 to 69	12.1 (4.6–24.4)	12.1 (4.5–24.5)	12.2 (4.7–24.3)	12.3 (4.9–25.7)	12.2 (4.9–25.4)
South Sudan	70 to 74	9.6 (3.6–19.9)	9.7 (3.5–20.0)	9.8 (3.6–19.8)	9.8 (3.8–21.0)	9.7 (3.8–20.7)
South Sudan	75 to 79	8.6 (3.2–18.1)	8.7 (3.1–18.2)	8.8 (3.2–18.0)	8.8 (3.4–19.1)	8.8 (3.4–18.8)
South Sudan	80 to 84	6.1 (2.2–13.1)	6.1 (2.1–13.1)	6.2 (2.2–13.0)	6.2 (2.3–13.8)	6.2 (2.3–13.6)
South Sudan	85 to 89	5.5 (2.0–11.8)	5.5 (1.9–11.8)	5.5 (2.0–11.7)	5.6 (2.1–12.5)	5.5 (2.1–12.3)
South Sudan	90 to 94	4.9 (1.8–10.7)	4.9 (1.7–10.7)	5.0 (1.8–10.6)	5.0 (1.9–11.4)	5.0 (1.9–11.2)
South Sudan	95 plus	4.9 (1.7–10.6)	4.9 (1.7–10.7)	5.0 (1.8–10.5)	5.0 (1.8–11.3)	5.0 (1.9–11.1)
South Sudan	Age-standardized	13.3 (5.2–26.6)	13.4 (5.0–26.7)	13.5 (5.2–26.5)	13.6 (5.5–28.0)	13.6 (5.5–27.6)
South Sudan	All age	13.5 (5.2–27.0)	13.7 (5.1–27.2)	13.9 (5.4–27.2)	14.0 (5.6–28.7)	13.9 (5.7–28.3)
Uganda	20 to 24	19.9 (11.5–31.4)	19.2 (13.1–27.0)	20.7 (14.5–27.7)	22.3 (14.7–31.7)	22.5 (13.5–34.1)
Uganda	25 to 29	20.8 (11.1–34.5)	20.7 (14.0–28.9)	21.0 (14.2–29.0)	22.0 (15.7–29.8)	22.2 (15.4–31.1)
Uganda	30 to 34	22.4 (11.0–37.5)	21.9 (14.0–31.2)	20.3 (13.3–28.7)	19.7 (10.2–32.6)	19.7 (9.2–34.4)
Uganda	35 to 39	19.9 (9.3–34.9)	19.3 (11.5–29.0)	19.3 (12.7–27.0)	19.3 (11.2–29.7)	19.3 (10.0–31.1)
Uganda	40 to 44	19.0 (8.3–34.0)	18.2 (10.0–28.3)	19.5 (12.1–28.7)	21.3 (12.3–32.8)	21.7 (11.4–35.0)
Uganda	45 to 49	20.0 (9.1–36.0)	19.6 (10.2–32.0)	20.4 (11.3–32.4)	21.6 (12.0–34.8)	21.8 (11.0–36.6)

Supplementary Table S11: Prevalence of male SVAC by age and location for 1990, 2000, 2010, 2020, and 2023						
**Location**	**Age Range**	**1990**	**2000**	**2010**	**2020**	**2023**
Uganda	50 to 54	20.6 (8.6–38.6)	20.5 (8.2–38.3)	20.5 (8.5–37.8)	20.7 (8.9–39.7)	20.7 (8.9–40.4)
Uganda	55 to 59	20.0 (8.3–37.6)	19.9 (7.9–37.4)	19.9 (8.2–36.9)	20.1 (8.6–38.8)	20.1 (8.6–39.4)
Uganda	60 to 64	19.4 (7.8–36.7)	19.3 (7.6–36.5)	19.3 (7.9–36.0)	19.5 (8.3–37.9)	19.5 (8.3–38.4)
Uganda	65 to 69	18.1 (7.4–34.8)	18.0 (7.0–34.5)	18.1 (7.3–34.0)	18.2 (7.6–35.9)	18.3 (7.6–36.4)
Uganda	70 to 74	14.7 (5.8–29.1)	14.6 (5.5–28.8)	14.6 (5.7–28.4)	14.7 (6.0–30.1)	14.8 (6.0–30.6)
Uganda	75 to 79	13.3 (5.2–26.7)	13.2 (4.9–26.5)	13.2 (5.1–26.0)	13.3 (5.3–27.6)	13.4 (5.4–28.1)
Uganda	80 to 84	9.5 (3.6–19.8)	9.4 (3.4–19.6)	9.5 (3.5–19.3)	9.5 (3.7–20.6)	9.6 (3.7–21.1)
Uganda	85 to 89	8.6 (3.2–18.1)	8.5 (3.0–17.9)	8.5 (3.1–17.6)	8.6 (3.3–18.8)	8.7 (3.3–19.2)
Uganda	90 to 94	7.8 (2.9–16.5)	7.7 (2.7–16.3)	7.7 (2.8–16.0)	7.8 (3.0–17.2)	7.8 (3.0–17.5)
Uganda	95 plus	7.7 (2.8–16.2)	7.7 (2.7–16.2)	7.7 (2.8–15.9)	7.7 (2.9–17.0)	7.8 (3.0–17.4)
Uganda	Age-standardized	19.5 (9.4–33.4)	19.1 (10.6–30.3)	19.3 (12.0–29.0)	19.9 (11.7–31.9)	20.0 (10.7–33.2)
Uganda	All age	20.0 (10.1–33.1)	19.7 (12.0–29.2)	20.1 (13.4–28.1)	20.8 (12.9–31.6)	20.9 (12.2–32.6)
Tanzania	20 to 24	20.7 (9.2–36.5)	20.3 (11.3–31.6)	20.0 (14.5–26.3)	20.2 (11.2–33.6)	20.3 (9.9–35.7)
Tanzania	25 to 29	21.0 (8.9–38.3)	20.8 (9.8–35.7)	20.5 (12.2–31.0)	20.6 (11.6–32.2)	20.7 (10.8–34.0)
Tanzania	30 to 34	22.1 (9.6–40.6)	21.8 (9.7–38.5)	21.4 (11.3–34.7)	20.9 (12.8–31.1)	20.9 (12.5–31.8)

Supplementary Table S11: Prevalence of male SVAC by age and location for 1990, 2000, 2010, 2020, and 2023						
**Location**	**Age Range**	**1990**	**2000**	**2010**	**2020**	**2023**
Tanzania	35 to 39	22.4 (9.5–41.1)	22.4 (9.1–41.2)	22.6 (9.5–40.8)	22.6 (9.9–42.5)	22.5 (9.9–42.1)
Tanzania	40 to 44	22.9 (9.7–41.9)	23.0 (9.4–42.0)	23.2 (9.8–41.6)	23.1 (10.1–43.3)	23.1 (10.3–42.9)
Tanzania	45 to 49	22.3 (9.4–41.0)	22.3 (9.1–41.0)	22.5 (9.5–40.7)	22.5 (9.8–42.4)	22.5 (9.9–42.0)
Tanzania	50 to 54	21.8 (9.2–40.3)	21.8 (8.8–40.3)	22.0 (9.2–39.9)	22.0 (9.5–41.6)	21.9 (9.6–41.2)
Tanzania	55 to 59	21.1 (8.8–39.3)	21.2 (8.5–39.3)	21.3 (8.8–38.9)	21.3 (9.2–40.6)	21.2 (9.3–40.2)
Tanzania	60 to 64	20.5 (8.5–38.3)	20.5 (8.2–38.3)	20.7 (8.5–38.0)	20.6 (8.8–39.6)	20.6 (8.9–39.2)
Tanzania	65 to 69	19.2 (7.8–36.3)	19.2 (7.6–36.3)	19.3 (7.9–36.0)	19.3 (8.2–37.6)	19.3 (8.3–37.2)
Tanzania	70 to 74	15.5 (6.2–30.5)	15.6 (5.9–30.5)	15.7 (6.2–30.2)	15.7 (6.4–31.7)	15.6 (6.5–31.3)
Tanzania	75 to 79	14.1 (5.5–28.0)	14.1 (5.3–28.0)	14.2 (5.5–27.7)	14.2 (5.7–29.1)	14.2 (5.8–28.8)
Tanzania	80 to 84	10.1 (3.8–20.9)	10.1 (3.7–20.9)	10.2 (3.8–20.7)	10.2 (4.0–21.8)	10.2 (4.0–21.6)
Tanzania	85 to 89	9.1 (3.4–19.1)	9.2 (3.3–19.1)	9.2 (3.4–18.9)	9.2 (3.5–19.9)	9.2 (3.6–19.7)
Tanzania	90 to 94	8.3 (3.1–17.4)	8.3 (2.9–17.4)	8.4 (3.1–17.2)	8.3 (3.2–18.2)	8.3 (3.2–18.0)
Tanzania	95 plus	8.2 (3.0–17.3)	8.2 (2.9–17.3)	8.3 (3.0–17.1)	8.3 (3.2–18.1)	8.3 (3.2–17.8)
Tanzania	Age-standardized	20.8 (9.0–37.5)	20.7 (9.2–37.4)	20.7 (10.9–34.0)	20.6 (12.4–33.7)	20.6 (11.9–33.8)
Tanzania	All age	21.2 (9.4–38.4)	21.1 (9.7–37.2)	21.0 (12.3–32.8)	21.0 (13.9–32.2)	21.0 (13.6–32.4)

Supplementary Table S11: Prevalence of male SVAC by age and location for 1990, 2000, 2010, 2020, and 2023						
**Location**	**Age Range**	**1990**	**2000**	**2010**	**2020**	**2023**
Zambia	20 to 24	16.2 (6.3–31.7)	16.3 (7.8–28.2)	16.4 (11.1–23.3)	16.4 (10.2–24.1)	16.5 (9.3–25.7)
Zambia	25 to 29	16.3 (6.3–33.0)	16.3 (7.1–29.7)	16.5 (8.8–27.6)	16.4 (10.9–23.5)	16.5 (10.5–24.8)
Zambia	30 to 34	17.2 (6.4–34.1)	17.1 (6.6–33.1)	17.3 (6.9–32.8)	17.5 (7.2–34.8)	17.6 (7.2–35.4)
Zambia	35 to 39	17.3 (6.4–34.3)	17.2 (6.7–33.2)	17.4 (6.9–32.9)	17.6 (7.3–34.9)	17.6 (7.2–35.5)
Zambia	40 to 44	17.8 (6.6–35.1)	17.7 (6.9–34.0)	17.9 (7.2–33.7)	18.1 (7.5–35.7)	18.1 (7.5–36.3)
Zambia	45 to 49	17.3 (6.4–34.3)	17.2 (6.7–33.2)	17.4 (6.9–32.9)	17.5 (7.3–34.9)	17.6 (7.2–35.5)
Zambia	50 to 54	16.9 (6.2–33.6)	16.8 (6.5–32.6)	17.0 (6.7–32.2)	17.1 (7.1–34.1)	17.2 (7.0–34.8)
Zambia	55 to 59	16.4 (6.0–32.7)	16.3 (6.2–31.7)	16.4 (6.5–31.3)	16.6 (6.8–33.3)	16.6 (6.7–33.8)
Zambia	60 to 64	15.8 (5.8–31.9)	15.8 (6.0–30.8)	15.9 (6.3–30.5)	16.0 (6.5–32.3)	16.1 (6.5–33.0)
Zambia	65 to 69	14.8 (5.3–30.0)	14.7 (5.5–29.0)	14.8 (5.8–28.7)	14.9 (6.0–30.6)	15.0 (6.0–30.8)
Zambia	70 to 74	11.9 (4.2–24.9)	11.8 (4.3–23.9)	11.9 (4.5–23.7)	12.0 (4.7–25.2)	12.1 (4.7–25.8)
Zambia	75 to 79	10.7 (3.7–22.7)	10.6 (3.9–21.8)	10.7 (4.0–21.6)	10.8 (4.2–23.0)	10.9 (4.2–23.5)
Zambia	80 to 84	7.6 (2.5–16.6)	7.5 (2.7–15.9)	7.6 (2.8–15.7)	7.7 (2.9–16.9)	7.7 (2.9–17.3)
Zambia	85 to 89	6.8 (2.3–15.1)	6.8 (2.4–14.5)	6.8 (2.5–14.3)	6.9 (2.6–15.3)	6.9 (2.6–15.7)
Zambia	90 to 94	6.2 (2.0–13.8)	6.1 (2.1–13.1)	6.2 (2.2–13.0)	6.2 (2.3–14.0)	6.3 (2.3–14.2)

Supplementary Table S11: Prevalence of male SVAC by age and location for 1990, 2000, 2010, 2020, and 2023						
**Location**	**Age Range**	**1990**	**2000**	**2010**	**2020**	**2023**
Zambia	95 plus	6.1 (2.0–13.7)	6.1 (2.1–13.0)	6.1 (2.2–12.9)	6.2 (2.3–13.9)	6.2 (2.3–14.2)
Zambia	Age-standardized	16.1 (6.3–31.2)	16.1 (6.8–30.7)	16.2 (7.5–29.1)	16.3 (8.2–29.6)	16.4 (8.0–30.1)
Zambia	All age	16.5 (6.6–31.4)	16.5 (7.2–30.5)	16.7 (8.2–29.1)	16.8 (9.2–28.7)	16.9 (8.9–29.7)
Southern sub-Saharan Africa	20 to 24	16.5 (6.9–30.6)	16.2 (6.8–30.0)	16.6 (7.1–30.2)	16.8 (7.3–32.6)	16.8 (7.4–32.7)
Southern sub-Saharan Africa	25 to 29	17.2 (7.1–32.1)	16.8 (7.0–31.6)	17.0 (7.1–31.6)	17.4 (7.3–34.6)	17.5 (7.3–34.7)
Southern sub-Saharan Africa	30 to 34	18.9 (8.0–34.7)	18.6 (7.9–34.5)	18.6 (7.9–33.8)	19.2 (8.0–37.7)	19.2 (8.0–37.5)
Southern sub-Saharan Africa	35 to 39	19.0 (7.8–35.2)	19.2 (7.9–35.8)	19.0 (8.0–34.8)	19.4 (8.0–38.6)	19.6 (8.0–38.7)
Southern sub-Saharan Africa	40 to 44	20.0 (8.4–36.8)	20.2 (8.6–37.3)	20.2 (8.7–36.7)	20.1 (8.5–38.9)	20.3 (8.3–39.6)
Southern sub-Saharan Africa	45 to 49	19.4 (8.1–35.7)	19.6 (8.2–36.3)	19.9 (8.5–36.2)	19.8 (8.2–39.2)	19.7 (8.1–39.1)
Southern sub-Saharan Africa	50 to 54	18.9 (7.9–35.0)	19.2 (7.9–35.8)	19.6 (8.3–35.8)	19.7 (8.1–39.1)	19.6 (8.0–38.8)
Southern sub-Saharan Africa	55 to 59	18.6 (7.8–34.5)	18.8 (7.9–35.1)	19.2 (8.1–35.2)	19.5 (8.0–38.7)	19.5 (8.0–38.8)
Southern sub-Saharan Africa	60 to 64	18.1 (7.5–33.7)	18.2 (7.7–34.1)	18.6 (7.9–34.2)	18.9 (7.6–37.9)	19.0 (7.6–37.8)
Southern sub-Saharan Africa	65 to 69	17.0 (7.1–31.7)	17.1 (7.2–32.2)	17.4 (7.3–32.2)	17.8 (7.2–35.9)	17.9 (7.2–36.1)
Southern sub-Saharan Africa	70 to 74	13.6 (5.3–26.6)	13.7 (5.4–26.9)	14.0 (5.6–27.0)	14.3 (5.6–30.3)	14.4 (5.6–30.4)
Southern sub-Saharan Africa	75 to 79	12.4 (4.7–24.7)	12.3 (4.6–24.8)	12.6 (4.9–24.7)	12.9 (4.8–27.9)	13.0 (4.8–27.8)

Supplementary Table S11: Prevalence of male SVAC by age and location for 1990, 2000, 2010, 2020, and 2023						
**Location**	**Age Range**	**1990**	**2000**	**2010**	**2020**	**2023**
Southern sub-Saharan Africa	80 to 84	9.3 (3.3–19.5)	9.0 (3.3–18.9)	9.2 (3.4–18.7)	9.4 (3.4–20.8)	9.5 (3.3–21.3)
Southern sub-Saharan Africa	85 to 89	8.6 (3.0–18.4)	8.3 (3.0–17.5)	8.4 (3.1–17.3)	8.7 (3.1–20.1)	8.8 (3.1–20.4)
Southern sub-Saharan Africa	90 to 94	7.9 (2.7–16.9)	7.8 (2.8–16.4)	7.7 (2.8–15.9)	7.9 (2.7–18.0)	8.0 (2.7–18.5)
Southern sub-Saharan Africa	95 plus	7.9 (2.7–17.0)	7.8 (2.8–16.4)	7.7 (2.8–15.9)	7.9 (2.7–18.0)	8.0 (2.7–18.6)
Southern sub-Saharan Africa	Age-standardized	17.7 (7.4–33.0)	17.7 (7.5–33.1)	17.9 (7.5–32.9)	18.2 (7.5–36.1)	18.2 (7.5–36.1)
Southern sub-Saharan Africa	All age	17.9 (7.5–33.2)	17.9 (7.6–33.4)	18.1 (7.6–33.1)	18.5 (7.7–36.5)	18.6 (7.7–36.6)
Botswana	20 to 24	17.3 (8.2–30.0)	17.1 (8.7–28.4)	16.6 (9.9–25.3)	16.7 (9.0–26.9)	17.0 (8.5–29.1)
Botswana	25 to 29	18.8 (8.4–33.2)	18.4 (8.9–32.0)	17.5 (9.8–27.9)	16.7 (9.8–26.1)	16.8 (9.1–27.1)
Botswana	30 to 34	31.5 (17.9–47.5)	31.2 (18.3–45.0)	30.2 (19.6–41.9)	29.1 (16.8–44.2)	28.9 (14.7–45.3)
Botswana	35 to 39	28.8 (15.6–44.8)	28.6 (16.2–42.9)	27.9 (17.5–39.8)	27.5 (15.5–43.0)	27.5 (13.5–43.6)
Botswana	40 to 44	40.0 (23.2–57.4)	39.6 (23.7–55.5)	38.3 (25.1–52.1)	36.0 (21.2–53.6)	35.5 (18.8–54.5)
Botswana	45 to 49	36.2 (20.8–53.8)	35.8 (21.5–52.1)	34.8 (22.5–48.2)	33.3 (19.4–50.3)	33.0 (17.3–50.6)
Botswana	50 to 54	32.9 (19.0–49.5)	32.6 (19.8–46.8)	31.8 (20.9–43.7)	31.0 (18.1–46.4)	30.9 (15.9–48.2)
Botswana	55 to 59	40.6 (24.3–57.7)	40.2 (24.9–56.2)	39.0 (26.0–52.8)	36.6 (21.8–54.1)	36.1 (19.4–54.8)
Botswana	60 to 64	37.7 (23.3–54.5)	37.3 (23.4–52.2)	36.2 (24.7–48.0)	34.3 (20.6–49.9)	33.9 (18.2–51.7)

Supplementary Table S11: Prevalence of male SVAC by age and location for 1990, 2000, 2010, 2020, and 2023						
**Location**	**Age Range**	**1990**	**2000**	**2010**	**2020**	**2023**
Botswana	65 to 69	40.5 (26.0–56.6)	40.0 (26.6–54.4)	38.7 (27.2–50.7)	35.9 (21.9–51.7)	35.3 (19.6–52.9)
Botswana	70 to 74	26.1 (13.4–41.7)	25.9 (14.0–39.7)	25.3 (15.4–37.0)	24.5 (13.3–39.5)	24.3 (11.6–40.1)
Botswana	75 to 79	17.2 (7.4–32.6)	17.1 (7.8–30.6)	16.9 (8.4–28.8)	17.4 (8.5–31.1)	17.6 (7.9–32.6)
Botswana	80 to 84	13.2 (5.1–26.4)	13.1 (5.4–25.1)	12.9 (5.8–23.6)	13.1 (5.8–25.3)	13.2 (5.6–26.1)
Botswana	85 to 89	12.3 (4.8–24.6)	12.2 (5.1–23.6)	12.0 (5.3–22.2)	12.1 (5.3–23.7)	12.2 (5.1–24.4)
Botswana	90 to 94	11.5 (4.4–23.5)	11.4 (4.6–22.5)	11.2 (5.0–20.9)	11.3 (4.9–22.2)	11.3 (4.7–22.8)
Botswana	95 plus	11.5 (4.3–23.2)	11.3 (4.6–22.1)	11.1 (4.9–20.8)	11.2 (4.9–22.0)	11.3 (4.7–22.6)
Botswana	Age-standardized	29.5 (16.6–44.4)	29.2 (17.1–42.9)	28.3 (18.1–40.0)	27.2 (15.7–41.8)	27.1 (14.0–42.2)
Botswana	All age	27.9 (15.4–42.4)	27.3 (15.7–41.0)	26.6 (16.9–37.8)	26.9 (15.5–41.0)	27.0 (14.1–42.1)
Eswatini	20 to 24	7.0 (2.6–14.8)	6.9 (2.8–13.9)	6.9 (3.3–12.6)	7.0 (3.9–11.5)	7.1 (3.8–12.0)
Eswatini	25 to 29	7.1 (2.5–14.9)	7.0 (2.6–14.2)	6.9 (3.1–13.2)	7.1 (3.3–13.7)	7.2 (3.1–14.3)
Eswatini	30 to 34	10.4 (4.6–18.4)	10.3 (5.0–17.8)	10.2 (5.9–16.0)	10.1 (5.4–16.8)	10.2 (4.9–17.8)
Eswatini	35 to 39	9.7 (4.1–17.7)	9.6 (4.4–17.2)	9.4 (5.2–15.3)	9.5 (5.0–16.2)	9.6 (4.5–17.5)
Eswatini	40 to 44	13.7 (6.4–22.9)	13.5 (7.1–22.1)	13.3 (8.3–19.9)	13.1 (7.3–20.9)	12.9 (6.6–22.0)
Eswatini	45 to 49	12.2 (5.6–21.0)	12.1 (6.1–20.2)	11.9 (7.1–18.3)	11.8 (6.5–19.1)	11.8 (5.8–20.4)

Supplementary Table S11: Prevalence of male SVAC by age and location for 1990, 2000, 2010, 2020, and 2023						
**Location**	**Age Range**	**1990**	**2000**	**2010**	**2020**	**2023**
Eswatini	50 to 54	10.9 (4.9–19.3)	10.9 (5.3–18.6)	10.7 (6.2–16.7)	10.7 (5.8–17.6)	10.8 (5.3–18.8)
Eswatini	55 to 59	14.0 (6.7–23.3)	13.8 (7.3–22.4)	13.6 (8.6–20.1)	13.3 (7.6–21.2)	13.1 (6.7–22.3)
Eswatini	60 to 64	12.5 (5.9–21.2)	12.4 (6.5–20.4)	12.2 (7.6–18.3)	12.1 (6.8–19.4)	12.0 (6.1–20.5)
Eswatini	65 to 69	13.6 (6.6–22.5)	13.4 (7.2–21.8)	13.1 (8.5–19.2)	12.8 (7.4–20.2)	12.6 (6.5–21.5)
Eswatini	70 to 74	8.2 (2.4–19.7)	8.1 (2.5–18.5)	7.9 (2.7–17.2)	7.9 (3.0–17.3)	7.9 (3.0–17.4)
Eswatini	75 to 79	7.4 (2.1–17.8)	7.3 (2.2–16.8)	7.1 (2.4–15.6)	7.1 (2.7–15.7)	7.1 (2.7–15.7)
Eswatini	80 to 84	5.2 (1.5–12.9)	5.1 (1.5–12.1)	5.0 (1.7–11.1)	5.0 (1.8–11.3)	5.0 (1.9–11.3)
Eswatini	85 to 89	4.6 (1.3–11.6)	4.6 (1.3–10.9)	4.5 (1.5–10.1)	4.5 (1.6–10.2)	4.5 (1.7–10.2)
Eswatini	90 to 94	4.2 (1.2–10.6)	4.1 (1.2– 9.9)	4.0 (1.3– 9.1)	4.0 (1.5– 9.2)	4.0 (1.5– 9.2)
Eswatini	95 plus	4.1 (1.2–10.5)	4.1 (1.2– 9.8)	4.0 (1.3– 9.0)	4.0 (1.5– 9.1)	4.0 (1.5– 9.1)
Eswatini	Age-standardized	10.2 (4.5–18.7)	10.1 (4.8–18.0)	9.9 (5.7–16.1)	9.9 (5.4–16.5)	9.9 (4.9–17.2)
Eswatini	All age	9.7 (4.3–18.2)	9.6 (4.5–17.4)	9.4 (5.3–15.5)	9.6 (5.2–16.1)	9.7 (4.8–16.9)
Lesotho	20 to 24	11.2 (4.4–22.7)	11.0 (4.4–21.1)	10.6 (5.3–18.3)	10.4 (5.8–16.9)	10.5 (5.7–17.4)
Lesotho	25 to 29	11.7 (4.4–23.7)	11.6 (4.3–23.7)	11.7 (4.4–23.3)	11.7 (4.6–24.6)	11.7 (4.7–24.3)
Lesotho	30 to 34	12.7 (4.9–25.6)	12.7 (4.7–25.6)	12.7 (4.9–25.2)	12.7 (5.1–26.6)	12.7 (5.1–26.3)

Supplementary Table S11: Prevalence of male SVAC by age and location for 1990, 2000, 2010, 2020, and 2023						
**Location**	**Age Range**	**1990**	**2000**	**2010**	**2020**	**2023**
Lesotho	35 to 39	13.0 (5.0–26.0)	12.9 (4.8–26.0)	13.0 (5.0–25.6)	13.0 (5.2–27.0)	13.0 (5.2–26.7)
Lesotho	40 to 44	13.5 (5.2–27.0)	13.5 (5.0–27.0)	13.5 (5.2–26.6)	13.5 (5.4–28.0)	13.5 (5.5–27.6)
Lesotho	45 to 49	13.3 (5.1–26.6)	13.3 (4.9–26.6)	13.3 (5.1–26.2)	13.3 (5.3–27.5)	13.3 (5.4–27.2)
Lesotho	50 to 54	13.1 (5.1–26.3)	13.1 (4.9–26.2)	13.1 (5.0–25.8)	13.1 (5.2–27.2)	13.1 (5.3–26.9)
Lesotho	55 to 59	12.8 (4.9–25.8)	12.8 (4.7–25.7)	12.8 (4.9–25.3)	12.8 (5.1–26.6)	12.8 (5.2–26.3)
Lesotho	60 to 64	12.5 (4.8–25.2)	12.5 (4.6–25.2)	12.5 (4.8–24.8)	12.5 (4.9–26.0)	12.4 (5.0–25.7)
Lesotho	65 to 69	11.7 (4.5–23.8)	11.7 (4.3–23.7)	11.7 (4.4–23.3)	11.6 (4.6–24.6)	11.6 (4.7–24.2)
Lesotho	70 to 74	9.4 (3.5–19.5)	9.3 (3.3–19.4)	9.4 (3.5–19.1)	9.3 (3.6–20.1)	9.3 (3.6–19.9)
Lesotho	75 to 79	8.5 (3.1–17.7)	8.4 (3.0–17.7)	8.4 (3.1–17.4)	8.4 (3.2–18.3)	8.4 (3.3–18.1)
Lesotho	80 to 84	6.0 (2.2–12.8)	6.0 (2.1–12.8)	6.0 (2.1–12.6)	5.9 (2.2–13.3)	5.9 (2.2–13.1)
Lesotho	85 to 89	5.4 (1.9–11.7)	5.4 (1.9–11.6)	5.4 (1.9–11.4)	5.3 (2.0–12.1)	5.3 (2.0–11.9)
Lesotho	90 to 94	4.9 (1.7–10.6)	4.9 (1.7–10.6)	4.9 (1.7–10.4)	4.8 (1.8–11.0)	4.8 (1.8–10.8)
Lesotho	95 plus	4.8 (1.7–10.5)	4.8 (1.7–10.5)	4.8 (1.7–10.3)	4.8 (1.8–10.9)	4.8 (1.8–10.8)
Lesotho	Age-standardized	12.1 (4.7–24.3)	12.0 (4.5–24.2)	12.0 (4.7–23.5)	12.0 (5.1–24.2)	12.0 (5.1–24.2)
Lesotho	All age	12.2 (4.7–24.6)	12.2 (4.7–24.6)	12.1 (4.9–23.5)	12.1 (5.3–24.1)	12.1 (5.3–24.2)

Supplementary Table S11: Prevalence of male SVAC by age and location for 1990, 2000, 2010, 2020, and 2023						
**Location**	**Age Range**	**1990**	**2000**	**2010**	**2020**	**2023**
Namibia	20 to 24	14.2 (5.6–27.9)	14.2 (6.1–26.3)	14.0 (7.3–24.1)	13.7 (8.9–20.2)	13.8 (8.5–21.5)
Namibia	25 to 29	14.6 (5.7–28.9)	14.6 (5.5–28.8)	14.6 (5.7–28.4)	14.6 (5.9–29.9)	14.6 (6.0–29.6)
Namibia	30 to 34	15.9 (6.3–31.1)	15.9 (6.1–31.0)	15.9 (6.3–30.5)	15.9 (6.5–32.1)	15.9 (6.6–31.8)
Namibia	35 to 39	16.2 (6.5–31.6)	16.2 (6.2–31.6)	16.3 (6.4–31.1)	16.3 (6.7–32.7)	16.2 (6.8–32.3)
Namibia	40 to 44	17.0 (6.8–32.9)	17.0 (6.5–32.8)	17.0 (6.8–32.3)	17.0 (7.0–33.9)	17.0 (7.1–33.5)
Namibia	45 to 49	16.8 (6.7–32.5)	16.7 (6.4–32.4)	16.8 (6.7–32.0)	16.7 (6.9–33.5)	16.7 (7.0–33.1)
Namibia	50 to 54	16.6 (6.6–32.2)	16.6 (6.4–32.2)	16.6 (6.6–31.7)	16.6 (6.8–33.1)	16.5 (6.9–32.8)
Namibia	55 to 59	16.3 (6.5–31.7)	16.3 (6.2–31.6)	16.3 (6.4–31.2)	16.2 (6.7–32.6)	16.2 (6.7–32.3)
Namibia	60 to 64	16.0 (6.3–31.2)	15.9 (6.1–31.1)	16.0 (6.3–30.6)	15.9 (6.5–32.0)	15.9 (6.6–31.7)
Namibia	65 to 69	15.0 (5.9–29.6)	15.0 (5.7–29.5)	15.0 (5.9–29.1)	14.9 (6.1–30.4)	14.9 (6.1–30.1)
Namibia	70 to 74	12.2 (4.7–24.6)	12.1 (4.5–24.6)	12.2 (4.6–24.2)	12.1 (4.8–25.3)	12.1 (4.8–25.0)
Namibia	75 to 79	11.0 (4.2–22.6)	11.0 (4.0–22.5)	11.0 (4.1–22.2)	10.9 (4.3–23.2)	10.9 (4.3–23.0)
Namibia	80 to 84	7.9 (2.9–16.6)	7.9 (2.8–16.6)	7.9 (2.9–16.3)	7.8 (3.0–17.1)	7.8 (3.0–16.9)
Namibia	85 to 89	7.1 (2.6–15.2)	7.1 (2.5–15.1)	7.1 (2.6–14.9)	7.1 (2.7–15.6)	7.1 (2.7–15.4)
Namibia	90 to 94	6.5 (2.4–13.9)	6.5 (2.3–13.8)	6.5 (2.3–13.6)	6.4 (2.4–14.3)	6.4 (2.4–14.1)

Supplementary Table S11: Prevalence of male SVAC by age and location for 1990, 2000, 2010, 2020, and 2023						
**Location**	**Age Range**	**1990**	**2000**	**2010**	**2020**	**2023**
Namibia	95 plus	6.4 (2.3–13.8)	6.4 (2.2–13.8)	6.4 (2.3–13.5)	6.4 (2.4–14.2)	6.4 (2.4–14.0)
Namibia	Age-standardized	15.3 (6.1–29.6)	15.2 (6.0–29.8)	15.2 (6.2–28.6)	15.2 (6.8–29.5)	15.2 (6.9–29.2)
Namibia	All age	15.4 (6.2–30.0)	15.3 (6.2–29.7)	15.4 (6.5–28.6)	15.4 (7.1–29.2)	15.4 (7.2–29.1)
South Africa	20 to 24	19.2 (7.3–37.3)	19.1 (7.5–36.1)	19.1 (7.7–35.6)	19.3 (7.3–38.8)	19.4 (7.1–40.0)
South Africa	25 to 29	19.6 (7.4–37.9)	19.4 (7.7–36.7)	19.4 (7.9–36.1)	19.7 (7.5–39.2)	19.8 (7.4–40.5)
South Africa	30 to 34	21.1 (8.1–40.4)	20.9 (8.4–38.9)	20.9 (8.7–38.4)	21.2 (8.3–41.6)	21.3 (8.4–42.8)
South Africa	35 to 39	21.3 (8.2–40.7)	21.2 (8.5–39.3)	21.2 (8.8–38.8)	21.4 (8.4–41.9)	21.5 (8.5–43.2)
South Africa	40 to 44	22.0 (8.6–41.8)	21.9 (8.9–40.4)	21.9 (9.2–39.8)	22.1 (8.8–42.6)	22.2 (8.5–43.7)
South Africa	45 to 49	21.6 (8.4–41.1)	21.4 (8.7–39.7)	21.5 (8.9–39.2)	21.7 (8.6–43.7)	21.8 (8.4–44.0)
South Africa	50 to 54	21.2 (8.2–40.6)	21.1 (8.5–39.2)	21.1 (8.8–38.7)	21.4 (8.3–42.3)	21.4 (8.2–43.1)
South Africa	55 to 59	20.7 (8.0–39.8)	20.6 (8.2–38.4)	20.6 (8.5–37.9)	20.8 (8.2–41.0)	20.9 (8.2–42.3)
South Africa	60 to 64	20.2 (7.7–39.2)	20.0 (8.0–37.6)	20.1 (8.2–37.1)	20.3 (7.7–40.4)	20.4 (7.6–41.4)
South Africa	65 to 69	18.9 (7.2–36.9)	18.8 (7.4–35.7)	18.9 (7.7–35.3)	19.1 (7.3–38.5)	19.1 (7.1–39.5)
South Africa	70 to 74	15.4 (5.6–31.1)	15.3 (5.8–30.0)	15.3 (6.0–29.6)	15.5 (5.8–32.4)	15.6 (5.8–33.4)
South Africa	75 to 79	14.0 (5.0–28.7)	13.9 (5.2–27.6)	13.9 (5.4–27.2)	14.1 (5.1–29.8)	14.2 (5.0–30.9)

Supplementary Table S11: Prevalence of male SVAC by age and location for 1990, 2000, 2010, 2020, and 2023						
**Location**	**Age Range**	**1990**	**2000**	**2010**	**2020**	**2023**
South Africa	80 to 84	10.1 (3.5–21.5)	10.0 (3.6–20.6)	10.0 (3.7–20.3)	10.1 (3.6–22.8)	10.2 (3.4–23.3)
South Africa	85 to 89	9.1 (3.1–19.8)	9.0 (3.2–18.8)	9.1 (3.3–18.5)	9.2 (3.2–21.5)	9.3 (3.2–21.6)
South Africa	90 to 94	8.3 (2.8–18.0)	8.2 (2.9–17.2)	8.2 (3.0–17.0)	8.3 (2.8–19.0)	8.4 (2.8–19.5)
South Africa	95 plus	8.2 (2.8–17.8)	8.1 (2.9–17.1)	8.2 (3.0–16.8)	8.3 (2.8–18.9)	8.3 (2.7–19.5)
South Africa	Age-standardized	19.9 (7.6–38.3)	19.8 (7.9–37.1)	19.8 (8.1–36.6)	20.0 (7.8–39.4)	20.1 (7.5–40.7)
South Africa	All age	20.2 (7.7–38.8)	20.1 (8.0–37.7)	20.1 (8.3–37.1)	20.4 (8.0–40.2)	20.5 (7.7–41.4)
Zimbabwe	20 to 24	7.5 (3.1–14.5)	7.4 (3.4–13.4)	7.4 (4.1–12.2)	7.6 (4.0–12.7)	7.6 (3.7–13.6)
Zimbabwe	25 to 29	7.7 (2.8–16.6)	7.6 (3.0–15.6)	7.5 (3.6–13.6)	7.6 (4.1–12.8)	7.6 (4.0–12.7)
Zimbabwe	30 to 34	8.3 (2.8–18.6)	8.2 (3.0–17.5)	8.0 (3.4–15.6)	7.9 (3.9–14.5)	7.9 (3.8–14.2)
Zimbabwe	35 to 39	8.7 (2.7–20.1)	8.6 (2.9–19.2)	8.5 (3.1–17.9)	8.6 (3.3–18.8)	8.6 (3.3–18.5)
Zimbabwe	40 to 44	9.1 (2.8–20.8)	9.0 (3.0–20.0)	8.9 (3.3–18.6)	9.0 (3.5–19.5)	9.0 (3.5–19.2)
Zimbabwe	45 to 49	8.9 (2.8–20.4)	8.8 (3.0–19.5)	8.7 (3.2–18.2)	8.8 (3.4–19.1)	8.8 (3.4–19.0)
Zimbabwe	50 to 54	8.7 (2.7–20.1)	8.6 (2.9–19.3)	8.5 (3.1–17.9)	8.6 (3.3–18.8)	8.6 (3.3–18.5)
Zimbabwe	55 to 59	8.5 (2.6–19.7)	8.4 (2.9–18.8)	8.3 (3.0–17.5)	8.4 (3.2–18.3)	8.4 (3.2–18.2)
Zimbabwe	60 to 64	8.2 (2.5–19.1)	8.2 (2.7–18.2)	8.1 (2.9–17.0)	8.1 (3.1–17.8)	8.1 (3.1–17.5)

Supplementary Table S11: Prevalence of male SVAC by age and location for 1990, 2000, 2010, 2020, and 2023						
**Location**	**Age Range**	**1990**	**2000**	**2010**	**2020**	**2023**
Zimbabwe	65 to 69	7.7 (2.3–18.0)	7.6 (2.5–17.0)	7.5 (2.7–15.8)	7.6 (2.9–16.6)	7.6 (2.9–16.4)
Zimbabwe	70 to 74	6.1 (1.8–14.4)	6.0 (2.0–13.7)	5.9 (2.1–12.5)	6.0 (2.2–13.4)	6.0 (2.2–13.3)
Zimbabwe	75 to 79	5.5 (1.6–13.1)	5.4 (1.7–12.4)	5.3 (1.9–11.4)	5.3 (2.0–12.1)	5.4 (2.0–12.0)
Zimbabwe	80 to 84	3.8 (1.1– 9.4)	3.8 (1.2– 8.8)	3.7 (1.3– 8.1)	3.7 (1.4– 8.6)	3.7 (1.4– 8.4)
Zimbabwe	85 to 89	3.4 (1.0– 8.5)	3.4 (1.1– 8.0)	3.3 (1.2– 7.3)	3.3 (1.2– 7.7)	3.3 (1.2– 7.6)
Zimbabwe	90 to 94	3.1 (0.9– 7.6)	3.1 (1.0– 7.2)	3.0 (1.0– 6.6)	3.0 (1.1– 7.0)	3.0 (1.1– 6.9)
Zimbabwe	95 plus	3.1 (0.9– 7.5)	3.0 (1.0– 7.1)	3.0 (1.0– 6.6)	3.0 (1.1– 6.9)	3.0 (1.1– 6.8)
Zimbabwe	Age-standardized	8.0 (2.7–17.7)	7.9 (2.9–16.9)	7.8 (3.2–15.7)	7.9 (3.6–15.7)	7.9 (3.5–15.8)
Zimbabwe	All age	8.1 (2.9–17.3)	8.0 (3.1–16.4)	7.9 (3.4–15.2)	8.1 (3.8–15.7)	8.1 (3.7–15.9)
Western sub-Saharan Africa	20 to 24	24.0 (13.0–39.0)	22.8 (12.0–37.2)	20.3 (10.8–33.1)	19.5 (9.9–34.3)	19.6 (9.3–35.1)
Western sub-Saharan Africa	25 to 29	24.4 (13.6–39.2)	23.3 (12.7–37.5)	20.9 (10.9–34.5)	19.9 (10.3–34.3)	19.9 (9.9–35.1)
Western sub-Saharan Africa	30 to 34	25.7 (14.0–41.1)	25.2 (14.3–39.3)	24.1 (14.1–37.1)	23.4 (12.8–39.4)	23.5 (12.0–39.9)
Western sub-Saharan Africa	35 to 39	25.9 (13.6–42.2)	25.2 (14.3–39.4)	23.9 (14.3–36.4)	23.2 (12.6–38.6)	23.2 (11.9–39.6)
Western sub-Saharan Africa	40 to 44	26.5 (13.5–43.6)	26.4 (14.6–41.2)	26.6 (16.6–39.1)	26.6 (15.7–42.3)	26.6 (14.5–42.8)
Western sub-Saharan Africa	45 to 49	26.2 (13.0–43.6)	25.8 (14.0–41.0)	25.4 (15.8–38.0)	25.4 (15.1–39.9)	25.4 (14.2–40.8)

Supplementary Table S11: Prevalence of male SVAC by age and location for 1990, 2000, 2010, 2020, and 2023						
**Location**	**Age Range**	**1990**	**2000**	**2010**	**2020**	**2023**
Western sub-Saharan Africa	50 to 54	25.3 (12.3–43.0)	24.8 (13.1–40.0)	24.2 (14.5–37.0)	24.3 (14.0–39.3)	24.4 (12.9–39.8)
Western sub-Saharan Africa	55 to 59	25.5 (12.1–43.4)	25.6 (13.4–41.5)	25.6 (15.2–39.2)	25.5 (14.9–40.5)	25.5 (14.0–41.0)
Western sub-Saharan Africa	60 to 64	24.7 (11.6–42.4)	24.6 (12.7–40.3)	24.4 (14.1–37.9)	24.0 (13.7–39.2)	24.0 (12.7–39.7)
Western sub-Saharan Africa	65 to 69	22.6 (9.6–41.2)	22.6 (9.3–41.3)	22.3 (9.4–40.2)	22.0 (9.6–41.5)	22.0 (9.7–41.8)
Western sub-Saharan Africa	70 to 74	18.5 (7.6–35.1)	18.5 (7.3–35.2)	18.3 (7.4–34.2)	18.1 (7.6–35.5)	18.1 (7.6–35.8)
Western sub-Saharan Africa	75 to 79	16.8 (6.7–32.4)	16.7 (6.5–32.2)	16.7 (6.7–31.7)	16.5 (6.9–33.0)	16.5 (6.8–33.2)
Western sub-Saharan Africa	80 to 84	12.2 (4.7–24.7)	12.2 (4.5–24.5)	12.2 (4.7–24.2)	12.1 (4.8–25.3)	12.1 (4.8–25.6)
Western sub-Saharan Africa	85 to 89	11.1 (4.2–22.7)	11.0 (4.0–22.5)	11.0 (4.1–22.0)	11.1 (4.4–23.4)	11.1 (4.4–23.7)
Western sub-Saharan Africa	90 to 94	10.2 (3.8–21.0)	10.1 (3.6–20.7)	10.0 (3.7–20.3)	10.2 (4.0–21.7)	10.2 (4.0–22.1)
Western sub-Saharan Africa	95 plus	10.2 (3.9–21.1)	10.0 (3.6–20.7)	10.0 (3.7–20.2)	10.1 (3.9–21.5)	10.2 (4.0–22.1)
Western sub-Saharan Africa	Age-standardized	24.3 (12.4–40.1)	23.8 (12.6–38.8)	22.8 (12.8–36.0)	22.4 (12.2–37.8)	22.4 (11.6–38.4)
Western sub-Saharan Africa	All age	24.8 (12.9–40.5)	24.1 (13.0–38.9)	22.9 (13.0–35.8)	22.3 (12.2–37.8)	22.4 (11.6–38.2)
Benin	20 to 24	20.4 (8.4–38.1)	20.1 (8.0–37.7)	19.8 (8.1–36.7)	19.7 (8.3–38.1)	19.6 (8.5–37.8)
Benin	25 to 29	20.9 (8.7–38.9)	20.7 (8.3–38.6)	20.4 (8.4–37.5)	20.2 (8.6–38.9)	20.2 (8.7–38.6)
Benin	30 to 34	22.6 (9.5–41.4)	22.3 (9.1–41.0)	22.0 (9.2–40.0)	21.8 (9.5–41.4)	21.8 (9.6–41.0)

Supplementary Table S11: Prevalence of male SVAC by age and location for 1990, 2000, 2010, 2020, and 2023						
**Location**	**Age Range**	**1990**	**2000**	**2010**	**2020**	**2023**
Benin	35 to 39	22.9 (9.7–41.9)	22.7 (9.3–41.6)	22.4 (9.4–40.5)	22.2 (9.7–42.0)	22.2 (9.8–41.6)
Benin	40 to 44	23.8 (10.2–43.1)	23.6 (9.7–42.8)	23.3 (9.8–41.8)	23.1 (10.1–43.3)	23.1 (10.2–42.9)
Benin	45 to 49	23.3 (9.9–42.5)	23.1 (9.5–42.2)	22.9 (9.6–41.2)	22.7 (9.9–42.7)	22.7 (10.0–42.3)
Benin	50 to 54	22.9 (9.7–41.9)	22.7 (9.3–41.6)	22.5 (9.4–40.7)	22.3 (9.7–42.1)	22.3 (9.8–41.8)
Benin	55 to 59	22.3 (9.4–41.0)	22.1 (9.0–40.7)	21.9 (9.1–39.8)	21.7 (9.4–41.2)	21.7 (9.5–40.9)
Benin	60 to 64	21.7 (9.1–40.1)	21.5 (8.7–39.8)	21.3 (8.8–38.9)	21.1 (9.1–40.3)	21.1 (9.2–39.9)
Benin	65 to 69	20.2 (8.4–37.9)	20.1 (8.0–37.7)	19.8 (8.1–36.8)	19.7 (8.4–38.1)	19.7 (8.5–37.8)
Benin	70 to 74	16.5 (6.6–32.0)	16.3 (6.3–31.7)	16.1 (6.4–30.9)	16.0 (6.6–32.2)	16.0 (6.6–31.9)
Benin	75 to 79	14.9 (5.9–29.4)	14.8 (5.6–29.2)	14.6 (5.7–28.4)	14.5 (5.9–29.6)	14.5 (5.9–29.3)
Benin	80 to 84	10.8 (4.1–22.1)	10.7 (3.9–21.9)	10.5 (3.9–21.2)	10.4 (4.1–22.2)	10.4 (4.1–22.0)
Benin	85 to 89	9.7 (3.6–20.2)	9.6 (3.5–20.0)	9.5 (3.5–19.4)	9.4 (3.6–20.3)	9.4 (3.7–20.1)
Benin	90 to 94	8.8 (3.3–18.5)	8.7 (3.1–18.3)	8.6 (3.2–17.7)	8.5 (3.3–18.6)	8.5 (3.3–18.4)
Benin	95 plus	8.7 (3.2–18.3)	8.7 (3.1–18.1)	8.5 (3.1–17.6)	8.5 (3.2–18.4)	8.5 (3.3–18.2)
Benin	Age-standardized	21.3 (9.0–39.5)	21.1 (8.5–39.2)	20.9 (8.7–38.2)	20.7 (8.9–39.6)	20.7 (9.0–39.2)
Benin	All age	21.5 (9.0–39.8)	21.4 (8.6–39.6)	21.2 (8.8–38.7)	21.0 (9.0–40.1)	21.0 (9.2–39.8)

Supplementary Table S11: Prevalence of male SVAC by age and location for 1990, 2000, 2010, 2020, and 2023						
**Location**	**Age Range**	**1990**	**2000**	**2010**	**2020**	**2023**
Burkina Faso	20 to 24	12.3 (4.7–24.8)	12.1 (4.5–24.5)	11.9 (4.5–23.7)	11.7 (4.6–24.6)	11.7 (4.7–24.3)
Burkina Faso	25 to 29	13.0 (5.0–26.1)	12.8 (4.8–25.8)	12.6 (4.8–24.9)	12.3 (4.9–25.8)	12.3 (4.9–25.5)
Burkina Faso	30 to 34	14.5 (5.7–28.6)	14.3 (5.4–28.3)	14.0 (5.4–27.3)	13.7 (5.5–28.3)	13.7 (5.6–28.0)
Burkina Faso	35 to 39	15.0 (5.9–29.5)	14.8 (5.6–29.2)	14.5 (5.6–28.2)	14.2 (5.7–29.1)	14.2 (5.8–28.8)
Burkina Faso	40 to 44	15.8 (6.3–30.9)	15.6 (5.9–30.6)	15.3 (6.0–29.5)	15.0 (6.1–30.5)	15.0 (6.2–30.2)
Burkina Faso	45 to 49	15.6 (6.2–30.6)	15.5 (5.9–30.3)	15.2 (5.9–29.3)	14.9 (6.0–30.3)	14.9 (6.1–30.0)
Burkina Faso	50 to 54	15.5 (6.1–30.4)	15.3 (5.8–30.1)	15.1 (5.9–29.1)	14.8 (6.0–30.1)	14.7 (6.1–29.8)
Burkina Faso	55 to 59	15.1 (6.0–29.8)	15.0 (5.7–29.5)	14.7 (5.7–28.6)	14.4 (5.8–29.5)	14.4 (5.9–29.2)
Burkina Faso	60 to 64	14.7 (5.8–29.1)	14.6 (5.5–28.8)	14.3 (5.6–27.9)	14.0 (5.7–28.9)	14.0 (5.7–28.5)
Burkina Faso	65 to 69	13.7 (5.3–27.4)	13.6 (5.1–27.1)	13.3 (5.1–26.2)	13.0 (5.2–27.1)	13.0 (5.3–26.8)
Burkina Faso	70 to 74	11.0 (4.2–22.5)	10.9 (3.9–22.2)	10.6 (4.0–21.4)	10.4 (4.1–22.2)	10.4 (4.1–22.0)
Burkina Faso	75 to 79	9.9 (3.7–20.5)	9.8 (3.5–20.2)	9.6 (3.6–19.5)	9.4 (3.6–20.2)	9.4 (3.7–20.0)
Burkina Faso	80 to 84	7.0 (2.5–14.9)	6.9 (2.4–14.7)	6.8 (2.4–14.1)	6.6 (2.5–14.7)	6.6 (2.5–14.5)
Burkina Faso	85 to 89	6.3 (2.3–13.5)	6.2 (2.2–13.3)	6.1 (2.2–12.8)	5.9 (2.2–13.3)	5.9 (2.2–13.1)
Burkina Faso	90 to 94	5.7 (2.0–12.2)	5.6 (1.9–12.1)	5.5 (2.0–11.6)	5.4 (2.0–12.1)	5.4 (2.0–11.9)

Supplementary Table S11: Prevalence of male SVAC by age and location for 1990, 2000, 2010, 2020, and 2023						
**Location**	**Age Range**	**1990**	**2000**	**2010**	**2020**	**2023**
Burkina Faso	95 plus	5.6 (2.0–12.1)	5.6 (1.9–12.0)	5.4 (1.9–11.5)	5.3 (2.0–12.0)	5.3 (2.0–11.8)
Burkina Faso	Age-standardized	13.9 (5.4–27.6)	13.8 (5.2–27.3)	13.5 (5.2–26.4)	13.2 (5.3–27.3)	13.2 (5.4–27.0)
Burkina Faso	All age	13.9 (5.4–27.7)	13.7 (5.1–27.3)	13.5 (5.2–26.4)	13.3 (5.3–27.5)	13.3 (5.4–27.2)
Cabo Verde	20 to 24	20.4 (8.4–38.1)	20.1 (8.0–37.7)	19.8 (8.1–36.7)	19.7 (8.3–38.1)	19.6 (8.5–37.8)
Cabo Verde	25 to 29	20.9 (8.7–38.9)	20.7 (8.3–38.6)	20.4 (8.4–37.5)	20.2 (8.6–38.9)	20.2 (8.7–38.6)
Cabo Verde	30 to 34	22.6 (9.5–41.4)	22.3 (9.1–41.0)	22.0 (9.2–40.0)	21.8 (9.5–41.4)	21.8 (9.6–41.0)
Cabo Verde	35 to 39	22.9 (9.7–41.9)	22.7 (9.3–41.6)	22.4 (9.4–40.5)	22.2 (9.7–42.0)	22.2 (9.8–41.6)
Cabo Verde	40 to 44	23.8 (10.2–43.1)	23.6 (9.7–42.8)	23.3 (9.8–41.8)	23.1 (10.1–43.3)	23.1 (10.2–42.9)
Cabo Verde	45 to 49	23.3 (9.9–42.5)	23.1 (9.5–42.2)	22.9 (9.6–41.2)	22.7 (9.9–42.7)	22.7 (10.0–42.3)
Cabo Verde	50 to 54	22.9 (9.7–41.9)	22.7 (9.3–41.6)	22.5 (9.4–40.7)	22.3 (9.7–42.1)	22.3 (9.8–41.8)
Cabo Verde	55 to 59	22.3 (9.4–41.0)	22.1 (9.0–40.7)	21.9 (9.1–39.8)	21.7 (9.4–41.2)	21.7 (9.5–40.9)
Cabo Verde	60 to 64	21.7 (9.1–40.1)	21.5 (8.7–39.8)	21.3 (8.8–38.9)	21.1 (9.1–40.3)	21.1 (9.2–39.9)
Cabo Verde	65 to 69	20.2 (8.4–37.9)	20.1 (8.0–37.7)	19.8 (8.1–36.8)	19.7 (8.4–38.1)	19.7 (8.5–37.8)
Cabo Verde	70 to 74	16.5 (6.6–32.0)	16.3 (6.3–31.7)	16.1 (6.4–30.9)	16.0 (6.6–32.2)	16.0 (6.6–31.9)
Cabo Verde	75 to 79	14.9 (5.9–29.4)	14.8 (5.6–29.2)	14.6 (5.7–28.4)	14.5 (5.9–29.6)	14.5 (5.9–29.3)

Supplementary Table S11: Prevalence of male SVAC by age and location for 1990, 2000, 2010, 2020, and 2023						
**Location**	**Age Range**	**1990**	**2000**	**2010**	**2020**	**2023**
Cabo Verde	80 to 84	10.8 (4.1–22.1)	10.7 (3.9–21.9)	10.5 (3.9–21.2)	10.4 (4.1–22.2)	10.4 (4.1–22.0)
Cabo Verde	85 to 89	9.7 (3.6–20.2)	9.6 (3.5–20.0)	9.5 (3.5–19.4)	9.4 (3.6–20.3)	9.4 (3.7–20.1)
Cabo Verde	90 to 94	8.8 (3.3–18.5)	8.7 (3.1–18.3)	8.6 (3.2–17.7)	8.5 (3.3–18.6)	8.5 (3.3–18.4)
Cabo Verde	95 plus	8.7 (3.2–18.3)	8.7 (3.1–18.1)	8.5 (3.1–17.6)	8.5 (3.2–18.4)	8.5 (3.3–18.2)
Cabo Verde	Age-standardized	21.3 (9.0–39.5)	21.1 (8.5–39.2)	20.9 (8.7–38.2)	20.7 (8.9–39.6)	20.7 (9.0–39.2)
Cabo Verde	All age	20.9 (8.8–38.9)	21.1 (8.5–39.1)	21.0 (8.7–38.4)	21.1 (9.1–40.2)	21.1 (9.2–40.0)
Cameroon	20 to 24	20.4 (8.4–38.1)	20.1 (8.0–37.7)	19.8 (8.1–36.7)	19.7 (8.3–38.1)	19.6 (8.5–37.8)
Cameroon	25 to 29	20.9 (8.7–38.9)	20.7 (8.3–38.6)	20.4 (8.4–37.5)	20.2 (8.6–38.9)	20.2 (8.7–38.6)
Cameroon	30 to 34	22.6 (9.5–41.4)	22.3 (9.1–41.0)	22.0 (9.2–40.0)	21.8 (9.5–41.4)	21.8 (9.6–41.0)
Cameroon	35 to 39	22.9 (9.7–41.9)	22.7 (9.3–41.6)	22.4 (9.4–40.5)	22.2 (9.7–42.0)	22.2 (9.8–41.6)
Cameroon	40 to 44	23.8 (10.2–43.1)	23.6 (9.7–42.8)	23.3 (9.8–41.8)	23.1 (10.1–43.3)	23.1 (10.2–42.9)
Cameroon	45 to 49	23.3 (9.9–42.5)	23.1 (9.5–42.2)	22.9 (9.6–41.2)	22.7 (9.9–42.7)	22.7 (10.0–42.3)
Cameroon	50 to 54	22.9 (9.7–41.9)	22.7 (9.3–41.6)	22.5 (9.4–40.7)	22.3 (9.7–42.1)	22.3 (9.8–41.8)
Cameroon	55 to 59	22.3 (9.4–41.0)	22.1 (9.0–40.7)	21.9 (9.1–39.8)	21.7 (9.4–41.2)	21.7 (9.5–40.9)
Cameroon	60 to 64	21.7 (9.1–40.1)	21.5 (8.7–39.8)	21.3 (8.8–38.9)	21.1 (9.1–40.3)	21.1 (9.2–39.9)

Supplementary Table S11: Prevalence of male SVAC by age and location for 1990, 2000, 2010, 2020, and 2023						
**Location**	**Age Range**	**1990**	**2000**	**2010**	**2020**	**2023**
Cameroon	65 to 69	20.2 (8.4–37.9)	20.1 (8.0–37.7)	19.8 (8.1–36.8)	19.7 (8.4–38.1)	19.7 (8.5–37.8)
Cameroon	70 to 74	16.5 (6.6–32.0)	16.3 (6.3–31.7)	16.1 (6.4–30.9)	16.0 (6.6–32.2)	16.0 (6.6–31.9)
Cameroon	75 to 79	14.9 (5.9–29.4)	14.8 (5.6–29.2)	14.6 (5.7–28.4)	14.5 (5.9–29.6)	14.5 (5.9–29.3)
Cameroon	80 to 84	10.8 (4.1–22.1)	10.7 (3.9–21.9)	10.5 (3.9–21.2)	10.4 (4.1–22.2)	10.4 (4.1–22.0)
Cameroon	85 to 89	9.7 (3.6–20.2)	9.6 (3.5–20.0)	9.5 (3.5–19.4)	9.4 (3.6–20.3)	9.4 (3.7–20.1)
Cameroon	90 to 94	8.8 (3.3–18.5)	8.7 (3.1–18.3)	8.6 (3.2–17.7)	8.5 (3.3–18.6)	8.5 (3.3–18.4)
Cameroon	95 plus	8.7 (3.2–18.3)	8.7 (3.1–18.1)	8.5 (3.1–17.6)	8.5 (3.2–18.4)	8.5 (3.3–18.2)
Cameroon	Age-standardized	21.3 (9.0–39.5)	21.1 (8.5–39.2)	20.9 (8.7–38.2)	20.7 (8.9–39.6)	20.7 (9.0–39.2)
Cameroon	All age	21.6 (9.1–40.0)	21.4 (8.7–39.7)	21.2 (8.8–38.7)	21.1 (9.1–40.2)	21.1 (9.2–39.9)
Chad	20 to 24	20.4 (8.4–38.1)	20.1 (8.0–37.7)	19.8 (8.1–36.7)	19.7 (8.3–38.1)	19.6 (8.5–37.8)
Chad	25 to 29	20.9 (8.7–38.9)	20.7 (8.3–38.6)	20.4 (8.4–37.5)	20.2 (8.6–38.9)	20.2 (8.7–38.6)
Chad	30 to 34	22.6 (9.5–41.4)	22.3 (9.1–41.0)	22.0 (9.2–40.0)	21.8 (9.5–41.4)	21.8 (9.6–41.0)
Chad	35 to 39	22.9 (9.7–41.9)	22.7 (9.3–41.6)	22.4 (9.4–40.5)	22.2 (9.7–42.0)	22.2 (9.8–41.6)
Chad	40 to 44	23.8 (10.2–43.1)	23.6 (9.7–42.8)	23.3 (9.8–41.8)	23.1 (10.1–43.3)	23.1 (10.2–42.9)
Chad	45 to 49	23.3 (9.9–42.5)	23.1 (9.5–42.2)	22.9 (9.6–41.2)	22.7 (9.9–42.7)	22.7 (10.0–42.3)

Supplementary Table S11: Prevalence of male SVAC by age and location for 1990, 2000, 2010, 2020, and 2023						
**Location**	**Age Range**	**1990**	**2000**	**2010**	**2020**	**2023**
Chad	50 to 54	22.9 (9.7–41.9)	22.7 (9.3–41.6)	22.5 (9.4–40.7)	22.3 (9.7–42.1)	22.3 (9.8–41.8)
Chad	55 to 59	22.3 (9.4–41.0)	22.1 (9.0–40.7)	21.9 (9.1–39.8)	21.7 (9.4–41.2)	21.7 (9.5–40.9)
Chad	60 to 64	21.7 (9.1–40.1)	21.5 (8.7–39.8)	21.3 (8.8–38.9)	21.1 (9.1–40.3)	21.1 (9.2–39.9)
Chad	65 to 69	20.2 (8.4–37.9)	20.1 (8.0–37.7)	19.8 (8.1–36.8)	19.7 (8.4–38.1)	19.7 (8.5–37.8)
Chad	70 to 74	16.5 (6.6–32.0)	16.3 (6.3–31.7)	16.1 (6.4–30.9)	16.0 (6.6–32.2)	16.0 (6.6–31.9)
Chad	75 to 79	14.9 (5.9–29.4)	14.8 (5.6–29.2)	14.6 (5.7–28.4)	14.5 (5.9–29.6)	14.5 (5.9–29.3)
Chad	80 to 84	10.8 (4.1–22.1)	10.7 (3.9–21.9)	10.5 (3.9–21.2)	10.4 (4.1–22.2)	10.4 (4.1–22.0)
Chad	85 to 89	9.7 (3.6–20.2)	9.6 (3.5–20.0)	9.5 (3.5–19.4)	9.4 (3.6–20.3)	9.4 (3.7–20.1)
Chad	90 to 94	8.8 (3.3–18.5)	8.7 (3.1–18.3)	8.6 (3.2–17.7)	8.5 (3.3–18.6)	8.5 (3.3–18.4)
Chad	95 plus	8.7 (3.2–18.3)	8.7 (3.1–18.1)	8.5 (3.1–17.6)	8.5 (3.2–18.4)	8.5 (3.3–18.2)
Chad	Age-standardized	21.3 (9.0–39.5)	21.1 (8.5–39.2)	20.9 (8.7–38.2)	20.7 (8.9–39.6)	20.7 (9.0–39.2)
Chad	All age	21.4 (9.0–39.7)	21.3 (8.6–39.5)	21.1 (8.8–38.6)	20.9 (9.0–40.0)	20.9 (9.1–39.6)
Côte d'Ivoire	20 to 24	28.3 (12.7–49.3)	28.3 (12.2–49.2)	28.4 (12.6–48.8)	28.8 (13.3–51.0)	28.8 (13.5–50.6)
Côte d'Ivoire	25 to 29	28.4 (12.7–49.4)	28.3 (12.2–49.3)	28.5 (12.7–48.8)	28.9 (13.4–51.1)	28.8 (13.5–50.7)
Côte d'Ivoire	30 to 34	29.9 (13.6–51.3)	29.9 (13.1–51.2)	30.0 (13.6–50.8)	30.4 (14.3–53.0)	30.4 (14.4–52.6)

Supplementary Table S11: Prevalence of male SVAC by age and location for 1990, 2000, 2010, 2020, and 2023						
**Location**	**Age Range**	**1990**	**2000**	**2010**	**2020**	**2023**
Côte d'Ivoire	35 to 39	29.9 (13.6–51.3)	29.9 (13.1–51.2)	30.0 (13.5–50.7)	30.3 (14.3–52.9)	30.3 (14.4–52.6)
Côte d'Ivoire	40 to 44	30.5 (14.0–52.1)	30.5 (13.5–52.0)	30.6 (13.9–51.5)	31.0 (14.6–53.7)	30.9 (14.8–53.3)
Côte d'Ivoire	45 to 49	29.7 (13.5–51.1)	29.7 (13.0–51.0)	29.8 (13.4–50.5)	30.1 (14.1–52.7)	30.1 (14.3–52.3)
Côte d'Ivoire	50 to 54	29.0 (13.1–50.2)	29.0 (12.6–50.1)	29.1 (13.0–49.6)	29.4 (13.7–51.8)	29.4 (13.8–51.4)
Côte d'Ivoire	55 to 59	28.2 (12.6–49.1)	28.1 (12.1–49.0)	28.3 (12.5–48.5)	28.5 (13.2–50.7)	28.5 (13.3–50.3)
Côte d'Ivoire	60 to 64	27.4 (12.2–48.1)	27.4 (11.7–48.0)	27.5 (12.1–47.5)	27.7 (12.7–49.6)	27.7 (12.8–49.2)
Côte d'Ivoire	65 to 69	25.7 (11.2–45.9)	25.7 (10.8–45.8)	25.8 (11.2–45.3)	26.0 (11.7–47.4)	26.0 (11.9–47.0)
Côte d'Ivoire	70 to 74	21.2 (8.9–39.5)	21.2 (8.5–39.4)	21.3 (8.8–38.9)	21.5 (9.3–40.9)	21.5 (9.4–40.6)
Côte d'Ivoire	75 to 79	19.4 (8.0–36.6)	19.3 (7.6–36.6)	19.4 (7.9–36.1)	19.6 (8.3–38.1)	19.6 (8.4–37.7)
Côte d'Ivoire	80 to 84	14.2 (5.5–28.2)	14.2 (5.3–28.1)	14.2 (5.5–27.7)	14.4 (5.8–29.5)	14.4 (5.9–29.1)
Côte d'Ivoire	85 to 89	12.9 (5.0–25.9)	12.9 (4.8–25.9)	12.9 (4.9–25.5)	13.1 (5.2–27.1)	13.1 (5.3–26.8)
Côte d'Ivoire	90 to 94	11.7 (4.5–23.9)	11.7 (4.3–23.8)	11.8 (4.5–23.5)	11.9 (4.7–25.0)	11.9 (4.8–24.7)
Côte d'Ivoire	95 plus	11.6 (4.4–23.7)	11.6 (4.3–23.6)	11.7 (4.4–23.3)	11.8 (4.7–24.8)	11.8 (4.7–24.5)
Côte d'Ivoire	Age-standardized	27.9 (12.5–48.6)	27.9 (12.1–48.5)	28.0 (12.5–48.1)	28.3 (13.1–50.2)	28.3 (13.2–49.8)
Côte d'Ivoire	All age	28.8 (13.0–49.8)	28.7 (12.5–49.7)	28.9 (12.9–49.3)	29.3 (13.6–51.5)	29.2 (13.8–51.2)

Supplementary Table S11: Prevalence of male SVAC by age and location for 1990, 2000, 2010, 2020, and 2023						
**Location**	**Age Range**	**1990**	**2000**	**2010**	**2020**	**2023**
The Gambia	20 to 24	20.4 (8.4–38.1)	20.1 (8.0–37.7)	19.8 (8.1–36.7)	19.7 (8.3–38.1)	19.6 (8.5–37.8)
The Gambia	25 to 29	20.9 (8.7–38.9)	20.7 (8.3–38.6)	20.4 (8.4–37.5)	20.2 (8.6–38.9)	20.2 (8.7–38.6)
The Gambia	30 to 34	22.6 (9.5–41.4)	22.3 (9.1–41.0)	22.0 (9.2–40.0)	21.8 (9.5–41.4)	21.8 (9.6–41.0)
The Gambia	35 to 39	22.9 (9.7–41.9)	22.7 (9.3–41.6)	22.4 (9.4–40.5)	22.2 (9.7–42.0)	22.2 (9.8–41.6)
The Gambia	40 to 44	23.8 (10.2–43.1)	23.6 (9.7–42.8)	23.3 (9.8–41.8)	23.1 (10.1–43.3)	23.1 (10.2–42.9)
The Gambia	45 to 49	23.3 (9.9–42.5)	23.1 (9.5–42.2)	22.9 (9.6–41.2)	22.7 (9.9–42.7)	22.7 (10.0–42.3)
The Gambia	50 to 54	22.9 (9.7–41.9)	22.7 (9.3–41.6)	22.5 (9.4–40.7)	22.3 (9.7–42.1)	22.3 (9.8–41.8)
The Gambia	55 to 59	22.3 (9.4–41.0)	22.1 (9.0–40.7)	21.9 (9.1–39.8)	21.7 (9.4–41.2)	21.7 (9.5–40.9)
The Gambia	60 to 64	21.7 (9.1–40.1)	21.5 (8.7–39.8)	21.3 (8.8–38.9)	21.1 (9.1–40.3)	21.1 (9.2–39.9)
The Gambia	65 to 69	20.2 (8.4–37.9)	20.1 (8.0–37.7)	19.8 (8.1–36.8)	19.7 (8.4–38.1)	19.7 (8.5–37.8)
The Gambia	70 to 74	16.5 (6.6–32.0)	16.3 (6.3–31.7)	16.1 (6.4–30.9)	16.0 (6.6–32.2)	16.0 (6.6–31.9)
The Gambia	75 to 79	14.9 (5.9–29.4)	14.8 (5.6–29.2)	14.6 (5.7–28.4)	14.5 (5.9–29.6)	14.5 (5.9–29.3)
The Gambia	80 to 84	10.8 (4.1–22.1)	10.7 (3.9–21.9)	10.5 (3.9–21.2)	10.4 (4.1–22.2)	10.4 (4.1–22.0)
The Gambia	85 to 89	9.7 (3.6–20.2)	9.6 (3.5–20.0)	9.5 (3.5–19.4)	9.4 (3.6–20.3)	9.4 (3.7–20.1)
The Gambia	90 to 94	8.8 (3.3–18.5)	8.7 (3.1–18.3)	8.6 (3.2–17.7)	8.5 (3.3–18.6)	8.5 (3.3–18.4)

Supplementary Table S11: Prevalence of male SVAC by age and location for 1990, 2000, 2010, 2020, and 2023						
**Location**	**Age Range**	**1990**	**2000**	**2010**	**2020**	**2023**
The Gambia	95 plus	8.7 (3.2–18.3)	8.7 (3.1–18.1)	8.5 (3.1–17.6)	8.5 (3.2–18.4)	8.5 (3.3–18.2)
The Gambia	Age-standardized	21.3 (9.0–39.5)	21.1 (8.5–39.2)	20.9 (8.7–38.2)	20.7 (8.9–39.6)	20.7 (9.0–39.2)
The Gambia	All age	21.7 (9.1–40.1)	21.4 (8.6–39.6)	21.1 (8.7–38.5)	21.0 (9.0–40.1)	21.0 (9.1–39.7)
Ghana	20 to 24	20.4 (8.4–38.1)	20.1 (8.0–37.7)	19.8 (8.1–36.7)	19.7 (8.3–38.1)	19.6 (8.5–37.8)
Ghana	25 to 29	20.9 (8.7–38.9)	20.7 (8.3–38.6)	20.4 (8.4–37.5)	20.2 (8.6–38.9)	20.2 (8.7–38.6)
Ghana	30 to 34	22.6 (9.5–41.4)	22.3 (9.1–41.0)	22.0 (9.2–40.0)	21.8 (9.5–41.4)	21.8 (9.6–41.0)
Ghana	35 to 39	22.9 (9.7–41.9)	22.7 (9.3–41.6)	22.4 (9.4–40.5)	22.2 (9.7–42.0)	22.2 (9.8–41.6)
Ghana	40 to 44	23.8 (10.2–43.1)	23.6 (9.7–42.8)	23.3 (9.8–41.8)	23.1 (10.1–43.3)	23.1 (10.2–42.9)
Ghana	45 to 49	23.3 (9.9–42.5)	23.1 (9.5–42.2)	22.9 (9.6–41.2)	22.7 (9.9–42.7)	22.7 (10.0–42.3)
Ghana	50 to 54	22.9 (9.7–41.9)	22.7 (9.3–41.6)	22.5 (9.4–40.7)	22.3 (9.7–42.1)	22.3 (9.8–41.8)
Ghana	55 to 59	22.3 (9.4–41.0)	22.1 (9.0–40.7)	21.9 (9.1–39.8)	21.7 (9.4–41.2)	21.7 (9.5–40.9)
Ghana	60 to 64	21.7 (9.1–40.1)	21.5 (8.7–39.8)	21.3 (8.8–38.9)	21.1 (9.1–40.3)	21.1 (9.2–39.9)
Ghana	65 to 69	20.2 (8.4–37.9)	20.1 (8.0–37.7)	19.8 (8.1–36.8)	19.7 (8.4–38.1)	19.7 (8.5–37.8)
Ghana	70 to 74	16.5 (6.6–32.0)	16.3 (6.3–31.7)	16.1 (6.4–30.9)	16.0 (6.6–32.2)	16.0 (6.6–31.9)
Ghana	75 to 79	14.9 (5.9–29.4)	14.8 (5.6–29.2)	14.6 (5.7–28.4)	14.5 (5.9–29.6)	14.5 (5.9–29.3)

Supplementary Table S11: Prevalence of male SVAC by age and location for 1990, 2000, 2010, 2020, and 2023						
**Location**	**Age Range**	**1990**	**2000**	**2010**	**2020**	**2023**
Ghana	80 to 84	10.8 (4.1–22.1)	10.7 (3.9–21.9)	10.5 (3.9–21.2)	10.4 (4.1–22.2)	10.4 (4.1–22.0)
Ghana	85 to 89	9.7 (3.6–20.2)	9.6 (3.5–20.0)	9.5 (3.5–19.4)	9.4 (3.6–20.3)	9.4 (3.7–20.1)
Ghana	90 to 94	8.8 (3.3–18.5)	8.7 (3.1–18.3)	8.6 (3.2–17.7)	8.5 (3.3–18.6)	8.5 (3.3–18.4)
Ghana	95 plus	8.7 (3.2–18.3)	8.7 (3.1–18.1)	8.5 (3.1–17.6)	8.5 (3.2–18.4)	8.5 (3.3–18.2)
Ghana	Age-standardized	21.3 (9.0–39.5)	21.1 (8.5–39.2)	20.9 (8.7–38.2)	20.7 (8.9–39.6)	20.7 (9.0–39.2)
Ghana	All age	21.7 (9.1–40.1)	21.5 (8.7–39.8)	21.2 (8.8–38.8)	21.1 (9.1–40.2)	21.1 (9.2–39.9)
Guinea	20 to 24	20.4 (8.4–38.1)	20.1 (8.0–37.7)	19.8 (8.1–36.7)	19.7 (8.3–38.1)	19.6 (8.5–37.8)
Guinea	25 to 29	20.9 (8.7–38.9)	20.7 (8.3–38.6)	20.4 (8.4–37.5)	20.2 (8.6–38.9)	20.2 (8.7–38.6)
Guinea	30 to 34	22.6 (9.5–41.4)	22.3 (9.1–41.0)	22.0 (9.2–40.0)	21.8 (9.5–41.4)	21.8 (9.6–41.0)
Guinea	35 to 39	22.9 (9.7–41.9)	22.7 (9.3–41.6)	22.4 (9.4–40.5)	22.2 (9.7–42.0)	22.2 (9.8–41.6)
Guinea	40 to 44	23.8 (10.2–43.1)	23.6 (9.7–42.8)	23.3 (9.8–41.8)	23.1 (10.1–43.3)	23.1 (10.2–42.9)
Guinea	45 to 49	23.3 (9.9–42.5)	23.1 (9.5–42.2)	22.9 (9.6–41.2)	22.7 (9.9–42.7)	22.7 (10.0–42.3)
Guinea	50 to 54	22.9 (9.7–41.9)	22.7 (9.3–41.6)	22.5 (9.4–40.7)	22.3 (9.7–42.1)	22.3 (9.8–41.8)
Guinea	55 to 59	22.3 (9.4–41.0)	22.1 (9.0–40.7)	21.9 (9.1–39.8)	21.7 (9.4–41.2)	21.7 (9.5–40.9)
Guinea	60 to 64	21.7 (9.1–40.1)	21.5 (8.7–39.8)	21.3 (8.8–38.9)	21.1 (9.1–40.3)	21.1 (9.2–39.9)

Supplementary Table S11: Prevalence of male SVAC by age and location for 1990, 2000, 2010, 2020, and 2023						
**Location**	**Age Range**	**1990**	**2000**	**2010**	**2020**	**2023**
Guinea	65 to 69	20.2 (8.4–37.9)	20.1 (8.0–37.7)	19.8 (8.1–36.8)	19.7 (8.4–38.1)	19.7 (8.5–37.8)
Guinea	70 to 74	16.5 (6.6–32.0)	16.3 (6.3–31.7)	16.1 (6.4–30.9)	16.0 (6.6–32.2)	16.0 (6.6–31.9)
Guinea	75 to 79	14.9 (5.9–29.4)	14.8 (5.6–29.2)	14.6 (5.7–28.4)	14.5 (5.9–29.6)	14.5 (5.9–29.3)
Guinea	80 to 84	10.8 (4.1–22.1)	10.7 (3.9–21.9)	10.5 (3.9–21.2)	10.4 (4.1–22.2)	10.4 (4.1–22.0)
Guinea	85 to 89	9.7 (3.6–20.2)	9.6 (3.5–20.0)	9.5 (3.5–19.4)	9.4 (3.6–20.3)	9.4 (3.7–20.1)
Guinea	90 to 94	8.8 (3.3–18.5)	8.7 (3.1–18.3)	8.6 (3.2–17.7)	8.5 (3.3–18.6)	8.5 (3.3–18.4)
Guinea	95 plus	8.7 (3.2–18.3)	8.7 (3.1–18.1)	8.5 (3.1–17.6)	8.5 (3.2–18.4)	8.5 (3.3–18.2)
Guinea	Age-standardized	21.3 (9.0–39.5)	21.1 (8.5–39.2)	20.9 (8.7–38.2)	20.7 (8.9–39.6)	20.7 (9.0–39.2)
Guinea	All age	21.5 (9.0–39.8)	21.3 (8.6–39.4)	21.0 (8.7–38.4)	20.9 (9.0–39.9)	20.9 (9.1–39.6)
Guinea-Bissau	20 to 24	20.4 (8.4–38.1)	20.1 (8.0–37.7)	19.8 (8.1–36.7)	19.7 (8.3–38.1)	19.6 (8.5–37.8)
Guinea-Bissau	25 to 29	20.9 (8.7–38.9)	20.7 (8.3–38.6)	20.4 (8.4–37.5)	20.2 (8.6–38.9)	20.2 (8.7–38.6)
Guinea-Bissau	30 to 34	22.6 (9.5–41.4)	22.3 (9.1–41.0)	22.0 (9.2–40.0)	21.8 (9.5–41.4)	21.8 (9.6–41.0)
Guinea-Bissau	35 to 39	22.9 (9.7–41.9)	22.7 (9.3–41.6)	22.4 (9.4–40.5)	22.2 (9.7–42.0)	22.2 (9.8–41.6)
Guinea-Bissau	40 to 44	23.8 (10.2–43.1)	23.6 (9.7–42.8)	23.3 (9.8–41.8)	23.1 (10.1–43.3)	23.1 (10.2–42.9)
Guinea-Bissau	45 to 49	23.3 (9.9–42.5)	23.1 (9.5–42.2)	22.9 (9.6–41.2)	22.7 (9.9–42.7)	22.7 (10.0–42.3)

Supplementary Table S11: Prevalence of male SVAC by age and location for 1990, 2000, 2010, 2020, and 2023						
**Location**	**Age Range**	**1990**	**2000**	**2010**	**2020**	**2023**
Guinea-Bissau	50 to 54	22.9 (9.7–41.9)	22.7 (9.3–41.6)	22.5 (9.4–40.7)	22.3 (9.7–42.1)	22.3 (9.8–41.8)
Guinea-Bissau	55 to 59	22.3 (9.4–41.0)	22.1 (9.0–40.7)	21.9 (9.1–39.8)	21.7 (9.4–41.2)	21.7 (9.5–40.9)
Guinea-Bissau	60 to 64	21.7 (9.1–40.1)	21.5 (8.7–39.8)	21.3 (8.8–38.9)	21.1 (9.1–40.3)	21.1 (9.2–39.9)
Guinea-Bissau	65 to 69	20.2 (8.4–37.9)	20.1 (8.0–37.7)	19.8 (8.1–36.8)	19.7 (8.4–38.1)	19.7 (8.5–37.8)
Guinea-Bissau	70 to 74	16.5 (6.6–32.0)	16.3 (6.3–31.7)	16.1 (6.4–30.9)	16.0 (6.6–32.2)	16.0 (6.6–31.9)
Guinea-Bissau	75 to 79	14.9 (5.9–29.4)	14.8 (5.6–29.2)	14.6 (5.7–28.4)	14.5 (5.9–29.6)	14.5 (5.9–29.3)
Guinea-Bissau	80 to 84	10.8 (4.1–22.1)	10.7 (3.9–21.9)	10.5 (3.9–21.2)	10.4 (4.1–22.2)	10.4 (4.1–22.0)
Guinea-Bissau	85 to 89	9.7 (3.6–20.2)	9.6 (3.5–20.0)	9.5 (3.5–19.4)	9.4 (3.6–20.3)	9.4 (3.7–20.1)
Guinea-Bissau	90 to 94	8.8 (3.3–18.5)	8.7 (3.1–18.3)	8.6 (3.2–17.7)	8.5 (3.3–18.6)	8.5 (3.3–18.4)
Guinea-Bissau	95 plus	8.7 (3.2–18.3)	8.7 (3.1–18.1)	8.5 (3.1–17.6)	8.5 (3.2–18.4)	8.5 (3.3–18.2)
Guinea-Bissau	Age-standardized	21.3 (9.0–39.5)	21.1 (8.5–39.2)	20.9 (8.7–38.2)	20.7 (8.9–39.6)	20.7 (9.0–39.2)
Guinea-Bissau	All age	21.6 (9.1–39.9)	21.4 (8.7–39.7)	21.2 (8.8–38.7)	21.1 (9.1–40.2)	21.1 (9.2–39.9)
Liberia	20 to 24	20.4 (8.4–38.1)	20.1 (8.0–37.7)	19.8 (8.1–36.7)	19.7 (8.3–38.1)	19.6 (8.5–37.8)
Liberia	25 to 29	20.9 (8.7–38.9)	20.7 (8.3–38.6)	20.4 (8.4–37.5)	20.2 (8.6–38.9)	20.2 (8.7–38.6)
Liberia	30 to 34	22.6 (9.5–41.4)	22.3 (9.1–41.0)	22.0 (9.2–40.0)	21.8 (9.5–41.4)	21.8 (9.6–41.0)

Supplementary Table S11: Prevalence of male SVAC by age and location for 1990, 2000, 2010, 2020, and 2023						
**Location**	**Age Range**	**1990**	**2000**	**2010**	**2020**	**2023**
Liberia	35 to 39	22.9 (9.7–41.9)	22.7 (9.3–41.6)	22.4 (9.4–40.5)	22.2 (9.7–42.0)	22.2 (9.8–41.6)
Liberia	40 to 44	23.8 (10.2–43.1)	23.6 (9.7–42.8)	23.3 (9.8–41.8)	23.1 (10.1–43.3)	23.1 (10.2–42.9)
Liberia	45 to 49	23.3 (9.9–42.5)	23.1 (9.5–42.2)	22.9 (9.6–41.2)	22.7 (9.9–42.7)	22.7 (10.0–42.3)
Liberia	50 to 54	22.9 (9.7–41.9)	22.7 (9.3–41.6)	22.5 (9.4–40.7)	22.3 (9.7–42.1)	22.3 (9.8–41.8)
Liberia	55 to 59	22.3 (9.4–41.0)	22.1 (9.0–40.7)	21.9 (9.1–39.8)	21.7 (9.4–41.2)	21.7 (9.5–40.9)
Liberia	60 to 64	21.7 (9.1–40.1)	21.5 (8.7–39.8)	21.3 (8.8–38.9)	21.1 (9.1–40.3)	21.1 (9.2–39.9)
Liberia	65 to 69	20.2 (8.4–37.9)	20.1 (8.0–37.7)	19.8 (8.1–36.8)	19.7 (8.4–38.1)	19.7 (8.5–37.8)
Liberia	70 to 74	16.5 (6.6–32.0)	16.3 (6.3–31.7)	16.1 (6.4–30.9)	16.0 (6.6–32.2)	16.0 (6.6–31.9)
Liberia	75 to 79	14.9 (5.9–29.4)	14.8 (5.6–29.2)	14.6 (5.7–28.4)	14.5 (5.9–29.6)	14.5 (5.9–29.3)
Liberia	80 to 84	10.8 (4.1–22.1)	10.7 (3.9–21.9)	10.5 (3.9–21.2)	10.4 (4.1–22.2)	10.4 (4.1–22.0)
Liberia	85 to 89	9.7 (3.6–20.2)	9.6 (3.5–20.0)	9.5 (3.5–19.4)	9.4 (3.6–20.3)	9.4 (3.7–20.1)
Liberia	90 to 94	8.8 (3.3–18.5)	8.7 (3.1–18.3)	8.6 (3.2–17.7)	8.5 (3.3–18.6)	8.5 (3.3–18.4)
Liberia	95 plus	8.7 (3.2–18.3)	8.7 (3.1–18.1)	8.5 (3.1–17.6)	8.5 (3.2–18.4)	8.5 (3.3–18.2)
Liberia	Age-standardized	21.3 (9.0–39.5)	21.1 (8.5–39.2)	20.9 (8.7–38.2)	20.7 (8.9–39.6)	20.7 (9.0–39.2)
Liberia	All age	21.5 (9.0–39.7)	21.3 (8.6–39.4)	21.2 (8.8–38.7)	21.1 (9.1–40.3)	21.1 (9.2–40.0)

Supplementary Table S11: Prevalence of male SVAC by age and location for 1990, 2000, 2010, 2020, and 2023						
**Location**	**Age Range**	**1990**	**2000**	**2010**	**2020**	**2023**
Mali	20 to 24	20.4 (8.4–38.1)	20.1 (8.0–37.7)	19.8 (8.1–36.7)	19.7 (8.3–38.1)	19.6 (8.5–37.8)
Mali	25 to 29	20.9 (8.7–38.9)	20.7 (8.3–38.6)	20.4 (8.4–37.5)	20.2 (8.6–38.9)	20.2 (8.7–38.6)
Mali	30 to 34	22.6 (9.5–41.4)	22.3 (9.1–41.0)	22.0 (9.2–40.0)	21.8 (9.5–41.4)	21.8 (9.6–41.0)
Mali	35 to 39	22.9 (9.7–41.9)	22.7 (9.3–41.6)	22.4 (9.4–40.5)	22.2 (9.7–42.0)	22.2 (9.8–41.6)
Mali	40 to 44	23.8 (10.2–43.1)	23.6 (9.7–42.8)	23.3 (9.8–41.8)	23.1 (10.1–43.3)	23.1 (10.2–42.9)
Mali	45 to 49	23.3 (9.9–42.5)	23.1 (9.5–42.2)	22.9 (9.6–41.2)	22.7 (9.9–42.7)	22.7 (10.0–42.3)
Mali	50 to 54	22.9 (9.7–41.9)	22.7 (9.3–41.6)	22.5 (9.4–40.7)	22.3 (9.7–42.1)	22.3 (9.8–41.8)
Mali	55 to 59	22.3 (9.4–41.0)	22.1 (9.0–40.7)	21.9 (9.1–39.8)	21.7 (9.4–41.2)	21.7 (9.5–40.9)
Mali	60 to 64	21.7 (9.1–40.1)	21.5 (8.7–39.8)	21.3 (8.8–38.9)	21.1 (9.1–40.3)	21.1 (9.2–39.9)
Mali	65 to 69	20.2 (8.4–37.9)	20.1 (8.0–37.7)	19.8 (8.1–36.8)	19.7 (8.4–38.1)	19.7 (8.5–37.8)
Mali	70 to 74	16.5 (6.6–32.0)	16.3 (6.3–31.7)	16.1 (6.4–30.9)	16.0 (6.6–32.2)	16.0 (6.6–31.9)
Mali	75 to 79	14.9 (5.9–29.4)	14.8 (5.6–29.2)	14.6 (5.7–28.4)	14.5 (5.9–29.6)	14.5 (5.9–29.3)
Mali	80 to 84	10.8 (4.1–22.1)	10.7 (3.9–21.9)	10.5 (3.9–21.2)	10.4 (4.1–22.2)	10.4 (4.1–22.0)
Mali	85 to 89	9.7 (3.6–20.2)	9.6 (3.5–20.0)	9.5 (3.5–19.4)	9.4 (3.6–20.3)	9.4 (3.7–20.1)
Mali	90 to 94	8.8 (3.3–18.5)	8.7 (3.1–18.3)	8.6 (3.2–17.7)	8.5 (3.3–18.6)	8.5 (3.3–18.4)

Supplementary Table S11: Prevalence of male SVAC by age and location for 1990, 2000, 2010, 2020, and 2023						
**Location**	**Age Range**	**1990**	**2000**	**2010**	**2020**	**2023**
Mali	95 plus	8.7 (3.2–18.3)	8.7 (3.1–18.1)	8.5 (3.1–17.6)	8.5 (3.2–18.4)	8.5 (3.3–18.2)
Mali	Age-standardized	21.3 (9.0–39.5)	21.1 (8.5–39.2)	20.9 (8.7–38.2)	20.7 (8.9–39.6)	20.7 (9.0–39.2)
Mali	All age	21.7 (9.1–40.0)	21.4 (8.7–39.7)	21.1 (8.8–38.6)	21.0 (9.0–40.1)	21.0 (9.2–39.7)
Mauritania	20 to 24	20.4 (8.4–38.1)	20.1 (8.0–37.7)	19.8 (8.1–36.7)	19.7 (8.3–38.1)	19.6 (8.5–37.8)
Mauritania	25 to 29	20.9 (8.7–38.9)	20.7 (8.3–38.6)	20.4 (8.4–37.5)	20.2 (8.6–38.9)	20.2 (8.7–38.6)
Mauritania	30 to 34	22.6 (9.5–41.4)	22.3 (9.1–41.0)	22.0 (9.2–40.0)	21.8 (9.5–41.4)	21.8 (9.6–41.0)
Mauritania	35 to 39	22.9 (9.7–41.9)	22.7 (9.3–41.6)	22.4 (9.4–40.5)	22.2 (9.7–42.0)	22.2 (9.8–41.6)
Mauritania	40 to 44	23.8 (10.2–43.1)	23.6 (9.7–42.8)	23.3 (9.8–41.8)	23.1 (10.1–43.3)	23.1 (10.2–42.9)
Mauritania	45 to 49	23.3 (9.9–42.5)	23.1 (9.5–42.2)	22.9 (9.6–41.2)	22.7 (9.9–42.7)	22.7 (10.0–42.3)
Mauritania	50 to 54	22.9 (9.7–41.9)	22.7 (9.3–41.6)	22.5 (9.4–40.7)	22.3 (9.7–42.1)	22.3 (9.8–41.8)
Mauritania	55 to 59	22.3 (9.4–41.0)	22.1 (9.0–40.7)	21.9 (9.1–39.8)	21.7 (9.4–41.2)	21.7 (9.5–40.9)
Mauritania	60 to 64	21.7 (9.1–40.1)	21.5 (8.7–39.8)	21.3 (8.8–38.9)	21.1 (9.1–40.3)	21.1 (9.2–39.9)
Mauritania	65 to 69	20.2 (8.4–37.9)	20.1 (8.0–37.7)	19.8 (8.1–36.8)	19.7 (8.4–38.1)	19.7 (8.5–37.8)
Mauritania	70 to 74	16.5 (6.6–32.0)	16.3 (6.3–31.7)	16.1 (6.4–30.9)	16.0 (6.6–32.2)	16.0 (6.6–31.9)
Mauritania	75 to 79	14.9 (5.9–29.4)	14.8 (5.6–29.2)	14.6 (5.7–28.4)	14.5 (5.9–29.6)	14.5 (5.9–29.3)

Supplementary Table S11: Prevalence of male SVAC by age and location for 1990, 2000, 2010, 2020, and 2023						
**Location**	**Age Range**	**1990**	**2000**	**2010**	**2020**	**2023**
Mauritania	80 to 84	10.8 (4.1–22.1)	10.7 (3.9–21.9)	10.5 (3.9–21.2)	10.4 (4.1–22.2)	10.4 (4.1–22.0)
Mauritania	85 to 89	9.7 (3.6–20.2)	9.6 (3.5–20.0)	9.5 (3.5–19.4)	9.4 (3.6–20.3)	9.4 (3.7–20.1)
Mauritania	90 to 94	8.8 (3.3–18.5)	8.7 (3.1–18.3)	8.6 (3.2–17.7)	8.5 (3.3–18.6)	8.5 (3.3–18.4)
Mauritania	95 plus	8.7 (3.2–18.3)	8.7 (3.1–18.1)	8.5 (3.1–17.6)	8.5 (3.2–18.4)	8.5 (3.3–18.2)
Mauritania	Age-standardized	21.3 (9.0–39.5)	21.1 (8.5–39.2)	20.9 (8.7–38.2)	20.7 (8.9–39.6)	20.7 (9.0–39.2)
Mauritania	All age	21.6 (9.1–39.9)	21.4 (8.6–39.6)	21.1 (8.7–38.5)	20.9 (9.0–39.9)	20.9 (9.1–39.5)
Niger	20 to 24	17.0 (6.8–32.9)	16.9 (6.5–32.7)	16.8 (6.7–32.0)	16.9 (7.0–33.6)	16.8 (7.1–33.3)
Niger	25 to 29	17.5 (7.1–33.7)	17.4 (6.8–33.5)	17.3 (6.9–32.8)	17.4 (7.2–34.5)	17.3 (7.3–34.1)
Niger	30 to 34	19.0 (7.8–36.1)	18.9 (7.4–35.9)	18.8 (7.6–35.2)	18.8 (7.9–36.8)	18.8 (8.0–36.5)
Niger	35 to 39	19.4 (7.9–36.6)	19.3 (7.6–36.4)	19.1 (7.8–35.7)	19.1 (8.1–37.3)	19.1 (8.2–36.9)
Niger	40 to 44	20.1 (8.3–37.8)	20.0 (8.0–37.6)	19.9 (8.1–36.8)	19.9 (8.5–38.5)	19.9 (8.6–38.1)
Niger	45 to 49	19.7 (8.1–37.2)	19.6 (7.8–37.0)	19.5 (8.0–36.3)	19.5 (8.3–37.9)	19.5 (8.3–37.6)
Niger	50 to 54	19.4 (8.0–36.7)	19.3 (7.6–36.5)	19.2 (7.8–35.8)	19.2 (8.1–37.4)	19.2 (8.2–37.0)
Niger	55 to 59	18.9 (7.7–35.9)	18.8 (7.4–35.7)	18.7 (7.6–35.0)	18.7 (7.9–36.6)	18.7 (7.9–36.2)
Niger	60 to 64	18.4 (7.5–35.1)	18.3 (7.1–34.9)	18.2 (7.3–34.2)	18.1 (7.6–35.7)	18.1 (7.7–35.4)

Supplementary Table S11: Prevalence of male SVAC by age and location for 1990, 2000, 2010, 2020, and 2023						
**Location**	**Age Range**	**1990**	**2000**	**2010**	**2020**	**2023**
Niger	65 to 69	17.1 (6.9–33.1)	17.1 (6.6–32.9)	16.9 (6.7–32.2)	16.9 (7.0–33.8)	16.9 (7.1–33.4)
Niger	70 to 74	13.8 (5.4–27.6)	13.8 (5.1–27.4)	13.7 (5.3–26.8)	13.7 (5.5–28.2)	13.7 (5.6–27.9)
Niger	75 to 79	12.5 (4.8–25.3)	12.4 (4.6–25.1)	12.3 (4.7–24.5)	12.3 (4.9–25.8)	12.3 (5.0–25.5)
Niger	80 to 84	8.9 (3.3–18.7)	8.9 (3.2–18.5)	8.8 (3.2–18.1)	8.8 (3.4–19.1)	8.8 (3.4–18.9)
Niger	85 to 89	8.1 (3.0–17.0)	8.0 (2.8–16.9)	7.9 (2.9–16.4)	7.9 (3.0–17.4)	7.9 (3.1–17.2)
Niger	90 to 94	7.3 (2.7–15.5)	7.2 (2.5–15.4)	7.2 (2.6–15.0)	7.2 (2.7–15.9)	7.2 (2.8–15.7)
Niger	95 plus	7.2 (2.6–15.4)	7.2 (2.5–15.3)	7.1 (2.6–14.8)	7.1 (2.7–15.8)	7.1 (2.7–15.6)
Niger	Age-standardized	18.0 (7.3–34.4)	17.9 (7.0–34.2)	17.8 (7.2–33.5)	17.8 (7.5–35.1)	17.8 (7.5–34.7)
Niger	All age	18.3 (7.5–35.0)	18.2 (7.1–34.8)	18.1 (7.3–34.0)	18.0 (7.5–35.4)	17.9 (7.6–35.0)
Nigeria	20 to 24	27.7 (17.6–40.2)	25.2 (15.7–36.7)	20.6 (13.3–29.3)	18.9 (10.8–30.0)	19.0 (9.9–31.5)
Nigeria	25 to 29	27.9 (18.1–39.6)	25.7 (16.7–35.9)	21.2 (13.5–30.4)	19.0 (11.6–29.7)	19.0 (10.5–30.2)
Nigeria	30 to 34	28.6 (17.4–41.5)	27.9 (18.9–38.4)	26.0 (17.7–35.6)	25.0 (14.8–38.0)	25.1 (13.7–39.5)
Nigeria	35 to 39	28.8 (16.5–43.6)	27.8 (18.6–38.3)	25.3 (17.3–34.3)	24.1 (14.2–36.2)	24.1 (12.6–37.8)
Nigeria	40 to 44	29.1 (15.5–45.7)	29.1 (19.3–40.3)	29.8 (21.8–38.9)	30.4 (19.5–43.0)	30.5 (17.7–45.2)
Nigeria	45 to 49	28.7 (14.3–46.8)	28.3 (17.7–40.4)	28.1 (19.7–37.5)	28.1 (18.6–39.7)	28.2 (17.2–42.2)

Supplementary Table S11: Prevalence of male SVAC by age and location for 1990, 2000, 2010, 2020, and 2023						
**Location**	**Age Range**	**1990**	**2000**	**2010**	**2020**	**2023**
Nigeria	50 to 54	27.6 (13.7–45.3)	26.7 (16.1–39.2)	26.1 (18.3–35.0)	26.4 (16.4–37.9)	26.5 (15.5–39.9)
Nigeria	55 to 59	28.3 (14.0–46.7)	28.9 (16.8–43.3)	29.8 (21.0–40.3)	30.1 (19.7–42.4)	30.1 (17.9–44.3)
Nigeria	60 to 64	27.6 (13.6–45.7)	27.8 (16.2–42.1)	28.0 (19.0–38.6)	28.0 (17.8–40.1)	28.0 (16.3–41.7)
Nigeria	65 to 69	25.4 (11.0–45.4)	25.3 (10.6–45.2)	25.3 (10.9–44.6)	25.3 (11.3–46.5)	25.4 (11.3–47.2)
Nigeria	70 to 74	20.9 (8.7–39.0)	20.9 (8.4–38.9)	20.8 (8.6–38.2)	20.9 (9.0–40.1)	20.9 (8.9–40.7)
Nigeria	75 to 79	19.1 (7.8–36.2)	19.0 (7.5–36.1)	19.0 (7.7–35.4)	19.0 (8.0–37.2)	19.1 (8.0–37.7)
Nigeria	80 to 84	14.0 (5.4–27.8)	13.9 (5.2–27.7)	13.9 (5.4–27.1)	13.9 (5.6–28.7)	14.0 (5.6–29.3)
Nigeria	85 to 89	12.7 (4.9–25.6)	12.6 (4.7–25.4)	12.6 (4.8–24.9)	12.6 (5.0–26.4)	12.7 (5.0–26.9)
Nigeria	90 to 94	11.6 (4.4–23.5)	11.5 (4.2–23.4)	11.5 (4.3–22.9)	11.5 (4.5–24.3)	11.6 (4.5–24.8)
Nigeria	95 plus	11.5 (4.4–23.4)	11.4 (4.2–23.2)	11.4 (4.3–22.8)	11.4 (4.5–24.2)	11.5 (4.4–24.7)
Nigeria	Age-standardized	27.2 (15.3–42.0)	26.3 (16.4–38.3)	24.8 (16.5–34.6)	24.2 (15.1–36.0)	24.2 (13.5–38.2)
Nigeria	All age	27.8 (16.1–42.1)	26.7 (16.9–38.1)	24.5 (16.4–33.9)	23.6 (14.6–35.4)	23.6 (13.2–37.2)
São Tomé and Príncipe	20 to 24	13.0 (6.0–23.6)	12.5 (7.3–19.5)	11.8 (7.3–17.4)	11.1 (6.7–16.9)	11.0 (6.3–18.1)
São Tomé and Príncipe	25 to 29	13.1 (5.5–24.9)	13.0 (7.0–21.9)	13.2 (8.2–19.6)	13.8 (8.8–20.5)	13.9 (8.5–21.9)
São Tomé and Príncipe	30 to 34	13.8 (5.6–26.4)	13.4 (6.3–24.1)	12.5 (7.2–19.6)	11.8 (7.0–18.1)	11.8 (6.6–18.9)

Supplementary Table S11: Prevalence of male SVAC by age and location for 1990, 2000, 2010, 2020, and 2023						
**Location**	**Age Range**	**1990**	**2000**	**2010**	**2020**	**2023**
São Tomé and Príncipe	35 to 39	13.8 (5.6–27.0)	13.6 (5.9–25.5)	13.0 (6.9–21.5)	12.5 (7.2–19.6)	12.5 (6.9–20.2)
São Tomé and Príncipe	40 to 44	14.4 (5.8–28.4)	14.5 (6.0–27.2)	14.5 (7.3–24.6)	14.3 (8.6–22.1)	14.3 (8.3–22.5)
São Tomé and Príncipe	45 to 49	13.4 (5.3–26.6)	12.9 (5.2–24.9)	12.1 (5.8–21.7)	11.7 (6.3–19.8)	11.7 (6.1–19.9)
São Tomé and Príncipe	50 to 54	12.7 (5.0–25.5)	12.0 (4.8–23.5)	11.0 (5.2–20.0)	10.4 (5.5–18.2)	10.5 (5.3–18.3)
São Tomé and Príncipe	55 to 59	12.4 (4.7–24.6)	11.9 (4.6–24.2)	11.3 (4.6–21.9)	10.9 (4.9–21.5)	11.0 (4.8–21.6)
São Tomé and Príncipe	60 to 64	13.2 (5.2–26.4)	14.0 (5.6–27.2)	15.1 (7.2–26.8)	15.6 (8.4–26.7)	15.5 (8.0–26.3)
São Tomé and Príncipe	65 to 69	11.3 (4.3–23.0)	11.0 (4.1–22.6)	10.7 (4.2–21.2)	10.5 (4.4–21.3)	10.6 (4.4–21.5)
São Tomé and Príncipe	70 to 74	9.1 (3.4–19.0)	9.1 (3.2–18.9)	9.0 (3.3–18.5)	9.0 (3.5–19.6)	9.0 (3.5–19.4)
São Tomé and Príncipe	75 to 79	8.2 (3.0–17.2)	8.2 (2.9–17.2)	8.1 (3.0–16.8)	8.1 (3.1–17.8)	8.1 (3.1–17.6)
São Tomé and Príncipe	80 to 84	5.7 (2.1–12.4)	5.7 (2.0–12.3)	5.7 (2.0–12.0)	5.7 (2.1–12.8)	5.7 (2.2–12.6)
São Tomé and Príncipe	85 to 89	5.2 (1.8–11.2)	5.1 (1.8–11.1)	5.1 (1.8–10.9)	5.1 (1.9–11.6)	5.1 (1.9–11.4)
São Tomé and Príncipe	90 to 94	4.7 (1.7–10.1)	4.6 (1.6–10.1)	4.6 (1.6– 9.9)	4.6 (1.7–10.5)	4.6 (1.7–10.3)
São Tomé and Príncipe	95 plus	4.6 (1.6–10.0)	4.6 (1.6–10.0)	4.6 (1.6– 9.8)	4.6 (1.7–10.4)	4.6 (1.7–10.3)
São Tomé and Príncipe	Age-standardized	12.7 (5.3–24.4)	12.5 (5.7–22.9)	12.1 (6.5–20.0)	11.8 (6.8–19.3)	11.9 (6.6–19.6)
São Tomé and Príncipe	All age	12.9 (5.5–24.6)	12.7 (6.2–22.3)	12.4 (7.2–19.5)	12.1 (7.1–19.4)	12.2 (6.9–19.7)

Supplementary Table S11: Prevalence of male SVAC by age and location for 1990, 2000, 2010, 2020, and 2023						
**Location**	**Age Range**	**1990**	**2000**	**2010**	**2020**	**2023**
Senegal	20 to 24	20.4 (8.4–38.1)	20.1 (8.0–37.7)	19.8 (8.1–36.7)	19.7 (8.3–38.1)	19.6 (8.5–37.8)
Senegal	25 to 29	20.9 (8.7–38.9)	20.7 (8.3–38.6)	20.4 (8.4–37.5)	20.2 (8.6–38.9)	20.2 (8.7–38.6)
Senegal	30 to 34	22.6 (9.5–41.4)	22.3 (9.1–41.0)	22.0 (9.2–40.0)	21.8 (9.5–41.4)	21.8 (9.6–41.0)
Senegal	35 to 39	22.9 (9.7–41.9)	22.7 (9.3–41.6)	22.4 (9.4–40.5)	22.2 (9.7–42.0)	22.2 (9.8–41.6)
Senegal	40 to 44	23.8 (10.2–43.1)	23.6 (9.7–42.8)	23.3 (9.8–41.8)	23.1 (10.1–43.3)	23.1 (10.2–42.9)
Senegal	45 to 49	23.3 (9.9–42.5)	23.1 (9.5–42.2)	22.9 (9.6–41.2)	22.7 (9.9–42.7)	22.7 (10.0–42.3)
Senegal	50 to 54	22.9 (9.7–41.9)	22.7 (9.3–41.6)	22.5 (9.4–40.7)	22.3 (9.7–42.1)	22.3 (9.8–41.8)
Senegal	55 to 59	22.3 (9.4–41.0)	22.1 (9.0–40.7)	21.9 (9.1–39.8)	21.7 (9.4–41.2)	21.7 (9.5–40.9)
Senegal	60 to 64	21.7 (9.1–40.1)	21.5 (8.7–39.8)	21.3 (8.8–38.9)	21.1 (9.1–40.3)	21.1 (9.2–39.9)
Senegal	65 to 69	20.2 (8.4–37.9)	20.1 (8.0–37.7)	19.8 (8.1–36.8)	19.7 (8.4–38.1)	19.7 (8.5–37.8)
Senegal	70 to 74	16.5 (6.6–32.0)	16.3 (6.3–31.7)	16.1 (6.4–30.9)	16.0 (6.6–32.2)	16.0 (6.6–31.9)
Senegal	75 to 79	14.9 (5.9–29.4)	14.8 (5.6–29.2)	14.6 (5.7–28.4)	14.5 (5.9–29.6)	14.5 (5.9–29.3)
Senegal	80 to 84	10.8 (4.1–22.1)	10.7 (3.9–21.9)	10.5 (3.9–21.2)	10.4 (4.1–22.2)	10.4 (4.1–22.0)
Senegal	85 to 89	9.7 (3.6–20.2)	9.6 (3.5–20.0)	9.5 (3.5–19.4)	9.4 (3.6–20.3)	9.4 (3.7–20.1)
Senegal	90 to 94	8.8 (3.3–18.5)	8.7 (3.1–18.3)	8.6 (3.2–17.7)	8.5 (3.3–18.6)	8.5 (3.3–18.4)

Supplementary Table S11: Prevalence of male SVAC by age and location for 1990, 2000, 2010, 2020, and 2023						
**Location**	**Age Range**	**1990**	**2000**	**2010**	**2020**	**2023**
Senegal	95 plus	8.7 (3.2–18.3)	8.7 (3.1–18.1)	8.5 (3.1–17.6)	8.5 (3.2–18.4)	8.5 (3.3–18.2)
Senegal	Age-standardized	21.3 (9.0–39.5)	21.1 (8.5–39.2)	20.9 (8.7–38.2)	20.7 (8.9–39.6)	20.7 (9.0–39.2)
Senegal	All age	21.5 (9.0–39.8)	21.3 (8.6–39.5)	21.1 (8.7–38.5)	20.9 (9.0–40.0)	20.9 (9.1–39.6)
Sierra Leone	20 to 24	20.4 (8.4–38.1)	20.1 (8.0–37.7)	19.8 (8.1–36.7)	19.7 (8.3–38.1)	19.6 (8.5–37.8)
Sierra Leone	25 to 29	20.9 (8.7–38.9)	20.7 (8.3–38.6)	20.4 (8.4–37.5)	20.2 (8.6–38.9)	20.2 (8.7–38.6)
Sierra Leone	30 to 34	22.6 (9.5–41.4)	22.3 (9.1–41.0)	22.0 (9.2–40.0)	21.8 (9.5–41.4)	21.8 (9.6–41.0)
Sierra Leone	35 to 39	22.9 (9.7–41.9)	22.7 (9.3–41.6)	22.4 (9.4–40.5)	22.2 (9.7–42.0)	22.2 (9.8–41.6)
Sierra Leone	40 to 44	23.8 (10.2–43.1)	23.6 (9.7–42.8)	23.3 (9.8–41.8)	23.1 (10.1–43.3)	23.1 (10.2–42.9)
Sierra Leone	45 to 49	23.3 (9.9–42.5)	23.1 (9.5–42.2)	22.9 (9.6–41.2)	22.7 (9.9–42.7)	22.7 (10.0–42.3)
Sierra Leone	50 to 54	22.9 (9.7–41.9)	22.7 (9.3–41.6)	22.5 (9.4–40.7)	22.3 (9.7–42.1)	22.3 (9.8–41.8)
Sierra Leone	55 to 59	22.3 (9.4–41.0)	22.1 (9.0–40.7)	21.9 (9.1–39.8)	21.7 (9.4–41.2)	21.7 (9.5–40.9)
Sierra Leone	60 to 64	21.7 (9.1–40.1)	21.5 (8.7–39.8)	21.3 (8.8–38.9)	21.1 (9.1–40.3)	21.1 (9.2–39.9)
Sierra Leone	65 to 69	20.2 (8.4–37.9)	20.1 (8.0–37.7)	19.8 (8.1–36.8)	19.7 (8.4–38.1)	19.7 (8.5–37.8)
Sierra Leone	70 to 74	16.5 (6.6–32.0)	16.3 (6.3–31.7)	16.1 (6.4–30.9)	16.0 (6.6–32.2)	16.0 (6.6–31.9)
Sierra Leone	75 to 79	14.9 (5.9–29.4)	14.8 (5.6–29.2)	14.6 (5.7–28.4)	14.5 (5.9–29.6)	14.5 (5.9–29.3)

Supplementary Table S11: Prevalence of male SVAC by age and location for 1990, 2000, 2010, 2020, and 2023						
**Location**	**Age Range**	**1990**	**2000**	**2010**	**2020**	**2023**
Sierra Leone	80 to 84	10.8 (4.1–22.1)	10.7 (3.9–21.9)	10.5 (3.9–21.2)	10.4 (4.1–22.2)	10.4 (4.1–22.0)
Sierra Leone	85 to 89	9.7 (3.6–20.2)	9.6 (3.5–20.0)	9.5 (3.5–19.4)	9.4 (3.6–20.3)	9.4 (3.7–20.1)
Sierra Leone	90 to 94	8.8 (3.3–18.5)	8.7 (3.1–18.3)	8.6 (3.2–17.7)	8.5 (3.3–18.6)	8.5 (3.3–18.4)
Sierra Leone	95 plus	8.7 (3.2–18.3)	8.7 (3.1–18.1)	8.5 (3.1–17.6)	8.5 (3.2–18.4)	8.5 (3.3–18.2)
Sierra Leone	Age-standardized	21.3 (9.0–39.5)	21.1 (8.5–39.2)	20.9 (8.7–38.2)	20.7 (8.9–39.6)	20.7 (9.0–39.2)
Sierra Leone	All age	21.6 (9.1–39.9)	21.5 (8.7–39.7)	21.1 (8.8–38.6)	21.0 (9.0–40.1)	21.0 (9.2–39.8)
Togo	20 to 24	20.4 (8.4–38.1)	20.1 (8.0–37.7)	19.8 (8.1–36.7)	19.7 (8.3–38.1)	19.6 (8.5–37.8)
Togo	25 to 29	20.9 (8.7–38.9)	20.7 (8.3–38.6)	20.4 (8.4–37.5)	20.2 (8.6–38.9)	20.2 (8.7–38.6)
Togo	30 to 34	22.6 (9.5–41.4)	22.3 (9.1–41.0)	22.0 (9.2–40.0)	21.8 (9.5–41.4)	21.8 (9.6–41.0)
Togo	35 to 39	22.9 (9.7–41.9)	22.7 (9.3–41.6)	22.4 (9.4–40.5)	22.2 (9.7–42.0)	22.2 (9.8–41.6)
Togo	40 to 44	23.8 (10.2–43.1)	23.6 (9.7–42.8)	23.3 (9.8–41.8)	23.1 (10.1–43.3)	23.1 (10.2–42.9)
Togo	45 to 49	23.3 (9.9–42.5)	23.1 (9.5–42.2)	22.9 (9.6–41.2)	22.7 (9.9–42.7)	22.7 (10.0–42.3)
Togo	50 to 54	22.9 (9.7–41.9)	22.7 (9.3–41.6)	22.5 (9.4–40.7)	22.3 (9.7–42.1)	22.3 (9.8–41.8)
Togo	55 to 59	22.3 (9.4–41.0)	22.1 (9.0–40.7)	21.9 (9.1–39.8)	21.7 (9.4–41.2)	21.7 (9.5–40.9)
Togo	60 to 64	21.7 (9.1–40.1)	21.5 (8.7–39.8)	21.3 (8.8–38.9)	21.1 (9.1–40.3)	21.1 (9.2–39.9)

Supplementary Table S11: Prevalence of male SVAC by age and location for 1990, 2000, 2010, 2020, and 2023						
**Location**	**Age Range**	**1990**	**2000**	**2010**	**2020**	**2023**
Togo	65 to 69	20.2 (8.4–37.9)	20.1 (8.0–37.7)	19.8 (8.1–36.8)	19.7 (8.4–38.1)	19.7 (8.5–37.8)
Togo	70 to 74	16.5 (6.6–32.0)	16.3 (6.3–31.7)	16.1 (6.4–30.9)	16.0 (6.6–32.2)	16.0 (6.6–31.9)
Togo	75 to 79	14.9 (5.9–29.4)	14.8 (5.6–29.2)	14.6 (5.7–28.4)	14.5 (5.9–29.6)	14.5 (5.9–29.3)
Togo	80 to 84	10.8 (4.1–22.1)	10.7 (3.9–21.9)	10.5 (3.9–21.2)	10.4 (4.1–22.2)	10.4 (4.1–22.0)
Togo	85 to 89	9.7 (3.6–20.2)	9.6 (3.5–20.0)	9.5 (3.5–19.4)	9.4 (3.6–20.3)	9.4 (3.7–20.1)
Togo	90 to 94	8.8 (3.3–18.5)	8.7 (3.1–18.3)	8.6 (3.2–17.7)	8.5 (3.3–18.6)	8.5 (3.3–18.4)
Togo	95 plus	8.7 (3.2–18.3)	8.7 (3.1–18.1)	8.5 (3.1–17.6)	8.5 (3.2–18.4)	8.5 (3.3–18.2)
Togo	Age-standardized	21.3 (9.0–39.5)	21.1 (8.5–39.2)	20.9 (8.7–38.2)	20.7 (8.9–39.6)	20.7 (9.0–39.2)
Togo	All age	21.6 (9.1–40.0)	21.5 (8.7–39.8)	21.3 (8.9–38.9)	21.2 (9.1–40.4)	21.1 (9.2–40.0)

Section 7.2 Additional prevalence figures

Figure S7.2.1A

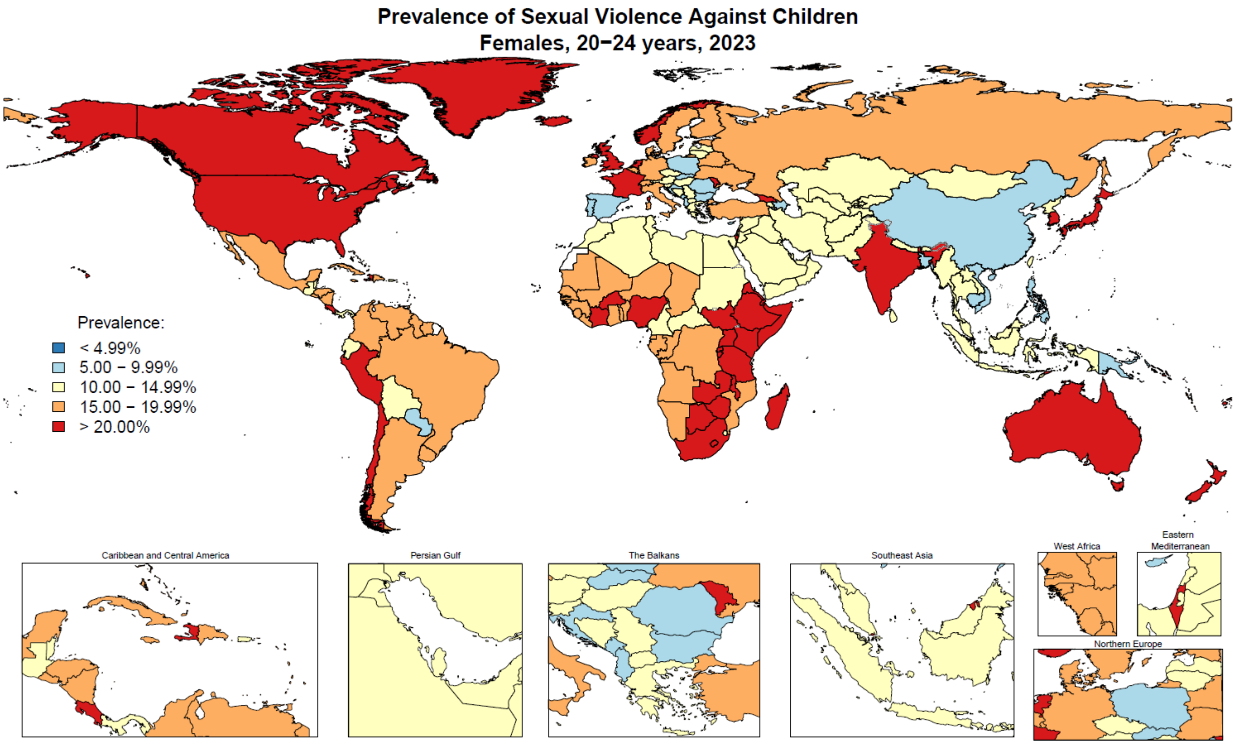

Figure S7.2.1B

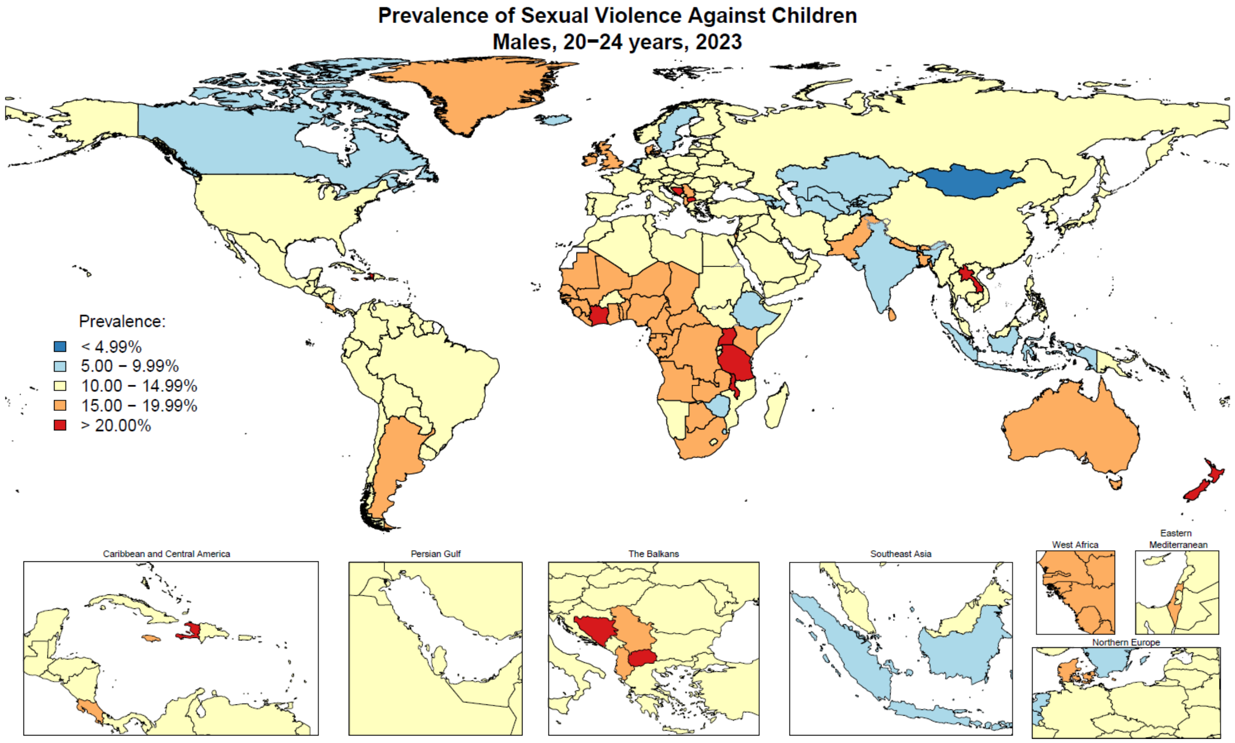

Section 7.3 Trends in SVAC prevalence

Figure S7.3.1A

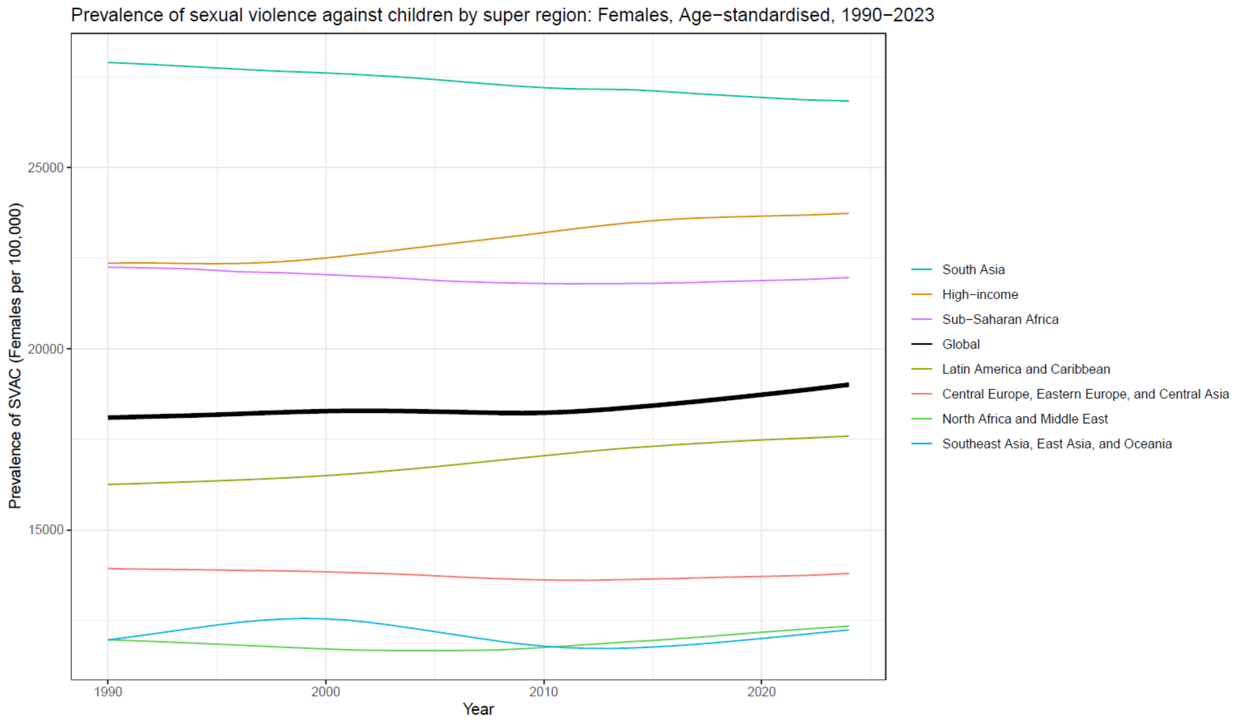

Figure S7.3.1B

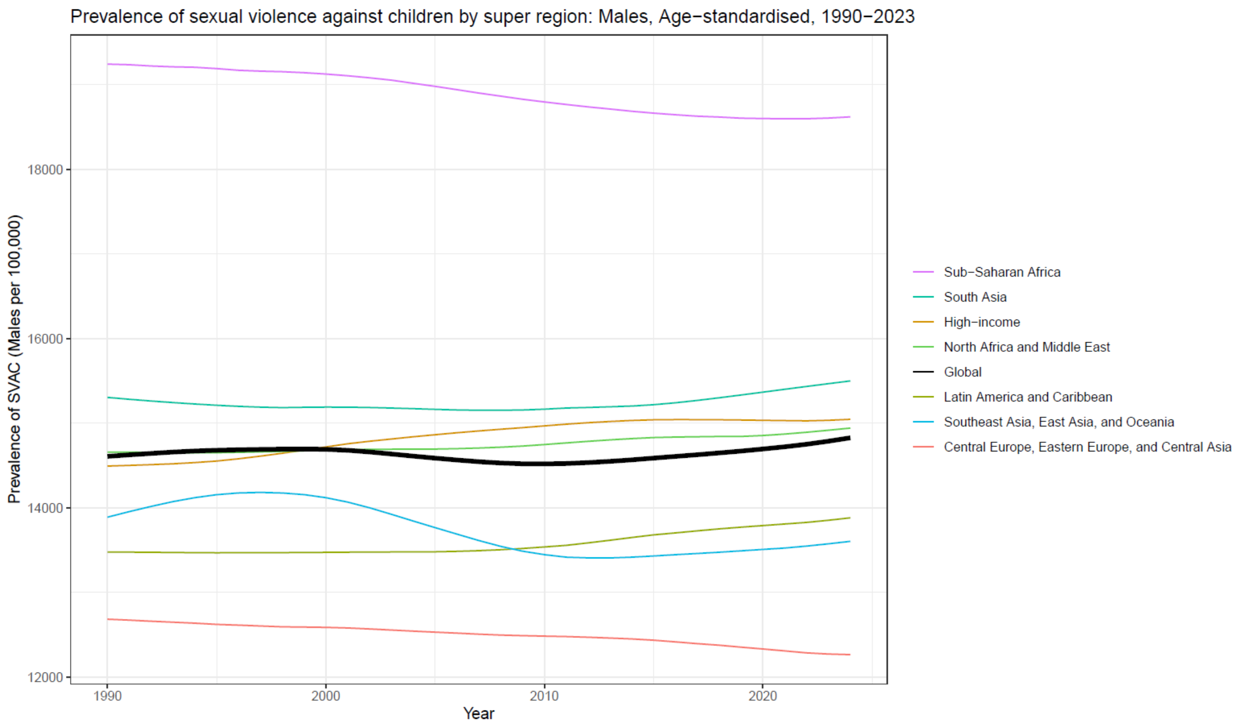

Figure S7.3.3A

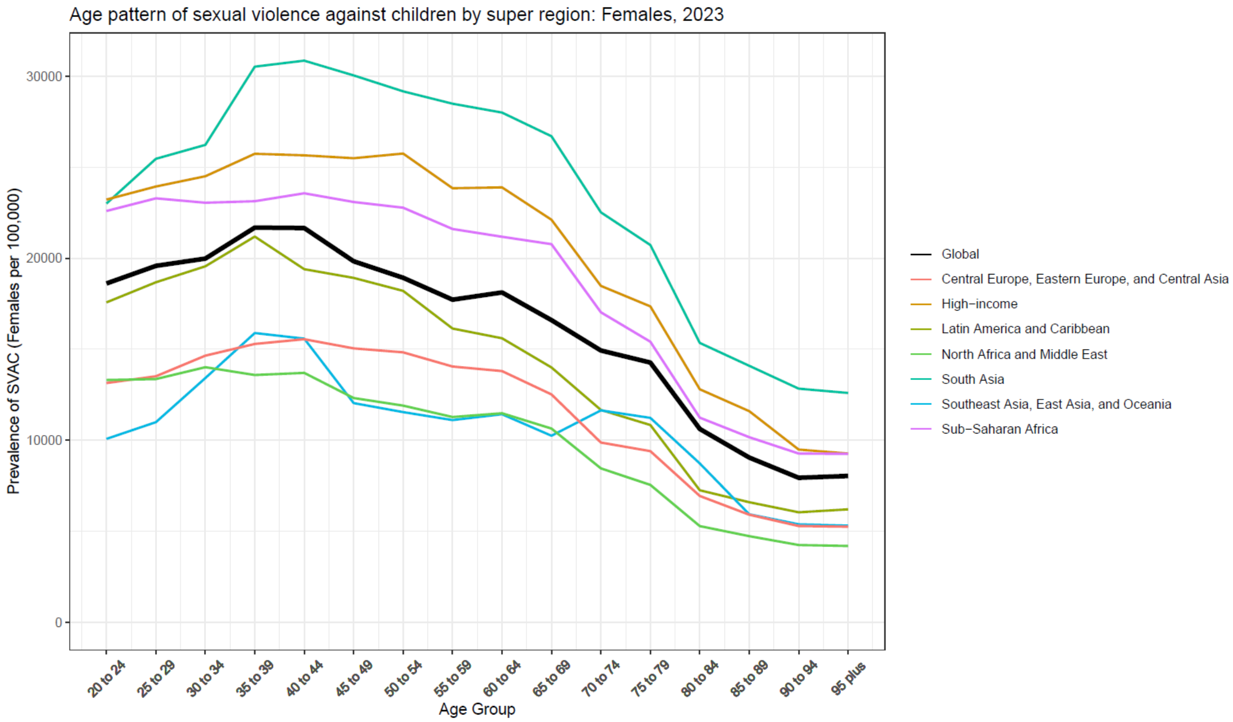

Figure S7.3.3B

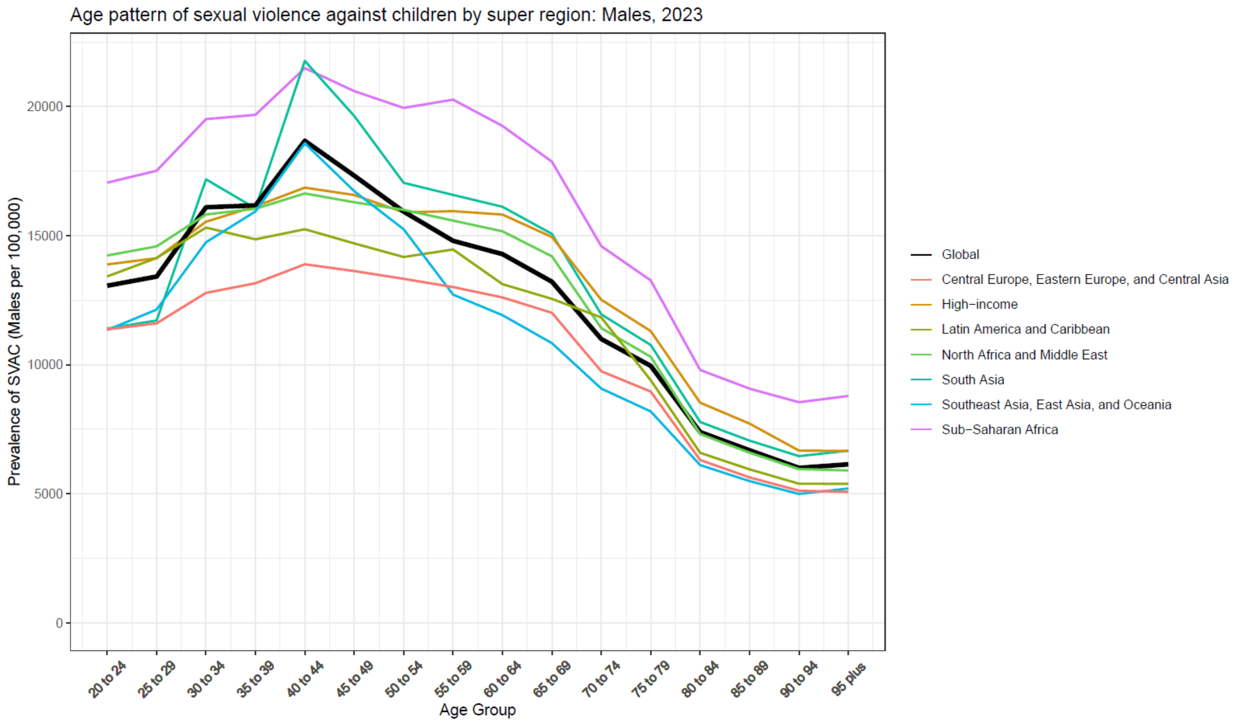

Section 8. Data Sources

Supplementary Table S12. Data sources included in the prevalence analysis

Supplementary Table S12. Data sources included in the prevalence analysis		
Year	Title	Citation
Afghanistan		
2015	Afghanistan Demographic and Health Survey 2015-2016	Central Statistics Organization (Afghanistan), ICF International, Ministry of Public Health (Afghanistan). Afghanistan Demographic and Health Survey 2015-2016. Fairfax, United States of America: ICF International, 2017.
Albania		
2002	Albania Reproductive Health Survey 2002	Albania Institute of Public Health (IPH), Ministry of Health (Albania), National Institute of Statistics (Albania), and Centers for Disease Control and Prevention. (2005) Albania Reproductive Health Survey 2001. Atlanta, United States: Centers for Disease Control and Prevention (CDC).
2018	Albania National Violence Against Women Survey 2018	Albania Institute of Statistics (INSTAT), United Nations Development Programme (UNDP), United Nations Entity for Gender Equality and the Empowerment of Women (UN Women). Albania National Violence Against Women Survey 2018.
2011	Albania Balkan Epidemiological Study on Child Abuse and Neglect 2011	Children's Human Rights Centre of Albania (CRCA), Institute of Child Health (Greece). Albania Balkan Epidemiological Study on Child Abuse and Neglect 2011.
American Samoa		
2017	American Samoa Adult Hybrid Survey 2017-2018	American Samoa Department of Human and Social Services, Department of Health (American Samoa), Pacific Islands Health Officers Association (PIHOA). American Samoa Adult Hybrid Survey 2017-2018.
Argentina		
2003	Argentina - Buenos Aires Gender, Alcohol and Culture: An International Study (GENACIS) 2003	Aarhus University, Addiction Switzerland Research Institute, Alcohol Research Group, Public Health Institute, Centre for Addiction and Mental Health (Canada), Centre for Alcohol Policy Research, Turning Point Alcohol and Drug Centre (Australia), Kettil Bruun Society for Social and Epidemiological Research on Alcohol, University of North Dakota. Argentina - Buenos Aires Gender, Alcohol and Culture: An International Study (GENACIS) 2003.
Armenia		
2015	Armenia Demographic and Health Survey 2015-2016	ICF International, Ministry of Health (Armenia), National Statistical Service of the Republic of Armenia. Armenia Demographic and Health Survey 2015-2016. Fairfax, United States of America: ICF International, 2017.

Supplementary Table S12. Data sources included in the prevalence analysis		
Year	Title	Citation
2021	Armenia Survey on Domestic Violence Against Women 2021	International Center for Human Development (ICHD), Statistical Committee of the Republic of Armenia. Armenia Survey on Domestic Violence Against Women 2021.
2008	Armenia Nationwide Survey on Violence Against Women 2008	National Statistical Service of the Republic of Armenia, United Nations Population Fund (UNFPA). Armenia Nationwide Survey on Violence Against Women 2008.
Australia		
2016	Australia Personal Safety Survey 2016-2017	Australian Bureau of Statistics, Department of Social Services (Australia). Australia Personal Safety Survey 2016-2017.
2005	Australia Personal Safety Survey 2005	Australian Bureau of Statistics. Australia Personal Safety Survey 2005.
1995	Sexual Abuse and Suicidal Behavior: A Model Constructed from a Large Community Sample of Adolescents	Bergen HA, Martin G, Richardson AS, Allison S, Roeger L. Sexual Abuse and Suicidal Behavior: A Model Constructed from a Large Community Sample of Adolescents. J Am Acad Child Adolesc Psychiatry. 2003; 42(11): 1301-9.
2000	Is Child Sexual Abuse Declining? Evidence from a Population-Based Survey of Men and Women in Australia	Dunne MP, Purdie DM, Cook MD, Boyle FM, Najman JM. Is child sexual abuse declining? Evidence from a population-based survey of men and women in Australia. Child Abuse Negl. 2003; 27(2): 141-52.
2002	International Violence Against Women Surveys Data 2002-2005	European Institute for Crime Prevention and Control, affiliated with the United Nations (HEUNI), United Nations Office on Drugs and Crime (UNODC), Statistics Canada, United Nations Interregional Crime and Justice Research Institute (UNICRI). International Violence Against Women Surveys (IVAWS) Data 2002-2005. As provided by the Global Burden of Disease Child Sexual Abuse and Intimate Partner Violence Expert Group. [Unpublished].
1998	International Violence Against Women Surveys Data 2002-2005	European Institute for Crime Prevention and Control, affiliated with the United Nations (HEUNI), United Nations Office on Drugs and Crime (UNODC), Statistics Canada, United Nations Interregional Crime and Justice Research Institute (UNICRI). International Violence Against Women Surveys (IVAWS) Data 2002-2005. As provided by the Global Burden of Disease Child Sexual Abuse and Intimate Partner Violence Expert Group. [Unpublished].
2003	Violence Against Women: An International Perspective	Johnson H, Ollus N, Nevala S. Violence Against Women: An International Perspective. New York City, United States: Springer; 2008.
2021	The Prevalence of Child Maltreatment in Australia: Findings from a National Survey	Mathews B, Pacella R, Scott JG, Finkelhor D, Meinck F, Higgins DJ, Erskine HE, Thomas HJ, Lawrence DM, Haslam DM, Malacova E, Dunne MP. The prevalence of child maltreatment in Australia: findings from a national survey. Med J Aust. 2023; 218 Suppl 6(Suppl 6): S13-S18.

Supplementary Table S12. Data sources included in the prevalence analysis		
Year	Title	Citation
1998	Assessment of First Sexual Intercourse in Young Women with a History of Childhood Sexual Abuse	Moore EE, Jayasinghe Y, Olsson CA, Romaniuk H, Sasongko V, Patton GC, Garland SM. Assessment of first sexual intercourse in young women with a history of childhood sexual abuse. Aust N Z J Obstet Gynaecol. 2011; 51(3): 276-79.
1994	The Prevalence of Childhood Sexual Abuse and Adolescent Unwanted Sexual Contact Among Boys and Girls Living in Victoria, Australia	Moore EE, Romaniuk H, Olsson CA, Jayasinghe Y, Carlin JB, Patton GC. The prevalence of childhood sexual abuse and adolescent unwanted sexual contact among boys and girls living in Victoria, Australia. Child Abuse Negl. 2010; 34(5): 379-85.
2002	The Prevalence of Childhood Sexual Abuse and Adolescent Unwanted Sexual Contact Among Boys and Girls Living in Victoria, Australia	Moore EE, Romaniuk H, Olsson CA, Jayasinghe Y, Carlin JB, Patton GC. The prevalence of childhood sexual abuse and adolescent unwanted sexual contact among boys and girls living in Victoria, Australia. Child Abuse Negl. 2010; 34(5): 379-85.
1996	Intimate Partner Violence and Health Outcomes in Mid-Life Women: A Population-Based Cohort Study	Schei B, Guthrie JR, Dennerstein L, Alford S. Intimate partner violence and health outcomes in mid-life women: a population-based cohort study. Arch Womens Ment Health. 2006; 9(6): 317-24.
2017	Australian Longitudinal Study on Women's Health 1989-1995 Cohort Summary Surveys 1-5 (2013-2017)	University of Newcastle (Australia), University of Queensland (Australia). Australian Longitudinal Study on Women's Health 1989-1995 Cohort Summary Surveys 1-5 (2013-2017). Australia: Australian Longitudinal Study on Women's Health, 2019.
2001	Sex in Australia: Experiences of Sexual Coercion Among a Representative Sample of Adults	de Visser RO, Smith AM, Rissel CE, Richters J, Grulich AE. Sex in Australia: experiences of sexual coercion among a representative sample of adults. Aust N Z J Public Health. 2003; 27(2): 198-203.
Austria		
2012	European Union Violence Against Women Study 2012	European Union Agency for Fundamental Rights. European Union Violence Against Women Study 2012.
Azerbaijan		
2016	Azerbaijan International Men and Gender Equality Study 2016	International Center for Research on Women, International Center for Social Research (ICSR) (Azerbaijan), Promundo, State Committee for Family, Women and Children's Affairs (Azerbaijan), United Nations Population Fund (UNFPA). Azerbaijan International Men and Gender Equality Study 2016.
2006	Azerbaijan Demographic and Health Survey 2006	Macro International, Inc, State Statistical Committee of Azerbaijan. Azerbaijan Demographic and Health Survey 2006. Fairfax, United States of America: ICF International.
Bangladesh		

Supplementary Table S12. Data sources included in the prevalence analysis		
Year	Title	Citation
2006	Bangladesh Urban Health Survey 2006	Associates for Community and Population Research (ACPR), International Centre for Diarrhoeal Disease Research, Bangladesh (ICDDR,B), MEASURE Evaluation Project, Carolina Population Center, University of North Carolina, National Institute of Population Research and Training (NIPORT). Bangladesh Urban Health Survey 2006.
2011	Bangladesh Violence Against Women Survey 2011	Bangladesh Bureau of Statistics (BBS). Bangladesh Violence Against Women Survey 2011. Dhaka, Bangladesh: Bangladesh Bureau of Statistics (BBS).
2015	Bangladesh Violence Against Women Survey 2015	Bangladesh Bureau of Statistics (BBS). Bangladesh Violence Against Women Survey 2015. Dhaka, Bangladesh: Bangladesh Bureau of Statistics (BBS).
2011	Pathways Between Childhood Trauma, Intimate Partner Violence, and Harsh Parenting: Findings from the UN Multi-Country Study on Men and Violence in Asia and the Pacific	Fulu E, Miedema S, Roselli T, McCook S, Chan KL, Haardörfer R, Jewkes R, UN Multi-country Study on Men and Violence study team. Pathways between childhood trauma, intimate partner violence, and harsh parenting: findings from the UN Multi-country Study on Men and Violence in Asia and the Pacific. Lancet Glob Health. 2017; 5(5): e512–22.
2002	Bangladesh WHO Multi-Country Study on Women's Health and Domestic Violence Against Women 2001	International Centre for Diarrhoeal Disease Research, Bangladesh (ICDDR,B), Naripokkho, Uppsala University, World Health Organization (WHO). Bangladesh WHO Multi-country Study on Women's Health and Domestic Violence Against Women 2001.
2007	Bangladesh Demographic and Health Survey 2007	Macro International, Inc, Mitra and Associates, National Institute of Population Research and Training (NIPORT). Bangladesh Demographic and Health Survey 2007. Fairfax, United States of America: ICF International, 2009.
Belgium		
2012	European Union Violence Against Women Study 2012	European Union Agency for Fundamental Rights. European Union Violence Against Women Study 2012.
2009	Belgium Emotional, Physical and Sexual Abuse Study 2009	Ghent University, Institute for the Equality of Women and Men (Belgium), University of Liège (Belgium). Belgium Emotional, Physical and Sexual Abuse Study 2009.
Belize		
2005	Belize Gender, Alcohol and Culture: An International Study (GENACIS) 2005	Aarhus University, Addiction Switzerland Research Institute, Alcohol Research Group, Public Health Institute, Centre for Addiction and Mental Health (Canada), Centre for Alcohol Policy Research, Turning Point Alcohol and Drug Centre (Australia), Kettil Bruun Society for Social and Epidemiological Research on Alcohol, University of North Dakota. Belize Gender, Alcohol and Culture: An International Study (GENACIS) 2005.
Benin		

Supplementary Table S12. Data sources included in the prevalence analysis		
Year	Title	Citation
2017	Benin Demographic and Health Survey 2017-2018	Hubert Koutoukou Maga National University Hospital Center (CNHU-HKM)(Benin), ICF International, National Institute of Statistics and Economic Analysis (INSAE) (Benin), National Malaria Control Program, Ministry of Health (Benin), Permanent Secretariat of the Food Council and Nutrition (SP-CAN)(Benin). Benin Demographic and Health Survey 2017-2018. Fairfax, United States of America: ICF International, 2018.
2021	Benin Multiple Indicator Cluster Survey 2021-2022	National Institute of Statistics and Demography (INStaD) (Benin), United Nations Children's Fund (UNICEF). Benin Multiple Indicator Cluster Survey 2021-2022. New York, United States of America: United Nations Children's Fund (UNICEF), 2023.
Bhutan		
2012	Bhutan Health Survey 2012-2013	Ministry of Health (Bhutan), National Statistics Bureau (Bhutan), United Nations Population Fund (UNFPA). Bhutan Health Survey 2012-2013.
2017	Bhutan National Study on Women's Health and Life Experiences 2017	National Commission for Women and Children (NCWC) (Bhutan). Bhutan National Study on Women's Health and Life Experiences 2017.
Bosnia and Herzegovina		
2011	Bosnia and Herzegovina Balkan Epidemiological Study on Child Abuse and Neglect 2011	Institute of Child Health (Greece), University of Sarajevo. Bosnia and Herzegovina Balkan Epidemiological Study on Child Abuse and Neglect 2011.
Botswana		
2016	Botswana Violence Against Children Survey 2016	5 AM Holdings (PTY) LTD (Botswana), Centers for Disease Control and Prevention (CDC), Department of Social Protection (Botswana), Together for Girls, United Nations Children's Fund (UNICEF). Botswana Violence Against Children Survey 2016. Washington, DC, United States of America: Together for Girls.
2008	Botswana AIDS Impact Survey 2008	Central Statistics Office (Botswana), National AIDS Coordinating Agency (Botswana). Botswana AIDS Impact Survey 2008. Gaborone, Botswana: Statistics Botswana, 2015.
2011	Botswana Gender Based Violence Indicators Study 2011	Gender Links (South Africa), Women's Affairs Department, Ministry of Labour and Home Affairs (Botswana). Botswana Gender Based Violence Indicators Study 2011.
2013	Botswana AIDS Impact Survey 2013	Ministry of Health (Botswana), National AIDS Coordinating Agency (Botswana), Statistics Botswana. Botswana AIDS Impact Survey 2013. Gaborone, Botswana: Statistics Botswana, 2015.
Brazil		

Supplementary Table S12. Data sources included in the prevalence analysis		
Year	Title	Citation
2001	Brazil - Botucatu Gender, Alcohol and Culture: An International Study (GENACIS) 2001-2002	Aarhus University, Addiction Switzerland Research Institute, Alcohol Research Group, Public Health Institute, Centre for Addiction and Mental Health (Canada), Centre for Alcohol Policy Research, Turning Point Alcohol and Drug Centre (Australia), Kettil Bruun Society for Social and Epidemiological Research on Alcohol, University of North Dakota. Brazil - Botucatu Gender, Alcohol and Culture: An International Study (GENACIS) 2001-2002.
2002	Child Sexual Abuse in Southern Brazil and Associated Factors: A Population-Based Study	Bassani DG, Palazzo LS, Béria JU, Gigante LP, Figueiredo AC, Aerts DR, Raymann BC. Child sexual abuse in southern Brazil and associated factors: a population-based study. BMC Public Health. 2009; 9(1): 133.
2015	Brazil National Survey of School Health 2015	Brazilian Institute of Geography and Statistics (IBGE). Brazil National Survey of School Health 2015. Rio de Janeiro, Brazil: Brazilian Institute of Geography and Statistics (IBGE), 2016.
2000	Brazil WHO Multi-Country Study on Women's Health and Domestic Violence Against Women 2000-2001	Federal University of Pernambuco, Feminist Collective for Health and Sexuality (São Paulo), University of São Paulo, World Health Organization (WHO). Brazil WHO Multi-country Study on Women's Health and Domestic Violence Against Women 2000-2001.
2011	Brazil National Alcohol and Drugs Survey 2011-2012	Ipsos, National Institute of Public Policy for Alcohol and Other Drugs (INPAD) (Brazil), University of São Paulo. Brazil National Alcohol and Drugs Survey 2011-2012.
Bulgaria		
2019	Bulgaria Violence Against Children Study 2019	Coram International, United Nations Children's Fund (UNICEF). Bulgaria Violence Against Children Study 2019.
2012	European Union Violence Against Women Study 2012	European Union Agency for Fundamental Rights. European Union Violence Against Women Study 2012.
2011	Bulgaria Balkan Epidemiological Study on Child Abuse and Neglect 2011	Institute of Child Health (Greece), South-West University "Neofit Rilski". Bulgaria Balkan Epidemiological Study on Child Abuse and Neglect 2011.
Burkina Faso		
2020	Burkina Faso Performance Monitoring and Action Phase 2 Household and Female Survey 2020-2021	Bill and Melinda Gates Institute for Population and Reproductive Health, Johns Hopkins Bloomberg School of Public Health, Higher Institute of Population Sciences (ISSP) (Burkina Faso), Jhpiego. Burkina Faso Performance Monitoring and Action Phase 2 Household and Female Survey 2020-2021. Baltimore, United States of America: Johns Hopkins Bloomberg School of Public Health, 2021.
2021	Burkina Faso Demographic and Health Survey 2021	ICF International, National Institute of Public Health (INSP) (Burkina Faso), National Institute of Statistics and Demography (Burkina Faso). Burkina Faso Demographic and Health Survey 2021.

Supplementary Table S12. Data sources included in the prevalence analysis		
Year	Title	Citation
2010	Burkina Faso Demographic and Health Survey 2010-2011	ICF Macro, Ministry of Health (Burkina Faso), National Institute of Statistics and Demography (Burkina Faso). Burkina Faso Demographic and Health Survey 2010-2011. Fairfax, United States of America: ICF International.
2018	Burkina Faso National Study on Violence Against Children 2018	Ministry of Women, National Solidarity, Family and Humanitarian Action (Burkina Faso). Burkina Faso National Study on Violence Against Children 2018.
Burundi		
2016	Burundi Demographic and Health Survey 2016-2017	Burundi Institute of Statistics and Economic Studies, ICF International, Ministry of Public Health and the Fight Against AIDS (Burundi). Burundi Demographic and Health Survey 2016-2017. Fairfax, United States of America: ICF International, 2018.
Cabo Verde		
2018	Cape Verde Demographic and Reproductive Health Survey 2018	National Institute of Statistics (Cape Verde), Utica International. Cape Verde Demographic and Reproductive Health Survey 2018. Praia, Cabo Verde: National Institute of Statistics (Cape Verde), 2020.
Cambodia		
2013	Cambodia Violence Against Children Survey 2013	Centers for Disease Control and Prevention (CDC), Ministry of Social Affairs, Veterans and Youth Rehabilitation (MoSVY) (Cambodia), Ministry of Women's Affairs (Cambodia), National Institute of Statistics (Cambodia), Together for Girls, United Nations Children's Fund (UNICEF). Cambodia Violence Against Children Survey 2013. Washington, DC, United States of America: Together for Girls.
2011	Pathways Between Childhood Trauma, Intimate Partner Violence, and Harsh Parenting: Findings from the UN Multi-Country Study on Men and Violence in Asia and the Pacific	Fulu E, Miedema S, Roselli T, McCook S, Chan KL, Haardörfer R, Jewkes R, UN Multi-country Study on Men and Violence study team. Pathways between childhood trauma, intimate partner violence, and harsh parenting: findings from the UN Multi-country Study on Men and Violence in Asia and the Pacific. Lancet Glob Health. 2017; 5(5): e512–22.
2014	Cambodia Demographic and Health Survey 2014	ICF International, Ministry of Health (Cambodia), National Institute of Statistics (Cambodia). Cambodia Demographic and Health Survey 2014. Fairfax, United States of America: ICF International, 2017.
2021	Cambodia Demographic and Health Survey 2021-2022	ICF International, Ministry of Health (Cambodia), National Institute of Statistics (Cambodia). Cambodia Demographic and Health Survey 2021-2022. Fairfax, United States of America: ICF International, 2023.
2005	Cambodia Demographic and Health Survey 2005-2006	Macro International, Inc, National Institute of Public Health (Cambodia), National Institute of Statistics (Cambodia). Cambodia Demographic and Health Survey 2005-2006. Fairfax, United States of America: ICF International.

Supplementary Table S12. Data sources included in the prevalence analysis		
Year	Title	Citation
2015	Cambodia WHO Multi-Country Study on Women's Health and Domestic Violence Against Women 2015	Ministry of Women's Affairs (Cambodia), National Institute of Statistics (Cambodia), World Health Organization (WHO). Cambodia WHO Multi-country Study on Women's Health and Domestic Violence Against Women 2015. Phnom Penh, Cambodia: National Institute of Statistics (Cambodia).
2012	Cambodia United Nations Multi-Country Study on Men and Violence 2012 - Equimundo	Partners for Prevention, United Nations Development Programme (UNDP), United Nations Entity for Gender Equality and the Empowerment of Women (UN Women), United Nations Population Fund (UNFPA), United Nations Volunteers (UNV). Cambodia United Nations Multi-country Study on Men and Violence 2012 - Equimundo.
Cameroon		
2017	Cameroon Population-Based HIV Impact Assessment 2017-2018	Centers for Disease Control and Prevention (CDC), Global Health System Solutions (GHSS) (Cameroon), ICAP, Columbia University Mailman School of Public Health, International Reference Center "Chantal Biya" (CIRCB) (Cameroon), Ministry of Public Health (Cameroon), National AIDS Control Committee (CNLS) (Cameroon), National Institute of Statistics (Cameroon), National Public Health Laboratory (Cameroon), Pasteur Center of Cameroon. Cameroon Population-Based HIV Impact Assessment 2017-2018. New York, New York: ICAP, Columbia University Mailman School of Public Health, 2021.
2011	Cameroon Demographic and Health Survey 2011	ICF International, Ministry of Economy, Planning and Regional Development (Cameroon), Ministry of Public Health (Cameroon), National Institute of Statistics (Cameroon), Pasteur Center of Cameroon. Cameroon Demographic and Health Survey 2011. Fairfax, United States of America: ICF International.
2018	Cameroon Demographic and Health Survey 2018-2019	ICF International, Ministry of Public Health (Cameroon), National Institute of Statistics (Cameroon). Cameroon Demographic and Health Survey 2018-2019. Fairfax, United States of America: ICF International.
2004	Cameroon Demographic and Health Survey 2004	Macro International, Inc, National Institute of Statistics (Cameroon). Cameroon Demographic and Health Survey 2004. Fairfax, United States of America: ICF International.
Canada		
2004	Canada Gender, Alcohol and Culture: An International Study (GENACIS) 2004	Aarhus University, Addiction Switzerland Research Institute, Alcohol Research Group, Public Health Institute, Centre for Addiction and Mental Health (Canada), Centre for Alcohol Policy Research, Turning Point Alcohol and Drug Centre (Australia), Kettil Bruun Society for Social and Epidemiological Research on Alcohol, University of North Dakota. Canada Gender, Alcohol and Culture: An International Study (GENACIS) 2004.
2012	Relationship Between Child Abuse Exposure and Reported Contact with Child Protection Organizations: Results from the Canadian Community Health Survey	Afifi TO, MacMillan HL, Taillieu T, Cheung K, Turner S, Tonmyr L, Hovdestad W. Relationship between child abuse exposure and reported contact with child protection organizations: results from the Canadian Community Health Survey. Child Abuse Negl. 2015; 46: 198–206.

Supplementary Table S12. Data sources included in the prevalence analysis		
Year	Title	Citation
1999	Men's and Women's Childhood Sexual Abuse and Victimization in Adult Partner Relationships: A Study of Risk Factors	Daigneault I, Hébert M, McDuff P. Men's and women's childhood sexual abuse and victimization in adult partner relationships: A study of risk factors. Child Abuse Negl. 2009; 33(9): 638-47.
1990	Childhood Abuse and Lifetime Psychopathology in a Community Sample	MacMillan HL, Fleming JE, Streiner DL, Lin E, Boyle MH, Jamieson E, Duku EK, Walsh CA, Wong MY, Beardslee WR. Childhood abuse and lifetime psychopathology in a community sample. Am J Psychiatry. 2001; 158(11): 1878–83.
2014	Canada General Social Survey on Victimization 2014	Statistics Canada (StatCan). Canada General Social Survey on Victimization 2014. 2015.
2006	Prevalence and Co-Occurrence of Violence Against Children in the Quebec Population	Tourigny M, Hébert M, Joly J, Cyr M, Baril K. Prevalence and co-occurrence of violence against children in the Quebec population. Aust N Z J Public Health. 2008; 32(4): 331-5.
Central African Republic		
2006	Central African Republic Multiple Indicator Cluster Survey 2006	United Nations Children's Fund (UNICEF). Central African Republic Multiple Indicator Cluster Survey 2006. New York, United States: United Nations Children's Fund (UNICEF).
Chad		
2014	Chad Demographic and Health Survey 2014-2015	ICF International, National Institute for Statistics, Economic and Demographic Studies (INSEED) (Chad). Chad Demographic and Health Survey 2014-2015. Fairfax, United States of America: ICF International, 2016.
Chile		
2008	Chile National Survey of Victimization for Domestic Violence and Sexual Crimes 2008	Center for the Study and Analysis of Crime (CEAD) (Chile), Directorate of Social Studies UC (DESUC), Pontifical Catholic University of Chile (Chile). Chile National Survey of Victimization for Domestic Violence and Sexual Crimes 2008. Chile: Center for the Study and Analysis of Crime (CEAD) (Chile).
2012	Chile National Survey of Victimization for Domestic Violence and Sexual Crimes 2012	Center for the Study and Analysis of Crime (CEAD) (Chile), GfK Adimark (Chile). Chile National Survey of Victimization for Domestic Violence and Sexual Crimes 2012. Chile: Center for the Study and Analysis of Crime (CEAD) (Chile).
2009	Chile International Men and Gender Equality Study 2009	EME Masculinities and Gender Equity (Chile), International Center for Research on Women, Promundo. Chile International Men and Gender Equality Study 2009.
China		

Supplementary Table S12. Data sources included in the prevalence analysis		
Year	Title	Citation
2000	Childhood Sexual Abuse, An Investigation Among 239 Male High School Students	Chen J, Dunne M, Wang X. Childhood Sexual Abuse, An Investigation among 239 Male High School Students. Chin Ment Health J. 2003; 17(5): 345–7.
2001	Child Sexual Abuse in China: A Study of Adolescents in Four Provinces	Chen J, Dunne MP, Han P. Child sexual abuse in China: a study of adolescents in four provinces. Child Abuse Negl. 2004; 28(11): 1171–86.
2000	Prevalence of Childhood Sexual Abuse Among Female Students in a Senior High School	Chen J. Prevalence of childhood sexual abuse among female students in a senior high school. Chin J Sch Health. 2002; 23: 108–10.
2011	Pathways Between Childhood Trauma, Intimate Partner Violence, and Harsh Parenting: Findings from the UN Multi-Country Study on Men and Violence in Asia and the Pacific	Fulu E, Miedema S, Roselli T, McCook S, Chan KL, Haardörfer R, Jewkes R, UN Multi-country Study on Men and Violence study team. Pathways between childhood trauma, intimate partner violence, and harsh parenting: findings from the UN Multi-country Study on Men and Violence in Asia and the Pacific. Lancet Glob Health. 2017; 5(5): e512–22.
1999	A Population-Based Study of Childhood Sexual Contact in China: Prevalence and Long-Term Consequences	Luo Y, Parish WL, Laumann EO. A population-based study of childhood sexual contact in China: prevalence and long-term consequences. Child Abuse Negl. 2008; 32(7): 721-31.
1999	China Health and Family Life Survey 1999-2000	National Opinion Research Center, University of Chicago (NORC), Renmin University, Beijing, Peking Union Medical College, Beijing, University of North Carolina. China Health and Family Life Survey 1999-2000. Chicago, USA: Population Research Center, University of Chicago.
2009	China Optimus Study 2009-2010	UBS Optimus Foundation, University of Hong Kong. China Optimus Study 2009-2010.
2005	To Unfold a Hidden Epidemic: Prevalence of Child Maltreatment and its Health Implications among High School Students in Guangzhou, China	Wong WCW, Leung PWS, Tang CSK, Chen W-Q, Lee A, Ling DC. To unfold a hidden epidemic: prevalence of child maltreatment and its health implications among high school students in Guangzhou, China. Child Abuse Negl. 2009; 33(7): 441-50.
Colombia		
2018	Colombia Violence Against Children and Youth Survey 2018	Centers for Disease Control and Prevention (CDC), International Organization for Migration (IOM), Ministry of Health and Social Protection (Colombia), Together for Girls. Colombia Violence Against Children and Youth Survey 2018. Bogotá, Colombia: Ministry of Health and Social Protection (Colombia).
2015	Colombia Demographic and Health Survey 2015-2016	ICF International, Ministry of Health (Colombia), Profamilia (Colombia). Colombia Demographic and Health Survey 2015-2016. Fairfax, United States of America: ICF International, 2017.

Supplementary Table S12. Data sources included in the prevalence analysis		
Year	Title	Citation
2009	Colombia Demographic and Health Survey 2009-2010	ICF Macro, Profamilia (Colombia). Colombia Demographic and Health Survey 2009-2010. Fairfax, United States of America: ICF International, 2011.
2000	Colombia Demographic and Health Survey 2000	Macro International, Inc, Profamilia (Colombia). Colombia Demographic and Health Survey 2000. Fairfax, United States of America: ICF International, 2000.
2004	Colombia Demographic and Health Survey 2004-2005	Macro International, Inc, Profamilia (Colombia). Colombia Demographic and Health Survey 2004-2005. Fairfax, United States of America: ICF International, 2005.
Comoros		
2012	Comoros Demographic and Health Survey 2012-2013	General Directorate of Statistics and Forecasting (Comoros), ICF International. Comoros Demographic and Health Survey 2012-2013. Fairfax, United States of America: ICF International.
2022	Comoros Multiple Indicator Cluster Survey 2022	National Institute of Statistics and Economic and Demographic Studies (INSEED) (Comoros), United Nations Children's Fund (UNICEF). Comoros Multiple Indicator Cluster Survey 2022. New York, United States of America: United Nations Children's Fund (UNICEF), 2023.
Cook Islands		
2012	Cook Islands Family Health and Safety Study 2012-2013	Cook Islands Statistics Office, Ministry of Health (Cook Islands), National Council of Women (Cook Islands). Cook Islands Family Health and Safety Study 2012-2013. 2014.
Costa Rica		
2003	Costa Rica Gender, Alcohol and Culture: An International Study (GENACIS) 2003	Aarhus University, Addiction Switzerland Research Institute, Alcohol Research Group, Public Health Institute, Centre for Addiction and Mental Health (Canada), Centre for Alcohol Policy Research, Turning Point Alcohol and Drug Centre (Australia), Kettil Bruun Society for Social and Epidemiological Research on Alcohol, University of North Dakota. Costa Rica Gender, Alcohol and Culture: An International Study (GENACIS) 2003.
2003	International Violence Against Women Surveys Data 2002-2005	European Institute for Crime Prevention and Control, affiliated with the United Nations (HEUNI), United Nations Office on Drugs and Crime (UNODC), Statistics Canada, United Nations Interregional Crime and Justice Research Institute (UNICRI). International Violence Against Women Surveys (IVAWS) Data 2002-2005. As provided by the Global Burden of Disease Child Sexual Abuse and Intimate Partner Violence Expert Group. [Unpublished].
2003	Violence Against Women: An International Perspective	Johnson H, Ollus N, Nevala S. Violence Against Women: An International Perspective. New York City, United States: Springer; 2008.
Croatia		

Supplementary Table S12. Data sources included in the prevalence analysis		
Year	Title	Citation
2012	European Union Violence Against Women Study 2012	European Union Agency for Fundamental Rights. European Union Violence Against Women Study 2012.
2011	Croatia Balkan Epidemiological Study on Child Abuse and Neglect 2011	Institute of Child Health (Greece), University of Zagreb. Croatia Balkan Epidemiological Study on Child Abuse and Neglect 2011.
Cyprus		
2012	European Union Violence Against Women Study 2012	European Union Agency for Fundamental Rights. European Union Violence Against Women Study 2012.
Czechia		
2002	Czech Republic Gender, Alcohol and Culture: An International Study (GENACIS) 2002	Aarhus University, Addiction Switzerland Research Institute, Alcohol Research Group, Public Health Institute, Centre for Addiction and Mental Health (Canada), Centre for Alcohol Policy Research, Turning Point Alcohol and Drug Centre (Australia), Kettil Bruun Society for Social and Epidemiological Research on Alcohol, University of North Dakota. Czech Republic Gender, Alcohol and Culture: An International Study (GENACIS) 2002.
2003	International Violence Against Women Surveys Data 2002-2005	European Institute for Crime Prevention and Control, affiliated with the United Nations (HEUNI), United Nations Office on Drugs and Crime (UNODC), Statistics Canada, United Nations Interregional Crime and Justice Research Institute (UNICRI). International Violence Against Women Surveys (IVAWS) Data 2002-2005. As provided by the Global Burden of Disease Child Sexual Abuse and Intimate Partner Violence Expert Group. [Unpublished].
2012	European Union Violence Against Women Study 2012	European Union Agency for Fundamental Rights. European Union Violence Against Women Study 2012.
2003	Violence Against Women: An International Perspective	Johnson H, Ollus N, Nevala S. Violence Against Women: An International Perspective. New York City, United States: Springer; 2008.
1991	Experiences with Sexual Aggression within the General Population in the Czech Republic	Weiss P, Zverina J. Experiences with sexual aggression within the general population in the Czech Republic. Arch Sex Behav. 1999; 28(3): 265-9.
Côte d'Ivoire		
2004	Côte d’Ivoire AIDS Indicator Survey 2005	CDC Retro-CI, Ministry of the Fight Against AIDS (Côte d’Ivoire), National Institute of Statistics (Côte d’Ivoire), ORC Macro. Côte d’Ivoire AIDS Indicator Survey 2005. Fairfax, United States of America: ICF International.
2018	Côte d’Ivoire Violence Against Children and Youth Survey 2018	Centers for Disease Control and Prevention (CDC), Ministry of Women, Family and Children (Côte d’Ivoire), National Institute of Statistics (Côte d’Ivoire), National Program for the Care of Orphans and Other Children Made Vulnerable by HIV/AIDS (PNOEV) (Côte d’Ivoire), Together for Girls.

Supplementary Table S12. Data sources included in the prevalence analysis		
Year	Title	Citation
		Côte d'Ivoire Violence Against Children and Youth Survey 2018. Côte d'Ivoire: Ministry of Women, Family and Children (Côte d'Ivoire).
2021	Côte d'Ivoire Demographic and Health Survey 2021	ICF International, Ministry of Health and Public Hygiene (MSHP) (Côte d'Ivoire), National Institute of Statistics (Côte d'Ivoire). Côte d'Ivoire Demographic and Health Survey 2021. Fairfax, United States of America: ICF International, 2023.
Democratic Republic of the Congo		
2013	Democratic Republic of the Congo Demographic and Health Survey 2013-2014	ICF International, Ministry of Planning and Monitoring Implementation of the Revolution of Modernity (Congo, DR), Ministry of Public Health (Congo, DR), National Institute of Statistics (Congo, DR). Democratic Republic of the Congo Demographic and Health Survey 2013-2014. Fairfax, United States of America: ICF International, 2014.
2007	Democratic Republic of the Congo Demographic and Health Survey 2007	Macro International, Inc, Ministry of Planning (Congo, DR). Democratic Republic of the Congo Demographic and Health Survey 2007. Fairfax, United States of America: ICF International.
Denmark		
2012	European Union Violence Against Women Study 2012	European Union Agency for Fundamental Rights. European Union Violence Against Women Study 2012.
2002	The Prevalence of Unwanted and Unlawful Sexual Experiences Reported by Danish Adolescents: Results from A National Youth Survey in 2002	Helweg-Larsen K, Larsen HB. The prevalence of unwanted and unlawful sexual experiences reported by Danish adolescents: Results from a national youth survey in 2002. Acta Paediatr. 2006; 95(10): 1270-6.
2008	Denmark Youth Welfare Survey 2008	National Institute of Public Health (Denmark), University of Copenhagen, University of Southern Denmark. Denmark Youth Welfare Survey 2008.
2002	Denmark Youth Welfare Survey 2002	National Institute of Public Health (Denmark). Denmark Youth Welfare Survey 2002.
Dominican Republic		
2013	Dominican Republic Demographic and Health Survey 2013	Center for Social and Demographic Studies (Dominican Republic) (CESDEM), ICF International, Ministry of Public Health and Social Assistance (Dominican Republic). Dominican Republic Demographic and Health Survey 2013. Fairfax, United States of America: ICF International, 2014.
2002	Dominican Republic Demographic and Health Survey 2002	Center for Social and Demographic Studies (Dominican Republic) (CESDEM), Macro International, Inc. Dominican Republic Demographic and Health Survey 2002. Fairfax, United States of America: ICF International.

Supplementary Table S12. Data sources included in the prevalence analysis		
Year	Title	Citation
2007	Dominican Republic Demographic and Health Survey 2007	Center for Social and Demographic Studies (Dominican Republic) (CESDEM), Macro International, Inc. Dominican Republic Demographic and Health Survey 2007. Fairfax, United States of America: ICF International.
1999	Dominican Republic Experimental Demographic and Health Survey 1999	Center for Social and Demographic Studies (Dominican Republic) (CESDEM), Macro International, Inc. Dominican Republic Experimental Demographic and Health Survey 1999. Fairfax, United States of America: ICF International.
Ecuador		
2004	Ecuador Reproductive Health Survey 2004	Center for Studies of Population and Social Development (CEPAR) (Ecuador) and Division of Reproductive Health-Centers for Disease Control and Prevention (CDC). (2005) Ecuador Reproductive Health Survey 2004. Quito, Ecuador: CEPAR.
1999	Ecuador Reproductive Health Survey 1999	Center for Studies of Population and Social Development (CEPAR) (Ecuador), Division of Reproductive Health-Centers for Disease Control and Prevention (CDC). Ecuador Reproductive Health Survey 1999. Atlanta, United States: Centers for Disease Control and Prevention (CDC), 2001.
2011	Ecuador National Survey of Family Relationships and Gender Violence Against Women 2011	Ministry of the Interior (Ecuador), National Institute of Statistics and Censuses (Ecuador), Transition Committee to the Council on Women and Gender Equality (Ecuador). Ecuador National Survey of Family Relationships and Gender Violence Against Women 2011.
Egypt		
2015	Egypt Economic Gender-Based Violence Survey 2015	Central Agency for Public Mobilization and Statistics (CAPMAS) (Egypt), United Nations Population Fund (UNFPA). Egypt Economic Gender-Based Violence Survey 2015.
El Salvador		
2002	El Salvador Reproductive Health Survey 2002-2003	Asociación Demográfica Salvadoreña (ADS), Division of Reproductive Health-Centers for Disease Control and Prevention (CDC). (2004) El Salvador Reproductive Health Survey 2002-2003. San Salvador, El Salvador: ADS.
2008	El Salvador Reproductive Health Survey 2008	Asociación Demográfica Salvadoreña (ADS), Division of Reproductive Health-Centers for Disease Control and Prevention (CDC). (2009) El Salvador Reproductive Health Survey 2008. San Salvador, El Salvador: ADS.
2017	El Salvador Violence Against Children, Girls and Adolescents Survey 2017	Centers for Disease Control and Prevention (CDC), General Directorate of Statistics and Census (DIGESTYC) (El Salvador), International Organization for Migration (IOM), Ministry of Justice and Public Safety (El Salvador), Together for Girls. El Salvador Violence Against Children, Girls and Adolescents Survey 2017.
2002	Childhood Abuse as a Risk Factor For Adolescent Pregnancy in El Salvador	Pallitto CC, Murillo V. Childhood Abuse as a Risk Factor for Adolescent Pregnancy in El Salvador. J Adolesc Health. 2008; 42(6): 580-6.

Supplementary Table S12. Data sources included in the prevalence analysis		
Year	Title	Citation
2014	El Salvador Violence Against Women Study 2014	Pan American Health Organization (PAHO), Technological University of El Salvador, University of Granada (Spain), World Health Organization (WHO). El Salvador Violence Against Women Study 2014.
2002	Dimensions of Child Sexual Abuse Before Age 15 in Three Central American Countries: Honduras, El Salvador, and Guatemala	Speizer IS, Goodwin M, Whittle L, Clyde M, Rogers J. Dimensions of child sexual abuse before age 15 in three Central American countries: Honduras, El Salvador, and Guatemala. Child Abuse Negl. 2008; 32(4): 455-62.
Estonia		
2012	European Union Violence Against Women Study 2012	European Union Agency for Fundamental Rights. European Union Violence Against Women Study 2012.
Eswatini		
2021	Eswatini Violence Against Children and Youth Survey 2021	Centers for Disease Control and Prevention (CDC), Central Statistical Office (Eswatini), ICAP, Columbia University Mailman School of Public Health, Ministry of Health (Eswatini), President's Emergency Plan for AIDS Relief (PEPFAR). Eswatini Violence Against Children and Youth Survey 2021.
2007	Swaziland National Study on Violence Against Children and Young Women 2007	Centers for Disease Control and Prevention (CDC), United Nations Children's Fund (UNICEF). Swaziland National Study on Violence Against Children and Young Women 2007.
2021	Eswatini Multiple Indicator Cluster Survey 2021-2022	Central Statistical Office (Eswatini), United Nations Children's Fund (UNICEF). Eswatini Multiple Indicator Cluster Survey 2021-2022. 2023.
2014	Swaziland Steps Noncommunicable Disease Risk Factors Survey 2014	Ministry of Health (Swaziland), World Health Organization (WHO). Swaziland STEPS Noncommunicable Disease Risk Factors Survey 2014.
Ethiopia		
2017	Ethiopia Population-Based HIV Impact Assessment 2017-2018	Centers for Disease Control and Prevention (CDC), Central Statistics Agency (Ethiopia), Ethiopian Public Health Institute (EPHI), Government of Ethiopia, ICAP, Columbia University Mailman School of Public Health, Ministry of Health (Ethiopia). Ethiopia Population-Based HIV Impact Assessment 2017-2018. New York, New York: ICAP, Columbia University Mailman School of Public Health, 2021.
2016	Ethiopia Demographic and Health Survey 2016	Central Statistics Agency (Ethiopia), ICF International. Ethiopia Demographic and Health Survey 2016. Fairfax, United States of America: ICF International, 2017.
2015	Ethiopia Steps Noncommunicable Disease Risk Factors Survey 2015	Ethiopian Public Health Institute (EPHI), World Health Organization (WHO). Ethiopia STEPS Noncommunicable Disease Risk Factors Survey 2015.

Supplementary Table S12. Data sources included in the prevalence analysis		
Year	Title	Citation
2014	Childhood Sexual Abuse Experiences and Its Associated Factors Among Adolescent Female High School Students in Arbaminch Town, Gammo Goffa Zone, Southern Ethiopia: A Mixed Method Study	Mekuria A, Nigussie A, Abera M. Childhood sexual abuse experiences and its associated factors among adolescent female high school students in Arbaminch town, Gammo Goffa zone, Southern Ethiopia: a mixed method study. BMC Int Health Hum Rights. 2015; 15: 21.
2005	Child Sexual Abuse and Its Outcomes Among High School Students in Southwest Ethiopia	Worku D, Gebremariam A, Jayalakshmi S. Child sexual abuse and its outcomes among high school students in southwest Ethiopia. Trop Doct. 2006; 36(3): 137-40.
Fiji		
2011	Fiji National Research on Women's Health and Life Experiences 2010-2011	Fiji Women's Crisis Centre. Fiji National Research on Women's Health and Life Experiences 2010-2011.
Finland		
2012	European Union Violence Against Women Study 2012	European Union Agency for Fundamental Rights. European Union Violence Against Women Study 2012.
1991	Finland Sex Survey 1991-1992	Haavio-Mannila, Elina (University of Helsinki) & Kontula, Osmo (University of Helsinki): Finnish Sex Survey 1992 [dataset]. Version 2.0 (2018-08-10). Finnish Social Science Data Archive [distributor]. http://urn.fi/urn:nbn:fi:fsd:T-FSD1243
1991	The Prevalence of Child Sexual Abuse in Finland	Sariola H, Uutela A. The prevalence of child sexual abuse in Finland. Child Abuse Negl. 1994; 18(10): 827-35.
France		
1993	Self-Reported Health and Behavioral Problems Among Adolescent Victims of Rape in France: Results of A Cross-Sectional Survey	Choquet M, Darves-Bornoz J-M, Ledoux S, Manfredi R, Hassler C. Self-reported health and behavioral problems among adolescent victims of rape in France: Results of a cross-sectional survey. Child Abuse Negl. 1997; 21(9): 823-32.
2012	European Union Violence Against Women Study 2012	European Union Agency for Fundamental Rights. European Union Violence Against Women Study 2012.
Gabon		
2012	Gabon Demographic and Health Survey 2012	General Directorate of Statistics (Gabon), ICF International, Ministry of Economy, Employment and Sustainable Development (Gabon), Ministry of Health and Social Affairs (Gabon). Gabon Demographic and Health Survey 2012. Fairfax, United States of America: ICF International, 2013.

Supplementary Table S12. Data sources included in the prevalence analysis		
Year	Title	Citation
Gambia		
2013	Gambia Demographic and Health Survey 2013	Gambia Bureau of Statistics (GBOS), ICF International, Ministry of Health and Social Welfare (The Gambia). Gambia Demographic and Health Survey 2013. Fairfax, United States of America: ICF International, 2015.
2019	Gambia Demographic and Health Survey 2019-2020	Gambia Bureau of Statistics (GBOS), ICF International, Ministry of Health and Social Welfare (The Gambia). Gambia Demographic and Health Survey 2019-2020. Fairfax, United States of America: ICF International, 2021.
Georgia		
2009	Georgia National Research on Domestic Violence Against Women 2009	ACT Research (Georgia), Ivane Javakhishvili Tbilisi State University (Georgia). Georgia National Research on Domestic Violence Against Women 2009.
2010	Georgia Reproductive Health Survey 2010-2011	Division of Reproductive Health, Centers for Disease Control and Prevention (CDC), Georgia Ministry of Labor, Health and Social Affairs, National Center for Disease Control and Public Health (Georgia), National Statistics Office of Georgia (GeoStat). Georgia Reproductive Health Survey 2010-2011.
2017	National Study on Violence Against Women in Georgia 2017	European Union (EU), National Statistics Office of Georgia (GeoStat), United Nations Entity for Gender Equality and the Empowerment of Women (UN Women). National Study on Violence Against Women in Georgia 2017. 2018.
2022	National Study on Violence Against Women in Georgia 2022	European Union (EU), National Statistics Office of Georgia (GeoStat), United Nations Entity for Gender Equality and the Empowerment of Women (UN Women). National Study on Violence Against Women in Georgia 2022. 2023.
1999	Georgia Reproductive Health Survey 1999-2000	Georgia Center for Disease Control (NCDC), Georgia Ministry of Labor, Health and Social Affairs (MOLHSA), Division of Reproductive Health-Centers for Disease Control and Prevention (CDC). (2001) Georgia Reproductive Health Survey 1999-2000. Atlanta, United States: Centers for Disease Control and Prevention (CDC).
2005	Georgia Reproductive Health Survey 2005	Georgia Center for Disease Control (NCDC), Georgian Ministry of Labor Health and Social Affairs (MOLHSA), Division of Reproductive Health, Centers for Disease Control and Prevention (CDC). Georgia Reproductive Health Survey 2005. Atlanta, United States: Centers for Disease Control and Prevention (CDC).
2019	Georgia International Men and Gender Equality Study 2019	National Center for Disease Control and Public Health (Georgia), Promundo, United Nations Development Programme (UNDP), United Nations Entity for Gender Equality and the Empowerment of Women (UN Women), United Nations Population Fund (UNFPA). Georgia International Men and Gender Equality Study 2019.
Germany		

Supplementary Table S12. Data sources included in the prevalence analysis		
Year	Title	Citation
2012	European Union Violence Against Women Study 2012	European Union Agency for Fundamental Rights. European Union Violence Against Women Study 2012.
Ghana		
2008	Ghana Demographic and Health Survey 2008	Ghana Statistical Service, Macro International, Inc, Ministry of Health (Ghana). Ghana Demographic and Health Survey 2008. Fairfax, United States of America: ICF International.
Greece		
2012	European Union Violence Against Women Study 2012	European Union Agency for Fundamental Rights. European Union Violence Against Women Study 2012.
2011	Greece Balkan Epidemiological Study on Child Abuse and Neglect 2010-2012	Institute of Child Health (Greece). Greece Balkan Epidemiological Study on Child Abuse and Neglect 2010-2012.
Greenland		
2011	Greenland Youth Welfare 2011	Department of Family, Culture, Church and Gender Equality (Greenland), National Institute of Public Health (Denmark). Greenland Youth Welfare 2011.
2004	Greenland Youth Welfare 2004-2005	National Institute of Public Health (Denmark). Greenland Youth Welfare 2004-2005.
Grenada		
2018	Grenada Women's Health and Life Experiences Survey 2018	Caribbean Development Bank, Central Statistical Office (Grenada), Global Women’s Institute, George Washington University, United Nations Entity for Gender Equality and the Empowerment of Women (UN Women). Grenada Women's Health and Life Experiences Survey 2018.
Guatemala		
2008	Guatemala Reproductive Health Survey 2008-2009	Guatemala Ministry of Health and Social Assistance, University of Valle and Division of Reproductive Health-Centers for Disease Control and Prevention (CDC). Guatemala Reproductive Health Survey 2008-2009. Atlanta, United States: Centers for Disease Control and Prevention (CDC).
2002	Guatemala Reproductive Health Survey 2002	Guatemala Ministry of Health and Social Assistance, University of Valle, Division of Reproductive Health-Centers for Disease Control and Prevention (CDC). (2003) Guatemala Reproductive Health Survey 2002. Atlanta, United States: Centers for Disease Control and Prevention (CDC).
2014	Guatemala Demographic and Health Survey 2014-2015	ICF International, Institute of Nutrition of Central America and Panama, Ministry of Public Health and Social Assistance (Guatemala), National Statistics Institute (Guatemala), Secretary of Planning and Programming of

Supplementary Table S12. Data sources included in the prevalence analysis		
Year	Title	Citation
		the Presidency (Segeplán) (Guatemala). Guatemala Demographic and Health Survey 2014-2015. Fairfax, United States of America: ICF International, 2017.
2002	Dimensions of Child Sexual Abuse Before Age 15 in Three Central American Countries: Honduras, El Salvador, and Guatemala	Speizer IS, Goodwin M, Whittle L, Clyde M, Rogers J. Dimensions of child sexual abuse before age 15 in three Central American countries: Honduras, El Salvador, and Guatemala. Child Abuse Negl. 2008; 32(4): 455-62.
Guinea-Bissau		
2010	Guinea-Bissau Multiple Indicator Cluster Survey 2010	Centers for Disease Control and Prevention (CDC), National Statistics Institute (Guinea-Bissau), United Nations Children's Fund (UNICEF). Guinea-Bissau Multiple Indicator Cluster Survey 2010. New York, United States of America: United Nations Children's Fund (UNICEF), 2018.
Haiti		
2012	Haiti Violence Against Children Survey 2012	Centers for Disease Control and Prevention (CDC), Government of Haiti, Interuniversity Institute for Research and Development (INURED), Multi-Sector Coordination Committee (CCMHAITI) (Haiti), Together for Girls. Haiti Violence Against Children Survey 2012. Washington, DC, United States of America: Together for Girls.
2012	Haiti Demographic and Health Survey 2012	Centers for Disease Control and Prevention (CDC), Haitian Institute of Childhood (IHE), Haitian Institute of Statistics and Informatics, Macro International, Inc. Haiti Demographic and Health Survey 2012. Fairfax, United States of America: ICF International.
2016	Haiti Demographic and Health Survey 2016-2017	Haitian Institute of Childhood (IHE), Haitian Institute of Statistics and Informatics, ICF International, Ministry of Public Health and Population (Haiti). Haiti Demographic and Health Survey 2016-2017. Fairfax, United States of America: ICF International.
2005	Haiti Demographic and Health Survey 2005-2006	Haitian Institute of Childhood (IHE), Haitian Institute of Statistics and Informatics, Macro International, Inc. Haiti Demographic and Health Survey 2005-2006. Fairfax, United States of America: ICF International.
2000	Haiti Demographic and Health Survey 2000	Haitian Institute of Childhood (IHE), Macro International, Inc. Haiti Demographic and Health Survey 2000. Fairfax, United States of America: ICF International.
Honduras		
2017	Honduras Violence Against Children, Girls and Adolescents Survey 2017	Centers for Disease Control and Prevention (CDC), International Organization for Migration (IOM), Ministry of Security (Honduras), Together for Girls, United Nations Children's Fund (UNICEF). Honduras Violence Against Children, Girls and Adolescents Survey 2017.
2019	Honduras Multiple Indicator Cluster Survey 2019	European Union (EU), Government of Canada, Government of Honduras, National Institute of Statistics (Honduras), Secretary of Health (Honduras),

Supplementary Table S12. Data sources included in the prevalence analysis		
Year	Title	Citation
		United Nations Children's Fund (UNICEF), United Nations Population Fund (UNFPA). Honduras Multiple Indicator Cluster Survey 2019. New York, United States of America: United Nations Children's Fund (UNICEF), 2021.
2001	Honduras Reproductive Health Survey 2001	Honduras Family Planning Association (ASHONPLAFA), Ministry of Health (Honduras), and Division of Reproductive Health-Centers for Disease Control and Prevention (CDC). Honduras Reproductive Health Survey 2001. Tegucigalpa, Honduras: Honduras Family Planning Association (ASHONPLAFA).
2011	Honduras Demographic and Health Survey 2011-2012	ICF Macro, National Institute of Statistics (Honduras). Honduras Demographic and Health Survey 2011-2012. Fairfax, United States of America: ICF International.
2005	Honduras Demographic and Health Survey 2005-2006	Macro International, Inc, National Institute of Statistics (Honduras), Secretary of Health (Honduras). Honduras Demographic and Health Survey 2005-2006. Fairfax, United States of America: ICF International.
2002	Dimensions of Child Sexual Abuse Before Age 15 in Three Central American Countries: Honduras, El Salvador, and Guatemala	Speizer IS, Goodwin M, Whittle L, Clyde M, Rogers J. Dimensions of child sexual abuse before age 15 in three Central American countries: Honduras, El Salvador, and Guatemala. Child Abuse Negl. 2008; 32(4): 455-62.
Hungary		
2012	European Union Violence Against Women Study 2012	European Union Agency for Fundamental Rights. European Union Violence Against Women Study 2012.
Iceland		
2004	Associations Between Sexual Abuse and Family Conflict/Violence, Self-Injurious Behavior, and Substance Use: The Mediating Role of Depressed Mood and Anger	Asgeirsdottir BB, Sigfúsdóttir ID, Gudjonsson GH, Sigurdsson JF. Associations between sexual abuse and family conflict/violence, self-injurious behavior, and substance use: the mediating role of depressed mood and anger. Child Abuse Negl. 2011; 35(3): 210-9.
2004	Gender and the Associated Impairments of Childhood Sexual Abuse: A National Study of Icelandic Youth	Gault-Sherman M, Silver E, Sigfúsdóttir ID. Gender and the associated impairments of childhood sexual abuse: A national study of Icelandic youth. Soc Sci Med. 2009; 69(10): 1515-22.
2003	Iceland Youth Survey 2003	Icelandic Centre for Social Research and Analysis (ICSRA). Iceland Youth Survey 2003.
2004	Iceland Youth Survey 2004	Icelandic Centre for Social Research and Analysis (ICSRA). Iceland Youth Survey 2004.
2006	Iceland Youth Survey 2006	Icelandic Centre for Social Research and Analysis (ICSRA). Iceland Youth Survey 2006.

Supplementary Table S12. Data sources included in the prevalence analysis		
Year	Title	Citation
2007	Iceland Youth Survey 2007	Icelandic Centre for Social Research and Analysis (ICSRA). Iceland Youth Survey 2007.
2009	Iceland Youth Survey 2009	Icelandic Centre for Social Research and Analysis (ICSRA). Iceland Youth Survey 2009.
2010	Iceland Youth Survey 2010	Icelandic Centre for Social Research and Analysis (ICSRA). Iceland Youth Survey 2010.
2012	Iceland Youth Survey 2012	Icelandic Centre for Social Research and Analysis (ICSRA). Iceland Youth Survey 2012.
2013	Iceland Youth Survey 2013	Icelandic Centre for Social Research and Analysis (ICSRA). Iceland Youth Survey 2013.
2014	Iceland Youth Survey 2014	Icelandic Centre for Social Research and Analysis (ICSRA). Iceland Youth Survey 2014.
India		
2003	India - Karnataka Gender, Alcohol and Culture: An International Study (GENACIS) 2003	Aarhus University, Addiction Switzerland Research Institute, Alcohol Research Group, Public Health Institute, Centre for Addiction and Mental Health (Canada), Centre for Alcohol Policy Research, Turning Point Alcohol and Drug Centre (Australia), Kettil Bruun Society for Social and Epidemiological Research on Alcohol, University of North Dakota. India - Karnataka Gender, Alcohol and Culture: An International Study (GENACIS) 2003.
2015	India Demographic and Health Survey 2015-2016	ICF International, International Institute for Population Sciences (India), Ministry of Health and Family Welfare (India). India Demographic and Health Survey 2015-2016. Fairfax, United States of America: ICF International, 2018.
2014	India Masculinity, Intimate Partner Violence and Son Preference 2014	International Center for Research on Women, United Nations Population Fund (UNFPA). India Masculinity, Intimate Partner Violence and Son Preference 2014.
2005	India Demographic and Health Survey 2005-2006	International Institute for Population Sciences (India), Macro International, Inc. India Demographic and Health Survey 2005-2006. Fairfax, United States of America: ICF International.
2004	Nonconsensual Sexual Experiences of Adolescents in Urban India	Jaya J, Hindin MJ. Nonconsensual Sexual Experiences of Adolescents in Urban India. J Adolesc Health. 2007; 40(6): 573e7–573e.
1998	Gender, Sexual Abuse and Risk Behaviours in Adolescents: A Cross-Sectional Survey in Schools in Goa	Patel V, Andrew G. Gender, sexual abuse and risk behaviours in adolescents: a cross-sectional survey in schools in Goa. Natl Med J India. 2001; 14(5): 263-7.
2015	India - Bihar and Uttar Pradesh Understanding the Lives of	Population Council (India). India - Bihar and Uttar Pradesh Understanding the Lives of Adolescents and Young Adults 2015-2016, Wave 1. New Delhi, India: Population Council (India), 2017.

Supplementary Table S12. Data sources included in the prevalence analysis		
Year	Title	Citation
	Adolescents and Young Adults 2015-2016, Wave 1	
Indonesia		
2011	Pathways Between Childhood Trauma, Intimate Partner Violence, and Harsh Parenting: Findings from the UN Multi-Country Study on Men and Violence in Asia and the Pacific	Fulu E, Miedema S, Roselli T, McCook S, Chan KL, Haardörfer R, Jewkes R, UN Multi-country Study on Men and Violence study team. Pathways between childhood trauma, intimate partner violence, and harsh parenting: findings from the UN Multi-country Study on Men and Violence in Asia and the Pacific. Lancet Glob Health. 2017; 5(5): e512–22.
2012	Indonesia United Nations Multi-Country Study on Men and Violence 2012 - Equimundo	Partners for Prevention, Rifka Annisa Women's Crisis Center (Indonesia), United Nations Development Programme (UNDP), United Nations Entity for Gender Equality and the Empowerment of Women (UN Women), United Nations Population Fund (UNFPA), United Nations Volunteers (UNV). Indonesia United Nations Multi-country Study on Men and Violence 2012 - Equimundo.
2016	Indonesia - Papua and West Papua Study on Women's and Men's Life Experiences 2016	Rifka Annisa Women's Crisis Center (Indonesia), Statistics Indonesia, United Nations Development Programme (UNDP), United States Agency for International Development (USAID). Indonesia - Papua and West Papua Study on Women's and Men's Life Experiences 2016.
Ireland		
2001	Ireland Sexual Abuse and Violence Study 2001	Department of Health and Children (Ireland), Department of Justice, Equality and Law Reform (Ireland), Dublin Rape Crisis Center, Royal College of Surgeons in Ireland (RCSI). Ireland Sexual Abuse and Violence Study 2001.
2012	European Union Violence Against Women Study 2012	European Union Agency for Fundamental Rights. European Union Violence Against Women Study 2012.
2001	Secular Trends in Child and Adult Sexual Violence - One Decreasing and the Other Increasing: A Population Survey in Ireland	McGee H, Garavan R, Byrne J, O'Higgins M, Conroy RM. Secular trends in child and adult sexual violence - one decreasing and the other increasing: a population survey in Ireland. Eur J Public Health. 2011; 21(1): 98-103.
Israel		
2002	Student Victimization by Educational Staff in Israel	Khoury-Kassabri M. Student victimization by educational staff in Israel. Child Abuse Negl. 2006; 30(6): 691-707.
Italy		
2006	Italy Violence Against Women Survey 2006	Department for Equal Opportunities (Italy), National Institute of Statistics (Italy). Italy Violence Against Women Survey 2006.

Supplementary Table S12. Data sources included in the prevalence analysis		
Year	Title	Citation
2012	European Union Violence Against Women Study 2012	European Union Agency for Fundamental Rights. European Union Violence Against Women Study 2012.
2014	Italy Violence Against Women Survey 2014	National Institute of Statistics (Italy). Italy Violence Against Women Survey 2014. Rome, Italy: National Institute of Statistics (Italy).
Jamaica		
2002	Jamaica Reproductive Health Survey 2002-2003	Division of Reproductive Health, Centers for Disease Control and Prevention (CDC), Jamaica National Family Planning Board, Statistical Institute of Jamaica. Jamaica Reproductive Health Survey 2002-2003. Kingston, Jamaica: Derek Gordon Databank, University of the West Indies.
2000	Sexual Violence and Reproductive Health Among Young People in Three Communities in Jamaica	Geary CW, Wedderburn M, McCarraher D, Cuthbertson C, Pottinger A. Sexual Violence and Reproductive Health Among Young People in Three Communities in Jamaica. J Interpers Violence. 2006; 21(11): 1512–33.
2016	Jamaica Women’s Health Survey 2016	Global Women’s Institute, George Washington University, Statistical Institute of Jamaica. Jamaica Women’s Health Survey 2016. Inter-American Development Bank (IDB), 2018.
2008	Jamaica Reproductive Health Survey 2008-2009	Jamaica Family Planning Board, Jamaica Division of Reproductive Health-Centers for Disease Control and Prevention (CDC). Jamaica Reproductive Health Survey 2008. Atlanta, United States: Centers for Disease Control and Prevention (CDC).
Kazakhstan		
2002	Kazakhstan Gender, Alcohol and Culture: An International Study (GENACIS) 2002-2003	Aarhus University, Addiction Switzerland Research Institute, Alcohol Research Group, Public Health Institute, Centre for Addiction and Mental Health (Canada), Centre for Alcohol Policy Research, Turning Point Alcohol and Drug Centre (Australia), Kettil Bruun Society for Social and Epidemiological Research on Alcohol, University of North Dakota. Kazakhstan Gender, Alcohol and Culture: An International Study (GENACIS) 2002-2003.
2010	Kazakhstan Multiple Indicator Cluster Survey 2010-2011	Agency of the Republic of Kazakhstan on Statistics, United Nations Children's Fund (UNICEF). Kazakhstan Multiple Indicator Cluster Survey 2010-2011. New York, United States of America: United Nations Children's Fund (UNICEF), 2013.
Kenya		
2003	Kenya Demographic and Health Survey 2003	Centers for Disease Control and Prevention (CDC), Central Bureau of Statistics (Kenya), Macro International, Inc, Ministry of Health (Kenya), National Council for Population and Development (Kenya). Kenya Demographic and Health Survey 2003. Fairfax, United States of America: ICF International.

Supplementary Table S12. Data sources included in the prevalence analysis		
Year	Title	Citation
2018	Kenya Violence Against Children Study 2018-2019	Centers for Disease Control and Prevention (CDC), Kenya National Bureau of Statistics, Ministry of Labour and Social Protection (Kenya), Population Council, Together for Girls, United Nations Children's Fund (UNICEF). Kenya Violence Against Children Study 2018-2019. Washington, DC, United States of America: Together for Girls.
2010	Kenya Violence Against Children Study 2010	Centers for Disease Control and Prevention (CDC), Kenya National Bureau of Statistics, United Nations Children's Fund (UNICEF). Kenya Violence Against Children Study 2010. Nairobi, Kenya: Kenya National Bureau of Statistics.
2001	The Experience of Sexual Coercion among Young People in Kenya	Erulkar AS. The Experience of Sexual Coercion among Young People in Kenya. Int Fam Plan Perspect. 2004; 30(4): 182-9.
2014	Kenya Demographic and Health Survey 2014	ICF International, Kenya Medical Research Institute (KEMRI), Kenya National Bureau of Statistics, Ministry of Health (Kenya), National AIDS Control Council (Kenya), National Council for Population and Development (Kenya). Kenya Demographic and Health Survey 2014. Fairfax, United States of America: ICF International.
2022	Kenya Demographic and Health Survey 2022	ICF International, Kenya National Bureau of Statistics, Ministry of Health (Kenya). Kenya Demographic and Health Survey 2022. Fairfax, United States of America: ICF International, 2023.
2008	Kenya Demographic and Health Survey 2008-2009	ICF Macro, Kenya Medical Research Institute (KEMRI), Kenya National Bureau of Statistics, Ministry of Public Health and Sanitation (Kenya), National AIDS and STI Control Programme (NASCOP) (Kenya), National Association of County and City Health Officials (NACCHO) (United States), National Coordinating Agency for Population and Development (Kenya). Kenya Demographic and Health Survey 2008-2009. Fairfax, United States of America: ICF International.
2000	Trauma Exposure and Post-Traumatic Stress Symptoms in Urban African Schools Survey in CapeTown and Nairobi	Seedat S, Nyamai C, Njenga F, Vythilingum B, Stein DJ. Trauma exposure and post-traumatic stress symptoms in urban African schools Survey in CapeTown and Nairobi. Br J Psychiatry. 2004; 184(2): 169-75.
Kiribati		
2008	Kiribati Family Health and Support Study 2008	Kiribati National Statistics Office, Ministry of Internal and Social Affairs (Kiribati), Secretariat of the Pacific Community (SPC), United Nations Children's Fund (UNICEF), World Health Organization (WHO). Kiribati Family Health and Support Study 2008.
Kyrgyzstan		
2012	Kyrgyzstan Demographic and Health Survey 2012	ICF International, Ministry of Health (Kyrgyzstan), National Statistical Committee of the Kyrgyz Republic. Kyrgyzstan Demographic and Health Survey 2012. Fairfax, United States of America: ICF International.

Supplementary Table S12. Data sources included in the prevalence analysis		
Year	Title	Citation
Lao People's Democratic Republic		
2014	Laos Violence Against Children Survey 2014	Centers for Disease Control and Prevention (CDC), Lao Statistics Bureau, Lao Women's Union, Ministry of Labour and Social Welfare (Laos), National Commission for Mothers and Children (Laos), Together for Girls, United Nations Children's Fund (UNICEF). Laos Violence Against Children Survey 2014.
2014	Laos National Survey on Women's Health and Life Experiences 2013-2014	Lao Statistics Bureau, Lao Women's Union, Ministry of Health (Laos), Ministry of Justice (Laos), Ministry of Public Security (Laos), National Commission for the Advancement of Women (NCAW) (Laos), United Nations Entity for Gender Equality and the Empowerment of Women (UN Women), United Nations Population Fund (UNFPA), World Health Organization (WHO). Laos National Survey on Women's Health and Life Experiences 2013-2014.
Latvia		
2012	European Union Violence Against Women Study 2012	European Union Agency for Fundamental Rights. European Union Violence Against Women Study 2012.
Lesotho		
2018	Lesotho Violence Against Children and Youth Survey 2018	Centers for Disease Control and Prevention (CDC), ICAP, Columbia University Mailman School of Public Health, Ministry of Social Development (Lesotho), Together for Girls. Lesotho Violence Against Children and Youth Survey 2018. Lesotho: Ministry of Social Development (Lesotho).
Liberia		
2006	Liberia Demographic and Health Survey 2006-2007	Liberia Institute for Statistics and Geo-information Services (LISGIS), Macro International, Inc. Liberia Demographic and Health Survey 2006-2007. Fairfax, United States of America: ICF International.
Lithuania		
2012	European Union Violence Against Women Study 2012	European Union Agency for Fundamental Rights. European Union Violence Against Women Study 2012.
Luxembourg		
2012	European Union Violence Against Women Study 2012	European Union Agency for Fundamental Rights. European Union Violence Against Women Study 2012.
Madagascar		
2021		
Malawi		

Supplementary Table S12. Data sources included in the prevalence analysis		
Year	Title	Citation
2013	Malawi Violence Against Children and Young Women Survey 2013	Center for Social Research, University of Malawi, Centers for Disease Control and Prevention (CDC), Ministry of Gender, Children, Disability and Social Welfare (Malawi), President's Emergency Plan for AIDS Relief (PEPFAR), Together for Girls, United Nations Children's Fund (UNICEF). Malawi Violence Against Children and Young Women Survey 2013. Washington, DC, United States of America: Together for Girls.
2015	Malawi Population-Based HIV Impact Assessment 2015-2016	Centers for Disease Control and Prevention (CDC), ICAP, Columbia University Mailman School of Public Health, Johns Hopkins University, Ministry of Health (Malawi), Statistical Center for HIV/AIDS Research and Prevention (SCHARP), University of Malawi College of Medicine, Westat. Malawi Population-Based HIV Impact Assessment 2015-2016.
2015	Malawi Demographic and Health Survey 2015-2016	Emory University and Centers for Disease Control & Prevention Collaboration, ICF International, Ministry of Health (Malawi), National Statistical Office of Malawi. Malawi Demographic and Health Survey 2015-2016. Fairfax, United States of America: ICF International, 2017.
2010	Malawi Demographic and Health Survey 2010	ICF Macro, National Statistical Office of Malawi. Malawi Demographic and Health Survey 2010. Fairfax, United States of America: ICF International.
2004	Malawi Demographic and Health Survey 2004-2005	Macro International, Inc, National Statistical Office of Malawi. Malawi Demographic and Health Survey 2004-2005. Fairfax, United States of America: ICF International.
Malaysia		
2006	Victimization Experiences of Adolescents in Malaysia	Choo W-Y, Dunne MP, Marret MJ, Fleming M, Wong Y-L. Victimization experiences of adolescents in Malaysia. J Adolesc Health. 2011; 49(6): 627-34.
Maldives		
2016	Maldives Demographic and Health Survey 2016-2017	ICF International, Ministry of Health (Maldives). Maldives Demographic and Health Survey 2016-2017. Fairfax, United States of America: ICF International, 2019.
2006	Maldives Study on Women's Health and Life Experiences 2006	Ministry of Gender, Family and Social Services (Maldives). Maldives Study on Women's Health and Life Experiences 2006.
Mali		
2012	Mali Demographic and Health Survey 2012-2013	ICF International, INFO-STAT (Mali), Ministry of Health (Mali), National Institute of Statistics (INSTAT) (Mali), Planning and Statistics Unit, Ministry of Health (Mali). Mali Demographic and Health Survey 2012-2013. Fairfax, United States of America: ICF International, 2014.
2018	Mali Demographic and Health Survey 2018	ICF International, National Institute of Statistics (INSTAT) (Mali), Sector Planning and Statistics Unit Health-Social Development and Family

Supplementary Table S12. Data sources included in the prevalence analysis		
Year	Title	Citation
		Promotion (CPS / SS-DS-PF) (Mali). Mali Demographic and Health Survey 2018. Fairfax, United States of America: ICF International, 2019.
2006	Mali Demographic and Health Survey 2006	Macro International, Inc, Ministry of Health (Mali), National Directorate of Statistics and Informatics (DNSI) (Mali). Mali Demographic and Health Survey 2006. Fairfax, United States of America: ICF International.
Malta		
2010	Malta Nationwide Research Study on the Prevalence of Domestic Violence Against Women 2010	Commission on Gender-Based Violence and Domestic Violence (Malta), M. Fsadni & Associates (Malta). Malta Nationwide Research Study on the Prevalence of Domestic Violence Against Women 2010.
2012	European Union Violence Against Women Study 2012	European Union Agency for Fundamental Rights. European Union Violence Against Women Study 2012.
Marshall Islands		
2012	Marshall Islands Family Health and Safety Study 2012	Ministry of Internal Affairs (Marshall Islands), Women United Together Marshall Islands (WUTMI). Marshall Islands Family Health and Safety Study 2012.
Mexico		
2005	Descriptive Epidemiology of Chronic Childhood Adversity in Mexican Adolescents	Benjet C, Borges G, Medina-Mora ME, Zambrano J, Cruz C, Méndez E. Descriptive Epidemiology of Chronic Childhood Adversity in Mexican Adolescents. J Adolesc Health. 2009; 45(5): 483-9.
2004	[Factors For Sexual Abuse During Childhood and Adolescence in Students of Morelos, Mexico]	Chavez Ayala R, Rivera-Rivera L, Angeles-Llerenas A, Díaz-Cerón E, Allen-Leigh B, Ponce EL. [Factors for sexual abuse during childhood and adolescence in students of Morelos, Mexico]. Rev Saude Publica. 2009; 43(3): 506-14.
2002	Mexico National Survey on Violence Against Women 2002-2003	Institute for Social Security and Services for State Workers (ISSSTE) (Mexico), Mexican Social Security Institute (IMSS), National Institute of Public Health (Mexico), Secretariat of Health (Mexico). Mexico National Survey on Violence Against Women 2002-2003.
2015	Mexico National Survey on the Dynamics of Household Relationships 2016	National Institute of Statistics and Geography (INEGI) (Mexico), National Institute of Women (Mexico), United Nations Women's Fund (UNIFEM). Mexico National Survey on the Dynamics of Household Relationships 2016. Mexico City, Mexico: National Institute of Statistics and Geography (INEGI) (Mexico).
2002	Prevalence of Childhood Sexual Abuse Among Mexican Adolescents	Pineda-Lucatero AG, Trujillo-Hernández B, Millán-Guerrero RO, Vásquez C. Prevalence of childhood sexual abuse among Mexican adolescents. Child Care Health Dev. 2009; 35(2): 184-9.

Supplementary Table S12. Data sources included in the prevalence analysis		
Year	Title	Citation
1991	[Prevalence of Sexual Abuse in Students and its Relation with Drug Abuse]	Ramos-Lira L, Saldívar-Hernández G, Medina-Mora ME, Rojas-Guiot E, Villatoro-Velázquez J. [Prevalence of sexual abuse in students and its relation with drug abuse]. Salud Publica Mex. 1998; 40(3): 221-33.
Micronesia (Federated States of)		
2014	Federated States of Micronesia Family Health and Safety Study 2014	Department of Health and Social Affairs (Micronesia). Federated States of Micronesia Family Health and Safety Study 2014. 2014.
Mongolia		
2017	Mongolia Gender Based Violence Survey 2017	National Statistical Office of Mongolia. Mongolia Gender Based Violence Survey 2017. Ulaanbaatar, Mongolia: National Statistical Office of Mongolia, 2018.
Montenegro		
2018	Montenegro OSCE-Led Survey on the Well-Being and Safety of Women 2018	Ipsos, Organization for Security and Co-operation in Europe (OSCE). Montenegro OSCE-Led Survey on the Well-Being and Safety of Women 2018. 2019.
Mozambique		
2015	Mozambique AIDS Indicator Survey 2015	Centers for Disease Control and Prevention (CDC), ICF International, Ministry of Health (Mozambique), National Institute of Health (Mozambique), National Institute of Statistics (INE) (Mozambique). Mozambique AIDS Indicator Survey 2015. Fairfax, United States of America: ICF International, 2018.
2019	Mozambique Violence Against Children and Youth Survey 2019	Centers for Disease Control and Prevention (CDC), Ministry of Gender, Children and Social Action (Mozambique), Ministry of Health (Mozambique), National Institute of Health (Mozambique), National Institute of Statistics (INE) (Mozambique), United Nations Children's Fund (UNICEF). Mozambique Violence Against Children and Youth Survey 2019.
2011	Mozambique Demographic and Health Survey 2011	ICF Macro, Manhica Health Research Center (CISM), Ministry of Health (Mozambique), National Institute of Statistics (INE) (Mozambique). Mozambique Demographic and Health Survey 2011. Fairfax, United States of America: ICF International.
2001	Mozambique Young Adult Reproductive Health Survey 2001	Mozambique National Institute of Statistics, Division of Reproductive Health-Centers for Disease Control and Prevention (CDC). (2003) Mozambique Young Adult Reproductive Health Survey 2001. Atlanta, United States: Centers for Disease Control and Prevention (CDC).
Namibia		

Supplementary Table S12. Data sources included in the prevalence analysis		
Year	Title	Citation
2019	Namibia Violence Against Children and Youth Survey 2019	Centers for Disease Control and Prevention (CDC), International Training and Education Center for Health (I-TECH), University of Washington, Ministry of Gender Equality, Poverty Eradication and Social Welfare (Namibia), Namibia Statistics Agency, United Nations Children's Fund (UNICEF). Namibia Violence Against Children and Youth Survey 2019.
2013	Namibia Demographic and Health Survey 2013	ICF International, Ministry of Health and Social Services (Namibia), Namibia Institute of Pathology, Namibia Statistics Agency. Namibia Demographic and Health Survey 2013. Fairfax, United States of America: ICF International.
Nauru		
2013	Nauru Family Health and Support Study 2013	Department of Women's Affairs (Nauru), Ministry of Home Affairs (Nauru). Nauru Family Health and Support Study 2013. Nauru: Ministry of Home Affairs (Nauru).
Nepal		
2016	Nepal Demographic and Health Survey 2016-2017	ICF International, Ministry of Health (Nepal), New ERA. Nepal Demographic and Health Survey 2016-2017. Fairfax, United States of America: ICF International, 2017.
2022	Nepal Demographic and Health Survey 2022	ICF International, Ministry of Health and Population (Nepal), New ERA. Nepal Demographic and Health Survey 2022. Fairfax, United States of America: ICF International, 2023.
2011	Nepal Demographic and Health Survey 2011	ICF Macro, Ministry of Health and Population (Nepal), New ERA. Nepal Demographic and Health Survey 2011. Fairfax, United States of America: ICF International.
Netherlands		
2008	Prevalence and Characteristics of Sexual Violence in the Netherlands, the Risk of Revictimization and Pregnancy: Results from A National Population Survey	De Haas S, van Berlo W, Bakker F, Vanwesenbeeck I. Prevalence and characteristics of sexual violence in the Netherlands, the risk of revictimization and pregnancy: results from a national population survey. Violence Vict. 2012; 27(4): 592-608.
2012	European Union Violence Against Women Study 2012	European Union Agency for Fundamental Rights. European Union Violence Against Women Study 2012.
2017	Netherlands Sexual Health Survey 2017	Rutgers Nisso Group. Netherlands Sexual Health Survey 2017.
New Zealand		

Supplementary Table S12. Data sources included in the prevalence analysis		
Year	Title	Citation
2007	New Zealand Gender, Alcohol and Culture: An International Study (GENACIS) 2007	Aarhus University, Addiction Switzerland Research Institute, Alcohol Research Group, Public Health Institute, Centre for Addiction and Mental Health (Canada), Centre for Alcohol Policy Research, Turning Point Alcohol and Drug Centre (Australia), Kettil Bruun Society for Social and Epidemiological Research on Alcohol, University of North Dakota. New Zealand Gender, Alcohol and Culture: An International Study (GENACIS) 2007.
2012	New Zealand Youth2012 Health and Wellbeing of Secondary School Students 2012	Adolescent Health Research Group, University of Auckland, Auckland UniServices, University of Auckland (New Zealand), Faculty of Medical and Health Sciences, University of Auckland. New Zealand Youth2012 Health and Wellbeing of Secondary School Students 2012.
2001	New Zealand Youth2000 Health and Wellbeing of Secondary School Students 2001	Adolescent Health Research Group, University of Auckland, Faculty of Medical and Health Sciences, University of Auckland. New Zealand Youth2000 Health and Wellbeing of Secondary School Students 2001.
2007	New Zealand Youth2007 Health and Wellbeing of Secondary School Students 2007	Adolescent Health Research Group, University of Auckland, Faculty of Medical and Health Sciences, University of Auckland. New Zealand Youth2007 Health and Wellbeing of Secondary School Students 2007.
1990	Prevalence of Childhood Sexual Abuse Experiences in a Community Sample of Women	Anderson J, Martin J, Mullen P, Romans S, Herbison P. Prevalence of Childhood Sexual Abuse Experiences in a Community Sample of Women. J Am Acad Child Adolesc Psychiatry. 1993; 32(5): 911-9.
2007	Prevalence of Child Sexual Abuse Reported by a Cross-Sectional Sample of New Zealand Women	Fanslow JL, Robinson EM, Crengle S, Perese L. Prevalence of child sexual abuse reported by a cross-sectional sample of New Zealand women. Child Abuse Negl. 2007; 31(9): 935-45.
2001	Self-Reported Suicide Attempts and Associated Risk and Protective Factors Among Secondary School Students in New Zealand	Fleming TM, Merry SN, Robinson EM, Denny SJ, Watson PD. Self-reported suicide attempts and associated risk and protective factors among secondary school students in New Zealand. Aust N Z J Psychiatry. 2007; 41(3): 213-21.
2019	New Zealand Youth 2019 Rangatahi Smart Survey 2019	University of Auckland (New Zealand), University of Otago (New Zealand), Victoria University of Wellington (New Zealand). New Zealand Youth 2019 Rangatahi Smart Survey 2019.
1999	Child Sexual Abuse and Persistence of Risky Sexual Behaviors and Negative Sexual Outcomes Over Adulthood: Findings from A Birth Cohort	Van Roode T, Dickson N, Herbison P, Paul C. Child sexual abuse and persistence of risky sexual behaviors and negative sexual outcomes over adulthood: Findings from a birth cohort. Child Abuse Negl. 2009; 33(3): 161-72.
Nicaragua		

Supplementary Table S12. Data sources included in the prevalence analysis		
Year	Title	Citation
2005	Nicaragua Gender, Alcohol and Culture: An International Study (GENACIS) 2005	Aarhus University, Addiction Switzerland Research Institute, Alcohol Research Group, Public Health Institute, Centre for Addiction and Mental Health (Canada), Centre for Alcohol Policy Research, Turning Point Alcohol and Drug Centre (Australia), Kettil Bruun Society for Social and Epidemiological Research on Alcohol, University of North Dakota. Nicaragua Gender, Alcohol and Culture: An International Study (GENACIS) 2005.
2006	Nicaragua Reproductive Health Survey 2006-2007	Division of Reproductive Health, Centers for Disease Control and Prevention (CDC), National Institute for Development Information (Nicaragua). Nicaragua Reproductive Health Survey 2006-2007. Managua, Nicaragua: National Institute for Development Information (Nicaragua).
1997	Nicaragua Demographic and Health Survey 1997-1998	Macro International, Inc, Ministry of Health (Nicaragua), National Institute of Statistics and Censuses (Nicaragua). Nicaragua Demographic and Health Survey 1997-1998. Fairfax, United States of America: ICF International.
2011	Nicaragua National Demographic and Health Survey 2011-2012	Ministry of Health (Nicaragua), National Institute for Development Information (Nicaragua). Nicaragua National Demographic and Health Survey 2011-2012. Managua, Nicaragua: National Institute for Development Information (Nicaragua).
Niger		
2015	Niger Extent and Determinants of Gender-Based Violence 2015	Ministry for the Promotion of Women and the Protection of Children (Niger). Niger Extent and Determinants of Gender-Based Violence 2015.
Nigeria		
2003	Nigeria Gender, Alcohol and Culture: An International Study (GENACIS) 2003	Aarhus University, Addiction Switzerland Research Institute, Alcohol Research Group, Public Health Institute, Centre for Addiction and Mental Health (Canada), Centre for Alcohol Policy Research, Turning Point Alcohol and Drug Centre (Australia), Kettil Bruun Society for Social and Epidemiological Research on Alcohol, University of North Dakota. Nigeria Gender, Alcohol and Culture: An International Study (GENACIS) 2003.
2014	Nigeria Violence Against Children Survey 2014	Centers for Disease Control and Prevention (CDC), Government of Nigeria, Together for Girls, United Nations Children's Fund (UNICEF). Nigeria Violence Against Children Survey 2014.
2018	Nigeria Demographic and Health Survey 2018	Federal Ministry of Health (Nigeria), ICF International, National Population Commission (NPC). Nigeria Demographic and Health Survey 2018. Fairfax, United States of America: ICF International, 2020.
2013	Nigeria Demographic and Health Survey 2013	ICF International, National Population Commission of Nigeria. Nigeria Demographic and Health Survey 2013. Fairfax, United States of America: ICF International.

Supplementary Table S12. Data sources included in the prevalence analysis		
Year	Title	Citation
2008	Nigeria Demographic and Health Survey 2008	Macro International, Inc, National Population Commission of Nigeria. Nigeria Demographic and Health Survey 2008. Fairfax, United States of America: ICF International, 2009.
2015	Nigeria International Men and Gender Equality Study 2015	Promundo. Nigeria International Men and Gender Equality Study 2015.
North Macedonia		
2011	Macedonia Balkan Epidemiological Study on Child Abuse and Neglect 2011	Institute of Child Health (Greece), Saints Cyril and Methodius University of Skopje. Macedonia Balkan Epidemiological Study on Child Abuse and Neglect 2011.
2010	Macedonia Survey of Adverse Childhood Experiences Among Young People 2010	World Health Organization Regional Office for Europe (WHO/Europe). Macedonia Survey of Adverse Childhood Experiences Among Young People 2010.
Norway		
2013	Norway Violence and Rape: A National Prevalence Study of Violence in A Lifespan Perspective 2013	Ipsos MMI (Norway), Norwegian Centre for Violence and Traumatic Stress Studies (NKVTS). Norway Violence and Rape: A National Prevalence Study of Violence in a Lifespan Perspective 2013.
2013	Norway Safety, Violence and Quality of Life Survey 2013	Norwegian Centre for Violence and Traumatic Stress Studies (NKVTS). Norway Safety, Violence and Quality of Life Survey 2013.
1990	Alcohol and Sexual Victimization: A Longitudinal Study of Norwegian Girls	Pedersen W, Skrondal A. Alcohol and sexual victimization: a longitudinal study of Norwegian girls. Addiction. 1996; 91(4): 565-81.
1993	Alcohol and Sexual Victimization: A Longitudinal Study of Norwegian Girls	Pedersen W, Skrondal A. Alcohol and sexual victimization: a longitudinal study of Norwegian girls. Addiction. 1996; 91(4): 565-81.
2013	Violence Against Children, Later Victimisation, and Mental Health: A Cross-Sectional Study of the General Norwegian Population	Thoresen S, Myhre M, Wentzel-Larsen T, Aakvaag HF, Hjemdal OK. Violence against children, later victimisation, and mental health: a cross-sectional study of the general Norwegian population. Eur J Psychotraumatol. 2015; 6(1): 26259.
Palau		
2013	Palau Family Health and Safety Study 2013	Ministry of Health (Palau). Palau Family Health and Safety Study 2013.
Panama		

Supplementary Table S12. Data sources included in the prevalence analysis		
Year	Title	Citation
2009	Panama Sexual and Reproductive Health Survey 2009	Gorgas Memorial Institute for Health Studies. Panama Sexual and Reproductive Health Survey 2009. 2011.
Papua New Guinea		
2014	Papua New Guinea - Western Highlands and West Sepik Family Wellbeing Survey 2014	Australian Government Department of Foreign Affairs and Trade (DFAT), FHI 360. Papua New Guinea - Western Highlands and West Sepik Family Wellbeing Survey 2014.
2011	Pathways Between Childhood Trauma, Intimate Partner Violence, and Harsh Parenting: Findings from the UN Multi-Country Study on Men and Violence in Asia and the Pacific	Fulu E, Miedema S, Roselli T, McCook S, Chan KL, Haardörfer R, Jewkes R, UN Multi-country Study on Men and Violence study team. Pathways between childhood trauma, intimate partner violence, and harsh parenting: findings from the UN Multi-country Study on Men and Violence in Asia and the Pacific. Lancet Glob Health. 2017; 5(5): e512–22.
2017	Papua New Guinea Demographic and Health Survey 2016-2018	ICF International, National Statistical Office (Papua New Guinea). Papua New Guinea Demographic and Health Survey 2016-2018. Fairfax, United States of America: ICF International, 2019.
Paraguay		
2004	Paraguay Reproductive Health Survey 2004	Division of Reproductive Health-Centers for Disease Control and Prevention (CDC). (2005): Paraguay Reproductive Health Survey 2004. Asunción, Paraguay, Paraguayan Center for Population Studies (CEPEP).
1998	Paraguay Contraceptive Prevalence Survey 1998	Division of Reproductive Health-Centers for Disease Control and Prevention (CDC). Paraguay Contraceptive Prevalence Survey 1998. Atlanta, United States: Centers for Disease Control and Prevention (CDC).
2008	Exploring the Associations Between Intimate Partner Violence and Women's Mental Health: Evidence from A Population-Based Study in Paraguay	Ishida K, Stupp P, Melian M, Serbanescu F, Goodwin M. Exploring the associations between intimate partner violence and women's mental health: Evidence from a population-based study in Paraguay. Soc Sci Med. 2010; 71(9): 1653-61.
2008	Paraguay Reproductive Health Survey 2008	Paraguay Center for Population Studies (CEPEP). Paraguay Reproductive Health Survey 2008. Asunción, Paraguay: Paraguayan Center for Population Studies (CEPEP).
Peru		
2005	Peru - Lima and Ayacucho Gender, Alcohol and Culture: An International Study (GENACIS) 2005	Aarhus University, Addiction Switzerland Research Institute, Alcohol Research Group, Public Health Institute, Centre for Addiction and Mental Health (Canada), Centre for Alcohol Policy Research, Turning Point Alcohol and Drug Centre (Australia), Kettil Bruun Society for Social and Epidemiological Research on Alcohol, University of North Dakota. Peru - Lima and Ayacucho Gender, Alcohol and Culture: An International Study (GENACIS) 2005.

Supplementary Table S12. Data sources included in the prevalence analysis		
Year	Title	Citation
2000	Peru WHO Multi-Country Study on Women's Health and Domestic Violence Against Women 2000	Cayetano Heredia University, Flora Tristan Center of Peruvian Women, World Health Organization (WHO). Peru WHO Multi-country Study on Women's Health and Domestic Violence Against Women 2000.
2014	Peru Continuous Demographic and Health Survey 2014 - INEI	ICF International, Ministry of Health (Peru), National Institute of Statistics and Informatics (Peru), National Police of Peru (PNP). Peru Continuous Demographic and Health Survey 2014 - INEI. Lima, Peru: National Institute of Statistics and Informatics (Peru), 2015.
2013	Peru Continuous Demographic and Health Survey 2013 - INEI	ICF International, National Institute of Statistics and Informatics (Peru). Peru Continuous Demographic and Health Survey 2013 - INEI. Lima, Peru: National Institute of Statistics and Informatics (Peru), 2014.
2011	Peru Continuous Demographic and Health Survey 2011	Macro International, Inc, National Institute of Statistics and Informatics (Peru). Peru Continuous Demographic and Health Survey 2011. Fairfax, United States of America: ICF International.
2012	Peru Continuous Demographic and Health Survey 2012	Macro International, Inc, National Institute of Statistics and Informatics (Peru). Peru Continuous Demographic and Health Survey 2012. Fairfax, United States of America: ICF International.
2005	Peru Continuous Demographic and Health Survey 2003-2008	Ministry of Economy and Finance (Peru), National Institute of Statistics and Informatics (Peru), ORC Macro. Peru Continuous Demographic and Health Survey 2003-2008. Fairfax, United States of America: ICF International.
2017	Peru Demographic and Family Health Survey 2017	National Center for Food and Nutrition, National Institute of Health (Peru), National Institute of Statistics and Informatics (Peru), National Police of Peru (PNP). Peru Demographic and Family Health Survey 2017. Lima, Peru: National Institute of Statistics and Informatics (Peru).
2009	Peru Continuous Demographic and Health Survey 2009	National Institute of Statistics and Informatics (Peru), ORC Macro. Peru Continuous Demographic and Health Survey 2009. Fairfax, United States of America: ICF International.
2010	Peru Continuous Demographic and Health Survey 2010	National Institute of Statistics and Informatics (Peru). Peru Continuous Demographic and Health Survey 2010. Fairfax, United States of America: ICF International.
2019	Peru Demographic and Family Health Survey 2019	National Institute of Statistics and Informatics (Peru). Peru Demographic and Family Health Survey 2019. Lima, Peru: National Institute of Statistics and Informatics (Peru).
Philippines		
2005	International Violence Against Women Surveys Data 2002-2005	European Institute for Crime Prevention and Control, affiliated with the United Nations (HEUNI), United Nations Office on Drugs and Crime (UNODC), Statistics Canada, United Nations Interregional Crime and Justice Research Institute (UNICRI). International Violence Against Women Surveys (IVAWS) Data 2002-2005. As provided by the Global Burden of Disease Child Sexual Abuse and Intimate Partner Violence Expert Group. [Unpublished].

Supplementary Table S12. Data sources included in the prevalence analysis		
Year	Title	Citation
2022	Philippines Demographic and Health Survey 2022	ICF International, Philippine Statistics Authority, United States Agency for International Development (USAID). Philippines Demographic and Health Survey 2022. Fairfax, United States of America: ICF International, 2023.
2017	Philippines Demographic and Health Survey 2017	ICF International, Philippines Statistics Authority, United States Agency for International Development (USAID). Philippines Demographic and Health Survey 2017. Fairfax, United States of America: ICF International, 2018.
2013	Philippines Demographic and Health Survey 2013	ICF International, Philippines Statistics Authority. Philippines Demographic and Health Survey 2013. Fairfax, United States of America: ICF International, 2014.
2005	Violence Against Women: An International Perspective	Johnson H, Ollus N, Nevala S. Violence Against Women: An International Perspective. New York City, United States: Springer; 2008.
2008	Philippines Demographic and Health Survey 2008	Macro International, Inc, National Statistics Office (Philippines). Philippines Demographic and Health Survey 2008. Fairfax, United States of America: ICF International, 2010.
Poland		
2004	International Violence Against Women Surveys Data 2002-2005	European Institute for Crime Prevention and Control, affiliated with the United Nations (HEUNI), United Nations Office on Drugs and Crime (UNODC), Statistics Canada, United Nations Interregional Crime and Justice Research Institute (UNICRI). International Violence Against Women Surveys (IVAWS) Data 2002-2005. As provided by the Global Burden of Disease Child Sexual Abuse and Intimate Partner Violence Expert Group. [Unpublished].
2012	European Union Violence Against Women Study 2012	European Union Agency for Fundamental Rights. European Union Violence Against Women Study 2012.
2004	Violence Against Women: An International Perspective	Johnson H, Ollus N, Nevala S. Violence Against Women: An International Perspective. New York City, United States: Springer; 2008.
Portugal		
2012	European Union Violence Against Women Study 2012	European Union Agency for Fundamental Rights. European Union Violence Against Women Study 2012.
Puerto Rico		
1995	Puerto Rico Reproductive Health Survey 1995-1996	University of Puerto Rico. Puerto Rico Reproductive Health Survey 1995-1996. San Juan, Puerto Rico: University of Puerto Rico, 1998.
Republic of Moldova		

Supplementary Table S12. Data sources included in the prevalence analysis		
Year	Title	Citation
2018	Republic of Moldova Violence Against Children and Youth Survey 2018-2019	Centers for Disease Control and Prevention (CDC), International Organization for Migration (IOM), Ministry of Health (Moldova). Republic of Moldova Violence Against Children and Youth Survey 2018-2019. Washington, DC, United States of America: Together for Girls.
1997	Moldova Reproductive Health Survey 1997	Division of Reproductive Health-Centers for Disease Control and Prevention (CDC) and Moldova Ministry of Health. (1998) Moldova Reproductive Health Survey 1997. Atlanta, United States: Centers for Disease Control and Prevention (CDC).
2005	Moldova Demographic and Health Survey 2005	Macro International, Inc, National Scientific and Applied Center for Preventive Medicine (Moldova). Moldova Demographic and Health Survey 2005. Fairfax, United States of America: ICF International.
Romania		
2011	Romania Balkan Epidemiological Study on Child Abuse and Neglect 2011	Babeş-Bolyai University, Institute of Child Health (Greece). Romania Balkan Epidemiological Study on Child Abuse and Neglect 2011.
2012	European Union Violence Against Women Study 2012	European Union Agency for Fundamental Rights. European Union Violence Against Women Study 2012.
Russian Federation		
1993	Adolescent Sexuality in Saint Petersburg, Russia	Lunin I, Hall TL, Mandel JS, Kay J, Hearst N. Adolescent sexuality in Saint Petersburg, Russia. AIDS. 1995; 9(Suppl 1): S53-60.
Rwanda		
2015	Rwanda Violence Against Children and Youth Survey 2015	Centers for Disease Control and Prevention (CDC), Ministry of Education (Rwanda), Ministry of Gender and Family Promotion (Rwanda), Ministry of Health (Rwanda), National Commission for Children (NCC) (Rwanda), National Institute of Statistics (Rwanda), Rwanda National Police (RNP), Together for Girls, United Nations Children's Fund (UNICEF). Rwanda Violence Against Children and Youth Survey 2015.
2014	Rwanda Demographic and Health Survey 2014-2015	ICF International, Ministry of Health (Rwanda), National Institute of Statistics of Rwanda. Rwanda Demographic and Health Survey 2014-2015. Fairfax, United States of America: ICF International, 2016.
2010	Rwanda Demographic and Health Survey 2010-2011	ICF Macro, Ministry of Health (Rwanda), National Institute of Statistics of Rwanda. Rwanda Demographic and Health Survey 2010-2011. Fairfax, United States of America: ICF International.
2010	Rwanda Masculinity and Gender Based Violence Survey 2010	International Center for Research on Women, Rwanda Men's Resource Centre (RWAMREC), Rwanda MenEngage Network, United Nations Development Programme (UNDP). Rwanda Masculinity and Gender Based Violence Survey 2010.

Supplementary Table S12. Data sources included in the prevalence analysis		
Year	Title	Citation
2005	Rwanda Demographic and Health Survey 2005	Macro International, Inc, National Institute of Statistics of Rwanda. Rwanda Demographic and Health Survey 2005. Fairfax, United States of America: ICF International.
2019	Rwanda Demographic and Health Survey 2019-2020	Ministry of Health (Rwanda), National Institute of Statistics (Rwanda), Rwanda Biomedical Center (RBC). Rwanda Demographic and Health Survey 2019-2020. Fairfax, United States of America: ICF International, 2021.
Samoa		
2000	Samoa Family Health and Safety Study 2000	Ministry of Women Affairs (Samoa), Secretariat of the Pacific Community (SPC). Samoa Family Health and Safety Study 2000.
São Tomé and Príncipe		
2008	São Tomé and Príncipe Demographic and Health Survey 2008-2009	ICF Macro, Ministry of Health (São Tomé and Príncipe), National Institute of Statistics (São Tomé and Príncipe). São Tomé and Príncipe Demographic and Health Survey 2008-2009. Fairfax, United States of America: ICF International.
2019	São Tomé and Príncipe Steps Noncommunicable Disease Risk Factors Survey 2019	Ministry of Health (São Tomé and Príncipe), World Health Organization (WHO). São Tomé and Príncipe STEPS Noncommunicable Disease Risk Factors Survey 2019. WHO NCD Microdata Repository, 2022.
Senegal		
2018	Senegal Continuous Demographic and Health Survey 2018	Directorate of Forecasting and Statistics, Ministry of the Economy, Finance and Planning (Senegal), ICF International, Ministry of Health and Social Action (Senegal), United States Agency for International Development (USAID). Senegal Continuous Demographic and Health Survey 2018. Fairfax, United States of America: ICF International, 2020.
2017	Senegal Continuous Demographic and Health Survey 2017	ICF International, Ministry of Health and Social Action (Senegal), National Agency of Statistics and Demography (Senegal), Unit for the Fight Against Malnutrition (Senegal). Senegal Continuous Demographic and Health Survey 2017. Fairfax, United States of America: ICF International, 2018.
2019	Senegal Continuous Demographic and Health Survey 2019	ICF International, Ministry of Health and Social Action (Senegal), National Agency of Statistics and Demography (Senegal), United States Agency for International Development (USAID). Senegal Continuous Demographic and Health Survey 2019. Fairfax, United States of America: ICF International, 2020.
Serbia		
2011	Serbia Balkan Epidemiological Study on Child Abuse and Neglect 2011	Institute of Child Health (Greece), University of Belgrade. Serbia Balkan Epidemiological Study on Child Abuse and Neglect 2011.
Sierra Leone		

Supplementary Table S12. Data sources included in the prevalence analysis		
Year	Title	Citation
2013	Sierra Leone Demographic and Health Survey 2013	ICF International, Ministry of Health and Sanitation (Sierra Leone), Statistics Sierra Leone. Sierra Leone Demographic and Health Survey 2013. Fairfax, United States of America: ICF International, 2014.
Slovakia		
2012	European Union Violence Against Women Study 2012	European Union Agency for Fundamental Rights. European Union Violence Against Women Study 2012.
Slovenia		
2012	European Union Violence Against Women Study 2012	European Union Agency for Fundamental Rights. European Union Violence Against Women Study 2012.
Solomon Islands		
2008	Solomon Islands Family Health and Safety Study 2008	Solomon Islands National Statistics Office (SINSO). Solomon Islands Family Health and Safety Study 2008.
South Africa		
2013	South Africa Optimus Study 2014	Centre for Justice and Crime Prevention (South Africa), UBS Optimus Foundation, University of Cape Town. South Africa Optimus Study 2014. 2016.
2016	South Africa Demographic and Health Survey 2016	Department of Health (South Africa), ICF International, South African Medical Research Council, Statistics South Africa. South Africa Demographic and Health Survey 2016. Fairfax, United States of America: ICF International, 2019.
1998	South Africa Demographic and Health Survey 1998	Department of Health (South Africa), Macro International, Inc, South African Medical Research Council. South Africa Demographic and Health Survey 1998. Fairfax, United States of America: ICF International.
2011	South Africa Gender Based Violence Indicators Study 2010-2013	Gender Links (South Africa). South Africa Gender Based Violence Indicators Study 2010-2013.
1997	Substance Abuse and Behavioral Correlates of Sexual Assault Among South African Adolescents	King G, Flisher AJ, Noubary F, Reece R, Marais A, Lombard C. Substance abuse and behavioral correlates of sexual assault among South African adolescents. Child Abuse Negl. 2004; 28(6): 683-96.
Spain		
2003	Spain Gender, Alcohol and Culture: An International Study (GENACIS) 2003	Aarhus University, Addiction Switzerland Research Institute, Alcohol Research Group, Public Health Institute, Centre for Addiction and Mental Health (Canada), Centre for Alcohol Policy Research, Turning Point Alcohol and Drug Centre (Australia), Kettil Bruun Society for Social and

Supplementary Table S12. Data sources included in the prevalence analysis		
Year	Title	Citation
		Epidemiological Research on Alcohol, University of North Dakota. Spain Gender, Alcohol and Culture: An International Study (GENACIS) 2003.
2012	European Union Violence Against Women Study 2012	European Union Agency for Fundamental Rights. European Union Violence Against Women Study 2012.
2014	Spain Violence Against Women Macro Survey 2014	Government Delegation for Gender Violence (Spain), Ministry of Health, Social Services and Equality (Spain), Sociological Research Center (Spain). Spain Violence Against Women Macro Survey 2014.
2019	Spain Violence Against Women Macro Survey 2019	Government Delegation for Gender Violence (Spain), Ministry of Health, Social Services and Equality (Spain), Sociological Research Center (Spain). Spain Violence Against Women Macro Survey 2019.
1991	[Prevalence and Consequences of Child Sexual Abuse in Spain]	López F, Carpintero E, Hernández A, Martín MJ, Fuertes A. [Prevalence and consequences of child sexual abuse in Spain]. Child Abuse Negl. 1995; 19(9): 1039-50.
Sri Lanka		
2002	Sri Lanka Gender, Alcohol and Culture: An International Study (GENACIS) 2002	Aarhus University, Addiction Switzerland Research Institute, Alcohol Research Group, Public Health Institute, Centre for Addiction and Mental Health (Canada), Centre for Alcohol Policy Research, Turning Point Alcohol and Drug Centre (Australia), Kettil Bruun Society for Social and Epidemiological Research on Alcohol, University of North Dakota. Sri Lanka Gender, Alcohol and Culture: An International Study (GENACIS) 2002.
2012	Sri Lanka United Nations Multi-Country Study on Men and Violence 2012-2013 - Equimundo	CARE International, Partners for Prevention, United Nations Development Programme (UNDP), United Nations Entity for Gender Equality and the Empowerment of Women (UN Women), United Nations Population Fund (UNFPA), United Nations Volunteers (UNV). Sri Lanka United Nations Multi-country Study on Men and Violence 2012-2013 - Equimundo.
2019	Sri Lanka Women's Wellbeing Survey 2019	Department of Census and Statistics (Sri Lanka). Sri Lanka Women's Wellbeing Survey 2019.
2011	Pathways Between Childhood Trauma, Intimate Partner Violence, and Harsh Parenting: Findings from the UN Multi-Country Study on Men and Violence in Asia and the Pacific	Fulu E, Miedema S, Roselli T, McCook S, Chan KL, Haardörfer R, Jewkes R, UN Multi-country Study on Men and Violence study team. Pathways between childhood trauma, intimate partner violence, and harsh parenting: findings from the UN Multi-country Study on Men and Violence in Asia and the Pacific. Lancet Glob Health. 2017; 5(5): e512–22.
2006	Prevalence and Correlates of Sexual Abuse Reported by Late Adolescent School Children in Sri Lanka	Perera B, Ostbye T. Prevalence and correlates of sexual abuse reported by late adolescent school children in Sri Lanka. Int J Adolesc Med Health. 2009; 21(2): 203-11.
Sweden		

Supplementary Table S12. Data sources included in the prevalence analysis		
Year	Title	Citation
2004	Shaming Experiences and the Association Between Adolescent Depression and Psychosocial Risk Factors	Åslund C, Nilsson K, Starrin B, Sjöberg R. Shaming experiences and the association between adolescent depression and psychosocial risk factors. Eur Child Adolesc Psychiatry. 2007; 16(5): 298-304.
1990	Prevalence and Characteristics of Sexual Abuse in A National Sample of Swedish Seventeen-Year-Old Boys and Girls	Edgardh K, Ormstad K. Prevalence and characteristics of sexual abuse in a national sample of Swedish seventeen-year-old boys and girls. Acta Paediatr. 2000; 89(3): 310-9.
2012	European Union Violence Against Women Study 2012	European Union Agency for Fundamental Rights. European Union Violence Against Women Study 2012.
2012	Sweden National Prevalence Study on Exposure to Violence 2012	National Board of Health and Welfare (Sweden), National Center for Knowledge on Men's Violence Against Women, Uppsala University, Statistics Sweden. Sweden National Prevalence Study on Exposure to Violence 2012.
1996	The Association Between Childhood and Adolescent Sexual Abuse and Proxies for Sexual Risk Behavior: A Random Sample of the General Population of Sweden	Steel JL, Herlitz CA. The association between childhood and adolescent sexual abuse and proxies for sexual risk behavior: a random sample of the general population of Sweden. Child Abuse Negl. 2005; 29(10): 1141-53.
Switzerland		
2003	Switzerland International Violence Against Women Survey 2003	European Institute for Crime Prevention and Control, affiliated with the United Nations (HEUNI), United Nations Interregional Crime and Justice Research Institute (UNICRI), University of Lausanne. Switzerland International Violence Against Women Survey 2003.
2003	International Violence Against Women Surveys Data 2002-2005	European Institute for Crime Prevention and Control, affiliated with the United Nations (HEUNI), United Nations Office on Drugs and Crime (UNODC), Statistics Canada, United Nations Interregional Crime and Justice Research Institute (UNICRI). International Violence Against Women Surveys (IVAWS) Data 2002-2005. As provided by the Global Burden of Disease Child Sexual Abuse and Intimate Partner Violence Expert Group. [Unpublished].
2003	Violence Against Women: An International Perspective	Johnson H, Ollus N, Nevala S. Violence Against Women: An International Perspective. New York City, United States: Springer; 2008.
2009	Child Sexual Abuse Revisited: A Population-Based Cross-Sectional Study among Swiss Adolescents	Mohler-Kuo M, Landolt MA, Maier T, Meidert U, Schönbucher V, Schnyder U. Child sexual abuse revisited: a population-based cross-sectional study among swiss adolescents. J Adolesc Health. 2014; 54(3): 304–311e1.
1992	Sexual Victimization in Adolescent Girls (Age 15-20 Years) Enrolled in Post-Mandatory Schools or	Tschumper A, Narring F, Meier C, Michaud P. Sexual victimization in adolescent girls (age 15-20 years) enrolled in post-mandatory schools or

Supplementary Table S12. Data sources included in the prevalence analysis		
Year	Title	Citation
	Professional Training Programmes in Switzerland	professional training programmes in Switzerland. Acta Paediatr. 1998; 87(2): 212-7.
2009	Switzerland Optimus Study 2009-2010	UBS Optimus Foundation, University of Zurich. Switzerland Optimus Study 2009-2010. 2012.
Tajikistan		
2012	Tajikistan Demographic and Health Survey 2012	ICF International, Ministry of Health (Tajikistan), Statistical Agency under the President of the Republic of Tajikistan. Tajikistan Demographic and Health Survey 2012. Fairfax, United States of America: ICF International, 2013.
2017	Tajikistan Demographic and Health Survey 2017	ICF International, Statistical Agency under the President of the Republic of Tajikistan. Tajikistan Demographic and Health Survey 2017. Fairfax, United States of America: ICF International, 2018.
Thailand		
2000	Thailand WHO Multi-Country Study on Women's Health and Domestic Violence Against Women 2000	Foundation for Women (Thailand), Institute for Population and Social Research, Mahidol University (Thailand), World Health Organization (WHO). Thailand WHO Multi-country Study on Women's Health and Domestic Violence Against Women 2000.
Timor-Leste		
2004	A Determination of the Prevalence of Gender-Based Violence Among Conflict-Affected Populations in East Timor	Hynes M, Robertson K, Ward J, Crouse C. A determination of the prevalence of gender-based violence among conflict-affected populations in East Timor. Disasters. 2004; 28(3): 294-321.
2016	Timor-Leste Demographic and Health Survey 2016	ICF International, National Statistics Directorate (Timor-Leste). Timor-Leste Demographic and Health Survey 2016. Fairfax, United States of America: ICF International, 2018.
2009	Timor-Leste Demographic and Health Survey 2009-2010	ICF Macro, Ministry of Finance (Timor-Leste), National Statistics Directorate (Timor-Leste). Timor-Leste Demographic and Health Survey 2009-2010. Fairfax, United States of America: ICF International.
2015	Timor-Leste Nabilan Health and Life Experiences Baseline Study 2015	The Asia Foundation. Timor-Leste Nabilan Health and Life Experiences Baseline Study 2015.
Togo		
2013	Togo Demographic and Health Survey 2013-2014	Directorate General of Statistics and National Accounts (Togo), ICF International, Ministry of Health (Togo), Ministry of Planning, Development and Zoning (Togo). Togo Demographic and Health Survey 2013-2014. Fairfax, United States of America: ICF International, 2015.
Tonga		

Supplementary Table S12. Data sources included in the prevalence analysis		
Year	Title	Citation
2008	Tonga National Study on Domestic Violence Against Women 2008-2009	Ma’a Fafine mo e Famili (Tonga). Tonga National Study on Domestic Violence Against Women 2008-2009.
Türkiye		
2002	Reported Childhood Trauma, Attempted Suicide and Self-Mutilative Behavior Among Women in the General Population	Akyuz G, Sar V, Kugu N, Dogan O. Reported childhood trauma, attempted suicide and self-mutilative behavior among women in the general population. Eur Psychiatry. 2005; 20(3): 268-73.
2008	Turkey National Research on Domestic Violence Against Women 2008	BNB Consulting (Turkey), ICON-INSTITUTE Consulting Group, Institute of Population Studies, Hacettepe University. Turkey National Research on Domestic Violence Against Women 2008.
2014	Turkey National Research on Domestic Violence Against Women 2014	Institute of Population Studies, Hacettepe University, Ministry of Family and Social Policies (Turkey). Turkey National Research on Domestic Violence Against Women 2014.
Uganda		
2003	Uganda Gender, Alcohol and Culture: An International Study (GENACIS) 2003	Aarhus University, Addiction Switzerland Research Institute, Alcohol Research Group, Public Health Institute, Centre for Addiction and Mental Health (Canada), Centre for Alcohol Policy Research, Turning Point Alcohol and Drug Centre (Australia), Kettil Bruun Society for Social and Epidemiological Research on Alcohol, University of North Dakota. Uganda Gender, Alcohol and Culture: An International Study (GENACIS) 2003.
2015	Uganda Violence Against Children Survey 2015	AfriChild Centre, Centers for Disease Control and Prevention (CDC), ChildFund International (CFI), Makerere University School of Public Health (Uganda), Ministry of Gender, Labour and Social Development (MGLSD) (Uganda), Together for Girls, Transcultural Psychological Organisation (Uganda), UNICEF Uganda, Uganda Bureau of Statistics, United Nations Children's Fund (UNICEF). Uganda Violence Against Children Survey 2015.
2016	Uganda Demographic and Health Survey 2016	ICF International, Uganda Bureau of Statistics. Uganda Demographic and Health Survey 2016. Fairfax, United States of America: ICF International, 2018.
2011	Uganda Demographic and Health Survey 2011	ICF Macro, Uganda Bureau of Statistics. Uganda Demographic and Health Survey 2011. Fairfax, United States of America: ICF International.
2006	Uganda Child Verbal Autopsy Study 2007	MEASURE Evaluation Project, Carolina Population Center, University of North Carolina, Macro International, Inc, Ministry of Health (Uganda), Uganda Bureau of Statistics. Uganda Child Verbal Autopsy Study 2007. Calverton, United States of America: Macro International, Inc.
2006	Uganda Demographic and Health Survey 2006	Macro International, Inc, Uganda Bureau of Statistics. Uganda Demographic and Health Survey 2006. Fairfax, United States of America: ICF International.

Supplementary Table S12. Data sources included in the prevalence analysis		
Year	Title	Citation
2020	Uganda National Survey on Violence 2020	Uganda Bureau of Statistics. Uganda National Survey on Violence 2020.
Ukraine		
1999	Ukraine Reproductive Health Survey 1999	Division of Reproductive Health-Centers for Disease Control and Prevention (CDC) and Kiev International Institute of Sociology. (2001) Ukraine Reproductive Health Survey 1999. Atlanta, United States: Centers for Disease Control and Prevention (CDC).
2007	Ukraine Demographic and Health Survey 2007	Macro International, Inc, State Statistical Committee (Ukraine), Ukrainian Center for Social Reforms (UCSR). Ukraine Demographic and Health Survey 2007. Fairfax, United States of America: ICF International.
United Kingdom		
2000	United Kingdom Gender, Alcohol and Culture: An International Study (GENACIS) 2000	Aarhus University, Addiction Switzerland Research Institute, Alcohol Research Group, Public Health Institute, Centre for Addiction and Mental Health (Canada), Centre for Alcohol Policy Research, Turning Point Alcohol and Drug Centre (Australia), Kettil Bruun Society for Social and Epidemiological Research on Alcohol, University of North Dakota. United Kingdom Gender, Alcohol and Culture: An International Study (GENACIS) 2000.
1982	Child Sexual Abuse: A Study of Prevalence in Great Britain	Baker AW, Duncan SP. Child sexual abuse: A study of prevalence in Great Britain. Child Abuse Negl. 1985; 9(4): 457-67.
2012	European Union Violence Against Women Study 2012	European Union Agency for Fundamental Rights. European Union Violence Against Women Study 2012.
1996	Attempted and Completed Incidents of Stranger-Perpetrated Child Sexual Abuse and Abduction	Gallagher B, Bradford M, Pease K. Attempted and completed incidents of stranger-perpetrated child sexual abuse and abduction. Child Abuse Negl. 2008; 32(5): 517-28.
1998	Measuring Child Maltreatment in the United Kingdom: A Study of the Prevalence of Child Abuse and Neglect	May-Chahal C, Cawson P. Measuring child maltreatment in the United Kingdom: a study of the prevalence of child abuse and neglect. Child Abuse Negl. 2005; 29(9): 969-84.
2006	United Kingdom - England Adult Psychiatric Morbidity Survey 2006-2007 - Uk Data Service	National Centre for Social Research and University of Leicester, Adult Psychiatric Morbidity Survey, 2007 [computer file]. 3rd Edition. Colchester, Essex: UK Data Archive [distributor], January 2011. SN: 6379, http://dx.doi.org/10.5255/UKDA-SN-6379-1
2019	United Kingdom - Scottish Health Survey 2019 - Scottish Government	Scottish Centre for Social Research (ScotCen), University of Glasgow. United Kingdom - Scottish Health Survey 2019 - Scottish Government.
United Republic of Tanzania		

Supplementary Table S12. Data sources included in the prevalence analysis		
Year	Title	Citation
2022	Tanzania Demographic and Health Survey 2022	Bureau of Statistics (Tanzania), ICF International, Ministry of Health (Tanzania), Ministry of Health (Zanzibar), Office of the Chief Government Statistician (OCGS) (Zanzibar), Tanzania Food and Nutrition Centre. Tanzania Demographic and Health Survey 2022. Fairfax, United States of America: ICF International, 2023.
2015	Tanzania Demographic and Health Survey 2015-2016	ICF International, Ministry of Health (Zanzibar), Ministry of Health, Community Development, Gender, Elderly and Children (MoHCDEC) (Tanzania), National Bureau of Statistics (Tanzania), Office of the Chief Government Statistician (OCGS) (Zanzibar). Tanzania Demographic and Health Survey 2015-2016. Fairfax, United States of America: ICF International, 2016.
2009	Tanzania Demographic and Health Survey 2009-2010	ICF Macro, National Bureau of Statistics (Tanzania). Tanzania Demographic and Health Survey 2009-2010. Fairfax, United States of America: ICF International.
2009	Tanzania Violence Against Children Study 2009	Ministry of Community Development, Gender and Children (MCDGC) (Tanzania), Muhimbili University of Health and Allied Sciences (Tanzania), National Center for Injury Prevention and Control, Centers for Disease Control and Prevention (CDC), United Nations Children's Fund (UNICEF). Tanzania Violence Against Children Study 2009.
2001	Tanzania WHO Multi-Country Study on Women's Health and Domestic Violence Against Women 2001-2002	Muhimbili University of Health and Allied Sciences (Tanzania), University of Dar es Salaam, World Health Organization (WHO). Tanzania WHO Multi-country Study on Women's Health and Domestic Violence Against Women 2001-2002.
United States Virgin Islands		
2017	United States Virgin Islands Youth Risk Behavior Survey 2017	Department of Health (United States Virgin Islands), University of the Virgin Islands (United States). United States Virgin Islands Youth Risk Behavior Survey 2017.
United States of America		
2001	United States Gender, Alcohol and Culture: An International Study (GENACIS) 2001	Aarhus University, Addiction Switzerland Research Institute, Alcohol Research Group, Public Health Institute, Centre for Addiction and Mental Health (Canada), Centre for Alcohol Policy Research, Turning Point Alcohol and Drug Centre (Australia), Kettil Bruun Society for Social and Epidemiological Research on Alcohol, University of North Dakota. United States Gender, Alcohol and Culture: An International Study (GENACIS) 2001.
2003	The Relationship Between Child Abuse and Adult Obesity Among California Women	Alvarez J, Pavao J, Baumrind N, Kimerling R. The Relationship Between Child Abuse and Adult Obesity Among California Women. Am J Prev Med. 2007; 33(1): 28-33.

Supplementary Table S12. Data sources included in the prevalence analysis		
Year	Title	Citation
2004	Lifetime Interpersonal Violence and Self-Reported Chlamydia Trachomatis Diagnosis Among California Women	Alvarez J, Pavao J, Mack KP, Chow JM, Baumrind N, Kimerling R. Lifetime interpersonal violence and self-reported chlamydia trachomatis diagnosis among California women. J Womens Health (Larchmt). 2009; 18(1): 57-63.
1993	Psychosocial Sequelae of Violent Victimization in A National Youth Sample	Boney-McCoy S, Finkelhor D. Psychosocial sequelae of violent victimization in a national youth sample. J Consult Clin Psychol. 1995; 63(5): 726-36.
1995	United States National Violence Against Women Survey 1995-1996	Centers for Disease Control and Prevention (CDC), National Institute of Justice (United States), Schulman, Ronca and Bucuvalas Inc. (SRBI). United States National Violence Against Women Survey 1995-1996.
2009	United States Behavioral Risk Factor Surveillance System 2009	Centers for Disease Control and Prevention (CDC). United States Behavioral Risk Factor Surveillance System 2009. Atlanta, Georgia: CDC, US Department of Health and Human Services.
2010	United States Behavioral Risk Factor Surveillance System 2010	Centers for Disease Control and Prevention (CDC). United States Behavioral Risk Factor Surveillance System 2010. Atlanta, United States of America: Centers for Disease Control and Prevention (CDC).
2011	United States Behavioral Risk Factor Surveillance System 2011	Centers for Disease Control and Prevention (CDC). United States Behavioral Risk Factor Surveillance System 2011. Atlanta, Georgia: CDC, US Department of Health and Human Services.
2012	United States Behavioral Risk Factor Surveillance System 2012	Centers for Disease Control and Prevention (CDC). United States Behavioral Risk Factor Surveillance System 2012. Atlanta, Georgia: CDC, US Department of Health and Human Services, 2013.
2022	United States Behavioral Risk Factor Surveillance System 2022	Centers for Disease Control and Prevention (CDC). United States Behavioral Risk Factor Surveillance System 2022. Atlanta, United States of America: Centers for Disease Control and Prevention (CDC), 2023.
2001	United States High School Youth Risk Behavior Survey 1991-2015	Centers for Disease Control and Prevention (CDC). United States High School Youth Risk Behavior Survey 1991-2015. Atlanta, United States of America: Centers for Disease Control and Prevention (CDC).
2003	United States High School Youth Risk Behavior Survey 1991-2015	Centers for Disease Control and Prevention (CDC). United States High School Youth Risk Behavior Survey 1991-2015. Atlanta, United States of America: Centers for Disease Control and Prevention (CDC).
2005	United States High School Youth Risk Behavior Survey 1991-2015	Centers for Disease Control and Prevention (CDC). United States High School Youth Risk Behavior Survey 1991-2015. Atlanta, United States of America: Centers for Disease Control and Prevention (CDC).
2007	United States High School Youth Risk Behavior Survey 1991-2015	Centers for Disease Control and Prevention (CDC). United States High School Youth Risk Behavior Survey 1991-2015. Atlanta, United States of America: Centers for Disease Control and Prevention (CDC).

Supplementary Table S12. Data sources included in the prevalence analysis		
Year	Title	Citation
2009	United States High School Youth Risk Behavior Survey 1991-2015	Centers for Disease Control and Prevention (CDC). United States High School Youth Risk Behavior Survey 1991-2015. Atlanta, United States of America: Centers for Disease Control and Prevention (CDC).
2011	United States High School Youth Risk Behavior Survey 1991-2015	Centers for Disease Control and Prevention (CDC). United States High School Youth Risk Behavior Survey 1991-2015. Atlanta, United States of America: Centers for Disease Control and Prevention (CDC).
2013	United States High School Youth Risk Behavior Survey 1991-2015	Centers for Disease Control and Prevention (CDC). United States High School Youth Risk Behavior Survey 1991-2015. Atlanta, United States of America: Centers for Disease Control and Prevention (CDC).
2015	United States High School Youth Risk Behavior Survey 1991-2015	Centers for Disease Control and Prevention (CDC). United States High School Youth Risk Behavior Survey 1991-2015. Atlanta, United States of America: Centers for Disease Control and Prevention (CDC).
2019	United States National Youth Risk Behavior Survey 2019	Centers for Disease Control and Prevention (CDC). United States National Youth Risk Behavior Survey 2019. Atlanta, United States of America: Centers for Disease Control and Prevention (CDC).
2007	Adolescent Violence Perpetration: Associations with Multiple Types of Adverse Childhood Experiences	Duke NN, Pettingell SL, McMorris BJ, Borowsky IW. Adolescent Violence Perpetration: Associations With Multiple Types of Adverse Childhood Experiences. Pediatrics. 2010; 125(4): e778–e786.
1991	Children As Victims of Violence: A National Survey	Finkelhor D, Dziuba-Leatherman J. Children as Victims of Violence: A National Survey. Pediatrics. 1994; 94(4): 413-20.
1985	Sexual Abuse in a National Survey of Adult Men and Women: Prevalence, Characteristics, and Risk Factors	Finkelhor D, Hotaling G, Lewis IA, Smith C. Sexual abuse in a national survey of adult men and women: prevalence, characteristics, and risk factors. Child Abuse Negl. 1990; 14(1): 19-28.
2001	Adverse Childhood Events Are Associated with Obesity and Disordered Eating: Results from A U.S. Population-Based Survey of Young Adults	Fuemmeler BF, Dedert E, McClernon FJ, Beckham JC. Adverse childhood events are associated with obesity and disordered eating: Results from a U.S. population-based survey of young adults. J Trauma Stress. 2009; 22(4): 329-33.
2004	Childhood Maltreatment and Early Alcohol Use Among High-Risk Adolescents	Hamburger ME, Leeb RT, Swahn MH. Childhood maltreatment and early alcohol use among high-risk adolescents. J Stud Alcohol Drugs. 2008; 69(2): 291–5.
2008	United States National Longitudinal Study of Adolescent to Adult Health 2007-2009, Wave 4	Harris, Kathleen Mullan, and J. Richard Udry. National Longitudinal Study of Adolescent to Adult Health (Add Health), 1994-2008. ICPSR21600-v12. Ann Arbor, MI: Inter-university Consortium for Political and Social Research [distributor], 2013-03-08. doi:10.3886/ICPSR21600.v12

Supplementary Table S12. Data sources included in the prevalence analysis		
Year	Title	Citation
1997	Health Status and Health Care Use of Massachusetts Women Reporting Partner Abuse	Hathaway JE, Mucci LA, Silverman JG, Brooks DR, Mathews R, Pavlos CA. Health status and health care use of Massachusetts women reporting partner abuse. Am J Prev Med. 2000; 19(4): 302-7.
1999	Health Status and Health Care Use of Massachusetts Women Reporting Partner Abuse	Hathaway JE, Mucci LA, Silverman JG, Brooks DR, Mathews R, Pavlos CA. Health status and health care use of Massachusetts women reporting partner abuse. Am J Prev Med. 2000; 19(4): 302-7.
1991	United States National Comorbidity Survey: Baseline 1990-1992	Kessler, Ronald C. National Comorbidity Survey: Baseline (NCS-1), 1990-1992. ICPSR06693-v6. Ann Arbor, MI: Inter-university Consortium for Political and Social Research [distributor], 2008-09-12. http://doi.org/10.3886/ICPSR06693.v6
1998	United States Commonwealth Fund Survey of Women's Health 1998	Louis Harris and Associates. United States Commonwealth Fund Survey of Women's Health 1998. New York, United States of America: Commonwealth Fund, 1999.
1996	United States Commonwealth Fund Survey of the Health of Adolescent Girls and Boys 1996-1997	Louis Harris and Associates. United States Commonwealth Fund Survey of the Health of Adolescent Girls and Boys 1996-1997. New York, United States of America: Commonwealth Fund, 1997.
2006	Prevalence and Correlates of Drug/Alcohol-Facilitated and Incapacitated Sexual Assault in A Nationally Representative Sample of Adolescent Girls	McCauley JL, Conoscenti LM, Ruggiero KJ, Resnick HS, Saunders BE, Kilpatrick DG. Prevalence and correlates of drug/alcohol-facilitated and incapacitated sexual assault in a nationally representative sample of adolescent girls. J Clin Child Adolesc Psychol. 2009; 38(2): 295-300.
2004	United States National Epidemiologic Survey on Alcohol and Related Conditions 2004-2005	National Institute on Alcohol Abuse and Alcoholism (NIAAA), National Institutes of Health (NIH), U.S. Department of Health and Human Services. United States National Epidemiologic Survey on Alcohol and Related Conditions 2004-2005.
1997	Dating Violence Against Adolescent Girls and Associated Substance Use, Unhealthy Weight Control, Sexual Risk Behavior, Pregnancy, and Suicidality	Silverman JG, Raj A, Mucci LA, Hathaway JE. Dating violence against adolescent girls and associated substance use, unhealthy weight control, sexual risk behavior, pregnancy, and suicidality. JAMA. 2001; 286(5): 572-9.
1999	Dating Violence Against Adolescent Girls and Associated Substance Use, Unhealthy Weight Control, Sexual Risk Behavior, Pregnancy, and Suicidality	Silverman JG, Raj A, Mucci LA, Hathaway JE. Dating violence against adolescent girls and associated substance use, unhealthy weight control, sexual risk behavior, pregnancy, and suicidality. JAMA. 2001; 286(5): 572-9.
1991	Prevalence and Risk Factors for Childhood Sexual Abuse in Women: National Survey Findings	Vogeltanz ND, Wilsnack SC, Harris TR, Wilsnack RW, Wonderlich SA, Kristjanson AF. Prevalence and risk factors for childhood sexual abuse in women: national survey findings. Child Abuse Negl. 1999; 23(6): 579-92.

Supplementary Table S12. Data sources included in the prevalence analysis		
Year	Title	Citation
1991	Childhood Sexual Abuse and Women's Substance Abuse: National Survey Findings	Wilsnack SC, Vogeltanz ND, Klassen AD, Harris TR. Childhood sexual abuse and women's substance abuse: national survey findings. J Stud Alcohol. 1997; 58(3): 264–71.
Uruguay		
2004	Uruguay Gender, Alcohol and Culture: An International Study (GENACIS) 2004	Aarhus University, Addiction Switzerland Research Institute, Alcohol Research Group, Public Health Institute, Centre for Addiction and Mental Health (Canada), Centre for Alcohol Policy Research, Turning Point Alcohol and Drug Centre (Australia), Kettil Bruun Society for Social and Epidemiological Research on Alcohol, University of North Dakota. Uruguay Gender, Alcohol and Culture: An International Study (GENACIS) 2004.
Viet Nam		
2018	Viet Nam National Study on Violence Against Women 2018-2019	General Statistics Office (Vietnam), United Nations Population Fund (UNFPA). Viet Nam National Study on Violence Against Women 2018-2019.
2009	Vietnam National Study of Domestic Violence Against Women 2009-2010	General Statistics Office (Vietnam), World Health Organization (WHO). Vietnam National Study of Domestic Violence Against Women 2009-2010.
Zambia		
2014	Zambia Violence Against Children Survey (VACS) 2014	Centers for Disease Control and Prevention (CDC), Central Statistical Office (Zambia), Ministry of Community Development and Social Services (MCDSS) (Zambia), Ministry of Youth Sport and Child Development (Zambia), Save the Children International, Together for Girls, United Nations Children's Fund (UNICEF), University of Zambia. Zambia Violence Against Children Survey (VACS) 2014.
2016	Zambia Population-Based HIV Impact Assessment 2016	Centers for Disease Control and Prevention (CDC), ICAP, Columbia University Mailman School of Public Health, Ministry of Health (Zambia), Statistical Center for HIV/AIDS Research and Prevention (SCHARP), Tropical Diseases Research Centre, University of Zambia, Westat. Zambia Population-Based HIV Impact Assessment 2016. New York, New York: ICAP, Columbia University Mailman School of Public Health.
2001	Zambia Demographic and Health Survey 2001-2002	Central Board of Health (Zambia), Central Statistical Office (Zambia), Macro International, Inc. Zambia Demographic and Health Survey 2001-2002. Fairfax, United States of America: ICF International.
2013	Zambia Demographic and Health Survey 2013-2014	Central Statistical Office (Zambia), ICF International, Ministry of Health (Zambia), Tropical Diseases Research Centre, University Teaching Hospital (Zambia), University of Zambia. Zambia Demographic and Health Survey 2013-2014. Fairfax, United States of America: ICF International.
2018	Zambia Demographic and Health Survey 2018-2019	Central Statistical Office (Zambia), ICF International, Ministry of Health (Zambia), University Teaching Hospital (Zambia), University of Zambia.

Supplementary Table S12. Data sources included in the prevalence analysis		
Year	Title	Citation
		Zambia Demographic and Health Survey 2018-2019. Fairfax, United States of America: ICF International, 2020.
2007	Zambia Demographic and Health Survey 2007	Central Statistical Office (Zambia), Macro International, Inc. Zambia Demographic and Health Survey 2007. Fairfax, United States of America: ICF International.
1997	Child Abuse and AIDS-Related Knowledge, Attitudes and Behavior Among Adolescents in Zambia	Slonim-Nevo V, Mukuka L. Child abuse and AIDS-related knowledge, attitudes and behavior among adolescents in Zambia. Child Abuse Negl. 2007; 31(2): 143-59.
Zimbabwe		
2011	Zimbabwe National Baseline Survey on Life Experiences of Adolescents 2011	Centers for Disease Control and Prevention (CDC), Centre for Collaborative Operational Research and Evaluation (Zimbabwe), United Nations Children's Fund (UNICEF), Zimbabwe National Statistics Agency. Zimbabwe National Baseline Survey on Life Experiences of Adolescents 2011.
2017	Zimbabwe Young Adult Survey: A Violence Against Children Study, 2017	Centers for Disease Control and Prevention (CDC), Elizabeth Glaser Pediatric AIDS Foundation (EGPAF), Ministry of Health and Child Welfare (Zimbabwe), Together for Girls, ZimStat. Zimbabwe Young Adult Survey: A Violence Against Children Study, 2017. Washington, DC, United States of America: Together for Girls.
2005	Zimbabwe Demographic and Health Survey 2005-2006	Central Statistical Office (Zimbabwe), Macro International, Inc. Zimbabwe Demographic and Health Survey 2005-2006. Fairfax, United States of America: ICF International.
2015	Zimbabwe Demographic and Health Survey 2015	ICF International, National Microbiology Reference Laboratory, Harare Central Hospital (NMRL) (Zimbabwe), Zimbabwe National Statistics Agency. Zimbabwe Demographic and Health Survey 2015. Fairfax, United States of America: ICF International, 2016.
2010	Zimbabwe Demographic and Health Survey 2010-2011	ICF Macro, Zimbabwe National Statistics Agency. Zimbabwe Demographic and Health Survey 2010-2011. Calverton, United States of America: ICF Macro, 2012.

Supplementary Table S13: DHS and VACS modules included in the age at first sexual violence analysis

Supplementary Table S13: DHS and VACS DHS and VACS modules included in the age at first sexual violence analysis		
Title	Time Period	Citation
Swaziland National Study on Violence Against Children and Young Women 2007	05/2007 to 06/2007	Centers for Disease Control and Prevention (CDC), United Nations Children's Fund (UNICEF). Swaziland National Study on Violence Against Children and Young Women 2007.
Tanzania Violence Against Children Study 2009	11/2009 to 12/2009	Ministry of Community Development, Gender and Children (MCDGC) (Tanzania), Muhimbili University of Health and Allied Sciences (Tanzania), National Center for Injury Prevention and Control, Centers for Disease Control and Prevention (CDC), United Nations Children's Fund (UNICEF). Tanzania Violence Against Children Study 2009.
Kenya Violence Against Children Study 2010	11/2010 to 12/2010	Centers for Disease Control and Prevention (CDC), Kenya National Bureau of Statistics, United Nations Children's Fund (UNICEF). Kenya Violence Against Children Study 2010. Nairobi, Kenya: Kenya National Bureau of Statistics.
Malawi Violence Against Children and Young Women Survey 2013	09/2013 to 10/2013	Center for Social Research, University of Malawi, Centers for Disease Control and Prevention (CDC), Ministry of Gender, Children, Disability and Social Welfare (Malawi), President's Emergency Plan for AIDS Relief (PEPFAR), Together for Girls, United Nations Children's Fund (UNICEF). Malawi Violence Against Children and Young Women Survey 2013. Washington, DC, United States of America: Together for Girls.
Nigeria Violence Against Children Survey 2014	05/2014 to 07/2014	Centers for Disease Control and Prevention (CDC), Government of Nigeria, Together for Girls, United Nations Children's Fund (UNICEF). Nigeria Violence Against Children Survey 2014.
Haiti Violence Against Children Survey 2012	04/2012 to 06/2012	Centers for Disease Control and Prevention (CDC), Government of Haiti, Interuniversity Institute for Research and Development (INURED), Multi-Sector Coordination Committee (CCMHAITI) (Haiti), Together for Girls. Haiti Violence Against Children Survey 2012. Washington, DC, United States of America: Together for Girls.
Cambodia Violence Against Children Survey 2013	02/2013 to 03/2013	Centers for Disease Control and Prevention (CDC), Ministry of Social Affairs, Veterans and Youth Rehabilitation (MoSVY) (Cambodia), Ministry of Women's Affairs (Cambodia), National Institute of Statistics (Cambodia), Together for Girls, United Nations Children's Fund (UNICEF). Cambodia Violence Against Children Survey 2013. Washington, DC, United States of America: Together for Girls.
Zambia Violence Against Children Survey (VACS) 2014	08/2014 to 09/2014	Centers for Disease Control and Prevention (CDC), Central Statistical Office (Zambia), Ministry of Community Development and Social Services (MCDSS) (Zambia), Ministry of Youth Sport and Child Development (Zambia), Save the Children International, Together for Girls, United Nations Children's Fund (UNICEF), University of Zambia. Zambia Violence Against Children Survey (VACS) 2014.
El Salvador Violence Against Children, Girls and Adolescents Survey 2017	01/2017 to 12/2017	Centers for Disease Control and Prevention (CDC), General Directorate of Statistics and Census (DIGESTYC) (El Salvador), International Organization for Migration (IOM), Ministry of Justice and Public Safety (El Salvador), Together for Girls. El Salvador Violence Against Children, Girls and Adolescents Survey 2017.
Honduras Violence Against Children, Girls and Adolescents Survey 2017	08/2017 to 09/2017	Centers for Disease Control and Prevention (CDC), International Organization for Migration (IOM), Ministry of Security (Honduras), Together for Girls, United Nations Children's Fund (UNICEF). Honduras Violence Against Children, Girls and Adolescents Survey 2017.

Supplementary Table S13: DHS and VACS DHS and VACS modules included in the age at first sexual violence analysis		
Title	Time Period	Citation
Kenya Violence Against Children Study 2018-2019	12/2018 to 01/2019	Centers for Disease Control and Prevention (CDC), Kenya National Bureau of Statistics, Ministry of Labour and Social Protection (Kenya), Population Council, Together for Girls, United Nations Children's Fund (UNICEF), University of California, San Francisco. Kenya Violence Against Children Study 2018-2019.
Republic of Moldova Violence Against Children and Youth Survey 2018-2019	10/2018 to 02/2019	Centers for Disease Control and Prevention (CDC), International Organization for Migration (IOM), Ministry of Health (Moldova), Together for Girls. Republic of Moldova Violence Against Children and Youth Survey 2018-2019.
Zimbabwe Young Adult Survey: A Violence Against Children Study, 2017	01/2017 to 12/2017	Centers for Disease Control and Prevention (CDC), Elizabeth Glaser Pediatric AIDS Foundation (EGPAF), Ministry of Health and Child Welfare (Zimbabwe), Together for Girls, ZimStat. Zimbabwe Young Adult Survey: A Violence Against Children Study, 2017.
Lesotho Violence Against Children and Youth Survey 2018	06/2018 to 09/2018	Centers for Disease Control and Prevention (CDC), ICAP, Columbia University Mailman School of Public Health, Ministry of Social Development (Lesotho), Together for Girls. Lesotho Violence Against Children and Youth Survey 2018. Lesotho: Ministry of Social Development (Lesotho).
Colombia Violence Against Children and Youth Survey 2018	01/2018 to 12/2018	Centers for Disease Control and Prevention (CDC), International Organization for Migration (IOM), Ministry of Health and Social Protection (Colombia), Together for Girls. Colombia Violence Against Children and Youth Survey 2018. Bogotá, Colombia: Ministry of Health and Social Protection (Colombia).
Côte d'Ivoire Violence Against Children and Youth Survey 2018	06/2018 to 09/2018	Centers for Disease Control and Prevention (CDC), Ministry of Women, Family and Children (Côte d'Ivoire), National Institute of Statistics (Côte d'Ivoire), National Program for the Care of Orphans and Other Children Made Vulnerable by HIV/AIDS (PNOEV) (Côte d'Ivoire), Together for Girls. Côte d'Ivoire Violence Against Children and Youth Survey 2018. Côte d'Ivoire: Ministry of Women, Family and Children (Côte d'Ivoire).
Azerbaijan Demographic and Health Survey 2006	04/2006 to 08/2006	Macro International, Inc, State Statistical Committee of Azerbaijan. Azerbaijan Demographic and Health Survey 2006. Fairfax, United States of America: ICF International.
Cameroon Demographic and Health Survey 2011	01/2011 to 08/2011	ICF International, Ministry of Economy, Planning and Regional Development (Cameroon), Ministry of Public Health (Cameroon), National Institute of Statistics (Cameroon), Pasteur Center of Cameroon. Cameroon Demographic and Health Survey 2011. Fairfax, United States of America: ICF International.
Dominican Republic Demographic and Health Survey 2007	03/2007 to 08/2007	Center for Social and Demographic Studies (Dominican Republic) (CESDEM), Macro International, Inc. Dominican Republic Demographic and Health Survey 2007. Fairfax, United States of America: ICF International.
Haiti Demographic and Health Survey 2005-2006	10/2005 to 06/2006	Haitian Institute of Childhood (IHE), Haitian Institute of Statistics and Informatics, Macro International, Inc. Haiti Demographic and Health Survey 2005-2006. Fairfax, United States of America: ICF International.
India Demographic and Health Survey 2005-2006	11/2005 to 08/2006	International Institute for Population Sciences (India), Macro International, Inc. India Demographic and Health Survey 2005-2006. Fairfax, United States of America: ICF International.
Liberia Demographic and Health Survey 2006-2007	12/2006 to 04/2007	Liberia Institute for Statistics and Geo-information Services (LISGIS), Macro International, Inc. Liberia Demographic and Health Survey 2006-2007. Fairfax, United States of America: ICF International.

Supplementary Table S13: DHS and VACS DHS and VACS modules included in the age at first sexual violence analysis		
Title	Time Period	Citation
Uganda Demographic and Health Survey 2006	04/2006 to 10/2006	Macro International, Inc, Uganda Bureau of Statistics. Uganda Demographic and Health Survey 2006. Fairfax, United States of America: ICF International.
Ukraine Demographic and Health Survey 2007	07/2007 to 11/2007	Macro International, Inc, State Statistical Committee (Ukraine), Ukrainian Center for Social Reforms (UCSR). Ukraine Demographic and Health Survey 2007. Fairfax, United States of America: ICF International.
Zambia Demographic and Health Survey 2007	04/2007 to 10/2007	Central Statistical Office (Zambia), Macro International, Inc. Zambia Demographic and Health Survey 2007. Fairfax, United States of America: ICF International.
Zimbabwe Demographic and Health Survey 2005-2006	08/2005 to 02/2006	Central Statistical Office (Zimbabwe), Macro International, Inc. Zimbabwe Demographic and Health Survey 2005-2006. Fairfax, United States of America: ICF International.
Ghana Demographic and Health Survey 2008	09/2008 to 11/2008	Ghana Statistical Service, Macro International, Inc, Ministry of Health (Ghana). Ghana Demographic and Health Survey 2008. Fairfax, United States of America: ICF International.
Nepal Demographic and Health Survey 2011	01/2011 to 06/2011	ICF Macro, Ministry of Health and Population (Nepal), New ERA. Nepal Demographic and Health Survey 2011. Fairfax, United States of America: ICF International.
Timor-Leste Demographic and Health Survey 2009-2010	08/2009 to 02/2010	ICF Macro, Ministry of Finance (Timor-Leste), National Statistics Directorate (Timor-Leste). Timor-Leste Demographic and Health Survey 2009-2010. Fairfax, United States of America: ICF International.
Colombia Demographic and Health Survey 2009-2010	11/2009 to 11/2010	ICF Macro, Profamilia (Colombia). Colombia Demographic and Health Survey 2009-2010. Fairfax, United States of America: ICF International, 2011.
Tanzania Demographic and Health Survey 2009-2010	12/2009 to 05/2010	ICF Macro, National Bureau of Statistics (Tanzania). Tanzania Demographic and Health Survey 2009-2010. Fairfax, United States of America: ICF International.
Kenya Demographic and Health Survey 2008-2009	11/2008 to 02/2009	ICF Macro, Kenya Medical Research Institute (KEMRI), Kenya National Bureau of Statistics, Ministry of Public Health and Sanitation (Kenya), National AIDS and STI Control Programme (NASCOP) (Kenya), National Association of County and City Health Officials (NACCHO) (United States), National Coordinating Agency for Population and Development (Kenya). Kenya Demographic and Health Survey 2008-2009. Fairfax, United States of America: ICF International.
Malawi Demographic and Health Survey 2010	06/2010 to 11/2010	ICF Macro, National Statistical Office of Malawi. Malawi Demographic and Health Survey 2010. Fairfax, United States of America: ICF International.
Philippines Demographic and Health Survey 2008	01/2008 to 12/2008	Macro International, Inc, National Statistics Office (Philippines). Philippines Demographic and Health Survey 2008. Fairfax, United States of America: ICF International, 2010.
Nigeria Demographic and Health Survey 2008	06/2008 to 10/2008	Macro International, Inc, National Population Commission of Nigeria. Nigeria Demographic and Health Survey 2008. Fairfax, United States of America: ICF International, 2009.
Sao Tome and Principe Demographic and Health Survey 2008-2009	09/2008 to 01/2009	ICF Macro, Ministry of Health (Sao Tome and Principe), National Institute of Statistics (Sao Tome and Principe). Sao Tome and Principe Demographic and Health Survey 2008-2009. Fairfax, United States of America: ICF International.
Mozambique Demographic and Health Survey 2011	05/2011 to 11/2011	ICF Macro, Manhica Health Research Center (CISM), Ministry of Health (Mozambique), National Institute of Statistics (INE) (Mozambique). Mozambique Demographic and Health Survey 2011. Fairfax, United States of America: ICF International.

Supplementary Table S13: DHS and VACS DHS and VACS modules included in the age at first sexual violence analysis		
Title	Time Period	Citation
Zimbabwe Demographic and Health Survey 2010-2011	09/2010 to 03/2011	ICF Macro, Zimbabwe National Statistics Agency. Zimbabwe Demographic and Health Survey 2010-2011. Calverton, United States of America: ICF Macro, 2012.
Uganda Demographic and Health Survey 2011	06/2011 to 12/2011	ICF Macro, Uganda Bureau of Statistics. Uganda Demographic and Health Survey 2011. Fairfax, United States of America: ICF International.
Rwanda Demographic and Health Survey 2010-2011	09/2010 to 03/2011	ICF Macro, Ministry of Health (Rwanda), National Institute of Statistics of Rwanda. Rwanda Demographic and Health Survey 2010-2011. Fairfax, United States of America: ICF International.
Haiti Demographic and Health Survey 2012	01/2012 to 06/2012	Centers for Disease Control and Prevention (CDC), Haitian Institute of Childhood (IHE), Haitian Institute of Statistics and Informatics, Macro International, Inc. Haiti Demographic and Health Survey 2012. Fairfax, United States of America: ICF International.
Tajikistan Demographic and Health Survey 2012	07/2012 to 09/2012	ICF International, Ministry of Health (Tajikistan), Statistical Agency under the President of the Republic of Tajikistan. Tajikistan Demographic and Health Survey 2012. Fairfax, United States of America: ICF International, 2013.
Gabon Demographic and Health Survey 2012	01/2012 to 05/2012	General Directorate of Statistics (Gabon), ICF International, Ministry of Economy, Employment and Sustainable Development (Gabon), Ministry of Health and Social Affairs (Gabon). Gabon Demographic and Health Survey 2012. Fairfax, United States of America: ICF International, 2013.
Comoros Demographic and Health Survey 2012-2013	07/2012 to 01/2013	General Directorate of Statistics and Forecasting (Comoros), ICF International. Comoros Demographic and Health Survey 2012-2013. Fairfax, United States of America: ICF International.
Democratic Republic of the Congo Demographic and Health Survey 2013-2014	11/2013 to 02/2014	ICF International, Ministry of Planning and Monitoring Implementation of the Revolution of Modernity (Congo, DR), Ministry of Public Health (Congo, DR), National Institute of Statistics (Congo, DR). Democratic Republic of the Congo Demographic and Health Survey 2013-2014. Fairfax, United States of America: ICF International, 2014.
Gambia Demographic and Health Survey 2013	02/2013 to 04/2013	Gambia Bureau of Statistics (GBOS), ICF International, Ministry of Health and Social Welfare (The Gambia). Gambia Demographic and Health Survey 2013. Fairfax, United States of America: ICF International, 2015.
Mali Demographic and Health Survey 2012-2013	11/2012 to 02/2013	ICF International, INFO-STAT (Mali), Ministry of Health (Mali), National Institute of Statistics (INSTAT) (Mali), Planning and Statistics Unit, Ministry of Health (Mali). Mali Demographic and Health Survey 2012-2013. Fairfax, United States of America: ICF International, 2014.
Nigeria Demographic and Health Survey 2013	02/2013 to 06/2013	ICF International, National Population Commission of Nigeria. Nigeria Demographic and Health Survey 2013. Fairfax, United States of America: ICF International.
Togo Demographic and Health Survey 2013-2014	11/2013 to 04/2014	Directorate General of Statistics and National Accounts (Togo), ICF International, Ministry of Health (Togo), Ministry of Planning, Development and Zoning (Togo). Togo Demographic and Health Survey 2013-2014. Fairfax, United States of America: ICF International, 2015.
Zambia Demographic and Health Survey 2013-2014	08/2013 to 04/2014	Central Statistical Office (Zambia), ICF International, Ministry of Health (Zambia), Tropical Diseases Research Centre, University Teaching Hospital (Zambia), University of Zambia. Zambia Demographic and Health Survey 2013-2014. Fairfax, United States of America: ICF International.

Supplementary Table S13: DHS and VACS DHS and VACS modules included in the age at first sexual violence analysis		
Title	Time Period	Citation
Kyrgyzstan Demographic and Health Survey 2012	09/2012 to 12/2012	ICF International, Ministry of Health (Kyrgyzstan), National Statistical Committee of the Kyrgyz Republic. Kyrgyzstan Demographic and Health Survey 2012. Fairfax, United States of America: ICF International.
Dominican Republic Demographic and Health Survey 2013	07/2013 to 10/2013	Center for Social and Demographic Studies (Dominican Republic) (CESDEM), ICF International, Ministry of Public Health and Social Assistance (Dominican Republic). Dominican Republic Demographic and Health Survey 2013. Fairfax, United States of America: ICF International, 2014.
Honduras Demographic and Health Survey 2011-2012	09/2011 to 07/2012	ICF Macro, National Institute of Statistics (Honduras). Honduras Demographic and Health Survey 2011-2012. Fairfax, United States of America: ICF International.
Sierra Leone Demographic and Health Survey 2013	06/2013 to 10/2013	ICF International, Ministry of Health and Sanitation (Sierra Leone), Statistics Sierra Leone. Sierra Leone Demographic and Health Survey 2013. Fairfax, United States of America: ICF International, 2014.
Philippines Demographic and Health Survey 2013	08/2013 to 10/2013	ICF International, Philippines Statistics Authority. Philippines Demographic and Health Survey 2013. Fairfax, United States of America: ICF International, 2014.
Namibia Demographic and Health Survey 2013	05/2013 to 09/2013	ICF International, Ministry of Health and Social Services (Namibia), Namibia Institute of Pathology, Namibia Statistics Agency. Namibia Demographic and Health Survey 2013. Fairfax, United States of America: ICF International.
Afghanistan Demographic and Health Survey 2015-2016	06/2015 to 02/2016	Central Statistics Organization (Afghanistan), ICF International, Ministry of Public Health (Afghanistan). Afghanistan Demographic and Health Survey 2015-2016. Fairfax, United States of America: ICF International, 2017.
Cambodia Demographic and Health Survey 2014	06/2014 to 12/2014	ICF International, Ministry of Health (Cambodia), National Institute of Statistics (Cambodia). Cambodia Demographic and Health Survey 2014. Fairfax, United States of America: ICF International, 2017.
Chad Demographic and Health Survey 2014-2015	10/2014 to 04/2015	ICF International, National Institute for Statistics, Economic and Demographic Studies (INSEED) (Chad). Chad Demographic and Health Survey 2014-2015. Fairfax, United States of America: ICF International, 2016.
Guatemala Demographic and Health Survey 2014-2015	10/2014 to 04/2015	ICF International, Institute of Nutrition of Central America and Panama, Ministry of Public Health and Social Assistance (Guatemala), National Statistics Institute (Guatemala), Secretary of Planning and Programming of the Presidency (Segeplán) (Guatemala). Guatemala Demographic and Health Survey 2014-2015. Fairfax, United States of America: ICF International, 2017.
Kenya Demographic and Health Survey 2014	05/2014 to 10/2014	ICF International, Kenya Medical Research Institute (KEMRI), Kenya National Bureau of Statistics, Ministry of Health (Kenya), National AIDS Control Council (Kenya), National Council for Population and Development (Kenya). Kenya Demographic and Health Survey 2014. Fairfax, United States of America: ICF International.
Mozambique AIDS Indicator Survey 2015	05/2015 to 10/2015	Centers for Disease Control and Prevention (CDC), ICF International, Ministry of Health (Mozambique), National Institute of Health (Mozambique), National Institute of Statistics (INE) (Mozambique). Mozambique AIDS Indicator Survey 2015. Fairfax, United States of America: ICF International, 2018.
Myanmar Demographic and Health Survey 2015-2016	12/2015 to 07/2016	ICF International, Ministry of Health and Sports (Myanmar). Myanmar Demographic and Health Survey 2015-2016. Fairfax, United States of America: ICF International, 2017.

Supplementary Table S13: DHS and VACS DHS and VACS modules included in the age at first sexual violence analysis		
Title	Time Period	Citation
Rwanda Demographic and Health Survey 2014-2015	11/2014 to 04/2015	ICF International, Ministry of Health (Rwanda), National Institute of Statistics of Rwanda. Rwanda Demographic and Health Survey 2014-2015. Fairfax, United States of America: ICF International, 2016.
South Africa Demographic and Health Survey 2016	06/2016 to 11/2016	Department of Health (South Africa), ICF International, South African Medical Research Council, Statistics South Africa. South Africa Demographic and Health Survey 2016. Fairfax, United States of America: ICF International, 2019.
Zimbabwe Demographic and Health Survey 2015	07/2015 to 12/2015	ICF International, National Microbiology Reference Laboratory, Harare Central Hospital (NMRL) (Zimbabwe), Zimbabwe National Statistics Agency. Zimbabwe Demographic and Health Survey 2015. Fairfax, United States of America: ICF International, 2016.
Angola Demographic and Health Survey 2015-2016	10/2015 to 03/2016	ICF International, Ministry of Health (Angola), National Institute of Statistics (Angola), United Nations Children's Fund (UNICEF). Angola Demographic and Health Survey 2015-2016. Fairfax, United States of America: ICF International, 2017.
Armenia Demographic and Health Survey 2015-2016	12/2015 to 04/2016	ICF International, Ministry of Health (Armenia), National Statistical Service of the Republic of Armenia. Armenia Demographic and Health Survey 2015-2016. Fairfax, United States of America: ICF International, 2017.
Benin Demographic and Health Survey 2017-2018	11/2017 to 02/2018	Hubert Koutoukou Maga National University Hospital Center (CNHU-HKM) (Benin), ICF International, National Institute of Statistics and Economic Analysis (INSAE) (Benin), National Malaria Control Program, Ministry of Health (Benin), Permanent Secretariat of the Food Council and Nutrition (SP-CAN) (Benin). Benin Demographic and Health Survey 2017-2018. Fairfax, United States of America: ICF International, 2018.
Colombia Demographic and Health Survey 2015-2016	02/2015 to 03/2016	ICF International, Ministry of Health (Colombia), Profamilia (Colombia). Colombia Demographic and Health Survey 2015-2016. Fairfax, United States of America: ICF International, 2017.
Ethiopia Demographic and Health Survey 2016	01/2016 to 06/2016	Central Statistics Agency (Ethiopia), ICF International. Ethiopia Demographic and Health Survey 2016. Fairfax, United States of America: ICF International, 2017.
Haiti Demographic and Health Survey 2016-2017	11/2016 to 04/2017	Haitian Institute of Childhood (IHE), Haitian Institute of Statistics and Informatics, ICF International, Ministry of Public Health and Population (Haiti). Haiti Demographic and Health Survey 2016-2017. Fairfax, United States of America: ICF International.
Malawi Demographic and Health Survey 2015-2016	10/2015 to 02/2016	Emory University and Centers for Disease Control & Prevention Collaboration, ICF International, Ministry of Health (Malawi), National Statistical Office of Malawi. Malawi Demographic and Health Survey 2015-2016. Fairfax, United States of America: ICF International, 2017.
Maldives Demographic and Health Survey 2016-2017	03/2016 to 11/2017	ICF International, Ministry of Health (Maldives). Maldives Demographic and Health Survey 2016-2017. Fairfax, United States of America: ICF International, 2019.
Tanzania Demographic and Health Survey 2015-2016	08/2015 to 02/2016	ICF International, Ministry of Health (Zanzibar), Ministry of Health, Community Development, Gender, Elderly and Children (MoHCDEC) (Tanzania), National Bureau of Statistics (Tanzania), Office of the Chief Government Statistician (OCGS) (Zanzibar). Tanzania Demographic and Health Survey 2015-2016. Fairfax, United States of America: ICF International, 2016.
Burundi Demographic and Health Survey 2016-2017	10/2016 to 03/2017	Burundi Institute of Statistics and Economic Studies, ICF International, Ministry of Public Health and the Fight Against AIDS

Supplementary Table S13: DHS and VACS DHS and VACS modules included in the age at first sexual violence analysis		
Title	Time Period	Citation
		(Burundi). Burundi Demographic and Health Survey 2016-2017. Fairfax, United States of America: ICF International, 2018.
Uganda Demographic and Health Survey 2016	06/2016 to 11/2016	ICF International, Uganda Bureau of Statistics. Uganda Demographic and Health Survey 2016. Fairfax, United States of America: ICF International, 2018.
Nepal Demographic and Health Survey 2016-2017	06/2016 to 01/2017	ICF International, Ministry of Health (Nepal), New ERA. Nepal Demographic and Health Survey 2016-2017. Fairfax, United States of America: ICF International, 2017.
Pakistan Demographic and Health Survey 2017-2018	11/2017 to 04/2018	ICF International, Ministry of National Health Services, Regulations & Coordination (Pakistan), National Institute of Population Studies (Pakistan). Pakistan Demographic and Health Survey 2017-2018. Fairfax, United States of America: ICF International, 2018.
Timor-Leste Demographic and Health Survey 2016	09/2016 to 12/2016	ICF International, National Statistics Directorate (Timor-Leste). Timor-Leste Demographic and Health Survey 2016. Fairfax, United States of America: ICF International, 2018.
Philippines Demographic and Health Survey 2017	08/2017 to 10/2017	ICF International, Philippines Statistics Authority, United States Agency for International Development (USAID). Philippines Demographic and Health Survey 2017. Fairfax, United States of America: ICF International, 2018.
Tajikistan Demographic and Health Survey 2017	08/2017 to 11/2017	ICF International, Statistical Agency under the President of the Republic of Tajikistan. Tajikistan Demographic and Health Survey 2017. Fairfax, United States of America: ICF International, 2018.
Senegal Continuous Demographic and Health Survey 2017	04/2017 to 12/2017	ICF International, Ministry of Health and Social Action (Senegal), National Agency of Statistics and Demography (Senegal), Unit for the Fight Against Malnutrition (Senegal). Senegal Continuous Demographic and Health Survey 2017. Fairfax, United States of America: ICF International, 2018.
Mali Demographic and Health Survey 2018	09/2018 to 11/2018	ICF International, National Institute of Statistics (INSTAT) (Mali), Sector Planning and Statistics Unit Health-Social Development and Family Promotion (CPS / SS-DS-PF) (Mali). Mali Demographic and Health Survey 2018. Fairfax, United States of America: ICF International, 2019.
Nigeria Demographic and Health Survey 2018	08/2018 to 12/2018	Federal Ministry of Health (Nigeria), ICF International, National Population Commission (NPC). Nigeria Demographic and Health Survey 2018. Fairfax, United States of America: ICF International, 2020.
Zambia Demographic and Health Survey 2018-2019	07/2018 to 01/2019	Central Statistical Office (Zambia), ICF International, Ministry of Health (Zambia), University Teaching Hospital (Zambia), University of Zambia. Zambia Demographic and Health Survey 2018-2019. Fairfax, United States of America: ICF International, 2020.
Cameroon Demographic and Health Survey 2018-2019	06/2018 to 01/2019	ICF International, Ministry of Public Health (Cameroon), National Institute of Statistics (Cameroon). Cameroon Demographic and Health Survey 2018-2019. Fairfax, United States of America: ICF International.
Sierra Leone Demographic and Health Survey 2019	05/2019 to 08/2019	ICF International, Ministry of Health and Sanitation (Sierra Leone), Statistics Sierra Leone. Sierra Leone Demographic and Health Survey 2019. Fairfax, United States of America: ICF International, 2020.
Papua New Guinea Demographic and Health Survey 2016-2018	09/2016 to 12/2018	ICF International, National Statistical Office (Papua New Guinea). Papua New Guinea Demographic and Health Survey 2016-2018. Fairfax, United States of America: ICF International, 2019.
Senegal Continuous Demographic and Health Survey 2018	06/2018 to 12/2018	Directorate of Forecasting and Statistics, Ministry of the Economy, Finance and Planning (Senegal), ICF International, Ministry of Health and Social Action (Senegal), United States Agency for

Supplementary Table S13: DHS and VACS DHS and VACS modules included in the age at first sexual violence analysis		
Title	Time Period	Citation
		International Development (USAID). Senegal Continuous Demographic and Health Survey 2018. Fairfax, United States of America: ICF International, 2020.
Liberia Demographic and Health Survey 2019-2020	10/2019 to 02/2020	ICF International, Liberia Institute for Statistics and Geo-information Services (LISGIS), Ministry of Health and Social Welfare (Liberia). Liberia Demographic and Health Survey 2019-2020. 2021.
Gambia Demographic and Health Survey 2019-2020	11/2019 to 03/2020	Gambia Bureau of Statistics (GBOS), ICF International, Ministry of Health and Social Welfare (The Gambia). Gambia Demographic and Health Survey 2019-2020. Fairfax, United States of America: ICF International, 2021.
Senegal Continuous Demographic and Health Survey 2019	04/2019 to 12/2019	ICF International, Ministry of Health and Social Action (Senegal), National Agency of Statistics and Demography (Senegal), United States Agency for International Development (USAID). Senegal Continuous Demographic and Health Survey 2019. Fairfax, United States of America: ICF International, 2020.
Rwanda Demographic and Health Survey 2019-2020	11/2019 to 07/2020	Ministry of Health (Rwanda), National Institute of Statistics (Rwanda), Rwanda Biomedical Center (RBC). Rwanda Demographic and Health Survey 2019-2020. Fairfax, United States of America: ICF International, 2021.
Mauritania Demographic and Health Survey 2019-2020	11/2019 to 03/2021	National Office of Statistics (Mauritania). Mauritania Demographic and Health Survey 2019-2020. Fairfax, United States of America: ICF International, 2022.
Madagascar Demographic and Health Survey 2021	03/2021 to 07/2021	ICF Macro, National Institute of Statistics (Madagascar). Madagascar Demographic and Health Survey 2021. Fairfax, United States of America: ICF International, 2022.
Cambodia Demographic and Health Survey 2021-2022	09/2021 to 02/2022	ICF International, Ministry of Health (Cambodia), National Institute of Statistics (Cambodia). Cambodia Demographic and Health Survey 2021-2022. Fairfax, United States of America: ICF International, 2023.
Philippines Demographic and Health Survey 2022	05/2022 to 06/2022	ICF International, Philippine Statistics Authority, United States Agency for International Development (USAID). Philippines Demographic and Health Survey 2022. Fairfax, United States of America: ICF International, 2023.
Burkina Faso Demographic and Health Survey 2021	07/2021 to 11/2021	ICF International, National Institute of Public Health (INSP) (Burkina Faso), National Institute of Statistics and Demography (Burkina Faso). Burkina Faso Demographic and Health Survey 2021.
Nepal Demographic and Health Survey 2022	01/2022 to 06/2022	ICF International, Ministry of Health and Population (Nepal), New ERA. Nepal Demographic and Health Survey 2022. Fairfax, United States of America: ICF International, 2023.
Kenya Demographic and Health Survey 2022	02/2022 to 07/2022	ICF International, Kenya National Bureau of Statistics, Ministry of Health (Kenya). Kenya Demographic and Health Survey 2022. Fairfax, United States of America: ICF International, 2023.
Gabon Demographic and Health Survey 2019-2021	11/2019 to 10/2021	General Directorate of Statistics (Gabon), ICF International, Ministry of Economy, Employment and Sustainable Development (Gabon), Ministry of Health and Social Affairs (Gabon), Ministry of Higher Education and Scientific Research (Gabon). Gabon Demographic and Health Survey 2019-2021. Fairfax, United States of America: ICF International, 2023.
Côte d'Ivoire Demographic and Health Survey 2021	09/2021 to 12/2021	ICF International, Ministry of Health and Public Hygiene (MSHP) (Côte d'Ivoire), National Institute of Statistics (Côte d'Ivoire). Côte d'Ivoire Demographic and Health Survey 2021. Fairfax, United States of America: ICF International, 2023.
Tanzania Demographic and Health Survey 2022	02/2022 to 07/2022	Bureau of Statistics (Tanzania), ICF International, Ministry of Health (Tanzania), Ministry of Health (Zanzibar), Office of the Chief

Supplementary Table S13: DHS and VACS DHS and VACS modules included in the age at first sexual violence analysis		
Title	Time Period	Citation
		Government Statistician (OCGS) (Zanzibar), Tanzania Food and Nutrition Centre. Tanzania Demographic and Health Survey 2022. Fairfax, United States of America: ICF International, 2023.
